# Supplementary material for: RBAD: The first database dedicated alterations of blood RNA in individuals with Alzheimer’s disease and their clinical relevance
Source: Neural Regen Res. 2025 Mar 25;21(6):2553–62. doi: 10.4103/NRR.NRR-D-24-01165 (PMC13211806; doi:10.4103/NRR.NRR-D-24-01165)
Supplement: Supplementary file 3 [file NRR-21-2553_Suppl3.pdf]

Additional Table 6. Clinical-associated genes detected in RBAD

Symbol	Group (dataset, subgroup, clinical outcome)
IGLON5	ACOM,female,AD.vs.control; ACOM,male,AD.vs.control; Homo_723,female,AD.vs.control; Homo_723,female,AD.vs.MCI; ROSMAP,female,MCI.vs.control; ROSMAP,control,male.vs.female; Emory,totalSample,immune-CD4_T; Emory,totalSample,immune-iTreg; ROSMAP,male-excludeAge90+,survival
CRIM1	ACOM,female,AD.vs.control; Homo_723,female,AD.vs.control; Homo_723,male,AD.vs.control; Homo_723,female,AD.vs.MCI; Homo_723,male,AD.vs.MCI; Homo_723,female,MCI.vs.control; Homo_723,MCI,male.vs.female; Homo_714,female,AD.vs.control; Homo_714,female,AD.vs.MCI; Homo_714,control,male.vs.female; Homo_714,MCI,male.vs.female; Homo_633,female,AD.vs.MCI; Homo_633,female,MCI.vs.control; Homo_633,control,male.vs.female; Homo_633,MCI,male.vs.female; ROSMAP,female,MCI.vs.control; Emory,MCI,age; ROSMAP,AD,last.visit.age; ROSMAP,MCI,last.visit.age; ROSMAP,AD,MMSE; Emory,totalSample,immune-InfiltrationScore; Homo_723,totalSample,immune-Monocyte; Homo_723,totalSample,immune-NKT; Homo_723,totalSample,immune-CD4_naive; Homo_723,totalSample,immune-InfiltrationScore; Homo_723,totalSample,immune-Central_memory; Homo_714,totalSample,immune-NKT; Homo_714,totalSample,immune-CD4_naive; Homo_714,totalSample,immune-Monocyte; Homo_714,totalSample,immune-InfiltrationScore; Homo_714,totalSample,immune-Th1; Homo_633,totalSample,immune-NKT; Homo_633,totalSample,immune-Monocyte; Homo_633,totalSample,immune-InfiltrationScore; Homo_633,totalSample,immune-CD4_naive; MCSA,totalSample,immune-DC; MCSA,totalSample,immune-Macrophage; MCSA,totalSample,immune-CD4_T; MCSA,totalSample,immune-CD4_naive; MCSA,totalSample,immune-Tr1; MCSA,totalSample,immune-nTreg; MCSA,totalSample,immune-iTreg; MCSA,totalSample,immune-Th17; MCSA,totalSample,immune-Tfh; MCSA,totalSample,immune-CD8_naive; MCSA,totalSample,immune-Cytotoxic; MCSA,totalSample,immune-Exhausted; MCSA,totalSample,immune-MAIT; MCSA,totalSample,immune-Central_memory;
ZNF175	ACOM,female,AD.vs.control; ACOM,male,AD.vs.control; ACOM,AD,age; MCSA,control,age; Emory,totalSample,immune-Macrophage; Emory,totalSample,immune-Neutrophil; Emory,totalSample,immune-CD4_T; Emory,totalSample,immune-Tr1; Emory,totalSample,immune-iTreg; Emory,totalSample,immune-Tfh; Emory,totalSample,immune-InfiltrationScore; Homo_723,totalSample,immune-CD4_naive; Homo_723,totalSample,immune-CD4_T; Homo_723,totalSample,immune-Tr1; Homo_723,totalSample,immune-iTreg; Homo_723,totalSample,immune-Tfh; Homo_723,totalSample,immune-Central_memory; Homo_723,totalSample,immune-Th2; Homo_723,totalSample,immune-nTreg; Homo_723,totalSample,immune-InfiltrationScore; Homo_723,totalSample,immune-NKT; Homo_723,totalSample,immune-Effector_memory; Homo_723,totalSample,immune-Th1; Homo_723,totalSample,immune-Cytotoxic; Homo_723,totalSample,immune-Macrophage; Homo_714,totalSample,immune-CD4_naive; Homo_714,totalSample,immune-CD4_T; Homo_714,totalSample,immune-Tfh; Homo_714,totalSample,immune-Tr1; Homo_714,totalSample,immune-iTreg; Homo_714,totalSample,immune-Central_memory; Homo_714,totalSample,immune-Effector_memory; Homo_714,totalSample,immune-NKT; Homo_714,totalSample,immune-Th2; Homo_714,totalSample,immune-nTreg; Homo_714,totalSample,immune-InfiltrationScore; Homo_714,totalSample,immune-Th1; Homo_714,totalSample,immune-Neutrophil; Homo_633,totalSample,immune-CD4_T; Homo_633,totalSample,immune-CD4_naive; Homo_633,totalSample,immune-Tfh; Homo_633,totalSample,immune-Tr1; Homo_633,totalSample,immune-Effector_memory; Homo_633,totalSample,immune-Central_memory; Homo_633,totalSample,immune-NKT; Homo_633,totalSample,immune-iTreg; Homo_633,totalSample,immune-nTreg; Homo_633,totalSample,immune-InfiltrationScore; Homo_633,totalSample,immune-Th1; Homo_633,totalSample,immune-Th2; Homo_633,totalSample,immune-DC; Homo_633,totalSample,immune-MAIT; Homo_633,totalSample,immune-Neutrophil; MCSA,totalSample,immune-DC; MCSA,totalSample,immune-Bcell; MCSA,totalSample,immune-Macrophage; MCSA,totalSample,immune-CD4_T; MCSA,totalSample,immune-Gamma_delta; MCSA,totalSample,immune-CD4_naive; MCSA,totalSample,immune-Tr1; MCSA,totalSample,immune-nTreg; MCSA,totalSample,immune-iTreg; MCSA,totalSample,immune-Th17; MCSA,totalSample,immune-Tfh; MCSA,totalSample,immune-CD8_naive; MCSA,totalSample,immune-Cytotoxic; MCSA,totalSample,immune-Exhausted; MCSA,totalSample,immune-MAIT; MCSA,totalSample,immune-Central_memory; ROSMAP,totalSample,immune-InfiltrationScore; SRP223445,totalSample,immune-InfiltrationScore; ROSMAP,male- [illegible]
EPS8L1	ACOM,female,AD.vs.control; ACOM,AD,male.vs.female; ROSMAP,male,AD.vs.control; ROSMAP,male,AD.vs.MCI; ROSMAP,AD,male.vs.female; ROSMAP,MCI,male.vs.female; Homo_723,totalSample,immune-DC; Homo_723,totalSample,immune-Cytotoxic; Homo_723,totalSample,immune-InfiltrationScore; Homo_714,totalSample,immune-DC; Homo_714,totalSample,immune-InfiltrationScore; Homo_633,totalSample,immune-DC; Homo_633,totalSample,immune-InfiltrationScore; MCSA,totalSample,immune-DC; MCSA,totalSample,immune-Monocyte; MCSA,totalSample,immune-NK; MCSA,totalSample,immune-Neutrophil; MCSA,totalSample,immune-CD8_T; MCSA,totalSample,immune-Th2; MCSA,totalSample,immune-Cytotoxic; MCSA,totalSample,immune-Effector_memory; MCSA,totalSample,immune-InfiltrationScore
RGPD2	ACOM,female,AD.vs.control; Homo_723,female,AD.vs.control; Homo_723,male,AD.vs.control; Homo_723,male,AD.vs.MCI; Homo_723,male,MCI.vs.control; Homo_723,AD,male.vs.female; Homo_723,control,male.vs.female; Homo_714,female,AD.vs.control; Homo_714,male,AD.vs.control; Homo_714,male,AD.vs.MCI; Homo_714,male,MCI.vs.control; Homo_714,AD,male.vs.female; Homo_714,control,male.vs.female; Homo_633,male,MCI.vs.control; Homo_633,control,male.vs.female; ROSMAP,female,AD.vs.control; ACOM,totalSample,age; Emory,MCI,age; Emory,totalSample,immune-iTreg; Homo_633,totalSample,immune-NKT; MCSA,totalSample,immune-DC; MCSA,totalSample,immune-CD4_T; MCSA,totalSample,immune-Tr1; MCSA,totalSample,immune-iTreg; MCSA,totalSample,immune-MAIT; ROSMAP,totalSample,immune-Effector_memory
SPACA6	ACOM,female,AD.vs.control; ROSMAP,female,AD.vs.control; Emory,MCI,age; Emory,totalSample,immune-Monocyte; Emory,totalSample,immune-Neutrophil; Emory,totalSample,immune-CD8_T; Emory,totalSample,immune-Gamma_delta; Emory,totalSample,immune-CD8_naive; Emory,totalSample,immune-Exhausted; Emory,totalSample,immune-MAIT; Homo_723,totalSample,immune-Neutrophil; Homo_714,totalSample,immune-Neutrophil; Homo_633,totalSample,immune-Neutrophil; Homo_633,totalSample,immune-Exhausted; MCSA,totalSample,immune-Exhausted; MCSA,totalSample,immune-DC; MCSA,totalSample,immune-Monocyte; MCSA,totalSample,immune-Th1; MCSA,totalSample,immune-Th2; SRP223445,totalSample,immune-CD4_naive
RGPD1	ACOM,female,AD.vs.control; Homo_723,female,AD.vs.control; Homo_723,male,AD.vs.control; Homo_723,female,AD.vs.MCI; Homo_723,male,AD.vs.MCI; Homo_723,male,MCI.vs.control; Homo_723,AD,male.vs.female; Homo_723,control,male.vs.female; Homo_714,female,AD.vs.control; Homo_714,male,AD.vs.control; Homo_714,female,AD.vs.MCI; Homo_714,male,AD.vs.MCI; Homo_714,male,MCI.vs.control; Homo_714,AD,male.vs.female; Homo_714,control,male.vs.female; Homo_633,female,AD.vs.control; Homo_633,female,AD.vs.MCI; Homo_633,male,MCI.vs.control; Homo_633,control,male.vs.female; ACOM,totalSample,age; Emory,MCI,age; Emory,totalSample,immune-iTreg; Homo_633,totalSample,immune-NKT; MCSA,totalSample,immune-DC; MCSA,totalSample,immune-CD4_T; MCSA,totalSample,immune-Tr1; MCSA,totalSample,immune-iTreg; MCSA,totalSample,immune-MAIT; ROSMAP,totalSample,immune-Effector_memory
WNT9B	ACOM,female,AD.vs.control; Homo_723,female,AD.vs.control; Homo_723,male,AD.vs.control; Homo_723,female,AD.vs.MCI; Homo_723,male,AD.vs.MCI; Homo_714,female,AD.vs.control; Homo_714,male,AD.vs.control; Homo_714,male,AD.vs.MCI; Homo_714,female,MCI.vs.control; Homo_633,female,MCI.vs.control; ACOM,totalSample,age; Emory,totalSample,immune-CD8_T; Homo_723,totalSample,immune-DC; Homo_714,totalSample,immune-InfiltrationScore; Homo_633,totalSample,immune-InfiltrationScore; MCSA,totalSample,immune-DC; MCSA,totalSample,immune-InfiltrationScore; SRP223445,totalSample,immune-Neutrophil; SRP223445,totalSample,immune-InfiltrationScore
ETFB	ACOM,female,AD.vs.control; ACOM,male,AD.vs.control; Homo_633,male,MCI.vs.control; ACOM,totalSample,age; MCSA,MCI,age; Emory,totalSample,immune-Monocyte; Emory,totalSample,immune-Macrophage; Emory,totalSample,immune-NK; Emory,totalSample,immune-Neutrophil; Emory,totalSample,immune-CD4_T; Emory,totalSample,immune-CD8_T; Emory,totalSample,immune-Tr1; Emory,totalSample,immune-iTreg; Emory,totalSample,immune-Th1; Emory,totalSample,immune-Tfh; Emory,totalSample,immune-Exhausted; Emory,totalSample,immune-InfiltrationScore; Homo_723,totalSample,immune-DC; Homo_723,totalSample,immune-Cytotoxic; Homo_723,totalSample,immune-CD8_T; Homo_723,totalSample,immune-Neutrophil; Homo_723,totalSample,immune-Gamma_delta; Homo_723,totalSample,immune-NKT; Homo_723,totalSample,immune-Exhausted; Homo_723,totalSample,immune-Monocyte; Homo_714,totalSample,immune-DC; Homo_714,totalSample,immune-CD8_T; Homo_714,totalSample,immune-Cytotoxic; Homo_714,totalSample,immune-Neutrophil; Homo_714,totalSample,immune-Exhausted; Homo_714,totalSample,immune-Gamma_delta; Homo_714,totalSample,immune-Monocyte; Homo_714,totalSample,immune-Th17; Homo_714,totalSample,immune-Th2; Homo_714,totalSample,immune-NKT; Homo_633,totalSample,immune-DC; Homo_633,totalSample,immune-CD8_T; Homo_633,totalSample,immune-Cytotoxic; Homo_633,totalSample,immune-NKT; Homo_633,totalSample,immune-Th2; Homo_633,totalSample,immune-Neutrophil; Homo_633,totalSample,immune-Th17; Homo_633,totalSample,immune-Monocyte; Homo_633,totalSample,immune-Gamma_delta; MCSA,totalSample,immune-DC; MCSA,totalSample,immune-Monocyte; MCSA,totalSample,immune-Macrophage; MCSA,totalSample,immune-Neutrophil; MCSA,totalSample,immune-CD8_T; MCSA,totalSample,immune-NKT; MCSA,totalSample,immune-Tr1; MCSA,totalSample,immune-iTreg; MCSA,totalSample,immune-Th2; MCSA,totalSample,immune-Th17; MCSA,totalSample,immune-Cytotoxic; MCSA,totalSample,immune-Exhausted; MCSA,totalSample,immune-Effector_memory; MCSA,totalSample,immune-InfiltrationScore; ROSMAP,totalSample,immune-Effector_memory; SRP223445,totalSample,immune-NK; SRP223445,totalSample,immune-CD4_naive; SRP223445,totalSample,immune-Th17; SRP223445,totalSample,immune-InfiltrationScore
APOC2	ACOM,female,AD.vs.control; ACOM,totalSample,age; SRP223445,totalSample,immune-InfiltrationScore
DBX1	ACOM,female,AD.vs.control; Homo_723,female,AD.vs.control; ACOM,AD,age; Homo_723,totalSample,immune-DC; Homo_723,totalSample,immune-Neutrophil; SRP223445,totalSample,immune-NK
SERPINC1	ACOM,female,AD.vs.control; Homo_723,female,AD.vs.MCI; Homo_714,female,AD.vs.MCI; Homo_714,male,AD.vs.MCI; Homo_633,female,AD.vs.control; ROSMAP,female,AD.vs.control; ROSMAP,male,AD.vs.control; Homo_723,totalSample,immune-Monocyte; Homo_714,totalSample,immune-Monocyte; Homo_633,totalSample,immune-Monocyte;
FEZF2	ACOM,female,AD.vs.control; Homo_723,female,AD.vs.control; Homo_723,female,AD.vs.MCI; ROSMAP,female,AD.vs.control; ROSMAP,AD,sample.age; Homo_723,totalSample,immune-DC
VSIG10L	ACOM,female,AD.vs.control; Homo_723,female,AD.vs.control; Homo_723,male,AD.vs.control; Homo_723,female,AD.vs.MCI; Homo_723,male,AD.vs.MCI; Homo_723,female,MCI.vs.control; Homo_723,male,MCI.vs.control; Homo_714,female,AD.vs.control; Homo_714,male,AD.vs.control; Homo_714,female,AD.vs.MCI; Homo_714,female,MCI.vs.control; Homo_714,male,MCI.vs.control; Homo_633,female,AD.vs.control; Homo_633,female,AD.vs.MCI; Homo_633,female,MCI.vs.control; Homo_633,male,MCI.vs.control; ROSMAP,female,AD.vs.control; ROSMAP,male,AD.vs.control; ROSMAP,female,AD.vs.MCI; Emory,totalSample,immune-DC; Emory,totalSample,immune-InfiltrationScore; Homo_723,totalSample,immune-DC; Homo_723,totalSample,immune-Monocyte; Homo_714,totalSample,immune-DC; Homo_714,totalSample,immune-Monocyte; Homo_633,totalSample,immune-Monocyte; MCSA,totalSample,immune-Monocyte; ROSMAP,female-excludeAge90+,survival; ROSMAP,female-includeAge90+,survival; ROSMAP,male-excludeAge90+,survival; ROSMAP,male and female-
TNXB	ACOM,female,AD.vs.control; Homo_723,female,AD.vs.control; Homo_723,male,AD.vs.control; Homo_723,female,AD.vs.MCI; Homo_723,male,AD.vs.MCI; Homo_714,female,AD.vs.control; Homo_714,male,AD.vs.control; Homo_714,female,AD.vs.MCI; Homo_714,male,AD.vs.MCI; Homo_633,female,AD.vs.control; Homo_633,female,AD.vs.MCI; Homo_633,female,MCI.vs.control; Homo_633,male,MCI.vs.control; ROSMAP,female,AD.vs.control; ROSMAP,male,AD.vs.control; ROSMAP,male,MCI.vs.control; ACOM,AD,age; MCSA,MCI,age; Homo_723,totalSample,immune-DC; Homo_723,totalSample,immune-Monocyte; Homo_723,totalSample,immune-Th2; Homo_723,totalSample,immune-Neutrophil; Homo_714,totalSample,immune-Th2; Homo_714,totalSample,immune-DC; Homo_633,totalSample,immune-Th2; Homo_633,totalSample,immune-DC; Homo_633,totalSample,immune-Monocyte; MCSA,totalSample,immune-DC; MCSA,totalSample,immune-Monocyte; MCSA,totalSample,immune-CD8_T; MCSA,totalSample,immune-Th2; MCSA,totalSample,immune-
POU3F4	ACOM,female,AD.vs.control; ACOM,AD,male.vs.female; Homo_723,male,AD.vs.control; Homo_723,female,AD.vs.MCI; Homo_723,female,MCI.vs.control; Homo_723,male,MCI.vs.control; Homo_723,control,male.vs.female; Homo_714,female,AD.vs.control; Homo_714,male,AD.vs.control; Homo_714,female,MCI.vs.control; Homo_714,male,MCI.vs.control; Homo_714,control,male.vs.female; Homo_633,female,MCI.vs.control; Homo_633,male,MCI.vs.control; Emory,totalSample,immune-Neutrophil; Emory,totalSample,immune-CD8_T; Emory,totalSample,immune-Gamma_delta
OPN5	ACOM,female,AD.vs.control; Homo_714,female,AD.vs.control; ROSMAP,female,AD.vs.control; ROSMAP,control,male.vs.female; ROSMAP,AD,last.visit.age; Emory,totalSample,immune-Gamma_delta; Emory,totalSample,immune-Exhausted; ROSMAP,totalSample,immune-InfiltrationScore
MSANTD3-TMEFF1	ACOM,female,AD.vs.control; ROSMAP,female,AD.vs.control; ROSMAP,male,AD.vs.control; ROSMAP,female,MCI.vs.control; ROSMAP,MCI,last.visit.age; ROSMAP,totalSample,immune-InfiltrationScore; SRP223445,totalSample,immune-CD4_naive; SRP223445,totalSample,immune-Exhausted; ROSMAP,male and female-excludeAge90+,survival
TYR	ACOM,female,AD.vs.control; ACOM,AD,male.vs.female; Homo_723,female,AD.vs.control; ROSMAP,female,AD.vs.control; Homo_723,totalSample,immune-Th17; Homo_723,totalSample,immune-DC; ROSMAP,totalSample,immune-Central_memory; ROSMAP,totalSample,immune-InfiltrationScore
C8B	ACOM,female,AD.vs.control; ROSMAP,female,AD.vs.control; ROSMAP,female,MCI.vs.control; ROSMAP,control,male.vs.female; ACOM,totalSample,age; ROSMAP,MCI,last.visit.age; ROSMAP,totalSample,immune-InfiltrationScore
SIGLEC10	ACOM,female,AD.vs.control; Emory,MCI,age; Emory,totalSample,immune-Neutrophil; Emory,totalSample,immune-CD8_T; Emory,totalSample,immune-NKT; Emory,totalSample,immune-Gamma_delta; Emory,totalSample,immune-Th1; Emory,totalSample,immune-Tfh; Emory,totalSample,immune-CD8_naive; Emory,totalSample,immune-Exhausted; Homo_723,totalSample,immune-Neutrophil; Homo_723,totalSample,immune-Exhausted; Homo_723,totalSample,immune-Gamma_delta; Homo_723,totalSample,immune-Th1; Homo_714,totalSample,immune-Neutrophil; Homo_714,totalSample,immune-Exhausted; Homo_714,totalSample,immune-Gamma_delta; Homo_714,totalSample,immune-Th1; Homo_633,totalSample,immune-Exhausted; Homo_633,totalSample,immune-Neutrophil; Homo_633,totalSample,immune-MAIT; Homo_633,totalSample,immune-CD8_T; Homo_633,totalSample,immune-Gamma_delta; Homo_633,totalSample,immune-Macrophage; MCSA,totalSample,immune-DC; MCSA,totalSample,immune-Monocyte; MCSA,totalSample,immune-Macrophage; MCSA,totalSample,immune-Gamma_delta; MCSA,totalSample,immune-Th1; MCSA,totalSample,immune-Th2; MCSA,totalSample,immune-Th17; MCSA,totalSample,immune-Exhausted; ROSMAP,totalSample,immune-Effector_memory
ZNF283	ACOM,female,AD.vs.control; MCSA,MCI,age; ROSMAP,AD,last.visit.age; Emory,totalSample,immune-Macrophage; Emory,totalSample,immune-CD4_T; Emory,totalSample,immune-Tr1; Emory,totalSample,immune-iTreg; Emory,totalSample,immune-Tfh; Emory,totalSample,immune-Central_memory; Emory,totalSample,immune-InfiltrationScore; Homo_723,totalSample,immune-Tr1; Homo_723,totalSample,immune-CD4_naive; Homo_723,totalSample,immune-iTreg; Homo_723,totalSample,immune-CD4_T; Homo_723,totalSample,immune-Tfh; Homo_723,totalSample,immune-Central_memory; Homo_723,totalSample,immune-Th2; Homo_723,totalSample,immune-Th1; Homo_723,totalSample,immune-nTreg; Homo_723,totalSample,immune-Cytotoxic; Homo_723,totalSample,immune-NKT; Homo_723,totalSample,immune-Effector_memory; Homo_723,totalSample,immune-InfiltrationScore; Homo_723,totalSample,immune-DC; Homo_714,totalSample,immune-CD4_naive; Homo_714,totalSample,immune-CD4_T; Homo_714,totalSample,immune-Tfh; Homo_714,totalSample,immune-Tr1; Homo_714,totalSample,immune-iTreg; Homo_714,totalSample,immune-NKT; Homo_714,totalSample,immune-Central_memory; Homo_714,totalSample,immune-Th2; Homo_714,totalSample,immune-Effector_memory; Homo_714,totalSample,immune-Th1; Homo_714,totalSample,immune-DC; Homo_714,totalSample,immune-nTreg; Homo_714,totalSample,immune-InfiltrationScore; Homo_714,totalSample,immune-Neutrophil; Homo_633,totalSample,immune-CD4_naive; Homo_633,totalSample,immune-CD4_T; Homo_633,totalSample,immune-Tfh; Homo_633,totalSample,immune-NKT; Homo_633,totalSample,immune-Tr1; Homo_633,totalSample,immune-Effector_memory; Homo_633,totalSample,immune-Central_memory; Homo_633,totalSample,immune-iTreg; Homo_633,totalSample,immune-DC; Homo_633,totalSample,immune-Th1; Homo_633,totalSample,immune-nTreg; Homo_633,totalSample,immune-Th2; Homo_633,totalSample,immune-InfiltrationScore; Homo_633,totalSample,immune-MAIT; Homo_633,totalSample,immune-Gamma_delta; Homo_633,totalSample,immune-Cytotoxic; MCSA,totalSample,immune-DC; MCSA,totalSample,immune-Monocyte; MCSA,totalSample,immune-Macrophage; MCSA,totalSample,immune-CD4_T; MCSA,totalSample,immune-CD8_T; MCSA,totalSample,immune-CD4_naive; MCSA,totalSample,immune-Tr1; MCSA,totalSample,immune-nTreg; MCSA,totalSample,immune-iTreg; MCSA,totalSample,immune-Th17; MCSA,totalSample,immune-Tfh; MCSA,totalSample,immune-CD8_naive; MCSA,totalSample,immune-Cytotoxic; MCSA,totalSample,immune-Exhausted; MCSA,totalSample,immune-MAIT; MCSA,totalSample,immune-Central_memory; ROSMAP,totalSample,immune-InfiltrationScore; [illegible]
ERICH1	ACOM,female,AD.vs.control; Homo_723,female,AD.vs.control; Homo_723,male,AD.vs.control; Homo_723,female,AD.vs.MCI; Homo_723,male,AD.vs.MCI; Homo_723,male,MCI.vs.control; Homo_714,female,AD.vs.control; Homo_714,male,AD.vs.control; Homo_714,female,AD.vs.MCI; Homo_714,male,AD.vs.MCI; Homo_714,female,MCI.vs.control; Homo_714,male,MCI.vs.control; Homo_633,male,MCI.vs.control; Emory,totalSample,age; Emory,MCI,age; Homo_723,totalSample,immune-Monocyte; Homo_723,totalSample,immune-DC; Homo_714,totalSample,immune-Monocyte; Homo_633,totalSample,immune-Monocyte; Homo_633,totalSample,immune-InfiltrationScore; MCSA,totalSample,immune-CD4_T; MCSA,totalSample,immune-CD4_naive; MCSA,totalSample,immune-Tr1; MCSA,totalSample,immune-nTreg; MCSA,totalSample,immune-iTreg; MCSA,totalSample,immune-Th17; MCSA,totalSample,immune-Tfh; MCSA,totalSample,immune-Central_memory; MCSA,totalSample,immune-Effector_memory; ROSMAP,totalSample,immune-Effector_memory
CLDND2	ACOM,female,AD.vs.control; ROSMAP,female,AD.vs.control; Emory,totalSample,immune-Neutrophil; Emory,totalSample,immune-Gamma_delta; Emory,totalSample,immune-Cytotoxic; Emory,totalSample,immune-Exhausted; Emory,totalSample,immune-Central_memory; Emory,totalSample,immune-Effector_memory; Homo_723,totalSample,immune-Cytotoxic; Homo_723,totalSample,immune-Gamma_delta; Homo_723,totalSample,immune-InfiltrationScore; Homo_723,totalSample,immune-DC; Homo_723,totalSample,immune-Neutrophil; Homo_723,totalSample,immune-CD8_T; Homo_723,totalSample,immune-Monocyte; Homo_723,totalSample,immune-NK; Homo_723,totalSample,immune-Exhausted; Homo_723,totalSample,immune-Effector_memory; Homo_723,totalSample,immune-Th1; Homo_714,totalSample,immune-Cytotoxic; Homo_714,totalSample,immune-InfiltrationScore; Homo_714,totalSample,immune-Gamma_delta; Homo_714,totalSample,immune-CD8_T; Homo_714,totalSample,immune-Neutrophil; Homo_714,totalSample,immune-Exhausted; Homo_714,totalSample,immune-DC; Homo_714,totalSample,immune-Th2; Homo_714,totalSample,immune-Macrophage; Homo_714,totalSample,immune-MAIT; Homo_714,totalSample,immune-Effector_memory; Homo_714,totalSample,immune-Th1; Homo_714,totalSample,immune-Th17; Homo_714,totalSample,immune-Monocyte; Homo_714,totalSample,immune-NK; Homo_633,totalSample,immune-Cytotoxic; Homo_633,totalSample,immune-InfiltrationScore; Homo_633,totalSample,immune-Gamma_delta; Homo_633,totalSample,immune-CD8_T; Homo_633,totalSample,immune-Neutrophil; Homo_633,totalSample,immune-Macrophage; Homo_633,totalSample,immune-DC; Homo_633,totalSample,immune-Th2; Homo_633,totalSample,immune-Th1; Homo_633,totalSample,immune-NKT; Homo_633,totalSample,immune-Th17; Homo_633,totalSample,immune-Exhausted; Homo_633,totalSample,immune-MAIT; Homo_633,totalSample,immune-Effector_memory; Homo_633,totalSample,immune-NK; Homo_633,totalSample,immune-Monocyte; Homo_633,totalSample,immune-Bcell; MCSA,totalSample,immune-DC; MCSA,totalSample,immune-Monocyte; MCSA,totalSample,immune-Neutrophil; MCSA,totalSample,immune-CD8_T; MCSA,totalSample,immune-NKT; MCSA,totalSample,immune-Gamma_delta; MCSA,totalSample,immune-iTreg; MCSA,totalSample,immune-Th2; MCSA,totalSample,immune-Cytotoxic; MCSA,totalSample,immune- [illegible]
LIM2	ACOM,female,AD.vs.control; Homo_714,female,AD.vs.control; Homo_714,male,AD.vs.control; Homo_714,female,AD.vs.MCI; Homo_633,female,AD.vs.control; Homo_633,female,AD.vs.MCI; ROSMAP,AD,sample.age; ROSMAP,MCI,sample.age; Emory,totalSample,immune-NK; Emory,totalSample,immune-Effector_memory; Homo_723,totalSample,immune-InfiltrationScore; Homo_723,totalSample,immune-Effector_memory; Homo_723,totalSample,immune-NK; Homo_723,totalSample,immune-Cytotoxic; Homo_714,totalSample,immune-InfiltrationScore; Homo_714,totalSample,immune-NK; Homo_714,totalSample,immune-Effector_memory; Homo_633,totalSample,immune-NK; Homo_633,totalSample,immune-InfiltrationScore; Homo_633,totalSample,immune-Effector_memory; Homo_633,totalSample,immune-DC; Homo_633,totalSample,immune-Neutrophil; MCSA,totalSample,immune-DC; MCSA,totalSample,immune-Monocyte; MCSA,totalSample,immune-NK; MCSA,totalSample,immune-Neutrophil; MCSA,totalSample,immune-CD8_T; MCSA,totalSample,immune-NKT; MCSA,totalSample,immune-Cytotoxic; MCSA,totalSample,immune-Effector_memory; MCSA,totalSample,immune-InfiltrationScore
NBL1	ACOM,female,AD.vs.control; Homo_633,female,AD.vs.control; Homo_633,female,AD.vs.MCI; ROSMAP,male,AD.vs.control; Emory,control,age; Emory,MCI,age; Emory,totalSample,immune-CD4_T; Emory,totalSample,immune-CD8_T; Emory,totalSample,immune-Tr1; Emory,totalSample,immune-nTreg; Emory,totalSample,immune-iTreg; Emory,totalSample,immune-Th17; Emory,totalSample,immune-Tfh; Emory,totalSample,immune-CD8_naive; Homo_723,totalSample,immune-Monocyte; Homo_723,totalSample,immune-DC; Homo_723,totalSample,immune-Neutrophil; Homo_723,totalSample,immune-Central_memory; Homo_714,totalSample,immune-Monocyte; Homo_633,totalSample,immune-Monocyte; MCSA,totalSample,immune-Monocyte; MCSA,totalSample,immune-CD8_T; MCSA,totalSample,immune-Cytotoxic; MCSA,totalSample,immune-Effector_memory; ROSMAP,totalSample,immune-Effector_memory; SRP223445,totalSample,immune-Neutrophil; SRP223445,totalSample,immune-InfiltrationScore; ROSMAP,female-excludeAge90+,survival; ROSMAP,male and female-
CYP2A6	ACOM,female,AD.vs.control; Homo_723,female,AD.vs.control; Homo_723,male,AD.vs.control; Homo_723,female,AD.vs.MCI; Homo_723,male,AD.vs.MCI; Homo_714,female,AD.vs.control; Homo_714,male,AD.vs.control; Homo_714,female,AD.vs.MCI; Homo_714,male,AD.vs.MCI; ROSMAP,female,AD.vs.control; ROSMAP,male,AD.vs.control; ROSMAP,male-

KCNJ12	ACOM,female,AD.vs.control; Homo_723,female,AD.vs.control; Homo_723,male,AD.vs.control; Homo_723,female,AD.vs.MCI; Homo_723,male,AD.vs.MCI; Homo_714,female,AD.vs.control; Homo_714,male,AD.vs.control; Homo_714,female,AD.vs.MCI; Homo_714,male,AD.vs.MCI; Homo_633,female,MCI.vs.control; Homo_633,male,MCI.vs.control; ROSMAP,female,AD.vs.control; ROSMAP,female,MCI.vs.control; ACOM,totalSample,age; MCSA,MCI,age; ROSMAP,AD,last.visit.age; Emory,totalSample,immune-MAIT; Homo_723,totalSample,immune-DC; Homo_714,totalSample,immune-Th2; SRP223445,totalSample,immune-Neutrophil; SRP223445,totalSample,immune-InfiltrationScore; ROSMAP,male-
NKX1-2	ACOM,female,AD.vs.control; Homo_723,totalSample,immune-CD8_naive
GREM2	ACOM,female,AD.vs.control; ACOM,AD,male.vs.female; Homo_723,female,AD.vs.control; Homo_723,male,AD.vs.control; Homo_723,female,AD.vs.MCI; Homo_723,male,AD.vs.MCI; Homo_714,female,AD.vs.control; Homo_714,male,AD.vs.control; Homo_714,female,AD.vs.MCI; Homo_714,male,AD.vs.MCI; Homo_633,female,AD.vs.control; Homo_633,female,AD.vs.MCI; Emory,totalSample,immune-CD4_T; Emory,totalSample,immune-Gamma_delta; Emory,totalSample,immune-Tr1; Emory,totalSample,immune-nTreg; Emory,totalSample,immune-iTreg; Emory,totalSample,immune-Th17; Emory,totalSample,immune-Cytotoxic; Homo_723,totalSample,immune-CD4_T; Homo_723,totalSample,immune-Th2; Homo_723,totalSample,immune-iTreg; Homo_723,totalSample,immune-Tfh; Homo_723,totalSample,immune-Tr1; Homo_723,totalSample,immune-nTreg; Homo_723,totalSample,immune-Central_memory; Homo_723,totalSample,immune-CD4_naive; Homo_714,totalSample,immune-Tfh; Homo_714,totalSample,immune-Tr1; Homo_714,totalSample,immune-CD4_T; Homo_714,totalSample,immune-iTreg; Homo_714,totalSample,immune-Th2; Homo_714,totalSample,immune-nTreg; Homo_633,totalSample,immune-Tfh; Homo_633,totalSample,immune-CD4_T; Homo_633,totalSample,immune-Tr1; Homo_633,totalSample,immune-Th17; Homo_633,totalSample,immune-nTreg; Homo_633,totalSample,immune-Th2; Homo_633,totalSample,immune-Gamma_delta; Homo_633,totalSample,immune-iTreg; Homo_633,totalSample,immune-Th1; Homo_633,totalSample,immune-CD4_naive; MCSA,totalSample,immune-CD4_T; MCSA,totalSample,immune-CD4_naive; MCSA,totalSample,immune-Tr1; MCSA,totalSample,immune-nTreg; MCSA,totalSample,immune-iTreg;
SCUBE2	ACOM,female,AD.vs.control; Homo_723,female,AD.vs.control; Homo_723,male,AD.vs.control; Homo_723,female,AD.vs.MCI; Homo_723,male,AD.vs.MCI; Homo_723,female,MCI.vs.control; Homo_723,male,MCI.vs.control; Homo_714,male,AD.vs.MCI; Homo_714,female,MCI.vs.control; Homo_714,male,MCI.vs.control; Homo_633,female,AD.vs.control; Homo_633,male,MCI.vs.control; ROSMAP,female,AD.vs.control; ROSMAP,male,AD.vs.control; ROSMAP,female,AD.vs.MCI; ROSMAP,male,MCI.vs.control; ROSMAP,MCI,male.vs.female; Emory,control,age; Homo_723,totalSample,immune-Exhausted; Homo_723,totalSample,immune-Monocyte; Homo_723,totalSample,immune-Th17; Homo_714,totalSample,immune-Monocyte; Homo_633,totalSample,immune-Monocyte; Homo_633,totalSample,immune-Exhausted; Homo_633,totalSample,immune-Th2; SRP223445,totalSample,immune-Neutrophil
IAPP	ACOM,female,AD.vs.control; Homo_723,female,AD.vs.control; Emory,totalSample,immune-Th17; Homo_723,totalSample,immune-Th17
TAFA4	ACOM,female,AD.vs.control; ROSMAP,female,AD.vs.control; ROSMAP,totalSample,immune-InfiltrationScore; ROSMAP,male and female-excludeAge90+,survival
ATP5MF-PTCD1	ACOM,female,AD.vs.control; ACOM,AD,male.vs.female; Homo_633,female,AD.vs.control; Homo_633,female,AD.vs.MCI; MCSA,male,MCI.vs.control; Homo_723,totalSample,immune-Th2; Homo_723,totalSample,immune-DC; Homo_714,totalSample,immune-Th2; ROSMAP,totalSample,immune-MAIT; ROSMAP,totalSample,immune-Effector_memory
URGCP-MRPS24	ACOM,female,AD.vs.control; Homo_723,female,AD.vs.MCI; Homo_723,female,MCI.vs.control; Homo_723,control,male.vs.female; Homo_714,female,AD.vs.MCI; Homo_714,female,MCI.vs.control; Homo_714,control,male.vs.female; Homo_633,female,AD.vs.control; Homo_633,female,AD.vs.MCI; Homo_633,female,MCI.vs.control; Homo_633,control,male.vs.female; MCSA,control,male.vs.female; ROSMAP,female,AD.vs.control
ZNF558	ACOM,female,AD.vs.control; ACOM,male,AD.vs.control; Homo_723,male,AD.vs.control; Homo_714,female,AD.vs.control; Homo_714,male,AD.vs.control; Homo_714,female,AD.vs.MCI; Homo_714,male,AD.vs.MCI; Homo_633,female,AD.vs.control; Homo_633,female,AD.vs.MCI; MCSA,MCI,age; Emory,totalSample,immune-Macrophage; Emory,totalSample,immune-Neutrophil; Emory,totalSample,immune-CD4_T; Emory,totalSample,immune-Tr1; Emory,totalSample,immune-iTreg; Emory,totalSample,immune-Tfh; Emory,totalSample,immune-Central_memory; Emory,totalSample,immune-InfiltrationScore; Homo_723,totalSample,immune-Th2; Homo_723,totalSample,immune-Tfh; Homo_723,totalSample,immune-iTreg; Homo_723,totalSample,immune-CD4_T; Homo_723,totalSample,immune-Tr1; Homo_723,totalSample,immune-CD4_naive; Homo_723,totalSample,immune-Effector_memory; Homo_723,totalSample,immune-DC; Homo_723,totalSample,immune-InfiltrationScore; Homo_723,totalSample,immune-Central_memory; Homo_723,totalSample,immune-nTreg; Homo_723,totalSample,immune-Macrophage; Homo_723,totalSample,immune-Monocyte; Homo_723,totalSample,immune-MAIT; Homo_714,totalSample,immune-Tfh; Homo_714,totalSample,immune-Th2; Homo_714,totalSample,immune-CD4_T; Homo_714,totalSample,immune-Effector_memory; Homo_714,totalSample,immune-Tr1; Homo_714,totalSample,immune-CD4_naive; Homo_714,totalSample,immune-Central_memory; Homo_714,totalSample,immune-iTreg; Homo_714,totalSample,immune-InfiltrationScore; Homo_714,totalSample,immune-nTreg; Homo_714,totalSample,immune-DC; Homo_714,totalSample,immune-Monocyte; Homo_633,totalSample,immune-Tfh; Homo_633,totalSample,immune-CD4_T; Homo_633,totalSample,immune-Th2; Homo_633,totalSample,immune-Effector_memory; Homo_633,totalSample,immune-CD4_naive; Homo_633,totalSample,immune-Central_memory; Homo_633,totalSample,immune-Tr1; Homo_633,totalSample,immune-InfiltrationScore; Homo_633,totalSample,immune-Monocyte; Homo_633,totalSample,immune-iTreg; Homo_633,totalSample,immune-Th17; Homo_633,totalSample,immune-nTreg; Homo_633,totalSample,immune-Exhausted; Homo_633,totalSample,immune-DC; Homo_633,totalSample,immune-Gamma_delta; Homo_633,totalSample,immune-NKT; Homo_633,totalSample,immune-Bcell; MCSA,totalSample,immune-DC; MCSA,totalSample,immune-Bcell; MCSA,totalSample,immune-Macrophage; MCSA,totalSample,immune-CD4_T; MCSA,totalSample,immune-CD4_naive; MCSA,totalSample,immune-Tr1; MCSA,totalSample,immune-nTreg; MCSA,totalSample,immune-iTreg; MCSA,totalSample,immune-Th17; MCSA,totalSample,immune-Tfh; MCSA,totalSample,immune-CD8_naive; MCSA,totalSample,immune-Cytotoxic; MCSA,totalSample,immune-Exhausted; MCSA,totalSample,immune-MAIT; MCSA,totalSample,immune-Central_memory; ROSMAP,totalSample,immune-InfiltrationScore; SRP223445,totalSample,immune-CD4_naive; SRP223445,totalSample,immune-InfiltrationScore; ROSMAP,male-includeAge90+,survival
RBP4	ACOM,female,AD.vs.control; Homo_723,female,AD.vs.control; Homo_723,female,AD.vs.MCI; Homo_723,female,MCI.vs.control; Homo_714,female,AD.vs.control; Homo_714,female,AD.vs.MCI; Homo_714,female,MCI.vs.control; ACOM,totalSample,age; ROSMAP,MCI,sample.age; Homo_723,totalSample,immune-Exhausted; Homo_723,totalSample,immune-DC; Homo_723,totalSample,immune-CD8_T; Homo_723,totalSample,immune-Th17; Homo_723,totalSample,immune-Macrophage; SRP223445,totalSample,immune-Exhausted
GABRG1	ACOM,female,AD.vs.control; Homo_723,female,AD.vs.control; Homo_723,female,AD.vs.MCI; ROSMAP,female,AD.vs.control; ROSMAP,AD,sample.age; ROSMAP,totalSample,age; ROSMAP,control,sample.age; ROSMAP,MCI,sample.age; ROSMAP,AD,MMSE; ROSMAP,totalSample,MMSE; ROSMAP,totalSample,immune-Monocyte; ROSMAP,totalSample,immune-CD4_T; ROSMAP,totalSample,immune-nTreg; ROSMAP,totalSample,immune-iTreg; ROSMAP,totalSample,immune-Th17; ROSMAP,totalSample,immune-Central_memory; ROSMAP,totalSample,immune-InfiltrationScore
HAS1	ACOM,female,AD.vs.control; Homo_723,male,AD.vs.control; Homo_723,male,AD.vs.MCI; Homo_723,female,MCI.vs.control; Homo_723,male,MCI.vs.control; Homo_714,male,AD.vs.control; Homo_714,female,AD.vs.MCI; Homo_714,male,AD.vs.MCI; Homo_714,female,MCI.vs.control; Homo_714,male,MCI.vs.control; Homo_633,female,AD.vs.control; Homo_633,female,AD.vs.MCI; Homo_633,female,MCI.vs.control; Homo_633,male,MCI.vs.control; ROSMAP,male,AD.vs.control; ROSMAP,male,AD.vs.MCI; ROSMAP,female,MCI.vs.control; ROSMAP,male,MCI.vs.control; ROSMAP,AD,male.vs.female; ROSMAP,MCI,male.vs.female; Emory,MCI,age; Emory,totalSample,immune-Neutrophil; Emory,totalSample,immune-CD8_T; Emory,totalSample,immune-Tfh; Emory,totalSample,immune-CD8_naive; Emory,totalSample,immune-Exhausted; Emory,totalSample,immune-MAIT; Homo_723,totalSample,immune-CD8_naive; MCSA,totalSample,immune-Monocyte; MCSA,totalSample,immune-Th2; ROSMAP,totalSample,immune-Effector_memory; ROSMAP,male and female-
SYT2	ACOM,female,AD.vs.control; Homo_723,female,AD.vs.control; Homo_723,male,AD.vs.control; Homo_723,female,MCI.vs.control; Homo_714,female,AD.vs.MCI; Homo_714,male,AD.vs.MCI; Homo_714,female,MCI.vs.control; Homo_714,male,MCI.vs.control; Homo_633,female,AD.vs.control; Homo_633,female,AD.vs.MCI; Homo_633,female,MCI.vs.control; Homo_633,male,MCI.vs.control; ROSMAP,male,AD.vs.control; ROSMAP,female,MCI.vs.control; ROSMAP,male,MCI.vs.control; ROSMAP,AD,male.vs.female; ROSMAP,MCI,male.vs.female; Emory,totalSample,age; Emory,MCI,age; MCSA,totalSample,age; MCSA,control,age; Emory,totalSample,immune-NK; Emory,totalSample,immune-Neutrophil; Emory,totalSample,immune-CD8_T; Emory,totalSample,immune-Gamma_delta; Emory,totalSample,immune-Th17; Emory,totalSample,immune-Tfh; Emory,totalSample,immune-CD8_naive; Emory,totalSample,immune-Cytotoxic; Emory,totalSample,immune-Exhausted; Emory,totalSample,immune-MAIT; Homo_723,totalSample,immune-CD8_naive; Homo_723,totalSample,immune-Monocyte; Homo_714,totalSample,immune-Monocyte; Homo_633,totalSample,immune-Monocyte; Homo_633,totalSample,immune-Neutrophil; MCSA,totalSample,immune-Monocyte; MCSA,totalSample,immune-Neutrophil; MCSA,totalSample,immune-Gamma_delta; MCSA,totalSample,immune-Th1; MCSA,totalSample,immune-Th2; MCSA,totalSample,immune-Th17; MCSA,totalSample,immune-Exhausted; ROSMAP,totalSample,immune-Effector_memory; ROSMAP,totalSample,immune-InfiltrationScore
RAX	ACOM,female,AD.vs.control; Homo_723,female,AD.vs.control; Homo_723,female,AD.vs.MCI; Homo_723,totalSample,immune-DC
ATP6V0D2	ACOM,female,AD.vs.control; ACOM,AD,male.vs.female; ACOM,totalSample,age
KRT24	ACOM,female,AD.vs.control
ZNF572	ACOM,female,AD.vs.control; Homo_723,female,AD.vs.MCI; Homo_723,female,MCI.vs.control; Homo_714,female,MCI.vs.control; Homo_633,female,AD.vs.control; Homo_633,female,AD.vs.MCI; Homo_633,female,MCI.vs.control; MCSA,MCI,age; ROSMAP,AD,last.visit.age; Emory,totalSample,immune-CD4_T; Emory,totalSample,immune-Tr1; Emory,totalSample,immune-iTreg; Emory,totalSample,immune-Effector_memory; Emory,totalSample,immune-InfiltrationScore; Homo_723,totalSample,immune-Tfh; Homo_723,totalSample,immune-Th2; Homo_723,totalSample,immune-CD4_T; Homo_723,totalSample,immune-CD4_naive; Homo_723,totalSample,immune-Tr1; Homo_723,totalSample,immune-iTreg; Homo_723,totalSample,immune-Exhausted; Homo_723,totalSample,immune-Monocyte; Homo_723,totalSample,immune-Effector_memory; Homo_723,totalSample,immune-Central_memory; Homo_723,totalSample,immune-InfiltrationScore; Homo_723,totalSample,immune-DC; Homo_723,totalSample,immune-nTreg; Homo_714,totalSample,immune-Tfh; Homo_714,totalSample,immune-Th2; Homo_714,totalSample,immune-CD4_T; Homo_714,totalSample,immune-CD4_naive; Homo_714,totalSample,immune-Central_memory; Homo_714,totalSample,immune-Tr1; Homo_714,totalSample,immune-Effector_memory; Homo_714,totalSample,immune-Monocyte; Homo_714,totalSample,immune-iTreg; Homo_714,totalSample,immune-InfiltrationScore; Homo_714,totalSample,immune-DC; Homo_714,totalSample,immune-Gamma_delta; Homo_633,totalSample,immune-Tfh; Homo_633,totalSample,immune-Th2; Homo_633,totalSample,immune-CD4_T; Homo_633,totalSample,immune-Central_memory; Homo_633,totalSample,immune-Effector_memory; Homo_633,totalSample,immune-CD4_naive; Homo_633,totalSample,immune-Monocyte; Homo_633,totalSample,immune-Tr1; Homo_633,totalSample,immune-Exhausted; Homo_633,totalSample,immune-Gamma_delta; Homo_633,totalSample,immune-DC; Homo_633,totalSample,immune-iTreg; Homo_633,totalSample,immune-InfiltrationScore; Homo_633,totalSample,immune-NKT; Homo_633,totalSample,immune-Th17; MCSA,totalSample,immune-DC; MCSA,totalSample,immune-Bcell; MCSA,totalSample,immune-Monocyte; MCSA,totalSample,immune-Macrophage; MCSA,totalSample,immune-CD4_T; MCSA,totalSample,immune-CD4_naive; MCSA,totalSample,immune-Tr1; MCSA,totalSample,immune-nTreg; MCSA,totalSample,immune-iTreg; MCSA,totalSample,immune-Th2; MCSA,totalSample,immune-Tfh; MCSA,totalSample,immune-CD8_naive; MCSA,totalSample,immune-Cytotoxic; MCSA,totalSample,immune-Exhausted; MCSA,totalSample,immune-MAIT; MCSA,totalSample,immune-Central_memory; ROSMAP,totalSample,immune-InfiltrationScore; SRP223445,totalSample,immune-Th17
SLC25A27	ACOM,female,AD.vs.control; ACOM,AD,male.vs.female; Homo_723,female,AD.vs.control; Homo_723,male,AD.vs.control; Homo_723,female,AD.vs.MCI; Homo_723,female,MCI.vs.control; Homo_723,male,MCI.vs.control; Homo_714,female,AD.vs.control; Homo_714,male,AD.vs.control; Homo_714,female,MCI.vs.control; Homo_714,male,MCI.vs.control; Homo_633,female,AD.vs.control; Homo_633,female,MCI.vs.control; Homo_633,male,MCI.vs.control; ROSMAP,female,AD.vs.control; ROSMAP,female,MCI.vs.control; Homo_723,totalSample,immune-Th2; Homo_723,totalSample,immune-Monocyte; Homo_723,totalSample,immune-Tfh; Homo_723,totalSample,immune-Exhausted; Homo_723,totalSample,immune-CD4_T; Homo_723,totalSample,immune-iTreg; Homo_723,totalSample,immune-Tr1; Homo_723,totalSample,immune-Gamma_delta; Homo_723,totalSample,immune-DC; Homo_723,totalSample,immune-Th17; Homo_714,totalSample,immune-Th2; Homo_714,totalSample,immune-Tfh; Homo_714,totalSample,immune-Gamma_delta; Homo_714,totalSample,immune-CD4_T; Homo_714,totalSample,immune-Monocyte; Homo_714,totalSample,immune-Tr1; Homo_714,totalSample,immune-Th17; Homo_633,totalSample,immune-Th2; Homo_633,totalSample,immune-Exhausted; Homo_633,totalSample,immune-Monocyte; Homo_633,totalSample,immune-Tfh; Homo_633,totalSample,immune-Gamma_delta; Homo_633,totalSample,immune-Th17; Homo_633,totalSample,immune-CD4_T; MCSA,totalSample,immune-DC; MCSA,totalSample,immune-CD4_T; MCSA,totalSample,immune-Gamma_delta; MCSA,totalSample,immune-CD4_naive; MCSA,totalSample,immune-Tr1; MCSA,totalSample,immune-nTreg; MCSA,totalSample,immune-iTreg; MCSA,totalSample,immune-Th1; MCSA,totalSample,immune-Th17; MCSA,totalSample,immune-Tfh; MCSA,totalSample,immune-CD8_naive; MCSA,totalSample,immune-Cytotoxic; MCSA,totalSample,immune-Exhausted; MCSA,totalSample,immune-Central_memory; ROSMAP,totalSample,immune-CD4_T; ROSMAP,totalSample,immune-Th17; ROSMAP,totalSample,immune-InfiltrationScore
SLC34A2	ACOM,female,AD.vs.control; Homo_723,male,AD.vs.control; Homo_723,female,AD.vs.MCI; Homo_714,male,AD.vs.control; Homo_714,female,AD.vs.MCI; Homo_633,male,MCI.vs.control; ROSMAP,totalSample,immune-InfiltrationScore
TF	ACOM,female,AD.vs.control; ACOM,male,AD.vs.control; Homo_633,female,AD.vs.MCI; MCSA,MCI,age; Homo_723,totalSample,immune-InfiltrationScore; Homo_723,totalSample,immune-CD4_naive; Homo_723,totalSample,immune-CD4_T; Homo_723,totalSample,immune-Tfh; Homo_723,totalSample,immune-NK; Homo_714,totalSample,immune-InfiltrationScore; Homo_714,totalSample,immune-CD4_naive; Homo_714,totalSample,immune-Effector_memory; Homo_633,totalSample,immune-InfiltrationScore; Homo_633,totalSample,immune-Effector_memory; Homo_633,totalSample,immune-Tfh; Homo_633,totalSample,immune-CD4_T; Homo_633,totalSample,immune-CD4_naive; MCSA,totalSample,immune-Macrophage; MCSA,totalSample,immune-CD4_T; MCSA,totalSample,immune-CD4_naive; MCSA,totalSample,immune-Tr1; MCSA,totalSample,immune-nTreg; MCSA,totalSample,immune-iTreg; MCSA,totalSample,immune-Th17; MCSA,totalSample,immune-Tfh; MCSA,totalSample,immune-MAIT; MCSA,totalSample,immune-Central_memory; MCSA,totalSample,immune-
TP53TG3F	ACOM,female,AD.vs.control; Homo_723,female,AD.vs.MCI; Homo_723,female,MCI.vs.control; Homo_714,female,AD.vs.control; Homo_714,female,AD.vs.MCI; Homo_714,male,AD.vs.MCI; Homo_714,female,MCI.vs.control; Homo_633,female,MCI.vs.control; MCSA,MCI,age; Emory,totalSample,immune-Th17; Homo_723,totalSample,immune-Gamma_delta; Homo_633,totalSample,immune-Th17; Homo_633,totalSample,immune-Gamma_delta
CHST2	ACOM,female,AD.vs.control; Homo_633,female,AD.vs.control; Homo_633,female,AD.vs.MCI; Emory,MCI,age; Emory,totalSample,immune-NK; Emory,totalSample,immune-CD8_naive; Emory,totalSample,immune-Effector_memory; Emory,totalSample,immune-InfiltrationScore; Homo_723,totalSample,immune-DC; Homo_723,totalSample,immune-InfiltrationScore; Homo_723,totalSample,immune-NK; Homo_723,totalSample,immune-Monocyte; Homo_723,totalSample,immune-Neutrophil; Homo_714,totalSample,immune-DC; Homo_714,totalSample,immune-NK; Homo_714,totalSample,immune-InfiltrationScore; Homo_714,totalSample,immune-Monocyte; Homo_714,totalSample,immune-Th2; Homo_633,totalSample,immune-NK; Homo_633,totalSample,immune-DC; Homo_633,totalSample,immune-InfiltrationScore; Homo_633,totalSample,immune-Monocyte; MCSA,totalSample,immune-DC; MCSA,totalSample,immune-Monocyte; MCSA,totalSample,immune-NK; MCSA,totalSample,immune-NKT; MCSA,totalSample,immune-Th2; MCSA,totalSample,immune-Effector_memory; MCSA,totalSample,immune-InfiltrationScore; SRP223445,totalSample,immune-NK; SRP223445,totalSample,immune-Neutrophil; SRP223445,totalSample,immune-nTreg; SRP223445,totalSample,immune-InfiltrationScore
FTCD	ACOM,female,AD.vs.control; ACOM,AD,male.vs.female; ROSMAP,female,AD.vs.control; MCSA,MCI,age; ROSMAP,AD,last.visit.age; ROSMAP,AD,sample.age; ROSMAP,MCI,sample.age; Emory,totalSample,immune-CD8_T; MCSA,totalSample,immune-Monocyte; MCSA,totalSample,immune-CD8_T; MCSA,totalSample,immune-Cytotoxic; MCSA,totalSample,immune-Effector_memory; ROSMAP,totalSample,immune-CD8_naive
DPEP1	ACOM,female,AD.vs.control; Homo_723,female,AD.vs.MCI; Homo_723,male,AD.vs.MCI; Homo_714,female,AD.vs.MCI; ROSMAP,female,AD.vs.control; ROSMAP,female,AD.vs.MCI; ROSMAP,AD,sample.age; ROSMAP,totalSample,age; SRP223445,totalSample,immune-InfiltrationScore
SMYD1	ACOM,female,AD.vs.control; ACOM,AD,male.vs.female; Homo_723,female,AD.vs.MCI; Homo_723,control,male.vs.female; Homo_714,control,male.vs.female; ROSMAP,male,AD.vs.control; MCSA,MCI,age; MCSA,totalSample,immune-Central_memory; ROSMAP,totalSample,immune-InfiltrationScore
SULT1A4	ACOM,female,AD.vs.control; Homo_723,female,AD.vs.control; Homo_723,male,AD.vs.control; Homo_723,female,AD.vs.MCI; Homo_723,male,AD.vs.MCI; Homo_714,female,AD.vs.control; Homo_714,male,AD.vs.control; Homo_714,female,AD.vs.MCI; Homo_714,male,AD.vs.MCI; Homo_633,female,AD.vs.control; Homo_633,female,AD.vs.MCI; Emory,MCI,age; MCSA,MCI,age; Homo_723,totalSample,immune-InfiltrationScore; Homo_723,totalSample,immune-Effector_memory; Homo_723,totalSample,immune-Th1; Homo_723,totalSample,immune-Monocyte; Homo_723,totalSample,immune-Bcell; Homo_723,totalSample,immune-Cytotoxic; Homo_714,totalSample,immune-Th1; Homo_714,totalSample,immune-Effector_memory; Homo_714,totalSample,immune-Th2; Homo_714,totalSample,immune-InfiltrationScore; Homo_633,totalSample,immune-InfiltrationScore; Homo_633,totalSample,immune-DC; Homo_633,totalSample,immune-Monocyte; Homo_633,totalSample,immune-Exhausted; Homo_633,totalSample,immune-Th1; MCSA,totalSample,immune-DC; MCSA,totalSample,immune-Monocyte; MCSA,totalSample,immune-Macrophage; MCSA,totalSample,immune-iTreg; MCSA,totalSample,immune-Th2; MCSA,totalSample,immune-Cytotoxic; MCSA,totalSample,immune-InfiltrationScore; ROSMAP,totalSample,immune-iTreg; ROSMAP,totalSample,immune-Th17; ROSMAP,totalSample,immune-Cytotoxic; ROSMAP,totalSample,immune-Effector_memory;
TP53TG3C	ACOM,female,AD.vs.control; Homo_723,female,AD.vs.MCI; Homo_723,female,MCI.vs.control; Homo_714,female,AD.vs.control; Homo_714,female,AD.vs.MCI; Homo_714,female,MCI.vs.control; Homo_633,female,MCI.vs.control; ROSMAP,female,AD.vs.MCI; Emory,totalSample,immune-Neutrophil; Emory,totalSample,immune-CD8_T; Emory,totalSample,immune-Gamma_delta; Emory,totalSample,immune-Th17; Emory,totalSample,immune-Cytotoxic; Emory,totalSample,immune-Exhausted; Emory,totalSample,immune-Effector_memory; Homo_723,totalSample,immune-Gamma_delta; Homo_714,totalSample,immune-Gamma_delta; Homo_633,totalSample,immune-Gamma_delta; Homo_633,totalSample,immune-Th17
ZNF229	ACOM,female,AD.vs.control; ACOM,male,AD.vs.control; Homo_723,female,AD.vs.control; Homo_723,male,AD.vs.control; Homo_723,female,AD.vs.MCI; Homo_723,male,AD.vs.MCI; Homo_723,female,MCI.vs.control; Homo_723,male,MCI.vs.control; Homo_714,female,AD.vs.control; Homo_714,male,AD.vs.control; Homo_714,female,AD.vs.MCI; Homo_714,male,AD.vs.MCI; Homo_714,female,MCI.vs.control; Homo_633,female,AD.vs.control; Homo_633,female,AD.vs.MCI; Homo_633,female,MCI.vs.control; Emory,totalSample,immune-Tr1; Emory,totalSample,immune-iTreg; Homo_723,totalSample,immune-Exhausted; Homo_723,totalSample,immune-CD4_naive; Homo_723,totalSample,immune-Tr1; Homo_723,totalSample,immune-Tfh; Homo_723,totalSample,immune-Th2; Homo_723,totalSample,immune-Cytotoxic; Homo_723,totalSample,immune-Th17; Homo_723,totalSample,immune-Central_memory; Homo_723,totalSample,immune-iTreg; Homo_723,totalSample,immune-Th1; Homo_723,totalSample,immune-nTreg; Homo_723,totalSample,immune-Effector_memory; Homo_723,totalSample,immune-Gamma_delta; Homo_714,totalSample,immune-Tfh; Homo_714,totalSample,immune-CD4_naive; Homo_714,totalSample,immune-CD4_T; Homo_714,totalSample,immune-Tr1; Homo_714,totalSample,immune-Central_memory; Homo_714,totalSample,immune-Exhausted; Homo_714,totalSample,immune-Th2; Homo_714,totalSample,immune-Gamma_delta; Homo_714,totalSample,immune-Effector_memory; Homo_714,totalSample,immune-iTreg; Homo_714,totalSample,immune-Th17; Homo_714,totalSample,immune-Monocyte; Homo_714,totalSample,immune-Th1; Homo_714,totalSample,immune-nTreg; Homo_714,totalSample,immune-Cytotoxic; Homo_633,totalSample,immune-Tfh; Homo_633,totalSample,immune-CD4_T; Homo_633,totalSample,immune-Th2; Homo_633,totalSample,immune-CD4_naive; Homo_633,totalSample,immune-Central_memory; Homo_633,totalSample,immune-Effector_memory; Homo_633,totalSample,immune-Gamma_delta; Homo_633,totalSample,immune-Exhausted; Homo_633,totalSample,immune-iTreg; Homo_633,totalSample,immune-NKT; Homo_633,totalSample,immune-nTreg; Homo_633,totalSample,immune-Th17; Homo_633,totalSample,immune-Monocyte; MCSA,totalSample,immune-DC; MCSA,totalSample,immune-Bcell; MCSA,totalSample,immune-Macrophage; MCSA,totalSample,immune-CD4_T; MCSA,totalSample,immune-NKT; MCSA,totalSample,immune-CD4_naive; MCSA,totalSample,immune-Tr1; MCSA,totalSample,immune-nTreg; MCSA,totalSample,immune-iTreg; MCSA,totalSample,immune-Tfh; MCSA,totalSample,immune-CD8_naive; MCSA,totalSample,immune-Cytotoxic; MCSA,totalSample,immune-Exhausted; MCSA,totalSample,immune-MAIT; MCSA,totalSample,immune-Central_memory; ROSMAP,totalSample,immune-InfiltrationScore
DIO1	ACOM,female,AD.vs.control; Homo_723,male,AD.vs.MCI; Homo_723,totalSample,immune-Th2; Homo_723,totalSample,immune-CD4_T; Homo_723,totalSample,immune-Tfh; Homo_723,totalSample,immune-iTreg; Homo_714,totalSample,immune-Th2; Homo_714,totalSample,immune-Tfh; Homo_633,totalSample,immune-Th2; Homo_633,totalSample,immune-Tfh; Homo_633,totalSample,immune-CD4_T; ROSMAP,totalSample,immune-Th17; ROSMAP,totalSample,immune-InfiltrationScore

SERPINA10	ACOM,female,AD.vs.control; Homo_723,male,AD.vs.control; Homo_723,female,AD.vs.MCI; Homo_723,male,AD.vs.MCI; Homo_723,female,MCI.vs.control; Homo_714,male,AD.vs.control; Homo_714,male,AD.vs.MCI; ROSMAP,AD,sample.age; ROSMAP,MCI,MMSE; Homo_723,totalSample,immune-Th2; Homo_723,totalSample,immune-Exhausted; Homo_723,totalSample,immune-CD4_T; Homo_723,totalSample,immune-Tfh; Homo_723,totalSample,immune-Tr1; Homo_723,totalSample,immune-CD4_naive; Homo_723,totalSample,immune-Th17; Homo_723,totalSample,immune-Cytotoxic; Homo_714,totalSample,immune-Th2; Homo_714,totalSample,immune-Tfh; Homo_714,totalSample,immune-CD4_T; Homo_714,totalSample,immune-Tr1; Homo_633,totalSample,immune-Th2; Homo_633,totalSample,immune-Tfh; Homo_633,totalSample,immune-CD4_T; Homo_633,totalSample,immune-Exhausted; Homo_633,totalSample,immune-CD4_naive; Homo_633,totalSample,immune-Th17; Homo_633,totalSample,immune-Central_memory; Homo_633,totalSample,immune-Effector_memory; MCSA,totalSample,immune-DC; MCSA,totalSample,immune-CD4_T; MCSA,totalSample,immune-CD4_naive; MCSA,totalSample,immune-Tr1; MCSA,totalSample,immune-nTreg; MCSA,totalSample,immune-iTreg; MCSA,totalSample,immune-Th17; MCSA,totalSample,immune-Tfh; MCSA,totalSample,immune-CD8_naive; MCSA,totalSample,immune-Cytotoxic; MCSA,totalSample,immune-Exhausted; MCSA,totalSample,immune-Central_memory; ROSMAP,male and female-excludeAge90+,survival; ROSMAP,male and female-includeAge90+,survival
UNC45B	ACOM,female,AD.vs.control; ACOM,AD,male.vs.female; Homo_723,male,AD.vs.control; Homo_633,female,AD.vs.control; Homo_633,female,AD.vs.MCI; ACOM,totalSample,immune-Effector_memory; Emory,totalSample,immune-Effector_memory; Homo_723,totalSample,immune-InfiltrationScore; Homo_723,totalSample,immune-Effector_memory; Homo_723,totalSample,immune-Tfh; Homo_723,totalSample,immune-CD4_naive; Homo_723,totalSample,immune-Bcell; Homo_723,totalSample,immune-CD4_T; Homo_723,totalSample,immune-MAIT; Homo_714,totalSample,immune-InfiltrationScore; Homo_714,totalSample,immune-Effector_memory; Homo_714,totalSample,immune-Tfh; Homo_714,totalSample,immune-CD4_naive; Homo_714,totalSample,immune-MAIT; Homo_714,totalSample,immune-Bcell; Homo_633,totalSample,immune-InfiltrationScore; Homo_633,totalSample,immune-Effector_memory; Homo_633,totalSample,immune-Tfh; Homo_633,totalSample,immune-CD4_naive; Homo_633,totalSample,immune-Bcell; Homo_633,totalSample,immune-MAIT; Homo_633,totalSample,immune-CD4_T; Homo_633,totalSample,immune-Central_memory; Homo_633,totalSample,immune-Neutrophil; Homo_633,totalSample,immune-Macrophage; MCSA,totalSample,immune-DC; MCSA,totalSample,immune-Macrophage; MCSA,totalSample,immune-Neutrophil; MCSA,totalSample,immune-CD4_T; MCSA,totalSample,immune-CD8_T; MCSA,totalSample,immune-NKT; MCSA,totalSample,immune-Gamma_delta; MCSA,totalSample,immune-CD4_naive; MCSA,totalSample,immune-Tr1; MCSA,totalSample,immune-nTreg; MCSA,totalSample,immune-iTreg; MCSA,totalSample,immune-Tfh; MCSA,totalSample,immune-CD8_naive; MCSA,totalSample,immune-MAIT; MCSA,totalSample,immune-[illegible]
RASD1	ACOM,female,AD.vs.control; ROSMAP,female,AD.vs.control; Emory,MCI,age; MCSA,totalSample,age; MCSA,control,age; ROSMAP,MCI,sample.age; Homo_723,totalSample,immune-DC; Homo_714,totalSample,immune-DC; MCSA,totalSample,immune-DC; MCSA,totalSample,immune-Monocyte; MCSA,totalSample,immune-Th2; SRP223445,totalSample,immune-nTreg
TP53TG3E	ACOM,female,AD.vs.control; Homo_723,female,AD.vs.control; Homo_723,female,AD.vs.MCI; Homo_723,female,MCI.vs.control; Homo_714,female,AD.vs.control; Homo_714,female,AD.vs.MCI; Homo_714,male,AD.vs.MCI; Homo_714,female,MCI.vs.control; Homo_633,female,MCI.vs.control; MCSA,MCI,age; Emory,totalSample,immune-Th17; Homo_723,totalSample,immune-Gamma_delta; Homo_633,totalSample,immune-Gamma_delta; MCSA,totalSample,immune-iTreg; ROSMAP,female-excludeAge90+,survival; ROSMAP,female-includeAge90+,survival; ROSMAP,male and female-excludeAge90+,survival
SGK3	ACOM,female,AD.vs.control; ACOM,AD,male.vs.female; Homo_633,female,AD.vs.control; Homo_633,female,AD.vs.MCI; MCSA,MCI,age; Emory,totalSample,immune-Monocyte; Emory,totalSample,immune-CD4_T; Emory,totalSample,immune-Tr1; Emory,totalSample,immune-iTreg; Emory,totalSample,immune-Effector_memory; Emory,totalSample,immune-InfiltrationScore; Homo_723,totalSample,immune-InfiltrationScore; Homo_723,totalSample,immune-Tfh; Homo_723,totalSample,immune-iTreg; Homo_723,totalSample,immune-Tr1; Homo_723,totalSample,immune-Monocyte; Homo_723,totalSample,immune-Th2; Homo_723,totalSample,immune-Exhausted; Homo_723,totalSample,immune-Effector_memory; Homo_723,totalSample,immune-CD4_naive; Homo_723,totalSample,immune-CD4_T; Homo_723,totalSample,immune-DC; Homo_723,totalSample,immune-Gamma_delta; Homo_714,totalSample,immune-Tfh; Homo_714,totalSample,immune-Monocyte; Homo_714,totalSample,immune-Tr1; Homo_714,totalSample,immune-Th2; Homo_714,totalSample,immune-InfiltrationScore; Homo_714,totalSample,immune-Effector_memory; Homo_714,totalSample,immune-CD4_T; Homo_714,totalSample,immune-CD4_naive; Homo_714,totalSample,immune-iTreg; Homo_714,totalSample,immune-Central_memory; Homo_714,totalSample,immune-Exhausted; Homo_633,totalSample,immune-Tfh; Homo_633,totalSample,immune-Effector_memory; Homo_633,totalSample,immune-CD4_T; Homo_633,totalSample,immune-Monocyte; Homo_633,totalSample,immune-CD4_naive; Homo_633,totalSample,immune-Central_memory; Homo_633,totalSample,immune-Th2; Homo_633,totalSample,immune-NKT; Homo_633,totalSample,immune-InfiltrationScore; Homo_633,totalSample,immune-Exhausted; Homo_633,totalSample,immune-Tr1; Homo_633,totalSample,immune-Gamma_delta; Homo_633,totalSample,immune-iTreg; Homo_633,totalSample,immune-Bcell; MCSA,totalSample,immune-DC; MCSA,totalSample,immune-Monocyte; MCSA,totalSample,immune-CD4_T; MCSA,totalSample,immune-CD8_T; MCSA,totalSample,immune-CD4_naive; MCSA,totalSample,immune-Tr1; MCSA,totalSample,immune-nTreg; MCSA,totalSample,immune-iTreg; MCSA,totalSample,immune-Tfh; MCSA,totalSample,immune-CD8_naive; MCSA,totalSample,immune-Cytotoxic; MCSA,totalSample,immune-Exhausted; MCSA,totalSample,immune-MAIT; MCSA,totalSample,immune-Central_memory; ROSMAP,totalSample,immune-[illegible]
PCDH20	ACOM,female,AD.vs.control; ROSMAP,female,AD.vs.control; ROSMAP,MCI,sample.age; Emory,totalSample,immune-CD8_T; ROSMAP,totalSample,immune-InfiltrationScore
SLC13A5	ACOM,female,AD.vs.control; Homo_723,female,AD.vs.control; Homo_723,female,AD.vs.MCI; Homo_714,female,AD.vs.control; Homo_714,female,AD.vs.MCI; ROSMAP,female,AD.vs.control; MCSA,MCI,age; Emory,totalSample,immune-InfiltrationScore; MCSA,totalSample,immune-Macrophage; MCSA,totalSample,immune-CD4_naive; MCSA,totalSample,immune-nTreg; MCSA,totalSample,immune-Tfh; ROSMAP,totalSample,immune-InfiltrationScore
CAPN12	ACOM,female,AD.vs.control; Homo_723,female,AD.vs.control; Homo_723,male,AD.vs.control; Homo_723,female,AD.vs.MCI; Homo_723,male,AD.vs.MCI; Homo_714,female,AD.vs.control; Homo_714,male,AD.vs.control; Homo_714,female,AD.vs.MCI; Homo_714,male,AD.vs.MCI; Homo_633,female,AD.vs.control; Homo_633,female,AD.vs.MCI; ROSMAP,female,AD.vs.MCI; MCSA,totalSample,age; MCSA,control,age; Emory,totalSample,immune-Macrophage; Emory,totalSample,immune-NK; Emory,totalSample,immune-Neutrophil; Emory,totalSample,immune-CD4_T; Emory,totalSample,immune-CD8_T; Emory,totalSample,immune-Tr1; Emory,totalSample,immune-iTreg; Emory,totalSample,immune-Tfh; Emory,totalSample,immune-Exhausted; Emory,totalSample,immune-Effector_memory; Emory,totalSample,immune-InfiltrationScore; Homo_723,totalSample,immune-InfiltrationScore; Homo_723,totalSample,immune-CD8_T; Homo_723,totalSample,immune-Effector_memory; Homo_723,totalSample,immune-Cytotoxic; Homo_723,totalSample,immune-iTreg; Homo_723,totalSample,immune-Macrophage; Homo_723,totalSample,immune-nTreg; Homo_723,totalSample,immune-Monocyte; Homo_723,totalSample,immune-Tfh; Homo_723,totalSample,immune-Exhausted; Homo_723,totalSample,immune-MAIT; Homo_723,totalSample,immune-Central_memory; Homo_714,totalSample,immune-InfiltrationScore; Homo_714,totalSample,immune-Effector_memory; Homo_714,totalSample,immune-nTreg; Homo_714,totalSample,immune-CD8_T; Homo_714,totalSample,immune-Macrophage; Homo_714,totalSample,immune-Neutrophil; Homo_714,totalSample,immune-Monocyte; Homo_714,totalSample,immune-Tfh; Homo_633,totalSample,immune-InfiltrationScore; Homo_633,totalSample,immune-Effector_memory; Homo_633,totalSample,immune-Neutrophil; Homo_633,totalSample,immune-nTreg; Homo_633,totalSample,immune-Monocyte; Homo_633,totalSample,immune-DC; Homo_633,totalSample,immune-Macrophage; Homo_633,totalSample,immune-CD8_T; Homo_633,totalSample,immune-NK; MCSA,totalSample,immune-Monocyte; MCSA,totalSample,immune-Neutrophil; MCSA,totalSample,immune-CD8_T; MCSA,totalSample,immune-NKT; MCSA,totalSample,immune-CD4_naive; MCSA,totalSample,immune-nTreg; MCSA,totalSample,immune-Th2; MCSA,totalSample,immune-Tfh; MCSA,totalSample,immune-Cytotoxic; MCSA,totalSample,immune-MAIT; MCSA,totalSample,immune-[illegible]
CPLX4	ACOM,female,AD.vs.control; Homo_723,female,AD.vs.control; Homo_714,female,AD.vs.control; MCSA,MCI,age; ROSMAP,totalSample,immune-InfiltrationScore; SRP223445,totalSample,immune-NK
EPHB2	ACOM,female,AD.vs.control; ROSMAP,male,AD.vs.control; MCSA,MCI,age; Emory,totalSample,immune-DC; Emory,totalSample,immune-CD8_T; Emory,totalSample,immune-Gamma_delta; Emory,totalSample,immune-Tfh; Emory,totalSample,immune-Cytotoxic; Emory,totalSample,immune-InfiltrationScore; Homo_723,totalSample,immune-DC; Homo_714,totalSample,immune-DC; Homo_633,totalSample,immune-DC; MCSA,totalSample,immune-DC; MCSA,totalSample,immune-Monocyte; MCSA,totalSample,immune-Macrophage; MCSA,totalSample,immune-iTreg; MCSA,totalSample,immune-Th2; MCSA,totalSample,immune-InfiltrationScore; ROSMAP,totalSample,immune-Effector_memory
SCIN	ACOM,female,AD.vs.control; Homo_723,female,AD.vs.control; Homo_723,female,AD.vs.MCI; Homo_714,male,AD.vs.control; Homo_633,female,AD.vs.control; Homo_633,female,AD.vs.MCI; MCSA,MCI,age; Homo_723,totalSample,immune-Tfh; Homo_723,totalSample,immune-Th2; Homo_723,totalSample,immune-CD4_T; Homo_723,totalSample,immune-iTreg; Homo_723,totalSample,immune-Tr1; Homo_723,totalSample,immune-CD4_naive; Homo_714,totalSample,immune-Tfh; Homo_714,totalSample,immune-Th2; Homo_714,totalSample,immune-CD4_T; Homo_714,totalSample,immune-Tr1; Homo_633,totalSample,immune-Tfh; Homo_633,totalSample,immune-CD4_T; Homo_633,totalSample,immune-CD4_naive; Homo_633,totalSample,immune-Th2; Homo_633,totalSample,immune-Tr1; MCSA,totalSample,immune-DC; MCSA,totalSample,immune-CD4_T; MCSA,totalSample,immune-CD4_naive; MCSA,totalSample,immune-Tr1; MCSA,totalSample,immune-nTreg; MCSA,totalSample,immune-iTreg; MCSA,totalSample,immune-Tfh; MCSA,totalSample,immune-CD8_naive; MCSA,totalSample,immune-Cytotoxic; MCSA,totalSample,immune-MAIT; MCSA,totalSample,immune-Central_memory; ROSMAP,totalSample,immune-InfiltrationScore
HP	ACOM,female,AD.vs.control; Homo_723,female,MCI.vs.control; Homo_714,control,male.vs.female; Homo_633,female,MCI.vs.control; Homo_633,control,male.vs.female; ROSMAP,female,AD.vs.control; ACOM,AD,age; Emory,MCI,age; Emory,totalSample,immune-Macrophage; Emory,totalSample,immune-NK; Emory,totalSample,immune-Neutrophil; Emory,totalSample,immune-CD4_T; Emory,totalSample,immune-CD8_T; Emory,totalSample,immune-Gamma_delta; Emory,totalSample,immune-Tr1; Emory,totalSample,immune-iTreg; Emory,totalSample,immune-Tfh; Emory,totalSample,immune-CD8_naive; Emory,totalSample,immune-Exhausted; Emory,totalSample,immune-MAIT; Homo_723,totalSample,immune-Macrophage; Homo_723,totalSample,immune-CD4_T; Homo_723,totalSample,immune-Th2; Homo_723,totalSample,immune-iTreg; Homo_723,totalSample,immune-Tr1; Homo_723,totalSample,immune-CD4_naive; Homo_723,totalSample,immune-nTreg; Homo_723,totalSample,immune-Tfh; Homo_723,totalSample,immune-Central_memory; Homo_714,totalSample,immune-Macrophage; Homo_714,totalSample,immune-CD4_T; Homo_714,totalSample,immune-Th2; Homo_714,totalSample,immune-Central_memory; Homo_714,totalSample,immune-iTreg; Homo_714,totalSample,immune-CD4_naive; Homo_714,totalSample,immune-Tr1; Homo_714,totalSample,immune-nTreg; Homo_714,totalSample,immune-Tfh; Homo_714,totalSample,immune-CD8_T; Homo_633,totalSample,immune-Macrophage; Homo_633,totalSample,immune-CD4_T; Homo_633,totalSample,immune-CD4_naive; Homo_633,totalSample,immune-Central_memory; Homo_633,totalSample,immune-iTreg; Homo_633,totalSample,immune-Tr1; Homo_633,totalSample,immune-Th2; Homo_633,totalSample,immune-nTreg; Homo_633,totalSample,immune-Tfh; MCSA,totalSample,immune-Macrophage; MCSA,totalSample,immune-Neutrophil; MCSA,totalSample,immune-CD4_T; MCSA,totalSample,immune-CD8_T; MCSA,totalSample,immune-CD4_naive; MCSA,totalSample,immune-Tr1; MCSA,totalSample,immune-nTreg; MCSA,totalSample,immune-iTreg; MCSA,totalSample,immune-Tfh; [illegible]
GABRG3	ACOM,female,AD.vs.control; Homo_723,female,AD.vs.control; Homo_633,female,MCI.vs.control; ROSMAP,AD,last.visit.age; Emory,totalSample,immune-Neutrophil; Emory,totalSample,immune-CD8_T; Emory,totalSample,immune-Gamma_delta; ROSMAP,totalSample,immune-InfiltrationScore
TYW3	ACOM,female,AD.vs.control; Homo_723,female,MCI.vs.control; Homo_714,female,MCI.vs.control; Homo_633,female,AD.vs.control; Homo_633,female,AD.vs.MCI; Homo_633,female,MCI.vs.control; ROSMAP,female,AD.vs.control; ACOM,AD,age; Emory,MCI,age; MCSA,MCI,age; Emory,totalSample,immune-DC; Emory,totalSample,immune-Macrophage; Emory,totalSample,immune-Neutrophil; Emory,totalSample,immune-CD4_T; Emory,totalSample,immune-CD8_T; Emory,totalSample,immune-Tr1; Emory,totalSample,immune-iTreg; Emory,totalSample,immune-Tfh; Emory,totalSample,immune-Exhausted; Emory,totalSample,immune-Central_memory; Emory,totalSample,immune-Effector_memory; Emory,totalSample,immune-InfiltrationScore; Homo_723,totalSample,immune-Monocyte; Homo_723,totalSample,immune-Tfh; Homo_723,totalSample,immune-Exhausted; Homo_723,totalSample,immune-InfiltrationScore; Homo_723,totalSample,immune-CD4_naive; Homo_723,totalSample,immune-CD4_T; Homo_723,totalSample,immune-Th2; Homo_723,totalSample,immune-Effector_memory; Homo_723,totalSample,immune-Tr1; Homo_723,totalSample,immune-iTreg; Homo_723,totalSample,immune-Central_memory; Homo_714,totalSample,immune-Monocyte; Homo_714,totalSample,immune-Tfh; Homo_714,totalSample,immune-CD4_naive; Homo_714,totalSample,immune-Th2; Homo_714,totalSample,immune-Effector_memory; Homo_714,totalSample,immune-CD4_T; Homo_714,totalSample,immune-InfiltrationScore; Homo_714,totalSample,immune-Central_memory; Homo_714,totalSample,immune-Neutrophil; Homo_714,totalSample,immune-Tr1; Homo_633,totalSample,immune-Tfh; Homo_633,totalSample,immune-Monocyte; Homo_633,totalSample,immune-Th2; Homo_633,totalSample,immune-Central_memory; Homo_633,totalSample,immune-CD4_T; Homo_633,totalSample,immune-CD4_naive; Homo_633,totalSample,immune-Effector_memory; Homo_633,totalSample,immune-Tr1; Homo_633,totalSample,immune-InfiltrationScore; Homo_633,totalSample,immune-Bcell; Homo_633,totalSample,immune-Exhausted; MCSA,totalSample,immune-DC; MCSA,totalSample,immune-Monocyte; MCSA,totalSample,immune-Macrophage; MCSA,totalSample,immune-CD4_T; MCSA,totalSample,immune-CD4_naive; MCSA,totalSample,immune-Tr1; MCSA,totalSample,immune-nTreg; MCSA,totalSample,immune-iTreg; MCSA,totalSample,immune-Th2; MCSA,totalSample,immune-Tfh; MCSA,totalSample,immune-CD8_naive; MCSA,totalSample,immune-Cytotoxic; MCSA,totalSample,immune-Exhausted; MCSA,totalSample,immune-MAIT; MCSA,totalSample,immune-Central_memory; ROSMAP,totalSample,immune-Central_memory; ROSMAP,totalSample,immune-Effector_memory; SRP223445,totalSample,immune-NK; [illegible]
CHCHD2	ACOM,female,AD.vs.control; ACOM,male,AD.vs.control; Homo_723,female,AD.vs.control; ROSMAP,female,AD.vs.control; ROSMAP,female,MCI.vs.control; ROSMAP,MCI,sample.age; Emory,totalSample,immune-DC; Emory,totalSample,immune-Monocyte; Emory,totalSample,immune-Macrophage; Emory,totalSample,immune-Neutrophil; Emory,totalSample,immune-CD4_T; Emory,totalSample,immune-CD8_T; Emory,totalSample,immune-NKT; Emory,totalSample,immune-CD4_naive; Emory,totalSample,immune-Tr1; Emory,totalSample,immune-iTreg; Emory,totalSample,immune-Th1; Emory,totalSample,immune-Tfh; Emory,totalSample,immune-Cytotoxic; Emory,totalSample,immune-Effector_memory; Homo_723,totalSample,immune-Cytotoxic; Homo_723,totalSample,immune-Gamma_delta; Homo_723,totalSample,immune-Neutrophil; Homo_723,totalSample,immune-Th17; Homo_723,totalSample,immune-Th2; Homo_723,totalSample,immune-Exhausted; Homo_723,totalSample,immune-CD8_T; Homo_723,totalSample,immune-DC; Homo_723,totalSample,immune-iTreg; Homo_723,totalSample,immune-CD4_T; Homo_714,totalSample,immune-Th2; Homo_714,totalSample,immune-Th17; Homo_714,totalSample,immune-Cytotoxic; Homo_714,totalSample,immune-Gamma_delta; Homo_714,totalSample,immune-Neutrophil; Homo_714,totalSample,immune-Exhausted; Homo_714,totalSample,immune-CD8_T; Homo_714,totalSample,immune-DC; Homo_714,totalSample,immune-CD4_T; Homo_714,totalSample,immune-iTreg; Homo_633,totalSample,immune-Th2; Homo_633,totalSample,immune-Th17; Homo_633,totalSample,immune-Cytotoxic; Homo_633,totalSample,immune-Gamma_delta; Homo_633,totalSample,immune-Neutrophil; Homo_633,totalSample,immune-DC; Homo_633,totalSample,immune-CD8_T; Homo_633,totalSample,immune-Exhausted; Homo_633,totalSample,immune-CD4_T; MCSA,totalSample,immune-DC; MCSA,totalSample,immune-Neutrophil; MCSA,totalSample,immune-CD4_T; MCSA,totalSample,immune-CD8_T; MCSA,totalSample,immune-NKT; MCSA,totalSample,immune-Gamma_delta; MCSA,totalSample,immune-Tr1; MCSA,totalSample,immune-nTreg; MCSA,totalSample,immune-iTreg; MCSA,totalSample,immune-Th17; MCSA,totalSample,immune-Cytotoxic; MCSA,totalSample,immune-Exhausted; MCSA,totalSample,immune-Effector_memory; ROSMAP,totalSample,immune-CD4_T; ROSMAP,totalSample,immune-nTreg; ROSMAP,totalSample,immune-Th2; ROSMAP,totalSample,immune-Th17; [illegible]
FOXG1	ACOM,female,AD.vs.control; Homo_723,female,AD.vs.control; Homo_723,male,AD.vs.control; Homo_723,female,AD.vs.MCI; Homo_714,female,AD.vs.control; Homo_714,female,AD.vs.MCI; Homo_633,female,MCI.vs.control; Homo_633,male,MCI.vs.control; ROSMAP,female,AD.vs.control
FABP2	ACOM,female,AD.vs.control; ROSMAP,female,AD.vs.control; ROSMAP,male and female-excludeAge90+,survival
HES5	ACOM,female,AD.vs.control; ACOM,AD,male.vs.female; Homo_723,male,AD.vs.control; Homo_723,female,AD.vs.MCI; Homo_723,male,AD.vs.MCI; Homo_723,female,MCI.vs.control; ROSMAP,female,AD.vs.control; ROSMAP,female,MCI.vs.control; ROSMAP,totalSample,age; ROSMAP,MCI,sample.age; ROSMAP,totalSample,MMSE; Emory,totalSample,immune-Central_memory; Homo_723,totalSample,immune-DC; Homo_723,totalSample,immune-Neutrophil; Homo_723,totalSample,immune-Th17; Homo_723,totalSample,immune-CD8_T; Homo_723,totalSample,immune-Exhausted; Homo_723,totalSample,immune-MAIT; Homo_714,totalSample,immune-Th2; ROSMAP,totalSample,immune-CD4_T; ROSMAP,totalSample,immune-Tr1; ROSMAP,totalSample,immune-iTreg; ROSMAP,totalSample,immune-Th17; ROSMAP,totalSample,immune-CD8_naive; ROSMAP,totalSample,immune-Central_memory; SRP223445,totalSample,immune-Neutrophil
SNTB2	ACOM,male,AD.vs.control; Emory,totalSample,immune-NK; Emory,totalSample,immune-Tr1; Emory,totalSample,immune-iTreg; Emory,totalSample,immune-Th17; Emory,totalSample,immune-InfiltrationScore; Homo_723,totalSample,immune-InfiltrationScore; Homo_723,totalSample,immune-Tfh; Homo_723,totalSample,immune-CD4_naive; Homo_723,totalSample,immune-iTreg; Homo_723,totalSample,immune-CD4_T; Homo_723,totalSample,immune-Th2; Homo_723,totalSample,immune-Tr1; Homo_723,totalSample,immune-Effector_memory; Homo_723,totalSample,immune-DC; Homo_714,totalSample,immune-Tfh; Homo_714,totalSample,immune-InfiltrationScore; Homo_714,totalSample,immune-CD4_naive; Homo_714,totalSample,immune-CD4_T; Homo_714,totalSample,immune-Effector_memory; Homo_714,totalSample,immune-iTreg; Homo_714,totalSample,immune-Th2; Homo_714,totalSample,immune-Tr1; Homo_714,totalSample,immune-NKT; Homo_714,totalSample,immune-DC; Homo_633,totalSample,immune-InfiltrationScore; Homo_633,totalSample,immune-Tfh; Homo_633,totalSample,immune-NKT; Homo_633,totalSample,immune-CD4_naive; Homo_633,totalSample,immune-CD4_T; Homo_633,totalSample,immune-Effector_memory; Homo_633,totalSample,immune-Th2; Homo_633,totalSample,immune-Tr1; Homo_633,totalSample,immune-iTreg; Homo_633,totalSample,immune-Th17; Homo_633,totalSample,immune-DC; Homo_633,totalSample,immune-Bcell; Homo_633,totalSample,immune-Central_memory; MCSA,totalSample,immune-DC; MCSA,totalSample,immune-Monocyte; MCSA,totalSample,immune-Macrophage; MCSA,totalSample,immune-CD4_T; MCSA,totalSample,immune-CD8_T; MCSA,totalSample,immune-CD4_naive; MCSA,totalSample,immune-Tr1; MCSA,totalSample,immune-nTreg; MCSA,totalSample,immune-iTreg; MCSA,totalSample,immune-Th17; MCSA,totalSample,immune-Tfh; MCSA,totalSample,immune-CD8_naive; MCSA,totalSample,immune-Cytotoxic; MCSA,totalSample,immune-Exhausted; MCSA,totalSample,immune-MAIT; MCSA,totalSample,immune-Central_memory; ROSMAP,totalSample,immune-Th17; ROSMAP,totalSample,immune-Cytotoxic; ROSMAP,totalSample,immune-Effector_memory; ROSMAP,totalSample,immune-InfiltrationScore; SRP223445,totalSample,immune-CD4_naive; SRP223445,totalSample,immune-Exhausted; ROSMAP,male and female-includeAge90+,survival
MTRNR2L1	ACOM,male,AD.vs.control; Emory,control,male.vs.female; Homo_723,female,AD.vs.control; Homo_723,female,MCI.vs.control; Homo_723,male,MCI.vs.control; Homo_714,female,AD.vs.control; Homo_714,female,MCI.vs.control; Homo_714,male,MCI.vs.control; Homo_633,female,MCI.vs.control; Homo_633,male,MCI.vs.control; ROSMAP,female,AD.vs.control; ROSMAP,AD,last.visit.age; Homo_723,totalSample,immune-Monocyte; Homo_723,totalSample,immune-DC; Homo_723,totalSample,immune-Exhausted; Homo_723,totalSample,immune-Th2; Homo_714,totalSample,immune-Th2; Homo_714,totalSample,immune-Monocyte; Homo_633,totalSample,immune-Monocyte; Homo_633,totalSample,immune-Th2
LIN28A	ACOM,male,AD.vs.control; Homo_723,female,AD.vs.control; Homo_723,female,MCI.vs.control; Homo_714,female,MCI.vs.control; Homo_633,female,MCI.vs.control; ACOM,totalSample,age; Homo_723,totalSample,immune-CD8_naive
RUVBL1	ACOM,male,AD.vs.control; ROSMAP,female,AD.vs.control; ACOM,totalSample,age; MCSA,totalSample,age; MCSA,control,age; Emory,totalSample,immune-Macrophage; Emory,totalSample,immune-NK; Emory,totalSample,immune-Neutrophil; Emory,totalSample,immune-CD4_T; Emory,totalSample,immune-CD8_T; Emory,totalSample,immune-Tr1; Emory,totalSample,immune-iTreg; Emory,totalSample,immune-Tfh; Emory,totalSample,immune-Exhausted; Emory,totalSample,immune-InfiltrationScore; Homo_723,totalSample,immune-InfiltrationScore; Homo_723,totalSample,immune-CD4_naive; Homo_723,totalSample,immune-iTreg; Homo_723,totalSample,immune-Effector_memory; Homo_723,totalSample,immune-Tfh; Homo_723,totalSample,immune-nTreg; Homo_723,totalSample,immune-CD4_T; Homo_723,totalSample,immune-Monocyte; Homo_723,totalSample,immune-Tr1; Homo_723,totalSample,immune-Central_memory; Homo_723,totalSample,immune-Macrophage; Homo_714,totalSample,immune-InfiltrationScore; Homo_714,totalSample,immune-Effector_memory; Homo_714,totalSample,immune-CD4_naive; Homo_714,totalSample,immune-Tfh; Homo_714,totalSample,immune-nTreg; Homo_714,totalSample,immune-iTreg; Homo_714,totalSample,immune-CD4_T; Homo_714,totalSample,immune-Neutrophil; Homo_714,totalSample,immune-Tr1; Homo_714,totalSample,immune-Central_memory; Homo_633,totalSample,immune-InfiltrationScore; Homo_633,totalSample,immune-Effector_memory; Homo_633,totalSample,immune-CD4_naive; Homo_633,totalSample,immune-Central_memory; Homo_633,totalSample,immune-nTreg; Homo_633,totalSample,immune-Tfh; Homo_633,totalSample,immune-CD4_T; Homo_633,totalSample,immune-Tr1; Homo_633,totalSample,immune-Monocyte; Homo_633,totalSample,immune-Neutrophil; Homo_633,totalSample,immune-iTreg; MCSA,totalSample,immune-Monocyte; MCSA,totalSample,immune-Macrophage; MCSA,totalSample,immune-Neutrophil; MCSA,totalSample,immune-CD4_T; MCSA,totalSample,immune-CD8_T; MCSA,totalSample,immune-CD4_naive; MCSA,totalSample,immune-Tr1; MCSA,totalSample,immune-nTreg; MCSA,totalSample,immune-iTreg; MCSA,totalSample,immune-Th17; MCSA,totalSample,immune-Tfh; MCSA,totalSample,immune-CD8_naive; MCSA,totalSample,immune-MAIT; MCSA,totalSample,immune-Central_memory; MCSA,totalSample,immune-Effector_memory; MCSA,totalSample,immune-InfiltrationScore; ROSMAP,totalSample,immune-Cytotoxic; ROSMAP,totalSample,immune-Central_memory; ROSMAP,totalSample,immune-Effector_memory; SRP223445,totalSample,immune-CD4_naive; SRP223445,totalSample,immune-InfiltrationScore

SEPTIN9	ACOM,male,AD.vs.control; ROSMAP,female,AD.vs.control; ROSMAP,male,AD.vs.control; ACOM,totalSample,age; MCSA,totalSample,age; MCSA,control,age; MCSA,MCI,age; ROSMAP,AD,last.visit.age; Emory,totalSample,immune-Monocyte; Emory,totalSample,immune-Macrophage; Emory,totalSample,immune-NK; Emory,totalSample,immune-Neutrophil; Emory,totalSample,immune-CD4_T; Emory,totalSample,immune-CD8_T; Emory,totalSample,immune-CD4_naive; Emory,totalSample,immune-Tr1; Emory,totalSample,immune-nTreg; Emory,totalSample,immune-iTreg; Emory,totalSample,immune-Tfh; Emory,totalSample,immune-Exhausted; Emory,totalSample,immune-MAIT; Emory,totalSample,immune-Central_memory; Emory,totalSample,immune-Effector_memory; Emory,totalSample,immune-InfiltrationScore; Homo_723,totalSample,immune-CD8_T; Homo_723,totalSample,immune-DC; Homo_723,totalSample,immune-nTreg; Homo_723,totalSample,immune-Monocyte; Homo_723,totalSample,immune-InfiltrationScore; Homo_723,totalSample,immune-Effector_memory; Homo_723,totalSample,immune-Neutrophil; Homo_723,totalSample,immune-Cytotoxic; Homo_723,totalSample,immune-Macrophage; Homo_723,totalSample,immune-Central_memory; Homo_723,totalSample,immune-CD4_naive; Homo_723,totalSample,immune-Tfh; Homo_723,totalSample,immune-Exhausted; Homo_723,totalSample,immune-Gamma_delta; Homo_714,totalSample,immune-InfiltrationScore; Homo_714,totalSample,immune-nTreg; Homo_714,totalSample,immune-Effector_memory; Homo_714,totalSample,immune-DC; Homo_714,totalSample,immune-Monocyte; Homo_714,totalSample,immune-CD8_T; Homo_714,totalSample,immune-Neutrophil; Homo_714,totalSample,immune-Central_memory; Homo_714,totalSample,immune-CD4_naive; Homo_714,totalSample,immune-Macrophage; Homo_714,totalSample,immune-Tfh; Homo_714,totalSample,immune-iTreg; Homo_714,totalSample,immune-CD4_T; Homo_633,totalSample,immune-nTreg; Homo_633,totalSample,immune-InfiltrationScore; Homo_633,totalSample,immune-DC; Homo_633,totalSample,immune-Effector_memory; Homo_633,totalSample,immune-Monocyte; Homo_633,totalSample,immune-Central_memory; Homo_633,totalSample,immune-CD8_T; Homo_633,totalSample,immune-CD4_naive; Homo_633,totalSample,immune-Tr1; Homo_633,totalSample,immune-iTreg; Homo_633,totalSample,immune-Neutrophil; Homo_633,totalSample,immune-CD4_T; Homo_633,totalSample,immune-Tfh; Homo_633,totalSample,immune-Macrophage; MCSA,totalSample,immune-DC; MCSA,totalSample,immune-Monocyte; MCSA,totalSample,immune-Neutrophil; MCSA,totalSample,immune-CD8_T; MCSA,totalSample,immune-NKT; MCSA,totalSample,immune-Th2; MCSA,totalSample,immune-Tfh; MCSA,totalSample,immune-Cytotoxic; MCSA,totalSample,immune-Central_memory; MCSA,totalSample,immune-Effector_memory; MCSA,totalSample,immune-InfiltrationScore; ROSMAP,totalSample,immune-Effector_memory; SRP223445,totalSample,immune-NK; SRP223445,totalSample,immune-Neutrophil; SRP223445,totalSample,immune-CD4_naive; SRP223445,totalSample,immune-nTreg; [illegible]
DPP6	ACOM,male,AD.vs.control; Homo_633,male,MCI.vs.control; Emory,totalSample,immune-CD8_T; Homo_723,totalSample,immune-DC; Homo_714,totalSample,immune-Th2; ROSMAP,totalSample,immune-InfiltrationScore
ANKHD1-EIF4EBP3	ACOM,male,AD.vs.control; ACOM,AD,male.vs.female; Homo_723,male,AD.vs.control; Homo_723,male,AD.vs.MCI; Homo_714,male,AD.vs.control; Homo_714,male,AD.vs.MCI; ACOM,totalSample,age; MCSA,totalSample,age; MCSA,control,age; Homo_723,totalSample,immune-CD4_naive; Homo_723,totalSample,immune-iTreg; Homo_723,totalSample,immune-InfiltrationScore; Homo_723,totalSample,immune-Monocyte; Homo_723,totalSample,immune-CD4_T; Homo_723,totalSample,immune-nTreg; Homo_723,totalSample,immune-Effector_memory; Homo_723,totalSample,immune-Tfh; Homo_723,totalSample,immune-Central_memory; Homo_714,totalSample,immune-CD4_naive; Homo_714,totalSample,immune-nTreg; Homo_714,totalSample,immune-iTreg; Homo_714,totalSample,immune-Effector_memory; Homo_714,totalSample,immune-CD4_T; Homo_714,totalSample,immune-InfiltrationScore; Homo_714,totalSample,immune-Monocyte; Homo_714,totalSample,immune-Tfh; Homo_633,totalSample,immune-CD4_naive; Homo_633,totalSample,immune-CD4_T; Homo_633,totalSample,immune-nTreg; Homo_633,totalSample,immune-iTreg; Homo_633,totalSample,immune-InfiltrationScore; Homo_633,totalSample,immune-Effector_memory; Homo_633,totalSample,immune-Tfh; Homo_633,totalSample,immune-Monocyte; Homo_633,totalSample,immune-Central_memory; Homo_633,totalSample,immune-Tr1; MCSA,totalSample,immune-Monocyte; MCSA,totalSample,immune-CD4_T; MCSA,totalSample,immune-Gamma_delta; MCSA,totalSample,immune-CD4_naive; MCSA,totalSample,immune-Tr1; MCSA,totalSample,immune-nTreg; MCSA,totalSample,immune-iTreg; MCSA,totalSample,immune-Th17; MCSA,totalSample,immune-Tfh; MCSA,totalSample,immune-Exhausted;
MCCC2	ACOM,male,AD.vs.control; ACOM,AD,male.vs.female; Homo_633,female,AD.vs.control; Homo_633,female,AD.vs.MCI; Homo_633,male,MCI.vs.control; ACOM,totalSample,age; MCSA,MCI,age; Emory,totalSample,immune-Macrophage; Emory,totalSample,immune-Neutrophil; Emory,totalSample,immune-CD4_T; Emory,totalSample,immune-CD8_T; Emory,totalSample,immune-Tr1; Emory,totalSample,immune-iTreg; Emory,totalSample,immune-MAIT; Emory,totalSample,immune-InfiltrationScore; Homo_723,totalSample,immune-DC; Homo_714,totalSample,immune-InfiltrationScore; Homo_633,totalSample,immune-InfiltrationScore; ROSMAP,totalSample,immune-Th17; ROSMAP,totalSample,immune-Effector_memory; ROSMAP,totalSample,immune-InfiltrationScore; SRP223445,totalSample,immune-Neutrophil; SRP223445,totalSample,immune-InfiltrationScore
TBCEL-TECTA	ACOM,male,AD.vs.control; ROSMAP,male,AD.vs.control; ROSMAP,male,MCI.vs.control; ROSMAP,totalSample,last.visit.age; ROSMAP,totalSample,age; ROSMAP,control,last.visit.age; ROSMAP,control,sample.age; ROSMAP,MCI,last.visit.age; ROSMAP,MCI,sample.age; Homo_723,totalSample,immune-Bcell; Homo_633,totalSample,immune-Bcell; MCSA,totalSample,immune-Neutrophil; MCSA,totalSample,immune-CD4_T; MCSA,totalSample,immune-CD4_naive; MCSA,totalSample,immune-Tr1; MCSA,totalSample,immune-nTreg; MCSA,totalSample,immune-Tfh; MCSA,totalSample,immune-CD8_naive; MCSA,totalSample,immune-Central_memory; MCSA,totalSample,immune-Effector_memory; ROSMAP,totalSample,immune-CD4_T; ROSMAP,totalSample,immune-Th17; ROSMAP,totalSample,immune-InfiltrationScore; SRP223445,totalSample,immune-Th17
EOMES	ACOM,male,AD.vs.control; Homo_723,female,AD.vs.control; Homo_723,male,AD.vs.control; Homo_723,female,AD.vs.MCI; Homo_723,male,AD.vs.MCI; Homo_723,control,male.vs.female; Homo_714,female,AD.vs.control; Homo_714,male,AD.vs.control; Homo_714,female,AD.vs.MCI; Homo_714,male,AD.vs.MCI; Homo_714,female,MCI.vs.control; Homo_714,control,male.vs.female; Homo_633,control,male.vs.female; ACOM,totalSample,age; Emory,totalSample,immune-Macrophage; Emory,totalSample,immune-NK; Emory,totalSample,immune-Neutrophil; Emory,totalSample,immune-CD8_T; Emory,totalSample,immune-Gamma_delta; Emory,totalSample,immune-CD4_naive; Emory,totalSample,immune-Cytotoxic; Emory,totalSample,immune-Exhausted; Emory,totalSample,immune-Central_memory; Emory,totalSample,immune-Effector_memory; Emory,totalSample,immune-InfiltrationScore; Homo_723,totalSample,immune-Monocyte; Homo_723,totalSample,immune-InfiltrationScore; Homo_723,totalSample,immune-Neutrophil; Homo_723,totalSample,immune-MAIT; Homo_723,totalSample,immune-NK; Homo_723,totalSample,immune-NKT; Homo_723,totalSample,immune-Exhausted; Homo_723,totalSample,immune-CD4_naive; Homo_723,totalSample,immune-DC; Homo_714,totalSample,immune-InfiltrationScore; Homo_714,totalSample,immune-NKT; Homo_714,totalSample,immune-Monocyte; Homo_714,totalSample,immune-Neutrophil; Homo_714,totalSample,immune-MAIT; Homo_714,totalSample,immune-CD4_naive; Homo_714,totalSample,immune-NK; Homo_633,totalSample,immune-InfiltrationScore; Homo_633,totalSample,immune-Monocyte; Homo_633,totalSample,immune-NKT; Homo_633,totalSample,immune-MAIT; Homo_633,totalSample,immune-CD4_naive; Homo_633,totalSample,immune-Th2; MCSA,totalSample,immune-DC; MCSA,totalSample,immune-Macrophage; MCSA,totalSample,immune-Neutrophil; MCSA,totalSample,immune-CD4_T; MCSA,totalSample,immune-CD8_T; MCSA,totalSample,immune-Gamma_delta; MCSA,totalSample,immune-CD4_naive; MCSA,totalSample,immune-Tr1; MCSA,totalSample,immune-nTreg; MCSA,totalSample,immune-iTreg; MCSA,totalSample,immune-Tfh; MCSA,totalSample,immune-Cytotoxic; MCSA,totalSample,immune-MAIT; MCSA,totalSample,immune-Effector_memory; MCSA,totalSample,immune-InfiltrationScore; SRP223445,totalSample,immune-NK; SRP223445,totalSample,immune-Neutrophil; SRP223445,totalSample,immune-CD8_T; SRP223445,totalSample,immune-CD4_naive; SRP223445,totalSample,immune-Th17; SRP223445,totalSample,immune-Cytotoxic; SRP223445,totalSample,immune-InfiltrationScore; ROSMAP,female-excludeAge90+,survival; ROSMAP,male and female-excludeAge90+,survival
LOC112694756	ACOM,male,AD.vs.control; Homo_723,female,AD.vs.MCI; Homo_723,female,MCI.vs.control; Homo_714,male,AD.vs.control; Homo_714,female,AD.vs.MCI; Homo_714,female,MCI.vs.control; Homo_633,female,MCI.vs.control; MCSA,totalSample,age; MCSA,control,age; Emory,totalSample,immune-Monocyte; Emory,totalSample,immune-Neutrophil; Emory,totalSample,immune-CD4_T; Emory,totalSample,immune-CD8_T; Emory,totalSample,immune-Tr1; Emory,totalSample,immune-nTreg; Emory,totalSample,immune-iTreg; Emory,totalSample,immune-Tfh; Emory,totalSample,immune-Exhausted; Emory,totalSample,immune-MAIT; Emory,totalSample,immune-Central_memory; Emory,totalSample,immune-InfiltrationScore; Homo_723,totalSample,immune-nTreg; Homo_723,totalSample,immune-Central_memory; Homo_723,totalSample,immune-CD4_naive; Homo_723,totalSample,immune-Monocyte; Homo_723,totalSample,immune-CD4_T; Homo_723,totalSample,immune-Effector_memory; Homo_723,totalSample,immune-iTreg; Homo_723,totalSample,immune-Tfh; Homo_723,totalSample,immune-Tr1; Homo_723,totalSample,immune-CD8_T; Homo_723,totalSample,immune-Th2; Homo_723,totalSample,immune-Neutrophil; Homo_723,totalSample,immune-DC; Homo_723,totalSample,immune-InfiltrationScore; Homo_714,totalSample,immune-nTreg; Homo_714,totalSample,immune-Monocyte; Homo_714,totalSample,immune-Central_memory; Homo_714,totalSample,immune-Effector_memory; Homo_714,totalSample,immune-CD4_naive; Homo_714,totalSample,immune-CD4_T; Homo_714,totalSample,immune-Tfh; Homo_714,totalSample,immune-Tr1; Homo_714,totalSample,immune-iTreg; Homo_714,totalSample,immune-Neutrophil; Homo_714,totalSample,immune-Th2; Homo_714,totalSample,immune-CD8_T; Homo_714,totalSample,immune-InfiltrationScore; Homo_714,totalSample,immune-DC; Homo_633,totalSample,immune-nTreg; Homo_633,totalSample,immune-Effector_memory; Homo_633,totalSample,immune-Monocyte; Homo_633,totalSample,immune-Central_memory; Homo_633,totalSample,immune-CD4_naive; Homo_633,totalSample,immune-CD4_T; Homo_633,totalSample,immune-Tfh; Homo_633,totalSample,immune-Tr1; Homo_633,totalSample,immune-Neutrophil; Homo_633,totalSample,immune-iTreg; Homo_633,totalSample,immune-InfiltrationScore; Homo_633,totalSample,immune-CD8_T; Homo_633,totalSample,immune-DC; MCSA,totalSample,immune-DC; MCSA,totalSample,immune-Monocyte; MCSA,totalSample,immune-Neutrophil; MCSA,totalSample,immune-CD4_T; MCSA,totalSample,immune-CD8_T; MCSA,totalSample,immune-NKT; MCSA,totalSample,immune-CD4_naive; MCSA,totalSample,immune-Tr1; MCSA,totalSample,immune-nTreg; MCSA,totalSample,immune-iTreg; MCSA,totalSample,immune-Th2; MCSA,totalSample,immune-Th17; MCSA,totalSample,immune-Tfh; MCSA,totalSample,immune-CD8_naive; MCSA,totalSample,immune-Cytotoxic; MCSA,totalSample,immune-Central_memory; MCSA,totalSample,immune-Effector_memory; MCSA,totalSample,immune-InfiltrationScore; SRP223445,totalSample,immune-CD4_naive; SRP223445,totalSample,immune-[illegible]
ZNF90	ACOM,male,AD.vs.control; ACOM,AD,male.vs.female; ROSMAP,female,AD.vs.control; ROSMAP,female,MCI.vs.control; ROSMAP,control,male.vs.female; ACOM,totalSample,age; ROSMAP,AD,last.visit.age; Emory,totalSample,immune-Macrophage; Emory,totalSample,immune-CD4_T; Emory,totalSample,immune-CD8_T; Emory,totalSample,immune-Tr1; Emory,totalSample,immune-iTreg; Emory,totalSample,immune-Tfh
VGLL3	ACOM,male,AD.vs.control; Homo_723,female,AD.vs.control; Homo_723,male,AD.vs.control; Homo_723,female,AD.vs.MCI; Homo_723,male,AD.vs.MCI; Homo_723,female,MCI.vs.control; Homo_714,female,AD.vs.MCI; Homo_633,female,MCI.vs.control; Homo_633,male,MCI.vs.control; MCSA,MCI,age; ROSMAP,MCI,last.visit.age; Emory,totalSample,immune-CD8_T; Homo_723,totalSample,immune-Th17; Homo_723,totalSample,immune-DC; Homo_723,totalSample,immune-CD8_T; Homo_723,totalSample,immune-Cytotoxic; Homo_723,totalSample,immune-Th1; Homo_714,totalSample,immune-Th2; Homo_714,totalSample,immune-CD8_T; ROSMAP,totalSample,immune-InfiltrationScore
EIF1AY	ACOM,male,AD.vs.control; ACOM,AD,male.vs.female; ACOM,control,male.vs.female; Emory,control,male.vs.female; Emory,MCI,male.vs.female; Homo_723,male,AD.vs.control; Homo_723,male,AD.vs.MCI; Homo_723,AD,male.vs.female; Homo_723,control,male.vs.female; Homo_723,MCI,male.vs.female; Homo_714,male,AD.vs.control; Homo_714,male,AD.vs.MCI; Homo_714,AD,male.vs.female; Homo_714,control,male.vs.female; Homo_714,MCI,male.vs.female; Homo_633,control,male.vs.female; Homo_633,MCI,male.vs.female; ROSMAP,female,AD.vs.control; ROSMAP,AD,male.vs.female; ROSMAP,control,male.vs.female; ROSMAP,MCI,male.vs.female; SRP223445,AD,male.vs.female; SRP223445,control,male.vs.female;
ADA2	ACOM,male,AD.vs.control; ACOM,AD,male.vs.female; Homo_723,female,AD.vs.control; Homo_723,male,AD.vs.control; Homo_723,female,AD.vs.MCI; Homo_723,male,AD.vs.MCI; Homo_723,MCI,male.vs.female; Homo_714,male,AD.vs.control; Homo_714,female,AD.vs.MCI; Homo_714,male,AD.vs.MCI; Homo_714,female,MCI.vs.control; Homo_714,AD,male.vs.female; Homo_714,MCI,male.vs.female; Homo_633,MCI,male.vs.female; ACOM,totalSample,age; Emory,MCI,age; MCSA,totalSample,age; MCSA,control,age; Emory,totalSample,immune-DC; Emory,totalSample,immune-NKT; Emory,totalSample,immune-Gamma_delta; Emory,totalSample,immune-Tfh; Emory,totalSample,immune-CD8_naive; Emory,totalSample,immune-Cytotoxic; Emory,totalSample,immune-InfiltrationScore; Homo_723,totalSample,immune-InfiltrationScore; Homo_723,totalSample,immune-CD4_naive; Homo_723,totalSample,immune-Effector_memory; Homo_723,totalSample,immune-Bcell; Homo_723,totalSample,immune-nTreg; Homo_723,totalSample,immune-Tfh; Homo_723,totalSample,immune-DC; Homo_723,totalSample,immune-Central_memory; Homo_714,totalSample,immune-Effector_memory; Homo_714,totalSample,immune-InfiltrationScore; Homo_714,totalSample,immune-CD4_naive; Homo_714,totalSample,immune-nTreg; Homo_714,totalSample,immune-DC; Homo_714,totalSample,immune-Tfh; Homo_714,totalSample,immune-Central_memory; Homo_633,totalSample,immune-Effector_memory; Homo_633,totalSample,immune-InfiltrationScore; Homo_633,totalSample,immune-CD4_naive; Homo_633,totalSample,immune-nTreg; Homo_633,totalSample,immune-Monocyte; Homo_633,totalSample,immune-Tfh; Homo_633,totalSample,immune-CD4_T; Homo_633,totalSample,immune-iTreg; Homo_633,totalSample,immune-Central_memory; MCSA,totalSample,immune-DC; MCSA,totalSample,immune-Monocyte; MCSA,totalSample,immune-CD4_T; MCSA,totalSample,immune-NKT; MCSA,totalSample,immune-Gamma_delta; MCSA,totalSample,immune-CD4_naive; MCSA,totalSample,immune-Tr1; MCSA,totalSample,immune-nTreg; MCSA,totalSample,immune-iTreg; MCSA,totalSample,immune-Th2; MCSA,totalSample,immune-Th17; MCSA,totalSample,immune-Tfh; MCSA,totalSample,immune-Exhausted; MCSA,totalSample,immune-Central_memory; MCSA,totalSample,immune-Effector_memory; MCSA,totalSample,immune-InfiltrationScore; [illegible]
ZNF486	ACOM,male,AD.vs.control; ACOM,AD,male.vs.female; ACOM,totalSample,age; ROSMAP,AD,last.visit.age; Emory,totalSample,immune-Monocyte; Emory,totalSample,immune-Neutrophil; Emory,totalSample,immune-Gamma_delta; Emory,totalSample,immune-iTreg; Emory,totalSample,immune-Cytotoxic; Emory,totalSample,immune-Exhausted; Emory,totalSample,immune-Central_memory; Homo_723,totalSample,immune-Cytotoxic; Homo_723,totalSample,immune-Exhausted; Homo_723,totalSample,immune-Tr1; Homo_723,totalSample,immune-Gamma_delta; Homo_723,totalSample,immune-iTreg; Homo_723,totalSample,immune-NKT; Homo_723,totalSample,immune-Th2; Homo_723,totalSample,immune-CD8_T; Homo_723,totalSample,immune-CD4_naive; Homo_723,totalSample,immune-CD4_T; Homo_723,totalSample,immune-DC; Homo_723,totalSample,immune-Tfh; Homo_723,totalSample,immune-Central_memory; Homo_714,totalSample,immune-Exhausted; Homo_714,totalSample,immune-Cytotoxic; Homo_714,totalSample,immune-Tr1; Homo_714,totalSample,immune-Gamma_delta; Homo_714,totalSample,immune-NKT; Homo_714,totalSample,immune-iTreg; Homo_714,totalSample,immune-CD8_T; Homo_714,totalSample,immune-Th2; Homo_714,totalSample,immune-CD4_T; Homo_714,totalSample,immune-Tfh; Homo_714,totalSample,immune-DC; Homo_714,totalSample,immune-CD4_naive; Homo_714,totalSample,immune-Th17; Homo_633,totalSample,immune-NKT; Homo_633,totalSample,immune-Exhausted; Homo_633,totalSample,immune-Gamma_delta; Homo_633,totalSample,immune-Cytotoxic; Homo_633,totalSample,immune-Tr1; Homo_633,totalSample,immune-CD8_T; Homo_633,totalSample,immune-CD4_T; Homo_633,totalSample,immune-Tfh; Homo_633,totalSample,immune-iTreg; Homo_633,totalSample,immune-Th2; Homo_633,totalSample,immune-Th17; Homo_633,totalSample,immune-DC; Homo_633,totalSample,immune-CD4_naive; Homo_633,totalSample,immune-Central_memory; Homo_633,totalSample,immune-Neutrophil; MCSA,totalSample,immune-DC; MCSA,totalSample,immune-CD4_T; MCSA,totalSample,immune-CD8_T; MCSA,totalSample,immune-Gamma_delta; MCSA,totalSample,immune-CD4_naive; MCSA,totalSample,immune-Tr1; MCSA,totalSample,immune-nTreg; MCSA,totalSample,immune-iTreg; MCSA,totalSample,immune-Th1; MCSA,totalSample,immune-Th17; MCSA,totalSample,immune-Tfh; MCSA,totalSample,immune-CD8_naive; MCSA,totalSample,immune-Cytotoxic; [illegible]
NEUROG2	ACOM,male,AD.vs.control; Homo_723,female,AD.vs.control; Homo_723,female,AD.vs.MCI; ROSMAP,MCI,last.visit.age
PCDHGA7	ACOM,male,AD.vs.control; ACOM,AD,male.vs.female; Homo_714,female,MCI.vs.control; Homo_633,female,MCI.vs.control; ROSMAP,female,AD.vs.MCI; ROSMAP,MCI,male.vs.female
CD302	ACOM,male,AD.vs.control; Homo_723,female,AD.vs.control; Homo_723,male,AD.vs.control; Homo_723,female,AD.vs.MCI; Homo_723,male,AD.vs.MCI; Homo_714,female,AD.vs.control; Homo_714,male,AD.vs.control; Homo_714,female,AD.vs.MCI; Homo_714,male,AD.vs.MCI; Homo_633,female,AD.vs.control; Homo_633,female,AD.vs.MCI; Emory,MCI,age; MCSA,MCI,age; Emory,totalSample,immune-DC; Emory,totalSample,immune-Monocyte; Emory,totalSample,immune-Neutrophil; Emory,totalSample,immune-CD8_T; Emory,totalSample,immune-Gamma_delta; Emory,totalSample,immune-Th1; Emory,totalSample,immune-Tfh; Emory,totalSample,immune-CD8_naive; Emory,totalSample,immune-Cytotoxic; Emory,totalSample,immune-Exhausted; Homo_723,totalSample,immune-Monocyte; Homo_723,totalSample,immune-Neutrophil; Homo_723,totalSample,immune-Gamma_delta; Homo_723,totalSample,immune-Th1; Homo_723,totalSample,immune-InfiltrationScore; Homo_723,totalSample,immune-DC; Homo_714,totalSample,immune-Monocyte; Homo_714,totalSample,immune-Gamma_delta; Homo_714,totalSample,immune-Neutrophil; Homo_714,totalSample,immune-Th1; Homo_714,totalSample,immune-Exhausted; Homo_633,totalSample,immune-Exhausted; Homo_633,totalSample,immune-Monocyte; Homo_633,totalSample,immune-Gamma_delta; Homo_633,totalSample,immune-Macrophage; Homo_633,totalSample,immune-CD8_T; Homo_633,totalSample,immune-Neutrophil; MCSA,totalSample,immune-Monocyte; MCSA,totalSample,immune-Macrophage; MCSA,totalSample,immune-Neutrophil; MCSA,totalSample,immune-CD8_T; MCSA,totalSample,immune-Cytotoxic; MCSA,totalSample,immune-Exhausted; MCSA,totalSample,immune-Effector_memory; ROSMAP,totalSample,immune-Neutrophil; ROSMAP,totalSample,immune-Effector_memory; SRP223445,totalSample,immune-Monocyte; SRP223445,totalSample,immune-CD4_T; ROSMAP,male-excludeAge90+,survival
POU5F1	ACOM,male,AD.vs.control; Homo_723,male,AD.vs.control; Homo_723,male,AD.vs.MCI; Homo_714,male,AD.vs.MCI; Emory,totalSample,immune-CD8_T; Emory,totalSample,immune-Gamma_delta; Emory,totalSample,immune-CD4_naive; Emory,totalSample,immune-Th17; Emory,totalSample,immune-Cytotoxic; Emory,totalSample,immune-Exhausted;
PCDHGA3	ACOM,male,AD.vs.control; Homo_723,female,AD.vs.control; Homo_723,female,MCI.vs.control; Homo_714,female,AD.vs.control; Homo_714,female,MCI.vs.control; Homo_633,female,MCI.vs.control; ROSMAP,female,AD.vs.MCI; ROSMAP,MCI,male.vs.female; ROSMAP,MCI,last.visit.age; Homo_723,totalSample,immune-Monocyte; Homo_633,totalSample,immune-[illegible]
TNFRSF1B	ACOM,male,AD.vs.control; SRP223445,female,AD.vs.control; ACOM,totalSample,age; Emory,totalSample,age; Emory,MCI,age; MCSA,MCI,age; Emory,totalSample,immune-B_cell; Emory,totalSample,immune-Neutrophil; Emory,totalSample,immune-CD8_T; Emory,totalSample,immune-Gamma_delta; Emory,totalSample,immune-Th1; Emory,totalSample,immune-Th17; Emory,totalSample,immune-Tfh; Emory,totalSample,immune-CD8_naive; Emory,totalSample,immune-Cytotoxic; Emory,totalSample,immune-Exhausted; Homo_723,totalSample,immune-Th1; Homo_723,totalSample,immune-Neutrophil; Homo_723,totalSample,immune-DC; Homo_723,totalSample,immune-Bcell; Homo_723,totalSample,immune-InfiltrationScore; Homo_714,totalSample,immune-Neutrophil; Homo_714,totalSample,immune-Th1; Homo_714,totalSample,immune-DC; Homo_714,totalSample,immune-InfiltrationScore; Homo_714,totalSample,immune-Exhausted; Homo_633,totalSample,immune-InfiltrationScore; Homo_633,totalSample,immune-DC; Homo_633,totalSample,immune-Exhausted; Homo_633,totalSample,immune-Neutrophil; Homo_633,totalSample,immune-Th1; Homo_633,totalSample,immune-MAIT; Homo_633,totalSample,immune-CD8_T; MCSA,totalSample,immune-DC; MCSA,totalSample,immune-Bcell; MCSA,totalSample,immune-Monocyte; MCSA,totalSample,immune-Macrophage; MCSA,totalSample,immune-CD4_T; MCSA,totalSample,immune-Gamma_delta; MCSA,totalSample,immune-Tr1; MCSA,totalSample,immune-iTreg; MCSA,totalSample,immune-Th2; MCSA,totalSample,immune-CD8_naive; MCSA,totalSample,immune-MAIT; MCSA,totalSample,immune-InfiltrationScore; SRP223445,totalSample,immune-Exhausted
SEPTIN5	ACOM,male,AD.vs.control; Homo_723,female,AD.vs.control; Homo_723,female,AD.vs.MCI; Homo_723,male,AD.vs.MCI; Homo_723,female,MCI.vs.control; Homo_714,female,AD.vs.control; Homo_714,female,AD.vs.MCI; Homo_714,male,AD.vs.MCI; Homo_714,female,MCI.vs.control; Homo_633,female,AD.vs.control; Homo_633,female,AD.vs.MCI; ROSMAP,female,AD.vs.control; ROSMAP,female,MCI.vs.control; Homo_723,totalSample,immune-DC; Homo_714,totalSample,immune-Th2; SRP223445,totalSample,immune-CD4_naive; SRP223445,totalSample,immune-Exhausted
PRTG	ACOM,male,AD.vs.control; Homo_723,female,AD.vs.control; Homo_723,male,AD.vs.control; Homo_723,female,AD.vs.MCI; Homo_723,male,AD.vs.MCI; Homo_723,AD,male.vs.female; Homo_714,female,AD.vs.control; Homo_714,male,AD.vs.MCI; Homo_633,female,MCI.vs.control; MCSA,MCI,age; Emory,totalSample,immune-CD8_T; Emory,totalSample,immune-Gamma_delta; ROSMAP,totalSample,immune-InfiltrationScore
PLS3	ACOM,male,AD.vs.control; Homo_723,female,AD.vs.control; Homo_723,male,AD.vs.control; Homo_723,female,AD.vs.MCI; Homo_723,male,AD.vs.MCI; Homo_723,female,MCI.vs.control; Homo_723,male,MCI.vs.control; Homo_714,female,AD.vs.control; Homo_714,male,AD.vs.control; Homo_714,female,AD.vs.MCI; Homo_714,male,AD.vs.MCI; Homo_714,female,MCI.vs.control; Homo_714,male,MCI.vs.control; Homo_714,control,male.vs.female; Homo_633,female,AD.vs.control; Homo_633,female,AD.vs.MCI; Homo_633,female,MCI.vs.control; ROSMAP,MCI,male.vs.female; MCSA,MCI,age; Homo_723,totalSample,immune-Exhausted; Homo_723,totalSample,immune-Th17; Homo_723,totalSample,immune-CD8_T; Homo_723,totalSample,immune-Cytotoxic; Homo_723,totalSample,immune-CD4_naive; Homo_714,totalSample,immune-Monocyte; Homo_714,totalSample,immune-CD4_naive; Homo_714,totalSample,immune-Exhausted; Homo_714,totalSample,immune-InfiltrationScore; Homo_633,totalSample,immune-Tfh; Homo_633,totalSample,immune-Effector_memory; Homo_633,totalSample,immune-InfiltrationScore; Homo_633,totalSample,immune-Monocyte; Homo_633,totalSample,immune-Th2; Homo_633,totalSample,immune-CD4_T; Homo_633,totalSample,immune-CD4_naive; Homo_633,totalSample,immune-Central_memory; MCSA,totalSample,immune-DC; MCSA,totalSample,immune-CD4_T; MCSA,totalSample,immune-CD4_naive; MCSA,totalSample,immune-Tr1; MCSA,totalSample,immune-nTreg; MCSA,totalSample,immune-iTreg; MCSA,totalSample,immune-Tfh; MCSA,totalSample,immune-CD8_naive; MCSA,totalSample,immune-Cytotoxic; MCSA,totalSample,immune-Exhausted; MCSA,totalSample,immune-MAIT; MCSA,totalSample,immune-Central_memory; ROSMAP,totalSample,immune-InfiltrationScore; SRP223445,totalSample,immune-NK; SRP223445,totalSample,immune-Neutrophil; SRP223445,totalSample,immune-Exhausted; SRP223445,totalSample,immune-InfiltrationScore

ZNF682	ACOM,male,AD.vs.control; ACOM,AD,male.vs.female; Homo_723,female,AD.vs.control; Homo_723,male,AD.vs.control; Homo_723,male,AD.vs.MCI; Homo_723,female,MCI.vs.control; Homo_714,female,AD.vs.control; Homo_714,male,AD.vs.control; Homo_714,male,AD.vs.MCI; Homo_714,female,MCI.vs.control; Homo_633,female,AD.vs.control; Homo_633,female,AD.vs.MCI; Homo_633,female,MCI.vs.control; ROSMAP,female,AD.vs.control; ROSMAP,control,male.vs.female; ACOM,totalSample,age; MCSA,MCI,age; Emory,totalSample,immune-DC; Emory,totalSample,immune-Macrophage; Emory,totalSample,immune-CD4_T; Emory,totalSample,immune-CD8_T; Emory,totalSample,immune-Tr1; Emory,totalSample,immune-iTreg; Emory,totalSample,immune-Tfh; Emory,totalSample,immune-Central_memory; Emory,totalSample,immune-InfiltrationScore; Homo_723,totalSample,immune-iTreg; Homo_723,totalSample,immune-CD4_naive; Homo_723,totalSample,immune-Tr1; Homo_723,totalSample,immune-Central_memory; Homo_723,totalSample,immune-Th1; Homo_723,totalSample,immune-Tfh; Homo_723,totalSample,immune-CD4_T; Homo_723,totalSample,immune-NKT; Homo_723,totalSample,immune-nTreg; Homo_723,totalSample,immune-Neutrophil; Homo_714,totalSample,immune-CD4_naive; Homo_714,totalSample,immune-Th1; Homo_714,totalSample,immune-NKT; Homo_714,totalSample,immune-Tfh; Homo_714,totalSample,immune-iTreg; Homo_714,totalSample,immune-Tr1; Homo_714,totalSample,immune-CD4_T; Homo_714,totalSample,immune-Central_memory; Homo_714,totalSample,immune-Neutrophil; Homo_633,totalSample,immune-CD4_naive; Homo_633,totalSample,immune-Tfh; Homo_633,totalSample,immune-NKT; Homo_633,totalSample,immune-Th1; Homo_633,totalSample,immune-CD4_T; Homo_633,totalSample,immune-Tr1; Homo_633,totalSample,immune-iTreg; Homo_633,totalSample,immune-Central_memory; MCSA,totalSample,immune-DC; MCSA,totalSample,immune-Bcell; MCSA,totalSample,immune-Monocyte; MCSA,totalSample,immune-Macrophage; MCSA,totalSample,immune-CD4_T; MCSA,totalSample,immune-CD4_naive; MCSA,totalSample,immune-Tr1; MCSA,totalSample,immune-nTreg; MCSA,totalSample,immune-iTreg; MCSA,totalSample,immune-Th2; MCSA,totalSample,immune-Tfh; MCSA,totalSample,immune-CD8_naive; MCSA,totalSample,immune-Cytotoxic; MCSA,totalSample,immune-Exhausted; MCSA,totalSample,immune-MAIT; [illegible]
TGIF2-RAB5IF	ACOM,male,AD.vs.control; ACOM,AD,male.vs.female; Homo_723,male,AD.vs.control; Homo_723,male,AD.vs.MCI; Homo_714,male,AD.vs.control; Homo_714,male,AD.vs.MCI; Homo_633,female,AD.vs.control; Homo_633,female,AD.vs.MCI; ACOM,AD,age; ACOM,totalSample,age
PODXL	ACOM,male,AD.vs.control; ROSMAP,female,AD.vs.control; ROSMAP,male,AD.vs.control; ROSMAP,female,AD.vs.MCI; ROSMAP,male,AD.vs.MCI; ROSMAP,AD,male.vs.female; ROSMAP,control,male.vs.female; Emory,totalSample,immune-CD4_T; Emory,totalSample,immune-Tr1; Emory,totalSample,immune-iTreg; Homo_723,totalSample,immune-InfiltrationScore; Homo_723,totalSample,immune-CD4_naive; Homo_723,totalSample,immune-CD4_T; Homo_723,totalSample,immune-iTreg; Homo_723,totalSample,immune-Tr1; Homo_723,totalSample,immune-Tfh; Homo_723,totalSample,immune-Th2; Homo_723,totalSample,immune-nTreg; Homo_723,totalSample,immune-NKT; Homo_723,totalSample,immune-Th17; Homo_714,totalSample,immune-InfiltrationScore; Homo_714,totalSample,immune-CD4_naive; Homo_714,totalSample,immune-CD4_T; Homo_714,totalSample,immune-iTreg; Homo_714,totalSample,immune-NKT; Homo_714,totalSample,immune-Tfh; Homo_714,totalSample,immune-Tr1; Homo_714,totalSample,immune-Effector_memory; Homo_714,totalSample,immune-Th2; Homo_633,totalSample,immune-CD4_T; Homo_633,totalSample,immune-InfiltrationScore; Homo_633,totalSample,immune-CD4_naive; Homo_633,totalSample,immune-NKT; Homo_633,totalSample,immune-Tfh; Homo_633,totalSample,immune-Tr1; Homo_633,totalSample,immune-iTreg; Homo_633,totalSample,immune-Effector_memory; Homo_633,totalSample,immune-DC; Homo_633,totalSample,immune-Th2; Homo_633,totalSample,immune-Th17; MCSA,totalSample,immune-DC; MCSA,totalSample,immune-CD4_T; MCSA,totalSample,immune-CD4_naive; MCSA,totalSample,immune-Tr1; MCSA,totalSample,immune-nTreg; MCSA,totalSample,immune-iTreg; MCSA,totalSample,immune-Th17; MCSA,totalSample,immune-Tfh; MCSA,totalSample,immune-Cytotoxic; MCSA,totalSample,immune-Exhausted; MCSA,totalSample,immune-Central_memory; SRP223445,totalSample,immune-CD4_naive; SRP223445,totalSample,immune-nTreg; SRP223445,totalSample,immune-InfiltrationScore
FOXH1	ACOM,male,AD.vs.control; ROSMAP,MCI,last.visit.age; ROSMAP,totalSample,immune-InfiltrationScore
BHLHB9	ACOM,male,AD.vs.control; ROSMAP,female,MCI.vs.control; ROSMAP,MCI,last.visit.age; MCSA,totalSample,immune-CD4_T; MCSA,totalSample,immune-CD4_naive; MCSA,totalSample,immune-Tr1; MCSA,totalSample,immune-nTreg; MCSA,totalSample,immune-iTreg; MCSA,totalSample,immune-Tfh; MCSA,totalSample,immune-Central_memory;
PRG2	ACOM,male,AD.vs.control; Homo_723,female,AD.vs.control; Homo_723,male,AD.vs.control; Homo_723,male,AD.vs.MCI; Homo_723,female,MCI.vs.control; Homo_723,male,MCI.vs.control; Homo_714,female,AD.vs.control; Homo_714,male,AD.vs.control; Homo_714,male,AD.vs.MCI; Homo_714,female,MCI.vs.control; Homo_714,male,MCI.vs.control; Homo_633,female,AD.vs.control; Homo_633,female,AD.vs.MCI; Homo_633,female,MCI.vs.control; Homo_633,male,MCI.vs.control; ROSMAP,female,AD.vs.control; MCSA,MCI,age; ROSMAP,AD,sample.age; ROSMAP,MCI,last.visit.age; ROSMAP,MCI,sample.age; Homo_723,totalSample,immune-Monocyte; Homo_714,totalSample,immune-Monocyte; Homo_714,totalSample,immune-Th2; Homo_633,totalSample,immune-Monocyte; Homo_633,totalSample,immune-Th2; MCSA,totalSample,immune-Monocyte; MCSA,totalSample,immune-Macrophage; ROSMAP,totalSample,immune-Monocyte; ROSMAP,totalSample,immune-Central_memory; ROSMAP,totalSample,immune-InfiltrationScore
IL33	ACOM,male,AD.vs.control; Homo_723,female,AD.vs.control; Homo_723,female,AD.vs.MCI; Homo_723,male,AD.vs.MCI; Homo_723,female,MCI.vs.control; Homo_723,male,MCI.vs.control; Homo_714,female,AD.vs.control; Homo_714,female,AD.vs.MCI; Homo_714,male,AD.vs.MCI; Homo_714,female,MCI.vs.control; Homo_714,male,MCI.vs.control; ROSMAP,female,AD.vs.control; ROSMAP,female,MCI.vs.control; MCSA,MCI,age; Emory,totalSample,immune-Gamma_delta; Homo_723,totalSample,immune-Exhausted; Homo_723,totalSample,immune-CD8_T; Homo_723,totalSample,immune-Th17; Homo_723,totalSample,immune-Cytotoxic; Homo_714,totalSample,immune-Monocyte; Homo_714,totalSample,immune-Th17; Homo_633,totalSample,immune-Monocyte; MCSA,totalSample,immune-DC; MCSA,totalSample,immune-Monocyte; ROSMAP,totalSample,immune-InfiltrationScore; SRP223445,totalSample,immune-NK; SRP223445,totalSample,immune-Neutrophil; SRP223445,totalSample,immune-CD4_naive; SRP223445,totalSample,immune-Exhausted;
IFI44	ACOM,male,AD.vs.control; Homo_723,female,AD.vs.control; Homo_723,male,AD.vs.control; Homo_723,female,AD.vs.MCI; Homo_723,male,AD.vs.MCI; Homo_723,female,MCI.vs.control; Homo_723,male,MCI.vs.control; Homo_714,female,AD.vs.control; Homo_714,male,AD.vs.control; Homo_714,female,AD.vs.MCI; Homo_714,male,AD.vs.MCI; Homo_714,female,MCI.vs.control; Homo_714,male,MCI.vs.control; Homo_633,female,AD.vs.control; Homo_633,female,AD.vs.MCI; Homo_633,female,MCI.vs.control; Homo_633,male,MCI.vs.control; ROSMAP,female,AD.vs.control; ACOM,totalSample,age; MCSA,totalSample,age; MCSA,control,age; ROSMAP,MCI,MMSE; Emory,totalSample,immune-Monocyte; Emory,totalSample,immune-InfiltrationScore; Homo_723,totalSample,immune-Monocyte; Homo_723,totalSample,immune-Th2; Homo_723,totalSample,immune-Tfh; Homo_723,totalSample,immune-DC; Homo_723,totalSample,immune-InfiltrationScore; Homo_714,totalSample,immune-Monocyte; Homo_714,totalSample,immune-Th2; Homo_714,totalSample,immune-Tfh; Homo_633,totalSample,immune-Monocyte; Homo_633,totalSample,immune-Th2; Homo_633,totalSample,immune-Bcell; MCSA,totalSample,immune-Monocyte; MCSA,totalSample,immune-CD8_T; MCSA,totalSample,immune-Cytotoxic; MCSA,totalSample,immune-Effector_memory; SRP223445,totalSample,immune-Exhausted; SRP223445,totalSample,immune-
FIGNL2	ACOM,male,AD.vs.control; Homo_723,female,AD.vs.control; Homo_723,male,AD.vs.control; Homo_723,female,AD.vs.MCI; Homo_723,male,AD.vs.MCI; Homo_714,male,AD.vs.control; Homo_714,male,AD.vs.MCI; Homo_633,female,AD.vs.control; Homo_633,female,AD.vs.MCI; ROSMAP,female,AD.vs.control; ROSMAP,male,AD.vs.control; ROSMAP,female,MCI.vs.control; ACOM,totalSample,age; MCSA,totalSample,age; MCSA,control,age; ROSMAP,totalSample,MMSE; Emory,totalSample,immune-CD4_T; Emory,totalSample,immune-Tr1; Emory,totalSample,immune-iTreg; Homo_723,totalSample,immune-nTreg; Homo_723,totalSample,immune-iTreg; Homo_723,totalSample,immune-CD4_naive; Homo_723,totalSample,immune-Tfh; Homo_723,totalSample,immune-CD4_T; Homo_723,totalSample,immune-Central_memory; Homo_723,totalSample,immune-Monocyte; Homo_723,totalSample,immune-CD8_T; Homo_723,totalSample,immune-InfiltrationScore; Homo_723,totalSample,immune-Th2; Homo_723,totalSample,immune-Tr1; Homo_723,totalSample,immune-Macrophage; Homo_714,totalSample,immune-nTreg; Homo_714,totalSample,immune-Tfh; Homo_714,totalSample,immune-InfiltrationScore; Homo_714,totalSample,immune-CD4_naive; Homo_714,totalSample,immune-iTreg; Homo_714,totalSample,immune-Effector_memory; Homo_714,totalSample,immune-CD4_T; Homo_714,totalSample,immune-Monocyte; Homo_714,totalSample,immune-Central_memory; Homo_714,totalSample,immune-Tr1; Homo_714,totalSample,immune-Macrophage; Homo_633,totalSample,immune-nTreg; Homo_633,totalSample,immune-InfiltrationScore; Homo_633,totalSample,immune-Tfh; Homo_633,totalSample,immune-CD4_naive; Homo_633,totalSample,immune-Effector_memory; Homo_633,totalSample,immune-Monocyte; Homo_633,totalSample,immune-CD4_T; Homo_633,totalSample,immune-iTreg; Homo_633,totalSample,immune-Tr1; Homo_633,totalSample,immune-Central_memory; MCSA,totalSample,immune-DC; MCSA,totalSample,immune-Monocyte; MCSA,totalSample,immune-Neutrophil; MCSA,totalSample,immune-CD4_T; MCSA,totalSample,immune-CD8_T; MCSA,totalSample,immune-CD4_naive; MCSA,totalSample,immune-Tr1; MCSA,totalSample,immune-nTreg; MCSA,totalSample,immune-iTreg; MCSA,totalSample,immune-Th2; MCSA,totalSample,immune-Th17; MCSA,totalSample,immune-Tfh; MCSA,totalSample,immune-Central_memory; MCSA,totalSample,immune- [illegible]
PRDM8	ACOM,male,AD.vs.control; Homo_723,female,AD.vs.control; Homo_723,female,AD.vs.MCI; Homo_723,female,MCI.vs.control; Homo_723,male,MCI.vs.control; Homo_723,MCI,male.vs.female; Homo_714,male,AD.vs.control; Homo_714,female,AD.vs.MCI; Homo_714,female,MCI.vs.control; Homo_714,male,MCI.vs.control; Homo_714,MCI,male.vs.female; Homo_633,female,MCI.vs.control; Homo_633,MCI,male.vs.female; ROSMAP,female,AD.vs.control; ROSMAP,male,AD.vs.control; ROSMAP,female,MCI.vs.control; MCSA,totalSample,age; MCSA,control,age; ROSMAP,MCI,last.visit.age; Emory,totalSample,immune-CD4_T; Emory,totalSample,immune-Tr1; Emory,totalSample,immune-iTreg; Emory,totalSample,immune-Th17; Emory,totalSample,immune-InfiltrationScore; Homo_723,totalSample,immune-Monocyte; Homo_723,totalSample,immune-Central_memory; Homo_723,totalSample,immune-CD4_naive; Homo_714,totalSample,immune-NKT; Homo_714,totalSample,immune-Monocyte; Homo_714,totalSample,immune-Th1; Homo_633,totalSample,immune-Monocyte; Homo_633,totalSample,immune-NKT; Homo_633,totalSample,immune-Th1; Homo_633,totalSample,immune-iTreg; MCSA,totalSample,immune-CD4_T; MCSA,totalSample,immune-Gamma_delta; MCSA,totalSample,immune-CD4_naive; MCSA,totalSample,immune-Tr1; MCSA,totalSample,immune-nTreg; MCSA,totalSample,immune-iTreg; MCSA,totalSample,immune-Th17; MCSA,totalSample,immune-Tfh; MCSA,totalSample,immune-CD8_naive; MCSA,totalSample,immune-Cytotoxic; MCSA,totalSample,immune-Exhausted; MCSA,totalSample,immune-Central_memory; MCSA,totalSample,immune-Effector_memory; SRP223445,totalSample,immune-CD4_naive
NFIB	ACOM,male,AD.vs.control; Homo_723,female,AD.vs.control; Homo_723,male,AD.vs.control; Homo_723,female,AD.vs.MCI; Homo_723,male,AD.vs.MCI; Homo_723,female,MCI.vs.control; Homo_723,male,MCI.vs.control; Homo_723,control,male.vs.female; Homo_714,female,AD.vs.control; Homo_714,male,AD.vs.control; Homo_714,female,AD.vs.MCI; Homo_714,male,AD.vs.MCI; Homo_714,female,MCI.vs.control; Homo_714,male,MCI.vs.control; Homo_714,control,male.vs.female; Homo_633,female,AD.vs.control; Homo_633,female,AD.vs.MCI; Homo_633,female,MCI.vs.control; Homo_633,male,MCI.vs.control; ACOM,totalSample,age; MCSA,MCI,age; ROSMAP,AD,last.visit.age; ROSMAP,AD,sample.age; Homo_723,totalSample,immune-DC; Homo_723,totalSample,immune-Th17; MCSA,totalSample,immune-Th17; SRP223445,totalSample,immune-Neutrophil; SRP223445,totalSample,immune-InfiltrationScore
TIE1	ACOM,male,AD.vs.control; Homo_723,male,AD.vs.control; Homo_714,male,AD.vs.control; ROSMAP,female,AD.vs.control; ROSMAP,male,AD.vs.control; ACOM,totalSample,age; MCSA,totalSample,age; MCSA,control,age; Emory,totalSample,immune-Th17; Homo_723,totalSample,immune-InfiltrationScore; MCSA,totalSample,immune-DC; MCSA,totalSample,immune-Monocyte; MCSA,totalSample,immune-Th2; MCSA,totalSample,immune-Effector_memory; MCSA,totalSample,immune-Central_memory; ROSMAP,totalSample,immune-InfiltrationScore; SRP223445,totalSample,immune-CD4_naive
HSPA6	ACOM,male,AD.vs.control; ROSMAP,female,AD.vs.control; ROSMAP,male,AD.vs.control; ROSMAP,female,MCI.vs.control; ACOM,totalSample,age; Emory,totalSample,age; Emory,MCI,age; MCSA,MCI,age; ROSMAP,MCI,sample.age; Emory,totalSample,immune-B_cell; Emory,totalSample,immune-Monocyte; Emory,totalSample,immune-Neutrophil; Emory,totalSample,immune-CD4_T; Emory,totalSample,immune-CD8_T; Emory,totalSample,immune-Gamma_delta; Emory,totalSample,immune-Th1; Emory,totalSample,immune-Th17; Emory,totalSample,immune-Tfh; Emory,totalSample,immune-CD8_naive; Emory,totalSample,immune-Cytotoxic; Emory,totalSample,immune-Exhausted; Emory,totalSample,immune-MAIT; Homo_723,totalSample,immune-Neutrophil; Homo_723,totalSample,immune-Th1; Homo_723,totalSample,immune-CD4_naive; Homo_723,totalSample,immune-CD4_T; Homo_723,totalSample,immune-Tfh; Homo_723,totalSample,immune-iTreg; Homo_723,totalSample,immune-Central_memory; Homo_723,totalSample,immune-Tr1; Homo_723,totalSample,immune-CD8_T; Homo_723,totalSample,immune-MAIT; Homo_714,totalSample,immune-Neutrophil; Homo_714,totalSample,immune-CD4_naive; Homo_714,totalSample,immune-Exhausted; Homo_714,totalSample,immune-Th1; Homo_714,totalSample,immune-MAIT; Homo_714,totalSample,immune-CD4_T; Homo_714,totalSample,immune-CD8_T; Homo_714,totalSample,immune-Central_memory; Homo_714,totalSample,immune-Tfh; Homo_714,totalSample,immune-iTreg; Homo_633,totalSample,immune-Neutrophil; Homo_633,totalSample,immune-CD8_T; Homo_633,totalSample,immune-Exhausted; Homo_633,totalSample,immune-MAIT; Homo_633,totalSample,immune-CD4_naive; Homo_633,totalSample,immune-Tfh; Homo_633,totalSample,immune-Macrophage; Homo_633,totalSample,immune-Central_memory; Homo_633,totalSample,immune-CD4_T; Homo_633,totalSample,immune-Th1; Homo_633,totalSample,immune-DC; MCSA,totalSample,immune-Bcell; MCSA,totalSample,immune-Monocyte; MCSA,totalSample,immune-Macrophage; MCSA,totalSample,immune-Neutrophil; MCSA,totalSample,immune-CD4_T; MCSA,totalSample,immune-Gamma_delta; MCSA,totalSample,immune-CD4_naive; MCSA,totalSample,immune-Tr1; MCSA,totalSample,immune-nTreg; MCSA,totalSample,immune-iTreg; MCSA,totalSample,immune-Th2; MCSA,totalSample,immune-Tfh; MCSA,totalSample,immune-CD8_naive; MCSA,totalSample,immune-MAIT; MCSA,totalSample,immune-Central_memory; ROSMAP,totalSample,immune-Th17; ROSMAP,totalSample,immune-Effector_memory; ROSMAP,totalSample,immune-InfiltrationScore; SRP223445,totalSample,immune-Neutrophil; SRP223445,totalSample,immune-CD4_naive; SRP223445,totalSample,immune-Exhausted; ROSMAP,female-excludeAge90+,survival; [illegible]
MPPED2	ACOM,male,AD.vs.control; Homo_723,female,AD.vs.control; Homo_723,female,AD.vs.MCI; Homo_723,male,AD.vs.MCI; Homo_723,male,MCI.vs.control; Homo_714,female,AD.vs.control; Homo_714,male,AD.vs.control; Homo_714,female,AD.vs.MCI; Homo_714,male,AD.vs.MCI; Homo_633,female,AD.vs.MCI; Homo_633,female,MCI.vs.control; ACOM,totalSample,age; Emory,MCI,age; MCSA,totalSample,age; Emory,totalSample,immune-Th17; Homo_723,totalSample,immune-Th17; Homo_714,totalSample,immune-Th17; MCSA,totalSample,immune-CD4_T; MCSA,totalSample,immune-CD4_naive; MCSA,totalSample,immune-Tr1; MCSA,totalSample,immune-iTreg;
FUT9	ACOM,male,AD.vs.control; Homo_723,female,AD.vs.control; Homo_723,female,AD.vs.MCI; Homo_723,AD,male.vs.female; ACOM,AD,age; ROSMAP,MCI,last.visit.age; Emory,totalSample,immune-CD8_T; ROSMAP,totalSample,immune-InfiltrationScore; ROSMAP,male-includeAge90+,survival
CLEC18B	ACOM,male,AD.vs.control; Homo_723,female,AD.vs.control; Homo_723,male,AD.vs.control; Homo_723,male,AD.vs.MCI; Homo_723,female,MCI.vs.control; Homo_723,male,MCI.vs.control; Homo_714,female,AD.vs.control; Homo_714,female,MCI.vs.control; Homo_714,male,MCI.vs.control; Homo_633,female,MCI.vs.control; Homo_633,MCI,male.vs.female; ROSMAP,female,AD.vs.control; ROSMAP,male,AD.vs.control; ROSMAP,female,AD.vs.MCI; ACOM,totalSample,age; MCSA,totalSample,age; MCSA,control,age; MCSA,MCI,age; Emory,totalSample,immune-Neutrophil; Emory,totalSample,immune-CD8_T; Emory,totalSample,immune-Gamma_delta; Emory,totalSample,immune-Th17; Emory,totalSample,immune-Exhausted; Homo_723,totalSample,immune-Monocyte; Homo_723,totalSample,immune-CD8_T; Homo_723,totalSample,immune-Exhausted; Homo_723,totalSample,immune-Cytotoxic; Homo_714,totalSample,immune-Monocyte; Homo_633,totalSample,immune-Monocyte; MCSA,totalSample,immune-DC; MCSA,totalSample,immune-Monocyte; MCSA,totalSample,immune-Macrophage; MCSA,totalSample,immune-CD8_T; MCSA,totalSample,immune-Gamma_delta; MCSA,totalSample,immune-iTreg; MCSA,totalSample,immune-Th2; MCSA,totalSample,immune-Th17; MCSA,totalSample,immune-Cytotoxic; MCSA,totalSample,immune-MAIT; MCSA,totalSample,immune-Effector_memory;
PCDH8	ACOM,male,AD.vs.control; Homo_723,female,AD.vs.control; Homo_723,female,AD.vs.MCI; Homo_633,female,MCI.vs.control; SRP223445,totalSample,immune-Neutrophil
HEY1	ACOM,male,AD.vs.control; Homo_723,female,AD.vs.control; Homo_723,male,AD.vs.control; Homo_723,female,AD.vs.MCI; Homo_723,male,AD.vs.MCI; Homo_723,female,MCI.vs.control; Homo_714,female,AD.vs.control; Homo_714,male,AD.vs.control; Homo_714,female,AD.vs.MCI; Homo_714,male,AD.vs.MCI; Homo_714,female,MCI.vs.control; Homo_633,female,AD.vs.control; Homo_633,female,AD.vs.MCI; Homo_633,male,MCI.vs.control; ACOM,totalSample,age; Emory,totalSample,immune-Neutrophil; Emory,totalSample,immune-Gamma_delta; Emory,totalSample,immune-Cytotoxic; Emory,totalSample,immune-Exhausted; Homo_723,totalSample,immune-Effector_memory; Homo_723,totalSample,immune-CD8_T; Homo_723,totalSample,immune-Th17; Homo_714,totalSample,immune-Effector_memory; Homo_714,totalSample,immune-Th2; Homo_714,totalSample,immune-CD8_T; Homo_714,totalSample,immune-Exhausted; Homo_633,totalSample,immune-Effector_memory; Homo_633,totalSample,immune-Neutrophil; MCSA,totalSample,immune-DC; MCSA,totalSample,immune-Monocyte; MCSA,totalSample,immune-Neutrophil; MCSA,totalSample,immune-CD4_T; MCSA,totalSample,immune-CD8_T; MCSA,totalSample,immune-NKT; MCSA,totalSample,immune-CD4_naive; MCSA,totalSample,immune-nTreg; MCSA,totalSample,immune-Th2; MCSA,totalSample,immune-Tfh; MCSA,totalSample,immune-Central_memory; MCSA,totalSample,immune-Effector_memory; SRP223445,totalSample,immune-CD4_naive; SRP223445,totalSample,immune-Exhausted
HSPE1-	ACOM,male,AD.vs.control; ROSMAP,female,AD.vs.control; ROSMAP,female,MCI.vs.control; ROSMAP,AD,sample.age; ROSMAP,MCI,last.visit.age; ROSMAP,MCI,sample.age; Emory,totalSample,immune-Monocyte; ROSMAP,totalSample,immune-CD4_T; ROSMAP,totalSample,immune-Th17
TNC	ACOM,male,AD.vs.control; Homo_723,female,AD.vs.control; Homo_723,male,AD.vs.control; Homo_723,female,AD.vs.MCI; Homo_723,male,AD.vs.MCI; Homo_714,female,AD.vs.control; Homo_714,male,AD.vs.control; Homo_714,female,AD.vs.MCI; Homo_714,male,AD.vs.MCI; Homo_633,female,AD.vs.MCI; Homo_633,female,MCI.vs.control; ROSMAP,female,AD.vs.MCI; ROSMAP,male,AD.vs.MCI; ACOM,totalSample,age; Homo_723,totalSample,immune-Exhausted; Homo_633,totalSample,immune-InfiltrationScore; MCSA,totalSample,immune-Exhausted; ROSMAP,totalSample,immune-InfiltrationScore; SRP223445,totalSample,immune-CD4_naive
LY75-CD302	ACOM,male,AD.vs.control; MCSA,MCI,age; Homo_723,totalSample,immune-Exhausted; Homo_723,totalSample,immune-Th17; Homo_723,totalSample,immune-Cytotoxic; Homo_723,totalSample,immune-CD8_T; Homo_723,totalSample,immune-Th2; Homo_723,totalSample,immune-DC; Homo_714,totalSample,immune-Th17; Homo_714,totalSample,immune-Th2; Homo_633,totalSample,immune-Th2; Homo_633,totalSample,immune-DC; Homo_633,totalSample,immune-Th17; Homo_633,totalSample,immune-Monocyte; Homo_633,totalSample,immune-Cytotoxic; Homo_633,totalSample,immune-Exhausted; MCSA,totalSample,immune-DC; MCSA,totalSample,immune-CD4_T; MCSA,totalSample,immune-CD8_T; MCSA,totalSample,immune-CD4_naive; MCSA,totalSample,immune-Tr1; MCSA,totalSample,immune-nTreg; MCSA,totalSample,immune-iTreg; MCSA,totalSample,immune-CD8_naive; MCSA,totalSample,immune-Cytotoxic; MCSA,totalSample,immune-Exhausted; MCSA,totalSample,immune-Central_memory; ROSMAP,totalSample,immune-Neutrophil; ROSMAP,totalSample,immune-Effector_memory
CSNK1E	ACOM,male,AD.vs.control; Homo_723,female,AD.vs.control; Homo_723,male,AD.vs.control; Homo_723,female,AD.vs.MCI; Homo_723,male,AD.vs.MCI; Homo_633,female,AD.vs.control; Homo_633,female,AD.vs.MCI; Emory,totalSample,immune-Monocyte; Emory,totalSample,immune-Neutrophil; Emory,totalSample,immune-CD4_T; Emory,totalSample,immune-CD8_T; Emory,totalSample,immune-Tr1; Emory,totalSample,immune-iTreg; Emory,totalSample,immune-Th1; Emory,totalSample,immune-CD8_naive; Emory,totalSample,immune-Exhausted; Emory,totalSample,immune-InfiltrationScore; Homo_723,totalSample,immune-DC; Homo_723,totalSample,immune-Neutrophil; Homo_723,totalSample,immune-nTreg; Homo_723,totalSample,immune-Monocyte; Homo_723,totalSample,immune-CD8_T; Homo_723,totalSample,immune-InfiltrationScore; Homo_714,totalSample,immune-DC; Homo_714,totalSample,immune-InfiltrationScore; Homo_714,totalSample,immune-Neutrophil; Homo_714,totalSample,immune-Effector_memory; Homo_714,totalSample,immune-nTreg; Homo_714,totalSample,immune-Monocyte; Homo_633,totalSample,immune-DC; Homo_633,totalSample,immune-InfiltrationScore; Homo_633,totalSample,immune-Neutrophil; Homo_633,totalSample,immune-Effector_memory; Homo_633,totalSample,immune-nTreg; Homo_633,totalSample,immune-Monocyte; Homo_633,totalSample,immune-CD8_T; MCSA,totalSample,immune-DC; MCSA,totalSample,immune-Monocyte; MCSA,totalSample,immune-Neutrophil; MCSA,totalSample,immune-CD8_T; MCSA,totalSample,immune-NKT; MCSA,totalSample,immune-Th2; MCSA,totalSample,immune-Tfh; MCSA,totalSample,immune-Central_memory; MCSA,totalSample,immune-
NOS3	ACOM,male,AD.vs.control; Homo_723,female,AD.vs.control; Homo_723,male,AD.vs.control; Homo_723,female,AD.vs.MCI; Homo_723,male,AD.vs.MCI; Homo_723,MCI,male.vs.female; Homo_714,female,AD.vs.control; Homo_714,male,AD.vs.control; Homo_714,female,AD.vs.MCI; Homo_714,male,AD.vs.MCI; Homo_714,MCI,male.vs.female; Homo_633,female,AD.vs.control; Homo_633,female,AD.vs.MCI; Homo_633,female,MCI.vs.control; Homo_633,male,MCI.vs.control; Homo_633,control,male.vs.female; Homo_633,MCI,male.vs.female; ROSMAP,female,AD.vs.control; ROSMAP,male,AD.vs.control; SRP223445,female,AD.vs.control; ACOM,totalSample,age; MCSA,totalSample,age; MCSA,control,age; Emory,totalSample,immune-Macrophage; Emory,totalSample,immune-Neutrophil; Emory,totalSample,immune-CD4_T; Emory,totalSample,immune-CD8_T; Emory,totalSample,immune-CD4_naive; Emory,totalSample,immune-Tr1; Emory,totalSample,immune-nTreg; Emory,totalSample,immune-iTreg; Emory,totalSample,immune-Th17; Emory,totalSample,immune-Tfh; Emory,totalSample,immune-CD8_naive; Emory,totalSample,immune-Central_memory; Emory,totalSample,immune-InfiltrationScore; Homo_723,totalSample,immune-DC; Homo_723,totalSample,immune-Monocyte; Homo_723,totalSample,immune-Central_memory; Homo_723,totalSample,immune-nTreg; Homo_723,totalSample,immune-Neutrophil; Homo_723,totalSample,immune-CD4_naive; Homo_714,totalSample,immune-DC; Homo_714,totalSample,immune-Monocyte; Homo_714,totalSample,immune-nTreg; Homo_714,totalSample,immune-Th2; Homo_633,totalSample,immune-Monocyte; Homo_633,totalSample,immune-nTreg; Homo_633,totalSample,immune-DC; Homo_633,totalSample,immune-Central_memory; Homo_633,totalSample,immune-InfiltrationScore; Homo_633,totalSample,immune-iTreg; MCSA,totalSample,immune-DC; MCSA,totalSample,immune-Monocyte; MCSA,totalSample,immune-Neutrophil; MCSA,totalSample,immune-CD4_T; MCSA,totalSample,immune-CD8_T; MCSA,totalSample,immune-NKT; MCSA,totalSample,immune-CD4_naive; MCSA,totalSample,immune-Tr1; MCSA,totalSample,immune-nTreg; MCSA,totalSample,immune-Th2; MCSA,totalSample,immune-Tfh; MCSA,totalSample,immune-Cytotoxic; MCSA,totalSample,immune-Central_memory; MCSA,totalSample,immune-Effector_memory; MCSA,totalSample,immune-InfiltrationScore; SRP223445,totalSample,immune-Neutrophil; [illegible]

SLFN13	ACOM,male,AD.vs.control; Homo_723,female,AD.vs.control; Homo_723,female,AD.vs.MCI; Homo_723,female,MCI.vs.control; Homo_723,AD,male.vs.female; Homo_723,MCI,male.vs.female; Homo_714,female,AD.vs.control; Homo_714,male,AD.vs.MCI; Homo_714,female,MCI.vs.control; Homo_714,AD,male.vs.female; Homo_714,MCI,male.vs.female; Homo_633,female,AD.vs.control; Homo_633,female,AD.vs.MCI; Homo_633,female,MCI.vs.control; Homo_633,MCI,male.vs.female; ACOM,totalSample,age; Emory,MCI,age; MCSA,MCI,age; ROSMAP,MCI,last.visit.age; Emory,totalSample,immune-Macrophage; Emory,totalSample,immune-NK; Emory,totalSample,immune-Neutrophil; Emory,totalSample,immune-CD4_T; Emory,totalSample,immune-CD8_T; Emory,totalSample,immune-Tr1; Emory,totalSample,immune-iTreg; Emory,totalSample,immune-Exhausted; Emory,totalSample,immune-Effector_memory; Emory,totalSample,immune-InfiltrationScore; Homo_723,totalSample,immune-Monocyte; Homo_723,totalSample,immune-CD4_naive; Homo_723,totalSample,immune-InfiltrationScore; Homo_723,totalSample,immune-CD4_T; Homo_723,totalSample,immune-Th1; Homo_723,totalSample,immune-Central_memory; Homo_723,totalSample,immune-Neutrophil; Homo_723,totalSample,immune-Tfh; Homo_723,totalSample,immune-Th2; Homo_723,totalSample,immune-Tr1; Homo_723,totalSample,immune-NK; Homo_723,totalSample,immune-NKT; Homo_714,totalSample,immune-CD4_naive; Homo_714,totalSample,immune-InfiltrationScore; Homo_714,totalSample,immune-NKT; Homo_714,totalSample,immune-Monocyte; Homo_714,totalSample,immune-Th1; Homo_714,totalSample,immune-iTreg; Homo_714,totalSample,immune-Neutrophil; Homo_714,totalSample,immune-CD4_T; Homo_633,totalSample,immune-InfiltrationScore; Homo_633,totalSample,immune-Monocyte; Homo_633,totalSample,immune-NKT; Homo_633,totalSample,immune-CD4_naive; Homo_633,totalSample,immune-Tfh; Homo_633,totalSample,immune-iTreg; Homo_633,totalSample,immune-Th1; Homo_633,totalSample,immune-CD4_T; MCSA,totalSample,immune-DC; MCSA,totalSample,immune-Macrophage; MCSA,totalSample,immune-CD4_T; MCSA,totalSample,immune-CD4_naive; MCSA,totalSample,immune-Tr1; MCSA,totalSample,immune-nTreg; MCSA,totalSample,immune-iTreg; MCSA,totalSample,immune-Th17; MCSA,totalSample,immune-Tfh; MCSA,totalSample,immune-CD8_naive; MCSA,totalSample,immune-Exhausted; MCSA,totalSample,immune-MAIT; MCSA,totalSample,immune-Central_memory; MCSA,totalSample,immune-Effector_memory; MCSA,totalSample,immune-InfiltrationScore; ROSMAP,totalSample,immune-Th17; ROSMAP,totalSample,immune-Effector_memory; SRP223445,totalSample,immune-NK; SRP223445,totalSample,immune-Th17; [illegible]
INHBB	ACOM,male,AD.vs.control; ROSMAP,female,AD.vs.control; ACOM,totalSample,age; Emory,MCI,age; ROSMAP,AD,sample.age; Emory,totalSample,immune-NK; Emory,totalSample,immune-Neutrophil; Emory,totalSample,immune-CD8_T; Emory,totalSample,immune-Gamma_delta; Emory,totalSample,immune-Tfh; Emory,totalSample,immune-CD8_naive; Emory,totalSample,immune-Exhausted; Homo_723,totalSample,immune-Neutrophil; Homo_714,totalSample,immune-Neutrophil; Homo_633,totalSample,immune-Neutrophil; Homo_633,totalSample,immune-Exhausted; MCSA,totalSample,immune-Neutrophil; MCSA,totalSample,immune-Gamma_delta; MCSA,totalSample,immune-Th1; MCSA,totalSample,immune-Exhausted
TRIM71	ACOM,male,AD.vs.control; Homo_723,female,AD.vs.control; Homo_723,female,AD.vs.MCI; Homo_714,female,AD.vs.control; Homo_714,female,AD.vs.MCI; Homo_633,female,AD.vs.control; Homo_633,female,AD.vs.MCI; ACOM,totalSample,age; Emory,totalSample,immune-CD8_T; Emory,totalSample,immune-Gamma_delta; Homo_723,totalSample,immune-Th17; Homo_714,totalSample,immune-Monocyte; Homo_633,totalSample,immune-Gamma_delta; Homo_633,totalSample,immune-CD8_T; MCSA,totalSample,immune-iTreg; MCSA,totalSample,immune-Th17; MCSA,totalSample,immune-Cytotoxic; ROSMAP,totalSample,immune-InfiltrationScore
ZNF560	ACOM,male,AD.vs.control; Homo_723,female,AD.vs.control; Homo_723,male,AD.vs.control; Homo_723,female,MCI.vs.control; Homo_714,female,MCI.vs.control; Homo_633,female,MCI.vs.control
ZNF300	ACOM,male,AD.vs.control; Homo_723,female,AD.vs.control; Homo_723,female,MCI.vs.control; Homo_723,control,male.vs.female; Homo_714,female,AD.vs.control; Homo_714,female,MCI.vs.control; Homo_714,control,male.vs.female; Homo_633,female,AD.vs.control; Homo_633,female,MCI.vs.control; Homo_633,control,male.vs.female; ROSMAP,female,MCI.vs.control; ROSMAP,control,male.vs.female; MCSA,MCI,age; ROSMAP,MCI,sample.age; ROSMAP,AD,MMSE; Emory,totalSample,immune-Macrophage; Emory,totalSample,immune-Neutrophil; Emory,totalSample,immune-CD4_T; Emory,totalSample,immune-Tr1; Emory,totalSample,immune-iTreg; Emory,totalSample,immune-Tfh; Emory,totalSample,immune-CD8_naive; Emory,totalSample,immune-Central_memory; Emory,totalSample,immune-InfiltrationScore; Homo_723,totalSample,immune-Central_memory; Homo_723,totalSample,immune-CD4_naive; Homo_723,totalSample,immune-iTreg; Homo_723,totalSample,immune-Th1; Homo_723,totalSample,immune-Tfh; Homo_723,totalSample,immune-Tr1; Homo_723,totalSample,immune-CD4_T; Homo_723,totalSample,immune-NKT; Homo_714,totalSample,immune-CD4_naive; Homo_714,totalSample,immune-iTreg; Homo_714,totalSample,immune-NKT; Homo_714,totalSample,immune-Th1; Homo_714,totalSample,immune-Tfh; Homo_714,totalSample,immune-CD4_T; Homo_714,totalSample,immune-Tr1; Homo_633,totalSample,immune-NKT; Homo_633,totalSample,immune-Th1; Homo_633,totalSample,immune-CD4_naive; Homo_633,totalSample,immune-Tfh; Homo_633,totalSample,immune-iTreg; Homo_633,totalSample,immune-CD4_T; Homo_633,totalSample,immune-Tr1; MCSA,totalSample,immune-DC; MCSA,totalSample,immune-Bcell; MCSA,totalSample,immune-Monocyte; MCSA,totalSample,immune-Macrophage; MCSA,totalSample,immune-CD4_T; MCSA,totalSample,immune-CD4_naive; MCSA,totalSample,immune-Tr1; MCSA,totalSample,immune-nTreg; MCSA,totalSample,immune-iTreg; MCSA,totalSample,immune-Th17; MCSA,totalSample,immune-Tfh; MCSA,totalSample,immune-CD8_naive; MCSA,totalSample,immune-Cytotoxic; MCSA,totalSample,immune-Exhausted; MCSA,totalSample,immune-MAIT; MCSA,totalSample,immune-Central_memory; ROSMAP,totalSample,immune-Th17; ROSMAP,totalSample,immune-Central_memory; ROSMAP,totalSample,immune-InfiltrationScore
CRABP1	ACOM,male,AD.vs.control; Homo_723,female,AD.vs.MCI; Homo_723,female,MCI.vs.control; ROSMAP,MCI,MMSE; Homo_723,totalSample,immune-Th17
PLEKHA4	ACOM,male,AD.vs.control; Homo_723,female,AD.vs.control; Homo_723,male,AD.vs.control; Homo_723,female,AD.vs.MCI; Homo_723,male,AD.vs.MCI; Homo_723,female,MCI.vs.control; Homo_723,male,MCI.vs.control; Homo_723,control,male.vs.female; Homo_714,female,AD.vs.control; Homo_714,male,AD.vs.control; Homo_714,female,AD.vs.MCI; Homo_714,male,AD.vs.MCI; Homo_714,female,MCI.vs.control; Homo_714,male,MCI.vs.control; Homo_714,control,male.vs.female; Homo_633,female,AD.vs.control; Homo_633,female,AD.vs.MCI; Homo_633,female,MCI.vs.control; SRP223445,female,AD.vs.control; ACOM,totalSample,age; MCSA,MCI,age; ROSMAP,AD,last.visit.age; ROSMAP,MCI,last.visit.age; Emory,totalSample,immune-B_cell; Emory,totalSample,immune-CD8_naive; Homo_723,totalSample,immune-DC; Homo_723,totalSample,immune-Neutrophil; Homo_723,totalSample,immune-Monocyte; Homo_723,totalSample,immune-nTreg; Homo_723,totalSample,immune-Central_memory; Homo_714,totalSample,immune-DC; Homo_714,totalSample,immune-Neutrophil; Homo_633,totalSample,immune-Monocyte; Homo_633,totalSample,immune-nTreg; MCSA,totalSample,immune-DC; MCSA,totalSample,immune-Monocyte; MCSA,totalSample,immune-Neutrophil; MCSA,totalSample,immune-CD8_T; MCSA,totalSample,immune-Th2; MCSA,totalSample,immune-Tfh; MCSA,totalSample,immune-Cytotoxic; MCSA,totalSample,immune-Central_memory; MCSA,totalSample,immune-Effector_memory; MCSA,totalSample,immune-InfiltrationScore; ROSMAP,totalSample,immune-InfiltrationScore; SRP223445,totalSample,immune-Neutrophil; SRP223445,totalSample,immune-nTreg; ROSMAP,male-excludeAge90+,survival
FXYD5	ACOM,male,AD.vs.control; Homo_723,control,male.vs.female; Homo_714,control,male.vs.female; Homo_633,control,male.vs.female; ACOM,totalSample,age; MCSA,totalSample,age; MCSA,control,age; MCSA,MCI,age; ROSMAP,MCI,last.visit.age; Emory,totalSample,immune-Monocyte; Emory,totalSample,immune-Macrophage; Emory,totalSample,immune-Neutrophil; Emory,totalSample,immune-CD4_T; Emory,totalSample,immune-CD8_T; Emory,totalSample,immune-Tr1; Emory,totalSample,immune-nTreg; Emory,totalSample,immune-iTreg; Emory,totalSample,immune-Tfh; Emory,totalSample,immune-Exhausted; Emory,totalSample,immune-InfiltrationScore; Homo_723,totalSample,immune-nTreg; Homo_723,totalSample,immune-CD8_T; Homo_723,totalSample,immune-Monocyte; Homo_723,totalSample,immune-DC; Homo_723,totalSample,immune-CD4_naive; Homo_723,totalSample,immune-Central_memory; Homo_723,totalSample,immune-Neutrophil; Homo_723,totalSample,immune-CD4_T; Homo_714,totalSample,immune-Monocyte; Homo_714,totalSample,immune-nTreg; Homo_714,totalSample,immune-DC; Homo_714,totalSample,immune-CD8_T; Homo_714,totalSample,immune-Central_memory; Homo_714,totalSample,immune-CD4_naive; Homo_714,totalSample,immune-Neutrophil; Homo_633,totalSample,immune-Monocyte; Homo_633,totalSample,immune-nTreg; Homo_633,totalSample,immune-CD8_T; Homo_633,totalSample,immune-DC; Homo_633,totalSample,immune-Neutrophil; Homo_633,totalSample,immune-Effector_memory; Homo_633,totalSample,immune-Central_memory; Homo_633,totalSample,immune-Macrophage; Homo_633,totalSample,immune-CD4_naive; MCSA,totalSample,immune-DC; MCSA,totalSample,immune-Monocyte; MCSA,totalSample,immune-Neutrophil; MCSA,totalSample,immune-CD4_T; MCSA,totalSample,immune-CD8_T; MCSA,totalSample,immune-Gamma_delta; MCSA,totalSample,immune-CD4_naive; MCSA,totalSample,immune-Tr1; MCSA,totalSample,immune-nTreg; MCSA,totalSample,immune-Th2; MCSA,totalSample,immune-Th17; MCSA,totalSample,immune-Tfh;
DNAJB1	ACOM,male,AD.vs.control; Homo_723,female,AD.vs.MCI; Homo_723,female,MCI.vs.control; Homo_723,control,male.vs.female; Homo_714,female,AD.vs.control; Homo_714,male,AD.vs.control; Homo_714,female,AD.vs.MCI; Homo_714,male,AD.vs.MCI; Homo_714,female,MCI.vs.control; Homo_714,male,MCI.vs.control; Homo_714,AD,male.vs.female; Homo_714,control,male.vs.female; Homo_633,control,male.vs.female; ROSMAP,male,AD.vs.control; MCSA,totalSample,age; MCSA,control,age; MCSA,MCI,age; ROSMAP,MCI,MMSE; Emory,totalSample,immune-Macrophage; Emory,totalSample,immune-Neutrophil; Emory,totalSample,immune-CD4_T; Emory,totalSample,immune-CD8_T; Emory,totalSample,immune-Tr1; Emory,totalSample,immune-iTreg; Emory,totalSample,immune-Tfh; Emory,totalSample,immune-Exhausted; Emory,totalSample,immune-MAIT; Emory,totalSample,immune-Central_memory; Emory,totalSample,immune-InfiltrationScore; Homo_723,totalSample,immune-nTreg; Homo_723,totalSample,immune-CD4_naive; Homo_723,totalSample,immune-Tfh; Homo_723,totalSample,immune-Central_memory; Homo_723,totalSample,immune-Monocyte; Homo_723,totalSample,immune-CD8_T; Homo_723,totalSample,immune-CD4_T; Homo_723,totalSample,immune-InfiltrationScore; Homo_723,totalSample,immune-Effector_memory; Homo_723,totalSample,immune-iTreg; Homo_723,totalSample,immune-Macrophage; Homo_723,totalSample,immune-Tr1; Homo_723,totalSample,immune-Exhausted; Homo_723,totalSample,immune-Bcell; Homo_723,totalSample,immune-Neutrophil; Homo_723,totalSample,immune-DC; Homo_714,totalSample,immune-nTreg; Homo_714,totalSample,immune-CD4_naive; Homo_714,totalSample,immune-Monocyte; Homo_714,totalSample,immune-Central_memory; Homo_714,totalSample,immune-Tfh; Homo_714,totalSample,immune-Effector_memory; Homo_714,totalSample,immune-CD4_T; Homo_714,totalSample,immune-InfiltrationScore; Homo_714,totalSample,immune-iTreg; Homo_714,totalSample,immune-CD8_T; Homo_714,totalSample,immune-Macrophage; Homo_714,totalSample,immune-Neutrophil; Homo_714,totalSample,immune-Tr1; Homo_714,totalSample,immune-MAIT; Homo_714,totalSample,immune-Bcell; Homo_633,totalSample,immune-nTreg; Homo_633,totalSample,immune-Monocyte; Homo_633,totalSample,immune-CD4_naive; Homo_633,totalSample,immune-Tfh; Homo_633,totalSample,immune-Central_memory; Homo_633,totalSample,immune-CD4_T; Homo_633,totalSample,immune-Effector_memory; Homo_633,totalSample,immune-iTreg; Homo_633,totalSample,immune-InfiltrationScore; Homo_633,totalSample,immune-Macrophage; Homo_633,totalSample,immune-CD8_T; Homo_633,totalSample,immune-Neutrophil; Homo_633,totalSample,immune-Tr1; Homo_633,totalSample,immune-MAIT; Homo_633,totalSample,immune-Bcell; MCSA,totalSample,immune-Monocyte; MCSA,totalSample,immune-Neutrophil; MCSA,totalSample,immune-CD4_T; MCSA,totalSample,immune-CD8_T; MCSA,totalSample,immune-CD4_naive; MCSA,totalSample,immune-Tr1; MCSA,totalSample,immune-nTreg; MCSA,totalSample,immune-iTreg; MCSA,totalSample,immune-Th2; MCSA,totalSample,immune-Th17; MCSA,totalSample,immune-Tfh; MCSA,totalSample,immune-MAIT; MCSA,totalSample,immune-Central_memory; MCSA,totalSample,immune-Effector_memory; MCSA,totalSample,immune-InfiltrationScore; SRP223445,totalSample,immune-CD4_naive; SRP223445,totalSample,immune-Th17; SRP223445,totalSample,immune-Exhausted; [illegible]
CRABP2	ACOM,male,AD.vs.control; Homo_723,female,AD.vs.MCI; Homo_723,female,MCI.vs.control; Homo_714,female,AD.vs.control; Homo_714,female,AD.vs.MCI; ROSMAP,female,AD.vs.control; ROSMAP,female,AD.vs.MCI; ROSMAP,male,MCI.vs.control; ROSMAP,MCI,male.vs.female; ROSMAP,AD,last.visit.age; ROSMAP,male and
POU5F1B	ACOM,male,AD.vs.control; ACOM,totalSample,age; MCSA,totalSample,age; MCSA,control,age; MCSA,MCI,age; Emory,totalSample,immune-Th17; Homo_723,totalSample,immune-nTreg; Homo_723,totalSample,immune-CD8_T; Homo_723,totalSample,immune-iTreg; Homo_723,totalSample,immune-Tfh; Homo_723,totalSample,immune-Macrophage; Homo_723,totalSample,immune-Monocyte; Homo_723,totalSample,immune-Effector_memory; Homo_714,totalSample,immune-nTreg; Homo_714,totalSample,immune-CD8_T; Homo_714,totalSample,immune-Monocyte; Homo_714,totalSample,immune-Effector_memory; Homo_714,totalSample,immune-Macrophage; Homo_633,totalSample,immune-nTreg; Homo_633,totalSample,immune-Monocyte; Homo_633,totalSample,immune-Tr1; Homo_633,totalSample,immune-iTreg; Homo_633,totalSample,immune-CD4_T; Homo_633,totalSample,immune-Central_memory; Homo_633,totalSample,immune-Tfh; Homo_633,totalSample,immune-Effector_memory; Homo_633,totalSample,immune-CD4_naive; MCSA,totalSample,immune-Monocyte; MCSA,totalSample,immune-Neutrophil; MCSA,totalSample,immune-CD4_T; MCSA,totalSample,immune-CD8_T; MCSA,totalSample,immune-CD4_naive; MCSA,totalSample,immune-Tr1; MCSA,totalSample,immune-nTreg; MCSA,totalSample,immune-Th17; MCSA,totalSample,immune-Tfh; MCSA,totalSample,immune-Cytotoxic; MCSA,totalSample,immune-Central_memory; MCSA,totalSample,immune-Effector_memory; MCSA,totalSample,immune-InfiltrationScore; SRP223445,totalSample,immune-InfiltrationScore
NR6A1	ACOM,male,AD.vs.control; Homo_723,female,AD.vs.control; Homo_723,male,AD.vs.control; Homo_723,female,AD.vs.MCI; Homo_714,female,AD.vs.MCI; Homo_714,male,AD.vs.MCI; Homo_633,female,AD.vs.control; Homo_633,female,AD.vs.MCI; ROSMAP,female,AD.vs.control; ROSMAP,male,AD.vs.control; ROSMAP,female,AD.vs.MCI; ACOM,totalSample,age; Emory,totalSample,age; Emory,MCI,age; MCSA,MCI,age; ROSMAP,MCI,sample.age; Emory,totalSample,immune-Macrophage; Emory,totalSample,immune-NK; Emory,totalSample,immune-Neutrophil; Emory,totalSample,immune-CD4_T; Emory,totalSample,immune-CD8_T; Emory,totalSample,immune-Gamma_delta; Emory,totalSample,immune-Th17; Emory,totalSample,immune-Tfh; Emory,totalSample,immune-CD8_naive; Emory,totalSample,immune-Cytotoxic; Emory,totalSample,immune-Exhausted; Emory,totalSample,immune-MAIT; Homo_723,totalSample,immune-Neutrophil; Homo_714,totalSample,immune-Neutrophil; Homo_714,totalSample,immune-CD4_naive; Homo_714,totalSample,immune-Central_memory; Homo_714,totalSample,immune-CD4_T; Homo_714,totalSample,immune-MAIT; Homo_714,totalSample,immune-Th1; Homo_633,totalSample,immune-Neutrophil; Homo_633,totalSample,immune-MAIT; Homo_633,totalSample,immune-Central_memory; Homo_633,totalSample,immune-CD4_naive; Homo_633,totalSample,immune-Effector_memory; Homo_633,totalSample,immune-CD4_T; Homo_633,totalSample,immune-CD8_T; Homo_633,totalSample,immune-Tfh; MCSA,totalSample,immune-DC; MCSA,totalSample,immune-Bcell; MCSA,totalSample,immune-Monocyte; MCSA,totalSample,immune-Macrophage; MCSA,totalSample,immune-Neutrophil; MCSA,totalSample,immune-CD4_T; MCSA,totalSample,immune-CD4_naive; MCSA,totalSample,immune-Tr1; MCSA,totalSample,immune-nTreg; MCSA,totalSample,immune-iTreg; MCSA,totalSample,immune-Th17; MCSA,totalSample,immune-Tfh; MCSA,totalSample,immune-CD8_naive; MCSA,totalSample,immune-MAIT; MCSA,totalSample,immune-Central_memory; ROSMAP,totalSample,immune-[illegible]
ANP32D	ACOM,male,AD.vs.control; Homo_723,female,AD.vs.MCI; Homo_714,female,AD.vs.control; Homo_714,female,AD.vs.MCI; Homo_633,female,AD.vs.MCI; Homo_723,totalSample,immune-Exhausted; Homo_723,totalSample,immune-CD8_T; Homo_723,totalSample,immune-Th17; Homo_723,totalSample,immune-Monocyte; Homo_714,totalSample,immune-Exhausted; Homo_714,totalSample,immune-Th17; Homo_633,totalSample,immune-Th2; Homo_633,totalSample,immune-Exhausted; Homo_633,totalSample,immune-Monocyte; MCSA,totalSample,immune-Neutrophil; MCSA,totalSample,immune-iTreg; MCSA,totalSample,immune-Th1; MCSA,totalSample,immune-Cytotoxic; MCSA,totalSample,immune-Exhausted;
PLA2G4B	ACOM,male,AD.vs.control; ACOM,AD,male.vs.female; Homo_723,female,AD.vs.control; Homo_723,female,AD.vs.MCI; Homo_714,female,AD.vs.control; Homo_714,male,AD.vs.control; Homo_714,female,AD.vs.MCI; Homo_714,male,AD.vs.MCI; Homo_633,female,AD.vs.control; Homo_633,female,AD.vs.MCI; ROSMAP,female,AD.vs.MCI; Emory,totalSample,immune-iTreg; Homo_723,totalSample,immune-Effector_memory; Homo_723,totalSample,immune-Th2; Homo_714,totalSample,immune-Th2; Homo_714,totalSample,immune-Effector_memory; Homo_714,totalSample,immune-Tfh; MCSA,totalSample,immune-Monocyte; MCSA,totalSample,immune-Effector_memory; ROSMAP,totalSample,immune-Cytotoxic;
ANO4	ACOM,male,AD.vs.control; ROSMAP,female,AD.vs.control; ROSMAP,AD,last.visit.age; ROSMAP,AD,MMSE; ROSMAP,totalSample,immune-InfiltrationScore
RARRES2	ACOM,male,AD.vs.control; Homo_723,female,AD.vs.MCI; Homo_723,female,MCI.vs.control; ACOM,totalSample,age; MCSA,MCI,age; Homo_723,totalSample,immune-DC; Homo_723,totalSample,immune-Th17; Homo_723,totalSample,immune-Exhausted; ROSMAP,male-includeAge90+,survival
ZNF43	ACOM,male,AD.vs.control; ACOM,totalSample,age; ROSMAP,MCI,sample.age; Emory,totalSample,immune-Tr1; Emory,totalSample,immune-iTreg; Emory,totalSample,immune-InfiltrationScore
ZNF559	ACOM,male,AD.vs.control; ROSMAP,female,AD.vs.MCI; MCSA,MCI,age; Emory,totalSample,immune-DC; Emory,totalSample,immune-Macrophage; Emory,totalSample,immune-Neutrophil; Emory,totalSample,immune-CD4_T; Emory,totalSample,immune-CD8_T; Emory,totalSample,immune-Tr1; Emory,totalSample,immune-iTreg; Emory,totalSample,immune-Tfh; Emory,totalSample,immune-Central_memory; Emory,totalSample,immune-InfiltrationScore; Homo_723,totalSample,immune-Tr1; Homo_723,totalSample,immune-iTreg; Homo_723,totalSample,immune-CD4_naive; Homo_723,totalSample,immune-Tfh; Homo_723,totalSample,immune-CD4_T; Homo_723,totalSample,immune-Central_memory; Homo_723,totalSample,immune-Th2; Homo_723,totalSample,immune-nTreg; Homo_723,totalSample,immune-Th1; Homo_723,totalSample,immune-InfiltrationScore; Homo_723,totalSample,immune-Effector_memory; Homo_723,totalSample,immune-DC; Homo_723,totalSample,immune-Macrophage; Homo_723,totalSample,immune-NKT; Homo_723,totalSample,immune-Neutrophil; Homo_714,totalSample,immune-Tr1; Homo_714,totalSample,immune-CD4_naive; Homo_714,totalSample,immune-Tfh; Homo_714,totalSample,immune-CD4_T; Homo_714,totalSample,immune-iTreg; Homo_714,totalSample,immune-Central_memory; Homo_714,totalSample,immune-Th2; Homo_714,totalSample,immune-Effector_memory; Homo_714,totalSample,immune-NKT; Homo_714,totalSample,immune-InfiltrationScore; Homo_714,totalSample,immune-nTreg; Homo_714,totalSample,immune-Th1; Homo_714,totalSample,immune-DC; Homo_714,totalSample,immune-Neutrophil; Homo_714,totalSample,immune-Macrophage; Homo_714,totalSample,immune-MAIT; Homo_633,totalSample,immune-Tfh; Homo_633,totalSample,immune-CD4_T; Homo_633,totalSample,immune-Tr1; Homo_633,totalSample,immune-CD4_naive; Homo_633,totalSample,immune-NKT; Homo_633,totalSample,immune-Central_memory; Homo_633,totalSample,immune-iTreg; Homo_633,totalSample,immune-Effector_memory; Homo_633,totalSample,immune-InfiltrationScore; Homo_633,totalSample,immune-nTreg; Homo_633,totalSample,immune-Th1; Homo_633,totalSample,immune-DC; Homo_633,totalSample,immune-Th2; Homo_633,totalSample,immune-MAIT; MCSA,totalSample,immune-DC; MCSA,totalSample,immune-Bcell; MCSA,totalSample,immune-Monocyte; MCSA,totalSample,immune-Macrophage; MCSA,totalSample,immune-CD4_T; MCSA,totalSample,immune-CD4_naive; MCSA,totalSample,immune-Tr1; MCSA,totalSample,immune-nTreg; MCSA,totalSample,immune-iTreg; MCSA,totalSample,immune-Th2; MCSA,totalSample,immune-Tfh; MCSA,totalSample,immune-CD8_naive; MCSA,totalSample,immune-Cytotoxic; MCSA,totalSample,immune-Exhausted; MCSA,totalSample,immune-MAIT; MCSA,totalSample,immune-Central_memory; ROSMAP,totalSample,immune-CD4_T; ROSMAP,totalSample,immune-nTreg; ROSMAP,totalSample,immune-iTreg; ROSMAP,totalSample,immune-Th1; ROSMAP,totalSample,immune-Th17; ROSMAP,totalSample,immune-Cytotoxic; ROSMAP,totalSample,immune-Effector_memory; ROSMAP,totalSample,immune-[illegible]
CLEC18C	ACOM,male,AD.vs.control; Homo_723,female,MCI.vs.control; Homo_723,male,MCI.vs.control; Homo_714,female,AD.vs.control; Homo_714,male,AD.vs.control; Homo_714,female,AD.vs.MCI; Homo_714,male,AD.vs.MCI; Homo_714,female,MCI.vs.control; Homo_633,female,AD.vs.control; Homo_633,female,AD.vs.MCI; Homo_633,female,MCI.vs.control; Homo_633,male,MCI.vs.control; ROSMAP,female,AD.vs.control; ROSMAP,male,AD.vs.control; ACOM,totalSample,age; Emory,MCI,age; MCSA,totalSample,age; MCSA,control,age; Emory,totalSample,immune-Neutrophil; Emory,totalSample,immune-CD8_T; Emory,totalSample,immune-Gamma_delta; Emory,totalSample,immune-Th17; Emory,totalSample,immune-Cytotoxic; Emory,totalSample,immune-Exhausted; Homo_723,totalSample,immune-Th1; Homo_723,totalSample,immune-Th2; Homo_714,totalSample,immune-Th1; Homo_714,totalSample,immune-Th2; Homo_714,totalSample,immune-NKT; Homo_633,totalSample,immune-Th1; Homo_633,totalSample,immune-Exhausted; Homo_633,totalSample,immune-NKT; Homo_633,totalSample,immune-Th17; MCSA,totalSample,immune-DC; MCSA,totalSample,immune-Monocyte; MCSA,totalSample,immune-Macrophage; MCSA,totalSample,immune-CD8_T; MCSA,totalSample,immune-Gamma_delta; MCSA,totalSample,immune-iTreg; MCSA,totalSample,immune-Th2; MCSA,totalSample,immune-Cytotoxic; MCSA,totalSample,immune-[illegible]
PMEL	ACOM,male,AD.vs.control; ACOM,AD,male.vs.female; Homo_723,male,AD.vs.control; Homo_723,male,AD.vs.MCI; Homo_723,AD,male.vs.female; Homo_723,control,male.vs.female; Homo_714,male,AD.vs.control; Homo_714,male,AD.vs.MCI; Homo_714,control,male.vs.female; ROSMAP,female,AD.vs.control; MCSA,totalSample,age; MCSA,control,age; ROSMAP,MCI,last.visit.age; Emory,totalSample,immune-CD4_T; Emory,totalSample,immune-CD4_naive; Emory,totalSample,immune-Tr1; Emory,totalSample,immune-iTreg; Emory,totalSample,immune-Central_memory; Homo_723,totalSample,immune-nTreg; Homo_723,totalSample,immune-CD4_T; Homo_723,totalSample,immune-Tfh; Homo_723,totalSample,immune-iTreg; Homo_723,totalSample,immune-CD4_naive; Homo_723,totalSample,immune-Central_memory; Homo_723,totalSample,immune-Th2; Homo_723,totalSample,immune-Tr1; Homo_723,totalSample,immune-CD8_T; Homo_723,totalSample,immune-Effector_memory; Homo_723,totalSample,immune-Macrophage; Homo_714,totalSample,immune-nTreg; Homo_714,totalSample,immune-CD4_T; Homo_714,totalSample,immune-Tfh; Homo_714,totalSample,immune-Th2; Homo_714,totalSample,immune-Central_memory; Homo_714,totalSample,immune-CD4_naive; Homo_714,totalSample,immune-iTreg; Homo_714,totalSample,immune-Tr1; Homo_714,totalSample,immune-Effector_memory; Homo_714,totalSample,immune-Monocyte; Homo_714,totalSample,immune-Th1; Homo_714,totalSample,immune-Macrophage; Homo_633,totalSample,immune-nTreg; Homo_633,totalSample,immune-CD4_T; Homo_633,totalSample,immune-Tfh; Homo_633,totalSample,immune-CD4_naive; Homo_633,totalSample,immune-Tr1; Homo_633,totalSample,immune-Central_memory; Homo_633,totalSample,immune-iTreg; Homo_633,totalSample,immune-Effector_memory; Homo_633,totalSample,immune-Th2; Homo_633,totalSample,immune-Monocyte; Homo_633,totalSample,immune-Th17; Homo_633,totalSample,immune-Th1; MCSA,totalSample,immune-Monocyte; MCSA,totalSample,immune-Macrophage; MCSA,totalSample,immune-Neutrophil; MCSA,totalSample,immune-CD4_T; MCSA,totalSample,immune-Gamma_delta; MCSA,totalSample,immune-CD4_naive; MCSA,totalSample,immune-Tr1; MCSA,totalSample,immune-nTreg; MCSA,totalSample,immune-iTreg; MCSA,totalSample,immune-Th17; MCSA,totalSample,immune-Tfh; MCSA,totalSample,immune-CD8_naive; MCSA,totalSample,immune-[illegible]
NLGN1	ACOM,male,AD.vs.control; Homo_723,female,AD.vs.control; Homo_723,male,AD.vs.control; Homo_723,female,AD.vs.MCI; Homo_723,male,AD.vs.MCI; Homo_723,male,MCI.vs.control; Homo_714,female,AD.vs.control; Homo_714,male,AD.vs.control; Homo_714,female,AD.vs.MCI; Homo_714,male,AD.vs.MCI; Homo_714,male,MCI.vs.control; Homo_633,female,MCI.vs.control; ROSMAP,female,AD.vs.control; ROSMAP,male,AD.vs.control; ACOM,totalSample,age; Homo_723,totalSample,immune-DC; Homo_723,totalSample,immune-Th17; Homo_723,totalSample,immune-Th1; MCSA,totalSample,immune-Tr1; MCSA,totalSample,immune-iTreg; ROSMAP,totalSample,immune-Monocyte; ROSMAP,totalSample,immune-InfiltrationScore; ROSMAP,male-excludeAge90+,survival; ROSMAP,male and female-excludeAge90+,survival
FGF3	ACOM,male,AD.vs.control; ROSMAP,female,AD.vs.control; ROSMAP,AD,last.visit.age

HOXC12	ACOM,male,AD.vs.control; ROSMAP,female,AD.vs.control; ROSMAP,MCI,sample.age; Emory,totalSample,immune-CD8_T; Emory,totalSample,immune-Gamma_delta; ROSMAP,female-includeAge90+,survival
NT5C1B-RDH14	ACOM,male,AD.vs.control; ACOM,AD,male.vs.female; Homo_723,female,AD.vs.MCI; Homo_723,male,AD.vs.MCI; Homo_714,female,AD.vs.MCI; Homo_714,female,MCI.vs.control; Emory,MCI,age; ROSMAP,control,sample.age; ROSMAP,totalSample,immune-InfiltrationScore
TBC1D26	ACOM,male,AD.vs.control; ROSMAP,AD,last.visit.age; ROSMAP,male-includeAge90+,survival
NDNF	ACOM,male,AD.vs.control; Homo_723,female,AD.vs.control; Homo_723,male,AD.vs.control; Homo_723,female,AD.vs.MCI; Homo_723,male,AD.vs.MCI; Homo_633,female,MCI.vs.control; Emory,totalSample,immune-Neutrophil; Emory,totalSample,immune-CD8_T
HOXC11	ACOM,male,AD.vs.control; MCSA,MCI,age; Emory,totalSample,immune-CD8_T; Emory,totalSample,immune-Gamma_delta; SRP223445,totalSample,immune-InfiltrationScore; ROSMAP,female-excludeAge90+,survival; ROSMAP,female-includeAge90+,survival; ROSMAP,male-excludeAge90+,survival; ROSMAP,male-includeAge90+,survival
ALDOA	ACOM,male,AD.vs.control; Emory,totalSample,age; Emory,control,age; Emory,MCI,age; MCSA,totalSample,age; MCSA,control,age; MCSA,MCI,age; Emory,totalSample,immune-DC; Emory,totalSample,immune-Macrophage; Emory,totalSample,immune-Neutrophil; Emory,totalSample,immune-CD4_T; Emory,totalSample,immune-CD8_T; Emory,totalSample,immune-Gamma_delta; Emory,totalSample,immune-Tr1; Emory,totalSample,immune-Th17; Emory,totalSample,immune-Tfh; Emory,totalSample,immune-CD8_naive; Emory,totalSample,immune-Cytotoxic; Emory,totalSample,immune-Exhausted; Homo_723,totalSample,immune-DC; Homo_723,totalSample,immune-Th1; Homo_714,totalSample,immune-DC; Homo_714,totalSample,immune-Th1; Homo_714,totalSample,immune-Monocyte; Homo_633,totalSample,immune-DC; Homo_633,totalSample,immune-Th1; Homo_633,totalSample,immune-NKT; MCSA,totalSample,immune-DC; MCSA,totalSample,immune-Monocyte; MCSA,totalSample,immune-Macrophage; MCSA,totalSample,immune-CD8_T; MCSA,totalSample,immune-iTreg; MCSA,totalSample,immune-Th2; MCSA,totalSample,immune-Cytotoxic; MCSA,totalSample,immune-Effector_memory; MCSA,totalSample,immune-InfiltrationScore
LRRTM2	ACOM,male,AD.vs.control; Homo_723,female,AD.vs.control; Homo_723,female,AD.vs.MCI; Homo_723,male,MCI.vs.control; Homo_714,female,MCI.vs.control; Homo_714,male,MCI.vs.control; Homo_633,female,AD.vs.MCI; Homo_633,female,MCI.vs.control; Homo_633,male,MCI.vs.control; ACOM,totalSample,age; Emory,MCI,age; MCSA,MCI,age; ROSMAP,MCI,sample.age; Homo_723,totalSample,immune-CD8_naive; Homo_723,totalSample,immune-Monocyte; MCSA,totalSample,immune-CD4_T; MCSA,totalSample,immune-Tr1; MCSA,totalSample,immune-iTreg; MCSA,totalSample,immune-CD8_naive; MCSA,totalSample,immune-Exhausted; SRP223445,totalSample,immune-Neutrophil
CLEC18A	ACOM,male,AD.vs.control; Homo_723,female,AD.vs.control; Homo_723,male,AD.vs.control; Homo_723,male,AD.vs.MCI; Homo_714,female,AD.vs.control; Homo_714,male,AD.vs.control; Homo_714,female,AD.vs.MCI; Homo_714,male,AD.vs.MCI; Homo_633,female,AD.vs.control; Homo_633,female,AD.vs.MCI; ROSMAP,female,AD.vs.control; ROSMAP,male,AD.vs.control; ACOM,totalSample,age; MCSA,totalSample,age; MCSA,control,age; Emory,totalSample,immune-Neutrophil; Emory,totalSample,immune-CD8_T; Emory,totalSample,immune-Gamma_delta; Emory,totalSample,immune-Th17; Emory,totalSample,immune-CD8_naive; Emory,totalSample,immune-Cytotoxic; Emory,totalSample,immune-Exhausted; Homo_723,totalSample,immune-Th1; Homo_723,totalSample,immune-Th2; Homo_714,totalSample,immune-Th1; Homo_714,totalSample,immune-Th2; Homo_714,totalSample,immune-NKT; Homo_633,totalSample,immune-Th1; Homo_633,totalSample,immune-Exhausted; Homo_633,totalSample,immune-NKT; MCSA,totalSample,immune-DC; MCSA,totalSample,immune-Monocyte; MCSA,totalSample,immune-Macrophage; MCSA,totalSample,immune-Gamma_delta; MCSA,totalSample,immune-iTreg; MCSA,totalSample,immune-Th2
PLG	ACOM,male,AD.vs.control; Homo_723,female,AD.vs.control; Homo_723,female,AD.vs.MCI; Homo_723,female,MCI.vs.control; Homo_723,male,MCI.vs.control; Homo_714,female,AD.vs.MCI; Homo_714,female,MCI.vs.control; Homo_633,female,MCI.vs.control; ROSMAP,MCI,last.visit.age; ROSMAP,MCI,sample.age; Homo_723,totalSample,immune-Th2; Homo_723,totalSample,immune-Tfh; Homo_723,totalSample,immune-Effector_memory; Homo_723,totalSample,immune-CD4_T; Homo_723,totalSample,immune-CD4_naive; Homo_723,totalSample,immune-InfiltrationScore; Homo_714,totalSample,immune-Tfh; Homo_714,totalSample,immune-Th2; Homo_714,totalSample,immune-InfiltrationScore; Homo_714,totalSample,immune-Effector_memory; Homo_633,totalSample,immune-Th2; Homo_633,totalSample,immune-Tfh; Homo_633,totalSample,immune-Effector_memory; Homo_633,totalSample,immune-InfiltrationScore; Homo_633,totalSample,immune-CD4_T; Homo_633,totalSample,immune-CD4_naive; Homo_633,totalSample,immune-Bcell; MCSA,totalSample,immune-Macrophage; MCSA,totalSample,immune-CD4_T; MCSA,totalSample,immune-CD4_naive; MCSA,totalSample,immune-Tr1; MCSA,totalSample,immune-nTreg; MCSA,totalSample,immune-iTreg; MCSA,totalSample,immune-Th17; MCSA,totalSample,immune-Tfh; MCSA,totalSample,immune-MAIT; MCSA,totalSample,immune-Central_memory; MCSA,totalSample,immune-Effector_memory; MCSA,totalSample,immune-InfiltrationScore; ROSMAP,totalSample,immune-InfiltrationScore; ROSMAP,male-excludeAge90+,survival
NFIA	ACOM,male,AD.vs.control; Emory,control,male.vs.female; Homo_723,control,male.vs.female; Homo_714,male,AD.vs.control; Homo_714,control,male.vs.female; Homo_633,control,male.vs.female; MCSA,totalSample,age; MCSA,control,age; Emory,totalSample,immune-Neutrophil; Emory,totalSample,immune-Gamma_delta; Homo_723,totalSample,immune-Tfh; Homo_723,totalSample,immune-CD4_naive; Homo_723,totalSample,immune-CD4_T; Homo_723,totalSample,immune-InfiltrationScore; Homo_714,totalSample,immune-CD4_naive; Homo_714,totalSample,immune-Tfh; Homo_714,totalSample,immune-CD4_T; Homo_714,totalSample,immune-InfiltrationScore; Homo_633,totalSample,immune-CD4_T; Homo_633,totalSample,immune-CD4_naive; Homo_633,totalSample,immune-Tfh; Homo_633,totalSample,immune-iTreg; MCSA,totalSample,immune-CD4_T; MCSA,totalSample,immune-CD4_naive; MCSA,totalSample,immune-Tr1; MCSA,totalSample,immune-nTreg; MCSA,totalSample,immune-iTreg; MCSA,totalSample,immune-Th17; MCSA,totalSample,immune-Tfh; MCSA,totalSample,immune-Cytotoxic; MCSA,totalSample,immune-Central_memory; ROSMAP,totalSample,immune-Cytotoxic; ROSMAP,totalSample,immune-Central_memory; ROSMAP,totalSample,immune-Effector_memory; SRP223445,totalSample,immune-CD4_naive; SRP223445,totalSample,immune-Exhausted; SRP223445,totalSample,immune-InfiltrationScore
PRSS33	ACOM,male,AD.vs.control; Homo_723,female,AD.vs.control; Homo_723,male,AD.vs.control; Homo_723,female,AD.vs.MCI; Homo_723,male,AD.vs.MCI; Homo_723,female,MCI.vs.control; Homo_723,male,MCI.vs.control; Homo_714,female,AD.vs.control; Homo_714,male,AD.vs.control; Homo_714,female,AD.vs.MCI; Homo_714,male,AD.vs.MCI; Homo_714,female,MCI.vs.control; Homo_714,male,MCI.vs.control; Homo_633,female,AD.vs.control; Homo_633,female,AD.vs.MCI; Homo_633,female,MCI.vs.control; ROSMAP,female,AD.vs.control; ROSMAP,male,AD.vs.control; ROSMAP,female,AD.vs.MCI; ACOM,totalSample,age; Emory,totalSample,age; Emory,control,age; Emory,MCI,age; MCSA,MCI,age; ROSMAP,totalSample,age; ROSMAP,control,sample.age; ROSMAP,totalSample,MMSE; Emory,totalSample,immune-CD8_naive; Homo_723,totalSample,immune-Exhausted; Homo_723,totalSample,immune-CD8_T; Homo_723,totalSample,immune-Cytotoxic; Homo_723,totalSample,immune-Th17; Homo_633,totalSample,immune-DC; MCSA,totalSample,immune-DC; MCSA,totalSample,immune-Monocyte; MCSA,totalSample,immune-Cytotoxic; ROSMAP,totalSample,immune-CD4_T; ROSMAP,totalSample,immune-Tr1; ROSMAP,totalSample,immune-iTreg; ROSMAP,totalSample,immune-Th17; ROSMAP,totalSample,immune-Tfh
OGN	ACOM,male,AD.vs.control; Homo_723,female,AD.vs.control; Homo_723,male,AD.vs.control; Homo_723,female,AD.vs.MCI; Homo_723,male,AD.vs.MCI; Homo_714,female,AD.vs.control; Homo_714,male,AD.vs.control; Homo_714,female,AD.vs.MCI; Homo_714,male,AD.vs.MCI; Homo_633,female,MCI.vs.control; MCSA,MCI,age; Emory,totalSample,immune-Tr1; Emory,totalSample,immune-CD8_naive; Homo_723,totalSample,immune-Tr1; Homo_723,totalSample,immune-CD4_T; Homo_633,totalSample,immune-Tfh; Homo_633,totalSample,immune-CD4_T; Homo_633,totalSample,immune-Tr1; Homo_633,totalSample,immune-CD4_naive; MCSA,totalSample,immune-DC; MCSA,totalSample,immune-Macrophage; MCSA,totalSample,immune-CD4_T; MCSA,totalSample,immune-CD4_naive; MCSA,totalSample,immune-Tr1; MCSA,totalSample,immune-nTreg; MCSA,totalSample,immune-iTreg; MCSA,totalSample,immune-Tfh; MCSA,totalSample,immune-Cytotoxic; ROSMAP,totalSample,immune-InfiltrationScore
HOXD12	ACOM,male,AD.vs.control; ROSMAP,female,AD.vs.control; ROSMAP,totalSample,immune-CD8_naive
SALL4	ACOM,male,AD.vs.control; Homo_723,female,AD.vs.control; Homo_723,male,AD.vs.control; Homo_723,female,AD.vs.MCI; Homo_723,male,AD.vs.MCI; Homo_714,male,AD.vs.control; Homo_714,male,AD.vs.MCI; ROSMAP,female,AD.vs.control; ACOM,totalSample,age; Emory,totalSample,immune-Macrophage; Emory,totalSample,immune-CD8_T;
ZFPM2	ACOM,male,AD.vs.control; Homo_723,female,AD.vs.control; Homo_723,female,AD.vs.MCI; Homo_723,male,AD.vs.MCI; Homo_723,female,MCI.vs.control; Homo_723,male,MCI.vs.control; Homo_714,female,AD.vs.control; Homo_714,female,AD.vs.MCI; Homo_714,male,AD.vs.MCI; Homo_714,female,MCI.vs.control; Homo_714,male,MCI.vs.control; Homo_633,female,MCI.vs.control; Homo_633,male,MCI.vs.control; ROSMAP,female,AD.vs.control; ROSMAP,male,AD.vs.control; Emory,totalSample,immune-CD8_T; Homo_723,totalSample,immune-CD8_T; Homo_723,totalSample,immune-Th17; Homo_723,totalSample,immune-DC; Homo_723,totalSample,immune-Exhausted; Homo_723,totalSample,immune-Cytotoxic; Homo_723,totalSample,immune-Macrophage; Homo_714,totalSample,immune-Th2; Homo_714,totalSample,immune-CD8_T; ROSMAP,totalSample,immune-InfiltrationScore; SRP223445,totalSample,immune-CD4_naive; SRP223445,totalSample,immune-Exhausted; ROSMAP,male-excludeAge90+,survival
ENPP2	ACOM,male,AD.vs.control; Homo_723,female,AD.vs.control; Homo_723,male,AD.vs.control; Homo_723,female,MCI.vs.control; Homo_723,control,male.vs.female; Homo_714,female,AD.vs.control; Homo_714,female,MCI.vs.control; Homo_714,MCI,male.vs.female; Homo_633,female,MCI.vs.control; Homo_633,MCI,male.vs.female; ROSMAP,control,male.vs.female; ACOM,totalSample,age; Emory,totalSample,immune-Neutrophil; Emory,totalSample,immune-Gamma_delta; Emory,totalSample,immune-Th17; Emory,totalSample,immune-Cytotoxic; Homo_723,totalSample,immune-CD4_naive; Homo_723,totalSample,immune-InfiltrationScore; Homo_723,totalSample,immune-iTreg; Homo_723,totalSample,immune-CD4_T; Homo_723,totalSample,immune-Tr1; Homo_723,totalSample,immune-Tfh; Homo_723,totalSample,immune-Central_memory; Homo_723,totalSample,immune-nTreg; Homo_723,totalSample,immune-NKT; Homo_714,totalSample,immune-NKT; Homo_714,totalSample,immune-iTreg; Homo_714,totalSample,immune-InfiltrationScore; Homo_714,totalSample,immune-CD4_naive; Homo_714,totalSample,immune-Tfh; Homo_633,totalSample,immune-NKT; Homo_633,totalSample,immune-iTreg; Homo_633,totalSample,immune-CD4_naive; Homo_633,totalSample,immune-Tfh; Homo_633,totalSample,immune-InfiltrationScore; Homo_633,totalSample,immune-Tr1; Homo_633,totalSample,immune-CD4_T; Homo_633,totalSample,immune-Th1; MCSA,totalSample,immune-CD4_T; MCSA,totalSample,immune-CD8_T; MCSA,totalSample,immune-Gamma_delta; MCSA,totalSample,immune-CD4_naive; MCSA,totalSample,immune-Tr1; MCSA,totalSample,immune-nTreg; MCSA,totalSample,immune-iTreg; MCSA,totalSample,immune-Th17; MCSA,totalSample,immune-Tfh; MCSA,totalSample,immune-CD8_naive; MCSA,totalSample,immune-Cytotoxic; MCSA,totalSample,immune-Exhausted; MCSA,totalSample,immune-Central_memory; ROSMAP,totalSample,immune-InfiltrationScore; SRP223445,totalSample,immune-Exhausted; SRP223445,totalSample,immune-InfiltrationScore
SLITRK3	ACOM,male,AD.vs.control; Homo_723,female,AD.vs.control; Homo_723,male,AD.vs.control; Homo_723,female,AD.vs.MCI; Homo_723,male,AD.vs.MCI; ACOM,totalSample,age; Homo_723,totalSample,immune-DC; Homo_723,totalSample,immune-Th17; ROSMAP,female-includeAge90+,survival; ROSMAP,male and female-excludeAge90+,survival; ROSMAP,male and
LAMC3	ACOM,male,AD.vs.control; Homo_723,female,AD.vs.control; Homo_723,male,AD.vs.control; Homo_714,female,AD.vs.control; Homo_714,male,AD.vs.control; Homo_633,female,AD.vs.control; Homo_633,female,AD.vs.MCI; Homo_633,female,MCI.vs.control; ROSMAP,female,AD.vs.control; ROSMAP,male,AD.vs.control; ACOM,totalSample,age; Emory,totalSample,age; Emory,control,age; MCSA,MCI,age; Emory,totalSample,immune-CD4_naive; Emory,totalSample,immune-Tr1; Emory,totalSample,immune-nTreg; Emory,totalSample,immune-iTreg; Emory,totalSample,immune-Cytotoxic; Emory,totalSample,immune-Central_memory; Homo_723,totalSample,immune-Central_memory; Homo_723,totalSample,immune-DC; Homo_723,totalSample,immune-nTreg; Homo_723,totalSample,immune-Monocyte; Homo_714,totalSample,immune-nTreg; Homo_714,totalSample,immune-DC; Homo_633,totalSample,immune-nTreg; Homo_633,totalSample,immune-Central_memory; Homo_633,totalSample,immune-Monocyte; MCSA,totalSample,immune-DC; MCSA,totalSample,immune-Monocyte; MCSA,totalSample,immune-Neutrophil; MCSA,totalSample,immune-CD8_T; MCSA,totalSample,immune-CD4_naive; MCSA,totalSample,immune-nTreg; MCSA,totalSample,immune-Th2; MCSA,totalSample,immune-Tfh; MCSA,totalSample,immune-Cytotoxic; MCSA,totalSample,immune-Central_memory; MCSA,totalSample,immune-Effector_memory;
AGAP6	ACOM,male,AD.vs.control; Homo_723,female,AD.vs.control; Homo_723,male,AD.vs.control; Homo_723,female,MCI.vs.control; Homo_723,male,MCI.vs.control; Homo_714,female,AD.vs.control; Homo_714,male,AD.vs.control; Homo_714,female,AD.vs.MCI; Homo_714,male,AD.vs.MCI; Homo_714,female,MCI.vs.control; Homo_714,male,MCI.vs.control; Homo_633,female,AD.vs.control; Homo_633,female,AD.vs.MCI; Homo_633,female,MCI.vs.control; Homo_633,male,MCI.vs.control; ROSMAP,female,AD.vs.control; ROSMAP,female,AD.vs.MCI; ACOM,totalSample,age; MCSA,totalSample,age; MCSA,control,age; Homo_723,totalSample,immune-Th2; Homo_723,totalSample,immune-Tfh; Homo_723,totalSample,immune-Effector_memory; Homo_723,totalSample,immune-iTreg; Homo_723,totalSample,immune-CD4_T; Homo_723,totalSample,immune-Tr1; Homo_723,totalSample,immune-nTreg; Homo_723,totalSample,immune-CD4_naive; Homo_723,totalSample,immune-Macrophage; Homo_723,totalSample,immune-InfiltrationScore; Homo_714,totalSample,immune-Th2; Homo_714,totalSample,immune-Tfh; Homo_714,totalSample,immune-Effector_memory; Homo_714,totalSample,immune-CD4_T; Homo_714,totalSample,immune-Tr1; Homo_714,totalSample,immune-Central_memory; Homo_714,totalSample,immune-nTreg; Homo_714,totalSample,immune-InfiltrationScore; Homo_633,totalSample,immune-Effector_memory; Homo_633,totalSample,immune-Tfh; Homo_633,totalSample,immune-Th2; Homo_633,totalSample,immune-Central_memory; Homo_633,totalSample,immune-InfiltrationScore; Homo_633,totalSample,immune-CD4_T; Homo_633,totalSample,immune-CD4_naive; Homo_633,totalSample,immune-Exhausted; Homo_633,totalSample,immune-nTreg; Homo_633,totalSample,immune-Tr1; MCSA,totalSample,immune-Neutrophil; MCSA,totalSample,immune-CD4_T; MCSA,totalSample,immune-NKT; MCSA,totalSample,immune-CD4_naive; MCSA,totalSample,immune-Tr1; MCSA,totalSample,immune-nTreg; MCSA,totalSample,immune-iTreg; MCSA,totalSample,immune-Th17; MCSA,totalSample,immune-Tfh; MCSA,totalSample,immune-CD8_naive; MCSA,totalSample,immune-Exhausted; MCSA,totalSample,immune-MAIT; MCSA,totalSample,immune-Central_memory; MCSA,totalSample,immune-Effector_memory; ROSMAP,totalSample,immune-Effector_memory
IGDCC3	ACOM,male,AD.vs.control; Homo_723,female,AD.vs.control; Homo_723,female,AD.vs.MCI; Homo_714,female,AD.vs.control; Homo_633,female,MCI.vs.control; Homo_633,male,MCI.vs.control; MCSA,MCI,age; Emory,totalSample,immune-Macrophage; Emory,totalSample,immune-CD8_T; Emory,totalSample,immune-Gamma_delta
TMEM176A	ACOM,male,AD.vs.control; Emory,control,male.vs.female; Homo_723,female,AD.vs.control; Homo_723,male,AD.vs.control; Homo_723,female,AD.vs.MCI; Homo_723,male,AD.vs.MCI; Homo_714,female,AD.vs.control; Homo_714,male,AD.vs.control; Homo_714,female,AD.vs.MCI; Homo_714,male,AD.vs.MCI; Homo_633,female,AD.vs.control; Homo_633,female,AD.vs.MCI; ROSMAP,female,AD.vs.control; ROSMAP,control,male.vs.female; ACOM,totalSample,age; Emory,MCI,age; Emory,totalSample,immune-Neutrophil; Emory,totalSample,immune-CD8_naive
LRTOMT	ACOM,male,AD.vs.control; Homo_723,female,AD.vs.control; Homo_723,male,AD.vs.control; Homo_723,female,AD.vs.MCI; Homo_723,male,AD.vs.MCI; Homo_714,female,AD.vs.control; Homo_714,male,AD.vs.control; Homo_714,female,AD.vs.MCI; Homo_714,male,AD.vs.MCI; Homo_633,female,AD.vs.control; Homo_633,female,AD.vs.MCI; MCSA,totalSample,age; MCSA,control,age; ROSMAP,MCI,last.visit.age; ROSMAP,MCI,sample.age; Emory,totalSample,immune-CD4_T; Emory,totalSample,immune-Tr1; Emory,totalSample,immune-iTreg; Emory,totalSample,immune-Th17; Emory,totalSample,immune-Cytotoxic; Homo_723,totalSample,immune-DC; Homo_723,totalSample,immune-nTreg; Homo_723,totalSample,immune-Neutrophil; Homo_723,totalSample,immune-Th17; Homo_723,totalSample,immune-Central_memory; Homo_633,totalSample,immune-nTreg; MCSA,totalSample,immune-Monocyte; MCSA,totalSample,immune-Th17; ROSMAP,male-excludeAge90+,survival
AFM	ACOM,male,AD.vs.control; ROSMAP,female,AD.vs.control; ROSMAP,AD,MMSE; ROSMAP,totalSample,immune-InfiltrationScore
FMC1-LUC7L2	ACOM,male,AD.vs.control; ROSMAP,female,AD.vs.control; ROSMAP,female,MCI.vs.control; ROSMAP,MCI,last.visit.age; ROSMAP,MCI,sample.age
SPRY1	ACOM,male,AD.vs.control; Homo_723,female,AD.vs.control; Homo_723,male,AD.vs.control; Homo_723,female,AD.vs.MCI; Homo_723,male,AD.vs.MCI; Homo_714,female,AD.vs.control; Homo_714,male,AD.vs.control; Homo_714,female,AD.vs.MCI; Homo_714,male,AD.vs.MCI; Homo_633,female,AD.vs.control; Homo_633,female,AD.vs.MCI; Homo_633,female,MCI.vs.control; ROSMAP,control,male.vs.female; MCSA,totalSample,age; MCSA,control,age; Emory,totalSample,immune-B_cell; Emory,totalSample,immune-Macrophage; Emory,totalSample,immune-Neutrophil; Emory,totalSample,immune-CD4_T; Emory,totalSample,immune-Tr1; Emory,totalSample,immune-iTreg; Emory,totalSample,immune-Th17; Emory,totalSample,immune-Tfh; Emory,totalSample,immune-CD8_naive; Emory,totalSample,immune-InfiltrationScore; Homo_723,totalSample,immune-Th1; Homo_723,totalSample,immune-CD4_naive; Homo_723,totalSample,immune-Central_memory; Homo_723,totalSample,immune-CD4_T; Homo_723,totalSample,immune-Th17; Homo_723,totalSample,immune-nTreg; Homo_723,totalSample,immune-Neutrophil; Homo_723,totalSample,immune-Cytotoxic; Homo_723,totalSample,immune-Monocyte; Homo_723,totalSample,immune-DC; Homo_723,totalSample,immune-Tr1; Homo_723,totalSample,immune-Tfh; Homo_714,totalSample,immune-Th1; Homo_714,totalSample,immune-NKT; Homo_714,totalSample,immune-CD4_naive; Homo_714,totalSample,immune-iTreg; Homo_714,totalSample,immune-InfiltrationScore; Homo_714,totalSample,immune-CD4_T; Homo_714,totalSample,immune-Exhausted; Homo_633,totalSample,immune-NKT; Homo_633,totalSample,immune-iTreg; Homo_633,totalSample,immune-Tfh; Homo_633,totalSample,immune-CD4_T; Homo_633,totalSample,immune-CD4_naive; Homo_633,totalSample,immune-Th1; Homo_633,totalSample,immune-Tr1; Homo_633,totalSample,immune-nTreg; Homo_633,totalSample,immune-InfiltrationScore; Homo_633,totalSample,immune-Central_memory; MCSA,totalSample,immune-DC; MCSA,totalSample,immune-Bcell; MCSA,totalSample,immune-Macrophage; MCSA,totalSample,immune-CD4_T; MCSA,totalSample,immune-Gamma_delta; MCSA,totalSample,immune-CD4_naive; MCSA,totalSample,immune-Tr1; MCSA,totalSample,immune-nTreg; MCSA,totalSample,immune-iTreg; MCSA,totalSample,immune-Th17; MCSA,totalSample,immune-Tfh; MCSA,totalSample,immune-CD8_naive; MCSA,totalSample,immune-Cytotoxic; MCSA,totalSample,immune-[illegible]
EPS8L3	ACOM,male,AD.vs.control; Homo_723,female,AD.vs.control
KCNQ3	ACOM,male,AD.vs.control; Homo_723,female,AD.vs.control; Homo_723,male,AD.vs.control; Homo_723,female,AD.vs.MCI; Homo_723,male,AD.vs.MCI; Homo_714,female,AD.vs.control; Homo_714,female,AD.vs.MCI; Homo_714,female,MCI.vs.control; Homo_633,female,AD.vs.MCI; Homo_633,female,MCI.vs.control; ROSMAP,MCI,sample.age; Emory,totalSample,immune-CD8_T; ROSMAP,totalSample,immune-InfiltrationScore; ROSMAP,male-excludeAge90+,survival
LUZP6	ACOM,male,AD.vs.control; Homo_723,male,AD.vs.control; Homo_723,male,AD.vs.MCI; Homo_723,AD,male.vs.female; Homo_723,MCI,male.vs.female; Homo_714,male,AD.vs.control; Homo_714,male,AD.vs.MCI; Homo_714,male,MCI.vs.control; Homo_714,AD,male.vs.female; Homo_714,MCI,male.vs.female; Homo_633,female,AD.vs.control; Homo_633,female,AD.vs.MCI; Homo_633,male,MCI.vs.control; Homo_633,control,male.vs.female; Homo_633,MCI,male.vs.female; MCSA,MCI,age; Homo_723,totalSample,immune-Th1; Homo_714,totalSample,immune-Th1; Homo_714,totalSample,immune-NKT; Homo_633,totalSample,immune-NKT; Homo_633,totalSample,immune-Th1; MCSA,totalSample,immune-DC; MCSA,totalSample,immune-Monocyte; MCSA,totalSample,immune-Neutrophil; MCSA,totalSample,immune-CD8_T; MCSA,totalSample,immune-Tr1; MCSA,totalSample,immune-iTreg; MCSA,totalSample,immune-Th2; MCSA,totalSample,immune-Th17; MCSA,totalSample,immune-Cytotoxic; MCSA,totalSample,immune-Exhausted; MCSA,totalSample,immune-
QPRT	ACOM,male,AD.vs.control; Homo_723,female,AD.vs.control; Homo_723,male,AD.vs.control; Homo_723,female,AD.vs.MCI; Homo_723,control,male.vs.female; Homo_714,female,AD.vs.control; Homo_714,male,AD.vs.control; Homo_714,female,AD.vs.MCI; Homo_714,control,male.vs.female; Homo_633,control,male.vs.female; ACOM,totalSample,age; Emory,totalSample,age; Emory,MCI,age; Emory,totalSample,immune-DC; Emory,totalSample,immune-NK; Emory,totalSample,immune-Effector_memory; Emory,totalSample,immune-InfiltrationScore; Homo_723,totalSample,immune-DC; Homo_723,totalSample,immune-Monocyte; Homo_723,totalSample,immune-Neutrophil; Homo_723,totalSample,immune-Cytotoxic; Homo_723,totalSample,immune-Th2; Homo_723,totalSample,immune-Exhausted; Homo_723,totalSample,immune-NK; Homo_723,totalSample,immune-InfiltrationScore; Homo_714,totalSample,immune-DC; Homo_714,totalSample,immune-Th2; Homo_714,totalSample,immune-Monocyte; Homo_714,totalSample,immune-Neutrophil; Homo_714,totalSample,immune-InfiltrationScore; Homo_714,totalSample,immune-NK; Homo_633,totalSample,immune-DC; Homo_633,totalSample,immune-Th2; Homo_633,totalSample,immune-Monocyte; Homo_633,totalSample,immune-Th17; Homo_633,totalSample,immune-InfiltrationScore; Homo_633,totalSample,immune-NK; MCSA,totalSample,immune-DC; MCSA,totalSample,immune-Monocyte; MCSA,totalSample,immune-NK; MCSA,totalSample,immune-Neutrophil; MCSA,totalSample,immune-CD8_T; MCSA,totalSample,immune-NKT; MCSA,totalSample,immune-Th2; MCSA,totalSample,immune-Cytotoxic; MCSA,totalSample,immune-Effector_memory; MCSA,totalSample,immune-InfiltrationScore; ROSMAP,totalSample,immune-MAIT;

EIF4EBP3	ACOM,AD,male.vs.female; Homo_723,female,AD.vs.MCI; Homo_723,male,AD.vs.MCI; Homo_723,female,MCI.vs.control; Homo_723,male,MCI.vs.control; Homo_714,female,AD.vs.MCI; Homo_714,male,AD.vs.MCI; Homo_714,female,MCI.vs.control; Homo_714,male,MCI.vs.control; Homo_714,MCI,male.vs.female; Homo_633,female,AD.vs.control; Homo_633,female,AD.vs.MCI; Homo_633,female,MCI.vs.control; Homo_633,male,MCI.vs.control; Homo_633,MCI,male.vs.female; ROSMAP,female,AD.vs.control; ROSMAP,female,AD.vs.MCI; ACOM,totalSample,age; MCSA,MCI,age; ROSMAP,MCI,last.visit.age; Emory,totalSample,immune-CD4_T; Emory,totalSample,immune-Tr1; Emory,totalSample,immune-nTreg; Emory,totalSample,immune-iTreg; Emory,totalSample,immune-Central_memory; Homo_723,totalSample,immune-DC; Homo_723,totalSample,immune-Exhausted; Homo_723,totalSample,immune-Monocyte; Homo_723,totalSample,immune-CD8_T; Homo_723,totalSample,immune-Cytotoxic; Homo_723,totalSample,immune-Th2; Homo_723,totalSample,immune-Th17; Homo_714,totalSample,immune-DC; Homo_714,totalSample,immune-Th2; Homo_714,totalSample,immune-Monocyte; Homo_714,totalSample,immune-Th17; Homo_633,totalSample,immune-DC; Homo_633,totalSample,immune-Th2; Homo_633,totalSample,immune-Monocyte; Homo_633,totalSample,immune-Th17; MCSA,totalSample,immune-DC; MCSA,totalSample,immune-Monocyte; MCSA,totalSample,immune-Macrophage; MCSA,totalSample,immune-Neutrophil; MCSA,totalSample,immune-CD4_T; MCSA,totalSample,immune-CD8_T; MCSA,totalSample,immune-Tr1; MCSA,totalSample,immune-iTreg; MCSA,totalSample,immune-Th2; MCSA,totalSample,immune-Cytotoxic; MCSA,totalSample,immune-Exhausted; MCSA,totalSample,immune-Effector_memory; MCSA,totalSample,immune-InfiltrationScore; ROSMAP,totalSample,immune-Effector_memory; SRP223445,totalSample,immune-Th17; SRP223445,totalSample,immune-InfiltrationScore
HLA-DPB1	ACOM,AD,male.vs.female; ACOM,totalSample,age; Emory,totalSample,immune-B_cell; Homo_723,totalSample,immune-DC; Homo_714,totalSample,immune-InfiltrationScore; Homo_633,totalSample,immune-DC; MCSA,totalSample,immune-DC; MCSA,totalSample,immune-Bcell; MCSA,totalSample,immune-Monocyte; MCSA,totalSample,immune-NK; MCSA,totalSample,immune-Neutrophil; MCSA,totalSample,immune-NKT; MCSA,totalSample,immune-Effector_memory; MCSA,totalSample,immune-InfiltrationScore; SRP223445,totalSample,immune-InfiltrationScore
HES6	ACOM,AD,male.vs.female; Homo_723,female,AD.vs.control; Homo_723,male,AD.vs.control; Homo_723,female,AD.vs.MCI; Homo_723,male,AD.vs.MCI; Homo_714,female,AD.vs.MCI; Homo_714,male,AD.vs.MCI; Homo_633,female,AD.vs.control; Homo_633,female,AD.vs.MCI; Emory,totalSample,immune-Monocyte; Emory,totalSample,immune-Cytotoxic; Emory,totalSample,immune-Effector_memory; Homo_723,totalSample,immune-DC; Homo_723,totalSample,immune-Monocyte; Homo_723,totalSample,immune-Cytotoxic; Homo_723,totalSample,immune-Neutrophil; Homo_723,totalSample,immune-CD8_T; Homo_723,totalSample,immune-InfiltrationScore; Homo_714,totalSample,immune-DC; Homo_714,totalSample,immune-InfiltrationScore; Homo_714,totalSample,immune-NK; Homo_714,totalSample,immune-Monocyte; Homo_633,totalSample,immune-DC; Homo_633,totalSample,immune-NK; Homo_633,totalSample,immune-Th2; Homo_633,totalSample,immune-InfiltrationScore; Homo_633,totalSample,immune-Monocyte; MCSA,totalSample,immune-DC; MCSA,totalSample,immune-Monocyte; MCSA,totalSample,immune-Neutrophil; MCSA,totalSample,immune-CD8_T; MCSA,totalSample,immune-NKT; MCSA,totalSample,immune-Th2; MCSA,totalSample,immune-Cytotoxic; MCSA,totalSample,immune-Effector_memory; MCSA,totalSample,immune-InfiltrationScore
XK	ACOM,AD,male.vs.female; Emory,control,male.vs.female; Homo_723,male,AD.vs.control; Homo_723,male,AD.vs.MCI; Homo_723,AD,male.vs.female; Homo_714,male,AD.vs.control; Homo_714,male,AD.vs.MCI; Homo_714,AD,male.vs.female; Homo_633,male,MCI.vs.control; Homo_633,MCI,male.vs.female; Emory,MCI,age; Emory,totalSample,immune-Th17; Homo_723,totalSample,immune-Bcell; Homo_714,totalSample,immune-Bcell; Homo_633,totalSample,immune-Bcell; MCSA,totalSample,immune-CD8_T; MCSA,totalSample,immune-iTreg; MCSA,totalSample,immune-Cytotoxic; ROSMAP,totalSample,immune-Th17; ROSMAP,totalSample,immune-InfiltrationScore; SRP223445,totalSample,immune-CD4_naive;
TNNT2	ACOM,AD,male.vs.female; ROSMAP,totalSample,immune-InfiltrationScore; ROSMAP,male-excludeAge90+,survival
DLX5	ACOM,AD,male.vs.female; Homo_723,totalSample,immune-CD8_naive
PAQR5	ACOM,AD,male.vs.female; Homo_723,female,AD.vs.control; Homo_723,male,AD.vs.control; Homo_723,female,AD.vs.MCI; Homo_723,male,AD.vs.MCI; Homo_723,male,MCI.vs.control; Homo_714,female,AD.vs.control; Homo_714,male,AD.vs.control; Homo_714,female,AD.vs.MCI; Homo_714,male,AD.vs.MCI; Homo_714,male,MCI.vs.control; Homo_633,female,AD.vs.control; Homo_633,female,AD.vs.MCI; ROSMAP,female,AD.vs.control; Emory,totalSample,immune-Neutrophil; Emory,totalSample,immune-CD8_T; Emory,totalSample,immune-Gamma_delta; Emory,totalSample,immune-Th17; Emory,totalSample,immune-Exhausted; Emory,totalSample,immune-MAIT; Homo_723,totalSample,immune-Exhausted; Homo_723,totalSample,immune-Th17; Homo_723,totalSample,immune-CD8_T; Homo_723,totalSample,immune-Monocyte; Homo_723,totalSample,immune-Cytotoxic; Homo_714,totalSample,immune-Exhausted; Homo_714,totalSample,immune-Th17; Homo_714,totalSample,immune-Monocyte; Homo_714,totalSample,immune-CD8_T; Homo_714,totalSample,immune-Gamma_delta; Homo_633,totalSample,immune-Exhausted; Homo_633,totalSample,immune-Monocyte; Homo_633,totalSample,immune-Th17; Homo_633,totalSample,immune-Th2; Homo_633,totalSample,immune-Gamma_delta; Homo_633,totalSample,immune-Neutrophil; MCSA,totalSample,immune-Neutrophil; MCSA,totalSample,immune-CD8_T;
GAREM1	ACOM,AD,male.vs.female; Homo_723,female,AD.vs.control; Homo_723,male,AD.vs.control; Homo_723,female,AD.vs.MCI; Homo_723,male,AD.vs.MCI; Homo_714,female,AD.vs.control; Homo_714,male,AD.vs.control; Homo_714,female,AD.vs.MCI; Homo_714,male,AD.vs.MCI; Homo_633,female,MCI.vs.control; Homo_633,male,MCI.vs.control; ROSMAP,female,AD.vs.control; ROSMAP,male,AD.vs.control; MCSA,MCI,age; ROSMAP,MCI,last.visit.age; ROSMAP,MCI,sample.age; MCSA,totalSample,immune-iTreg
ICAM1	ACOM,AD,male.vs.female; Homo_723,male,AD.vs.control; Homo_723,male,AD.vs.MCI; Homo_723,AD,male.vs.female; Homo_714,male,AD.vs.control; Homo_714,male,AD.vs.MCI; Homo_714,AD,male.vs.female; SRP223445,female,AD.vs.control; ACOM,totalSample,age; Emory,MCI,age; MCSA,totalSample,age; MCSA,control,age; ROSMAP,MCI,last.visit.age; ROSMAP,MCI,sample.age; Emory,totalSample,immune-B_cell; Emory,totalSample,immune-Macrophage; Emory,totalSample,immune-Neutrophil; Emory,totalSample,immune-CD8_T; Emory,totalSample,immune-Gamma_delta; Emory,totalSample,immune-Th1; Emory,totalSample,immune-Tfh; Emory,totalSample,immune-CD8_naive; Emory,totalSample,immune-Exhausted; Homo_723,totalSample,immune-Neutrophil; Homo_723,totalSample,immune-Th1; Homo_723,totalSample,immune-DC; Homo_723,totalSample,immune-Tr1; Homo_723,totalSample,immune-MAIT; Homo_714,totalSample,immune-Neutrophil; Homo_714,totalSample,immune-MAIT; Homo_633,totalSample,immune-Neutrophil; Homo_633,totalSample,immune-MAIT; Homo_633,totalSample,immune-DC; MCSA,totalSample,immune-DC; MCSA,totalSample,immune-Bcell; MCSA,totalSample,immune-Monocyte; MCSA,totalSample,immune-Macrophage; MCSA,totalSample,immune-Neutrophil; MCSA,totalSample,immune-Gamma_delta; MCSA,totalSample,immune-Th1; MCSA,totalSample,immune-Th2; MCSA,totalSample,immune-Th17;
SLC46A1	ACOM,AD,male.vs.female; Homo_723,female,AD.vs.MCI; Homo_714,female,AD.vs.control; Homo_714,male,AD.vs.control; Homo_714,female,AD.vs.MCI; Homo_714,male,AD.vs.MCI; Homo_633,female,AD.vs.control; Homo_633,female,AD.vs.MCI; ROSMAP,female,AD.vs.control; ROSMAP,male,AD.vs.control; MCSA,totalSample,age; MCSA,control,age; ROSMAP,AD,last.visit.age; ROSMAP,MCI,sample.age; Emory,totalSample,immune-Gamma_delta; Emory,totalSample,immune-InfiltrationScore; Homo_723,totalSample,immune-Th2; Homo_723,totalSample,immune-Tfh; Homo_723,totalSample,immune-Effector_memory; Homo_723,totalSample,immune-iTreg; Homo_723,totalSample,immune-CD4_T; Homo_723,totalSample,immune-CD4_naive; Homo_723,totalSample,immune-InfiltrationScore; Homo_723,totalSample,immune-nTreg; Homo_723,totalSample,immune-Tr1; Homo_714,totalSample,immune-Th2; Homo_714,totalSample,immune-Tfh; Homo_714,totalSample,immune-Effector_memory; Homo_714,totalSample,immune-CD4_T; Homo_714,totalSample,immune-Central_memory; Homo_714,totalSample,immune-CD4_naive; Homo_714,totalSample,immune-Tr1; Homo_714,totalSample,immune-Gamma_delta; Homo_714,totalSample,immune-nTreg; Homo_633,totalSample,immune-Effector_memory; Homo_633,totalSample,immune-Tfh; Homo_633,totalSample,immune-Th2; Homo_633,totalSample,immune-CD4_T; Homo_633,totalSample,immune-Central_memory; Homo_633,totalSample,immune-Gamma_delta; Homo_633,totalSample,immune-CD4_naive; Homo_633,totalSample,immune-Exhausted; Homo_633,totalSample,immune-InfiltrationScore; Homo_633,totalSample,immune-Th17; Homo_633,totalSample,immune-nTreg; Homo_633,totalSample,immune-Tr1; MCSA,totalSample,immune-NK; MCSA,totalSample,immune-CD4_T; MCSA,totalSample,immune-NKT; MCSA,totalSample,immune-Gamma_delta; MCSA,totalSample,immune-CD4_naive; MCSA,totalSample,immune-Tr1; MCSA,totalSample,immune-nTreg; MCSA,totalSample,immune-iTreg; MCSA,totalSample,immune-Th2; MCSA,totalSample,immune-Th17;
ZNF737	ACOM,AD,male.vs.female; MCSA,MCI,age; Emory,totalSample,immune-Macrophage; Emory,totalSample,immune-CD4_T; Emory,totalSample,immune-Tr1; Emory,totalSample,immune-iTreg; Emory,totalSample,immune-Tfh; Emory,totalSample,immune-Central_memory; Emory,totalSample,immune-InfiltrationScore; Homo_723,totalSample,immune-CD4_naive; Homo_723,totalSample,immune-iTreg; Homo_723,totalSample,immune-Tr1; Homo_723,totalSample,immune-CD4_T; Homo_723,totalSample,immune-Tfh; Homo_723,totalSample,immune-Central_memory; Homo_723,totalSample,immune-Th2; Homo_723,totalSample,immune-nTreg; Homo_723,totalSample,immune-Cytotoxic; Homo_723,totalSample,immune-Effector_memory; Homo_723,totalSample,immune-NKT; Homo_723,totalSample,immune-InfiltrationScore; Homo_723,totalSample,immune-Th1; Homo_723,totalSample,immune-Gamma_delta; Homo_723,totalSample,immune-DC; Homo_714,totalSample,immune-CD4_naive; Homo_714,totalSample,immune-CD4_T; Homo_714,totalSample,immune-Tfh; Homo_714,totalSample,immune-iTreg; Homo_714,totalSample,immune-Tr1; Homo_714,totalSample,immune-Central_memory; Homo_714,totalSample,immune-Effector_memory; Homo_714,totalSample,immune-NKT; Homo_714,totalSample,immune-Th2; Homo_714,totalSample,immune-nTreg; Homo_714,totalSample,immune-Th1; Homo_714,totalSample,immune-InfiltrationScore; Homo_714,totalSample,immune-Cytotoxic; Homo_714,totalSample,immune-Gamma_delta; Homo_714,totalSample,immune-DC; Homo_633,totalSample,immune-Tfh; Homo_633,totalSample,immune-CD4_naive; Homo_633,totalSample,immune-CD4_T; Homo_633,totalSample,immune-Tr1; Homo_633,totalSample,immune-Central_memory; Homo_633,totalSample,immune-NKT; Homo_633,totalSample,immune-iTreg; Homo_633,totalSample,immune-Effector_memory; Homo_633,totalSample,immune-nTreg; Homo_633,totalSample,immune-InfiltrationScore; Homo_633,totalSample,immune-DC; Homo_633,totalSample,immune-Th2; Homo_633,totalSample,immune-Gamma_delta; Homo_633,totalSample,immune-Th1; Homo_633,totalSample,immune-Cytotoxic; Homo_633,totalSample,immune-Exhausted; MCSA,totalSample,immune-DC; MCSA,totalSample,immune-Macrophage; MCSA,totalSample,immune-CD4_T; MCSA,totalSample,immune-CD4_naive; MCSA,totalSample,immune-Tr1; MCSA,totalSample,immune-nTreg; MCSA,totalSample,immune-iTreg; MCSA,totalSample,immune-Th17; MCSA,totalSample,immune-Tfh; MCSA,totalSample,immune-CD8_naive; MCSA,totalSample,immune-Cytotoxic; MCSA,totalSample,immune-Exhausted; MCSA,totalSample,immune-MAIT; MCSA,totalSample,immune-Central_memory; ROSMAP,totalSample,immune-InfiltrationScore
MFNG	ACOM,AD,male.vs.female; Homo_714,male,AD.vs.control; Homo_714,male,AD.vs.MCI; MCSA,totalSample,age; MCSA,control,age; MCSA,MCI,age; ROSMAP,MCI,last.visit.age; Emory,totalSample,immune-Macrophage; Emory,totalSample,immune-Neutrophil; Emory,totalSample,immune-CD4_T; Emory,totalSample,immune-CD8_T; Emory,totalSample,immune-Tr1; Emory,totalSample,immune-iTreg; Emory,totalSample,immune-Th17; Emory,totalSample,immune-Tfh; Emory,totalSample,immune-MAIT; Emory,totalSample,immune-Central_memory; Emory,totalSample,immune-InfiltrationScore; Homo_723,totalSample,immune-CD8_T; Homo_723,totalSample,immune-DC; Homo_723,totalSample,immune-nTreg; Homo_723,totalSample,immune-Cytotoxic; Homo_723,totalSample,immune-Monocyte; Homo_723,totalSample,immune-Effector_memory; Homo_723,totalSample,immune-InfiltrationScore; Homo_723,totalSample,immune-Neutrophil; Homo_723,totalSample,immune-Bcell; Homo_714,totalSample,immune-DC; Homo_714,totalSample,immune-CD8_T; Homo_714,totalSample,immune-nTreg; Homo_714,totalSample,immune-Monocyte; Homo_714,totalSample,immune-Effector_memory; Homo_714,totalSample,immune-InfiltrationScore; Homo_714,totalSample,immune-Neutrophil; Homo_714,totalSample,immune-Cytotoxic; Homo_714,totalSample,immune-Macrophage; Homo_633,totalSample,immune-DC; Homo_633,totalSample,immune-nTreg; Homo_633,totalSample,immune-CD8_T; Homo_633,totalSample,immune-Monocyte; Homo_633,totalSample,immune-Effector_memory; Homo_633,totalSample,immune-Neutrophil; Homo_633,totalSample,immune-Cytotoxic; Homo_633,totalSample,immune-InfiltrationScore; Homo_633,totalSample,immune-Macrophage; MCSA,totalSample,immune-DC; MCSA,totalSample,immune-Monocyte; MCSA,totalSample,immune-Neutrophil; MCSA,totalSample,immune-CD8_T; MCSA,totalSample,immune-NKT; MCSA,totalSample,immune-Th2; MCSA,totalSample,immune-Tfh; MCSA,totalSample,immune-Cytotoxic; MCSA,totalSample,immune-Central_memory; MCSA,totalSample,immune-
SNCA	ACOM,AD,male.vs.female; ROSMAP,female,AD.vs.control; Emory,totalSample,age; Emory,MCI,age; ROSMAP,MCI,last.visit.age; ROSMAP,MCI,sample.age; Emory,totalSample,immune-Gamma_delta; Emory,totalSample,immune-CD8_naive; Homo_723,totalSample,immune-Bcell; Homo_723,totalSample,immune-Gamma_delta; Homo_714,totalSample,immune-Bcell; Homo_633,totalSample,immune-Bcell; Homo_633,totalSample,immune-Monocyte; ROSMAP,totalSample,immune-Th17; ROSMAP,totalSample,immune-Cytotoxic; ROSMAP,totalSample,immune-Effector_memory; SRP223445,totalSample,immune-InfiltrationScore
SCG5	ACOM,AD,male.vs.female; Homo_723,female,AD.vs.control; Homo_723,female,AD.vs.MCI; Homo_723,female,MCI.vs.control; ROSMAP,female,AD.vs.control; ACOM,totalSample,age; ROSMAP,totalSample,immune-Central_memory; ROSMAP,totalSample,immune-InfiltrationScore; ROSMAP,male-includeAge90+,survival
CLEC19A	ACOM,AD,male.vs.female; Homo_723,female,AD.vs.control; ACOM,totalSample,age; ROSMAP,totalSample,immune-InfiltrationScore
REG3G	ACOM,AD,male.vs.female; ACOM,totalSample,immune-Effector_memory
CDHR4	ACOM,AD,male.vs.female; Homo_714,female,AD.vs.control; ROSMAP,MCI,last.visit.age; Emory,totalSample,immune-CD8_T; ROSMAP,totalSample,immune-InfiltrationScore
EBF2	ACOM,AD,male.vs.female; Homo_723,male,AD.vs.control; Homo_723,totalSample,immune-DC; ROSMAP,totalSample,immune-InfiltrationScore
RASSF2	ACOM,AD,male.vs.female; Emory,MCI,age; ROSMAP,MCI,last.visit.age; Emory,totalSample,immune-Monocyte; Emory,totalSample,immune-NK; Emory,totalSample,immune-Neutrophil; Emory,totalSample,immune-CD8_T; Emory,totalSample,immune-Gamma_delta; Emory,totalSample,immune-Th1; Emory,totalSample,immune-Th17; Emory,totalSample,immune-Tfh; Emory,totalSample,immune-CD8_naive; Emory,totalSample,immune-Cytotoxic; Emory,totalSample,immune-Exhausted; Homo_723,totalSample,immune-Neutrophil; Homo_723,totalSample,immune-CD8_T; Homo_723,totalSample,immune-Exhausted; Homo_723,totalSample,immune-Cytotoxic; Homo_723,totalSample,immune-Gamma_delta; Homo_723,totalSample,immune-DC; Homo_723,totalSample,immune-Macrophage; Homo_723,totalSample,immune-MAIT; Homo_714,totalSample,immune-Neutrophil; Homo_714,totalSample,immune-Exhausted; Homo_714,totalSample,immune-CD8_T; Homo_714,totalSample,immune-Cytotoxic; Homo_714,totalSample,immune-Gamma_delta; Homo_714,totalSample,immune-DC; Homo_714,totalSample,immune-MAIT; Homo_714,totalSample,immune-Macrophage; Homo_714,totalSample,immune-Monocyte; Homo_714,totalSample,immune-Th17; Homo_633,totalSample,immune-Neutrophil; Homo_633,totalSample,immune-CD8_T; Homo_633,totalSample,immune-Exhausted; Homo_633,totalSample,immune-Cytotoxic; Homo_633,totalSample,immune-Gamma_delta; Homo_633,totalSample,immune-Macrophage; Homo_633,totalSample,immune-DC; Homo_633,totalSample,immune-MAIT; Homo_633,totalSample,immune-Th17; Homo_633,totalSample,immune-Th2; Homo_633,totalSample,immune-NKT; MCSA,totalSample,immune-DC; MCSA,totalSample,immune-Monocyte; MCSA,totalSample,immune-Neutrophil; MCSA,totalSample,immune-CD8_T; MCSA,totalSample,immune-Gamma_delta; MCSA,totalSample,immune-iTreg; MCSA,totalSample,immune-Th1; MCSA,totalSample,immune-Th17; MCSA,totalSample,immune-Cytotoxic; MCSA,totalSample,immune-Exhausted; MCSA,totalSample,immune-Effector_memory; MCSA,totalSample,immune-InfiltrationScore; ROSMAP,totalSample,immune-Effector_memory; SRP223445,totalSample,immune-CD4_T; SRP223445,totalSample,immune-CD4_naive; SRP223445,totalSample,immune-Th17; SRP223445,totalSample,immune-Exhausted
CNTFR	ACOM,AD,male.vs.female; ROSMAP,female,AD.vs.control; ROSMAP,female,AD.vs.MCI; ROSMAP,MCI,male.vs.female; ACOM,AD,age; ROSMAP,male-excludeAge90+,survival
NMRK1	ACOM,AD,male.vs.female; Homo_723,female,AD.vs.control; Homo_723,male,AD.vs.control; Homo_723,female,AD.vs.MCI; Homo_723,male,AD.vs.MCI; Homo_714,female,AD.vs.control; Homo_714,male,AD.vs.control; Homo_714,female,AD.vs.MCI; Homo_714,male,AD.vs.MCI; Homo_633,female,AD.vs.control; Homo_633,female,AD.vs.MCI; MCSA,control,age; MCSA,MCI,age; Emory,totalSample,immune-DC; Emory,totalSample,immune-Macrophage; Emory,totalSample,immune-Neutrophil; Emory,totalSample,immune-CD4_T; Emory,totalSample,immune-CD8_T; Emory,totalSample,immune-Tr1; Emory,totalSample,immune-iTreg; Emory,totalSample,immune-Tfh; Emory,totalSample,immune-Central_memory; Emory,totalSample,immune-InfiltrationScore; Homo_723,totalSample,immune-Tfh; Homo_723,totalSample,immune-Th2; Homo_723,totalSample,immune-iTreg; Homo_723,totalSample,immune-Tr1; Homo_723,totalSample,immune-CD4_T; Homo_723,totalSample,immune-CD4_naive; Homo_723,totalSample,immune-DC; Homo_723,totalSample,immune-Effector_memory; Homo_723,totalSample,immune-Central_memory; Homo_723,totalSample,immune-InfiltrationScore; Homo_723,totalSample,immune-nTreg; Homo_723,totalSample,immune-Monocyte; Homo_723,totalSample,immune-Gamma_delta; Homo_723,totalSample,immune-Macrophage; Homo_723,totalSample,immune-MAIT; Homo_723,totalSample,immune-Bcell; Homo_714,totalSample,immune-Tfh; Homo_714,totalSample,immune-Th2; Homo_714,totalSample,immune-CD4_T; Homo_714,totalSample,immune-Tr1; Homo_714,totalSample,immune-Central_memory; Homo_714,totalSample,immune-CD4_naive; Homo_714,totalSample,immune-Effector_memory; Homo_714,totalSample,immune-iTreg; Homo_714,totalSample,immune-nTreg; Homo_714,totalSample,immune-DC; Homo_714,totalSample,immune-Gamma_delta; Homo_714,totalSample,immune-InfiltrationScore; Homo_633,totalSample,immune-Tfh; Homo_633,totalSample,immune-CD4_T; Homo_633,totalSample,immune-Th2; Homo_633,totalSample,immune-CD4_naive; Homo_633,totalSample,immune-Central_memory; Homo_633,totalSample,immune-Tr1; Homo_633,totalSample,immune-Effector_memory; Homo_633,totalSample,immune-nTreg; Homo_633,totalSample,immune-iTreg; Homo_633,totalSample,immune-Th17; Homo_633,totalSample,immune-InfiltrationScore; Homo_633,totalSample,immune-Gamma_delta; Homo_633,totalSample,immune-Monocyte; Homo_633,totalSample,immune-DC; Homo_633,totalSample,immune-Exhausted; Homo_633,totalSample,immune-NKT; MCSA,totalSample,immune-DC; MCSA,totalSample,immune-Macrophage; MCSA,totalSample,immune-CD4_T; MCSA,totalSample,immune-CD4_naive; MCSA,totalSample,immune-Tr1; MCSA,totalSample,immune-nTreg; MCSA,totalSample,immune-iTreg; MCSA,totalSample,immune-Th17; MCSA,totalSample,immune-Tfh; MCSA,totalSample,immune-CD8_naive; MCSA,totalSample,immune-Cytotoxic; MCSA,totalSample,immune-Exhausted; MCSA,totalSample,immune-MAIT; MCSA,totalSample,immune-Central_memory; ROSMAP,female-excludeAge90+,survival; ROSMAP,male-excludeAge90+,survival; [illegible]
S100B	ACOM,AD,male.vs.female; Homo_723,female,AD.vs.control; Homo_723,female,AD.vs.MCI; Homo_723,female,MCI.vs.control; Homo_714,female,AD.vs.control; Homo_714,female,AD.vs.MCI; Homo_714,female,MCI.vs.control; Homo_633,female,AD.vs.control; Homo_633,female,AD.vs.MCI; Emory,totalSample,age; Emory,control,age; Emory,MCI,age; ROSMAP,AD,sample.age; ROSMAP,MCI,last.visit.age; ROSMAP,MCI,sample.age; Emory,totalSample,immune-Macrophage; Emory,totalSample,immune-Neutrophil; Emory,totalSample,immune-CD8_T; Emory,totalSample,immune-Tfh; Emory,totalSample,immune-CD8_naive; Emory,totalSample,immune-Exhausted; Emory,totalSample,immune-MAIT
TSPAN10	ACOM,AD,male.vs.female; Homo_723,control,male.vs.female; Homo_714,control,male.vs.female; ROSMAP,female,AD.vs.control; Emory,totalSample,immune-CD4_T; Emory,totalSample,immune-Tr1; Emory,totalSample,immune-iTreg; Homo_723,totalSample,immune-CD8_naive; MCSA,totalSample,immune-CD8_naive; ROSMAP,male and female-excludeAge90+,survival
TAGLN	ACOM,AD,male.vs.female; Homo_723,female,AD.vs.control; Homo_723,male,AD.vs.control; Homo_723,male,AD.vs.MCI; Homo_714,female,AD.vs.control; Homo_714,male,AD.vs.control; Homo_714,female,AD.vs.MCI; Homo_633,female,AD.vs.control; Homo_633,female,AD.vs.MCI; SRP223445,female,AD.vs.control; Emory,MCI,age; MCSA,totalSample,age; MCSA,MCI,age; ROSMAP,AD,sample.age; ROSMAP,MCI,last.visit.age; Emory,totalSample,immune-DC; Emory,totalSample,immune-NK; Emory,totalSample,immune-InfiltrationScore; Homo_723,totalSample,immune-DC; Homo_723,totalSample,immune-InfiltrationScore; Homo_723,totalSample,immune-Neutrophil; Homo_714,totalSample,immune-DC; Homo_714,totalSample,immune-InfiltrationScore; Homo_714,totalSample,immune-Neutrophil; Homo_633,totalSample,immune-DC; Homo_633,totalSample,immune-InfiltrationScore; MCSA,totalSample,immune-DC; MCSA,totalSample,immune-Monocyte; MCSA,totalSample,immune-Neutrophil; MCSA,totalSample,immune-CD8_T; MCSA,totalSample,immune-Cytotoxic; MCSA,totalSample,immune-Effector_memory; MCSA,totalSample,immune-InfiltrationScore; SRP223445,totalSample,immune-Neutrophil; SRP223445,totalSample,immune-nTreg; SRP223445,totalSample,immune-InfiltrationScore
QKI	ACOM,AD,male.vs.female; Emory,totalSample,age; Emory,MCI,age; MCSA,MCI,age; ROSMAP,MCI,last.visit.age; Emory,totalSample,immune-B_cell; Emory,totalSample,immune-Monocyte; Emory,totalSample,immune-Neutrophil; Emory,totalSample,immune-CD8_T; Emory,totalSample,immune-Gamma_delta; Emory,totalSample,immune-Th1; Emory,totalSample,immune-Th17; Emory,totalSample,immune-Tfh; Emory,totalSample,immune-CD8_naive; Emory,totalSample,immune-Cytotoxic; Emory,totalSample,immune-Exhausted; Homo_723,totalSample,immune-Cytotoxic; Homo_723,totalSample,immune-Gamma_delta; Homo_723,totalSample,immune-CD8_T; Homo_723,totalSample,immune-NKT; Homo_723,totalSample,immune-Exhausted; Homo_723,totalSample,immune-Neutrophil; Homo_723,totalSample,immune-Tr1; Homo_723,totalSample,immune-InfiltrationScore; Homo_723,totalSample,immune-iTreg; Homo_714,totalSample,immune-Exhausted; Homo_714,totalSample,immune-CD8_T; Homo_714,totalSample,immune-Cytotoxic; Homo_714,totalSample,immune-NKT; Homo_714,totalSample,immune-Gamma_delta; Homo_714,totalSample,immune-Neutrophil; Homo_714,totalSample,immune-Tr1; Homo_714,totalSample,immune-Monocyte; Homo_633,totalSample,immune-NKT; Homo_633,totalSample,immune-Exhausted; Homo_633,totalSample,immune-CD8_T; Homo_633,totalSample,immune-Gamma_delta; Homo_633,totalSample,immune-Cytotoxic; Homo_633,totalSample,immune-DC; Homo_633,totalSample,immune-Neutrophil; Homo_633,totalSample,immune-Th2; Homo_633,totalSample,immune-Tr1; Homo_633,totalSample,immune-Th17; Homo_633,totalSample,immune-Monocyte; Homo_633,totalSample,immune-iTreg; Homo_633,totalSample,immune-Macrophage; MCSA,totalSample,immune-DC; MCSA,totalSample,immune-Monocyte; MCSA,totalSample,immune-Neutrophil; MCSA,totalSample,immune-CD4_T; MCSA,totalSample,immune-CD8_T; MCSA,totalSample,immune-Gamma_delta; MCSA,totalSample,immune-CD4_naive; MCSA,totalSample,immune-Tr1; MCSA,totalSample,immune-nTreg; MCSA,totalSample,immune-iTreg; MCSA,totalSample,immune-Th1; MCSA,totalSample,immune-Th17; MCSA,totalSample,immune-CD8_naive; MCSA,totalSample,immune-Cytotoxic; MCSA,totalSample,immune-Exhausted; MCSA,totalSample,immune-Effector_memory; SRP223445,totalSample,immune-NK; ROSMAP,female-includeAge90+,survival

DLL1	ACOM,AD,male.vs.female; Homo_723,female,AD.vs.control; Homo_723,male,AD.vs.control; Homo_723,female,AD.vs.MCI; Homo_723,male,AD.vs.MCI; Homo_723,female,MCI.vs.control; Homo_714,female,AD.vs.control; Homo_714,male,AD.vs.control; Homo_714,female,AD.vs.MCI; Homo_714,male,AD.vs.MCI; Homo_633,female,AD.vs.control; Homo_633,female,AD.vs.MCI; Homo_633,male,MCI.vs.control; ROSMAP,female,AD.vs.MCI; ROSMAP,female,MCI.vs.control; MCSA,totalSample,age; MCSA,control,age; Emory,totalSample,immune-CD4_T; Emory,totalSample,immune-Gamma_delta; Emory,totalSample,immune-Tr1; Emory,totalSample,immune-iTreg; Homo_723,totalSample,immune-DC; Homo_723,totalSample,immune-nTreg; Homo_723,totalSample,immune-Monocyte; Homo_723,totalSample,immune-Central_memory; Homo_723,totalSample,immune-InfiltrationScore; Homo_723,totalSample,immune-Neutrophil; Homo_723,totalSample,immune-CD4_naive; Homo_714,totalSample,immune-InfiltrationScore; Homo_714,totalSample,immune-nTreg; Homo_633,totalSample,immune-nTreg; Homo_633,totalSample,immune-Monocyte; Homo_633,totalSample,immune-Tr1; Homo_633,totalSample,immune-iTreg; Homo_633,totalSample,immune-InfiltrationScore; Homo_633,totalSample,immune-Effector_memory; MCSA,totalSample,immune-DC; MCSA,totalSample,immune-Monocyte; MCSA,totalSample,immune-Neutrophil; MCSA,totalSample,immune-CD4_T; MCSA,totalSample,immune-CD8_T; MCSA,totalSample,immune-NKT; MCSA,totalSample,immune-Gamma_delta; MCSA,totalSample,immune-CD4_naive; MCSA,totalSample,immune-Tr1; MCSA,totalSample,immune-nTreg; MCSA,totalSample,immune-iTreg; MCSA,totalSample,immune-Th2; MCSA,totalSample,immune-Tfh; MCSA,totalSample,immune-CD8_naive; MCSA,totalSample,immune-Exhausted; MCSA,totalSample,immune-Central_memory; MCSA,totalSample,immune-Effector_memory; MCSA,totalSample,immune-InfiltrationScore; SRP223445,totalSample,immune-NK; SRP223445,totalSample,immune-InfiltrationScore
DEUP1	ACOM,AD,male.vs.female; ROSMAP,female,MCI.vs.control; ROSMAP,control,male.vs.female; ACOM,AD,age; ROSMAP,AD,sample.age; ROSMAP,AD,MMSE; ROSMAP,totalSample,immune-InfiltrationScore
RCOR2	ACOM,AD,male.vs.female; ROSMAP,female,AD.vs.control; ROSMAP,female,AD.vs.MCI; ROSMAP,MCI,sample.age; SRP223445,totalSample,immune-InfiltrationScore
BSX	ACOM,AD,male.vs.female; Homo_723,totalSample,immune-DC
MYO16	ACOM,AD,male.vs.female; Homo_723,male,AD.vs.control; Homo_723,female,AD.vs.MCI; Homo_723,female,MCI.vs.control; Homo_714,female,AD.vs.MCI; Homo_714,female,MCI.vs.control; Homo_633,female,AD.vs.control; Homo_633,female,AD.vs.MCI; Homo_633,female,MCI.vs.control; Emory,MCI,age; Emory,totalSample,immune-Exhausted; MCSA,totalSample,immune-Th17; ROSMAP,totalSample,immune-InfiltrationScore
OVCA2	ACOM,AD,male.vs.female; ROSMAP,MCI,last.visit.age; ROSMAP,MCI,sample.age; Emory,totalSample,immune-InfiltrationScore; Homo_723,totalSample,immune-DC; Homo_723,totalSample,immune-Cytotoxic; Homo_723,totalSample,immune-Effector_memory; Homo_723,totalSample,immune-InfiltrationScore; Homo_723,totalSample,immune-Neutrophil; Homo_714,totalSample,immune-DC; Homo_714,totalSample,immune-InfiltrationScore; Homo_714,totalSample,immune-Th17; Homo_633,totalSample,immune-DC; Homo_633,totalSample,immune-Th2; Homo_633,totalSample,immune-NKT; Homo_633,totalSample,immune-Th17; MCSA,totalSample,immune-DC; MCSA,totalSample,immune-Monocyte; MCSA,totalSample,immune-Macrophage; MCSA,totalSample,immune-Neutrophil; MCSA,totalSample,immune-CD8_T; MCSA,totalSample,immune-NKT; MCSA,totalSample,immune-iTreg; MCSA,totalSample,immune-Th2; MCSA,totalSample,immune-Th17; MCSA,totalSample,immune-Cytotoxic; MCSA,totalSample,immune-Effector_memory; MCSA,totalSample,immune-
OLIG3	ACOM,AD,male.vs.female; Homo_723,female,AD.vs.control; Homo_723,female,AD.vs.MCI; Homo_723,control,male.vs.female; Homo_633,female,MCI.vs.control; Emory,totalSample,immune-CD8_T; Emory,totalSample,immune-Exhausted; Homo_723,totalSample,immune-DC; Homo_714,totalSample,immune-Th2
CRYZL2P-SEC16B	ACOM,AD,male.vs.female; Homo_723,female,AD.vs.control; Homo_723,female,AD.vs.MCI; Homo_723,male,AD.vs.MCI; Homo_714,female,AD.vs.control; Homo_714,female,AD.vs.MCI; Homo_714,male,AD.vs.MCI; Homo_633,female,AD.vs.control; Homo_633,female,AD.vs.MCI; ACOM,totalSample,age; Emory,totalSample,immune-Central_memory; Homo_723,totalSample,immune-Exhausted; Homo_723,totalSample,immune-CD8_T; Homo_723,totalSample,immune-Th17; Homo_714,totalSample,immune-Exhausted; Homo_714,totalSample,immune-Monocyte; Homo_633,totalSample,immune-Exhausted; Homo_633,totalSample,immune-Monocyte; Homo_633,totalSample,immune-Th2; Homo_633,totalSample,immune-Gamma_delta; MCSA,totalSample,immune-Gamma_delta; MCSA,totalSample,immune-Tr1; MCSA,totalSample,immune-iTreg; MCSA,totalSample,immune-Cytotoxic; MCSA,totalSample,immune-Exhausted; SRP223445,totalSample,immune-InfiltrationScore
CRLF1	ACOM,AD,male.vs.female; Homo_723,female,AD.vs.control; Homo_723,female,AD.vs.MCI; Homo_714,female,AD.vs.control; Homo_714,female,AD.vs.MCI; ROSMAP,male,AD.vs.control; ROSMAP,AD,male.vs.female; ROSMAP,AD,last.visit.age; MCSA,totalSample,immune-CD8_T; ROSMAP,totalSample,immune-Th17
OPTC	ACOM,AD,male.vs.female; ACOM,AD,age; ACOM,totalSample,age; Homo_723,totalSample,immune-Th17; Homo_723,totalSample,immune-DC
SHOC1	ACOM,AD,male.vs.female; Homo_723,female,AD.vs.control; Homo_723,male,AD.vs.control; Homo_723,female,AD.vs.MCI; Homo_723,male,AD.vs.MCI; Homo_723,female,MCI.vs.control; Homo_723,male,MCI.vs.control; Homo_714,female,AD.vs.control; Homo_714,male,AD.vs.control; Homo_714,female,AD.vs.MCI; Homo_714,male,AD.vs.MCI; Homo_714,female,MCI.vs.control; Homo_714,male,MCI.vs.control; Homo_633,female,AD.vs.control; Homo_633,female,AD.vs.MCI; Homo_633,female,MCI.vs.control; Homo_633,male,MCI.vs.control; ROSMAP,female,MCI.vs.control; ROSMAP,control,male.vs.female; MCSA,MCI,age; ROSMAP,MCI,sample.age; Emory,totalSample,immune-Neutrophil; Emory,totalSample,immune-CD8_T; Emory,totalSample,immune-Gamma_delta; Emory,totalSample,immune-Th17; Emory,totalSample,immune-Tfh; Emory,totalSample,immune-Exhausted; Emory,totalSample,immune-Effector_memory; Homo_723,totalSample,immune-Neutrophil; Homo_723,totalSample,immune-Exhausted; Homo_723,totalSample,immune-CD8_naive; Homo_723,totalSample,immune-Gamma_delta; Homo_714,totalSample,immune-Neutrophil; Homo_633,totalSample,immune-Neutrophil; Homo_633,totalSample,immune-Effector_memory; Homo_633,totalSample,immune-CD8_T; MCSA,totalSample,immune-Neutrophil; MCSA,totalSample,immune-CD8_T; MCSA,totalSample,immune-NKT; MCSA,totalSample,immune-Gamma_delta; MCSA,totalSample,immune-Th1; MCSA,totalSample,immune-Th17; MCSA,totalSample,immune-Cytotoxic; MCSA,totalSample,immune-MAIT; MCSA,totalSample,immune-Central_memory; MCSA,totalSample,immune-Effector_memory; ROSMAP,totalSample,immune-InfiltrationScore
MAGEB17	ACOM,AD,male.vs.female; Homo_723,female,AD.vs.MCI; Homo_723,male,AD.vs.MCI; Homo_714,male,AD.vs.MCI; ACOM,AD,age
EGFL6	ACOM,AD,male.vs.female; Homo_723,female,AD.vs.control; Homo_714,female,AD.vs.control; ROSMAP,totalSample,immune-InfiltrationScore; SRP223445,totalSample,immune-Th17
SPHK1	ACOM,AD,male.vs.female; Homo_723,male,AD.vs.control; Homo_723,male,AD.vs.MCI; Homo_723,AD,male.vs.female; Homo_714,male,AD.vs.control; Homo_714,male,AD.vs.MCI; Homo_714,AD,male.vs.female; ROSMAP,female,AD.vs.control; ROSMAP,male,AD.vs.control; ROSMAP,male,AD.vs.MCI; ROSMAP,female,MCI.vs.control; ROSMAP,AD,male.vs.female; ACOM,totalSample,age; MCSA,totalSample,age; MCSA,MCI,age; ROSMAP,MCI,last.visit.age; ROSMAP,MCI,sample.age; Homo_723,totalSample,immune-DC; Homo_723,totalSample,immune-Cytotoxic; Homo_723,totalSample,immune-Th1; Homo_714,totalSample,immune-DC; Homo_714,totalSample,immune-Th1; Homo_633,totalSample,immune-DC; Homo_633,totalSample,immune-Th1; Homo_633,totalSample,immune-NKT; Homo_633,totalSample,immune-Th2; MCSA,totalSample,immune-DC; MCSA,totalSample,immune-Monocyte; MCSA,totalSample,immune-Macrophage; MCSA,totalSample,immune-Neutrophil; MCSA,totalSample,immune-CD8_T; MCSA,totalSample,immune-NKT; MCSA,totalSample,immune-iTreg; MCSA,totalSample,immune-Th2; MCSA,totalSample,immune-Cytotoxic; MCSA,totalSample,immune-Effector_memory; MCSA,totalSample,immune-InfiltrationScore; ROSMAP,totalSample,immune-Macrophage; ROSMAP,totalSample,immune-Effector_memory; SRP223445,totalSample,immune-Exhausted
ADAM11	ACOM,AD,male.vs.female; Homo_723,female,AD.vs.control; Homo_723,male,AD.vs.control; Homo_723,female,AD.vs.MCI; Homo_723,male,AD.vs.MCI; Homo_723,female,MCI.vs.control; Homo_714,female,AD.vs.MCI; ROSMAP,female,AD.vs.control; ROSMAP,female,MCI.vs.control; ROSMAP,control,male.vs.female; MCSA,totalSample,age; MCSA,control,age; MCSA,MCI,age; Homo_714,totalSample,immune-InfiltrationScore; MCSA,totalSample,immune-DC; MCSA,totalSample,immune-Monocyte; MCSA,totalSample,immune-Neutrophil; MCSA,totalSample,immune-CD8_T; MCSA,totalSample,immune-CD4_naive; MCSA,totalSample,immune-Th2; MCSA,totalSample,immune-Tfh; MCSA,totalSample,immune-Cytotoxic; MCSA,totalSample,immune-Central_memory; MCSA,totalSample,immune-Effector_memory; MCSA,totalSample,immune-InfiltrationScore; SRP223445,totalSample,immune-Neutrophil; ROSMAP,female-includeAge90+,survival; ROSMAP,male and female-excludeAge90+,survival; ROSMAP,male and female-includeAge90+,survival
HSPA2	ACOM,AD,male.vs.female; ROSMAP,female,AD.vs.control; ROSMAP,male,AD.vs.control; ROSMAP,AD,male.vs.female; MCSA,totalSample,age; MCSA,control,age; Homo_723,totalSample,immune-DC; Homo_723,totalSample,immune-InfiltrationScore; Homo_714,totalSample,immune-DC; Homo_714,totalSample,immune-InfiltrationScore; Homo_633,totalSample,immune-InfiltrationScore; MCSA,totalSample,immune-DC; MCSA,totalSample,immune-Monocyte; MCSA,totalSample,immune-CD8_T; MCSA,totalSample,immune-NKT; MCSA,totalSample,immune-CD4_naive; MCSA,totalSample,immune-Tr1; MCSA,totalSample,immune-nTreg; MCSA,totalSample,immune-Th2; MCSA,totalSample,immune-Tfh; MCSA,totalSample,immune-Central_memory; MCSA,totalSample,immune-Effector_memory
RYR1	ACOM,AD,male.vs.female; Homo_723,female,AD.vs.control; Homo_723,female,AD.vs.MCI; Homo_714,female,AD.vs.control; Homo_714,female,AD.vs.MCI; Homo_633,female,AD.vs.control; Homo_633,female,AD.vs.MCI; ROSMAP,female,AD.vs.control; ROSMAP,male,AD.vs.control; ROSMAP,male,MCI.vs.control; ROSMAP,MCI,male.vs.female; Emory,totalSample,immune-DC; Emory,totalSample,immune-InfiltrationScore; Homo_723,totalSample,immune-DC; Homo_723,totalSample,immune-InfiltrationScore; Homo_723,totalSample,immune-Neutrophil; Homo_723,totalSample,immune-Monocyte; Homo_714,totalSample,immune-InfiltrationScore; Homo_714,totalSample,immune-DC; Homo_714,totalSample,immune-NK; Homo_633,totalSample,immune-InfiltrationScore; Homo_633,totalSample,immune-DC; Homo_633,totalSample,immune-NK; MCSA,totalSample,immune-DC; MCSA,totalSample,immune-Monocyte; MCSA,totalSample,immune-NK; MCSA,totalSample,immune-Neutrophil; MCSA,totalSample,immune-CD8_T; MCSA,totalSample,immune-NKT; MCSA,totalSample,immune-Cytotoxic; MCSA,totalSample,immune-Effector_memory; MCSA,totalSample,immune-InfiltrationScore; ROSMAP,totalSample,immune-InfiltrationScore; SRP223445,totalSample,immune-DC
ERICH5	ACOM,AD,male.vs.female; Homo_723,female,AD.vs.control; Homo_723,female,AD.vs.MCI; Homo_714,male,AD.vs.MCI; ROSMAP,AD,last.visit.age; Emory,totalSample,immune-Neutrophil; Emory,totalSample,immune-CD8_T; Homo_723,totalSample,immune-Th2
NOTCH1	ACOM,AD,male.vs.female; Homo_714,female,AD.vs.control; Homo_714,male,MCI.vs.control; ROSMAP,male,AD.vs.control; ACOM,AD,age; Emory,totalSample,age; Emory,MCI,age; MCSA,MCI,age; Emory,totalSample,immune-Macrophage; Emory,totalSample,immune-NK; Emory,totalSample,immune-Neutrophil; Emory,totalSample,immune-CD8_T; Emory,totalSample,immune-Gamma_delta; Emory,totalSample,immune-Th1; Emory,totalSample,immune-Th17; Emory,totalSample,immune-Tfh; Emory,totalSample,immune-CD8_naive; Emory,totalSample,immune-Cytotoxic; Emory,totalSample,immune-Exhausted; Homo_723,totalSample,immune-Th1; Homo_723,totalSample,immune-CD4_T; Homo_723,totalSample,immune-Neutrophil; Homo_723,totalSample,immune-Tr1; Homo_723,totalSample,immune-Tfh; Homo_723,totalSample,immune-iTreg; Homo_723,totalSample,immune-CD4_naive; Homo_723,totalSample,immune-Th2; Homo_723,totalSample,immune-DC; Homo_723,totalSample,immune-Cytotoxic; Homo_723,totalSample,immune-Bcell; Homo_714,totalSample,immune-Th1; Homo_714,totalSample,immune-CD4_T; Homo_714,totalSample,immune-Neutrophil; Homo_714,totalSample,immune-Tfh; Homo_714,totalSample,immune-CD4_naive; Homo_714,totalSample,immune-Tr1; Homo_714,totalSample,immune-DC; Homo_714,totalSample,immune-Th2; Homo_714,totalSample,immune-Central_memory; Homo_714,totalSample,immune-NKT; Homo_633,totalSample,immune-Th1; Homo_633,totalSample,immune-Tfh; Homo_633,totalSample,immune-CD4_T; Homo_633,totalSample,immune-CD4_naive; Homo_633,totalSample,immune-Neutrophil; Homo_633,totalSample,immune-DC; Homo_633,totalSample,immune-NKT; Homo_633,totalSample,immune-MAIT; Homo_633,totalSample,immune-Central_memory; Homo_633,totalSample,immune-Tr1; MCSA,totalSample,immune-DC; MCSA,totalSample,immune-Bcell; MCSA,totalSample,immune-Monocyte; MCSA,totalSample,immune-Macrophage; MCSA,totalSample,immune-CD4_T; MCSA,totalSample,immune-CD4_naive; MCSA,totalSample,immune-Tr1; MCSA,totalSample,immune-nTreg; MCSA,totalSample,immune-iTreg; MCSA,totalSample,immune-Th1; MCSA,totalSample,immune-Th2; MCSA,totalSample,immune-Tfh; MCSA,totalSample,immune-CD8_naive; MCSA,totalSample,immune-Cytotoxic; MCSA,totalSample,immune-MAIT
BBOX1	ACOM,AD,male.vs.female; ROSMAP,female,AD.vs.control; ROSMAP,totalSample,immune-InfiltrationScore
LRRC69	ACOM,AD,male.vs.female; ROSMAP,female,AD.vs.control; ROSMAP,female,MCI.vs.control; ACOM,AD,age; ACOM,totalSample,age; ROSMAP,AD,MMSE; Emory,totalSample,immune-Tr1; Emory,totalSample,immune-iTreg; Homo_723,totalSample,immune-CD4_naive; Homo_723,totalSample,immune-CD4_T; Homo_723,totalSample,immune-iTreg; Homo_723,totalSample,immune-Tr1; Homo_723,totalSample,immune-Tfh; Homo_723,totalSample,immune-Central_memory; Homo_723,totalSample,immune-Th2; Homo_723,totalSample,immune-Th1; Homo_723,totalSample,immune-nTreg; Homo_714,totalSample,immune-CD4_naive; Homo_714,totalSample,immune-CD4_T; Homo_714,totalSample,immune-Tfh; Homo_714,totalSample,immune-Tr1; Homo_714,totalSample,immune-iTreg; Homo_714,totalSample,immune-Th1; Homo_714,totalSample,immune-Central_memory; Homo_633,totalSample,immune-Tfh; Homo_633,totalSample,immune-CD4_T; Homo_633,totalSample,immune-CD4_naive; Homo_633,totalSample,immune-Tr1; Homo_633,totalSample,immune-iTreg; Homo_633,totalSample,immune-Th1; Homo_633,totalSample,immune-Central_memory; MCSA,totalSample,immune-DC; MCSA,totalSample,immune-Macrophage; MCSA,totalSample,immune-CD4_T; MCSA,totalSample,immune-CD4_naive; MCSA,totalSample,immune-Tr1; MCSA,totalSample,immune-nTreg; MCSA,totalSample,immune-iTreg; MCSA,totalSample,immune-Th2; MCSA,totalSample,immune-Th17; MCSA,totalSample,immune-Tfh; MCSA,totalSample,immune-CD8_naive; MCSA,totalSample,immune-Central_memory; ROSMAP,totalSample,immune-InfiltrationScore
RSPH10B2	ACOM,AD,male.vs.female; Homo_723,female,AD.vs.control; Homo_723,female,AD.vs.MCI; Homo_723,male,AD.vs.MCI; Homo_714,male,AD.vs.MCI; Homo_633,female,AD.vs.control; Homo_633,female,AD.vs.MCI; ROSMAP,AD,last.visit.age; ROSMAP,MCI,last.visit.age; ROSMAP,MCI,sample.age; Emory,totalSample,immune-Th17; Homo_633,totalSample,immune-Th2; Homo_633,totalSample,immune-Exhausted; ROSMAP,totalSample,immune-InfiltrationScore; SRP223445,totalSample,immune-InfiltrationScore; ROSMAP,female-excludeAge90+,survival; ROSMAP,female-includeAge90+,survival
IDNK	ACOM,AD,male.vs.female; Homo_723,female,AD.vs.control; Homo_723,male,AD.vs.control; Homo_723,female,AD.vs.MCI; Homo_723,male,AD.vs.MCI; Homo_714,female,AD.vs.control; Homo_714,male,AD.vs.control; Homo_714,female,AD.vs.MCI; Homo_714,male,AD.vs.MCI; Homo_633,female,AD.vs.control; Homo_633,female,AD.vs.MCI; ROSMAP,male,AD.vs.control; MCSA,MCI,age; ROSMAP,MCI,last.visit.age; Emory,totalSample,immune-Macrophage; Emory,totalSample,immune-NK; Emory,totalSample,immune-Neutrophil; Emory,totalSample,immune-CD4_T; Emory,totalSample,immune-CD8_T; Emory,totalSample,immune-Tr1; Emory,totalSample,immune-iTreg; Emory,totalSample,immune-Tfh; Emory,totalSample,immune-Exhausted; Emory,totalSample,immune-Central_memory; Emory,totalSample,immune-Effector_memory; Emory,totalSample,immune-InfiltrationScore; Homo_723,totalSample,immune-Tfh; Homo_723,totalSample,immune-iTreg; Homo_723,totalSample,immune-Th2; Homo_723,totalSample,immune-CD4_T; Homo_723,totalSample,immune-Tr1; Homo_723,totalSample,immune-InfiltrationScore; Homo_723,totalSample,immune-CD4_naive; Homo_723,totalSample,immune-Effector_memory; Homo_723,totalSample,immune-Macrophage; Homo_723,totalSample,immune-Central_memory; Homo_723,totalSample,immune-MAIT; Homo_723,totalSample,immune-nTreg; Homo_723,totalSample,immune-CD8_T; Homo_723,totalSample,immune-DC; Homo_714,totalSample,immune-Tfh; Homo_714,totalSample,immune-Th2; Homo_714,totalSample,immune-Effector_memory; Homo_714,totalSample,immune-CD4_T; Homo_714,totalSample,immune-Tr1; Homo_714,totalSample,immune-CD4_naive; Homo_714,totalSample,immune-iTreg; Homo_714,totalSample,immune-InfiltrationScore; Homo_714,totalSample,immune-Central_memory; Homo_714,totalSample,immune-Macrophage; Homo_714,totalSample,immune-nTreg; Homo_714,totalSample,immune-MAIT; Homo_714,totalSample,immune-Neutrophil; Homo_714,totalSample,immune-CD8_T; Homo_633,totalSample,immune-Tfh; Homo_633,totalSample,immune-CD4_naive; Homo_633,totalSample,immune-CD4_T; Homo_633,totalSample,immune-Effector_memory; Homo_633,totalSample,immune-InfiltrationScore; Homo_633,totalSample,immune-Central_memory; Homo_633,totalSample,immune-Tr1; Homo_633,totalSample,immune-Neutrophil; Homo_633,totalSample,immune-iTreg; Homo_633,totalSample,immune-nTreg; Homo_633,totalSample,immune-Macrophage; Homo_633,totalSample,immune-Th2; Homo_633,totalSample,immune-MAIT; Homo_633,totalSample,immune-NKT; Homo_633,totalSample,immune-Th1; Homo_633,totalSample,immune-Bcell; MCSA,totalSample,immune-DC; MCSA,totalSample,immune-Macrophage; MCSA,totalSample,immune-Neutrophil; MCSA,totalSample,immune-CD4_T; MCSA,totalSample,immune-CD4_naive; MCSA,totalSample,immune-Tr1; MCSA,totalSample,immune-nTreg; MCSA,totalSample,immune-iTreg; MCSA,totalSample,immune-Th2; MCSA,totalSample,immune-Tfh; MCSA,totalSample,immune-CD8_naive; MCSA,totalSample,immune-MAIT; MCSA,totalSample,immune-Central_memory; [illegible]
FER1L6	ACOM,AD,male.vs.female; Homo_633,male,MCI.vs.control; ROSMAP,female,AD.vs.control; ROSMAP,male,AD.vs.control; ROSMAP,female,MCI.vs.control; Emory,totalSample,immune-Macrophage; ROSMAP,totalSample,immune-InfiltrationScore
TBX1	ACOM,AD,male.vs.female; Homo_723,female,AD.vs.control; Homo_723,male,AD.vs.control; Homo_723,female,AD.vs.MCI; Homo_723,male,AD.vs.MCI; Homo_714,female,AD.vs.control; Homo_714,male,AD.vs.control; Homo_714,female,AD.vs.MCI; Homo_714,male,AD.vs.MCI; Homo_633,female,MCI.vs.control; ROSMAP,female,AD.vs.control; ROSMAP,female,AD.vs.MCI; ROSMAP,MCI,last.visit.age
FYB2	ACOM,AD,male.vs.female; Homo_723,female,AD.vs.control; ROSMAP,female,AD.vs.control; ROSMAP,female,MCI.vs.control; ROSMAP,control,male.vs.female; ROSMAP,MCI,last.visit.age; ROSMAP,MCI,sample.age; Emory,totalSample,immune-CD8_T; Emory,totalSample,immune-Gamma_delta; Emory,totalSample,immune-CD4_naive; ROSMAP,totalSample,immune-
CLDN1	ACOM,AD,male.vs.female; Homo_723,female,AD.vs.control; Homo_723,female,AD.vs.MCI; Homo_723,male,AD.vs.MCI; Homo_633,female,MCI.vs.control; ROSMAP,male,AD.vs.control; MCSA,MCI,age
RGL3	ACOM,AD,male.vs.female; ROSMAP,female,AD.vs.control; ROSMAP,control,male.vs.female; ACOM,totalSample,age; Emory,MCI,age; Emory,totalSample,immune-Neutrophil; Emory,totalSample,immune-CD8_T; Emory,totalSample,immune-Gamma_delta; Emory,totalSample,immune-Th17; Emory,totalSample,immune-Tfh; Emory,totalSample,immune-CD8_naive; Emory,totalSample,immune-Cytotoxic; Emory,totalSample,immune-Exhausted; Homo_714,totalSample,immune-Neutrophil; Homo_633,totalSample,immune-Neutrophil; Homo_633,totalSample,immune-MAIT; MCSA,totalSample,immune-DC; MCSA,totalSample,immune-Bcell; MCSA,totalSample,immune-Monocyte; MCSA,totalSample,immune-Macrophage; MCSA,totalSample,immune-Neutrophil; MCSA,totalSample,immune-Gamma_delta; MCSA,totalSample,immune-iTreg; MCSA,totalSample,immune-Th1; MCSA,totalSample,immune-MAIT
ADHFE1	ACOM,AD,male.vs.female; ROSMAP,female,AD.vs.control; ACOM,totalSample,age; MCSA,totalSample,age; MCSA,control,age; ROSMAP,AD,last.visit.age; Emory,totalSample,immune-Macrophage; Emory,totalSample,immune-Neutrophil; Emory,totalSample,immune-CD4_T; Emory,totalSample,immune-CD8_T; Emory,totalSample,immune-Tr1; Emory,totalSample,immune-iTreg; Emory,totalSample,immune-Exhausted; Emory,totalSample,immune-Effector_memory; Emory,totalSample,immune-InfiltrationScore; Homo_723,totalSample,immune-CD4_naive; Homo_723,totalSample,immune-CD4_T; Homo_723,totalSample,immune-InfiltrationScore; Homo_723,totalSample,immune-Effector_memory; Homo_723,totalSample,immune-nTreg; Homo_723,totalSample,immune-Tfh; Homo_723,totalSample,immune-Tr1; Homo_723,totalSample,immune-iTreg; Homo_723,totalSample,immune-Monocyte; Homo_723,totalSample,immune-Th2; Homo_723,totalSample,immune-Neutrophil; Homo_714,totalSample,immune-CD4_naive; Homo_714,totalSample,immune-InfiltrationScore; Homo_714,totalSample,immune-Effector_memory; Homo_714,totalSample,immune-CD4_T; Homo_714,totalSample,immune-MAIT; Homo_714,totalSample,immune-nTreg; Homo_714,totalSample,immune-Neutrophil; Homo_714,totalSample,immune-Tr1; Homo_714,totalSample,immune-Tfh; Homo_714,totalSample,immune-iTreg; Homo_714,totalSample,immune-Macrophage; Homo_714,totalSample,immune-Monocyte; Homo_714,totalSample,immune-Th1; Homo_714,totalSample,immune-Bcell; Homo_633,totalSample,immune-Effector_memory; Homo_633,totalSample,immune-InfiltrationScore; Homo_633,totalSample,immune-CD4_naive; Homo_633,totalSample,immune-CD4_T; Homo_633,totalSample,immune-MAIT; Homo_633,totalSample,immune-nTreg; Homo_633,totalSample,immune-Tr1; Homo_633,totalSample,immune-Tfh; Homo_633,totalSample,immune-Macrophage; Homo_633,totalSample,immune-Neutrophil; Homo_633,totalSample,immune-iTreg; Homo_633,totalSample,immune-Bcell; Homo_633,totalSample,immune-Monocyte; Homo_633,totalSample,immune-CD8_T; Homo_633,totalSample,immune-Gamma_delta; MCSA,totalSample,immune-Monocyte; MCSA,totalSample,immune-Macrophage; MCSA,totalSample,immune-Neutrophil; MCSA,totalSample,immune-CD4_T; MCSA,totalSample,immune-CD4_naive; MCSA,totalSample,immune-Tr1; MCSA,totalSample,immune-nTreg; MCSA,totalSample,immune-iTreg; MCSA,totalSample,immune-Th17; MCSA,totalSample,immune-Tfh; MCSA,totalSample,immune-MAIT; MCSA,totalSample,immune-Central_memory; MCSA,totalSample,immune-Effector_memory; MCSA,totalSample,immune-InfiltrationScore; ROSMAP,totalSample,immune-Effector_memory
CDC20B	ACOM,AD,male.vs.female; Homo_723,female,AD.vs.control; Homo_723,male,AD.vs.control; Homo_723,female,AD.vs.MCI; Homo_723,male,MCI.vs.control; Homo_714,female,MCI.vs.control; Homo_714,male,MCI.vs.control; Homo_633,female,MCI.vs.control; Homo_633,male,MCI.vs.control; ROSMAP,female,AD.vs.control; ROSMAP,female,MCI.vs.control
LRRC2	ACOM,AD,male.vs.female; Homo_723,female,AD.vs.control; Homo_723,male,AD.vs.control; Homo_723,female,AD.vs.MCI; Homo_714,female,AD.vs.control; Homo_714,male,AD.vs.control; Homo_714,female,AD.vs.MCI; Homo_714,male,AD.vs.MCI; Homo_633,female,AD.vs.control; Homo_633,female,AD.vs.MCI; Homo_633,female,MCI.vs.control; ACOM,totalSample,age; Emory,MCI,age; Homo_723,totalSample,immune-Bcell; ROSMAP,totalSample,immune-InfiltrationScore; SRP223445,totalSample,immune-InfiltrationScore

MAL2	ACOM,AD,male.vs.female; Homo_723,female,AD.vs.control; Homo_723,male,AD.vs.control; Homo_723,female,AD.vs.MCI; Homo_723,male,AD.vs.MCI; Homo_714,male,AD.vs.control; Homo_714,male,AD.vs.MCI; Homo_723,totalSample,immune-Monocyte; Homo_714,totalSample,immune-Monocyte; Homo_633,totalSample,immune-Monocyte; MCSA,totalSample,immune-DC; MCSA,totalSample,immune-Monocyte; MCSA,totalSample,immune-iTreg
SYT17	ACOM,AD,male.vs.female; Homo_723,female,AD.vs.control; Homo_723,male,AD.vs.control; Homo_723,female,AD.vs.MCI; Homo_723,male,AD.vs.MCI; Homo_714,female,AD.vs.control; Homo_714,female,AD.vs.MCI; Homo_633,female,AD.vs.control; Homo_633,female,AD.vs.MCI; ROSMAP,female,AD.vs.control; ROSMAP,male,AD.vs.control; ROSMAP,female,MCI.vs.control; ROSMAP,male,MCI.vs.control; Emory,MCI,age; Emory,totalSample,immune-B_cell; Emory,totalSample,immune-Monocyte; Emory,totalSample,immune-Macrophage; Emory,totalSample,immune-NK; Emory,totalSample,immune-Neutrophil; Emory,totalSample,immune-CD4_T; Emory,totalSample,immune-Tr1; Emory,totalSample,immune-iTreg; Emory,totalSample,immune-InfiltrationScore; Homo_723,totalSample,immune-Neutrophil; Homo_723,totalSample,immune-DC; Homo_723,totalSample,immune-nTreg; Homo_723,totalSample,immune-InfiltrationScore; Homo_723,totalSample,immune-CD4_naive; Homo_723,totalSample,immune-Central_memory; Homo_723,totalSample,immune-Th1; Homo_723,totalSample,immune-CD4_T; Homo_714,totalSample,immune-Neutrophil; Homo_714,totalSample,immune-DC; Homo_714,totalSample,immune-InfiltrationScore; Homo_714,totalSample,immune-NK; Homo_714,totalSample,immune-nTreg; Homo_714,totalSample,immune-Th1; Homo_714,totalSample,immune-CD4_naive; Homo_633,totalSample,immune-Neutrophil; Homo_633,totalSample,immune-nTreg; Homo_633,totalSample,immune-DC; Homo_633,totalSample,immune-InfiltrationScore; Homo_633,totalSample,immune-Tfh; Homo_633,totalSample,immune-Tr1; Homo_633,totalSample,immune-CD4_T; Homo_633,totalSample,immune-Central_memory; Homo_633,totalSample,immune-CD4_naive; Homo_633,totalSample,immune-Effector_memory; Homo_633,totalSample,immune-NK; Homo_633,totalSample,immune-Macrophage; MCSA,totalSample,immune-DC; MCSA,totalSample,immune-Bcell; MCSA,totalSample,immune-Monocyte; MCSA,totalSample,immune-NK; MCSA,totalSample,immune-Neutrophil; MCSA,totalSample,immune-CD4_T; MCSA,totalSample,immune-Gamma_delta; MCSA,totalSample,immune-CD4_naive; MCSA,totalSample,immune-Tr1; MCSA,totalSample,immune-nTreg; MCSA,totalSample,immune-Tfh; MCSA,totalSample,immune-Central_memory; MCSA,totalSample,immune-Effector_memory; MCSA,totalSample,immune-InfiltrationScore; ROSMAP,totalSample,immune-InfiltrationScore; [illegible]
SETBP1	ACOM,AD,male.vs.female; Homo_723,female,AD.vs.MCI; Homo_723,male,AD.vs.MCI; Homo_723,female,MCI.vs.control; Homo_723,male,MCI.vs.control; Homo_723,AD,male.vs.female; Homo_723,MCI,male.vs.female; Homo_714,female,AD.vs.MCI; Homo_714,female,MCI.vs.control; Homo_714,male,MCI.vs.control; Homo_714,MCI,male.vs.female; Homo_633,female,AD.vs.MCI; Homo_633,female,MCI.vs.control; Homo_633,male,MCI.vs.control; Homo_633,control,male.vs.female; Homo_633,MCI,male.vs.female; ROSMAP,male,AD.vs.control; ROSMAP,AD,male.vs.female; Emory,totalSample,immune-B_cell; Emory,totalSample,immune-Monocyte; Emory,totalSample,immune-Macrophage; Emory,totalSample,immune-NK; Emory,totalSample,immune-Neutrophil; Emory,totalSample,immune-CD4_T; Emory,totalSample,immune-Tr1; Emory,totalSample,immune-iTreg; Emory,totalSample,immune-Exhausted; Emory,totalSample,immune-InfiltrationScore; Homo_723,totalSample,immune-Monocyte; Homo_723,totalSample,immune-NKT; Homo_723,totalSample,immune-CD4_naive; Homo_723,totalSample,immune-Th1; Homo_723,totalSample,immune-InfiltrationScore; Homo_723,totalSample,immune-Central_memory; Homo_723,totalSample,immune-Neutrophil; Homo_723,totalSample,immune-DC; Homo_714,totalSample,immune-NKT; Homo_714,totalSample,immune-Monocyte; Homo_714,totalSample,immune-Th1; Homo_714,totalSample,immune-CD4_naive; Homo_714,totalSample,immune-InfiltrationScore; Homo_633,totalSample,immune-NKT; Homo_633,totalSample,immune-Monocyte; Homo_633,totalSample,immune-InfiltrationScore; Homo_633,totalSample,immune-Th1; Homo_633,totalSample,immune-CD4_naive; MCSA,totalSample,immune-Bcell; MCSA,totalSample,immune-Monocyte; MCSA,totalSample,immune-Macrophage; MCSA,totalSample,immune-Neutrophil; MCSA,totalSample,immune-CD4_T; MCSA,totalSample,immune-CD4_naive; MCSA,totalSample,immune-Tr1; MCSA,totalSample,immune-nTreg; MCSA,totalSample,immune-iTreg; MCSA,totalSample,immune-Th17; MCSA,totalSample,immune-Tfh; MCSA,totalSample,immune-CD8_naive; MCSA,totalSample,immune-Exhausted; MCSA,totalSample,immune-MAIT; MCSA,totalSample,immune-Central_memory; MCSA,totalSample,immune-Effector_memory; MCSA,totalSample,immune-InfiltrationScore; SRP223445,totalSample,immune-Neutrophil; SRP223445,totalSample,immune-CD4_naive; SRP223445,totalSample,immune-Th17; [illegible]
VAX1	ACOM,AD,male.vs.female; Homo_723,female,AD.vs.control; Homo_723,female,AD.vs.MCI; Homo_714,female,AD.vs.control; ROSMAP,female,AD.vs.control; ROSMAP,AD,last.visit.age; Homo_714,totalSample,immune-Th2; SRP223445,totalSample,immune-InfiltrationScore
ACCS	ACOM,AD,male.vs.female; Homo_723,female,AD.vs.MCI; Homo_714,female,AD.vs.MCI; ACOM,totalSample,age; MCSA,totalSample,age; MCSA,control,age; MCSA,MCI,age; ROSMAP,AD,last.visit.age; Emory,totalSample,immune-CD4_T; Emory,totalSample,immune-Gamma_delta; Emory,totalSample,immune-Tr1; Emory,totalSample,immune-iTreg; Emory,totalSample,immune-Effector_memory; Emory,totalSample,immune-InfiltrationScore; Homo_723,totalSample,immune-nTreg; Homo_723,totalSample,immune-CD4_naive; Homo_723,totalSample,immune-CD4_T; Homo_723,totalSample,immune-Tr1; Homo_723,totalSample,immune-Central_memory; Homo_723,totalSample,immune-iTreg; Homo_723,totalSample,immune-Effector_memory; Homo_723,totalSample,immune-Tfh; Homo_723,totalSample,immune-Monocyte; Homo_714,totalSample,immune-nTreg; Homo_714,totalSample,immune-CD4_naive; Homo_714,totalSample,immune-CD4_T; Homo_714,totalSample,immune-Central_memory; Homo_714,totalSample,immune-Tr1; Homo_714,totalSample,immune-Effector_memory; Homo_714,totalSample,immune-iTreg; Homo_714,totalSample,immune-Monocyte; Homo_633,totalSample,immune-nTreg; Homo_633,totalSample,immune-Effector_memory; Homo_633,totalSample,immune-Tr1; Homo_633,totalSample,immune-CD4_T; Homo_633,totalSample,immune-iTreg; Homo_633,totalSample,immune-CD4_naive; Homo_633,totalSample,immune-Central_memory; Homo_633,totalSample,immune-Tfh; Homo_633,totalSample,immune-InfiltrationScore; Homo_633,totalSample,immune-Monocyte; MCSA,totalSample,immune-DC; MCSA,totalSample,immune-Monocyte; MCSA,totalSample,immune-Neutrophil; MCSA,totalSample,immune-CD4_T; MCSA,totalSample,immune-CD8_T; MCSA,totalSample,immune-CD4_naive; MCSA,totalSample,immune-Tr1; MCSA,totalSample,immune-nTreg; MCSA,totalSample,immune-Th2; MCSA,totalSample,immune-Th17; MCSA,totalSample,immune-Tfh; MCSA,totalSample,immune-Central_memory; MCSA,totalSample,immune-Effector_memory;
ALS2CL	ACOM,AD,male.vs.female; Homo_723,female,AD.vs.control; Homo_723,control,male.vs.female; Homo_714,female,AD.vs.control; Homo_714,control,male.vs.female; Homo_633,control,male.vs.female; ROSMAP,female,AD.vs.control; ROSMAP,male,AD.vs.control; ROSMAP,female,AD.vs.MCI; ROSMAP,male,MCI.vs.control; ROSMAP,MCI,male.vs.female; MCSA,totalSample,age; MCSA,control,age; MCSA,MCI,age; Emory,totalSample,immune-Macrophage; Emory,totalSample,immune-Neutrophil; Emory,totalSample,immune-CD4_T; Emory,totalSample,immune-CD8_T; Emory,totalSample,immune-Gamma_delta; Emory,totalSample,immune-CD4_naive; Emory,totalSample,immune-Tr1; Emory,totalSample,immune-nTreg; Emory,totalSample,immune-iTreg; Emory,totalSample,immune-Th17; Emory,totalSample,immune-Tfh; Emory,totalSample,immune-CD8_naive; Emory,totalSample,immune-Cytotoxic; Emory,totalSample,immune-Central_memory; Emory,totalSample,immune-InfiltrationScore; Homo_723,totalSample,immune-nTreg; Homo_723,totalSample,immune-Central_memory; Homo_723,totalSample,immune-CD4_naive; Homo_723,totalSample,immune-Tfh; Homo_723,totalSample,immune-CD4_T; Homo_723,totalSample,immune-iTreg; Homo_723,totalSample,immune-Tr1; Homo_723,totalSample,immune-Th2; Homo_723,totalSample,immune-Effector_memory; Homo_723,totalSample,immune-CD8_T; Homo_723,totalSample,immune-Monocyte; Homo_723,totalSample,immune-Th17; Homo_723,totalSample,immune-Neutrophil; Homo_714,totalSample,immune-nTreg; Homo_714,totalSample,immune-Central_memory; Homo_714,totalSample,immune-CD4_T; Homo_714,totalSample,immune-Tfh; Homo_714,totalSample,immune-CD4_naive; Homo_714,totalSample,immune-iTreg; Homo_714,totalSample,immune-Tr1; Homo_714,totalSample,immune-Effector_memory; Homo_714,totalSample,immune-Th2; Homo_714,totalSample,immune-Monocyte; Homo_714,totalSample,immune-Th17; Homo_714,totalSample,immune-CD8_T; Homo_714,totalSample,immune-Neutrophil; Homo_633,totalSample,immune-nTreg; Homo_633,totalSample,immune-Central_memory; Homo_633,totalSample,immune-CD4_T; Homo_633,totalSample,immune-Tfh; Homo_633,totalSample,immune-Tr1; Homo_633,totalSample,immune-CD4_naive; Homo_633,totalSample,immune-iTreg; Homo_633,totalSample,immune-Effector_memory; Homo_633,totalSample,immune-Monocyte; Homo_633,totalSample,immune-Th2; Homo_633,totalSample,immune-Th17; Homo_633,totalSample,immune-CD8_T; MCSA,totalSample,immune-DC; MCSA,totalSample,immune-Monocyte; MCSA,totalSample,immune-Neutrophil; MCSA,totalSample,immune-CD4_T; MCSA,totalSample,immune-CD8_T; MCSA,totalSample,immune-Gamma_delta; MCSA,totalSample,immune-CD4_naive; MCSA,totalSample,immune-Tr1; MCSA,totalSample,immune-nTreg; MCSA,totalSample,immune-iTreg; MCSA,totalSample,immune-Th2; MCSA,totalSample,immune-Th17; MCSA,totalSample,immune-Tfh; MCSA,totalSample,immune-CD8_naive; MCSA,totalSample,immune-Central_memory; MCSA,totalSample,immune-Effector_memory; MCSA,totalSample,immune-InfiltrationScore; [illegible]
ZNF626	ACOM,AD,male.vs.female; Emory,totalSample,age; Emory,control,age; ROSMAP,MCI,last.visit.age; Emory,totalSample,immune-DC; Emory,totalSample,immune-Macrophage; Emory,totalSample,immune-CD4_T; Emory,totalSample,immune-Tr1; Emory,totalSample,immune-iTreg; Emory,totalSample,immune-Tfh; Homo_723,totalSample,immune-Tr1; Homo_723,totalSample,immune-CD4_naive; Homo_723,totalSample,immune-CD4_T; Homo_723,totalSample,immune-iTreg; Homo_723,totalSample,immune-Tfh; Homo_723,totalSample,immune-Central_memory; Homo_723,totalSample,immune-Th2; Homo_723,totalSample,immune-nTreg; Homo_723,totalSample,immune-Th1; Homo_723,totalSample,immune-NKT; Homo_723,totalSample,immune-Cytotoxic; Homo_723,totalSample,immune-DC; Homo_723,totalSample,immune-Gamma_delta; Homo_714,totalSample,immune-CD4_T; Homo_714,totalSample,immune-CD4_naive; Homo_714,totalSample,immune-Tfh; Homo_714,totalSample,immune-Tr1; Homo_714,totalSample,immune-Central_memory; Homo_714,totalSample,immune-iTreg; Homo_714,totalSample,immune-NKT; Homo_714,totalSample,immune-Th2; Homo_714,totalSample,immune-nTreg; Homo_714,totalSample,immune-Effector_memory; Homo_714,totalSample,immune-Th1; Homo_714,totalSample,immune-DC; Homo_633,totalSample,immune-CD4_T; Homo_633,totalSample,immune-Tfh; Homo_633,totalSample,immune-CD4_naive; Homo_633,totalSample,immune-Tr1; Homo_633,totalSample,immune-NKT; Homo_633,totalSample,immune-Central_memory; Homo_633,totalSample,immune-iTreg; Homo_633,totalSample,immune-Effector_memory; Homo_633,totalSample,immune-nTreg; Homo_633,totalSample,immune-DC; Homo_633,totalSample,immune-Th1; Homo_633,totalSample,immune-Th2; Homo_633,totalSample,immune-InfiltrationScore; MCSA,totalSample,immune-DC; MCSA,totalSample,immune-Macrophage; MCSA,totalSample,immune-CD4_T; MCSA,totalSample,immune-CD4_naive; MCSA,totalSample,immune-Tr1; MCSA,totalSample,immune-nTreg; MCSA,totalSample,immune-iTreg; MCSA,totalSample,immune-Th17; MCSA,totalSample,immune-Tfh; MCSA,totalSample,immune-CD8_naive; MCSA,totalSample,immune-Cytotoxic; MCSA,totalSample,immune-Exhausted; MCSA,totalSample,immune-MAIT; MCSA,totalSample,immune-Central_memory; ROSMAP,totalSample,immune-InfiltrationScore; SRP223445,totalSample,immune-CD4_naive; ROSMAP,male-includeAge90+,survival
RSPH10B	ACOM,AD,male.vs.female; Homo_723,female,AD.vs.control; Homo_723,male,AD.vs.control; Homo_723,female,AD.vs.MCI; Homo_723,male,AD.vs.MCI; Homo_723,female,MCI.vs.control; Homo_714,female,AD.vs.control; Homo_714,male,AD.vs.control; Homo_714,female,AD.vs.MCI; Homo_714,male,AD.vs.MCI; Homo_714,female,MCI.vs.control; Homo_633,female,MCI.vs.control; ROSMAP,MCI,last.visit.age; ROSMAP,MCI,sample.age; Homo_723,totalSample,immune-Exhausted; Homo_723,totalSample,immune-Monocyte; Homo_723,totalSample,immune-Th17; Homo_723,totalSample,immune-Th2; Homo_714,totalSample,immune-Monocyte; Homo_633,totalSample,immune-Th2; Homo_633,totalSample,immune-Monocyte; Homo_633,totalSample,immune-Exhausted; Homo_633,totalSample,immune-Th17; MCSA,totalSample,immune-CD4_T; MCSA,totalSample,immune-CD4_naive; MCSA,totalSample,immune-Tr1; MCSA,totalSample,immune-nTreg; MCSA,totalSample,immune-iTreg; MCSA,totalSample,immune-Th17; ROSMAP,totalSample,immune-InfiltrationScore
CYP2C8	ACOM,AD,male.vs.female; Homo_723,female,AD.vs.control; Homo_723,male,AD.vs.control; Homo_723,female,AD.vs.MCI; Homo_723,male,AD.vs.MCI; Homo_723,female,MCI.vs.control; Homo_723,male,MCI.vs.control; Homo_714,female,AD.vs.MCI; Homo_714,male,AD.vs.MCI; Homo_714,female,MCI.vs.control; Homo_714,male,MCI.vs.control; Homo_633,female,MCI.vs.control; Homo_633,male,MCI.vs.control; ROSMAP,male,AD.vs.control; Emory,totalSample,immune-B_cell; Homo_723,totalSample,immune-Th2; Homo_723,totalSample,immune-Tfh; Homo_723,totalSample,immune-CD4_T; Homo_723,totalSample,immune-Effector_memory; Homo_723,totalSample,immune-Exhausted; Homo_723,totalSample,immune-CD4_naive; Homo_714,totalSample,immune-Th2; Homo_714,totalSample,immune-Tfh; Homo_714,totalSample,immune-Effector_memory; Homo_714,totalSample,immune-CD4_T; Homo_633,totalSample,immune-Th2; Homo_633,totalSample,immune-Tfh; Homo_633,totalSample,immune-CD4_T; Homo_633,totalSample,immune-Effector_memory; Homo_633,totalSample,immune-Central_memory; Homo_633,totalSample,immune-CD4_naive; Homo_633,totalSample,immune-Th17; Homo_633,totalSample,immune-nTreg; Homo_633,totalSample,immune-Tr1; MCSA,totalSample,immune-Macrophage; MCSA,totalSample,immune-Neutrophil; MCSA,totalSample,immune-CD4_T; MCSA,totalSample,immune-CD4_naive; MCSA,totalSample,immune-Tr1; MCSA,totalSample,immune-nTreg; MCSA,totalSample,immune-iTreg; MCSA,totalSample,immune-Th17; MCSA,totalSample,immune-Tfh; MCSA,totalSample,immune-CD8_naive; MCSA,totalSample,immune-MAIT; MCSA,totalSample,immune-Central_memory; MCSA,totalSample,immune-[illegible]
GZMA	ACOM,AD,male.vs.female; ROSMAP,female,AD.vs.control; ROSMAP,female,MCI.vs.control; ROSMAP,control,male.vs.female; MCSA,totalSample,age; MCSA,control,age; MCSA,MCI,age; ROSMAP,AD,last.visit.age; ROSMAP,AD,sample.age; ROSMAP,totalSample,age; ROSMAP,control,sample.age; ROSMAP,totalSample,MMSE; Emory,totalSample,immune-DC; Emory,totalSample,immune-Monocyte; Emory,totalSample,immune-Macrophage; Emory,totalSample,immune-NK; Emory,totalSample,immune-Neutrophil; Emory,totalSample,immune-CD8_T; Emory,totalSample,immune-Gamma_delta; Emory,totalSample,immune-CD4_naive; Emory,totalSample,immune-nTreg; Emory,totalSample,immune-Th17; Emory,totalSample,immune-Tfh; Emory,totalSample,immune-Cytotoxic; Emory,totalSample,immune-Exhausted; Emory,totalSample,immune-Effector_memory; Emory,totalSample,immune-InfiltrationScore; Homo_723,totalSample,immune-Gamma_delta; Homo_723,totalSample,immune-Cytotoxic; Homo_723,totalSample,immune-Neutrophil; Homo_723,totalSample,immune-Th17; Homo_723,totalSample,immune-MAIT; Homo_723,totalSample,immune-NK; Homo_723,totalSample,immune-Th2; Homo_723,totalSample,immune-InfiltrationScore; Homo_723,totalSample,immune-Exhausted; Homo_714,totalSample,immune-Gamma_delta; Homo_714,totalSample,immune-Cytotoxic; Homo_714,totalSample,immune-Th17; Homo_714,totalSample,immune-Neutrophil; Homo_714,totalSample,immune-MAIT; Homo_714,totalSample,immune-Exhausted; Homo_714,totalSample,immune-CD8_T; Homo_714,totalSample,immune-Th2; Homo_714,totalSample,immune-InfiltrationScore; Homo_714,totalSample,immune-NK; Homo_714,totalSample,immune-nTreg; Homo_714,totalSample,immune-Bcell; Homo_633,totalSample,immune-Gamma_delta; Homo_633,totalSample,immune-Cytotoxic; Homo_633,totalSample,immune-Th17; Homo_633,totalSample,immune-MAIT; Homo_633,totalSample,immune-Neutrophil; Homo_633,totalSample,immune-CD8_T; Homo_633,totalSample,immune-Th2; Homo_633,totalSample,immune-Exhausted; Homo_633,totalSample,immune-nTreg; Homo_633,totalSample,immune-iTreg; Homo_633,totalSample,immune-Tr1; Homo_633,totalSample,immune-InfiltrationScore; Homo_633,totalSample,immune-Bcell; Homo_633,totalSample,immune-NK; MCSA,totalSample,immune-DC; MCSA,totalSample,immune-Monocyte; MCSA,totalSample,immune-Macrophage; MCSA,totalSample,immune-CD4_T; MCSA,totalSample,immune-Gamma_delta; MCSA,totalSample,immune-CD4_naive; MCSA,totalSample,immune-Tr1; MCSA,totalSample,immune-nTreg; MCSA,totalSample,immune-Th2; MCSA,totalSample,immune-Th17; MCSA,totalSample,immune-Cytotoxic; MCSA,totalSample,immune-Exhausted; MCSA,totalSample,immune-MAIT; MCSA,totalSample,immune-Central_memory; ROSMAP,totalSample,immune-Bcell; ROSMAP,totalSample,immune-Neutrophil; ROSMAP,totalSample,immune-CD4_T; ROSMAP,totalSample,immune-Gamma_delta; ROSMAP,totalSample,immune-Th17; ROSMAP,totalSample,immune-Cytotoxic; ROSMAP,totalSample,immune-InfiltrationScore; SRP223445,totalSample,immune-NK; [illegible]
STRIT1	ACOM,AD,male.vs.female; ACOM,AD,age; ACOM,totalSample,age
NKX6-1	ACOM,AD,male.vs.female; ROSMAP,female,AD.vs.control; Emory,totalSample,immune-Tfh; Homo_723,totalSample,immune-DC
ACP5	ACOM,AD,male.vs.female; Homo_723,female,AD.vs.control; Homo_723,male,AD.vs.control; Homo_723,female,AD.vs.MCI; Homo_723,male,AD.vs.MCI; Homo_714,female,AD.vs.control; Homo_714,male,AD.vs.control; Homo_714,female,AD.vs.MCI; Homo_714,male,AD.vs.MCI; Homo_633,female,AD.vs.control; Homo_633,female,AD.vs.MCI; Emory,MCI,age; MCSA,MCI,age; Emory,totalSample,immune-B_cell; Emory,totalSample,immune-Macrophage; Emory,totalSample,immune-NK; Emory,totalSample,immune-Neutrophil; Emory,totalSample,immune-Gamma_delta; Emory,totalSample,immune-Th17; Emory,totalSample,immune-CD8_naive; Emory,totalSample,immune-Exhausted; Emory,totalSample,immune-InfiltrationScore; Homo_723,totalSample,immune-CD8_T; Homo_723,totalSample,immune-Cytotoxic; Homo_723,totalSample,immune-Exhausted; Homo_723,totalSample,immune-InfiltrationScore; Homo_723,totalSample,immune-Th17; Homo_714,totalSample,immune-DC; Homo_714,totalSample,immune-CD8_T; Homo_714,totalSample,immune-Cytotoxic; Homo_714,totalSample,immune-Macrophage; Homo_633,totalSample,immune-DC; Homo_633,totalSample,immune-Neutrophil; Homo_633,totalSample,immune-nTreg; Homo_633,totalSample,immune-NK; MCSA,totalSample,immune-DC; MCSA,totalSample,immune-Monocyte; MCSA,totalSample,immune-Neutrophil; MCSA,totalSample,immune-CD8_T; MCSA,totalSample,immune-NKT; MCSA,totalSample,immune-iTreg; MCSA,totalSample,immune-Th2; MCSA,totalSample,immune-Cytotoxic; MCSA,totalSample,immune-Effector_memory; MCSA,totalSample,immune-InfiltrationScore; SRP223445,totalSample,immune-Neutrophil; SRP223445,totalSample,immune-InfiltrationScore
INSC	ACOM,AD,male.vs.female; Homo_723,female,AD.vs.control; Homo_723,male,AD.vs.control; Homo_723,female,AD.vs.MCI; Homo_723,male,AD.vs.MCI; Homo_714,female,AD.vs.control; Homo_714,male,AD.vs.control; Homo_714,female,AD.vs.MCI; Homo_714,male,AD.vs.MCI; Homo_633,female,AD.vs.control; Homo_633,female,AD.vs.MCI; Emory,totalSample,immune-Monocyte; Emory,totalSample,immune-Macrophage; Emory,totalSample,immune-Neutrophil; Emory,totalSample,immune-CD8_T; Emory,totalSample,immune-Gamma_delta; Emory,totalSample,immune-Th1; Emory,totalSample,immune-CD8_naive; Emory,totalSample,immune-Exhausted; Emory,totalSample,immune-InfiltrationScore; Homo_723,totalSample,immune-Neutrophil; Homo_723,totalSample,immune-Gamma_delta; Homo_714,totalSample,immune-Neutrophil; Homo_714,totalSample,immune-Th1; Homo_714,totalSample,immune-Gamma_delta; Homo_633,totalSample,immune-Neutrophil; Homo_633,totalSample,immune-Exhausted; Homo_633,totalSample,immune-CD8_T; Homo_633,totalSample,immune-MAIT; Homo_633,totalSample,immune-Monocyte; Homo_633,totalSample,immune-Gamma_delta; MCSA,totalSample,immune-Neutrophil; MCSA,totalSample,immune-CD8_T; MCSA,totalSample,immune-Gamma_delta; MCSA,totalSample,immune-Th1; MCSA,totalSample,immune-Th17; MCSA,totalSample,immune-Cytotoxic;
TYRP1	ACOM,AD,male.vs.female; Homo_723,female,AD.vs.control; Homo_723,female,AD.vs.MCI; Homo_723,female,MCI.vs.control; ROSMAP,female,AD.vs.control; ROSMAP,male,AD.vs.control; Homo_723,totalSample,immune-DC; Homo_723,totalSample,immune-Th17; ROSMAP,totalSample,immune-CD4_T; ROSMAP,totalSample,immune-Th17; ROSMAP,totalSample,immune-InfiltrationScore; ROSMAP,male-includeAge90+,survival
TNNC1	ACOM,AD,male.vs.female; Homo_723,male,AD.vs.control; Homo_723,female,MCI.vs.control; Homo_723,male,MCI.vs.control; Homo_714,female,AD.vs.control; Homo_714,male,AD.vs.control; Homo_714,female,AD.vs.MCI; Homo_714,female,MCI.vs.control; Homo_714,male,MCI.vs.control; Homo_633,female,AD.vs.control; Homo_633,female,MCI.vs.control; Homo_633,male,MCI.vs.control; ACOM,totalSample,age; MCSA,totalSample,age; MCSA,control,age; MCSA,MCI,age; Emory,totalSample,immune-Macrophage; Emory,totalSample,immune-NK; Emory,totalSample,immune-Tr1; Emory,totalSample,immune-iTreg; Emory,totalSample,immune-Tfh; Emory,totalSample,immune-MAIT; Homo_723,totalSample,immune-Th2; Homo_723,totalSample,immune-Tfh; Homo_723,totalSample,immune-Effector_memory; Homo_723,totalSample,immune-CD4_T; Homo_723,totalSample,immune-iTreg; Homo_723,totalSample,immune-nTreg; Homo_723,totalSample,immune-Macrophage; Homo_723,totalSample,immune-CD4_naive; Homo_723,totalSample,immune-CD8_T; Homo_723,totalSample,immune-Monocyte; Homo_723,totalSample,immune-Tr1; Homo_714,totalSample,immune-Th2; Homo_714,totalSample,immune-Tfh; Homo_714,totalSample,immune-Central_memory; Homo_714,totalSample,immune-Effector_memory; Homo_714,totalSample,immune-CD4_T; Homo_714,totalSample,immune-nTreg; Homo_714,totalSample,immune-CD8_T; Homo_714,totalSample,immune-Tr1; Homo_633,totalSample,immune-Th2; Homo_633,totalSample,immune-Central_memory; Homo_633,totalSample,immune-Tfh; Homo_633,totalSample,immune-nTreg; Homo_633,totalSample,immune-CD4_T; Homo_633,totalSample,immune-Effector_memory; Homo_633,totalSample,immune-CD4_naive; Homo_633,totalSample,immune-CD8_T; MCSA,totalSample,immune-DC; MCSA,totalSample,immune-Monocyte; MCSA,totalSample,immune-Neutrophil; MCSA,totalSample,immune-CD4_T; MCSA,totalSample,immune-CD8_T; MCSA,totalSample,immune-CD4_naive; MCSA,totalSample,immune-Tr1; MCSA,totalSample,immune-nTreg; MCSA,totalSample,immune-Tfh; MCSA,totalSample,immune-[illegible]
SYT6	ACOM,AD,male.vs.female; Homo_723,female,AD.vs.control; Homo_723,male,AD.vs.control; Homo_723,female,AD.vs.MCI; Homo_723,male,AD.vs.MCI; Homo_633,female,MCI.vs.control; MCSA,totalSample,immune-iTreg; ROSMAP,totalSample,immune-Central_memory; ROSMAP,totalSample,immune-InfiltrationScore
CNPY1	ACOM,AD,male.vs.female; Homo_723,female,AD.vs.control; Homo_723,female,AD.vs.MCI; ROSMAP,female-includeAge90+,survival
PRUNE2	ACOM,AD,male.vs.female; Homo_723,female,AD.vs.control; Homo_723,male,AD.vs.control; Homo_723,female,AD.vs.MCI; Homo_723,male,AD.vs.MCI; Homo_723,male,MCI.vs.control; Homo_714,female,AD.vs.control; Homo_714,male,AD.vs.control; Homo_714,female,AD.vs.MCI; Homo_714,male,AD.vs.MCI; Homo_714,male,MCI.vs.control; Homo_633,female,AD.vs.control; Homo_633,female,AD.vs.MCI; Emory,totalSample,immune-Monocyte; Emory,totalSample,immune-Neutrophil; Emory,totalSample,immune-nTreg; Emory,totalSample,immune-Th1; Emory,totalSample,immune-Tfh; Homo_723,totalSample,immune-Exhausted; Homo_723,totalSample,immune-CD8_T; Homo_723,totalSample,immune-Th17; Homo_714,totalSample,immune-Monocyte; Homo_714,totalSample,immune-Exhausted; Homo_633,totalSample,immune-Monocyte; Homo_633,totalSample,immune-Exhausted

PLAAT3	ACOM,AD,male.vs.female; Homo_723,female,AD.vs.control; Homo_723,male,AD.vs.control; Homo_723,female,AD.vs.MCI; Homo_723,male,AD.vs.MCI; Homo_714,female,AD.vs.control; Homo_714,male,AD.vs.control; Homo_714,female,AD.vs.MCI; Homo_714,male,AD.vs.MCI; Homo_633,female,AD.vs.control; Homo_633,female,AD.vs.MCI; ROSMAP,female,MCI.vs.control; ACOM,AD,age; ROSMAP,AD,last.visit.age; Emory,totalSample,immune-NK; Emory,totalSample,immune-Neutrophil; Emory,totalSample,immune-Gamma_delta; Emory,totalSample,immune-Cytotoxic; Emory,totalSample,immune-Exhausted; Emory,totalSample,immune-Effector_memory; Emory,totalSample,immune-InfiltrationScore; Homo_723,totalSample,immune-NK; Homo_723,totalSample,immune-DC; Homo_723,totalSample,immune-InfiltrationScore; Homo_723,totalSample,immune-Neutrophil; Homo_723,totalSample,immune-Gamma_delta; Homo_723,totalSample,immune-CD8_naive; Homo_723,totalSample,immune-iTreg; Homo_723,totalSample,immune-Th2; Homo_723,totalSample,immune-Cytotoxic; Homo_714,totalSample,immune-InfiltrationScore; Homo_714,totalSample,immune-NK; Homo_714,totalSample,immune-DC; Homo_714,totalSample,immune-Cytotoxic; Homo_714,totalSample,immune-Neutrophil; Homo_714,totalSample,immune-Th2; Homo_714,totalSample,immune-Gamma_delta; Homo_714,totalSample,immune-iTreg; Homo_714,totalSample,immune-Tr1; Homo_714,totalSample,immune-Bcell; Homo_633,totalSample,immune-InfiltrationScore; Homo_633,totalSample,immune-Cytotoxic; Homo_633,totalSample,immune-NK; Homo_633,totalSample,immune-DC; Homo_633,totalSample,immune-Gamma_delta; Homo_633,totalSample,immune-Neutrophil; Homo_633,totalSample,immune-CD8_T; Homo_633,totalSample,immune-Bcell; Homo_633,totalSample,immune-Th17; Homo_633,totalSample,immune-iTreg; Homo_633,totalSample,immune-NKT; Homo_633,totalSample,immune-Th1; MCSA,totalSample,immune-DC; MCSA,totalSample,immune-NK; MCSA,totalSample,immune-Neutrophil; MCSA,totalSample,immune-CD4_T; MCSA,totalSample,immune-CD8_T; MCSA,totalSample,immune-Gamma_delta; MCSA,totalSample,immune-CD4_naive; MCSA,totalSample,immune-Tr1; MCSA,totalSample,immune-nTreg; MCSA,totalSample,immune-iTreg; MCSA,totalSample,immune-Th17; MCSA,totalSample,immune-Cytotoxic; MCSA,totalSample,immune-Exhausted; MCSA,totalSample,immune-Effector_memory; MCSA,totalSample,immune-InfiltrationScore; SRP223445,totalSample,immune-NK; SRP223445,totalSample,immune-Neutrophil; SRP223445,totalSample,immune-CD4_naive; SRP223445,totalSample,immune-InfiltrationScore; ROSMAP,female-excludeAge90+,survival; ROSMAP,male-excludeAge90+,survival
STPG4	ACOM,AD,male.vs.female; Homo_723,female,AD.vs.control; Homo_723,male,AD.vs.control; Homo_723,female,AD.vs.MCI; Homo_723,male,AD.vs.MCI; Homo_714,female,AD.vs.control; Homo_714,male,AD.vs.control; Homo_714,female,AD.vs.MCI; Homo_714,male,AD.vs.MCI; Homo_633,female,AD.vs.MCI; Homo_633,female,MCI.vs.control; Homo_633,male,MCI.vs.control; ROSMAP,female,AD.vs.MCI
GABRP	ACOM,AD,male.vs.female; Homo_723,female,AD.vs.MCI; Homo_723,totalSample,immune-DC
GDF6	ACOM,AD,male.vs.female; ROSMAP,female,AD.vs.control; MCSA,MCI,age; Emory,totalSample,immune-Neutrophil; Emory,totalSample,immune-Gamma_delta; Homo_723,totalSample,immune-Th17; Homo_723,totalSample,immune-DC; ROSMAP,totalSample,immune-iTreg; ROSMAP,female-excludeAge90+,survival; ROSMAP,female-includeAge90+,survival
IRAG2	ACOM,AD,male.vs.female; Emory,MCI,age; MCSA,totalSample,age; MCSA,control,age; MCSA,MCI,age; Emory,totalSample,immune-DC; Emory,totalSample,immune-Monocyte; Emory,totalSample,immune-Neutrophil; Emory,totalSample,immune-CD8_T; Emory,totalSample,immune-Gamma_delta; Emory,totalSample,immune-Th1; Emory,totalSample,immune-Th17; Emory,totalSample,immune-Tfh; Emory,totalSample,immune-CD8_naive; Emory,totalSample,immune-Cytotoxic; Emory,totalSample,immune-Exhausted; Emory,totalSample,immune-MAIT; Homo_723,totalSample,immune-CD8_T; Homo_723,totalSample,immune-Neutrophil; Homo_723,totalSample,immune-Cytotoxic; Homo_723,totalSample,immune-DC; Homo_723,totalSample,immune-Gamma_delta; Homo_723,totalSample,immune-Exhausted; Homo_723,totalSample,immune-NKT; Homo_714,totalSample,immune-CD8_T; Homo_714,totalSample,immune-Neutrophil; Homo_714,totalSample,immune-Exhausted; Homo_714,totalSample,immune-DC; Homo_714,totalSample,immune-Cytotoxic; Homo_714,totalSample,immune-Gamma_delta; Homo_714,totalSample,immune-Monocyte; Homo_714,totalSample,immune-Macrophage; Homo_633,totalSample,immune-DC; Homo_633,totalSample,immune-Neutrophil; Homo_633,totalSample,immune-CD8_T; Homo_633,totalSample,immune-Exhausted; Homo_633,totalSample,immune-NKT; Homo_633,totalSample,immune-Monocyte; Homo_633,totalSample,immune-Cytotoxic; Homo_633,totalSample,immune-Gamma_delta; Homo_633,totalSample,immune-Macrophage; Homo_633,totalSample,immune-Th2; MCSA,totalSample,immune-DC; MCSA,totalSample,immune-Monocyte; MCSA,totalSample,immune-Neutrophil; MCSA,totalSample,immune-CD8_T; MCSA,totalSample,immune-iTreg; MCSA,totalSample,immune-Th2; MCSA,totalSample,immune-Cytotoxic; MCSA,totalSample,immune-Exhausted; MCSA,totalSample,immune-Effector_memory; MCSA,totalSample,immune-InfiltrationScore; SRP223445,totalSample,immune-CD4_naive
SPP1	ACOM,AD,male.vs.female; Emory,control,male.vs.female; Homo_723,female,AD.vs.control; Homo_723,male,AD.vs.control; Homo_723,female,AD.vs.MCI; Homo_723,male,AD.vs.MCI; Homo_714,female,AD.vs.control; Homo_714,male,AD.vs.control; Homo_714,female,AD.vs.MCI; Homo_714,male,AD.vs.MCI; Homo_633,female,AD.vs.control; Homo_633,female,AD.vs.MCI; Emory,MCI,age; MCSA,MCI,age; Emory,totalSample,immune-Neutrophil; Emory,totalSample,immune-CD8_T; Emory,totalSample,immune-Gamma_delta; Emory,totalSample,immune-Exhausted; Homo_723,totalSample,immune-Neutrophil; Homo_714,totalSample,immune-Th2; Homo_633,totalSample,immune-Exhausted; MCSA,totalSample,immune-DC; MCSA,totalSample,immune-Neutrophil; MCSA,totalSample,immune-Exhausted; ROSMAP,totalSample,immune-Effector_memory; ROSMAP,male and female-excludeAge90+,survival; ROSMAP,male and female-includeAge90+,survival
ADAP2	ACOM,AD,male.vs.female; Homo_723,male,AD.vs.control; Homo_723,male,AD.vs.MCI; Homo_723,female,MCI.vs.control; Homo_723,AD,male.vs.female; Homo_723,control,male.vs.female; Homo_723,MCI,male.vs.female; Homo_714,male,AD.vs.control; Homo_714,female,AD.vs.MCI; Homo_714,male,AD.vs.MCI; Homo_714,female,MCI.vs.control; Homo_714,AD,male.vs.female; Homo_714,control,male.vs.female; Homo_714,MCI,male.vs.female; Homo_633,control,male.vs.female; Homo_633,MCI,male.vs.female; ACOM,totalSample,age; Emory,MCI,age; ROSMAP,AD,last.visit.age; Emory,totalSample,immune-DC; Emory,totalSample,immune-Macrophage; Emory,totalSample,immune-CD8_T; Emory,totalSample,immune-Gamma_delta; Emory,totalSample,immune-Tfh; Emory,totalSample,immune-CD8_naive; Emory,totalSample,immune-InfiltrationScore; Homo_723,totalSample,immune-DC; Homo_723,totalSample,immune-Th2; Homo_723,totalSample,immune-iTreg; Homo_723,totalSample,immune-Effector_memory; Homo_714,totalSample,immune-Th2; Homo_714,totalSample,immune-DC; Homo_633,totalSample,immune-Th2; Homo_633,totalSample,immune-DC; MCSA,totalSample,immune-DC; MCSA,totalSample,immune-NK; MCSA,totalSample,immune-CD4_T; MCSA,totalSample,immune-CD8_T; MCSA,totalSample,immune-NKT; MCSA,totalSample,immune-CD4_naive; MCSA,totalSample,immune-Tr1; MCSA,totalSample,immune-nTreg; MCSA,totalSample,immune-iTreg; MCSA,totalSample,immune-Tfh; MCSA,totalSample,immune-CD8_naive; MCSA,totalSample,immune-MAIT; MCSA,totalSample,immune-Central_memory; MCSA,totalSample,immune-Effector_memory; MCSA,totalSample,immune-InfiltrationScore; ROSMAP,totalSample,immune-Effector_memory; SRP223445,totalSample,immune-NK; SRP223445,totalSample,immune-CD4_naive; SRP223445,totalSample,immune-Th17; SRP223445,totalSample,immune-Exhausted; SRP223445,totalSample,immune-InfiltrationScore; ROSMAP,female-excludeAge90+,survival
P3H2	ACOM,AD,male.vs.female; Homo_723,male,AD.vs.MCI; ROSMAP,female,AD.vs.control; ROSMAP,male,MCI.vs.control; ROSMAP,MCI,male.vs.female; ROSMAP,AD,last.visit.age; Emory,totalSample,immune-Gamma_delta; ROSMAP,totalSample,immune-InfiltrationScore; SRP223445,totalSample,immune-Neutrophil
HSPB7	ACOM,AD,male.vs.female; Homo_723,female,AD.vs.MCI; Homo_714,female,AD.vs.MCI; Emory,totalSample,immune-CD8_T; Homo_723,totalSample,immune-DC; Homo_723,totalSample,immune-Th17; Homo_723,totalSample,immune-CD8_T; Homo_714,totalSample,immune-Th2
VEPH1	ACOM,AD,male.vs.female; Homo_723,female,AD.vs.control; Homo_723,male,AD.vs.control; Homo_723,female,AD.vs.MCI; Homo_723,male,AD.vs.MCI; Homo_723,female,MCI.vs.control; Homo_723,male,MCI.vs.control; Homo_714,female,AD.vs.control; Homo_714,male,AD.vs.control; Homo_714,female,AD.vs.MCI; Homo_714,male,AD.vs.MCI; Homo_714,female,MCI.vs.control; Homo_714,male,MCI.vs.control; Homo_633,female,AD.vs.control; Homo_633,female,AD.vs.MCI; SRP223445,female,AD.vs.control; Emory,totalSample,immune-NK; Emory,totalSample,immune-Neutrophil; Emory,totalSample,immune-Th1; Emory,totalSample,immune-Th17; Emory,totalSample,immune-Exhausted; Emory,totalSample,immune-InfiltrationScore; Homo_723,totalSample,immune-Th17; Homo_723,totalSample,immune-CD8_T; Homo_723,totalSample,immune-Cytotoxic; Homo_723,totalSample,immune-Exhausted; Homo_723,totalSample,immune-DC; Homo_714,totalSample,immune-Exhausted; Homo_714,totalSample,immune-Th17; Homo_714,totalSample,immune-CD8_T; Homo_633,totalSample,immune-Gamma_delta; Homo_633,totalSample,immune-Th17; MCSA,totalSample,immune-iTreg; MCSA,totalSample,immune-Cytotoxic; MCSA,totalSample,immune-Exhausted; ROSMAP,totalSample,immune-InfiltrationScore; SRP223445,totalSample,immune-CD4_naive; SRP223445,totalSample,immune-Exhausted
NEK11	ACOM,AD,male.vs.female; Homo_723,female,AD.vs.control; Homo_723,male,AD.vs.control; Homo_723,female,AD.vs.MCI; Homo_723,male,AD.vs.MCI; Homo_714,female,AD.vs.control; Homo_714,male,AD.vs.control; Homo_714,female,AD.vs.MCI; Homo_714,male,AD.vs.MCI; Homo_633,female,AD.vs.control; Homo_633,female,AD.vs.MCI; ROSMAP,female,MCI.vs.control; ACOM,AD,age; Emory,control,age; MCSA,MCI,age; ROSMAP,AD,MMSE; Emory,totalSample,immune-Macrophage; Emory,totalSample,immune-CD4_T; Emory,totalSample,immune-Tr1; Emory,totalSample,immune-iTreg; Emory,totalSample,immune-Th17; Emory,totalSample,immune-Central_memory; Homo_723,totalSample,immune-Th17; Homo_723,totalSample,immune-Exhausted; Homo_723,totalSample,immune-CD4_T; Homo_723,totalSample,immune-CD4_naive; Homo_723,totalSample,immune-Tfh; Homo_723,totalSample,immune-Th2; Homo_723,totalSample,immune-Central_memory; Homo_723,totalSample,immune-Tr1; Homo_723,totalSample,immune-Cytotoxic; Homo_723,totalSample,immune-nTreg; Homo_723,totalSample,immune-Monocyte; Homo_714,totalSample,immune-Th17; Homo_714,totalSample,immune-CD4_naive; Homo_714,totalSample,immune-Tfh; Homo_714,totalSample,immune-CD4_T; Homo_714,totalSample,immune-Central_memory; Homo_714,totalSample,immune-Monocyte; Homo_714,totalSample,immune-Th2; Homo_714,totalSample,immune-Tr1; Homo_633,totalSample,immune-Tfh; Homo_633,totalSample,immune-Th2; Homo_633,totalSample,immune-CD4_T; Homo_633,totalSample,immune-Central_memory; Homo_633,totalSample,immune-CD4_naive; Homo_633,totalSample,immune-Effector_memory; Homo_633,totalSample,immune-Th17; Homo_633,totalSample,immune-Tr1; Homo_633,totalSample,immune-Monocyte; Homo_633,totalSample,immune-Gamma_delta; Homo_633,totalSample,immune-Exhausted; Homo_633,totalSample,immune-nTreg; MCSA,totalSample,immune-DC; MCSA,totalSample,immune-Bcell; MCSA,totalSample,immune-Macrophage; MCSA,totalSample,immune-CD4_T; MCSA,totalSample,immune-CD4_naive; MCSA,totalSample,immune-Tr1; MCSA,totalSample,immune-nTreg; MCSA,totalSample,immune-iTreg; MCSA,totalSample,immune-Th17; MCSA,totalSample,immune-Tfh; MCSA,totalSample,immune-CD8_naive; MCSA,totalSample,immune-Cytotoxic; MCSA,totalSample,immune-Exhausted; MCSA,totalSample,immune-MAIT; [illegible]
DLX6	ACOM,AD,male.vs.female; Homo_723,female,AD.vs.control; ROSMAP,female,AD.vs.control; ROSMAP,male,AD.vs.control; ROSMAP,AD,last.visit.age; ROSMAP,AD,sample.age; Emory,totalSample,immune-CD4_T; Emory,totalSample,immune-Tr1; Emory,totalSample,immune-iTreg; Emory,totalSample,immune-Tfh; Homo_723,totalSample,immune-DC;
PARD6B	ACOM,AD,male.vs.female; MCSA,MCI,age; Emory,totalSample,immune-CD4_T; Emory,totalSample,immune-Gamma_delta; Emory,totalSample,immune-CD4_naive; Emory,totalSample,immune-Tr1; Emory,totalSample,immune-iTreg; Emory,totalSample,immune-Central_memory; Homo_723,totalSample,immune-Tr1; Homo_723,totalSample,immune-iTreg; Homo_723,totalSample,immune-CD4_naive; Homo_723,totalSample,immune-CD4_T; Homo_723,totalSample,immune-Tfh; Homo_723,totalSample,immune-Central_memory; Homo_723,totalSample,immune-Th2; Homo_723,totalSample,immune-Th1; Homo_723,totalSample,immune-DC; Homo_723,totalSample,immune-Cytotoxic; Homo_723,totalSample,immune-nTreg; Homo_723,totalSample,immune-NKT; Homo_714,totalSample,immune-CD4_naive; Homo_714,totalSample,immune-CD4_T; Homo_714,totalSample,immune-Tfh; Homo_714,totalSample,immune-iTreg; Homo_714,totalSample,immune-Tr1; Homo_714,totalSample,immune-DC; Homo_714,totalSample,immune-Th1; Homo_714,totalSample,immune-NKT; Homo_714,totalSample,immune-Th2; Homo_714,totalSample,immune-Central_memory; Homo_633,totalSample,immune-CD4_T; Homo_633,totalSample,immune-Tfh; Homo_633,totalSample,immune-CD4_naive; Homo_633,totalSample,immune-NKT; Homo_633,totalSample,immune-DC; Homo_633,totalSample,immune-Th1; Homo_633,totalSample,immune-iTreg; Homo_633,totalSample,immune-Tr1; Homo_633,totalSample,immune-Central_memory; Homo_633,totalSample,immune-Effector_memory; Homo_633,totalSample,immune-MAIT; Homo_633,totalSample,immune-Th2; MCSA,totalSample,immune-DC; MCSA,totalSample,immune-Monocyte; MCSA,totalSample,immune-Macrophage; MCSA,totalSample,immune-CD4_T; MCSA,totalSample,immune-CD8_T; MCSA,totalSample,immune-CD4_naive; MCSA,totalSample,immune-Tr1; MCSA,totalSample,immune-nTreg; MCSA,totalSample,immune-iTreg; MCSA,totalSample,immune-Th17; MCSA,totalSample,immune-Tfh; MCSA,totalSample,immune-CD8_naive; MCSA,totalSample,immune-Cytotoxic; MCSA,totalSample,immune-Exhausted;
IFNK	ACOM,AD,male.vs.female; Homo_723,female,AD.vs.control; Homo_723,female,AD.vs.MCI; Homo_714,female,AD.vs.control; Homo_714,female,AD.vs.MCI; ROSMAP,totalSample,age; ROSMAP,MCI,last.visit.age; ROSMAP,MCI,sample.age; ROSMAP,AD,MMSE; Homo_723,totalSample,immune-Th2; Homo_723,totalSample,immune-Exhausted; Homo_723,totalSample,immune-Tfh; Homo_723,totalSample,immune-CD4_T; Homo_714,totalSample,immune-Th2; Homo_714,totalSample,immune-Tfh; Homo_633,totalSample,immune-Th2; Homo_633,totalSample,immune-Tfh; Homo_633,totalSample,immune-Th17; Homo_633,totalSample,immune-CD4_T; Homo_633,totalSample,immune-Exhausted; Homo_633,totalSample,immune-Central_memory; MCSA,totalSample,immune-CD4_T; MCSA,totalSample,immune-Tr1; MCSA,totalSample,immune-nTreg; MCSA,totalSample,immune-iTreg; MCSA,totalSample,immune-CD8_naive; MCSA,totalSample,immune-Cytotoxic; MCSA,totalSample,immune-Exhausted; ROSMAP,totalSample,immune-CD4_T;
ACTRT3	ACOM,AD,male.vs.female; MCSA,MCI,age; Emory,totalSample,immune-CD8_T; ROSMAP,female-excludeAge90+,survival; ROSMAP,female-includeAge90+,survival
CDKL2	ACOM,AD,male.vs.female; Homo_723,female,AD.vs.control; Homo_723,female,AD.vs.MCI; Homo_714,male,AD.vs.control; MCSA,MCI,age; Homo_723,totalSample,immune-Th2; Homo_723,totalSample,immune-Tfh; Homo_723,totalSample,immune-CD4_T; Homo_723,totalSample,immune-iTreg; Homo_723,totalSample,immune-DC; Homo_723,totalSample,immune-Tr1; Homo_714,totalSample,immune-Th2; Homo_714,totalSample,immune-Tfh; Homo_633,totalSample,immune-Th2; Homo_633,totalSample,immune-Tfh; Homo_633,totalSample,immune-CD4_T; Homo_633,totalSample,immune-CD4_naive; Homo_633,totalSample,immune-Monocyte; Homo_633,totalSample,immune-DC; MCSA,totalSample,immune-DC; MCSA,totalSample,immune-Macrophage; MCSA,totalSample,immune-CD4_T; MCSA,totalSample,immune-CD4_naive; MCSA,totalSample,immune-Tr1; MCSA,totalSample,immune-nTreg; MCSA,totalSample,immune-iTreg; MCSA,totalSample,immune-Th2; MCSA,totalSample,immune-Tfh; MCSA,totalSample,immune-CD8_naive; MCSA,totalSample,immune-Cytotoxic; MCSA,totalSample,immune-Exhausted; MCSA,totalSample,immune-MAIT; MCSA,totalSample,immune-Central_memory; ROSMAP,totalSample,immune-InfiltrationScore; ROSMAP,male-excludeAge90+,survival
LCA5L	ACOM,AD,male.vs.female; Homo_723,female,AD.vs.control; Homo_723,female,AD.vs.MCI; Homo_714,male,AD.vs.control; Homo_714,male,AD.vs.MCI; Homo_633,female,AD.vs.control; Homo_633,female,AD.vs.MCI; ROSMAP,female,AD.vs.control; ROSMAP,male,AD.vs.control; ROSMAP,male,AD.vs.MCI; ROSMAP,AD,last.visit.age; Homo_723,totalSample,immune-Th2; Homo_723,totalSample,immune-Tfh; Homo_723,totalSample,immune-CD4_T; Homo_714,totalSample,immune-Th2; Homo_714,totalSample,immune-Tfh; Homo_633,totalSample,immune-Th2; Homo_633,totalSample,immune-Tfh; Homo_633,totalSample,immune-Th17; MCSA,totalSample,immune-CD4_T; MCSA,totalSample,immune-Tr1; MCSA,totalSample,immune-nTreg; MCSA,totalSample,immune-iTreg; MCSA,totalSample,immune-Th17; MCSA,totalSample,immune-Cytotoxic
RPS4Y1	ACOM,control,male.vs.female; Emory,control,male.vs.female; Emory,MCI,male.vs.female; Homo_723,female,AD.vs.control; Homo_723,male,AD.vs.control; Homo_723,AD,male.vs.female; Homo_723,control,male.vs.female; Homo_723,MCI,male.vs.female; Homo_714,female,AD.vs.control; Homo_714,male,AD.vs.control; Homo_714,AD,male.vs.female; Homo_714,control,male.vs.female; Homo_714,MCI,male.vs.female; Homo_633,control,male.vs.female; Homo_633,MCI,male.vs.female; ROSMAP,AD,male.vs.female; ROSMAP,control,male.vs.female; ROSMAP,MCI,male.vs.female; SRP223445,AD,male.vs.female; SRP223445,control,male.vs.female; Emory,totalSample,age; Emory,MCI,age; MCSA,totalSample,immune-DC; MCSA,totalSample,immune-Macrophage; MCSA,totalSample,immune-CD4_T; MCSA,totalSample,immune-Th2; MCSA,totalSample,immune-Tfh; ROSMAP,male and female-excludeAge90+,survival
USP9Y	ACOM,control,male.vs.female; Emory,control,male.vs.female; Emory,MCI,male.vs.female; Homo_723,female,AD.vs.MCI; Homo_723,male,AD.vs.MCI; Homo_723,male,MCI.vs.control; Homo_723,control,male.vs.female; Homo_723,MCI,male.vs.female; Homo_714,female,AD.vs.MCI; Homo_714,male,AD.vs.MCI; Homo_714,male,MCI.vs.control; Homo_714,AD,male.vs.female; Homo_714,control,male.vs.female; Homo_714,MCI,male.vs.female; Homo_633,control,male.vs.female; Homo_633,MCI,male.vs.female; ROSMAP,female,AD.vs.control; ROSMAP,AD,male.vs.female; ROSMAP,control,male.vs.female; ROSMAP,MCI,male.vs.female; SRP223445,AD,male.vs.female; SRP223445,control,male.vs.female; Emory,MCI,age; MCSA,MCI,age; ROSMAP,MCI,last.visit.age; MCSA,totalSample,immune-CD4_T; MCSA,totalSample,immune-Tr1; MCSA,totalSample,immune-nTreg; MCSA,totalSample,immune-iTreg; MCSA,totalSample,immune-Central_memory
UTY	ACOM,control,male.vs.female; Emory,control,male.vs.female; Emory,MCI,male.vs.female; Homo_723,female,AD.vs.control; Homo_723,female,AD.vs.MCI; Homo_723,male,AD.vs.MCI; Homo_723,control,male.vs.female; Homo_723,MCI,male.vs.female; Homo_714,female,AD.vs.control; Homo_714,female,AD.vs.MCI; Homo_714,male,AD.vs.MCI; Homo_714,AD,male.vs.female; Homo_714,control,male.vs.female; Homo_714,MCI,male.vs.female; Homo_633,control,male.vs.female; Homo_633,MCI,male.vs.female; ROSMAP,female,AD.vs.control; ROSMAP,AD,male.vs.female; ROSMAP,control,male.vs.female; ROSMAP,MCI,male.vs.female; SRP223445,AD,male.vs.female; SRP223445,control,male.vs.female; MCSA,MCI,age; ROSMAP,MCI,last.visit.age; MCSA,totalSample,immune-Tr1; MCSA,totalSample,immune-nTreg; MCSA,totalSample,immune-iTreg; MCSA,totalSample,immune-Central_memory; ROSMAP,male and female-excludeAge90+,survival
KDM5D	ACOM,control,male.vs.female; Emory,control,male.vs.female; Emory,MCI,male.vs.female; Homo_723,male,AD.vs.control; Homo_723,female,AD.vs.MCI; Homo_723,male,AD.vs.MCI; Homo_723,female,MCI.vs.control; Homo_723,control,male.vs.female; Homo_723,MCI,male.vs.female; Homo_714,male,AD.vs.control; Homo_714,female,AD.vs.MCI; Homo_714,male,AD.vs.MCI; Homo_714,female,MCI.vs.control; Homo_714,AD,male.vs.female; Homo_714,control,male.vs.female; Homo_714,MCI,male.vs.female; Homo_633,male,MCI.vs.control; Homo_633,control,male.vs.female; Homo_633,MCI,male.vs.female; ROSMAP,AD,male.vs.female; ROSMAP,control,male.vs.female; ROSMAP,MCI,male.vs.female; SRP223445,AD,male.vs.female; SRP223445,control,male.vs.female; Emory,MCI,age; Homo_723,totalSample,immune-Effector_memory; Homo_633,totalSample,immune-Effector_memory; MCSA,totalSample,immune-Neutrophil; MCSA,totalSample,immune-CD4_T; MCSA,totalSample,immune-NKT; MCSA,totalSample,immune-CD4_naive; MCSA,totalSample,immune-Tr1; MCSA,totalSample,immune-nTreg; MCSA,totalSample,immune-iTreg; MCSA,totalSample,immune-Tfh; MCSA,totalSample,immune-CD8_naive; MCSA,totalSample,immune-MAIT; MCSA,totalSample,immune-Central_memory; MCSA,totalSample,immune-Effector_memory; ROSMAP,female-excludeAge90+,survival; ROSMAP,male and female-excludeAge90+,survival
DDX3Y	ACOM,control,male.vs.female; Emory,control,male.vs.female; Emory,MCI,male.vs.female; Homo_723,female,AD.vs.control; Homo_723,male,AD.vs.control; Homo_723,male,MCI.vs.control; Homo_723,AD,male.vs.female; Homo_723,control,male.vs.female; Homo_723,MCI,male.vs.female; Homo_714,female,AD.vs.control; Homo_714,male,AD.vs.control; Homo_714,male,MCI.vs.control; Homo_714,AD,male.vs.female; Homo_714,control,male.vs.female; Homo_714,MCI,male.vs.female; Homo_633,male,MCI.vs.control; Homo_633,control,male.vs.female; Homo_633,MCI,male.vs.female; ROSMAP,female,AD.vs.control; ROSMAP,AD,male.vs.female; ROSMAP,control,male.vs.female; ROSMAP,MCI,male.vs.female; SRP223445,AD,male.vs.female; SRP223445,control,male.vs.female; ROSMAP,MCI,last.visit.age; MCSA,totalSample,immune-DC; MCSA,totalSample,immune-Macrophage; MCSA,totalSample,immune-CD4_T; MCSA,totalSample,immune-CD4_naive; MCSA,totalSample,immune-Tr1; MCSA,totalSample,immune-nTreg; MCSA,totalSample,immune-iTreg; MCSA,totalSample,immune-Tfh; MCSA,totalSample,immune-CD8_naive; MCSA,totalSample,immune-Exhausted; MCSA,totalSample,immune-Central_memory; ROSMAP,male and female-excludeAge90+,survival
TMSB4Y	ACOM,control,male.vs.female; Emory,control,male.vs.female; Emory,MCI,male.vs.female; Homo_723,male,AD.vs.control; Homo_723,male,AD.vs.MCI; Homo_723,AD,male.vs.female; Homo_723,control,male.vs.female; Homo_723,MCI,male.vs.female; Homo_714,male,AD.vs.control; Homo_714,male,AD.vs.MCI; Homo_714,AD,male.vs.female; Homo_714,control,male.vs.female; Homo_714,MCI,male.vs.female; Homo_633,control,male.vs.female; Homo_633,MCI,male.vs.female; ROSMAP,AD,male.vs.female; ROSMAP,control,male.vs.female; ROSMAP,MCI,male.vs.female; SRP223445,AD,male.vs.female; SRP223445,control,male.vs.female; Emory,MCI,age
NLGN4Y	ACOM,control,male.vs.female; Emory,MCI,male.vs.female; Homo_723,male,AD.vs.control; Homo_723,female,AD.vs.MCI; Homo_723,male,AD.vs.MCI; Homo_723,female,MCI.vs.control; Homo_723,control,male.vs.female; Homo_723,MCI,male.vs.female; Homo_714,male,AD.vs.control; Homo_714,male,AD.vs.MCI; Homo_714,control,male.vs.female; Homo_714,MCI,male.vs.female; Homo_633,control,male.vs.female; Homo_633,MCI,male.vs.female; ROSMAP,AD,male.vs.female; ROSMAP,control,male.vs.female; ROSMAP,AD,last.visit.age
ZFY	ACOM,control,male.vs.female; Emory,control,male.vs.female; Emory,MCI,male.vs.female; Homo_723,male,AD.vs.control; Homo_723,male,AD.vs.MCI; Homo_723,AD,male.vs.female; Homo_723,control,male.vs.female; Homo_723,MCI,male.vs.female; Homo_714,male,AD.vs.control; Homo_714,male,AD.vs.MCI; Homo_714,AD,male.vs.female; Homo_714,control,male.vs.female; Homo_714,MCI,male.vs.female; Homo_633,control,male.vs.female; ROSMAP,female,AD.vs.control; ROSMAP,AD,male.vs.female; ROSMAP,control,male.vs.female; ROSMAP,MCI,male.vs.female; SRP223445,AD,male.vs.female; SRP223445,control,male.vs.female; Emory,MCI,age; MCSA,totalSample,immune-iTreg
PRY	ACOM,control,male.vs.female; Emory,MCI,male.vs.female; Homo_723,male,AD.vs.control; Homo_723,male,AD.vs.MCI; Homo_723,control,male.vs.female; Homo_723,MCI,male.vs.female; Homo_714,male,AD.vs.control; Homo_714,male,AD.vs.MCI; Homo_714,control,male.vs.female; Homo_714,MCI,male.vs.female; Homo_633,control,male.vs.female; Homo_633,MCI,male.vs.female; ROSMAP,male and female-excludeAge90+,survival; ROSMAP,male and female-includeAge90+,survival

KDM5C	ACOM,control,male.vs.female; Homo_723,MCI,male.vs.female; Homo_714,MCI,male.vs.female; ROSMAP,male,AD.vs.control; ROSMAP,AD,male.vs.female; Emory,MCI,age; MCSA,totalSample,age; MCSA,control,age; ROSMAP,MCI,last.visit.age; ROSMAP,MCI,sample.age; Emory,totalSample,immune-Gamma_delta; Emory,totalSample,immune-CD4_naive; Emory,totalSample,immune-Tr1; Emory,totalSample,immune-iTreg; Emory,totalSample,immune-Th17; Emory,totalSample,immune-Cytotoxic; Emory,totalSample,immune-Central_memory; Emory,totalSample,immune-InfiltrationScore; Homo_723,totalSample,immune-InfiltrationScore; Homo_723,totalSample,immune-nTreg; Homo_723,totalSample,immune-Effector_memory; Homo_723,totalSample,immune-CD4_naive; Homo_723,totalSample,immune-Central_memory; Homo_723,totalSample,immune-Tfh; Homo_723,totalSample,immune-CD4_T; Homo_723,totalSample,immune-iTreg; Homo_723,totalSample,immune-Tr1; Homo_723,totalSample,immune-Monocyte; Homo_723,totalSample,immune-Th2; Homo_723,totalSample,immune-Gamma_delta; Homo_714,totalSample,immune-InfiltrationScore; Homo_714,totalSample,immune-Effector_memory; Homo_714,totalSample,immune-nTreg; Homo_714,totalSample,immune-CD4_naive; Homo_714,totalSample,immune-Tfh; Homo_714,totalSample,immune-Central_memory; Homo_714,totalSample,immune-CD4_T; Homo_714,totalSample,immune-iTreg; Homo_714,totalSample,immune-Tr1; Homo_714,totalSample,immune-Monocyte; Homo_714,totalSample,immune-Th2; Homo_633,totalSample,immune-InfiltrationScore; Homo_633,totalSample,immune-Effector_memory; Homo_633,totalSample,immune-nTreg; Homo_633,totalSample,immune-Central_memory; Homo_633,totalSample,immune-Tr1; Homo_633,totalSample,immune-CD4_T; Homo_633,totalSample,immune-Tfh; Homo_633,totalSample,immune-CD4_naive; Homo_633,totalSample,immune-iTreg; Homo_633,totalSample,immune-Exhausted; Homo_633,totalSample,immune-Monocyte; MCSA,totalSample,immune-DC; MCSA,totalSample,immune-Monocyte; MCSA,totalSample,immune-CD4_T; MCSA,totalSample,immune-NKT; MCSA,totalSample,immune-Gamma_delta; MCSA,totalSample,immune-CD4_naive; MCSA,totalSample,immune-Tr1; MCSA,totalSample,immune-nTreg; MCSA,totalSample,immune-iTreg; MCSA,totalSample,immune-Th1; MCSA,totalSample,immune-Th2; MCSA,totalSample,immune-Th17; MCSA,totalSample,immune-Tfh; MCSA,totalSample,immune-CD8_naive; MCSA,totalSample,immune-Exhausted; MCSA,totalSample,immune-Central_memory; MCSA,totalSample,immune-Effector_memory; MCSA,totalSample,immune-InfiltrationScore; ROSMAP,totalSample,immune-NK; SRP223445,totalSample,immune-NK; SRP223445,totalSample,immune-Neutrophil; SRP223445,totalSample,immune-[illegible]
PRY2	ACOM,control,male.vs.female; Emory,MCI,male.vs.female; Homo_723,male,AD.vs.control; Homo_723,male,AD.vs.MCI; Homo_723,control,male.vs.female; Homo_714,male,AD.vs.control; Homo_714,male,AD.vs.MCI; Homo_714,control,male.vs.female; Homo_633,control,male.vs.female; Homo_633,MCI,male.vs.female; Emory,MCI,age; Emory,totalSample,immune-iTreg; MCSA,totalSample,immune-Tr1; MCSA,totalSample,immune-iTreg; ROSMAP,male and female-excludeAge90+,survival; ROSMAP,male and female-includeAge90+,survival
RPS4X	ACOM,control,male.vs.female; Emory,MCI,male.vs.female; Homo_723,female,AD.vs.control; Homo_723,male,AD.vs.control; Homo_723,male,MCI.vs.control; Homo_723,AD,male.vs.female; Homo_723,control,male.vs.female; Homo_723,MCI,male.vs.female; Homo_714,female,AD.vs.control; Homo_714,male,AD.vs.control; Homo_714,male,MCI.vs.control; Homo_714,AD,male.vs.female; Homo_714,control,male.vs.female; Homo_714,MCI,male.vs.female; Homo_633,male,MCI.vs.control; Homo_633,control,male.vs.female; Homo_633,MCI,male.vs.female; ROSMAP,female,AD.vs.control; ROSMAP,female,MCI.vs.control; Emory,totalSample,immune-B_cell; Emory,totalSample,immune-Macrophage; Emory,totalSample,immune-NK; Emory,totalSample,immune-Neutrophil; Emory,totalSample,immune-CD4_T; Emory,totalSample,immune-CD8_T; Emory,totalSample,immune-Tr1; Emory,totalSample,immune-iTreg; Emory,totalSample,immune-Th1; Emory,totalSample,immune-Tfh; Emory,totalSample,immune-CD8_naive; Emory,totalSample,immune-Exhausted; Emory,totalSample,immune-MAIT; Emory,totalSample,immune-Central_memory; Emory,totalSample,immune-InfiltrationScore; Homo_723,totalSample,immune-Neutrophil; Homo_723,totalSample,immune-Tfh; Homo_723,totalSample,immune-CD8_T; Homo_723,totalSample,immune-CD4_naive; Homo_723,totalSample,immune-nTreg; Homo_723,totalSample,immune-Macrophage; Homo_723,totalSample,immune-CD4_T; Homo_723,totalSample,immune-iTreg; Homo_723,totalSample,immune-Th1; Homo_723,totalSample,immune-Central_memory; Homo_723,totalSample,immune-Tr1; Homo_723,totalSample,immune-Exhausted; Homo_723,totalSample,immune-Th2; Homo_714,totalSample,immune-Neutrophil; Homo_714,totalSample,immune-Macrophage; Homo_714,totalSample,immune-CD8_T; Homo_714,totalSample,immune-Tfh; Homo_714,totalSample,immune-Th1; Homo_714,totalSample,immune-CD4_naive; Homo_714,totalSample,immune-nTreg; Homo_714,totalSample,immune-MAIT; Homo_714,totalSample,immune-CD4_T; Homo_714,totalSample,immune-Central_memory; Homo_714,totalSample,immune-iTreg; Homo_714,totalSample,immune-Exhausted; Homo_714,totalSample,immune-Tr1; Homo_633,totalSample,immune-Neutrophil; Homo_633,totalSample,immune-Macrophage; Homo_633,totalSample,immune-CD8_T; Homo_633,totalSample,immune-CD4_naive; Homo_633,totalSample,immune-Th1; Homo_633,totalSample,immune-MAIT; Homo_633,totalSample,immune-Tfh; Homo_633,totalSample,immune-CD4_T; Homo_633,totalSample,immune-Exhausted; Homo_633,totalSample,immune-nTreg; Homo_633,totalSample,immune-Central_memory; Homo_633,totalSample,immune-Tr1; MCSA,totalSample,immune-DC; MCSA,totalSample,immune-Macrophage; MCSA,totalSample,immune-Neutrophil; MCSA,totalSample,immune-CD4_T; MCSA,totalSample,immune-CD8_T; MCSA,totalSample,immune-Gamma_delta; MCSA,totalSample,immune-CD4_naive; MCSA,totalSample,immune-Tr1; MCSA,totalSample,immune-nTreg; MCSA,totalSample,immune-iTreg; MCSA,totalSample,immune-Th2; MCSA,totalSample,immune-Tfh; MCSA,totalSample,immune-CD8_naive; [illegible]
MAP7D2	ACOM,control,male.vs.female; Emory,MCI,male.vs.female; Homo_723,control,male.vs.female; Homo_723,MCI,male.vs.female; Homo_714,AD,male.vs.female; Homo_714,control,male.vs.female; Homo_714,MCI,male.vs.female; Homo_633,control,male.vs.female; Homo_633,MCI,male.vs.female; ROSMAP,AD,male.vs.female; ROSMAP,control,male.vs.female; ROSMAP,MCI,male.vs.female; MCSA,MCI,age; Homo_723,totalSample,immune-Tr1; Homo_723,totalSample,immune-iTreg; Homo_723,totalSample,immune-CD4_T; Homo_723,totalSample,immune-Th2; Homo_723,totalSample,immune-CD4_naive; Homo_714,totalSample,immune-CD4_T; Homo_714,totalSample,immune-iTreg; Homo_714,totalSample,immune-Tr1; Homo_714,totalSample,immune-Th2; Homo_633,totalSample,immune-Tr1; Homo_633,totalSample,immune-CD4_T; Homo_633,totalSample,immune-InfiltrationScore; Homo_633,totalSample,immune-iTreg; MCSA,totalSample,immune-DC; MCSA,totalSample,immune-Macrophage; MCSA,totalSample,immune-CD4_T; MCSA,totalSample,immune-CD4_naive; MCSA,totalSample,immune-Tr1; MCSA,totalSample,immune-nTreg; MCSA,totalSample,immune-iTreg; MCSA,totalSample,immune-Th17; MCSA,totalSample,immune-Tfh; MCSA,totalSample,immune-CD8_naive; MCSA,totalSample,immune-MAIT; MCSA,totalSample,immune-Central_memory; ROSMAP,totalSample,immune-InfiltrationScore; ROSMAP,male and female-
PRKX	ACOM,control,male.vs.female; Emory,control,male.vs.female; Emory,MCI,male.vs.female; Homo_723,female,AD.vs.control; Homo_723,control,male.vs.female; Homo_723,MCI,male.vs.female; Homo_714,female,AD.vs.control; Homo_714,female,AD.vs.MCI; Homo_714,control,male.vs.female; Homo_714,MCI,male.vs.female; Homo_633,control,male.vs.female; Homo_633,MCI,male.vs.female; ROSMAP,female,AD.vs.control; ROSMAP,male,AD.vs.control; ROSMAP,AD,male.vs.female; ROSMAP,control,male.vs.female; ROSMAP,MCI,male.vs.female; MCSA,MCI,age; Emory,totalSample,immune-Macrophage; Emory,totalSample,immune-NK; Emory,totalSample,immune-Neutrophil; Emory,totalSample,immune-CD4_T; Emory,totalSample,immune-CD8_T; Emory,totalSample,immune-Tr1; Emory,totalSample,immune-iTreg; Emory,totalSample,immune-Tfh; Emory,totalSample,immune-Exhausted; Emory,totalSample,immune-Effector_memory; Emory,totalSample,immune-InfiltrationScore; Homo_723,totalSample,immune-CD4_naive; Homo_723,totalSample,immune-iTreg; Homo_723,totalSample,immune-Tfh; Homo_723,totalSample,immune-CD4_T; Homo_723,totalSample,immune-Tr1; Homo_723,totalSample,immune-InfiltrationScore; Homo_723,totalSample,immune-Effector_memory; Homo_723,totalSample,immune-nTreg; Homo_723,totalSample,immune-Th2; Homo_723,totalSample,immune-Central_memory; Homo_723,totalSample,immune-Th1; Homo_723,totalSample,immune-NKT; Homo_723,totalSample,immune-Macrophage; Homo_723,totalSample,immune-Neutrophil; Homo_714,totalSample,immune-CD4_naive; Homo_714,totalSample,immune-Tfh; Homo_714,totalSample,immune-Effector_memory; Homo_714,totalSample,immune-CD4_T; Homo_714,totalSample,immune-iTreg; Homo_714,totalSample,immune-Tr1; Homo_714,totalSample,immune-InfiltrationScore; Homo_714,totalSample,immune-nTreg; Homo_714,totalSample,immune-Central_memory; Homo_714,totalSample,immune-Th2; Homo_714,totalSample,immune-Th1; Homo_714,totalSample,immune-NKT; Homo_714,totalSample,immune-Neutrophil; Homo_714,totalSample,immune-MAIT; Homo_714,totalSample,immune-Bcell; Homo_714,totalSample,immune-Macrophage; Homo_633,totalSample,immune-CD4_naive; Homo_633,totalSample,immune-Effector_memory; Homo_633,totalSample,immune-CD4_T; Homo_633,totalSample,immune-Tfh; Homo_633,totalSample,immune-Tr1; Homo_633,totalSample,immune-iTreg; Homo_633,totalSample,immune-Central_memory; Homo_633,totalSample,immune-InfiltrationScore; Homo_633,totalSample,immune-NKT; Homo_633,totalSample,immune-nTreg; Homo_633,totalSample,immune-Th1; Homo_633,totalSample,immune-Neutrophil; Homo_633,totalSample,immune-Bcell; Homo_633,totalSample,immune-Th2; Homo_633,totalSample,immune-MAIT; MCSA,totalSample,immune-DC; MCSA,totalSample,immune-Macrophage; MCSA,totalSample,immune-CD4_T; MCSA,totalSample,immune-CD4_naive; MCSA,totalSample,immune-Tr1; MCSA,totalSample,immune-nTreg; MCSA,totalSample,immune-iTreg; MCSA,totalSample,immune-Th17; MCSA,totalSample,immune-Tfh; MCSA,totalSample,immune-CD8_naive; MCSA,totalSample,immune-Cytotoxic; MCSA,totalSample,immune-Exhausted; MCSA,totalSample,immune-MAIT; MCSA,totalSample,immune-Central_memory; ROSMAP,totalSample,immune-Cytotoxic; ROSMAP,totalSample,immune-Effector_memory; SRP223445,totalSample,immune-NK; SRP223445,totalSample,immune-Th17; [illegible]
EIF1AX	ACOM,control,male.vs.female; Emory,control,male.vs.female; Emory,MCI,male.vs.female; Homo_723,AD,male.vs.female; Homo_723,MCI,male.vs.female; Homo_714,AD,male.vs.female; Homo_714,MCI,male.vs.female; Homo_633,MCI,male.vs.female; MCSA,MCI,age; ROSMAP,AD,sample.age; Emory,totalSample,immune-DC; Emory,totalSample,immune-Macrophage; Emory,totalSample,immune-Neutrophil; Emory,totalSample,immune-CD4_T; Emory,totalSample,immune-CD8_T; Emory,totalSample,immune-Tr1; Emory,totalSample,immune-iTreg; Emory,totalSample,immune-Tfh; Emory,totalSample,immune-Exhausted; Emory,totalSample,immune-Central_memory; Emory,totalSample,immune-Effector_memory; Emory,totalSample,immune-InfiltrationScore; Homo_723,totalSample,immune-Tfh; Homo_723,totalSample,immune-Tr1; Homo_723,totalSample,immune-iTreg; Homo_723,totalSample,immune-CD4_T; Homo_723,totalSample,immune-CD4_naive; Homo_723,totalSample,immune-Central_memory; Homo_723,totalSample,immune-Th2; Homo_723,totalSample,immune-Th1; Homo_723,totalSample,immune-InfiltrationScore; Homo_723,totalSample,immune-nTreg; Homo_723,totalSample,immune-NKT; Homo_723,totalSample,immune-DC; Homo_723,totalSample,immune-Cytotoxic; Homo_723,totalSample,immune-MAIT; Homo_723,totalSample,immune-Effector_memory; Homo_714,totalSample,immune-Tfh; Homo_714,totalSample,immune-CD4_T; Homo_714,totalSample,immune-CD4_naive; Homo_714,totalSample,immune-Tr1; Homo_714,totalSample,immune-Central_memory; Homo_714,totalSample,immune-iTreg; Homo_714,totalSample,immune-NKT; Homo_714,totalSample,immune-Th2; Homo_714,totalSample,immune-Th1; Homo_714,totalSample,immune-Effector_memory; Homo_714,totalSample,immune-DC; Homo_714,totalSample,immune-MAIT; Homo_714,totalSample,immune-nTreg; Homo_714,totalSample,immune-InfiltrationScore; Homo_633,totalSample,immune-Tfh; Homo_633,totalSample,immune-CD4_T; Homo_633,totalSample,immune-CD4_naive; Homo_633,totalSample,immune-NKT; Homo_633,totalSample,immune-Tr1; Homo_633,totalSample,immune-Central_memory; Homo_633,totalSample,immune-iTreg; Homo_633,totalSample,immune-Effector_memory; Homo_633,totalSample,immune-DC; Homo_633,totalSample,immune-Th1; Homo_633,totalSample,immune-MAIT; Homo_633,totalSample,immune-InfiltrationScore; Homo_633,totalSample,immune-Th2; Homo_633,totalSample,immune-nTreg; MCSA,totalSample,immune-DC; MCSA,totalSample,immune-Bcell; MCSA,totalSample,immune-Monocyte; MCSA,totalSample,immune-Macrophage; MCSA,totalSample,immune-CD4_T; MCSA,totalSample,immune-CD8_T; MCSA,totalSample,immune-CD4_naive; MCSA,totalSample,immune-Tr1; MCSA,totalSample,immune-nTreg; MCSA,totalSample,immune-iTreg; MCSA,totalSample,immune-Tfh; MCSA,totalSample,immune-CD8_naive; MCSA,totalSample,immune-Cytotoxic; MCSA,totalSample,immune-Exhausted; MCSA,totalSample,immune-MAIT; MCSA,totalSample,immune-Central_memory; SRP223445,totalSample,immune-NK; SRP223445,totalSample,immune-InfiltrationScore
RPS4Y2	ACOM,control,male.vs.female; Homo_723,male,AD.vs.control; Homo_723,male,AD.vs.MCI; Homo_723,control,male.vs.female; Homo_723,MCI,male.vs.female; Homo_714,male,AD.vs.control; Homo_714,male,AD.vs.MCI; Homo_714,control,male.vs.female; Homo_714,MCI,male.vs.female; Homo_633,control,male.vs.female; Homo_633,MCI,male.vs.female; Emory,MCI,age; ROSMAP,male and female-excludeAge90+,survival
SUPT20HL2	ACOM,control,male.vs.female; Homo_723,male,AD.vs.control; Homo_723,male,MCI.vs.control; Homo_723,control,male.vs.female; Homo_723,MCI,male.vs.female; Homo_714,male,AD.vs.control; Homo_714,male,MCI.vs.control; Homo_714,control,male.vs.female; Homo_714,MCI,male.vs.female; Homo_633,male,MCI.vs.control; Homo_633,control,male.vs.female; Homo_633,MCI,male.vs.female; ROSMAP,control,male.vs.female; ROSMAP,MCI,male.vs.female; Homo_723,totalSample,immune-Th17; Homo_714,totalSample,immune-Th17; ROSMAP,totalSample,immune-InfiltrationScore
SEPTIN6	ACOM,control,male.vs.female; Homo_723,female,MCI.vs.control; Homo_723,control,male.vs.female; Homo_723,MCI,male.vs.female; Homo_714,female,AD.vs.MCI; Homo_714,female,MCI.vs.control; Homo_714,control,male.vs.female; Homo_714,MCI,male.vs.female; Homo_633,female,MCI.vs.control; Homo_633,control,male.vs.female; Homo_633,MCI,male.vs.female; ROSMAP,male,AD.vs.control; MCSA,totalSample,age; MCSA,control,age; Emory,totalSample,immune-Monocyte; Emory,totalSample,immune-Macrophage; Emory,totalSample,immune-NK; Emory,totalSample,immune-Neutrophil; Emory,totalSample,immune-CD4_T; Emory,totalSample,immune-CD8_T; Emory,totalSample,immune-CD4_naive; Emory,totalSample,immune-Tr1; Emory,totalSample,immune-nTreg; Emory,totalSample,immune-iTreg; Emory,totalSample,immune-Tfh; Emory,totalSample,immune-CD8_naive; Emory,totalSample,immune-Exhausted; Emory,totalSample,immune-MAIT; Emory,totalSample,immune-Central_memory; Emory,totalSample,immune-InfiltrationScore; Homo_723,totalSample,immune-CD4_naive; Homo_723,totalSample,immune-nTreg; Homo_723,totalSample,immune-CD4_T; Homo_723,totalSample,immune-Tfh; Homo_723,totalSample,immune-Central_memory; Homo_723,totalSample,immune-Th2; Homo_723,totalSample,immune-Tr1; Homo_723,totalSample,immune-iTreg; Homo_723,totalSample,immune-CD8_T; Homo_723,totalSample,immune-Macrophage; Homo_723,totalSample,immune-Th1; Homo_723,totalSample,immune-Neutrophil; Homo_723,totalSample,immune-Exhausted; Homo_723,totalSample,immune-Monocyte; Homo_714,totalSample,immune-CD4_naive; Homo_714,totalSample,immune-CD4_T; Homo_714,totalSample,immune-nTreg; Homo_714,totalSample,immune-Tfh; Homo_714,totalSample,immune-Central_memory; Homo_714,totalSample,immune-Th1; Homo_714,totalSample,immune-Tr1; Homo_714,totalSample,immune-iTreg; Homo_714,totalSample,immune-Macrophage; Homo_714,totalSample,immune-Th2; Homo_714,totalSample,immune-Monocyte; Homo_714,totalSample,immune-Neutrophil; Homo_714,totalSample,immune-CD8_T; Homo_714,totalSample,immune-Effector_memory; Homo_714,totalSample,immune-MAIT; Homo_714,totalSample,immune-Exhausted; Homo_633,totalSample,immune-nTreg; Homo_633,totalSample,immune-CD4_T; Homo_633,totalSample,immune-CD4_naive; Homo_633,totalSample,immune-Tfh; Homo_633,totalSample,immune-Tr1; Homo_633,totalSample,immune-Macrophage; Homo_633,totalSample,immune-Central_memory; Homo_633,totalSample,immune-Neutrophil; Homo_633,totalSample,immune-iTreg; Homo_633,totalSample,immune-Monocyte; Homo_633,totalSample,immune-Th1; Homo_633,totalSample,immune-Effector_memory; Homo_633,totalSample,immune-CD8_T; Homo_633,totalSample,immune-Th2; Homo_633,totalSample,immune-MAIT; Homo_633,totalSample,immune-Exhausted; MCSA,totalSample,immune-Monocyte; MCSA,totalSample,immune-Macrophage; MCSA,totalSample,immune-Neutrophil; MCSA,totalSample,immune-CD4_T; MCSA,totalSample,immune-CD8_T; MCSA,totalSample,immune-CD4_naive; MCSA,totalSample,immune-Tr1; MCSA,totalSample,immune-nTreg; MCSA,totalSample,immune-iTreg; MCSA,totalSample,immune-Th17; MCSA,totalSample,immune-Tfh; MCSA,totalSample,immune-CD8_naive; MCSA,totalSample,immune-MAIT; MCSA,totalSample,immune-Central_memory; MCSA,totalSample,immune-Effector_memory; MCSA,totalSample,immune-InfiltrationScore; [illegible]
LOC107985678	ACOM,control,male.vs.female; Emory,control,male.vs.female; Emory,MCI,male.vs.female; Homo_723,female,AD.vs.control; Homo_723,male,AD.vs.control; Homo_723,male,AD.vs.MCI; Homo_723,MCI,male.vs.female; Homo_714,MCI,male.vs.female; Homo_633,female,AD.vs.control; Homo_633,female,AD.vs.MCI; Homo_633,MCI,male.vs.female; MCSA,control,age; Emory,totalSample,immune-Th17; Homo_723,totalSample,immune-Central_memory; Homo_723,totalSample,immune-CD8_naive; Homo_723,totalSample,immune-Gamma_delta; Homo_633,totalSample,immune-Tfh; Homo_633,totalSample,immune-Exhausted; MCSA,totalSample,immune-CD4_T; MCSA,totalSample,immune-Gamma_delta; MCSA,totalSample,immune-CD4_naive; MCSA,totalSample,immune-Tr1; MCSA,totalSample,immune-nTreg; MCSA,totalSample,immune-iTreg; MCSA,totalSample,immune-Th17; MCSA,totalSample,immune-Tfh; MCSA,totalSample,immune-CD8_naive; MCSA,totalSample,immune-Cytotoxic; MCSA,totalSample,immune-Exhausted; MCSA,totalSample,immune-Central_memory
ZFX	ACOM,control,male.vs.female; Homo_723,control,male.vs.female; Homo_723,MCI,male.vs.female; Homo_714,control,male.vs.female; Homo_714,MCI,male.vs.female; Homo_633,control,male.vs.female; Homo_633,MCI,male.vs.female; ROSMAP,control,male.vs.female; MCSA,MCI,age; ROSMAP,MCI,last.visit.age; Emory,totalSample,immune-CD4_T; Emory,totalSample,immune-Gamma_delta; Emory,totalSample,immune-Tr1; Emory,totalSample,immune-iTreg; Emory,totalSample,immune-Th1; Emory,totalSample,immune-Central_memory; Emory,totalSample,immune-InfiltrationScore; Homo_723,totalSample,immune-iTreg; Homo_723,totalSample,immune-Tr1; Homo_723,totalSample,immune-CD4_naive; Homo_723,totalSample,immune-CD4_T; Homo_723,totalSample,immune-Tfh; Homo_723,totalSample,immune-Th2; Homo_723,totalSample,immune-InfiltrationScore; Homo_723,totalSample,immune-NKT; Homo_723,totalSample,immune-Central_memory; Homo_723,totalSample,immune-Cytotoxic; Homo_723,totalSample,immune-Effector_memory; Homo_723,totalSample,immune-Gamma_delta; Homo_723,totalSample,immune-DC; Homo_723,totalSample,immune-nTreg; Homo_723,totalSample,immune-Exhausted; Homo_714,totalSample,immune-Tfh; Homo_714,totalSample,immune-iTreg; Homo_714,totalSample,immune-CD4_naive; Homo_714,totalSample,immune-CD4_T; Homo_714,totalSample,immune-Tr1; Homo_714,totalSample,immune-NKT; Homo_714,totalSample,immune-Th2; Homo_714,totalSample,immune-Effector_memory; Homo_714,totalSample,immune-Central_memory; Homo_714,totalSample,immune-InfiltrationScore; Homo_714,totalSample,immune-DC; Homo_714,totalSample,immune-Cytotoxic; Homo_714,totalSample,immune-Exhausted; Homo_714,totalSample,immune-nTreg; Homo_714,totalSample,immune-Gamma_delta; Homo_633,totalSample,immune-NKT; Homo_633,totalSample,immune-Tfh; Homo_633,totalSample,immune-CD4_naive; Homo_633,totalSample,immune-CD4_T; Homo_633,totalSample,immune-Tr1; Homo_633,totalSample,immune-Effector_memory; Homo_633,totalSample,immune-iTreg; Homo_633,totalSample,immune-Central_memory; Homo_633,totalSample,immune-DC; Homo_633,totalSample,immune-Th2; Homo_633,totalSample,immune-InfiltrationScore; Homo_633,totalSample,immune-Exhausted; Homo_633,totalSample,immune-Gamma_delta; Homo_633,totalSample,immune-Cytotoxic; Homo_633,totalSample,immune-Th17; Homo_633,totalSample,immune-Th1; MCSA,totalSample,immune-DC; MCSA,totalSample,immune-Monocyte; MCSA,totalSample,immune-Macrophage; MCSA,totalSample,immune-CD4_T; MCSA,totalSample,immune-CD8_T; MCSA,totalSample,immune-CD4_naive; MCSA,totalSample,immune-Tr1; MCSA,totalSample,immune-nTreg; MCSA,totalSample,immune-iTreg; MCSA,totalSample,immune-Th17; MCSA,totalSample,immune-Tfh; MCSA,totalSample,immune-CD8_naive; MCSA,totalSample,immune-Cytotoxic; MCSA,totalSample,immune-Exhausted; MCSA,totalSample,immune-MAIT; MCSA,totalSample,immune-Central_memory; SRP223445,totalSample,immune-Exhausted; SRP223445,totalSample,immune-InfiltrationScore
H3C15	ACOM,control,male.vs.female; Homo_723,male,AD.vs.control; Homo_723,female,MCI.vs.control; Homo_723,MCI,male.vs.female; Homo_714,female,AD.vs.control; Homo_714,male,AD.vs.control; Homo_714,female,MCI.vs.control; Homo_714,MCI,male.vs.female; Homo_633,female,MCI.vs.control; Homo_633,male,MCI.vs.control; Homo_633,MCI,male.vs.female; ROSMAP,female,AD.vs.control; ROSMAP,male,AD.vs.MCI; ROSMAP,female,MCI.vs.control; ROSMAP,male,MCI.vs.control; ROSMAP,MCI,male.vs.female; ROSMAP,AD,last.visit.age; Homo_723,totalSample,immune-Monocyte; Homo_723,totalSample,immune-Th2; Homo_723,totalSample,immune-DC; Homo_714,totalSample,immune-Th2; Homo_714,totalSample,immune-Tfh; Homo_633,totalSample,immune-Monocyte; Homo_633,totalSample,immune-Th2; MCSA,totalSample,immune-DC; MCSA,totalSample,immune-Macrophage; SRP223445,totalSample,immune-CD4_naive
EIF2S3	ACOM,control,male.vs.female; ROSMAP,female,AD.vs.control; ROSMAP,female,MCI.vs.control; MCSA,MCI,age; ROSMAP,AD,last.visit.age; Emory,totalSample,immune-Macrophage; Emory,totalSample,immune-CD4_T; Emory,totalSample,immune-Gamma_delta; Emory,totalSample,immune-Tr1; Emory,totalSample,immune-iTreg; Emory,totalSample,immune-Central_memory; Emory,totalSample,immune-InfiltrationScore; Homo_723,totalSample,immune-iTreg; Homo_723,totalSample,immune-Tr1; Homo_723,totalSample,immune-Tfh; Homo_723,totalSample,immune-CD4_naive; Homo_723,totalSample,immune-CD4_T; Homo_723,totalSample,immune-Th2; Homo_723,totalSample,immune-Central_memory; Homo_723,totalSample,immune-InfiltrationScore; Homo_723,totalSample,immune-Cytotoxic; Homo_723,totalSample,immune-nTreg; Homo_723,totalSample,immune-Gamma_delta; Homo_723,totalSample,immune-NKT; Homo_723,totalSample,immune-Th1; Homo_723,totalSample,immune-Effector_memory; Homo_714,totalSample,immune-Tfh; Homo_714,totalSample,immune-Tr1; Homo_714,totalSample,immune-iTreg; Homo_714,totalSample,immune-CD4_T; Homo_714,totalSample,immune-CD4_naive; Homo_714,totalSample,immune-Th2; Homo_714,totalSample,immune-NKT; Homo_714,totalSample,immune-Central_memory; Homo_714,totalSample,immune-nTreg; Homo_714,totalSample,immune-Th1; Homo_714,totalSample,immune-Effector_memory; Homo_714,totalSample,immune-InfiltrationScore; Homo_714,totalSample,immune-Cytotoxic; Homo_714,totalSample,immune-Gamma_delta; Homo_714,totalSample,immune-DC; Homo_633,totalSample,immune-Tfh; Homo_633,totalSample,immune-CD4_T; Homo_633,totalSample,immune-Tr1; Homo_633,totalSample,immune-NKT; Homo_633,totalSample,immune-CD4_naive; Homo_633,totalSample,immune-iTreg; Homo_633,totalSample,immune-Central_memory; Homo_633,totalSample,immune-Th2; Homo_633,totalSample,immune-Effector_memory; Homo_633,totalSample,immune-Th1; Homo_633,totalSample,immune-InfiltrationScore; Homo_633,totalSample,immune-Gamma_delta; Homo_633,totalSample,immune-nTreg; Homo_633,totalSample,immune-Cytotoxic; Homo_633,totalSample,immune-DC; Homo_633,totalSample,immune-Th17; MCSA,totalSample,immune-DC; MCSA,totalSample,immune-Monocyte; MCSA,totalSample,immune-Macrophage; MCSA,totalSample,immune-CD4_T; MCSA,totalSample,immune-CD8_T; MCSA,totalSample,immune-CD4_naive; MCSA,totalSample,immune-Tr1; MCSA,totalSample,immune-nTreg; MCSA,totalSample,immune-iTreg; MCSA,totalSample,immune-Th2; MCSA,totalSample,immune-Th17; MCSA,totalSample,immune-Tfh; MCSA,totalSample,immune-CD8_naive; MCSA,totalSample,immune-Cytotoxic; MCSA,totalSample,immune-Exhausted; MCSA,totalSample,immune-MAIT; MCSA,totalSample,immune-Central_memory; ROSMAP,totalSample,immune-Effector_memory; SRP223445,totalSample,immune-NK; SRP223445,totalSample,immune-CD4_naive; SRP223445,totalSample,immune-Th17; SRP223445,totalSample,immune-Exhausted; [illegible]
AMFR	ACOM,control,male.vs.female; Homo_723,female,AD.vs.control; Homo_723,male,AD.vs.control; Homo_723,female,AD.vs.MCI; Homo_723,male,AD.vs.MCI; Homo_723,AD,male.vs.female; Homo_723,control,male.vs.female; Homo_723,MCI,male.vs.female; Homo_714,female,AD.vs.control; Homo_714,male,AD.vs.control; Homo_714,female,AD.vs.MCI; Homo_714,male,AD.vs.MCI; Homo_714,female,MCI.vs.control; Homo_714,AD,male.vs.female; Homo_714,control,male.vs.female; Homo_633,female,AD.vs.control; Homo_633,female,AD.vs.MCI; Homo_633,female,MCI.vs.control; Homo_633,control,male.vs.female; Homo_633,MCI,male.vs.female; Emory,totalSample,immune-Neutrophil; Emory,totalSample,immune-InfiltrationScore; Homo_723,totalSample,immune-Monocyte; Homo_723,totalSample,immune-DC; Homo_723,totalSample,immune-Central_memory; Homo_714,totalSample,immune-Monocyte; Homo_714,totalSample,immune-NKT; Homo_633,totalSample,immune-Monocyte; Homo_633,totalSample,immune-Th1; Homo_633,totalSample,immune-NKT; MCSA,totalSample,immune-Bcell; MCSA,totalSample,immune-Monocyte; MCSA,totalSample,immune-Neutrophil; MCSA,totalSample,immune-CD4_T; MCSA,totalSample,immune-NKT; MCSA,totalSample,immune-Gamma_delta; MCSA,totalSample,immune-CD4_naive; MCSA,totalSample,immune-Tr1; MCSA,totalSample,immune-nTreg; MCSA,totalSample,immune-iTreg; MCSA,totalSample,immune-Th2; MCSA,totalSample,immune-Th17; MCSA,totalSample,immune-Tfh; MCSA,totalSample,immune-CD8_naive; MCSA,totalSample,immune-Exhausted; MCSA,totalSample,immune-Central_memory; MCSA,totalSample,immune-Effector_memory; MCSA,totalSample,immune-InfiltrationScore; SRP223445,totalSample,immune-CD4_naive;

ZBED1	ACOM,control,male.vs.female; Homo_723,control,male.vs.female; Homo_723,MCI,male.vs.female; Homo_714,control,male.vs.female; Homo_714,MCI,male.vs.female; Homo_633,control,male.vs.female; Homo_633,MCI,male.vs.female; Emory,MCI,age; MCSA,MCI,age; Emory,totalSample,immune-Neutrophil; Emory,totalSample,immune-CD8_T; Emory,totalSample,immune-Gamma_delta; Emory,totalSample,immune-Th17; Emory,totalSample,immune-Tfh; Emory,totalSample,immune-CD8_naive; Emory,totalSample,immune-Cytotoxic; Homo_723,totalSample,immune-DC; Homo_723,totalSample,immune-Th1; Homo_723,totalSample,immune-InfiltrationScore; Homo_723,totalSample,immune-Bcell; Homo_714,totalSample,immune-DC; Homo_714,totalSample,immune-Th1; Homo_633,totalSample,immune-DC; Homo_633,totalSample,immune-Th1; MCSA,totalSample,immune-DC; MCSA,totalSample,immune-Monocyte; MCSA,totalSample,immune-Macrophage; MCSA,totalSample,immune-iTreg; MCSA,totalSample,immune-Th2; MCSA,totalSample,immune-Cytotoxic; MCSA,totalSample,immune-Effector_memory; SRP223445,totalSample,immune-InfiltrationScore; ROSMAP,female-excludeAge90+,survival
DACT1	ACOM,control,male.vs.female; Emory,control,male.vs.female; Homo_723,female,AD.vs.control; Homo_723,male,AD.vs.control; Homo_723,female,AD.vs.MCI; Homo_723,male,AD.vs.MCI; Homo_723,control,male.vs.female; Homo_723,MCI,male.vs.female; Homo_714,female,AD.vs.control; Homo_714,control,male.vs.female; Homo_714,MCI,male.vs.female; Homo_633,female,AD.vs.control; Homo_633,female,AD.vs.MCI; Homo_633,control,male.vs.female; Homo_633,MCI,male.vs.female; Emory,totalSample,immune-CD4_T; Emory,totalSample,immune-Gamma_delta; Emory,totalSample,immune-CD4_naive; Emory,totalSample,immune-Tr1; Emory,totalSample,immune-nTreg; Emory,totalSample,immune-iTreg; Emory,totalSample,immune-Tfh; Emory,totalSample,immune-Cytotoxic; Emory,totalSample,immune-Central_memory; Homo_723,totalSample,immune-iTreg; Homo_723,totalSample,immune-Tr1; Homo_723,totalSample,immune-Central_memory; Homo_723,totalSample,immune-CD4_T; Homo_723,totalSample,immune-Tfh; Homo_723,totalSample,immune-Th2; Homo_723,totalSample,immune-nTreg; Homo_723,totalSample,immune-CD4_naive; Homo_723,totalSample,immune-Gamma_delta; Homo_723,totalSample,immune-Cytotoxic; Homo_723,totalSample,immune-Th1; Homo_714,totalSample,immune-Tfh; Homo_714,totalSample,immune-Tr1; Homo_714,totalSample,immune-Central_memory; Homo_714,totalSample,immune-CD4_T; Homo_714,totalSample,immune-Th2; Homo_714,totalSample,immune-nTreg; Homo_714,totalSample,immune-Gamma_delta; Homo_714,totalSample,immune-iTreg; Homo_714,totalSample,immune-CD4_naive; Homo_714,totalSample,immune-Cytotoxic; Homo_714,totalSample,immune-Effector_memory; Homo_633,totalSample,immune-Tfh; Homo_633,totalSample,immune-Central_memory; Homo_633,totalSample,immune-Tr1; Homo_633,totalSample,immune-Gamma_delta; Homo_633,totalSample,immune-CD4_T; Homo_633,totalSample,immune-nTreg; Homo_633,totalSample,immune-Th2; Homo_633,totalSample,immune-CD4_naive; Homo_633,totalSample,immune-iTreg; Homo_633,totalSample,immune-Effector_memory; Homo_633,totalSample,immune-Exhausted; Homo_633,totalSample,immune-Cytotoxic; MCSA,totalSample,immune-CD4_T; MCSA,totalSample,immune-Gamma_delta; MCSA,totalSample,immune-CD4_naive; MCSA,totalSample,immune-Tr1; MCSA,totalSample,immune-nTreg; MCSA,totalSample,immune-iTreg; MCSA,totalSample,immune-Tfh; [illegible]
H1-1	ACOM,control,male.vs.female; Homo_723,female,AD.vs.control; Homo_723,female,MCI.vs.control; Homo_714,female,AD.vs.control; Homo_714,female,MCI.vs.control; Homo_633,female,AD.vs.control; Homo_633,female,MCI.vs.control; Homo_633,male,MCI.vs.control; Emory,totalSample,age; Emory,control,age; ROSMAP,MCI,last.visit.age; Emory,totalSample,immune-Neutrophil; Emory,totalSample,immune-CD4_T; Emory,totalSample,immune-Tr1; Emory,totalSample,immune-Tfh; Emory,totalSample,immune-CD8_naive; Emory,totalSample,immune-Exhausted; Emory,totalSample,immune-InfiltrationScore; Homo_723,totalSample,immune-Monocyte; Homo_723,totalSample,immune-Th2; Homo_723,totalSample,immune-DC; Homo_723,totalSample,immune-Exhausted; Homo_714,totalSample,immune-Monocyte; Homo_714,totalSample,immune-Th2; Homo_633,totalSample,immune-Monocyte; Homo_633,totalSample,immune-Th2; Homo_633,totalSample,immune-Exhausted
DIPK2B	ACOM,control,male.vs.female; Homo_723,female,AD.vs.control; Homo_723,female,AD.vs.MCI; Homo_723,male,AD.vs.MCI; Homo_723,female,MCI.vs.control; Homo_723,male,MCI.vs.control; Homo_723,MCI,male.vs.female; Homo_714,female,AD.vs.control; Homo_714,female,AD.vs.MCI; Homo_714,male,AD.vs.MCI; Homo_714,female,MCI.vs.control; Homo_714,male,MCI.vs.control; Homo_714,MCI,male.vs.female; Homo_633,female,AD.vs.MCI; Homo_633,control,male.vs.female; Homo_633,MCI,male.vs.female; ROSMAP,female,AD.vs.control; ROSMAP,female,MCI.vs.control; ROSMAP,control,male.vs.female; MCSA,MCI,age; ROSMAP,MCI,last.visit.age; ROSMAP,MCI,sample.age; Homo_723,totalSample,immune-Th17; SRP223445,totalSample,immune-CD4_naive; SRP223445,totalSample,immune-Exhausted; SRP223445,totalSample,immune-InfiltrationScore
BRSK2	ACOM,control,male.vs.female; Homo_723,female,AD.vs.control; Homo_723,female,AD.vs.MCI; Homo_723,male,MCI.vs.control; Homo_714,female,MCI.vs.control; Homo_714,male,MCI.vs.control; Homo_633,female,MCI.vs.control; Homo_633,male,MCI.vs.control; ROSMAP,female,AD.vs.control; ROSMAP,AD,last.visit.age; Homo_723,totalSample,immune-Monocyte; Homo_723,totalSample,immune-DC; Homo_723,totalSample,immune-Exhausted; Homo_714,totalSample,immune-Monocyte; Homo_633,totalSample,immune-Monocyte; Homo_633,totalSample,immune-Th2; MCSA,totalSample,immune-CD8_T; MCSA,totalSample,immune-Th2; MCSA,totalSample,immune-Cytotoxic; MCSA,totalSample,immune-Effector_memory
H3C14	ACOM,control,male.vs.female; Homo_723,male,AD.vs.control; Homo_723,female,MCI.vs.control; Homo_723,MCI,male.vs.female; Homo_714,female,AD.vs.control; Homo_714,female,MCI.vs.control; Homo_714,MCI,male.vs.female; Homo_633,female,MCI.vs.control; Homo_633,male,MCI.vs.control; Homo_633,MCI,male.vs.female; ROSMAP,female,AD.vs.control; ROSMAP,male,AD.vs.MCI; ROSMAP,female,MCI.vs.control; ROSMAP,male,MCI.vs.control; ROSMAP,MCI,male.vs.female; MCSA,MCI,age; ROSMAP,AD,last.visit.age; ROSMAP,MCI,last.visit.age; Homo_723,totalSample,immune-Th2; Homo_723,totalSample,immune-DC; Homo_723,totalSample,immune-Monocyte; Homo_714,totalSample,immune-Th2; Homo_714,totalSample,immune-Tfh; Homo_633,totalSample,immune-Monocyte; Homo_633,totalSample,immune-Th2; MCSA,totalSample,immune-DC; MCSA,totalSample,immune-Macrophage; MCSA,totalSample,immune-Th2; SRP223445,totalSample,immune-CD4_naive
SSTR3	ACOM,control,male.vs.female; Homo_723,female,AD.vs.control; Homo_723,male,MCI.vs.control; Homo_723,control,male.vs.female; Homo_723,MCI,male.vs.female; Homo_714,male,MCI.vs.control; Homo_714,control,male.vs.female; Homo_714,MCI,male.vs.female; Homo_633,female,AD.vs.control; Homo_633,female,AD.vs.MCI; Homo_633,male,MCI.vs.control; Homo_633,control,male.vs.female; Homo_633,MCI,male.vs.female; ROSMAP,female,AD.vs.control; ROSMAP,female,AD.vs.MCI; MCSA,totalSample,age; MCSA,control,age; ROSMAP,AD,last.visit.age; Emory,totalSample,immune-CD4_T; Emory,totalSample,immune-Gamma_delta; Emory,totalSample,immune-CD4_naive; Emory,totalSample,immune-Tr1; Emory,totalSample,immune-nTreg; Emory,totalSample,immune-iTreg; Emory,totalSample,immune-Tfh; Emory,totalSample,immune-Cytotoxic; Emory,totalSample,immune-Central_memory; Homo_723,totalSample,immune-nTreg; Homo_723,totalSample,immune-Central_memory; Homo_723,totalSample,immune-Tfh; Homo_723,totalSample,immune-CD4_naive; Homo_723,totalSample,immune-CD4_T; Homo_723,totalSample,immune-iTreg; Homo_723,totalSample,immune-Tr1; Homo_723,totalSample,immune-Th2; Homo_723,totalSample,immune-CD8_T; Homo_723,totalSample,immune-Gamma_delta; Homo_714,totalSample,immune-nTreg; Homo_714,totalSample,immune-Tfh; Homo_714,totalSample,immune-Central_memory; Homo_714,totalSample,immune-Tr1; Homo_714,totalSample,immune-CD4_T; Homo_714,totalSample,immune-CD4_naive; Homo_714,totalSample,immune-iTreg; Homo_714,totalSample,immune-Th2; Homo_714,totalSample,immune-Gamma_delta; Homo_714,totalSample,immune-Th1; Homo_714,totalSample,immune-Effector_memory; Homo_633,totalSample,immune-nTreg; Homo_633,totalSample,immune-Tr1; Homo_633,totalSample,immune-Central_memory; Homo_633,totalSample,immune-Tfh; Homo_633,totalSample,immune-CD4_T; Homo_633,totalSample,immune-CD4_naive; Homo_633,totalSample,immune-iTreg; Homo_633,totalSample,immune-Th2; Homo_633,totalSample,immune-Gamma_delta; Homo_633,totalSample,immune-Effector_memory; MCSA,totalSample,immune-Monocyte; MCSA,totalSample,immune-Neutrophil; MCSA,totalSample,immune-CD4_T; MCSA,totalSample,immune-CD8_T; MCSA,totalSample,immune-Gamma_delta; MCSA,totalSample,immune-CD4_naive; MCSA,totalSample,immune-Tr1; MCSA,totalSample,immune-nTreg; [illegible]
BAIAP2L1	ACOM,control,male.vs.female; Homo_723,MCI,male.vs.female; Homo_714,MCI,male.vs.female; Homo_633,MCI,male.vs.female; ACOM,totalSample,age; Emory,totalSample,age; MCSA,totalSample,age; MCSA,control,age; ROSMAP,MCI,sample.age; Emory,totalSample,immune-Gamma_delta; Emory,totalSample,immune-iTreg; Emory,totalSample,immune-Exhausted; Homo_723,totalSample,immune-nTreg; Homo_714,totalSample,immune-nTreg; Homo_633,totalSample,immune-nTreg; MCSA,totalSample,immune-DC; MCSA,totalSample,immune-Monocyte; MCSA,totalSample,immune-Gamma_delta; MCSA,totalSample,immune-CD4_naive; MCSA,totalSample,immune-nTreg; MCSA,totalSample,immune-Th17; MCSA,totalSample,immune-Tfh; MCSA,totalSample,immune-Central_memory; MCSA,totalSample,immune-InfiltrationScore; SRP223445,totalSample,immune-InfiltrationScore
ZNF608	ACOM,control,male.vs.female; Homo_723,female,AD.vs.control; Homo_723,male,AD.vs.control; Homo_723,female,AD.vs.MCI; Homo_723,male,AD.vs.MCI; Homo_723,female,MCI.vs.control; Homo_723,male,MCI.vs.control; Homo_714,female,AD.vs.control; Homo_714,male,AD.vs.control; Homo_714,female,AD.vs.MCI; Homo_714,male,AD.vs.MCI; Homo_714,female,MCI.vs.control; Homo_714,male,MCI.vs.control; Homo_714,control,male.vs.female; Homo_633,female,AD.vs.control; Homo_633,female,AD.vs.MCI; ACOM,totalSample,age; Emory,control,age; MCSA,MCI,age; ROSMAP,AD,MMSE; Emory,totalSample,immune-Neutrophil; Emory,totalSample,immune-CD8_T; Emory,totalSample,immune-Gamma_delta; Emory,totalSample,immune-Th1; Emory,totalSample,immune-Exhausted; Homo_723,totalSample,immune-Th17; Homo_723,totalSample,immune-Exhausted; Homo_723,totalSample,immune-CD8_T; Homo_723,totalSample,immune-Cytotoxic; Homo_723,totalSample,immune-Gamma_delta; Homo_714,totalSample,immune-Exhausted; Homo_714,totalSample,immune-CD8_T; Homo_714,totalSample,immune-Th17; Homo_714,totalSample,immune-Gamma_delta; Homo_633,totalSample,immune-Gamma_delta; Homo_633,totalSample,immune-Exhausted; Homo_633,totalSample,immune-CD8_T; Homo_633,totalSample,immune-Neutrophil; MCSA,totalSample,immune-DC; MCSA,totalSample,immune-Neutrophil; MCSA,totalSample,immune-CD8_T; MCSA,totalSample,immune-Gamma_delta; MCSA,totalSample,immune-iTreg; MCSA,totalSample,immune-Th1; MCSA,totalSample,immune-Th17; MCSA,totalSample,immune-Cytotoxic; MCSA,totalSample,immune-Exhausted; MCSA,totalSample,immune-Effector_memory; ROSMAP,totalSample,immune-InfiltrationScore; SRP223445,totalSample,immune-CD4_naive;
SLC16A4	ACOM,control,male.vs.female; Homo_723,female,AD.vs.MCI; Homo_723,female,MCI.vs.control; Homo_723,MCI,male.vs.female; Homo_714,female,AD.vs.MCI; Homo_714,female,MCI.vs.control; Homo_714,MCI,male.vs.female; Homo_633,female,AD.vs.MCI; Homo_633,female,MCI.vs.control; Homo_633,control,male.vs.female; Homo_633,MCI,male.vs.female; ROSMAP,control,male.vs.female; Emory,MCI,age; ROSMAP,MCI,MMSE; Emory,totalSample,immune-Gamma_delta; Homo_723,totalSample,immune-Monocyte; Homo_723,totalSample,immune-iTreg; Homo_723,totalSample,immune-NKT; Homo_714,totalSample,immune-NKT; Homo_714,totalSample,immune-Th1; Homo_633,totalSample,immune-Monocyte; Homo_633,totalSample,immune-NKT; Homo_633,totalSample,immune-Th1; Homo_633,totalSample,immune-iTreg; MCSA,totalSample,immune-DC; MCSA,totalSample,immune-Bcell; MCSA,totalSample,immune-CD4_T; MCSA,totalSample,immune-CD8_T; MCSA,totalSample,immune-Gamma_delta; MCSA,totalSample,immune-CD4_naive; MCSA,totalSample,immune-Tr1; MCSA,totalSample,immune-nTreg; MCSA,totalSample,immune-iTreg; MCSA,totalSample,immune-Th17; MCSA,totalSample,immune-Tfh; MCSA,totalSample,immune-CD8_naive; MCSA,totalSample,immune-Cytotoxic; MCSA,totalSample,immune-Exhausted; MCSA,totalSample,immune-Central_memory; MCSA,totalSample,immune-Effector_memory
DHRSX	ACOM,control,male.vs.female; Homo_723,male,AD.vs.control; Homo_723,female,AD.vs.MCI; Homo_723,male,AD.vs.MCI; Homo_723,female,MCI.vs.control; Homo_723,control,male.vs.female; Homo_723,MCI,male.vs.female; Homo_714,male,AD.vs.control; Homo_714,female,AD.vs.MCI; Homo_714,male,AD.vs.MCI; Homo_714,female,MCI.vs.control; Homo_714,control,male.vs.female; Homo_714,MCI,male.vs.female; Homo_633,female,MCI.vs.control; Homo_633,control,male.vs.female; Homo_633,MCI,male.vs.female; ROSMAP,female,AD.vs.control; ROSMAP,male,AD.vs.control; Emory,totalSample,age; Emory,MCI,age; Emory,totalSample,immune-Neutrophil; Emory,totalSample,immune-CD8_T; Emory,totalSample,immune-Gamma_delta; Emory,totalSample,immune-Th17; Emory,totalSample,immune-Tfh; Emory,totalSample,immune-CD8_naive; Emory,totalSample,immune-Cytotoxic; Emory,totalSample,immune-Exhausted; Homo_723,totalSample,immune-DC; Homo_723,totalSample,immune-Th2; Homo_723,totalSample,immune-Monocyte; Homo_714,totalSample,immune-Th2; Homo_714,totalSample,immune-DC; Homo_633,totalSample,immune-Th2; Homo_633,totalSample,immune-Monocyte; MCSA,totalSample,immune-DC; MCSA,totalSample,immune-Monocyte; MCSA,totalSample,immune-Macrophage; MCSA,totalSample,immune-Gamma_delta; MCSA,totalSample,immune-iTreg;
VPS9D1	ACOM,control,male.vs.female; Homo_723,control,male.vs.female; Homo_723,MCI,male.vs.female; Homo_714,female,AD.vs.MCI; Homo_714,female,MCI.vs.control; Homo_714,control,male.vs.female; Homo_714,MCI,male.vs.female; Homo_633,control,male.vs.female; Homo_633,MCI,male.vs.female; MCSA,totalSample,age; MCSA,control,age; MCSA,MCI,age; ROSMAP,MCI,last.visit.age; ROSMAP,MCI,sample.age; ROSMAP,MCI,MMSE; Emory,totalSample,immune-Neutrophil; Emory,totalSample,immune-CD8_T; Emory,totalSample,immune-Gamma_delta; Emory,totalSample,immune-Tfh; Emory,totalSample,immune-CD8_naive; Emory,totalSample,immune-Exhausted; Emory,totalSample,immune-MAIT; Homo_723,totalSample,immune-DC; Homo_723,totalSample,immune-Cytotoxic; Homo_723,totalSample,immune-Monocyte; Homo_723,totalSample,immune-NKT; Homo_723,totalSample,immune-CD8_T; Homo_714,totalSample,immune-DC; Homo_714,totalSample,immune-Monocyte; Homo_714,totalSample,immune-Th2; Homo_633,totalSample,immune-DC; Homo_633,totalSample,immune-NKT; Homo_633,totalSample,immune-Monocyte; Homo_633,totalSample,immune-Th2; MCSA,totalSample,immune-DC; MCSA,totalSample,immune-Bcell; MCSA,totalSample,immune-Monocyte; MCSA,totalSample,immune-Macrophage; MCSA,totalSample,immune-CD8_T; MCSA,totalSample,immune-iTreg; MCSA,totalSample,immune-Th2; MCSA,totalSample,immune-Cytotoxic; MCSA,totalSample,immune-MAIT; MCSA,totalSample,immune-Effector_memory; MCSA,totalSample,immune-InfiltrationScore; ROSMAP,totalSample,immune-InfiltrationScore
EBF1	ACOM,control,male.vs.female; Homo_723,female,AD.vs.control; Homo_723,male,AD.vs.control; Homo_723,male,AD.vs.MCI; Homo_723,female,MCI.vs.control; Homo_723,MCI,male.vs.female; Homo_714,female,AD.vs.control; Homo_714,male,AD.vs.control; Homo_714,female,MCI.vs.control; Homo_714,MCI,male.vs.female; Homo_633,female,AD.vs.control; Homo_633,female,AD.vs.MCI; Homo_633,female,MCI.vs.control; Homo_633,male,MCI.vs.control; Homo_633,MCI,male.vs.female; ROSMAP,control,male.vs.female; Emory,totalSample,immune-B_cell; Emory,totalSample,immune-Macrophage; Emory,totalSample,immune-Neutrophil; Emory,totalSample,immune-CD4_T; Emory,totalSample,immune-Tr1; Emory,totalSample,immune-iTreg; Emory,totalSample,immune-Tfh; Emory,totalSample,immune-CD8_naive; Emory,totalSample,immune-InfiltrationScore; Homo_723,totalSample,immune-Monocyte; Homo_723,totalSample,immune-Th1; Homo_723,totalSample,immune-Neutrophil; Homo_723,totalSample,immune-DC; Homo_723,totalSample,immune-Central_memory; Homo_723,totalSample,immune-nTreg; Homo_723,totalSample,immune-Bcell; Homo_714,totalSample,immune-Th1; Homo_714,totalSample,immune-NKT; Homo_714,totalSample,immune-Monocyte; Homo_633,totalSample,immune-Th1; Homo_633,totalSample,immune-Monocyte; Homo_633,totalSample,immune-NKT; MCSA,totalSample,immune-Bcell; MCSA,totalSample,immune-Macrophage; MCSA,totalSample,immune-CD4_T; MCSA,totalSample,immune-CD4_naive; MCSA,totalSample,immune-Tr1; MCSA,totalSample,immune-nTreg; MCSA,totalSample,immune-iTreg; MCSA,totalSample,immune-Th2; MCSA,totalSample,immune-Th17; MCSA,totalSample,immune-Tfh; MCSA,totalSample,immune-CD8_naive;
KCMF1	ACOM,control,male.vs.female; Homo_723,female,AD.vs.control; Homo_723,male,AD.vs.control; Homo_723,female,MCI.vs.control; Homo_723,male,MCI.vs.control; Homo_723,AD,male.vs.female; Homo_723,MCI,male.vs.female; Homo_714,female,AD.vs.control; Homo_714,male,AD.vs.control; Homo_714,female,MCI.vs.control; Homo_714,male,MCI.vs.control; Homo_714,AD,male.vs.female; Homo_714,MCI,male.vs.female; Homo_633,male,MCI.vs.control; Homo_633,control,male.vs.female; Homo_633,MCI,male.vs.female; ROSMAP,female,AD.vs.control; ROSMAP,control,male.vs.female; MCSA,MCI,age; Emory,totalSample,immune-DC; Emory,totalSample,immune-Monocyte; Emory,totalSample,immune-Neutrophil; Emory,totalSample,immune-Gamma_delta; Emory,totalSample,immune-Tr1; Emory,totalSample,immune-iTreg; Emory,totalSample,immune-Exhausted; Homo_723,totalSample,immune-Th1; Homo_714,totalSample,immune-NKT; Homo_714,totalSample,immune-Th1; Homo_633,totalSample,immune-NKT; Homo_633,totalSample,immune-Th1; MCSA,totalSample,immune-DC; MCSA,totalSample,immune-Monocyte; MCSA,totalSample,immune-Neutrophil; MCSA,totalSample,immune-CD4_T; MCSA,totalSample,immune-CD8_T; MCSA,totalSample,immune-Gamma_delta; MCSA,totalSample,immune-CD4_naive; MCSA,totalSample,immune-Tr1; MCSA,totalSample,immune-nTreg; MCSA,totalSample,immune-iTreg; MCSA,totalSample,immune-Th2; MCSA,totalSample,immune-Th17; MCSA,totalSample,immune-Cytotoxic; MCSA,totalSample,immune-Exhausted; MCSA,totalSample,immune-Effector_memory
DAD1	ACOM,control,male.vs.female; Homo_723,male,AD.vs.control; Homo_723,female,MCI.vs.control; Homo_723,male,MCI.vs.control; Homo_723,AD,male.vs.female; Homo_723,MCI,male.vs.female; Homo_714,male,AD.vs.control; Homo_714,female,AD.vs.MCI; Homo_714,female,MCI.vs.control; Homo_714,male,MCI.vs.control; Homo_714,AD,male.vs.female; Homo_714,MCI,male.vs.female; Homo_633,male,MCI.vs.control; Homo_633,control,male.vs.female; Homo_633,MCI,male.vs.female; ROSMAP,MCI,last.visit.age; Emory,totalSample,immune-Macrophage; Emory,totalSample,immune-Neutrophil; Emory,totalSample,immune-CD4_T; Emory,totalSample,immune-Tr1; Emory,totalSample,immune-iTreg; Emory,totalSample,immune-Th1; Emory,totalSample,immune-Tfh; Emory,totalSample,immune-InfiltrationScore; Homo_723,totalSample,immune-Monocyte; Homo_723,totalSample,immune-Exhausted; Homo_633,totalSample,immune-Monocyte; MCSA,totalSample,immune-DC; MCSA,totalSample,immune-Macrophage; MCSA,totalSample,immune-NKT; MCSA,totalSample,immune-Th2; ROSMAP,totalSample,immune-iTreg; ROSMAP,totalSample,immune-Effector_memory; SRP223445,totalSample,immune-CD4_naive; SRP223445,totalSample,immune-Exhausted; SRP223445,totalSample,immune-InfiltrationScore
CDC42EP1	ACOM,control,male.vs.female; Emory,control,male.vs.female; ROSMAP,female,AD.vs.control; ROSMAP,female,AD.vs.MCI; SRP223445,female,AD.vs.control; SRP223445,male,AD.vs.control; ROSMAP,AD,last.visit.age; Homo_723,totalSample,immune-DC; Homo_723,totalSample,immune-Monocyte; Homo_714,totalSample,immune-Th2; Homo_633,totalSample,immune-Th2; Homo_633,totalSample,immune-Monocyte; MCSA,totalSample,immune-DC; SRP223445,totalSample,immune-Neutrophil; SRP223445,totalSample,immune-CD4_naive; SRP223445,totalSample,immune-InfiltrationScore; ROSMAP,male-excludeAge90+,survival
SMC1A	ACOM,control,male.vs.female; Emory,MCI,age; MCSA,totalSample,age; MCSA,control,age; ROSMAP,MCI,sample.age; Emory,totalSample,immune-Monocyte; Emory,totalSample,immune-CD4_T; Emory,totalSample,immune-NKT; Emory,totalSample,immune-Tr1; Emory,totalSample,immune-iTreg; Emory,totalSample,immune-Central_memory; Emory,totalSample,immune-InfiltrationScore; Homo_723,totalSample,immune-CD4_naive; Homo_723,totalSample,immune-iTreg; Homo_723,totalSample,immune-CD4_T; Homo_723,totalSample,immune-Tr1; Homo_723,totalSample,immune-InfiltrationScore; Homo_723,totalSample,immune-Tfh; Homo_723,totalSample,immune-nTreg; Homo_723,totalSample,immune-Th2; Homo_723,totalSample,immune-Central_memory; Homo_723,totalSample,immune-Effector_memory; Homo_723,totalSample,immune-NKT; Homo_723,totalSample,immune-Gamma_delta; Homo_723,totalSample,immune-Th17; Homo_723,totalSample,immune-Monocyte; Homo_714,totalSample,immune-CD4_naive; Homo_714,totalSample,immune-CD4_T; Homo_714,totalSample,immune-Tfh; Homo_714,totalSample,immune-Tr1; Homo_714,totalSample,immune-iTreg; Homo_714,totalSample,immune-InfiltrationScore; Homo_714,totalSample,immune-Effector_memory; Homo_714,totalSample,immune-nTreg; Homo_714,totalSample,immune-Th2; Homo_714,totalSample,immune-Central_memory; Homo_714,totalSample,immune-NKT; Homo_714,totalSample,immune-Th17; Homo_714,totalSample,immune-Gamma_delta; Homo_633,totalSample,immune-CD4_T; Homo_633,totalSample,immune-Tfh; Homo_633,totalSample,immune-InfiltrationScore; Homo_633,totalSample,immune-Tr1; Homo_633,totalSample,immune-CD4_naive; Homo_633,totalSample,immune-Effector_memory; Homo_633,totalSample,immune-iTreg; Homo_633,totalSample,immune-NKT; Homo_633,totalSample,immune-nTreg; Homo_633,totalSample,immune-Central_memory; Homo_633,totalSample,immune-Th2; Homo_633,totalSample,immune-Th17; Homo_633,totalSample,immune-Cytotoxic; Homo_633,totalSample,immune-Gamma_delta; MCSA,totalSample,immune-Monocyte; MCSA,totalSample,immune-CD4_T; MCSA,totalSample,immune-Gamma_delta; MCSA,totalSample,immune-CD4_naive; MCSA,totalSample,immune-Tr1; MCSA,totalSample,immune-nTreg; MCSA,totalSample,immune-iTreg; MCSA,totalSample,immune-Th17; MCSA,totalSample,immune-Tfh; MCSA,totalSample,immune-CD8_naive; MCSA,totalSample,immune-Cytotoxic; MCSA,totalSample,immune-Exhausted; MCSA,totalSample,immune-Central_memory; MCSA,totalSample,immune-Effector_memory; MCSA,totalSample,immune-InfiltrationScore; ROSMAP,totalSample,immune-Effector_memory; SRP223445,totalSample,immune-nTreg
MACROH2A1	ACOM,control,male.vs.female; Homo_723,control,male.vs.female; Homo_714,control,male.vs.female; Homo_714,MCI,male.vs.female; Homo_633,control,male.vs.female; Homo_633,MCI,male.vs.female; Emory,totalSample,age; Emory,MCI,age; MCSA,MCI,age; ROSMAP,MCI,MMSE; Emory,totalSample,immune-B_cell; Emory,totalSample,immune-Neutrophil; Emory,totalSample,immune-CD8_T; Emory,totalSample,immune-Gamma_delta; Emory,totalSample,immune-Th1; Emory,totalSample,immune-Tfh; Emory,totalSample,immune-CD8_naive; Emory,totalSample,immune-Cytotoxic; Emory,totalSample,immune-Exhausted; Homo_723,totalSample,immune-Exhausted; Homo_723,totalSample,immune-CD8_T; Homo_723,totalSample,immune-Bcell; Homo_723,totalSample,immune-Macrophage; Homo_723,totalSample,immune-InfiltrationScore; Homo_723,totalSample,immune-Cytotoxic; Homo_723,totalSample,immune-Neutrophil; Homo_714,totalSample,immune-Exhausted; Homo_714,totalSample,immune-CD8_T; Homo_714,totalSample,immune-Macrophage; Homo_714,totalSample,immune-Cytotoxic; Homo_633,totalSample,immune-CD8_T; Homo_633,totalSample,immune-Exhausted; Homo_633,totalSample,immune-Macrophage; Homo_633,totalSample,immune-Neutrophil; Homo_633,totalSample,immune-Cytotoxic; Homo_633,totalSample,immune-Gamma_delta; MCSA,totalSample,immune-Neutrophil; MCSA,totalSample,immune-CD4_T; MCSA,totalSample,immune-CD8_T; MCSA,totalSample,immune-Gamma_delta; MCSA,totalSample,immune-Tr1; MCSA,totalSample,immune-nTreg; MCSA,totalSample,immune-iTreg; MCSA,totalSample,immune-Th1; MCSA,totalSample,immune-Th17; MCSA,totalSample,immune-Cytotoxic; MCSA,totalSample,immune-Exhausted;
SNAP29	ACOM,control,male.vs.female; ROSMAP,MCI,last.visit.age; Emory,totalSample,immune-CD4_T; Emory,totalSample,immune-Gamma_delta; Emory,totalSample,immune-Tr1; Emory,totalSample,immune-iTreg; Emory,totalSample,immune-InfiltrationScore; Homo_723,totalSample,immune-InfiltrationScore; Homo_714,totalSample,immune-InfiltrationScore; Homo_633,totalSample,immune-InfiltrationScore; Homo_633,totalSample,immune-Bcell; MCSA,totalSample,immune-CD8_T; MCSA,totalSample,immune-iTreg; MCSA,totalSample,immune-Th1; MCSA,totalSample,immune-Th17; ROSMAP,totalSample,immune-Effector_memory
SUN3	ACOM,control,male.vs.female; Homo_723,female,AD.vs.control; Homo_723,male,MCI.vs.control; Homo_714,male,MCI.vs.control; Homo_633,male,MCI.vs.control; Emory,totalSample,immune-MAIT; Homo_723,totalSample,immune-Monocyte; Homo_723,totalSample,immune-Th2; Homo_723,totalSample,immune-Tfh; Homo_723,totalSample,immune-DC; Homo_714,totalSample,immune-Th2; Homo_714,totalSample,immune-Tfh; Homo_633,totalSample,immune-Th2; Homo_633,totalSample,immune-Tfh; MCSA,totalSample,immune-DC; MCSA,totalSample,immune-Macrophage; MCSA,totalSample,immune-CD4_T; MCSA,totalSample,immune-CD4_naive; MCSA,totalSample,immune-Tr1; MCSA,totalSample,immune-nTreg; MCSA,totalSample,immune-iTreg; MCSA,totalSample,immune-Tfh; MCSA,totalSample,immune-MAIT; MCSA,totalSample,immune-Central_memory; ROSMAP,female-excludeAge90+,survival; ROSMAP,male and female-excludeAge90+,survival; ROSMAP,male and female-includeAge90+,survival

CMTM8	ACOM,control,male.vs.female; Homo_723,male,MCI.vs.control; Homo_723,MCI,male.vs.female; Homo_714,male,MCI.vs.control; Homo_714,MCI,male.vs.female; Homo_633,female,AD.vs.control; Homo_633,female,AD.vs.MCI; Homo_633,male,MCI.vs.control; Homo_633,MCI,male.vs.female; ROSMAP,female,AD.vs.control; MCSA,totalSample,age; MCSA,control,age; MCSA,MCI,age; ROSMAP,AD,last.visit.age; ROSMAP,MCI,last.visit.age; ROSMAP,MCI,sample.age; Emory,totalSample,immune-CD4_T; Emory,totalSample,immune-Tr1; Emory,totalSample,immune-nTreg; Emory,totalSample,immune-iTreg; Emory,totalSample,immune-Th17; Emory,totalSample,immune-Tfh; Emory,totalSample,immune-CD8_naive; Emory,totalSample,immune-Cytotoxic; Emory,totalSample,immune-Central_memory; Homo_723,totalSample,immune-nTreg; Homo_723,totalSample,immune-CD4_naive; Homo_723,totalSample,immune-Central_memory; Homo_723,totalSample,immune-Tfh; Homo_723,totalSample,immune-iTreg; Homo_723,totalSample,immune-CD4_T; Homo_723,totalSample,immune-Tr1; Homo_714,totalSample,immune-nTreg; Homo_714,totalSample,immune-Tfh; Homo_714,totalSample,immune-CD4_naive; Homo_714,totalSample,immune-iTreg; Homo_714,totalSample,immune-CD4_T; Homo_714,totalSample,immune-Tr1; Homo_714,totalSample,immune-Central_memory; Homo_714,totalSample,immune-Th1; Homo_633,totalSample,immune-nTreg; Homo_633,totalSample,immune-iTreg; Homo_633,totalSample,immune-Tfh; Homo_633,totalSample,immune-CD4_naive; Homo_633,totalSample,immune-Tr1; Homo_633,totalSample,immune-CD4_T; Homo_633,totalSample,immune-Central_memory; Homo_633,totalSample,immune-Th1; MCSA,totalSample,immune-CD4_T; MCSA,totalSample,immune-CD4_naive; MCSA,totalSample,immune-Tr1; MCSA,totalSample,immune-nTreg; MCSA,totalSample,immune-iTreg; MCSA,totalSample,immune-Th17; MCSA,totalSample,immune-Tfh; MCSA,totalSample,immune-Central_memory; ROSMAP,totalSample,immune-Th17; ROSMAP,totalSample,immune-InfiltrationScore
ZFHX2	ACOM,control,male.vs.female; Homo_723,female,AD.vs.control; Homo_723,male,AD.vs.control; Homo_723,female,AD.vs.MCI; Homo_723,male,AD.vs.MCI; Homo_714,female,AD.vs.control; Homo_714,female,AD.vs.MCI; Homo_633,female,MCI.vs.control; ROSMAP,male,AD.vs.control; ROSMAP,female,MCI.vs.control; ROSMAP,AD,male.vs.female; ROSMAP,control,male.vs.female; ROSMAP,AD,last.visit.age; ROSMAP,MCI,sample.age; Emory,totalSample,immune-Gamma_delta; Emory,totalSample,immune-Th17; Homo_723,totalSample,immune-Monocyte; Homo_723,totalSample,immune-DC; Homo_714,totalSample,immune-Th2; Homo_633,totalSample,immune-Monocyte; ROSMAP,totalSample,immune- [illegible]
OR2T12	ACOM,control,male.vs.female; Homo_714,female,MCI.vs.control; Homo_633,female,MCI.vs.control; ROSMAP,totalSample,immune-InfiltrationScore
BCAM	ACOM,control,male.vs.female; Homo_723,female,AD.vs.control; Homo_723,female,AD.vs.MCI; Homo_723,male,AD.vs.MCI; Homo_714,female,AD.vs.control; Homo_714,female,AD.vs.MCI; Homo_633,female,MCI.vs.control; ROSMAP,female,AD.vs.control; Emory,MCI,age; Emory,totalSample,immune-Tr1; Emory,totalSample,immune-iTreg; Homo_723,totalSample,immune-DC; Homo_723,totalSample,immune-Th2; Homo_723,totalSample,immune-Bcell; Homo_723,totalSample,immune-Neutrophil; Homo_723,totalSample,immune-Monocyte; Homo_723,totalSample,immune-iTreg; Homo_714,totalSample,immune-Th2; Homo_714,totalSample,immune-DC; Homo_633,totalSample,immune-Th2; Homo_633,totalSample,immune-Monocyte; SRP223445,totalSample,immune-Neutrophil; SRP223445,totalSample,immune-CD4_naive; SRP223445,totalSample,immune-InfiltrationScore
KPNA1	ACOM,control,male.vs.female; Homo_723,MCI,male.vs.female; Homo_714,MCI,male.vs.female; Homo_633,MCI,male.vs.female; MCSA,MCI,age; Emory,totalSample,immune-Neutrophil; Emory,totalSample,immune-Gamma_delta; Emory,totalSample,immune-Tr1; Emory,totalSample,immune-iTreg; Emory,totalSample,immune-Th1; Emory,totalSample,immune-Th17; Emory,totalSample,immune-CD8_naive; Emory,totalSample,immune-Cytotoxic; Emory,totalSample,immune-Exhausted; Homo_723,totalSample,immune-Cytotoxic; Homo_723,totalSample,immune-Tr1; Homo_723,totalSample,immune-Th2; Homo_723,totalSample,immune-iTreg; Homo_723,totalSample,immune-CD4_naive; Homo_723,totalSample,immune-CD4_T; Homo_723,totalSample,immune-DC; Homo_723,totalSample,immune-Tfh; Homo_723,totalSample,immune-NKT; Homo_723,totalSample,immune-CD8_T; Homo_723,totalSample,immune-Exhausted; Homo_723,totalSample,immune-Gamma_delta; Homo_723,totalSample,immune-Th17; Homo_723,totalSample,immune-Neutrophil; Homo_714,totalSample,immune-DC; Homo_714,totalSample,immune-Th2; Homo_714,totalSample,immune-iTreg; Homo_714,totalSample,immune-CD4_naive; Homo_714,totalSample,immune-CD4_T; Homo_714,totalSample,immune-Tr1; Homo_714,totalSample,immune-Cytotoxic; Homo_714,totalSample,immune-NKT; Homo_714,totalSample,immune-Tfh; Homo_714,totalSample,immune-Exhausted; Homo_714,totalSample,immune-CD8_T; Homo_714,totalSample,immune-Th17; Homo_714,totalSample,immune-Th1; Homo_633,totalSample,immune-DC; Homo_633,totalSample,immune-NKT; Homo_633,totalSample,immune-CD4_T; Homo_633,totalSample,immune-Th2; Homo_633,totalSample,immune-CD4_naive; Homo_633,totalSample,immune-Cytotoxic; Homo_633,totalSample,immune-iTreg; Homo_633,totalSample,immune-Tr1; Homo_633,totalSample,immune-Tfh; Homo_633,totalSample,immune-Th17; Homo_633,totalSample,immune-Effector_memory; Homo_633,totalSample,immune-CD8_T; Homo_633,totalSample,immune-Gamma_delta; Homo_633,totalSample,immune-Exhausted; Homo_633,totalSample,immune-Neutrophil; Homo_633,totalSample,immune-Central_memory; Homo_633,totalSample,immune-Th1; MCSA,totalSample,immune-DC; MCSA,totalSample,immune-Macrophage; MCSA,totalSample,immune-Neutrophil; MCSA,totalSample,immune-CD4_T; MCSA,totalSample,immune-CD8_T; MCSA,totalSample,immune-Gamma_delta; MCSA,totalSample,immune-CD4_naive; MCSA,totalSample,immune-Tr1; MCSA,totalSample,immune-nTreg; MCSA,totalSample,immune-iTreg; MCSA,totalSample,immune-Th1; MCSA,totalSample,immune-Th17; MCSA,totalSample,immune-Tfh; MCSA,totalSample,immune-CD8_naive; MCSA,totalSample,immune-Cytotoxic; MCSA,totalSample,immune-Exhausted; MCSA,totalSample,immune-Central_memory; [illegible]
SCG3	ACOM,control,male.vs.female; Homo_723,female,AD.vs.control; Homo_723,male,AD.vs.control; Homo_723,female,AD.vs.MCI; Homo_723,male,AD.vs.MCI; Homo_723,female,MCI.vs.control; Homo_714,female,AD.vs.control; Homo_714,male,AD.vs.control; Homo_714,female,AD.vs.MCI; Homo_714,male,AD.vs.MCI; ROSMAP,female,AD.vs.control; ROSMAP,MCI,sample.age; Emory,totalSample,immune-MAIT; Homo_723,totalSample,immune-Th2; Homo_723,totalSample,immune-Exhausted; Homo_714,totalSample,immune-Th2; Homo_633,totalSample,immune-Th2; Homo_633,totalSample,immune-Monocyte; MCSA,totalSample,immune-Tr1; MCSA,totalSample,immune-iTreg; ROSMAP,totalSample,immune- [illegible]
SOBP	ACOM,control,male.vs.female; Homo_723,female,AD.vs.control; Homo_723,male,AD.vs.control; Homo_723,female,AD.vs.MCI; Homo_723,male,AD.vs.MCI; Homo_723,female,MCI.vs.control; Homo_723,male,MCI.vs.control; Homo_723,MCI,male.vs.female; Homo_714,female,AD.vs.control; Homo_714,male,AD.vs.control; Homo_714,female,AD.vs.MCI; Homo_714,male,AD.vs.MCI; Homo_714,MCI,male.vs.female; Homo_633,female,AD.vs.control; Homo_633,female,AD.vs.MCI; Homo_633,male,MCI.vs.control; Homo_633,MCI,male.vs.female; SRP223445,female,AD.vs.control; Emory,totalSample,immune-B_cell; Emory,totalSample,immune-Macrophage; Homo_723,totalSample,immune-Neutrophil; Homo_723,totalSample,immune-Th1; Homo_723,totalSample,immune-nTreg; Homo_714,totalSample,immune-Neutrophil; Homo_714,totalSample,immune-Th1; MCSA,totalSample,immune-Bcell; MCSA,totalSample,immune-Macrophage; MCSA,totalSample,immune-Neutrophil; MCSA,totalSample,immune-CD4_T; MCSA,totalSample,immune-CD4_naive; MCSA,totalSample,immune-Tr1; MCSA,totalSample,immune-nTreg; MCSA,totalSample,immune-iTreg; MCSA,totalSample,immune-Tfh; MCSA,totalSample,immune-CD8_naive; MCSA,totalSample,immune-Central_memory; SRP223445,totalSample,immune-CD4_naive; SRP223445,totalSample,immune-InfiltrationScore; ROSMAP,male and female-excludeAge90+,survival
PTCHD1	ACOM,control,male.vs.female; Homo_723,female,AD.vs.control; Homo_723,female,AD.vs.MCI; Homo_723,MCI,male.vs.female; Homo_714,female,AD.vs.control; Homo_714,female,MCI.vs.control; Homo_714,MCI,male.vs.female; Homo_633,female,MCI.vs.control; Homo_633,MCI,male.vs.female; ROSMAP,control,male.vs.female; Emory,totalSample,immune-CD8_T; Homo_723,totalSample,immune-Th17; MCSA,totalSample,immune-Th17
GRAPL	ACOM,control,male.vs.female; Homo_723,female,AD.vs.control; Homo_723,male,AD.vs.control; Homo_723,female,AD.vs.MCI; Homo_723,male,MCI.vs.control; Homo_723,control,male.vs.female; Homo_723,MCI,male.vs.female; Homo_714,female,AD.vs.control; Homo_714,male,AD.vs.control; Homo_714,female,AD.vs.MCI; Homo_714,male,MCI.vs.control; Homo_714,control,male.vs.female; Homo_714,MCI,male.vs.female; Homo_633,female,AD.vs.control; Homo_633,female,AD.vs.MCI; Homo_633,male,MCI.vs.control; Homo_633,control,male.vs.female; Homo_633,MCI,male.vs.female; ROSMAP,female,AD.vs.control; ROSMAP,female,MCI.vs.control; ROSMAP,control,male.vs.female; MCSA,totalSample,age; MCSA,control,age; Emory,totalSample,immune-B_cell; Emory,totalSample,immune-Monocyte; Emory,totalSample,immune-Macrophage; Emory,totalSample,immune-Neutrophil; Emory,totalSample,immune-CD4_T; Emory,totalSample,immune-CD8_T; Emory,totalSample,immune-CD4_naive; Emory,totalSample,immune-Tr1; Emory,totalSample,immune-nTreg; Emory,totalSample,immune-iTreg; Emory,totalSample,immune-Th1; Emory,totalSample,immune-Tfh; Emory,totalSample,immune-CD8_naive; Emory,totalSample,immune-Central_memory; Emory,totalSample,immune-InfiltrationScore; Homo_723,totalSample,immune-nTreg; Homo_723,totalSample,immune-Central_memory; Homo_723,totalSample,immune-CD4_naive; Homo_723,totalSample,immune-Monocyte; Homo_723,totalSample,immune-CD4_T; Homo_723,totalSample,immune-Tfh; Homo_723,totalSample,immune-iTreg; Homo_723,totalSample,immune-Neutrophil; Homo_723,totalSample,immune-Tr1; Homo_723,totalSample,immune-CD8_T; Homo_723,totalSample,immune-Th1; Homo_723,totalSample,immune-Th2; Homo_714,totalSample,immune-nTreg; Homo_714,totalSample,immune-CD4_naive; Homo_714,totalSample,immune-Monocyte; Homo_714,totalSample,immune-Central_memory; Homo_714,totalSample,immune-Th1; Homo_714,totalSample,immune-CD4_T; Homo_714,totalSample,immune-Tfh; Homo_714,totalSample,immune-iTreg; Homo_714,totalSample,immune-Neutrophil; Homo_714,totalSample,immune-Tr1; Homo_714,totalSample,immune-Macrophage; Homo_633,totalSample,immune-Monocyte; Homo_633,totalSample,immune-nTreg; Homo_633,totalSample,immune-Tfh; Homo_633,totalSample,immune-CD4_naive; Homo_633,totalSample,immune-CD4_T; Homo_633,totalSample,immune-Central_memory; Homo_633,totalSample,immune-iTreg; Homo_633,totalSample,immune-Tr1; Homo_633,totalSample,immune-Th1; Homo_633,totalSample,immune-Neutrophil; Homo_633,totalSample,immune-Macrophage; MCSA,totalSample,immune-Bcell; MCSA,totalSample,immune-Monocyte; MCSA,totalSample,immune-Macrophage; MCSA,totalSample,immune-Neutrophil; MCSA,totalSample,immune-CD4_T; MCSA,totalSample,immune-CD8_T; MCSA,totalSample,immune-Gamma_delta; MCSA,totalSample,immune-CD4_naive; MCSA,totalSample,immune-Tr1; MCSA,totalSample,immune-nTreg; MCSA,totalSample,immune-iTreg; MCSA,totalSample,immune-Th17; MCSA,totalSample,immune-Tfh; MCSA,totalSample,immune-CD8_naive; [illegible]
HOXB7	ACOM,control,male.vs.female; Homo_633,female,MCI.vs.control; ROSMAP,AD,sample.age; Emory,totalSample,immune-Effector_memory; Homo_723,totalSample,immune-Monocyte; Homo_723,totalSample,immune-Th2; Homo_714,totalSample,immune-Th2; Homo_633,totalSample,immune-Monocyte; Homo_633,totalSample,immune-Th2; SRP223445,totalSample,immune-Neutrophil; SRP223445,totalSample,immune-CD4_naive; SRP223445,totalSample,immune-InfiltrationScore; ROSMAP,male-includeAge90+,survival
CORO2B	ACOM,control,male.vs.female; Homo_723,female,AD.vs.control; Homo_723,male,AD.vs.control; Homo_723,female,AD.vs.MCI; Homo_723,male,AD.vs.MCI; Homo_633,female,AD.vs.MCI; Homo_633,female,MCI.vs.control; ROSMAP,female,AD.vs.control; ROSMAP,female,MCI.vs.control; MCSA,MCI,age; Emory,totalSample,immune-B_cell; Emory,totalSample,immune-Macrophage; Emory,totalSample,immune-CD4_T; Emory,totalSample,immune-Tr1; Emory,totalSample,immune-CD8_naive; Homo_723,totalSample,immune-Monocyte; Homo_723,totalSample,immune-DC; Homo_723,totalSample,immune-Neutrophil; Homo_723,totalSample,immune-Th1; Homo_714,totalSample,immune-Monocyte; Homo_714,totalSample,immune-Th2; Homo_714,totalSample,immune-Th1; Homo_633,totalSample,immune-Monocyte; Homo_633,totalSample,immune-Th2; Homo_633,totalSample,immune-Th1; SRP223445,totalSample,immune-Neutrophil; SRP223445,totalSample,immune-InfiltrationScore
WNK3	ACOM,control,male.vs.female; Homo_723,female,AD.vs.control; Homo_723,male,AD.vs.control; Homo_723,female,AD.vs.MCI; Homo_723,male,AD.vs.MCI; Homo_723,male,MCI.vs.control; Homo_723,MCI,male.vs.female; Homo_714,female,AD.vs.control; Homo_714,male,AD.vs.control; Homo_714,female,AD.vs.MCI; Homo_714,male,AD.vs.MCI; Homo_714,MCI,male.vs.female; Homo_633,female,AD.vs.control; Homo_633,female,AD.vs.MCI; Homo_633,female,MCI.vs.control; Homo_633,MCI,male.vs.female; ROSMAP,control,male.vs.female; MCSA,MCI,age; Emory,totalSample,immune-CD4_naive; Homo_723,totalSample,immune-CD4_naive; Homo_723,totalSample,immune-CD4_T; Homo_723,totalSample,immune-Central_memory; Homo_723,totalSample,immune-Tr1; Homo_723,totalSample,immune-Tfh; Homo_723,totalSample,immune-iTreg; Homo_723,totalSample,immune-Th1; Homo_723,totalSample,immune-nTreg; Homo_714,totalSample,immune-CD4_naive; Homo_714,totalSample,immune-Tfh; Homo_714,totalSample,immune-Tr1; Homo_714,totalSample,immune-CD4_T; Homo_714,totalSample,immune-iTreg; Homo_714,totalSample,immune-Th1; Homo_714,totalSample,immune-NKT; Homo_714,totalSample,immune-nTreg; Homo_714,totalSample,immune-Central_memory; Homo_633,totalSample,immune-Tfh; Homo_633,totalSample,immune-CD4_T; Homo_633,totalSample,immune-CD4_naive; Homo_633,totalSample,immune-Tr1; Homo_633,totalSample,immune-iTreg; Homo_633,totalSample,immune-Central_memory; Homo_633,totalSample,immune-NKT; Homo_633,totalSample,immune-nTreg; Homo_633,totalSample,immune-Th1; Homo_633,totalSample,immune-InfiltrationScore; MCSA,totalSample,immune-DC; MCSA,totalSample,immune-Macrophage; MCSA,totalSample,immune-CD4_T; MCSA,totalSample,immune-CD4_naive; MCSA,totalSample,immune-Tr1; MCSA,totalSample,immune-nTreg; MCSA,totalSample,immune-iTreg; MCSA,totalSample,immune-Th17; MCSA,totalSample,immune-Tfh; MCSA,totalSample,immune-CD8_naive; MCSA,totalSample,immune-Cytotoxic; MCSA,totalSample,immune-Exhausted; [illegible]
AGER	ACOM,control,male.vs.female; Homo_723,male,MCI.vs.control; Homo_723,MCI,male.vs.female; Homo_714,male,MCI.vs.control; Homo_714,MCI,male.vs.female; Homo_633,male,MCI.vs.control; MCSA,totalSample,age; MCSA,control,age; MCSA,MCI,age; Emory,totalSample,immune-Neutrophil; Emory,totalSample,immune-Th17; Homo_723,totalSample,immune-NKT; Homo_714,totalSample,immune-NKT; Homo_633,totalSample,immune-NKT; Homo_633,totalSample,immune-DC; Homo_633,totalSample,immune-Th1; MCSA,totalSample,immune-DC; MCSA,totalSample,immune-Monocyte; MCSA,totalSample,immune-Macrophage; MCSA,totalSample,immune-CD8_T; MCSA,totalSample,immune-Gamma_delta; MCSA,totalSample,immune-Th2; MCSA,totalSample,immune-Th17; MCSA,totalSample,immune-Cytotoxic; MCSA,totalSample,immune-MAIT; MCSA,totalSample,immune-Effector_memory; MCSA,totalSample,immune-InfiltrationScore
CELSR1	ACOM,control,male.vs.female; Homo_723,female,AD.vs.control; Homo_723,male,AD.vs.control; Homo_723,female,AD.vs.MCI; Homo_723,male,AD.vs.MCI; Homo_723,MCI,male.vs.female; Homo_714,female,AD.vs.control; Homo_714,male,AD.vs.control; Homo_714,female,AD.vs.MCI; Homo_714,MCI,male.vs.female; Homo_633,female,AD.vs.control; Homo_633,female,AD.vs.MCI; Homo_633,female,MCI.vs.control; Homo_633,MCI,male.vs.female; ROSMAP,female,AD.vs.control; MCSA,totalSample,age; MCSA,control,age; Emory,totalSample,immune-B_cell; Emory,totalSample,immune-Monocyte; Emory,totalSample,immune-Macrophage; Emory,totalSample,immune-Neutrophil; Emory,totalSample,immune-CD4_T; Emory,totalSample,immune-Tr1; Emory,totalSample,immune-iTreg; Emory,totalSample,immune-Tfh; Emory,totalSample,immune-CD8_naive; Emory,totalSample,immune-InfiltrationScore; Homo_723,totalSample,immune-Monocyte; Homo_723,totalSample,immune-nTreg; Homo_723,totalSample,immune-Central_memory; Homo_723,totalSample,immune-DC; Homo_723,totalSample,immune-Neutrophil; Homo_723,totalSample,immune-Th1; Homo_723,totalSample,immune-CD4_naive; Homo_723,totalSample,immune-CD4_T; Homo_714,totalSample,immune-nTreg; Homo_714,totalSample,immune-Th1; Homo_714,totalSample,immune-Monocyte; Homo_714,totalSample,immune-CD4_naive; Homo_714,totalSample,immune-NKT; Homo_714,totalSample,immune-Neutrophil; Homo_714,totalSample,immune-InfiltrationScore; Homo_633,totalSample,immune-Monocyte; Homo_633,totalSample,immune-nTreg; Homo_633,totalSample,immune-Th1; Homo_633,totalSample,immune-NKT; Homo_633,totalSample,immune-Tr1; Homo_633,totalSample,immune-iTreg; Homo_633,totalSample,immune-CD4_naive; Homo_633,totalSample,immune-Tfh; Homo_633,totalSample,immune-CD4_T; Homo_633,totalSample,immune-InfiltrationScore; Homo_633,totalSample,immune-Central_memory; MCSA,totalSample,immune-Bcell; MCSA,totalSample,immune-Monocyte; MCSA,totalSample,immune-Macrophage; MCSA,totalSample,immune-Neutrophil; MCSA,totalSample,immune-CD4_T; MCSA,totalSample,immune-Gamma_delta; MCSA,totalSample,immune-CD4_naive; MCSA,totalSample,immune-Tr1; MCSA,totalSample,immune-nTreg; MCSA,totalSample,immune-iTreg; MCSA,totalSample,immune-Th17; MCSA,totalSample,immune-Tfh; MCSA,totalSample,immune-CD8_naive; MCSA,totalSample,immune- [illegible]
DAP	ACOM,control,male.vs.female; Homo_723,female,AD.vs.control; Homo_723,male,AD.vs.control; Homo_723,female,AD.vs.MCI; Homo_723,male,AD.vs.MCI; Homo_723,control,male.vs.female; Homo_723,MCI,male.vs.female; Homo_714,female,AD.vs.control; Homo_714,male,AD.vs.control; Homo_714,female,AD.vs.MCI; Homo_714,male,AD.vs.MCI; Homo_714,male,MCI.vs.control; Homo_714,AD,male.vs.female; Homo_714,control,male.vs.female; Homo_714,MCI,male.vs.female; Homo_633,male,MCI.vs.control; Homo_633,control,male.vs.female; Homo_633,MCI,male.vs.female; MCSA,totalSample,age; MCSA,MCI,age; Emory,totalSample,immune-Tfh; Homo_723,totalSample,immune-DC; Homo_723,totalSample,immune-Tr1; Homo_723,totalSample,immune-iTreg; Homo_723,totalSample,immune-Tfh; Homo_723,totalSample,immune-CD4_T; Homo_723,totalSample,immune-Cytotoxic; Homo_723,totalSample,immune-Th2; Homo_723,totalSample,immune-CD4_naive; Homo_714,totalSample,immune-DC; Homo_714,totalSample,immune-Tfh; Homo_714,totalSample,immune-iTreg; Homo_714,totalSample,immune-CD4_T; Homo_714,totalSample,immune-Th2; Homo_714,totalSample,immune-Tr1; Homo_714,totalSample,immune-CD4_naive; Homo_633,totalSample,immune-DC; Homo_633,totalSample,immune-CD4_T; Homo_633,totalSample,immune-Tfh; Homo_633,totalSample,immune-Tr1; Homo_633,totalSample,immune-CD4_naive; Homo_633,totalSample,immune-NKT; Homo_633,totalSample,immune-Th2; Homo_633,totalSample,immune-iTreg; Homo_633,totalSample,immune-Central_memory; Homo_633,totalSample,immune-Effector_memory; MCSA,totalSample,immune-DC; MCSA,totalSample,immune-Monocyte; MCSA,totalSample,immune-Macrophage; MCSA,totalSample,immune-CD4_T; MCSA,totalSample,immune-Tr1; MCSA,totalSample,immune-nTreg; MCSA,totalSample,immune-iTreg; MCSA,totalSample,immune-Th2; MCSA,totalSample,immune-CD8_naive; MCSA,totalSample,immune-Cytotoxic; MCSA,totalSample,immune-Exhausted; MCSA,totalSample,immune-MAIT; MCSA,totalSample,immune- [illegible]
IFT20	ACOM,control,male.vs.female; Homo_723,MCI,male.vs.female; Homo_714,MCI,male.vs.female; Homo_633,MCI,male.vs.female; Emory,MCI,age; MCSA,totalSample,age; MCSA,control,age; MCSA,MCI,age; ROSMAP,MCI,last.visit.age; Emory,totalSample,immune-DC; Emory,totalSample,immune-Monocyte; Emory,totalSample,immune-CD4_naive; Emory,totalSample,immune-nTreg; Emory,totalSample,immune-Cytotoxic; Emory,totalSample,immune-Effector_memory; Homo_723,totalSample,immune-Monocyte; Homo_723,totalSample,immune-Th17; Homo_723,totalSample,immune-Gamma_delta; Homo_714,totalSample,immune-Monocyte; Homo_714,totalSample,immune-Th17; Homo_714,totalSample,immune-nTreg; Homo_714,totalSample,immune-Gamma_delta; Homo_714,totalSample,immune-CD4_T; Homo_714,totalSample,immune-Th2; Homo_633,totalSample,immune-Monocyte; Homo_633,totalSample,immune-nTreg; Homo_633,totalSample,immune-Th17; Homo_633,totalSample,immune-Cytotoxic; Homo_633,totalSample,immune-iTreg; Homo_633,totalSample,immune-CD4_T; Homo_633,totalSample,immune-Th2; MCSA,totalSample,immune-DC; MCSA,totalSample,immune-Monocyte; MCSA,totalSample,immune-CD4_T; MCSA,totalSample,immune-Gamma_delta; MCSA,totalSample,immune-CD4_naive; MCSA,totalSample,immune-Tr1; MCSA,totalSample,immune-nTreg; MCSA,totalSample,immune-Th2; MCSA,totalSample,immune-Th17; MCSA,totalSample,immune-Tfh; MCSA,totalSample,immune-Central_memory; MCSA,totalSample,immune-Effector_memory; MCSA,totalSample,immune-InfiltrationScore; ROSMAP,totalSample,immune-Effector_memory; SRP223445,totalSample,immune-NK; SRP223445,totalSample,immune-CD4_naive; SRP223445,totalSample,immune-Exhausted;
CCDC17	ACOM,control,male.vs.female; Homo_723,male,AD.vs.control; Homo_723,male,AD.vs.MCI; Homo_723,MCI,male.vs.female; Homo_714,male,AD.vs.control; Homo_714,male,MCI.vs.control; Homo_714,MCI,male.vs.female; MCSA,totalSample,age; MCSA,control,age; MCSA,MCI,age; ROSMAP,AD,last.visit.age; ROSMAP,MCI,last.visit.age; Emory,totalSample,immune-Neutrophil; Emory,totalSample,immune-CD8_T; Emory,totalSample,immune-Gamma_delta; Emory,totalSample,immune-Th17; Emory,totalSample,immune-Exhausted; Emory,totalSample,immune-MAIT; Homo_723,totalSample,immune-Neutrophil; Homo_723,totalSample,immune-Th1; Homo_714,totalSample,immune-Neutrophil; Homo_633,totalSample,immune-Neutrophil; Homo_633,totalSample,immune-Th17; Homo_633,totalSample,immune-Exhausted; MCSA,totalSample,immune-DC; MCSA,totalSample,immune-Monocyte; MCSA,totalSample,immune-Macrophage; MCSA,totalSample,immune-Gamma_delta; MCSA,totalSample,immune-Th2; MCSA,totalSample,immune-Th17; MCSA,totalSample,immune-MAIT; MCSA,totalSample,immune-InfiltrationScore; ROSMAP,totalSample,immune-Effector_memory; ROSMAP,male-includeAge90+,survival
RGCC	ACOM,control,male.vs.female; Homo_723,male,AD.vs.control; Homo_723,male,AD.vs.MCI; Homo_723,male,MCI.vs.control; Homo_723,AD,male.vs.female; Homo_723,control,male.vs.female; Homo_714,male,AD.vs.control; Homo_714,female,AD.vs.MCI; Homo_714,male,AD.vs.MCI; Homo_714,female,MCI.vs.control; Homo_714,male,MCI.vs.control; Homo_714,AD,male.vs.female; Homo_714,control,male.vs.female; Homo_714,MCI,male.vs.female; Homo_633,female,AD.vs.control; Homo_633,female,AD.vs.MCI; Homo_633,female,MCI.vs.control; Homo_633,male,MCI.vs.control; Homo_633,control,male.vs.female; ROSMAP,female,AD.vs.control; ROSMAP,female,MCI.vs.control; Emory,totalSample,age; Emory,control,age; Emory,MCI,age; MCSA,totalSample,age; MCSA,control,age; Emory,totalSample,immune-Macrophage; Emory,totalSample,immune-Neutrophil; Emory,totalSample,immune-CD4_T; Emory,totalSample,immune-CD8_T; Emory,totalSample,immune-Tr1; Emory,totalSample,immune-iTreg; Emory,totalSample,immune-Tfh; Emory,totalSample,immune-CD8_naive; Emory,totalSample,immune-MAIT; Emory,totalSample,immune-Central_memory; Homo_723,totalSample,immune-Central_memory; Homo_723,totalSample,immune-Th1; Homo_723,totalSample,immune-CD4_naive; Homo_723,totalSample,immune-nTreg; Homo_723,totalSample,immune-CD4_T; Homo_723,totalSample,immune-Neutrophil; Homo_723,totalSample,immune-Monocyte; Homo_723,totalSample,immune-Tfh; Homo_723,totalSample,immune-iTreg; Homo_723,totalSample,immune-Tr1; Homo_714,totalSample,immune-Th1; Homo_714,totalSample,immune-CD4_naive; Homo_714,totalSample,immune-nTreg; Homo_714,totalSample,immune-iTreg; Homo_714,totalSample,immune-Tfh; Homo_714,totalSample,immune-Central_memory; Homo_714,totalSample,immune-CD4_T; Homo_714,totalSample,immune-Neutrophil; Homo_633,totalSample,immune-Th1; Homo_633,totalSample,immune-Monocyte; Homo_633,totalSample,immune-Tfh; Homo_633,totalSample,immune-CD4_naive; Homo_633,totalSample,immune-iTreg; Homo_633,totalSample,immune-nTreg; Homo_633,totalSample,immune-CD4_T; MCSA,totalSample,immune-DC; MCSA,totalSample,immune-Macrophage; MCSA,totalSample,immune-Neutrophil; MCSA,totalSample,immune-CD4_T; MCSA,totalSample,immune-CD4_naive; MCSA,totalSample,immune-Tr1; MCSA,totalSample,immune-nTreg; MCSA,totalSample,immune-iTreg; MCSA,totalSample,immune-Th17; MCSA,totalSample,immune-Tfh; MCSA,totalSample,immune-CD8_naive; MCSA,totalSample,immune-MAIT; MCSA,totalSample,immune-Central_memory; MCSA,totalSample,immune-Effector_memory; ROSMAP,totalSample,immune-Macrophage; ROSMAP,totalSample,immune-Effector_memory
TRPC5	ACOM,control,male.vs.female; Homo_723,female,AD.vs.control; Homo_723,male,AD.vs.control; Homo_723,female,MCI.vs.control; Homo_723,control,male.vs.female; Homo_723,MCI,male.vs.female; Homo_714,female,MCI.vs.control; Homo_714,control,male.vs.female; Homo_714,MCI,male.vs.female; Homo_633,female,AD.vs.MCI; Homo_633,female,MCI.vs.control; Homo_633,MCI,male.vs.female; ROSMAP,MCI,male.vs.female; ROSMAP,MCI,sample.age; Homo_633,totalSample,immune-InfiltrationScore; MCSA,totalSample,immune-Macrophage; MCSA,totalSample,immune-CD4_T; MCSA,totalSample,immune-CD4_naive; MCSA,totalSample,immune-Tr1; MCSA,totalSample,immune-nTreg; MCSA,totalSample,immune-iTreg; MCSA,totalSample,immune-Tfh; MCSA,totalSample,immune-CD8_naive; MCSA,totalSample,immune-Central_memory; ROSMAP,totalSample,immune-InfiltrationScore

KCTD20	ACOM,control,male.vs.female; Homo_723,female,AD.vs.control; Homo_723,female,AD.vs.MCI; Homo_723,MCI,male.vs.female; Homo_714,female,AD.vs.control; Homo_714,female,AD.vs.MCI; Homo_714,MCI,male.vs.female; Homo_633,MCI,male.vs.female; Emory,MCI,age; MCSA,MCI,age; Emory,totalSample,immune-Monocyte; Emory,totalSample,immune-Gamma_delta; Emory,totalSample,immune-Tr1; Emory,totalSample,immune-iTreg; Emory,totalSample,immune-Th1; Emory,totalSample,immune-Tfh; Emory,totalSample,immune-CD8_naive; Homo_723,totalSample,immune-NKT; Homo_723,totalSample,immune-Cytotoxic; Homo_723,totalSample,immune-Tr1; Homo_723,totalSample,immune-Gamma_delta; Homo_723,totalSample,immune-iTreg; Homo_723,totalSample,immune-CD4_naive; Homo_723,totalSample,immune-Exhausted; Homo_723,totalSample,immune-CD8_T; Homo_723,totalSample,immune-CD4_T; Homo_723,totalSample,immune-DC; Homo_723,totalSample,immune-Th2; Homo_723,totalSample,immune-Bcell; Homo_714,totalSample,immune-NKT; Homo_714,totalSample,immune-Tr1; Homo_714,totalSample,immune-CD4_naive; Homo_714,totalSample,immune-Cytotoxic; Homo_714,totalSample,immune-Exhausted; Homo_714,totalSample,immune-iTreg; Homo_714,totalSample,immune-Gamma_delta; Homo_714,totalSample,immune-CD8_T; Homo_714,totalSample,immune-CD4_T; Homo_714,totalSample,immune-DC; Homo_714,totalSample,immune-Th2; Homo_633,totalSample,immune-NKT; Homo_633,totalSample,immune-CD4_naive; Homo_633,totalSample,immune-DC; Homo_633,totalSample,immune-Effector_memory; Homo_633,totalSample,immune-Exhausted; Homo_633,totalSample,immune-Tfh; Homo_633,totalSample,immune-Tr1; Homo_633,totalSample,immune-CD4_T; Homo_633,totalSample,immune-iTreg; Homo_633,totalSample,immune-Cytotoxic; Homo_633,totalSample,immune-InfiltrationScore; Homo_633,totalSample,immune-Gamma_delta; Homo_633,totalSample,immune-CD8_T; Homo_633,totalSample,immune-Th2; Homo_633,totalSample,immune-Central_memory; MCSA,totalSample,immune-DC; MCSA,totalSample,immune-Monocyte; MCSA,totalSample,immune-CD4_T; MCSA,totalSample,immune-CD8_T; MCSA,totalSample,immune-CD4_naive; MCSA,totalSample,immune-Tr1; MCSA,totalSample,immune-nTreg; MCSA,totalSample,immune-iTreg; MCSA,totalSample,immune-Th1; MCSA,totalSample,immune-Th17; MCSA,totalSample,immune-Tfh; MCSA,totalSample,immune-CD8_naive; MCSA,totalSample,immune-Cytotoxic; MCSA,totalSample,immune-Exhausted; MCSA,totalSample,immune-MAIT; MCSA,totalSample,immune-Central_memory; SRP223445,totalSample,immune-CD4_naive; SRP223445,totalSample,immune-Exhausted
SYNGR3	ACOM,control,male.vs.female; Homo_723,female,AD.vs.control; Homo_723,male,AD.vs.control; Homo_723,female,AD.vs.MCI; Homo_723,male,AD.vs.MCI; Homo_723,female,MCI.vs.control; Homo_723,MCI,male.vs.female; Homo_714,female,AD.vs.MCI; Homo_714,female,MCI.vs.control; Homo_714,male,MCI.vs.control; Homo_714,MCI,male.vs.female; Homo_633,female,AD.vs.control; Homo_633,female,AD.vs.MCI; Homo_633,female,MCI.vs.control; Homo_633,male,MCI.vs.control; Homo_633,MCI,male.vs.female; ROSMAP,AD,last.visit.age; ROSMAP,AD,sample.age; ROSMAP,MCI,sample.age; Emory,totalSample,immune-Monocyte; Emory,totalSample,immune-NK; Emory,totalSample,immune-Neutrophil; Emory,totalSample,immune-CD8_T; Emory,totalSample,immune-Gamma_delta; Emory,totalSample,immune-Th1; Emory,totalSample,immune-Cytotoxic; Emory,totalSample,immune-Exhausted; Emory,totalSample,immune-Effector_memory; Emory,totalSample,immune-InfiltrationScore; Homo_723,totalSample,immune-Monocyte; Homo_723,totalSample,immune-Exhausted; Homo_723,totalSample,immune-DC; Homo_723,totalSample,immune-CD8_T; Homo_723,totalSample,immune-Neutrophil; Homo_723,totalSample,immune-Cytotoxic; Homo_723,totalSample,immune-InfiltrationScore; Homo_714,totalSample,immune-Monocyte; Homo_714,totalSample,immune-InfiltrationScore; Homo_714,totalSample,immune-Th17; Homo_714,totalSample,immune-Neutrophil; Homo_714,totalSample,immune-Cytotoxic; Homo_714,totalSample,immune-CD8_T; Homo_714,totalSample,immune-DC; Homo_714,totalSample,immune-Exhausted; Homo_714,totalSample,immune-Macrophage; Homo_633,totalSample,immune-Monocyte; Homo_633,totalSample,immune-Th2; Homo_633,totalSample,immune-InfiltrationScore; Homo_633,totalSample,immune-Th17; Homo_633,totalSample,immune-Neutrophil; Homo_633,totalSample,immune-DC; Homo_633,totalSample,immune-Exhausted; Homo_633,totalSample,immune-Cytotoxic; Homo_633,totalSample,immune-Macrophage; MCSA,totalSample,immune-Monocyte; MCSA,totalSample,immune-Neutrophil; MCSA,totalSample,immune-CD8_T; MCSA,totalSample,immune-NKT; MCSA,totalSample,immune-Gamma_delta; MCSA,totalSample,immune-Th2; MCSA,totalSample,immune-Cytotoxic; MCSA,totalSample,immune-Central_memory; MCSA,totalSample,immune-Effector_memory; MCSA,totalSample,immune-InfiltrationScore
PABPC1L	ACOM,control,male.vs.female; Homo_723,female,AD.vs.control; Homo_723,male,AD.vs.control; Homo_723,female,AD.vs.MCI; Homo_723,female,MCI.vs.control; Homo_723,control,male.vs.female; Homo_723,MCI,male.vs.female; Homo_714,female,AD.vs.control; Homo_714,female,AD.vs.MCI; Homo_714,female,MCI.vs.control; Homo_714,control,male.vs.female; Homo_714,MCI,male.vs.female; Homo_633,female,AD.vs.control; Homo_633,female,AD.vs.MCI; Homo_633,female,MCI.vs.control; Homo_633,control,male.vs.female; Homo_633,MCI,male.vs.female; ROSMAP,female,AD.vs.control; ROSMAP,male,AD.vs.control; ROSMAP,female,AD.vs.MCI; Emory,MCI,age; MCSA,totalSample,age; MCSA,control,age; MCSA,MCI,age; ROSMAP,MCI,sample.age; Emory,totalSample,immune-Gamma_delta; Emory,totalSample,immune-iTreg; Homo_723,totalSample,immune-Monocyte; Homo_723,totalSample,immune-DC; Homo_723,totalSample,immune-Exhausted; Homo_723,totalSample,immune-CD8_T; Homo_714,totalSample,immune-Monocyte; Homo_633,totalSample,immune-Monocyte; Homo_633,totalSample,immune-nTreg; MCSA,totalSample,immune-DC; MCSA,totalSample,immune-Monocyte; MCSA,totalSample,immune-Gamma_delta; MCSA,totalSample,immune-CD4_naive; MCSA,totalSample,immune-nTreg; MCSA,totalSample,immune-Th2; MCSA,totalSample,immune-Th17; MCSA,totalSample,immune-MAIT; MCSA,totalSample,immune- [illegible]
DNAJB6	ACOM,control,male.vs.female; Homo_723,female,AD.vs.control; Homo_723,female,AD.vs.MCI; Homo_723,female,MCI.vs.control; Homo_723,MCI,male.vs.female; Homo_714,female,AD.vs.control; Homo_714,female,AD.vs.MCI; Homo_714,female,MCI.vs.control; Homo_714,MCI,male.vs.female; Homo_633,MCI,male.vs.female; ROSMAP,female,AD.vs.control; Emory,MCI,age; Emory,totalSample,immune-Neutrophil; Emory,totalSample,immune-Gamma_delta; Emory,totalSample,immune-Th1; Emory,totalSample,immune-Th17; Emory,totalSample,immune-Tfh; Emory,totalSample,immune-CD8_naive; Emory,totalSample,immune-Cytotoxic; Emory,totalSample,immune-Exhausted; Homo_723,totalSample,immune-Neutrophil; Homo_723,totalSample,immune-CD8_T; Homo_723,totalSample,immune-Bcell; Homo_723,totalSample,immune-Effector_memory; Homo_714,totalSample,immune-CD8_T; Homo_714,totalSample,immune-Neutrophil; Homo_633,totalSample,immune-Neutrophil; Homo_633,totalSample,immune-CD8_T; Homo_633,totalSample,immune-NKT; MCSA,totalSample,immune-DC; MCSA,totalSample,immune-Monocyte; MCSA,totalSample,immune-Neutrophil; MCSA,totalSample,immune-CD8_T; MCSA,totalSample,immune-NKT; MCSA,totalSample,immune-Tr1; MCSA,totalSample,immune-iTreg; MCSA,totalSample,immune-Th1; MCSA,totalSample,immune-Th17; MCSA,totalSample,immune-Cytotoxic; MCSA,totalSample,immune-Exhausted; MCSA,totalSample,immune-Effector_memory; ROSMAP,totalSample,immune-Monocyte; SRP223445,totalSample,immune-CD4_naive; SRP223445,totalSample,immune-Exhausted; ROSMAP,female-excludeAge90+,survival
PLCL2	ACOM,control,male.vs.female; MCSA,MCI,age; ROSMAP,AD,MMSE; Emory,totalSample,immune-Monocyte; Emory,totalSample,immune-Macrophage; Emory,totalSample,immune-CD4_T; Emory,totalSample,immune-Gamma_delta; Emory,totalSample,immune-Tr1; Emory,totalSample,immune-iTreg; Emory,totalSample,immune-Central_memory; Emory,totalSample,immune-Effector_memory; Emory,totalSample,immune-InfiltrationScore; Homo_723,totalSample,immune-Tr1; Homo_723,totalSample,immune-iTreg; Homo_723,totalSample,immune-Tfh; Homo_723,totalSample,immune-CD4_naive; Homo_723,totalSample,immune-CD4_T; Homo_723,totalSample,immune-NKT; Homo_723,totalSample,immune-InfiltrationScore; Homo_723,totalSample,immune-Th2; Homo_723,totalSample,immune-Cytotoxic; Homo_723,totalSample,immune-Central_memory; Homo_723,totalSample,immune-Gamma_delta; Homo_723,totalSample,immune-Effector_memory; Homo_714,totalSample,immune-Tfh; Homo_714,totalSample,immune-Tr1; Homo_714,totalSample,immune-NKT; Homo_714,totalSample,immune-CD4_T; Homo_714,totalSample,immune-CD4_naive; Homo_714,totalSample,immune-iTreg; Homo_714,totalSample,immune-Th2; Homo_714,totalSample,immune-InfiltrationScore; Homo_714,totalSample,immune-Effector_memory; Homo_714,totalSample,immune-Central_memory; Homo_714,totalSample,immune-DC; Homo_714,totalSample,immune-Th1; Homo_714,totalSample,immune-Cytotoxic; Homo_633,totalSample,immune-NKT; Homo_633,totalSample,immune-Tfh; Homo_633,totalSample,immune-CD4_T; Homo_633,totalSample,immune-CD4_naive; Homo_633,totalSample,immune-Tr1; Homo_633,totalSample,immune-iTreg; Homo_633,totalSample,immune-Central_memory; Homo_633,totalSample,immune-InfiltrationScore; Homo_633,totalSample,immune-Effector_memory; Homo_633,totalSample,immune-Gamma_delta; Homo_633,totalSample,immune-DC; Homo_633,totalSample,immune-Th2; Homo_633,totalSample,immune-Th1; Homo_633,totalSample,immune-Cytotoxic; Homo_633,totalSample,immune-Th17; MCSA,totalSample,immune-DC; MCSA,totalSample,immune-Monocyte; MCSA,totalSample,immune-Macrophage; MCSA,totalSample,immune-CD4_T; MCSA,totalSample,immune-CD8_T; MCSA,totalSample,immune-CD4_naive; MCSA,totalSample,immune-Tr1; MCSA,totalSample,immune-nTreg; MCSA,totalSample,immune-iTreg; MCSA,totalSample,immune-Th2; MCSA,totalSample,immune-Th17; MCSA,totalSample,immune-Tfh; MCSA,totalSample,immune-CD8_naive; MCSA,totalSample,immune-Cytotoxic; MCSA,totalSample,immune-Exhausted; MCSA,totalSample,immune-MAIT; MCSA,totalSample,immune-Central_memory; ROSMAP,totalSample,immune-Effector_memory; SRP223445,totalSample,immune-NK; SRP223445,totalSample,immune-CD4_naive; [illegible]
TGFBI	ACOM,control,male.vs.female; Homo_723,male,AD.vs.control; Homo_723,male,AD.vs.MCI; Homo_723,MCI,male.vs.female; Homo_714,male,AD.vs.control; Homo_714,male,AD.vs.MCI; Homo_714,MCI,male.vs.female; Homo_633,control,male.vs.female; Homo_633,MCI,male.vs.female; ROSMAP,female,AD.vs.control; Emory,MCI,age; MCSA,totalSample,age; MCSA,control,age; MCSA,MCI,age; Emory,totalSample,immune-Gamma_delta; Emory,totalSample,immune-Th1; Emory,totalSample,immune-Tfh; Emory,totalSample,immune-CD8_naive; Emory,totalSample,immune-InfiltrationScore; MCSA,totalSample,immune-DC; MCSA,totalSample,immune-Macrophage; MCSA,totalSample,immune-Gamma_delta; MCSA,totalSample,immune-Th17; MCSA,totalSample,immune-InfiltrationScore; ROSMAP,totalSample,immune-Effector_memory; SRP223445,totalSample,immune-CD4_naive; SRP223445,totalSample,immune-Exhausted; SRP223445,totalSample,immune-InfiltrationScore
HS3ST1	ACOM,control,male.vs.female; ROSMAP,male,AD.vs.control; SRP223445,female,AD.vs.control; MCSA,totalSample,age; MCSA,control,age; Emory,totalSample,immune-B_cell; Emory,totalSample,immune-Macrophage; Emory,totalSample,immune-Neutrophil; Emory,totalSample,immune-CD4_T; Emory,totalSample,immune-Tr1; Emory,totalSample,immune-iTreg; Emory,totalSample,immune-Tfh; Emory,totalSample,immune-CD8_naive; Emory,totalSample,immune-InfiltrationScore; Homo_723,totalSample,immune-iTreg; Homo_723,totalSample,immune-CD4_T; Homo_723,totalSample,immune-nTreg; Homo_723,totalSample,immune-Tr1; Homo_723,totalSample,immune-CD4_naive; Homo_723,totalSample,immune-Tfh; Homo_723,totalSample,immune-Central_memory; Homo_723,totalSample,immune-Th2; Homo_723,totalSample,immune-Th1; Homo_723,totalSample,immune-Bcell; Homo_723,totalSample,immune-Macrophage; Homo_714,totalSample,immune-CD4_T; Homo_714,totalSample,immune-iTreg; Homo_714,totalSample,immune-nTreg; Homo_714,totalSample,immune-Tr1; Homo_714,totalSample,immune-CD4_naive; Homo_714,totalSample,immune-Th1; Homo_714,totalSample,immune-Tfh; Homo_714,totalSample,immune-Th2; Homo_714,totalSample,immune-Central_memory; Homo_714,totalSample,immune-NKT; Homo_714,totalSample,immune-Bcell; Homo_633,totalSample,immune-CD4_T; Homo_633,totalSample,immune-nTreg; Homo_633,totalSample,immune-iTreg; Homo_633,totalSample,immune-Tr1; Homo_633,totalSample,immune-Tfh; Homo_633,totalSample,immune-CD4_naive; Homo_633,totalSample,immune-Th1; Homo_633,totalSample,immune-Central_memory; Homo_633,totalSample,immune-NKT; Homo_633,totalSample,immune-Gamma_delta; Homo_633,totalSample,immune-Effector_memory; Homo_633,totalSample,immune-Th2; Homo_633,totalSample,immune-Cytotoxic; MCSA,totalSample,immune-Bcell; MCSA,totalSample,immune-Monocyte; MCSA,totalSample,immune-Macrophage; MCSA,totalSample,immune-Neutrophil; MCSA,totalSample,immune-CD4_T; MCSA,totalSample,immune-Gamma_delta; MCSA,totalSample,immune-CD4_naive; MCSA,totalSample,immune-Tr1; MCSA,totalSample,immune-nTreg; MCSA,totalSample,immune-iTreg; MCSA,totalSample,immune-Th17; MCSA,totalSample,immune-Tfh; MCSA,totalSample,immune-CD8_naive; MCSA,totalSample,immune-Central_memory; MCSA,totalSample,immune-InfiltrationScore; SRP223445,totalSample,immune-Neutrophil; [illegible]
NUDT3	ACOM,control,male.vs.female; Emory,MCI,age; Emory,totalSample,immune-Gamma_delta; Emory,totalSample,immune-Tr1; Emory,totalSample,immune-iTreg; Emory,totalSample,immune-InfiltrationScore; Homo_723,totalSample,immune-InfiltrationScore; Homo_723,totalSample,immune-CD4_naive; Homo_723,totalSample,immune-Tr1; Homo_723,totalSample,immune-iTreg; Homo_723,totalSample,immune-CD4_T; Homo_723,totalSample,immune-Tfh; Homo_723,totalSample,immune-Th2; Homo_723,totalSample,immune-nTreg; Homo_723,totalSample,immune-Cytotoxic; Homo_723,totalSample,immune-Central_memory; Homo_723,totalSample,immune-Gamma_delta; Homo_723,totalSample,immune-Effector_memory; Homo_723,totalSample,immune-Th17; Homo_723,totalSample,immune-NKT; Homo_714,totalSample,immune-iTreg; Homo_714,totalSample,immune-CD4_naive; Homo_714,totalSample,immune-InfiltrationScore; Homo_714,totalSample,immune-Tr1; Homo_714,totalSample,immune-CD4_T; Homo_714,totalSample,immune-Tfh; Homo_714,totalSample,immune-NKT; Homo_714,totalSample,immune-Effector_memory; Homo_714,totalSample,immune-Th2; Homo_714,totalSample,immune-Exhausted; Homo_714,totalSample,immune-Cytotoxic; Homo_714,totalSample,immune-nTreg; Homo_714,totalSample,immune-Th17; Homo_633,totalSample,immune-iTreg; Homo_633,totalSample,immune-CD4_T; Homo_633,totalSample,immune-Tr1; Homo_633,totalSample,immune-CD4_naive; Homo_633,totalSample,immune-Tfh; Homo_633,totalSample,immune-NKT; Homo_633,totalSample,immune-InfiltrationScore; Homo_633,totalSample,immune-Th2; Homo_633,totalSample,immune-Effector_memory; Homo_633,totalSample,immune-Cytotoxic; Homo_633,totalSample,immune-nTreg; Homo_633,totalSample,immune-Exhausted; Homo_633,totalSample,immune-Th17; Homo_633,totalSample,immune-Gamma_delta; MCSA,totalSample,immune-CD4_T; MCSA,totalSample,immune-CD8_T; MCSA,totalSample,immune-Gamma_delta; MCSA,totalSample,immune-CD4_naive; MCSA,totalSample,immune-Tr1; MCSA,totalSample,immune-nTreg; MCSA,totalSample,immune-iTreg; MCSA,totalSample,immune-Th17; MCSA,totalSample,immune-Tfh; MCSA,totalSample,immune-Cytotoxic; MCSA,totalSample,immune-Exhausted; MCSA,totalSample,immune-Central_memory; ROSMAP,totalSample,immune-Effector_memory; SRP223445,totalSample,immune-NK; SRP223445,totalSample,immune-Neutrophil; [illegible]
DMPK	ACOM,control,male.vs.female; Homo_723,male,AD.vs.control; Homo_723,male,MCI.vs.control; Homo_723,MCI,male.vs.female; Homo_714,male,AD.vs.control; Homo_714,male,MCI.vs.control; Homo_714,MCI,male.vs.female; Homo_633,male,MCI.vs.control; Homo_633,MCI,male.vs.female; ROSMAP,female,AD.vs.control; ROSMAP,control,male.vs.female; ACOM,AD,age; MCSA,totalSample,age; MCSA,MCI,age; ROSMAP,MCI,last.visit.age; Emory,totalSample,immune-Monocyte; Emory,totalSample,immune-Macrophage; Emory,totalSample,immune-NK; Emory,totalSample,immune-Neutrophil; Emory,totalSample,immune-CD4_T; Emory,totalSample,immune-CD8_T; Emory,totalSample,immune-Gamma_delta; Emory,totalSample,immune-Tfh; Emory,totalSample,immune-Cytotoxic; Emory,totalSample,immune-Exhausted; Emory,totalSample,immune-InfiltrationScore; Homo_723,totalSample,immune-Cytotoxic; Homo_723,totalSample,immune-DC; Homo_723,totalSample,immune-CD8_T; Homo_723,totalSample,immune-Monocyte; Homo_723,totalSample,immune-Neutrophil; Homo_723,totalSample,immune-Gamma_delta; Homo_723,totalSample,immune-InfiltrationScore; Homo_723,totalSample,immune-Exhausted; Homo_723,totalSample,immune-Effector_memory; Homo_723,totalSample,immune-nTreg; Homo_723,totalSample,immune-Macrophage; Homo_714,totalSample,immune-CD8_T; Homo_714,totalSample,immune-DC; Homo_714,totalSample,immune-Cytotoxic; Homo_714,totalSample,immune-Monocyte; Homo_714,totalSample,immune-Neutrophil; Homo_714,totalSample,immune-InfiltrationScore; Homo_714,totalSample,immune-Gamma_delta; Homo_714,totalSample,immune-Exhausted; Homo_714,totalSample,immune-Macrophage; Homo_714,totalSample,immune-Effector_memory; Homo_714,totalSample,immune-nTreg; Homo_714,totalSample,immune-MAIT; Homo_633,totalSample,immune-CD8_T; Homo_633,totalSample,immune-DC; Homo_633,totalSample,immune-Cytotoxic; Homo_633,totalSample,immune-Monocyte; Homo_633,totalSample,immune-Gamma_delta; Homo_633,totalSample,immune-Neutrophil; Homo_633,totalSample,immune-InfiltrationScore; Homo_633,totalSample,immune-Macrophage; Homo_633,totalSample,immune-Th2; Homo_633,totalSample,immune-Exhausted; Homo_633,totalSample,immune-NKT; Homo_633,totalSample,immune-nTreg; Homo_633,totalSample,immune-Th17; Homo_633,totalSample,immune-Effector_memory; MCSA,totalSample,immune-DC; MCSA,totalSample,immune-Monocyte; MCSA,totalSample,immune-Neutrophil; MCSA,totalSample,immune-CD8_T; MCSA,totalSample,immune-NKT; MCSA,totalSample,immune-iTreg; MCSA,totalSample,immune-Th2; MCSA,totalSample,immune-Cytotoxic; MCSA,totalSample,immune-Exhausted; MCSA,totalSample,immune-Central_memory; [illegible]
LHFPL4	ACOM,control,male.vs.female; Homo_723,female,AD.vs.control; Homo_723,male,AD.vs.control; Homo_723,female,AD.vs.MCI; Homo_723,male,AD.vs.MCI; Homo_723,male,MCI.vs.control; Homo_714,female,AD.vs.MCI; Homo_714,female,MCI.vs.control; Homo_714,male,MCI.vs.control; Homo_633,female,MCI.vs.control; Homo_633,male,MCI.vs.control; ROSMAP,female,AD.vs.control; ROSMAP,female,MCI.vs.control; ROSMAP,control,male.vs.female; ROSMAP,AD,last.visit.age; ROSMAP,MCI,last.visit.age; Emory,totalSample,immune-Gamma_delta; Emory,totalSample,immune-Th17; Homo_723,totalSample,immune-Monocyte; Homo_723,totalSample,immune-Exhausted; Homo_723,totalSample,immune-CD8_naive; Homo_723,totalSample,immune-DC; Homo_714,totalSample,immune-Monocyte; Homo_633,totalSample,immune-Monocyte; Homo_633,totalSample,immune-Th2
DBNDD1	ACOM,control,male.vs.female; ROSMAP,female,AD.vs.control; ROSMAP,female,MCI.vs.control; ROSMAP,male,MCI.vs.control; ACOM,totalSample,age; Emory,totalSample,immune-B_cell; Emory,totalSample,immune-Monocyte; Emory,totalSample,immune-Macrophage; Emory,totalSample,immune-Neutrophil; Emory,totalSample,immune-CD4_T; Emory,totalSample,immune-Tr1; Emory,totalSample,immune-nTreg; Emory,totalSample,immune-iTreg; Emory,totalSample,immune-Tfh; Emory,totalSample,immune-CD8_naive; Emory,totalSample,immune-Central_memory; Emory,totalSample,immune-InfiltrationScore; Homo_723,totalSample,immune-nTreg; Homo_723,totalSample,immune-Monocyte; Homo_723,totalSample,immune-Central_memory; Homo_723,totalSample,immune-Tfh; Homo_723,totalSample,immune-CD4_naive; Homo_723,totalSample,immune-CD4_T; Homo_723,totalSample,immune-InfiltrationScore; Homo_723,totalSample,immune-iTreg; Homo_723,totalSample,immune-Tr1; Homo_723,totalSample,immune-DC; Homo_714,totalSample,immune-nTreg; Homo_714,totalSample,immune-Monocyte; Homo_714,totalSample,immune-Central_memory; Homo_714,totalSample,immune-iTreg; Homo_714,totalSample,immune-CD4_T; Homo_714,totalSample,immune-Tfh; Homo_714,totalSample,immune-CD4_naive; Homo_714,totalSample,immune-InfiltrationScore; Homo_633,totalSample,immune-nTreg; Homo_633,totalSample,immune-Monocyte; Homo_633,totalSample,immune-Tr1; Homo_633,totalSample,immune-Central_memory; Homo_633,totalSample,immune-iTreg; Homo_633,totalSample,immune-CD4_T; Homo_633,totalSample,immune-CD4_naive; Homo_633,totalSample,immune-Tfh; Homo_633,totalSample,immune-DC; MCSA,totalSample,immune-DC; MCSA,totalSample,immune-Monocyte; MCSA,totalSample,immune-Neutrophil; MCSA,totalSample,immune-CD4_T; MCSA,totalSample,immune-CD8_T; MCSA,totalSample,immune-CD4_naive; MCSA,totalSample,immune-nTreg; MCSA,totalSample,immune-Th2; MCSA,totalSample,immune-Tfh; MCSA,totalSample,immune-Cytotoxic; MCSA,totalSample,immune-Central_memory; MCSA,totalSample,immune-Effector_memory; MCSA,totalSample,immune-InfiltrationScore; SRP223445,totalSample,immune-Th17; SRP223445,totalSample,immune-InfiltrationScore
BECN1	ACOM,control,male.vs.female; Homo_723,male,AD.vs.MCI; Homo_723,female,MCI.vs.control; Homo_723,male,MCI.vs.control; Homo_723,AD,male.vs.female; Homo_723,control,male.vs.female; Homo_723,MCI,male.vs.female; Homo_714,female,AD.vs.MCI; Homo_714,male,AD.vs.MCI; Homo_714,female,MCI.vs.control; Homo_714,control,male.vs.female; Homo_714,MCI,male.vs.female; Homo_633,female,AD.vs.control; Homo_633,female,AD.vs.MCI; Homo_633,female,MCI.vs.control; Homo_633,male,MCI.vs.control; Homo_633,control,male.vs.female; Homo_633,MCI,male.vs.female; MCSA,MCI,age; ROSMAP,MCI,sample.age; Emory,totalSample,immune-Gamma_delta; Emory,totalSample,immune-Tr1; Emory,totalSample,immune-iTreg; Emory,totalSample,immune-InfiltrationScore; Homo_723,totalSample,immune-NKT; Homo_723,totalSample,immune-Th1; Homo_723,totalSample,immune-Cytotoxic; Homo_723,totalSample,immune-CD4_naive; Homo_723,totalSample,immune-Tr1; Homo_723,totalSample,immune-InfiltrationScore; Homo_723,totalSample,immune-CD4_T; Homo_714,totalSample,immune-NKT; Homo_714,totalSample,immune-Th1; Homo_714,totalSample,immune-CD4_naive; Homo_714,totalSample,immune-iTreg; Homo_633,totalSample,immune-NKT; Homo_633,totalSample,immune-Th1; Homo_633,totalSample,immune-Tfh; Homo_633,totalSample,immune-CD4_naive; Homo_633,totalSample,immune-DC; MCSA,totalSample,immune-DC; MCSA,totalSample,immune-Monocyte; MCSA,totalSample,immune-CD4_T; MCSA,totalSample,immune-CD8_T; MCSA,totalSample,immune-CD4_naive; MCSA,totalSample,immune-Tr1; MCSA,totalSample,immune-nTreg; MCSA,totalSample,immune-iTreg; MCSA,totalSample,immune-Th17; MCSA,totalSample,immune-Tfh; MCSA,totalSample,immune-CD8_naive; MCSA,totalSample,immune-Cytotoxic; MCSA,totalSample,immune-Exhausted; MCSA,totalSample,immune-MAIT; MCSA,totalSample,immune-Central_memory; ROSMAP,totalSample,immune-Effector_memory; SRP223445,totalSample,immune-InfiltrationScore
ANKRD37	ACOM,control,male.vs.female; Homo_723,male,MCI.vs.control; Homo_723,MCI,male.vs.female; Homo_714,male,AD.vs.control; Homo_714,male,MCI.vs.control; Homo_633,male,MCI.vs.control; Homo_633,MCI,male.vs.female; ROSMAP,male,AD.vs.control; Emory,totalSample,immune-Macrophage; Emory,totalSample,immune-Neutrophil; Emory,totalSample,immune-Tr1; Emory,totalSample,immune-Th1; Emory,totalSample,immune-Exhausted; Emory,totalSample,immune-Effector_memory; Emory,totalSample,immune-InfiltrationScore; Homo_723,totalSample,immune-Neutrophil; Homo_723,totalSample,immune-Th17; Homo_723,totalSample,immune-Exhausted; Homo_723,totalSample,immune-CD8_T; Homo_723,totalSample,immune-InfiltrationScore; Homo_714,totalSample,immune-Th17; Homo_714,totalSample,immune-Neutrophil; Homo_714,totalSample,immune-InfiltrationScore; Homo_633,totalSample,immune-Th17; Homo_633,totalSample,immune-Th2; Homo_633,totalSample,immune-Neutrophil; Homo_633,totalSample,immune-Monocyte; Homo_633,totalSample,immune-DC; MCSA,totalSample,immune-Neutrophil; MCSA,totalSample,immune-CD8_T; MCSA,totalSample,immune-NKT; MCSA,totalSample,immune-Cytotoxic; MCSA,totalSample,immune-Effector_memory
PCDH1	ACOM,control,male.vs.female; Emory,control,male.vs.female; Homo_723,female,AD.vs.control; Homo_723,male,AD.vs.control; Homo_723,female,AD.vs.MCI; Homo_723,male,AD.vs.MCI; Homo_723,female,MCI.vs.control; Homo_723,control,male.vs.female; Homo_723,MCI,male.vs.female; Homo_714,female,AD.vs.control; Homo_714,male,AD.vs.control; Homo_714,female,AD.vs.MCI; Homo_714,male,AD.vs.MCI; Homo_714,female,MCI.vs.control; Homo_714,control,male.vs.female; Homo_714,MCI,male.vs.female; Homo_633,female,AD.vs.control; Homo_633,female,MCI.vs.control; Homo_633,control,male.vs.female; Homo_633,MCI,male.vs.female; ROSMAP,female,AD.vs.control; SRP223445,female,AD.vs.control; ROSMAP,MCI,last.visit.age; Emory,totalSample,immune-Central_memory; Homo_723,totalSample,immune-InfiltrationScore; Homo_723,totalSample,immune-NK; Homo_723,totalSample,immune-DC; Homo_723,totalSample,immune-Monocyte; Homo_723,totalSample,immune-Cytotoxic; Homo_723,totalSample,immune-Neutrophil; Homo_714,totalSample,immune-InfiltrationScore; Homo_714,totalSample,immune-NK; Homo_714,totalSample,immune-Monocyte; Homo_714,totalSample,immune-DC; Homo_714,totalSample,immune-Gamma_delta; Homo_714,totalSample,immune-Th2; Homo_633,totalSample,immune-InfiltrationScore; Homo_633,totalSample,immune-Monocyte; Homo_633,totalSample,immune-NK; Homo_633,totalSample,immune-Th2; Homo_633,totalSample,immune-Gamma_delta; Homo_633,totalSample,immune-Cytotoxic; MCSA,totalSample,immune-Monocyte; MCSA,totalSample,immune-CD8_T; MCSA,totalSample,immune-NKT; MCSA,totalSample,immune-Th2; MCSA,totalSample,immune-Cytotoxic; MCSA,totalSample,immune-Effector_memory; ROSMAP,totalSample,immune-InfiltrationScore; SRP223445,totalSample,immune-NK; SRP223445,totalSample,immune-Neutrophil; SRP223445,totalSample,immune-InfiltrationScore
PER1	ACOM,control,male.vs.female; Homo_714,male,AD.vs.MCI; ROSMAP,female,AD.vs.control; Emory,totalSample,age; Emory,MCI,age; MCSA,totalSample,age; MCSA,control,age; MCSA,MCI,age; Emory,totalSample,immune-Monocyte; Emory,totalSample,immune-CD4_naive; Emory,totalSample,immune-InfiltrationScore; Homo_723,totalSample,immune-InfiltrationScore; Homo_723,totalSample,immune-DC; Homo_723,totalSample,immune-Monocyte; Homo_723,totalSample,immune-Effector_memory; Homo_723,totalSample,immune-nTreg; Homo_714,totalSample,immune-InfiltrationScore; Homo_714,totalSample,immune-DC; Homo_714,totalSample,immune-Monocyte; Homo_714,totalSample,immune-Effector_memory; Homo_633,totalSample,immune-InfiltrationScore; Homo_633,totalSample,immune-DC; Homo_633,totalSample,immune-Monocyte; Homo_633,totalSample,immune-Effector_memory; Homo_633,totalSample,immune-nTreg; MCSA,totalSample,immune-DC; MCSA,totalSample,immune-Monocyte; MCSA,totalSample,immune-CD8_T; MCSA,totalSample,immune-Th2; MCSA,totalSample,immune-Th17; MCSA,totalSample,immune-Cytotoxic; MCSA,totalSample,immune-Effector_memory; MCSA,totalSample,immune-InfiltrationScore; SRP223445,totalSample,immune-Exhausted

STMN2	ACOM,control,male.vs.female; Homo_723,female,AD.vs.control; Homo_723,female,AD.vs.MCI; Homo_723,female,MCI.vs.control; Homo_714,male,AD.vs.MCI; ROSMAP,male,AD.vs.control; ROSMAP,AD,sample.age; Emory,totalSample,immune-Th17
PCDHB3	ACOM,control,male.vs.female; Homo_723,female,AD.vs.control; Homo_723,male,AD.vs.control; Homo_723,female,MCI.vs.control; Homo_714,female,AD.vs.control; Homo_714,male,AD.vs.control; Homo_714,female,MCI.vs.control; Homo_633,female,MCI.vs.control; ROSMAP,female,AD.vs.control; ROSMAP,AD,last.visit.age; Emory,totalSample,immune-CD8_T; Emory,totalSample,immune-Th17; Homo_723,totalSample,immune-DC; Homo_714,totalSample,immune-Th2; ROSMAP,female-excludeAge90+,survival
RAB15	ACOM,control,male.vs.female; ROSMAP,male,MCI.vs.control; ROSMAP,MCI,male.vs.female; MCSA,totalSample,age; MCSA,control,age; MCSA,MCI,age; ROSMAP,AD,last.visit.age; Emory,totalSample,immune-Macrophage; Emory,totalSample,immune-CD4_T; Emory,totalSample,immune-Gamma_delta; Emory,totalSample,immune-CD4_naive; Emory,totalSample,immune-Tr1; Emory,totalSample,immune-iTreg; Emory,totalSample,immune-Th17; Emory,totalSample,immune-Tfh; Emory,totalSample,immune-Cytotoxic; Emory,totalSample,immune-Central_memory; Homo_723,totalSample,immune-nTreg; Homo_723,totalSample,immune-CD4_T; Homo_723,totalSample,immune-CD4_naive; Homo_723,totalSample,immune-Tr1; Homo_723,totalSample,immune-Tfh; Homo_723,totalSample,immune-iTreg; Homo_723,totalSample,immune-Central_memory; Homo_723,totalSample,immune-Th2; Homo_723,totalSample,immune-Th17; Homo_714,totalSample,immune-nTreg; Homo_714,totalSample,immune-CD4_T; Homo_714,totalSample,immune-Tr1; Homo_714,totalSample,immune-CD4_naive; Homo_714,totalSample,immune-iTreg; Homo_714,totalSample,immune-Tfh; Homo_714,totalSample,immune-Th2; Homo_714,totalSample,immune-Central_memory; Homo_714,totalSample,immune-Th17; Homo_633,totalSample,immune-nTreg; Homo_633,totalSample,immune-Tr1; Homo_633,totalSample,immune-CD4_T; Homo_633,totalSample,immune-iTreg; Homo_633,totalSample,immune-Tfh; Homo_633,totalSample,immune-CD4_naive; Homo_633,totalSample,immune-Th2; Homo_633,totalSample,immune-Central_memory; Homo_633,totalSample,immune-Th17; MCSA,totalSample,immune-Monocyte; MCSA,totalSample,immune-CD4_T; MCSA,totalSample,immune-NKT; MCSA,totalSample,immune-CD4_naive; MCSA,totalSample,immune-Tr1; MCSA,totalSample,immune-nTreg; MCSA,totalSample,immune-Th17; MCSA,totalSample,immune-Tfh; MCSA,totalSample,immune-Effector_memory; MCSA,totalSample,immune-InfiltrationScore
IRS2	ACOM,control,male.vs.female; Homo_714,female,AD.vs.control; Emory,totalSample,age; Emory,MCI,age; MCSA,MCI,age; Emory,totalSample,immune-Macrophage; Emory,totalSample,immune-Neutrophil; Emory,totalSample,immune-CD8_T; Emory,totalSample,immune-Gamma_delta; Emory,totalSample,immune-Th1; Emory,totalSample,immune-Th17; Emory,totalSample,immune-Tfh; Emory,totalSample,immune-CD8_naive; Emory,totalSample,immune-Cytotoxic; Emory,totalSample,immune-Exhausted; Homo_723,totalSample,immune-Neutrophil; Homo_723,totalSample,immune-CD8_T; Homo_723,totalSample,immune-Gamma_delta; Homo_723,totalSample,immune-Exhausted; Homo_723,totalSample,immune-Cytotoxic; Homo_723,totalSample,immune-Macrophage; Homo_714,totalSample,immune-Neutrophil; Homo_714,totalSample,immune-CD8_T; Homo_714,totalSample,immune-Exhausted; Homo_714,totalSample,immune-Gamma_delta; Homo_714,totalSample,immune-Cytotoxic; Homo_714,totalSample,immune-MAIT; Homo_714,totalSample,immune-Macrophage; Homo_714,totalSample,immune-Monocyte; Homo_633,totalSample,immune-Neutrophil; Homo_633,totalSample,immune-CD8_T; Homo_633,totalSample,immune-Exhausted; Homo_633,totalSample,immune-Cytotoxic; Homo_633,totalSample,immune-Gamma_delta; Homo_633,totalSample,immune-Macrophage; Homo_633,totalSample,immune-Th17; Homo_633,totalSample,immune-MAIT; Homo_633,totalSample,immune-DC; MCSA,totalSample,immune-DC; MCSA,totalSample,immune-Monocyte; MCSA,totalSample,immune-Neutrophil; MCSA,totalSample,immune-CD8_T; MCSA,totalSample,immune-Th1; MCSA,totalSample,immune-Th17; MCSA,totalSample,immune-Cytotoxic; MCSA,totalSample,immune-
EBF4	ACOM,control,male.vs.female; Homo_723,female,AD.vs.control; Homo_723,male,AD.vs.control; Homo_723,female,AD.vs.MCI; Homo_723,male,AD.vs.MCI; Homo_723,MCI,male.vs.female; Homo_714,female,AD.vs.control; Homo_714,male,AD.vs.control; Homo_714,female,AD.vs.MCI; Homo_714,male,AD.vs.MCI; Homo_714,MCI,male.vs.female; Homo_633,female,AD.vs.control; Homo_633,female,AD.vs.MCI; Homo_633,female,MCI.vs.control; Homo_633,MCI,male.vs.female; MCSA,MCI,age; Emory,totalSample,immune-NK; Emory,totalSample,immune-Neutrophil; Emory,totalSample,immune-CD8_T; Emory,totalSample,immune-Gamma_delta; Emory,totalSample,immune-Tr1; Emory,totalSample,immune-iTreg; Emory,totalSample,immune-Exhausted; Emory,totalSample,immune-Effector_memory; Homo_723,totalSample,immune-DC; Homo_723,totalSample,immune-Monocyte; Homo_723,totalSample,immune-Neutrophil; Homo_723,totalSample,immune-InfiltrationScore; Homo_723,totalSample,immune-NK; Homo_714,totalSample,immune-InfiltrationScore; Homo_714,totalSample,immune-DC; Homo_714,totalSample,immune-Th2; Homo_714,totalSample,immune-Monocyte; Homo_714,totalSample,immune-Neutrophil; Homo_714,totalSample,immune-NK; Homo_633,totalSample,immune-Monocyte; Homo_633,totalSample,immune-InfiltrationScore; Homo_633,totalSample,immune-DC; Homo_633,totalSample,immune-Th2; Homo_633,totalSample,immune-Cytotoxic; Homo_633,totalSample,immune-CD8_T; Homo_633,totalSample,immune-Gamma_delta; Homo_633,totalSample,immune-NK; Homo_633,totalSample,immune-Macrophage; MCSA,totalSample,immune-DC; MCSA,totalSample,immune-Monocyte; MCSA,totalSample,immune-Neutrophil; MCSA,totalSample,immune-CD8_T; MCSA,totalSample,immune-Th2; MCSA,totalSample,immune-Cytotoxic; MCSA,totalSample,immune-Effector_memory; MCSA,totalSample,immune-InfiltrationScore; SRP223445,totalSample,immune-Neutrophil; SRP223445,totalSample,immune-InfiltrationScore; ROSMAP,male and female-excludeAge90+,survival
FAM217B	ACOM,control,male.vs.female; Homo_633,MCI,male.vs.female; Emory,MCI,age; MCSA,MCI,age; Emory,totalSample,immune-Monocyte; Emory,totalSample,immune-Gamma_delta; Emory,totalSample,immune-Tr1; Emory,totalSample,immune-iTreg; Homo_723,totalSample,immune-Tr1; Homo_723,totalSample,immune-Cytotoxic; Homo_723,totalSample,immune-DC; Homo_723,totalSample,immune-iTreg; Homo_723,totalSample,immune-NKT; Homo_723,totalSample,immune-CD4_naive; Homo_723,totalSample,immune-InfiltrationScore; Homo_723,totalSample,immune-CD4_T; Homo_723,totalSample,immune-Tfh; Homo_723,totalSample,immune-Th2; Homo_723,totalSample,immune-Exhausted; Homo_714,totalSample,immune-DC; Homo_714,totalSample,immune-NKT; Homo_714,totalSample,immune-Tr1; Homo_714,totalSample,immune-iTreg; Homo_714,totalSample,immune-CD4_naive; Homo_714,totalSample,immune-Tfh; Homo_714,totalSample,immune-CD4_T; Homo_714,totalSample,immune-InfiltrationScore; Homo_714,totalSample,immune-Cytotoxic; Homo_714,totalSample,immune-Th2; Homo_714,totalSample,immune-Effector_memory; Homo_633,totalSample,immune-DC; Homo_633,totalSample,immune-NKT; Homo_633,totalSample,immune-CD4_naive; Homo_633,totalSample,immune-Tr1; Homo_633,totalSample,immune-CD4_T; Homo_633,totalSample,immune-Tfh; Homo_633,totalSample,immune-iTreg; Homo_633,totalSample,immune-Effector_memory; Homo_633,totalSample,immune-Cytotoxic; Homo_633,totalSample,immune-Th2; Homo_633,totalSample,immune-Central_memory; MCSA,totalSample,immune-DC; MCSA,totalSample,immune-Monocyte; MCSA,totalSample,immune-Macrophage; MCSA,totalSample,immune-CD4_T; MCSA,totalSample,immune-CD8_T; MCSA,totalSample,immune-CD4_naive; MCSA,totalSample,immune-Tr1; MCSA,totalSample,immune-nTreg; MCSA,totalSample,immune-iTreg; MCSA,totalSample,immune-Tfh; MCSA,totalSample,immune-CD8_naive; MCSA,totalSample,immune-Cytotoxic; MCSA,totalSample,immune-Exhausted; MCSA,totalSample,immune-MAIT; MCSA,totalSample,immune-Central_memory; ROSMAP,totalSample,immune-Cytotoxic; ROSMAP,totalSample,immune-Effector_memory; SRP223445,totalSample,immune-NK; SRP223445,totalSample,immune-CD4_naive; SRP223445,totalSample,immune-Th17; SRP223445,totalSample,immune-Exhausted; SRP223445,totalSample,immune-InfiltrationScore; ROSMAP,male-excludeAge90+,survival
MXD4	ACOM,control,male.vs.female; Emory,control,male.vs.female; Homo_723,male,AD.vs.MCI; Homo_714,male,AD.vs.control; Homo_714,male,AD.vs.MCI; ROSMAP,female,AD.vs.control; ROSMAP,female,MCI.vs.control; ROSMAP,AD,male.vs.female; ROSMAP,control,male.vs.female; MCSA,MCI,age; Emory,totalSample,immune-Monocyte; Emory,totalSample,immune-NK; Emory,totalSample,immune-Neutrophil; Emory,totalSample,immune-CD8_T; Emory,totalSample,immune-Gamma_delta; Emory,totalSample,immune-Th17; Emory,totalSample,immune-Cytotoxic; Emory,totalSample,immune-Exhausted; Emory,totalSample,immune-MAIT; Emory,totalSample,immune-Effector_memory; Homo_723,totalSample,immune-DC; Homo_723,totalSample,immune-InfiltrationScore; Homo_723,totalSample,immune-Cytotoxic; Homo_723,totalSample,immune-Neutrophil; Homo_723,totalSample,immune-Effector_memory; Homo_723,totalSample,immune-Monocyte; Homo_723,totalSample,immune-CD8_T; Homo_714,totalSample,immune-DC; Homo_714,totalSample,immune-InfiltrationScore; Homo_714,totalSample,immune-Neutrophil; Homo_714,totalSample,immune-Cytotoxic; Homo_714,totalSample,immune-CD8_T; Homo_714,totalSample,immune-Monocyte; Homo_714,totalSample,immune-Effector_memory; Homo_633,totalSample,immune-DC; Homo_633,totalSample,immune-Th2; Homo_633,totalSample,immune-Neutrophil; Homo_633,totalSample,immune-InfiltrationScore; Homo_633,totalSample,immune-CD8_T; Homo_633,totalSample,immune-Cytotoxic; Homo_633,totalSample,immune-Monocyte; Homo_633,totalSample,immune-Th1; MCSA,totalSample,immune-DC; MCSA,totalSample,immune-Monocyte; MCSA,totalSample,immune-Macrophage; MCSA,totalSample,immune-Neutrophil; MCSA,totalSample,immune-CD8_T; MCSA,totalSample,immune-NKT; MCSA,totalSample,immune-iTreg; MCSA,totalSample,immune-Th2; MCSA,totalSample,immune-Cytotoxic; MCSA,totalSample,immune-Central_memory; MCSA,totalSample,immune-Effector_memory; MCSA,totalSample,immune-InfiltrationScore; ROSMAP,totalSample,immune-Cytotoxic; ROSMAP,totalSample,immune-Central_memory; ROSMAP,totalSample,immune-Effector_memory; SRP223445,totalSample,immune-Neutrophil; SRP223445,totalSample,immune-CD4_naive; SRP223445,totalSample,immune-nTreg; SRP223445,totalSample,immune-Exhausted; SRP223445,totalSample,immune-InfiltrationScore
MAP1A	ACOM,control,male.vs.female; Homo_723,female,AD.vs.control; Homo_723,male,AD.vs.control; Homo_723,female,AD.vs.MCI; Homo_723,male,AD.vs.MCI; Homo_723,female,MCI.vs.control; Homo_723,male,MCI.vs.control; Homo_723,control,male.vs.female; Homo_714,female,AD.vs.control; Homo_714,male,AD.vs.control; Homo_714,female,AD.vs.MCI; Homo_714,male,AD.vs.MCI; Homo_714,female,MCI.vs.control; Homo_714,male,MCI.vs.control; Homo_714,control,male.vs.female; Homo_633,female,AD.vs.control; Homo_633,female,AD.vs.MCI; Homo_633,female,MCI.vs.control; Homo_633,male,MCI.vs.control; ROSMAP,female,AD.vs.control; SRP223445,AD,male.vs.female; MCSA,totalSample,age; MCSA,control,age; MCSA,MCI,age; ROSMAP,AD,last.visit.age; Emory,totalSample,immune-DC; Emory,totalSample,immune-Th1; Emory,totalSample,immune-Effector_memory; Homo_723,totalSample,immune-DC; Homo_723,totalSample,immune-Monocyte; Homo_714,totalSample,immune-DC; Homo_714,totalSample,immune-Th2; Homo_714,totalSample,immune-Monocyte; Homo_633,totalSample,immune-Monocyte; Homo_633,totalSample,immune-DC; MCSA,totalSample,immune-DC; MCSA,totalSample,immune-Monocyte; MCSA,totalSample,immune-NKT; MCSA,totalSample,immune-Gamma_delta; MCSA,totalSample,immune-Th17; MCSA,totalSample,immune-MAIT; MCSA,totalSample,immune-InfiltrationScore;
MED12L	ACOM,control,male.vs.female; MCSA,MCI,age; Homo_723,totalSample,immune-Gamma_delta; Homo_723,totalSample,immune-Cytotoxic; Homo_723,totalSample,immune-Th17; Homo_723,totalSample,immune-NKT; Homo_723,totalSample,immune-CD8_T; Homo_714,totalSample,immune-Th17; Homo_714,totalSample,immune-CD8_T; Homo_714,totalSample,immune-Gamma_delta; Homo_714,totalSample,immune-Cytotoxic; Homo_714,totalSample,immune-Exhausted; Homo_714,totalSample,immune-NKT; Homo_633,totalSample,immune-NKT; Homo_633,totalSample,immune-Th17; Homo_633,totalSample,immune-Gamma_delta; Homo_633,totalSample,immune-Exhausted; Homo_633,totalSample,immune-Tfh; Homo_633,totalSample,immune-CD8_T; Homo_633,totalSample,immune-Cytotoxic; MCSA,totalSample,immune-DC; MCSA,totalSample,immune-CD4_T; MCSA,totalSample,immune-CD8_T; MCSA,totalSample,immune-Gamma_delta; MCSA,totalSample,immune-CD4_naive; MCSA,totalSample,immune-Tr1; MCSA,totalSample,immune-nTreg; MCSA,totalSample,immune-iTreg; MCSA,totalSample,immune-Th17; MCSA,totalSample,immune-Tfh; MCSA,totalSample,immune-CD8_naive; MCSA,totalSample,immune-Cytotoxic; MCSA,totalSample,immune-Exhausted; ROSMAP,totalSample,immune-InfiltrationScore; SRP223445,totalSample,immune-CD4_naive; SRP223445,totalSample,immune-Exhausted; SRP223445,totalSample,immune-
SHISA8	ACOM,control,male.vs.female; Emory,control,male.vs.female; Homo_723,female,AD.vs.MCI; Homo_723,female,MCI.vs.control; Homo_714,female,AD.vs.control; Homo_714,male,AD.vs.control; Homo_714,female,AD.vs.MCI; Homo_714,male,AD.vs.MCI; Homo_714,female,MCI.vs.control; Homo_633,female,AD.vs.MCI; Homo_633,female,MCI.vs.control; ROSMAP,female,AD.vs.control; ROSMAP,AD,sample.age; ROSMAP,totalSample,age; ROSMAP,MCI,sample.age; ROSMAP,totalSample,MMSE; Emory,totalSample,immune-CD8_naive; Homo_723,totalSample,immune-Monocyte; Homo_723,totalSample,immune-Exhausted; Homo_723,totalSample,immune-CD8_T; Homo_714,totalSample,immune-Monocyte; Homo_633,totalSample,immune-Monocyte; MCSA,totalSample,immune-Monocyte; MCSA,totalSample,immune-Neutrophil; MCSA,totalSample,immune-CD8_T; MCSA,totalSample,immune-Cytotoxic; MCSA,totalSample,immune-Central_memory; MCSA,totalSample,immune-Effector_memory; ROSMAP,totalSample,immune-CD4_T; ROSMAP,totalSample,immune-Tr1;
OSBPL9	ACOM,control,male.vs.female; Homo_723,male,AD.vs.MCI; Homo_723,MCI,male.vs.female; Homo_714,MCI,male.vs.female; Homo_633,MCI,male.vs.female; Emory,MCI,age; MCSA,MCI,age; Emory,totalSample,immune-CD4_T; Emory,totalSample,immune-Gamma_delta; Emory,totalSample,immune-Tr1; Emory,totalSample,immune-iTreg; Emory,totalSample,immune-CD8_naive; Emory,totalSample,immune-Central_memory; Emory,totalSample,immune-InfiltrationScore; Homo_723,totalSample,immune-CD4_naive; Homo_723,totalSample,immune-iTreg; Homo_723,totalSample,immune-Tr1; Homo_723,totalSample,immune-CD4_T; Homo_723,totalSample,immune-Cytotoxic; Homo_723,totalSample,immune-Th2; Homo_723,totalSample,immune-Tfh; Homo_723,totalSample,immune-DC; Homo_723,totalSample,immune-Th1; Homo_723,totalSample,immune-NKT; Homo_723,totalSample,immune-InfiltrationScore; Homo_723,totalSample,immune-Effector_memory; Homo_723,totalSample,immune-nTreg; Homo_723,totalSample,immune-Exhausted; Homo_723,totalSample,immune-Central_memory; Homo_723,totalSample,immune-CD8_T; Homo_723,totalSample,immune-Bcell; Homo_714,totalSample,immune-CD4_naive; Homo_714,totalSample,immune-iTreg; Homo_714,totalSample,immune-DC; Homo_714,totalSample,immune-CD4_T; Homo_714,totalSample,immune-Tr1; Homo_714,totalSample,immune-Tfh; Homo_714,totalSample,immune-Th2; Homo_714,totalSample,immune-NKT; Homo_714,totalSample,immune-Effector_memory; Homo_714,totalSample,immune-Cytotoxic; Homo_714,totalSample,immune-Th1; Homo_714,totalSample,immune-InfiltrationScore; Homo_714,totalSample,immune-Central_memory; Homo_714,totalSample,immune-Exhausted; Homo_633,totalSample,immune-DC; Homo_633,totalSample,immune-CD4_naive; Homo_633,totalSample,immune-CD4_T; Homo_633,totalSample,immune-NKT; Homo_633,totalSample,immune-Tfh; Homo_633,totalSample,immune-iTreg; Homo_633,totalSample,immune-Tr1; Homo_633,totalSample,immune-Effector_memory; Homo_633,totalSample,immune-Th2; Homo_633,totalSample,immune-Cytotoxic; Homo_633,totalSample,immune-Th1; Homo_633,totalSample,immune-Central_memory; Homo_633,totalSample,immune-Gamma_delta; Homo_633,totalSample,immune-Bcell; Homo_633,totalSample,immune-CD8_T; Homo_633,totalSample,immune-Th17; MCSA,totalSample,immune-DC; MCSA,totalSample,immune-Neutrophil; MCSA,totalSample,immune-CD4_T; MCSA,totalSample,immune-CD8_T; MCSA,totalSample,immune-Gamma_delta; MCSA,totalSample,immune-CD4_naive; MCSA,totalSample,immune-Tr1; MCSA,totalSample,immune-nTreg; MCSA,totalSample,immune-iTreg; MCSA,totalSample,immune-Th17; MCSA,totalSample,immune-Tfh; MCSA,totalSample,immune-CD8_naive; MCSA,totalSample,immune-Cytotoxic; MCSA,totalSample,immune-Exhausted; MCSA,totalSample,immune-MAIT; MCSA,totalSample,immune-Central_memory; SRP223445,totalSample,immune-NK; SRP223445,totalSample,immune-CD4_naive; SRP223445,totalSample,immune-Exhausted; SRP223445,totalSample,immune-InfiltrationScore
SUPT20HL1	ACOM,control,male.vs.female; Homo_723,female,MCI.vs.control; Homo_723,control,male.vs.female; Homo_714,female,MCI.vs.control; Homo_714,control,male.vs.female; Homo_714,MCI,male.vs.female; Homo_633,female,MCI.vs.control; Homo_633,control,male.vs.female; Homo_633,MCI,male.vs.female; ROSMAP,control,male.vs.female; Emory,totalSample,immune-
PRAM1	ACOM,control,male.vs.female; Homo_723,male,AD.vs.MCI; Homo_714,male,AD.vs.control; Homo_714,male,AD.vs.MCI; Emory,totalSample,age; Emory,control,age; Emory,MCI,age; MCSA,totalSample,age; MCSA,MCI,age; Emory,totalSample,immune-DC; Emory,totalSample,immune-B_cell; Emory,totalSample,immune-Macrophage; Emory,totalSample,immune-Neutrophil; Emory,totalSample,immune-CD4_T; Emory,totalSample,immune-CD8_T; Emory,totalSample,immune-Gamma_delta; Emory,totalSample,immune-Tr1; Emory,totalSample,immune-Th1; Emory,totalSample,immune-Tfh; Emory,totalSample,immune-CD8_naive; Emory,totalSample,immune-Cytotoxic; Emory,totalSample,immune-Exhausted; Homo_723,totalSample,immune-DC; Homo_723,totalSample,immune-Th1; Homo_723,totalSample,immune-CD4_T; Homo_723,totalSample,immune-Tfh; Homo_723,totalSample,immune-Tr1; Homo_723,totalSample,immune-iTreg; Homo_723,totalSample,immune-Th2; Homo_723,totalSample,immune-CD4_naive; Homo_714,totalSample,immune-DC; Homo_714,totalSample,immune-CD4_T; Homo_714,totalSample,immune-Th1; Homo_714,totalSample,immune-Tfh; Homo_714,totalSample,immune-CD4_naive; Homo_714,totalSample,immune-Central_memory; Homo_714,totalSample,immune-Th2; Homo_714,totalSample,immune-Tr1; Homo_714,totalSample,immune-MAIT; Homo_714,totalSample,immune-iTreg; Homo_714,totalSample,immune-Neutrophil; Homo_633,totalSample,immune-DC; Homo_633,totalSample,immune-Tfh; Homo_633,totalSample,immune-Th1; Homo_633,totalSample,immune-CD4_T; Homo_633,totalSample,immune-CD4_naive; Homo_633,totalSample,immune-NKT; Homo_633,totalSample,immune-Neutrophil; Homo_633,totalSample,immune-MAIT; Homo_633,totalSample,immune-Central_memory; MCSA,totalSample,immune-DC; MCSA,totalSample,immune-Bcell; MCSA,totalSample,immune-Monocyte; MCSA,totalSample,immune-Macrophage; MCSA,totalSample,immune-CD4_T; MCSA,totalSample,immune-CD8_T; MCSA,totalSample,immune-CD4_naive; MCSA,totalSample,immune-Tr1; MCSA,totalSample,immune-nTreg; MCSA,totalSample,immune-iTreg; MCSA,totalSample,immune-Th2; MCSA,totalSample,immune-Tfh; MCSA,totalSample,immune-CD8_naive; MCSA,totalSample,immune-Cytotoxic; MCSA,totalSample,immune-MAIT; MCSA,totalSample,immune-InfiltrationScore; ROSMAP,totalSample,immune-MAIT; ROSMAP,totalSample,immune- [illegible]
RPS6	ACOM,control,male.vs.female; ROSMAP,female,AD.vs.control; ROSMAP,female,MCI.vs.control; Emory,totalSample,immune-DC; Emory,totalSample,immune-B_cell; Emory,totalSample,immune-Macrophage; Emory,totalSample,immune-Neutrophil; Emory,totalSample,immune-CD4_T; Emory,totalSample,immune-CD8_T; Emory,totalSample,immune-Tr1; Emory,totalSample,immune-iTreg; Emory,totalSample,immune-Th1; Emory,totalSample,immune-Tfh; Emory,totalSample,immune-CD8_naive; Emory,totalSample,immune-Exhausted; Emory,totalSample,immune-MAIT; Emory,totalSample,immune-Central_memory; Emory,totalSample,immune-InfiltrationScore; Homo_723,totalSample,immune-Tfh; Homo_723,totalSample,immune-nTreg; Homo_723,totalSample,immune-Neutrophil; Homo_723,totalSample,immune-CD4_T; Homo_723,totalSample,immune-iTreg; Homo_723,totalSample,immune-Tr1; Homo_723,totalSample,immune-Th1; Homo_723,totalSample,immune-Central_memory; Homo_723,totalSample,immune-CD4_naive; Homo_723,totalSample,immune-Macrophage; Homo_723,totalSample,immune-Th2; Homo_723,totalSample,immune-CD8_T; Homo_714,totalSample,immune-Neutrophil; Homo_714,totalSample,immune-Tfh; Homo_714,totalSample,immune-Th1; Homo_714,totalSample,immune-nTreg; Homo_714,totalSample,immune-CD4_T; Homo_714,totalSample,immune-Tr1; Homo_714,totalSample,immune-MAIT; Homo_714,totalSample,immune-iTreg; Homo_714,totalSample,immune-CD4_naive; Homo_714,totalSample,immune-Central_memory; Homo_714,totalSample,immune-Macrophage; Homo_633,totalSample,immune-Th1; Homo_633,totalSample,immune-Tfh; Homo_633,totalSample,immune-CD4_T; Homo_633,totalSample,immune-Tr1; Homo_633,totalSample,immune-Neutrophil; Homo_633,totalSample,immune-MAIT; Homo_633,totalSample,immune-nTreg; Homo_633,totalSample,immune-Central_memory; Homo_633,totalSample,immune-CD4_naive; Homo_633,totalSample,immune-iTreg; Homo_633,totalSample,immune-Macrophage; Homo_633,totalSample,immune-CD8_T; MCSA,totalSample,immune-DC; MCSA,totalSample,immune-Bcell; MCSA,totalSample,immune-Macrophage; MCSA,totalSample,immune-Neutrophil; MCSA,totalSample,immune-CD4_T; MCSA,totalSample,immune-CD4_naive; MCSA,totalSample,immune-Tr1; MCSA,totalSample,immune-nTreg; MCSA,totalSample,immune-iTreg; MCSA,totalSample,immune-Th1; MCSA,totalSample,immune-Th2; MCSA,totalSample,immune-Tfh; MCSA,totalSample,immune-CD8_naive; MCSA,totalSample,immune-Exhausted; MCSA,totalSample,immune-Central_memory; ROSMAP,totalSample,immune-Monocyte; SRP223445,totalSample,immune-Neutrophil; SRP223445,totalSample,immune-CD4_naive; SRP223445,totalSample,immune-nTreg; SRP223445,totalSample,immune- [illegible]
USP17L20	ACOM,control,male.vs.female; Homo_723,female,AD.vs.control; Homo_723,male,AD.vs.control; Homo_723,female,MCI.vs.control; Homo_723,male,MCI.vs.control; Homo_714,female,AD.vs.control; Homo_714,male,AD.vs.control; Homo_714,female,MCI.vs.control; Homo_714,male,MCI.vs.control; Homo_714,MCI,male.vs.female; Homo_633,female,MCI.vs.control; Homo_633,male,MCI.vs.control; Homo_633,MCI,male.vs.female; ACOM,totalSample,age; ROSMAP,AD,last.visit.age; Homo_723,totalSample,immune-Monocyte; Homo_723,totalSample,immune-DC; Homo_723,totalSample,immune-Th2; Homo_723,totalSample,immune-Effector_memory; Homo_714,totalSample,immune-Th2; Homo_714,totalSample,immune-Monocyte; Homo_633,totalSample,immune-Monocyte; Homo_633,totalSample,immune-Th2; ROSMAP,totalSample,immune-InfiltrationScore
TXLNG	Emory,control,male.vs.female; Homo_723,female,MCI.vs.control; Homo_714,female,MCI.vs.control; Homo_633,female,AD.vs.control; Homo_633,female,AD.vs.MCI; Homo_633,female,MCI.vs.control; ROSMAP,female,AD.vs.control; MCSA,MCI,age; ROSMAP,AD,last.visit.age; Emory,totalSample,immune-Macrophage; Emory,totalSample,immune-Neutrophil; Emory,totalSample,immune-CD4_T; Emory,totalSample,immune-CD8_T; Emory,totalSample,immune-CD4_naive; Emory,totalSample,immune-Tr1; Emory,totalSample,immune-nTreg; Emory,totalSample,immune-iTreg; Emory,totalSample,immune-Tfh; Emory,totalSample,immune-Exhausted; Emory,totalSample,immune-MAIT; Emory,totalSample,immune-Central_memory; Emory,totalSample,immune-InfiltrationScore; Homo_723,totalSample,immune-Th2; Homo_723,totalSample,immune-Tfh; Homo_723,totalSample,immune-CD4_T; Homo_723,totalSample,immune-iTreg; Homo_723,totalSample,immune-CD4_naive; Homo_723,totalSample,immune-Tr1; Homo_723,totalSample,immune-nTreg; Homo_723,totalSample,immune-Effector_memory; Homo_723,totalSample,immune-Central_memory; Homo_723,totalSample,immune-InfiltrationScore; Homo_723,totalSample,immune-Exhausted; Homo_723,totalSample,immune-Th17; Homo_723,totalSample,immune-Monocyte; Homo_723,totalSample,immune-DC; Homo_723,totalSample,immune-Macrophage; Homo_714,totalSample,immune-Tfh; Homo_714,totalSample,immune-Th2; Homo_714,totalSample,immune-CD4_T; Homo_714,totalSample,immune-CD4_naive; Homo_714,totalSample,immune-Central_memory; Homo_714,totalSample,immune-Tr1; Homo_714,totalSample,immune-Effector_memory; Homo_714,totalSample,immune-iTreg; Homo_714,totalSample,immune-nTreg; Homo_714,totalSample,immune-InfiltrationScore; Homo_714,totalSample,immune-Monocyte; Homo_714,totalSample,immune-Gamma_delta; Homo_714,totalSample,immune-Th17; Homo_633,totalSample,immune-Tfh; Homo_633,totalSample,immune-CD4_T; Homo_633,totalSample,immune-Th2; Homo_633,totalSample,immune-CD4_naive; Homo_633,totalSample,immune-Central_memory; Homo_633,totalSample,immune-Effector_memory; Homo_633,totalSample,immune-Tr1; Homo_633,totalSample,immune-InfiltrationScore; Homo_633,totalSample,immune-iTreg; Homo_633,totalSample,immune-nTreg; Homo_633,totalSample,immune-Th17; Homo_633,totalSample,immune-Exhausted; Homo_633,totalSample,immune-Gamma_delta; Homo_633,totalSample,immune-Monocyte; Homo_633,totalSample,immune-NKT; Homo_633,totalSample,immune-Bcell; Homo_633,totalSample,immune-DC; MCSA,totalSample,immune-DC; MCSA,totalSample,immune-Bcell; MCSA,totalSample,immune-Macrophage; MCSA,totalSample,immune-CD4_T; MCSA,totalSample,immune-CD4_naive; MCSA,totalSample,immune-Tr1; MCSA,totalSample,immune-nTreg; MCSA,totalSample,immune-iTreg; MCSA,totalSample,immune-Th17; MCSA,totalSample,immune-Tfh; MCSA,totalSample,immune-CD8_naive; MCSA,totalSample,immune-Cytotoxic; MCSA,totalSample,immune-Exhausted; MCSA,totalSample,immune-MAIT; [illegible]

PUDP	Emory,control,male.vs.female; Emory,MCI,male.vs.female; Homo_714,female,AD.vs.MCI; Homo_633,female,AD.vs.control; Homo_633,female,AD.vs.MCI; Emory,totalSample,immune-NK; Emory,totalSample,immune-CD4_T; Emory,totalSample,immune-Tr1; Emory,totalSample,immune-iTreg; Emory,totalSample,immune-Central_memory; Emory,totalSample,immune-Effector_memory; Emory,totalSample,immune-InfiltrationScore; Homo_723,totalSample,immune-InfiltrationScore; Homo_723,totalSample,immune-Tfh; Homo_723,totalSample,immune-CD4_naive; Homo_723,totalSample,immune-Tr1; Homo_723,totalSample,immune-iTreg; Homo_723,totalSample,immune-CD4_T; Homo_723,totalSample,immune-Central_memory; Homo_714,totalSample,immune-InfiltrationScore; Homo_714,totalSample,immune-Tfh; Homo_714,totalSample,immune-NKT; Homo_714,totalSample,immune-Tr1; Homo_633,totalSample,immune-InfiltrationScore; Homo_633,totalSample,immune-NKT; Homo_633,totalSample,immune-Th1; Homo_633,totalSample,immune-Tr1; Homo_633,totalSample,immune-Tfh; MCSA,totalSample,immune-Monocyte; MCSA,totalSample,immune-NK; MCSA,totalSample,immune-CD8_T; MCSA,totalSample,immune-NKT; MCSA,totalSample,immune-Tr1; MCSA,totalSample,immune-iTreg; MCSA,totalSample,immune-Th2; MCSA,totalSample,immune-Cytotoxic; ROSMAP,totalSample,immune-CD8_naive; ROSMAP,totalSample,immune-MAIT; ROSMAP,totalSample,immune-Effector_memory; SRP223445,totalSample,immune-InfiltrationScore; ROSMAP,female-excludeAge90+,survival; ROSMAP,male-excludeAge90+,survival
RNF187	Emory,control,male.vs.female; Homo_723,control,male.vs.female; Homo_714,control,male.vs.female; Homo_714,MCI,male.vs.female; Homo_633,control,male.vs.female; Homo_633,MCI,male.vs.female; ROSMAP,male,AD.vs.control; ROSMAP,male,MCI.vs.control; ROSMAP,MCI,male.vs.female; Emory,totalSample,age; Emory,MCI,age; MCSA,MCI,age; Emory,totalSample,immune-Neutrophil; Emory,totalSample,immune-CD8_T; Emory,totalSample,immune-Gamma_delta; Emory,totalSample,immune-CD8_naive; Emory,totalSample,immune-Cytotoxic; Emory,totalSample,immune-Exhausted; Emory,totalSample,immune-MAIT; Homo_723,totalSample,immune-Cytotoxic; Homo_723,totalSample,immune-DC; Homo_723,totalSample,immune-Bcell; Homo_723,totalSample,immune-Neutrophil; Homo_714,totalSample,immune-DC; Homo_714,totalSample,immune-Cytotoxic; Homo_714,totalSample,immune-Neutrophil; Homo_714,totalSample,immune-Th2; Homo_714,totalSample,immune-CD8_T; Homo_633,totalSample,immune-DC; Homo_633,totalSample,immune-Th2; Homo_633,totalSample,immune-NKT; Homo_633,totalSample,immune-Cytotoxic; Homo_633,totalSample,immune-Bcell; Homo_633,totalSample,immune-CD8_T; Homo_633,totalSample,immune-CD4_T; Homo_633,totalSample,immune-Neutrophil; MCSA,totalSample,immune-DC; MCSA,totalSample,immune-Monocyte; MCSA,totalSample,immune-CD8_T; MCSA,totalSample,immune-Tr1; MCSA,totalSample,immune-iTreg; MCSA,totalSample,immune-Th2; MCSA,totalSample,immune-Cytotoxic; MCSA,totalSample,immune-Effector_memory; SRP223445,totalSample,immune-NK; SRP223445,totalSample,immune-CD4_naive; SRP223445,totalSample,immune-Th17; SRP223445,totalSample,immune-InfiltrationScore
CAPN5	Emory,control,male.vs.female; ROSMAP,female,AD.vs.control; ROSMAP,male,AD.vs.control; ROSMAP,female,AD.vs.MCI; ROSMAP,male,AD.vs.MCI; MCSA,totalSample,age; MCSA,control,age; MCSA,MCI,age; Emory,totalSample,immune-Gamma_delta; Emory,totalSample,immune-MAIT; Homo_723,totalSample,immune-DC; Homo_714,totalSample,immune-DC; Homo_633,totalSample,immune-DC; MCSA,totalSample,immune-DC; MCSA,totalSample,immune-Monocyte; MCSA,totalSample,immune-CD8_T; MCSA,totalSample,immune-Th2; MCSA,totalSample,immune-Th17; MCSA,totalSample,immune-Cytotoxic; MCSA,totalSample,immune-Effector_memory; MCSA,totalSample,immune-InfiltrationScore; SRP223445,totalSample,immune-NK; SRP223445,totalSample,immune-Neutrophil; SRP223445,totalSample,immune-InfiltrationScore
AK1	Emory,control,male.vs.female; Homo_723,female,AD.vs.control; Homo_723,male,AD.vs.control; Homo_723,female,AD.vs.MCI; Homo_723,male,AD.vs.MCI; Homo_723,control,male.vs.female; Homo_723,MCI,male.vs.female; Homo_714,female,AD.vs.control; Homo_714,male,AD.vs.control; Homo_714,female,AD.vs.MCI; Homo_714,male,AD.vs.MCI; Homo_714,control,male.vs.female; Homo_633,female,AD.vs.control; Homo_633,female,AD.vs.MCI; Homo_633,female,MCI.vs.control; Homo_633,control,male.vs.female; Homo_633,MCI,male.vs.female; ROSMAP,female,MCI.vs.control; Emory,totalSample,age; Emory,MCI,age; MCSA,totalSample,age; MCSA,control,age; MCSA,MCI,age; Emory,totalSample,immune-CD8_T; Emory,totalSample,immune-Gamma_delta; Emory,totalSample,immune-CD8_naive; Emory,totalSample,immune-Cytotoxic; Emory,totalSample,immune-Exhausted; Homo_723,totalSample,immune-DC; Homo_723,totalSample,immune-Cytotoxic; Homo_723,totalSample,immune-CD8_T; Homo_723,totalSample,immune-Neutrophil; Homo_723,totalSample,immune-Exhausted; Homo_714,totalSample,immune-DC; Homo_714,totalSample,immune-Cytotoxic; Homo_714,totalSample,immune-CD8_T; Homo_714,totalSample,immune-Neutrophil; Homo_714,totalSample,immune-Exhausted; Homo_714,totalSample,immune-Th2; Homo_714,totalSample,immune-Monocyte; Homo_633,totalSample,immune-DC; Homo_633,totalSample,immune-Th2; Homo_633,totalSample,immune-CD8_T; Homo_633,totalSample,immune-Cytotoxic; Homo_633,totalSample,immune-NKT; Homo_633,totalSample,immune-Monocyte; Homo_633,totalSample,immune-Neutrophil; Homo_633,totalSample,immune-Exhausted; Homo_633,totalSample,immune-Macrophage; MCSA,totalSample,immune-DC; MCSA,totalSample,immune-Monocyte; MCSA,totalSample,immune-Neutrophil; MCSA,totalSample,immune-CD8_T; MCSA,totalSample,immune-iTreg; MCSA,totalSample,immune-Th2; MCSA,totalSample,immune-Cytotoxic; MCSA,totalSample,immune-Effector_memory; MCSA,totalSample,immune-InfiltrationScore
GCAT	Emory,control,male.vs.female; Homo_723,female,AD.vs.MCI; Homo_723,male,AD.vs.MCI; Homo_723,female,MCI.vs.control; Homo_723,control,male.vs.female; Homo_714,male,AD.vs.control; Homo_714,female,AD.vs.MCI; Homo_714,male,AD.vs.MCI; Homo_714,female,MCI.vs.control; Homo_714,control,male.vs.female; Homo_633,female,AD.vs.control; Homo_633,female,AD.vs.MCI; Homo_633,female,MCI.vs.control; Homo_633,control,male.vs.female; ROSMAP,male,MCI.vs.control; Emory,MCI,age; MCSA,MCI,age; ROSMAP,MCI,last.visit.age; Homo_723,totalSample,immune-DC; Homo_723,totalSample,immune-CD8_T; Homo_723,totalSample,immune-Exhausted; Homo_723,totalSample,immune-Cytotoxic; Homo_714,totalSample,immune-DC; Homo_633,totalSample,immune-DC; MCSA,totalSample,immune-DC; MCSA,totalSample,immune-Monocyte; MCSA,totalSample,immune-Macrophage; MCSA,totalSample,immune-Neutrophil; MCSA,totalSample,immune-CD8_T; MCSA,totalSample,immune-NKT; MCSA,totalSample,immune-iTreg; MCSA,totalSample,immune-Th2; MCSA,totalSample,immune-Cytotoxic; MCSA,totalSample,immune-Effector_memory; ROSMAP,totalSample,immune-InfiltrationScore; SRP223445,totalSample,immune-Exhausted; ROSMAP,female-excludeAge90+,survival; ROSMAP,female-includeAge90+,survival; ROSMAP,male and female-excludeAge90+,survival; ROSMAP,male and female-includeAge90+,survival
MICAL2	Emory,control,male.vs.female; Emory,MCI,age; Emory,totalSample,immune-Th1; Homo_723,totalSample,immune-Bcell; Homo_723,totalSample,immune-Gamma_delta; Homo_714,totalSample,immune-Bcell; Homo_633,totalSample,immune-Bcell; Homo_633,totalSample,immune-Gamma_delta; MCSA,totalSample,immune-Macrophage; MCSA,totalSample,immune-NK; MCSA,totalSample,immune-CD8_T; MCSA,totalSample,immune-Th17; MCSA,totalSample,immune-Exhausted; ROSMAP,totalSample,immune-Cytotoxic; ROSMAP,totalSample,immune-Effector_memory; ROSMAP,totalSample,immune-InfiltrationScore; SRP223445,totalSample,immune-CD4_naive
SLC48A1	Emory,control,male.vs.female; Homo_723,female,AD.vs.control; Homo_723,male,AD.vs.control; Homo_723,male,AD.vs.MCI; Homo_723,female,MCI.vs.control; Homo_723,male,MCI.vs.control; Homo_723,control,male.vs.female; Homo_723,MCI,male.vs.female; Homo_714,female,AD.vs.control; Homo_714,male,AD.vs.control; Homo_714,female,MCI.vs.control; Homo_714,male,MCI.vs.control; Homo_714,control,male.vs.female; Homo_714,MCI,male.vs.female; Homo_633,male,MCI.vs.control; Homo_633,control,male.vs.female; Homo_633,MCI,male.vs.female; ROSMAP,female,AD.vs.control; MCSA,totalSample,age; MCSA,control,age; MCSA,MCI,age; ROSMAP,MCI,sample.age; Emory,totalSample,immune-Macrophage; Emory,totalSample,immune-Tr1; Emory,totalSample,immune-iTreg; Homo_723,totalSample,immune-DC; Homo_723,totalSample,immune-Monocyte; Homo_723,totalSample,immune-Th2; Homo_723,totalSample,immune-Exhausted; Homo_714,totalSample,immune-Th2; Homo_714,totalSample,immune-DC; Homo_714,totalSample,immune-Monocyte; Homo_633,totalSample,immune-Monocyte; Homo_633,totalSample,immune-Th2; MCSA,totalSample,immune-DC; MCSA,totalSample,immune-Monocyte; MCSA,totalSample,immune-CD8_T; MCSA,totalSample,immune-iTreg; MCSA,totalSample,immune-Th2; MCSA,totalSample,immune-Cytotoxic; MCSA,totalSample,immune-MAIT; ROSMAP,totalSample,immune-
SERF2	Emory,control,male.vs.female; ROSMAP,female,AD.vs.control; ROSMAP,male,AD.vs.control; Emory,MCI,age; MCSA,MCI,age; Emory,totalSample,immune-Gamma_delta; Emory,totalSample,immune-Th17; Emory,totalSample,immune-CD8_naive; Emory,totalSample,immune-Cytotoxic; Homo_723,totalSample,immune-DC; Homo_723,totalSample,immune-Bcell; Homo_714,totalSample,immune-DC; Homo_714,totalSample,immune-Bcell; Homo_714,totalSample,immune-Th2; Homo_633,totalSample,immune-DC; Homo_633,totalSample,immune-Bcell; Homo_633,totalSample,immune-Th2; MCSA,totalSample,immune-DC; MCSA,totalSample,immune-Monocyte; MCSA,totalSample,immune-Macrophage; MCSA,totalSample,immune-Neutrophil; MCSA,totalSample,immune-CD4_T; MCSA,totalSample,immune-CD8_T; MCSA,totalSample,immune-NKT; MCSA,totalSample,immune-Tr1; MCSA,totalSample,immune-iTreg; MCSA,totalSample,immune-Th2; MCSA,totalSample,immune-Th17; MCSA,totalSample,immune-Cytotoxic; MCSA,totalSample,immune-Effector_memory; ROSMAP,totalSample,immune-iTreg; ROSMAP,totalSample,immune-Th17; ROSMAP,totalSample,immune-Effector_memory; SRP223445,totalSample,immune-CD4_naive; SRP223445,totalSample,immune-Exhausted; SRP223445,totalSample,immune-InfiltrationScore
ST6GALNAC4	Emory,control,male.vs.female; Homo_723,male,AD.vs.control; Homo_714,male,AD.vs.control; Homo_714,control,male.vs.female; Homo_633,control,male.vs.female; ROSMAP,female,AD.vs.control; ROSMAP,male,AD.vs.control; ROSMAP,female,MCI.vs.control; Emory,MCI,age; MCSA,MCI,age; ROSMAP,MCI,last.visit.age; Emory,totalSample,immune-Monocyte; Emory,totalSample,immune-Gamma_delta; Emory,totalSample,immune-Tr1; Emory,totalSample,immune-iTreg; Homo_723,totalSample,immune-DC; Homo_723,totalSample,immune-Bcell; Homo_723,totalSample,immune-Cytotoxic; Homo_723,totalSample,immune-Th1; Homo_723,totalSample,immune-Tr1; Homo_723,totalSample,immune-CD4_naive; Homo_723,totalSample,immune-Th2; Homo_714,totalSample,immune-DC; Homo_714,totalSample,immune-Bcell; Homo_714,totalSample,immune-CD4_naive; Homo_714,totalSample,immune-NKT; Homo_714,totalSample,immune-Th1; Homo_714,totalSample,immune-Cytotoxic; Homo_714,totalSample,immune-Th2; Homo_714,totalSample,immune-CD4_T; Homo_633,totalSample,immune-DC; Homo_633,totalSample,immune-Bcell; Homo_633,totalSample,immune-NKT; Homo_633,totalSample,immune-CD4_naive; Homo_633,totalSample,immune-Th2; Homo_633,totalSample,immune-Th1; Homo_633,totalSample,immune-CD4_T; Homo_633,totalSample,immune-iTreg; Homo_633,totalSample,immune-Tfh; Homo_633,totalSample,immune-Cytotoxic; MCSA,totalSample,immune-DC; MCSA,totalSample,immune-Monocyte; MCSA,totalSample,immune-Macrophage; MCSA,totalSample,immune-Neutrophil; MCSA,totalSample,immune-CD4_T; MCSA,totalSample,immune-CD8_T; MCSA,totalSample,immune-Tr1; MCSA,totalSample,immune-iTreg; MCSA,totalSample,immune-Th2; MCSA,totalSample,immune-Cytotoxic; MCSA,totalSample,immune-Exhausted; MCSA,totalSample,immune-MAIT; MCSA,totalSample,immune-Effector_memory; ROSMAP,totalSample,immune-Effector_memory
SLC6A8	Emory,control,male.vs.female; Homo_723,control,male.vs.female; Homo_714,male,AD.vs.control; Homo_714,male,AD.vs.MCI; Homo_714,control,male.vs.female; Homo_633,male,MCI.vs.control; Homo_633,control,male.vs.female; ROSMAP,female,AD.vs.control; ROSMAP,male,AD.vs.control; ROSMAP,male,AD.vs.MCI; ROSMAP,female,MCI.vs.control; ROSMAP,AD,male.vs.female; Emory,MCI,age; MCSA,MCI,age; Emory,totalSample,immune-Gamma_delta; Emory,totalSample,immune-Tr1; Emory,totalSample,immune-iTreg; Emory,totalSample,immune-Th17; Emory,totalSample,immune-CD8_naive; Homo_723,totalSample,immune-Bcell; Homo_723,totalSample,immune-DC; Homo_723,totalSample,immune-iTreg; Homo_723,totalSample,immune-Tr1; Homo_723,totalSample,immune-Th1; Homo_723,totalSample,immune-Cytotoxic; Homo_723,totalSample,immune-CD4_naive; Homo_723,totalSample,immune-Th2; Homo_723,totalSample,immune-CD4_T; Homo_723,totalSample,immune-NKT; Homo_714,totalSample,immune-Bcell; Homo_714,totalSample,immune-DC; Homo_714,totalSample,immune-Th2; Homo_714,totalSample,immune-iTreg; Homo_714,totalSample,immune-CD4_T; Homo_714,totalSample,immune-Th1; Homo_714,totalSample,immune-CD4_naive; Homo_714,totalSample,immune-NKT; Homo_714,totalSample,immune-Tr1; Homo_633,totalSample,immune-Bcell; Homo_633,totalSample,immune-DC; Homo_633,totalSample,immune-NKT; Homo_633,totalSample,immune-CD4_naive; Homo_633,totalSample,immune-CD4_T; Homo_633,totalSample,immune-Th2; Homo_633,totalSample,immune-Th1; Homo_633,totalSample,immune-iTreg; Homo_633,totalSample,immune-Effector_memory; MCSA,totalSample,immune-DC; MCSA,totalSample,immune-Monocyte; MCSA,totalSample,immune-Macrophage; MCSA,totalSample,immune-CD4_T; MCSA,totalSample,immune-Tr1; MCSA,totalSample,immune-iTreg; MCSA,totalSample,immune-Th2; MCSA,totalSample,immune-Cytotoxic; MCSA,totalSample,immune-MAIT; SRP223445,totalSample,immune-InfiltrationScore
MARCHF2	Emory,control,male.vs.female; Homo_723,female,AD.vs.control; Homo_723,female,AD.vs.MCI; Homo_714,female,AD.vs.control; Homo_714,female,AD.vs.MCI; Homo_633,control,male.vs.female; MCSA,MCI,age; Emory,totalSample,immune-CD4_T; Emory,totalSample,immune-Gamma_delta; Emory,totalSample,immune-Tr1; Emory,totalSample,immune-iTreg; Homo_723,totalSample,immune-DC; Homo_723,totalSample,immune-Cytotoxic; Homo_723,totalSample,immune-Th2; Homo_723,totalSample,immune-CD4_naive; Homo_723,totalSample,immune-Tr1; Homo_723,totalSample,immune-iTreg; Homo_723,totalSample,immune-CD4_T; Homo_723,totalSample,immune-Tfh; Homo_723,totalSample,immune-Bcell; Homo_723,totalSample,immune-Th1; Homo_714,totalSample,immune-DC; Homo_714,totalSample,immune-CD4_naive; Homo_714,totalSample,immune-iTreg; Homo_714,totalSample,immune-CD4_T; Homo_714,totalSample,immune-Th2; Homo_714,totalSample,immune-Tfh; Homo_714,totalSample,immune-Th1; Homo_714,totalSample,immune-Bcell; Homo_714,totalSample,immune-Tr1; Homo_633,totalSample,immune-DC; Homo_633,totalSample,immune-NKT; Homo_633,totalSample,immune-Th1; Homo_633,totalSample,immune-CD4_naive; Homo_633,totalSample,immune-CD4_T; Homo_633,totalSample,immune-Bcell; Homo_633,totalSample,immune-Th2; Homo_633,totalSample,immune-Tfh; Homo_633,totalSample,immune-iTreg; Homo_633,totalSample,immune-Tr1; MCSA,totalSample,immune-DC; MCSA,totalSample,immune-Monocyte; MCSA,totalSample,immune-Macrophage; MCSA,totalSample,immune-CD4_T; MCSA,totalSample,immune-CD8_T; MCSA,totalSample,immune-NKT; MCSA,totalSample,immune-Tr1; MCSA,totalSample,immune-iTreg; MCSA,totalSample,immune-Th2; MCSA,totalSample,immune-Cytotoxic; MCSA,totalSample,immune-Effector_memory; SRP223445,totalSample,immune-CD4_naive; SRP223445,totalSample,immune-Exhausted
RXRA	Emory,control,male.vs.female; ROSMAP,female,AD.vs.control; ROSMAP,male,AD.vs.control; Emory,MCI,age; MCSA,MCI,age; ROSMAP,AD,last.visit.age; ROSMAP,AD,sample.age; Emory,totalSample,immune-Macrophage; Emory,totalSample,immune-Neutrophil; Emory,totalSample,immune-CD4_T; Emory,totalSample,immune-CD8_T; Emory,totalSample,immune-Tr1; Emory,totalSample,immune-iTreg; Emory,totalSample,immune-Th1; Emory,totalSample,immune-Tfh; Emory,totalSample,immune-Exhausted; Emory,totalSample,immune-InfiltrationScore; Homo_723,totalSample,immune-DC; Homo_723,totalSample,immune-Th1; Homo_723,totalSample,immune-InfiltrationScore; Homo_714,totalSample,immune-DC; Homo_714,totalSample,immune-Th1; Homo_714,totalSample,immune-CD4_naive; Homo_633,totalSample,immune-DC; Homo_633,totalSample,immune-Th1; Homo_633,totalSample,immune-Exhausted; Homo_633,totalSample,immune-CD4_naive; MCSA,totalSample,immune-DC; MCSA,totalSample,immune-Monocyte; MCSA,totalSample,immune-Macrophage; MCSA,totalSample,immune-NKT; MCSA,totalSample,immune-Gamma_delta; MCSA,totalSample,immune-iTreg; MCSA,totalSample,immune-Th1; MCSA,totalSample,immune-Th2; MCSA,totalSample,immune-Cytotoxic; ROSMAP,totalSample,immune-Central_memory; ROSMAP,totalSample,immune-Effector_memory; SRP223445,totalSample,immune-CD4_naive;
PHOSPHO1	Emory,control,male.vs.female; ROSMAP,female,MCI.vs.control; ROSMAP,AD,male.vs.female; ROSMAP,control,male.vs.female; MCSA,MCI,age; Emory,totalSample,immune-Monocyte; Emory,totalSample,immune-Macrophage; Emory,totalSample,immune-CD4_T; Emory,totalSample,immune-Tr1; Emory,totalSample,immune-iTreg; Emory,totalSample,immune-InfiltrationScore; Homo_723,totalSample,immune-Th1; Homo_723,totalSample,immune-DC; Homo_723,totalSample,immune-Tr1; Homo_723,totalSample,immune-Th2; Homo_723,totalSample,immune-CD4_T; Homo_723,totalSample,immune-CD4_naive; Homo_723,totalSample,immune-Cytotoxic; Homo_723,totalSample,immune-iTreg; Homo_714,totalSample,immune-Th1; Homo_714,totalSample,immune-DC; Homo_714,totalSample,immune-CD4_naive; Homo_714,totalSample,immune-Bcell; Homo_633,totalSample,immune-DC; Homo_633,totalSample,immune-Th1; Homo_633,totalSample,immune-Bcell; Homo_633,totalSample,immune-CD4_naive; Homo_633,totalSample,immune-Th2; MCSA,totalSample,immune-DC; MCSA,totalSample,immune-Monocyte; MCSA,totalSample,immune-Macrophage; MCSA,totalSample,immune-CD4_T; MCSA,totalSample,immune-CD8_T; MCSA,totalSample,immune-NKT; MCSA,totalSample,immune-Tr1; MCSA,totalSample,immune-iTreg; MCSA,totalSample,immune-Th2; MCSA,totalSample,immune-Cytotoxic; MCSA,totalSample,immune-
NEDD4L	Emory,control,male.vs.female; Homo_723,female,AD.vs.control; Homo_723,male,AD.vs.control; Homo_723,female,AD.vs.MCI; Homo_723,male,AD.vs.MCI; Homo_714,female,AD.vs.control; Homo_714,female,AD.vs.MCI; Homo_633,female,AD.vs.control; Homo_633,female,AD.vs.MCI; Emory,totalSample,immune-Gamma_delta; Emory,totalSample,immune-Tr1; Emory,totalSample,immune-iTreg; Homo_723,totalSample,immune-Bcell; Homo_723,totalSample,immune-DC; Homo_723,totalSample,immune-Neutrophil; Homo_723,totalSample,immune-Th17; Homo_714,totalSample,immune-Bcell; Homo_714,totalSample,immune-DC; Homo_633,totalSample,immune-Bcell; SRP223445,totalSample,immune-InfiltrationScore
FOXO3	Emory,control,male.vs.female; Homo_714,male,AD.vs.control; Homo_714,male,AD.vs.MCI; Emory,totalSample,immune-CD4_T; Emory,totalSample,immune-Tr1; Emory,totalSample,immune-iTreg; Emory,totalSample,immune-Th1; Emory,totalSample,immune-Tfh; Homo_723,totalSample,immune-Gamma_delta; Homo_723,totalSample,immune-CD8_T; Homo_723,totalSample,immune-NKT; Homo_723,totalSample,immune-Th17; Homo_714,totalSample,immune-Gamma_delta; Homo_714,totalSample,immune-Exhausted; Homo_714,totalSample,immune-CD8_T; Homo_714,totalSample,immune-Th17; Homo_633,totalSample,immune-Th17; Homo_633,totalSample,immune-Gamma_delta; Homo_633,totalSample,immune-CD8_T; Homo_633,totalSample,immune-Exhausted; Homo_633,totalSample,immune-Monocyte; MCSA,totalSample,immune-DC; MCSA,totalSample,immune-Monocyte; MCSA,totalSample,immune-Neutrophil; MCSA,totalSample,immune-CD8_T; MCSA,totalSample,immune-Gamma_delta; MCSA,totalSample,immune-Tr1; MCSA,totalSample,immune-iTreg; MCSA,totalSample,immune-Th1; MCSA,totalSample,immune-Th17; MCSA,totalSample,immune-Cytotoxic; MCSA,totalSample,immune-Exhausted; MCSA,totalSample,immune-Effector_memory; ROSMAP,totalSample,immune-Macrophage; ROSMAP,totalSample,immune-Effector_memory; SRP223445,totalSample,immune-InfiltrationScore; ROSMAP,female-
SPTB	Emory,control,male.vs.female; ROSMAP,female,AD.vs.control; ROSMAP,female,AD.vs.MCI; ROSMAP,control,male.vs.female; Emory,MCI,age; MCSA,MCI,age; ROSMAP,control,sample.age; ROSMAP,MCI,last.visit.age; Emory,totalSample,immune-Gamma_delta; Emory,totalSample,immune-iTreg; Emory,totalSample,immune-CD8_naive; Homo_723,totalSample,immune-Bcell; Homo_714,totalSample,immune-Bcell; Homo_714,totalSample,immune-DC; Homo_633,totalSample,immune-Bcell; Homo_633,totalSample,immune-DC; Homo_633,totalSample,immune-Th1; MCSA,totalSample,immune-DC; MCSA,totalSample,immune-Macrophage; MCSA,totalSample,immune-NKT; MCSA,totalSample,immune-Th2; SRP223445,totalSample,immune-
MAF1	Emory,control,male.vs.female; Emory,MCI,age; MCSA,MCI,age; Emory,totalSample,immune-Neutrophil; Emory,totalSample,immune-CD8_T; Emory,totalSample,immune-Gamma_delta; Emory,totalSample,immune-CD8_naive; Emory,totalSample,immune-Cytotoxic; Emory,totalSample,immune-Exhausted; Emory,totalSample,immune-MAIT; Homo_723,totalSample,immune-DC; Homo_723,totalSample,immune-Bcell; Homo_723,totalSample,immune-InfiltrationScore; Homo_714,totalSample,immune-DC; Homo_714,totalSample,immune-Bcell; Homo_633,totalSample,immune-DC; Homo_633,totalSample,immune-Bcell; Homo_633,totalSample,immune-InfiltrationScore; MCSA,totalSample,immune-DC; MCSA,totalSample,immune-Monocyte; MCSA,totalSample,immune-Macrophage; MCSA,totalSample,immune-Neutrophil; MCSA,totalSample,immune-CD8_T; MCSA,totalSample,immune-NKT; MCSA,totalSample,immune-iTreg; MCSA,totalSample,immune-Th2; MCSA,totalSample,immune-Cytotoxic; MCSA,totalSample,immune-Central_memory; MCSA,totalSample,immune-Effector_memory;
GUK1	Emory,control,male.vs.female; ROSMAP,female,AD.vs.control; Emory,MCI,age; MCSA,MCI,age; ROSMAP,AD,sample.age; Emory,totalSample,immune-Gamma_delta; Emory,totalSample,immune-Tr1; Emory,totalSample,immune-iTreg; Emory,totalSample,immune-Cytotoxic; Emory,totalSample,immune-InfiltrationScore; Homo_723,totalSample,immune-Bcell; Homo_723,totalSample,immune-DC; Homo_723,totalSample,immune-Cytotoxic; Homo_723,totalSample,immune-Th2; Homo_723,totalSample,immune-Tr1; Homo_723,totalSample,immune-Neutrophil; Homo_714,totalSample,immune-DC; Homo_714,totalSample,immune-Bcell; Homo_714,totalSample,immune-Th2; Homo_714,totalSample,immune-Cytotoxic; Homo_714,totalSample,immune-CD4_naive; Homo_714,totalSample,immune-Th1; Homo_714,totalSample,immune-Tr1; Homo_714,totalSample,immune-CD4_T; Homo_714,totalSample,immune-iTreg; Homo_633,totalSample,immune-DC; Homo_633,totalSample,immune-Bcell; Homo_633,totalSample,immune-Th2; Homo_633,totalSample,immune-NKT; Homo_633,totalSample,immune-Th1; Homo_633,totalSample,immune-CD4_naive; Homo_633,totalSample,immune-CD4_T; Homo_633,totalSample,immune-iTreg; Homo_633,totalSample,immune-Cytotoxic; MCSA,totalSample,immune-DC; MCSA,totalSample,immune-Monocyte; MCSA,totalSample,immune-Macrophage; MCSA,totalSample,immune-Neutrophil; MCSA,totalSample,immune-CD4_T; MCSA,totalSample,immune-CD8_T; MCSA,totalSample,immune-NKT; MCSA,totalSample,immune-Tr1; MCSA,totalSample,immune-iTreg; MCSA,totalSample,immune-Th2; MCSA,totalSample,immune-Cytotoxic; MCSA,totalSample,immune-Effector_memory; SRP223445,totalSample,immune-CD4_naive;
FURIN	Emory,control,male.vs.female; MCSA,totalSample,age; MCSA,MCI,age; Emory,totalSample,immune-Macrophage; Emory,totalSample,immune-CD4_T; Emory,totalSample,immune-Tr1; Emory,totalSample,immune-iTreg; Emory,totalSample,immune-Th1; Emory,totalSample,immune-Tfh; Emory,totalSample,immune-InfiltrationScore; Homo_723,totalSample,immune-Th1; Homo_723,totalSample,immune-DC; Homo_714,totalSample,immune-DC; Homo_714,totalSample,immune-Th1; Homo_714,totalSample,immune-CD4_naive; Homo_633,totalSample,immune-DC; Homo_633,totalSample,immune-Th1; Homo_633,totalSample,immune-CD4_naive; Homo_633,totalSample,immune-Bcell; Homo_633,totalSample,immune-CD4_T; MCSA,totalSample,immune-DC; MCSA,totalSample,immune-Bcell; MCSA,totalSample,immune-Monocyte; MCSA,totalSample,immune-Macrophage; MCSA,totalSample,immune-CD4_T; MCSA,totalSample,immune-CD8_T; MCSA,totalSample,immune-Tr1; MCSA,totalSample,immune-iTreg; MCSA,totalSample,immune-Th2; MCSA,totalSample,immune-Cytotoxic;
HAGH	Emory,control,male.vs.female; Homo_714,female,AD.vs.MCI; Homo_633,control,male.vs.female; ROSMAP,female,AD.vs.control; Emory,MCI,age; MCSA,MCI,age; ROSMAP,AD,last.visit.age; ROSMAP,AD,sample.age; ROSMAP,MCI,last.visit.age; ROSMAP,AD,MMSE; Emory,totalSample,immune-Gamma_delta; Emory,totalSample,immune-Tr1; Emory,totalSample,immune-iTreg; Homo_723,totalSample,immune-Bcell; Homo_723,totalSample,immune-DC; Homo_723,totalSample,immune-Th2; Homo_723,totalSample,immune-Cytotoxic; Homo_723,totalSample,immune-iTreg; Homo_723,totalSample,immune-Tr1; Homo_723,totalSample,immune-CD4_naive; Homo_723,totalSample,immune-CD4_T; Homo_723,totalSample,immune-Th1; Homo_714,totalSample,immune-Bcell; Homo_714,totalSample,immune-DC; Homo_714,totalSample,immune-Th2; Homo_714,totalSample,immune-CD4_T; Homo_714,totalSample,immune-CD4_naive; Homo_714,totalSample,immune-iTreg; Homo_714,totalSample,immune-NKT; Homo_714,totalSample,immune-Th1; Homo_714,totalSample,immune-Tr1; Homo_633,totalSample,immune-Bcell; Homo_633,totalSample,immune-DC; Homo_633,totalSample,immune-NKT; Homo_633,totalSample,immune-CD4_naive; Homo_633,totalSample,immune-CD4_T; Homo_633,totalSample,immune-Th2; Homo_633,totalSample,immune-Th1; Homo_633,totalSample,immune-iTreg; MCSA,totalSample,immune-DC; MCSA,totalSample,immune-Monocyte; MCSA,totalSample,immune-Macrophage; MCSA,totalSample,immune-CD4_T; MCSA,totalSample,immune-CD8_T; MCSA,totalSample,immune-NKT; MCSA,totalSample,immune-Tr1; MCSA,totalSample,immune-iTreg; MCSA,totalSample,immune-Th2; MCSA,totalSample,immune-Cytotoxic;
IGF2	Emory,control,male.vs.female; Homo_723,male,AD.vs.control; Homo_723,male,AD.vs.MCI; Homo_723,control,male.vs.female; Homo_714,male,AD.vs.control; Homo_714,control,male.vs.female; Homo_633,control,male.vs.female; ROSMAP,female,AD.vs.control; SRP223445,female,AD.vs.control; SRP223445,male,AD.vs.control; Emory,totalSample,immune-Tr1; Emory,totalSample,immune-iTreg; Homo_723,totalSample,immune-DC; Homo_723,totalSample,immune-Bcell; Homo_714,totalSample,immune-DC; Homo_714,totalSample,immune-Th2; Homo_633,totalSample,immune-DC; Homo_633,totalSample,immune-Th2; Homo_633,totalSample,immune-Bcell; MCSA,totalSample,immune-DC; MCSA,totalSample,immune-iTreg; MCSA,totalSample,immune-Th2; SRP223445,totalSample,immune-Neutrophil; SRP223445,totalSample,immune-CD4_naive; SRP223445,totalSample,immune-nTreg; SRP223445,totalSample,immune-Exhausted; SRP223445,totalSample,immune-InfiltrationScore

HDGF	Emory,control,male.vs.female; Emory,MCI,age; Emory,totalSample,immune-Gamma_delta; Emory,totalSample,immune-Tr1; Emory,totalSample,immune-iTreg; Emory,totalSample,immune-CD8_naive; Homo_723,totalSample,immune-Bcell; Homo_723,totalSample,immune-Gamma_delta; Homo_714,totalSample,immune-Bcell; Homo_633,totalSample,immune-Bcell; MCSA,totalSample,immune-Macrophage; MCSA,totalSample,immune-Th17; SRP223445,totalSample,immune-InfiltrationScore; ROSMAP,male-includeAge90+,survival
YPEL3	Emory,control,male.vs.female; ROSMAP,control,male.vs.female; MCSA,MCI,age; Emory,totalSample,immune-Macrophage; Emory,totalSample,immune-NK; Emory,totalSample,immune-Neutrophil; Emory,totalSample,immune-CD4_T; Emory,totalSample,immune-Tr1; Emory,totalSample,immune-iTreg; Emory,totalSample,immune-Tfh; Emory,totalSample,immune-InfiltrationScore; Homo_723,totalSample,immune-DC; Homo_723,totalSample,immune-Th1; Homo_714,totalSample,immune-DC; Homo_714,totalSample,immune-Th1; Homo_714,totalSample,immune-CD4_naive; Homo_714,totalSample,immune-Bcell; Homo_714,totalSample,immune-CD4_T; Homo_633,totalSample,immune-DC; Homo_633,totalSample,immune-Th1; Homo_633,totalSample,immune-Bcell; Homo_633,totalSample,immune-CD4_naive; Homo_633,totalSample,immune-CD4_T; MCSA,totalSample,immune-DC; MCSA,totalSample,immune-Monocyte; MCSA,totalSample,immune-Macrophage; MCSA,totalSample,immune-CD4_T; MCSA,totalSample,immune-CD8_T; MCSA,totalSample,immune-Tr1; MCSA,totalSample,immune-iTreg; MCSA,totalSample,immune-Th2; MCSA,totalSample,immune-Cytotoxic; MCSA,totalSample,immune-MAIT; MCSA,totalSample,immune-Effector_memory
CHPT1	Emory,control,male.vs.female; Homo_723,female,AD.vs.control; Homo_723,male,AD.vs.control; Homo_723,female,AD.vs.MCI; Homo_723,male,AD.vs.MCI; Homo_723,female,MCI.vs.control; Homo_714,female,AD.vs.control; Homo_714,male,AD.vs.control; Homo_714,female,AD.vs.MCI; Homo_714,male,AD.vs.MCI; Homo_714,female,MCI.vs.control; Homo_633,female,AD.vs.control; Homo_633,female,AD.vs.MCI; Homo_633,female,MCI.vs.control; ROSMAP,female,AD.vs.control; ACOM,AD,age; Emory,MCI,age; ROSMAP,MCI,last.visit.age; Emory,totalSample,immune-Th17; Emory,totalSample,immune-InfiltrationScore; Homo_723,totalSample,immune-Gamma_delta; Homo_723,totalSample,immune-Exhausted; Homo_633,totalSample,immune-Bcell; MCSA,totalSample,immune-DC; MCSA,totalSample,immune-Monocyte; MCSA,totalSample,immune-CD8_T; MCSA,totalSample,immune-Cytotoxic; MCSA,totalSample,immune-Effector_memory; SRP223445,totalSample,immune-InfiltrationScore
BABAM1	Emory,control,male.vs.female; Emory,totalSample,age; Emory,MCI,age; MCSA,MCI,age; ROSMAP,AD,last.visit.age; Emory,totalSample,immune-Neutrophil; Emory,totalSample,immune-CD8_T; Emory,totalSample,immune-Gamma_delta; Emory,totalSample,immune-Th17; Emory,totalSample,immune-Tfh; Emory,totalSample,immune-CD8_naive; Emory,totalSample,immune-Cytotoxic; Emory,totalSample,immune-Exhausted; Homo_723,totalSample,immune-Bcell; Homo_723,totalSample,immune-Neutrophil; Homo_723,totalSample,immune-DC; Homo_723,totalSample,immune-InfiltrationScore; Homo_723,totalSample,immune-Cytotoxic; Homo_714,totalSample,immune-DC; Homo_714,totalSample,immune-Neutrophil; Homo_714,totalSample,immune-Bcell; Homo_714,totalSample,immune-InfiltrationScore; Homo_714,totalSample,immune-Cytotoxic; Homo_633,totalSample,immune-DC; Homo_633,totalSample,immune-Bcell; Homo_633,totalSample,immune-Neutrophil; Homo_633,totalSample,immune-Th2; MCSA,totalSample,immune-DC; MCSA,totalSample,immune-Monocyte; MCSA,totalSample,immune-Macrophage; MCSA,totalSample,immune-Neutrophil; MCSA,totalSample,immune-CD8_T; MCSA,totalSample,immune-NKT; MCSA,totalSample,immune-iTreg; MCSA,totalSample,immune-Th2; MCSA,totalSample,immune-Cytotoxic; MCSA,totalSample,immune-Effector_memory; MCSA,totalSample,immune-InfiltrationScore; ROSMAP,totalSample,immune-Effector_memory; SRP223445,totalSample,immune-Neutrophil; SRP223445,totalSample,immune-CD4_naive; SRP223445,totalSample,immune-InfiltrationScore
CASTOR2	Emory,control,male.vs.female; ACOM,totalSample,age; Emory,MCI,age; MCSA,totalSample,age; MCSA,control,age; MCSA,MCI,age; ROSMAP,AD,MMSE; Emory,totalSample,immune-Neutrophil; Emory,totalSample,immune-Th1; Emory,totalSample,immune-Tfh; Homo_723,totalSample,immune-Th1; Homo_723,totalSample,immune-Cytotoxic; Homo_714,totalSample,immune-Th1; Homo_633,totalSample,immune-Th1; Homo_633,totalSample,immune-NKT; Homo_633,totalSample,immune-DC; Homo_633,totalSample,immune-CD4_naive; MCSA,totalSample,immune-DC; MCSA,totalSample,immune-Monocyte; MCSA,totalSample,immune-CD8_T; MCSA,totalSample,immune-iTreg; MCSA,totalSample,immune-Th1; MCSA,totalSample,immune-Th2; MCSA,totalSample,immune-Th17; MCSA,totalSample,immune-Cytotoxic
GID4	Emory,control,male.vs.female; Homo_723,male,AD.vs.control; Homo_723,female,AD.vs.MCI; Homo_723,male,AD.vs.MCI; Homo_723,female,MCI.vs.control; Homo_723,male,MCI.vs.control; Homo_723,control,male.vs.female; Homo_714,female,AD.vs.control; Homo_714,male,AD.vs.control; Homo_714,female,AD.vs.MCI; Homo_714,male,AD.vs.MCI; Homo_714,female,MCI.vs.control; Homo_714,male,MCI.vs.control; Homo_714,control,male.vs.female; Homo_633,male,MCI.vs.control; Homo_633,control,male.vs.female; ROSMAP,female,AD.vs.control; ROSMAP,male,AD.vs.control; Emory,totalSample,age; Emory,MCI,age; Emory,totalSample,immune-Th1; Homo_633,totalSample,immune-Th1; MCSA,totalSample,immune-CD4_T; MCSA,totalSample,immune-CD4_naive; MCSA,totalSample,immune-Tr1; MCSA,totalSample,immune-nTreg; MCSA,totalSample,immune-iTreg; MCSA,totalSample,immune-Th17; MCSA,totalSample,immune-Tfh; MCSA,totalSample,immune-Cytotoxic; ROSMAP,totalSample,immune-Effector_memory; SRP223445,totalSample,immune-[illegible]
AP2A1	Emory,control,male.vs.female; ROSMAP,female,AD.vs.MCI; Emory,MCI,age; MCSA,totalSample,age; MCSA,MCI,age; ROSMAP,MCI,last.visit.age; Emory,totalSample,immune-Gamma_delta; Emory,totalSample,immune-Tr1; Emory,totalSample,immune-iTreg; Homo_723,totalSample,immune-DC; Homo_723,totalSample,immune-Th1; Homo_723,totalSample,immune-Cytotoxic; Homo_723,totalSample,immune-Bcell; Homo_714,totalSample,immune-DC; Homo_714,totalSample,immune-Th1; Homo_714,totalSample,immune-Bcell; Homo_633,totalSample,immune-DC; Homo_633,totalSample,immune-Th1; Homo_633,totalSample,immune-Bcell; MCSA,totalSample,immune-DC; MCSA,totalSample,immune-Monocyte; MCSA,totalSample,immune-Macrophage; MCSA,totalSample,immune-CD8_T; MCSA,totalSample,immune-iTreg; MCSA,totalSample,immune-Th2; MCSA,totalSample,immune-Cytotoxic; MCSA,totalSample,immune-MAIT; MCSA,totalSample,immune-Effector_memory; MCSA,totalSample,immune-InfiltrationScore; ROSMAP,totalSample,immune-Effector_memory; SRP223445,totalSample,immune-Neutrophil; SRP223445,totalSample,immune-CD4_naive; SRP223445,totalSample,immune-InfiltrationScore; ROSMAP,male-excludeAge90+,survival
ACHE	Emory,control,male.vs.female; Homo_723,female,AD.vs.control; Homo_723,female,MCI.vs.control; Homo_714,female,MCI.vs.control; Homo_714,control,male.vs.female; Homo_633,female,AD.vs.control; Homo_633,female,AD.vs.MCI; Homo_633,female,MCI.vs.control; Homo_633,male,MCI.vs.control; Homo_633,control,male.vs.female; ROSMAP,female,AD.vs.control; Emory,MCI,age; MCSA,MCI,age; Emory,totalSample,immune-Tr1; Emory,totalSample,immune-iTreg; Emory,totalSample,immune-CD8_naive; Homo_723,totalSample,immune-DC; Homo_723,totalSample,immune-Monocyte; Homo_723,totalSample,immune-Bcell; Homo_723,totalSample,immune-Th2; Homo_723,totalSample,immune-Neutrophil; Homo_714,totalSample,immune-Th2; Homo_714,totalSample,immune-DC; Homo_633,totalSample,immune-Th2; Homo_633,totalSample,immune-DC; Homo_633,totalSample,immune-Monocyte; MCSA,totalSample,immune-DC; MCSA,totalSample,immune-NKT; MCSA,totalSample,immune-Th2
ASGR2	Emory,control,male.vs.female; Homo_723,control,male.vs.female; Homo_714,control,male.vs.female; Homo_633,control,male.vs.female; Emory,totalSample,age; Emory,MCI,age; MCSA,totalSample,age; MCSA,MCI,age; ROSMAP,MCI,last.visit.age; ROSMAP,MCI,MMSE; Emory,totalSample,immune-Macrophage; Emory,totalSample,immune-Neutrophil; Emory,totalSample,immune-CD4_T; Emory,totalSample,immune-CD8_T; Emory,totalSample,immune-Gamma_delta; Emory,totalSample,immune-Tfh; Emory,totalSample,immune-CD8_naive; Emory,totalSample,immune-Exhausted; Emory,totalSample,immune-MAIT; Homo_723,totalSample,immune-CD4_T; Homo_723,totalSample,immune-Tfh; Homo_723,totalSample,immune-Tr1; Homo_723,totalSample,immune-iTreg; Homo_723,totalSample,immune-Macrophage; Homo_723,totalSample,immune-Th2; Homo_723,totalSample,immune-DC; Homo_723,totalSample,immune-CD4_naive; Homo_723,totalSample,immune-Th1; Homo_723,totalSample,immune-Central_memory; Homo_723,totalSample,immune-MAIT; Homo_714,totalSample,immune-CD4_T; Homo_714,totalSample,immune-Tfh; Homo_714,totalSample,immune-CD4_naive; Homo_714,totalSample,immune-Macrophage; Homo_714,totalSample,immune-Central_memory; Homo_714,totalSample,immune-Th2; Homo_714,totalSample,immune-DC; Homo_714,totalSample,immune-Tr1; Homo_714,totalSample,immune-iTreg; Homo_714,totalSample,immune-MAIT; Homo_633,totalSample,immune-Tfh; Homo_633,totalSample,immune-DC; Homo_633,totalSample,immune-CD4_T; Homo_633,totalSample,immune-CD4_naive; Homo_633,totalSample,immune-Macrophage; Homo_633,totalSample,immune-NKT; Homo_633,totalSample,immune-MAIT; Homo_633,totalSample,immune-Central_memory; MCSA,totalSample,immune-DC; MCSA,totalSample,immune-Monocyte; MCSA,totalSample,immune-Macrophage; MCSA,totalSample,immune-CD4_T; MCSA,totalSample,immune-Tr1; MCSA,totalSample,immune-iTreg; MCSA,totalSample,immune-Th2; MCSA,totalSample,immune-MAIT; MCSA,totalSample,immune-InfiltrationScore; ROSMAP,female-[illegible]
FZD5	Emory,control,male.vs.female; ROSMAP,female,AD.vs.control; ROSMAP,AD,last.visit.age; Emory,totalSample,immune-Tr1; Homo_723,totalSample,immune-Bcell; Homo_723,totalSample,immune-Gamma_delta; Homo_633,totalSample,immune-Gamma_delta; Homo_633,totalSample,immune-Bcell; MCSA,totalSample,immune-CD8_T; MCSA,totalSample,immune-Gamma_delta; MCSA,totalSample,immune-iTreg; MCSA,totalSample,immune-Th17; MCSA,totalSample,immune-Cytotoxic; MCSA,totalSample,immune-Exhausted
FTL	Emory,control,male.vs.female; ROSMAP,female,AD.vs.control; ROSMAP,female,MCI.vs.control; ACOM,AD,age; MCSA,MCI,age; ROSMAP,MCI,last.visit.age; Emory,totalSample,immune-Macrophage; Emory,totalSample,immune-CD4_T; Emory,totalSample,immune-Gamma_delta; Emory,totalSample,immune-Tr1; Emory,totalSample,immune-iTreg; Emory,totalSample,immune-Tfh; Emory,totalSample,immune-InfiltrationScore; Homo_723,totalSample,immune-DC; Homo_723,totalSample,immune-Th1; Homo_714,totalSample,immune-DC; Homo_714,totalSample,immune-CD4_naive; Homo_714,totalSample,immune-Bcell; Homo_714,totalSample,immune-Th1; Homo_714,totalSample,immune-CD4_T; Homo_714,totalSample,immune-Th2; Homo_633,totalSample,immune-DC; Homo_633,totalSample,immune-CD4_naive; Homo_633,totalSample,immune-Bcell; Homo_633,totalSample,immune-CD4_T; Homo_633,totalSample,immune-Th1; MCSA,totalSample,immune-DC; MCSA,totalSample,immune-Monocyte; MCSA,totalSample,immune-Macrophage; MCSA,totalSample,immune-CD4_T; MCSA,totalSample,immune-CD4_naive; MCSA,totalSample,immune-Tr1; MCSA,totalSample,immune-nTreg; MCSA,totalSample,immune-iTreg; MCSA,totalSample,immune-Th2; MCSA,totalSample,immune-Tfh; MCSA,totalSample,immune-Cytotoxic; MCSA,totalSample,immune-MAIT; ROSMAP,totalSample,immune-Macrophage; ROSMAP,totalSample,immune-CD4_T; ROSMAP,totalSample,immune-Tr1; SRP223445,totalSample,immune-CD4_naive; SRP223445,totalSample,immune-Exhausted
PTMS	Emory,control,male.vs.female; Homo_723,female,AD.vs.control; Homo_723,male,AD.vs.control; Homo_723,female,AD.vs.MCI; Homo_723,male,AD.vs.MCI; Homo_723,female,MCI.vs.control; Homo_723,male,MCI.vs.control; Homo_714,female,AD.vs.control; Homo_714,male,AD.vs.control; Homo_714,female,AD.vs.MCI; Homo_714,male,AD.vs.MCI; Homo_714,male,MCI.vs.control; Homo_633,female,AD.vs.control; Homo_633,female,AD.vs.MCI; Homo_633,female,MCI.vs.control; ROSMAP,female,AD.vs.control; ROSMAP,male,MCI.vs.control; SRP223445,male,AD.vs.control; MCSA,MCI,age; Emory,totalSample,immune-Monocyte; Emory,totalSample,immune-Gamma_delta; Emory,totalSample,immune-Tr1; Emory,totalSample,immune-iTreg; Emory,totalSample,immune-Th17; Homo_723,totalSample,immune-DC; Homo_723,totalSample,immune-Th2; Homo_723,totalSample,immune-Neutrophil; Homo_723,totalSample,immune-Monocyte; Homo_723,totalSample,immune-Bcell; Homo_714,totalSample,immune-Th2; Homo_714,totalSample,immune-DC; Homo_633,totalSample,immune-Th2; Homo_633,totalSample,immune-DC; MCSA,totalSample,immune-DC; MCSA,totalSample,immune-Monocyte; MCSA,totalSample,immune-Macrophage; MCSA,totalSample,immune-Th2; MCSA,totalSample,immune-Cytotoxic; SRP223445,totalSample,immune-Neutrophil; SRP223445,totalSample,immune-CD4_naive; SRP223445,totalSample,immune-nTreg; SRP223445,totalSample,immune-InfiltrationScore; ROSMAP,male-includeAge90+,survival; ROSMAP,male and female-excludeAge90+,survival; ROSMAP,male and female-includeAge90+,survival
PNPLA2	Emory,control,male.vs.female; ROSMAP,female,AD.vs.control; MCSA,totalSample,age; MCSA,control,age; MCSA,MCI,age; Emory,totalSample,immune-Monocyte; Emory,totalSample,immune-CD4_T; Emory,totalSample,immune-Gamma_delta; Emory,totalSample,immune-Tr1; Emory,totalSample,immune-iTreg; Emory,totalSample,immune-InfiltrationScore; Homo_723,totalSample,immune-DC; Homo_723,totalSample,immune-Monocyte; Homo_714,totalSample,immune-DC; Homo_714,totalSample,immune-Monocyte; Homo_633,totalSample,immune-DC; Homo_633,totalSample,immune-nTreg; Homo_633,totalSample,immune-Monocyte; MCSA,totalSample,immune-DC; MCSA,totalSample,immune-Monocyte; MCSA,totalSample,immune-CD8_T; MCSA,totalSample,immune-NKT; MCSA,totalSample,immune-Gamma_delta; MCSA,totalSample,immune-CD4_naive; MCSA,totalSample,immune-nTreg; MCSA,totalSample,immune-Th2; MCSA,totalSample,immune-Th17; MCSA,totalSample,immune-Cytotoxic; MCSA,totalSample,immune-Central_memory;
NT5E	Emory,control,male.vs.female; Homo_723,female,AD.vs.control; Homo_723,female,MCI.vs.control; Homo_714,female,MCI.vs.control; Homo_633,female,AD.vs.control; Homo_633,female,AD.vs.MCI; Homo_633,female,MCI.vs.control; ROSMAP,female,AD.vs.control; ROSMAP,female,MCI.vs.control; ROSMAP,control,male.vs.female; Emory,totalSample,age; Emory,control,age; Emory,MCI,age; MCSA,totalSample,age; MCSA,control,age; ROSMAP,MCI,last.visit.age; Emory,totalSample,immune-DC; Emory,totalSample,immune-Macrophage; Emory,totalSample,immune-Neutrophil; Emory,totalSample,immune-CD4_T; Emory,totalSample,immune-CD8_T; Emory,totalSample,immune-Tr1; Emory,totalSample,immune-iTreg; Emory,totalSample,immune-Th2; Emory,totalSample,immune-Tfh; Emory,totalSample,immune-CD8_naive; Emory,totalSample,immune-Central_memory; Emory,totalSample,immune-InfiltrationScore; Homo_723,totalSample,immune-Tfh; Homo_723,totalSample,immune-CD4_T; Homo_723,totalSample,immune-CD4_naive; Homo_723,totalSample,immune-Th2; Homo_723,totalSample,immune-Tr1; Homo_723,totalSample,immune-iTreg; Homo_723,totalSample,immune-Central_memory; Homo_723,totalSample,immune-nTreg; Homo_723,totalSample,immune-InfiltrationScore; Homo_723,totalSample,immune-Th17; Homo_723,totalSample,immune-Effector_memory; Homo_723,totalSample,immune-Gamma_delta; Homo_723,totalSample,immune-Macrophage; Homo_714,totalSample,immune-Tfh; Homo_714,totalSample,immune-CD4_T; Homo_714,totalSample,immune-Th2; Homo_714,totalSample,immune-CD4_naive; Homo_714,totalSample,immune-Central_memory; Homo_714,totalSample,immune-Tr1; Homo_714,totalSample,immune-nTreg; Homo_714,totalSample,immune-iTreg; Homo_714,totalSample,immune-Effector_memory; Homo_714,totalSample,immune-Th17; Homo_714,totalSample,immune-InfiltrationScore; Homo_714,totalSample,immune-Th1; Homo_714,totalSample,immune-Macrophage; Homo_714,totalSample,immune-Gamma_delta; Homo_714,totalSample,immune-Neutrophil; Homo_714,totalSample,immune-NKT; Homo_633,totalSample,immune-Tfh; Homo_633,totalSample,immune-CD4_T; Homo_633,totalSample,immune-Th2; Homo_633,totalSample,immune-Central_memory; Homo_633,totalSample,immune-CD4_naive; Homo_633,totalSample,immune-Tr1; Homo_633,totalSample,immune-nTreg; Homo_633,totalSample,immune-Effector_memory; Homo_633,totalSample,immune-iTreg; Homo_633,totalSample,immune-Th17; Homo_633,totalSample,immune-Gamma_delta; Homo_633,totalSample,immune-NKT; Homo_633,totalSample,immune-InfiltrationScore; Homo_633,totalSample,immune-Exhausted; Homo_633,totalSample,immune-Macrophage; MCSA,totalSample,immune-DC; MCSA,totalSample,immune-Bcell; MCSA,totalSample,immune-Macrophage; MCSA,totalSample,immune-CD4_T; MCSA,totalSample,immune-Gamma_delta; MCSA,totalSample,immune-CD4_naive; MCSA,totalSample,immune-Tr1; MCSA,totalSample,immune-nTreg; MCSA,totalSample,immune-iTreg; MCSA,totalSample,immune-Th17; MCSA,totalSample,immune-Tfh; MCSA,totalSample,immune-CD8_naive; MCSA,totalSample,immune-Exhausted; MCSA,totalSample,immune-Central_memory; MCSA,totalSample,immune-Effector_memory; ROSMAP,totalSample,immune-InfiltrationScore; SRP223445,totalSample,immune-NK; SRP223445,totalSample,immune-nTreg; SRP223445,totalSample,immune-Exhausted; SRP223445,totalSample,immune-InfiltrationScore
SHISA7	Emory,control,male.vs.female; Homo_723,female,AD.vs.control; Homo_723,male,AD.vs.control; Homo_723,female,AD.vs.MCI; Homo_723,male,AD.vs.MCI; Homo_723,female,MCI.vs.control; Homo_714,female,AD.vs.control; Homo_714,female,MCI.vs.control; Homo_633,female,AD.vs.MCI; Homo_633,female,MCI.vs.control; Homo_633,control,male.vs.female; MCSA,MCI,age; Emory,totalSample,immune-Monocyte; Emory,totalSample,immune-Tr1; Emory,totalSample,immune-iTreg; Homo_723,totalSample,immune-DC; Homo_723,totalSample,immune-Monocyte; Homo_723,totalSample,immune-Bcell; Homo_714,totalSample,immune-Th2; Homo_714,totalSample,immune-DC; Homo_633,totalSample,immune-Th2; Homo_633,totalSample,immune-Monocyte; SRP223445,totalSample,immune-InfiltrationScore
ROGDI	Emory,control,male.vs.female; Homo_723,male,AD.vs.control; Homo_723,female,AD.vs.MCI; Homo_723,male,AD.vs.MCI; Homo_723,male,MCI.vs.control; Homo_723,AD,male.vs.female; Homo_723,control,male.vs.female; Homo_714,male,AD.vs.control; Homo_714,male,AD.vs.MCI; Homo_714,female,MCI.vs.control; Homo_714,male,MCI.vs.control; Homo_714,control,male.vs.female; Homo_633,male,MCI.vs.control; Homo_633,control,male.vs.female; Homo_633,MCI,male.vs.female; ROSMAP,female,AD.vs.control; ROSMAP,male,AD.vs.control; MCSA,MCI,age; Emory,totalSample,immune-Gamma_delta; Emory,totalSample,immune-Tr1; Emory,totalSample,immune-iTreg; Emory,totalSample,immune-Th17; Emory,totalSample,immune-Cytotoxic; Homo_723,totalSample,immune-DC; Homo_723,totalSample,immune-Th2; Homo_723,totalSample,immune-Monocyte; Homo_714,totalSample,immune-Th2; Homo_714,totalSample,immune-DC; Homo_633,totalSample,immune-Th2; MCSA,totalSample,immune-DC; MCSA,totalSample,immune-Monocyte; MCSA,totalSample,immune-Macrophage; MCSA,totalSample,immune-Neutrophil; MCSA,totalSample,immune-CD4_T; MCSA,totalSample,immune-CD8_T; MCSA,totalSample,immune-NKT; MCSA,totalSample,immune-Tr1; MCSA,totalSample,immune-iTreg; MCSA,totalSample,immune-Th2; MCSA,totalSample,immune-Cytotoxic; MCSA,totalSample,immune-Effector_memory;
LGALS3	Emory,control,male.vs.female; Homo_723,male,AD.vs.control; Homo_723,male,AD.vs.MCI; Homo_723,female,MCI.vs.control; Homo_723,control,male.vs.female; Homo_714,male,AD.vs.control; Homo_714,male,AD.vs.MCI; Homo_714,female,MCI.vs.control; Homo_714,control,male.vs.female; Homo_633,female,MCI.vs.control; Homo_633,control,male.vs.female; ROSMAP,female,AD.vs.control; ROSMAP,female,MCI.vs.control; Emory,totalSample,age; Emory,MCI,age; ROSMAP,AD,sample.age; ROSMAP,MCI,last.visit.age; ROSMAP,MCI,sample.age; Emory,totalSample,immune-Gamma_delta; Emory,totalSample,immune-Th17; Emory,totalSample,immune-CD8_naive; Emory,totalSample,immune-Cytotoxic; Homo_723,totalSample,immune-Bcell; Homo_723,totalSample,immune-iTreg; Homo_723,totalSample,immune-Th2; Homo_723,totalSample,immune-Tr1; Homo_723,totalSample,immune-CD4_T; Homo_723,totalSample,immune-CD4_naive; Homo_723,totalSample,immune-Cytotoxic; Homo_723,totalSample,immune-Monocyte; Homo_723,totalSample,immune-NKT; Homo_723,totalSample,immune-DC; Homo_723,totalSample,immune-Neutrophil; Homo_714,totalSample,immune-Th2; Homo_714,totalSample,immune-iTreg; Homo_714,totalSample,immune-CD4_T; Homo_714,totalSample,immune-CD4_naive; Homo_714,totalSample,immune-Tr1; Homo_714,totalSample,immune-Bcell; Homo_714,totalSample,immune-Tfh; Homo_714,totalSample,immune-DC; Homo_714,totalSample,immune-NKT; Homo_714,totalSample,immune-Central_memory; Homo_714,totalSample,immune-Monocyte; Homo_633,totalSample,immune-Th2; Homo_633,totalSample,immune-CD4_T; Homo_633,totalSample,immune-CD4_naive; Homo_633,totalSample,immune-iTreg; Homo_633,totalSample,immune-Bcell; Homo_633,totalSample,immune-NKT; Homo_633,totalSample,immune-DC; Homo_633,totalSample,immune-Tfh; Homo_633,totalSample,immune-Effector_memory; Homo_633,totalSample,immune-Tr1; Homo_633,totalSample,immune-Central_memory; Homo_633,totalSample,immune-Cytotoxic; Homo_633,totalSample,immune-nTreg; MCSA,totalSample,immune-DC; MCSA,totalSample,immune-Macrophage; MCSA,totalSample,immune-CD4_T; MCSA,totalSample,immune-CD4_naive; MCSA,totalSample,immune-Tr1; MCSA,totalSample,immune-nTreg; MCSA,totalSample,immune-iTreg; MCSA,totalSample,immune-Tfh; MCSA,totalSample,immune-MAIT; MCSA,totalSample,immune-Central_memory; SRP223445,totalSample,immune-[illegible]
ELOB	Emory,control,male.vs.female; ROSMAP,female,AD.vs.control; Emory,MCI,age; MCSA,MCI,age; ROSMAP,MCI,last.visit.age; Emory,totalSample,immune-NKT; Emory,totalSample,immune-Gamma_delta; Emory,totalSample,immune-CD4_naive; Emory,totalSample,immune-Th17; Emory,totalSample,immune-Tfh; Emory,totalSample,immune-CD8_naive; Emory,totalSample,immune-Cytotoxic; Emory,totalSample,immune-Central_memory; Homo_723,totalSample,immune-Cytotoxic; Homo_723,totalSample,immune-Th2; Homo_723,totalSample,immune-DC; Homo_723,totalSample,immune-CD4_T; Homo_723,totalSample,immune-CD4_naive; Homo_723,totalSample,immune-Tr1; Homo_723,totalSample,immune-Bcell; Homo_723,totalSample,immune-iTreg; Homo_723,totalSample,immune-Neutrophil; Homo_723,totalSample,immune-NKT; Homo_723,totalSample,immune-Th1; Homo_714,totalSample,immune-Th2; Homo_714,totalSample,immune-DC; Homo_714,totalSample,immune-CD4_T; Homo_714,totalSample,immune-CD4_naive; Homo_714,totalSample,immune-Cytotoxic; Homo_714,totalSample,immune-NKT; Homo_714,totalSample,immune-iTreg; Homo_714,totalSample,immune-Th1; Homo_714,totalSample,immune-Tr1; Homo_714,totalSample,immune-Bcell; Homo_633,totalSample,immune-DC; Homo_633,totalSample,immune-Th2; Homo_633,totalSample,immune-NKT; Homo_633,totalSample,immune-CD4_T; Homo_633,totalSample,immune-Cytotoxic; Homo_633,totalSample,immune-CD4_naive; Homo_633,totalSample,immune-Bcell; Homo_633,totalSample,immune-Th1; Homo_633,totalSample,immune-iTreg; Homo_633,totalSample,immune-Tfh; Homo_633,totalSample,immune-CD8_T; Homo_633,totalSample,immune-Tr1; MCSA,totalSample,immune-DC; MCSA,totalSample,immune-Monocyte; MCSA,totalSample,immune-Macrophage; MCSA,totalSample,immune-Neutrophil; MCSA,totalSample,immune-CD4_T; MCSA,totalSample,immune-CD8_T; MCSA,totalSample,immune-NKT; MCSA,totalSample,immune-CD4_naive; MCSA,totalSample,immune-Tr1; MCSA,totalSample,immune-nTreg; MCSA,totalSample,immune-iTreg; MCSA,totalSample,immune-Th2; MCSA,totalSample,immune-Th17; MCSA,totalSample,immune-Cytotoxic; MCSA,totalSample,immune-Exhausted; MCSA,totalSample,immune-Effector_memory; SRP223445,totalSample,immune-NK; SRP223445,totalSample,immune-CD4_T; ROSMAP,male and female-includeAge90+,survival
RAD23A	Emory,control,male.vs.female; Homo_723,male,AD.vs.control; Homo_723,female,AD.vs.MCI; Homo_723,male,AD.vs.MCI; Homo_723,control,male.vs.female; Homo_714,male,AD.vs.control; Homo_714,female,AD.vs.MCI; Homo_714,male,AD.vs.MCI; Homo_714,female,MCI.vs.control; Homo_714,control,male.vs.female; Homo_633,female,MCI.vs.control; Homo_633,control,male.vs.female; Emory,totalSample,age; Emory,MCI,age; MCSA,totalSample,age; MCSA,MCI,age; Emory,totalSample,immune-Monocyte; Emory,totalSample,immune-Neutrophil; Emory,totalSample,immune-CD8_T; Emory,totalSample,immune-Gamma_delta; Emory,totalSample,immune-CD8_naive; Emory,totalSample,immune-Cytotoxic; Emory,totalSample,immune-Exhausted; Homo_723,totalSample,immune-DC; Homo_723,totalSample,immune-Cytotoxic; Homo_723,totalSample,immune-Neutrophil; Homo_723,totalSample,immune-Bcell; Homo_723,totalSample,immune-CD8_T; Homo_714,totalSample,immune-DC; Homo_714,totalSample,immune-Neutrophil; Homo_714,totalSample,immune-Cytotoxic; Homo_714,totalSample,immune-CD8_T; Homo_714,totalSample,immune-Bcell; Homo_633,totalSample,immune-DC; Homo_633,totalSample,immune-NKT; Homo_633,totalSample,immune-Th2; Homo_633,totalSample,immune-Bcell; Homo_633,totalSample,immune-Cytotoxic; Homo_633,totalSample,immune-Neutrophil; Homo_633,totalSample,immune-CD8_T; Homo_633,totalSample,immune-Th1; MCSA,totalSample,immune-DC; MCSA,totalSample,immune-Monocyte; MCSA,totalSample,immune-Neutrophil; MCSA,totalSample,immune-CD8_T; MCSA,totalSample,immune-Th2; MCSA,totalSample,immune-Cytotoxic; MCSA,totalSample,immune-Effector_memory; MCSA,totalSample,immune-InfiltrationScore;

PINK1	Emory,control,male.vs.female; ROSMAP,female,AD.vs.control; ROSMAP,female,AD.vs.MCI; MCSA,MCI,age; Emory,totalSample,immune-Macrophage; Emory,totalSample,immune-CD4_T; Emory,totalSample,immune-Tr1; Emory,totalSample,immune-iTreg; Emory,totalSample,immune-InfiltrationScore; Homo_723,totalSample,immune-Bcell; Homo_723,totalSample,immune-DC; Homo_723,totalSample,immune-Th1; Homo_714,totalSample,immune-Bcell; Homo_714,totalSample,immune-DC; Homo_714,totalSample,immune-Th1; Homo_714,totalSample,immune-CD4_naive; Homo_633,totalSample,immune-Bcell; Homo_633,totalSample,immune-DC; Homo_633,totalSample,immune-Th1; Homo_633,totalSample,immune-CD4_naive; Homo_633,totalSample,immune-Th2; MCSA,totalSample,immune-DC; MCSA,totalSample,immune-Monocyte; MCSA,totalSample,immune-Macrophage; MCSA,totalSample,immune-CD4_T; MCSA,totalSample,immune-CD8_T; MCSA,totalSample,immune-NKT; MCSA,totalSample,immune-Tr1; MCSA,totalSample,immune-iTreg; MCSA,totalSample,immune-Th2; MCSA,totalSample,immune-Cytotoxic; MCSA,totalSample,immune-Effector_memory; SRP223445,totalSample,immune-CD4_naive; SRP223445,totalSample,immune-Exhausted; SRP223445,totalSample,immune-InfiltrationScore
TESC	Emory,control,male.vs.female; Homo_723,male,AD.vs.control; Homo_723,male,AD.vs.MCI; Homo_723,control,male.vs.female; Homo_714,female,AD.vs.control; Homo_714,male,AD.vs.control; Homo_714,female,AD.vs.MCI; Homo_714,male,AD.vs.MCI; Homo_714,control,male.vs.female; Emory,MCI,age; MCSA,MCI,age; ROSMAP,MCI,last.visit.age; ROSMAP,MCI,sample.age; Emory,totalSample,immune-Gamma_delta; Emory,totalSample,immune-Tr1; Emory,totalSample,immune-iTreg; Emory,totalSample,immune-Th17; Emory,totalSample,immune-Cytotoxic; Homo_723,totalSample,immune-Bcell; Homo_723,totalSample,immune-Cytotoxic; Homo_723,totalSample,immune-DC; Homo_723,totalSample,immune-Th2; Homo_723,totalSample,immune-Tr1; Homo_723,totalSample,immune-iTreg; Homo_723,totalSample,immune-CD4_naive; Homo_723,totalSample,immune-Neutrophil; Homo_723,totalSample,immune-CD4_T; Homo_714,totalSample,immune-DC; Homo_714,totalSample,immune-Th2; Homo_714,totalSample,immune-Bcell; Homo_714,totalSample,immune-Cytotoxic; Homo_714,totalSample,immune-iTreg; Homo_714,totalSample,immune-CD4_naive; Homo_714,totalSample,immune-CD4_T; Homo_714,totalSample,immune-Tr1; Homo_714,totalSample,immune-NKT; Homo_714,totalSample,immune-Th1; Homo_633,totalSample,immune-DC; Homo_633,totalSample,immune-Th2; Homo_633,totalSample,immune-Bcell; Homo_633,totalSample,immune-NKT; Homo_633,totalSample,immune-CD4_naive; Homo_633,totalSample,immune-CD4_T; Homo_633,totalSample,immune-iTreg; Homo_633,totalSample,immune-Cytotoxic; Homo_633,totalSample,immune-Th1; Homo_633,totalSample,immune-Tfh; MCSA,totalSample,immune-DC; MCSA,totalSample,immune-Macrophage; MCSA,totalSample,immune-CD4_T; MCSA,totalSample,immune-CD8_T; MCSA,totalSample,immune-NKT; MCSA,totalSample,immune-Tr1; MCSA,totalSample,immune-nTreg; MCSA,totalSample,immune-iTreg; MCSA,totalSample,immune-Th2; MCSA,totalSample,immune-Cytotoxic; SRP223445,totalSample,immune-Neutrophil; SRP223445,totalSample,immune-[illegible]
SHARPIN	Emory,control,male.vs.female; Homo_723,male,AD.vs.control; Homo_723,male,MCI.vs.control; Homo_723,control,male.vs.female; Homo_714,female,AD.vs.control; Homo_714,male,AD.vs.control; Homo_714,female,AD.vs.MCI; Homo_714,male,MCI.vs.control; Homo_714,control,male.vs.female; Homo_633,male,MCI.vs.control; Homo_633,control,male.vs.female; ROSMAP,female,AD.vs.control; ACOM,AD,age; Emory,MCI,age; MCSA,totalSample,age; MCSA,control,age; MCSA,MCI,age; Emory,totalSample,immune-Monocyte; Emory,totalSample,immune-Gamma_delta; Emory,totalSample,immune-Tr1; Emory,totalSample,immune-iTreg; Emory,totalSample,immune-CD8_naive; Emory,totalSample,immune-Cytotoxic; Emory,totalSample,immune-Exhausted; Homo_723,totalSample,immune-DC; Homo_723,totalSample,immune-Cytotoxic; Homo_723,totalSample,immune-CD8_T; Homo_723,totalSample,immune-Neutrophil; Homo_723,totalSample,immune-Th2; Homo_714,totalSample,immune-DC; Homo_714,totalSample,immune-Th2; Homo_714,totalSample,immune-Cytotoxic; Homo_714,totalSample,immune-Tr1; Homo_714,totalSample,immune-CD8_T; Homo_714,totalSample,immune-Th1; Homo_633,totalSample,immune-DC; Homo_633,totalSample,immune-Th2; Homo_633,totalSample,immune-NKT; Homo_633,totalSample,immune-Cytotoxic; Homo_633,totalSample,immune-CD8_T; Homo_633,totalSample,immune-Th1; MCSA,totalSample,immune-DC; MCSA,totalSample,immune-Monocyte; MCSA,totalSample,immune-Macrophage; MCSA,totalSample,immune-Neutrophil; MCSA,totalSample,immune-CD8_T; MCSA,totalSample,immune-NKT; MCSA,totalSample,immune-iTreg; MCSA,totalSample,immune-Th2; MCSA,totalSample,immune-Cytotoxic; MCSA,totalSample,immune-[illegible]
AKT1S1	Emory,control,male.vs.female; Homo_723,female,AD.vs.control; Homo_723,male,AD.vs.control; Homo_723,female,AD.vs.MCI; Homo_723,male,AD.vs.MCI; Homo_723,control,male.vs.female; Homo_714,female,AD.vs.control; Homo_714,male,AD.vs.control; Homo_714,female,AD.vs.MCI; Homo_714,male,AD.vs.MCI; Homo_714,control,male.vs.female; Homo_633,female,AD.vs.control; Homo_633,female,AD.vs.MCI; Homo_633,control,male.vs.female; ROSMAP,female,AD.vs.control; Emory,MCI,age; MCSA,totalSample,age; MCSA,MCI,age; Emory,totalSample,immune-Monocyte; Emory,totalSample,immune-Gamma_delta; Emory,totalSample,immune-Tr1; Emory,totalSample,immune-iTreg; Emory,totalSample,immune-CD8_naive; Homo_723,totalSample,immune-DC; Homo_723,totalSample,immune-Cytotoxic; Homo_723,totalSample,immune-Tr1; Homo_723,totalSample,immune-Th1; Homo_723,totalSample,immune-Bcell; Homo_723,totalSample,immune-NKT; Homo_723,totalSample,immune-Th2; Homo_723,totalSample,immune-iTreg; Homo_723,totalSample,immune-CD8_T; Homo_714,totalSample,immune-DC; Homo_714,totalSample,immune-NKT; Homo_714,totalSample,immune-Th2; Homo_714,totalSample,immune-Th1; Homo_714,totalSample,immune-Cytotoxic; Homo_714,totalSample,immune-Tr1; Homo_714,totalSample,immune-iTreg; Homo_714,totalSample,immune-CD4_T; Homo_714,totalSample,immune-CD4_naive; Homo_714,totalSample,immune-Bcell; Homo_633,totalSample,immune-DC; Homo_633,totalSample,immune-NKT; Homo_633,totalSample,immune-Th2; Homo_633,totalSample,immune-Th1; Homo_633,totalSample,immune-CD4_naive; Homo_633,totalSample,immune-CD4_T; Homo_633,totalSample,immune-Cytotoxic; Homo_633,totalSample,immune-Bcell; Homo_633,totalSample,immune-iTreg; Homo_633,totalSample,immune-Tr1; Homo_633,totalSample,immune-Tfh; MCSA,totalSample,immune-DC; MCSA,totalSample,immune-Monocyte; MCSA,totalSample,immune-Macrophage; MCSA,totalSample,immune-CD8_T; MCSA,totalSample,immune-Tr1; MCSA,totalSample,immune-iTreg; MCSA,totalSample,immune-Th2; MCSA,totalSample,immune-Cytotoxic; MCSA,totalSample,immune-Exhausted; MCSA,totalSample,immune-MAIT; MCSA,totalSample,immune-Effector_memory; MCSA,totalSample,immune-InfiltrationScore; ROSMAP,totalSample,immune-CD8_T; SRP223445,totalSample,immune-CD4_naive; SRP223445,totalSample,immune-Exhausted; SRP223445,totalSample,immune-[illegible]
HBA2	Emory,control,male.vs.female; Emory,MCI,age; MCSA,totalSample,age; MCSA,control,age; MCSA,MCI,age; Emory,totalSample,immune-Monocyte; Emory,totalSample,immune-Gamma_delta; Emory,totalSample,immune-Tr1; Emory,totalSample,immune-iTreg; Emory,totalSample,immune-CD8_naive; Homo_723,totalSample,immune-DC; Homo_723,totalSample,immune-Th1; Homo_723,totalSample,immune-Bcell; Homo_723,totalSample,immune-Th2; Homo_723,totalSample,immune-Cytotoxic; Homo_714,totalSample,immune-DC; Homo_714,totalSample,immune-Bcell; Homo_633,totalSample,immune-DC; Homo_633,totalSample,immune-Bcell; Homo_633,totalSample,immune-Th2; MCSA,totalSample,immune-DC; MCSA,totalSample,immune-Monocyte; MCSA,totalSample,immune-Macrophage; MCSA,totalSample,immune-CD8_T; MCSA,totalSample,immune-NKT; MCSA,totalSample,immune-iTreg; MCSA,totalSample,immune-Th2; MCSA,totalSample,immune-Cytotoxic; MCSA,totalSample,immune-Effector_memory
PPP2R5B	Emory,control,male.vs.female; Homo_723,male,AD.vs.control; Homo_723,male,AD.vs.MCI; Homo_723,AD,male.vs.female; Homo_714,female,AD.vs.control; Homo_714,male,AD.vs.control; Homo_714,male,AD.vs.MCI; Homo_714,female,MCI.vs.control; Homo_714,AD,male.vs.female; Homo_714,control,male.vs.female; Homo_633,male,MCI.vs.control; Homo_633,control,male.vs.female; Homo_633,MCI,male.vs.female; ROSMAP,female,AD.vs.control; ROSMAP,male,AD.vs.control; Emory,MCI,age; MCSA,totalSample,age; MCSA,control,age; MCSA,MCI,age; ROSMAP,AD,sample.age; Emory,totalSample,immune-Gamma_delta; Homo_723,totalSample,immune-DC; Homo_723,totalSample,immune-Cytotoxic; Homo_723,totalSample,immune-Th2; Homo_723,totalSample,immune-Neutrophil; Homo_714,totalSample,immune-DC; Homo_714,totalSample,immune-Th2; Homo_633,totalSample,immune-Th2; Homo_633,totalSample,immune-DC; Homo_633,totalSample,immune-Monocyte; Homo_633,totalSample,immune-Bcell; MCSA,totalSample,immune-DC; MCSA,totalSample,immune-Monocyte; MCSA,totalSample,immune-Macrophage; MCSA,totalSample,immune-CD8_T; MCSA,totalSample,immune-NKT; MCSA,totalSample,immune-Th2; MCSA,totalSample,immune-Cytotoxic; MCSA,totalSample,immune-Effector_memory; MCSA,totalSample,immune-InfiltrationScore; SRP223445,totalSample,immune-CD4_naive;
UQCR11	Emory,control,male.vs.female; Homo_714,female,AD.vs.control; ROSMAP,female,AD.vs.control; Emory,MCI,age; MCSA,MCI,age; Emory,totalSample,immune-DC; Emory,totalSample,immune-Monocyte; Emory,totalSample,immune-CD8_T; Emory,totalSample,immune-NKT; Emory,totalSample,immune-Gamma_delta; Emory,totalSample,immune-CD4_naive; Emory,totalSample,immune-Th1; Emory,totalSample,immune-Th17; Emory,totalSample,immune-Tfh; Emory,totalSample,immune-Cytotoxic; Homo_723,totalSample,immune-Cytotoxic; Homo_723,totalSample,immune-Neutrophil; Homo_723,totalSample,immune-CD8_T; Homo_723,totalSample,immune-Th2; Homo_723,totalSample,immune-Gamma_delta; Homo_723,totalSample,immune-NKT; Homo_723,totalSample,immune-Exhausted; Homo_723,totalSample,immune-iTreg; Homo_714,totalSample,immune-Cytotoxic; Homo_714,totalSample,immune-Th2; Homo_714,totalSample,immune-Exhausted; Homo_714,totalSample,immune-NKT; Homo_714,totalSample,immune-CD8_T; Homo_714,totalSample,immune-Neutrophil; Homo_714,totalSample,immune-iTreg; Homo_714,totalSample,immune-Th17; Homo_714,totalSample,immune-DC; Homo_714,totalSample,immune-Gamma_delta; Homo_633,totalSample,immune-Th2; Homo_633,totalSample,immune-Cytotoxic; Homo_633,totalSample,immune-NKT; Homo_633,totalSample,immune-CD8_T; Homo_633,totalSample,immune-Neutrophil; Homo_633,totalSample,immune-DC; Homo_633,totalSample,immune-Th17; Homo_633,totalSample,immune-Gamma_delta; Homo_633,totalSample,immune-CD4_T; Homo_633,totalSample,immune-iTreg; Homo_633,totalSample,immune-Exhausted; MCSA,totalSample,immune-DC; MCSA,totalSample,immune-Monocyte; MCSA,totalSample,immune-Neutrophil; MCSA,totalSample,immune-CD8_T; MCSA,totalSample,immune-NKT; MCSA,totalSample,immune-Tr1; MCSA,totalSample,immune-iTreg; MCSA,totalSample,immune-Th2; MCSA,totalSample,immune-Th17; MCSA,totalSample,immune-Cytotoxic; MCSA,totalSample,immune-Exhausted; MCSA,totalSample,immune-Effector_memory; ROSMAP,totalSample,immune-[illegible]
SLC25A37	Emory,control,male.vs.female; Emory,totalSample,age; Emory,MCI,age; ROSMAP,MCI,last.visit.age; Emory,totalSample,immune-Gamma_delta; Emory,totalSample,immune-Tr1; Emory,totalSample,immune-iTreg; Emory,totalSample,immune-CD8_naive; Homo_723,totalSample,immune-Bcell; Homo_714,totalSample,immune-Bcell; Homo_633,totalSample,immune-Bcell; Homo_633,totalSample,immune-Monocyte; MCSA,totalSample,immune-Macrophage; MCSA,totalSample,immune-NK; MCSA,totalSample,immune-MAIT; ROSMAP,totalSample,immune-Effector_memory; SRP223445,totalSample,immune-CD4_naive; ROSMAP,male and female-excludeAge90+,survival
ATP5F1E	Emory,control,male.vs.female; Homo_723,female,AD.vs.control; Homo_723,female,AD.vs.MCI; Homo_723,control,male.vs.female; Homo_714,female,AD.vs.control; Homo_714,male,AD.vs.control; Homo_714,female,AD.vs.MCI; Homo_714,male,MCI.vs.control; Homo_714,control,male.vs.female; Homo_714,MCI,male.vs.female; Homo_633,male,MCI.vs.control; Homo_633,control,male.vs.female; Homo_633,MCI,male.vs.female; ROSMAP,female,AD.vs.control; Emory,totalSample,age; Emory,MCI,age; MCSA,totalSample,age; MCSA,control,age; Emory,totalSample,immune-DC; Emory,totalSample,immune-Monocyte; Emory,totalSample,immune-CD8_T; Emory,totalSample,immune-Gamma_delta; Emory,totalSample,immune-nTreg; Emory,totalSample,immune-Th17; Emory,totalSample,immune-Tfh; Emory,totalSample,immune-CD8_naive; Emory,totalSample,immune-Cytotoxic; Homo_723,totalSample,immune-iTreg; Homo_723,totalSample,immune-CD4_T; Homo_723,totalSample,immune-Th2; Homo_723,totalSample,immune-nTreg; Homo_723,totalSample,immune-CD4_naive; Homo_723,totalSample,immune-Effector_memory; Homo_723,totalSample,immune-Tr1; Homo_723,totalSample,immune-Tfh; Homo_723,totalSample,immune-Monocyte; Homo_723,totalSample,immune-Th17; Homo_723,totalSample,immune-Central_memory; Homo_714,totalSample,immune-iTreg; Homo_714,totalSample,immune-CD4_T; Homo_714,totalSample,immune-Th2; Homo_714,totalSample,immune-nTreg; Homo_714,totalSample,immune-Effector_memory; Homo_714,totalSample,immune-CD4_naive; Homo_714,totalSample,immune-Tfh; Homo_714,totalSample,immune-Tr1; Homo_714,totalSample,immune-Central_memory; Homo_714,totalSample,immune-Monocyte; Homo_714,totalSample,immune-Th17; Homo_714,totalSample,immune-NK; Homo_633,totalSample,immune-Effector_memory; Homo_633,totalSample,immune-CD4_T; Homo_633,totalSample,immune-nTreg; Homo_633,totalSample,immune-iTreg; Homo_633,totalSample,immune-Th2; Homo_633,totalSample,immune-Central_memory; Homo_633,totalSample,immune-CD4_naive; Homo_633,totalSample,immune-Th17; Homo_633,totalSample,immune-Tr1; Homo_633,totalSample,immune-Cytotoxic; Homo_633,totalSample,immune-Tfh; MCSA,totalSample,immune-DC; MCSA,totalSample,immune-Monocyte; MCSA,totalSample,immune-CD4_T; MCSA,totalSample,immune-Gamma_delta; MCSA,totalSample,immune-CD4_naive; MCSA,totalSample,immune-Tr1; MCSA,totalSample,immune-nTreg; MCSA,totalSample,immune-iTreg; MCSA,totalSample,immune-Th2; MCSA,totalSample,immune-Th17; MCSA,totalSample,immune-Tfh; MCSA,totalSample,immune-Exhausted; MCSA,totalSample,immune-Central_memory; MCSA,totalSample,immune-Effector_memory; MCSA,totalSample,immune-[illegible]
FOXO3B	Emory,control,male.vs.female; Homo_723,male,AD.vs.control; Homo_723,male,AD.vs.MCI; Homo_723,control,male.vs.female; Homo_714,male,AD.vs.control; Homo_714,male,AD.vs.MCI; Homo_714,control,male.vs.female; Homo_633,control,male.vs.female; ROSMAP,male,AD.vs.control; Emory,totalSample,immune-Th1; Homo_723,totalSample,immune-Tfh; Homo_723,totalSample,immune-iTreg; Homo_723,totalSample,immune-Central_memory; Homo_723,totalSample,immune-CD4_naive; Homo_723,totalSample,immune-CD4_T; Homo_723,totalSample,immune-Gamma_delta; Homo_723,totalSample,immune-InfiltrationScore; Homo_723,totalSample,immune-Effector_memory; Homo_723,totalSample,immune-NKT; Homo_723,totalSample,immune-Tr1; Homo_723,totalSample,immune-Th2; Homo_723,totalSample,immune-nTreg; Homo_714,totalSample,immune-Tfh; Homo_714,totalSample,immune-Central_memory; Homo_714,totalSample,immune-CD4_T; Homo_714,totalSample,immune-CD4_naive; Homo_714,totalSample,immune-Effector_memory; Homo_714,totalSample,immune-iTreg; Homo_714,totalSample,immune-Tr1; Homo_714,totalSample,immune-NKT; Homo_714,totalSample,immune-InfiltrationScore; Homo_714,totalSample,immune-Gamma_delta; Homo_714,totalSample,immune-nTreg; Homo_714,totalSample,immune-Th2; Homo_633,totalSample,immune-Tfh; Homo_633,totalSample,immune-Central_memory; Homo_633,totalSample,immune-CD4_naive; Homo_633,totalSample,immune-CD4_T; Homo_633,totalSample,immune-Effector_memory; Homo_633,totalSample,immune-iTreg; Homo_633,totalSample,immune-Tr1; Homo_633,totalSample,immune-NKT; Homo_633,totalSample,immune-InfiltrationScore; Homo_633,totalSample,immune-Gamma_delta; Homo_633,totalSample,immune-nTreg; MCSA,totalSample,immune-CD4_T; MCSA,totalSample,immune-NKT; MCSA,totalSample,immune-Gamma_delta; MCSA,totalSample,immune-CD4_naive; MCSA,totalSample,immune-Tr1; MCSA,totalSample,immune-nTreg; MCSA,totalSample,immune-iTreg; MCSA,totalSample,immune-Th1; MCSA,totalSample,immune-Th17; MCSA,totalSample,immune-Tfh; MCSA,totalSample,immune-CD8_naive; MCSA,totalSample,immune-Cytotoxic; MCSA,totalSample,immune-Exhausted; MCSA,totalSample,immune-MAIT; MCSA,totalSample,immune-Central_memory; MCSA,totalSample,immune-Effector_memory; ROSMAP,totalSample,immune-Macrophage; ROSMAP,totalSample,immune-Cytotoxic; [illegible]
SLC66A2	Emory,control,male.vs.female; Homo_723,female,AD.vs.control; Homo_723,male,AD.vs.control; Homo_723,female,AD.vs.MCI; Homo_723,male,AD.vs.MCI; Homo_714,female,AD.vs.control; Homo_714,male,AD.vs.control; Homo_714,female,AD.vs.MCI; Homo_714,male,AD.vs.MCI; Homo_633,female,AD.vs.control; Homo_633,female,AD.vs.MCI; Homo_633,male,MCI.vs.control; ROSMAP,female,AD.vs.control; ACOM,AD,age; MCSA,MCI,age; ROSMAP,AD,MMSE; Emory,totalSample,immune-Tr1; Emory,totalSample,immune-iTreg; Emory,totalSample,immune-InfiltrationScore; Homo_723,totalSample,immune-DC; Homo_723,totalSample,immune-Cytotoxic; Homo_723,totalSample,immune-Th2; Homo_723,totalSample,immune-Tr1; Homo_723,totalSample,immune-CD8_T; Homo_723,totalSample,immune-iTreg; Homo_723,totalSample,immune-Th1; Homo_723,totalSample,immune-Neutrophil; Homo_714,totalSample,immune-DC; Homo_714,totalSample,immune-Th2; Homo_714,totalSample,immune-Cytotoxic; Homo_714,totalSample,immune-Th1; Homo_714,totalSample,immune-Tr1; Homo_714,totalSample,immune-CD4_T; Homo_633,totalSample,immune-DC; Homo_633,totalSample,immune-Th2; Homo_633,totalSample,immune-NKT; Homo_633,totalSample,immune-Th1; Homo_633,totalSample,immune-Cytotoxic; Homo_633,totalSample,immune-CD8_T; MCSA,totalSample,immune-DC; MCSA,totalSample,immune-Monocyte; MCSA,totalSample,immune-Macrophage; MCSA,totalSample,immune-Neutrophil; MCSA,totalSample,immune-CD8_T; MCSA,totalSample,immune-NKT; MCSA,totalSample,immune-iTreg; MCSA,totalSample,immune-Th2; MCSA,totalSample,immune-Cytotoxic; MCSA,totalSample,immune-Effector_memory;
OAZ1	Emory,control,male.vs.female; Homo_723,female,AD.vs.control; Homo_723,control,male.vs.female; Homo_714,female,AD.vs.control; Homo_714,control,male.vs.female; Homo_633,control,male.vs.female; ROSMAP,female,AD.vs.control; ROSMAP,female,MCI.vs.control; Emory,MCI,age; MCSA,MCI,age; ROSMAP,MCI,last.visit.age; Emory,totalSample,immune-CD4_T; Emory,totalSample,immune-Gamma_delta; Emory,totalSample,immune-Tr1; Emory,totalSample,immune-iTreg; Emory,totalSample,immune-Cytotoxic; Homo_723,totalSample,immune-DC; Homo_723,totalSample,immune-iTreg; Homo_723,totalSample,immune-Th2; Homo_723,totalSample,immune-Tr1; Homo_723,totalSample,immune-CD4_naive; Homo_723,totalSample,immune-CD4_T; Homo_723,totalSample,immune-Cytotoxic; Homo_723,totalSample,immune-Bcell; Homo_723,totalSample,immune-Th1; Homo_723,totalSample,immune-Tfh; Homo_714,totalSample,immune-DC; Homo_714,totalSample,immune-iTreg; Homo_714,totalSample,immune-CD4_naive; Homo_714,totalSample,immune-Th2; Homo_714,totalSample,immune-CD4_T; Homo_714,totalSample,immune-Th1; Homo_714,totalSample,immune-Bcell; Homo_714,totalSample,immune-Tr1; Homo_714,totalSample,immune-NKT; Homo_714,totalSample,immune-Tfh; Homo_714,totalSample,immune-Cytotoxic; Homo_633,totalSample,immune-DC; Homo_633,totalSample,immune-Th2; Homo_633,totalSample,immune-NKT; Homo_633,totalSample,immune-CD4_T; Homo_633,totalSample,immune-CD4_naive; Homo_633,totalSample,immune-Bcell; Homo_633,totalSample,immune-Th1; Homo_633,totalSample,immune-iTreg; Homo_633,totalSample,immune-Tfh; Homo_633,totalSample,immune-Cytotoxic; Homo_633,totalSample,immune-Tr1; MCSA,totalSample,immune-DC; MCSA,totalSample,immune-Monocyte; MCSA,totalSample,immune-Macrophage; MCSA,totalSample,immune-CD4_T; MCSA,totalSample,immune-CD8_T; MCSA,totalSample,immune-NKT; MCSA,totalSample,immune-CD4_naive; MCSA,totalSample,immune-Tr1; MCSA,totalSample,immune-nTreg; MCSA,totalSample,immune-iTreg; MCSA,totalSample,immune-Th2; MCSA,totalSample,immune-Th17; MCSA,totalSample,immune-Cytotoxic; MCSA,totalSample,immune-Effector_memory; SRP223445,totalSample,immune-CD4_naive; SRP223445,totalSample,immune-Exhausted
ZDHHC2	Emory,control,male.vs.female; ROSMAP,female,AD.vs.control; MCSA,MCI,age; ROSMAP,MCI,last.visit.age; ROSMAP,MCI,sample.age; Emory,totalSample,immune-DC; Emory,totalSample,immune-Monocyte; Emory,totalSample,immune-Th17; Homo_723,totalSample,immune-DC; Homo_723,totalSample,immune-Cytotoxic; Homo_723,totalSample,immune-Tr1; Homo_723,totalSample,immune-iTreg; Homo_723,totalSample,immune-Monocyte; Homo_723,totalSample,immune-Th1; Homo_714,totalSample,immune-DC; Homo_714,totalSample,immune-Monocyte; Homo_714,totalSample,immune-Th1; Homo_633,totalSample,immune-DC; Homo_633,totalSample,immune-Monocyte; Homo_633,totalSample,immune-NKT; Homo_633,totalSample,immune-Th1; Homo_633,totalSample,immune-Tfh; MCSA,totalSample,immune-DC; MCSA,totalSample,immune-Monocyte; MCSA,totalSample,immune-CD4_T; MCSA,totalSample,immune-CD8_T; MCSA,totalSample,immune-CD4_naive; MCSA,totalSample,immune-Tr1; MCSA,totalSample,immune-nTreg; MCSA,totalSample,immune-iTreg; MCSA,totalSample,immune-Tfh; MCSA,totalSample,immune-CD8_naive; MCSA,totalSample,immune-Cytotoxic; MCSA,totalSample,immune-Exhausted; MCSA,totalSample,immune-MAIT; SRP223445,totalSample,immune-NK; SRP223445,totalSample,immune-CD4_naive; SRP223445,totalSample,immune-Exhausted; SRP223445,totalSample,immune-InfiltrationScore
HBA1	Emory,control,male.vs.female; Homo_723,female,AD.vs.control; Homo_723,female,AD.vs.MCI; Emory,MCI,age; MCSA,MCI,age; Emory,totalSample,immune-Monocyte; Emory,totalSample,immune-Gamma_delta; Emory,totalSample,immune-Tr1; Emory,totalSample,immune-iTreg; Emory,totalSample,immune-CD8_naive; Homo_723,totalSample,immune-DC; Homo_723,totalSample,immune-Th1; Homo_723,totalSample,immune-Bcell; Homo_714,totalSample,immune-DC; Homo_714,totalSample,immune-Bcell; Homo_633,totalSample,immune-DC; Homo_633,totalSample,immune-Bcell; Homo_633,totalSample,immune-Th2; MCSA,totalSample,immune-DC; MCSA,totalSample,immune-Monocyte; MCSA,totalSample,immune-Macrophage; MCSA,totalSample,immune-CD8_T; MCSA,totalSample,immune-NKT; MCSA,totalSample,immune-iTreg; MCSA,totalSample,immune-Th2; MCSA,totalSample,immune-Cytotoxic; MCSA,totalSample,immune-Effector_memory
ZER1	Emory,control,male.vs.female; Homo_723,female,AD.vs.MCI; Homo_723,male,AD.vs.MCI; Homo_723,female,MCI.vs.control; Homo_723,male,MCI.vs.control; Homo_714,female,AD.vs.MCI; Homo_714,male,AD.vs.MCI; Homo_714,female,MCI.vs.control; Homo_714,male,MCI.vs.control; Homo_633,female,AD.vs.control; Homo_633,female,AD.vs.MCI; Homo_633,female,MCI.vs.control; Homo_633,male,MCI.vs.control; ROSMAP,female,AD.vs.control; ROSMAP,female,AD.vs.MCI; Emory,MCI,age; MCSA,MCI,age; Emory,totalSample,immune-Gamma_delta; Homo_723,totalSample,immune-DC; Homo_723,totalSample,immune-Monocyte; Homo_723,totalSample,immune-Exhausted; Homo_723,totalSample,immune-Th2; Homo_714,totalSample,immune-Th2; Homo_714,totalSample,immune-DC; Homo_714,totalSample,immune-Monocyte; Homo_633,totalSample,immune-Th2; Homo_633,totalSample,immune-DC; Homo_633,totalSample,immune-Monocyte; MCSA,totalSample,immune-DC; MCSA,totalSample,immune-Monocyte; MCSA,totalSample,immune-Macrophage; MCSA,totalSample,immune-CD8_T; MCSA,totalSample,immune-Tr1; MCSA,totalSample,immune-iTreg; MCSA,totalSample,immune-Th2; MCSA,totalSample,immune-Cytotoxic; MCSA,totalSample,immune-MAIT; ROSMAP,totalSample,immune-Effector_memory; SRP223445,totalSample,immune-InfiltrationScore
MED25	Emory,control,male.vs.female; ROSMAP,female,AD.vs.control; MCSA,totalSample,age; MCSA,control,age; Emory,totalSample,immune-CD4_T; Emory,totalSample,immune-Tr1; Emory,totalSample,immune-iTreg; Emory,totalSample,immune-Th1; Emory,totalSample,immune-InfiltrationScore; Homo_723,totalSample,immune-Th17; Homo_714,totalSample,immune-Th17; Homo_633,totalSample,immune-Th17; MCSA,totalSample,immune-Neutrophil; MCSA,totalSample,immune-CD8_T; MCSA,totalSample,immune-Gamma_delta; MCSA,totalSample,immune-Th17; MCSA,totalSample,immune-Cytotoxic; MCSA,totalSample,immune-Exhausted; ROSMAP,totalSample,immune-Effector_memory; SRP223445,totalSample,immune-CD4_naive;
TFDP1	Emory,control,male.vs.female; Homo_723,male,AD.vs.control; Homo_723,male,MCI.vs.control; Homo_723,control,male.vs.female; Homo_714,female,AD.vs.control; Homo_714,male,AD.vs.control; Homo_714,male,MCI.vs.control; Homo_714,control,male.vs.female; Homo_633,female,AD.vs.control; Homo_633,female,AD.vs.MCI; Homo_633,male,MCI.vs.control; Homo_633,control,male.vs.female; Emory,MCI,age; Homo_723,totalSample,immune-Bcell; Homo_714,totalSample,immune-Bcell; Homo_633,totalSample,immune-Bcell; Homo_633,totalSample,immune-Gamma_delta; MCSA,totalSample,immune-CD4_T; MCSA,totalSample,immune-CD8_T; MCSA,totalSample,immune-NKT; MCSA,totalSample,immune-Gamma_delta; MCSA,totalSample,immune-CD4_naive; MCSA,totalSample,immune-Tr1; MCSA,totalSample,immune-nTreg; MCSA,totalSample,immune-iTreg; MCSA,totalSample,immune-Th17; MCSA,totalSample,immune-Tfh; MCSA,totalSample,immune-CD8_naive; MCSA,totalSample,immune-Cytotoxic; MCSA,totalSample,immune-Exhausted; SRP223445,totalSample,immune-[illegible]
NLRP2	Emory,control,male.vs.female; Homo_723,control,male.vs.female; Homo_714,control,male.vs.female; ACOM,totalSample,age; Emory,totalSample,immune-Effector_memory; Homo_723,totalSample,immune-InfiltrationScore; Homo_723,totalSample,immune-CD4_naive; Homo_723,totalSample,immune-Effector_memory; Homo_723,totalSample,immune-Tfh; Homo_714,totalSample,immune-InfiltrationScore; Homo_714,totalSample,immune-Effector_memory; Homo_714,totalSample,immune-Tfh; Homo_714,totalSample,immune-CD4_naive; Homo_633,totalSample,immune-InfiltrationScore; Homo_633,totalSample,immune-Effector_memory; Homo_633,totalSample,immune-Tfh; Homo_633,totalSample,immune-CD4_naive; MCSA,totalSample,immune-Tr1; MCSA,totalSample,immune-nTreg; MCSA,totalSample,immune-Tfh; SRP223445,totalSample,immune-nTreg; SRP223445,totalSample,immune-InfiltrationScore
TMEM176B	Emory,control,male.vs.female; Homo_723,female,AD.vs.control; Homo_723,male,AD.vs.MCI; Homo_723,female,MCI.vs.control; Homo_723,male,MCI.vs.control; Homo_723,control,male.vs.female; Homo_723,MCI,male.vs.female; Homo_714,female,AD.vs.control; Homo_714,male,AD.vs.control; Homo_714,male,AD.vs.MCI; Homo_714,female,MCI.vs.control; Homo_714,male,MCI.vs.control; Homo_714,MCI,male.vs.female; Homo_633,male,MCI.vs.control; Homo_633,MCI,male.vs.female; ROSMAP,female,AD.vs.control; ROSMAP,control,male.vs.female; ACOM,totalSample,age; Emory,MCI,age; Emory,totalSample,immune-CD8_naive; Homo_723,totalSample,immune-DC; Homo_714,totalSample,immune-Th2

BAG6	Emory,control,male.vs.female; ROSMAP,female,AD.vs.control; ROSMAP,male,AD.vs.control; MCSA,totalSample,age; MCSA,MCI,age; Emory,totalSample,immune-Tr1; Emory,totalSample,immune-iTreg; Homo_723,totalSample,immune-DC; Homo_723,totalSample,immune-Bcell; Homo_714,totalSample,immune-DC; Homo_714,totalSample,immune-Bcell; Homo_633,totalSample,immune-DC; Homo_633,totalSample,immune-Bcell; Homo_633,totalSample,immune-Th1; MCSA,totalSample,immune-DC; MCSA,totalSample,immune-Monocyte; MCSA,totalSample,immune-Macrophage; MCSA,totalSample,immune-CD8_T; MCSA,totalSample,immune-Tr1; MCSA,totalSample,immune-iTreg; MCSA,totalSample,immune-Th2; MCSA,totalSample,immune-Cytotoxic; MCSA,totalSample,immune-Exhausted; MCSA,totalSample,immune-MAIT; MCSA,totalSample,immune-InfiltrationScore; ROSMAP,totalSample,immune-Effector_memory; SRP223445,totalSample,immune-Neutrophil; SRP223445,totalSample,immune-CD4_naive; SRP223445,totalSample,immune-InfiltrationScore
SLC38A5	Emory,control,male.vs.female; Homo_723,male,AD.vs.control; Homo_723,male,AD.vs.MCI; Homo_714,male,AD.vs.control; Homo_714,male,AD.vs.MCI; ROSMAP,female,AD.vs.control; ROSMAP,male,AD.vs.MCI; ROSMAP,female,MCI.vs.control; ROSMAP,male,MCI.vs.control; Emory,MCI,age; MCSA,totalSample,age; MCSA,MCI,age; Emory,totalSample,immune-Monocyte; Emory,totalSample,immune-Gamma_delta; Emory,totalSample,immune-Tr1; Homo_723,totalSample,immune-DC; Homo_723,totalSample,immune-Cytotoxic; Homo_723,totalSample,immune-CD8_T; Homo_723,totalSample,immune-Exhausted; Homo_723,totalSample,immune-Bcell; Homo_723,totalSample,immune-Neutrophil; Homo_723,totalSample,immune-Th1; Homo_714,totalSample,immune-DC; Homo_714,totalSample,immune-Cytotoxic; Homo_714,totalSample,immune-CD8_T; Homo_714,totalSample,immune-Th1; Homo_633,totalSample,immune-DC; Homo_633,totalSample,immune-NKT; Homo_633,totalSample,immune-Th2; Homo_633,totalSample,immune-Th1; Homo_633,totalSample,immune-Cytotoxic; Homo_633,totalSample,immune-CD8_T; Homo_633,totalSample,immune-Bcell; MCSA,totalSample,immune-DC; MCSA,totalSample,immune-Monocyte; MCSA,totalSample,immune-Macrophage; MCSA,totalSample,immune-Neutrophil; MCSA,totalSample,immune-CD8_T; MCSA,totalSample,immune-iTreg; MCSA,totalSample,immune-Th2; MCSA,totalSample,immune-Cytotoxic; MCSA,totalSample,immune-Exhausted; MCSA,totalSample,immune-Effector_memory; MCSA,totalSample,immune-InfiltrationScore; SRP223445,totalSample,immune-Neutrophil
KLC3	Emory,control,male.vs.female; Homo_723,female,AD.vs.control; Homo_723,male,AD.vs.control; Homo_723,female,AD.vs.MCI; Homo_723,male,AD.vs.MCI; Homo_723,female,MCI.vs.control; Homo_714,female,AD.vs.control; Homo_714,male,AD.vs.control; Homo_714,female,AD.vs.MCI; Homo_714,male,AD.vs.MCI; Homo_714,female,MCI.vs.control; Homo_633,female,AD.vs.control; Homo_633,female,AD.vs.MCI; Homo_633,female,MCI.vs.control; ROSMAP,male,AD.vs.control; ROSMAP,female,AD.vs.MCI; Emory,totalSample,age; Emory,MCI,age; MCSA,MCI,age; Emory,totalSample,immune-Gamma_delta; Emory,totalSample,immune-CD8_naive; Homo_723,totalSample,immune-Exhausted; Homo_723,totalSample,immune-CD8_T; Homo_723,totalSample,immune-Cytotoxic; Homo_723,totalSample,immune-Th17; Homo_723,totalSample,immune-CD8_naive; Homo_714,totalSample,immune-DC; Homo_714,totalSample,immune-Th17; Homo_633,totalSample,immune-DC; Homo_633,totalSample,immune-Th2; Homo_633,totalSample,immune-Bcell; MCSA,totalSample,immune-DC; MCSA,totalSample,immune-Monocyte; MCSA,totalSample,immune-Macrophage; MCSA,totalSample,immune-Neutrophil; MCSA,totalSample,immune-CD4_T; MCSA,totalSample,immune-CD8_T; MCSA,totalSample,immune-NKT; MCSA,totalSample,immune-Tr1; MCSA,totalSample,immune-iTreg; MCSA,totalSample,immune-Th2;
DPM2	Emory,control,male.vs.female; Homo_723,female,AD.vs.MCI; Homo_714,female,AD.vs.MCI; Homo_714,female,MCI.vs.control; Homo_633,female,MCI.vs.control; Emory,MCI,age; MCSA,MCI,age; ROSMAP,MCI,last.visit.age; ROSMAP,MCI,sample.age; Emory,totalSample,immune-Gamma_delta; Emory,totalSample,immune-Tr1; Emory,totalSample,immune-iTreg; Emory,totalSample,immune-CD8_naive; Emory,totalSample,immune-Cytotoxic; Homo_723,totalSample,immune-Bcell; Homo_723,totalSample,immune-DC; Homo_723,totalSample,immune-Cytotoxic; Homo_723,totalSample,immune-Neutrophil; Homo_723,totalSample,immune-Tr1; Homo_723,totalSample,immune-Th2; Homo_714,totalSample,immune-Bcell; Homo_714,totalSample,immune-DC; Homo_714,totalSample,immune-Cytotoxic; Homo_714,totalSample,immune-Th2; Homo_714,totalSample,immune-CD4_naive; Homo_714,totalSample,immune-iTreg; Homo_714,totalSample,immune-CD4_T; Homo_714,totalSample,immune-Tr1; Homo_714,totalSample,immune-Th1; Homo_714,totalSample,immune-NKT; Homo_633,totalSample,immune-DC; Homo_633,totalSample,immune-Bcell; Homo_633,totalSample,immune-NKT; Homo_633,totalSample,immune-Th2; Homo_633,totalSample,immune-CD4_naive; Homo_633,totalSample,immune-CD4_T; Homo_633,totalSample,immune-Th1; Homo_633,totalSample,immune-iTreg; Homo_633,totalSample,immune-Cytotoxic; MCSA,totalSample,immune-DC; MCSA,totalSample,immune-Monocyte; MCSA,totalSample,immune-Macrophage; MCSA,totalSample,immune-Neutrophil; MCSA,totalSample,immune-CD4_T; MCSA,totalSample,immune-CD8_T; MCSA,totalSample,immune-Tr1; MCSA,totalSample,immune-iTreg; MCSA,totalSample,immune-Th2; MCSA,totalSample,immune-Cytotoxic; MCSA,totalSample,immune-Effector_memory; ROSMAP,totalSample,immune-Cytotoxic; ROSMAP,totalSample,immune-Central_memory; ROSMAP,totalSample,immune-Effector_memory; SRP223445,totalSample,immune-CD4_naive
UBXN6	Emory,control,male.vs.female; Emory,MCI,age; MCSA,MCI,age; ROSMAP,AD,last.visit.age; Emory,totalSample,immune-Gamma_delta; Emory,totalSample,immune-Tr1; Emory,totalSample,immune-iTreg; Emory,totalSample,immune-CD8_naive; Emory,totalSample,immune-Cytotoxic; Homo_723,totalSample,immune-Bcell; Homo_723,totalSample,immune-DC; Homo_723,totalSample,immune-Cytotoxic; Homo_723,totalSample,immune-Th2; Homo_714,totalSample,immune-DC; Homo_714,totalSample,immune-Bcell; Homo_714,totalSample,immune-Th2; Homo_714,totalSample,immune-Th1; Homo_633,totalSample,immune-DC; Homo_633,totalSample,immune-Bcell; Homo_633,totalSample,immune-Th2; Homo_633,totalSample,immune-Th1; Homo_633,totalSample,immune-NKT; Homo_633,totalSample,immune-CD4_naive; Homo_633,totalSample,immune-CD4_T; MCSA,totalSample,immune-DC; MCSA,totalSample,immune-Monocyte; MCSA,totalSample,immune-Macrophage; MCSA,totalSample,immune-Neutrophil; MCSA,totalSample,immune-CD4_T; MCSA,totalSample,immune-CD8_T; MCSA,totalSample,immune-NKT; MCSA,totalSample,immune-Tr1; MCSA,totalSample,immune-iTreg; MCSA,totalSample,immune-Th2; MCSA,totalSample,immune-Cytotoxic; MCSA,totalSample,immune-Effector_memory
JAZF1	Emory,control,male.vs.female; Homo_714,control,male.vs.female; Homo_633,control,male.vs.female; MCSA,totalSample,age; MCSA,control,age; MCSA,MCI,age; ROSMAP,MCI,last.visit.age; Emory,totalSample,immune-Gamma_delta; Emory,totalSample,immune-Th17; Emory,totalSample,immune-Cytotoxic; Homo_723,totalSample,immune-Bcell; Homo_714,totalSample,immune-Monocyte; Homo_714,totalSample,immune-nTreg; Homo_633,totalSample,immune-nTreg; Homo_633,totalSample,immune-Monocyte; MCSA,totalSample,immune-DC; MCSA,totalSample,immune-Monocyte; MCSA,totalSample,immune-CD8_T; MCSA,totalSample,immune-Th2; MCSA,totalSample,immune-Cytotoxic; MCSA,totalSample,immune-Central_memory; MCSA,totalSample,immune-Effector_memory; ROSMAP,totalSample,immune-Effector_memory; SRP223445,totalSample,immune-CD4_naive; SRP223445,totalSample,immune-Th17; SRP223445,totalSample,immune-InfiltrationScore; ROSMAP,male-excludeAge90+,survival
SLC7A5	Emory,control,male.vs.female; ROSMAP,female,AD.vs.control; ROSMAP,male,AD.vs.control; ACOM,AD,age; Emory,totalSample,age; Emory,MCI,age; MCSA,MCI,age; ROSMAP,AD,last.visit.age; Emory,totalSample,immune-Neutrophil; Emory,totalSample,immune-Gamma_delta; Emory,totalSample,immune-CD8_naive; Emory,totalSample,immune-Cytotoxic; Homo_723,totalSample,immune-DC; Homo_723,totalSample,immune-Cytotoxic; Homo_723,totalSample,immune-Th1; Homo_723,totalSample,immune-Bcell; Homo_723,totalSample,immune-CD8_T; Homo_714,totalSample,immune-DC; Homo_714,totalSample,immune-Th1; Homo_714,totalSample,immune-Cytotoxic; Homo_714,totalSample,immune-Bcell; Homo_633,totalSample,immune-DC; Homo_633,totalSample,immune-Th1; Homo_633,totalSample,immune-NKT; Homo_633,totalSample,immune-Bcell; Homo_633,totalSample,immune-Th2; MCSA,totalSample,immune-DC; MCSA,totalSample,immune-Monocyte; MCSA,totalSample,immune-Macrophage; MCSA,totalSample,immune-CD8_T; MCSA,totalSample,immune-iTreg; MCSA,totalSample,immune-Th2; MCSA,totalSample,immune-Cytotoxic; MCSA,totalSample,immune-Exhausted; MCSA,totalSample,immune-Effector_memory; MCSA,totalSample,immune-InfiltrationScore; ROSMAP,totalSample,immune-Macrophage; ROSMAP,totalSample,immune-Cytotoxic; ROSMAP,totalSample,immune-Central_memory;
ELOF1	Emory,control,male.vs.female; Homo_723,female,AD.vs.MCI; Homo_723,female,MCI.vs.control; Homo_714,female,AD.vs.control; Homo_714,male,AD.vs.control; Homo_714,female,AD.vs.MCI; Homo_714,male,AD.vs.MCI; Homo_714,female,MCI.vs.control; Homo_633,female,MCI.vs.control; Emory,totalSample,age; Emory,MCI,age; MCSA,MCI,age; ROSMAP,AD,last.visit.age; ROSMAP,MCI,last.visit.age; ROSMAP,MCI,sample.age; Emory,totalSample,immune-Monocyte; Emory,totalSample,immune-Neutrophil; Emory,totalSample,immune-CD8_T; Emory,totalSample,immune-Gamma_delta; Emory,totalSample,immune-iTreg; Emory,totalSample,immune-Th17; Emory,totalSample,immune-CD8_naive; Emory,totalSample,immune-Cytotoxic; Emory,totalSample,immune-Exhausted; Homo_723,totalSample,immune-DC; Homo_723,totalSample,immune-Cytotoxic; Homo_723,totalSample,immune-Bcell; Homo_723,totalSample,immune-Neutrophil; Homo_723,totalSample,immune-Th2; Homo_723,totalSample,immune-Tr1; Homo_723,totalSample,immune-NKT; Homo_723,totalSample,immune-iTreg; Homo_723,totalSample,immune-CD8_T; Homo_714,totalSample,immune-DC; Homo_714,totalSample,immune-Cytotoxic; Homo_714,totalSample,immune-Th2; Homo_714,totalSample,immune-Neutrophil; Homo_714,totalSample,immune-NKT; Homo_714,totalSample,immune-Th1; Homo_714,totalSample,immune-CD8_T; Homo_714,totalSample,immune-iTreg; Homo_714,totalSample,immune-CD4_T; Homo_714,totalSample,immune-Bcell; Homo_633,totalSample,immune-DC; Homo_633,totalSample,immune-NKT; Homo_633,totalSample,immune-Th2; Homo_633,totalSample,immune-Cytotoxic; Homo_633,totalSample,immune-Th1; Homo_633,totalSample,immune-Bcell; Homo_633,totalSample,immune-CD4_T; Homo_633,totalSample,immune-CD8_T; Homo_633,totalSample,immune-CD4_naive; Homo_633,totalSample,immune-Neutrophil; Homo_633,totalSample,immune-iTreg; MCSA,totalSample,immune-DC; MCSA,totalSample,immune-Monocyte; MCSA,totalSample,immune-Macrophage; MCSA,totalSample,immune-Neutrophil; MCSA,totalSample,immune-CD8_T; MCSA,totalSample,immune-NKT; MCSA,totalSample,immune-Tr1; MCSA,totalSample,immune-iTreg; MCSA,totalSample,immune-Th2; MCSA,totalSample,immune-Cytotoxic; MCSA,totalSample,immune-Effector_memory; SRP223445,totalSample,immune-Neutrophil; SRP223445,totalSample,immune-CD4_naive; [illegible]
DPCD	Emory,control,male.vs.female; Emory,MCI,age; MCSA,MCI,age; Emory,totalSample,immune-Neutrophil; Emory,totalSample,immune-Gamma_delta; Emory,totalSample,immune-Th17; Emory,totalSample,immune-CD8_naive; Emory,totalSample,immune-Cytotoxic; Emory,totalSample,immune-Central_memory; Homo_723,totalSample,immune-Bcell; Homo_714,totalSample,immune-Bcell; Homo_714,totalSample,immune-Th2; Homo_714,totalSample,immune-DC; Homo_714,totalSample,immune-CD4_naive; Homo_714,totalSample,immune-iTreg; Homo_633,totalSample,immune-Bcell; Homo_633,totalSample,immune-CD4_naive; Homo_633,totalSample,immune-Monocyte; Homo_633,totalSample,immune-DC; Homo_633,totalSample,immune-Th2; MCSA,totalSample,immune-Macrophage; MCSA,totalSample,immune-CD4_T; MCSA,totalSample,immune-CD4_naive; MCSA,totalSample,immune-Tr1; MCSA,totalSample,immune-nTreg; MCSA,totalSample,immune-iTreg; MCSA,totalSample,immune-Th17; MCSA,totalSample,immune-Tfh; MCSA,totalSample,immune-
GYPB	Emory,control,male.vs.female; Homo_723,male,AD.vs.control; Homo_723,male,AD.vs.MCI; Homo_723,female,MCI.vs.control; Homo_723,male,MCI.vs.control; Homo_723,control,male.vs.female; Homo_723,MCI,male.vs.female; Homo_714,male,AD.vs.control; Homo_714,male,AD.vs.MCI; Homo_714,female,MCI.vs.control; Homo_714,male,MCI.vs.control; Homo_714,control,male.vs.female; Homo_714,MCI,male.vs.female; Homo_633,male,MCI.vs.control; Homo_633,control,male.vs.female; Homo_633,MCI,male.vs.female; Emory,MCI,age; Emory,totalSample,immune-iTreg; Emory,totalSample,immune-CD8_naive; Homo_714,totalSample,immune-Th2; MCSA,totalSample,immune-Central_memory;
FIS1	Emory,control,male.vs.female; Homo_723,female,AD.vs.control; Homo_723,male,AD.vs.control; Homo_723,female,AD.vs.MCI; Homo_723,male,AD.vs.MCI; Homo_714,female,AD.vs.control; Homo_714,male,AD.vs.control; Homo_714,female,AD.vs.MCI; Homo_714,male,AD.vs.MCI; Homo_633,female,AD.vs.control; Homo_633,female,AD.vs.MCI; ROSMAP,female,AD.vs.control; ACOM,totalSample,age; Emory,totalSample,age; Emory,MCI,age; MCSA,MCI,age; ROSMAP,MCI,last.visit.age; ROSMAP,MCI,sample.age; Emory,totalSample,immune-Neutrophil; Emory,totalSample,immune-Gamma_delta; Emory,totalSample,immune-Th17; Emory,totalSample,immune-Tfh; Emory,totalSample,immune-CD8_naive; Emory,totalSample,immune-Cytotoxic; Homo_723,totalSample,immune-Cytotoxic; Homo_723,totalSample,immune-CD8_T; Homo_723,totalSample,immune-Th1; Homo_714,totalSample,immune-Cytotoxic; Homo_714,totalSample,immune-Th1; Homo_714,totalSample,immune-DC; Homo_714,totalSample,immune-NKT; Homo_714,totalSample,immune-CD8_T; Homo_633,totalSample,immune-DC; Homo_633,totalSample,immune-NKT; Homo_633,totalSample,immune-Cytotoxic; Homo_633,totalSample,immune-Th1; Homo_633,totalSample,immune-Neutrophil; Homo_633,totalSample,immune-Monocyte; Homo_633,totalSample,immune-CD8_T; Homo_633,totalSample,immune-Bcell; MCSA,totalSample,immune-DC; MCSA,totalSample,immune-Monocyte; MCSA,totalSample,immune-Macrophage; MCSA,totalSample,immune-Neutrophil; MCSA,totalSample,immune-CD4_T; MCSA,totalSample,immune-CD8_T; MCSA,totalSample,immune-NKT; MCSA,totalSample,immune-Tr1; MCSA,totalSample,immune-nTreg; MCSA,totalSample,immune-iTreg; MCSA,totalSample,immune-Th2; MCSA,totalSample,immune-Th17; MCSA,totalSample,immune-Cytotoxic; MCSA,totalSample,immune-Exhausted; MCSA,totalSample,immune-Effector_memory; ROSMAP,totalSample,immune-NK; ROSMAP,totalSample,immune-nTreg; ROSMAP,totalSample,immune-iTreg; ROSMAP,totalSample,immune-Th17; ROSMAP,totalSample,immune-Effector_memory;
YIPF2	Emory,control,male.vs.female; ROSMAP,female,AD.vs.control; ROSMAP,male,AD.vs.control; ROSMAP,male,AD.vs.MCI; ROSMAP,AD,male.vs.female; Emory,MCI,age; MCSA,totalSample,age; MCSA,MCI,age; ROSMAP,AD,last.visit.age; Emory,totalSample,immune-Monocyte; Emory,totalSample,immune-Neutrophil; Emory,totalSample,immune-CD8_T; Emory,totalSample,immune-Gamma_delta; Emory,totalSample,immune-CD8_naive; Emory,totalSample,immune-Cytotoxic; Emory,totalSample,immune-Exhausted; Emory,totalSample,immune-MAIT; Homo_723,totalSample,immune-DC; Homo_723,totalSample,immune-Neutrophil; Homo_723,totalSample,immune-Cytotoxic; Homo_723,totalSample,immune-CD8_T; Homo_723,totalSample,immune-Effector_memory; Homo_723,totalSample,immune-Monocyte; Homo_714,totalSample,immune-DC; Homo_714,totalSample,immune-Neutrophil; Homo_714,totalSample,immune-CD8_T; Homo_714,totalSample,immune-Monocyte; Homo_714,totalSample,immune-Cytotoxic; Homo_714,totalSample,immune-InfiltrationScore; Homo_633,totalSample,immune-DC; Homo_633,totalSample,immune-Neutrophil; Homo_633,totalSample,immune-CD8_T; Homo_633,totalSample,immune-Th2; Homo_633,totalSample,immune-Monocyte; Homo_633,totalSample,immune-Cytotoxic; Homo_633,totalSample,immune-nTreg; MCSA,totalSample,immune-DC; MCSA,totalSample,immune-Monocyte; MCSA,totalSample,immune-Neutrophil; MCSA,totalSample,immune-CD8_T; MCSA,totalSample,immune-NKT; MCSA,totalSample,immune-iTreg; MCSA,totalSample,immune-Th2; MCSA,totalSample,immune-Cytotoxic; MCSA,totalSample,immune-Central_memory; MCSA,totalSample,immune-Effector_memory; MCSA,totalSample,immune-InfiltrationScore;
NUDT4B	Emory,control,male.vs.female; ROSMAP,male,AD.vs.control; Emory,totalSample,age; Emory,control,age; Emory,MCI,age; MCSA,MCI,age; ROSMAP,MCI,sample.age; Emory,totalSample,immune-DC; Emory,totalSample,immune-Monocyte; Emory,totalSample,immune-nTreg; Emory,totalSample,immune-CD8_naive; Emory,totalSample,immune-Cytotoxic; Homo_723,totalSample,immune-Bcell; Homo_723,totalSample,immune-Gamma_delta; Homo_723,totalSample,immune-NKT; Homo_723,totalSample,immune-iTreg; Homo_723,totalSample,immune-Tr1; Homo_723,totalSample,immune-Cytotoxic; Homo_723,totalSample,immune-Tfh; Homo_714,totalSample,immune-Tfh; Homo_714,totalSample,immune-NKT; Homo_714,totalSample,immune-Tr1; Homo_633,totalSample,immune-NKT; Homo_633,totalSample,immune-Monocyte; Homo_633,totalSample,immune-Tfh; Homo_633,totalSample,immune-DC; Homo_633,totalSample,immune-Gamma_delta; MCSA,totalSample,immune-DC; MCSA,totalSample,immune-Bcell; MCSA,totalSample,immune-Monocyte; MCSA,totalSample,immune-Macrophage; MCSA,totalSample,immune-CD4_T; MCSA,totalSample,immune-CD8_T; MCSA,totalSample,immune-CD4_naive; MCSA,totalSample,immune-Tr1; MCSA,totalSample,immune-nTreg; MCSA,totalSample,immune-iTreg; MCSA,totalSample,immune-Th2; MCSA,totalSample,immune-Tfh; MCSA,totalSample,immune-CD8_naive; MCSA,totalSample,immune-Cytotoxic; MCSA,totalSample,immune-Exhausted; MCSA,totalSample,immune-Effector_memory; SRP223445,totalSample,immune-CD4_naive; SRP223445,totalSample,immune-InfiltrationScore
MCOLN1	Emory,control,male.vs.female; Homo_723,male,AD.vs.control; Homo_723,male,AD.vs.MCI; Homo_723,AD,male.vs.female; Homo_723,control,male.vs.female; Homo_714,male,AD.vs.control; Homo_714,male,AD.vs.MCI; Homo_714,control,male.vs.female; Homo_633,female,MCI.vs.control; Homo_633,control,male.vs.female; ACOM,AD,age; MCSA,MCI,age; Emory,totalSample,immune-CD4_T; Emory,totalSample,immune-Gamma_delta; Emory,totalSample,immune-Tr1; Emory,totalSample,immune-iTreg; Emory,totalSample,immune-InfiltrationScore; Homo_723,totalSample,immune-DC; Homo_723,totalSample,immune-Cytotoxic; Homo_723,totalSample,immune-Tr1; Homo_723,totalSample,immune-Th2; Homo_723,totalSample,immune-iTreg; Homo_723,totalSample,immune-Th1; Homo_723,totalSample,immune-CD4_T; Homo_723,totalSample,immune-CD4_naive; Homo_723,totalSample,immune-NKT; Homo_723,totalSample,immune-Tfh; Homo_714,totalSample,immune-DC; Homo_714,totalSample,immune-Th2; Homo_714,totalSample,immune-Tr1; Homo_714,totalSample,immune-CD4_T; Homo_714,totalSample,immune-iTreg; Homo_714,totalSample,immune-Th1; Homo_714,totalSample,immune-Tfh; Homo_714,totalSample,immune-CD4_naive; Homo_714,totalSample,immune-NKT; Homo_714,totalSample,immune-Cytotoxic; Homo_633,totalSample,immune-DC; Homo_633,totalSample,immune-Th2; Homo_633,totalSample,immune-NKT; Homo_633,totalSample,immune-CD4_T; Homo_633,totalSample,immune-Th1; Homo_633,totalSample,immune-CD4_naive; Homo_633,totalSample,immune-Tr1; Homo_633,totalSample,immune-Tfh; Homo_633,totalSample,immune-Cytotoxic; MCSA,totalSample,immune-DC; MCSA,totalSample,immune-Monocyte; MCSA,totalSample,immune-Macrophage; MCSA,totalSample,immune-CD4_T; MCSA,totalSample,immune-CD8_T; MCSA,totalSample,immune-Tr1; MCSA,totalSample,immune-iTreg; MCSA,totalSample,immune-Th2; MCSA,totalSample,immune-Cytotoxic; MCSA,totalSample,immune-MAIT; MCSA,totalSample,immune-Effector_memory; ROSMAP,totalSample,immune- [illegible]
TMEM63B	Emory,control,male.vs.female; Homo_723,male,AD.vs.control; Homo_723,male,MCI.vs.control; Homo_723,control,male.vs.female; Homo_714,male,AD.vs.control; Homo_714,male,MCI.vs.control; Homo_714,control,male.vs.female; Homo_633,control,male.vs.female; ROSMAP,female,AD.vs.control; MCSA,totalSample,age; MCSA,control,age; MCSA,MCI,age; Emory,totalSample,immune-Gamma_delta; Emory,totalSample,immune-Tr1; Emory,totalSample,immune-iTreg; Homo_723,totalSample,immune-DC; Homo_723,totalSample,immune-Cytotoxic; Homo_723,totalSample,immune-Exhausted; Homo_723,totalSample,immune-Tr1; Homo_723,totalSample,immune-iTreg; Homo_723,totalSample,immune-Bcell; Homo_723,totalSample,immune-NKT; Homo_723,totalSample,immune-Th1; Homo_723,totalSample,immune-CD8_T; Homo_714,totalSample,immune-DC; Homo_714,totalSample,immune-Th2; Homo_714,totalSample,immune-Tr1; Homo_714,totalSample,immune-iTreg; Homo_714,totalSample,immune-Cytotoxic; Homo_633,totalSample,immune-DC; Homo_633,totalSample,immune-Th2; Homo_633,totalSample,immune-NKT; Homo_633,totalSample,immune-Cytotoxic; MCSA,totalSample,immune-DC; MCSA,totalSample,immune-Monocyte; MCSA,totalSample,immune-Macrophage; MCSA,totalSample,immune-CD8_T; MCSA,totalSample,immune-iTreg; MCSA,totalSample,immune-Th2; MCSA,totalSample,immune-
HBQ1	Emory,control,male.vs.female; Homo_723,male,AD.vs.control; Homo_714,male,AD.vs.control; Homo_714,male,AD.vs.MCI; Homo_633,control,male.vs.female; Emory,MCI,age; MCSA,MCI,age; Emory,totalSample,immune-Gamma_delta; Emory,totalSample,immune-Tr1; Emory,totalSample,immune-iTreg; Emory,totalSample,immune-CD8_naive; Homo_723,totalSample,immune-DC; Homo_723,totalSample,immune-Cytotoxic; Homo_723,totalSample,immune-Bcell; Homo_723,totalSample,immune-Th2; Homo_723,totalSample,immune-Tr1; Homo_714,totalSample,immune-DC; Homo_714,totalSample,immune-Th2; Homo_714,totalSample,immune-Bcell; Homo_714,totalSample,immune-Cytotoxic; Homo_714,totalSample,immune-iTreg; Homo_714,totalSample,immune-Th1; Homo_714,totalSample,immune-NKT; Homo_633,totalSample,immune-DC; Homo_633,totalSample,immune-Th2; Homo_633,totalSample,immune-NKT; Homo_633,totalSample,immune-Bcell; Homo_633,totalSample,immune-Cytotoxic; Homo_633,totalSample,immune-Th1; Homo_633,totalSample,immune-CD4_T; Homo_633,totalSample,immune-CD4_naive; MCSA,totalSample,immune-DC; MCSA,totalSample,immune-Monocyte; MCSA,totalSample,immune-Macrophage; MCSA,totalSample,immune-Neutrophil; MCSA,totalSample,immune-CD4_T; MCSA,totalSample,immune-CD8_T; MCSA,totalSample,immune-NKT; MCSA,totalSample,immune-Tr1; MCSA,totalSample,immune-iTreg; MCSA,totalSample,immune-Th2; MCSA,totalSample,immune-Cytotoxic; MCSA,totalSample,immune-Effector_memory
ISM1	Emory,control,male.vs.female; Homo_714,female,AD.vs.control; Homo_633,female,AD.vs.control; Homo_633,female,AD.vs.MCI; Emory,MCI,age; MCSA,MCI,age; Emory,totalSample,immune-CD4_T; Emory,totalSample,immune-Gamma_delta; Emory,totalSample,immune-CD4_naive; Emory,totalSample,immune-Tr1; Emory,totalSample,immune-nTreg; Emory,totalSample,immune-iTreg; Emory,totalSample,immune-Tfh; Emory,totalSample,immune-Cytotoxic; Emory,totalSample,immune-Central_memory; Homo_723,totalSample,immune-Tfh; Homo_723,totalSample,immune-iTreg; Homo_723,totalSample,immune-CD4_naive; Homo_723,totalSample,immune-CD4_T; Homo_723,totalSample,immune-nTreg; Homo_723,totalSample,immune-Central_memory; Homo_723,totalSample,immune-Tr1; Homo_723,totalSample,immune-Th2; Homo_723,totalSample,immune-Gamma_delta; Homo_723,totalSample,immune-Effector_memory; Homo_723,totalSample,immune-InfiltrationScore; Homo_723,totalSample,immune-Th1; Homo_714,totalSample,immune-Tfh; Homo_714,totalSample,immune-nTreg; Homo_714,totalSample,immune-CD4_T; Homo_714,totalSample,immune-iTreg; Homo_714,totalSample,immune-Central_memory; Homo_714,totalSample,immune-CD4_naive; Homo_714,totalSample,immune-Tr1; Homo_714,totalSample,immune-Th2; Homo_714,totalSample,immune-Effector_memory; Homo_714,totalSample,immune-Gamma_delta; Homo_633,totalSample,immune-Tfh; Homo_633,totalSample,immune-nTreg; Homo_633,totalSample,immune-Central_memory; Homo_633,totalSample,immune-CD4_T; Homo_633,totalSample,immune-Tr1; Homo_633,totalSample,immune-CD4_naive; Homo_633,totalSample,immune-iTreg; Homo_633,totalSample,immune-Effector_memory; Homo_633,totalSample,immune-Th2; Homo_633,totalSample,immune-Gamma_delta; Homo_633,totalSample,immune-Cytotoxic; Homo_633,totalSample,immune-Th1; Homo_633,totalSample,immune-Exhausted; Homo_633,totalSample,immune-Th17; MCSA,totalSample,immune-Bcell; MCSA,totalSample,immune-Neutrophil; MCSA,totalSample,immune-CD4_T; MCSA,totalSample,immune-Gamma_delta; MCSA,totalSample,immune-CD4_naive; MCSA,totalSample,immune-Tr1; MCSA,totalSample,immune-nTreg; MCSA,totalSample,immune-iTreg; MCSA,totalSample,immune-Th17; MCSA,totalSample,immune-Tfh; MCSA,totalSample,immune-CD8_naive; MCSA,totalSample,immune-Exhausted; [illegible]
DMTN	Emory,control,male.vs.female; Homo_723,control,male.vs.female; Homo_714,control,male.vs.female; Homo_633,control,male.vs.female; ROSMAP,male,AD.vs.control; ROSMAP,male,AD.vs.MCI; ACOM,totalSample,age; Emory,MCI,age; MCSA,MCI,age; ROSMAP,MCI,last.visit.age; ROSMAP,MCI,sample.age; Emory,totalSample,immune-Gamma_delta; Emory,totalSample,immune-Tr1; Emory,totalSample,immune-iTreg; Emory,totalSample,immune-CD8_naive; Emory,totalSample,immune-Cytotoxic; Homo_723,totalSample,immune-Bcell; Homo_723,totalSample,immune-DC; Homo_714,totalSample,immune-DC; Homo_714,totalSample,immune-Bcell; Homo_633,totalSample,immune-Bcell; Homo_633,totalSample,immune-DC; Homo_633,totalSample,immune-NKT; Homo_633,totalSample,immune-Th1; Homo_633,totalSample,immune-Th2; Homo_633,totalSample,immune-CD4_naive; Homo_633,totalSample,immune-CD4_T; MCSA,totalSample,immune-DC; MCSA,totalSample,immune-Monocyte; MCSA,totalSample,immune-Macrophage; MCSA,totalSample,immune-CD8_T; MCSA,totalSample,immune-iTreg; MCSA,totalSample,immune-Th2; MCSA,totalSample,immune-Cytotoxic; SRP223445,totalSample,immune-Exhausted
KANK2	Emory,control,male.vs.female; Homo_723,female,AD.vs.control; Homo_723,male,AD.vs.control; Homo_723,female,AD.vs.MCI; Homo_723,male,AD.vs.MCI; Homo_723,female,MCI.vs.control; Homo_714,female,AD.vs.control; Homo_714,male,AD.vs.control; Homo_714,female,AD.vs.MCI; Homo_714,male,AD.vs.MCI; Homo_714,female,MCI.vs.control; Homo_633,female,AD.vs.control; Homo_633,female,AD.vs.MCI; Homo_633,female,MCI.vs.control; Homo_633,male,MCI.vs.control; Emory,MCI,age; MCSA,MCI,age; ROSMAP,MCI,sample.age; Emory,totalSample,immune-Gamma_delta; Emory,totalSample,immune-CD8_naive; Homo_723,totalSample,immune-DC; Homo_723,totalSample,immune-Bcell; Homo_723,totalSample,immune-Neutrophil; Homo_723,totalSample,immune-Th2; Homo_714,totalSample,immune-Th2; Homo_714,totalSample,immune-DC; Homo_633,totalSample,immune-Bcell; Homo_633,totalSample,immune-Th2; MCSA,totalSample,immune-Macrophage; MCSA,totalSample,immune-MAIT; SRP223445,totalSample,immune-Neutrophil; SRP223445,totalSample,immune-nTreg; SRP223445,totalSample,immune-InfiltrationScore; ROSMAP,female-excludeAge90+,survival; ROSMAP,female-includeAge90+,survival

GART	Emory,control,male.vs.female; Homo_633,male,MCI.vs.control; MCSA,totalSample,age; MCSA,control,age; ROSMAP,MCI,last.visit.age; Emory,totalSample,immune-NK; Emory,totalSample,immune-Neutrophil; Emory,totalSample,immune-CD4_T; Emory,totalSample,immune-Tr1; Emory,totalSample,immune-iTreg; Emory,totalSample,immune-Exhausted; Emory,totalSample,immune-InfiltrationScore; Homo_723,totalSample,immune-CD4_naive; Homo_723,totalSample,immune-CD4_T; Homo_723,totalSample,immune-Tr1; Homo_723,totalSample,immune-Tfh; Homo_723,totalSample,immune-nTreg; Homo_723,totalSample,immune-iTreg; Homo_723,totalSample,immune-Th2; Homo_723,totalSample,immune-Central_memory; Homo_723,totalSample,immune-InfiltrationScore; Homo_723,totalSample,immune-Effector_memory; Homo_723,totalSample,immune-Monocyte; Homo_714,totalSample,immune-CD4_naive; Homo_714,totalSample,immune-CD4_T; Homo_714,totalSample,immune-Tr1; Homo_714,totalSample,immune-Tfh; Homo_714,totalSample,immune-nTreg; Homo_714,totalSample,immune-iTreg; Homo_714,totalSample,immune-Effector_memory; Homo_714,totalSample,immune-Th1; Homo_714,totalSample,immune-Central_memory; Homo_714,totalSample,immune-Th2; Homo_714,totalSample,immune-Neutrophil; Homo_714,totalSample,immune-InfiltrationScore; Homo_714,totalSample,immune-NKT; Homo_714,totalSample,immune-Monocyte; Homo_633,totalSample,immune-CD4_naive; Homo_633,totalSample,immune-CD4_T; Homo_633,totalSample,immune-Tr1; Homo_633,totalSample,immune-Effector_memory; Homo_633,totalSample,immune-Tfh; Homo_633,totalSample,immune-nTreg; Homo_633,totalSample,immune-Th1; Homo_633,totalSample,immune-iTreg; Homo_633,totalSample,immune-NKT; Homo_633,totalSample,immune-InfiltrationScore; Homo_633,totalSample,immune-Central_memory; Homo_633,totalSample,immune-Neutrophil; Homo_633,totalSample,immune-Monocyte; Homo_633,totalSample,immune-Th2; Homo_633,totalSample,immune-MAIT; Homo_633,totalSample,immune-Macrophage; MCSA,totalSample,immune-Monocyte; MCSA,totalSample,immune-Macrophage; MCSA,totalSample,immune-Neutrophil; MCSA,totalSample,immune-CD4_T; MCSA,totalSample,immune-CD4_naive; MCSA,totalSample,immune-Tr1; MCSA,totalSample,immune-nTreg; MCSA,totalSample,immune-iTreg; MCSA,totalSample,immune-Th17; MCSA,totalSample,immune-Tfh; MCSA,totalSample,immune-CD8_naive; MCSA,totalSample,immune-MAIT; MCSA,totalSample,immune-Central_memory; MCSA,totalSample,immune-Effector_memory; MCSA,totalSample,immune-InfiltrationScore; ROSMAP,totalSample,immune-MAIT; ROSMAP,totalSample,immune-Effector_memory
STMP1	Emory,control,male.vs.female; Homo_723,female,AD.vs.control; Homo_714,female,AD.vs.control; Homo_714,female,AD.vs.MCI; ROSMAP,female,MCI.vs.control; SRP223445,female,AD.vs.control; Emory,MCI,age; ROSMAP,MCI,last.visit.age; Emory,totalSample,immune-Gamma_delta; Emory,totalSample,immune-iTreg; Emory,totalSample,immune-Th17; Emory,totalSample,immune-CD8_naive; Emory,totalSample,immune-Cytotoxic; Emory,totalSample,immune-InfiltrationScore; Homo_723,totalSample,immune-Bcell; Homo_723,totalSample,immune-Monocyte; Homo_723,totalSample,immune-Effector_memory; Homo_714,totalSample,immune-Monocyte; Homo_714,totalSample,immune-Effector_memory; Homo_714,totalSample,immune-nTreg; Homo_714,totalSample,immune-Bcell; Homo_633,totalSample,immune-Monocyte; Homo_633,totalSample,immune-Effector_memory; Homo_633,totalSample,immune-Bcell; Homo_633,totalSample,immune-nTreg; MCSA,totalSample,immune-DC; MCSA,totalSample,immune-Monocyte; MCSA,totalSample,immune-CD8_T; MCSA,totalSample,immune-iTreg; MCSA,totalSample,immune-Th2; MCSA,totalSample,immune-Cytotoxic; MCSA,totalSample,immune-Effector_memory; ROSMAP,totalSample,immune-Effector_memory; SRP223445,totalSample,immune-CD4_naive
TGM2	Emory,control,male.vs.female; Homo_723,female,AD.vs.control; Homo_723,male,AD.vs.control; Homo_723,female,AD.vs.MCI; Homo_723,male,AD.vs.MCI; Homo_723,male,MCI.vs.control; Homo_714,female,AD.vs.control; Homo_714,male,AD.vs.control; Homo_714,female,AD.vs.MCI; Homo_714,male,AD.vs.MCI; Homo_714,male,MCI.vs.control; Emory,MCI,age; MCSA,MCI,age; ROSMAP,AD,last.visit.age; Emory,totalSample,immune-Gamma_delta; Emory,totalSample,immune-Tr1; Emory,totalSample,immune-iTreg; Homo_723,totalSample,immune-DC; Homo_723,totalSample,immune-Th2; Homo_723,totalSample,immune-Bcell; Homo_723,totalSample,immune-Cytotoxic; Homo_723,totalSample,immune-Tr1; Homo_723,totalSample,immune-iTreg; Homo_723,totalSample,immune-Neutrophil; Homo_714,totalSample,immune-DC; Homo_714,totalSample,immune-Th2; Homo_714,totalSample,immune-Bcell; Homo_714,totalSample,immune-CD4_T; Homo_633,totalSample,immune-DC; Homo_633,totalSample,immune-Th2; Homo_633,totalSample,immune-Bcell; MCSA,totalSample,immune-DC; MCSA,totalSample,immune-Monocyte; MCSA,totalSample,immune-Macrophage; MCSA,totalSample,immune-CD4_T; MCSA,totalSample,immune-CD8_T; MCSA,totalSample,immune-Tr1; MCSA,totalSample,immune-iTreg; MCSA,totalSample,immune-Th2; MCSA,totalSample,immune-Cytotoxic; SRP223445,totalSample,immune-
RANBP10	Emory,control,male.vs.female; Homo_723,male,AD.vs.control; Homo_723,male,AD.vs.MCI; Homo_714,male,AD.vs.control; Homo_714,male,AD.vs.MCI; Homo_633,control,male.vs.female; ROSMAP,female,AD.vs.control; Emory,MCI,age; MCSA,totalSample,age; MCSA,control,age; MCSA,MCI,age; Emory,totalSample,immune-Gamma_delta; Emory,totalSample,immune-CD8_naive; Homo_723,totalSample,immune-Bcell; Homo_723,totalSample,immune-DC; Homo_723,totalSample,immune-Cytotoxic; Homo_714,totalSample,immune-Bcell; Homo_714,totalSample,immune-DC; Homo_633,totalSample,immune-DC; Homo_633,totalSample,immune-Bcell; Homo_633,totalSample,immune-NKT; MCSA,totalSample,immune-DC; MCSA,totalSample,immune-Monocyte; MCSA,totalSample,immune-Th2; MCSA,totalSample,immune-Th17; MCSA,totalSample,immune-Effector_memory; MCSA,totalSample,immune-InfiltrationScore; ROSMAP,totalSample,immune-Effector_memory; SRP223445,totalSample,immune-CD4_naive
ATP6V0C	Emory,control,male.vs.female; Emory,MCI,age; MCSA,MCI,age; Emory,totalSample,immune-Monocyte; Emory,totalSample,immune-Gamma_delta; Emory,totalSample,immune-Tr1; Emory,totalSample,immune-iTreg; Homo_723,totalSample,immune-DC; Homo_723,totalSample,immune-CD4_naive; Homo_723,totalSample,immune-Tr1; Homo_723,totalSample,immune-iTreg; Homo_723,totalSample,immune-CD4_T; Homo_723,totalSample,immune-Th1; Homo_723,totalSample,immune-Tfh; Homo_723,totalSample,immune-Th2; Homo_723,totalSample,immune-Cytotoxic; Homo_723,totalSample,immune-Bcell; Homo_714,totalSample,immune-DC; Homo_714,totalSample,immune-CD4_naive; Homo_714,totalSample,immune-CD4_T; Homo_714,totalSample,immune-Tfh; Homo_714,totalSample,immune-iTreg; Homo_714,totalSample,immune-Th1; Homo_714,totalSample,immune-Tr1; Homo_714,totalSample,immune-Th2; Homo_714,totalSample,immune-Effector_memory; Homo_714,totalSample,immune-NKT; Homo_714,totalSample,immune-Bcell; Homo_633,totalSample,immune-DC; Homo_633,totalSample,immune-CD4_naive; Homo_633,totalSample,immune-NKT; Homo_633,totalSample,immune-CD4_T; Homo_633,totalSample,immune-Tfh; Homo_633,totalSample,immune-Th1; Homo_633,totalSample,immune-Effector_memory; Homo_633,totalSample,immune-Bcell; Homo_633,totalSample,immune-Th2; Homo_633,totalSample,immune-Tr1; Homo_633,totalSample,immune-iTreg; Homo_633,totalSample,immune-Central_memory; MCSA,totalSample,immune-DC; MCSA,totalSample,immune-Monocyte; MCSA,totalSample,immune-Macrophage; MCSA,totalSample,immune-CD4_T; MCSA,totalSample,immune-CD8_T; MCSA,totalSample,immune-CD4_naive; MCSA,totalSample,immune-Tr1; MCSA,totalSample,immune-nTreg; MCSA,totalSample,immune-iTreg; MCSA,totalSample,immune-Th2; MCSA,totalSample,immune-Tfh; MCSA,totalSample,immune-CD8_naive; MCSA,totalSample,immune-Cytotoxic; MCSA,totalSample,immune-Exhausted; MCSA,totalSample,immune-MAIT; MCSA,totalSample,immune-Central_memory
ATP1B2	Emory,control,male.vs.female; Homo_723,female,MCI.vs.control; Homo_714,female,MCI.vs.control; Homo_714,MCI,male.vs.female; Homo_633,female,MCI.vs.control; Homo_633,MCI,male.vs.female; ROSMAP,female,AD.vs.control; ROSMAP,male,AD.vs.control; ROSMAP,female,AD.vs.MCI; ROSMAP,MCI,last.visit.age; ROSMAP,MCI,sample.age; Emory,totalSample,immune-Gamma_delta; Emory,totalSample,immune-Tr1; Emory,totalSample,immune-iTreg; Homo_723,totalSample,immune-Monocyte; Homo_723,totalSample,immune-DC; Homo_723,totalSample,immune-Th2; Homo_714,totalSample,immune-Th2; Homo_714,totalSample,immune-Monocyte; Homo_633,totalSample,immune-Monocyte; Homo_633,totalSample,immune-Th2; SRP223445,totalSample,immune-CD4_naive; ROSMAP,female-includeAge90+,survival; ROSMAP,male and female-includeAge90+,survival
GYPC	Emory,control,male.vs.female; ROSMAP,female,AD.vs.control; ACOM,totalSample,age; Emory,MCI,age; MCSA,MCI,age; Emory,totalSample,immune-Gamma_delta; Emory,totalSample,immune-Tr1; Emory,totalSample,immune-iTreg; Emory,totalSample,immune-Th17; Emory,totalSample,immune-Cytotoxic; Emory,totalSample,immune-InfiltrationScore; Homo_723,totalSample,immune-Bcell; Homo_723,totalSample,immune-Th2; Homo_723,totalSample,immune-DC; Homo_714,totalSample,immune-Bcell; Homo_714,totalSample,immune-CD4_naive; Homo_714,totalSample,immune-Th2; Homo_714,totalSample,immune-DC; Homo_633,totalSample,immune-Bcell; Homo_633,totalSample,immune-Th2; Homo_633,totalSample,immune-DC; Homo_633,totalSample,immune-CD4_naive; Homo_633,totalSample,immune-CD4_T; MCSA,totalSample,immune-DC; MCSA,totalSample,immune-Monocyte; MCSA,totalSample,immune-Macrophage; MCSA,totalSample,immune-CD4_T; MCSA,totalSample,immune-CD8_T; MCSA,totalSample,immune-CD4_naive; MCSA,totalSample,immune-Tr1; MCSA,totalSample,immune-nTreg; MCSA,totalSample,immune-iTreg; MCSA,totalSample,immune-Th2; MCSA,totalSample,immune-Cytotoxic; ROSMAP,totalSample,immune-Effector_memory; SRP223445,totalSample,immune-CD4_naive; SRP223445,totalSample,immune-InfiltrationScore
TANGO2	Emory,control,male.vs.female; ROSMAP,female,AD.vs.control; ROSMAP,female,MCI.vs.control; MCSA,MCI,age; Emory,totalSample,immune-Gamma_delta; Emory,totalSample,immune-Tr1; Emory,totalSample,immune-iTreg; Homo_723,totalSample,immune-DC; Homo_723,totalSample,immune-Th1; Homo_723,totalSample,immune-Bcell; Homo_714,totalSample,immune-DC; Homo_714,totalSample,immune-Bcell; Homo_714,totalSample,immune-Th1; Homo_633,totalSample,immune-DC; Homo_633,totalSample,immune-Bcell; Homo_633,totalSample,immune-Th1; MCSA,totalSample,immune-DC; MCSA,totalSample,immune-Monocyte; MCSA,totalSample,immune-Macrophage; MCSA,totalSample,immune-CD4_T; MCSA,totalSample,immune-CD8_T; MCSA,totalSample,immune-Tr1; MCSA,totalSample,immune-iTreg; MCSA,totalSample,immune-Th2; MCSA,totalSample,immune-Cytotoxic; MCSA,totalSample,immune-Effector_memory; ROSMAP,totalSample,immune-Effector_memory; SRP223445,totalSample,immune-Exhausted
FKBP8	Emory,control,male.vs.female; ROSMAP,female,AD.vs.control; ROSMAP,female,MCI.vs.control; Emory,MCI,age; MCSA,MCI,age; Emory,totalSample,immune-Gamma_delta; Emory,totalSample,immune-Tr1; Emory,totalSample,immune-iTreg; Emory,totalSample,immune-CD8_naive; Homo_723,totalSample,immune-DC; Homo_723,totalSample,immune-Bcell; Homo_723,totalSample,immune-Cytotoxic; Homo_723,totalSample,immune-Th2; Homo_723,totalSample,immune-Th1; Homo_714,totalSample,immune-DC; Homo_714,totalSample,immune-Bcell; Homo_714,totalSample,immune-Th1; Homo_714,totalSample,immune-Th2; Homo_714,totalSample,immune-CD4_naive; Homo_633,totalSample,immune-DC; Homo_633,totalSample,immune-Bcell; Homo_633,totalSample,immune-Th2; Homo_633,totalSample,immune-Th1; Homo_633,totalSample,immune-NKT; Homo_633,totalSample,immune-CD4_naive; Homo_633,totalSample,immune-CD4_T; MCSA,totalSample,immune-DC; MCSA,totalSample,immune-Monocyte; MCSA,totalSample,immune-Macrophage; MCSA,totalSample,immune-Neutrophil; MCSA,totalSample,immune-CD4_T; MCSA,totalSample,immune-CD8_T; MCSA,totalSample,immune-NKT; MCSA,totalSample,immune-Tr1; MCSA,totalSample,immune-iTreg; MCSA,totalSample,immune-Th2; MCSA,totalSample,immune-Cytotoxic; MCSA,totalSample,immune-Effector_memory
YBX3	Emory,control,male.vs.female; Emory,MCI,age; ROSMAP,MCI,sample.age; Emory,totalSample,immune-Gamma_delta; Emory,totalSample,immune-Tr1; Emory,totalSample,immune-iTreg; Emory,totalSample,immune-Th17; Emory,totalSample,immune-CD8_naive; Homo_723,totalSample,immune-Bcell; Homo_714,totalSample,immune-Bcell; Homo_633,totalSample,immune-Bcell; Homo_633,totalSample,immune-CD4_naive; SRP223445,totalSample,immune-CD4_naive; SRP223445,totalSample,immune-InfiltrationScore
PARM1	Emory,control,male.vs.female; Homo_723,female,AD.vs.control; Homo_723,male,AD.vs.control; Homo_723,female,AD.vs.MCI; Homo_723,male,AD.vs.MCI; Homo_714,female,AD.vs.control; Homo_714,male,AD.vs.control; Homo_714,female,AD.vs.MCI; Homo_714,male,AD.vs.MCI; Homo_633,female,AD.vs.control; Homo_633,female,AD.vs.MCI; ROSMAP,female,AD.vs.control; ROSMAP,male,AD.vs.control; ROSMAP,female,AD.vs.MCI; ROSMAP,male,AD.vs.MCI; Emory,totalSample,immune-B_cell; Emory,totalSample,immune-Macrophage; Emory,totalSample,immune-Neutrophil; Emory,totalSample,immune-CD4_T; Emory,totalSample,immune-CD8_T; Emory,totalSample,immune-Tr1; Emory,totalSample,immune-iTreg; Emory,totalSample,immune-Tfh; Emory,totalSample,immune-CD8_naive; Emory,totalSample,immune-Exhausted; Emory,totalSample,immune-InfiltrationScore; Homo_723,totalSample,immune-CD4_naive; Homo_723,totalSample,immune-nTreg; Homo_723,totalSample,immune-Neutrophil; Homo_723,totalSample,immune-Th1; Homo_723,totalSample,immune-CD4_T; Homo_723,totalSample,immune-Central_memory; Homo_723,totalSample,immune-DC; Homo_723,totalSample,immune-InfiltrationScore; Homo_723,totalSample,immune-Monocyte; Homo_723,totalSample,immune-Tfh; Homo_723,totalSample,immune-Tr1; Homo_723,totalSample,immune-iTreg; Homo_714,totalSample,immune-InfiltrationScore; Homo_714,totalSample,immune-NKT; Homo_714,totalSample,immune-Th1; Homo_714,totalSample,immune-CD4_naive; Homo_714,totalSample,immune-Neutrophil; Homo_714,totalSample,immune-nTreg; Homo_714,totalSample,immune-CD4_T; Homo_714,totalSample,immune-iTreg; Homo_714,totalSample,immune-Tfh; Homo_633,totalSample,immune-Tfh; Homo_633,totalSample,immune-CD4_T; Homo_633,totalSample,immune-CD4_naive; Homo_633,totalSample,immune-Tr1; Homo_633,totalSample,immune-NKT; Homo_633,totalSample,immune-iTreg; Homo_633,totalSample,immune-InfiltrationScore; Homo_633,totalSample,immune-nTreg; Homo_633,totalSample,immune-Th1; Homo_633,totalSample,immune-Monocyte; MCSA,totalSample,immune-Bcell; MCSA,totalSample,immune-CD4_T; MCSA,totalSample,immune-CD4_naive; MCSA,totalSample,immune-Tr1; MCSA,totalSample,immune-nTreg; MCSA,totalSample,immune-iTreg; MCSA,totalSample,immune-Th17; MCSA,totalSample,immune-Tfh; MCSA,totalSample,immune-CD8_naive; MCSA,totalSample,immune- [illegible]
MBNL3	Emory,control,male.vs.female; ROSMAP,female,AD.vs.control; Emory,MCI,age; Emory,totalSample,immune-Gamma_delta; Emory,totalSample,immune-Tr1; Emory,totalSample,immune-iTreg; Emory,totalSample,immune-CD8_naive; Homo_723,totalSample,immune-Bcell; Homo_714,totalSample,immune-Bcell; Homo_633,totalSample,immune-Bcell; Homo_633,totalSample,immune-Th17; MCSA,totalSample,immune-CD4_T; MCSA,totalSample,immune-Gamma_delta; MCSA,totalSample,immune-CD4_naive; MCSA,totalSample,immune-Tr1; MCSA,totalSample,immune-nTreg; MCSA,totalSample,immune-iTreg; MCSA,totalSample,immune-Th17; MCSA,totalSample,immune-Tfh; MCSA,totalSample,immune-CD8_naive; MCSA,totalSample,immune-Cytotoxic; MCSA,totalSample,immune-Exhausted; ROSMAP,totalSample,immune-Central_memory; ROSMAP,totalSample,immune-Effector_memory; SRP223445,totalSample,immune-NK; SRP223445,totalSample,immune-Neutrophil; SRP223445,totalSample,immune-InfiltrationScore; ROSMAP,female-excludeAge90+,survival
LTBP2	Emory,control,male.vs.female; Homo_723,female,AD.vs.control; Homo_723,male,AD.vs.control; Homo_723,female,AD.vs.MCI; Homo_723,male,AD.vs.MCI; Homo_714,female,AD.vs.control; Homo_714,male,AD.vs.control; Homo_714,female,AD.vs.MCI; Homo_714,male,AD.vs.MCI; Homo_633,female,AD.vs.control; Homo_633,female,AD.vs.MCI; ROSMAP,female,AD.vs.control; ROSMAP,male,AD.vs.control; ROSMAP,female,AD.vs.MCI; ROSMAP,female,MCI.vs.control; ROSMAP,male,MCI.vs.control; ROSMAP,MCI,last.visit.age; ROSMAP,MCI,sample.age; Emory,totalSample,immune-Tr1; Emory,totalSample,immune-iTreg; Emory,totalSample,immune-Th17; Homo_714,totalSample,immune-Bcell; Homo_633,totalSample,immune-Bcell; MCSA,totalSample,immune-NK; MCSA,totalSample,immune-Th2
GUCD1	Emory,control,male.vs.female; Homo_723,male,AD.vs.control; Homo_714,male,AD.vs.control; ROSMAP,female,AD.vs.control; ROSMAP,male,AD.vs.control; Emory,totalSample,age; Emory,MCI,age; MCSA,totalSample,age; MCSA,MCI,age; Emory,totalSample,immune-Gamma_delta; Homo_723,totalSample,immune-Bcell; Homo_723,totalSample,immune-DC; Homo_714,totalSample,immune-Bcell; Homo_714,totalSample,immune-DC; Homo_633,totalSample,immune-Bcell; Homo_633,totalSample,immune-DC; MCSA,totalSample,immune-DC; MCSA,totalSample,immune-Monocyte; MCSA,totalSample,immune-Macrophage; MCSA,totalSample,immune-Th2; MCSA,totalSample,immune-Th17; MCSA,totalSample,immune-MAIT; SRP223445,totalSample,immune-CD4_naive; SRP223445,totalSample,immune-InfiltrationScore
EMID1	Emory,control,male.vs.female; Homo_714,female,AD.vs.control; Homo_714,male,AD.vs.control; Homo_714,female,AD.vs.MCI; Homo_714,male,AD.vs.MCI; Homo_633,female,AD.vs.control; Homo_633,female,AD.vs.MCI; ROSMAP,female,AD.vs.control; ROSMAP,female,AD.vs.MCI; MCSA,MCI,age; Emory,totalSample,immune-Macrophage; Homo_723,totalSample,immune-Cytotoxic; Homo_723,totalSample,immune-CD8_T; Homo_723,totalSample,immune-Th1; Homo_714,totalSample,immune-Th1; Homo_714,totalSample,immune-DC; Homo_714,totalSample,immune-NKT; Homo_714,totalSample,immune-Cytotoxic; Homo_633,totalSample,immune-DC; Homo_633,totalSample,immune-Th1; Homo_633,totalSample,immune-NKT; Homo_633,totalSample,immune-Bcell; MCSA,totalSample,immune-DC; MCSA,totalSample,immune-Monocyte; MCSA,totalSample,immune-Macrophage; MCSA,totalSample,immune-CD8_T; MCSA,totalSample,immune-NKT; MCSA,totalSample,immune-iTreg; MCSA,totalSample,immune-Th2; MCSA,totalSample,immune-Cytotoxic;
HBD	Emory,control,male.vs.female; Emory,totalSample,age; Emory,MCI,age; Emory,totalSample,immune-Gamma_delta; Emory,totalSample,immune-CD8_naive; Homo_723,totalSample,immune-Bcell; Homo_714,totalSample,immune-Bcell; Homo_633,totalSample,immune-Bcell; ROSMAP,totalSample,immune-InfiltrationScore
PLEK2	Emory,control,male.vs.female; Homo_723,female,AD.vs.control; Homo_723,male,AD.vs.control; Homo_723,female,AD.vs.MCI; Homo_723,male,AD.vs.MCI; Homo_723,female,MCI.vs.control; Homo_714,female,AD.vs.control; Homo_714,male,AD.vs.control; Homo_714,female,AD.vs.MCI; Homo_714,male,AD.vs.MCI; Homo_714,female,MCI.vs.control; Homo_633,female,AD.vs.control; Homo_633,female,AD.vs.MCI; Homo_633,female,MCI.vs.control; ROSMAP,control,male.vs.female; Emory,MCI,age; Emory,totalSample,immune-Gamma_delta; Homo_723,totalSample,immune-Exhausted; Homo_723,totalSample,immune-CD8_T; Homo_723,totalSample,immune-Cytotoxic; Homo_723,totalSample,immune-Bcell; Homo_714,totalSample,immune-Bcell; Homo_633,totalSample,immune-Bcell; Homo_633,totalSample,immune-Th2; MCSA,totalSample,immune-Macrophage; MCSA,totalSample,immune-Central_memory; ROSMAP,totalSample,immune-InfiltrationScore
TLCD4	Emory,control,male.vs.female; Emory,totalSample,age; Emory,MCI,age; Emory,totalSample,immune-Gamma_delta; Emory,totalSample,immune-iTreg; Emory,totalSample,immune-Th17; Emory,totalSample,immune-CD8_naive; Emory,totalSample,immune-Cytotoxic; Homo_723,totalSample,immune-Bcell; Homo_714,totalSample,immune-Bcell; Homo_633,totalSample,immune-Bcell; MCSA,totalSample,immune-CD8_T; MCSA,totalSample,immune-Cytotoxic; ROSMAP,male-includeAge90+,survival
PRDX2	Emory,control,male.vs.female; Homo_723,male,AD.vs.control; Homo_723,female,AD.vs.MCI; Homo_723,male,AD.vs.MCI; Homo_714,male,AD.vs.control; Homo_714,female,AD.vs.MCI; Homo_714,male,AD.vs.MCI; ROSMAP,female,AD.vs.control; ROSMAP,female,MCI.vs.control; Emory,totalSample,age; Emory,MCI,age; MCSA,totalSample,age; MCSA,MCI,age; ROSMAP,MCI,last.visit.age; ROSMAP,MCI,sample.age; Emory,totalSample,immune-Neutrophil; Emory,totalSample,immune-CD8_T; Emory,totalSample,immune-Gamma_delta; Emory,totalSample,immune-Tfh; Emory,totalSample,immune-CD8_naive; Emory,totalSample,immune-Cytotoxic; Emory,totalSample,immune-Exhausted; Homo_723,totalSample,immune-Neutrophil; Homo_723,totalSample,immune-Bcell; Homo_723,totalSample,immune-Cytotoxic; Homo_723,totalSample,immune-DC; Homo_723,totalSample,immune-CD8_T; Homo_714,totalSample,immune-Neutrophil; Homo_714,totalSample,immune-DC; Homo_714,totalSample,immune-CD8_T; Homo_714,totalSample,immune-Bcell; Homo_714,totalSample,immune-Cytotoxic; Homo_633,totalSample,immune-DC; Homo_633,totalSample,immune-Neutrophil; Homo_633,totalSample,immune-CD8_T; Homo_633,totalSample,immune-Bcell; Homo_633,totalSample,immune-NKT; Homo_633,totalSample,immune-Cytotoxic; Homo_633,totalSample,immune-Th2; MCSA,totalSample,immune-DC; MCSA,totalSample,immune-Monocyte; MCSA,totalSample,immune-Neutrophil; MCSA,totalSample,immune-CD8_T; MCSA,totalSample,immune-NKT; MCSA,totalSample,immune-Th2; MCSA,totalSample,immune-Tfh; MCSA,totalSample,immune-Cytotoxic; MCSA,totalSample,immune-Effector_memory; MCSA,totalSample,immune-InfiltrationScore; ROSMAP,totalSample,immune-CD4_T; ROSMAP,totalSample,immune-iTreg; ROSMAP,totalSample,immune-Th17; ROSMAP,totalSample,immune-CD8_naive; ROSMAP,totalSample,immune-Effector_memory; ROSMAP,totalSample,immune-InfiltrationScore; SRP223445,totalSample,immune-Neutrophil; SRP223445,totalSample,immune-CD4_naive; SRP223445,totalSample,immune-InfiltrationScore
VWCE	Emory,control,male.vs.female; Homo_723,female,AD.vs.control; Homo_723,male,AD.vs.control; Homo_723,female,AD.vs.MCI; Homo_723,male,AD.vs.MCI; Homo_723,control,male.vs.female; Homo_714,female,AD.vs.control; Homo_714,male,AD.vs.control; Homo_714,male,AD.vs.MCI; Homo_714,control,male.vs.female; Homo_633,female,AD.vs.control; Homo_633,female,AD.vs.MCI; Homo_633,male,MCI.vs.control; Homo_633,control,male.vs.female; ROSMAP,female,MCI.vs.control; MCSA,MCI,age; ROSMAP,MCI,last.visit.age; Emory,totalSample,immune-Macrophage; Emory,totalSample,immune-NK; Emory,totalSample,immune-CD4_T; Emory,totalSample,immune-Tr1; Emory,totalSample,immune-iTreg; Emory,totalSample,immune-InfiltrationScore; Homo_723,totalSample,immune-DC; Homo_723,totalSample,immune-Cytotoxic; Homo_714,totalSample,immune-DC; Homo_714,totalSample,immune-Th2; Homo_714,totalSample,immune-CD4_T; Homo_714,totalSample,immune-CD4_naive; Homo_633,totalSample,immune-DC; Homo_633,totalSample,immune-Th2; Homo_633,totalSample,immune-NKT; Homo_633,totalSample,immune-CD4_T; MCSA,totalSample,immune-DC; MCSA,totalSample,immune-Monocyte; MCSA,totalSample,immune-Macrophage; MCSA,totalSample,immune-iTreg; MCSA,totalSample,immune-Cytotoxic; MCSA,totalSample,immune-MAIT
NUDT4	Emory,control,male.vs.female; Homo_723,female,AD.vs.control; Homo_723,male,AD.vs.control; Homo_723,female,AD.vs.MCI; Homo_723,male,AD.vs.MCI; Homo_723,male,MCI.vs.control; Homo_714,female,AD.vs.control; Homo_714,male,AD.vs.control; Homo_714,female,AD.vs.MCI; Homo_714,male,AD.vs.MCI; Homo_714,male,MCI.vs.control; Homo_633,female,AD.vs.control; Homo_633,female,AD.vs.MCI; Homo_633,male,MCI.vs.control; Homo_633,control,male.vs.female; ROSMAP,male,AD.vs.control; Emory,totalSample,age; Emory,control,age; Emory,MCI,age; MCSA,MCI,age; Emory,totalSample,immune-DC; Emory,totalSample,immune-CD8_naive; Homo_723,totalSample,immune-Th1; Homo_723,totalSample,immune-NKT; Homo_723,totalSample,immune-CD4_naive; Homo_723,totalSample,immune-Central_memory; Homo_723,totalSample,immune-CD4_T; Homo_723,totalSample,immune-Tr1; Homo_723,totalSample,immune-Tfh; Homo_723,totalSample,immune-Cytotoxic; Homo_723,totalSample,immune-Gamma_delta; Homo_723,totalSample,immune-iTreg; Homo_714,totalSample,immune-Th1; Homo_714,totalSample,immune-NKT; Homo_633,totalSample,immune-Th1; Homo_633,totalSample,immune-NKT; Homo_633,totalSample,immune-Tfh; Homo_633,totalSample,immune-DC; Homo_633,totalSample,immune-CD4_T; Homo_633,totalSample,immune-iTreg; Homo_633,totalSample,immune-CD4_naive; MCSA,totalSample,immune-DC; MCSA,totalSample,immune-Bcell; MCSA,totalSample,immune-Monocyte; MCSA,totalSample,immune-Macrophage; MCSA,totalSample,immune-CD4_T; MCSA,totalSample,immune-CD8_T; MCSA,totalSample,immune-CD4_naive; MCSA,totalSample,immune-Tr1; MCSA,totalSample,immune-nTreg; MCSA,totalSample,immune-iTreg; MCSA,totalSample,immune-Th2; MCSA,totalSample,immune-Th17; MCSA,totalSample,immune-Tfh; MCSA,totalSample,immune-CD8_naive; MCSA,totalSample,immune-Cytotoxic; MCSA,totalSample,immune-Exhausted; MCSA,totalSample,immune-MAIT; MCSA,totalSample,immune-Central_memory; SRP223445,totalSample,immune-CD4_naive;
GFUS	Emory,control,male.vs.female; Homo_723,female,AD.vs.control; Homo_723,female,AD.vs.MCI; Homo_714,female,AD.vs.control; Homo_714,male,AD.vs.control; Homo_714,female,AD.vs.MCI; Homo_714,male,AD.vs.MCI; Emory,MCI,age; MCSA,MCI,age; ROSMAP,AD,last.visit.age; Emory,totalSample,immune-Gamma_delta; Homo_723,totalSample,immune-DC; Homo_723,totalSample,immune-Bcell; Homo_723,totalSample,immune-Th2; Homo_723,totalSample,immune-Neutrophil; Homo_714,totalSample,immune-DC; Homo_714,totalSample,immune-Th2; Homo_714,totalSample,immune-Bcell; Homo_633,totalSample,immune-Th2; Homo_633,totalSample,immune-DC; Homo_633,totalSample,immune-Bcell; MCSA,totalSample,immune-DC; MCSA,totalSample,immune-Monocyte; MCSA,totalSample,immune-Macrophage; MCSA,totalSample,immune-CD8_T; MCSA,totalSample,immune-NKT; MCSA,totalSample,immune-iTreg; MCSA,totalSample,immune-Th2; MCSA,totalSample,immune-Cytotoxic; MCSA,totalSample,immune-Effector_memory; ROSMAP,totalSample,immune-Effector_memory; SRP223445,totalSample,immune-CD4_naive; SRP223445,totalSample,immune-InfiltrationScore

BACH2	Emory,control,male.vs.female; Homo_714,female,AD.vs.control; Homo_633,female,AD.vs.control; Emory,MCI,age; MCSA,totalSample,age; MCSA,control,age; ROSMAP,AD,last.visit.age; ROSMAP,MCI,last.visit.age; Emory,totalSample,immune-B_cell; Emory,totalSample,immune-Monocyte; Emory,totalSample,immune-Macrophage; Emory,totalSample,immune-Neutrophil; Emory,totalSample,immune-CD4_T; Emory,totalSample,immune-CD8_T; Emory,totalSample,immune-CD4_naive; Emory,totalSample,immune-Tr1; Emory,totalSample,immune-nTreg; Emory,totalSample,immune-iTreg; Emory,totalSample,immune-Tfh; Emory,totalSample,immune-CD8_naive; Emory,totalSample,immune-Central_memory; Emory,totalSample,immune-InfiltrationScore; Homo_723,totalSample,immune-CD4_T; Homo_723,totalSample,immune-iTreg; Homo_723,totalSample,immune-Tfh; Homo_723,totalSample,immune-Tr1; Homo_723,totalSample,immune-nTreg; Homo_723,totalSample,immune-CD4_naive; Homo_723,totalSample,immune-Central_memory; Homo_723,totalSample,immune-Th2; Homo_723,totalSample,immune-Macrophage; Homo_723,totalSample,immune-Effector_memory; Homo_723,totalSample,immune-Th1; Homo_723,totalSample,immune-Gamma_delta; Homo_723,totalSample,immune-Th17; Homo_723,totalSample,immune-InfiltrationScore; Homo_723,totalSample,immune-Cytotoxic; Homo_714,totalSample,immune-CD4_T; Homo_714,totalSample,immune-Tfh; Homo_714,totalSample,immune-nTreg; Homo_714,totalSample,immune-iTreg; Homo_714,totalSample,immune-Tr1; Homo_714,totalSample,immune-Central_memory; Homo_714,totalSample,immune-CD4_naive; Homo_714,totalSample,immune-Th2; Homo_714,totalSample,immune-Effector_memory; Homo_714,totalSample,immune-Th1; Homo_714,totalSample,immune-Macrophage; Homo_714,totalSample,immune-Gamma_delta; Homo_714,totalSample,immune-NKT; Homo_714,totalSample,immune-Th17; Homo_714,totalSample,immune-Cytotoxic; Homo_714,totalSample,immune-Neutrophil; Homo_633,totalSample,immune-CD4_T; Homo_633,totalSample,immune-Tfh; Homo_633,totalSample,immune-Tr1; Homo_633,totalSample,immune-nTreg; Homo_633,totalSample,immune-Central_memory; Homo_633,totalSample,immune-CD4_naive; Homo_633,totalSample,immune-iTreg; Homo_633,totalSample,immune-Effector_memory; Homo_633,totalSample,immune-Th2; Homo_633,totalSample,immune-NKT; Homo_633,totalSample,immune-Th1; Homo_633,totalSample,immune-Gamma_delta; Homo_633,totalSample,immune-Th17; Homo_633,totalSample,immune-Cytotoxic; Homo_633,totalSample,immune-Macrophage; Homo_633,totalSample,immune-Monocyte; Homo_633,totalSample,immune-Neutrophil; MCSA,totalSample,immune-Bcell; MCSA,totalSample,immune-Monocyte; MCSA,totalSample,immune-Macrophage; MCSA,totalSample,immune-Neutrophil; MCSA,totalSample,immune-CD4_T; MCSA,totalSample,immune-Gamma_delta; MCSA,totalSample,immune-CD4_naive; MCSA,totalSample,immune-Tr1; MCSA,totalSample,immune-nTreg; MCSA,totalSample,immune-iTreg; MCSA,totalSample,immune-Th17; MCSA,totalSample,immune-Tfh; MCSA,totalSample,immune-CD8_naive; MCSA,totalSample,immune-Cytotoxic; MCSA,totalSample,immune-Exhausted; MCSA,totalSample,immune-Central_memory; MCSA,totalSample,immune-Effector_memory; ROSMAP,totalSample,immune-InfiltrationScore; SRP223445,totalSample,immune-Th17; SRP223445,totalSample,immune-Exhausted
RPIA	Emory,control,male.vs.female; Homo_723,male,MCI.vs.control; Homo_723,MCI,male.vs.female; Homo_714,male,MCI.vs.control; Homo_714,MCI,male.vs.female; Homo_633,male,MCI.vs.control; ROSMAP,female,AD.vs.control; Emory,totalSample,age; Emory,MCI,age; ROSMAP,MCI,last.visit.age; ROSMAP,MCI,sample.age; Emory,totalSample,immune-Neutrophil; Emory,totalSample,immune-CD8_T; Emory,totalSample,immune-Gamma_delta; Emory,totalSample,immune-CD8_naive; Emory,totalSample,immune-Cytotoxic; Homo_723,totalSample,immune-Bcell; Homo_723,totalSample,immune-Neutrophil; Homo_723,totalSample,immune-Th1; Homo_714,totalSample,immune-Neutrophil; Homo_633,totalSample,immune-Th1; Homo_633,totalSample,immune-Neutrophil; MCSA,totalSample,immune-DC; MCSA,totalSample,immune-Monocyte; MCSA,totalSample,immune-CD4_T; MCSA,totalSample,immune-CD4_naive; MCSA,totalSample,immune-Tr1; MCSA,totalSample,immune-nTreg; MCSA,totalSample,immune-iTreg; MCSA,totalSample,immune-Tfh; MCSA,totalSample,immune-CD8_naive; MCSA,totalSample,immune-Cytotoxic; MCSA,totalSample,immune-Exhausted; ROSMAP,totalSample,immune-Th17; SRP223445,totalSample,immune-CD4_naive; SRP223445,totalSample,immune-Th17; SRP223445,totalSample,immune-InfiltrationScore; ROSMAP,male and female-includeAge90+,survival
SMIM1	Emory,control,male.vs.female; Homo_723,female,AD.vs.MCI; Homo_723,female,MCI.vs.control; Homo_714,female,AD.vs.MCI; Homo_714,female,MCI.vs.control; Homo_633,female,AD.vs.control; Homo_633,female,AD.vs.MCI; Homo_633,female,MCI.vs.control; ACOM,totalSample,age; Emory,MCI,age; MCSA,MCI,age; Emory,totalSample,immune-Gamma_delta; Homo_723,totalSample,immune-DC; Homo_723,totalSample,immune-Th2; Homo_723,totalSample,immune-Exhausted; Homo_714,totalSample,immune-DC; Homo_714,totalSample,immune-Th2; Homo_633,totalSample,immune-DC; Homo_633,totalSample,immune-Th2; MCSA,totalSample,immune-DC; MCSA,totalSample,immune-Monocyte; MCSA,totalSample,immune-Tr1; MCSA,totalSample,immune-iTreg; MCSA,totalSample,immune-Th2; MCSA,totalSample,immune-Cytotoxic
SVBP	Emory,control,male.vs.female; Homo_723,male,MCI.vs.control; Homo_723,control,male.vs.female; Homo_714,male,MCI.vs.control; Homo_714,control,male.vs.female; Homo_633,male,MCI.vs.control; Homo_633,control,male.vs.female; Emory,totalSample,immune-Gamma_delta; Emory,totalSample,immune-Th17; Emory,totalSample,immune-Cytotoxic; Homo_723,totalSample,immune-Effector_memory; Homo_723,totalSample,immune-Bcell; Homo_723,totalSample,immune-Th2; Homo_723,totalSample,immune-iTreg; Homo_723,totalSample,immune-Tr1; Homo_723,totalSample,immune-Central_memory; Homo_723,totalSample,immune-CD4_naive; Homo_723,totalSample,immune-CD4_T; Homo_714,totalSample,immune-Th2; Homo_714,totalSample,immune-Effector_memory; Homo_714,totalSample,immune-CD4_T; Homo_714,totalSample,immune-Central_memory; Homo_714,totalSample,immune-iTreg; Homo_714,totalSample,immune-Tr1; Homo_633,totalSample,immune-Effector_memory; Homo_633,totalSample,immune-Th2; Homo_633,totalSample,immune-CD4_T; Homo_633,totalSample,immune-Central_memory; Homo_633,totalSample,immune-CD4_naive; Homo_633,totalSample,immune-Exhausted; Homo_633,totalSample,immune-Tr1; Homo_633,totalSample,immune-nTreg; MCSA,totalSample,immune-DC; MCSA,totalSample,immune-Monocyte; MCSA,totalSample,immune-Th2; MCSA,totalSample,immune-Central_memory;
FAM210B	Emory,control,male.vs.female; Emory,MCI,age; Emory,totalSample,immune-Gamma_delta; Emory,totalSample,immune-Th17; Emory,totalSample,immune-CD8_naive; Homo_723,totalSample,immune-Bcell; Homo_714,totalSample,immune-Bcell; Homo_633,totalSample,immune-Bcell; Homo_633,totalSample,immune-Monocyte; ROSMAP,totalSample,immune-Th17; SRP223445,totalSample,immune-InfiltrationScore; ROSMAP,male-includeAge90+,survival
SLC1A5	Emory,control,male.vs.female; Homo_723,female,AD.vs.control; Homo_723,male,AD.vs.control; Homo_723,female,AD.vs.MCI; Homo_723,male,AD.vs.MCI; Homo_723,female,MCI.vs.control; Homo_723,control,male.vs.female; Homo_714,female,AD.vs.control; Homo_714,male,AD.vs.control; Homo_714,female,AD.vs.MCI; Homo_714,male,AD.vs.MCI; Homo_714,control,male.vs.female; Homo_633,female,AD.vs.control; Homo_633,female,AD.vs.MCI; Homo_633,male,MCI.vs.control; ROSMAP,female,AD.vs.control; Emory,totalSample,age; Emory,MCI,age; MCSA,MCI,age; Emory,totalSample,immune-Neutrophil; Emory,totalSample,immune-Gamma_delta; Emory,totalSample,immune-CD8_naive; Emory,totalSample,immune-Cytotoxic; Homo_723,totalSample,immune-DC; Homo_723,totalSample,immune-Cytotoxic; Homo_723,totalSample,immune-Neutrophil; Homo_723,totalSample,immune-Bcell; Homo_723,totalSample,immune-Exhausted; Homo_723,totalSample,immune-Th2; Homo_714,totalSample,immune-DC; Homo_714,totalSample,immune-Th2; Homo_633,totalSample,immune-DC; Homo_633,totalSample,immune-Th2; MCSA,totalSample,immune-DC; MCSA,totalSample,immune-Monocyte; MCSA,totalSample,immune-Macrophage; MCSA,totalSample,immune-Neutrophil; MCSA,totalSample,immune-CD8_T; MCSA,totalSample,immune-iTreg; MCSA,totalSample,immune-Th2; MCSA,totalSample,immune-Cytotoxic; MCSA,totalSample,immune-Effector_memory; MCSA,totalSample,immune-InfiltrationScore; ROSMAP,totalSample,immune-Cytotoxic; ROSMAP,totalSample,immune-MAIT; ROSMAP,totalSample,immune-Effector_memory; SRP223445,totalSample,immune-Neutrophil; SRP223445,totalSample,immune-nTreg; SRP223445,totalSample,immune-InfiltrationScore; ROSMAP,female-excludeAge90+,survival; ROSMAP,female-includeAge90+,survival; ROSMAP,male and female-excludeAge90+,survival; ROSMAP,male and female-includeAge90+,survival
C9orf78	Emory,control,male.vs.female; ROSMAP,female,AD.vs.control; Emory,totalSample,age; Emory,MCI,age; ROSMAP,MCI,last.visit.age; Emory,totalSample,immune-Neutrophil; Emory,totalSample,immune-Gamma_delta; Emory,totalSample,immune-Th17; Emory,totalSample,immune-CD8_naive; Emory,totalSample,immune-Cytotoxic; Homo_723,totalSample,immune-Bcell; Homo_714,totalSample,immune-Bcell; Homo_633,totalSample,immune-Bcell; SRP223445,totalSample,immune-CD4_naive; SRP223445,totalSample,immune-Th17; SRP223445,totalSample,immune-Exhausted; SRP223445,totalSample,immune-InfiltrationScore
CHST13	Emory,control,male.vs.female; Homo_723,female,AD.vs.control; Homo_723,male,AD.vs.MCI; Homo_723,control,male.vs.female; Homo_714,female,AD.vs.control; Homo_714,male,MCI.vs.control; Homo_714,control,male.vs.female; Homo_633,male,MCI.vs.control; Homo_633,control,male.vs.female; Homo_633,MCI,male.vs.female; ROSMAP,female,MCI.vs.control; Emory,totalSample,age; Emory,MCI,age; MCSA,MCI,age; ROSMAP,MCI,sample.age; Emory,totalSample,immune-Macrophage; Emory,totalSample,immune-Neutrophil; Emory,totalSample,immune-CD8_T; Emory,totalSample,immune-Gamma_delta; Emory,totalSample,immune-Tfh; Emory,totalSample,immune-CD8_naive; Emory,totalSample,immune-Exhausted; Emory,totalSample,immune-InfiltrationScore; Homo_723,totalSample,immune-DC; Homo_723,totalSample,immune-Monocyte; Homo_714,totalSample,immune-DC; Homo_633,totalSample,immune-Monocyte; Homo_633,totalSample,immune-Th2; MCSA,totalSample,immune-DC; MCSA,totalSample,immune-Monocyte; MCSA,totalSample,immune-Macrophage; MCSA,totalSample,immune-CD8_T; MCSA,totalSample,immune-iTreg; MCSA,totalSample,immune-Th2; MCSA,totalSample,immune-Cytotoxic; MCSA,totalSample,immune-InfiltrationScore; ROSMAP,totalSample,immune-Effector_memory
CDC34	Emory,control,male.vs.female; Homo_723,female,AD.vs.control; Homo_723,male,AD.vs.control; Homo_723,female,AD.vs.MCI; Homo_723,male,AD.vs.MCI; Homo_723,control,male.vs.female; Homo_714,female,AD.vs.control; Homo_714,male,AD.vs.control; Homo_714,female,AD.vs.MCI; Homo_714,male,AD.vs.MCI; Homo_714,control,male.vs.female; ROSMAP,female,AD.vs.control; ROSMAP,female,AD.vs.MCI; ROSMAP,female,MCI.vs.control; Emory,totalSample,age; Emory,MCI,age; MCSA,MCI,age; Emory,totalSample,immune-Gamma_delta; Emory,totalSample,immune-Tr1; Emory,totalSample,immune-iTreg; Emory,totalSample,immune-CD8_naive; Homo_723,totalSample,immune-DC; Homo_723,totalSample,immune-Bcell; Homo_723,totalSample,immune-Th2; Homo_723,totalSample,immune-Cytotoxic; Homo_723,totalSample,immune-Tr1; Homo_723,totalSample,immune-iTreg; Homo_723,totalSample,immune-CD4_T; Homo_714,totalSample,immune-DC; Homo_714,totalSample,immune-Th2; Homo_714,totalSample,immune-Bcell; Homo_714,totalSample,immune-CD4_T; Homo_714,totalSample,immune-CD4_naive; Homo_714,totalSample,immune-iTreg; Homo_714,totalSample,immune-Tr1; Homo_633,totalSample,immune-DC; Homo_633,totalSample,immune-Th2; Homo_633,totalSample,immune-Bcell; Homo_633,totalSample,immune-CD4_T; Homo_633,totalSample,immune-NKT; Homo_633,totalSample,immune-CD4_naive; MCSA,totalSample,immune-DC; MCSA,totalSample,immune-Monocyte; MCSA,totalSample,immune-Macrophage; MCSA,totalSample,immune-CD4_T; MCSA,totalSample,immune-CD8_T; MCSA,totalSample,immune-NKT; MCSA,totalSample,immune-Tr1; MCSA,totalSample,immune-iTreg; MCSA,totalSample,immune-Th2;
BLVRB	Emory,control,male.vs.female; ROSMAP,female,AD.vs.control; ROSMAP,female,MCI.vs.control; Emory,totalSample,age; Emory,MCI,age; MCSA,MCI,age; Emory,totalSample,immune-Gamma_delta; Emory,totalSample,immune-Tr1; Emory,totalSample,immune-iTreg; Emory,totalSample,immune-CD8_naive; Emory,totalSample,immune-Cytotoxic; Homo_723,totalSample,immune-Bcell; Homo_723,totalSample,immune-Cytotoxic; Homo_723,totalSample,immune-DC; Homo_723,totalSample,immune-Th2; Homo_723,totalSample,immune-Tr1; Homo_723,totalSample,immune-Th1; Homo_723,totalSample,immune-CD4_naive; Homo_723,totalSample,immune-iTreg; Homo_723,totalSample,immune-CD4_T; Homo_723,totalSample,immune-Neutrophil; Homo_714,totalSample,immune-Bcell; Homo_714,totalSample,immune-DC; Homo_714,totalSample,immune-Th2; Homo_714,totalSample,immune-CD4_naive; Homo_714,totalSample,immune-CD4_T; Homo_714,totalSample,immune-Th1; Homo_714,totalSample,immune-Cytotoxic; Homo_714,totalSample,immune-iTreg; Homo_714,totalSample,immune-NKT; Homo_714,totalSample,immune-Tr1; Homo_633,totalSample,immune-DC; Homo_633,totalSample,immune-Bcell; Homo_633,totalSample,immune-Th2; Homo_633,totalSample,immune-NKT; Homo_633,totalSample,immune-CD4_T; Homo_633,totalSample,immune-CD4_naive; Homo_633,totalSample,immune-Th1; Homo_633,totalSample,immune-iTreg; Homo_633,totalSample,immune-Cytotoxic; Homo_633,totalSample,immune-Tfh; Homo_633,totalSample,immune-Tr1; MCSA,totalSample,immune-DC; MCSA,totalSample,immune-Monocyte; MCSA,totalSample,immune-Macrophage; MCSA,totalSample,immune-Neutrophil; MCSA,totalSample,immune-CD4_T; MCSA,totalSample,immune-CD8_T; MCSA,totalSample,immune-NKT; MCSA,totalSample,immune-Tr1; MCSA,totalSample,immune-iTreg; MCSA,totalSample,immune-Th2; MCSA,totalSample,immune-Cytotoxic; MCSA,totalSample,immune-Effector_memory; ROSMAP,totalSample,immune-DC; ROSMAP,totalSample,immune-CD4_T; ROSMAP,totalSample,immune-iTreg; ROSMAP,totalSample,immune-Th1; ROSMAP,totalSample,immune-Th2; ROSMAP,totalSample,immune-Th17; ROSMAP,totalSample,immune-Effector_memory; ROSMAP,totalSample,immune-InfiltrationScore; SRP223445,totalSample,immune-CD4_naive; SRP223445,totalSample,immune-InfiltrationScore; ROSMAP,female-includeAge90+,survival
PRDX6	Emory,control,male.vs.female; Homo_723,female,AD.vs.control; Homo_723,male,AD.vs.control; Homo_723,control,male.vs.female; Homo_714,female,AD.vs.control; Homo_714,male,AD.vs.control; Homo_714,female,MCI.vs.control; Homo_714,male,MCI.vs.control; Homo_714,control,male.vs.female; Homo_633,female,MCI.vs.control; Homo_633,control,male.vs.female; SRP223445,female,AD.vs.control; Emory,MCI,age; Emory,totalSample,immune-Neutrophil; Emory,totalSample,immune-Gamma_delta; Emory,totalSample,immune-Tr1; Emory,totalSample,immune-iTreg; Emory,totalSample,immune-CD8_naive; Homo_723,totalSample,immune-Bcell; Homo_714,totalSample,immune-Bcell; Homo_714,totalSample,immune-iTreg; Homo_714,totalSample,immune-DC; Homo_633,totalSample,immune-Bcell; Homo_633,totalSample,immune-DC; SRP223445,totalSample,immune-CD4_naive; SRP223445,totalSample,immune-Exhausted
R3HDM4	Emory,control,male.vs.female; ROSMAP,male,AD.vs.control; Emory,MCI,age; MCSA,totalSample,age; MCSA,MCI,age; Emory,totalSample,immune-CD4_T; Emory,totalSample,immune-Tr1; Emory,totalSample,immune-iTreg; Emory,totalSample,immune-InfiltrationScore; Homo_723,totalSample,immune-DC; Homo_723,totalSample,immune-Th1; Homo_723,totalSample,immune-Bcell; Homo_714,totalSample,immune-DC; Homo_714,totalSample,immune-Th1; Homo_714,totalSample,immune-Bcell; Homo_714,totalSample,immune-CD4_naive; Homo_633,totalSample,immune-DC; Homo_633,totalSample,immune-Th1; Homo_633,totalSample,immune-Bcell; Homo_633,totalSample,immune-CD4_naive; Homo_633,totalSample,immune-NKT; Homo_633,totalSample,immune-CD4_T; MCSA,totalSample,immune-DC; MCSA,totalSample,immune-Monocyte; MCSA,totalSample,immune-Macrophage; MCSA,totalSample,immune-CD4_T; MCSA,totalSample,immune-CD8_T; MCSA,totalSample,immune-Tr1; MCSA,totalSample,immune-iTreg; MCSA,totalSample,immune-Th2;
UBA52	Emory,control,male.vs.female; ROSMAP,female,AD.vs.control; Emory,MCI,age; MCSA,MCI,age; ROSMAP,MCI,last.visit.age; Emory,totalSample,immune-Monocyte; Emory,totalSample,immune-Neutrophil; Emory,totalSample,immune-Gamma_delta; Emory,totalSample,immune-Tr1; Emory,totalSample,immune-iTreg; Emory,totalSample,immune-CD8_naive; Homo_723,totalSample,immune-Bcell; Homo_714,totalSample,immune-Bcell; Homo_714,totalSample,immune-DC; Homo_633,totalSample,immune-Bcell; Homo_633,totalSample,immune-DC; MCSA,totalSample,immune-DC; MCSA,totalSample,immune-Monocyte; MCSA,totalSample,immune-CD8_T; MCSA,totalSample,immune-iTreg; MCSA,totalSample,immune-Th2; MCSA,totalSample,immune-Cytotoxic; ROSMAP,totalSample,immune-CD4_T; ROSMAP,totalSample,immune-Th2; ROSMAP,totalSample,immune-Th17; SRP223445,totalSample,immune-Neutrophil; SRP223445,totalSample,immune-CD4_naive; SRP223445,totalSample,immune-Th17; SRP223445,totalSample,immune-Exhausted; SRP223445,totalSample,immune-
ERFE	Emory,control,male.vs.female; Homo_723,female,AD.vs.control; Homo_723,male,AD.vs.control; Homo_723,female,MCI.vs.control; Homo_723,male,MCI.vs.control; Homo_714,female,AD.vs.control; Homo_714,female,MCI.vs.control; Homo_714,male,MCI.vs.control; Homo_633,female,AD.vs.control; Homo_633,female,AD.vs.MCI; Homo_633,female,MCI.vs.control; ROSMAP,female,AD.vs.control; MCSA,MCI,age; Homo_723,totalSample,immune-DC; Homo_714,totalSample,immune-DC; Homo_633,totalSample,immune-DC; Homo_633,totalSample,immune-NK; MCSA,totalSample,immune-DC; MCSA,totalSample,immune-Monocyte; SRP223445,totalSample,immune-Monocyte
SESN3	Emory,control,male.vs.female; Homo_723,female,AD.vs.control; Homo_723,male,AD.vs.control; Homo_723,male,AD.vs.MCI; Homo_723,control,male.vs.female; Homo_714,male,AD.vs.control; Homo_714,male,AD.vs.MCI; Homo_714,control,male.vs.female; Homo_633,control,male.vs.female; Homo_633,MCI,male.vs.female; Emory,totalSample,age; Emory,MCI,age; MCSA,MCI,age; ROSMAP,AD,MMSE; ROSMAP,MCI,MMSE; Emory,totalSample,immune-CD8_T; Emory,totalSample,immune-Gamma_delta; Emory,totalSample,immune-CD8_naive; Homo_723,totalSample,immune-Bcell; Homo_723,totalSample,immune-Th1; Homo_714,totalSample,immune-Th1; Homo_633,totalSample,immune-Th1; MCSA,totalSample,immune-DC; MCSA,totalSample,immune-Bcell; MCSA,totalSample,immune-Monocyte; MCSA,totalSample,immune-CD4_T; MCSA,totalSample,immune-CD8_T; MCSA,totalSample,immune-Tr1; MCSA,totalSample,immune-iTreg; MCSA,totalSample,immune-CD8_naive; MCSA,totalSample,immune-Cytotoxic; MCSA,totalSample,immune-Exhausted; MCSA,totalSample,immune-Effector_memory; MCSA,totalSample,immune-InfiltrationScore; ROSMAP,totalSample,immune-Effector_memory; SRP223445,totalSample,immune-NK; SRP223445,totalSample,immune-Exhausted; SRP223445,totalSample,immune-InfiltrationScore
HPS1	Emory,control,male.vs.female; Emory,MCI,age; MCSA,totalSample,age; MCSA,MCI,age; ROSMAP,MCI,sample.age; Emory,totalSample,immune-Gamma_delta; Emory,totalSample,immune-Tr1; Emory,totalSample,immune-iTreg; Emory,totalSample,immune-CD8_naive; Homo_723,totalSample,immune-DC; Homo_723,totalSample,immune-Bcell; Homo_714,totalSample,immune-DC; Homo_714,totalSample,immune-Bcell; Homo_633,totalSample,immune-DC; Homo_633,totalSample,immune-Bcell; Homo_633,totalSample,immune-Th1; MCSA,totalSample,immune-DC; MCSA,totalSample,immune-Monocyte; MCSA,totalSample,immune-Macrophage; MCSA,totalSample,immune-CD8_T; MCSA,totalSample,immune-iTreg; MCSA,totalSample,immune-Th2; MCSA,totalSample,immune-Cytotoxic; MCSA,totalSample,immune-MAIT; MCSA,totalSample,immune-Effector_memory; MCSA,totalSample,immune-InfiltrationScore; ROSMAP,totalSample,immune-Effector_memory; SRP223445,totalSample,immune-InfiltrationScore
ANKRD9	Emory,control,male.vs.female; ROSMAP,female,AD.vs.control; ROSMAP,female,MCI.vs.control; Emory,MCI,age; MCSA,MCI,age; Emory,totalSample,immune-Gamma_delta; Emory,totalSample,immune-Tr1; Emory,totalSample,immune-iTreg; Homo_723,totalSample,immune-Bcell; Homo_723,totalSample,immune-DC; Homo_714,totalSample,immune-DC; Homo_714,totalSample,immune-Bcell; Homo_633,totalSample,immune-Bcell; Homo_633,totalSample,immune-DC; MCSA,totalSample,immune-DC; MCSA,totalSample,immune-Monocyte; MCSA,totalSample,immune-Macrophage; MCSA,totalSample,immune-NKT; MCSA,totalSample,immune-Th2; MCSA,totalSample,immune-Effector_memory;
RAP1GAP	Emory,control,male.vs.female; Homo_723,male,AD.vs.control; Homo_723,male,AD.vs.MCI; Homo_723,AD,male.vs.female; Homo_714,female,AD.vs.control; Homo_714,male,AD.vs.control; Homo_714,female,AD.vs.MCI; Homo_714,male,AD.vs.MCI; Homo_633,female,AD.vs.control; Homo_633,female,AD.vs.MCI; Homo_633,female,MCI.vs.control; Homo_633,control,male.vs.female; ROSMAP,female,AD.vs.control; MCSA,MCI,age
PLAU	Emory,control,male.vs.female; Homo_723,female,AD.vs.control; Homo_723,male,AD.vs.MCI; Homo_714,female,AD.vs.control; Homo_714,male,AD.vs.control; Homo_714,male,AD.vs.MCI; Homo_633,female,AD.vs.control; Homo_633,female,AD.vs.MCI; ROSMAP,MCI,last.visit.age; ROSMAP,MCI,sample.age; Emory,totalSample,immune-Macrophage; Emory,totalSample,immune-NK; Emory,totalSample,immune-Neutrophil; Emory,totalSample,immune-CD8_T; Emory,totalSample,immune-Gamma_delta; Emory,totalSample,immune-Th1; Emory,totalSample,immune-Th17; Emory,totalSample,immune-Tfh; Emory,totalSample,immune-CD8_naive; Emory,totalSample,immune-Cytotoxic; Emory,totalSample,immune-Exhausted; Homo_723,totalSample,immune-Neutrophil; Homo_723,totalSample,immune-Gamma_delta; Homo_714,totalSample,immune-Neutrophil; Homo_714,totalSample,immune-Gamma_delta; Homo_714,totalSample,immune-Exhausted; Homo_633,totalSample,immune-Neutrophil; Homo_633,totalSample,immune-CD8_T; Homo_633,totalSample,immune-Gamma_delta; Homo_633,totalSample,immune-Exhausted; Homo_633,totalSample,immune-MAIT; Homo_633,totalSample,immune-Macrophage; MCSA,totalSample,immune-Macrophage; MCSA,totalSample,immune-Neutrophil; MCSA,totalSample,immune-CD8_T; MCSA,totalSample,immune-Gamma_delta; MCSA,totalSample,immune-Th1; MCSA,totalSample,immune-Th17;
GRINA	Emory,control,male.vs.female; Emory,MCI,age; MCSA,MCI,age; Emory,totalSample,immune-Monocyte; Emory,totalSample,immune-Gamma_delta; Emory,totalSample,immune-Tr1; Emory,totalSample,immune-iTreg; Homo_723,totalSample,immune-DC; Homo_723,totalSample,immune-Th1; Homo_723,totalSample,immune-Cytotoxic; Homo_723,totalSample,immune-Bcell; Homo_723,totalSample,immune-Th2; Homo_723,totalSample,immune-Tr1; Homo_714,totalSample,immune-DC; Homo_714,totalSample,immune-Th1; Homo_714,totalSample,immune-CD4_naive; Homo_714,totalSample,immune-Bcell; Homo_714,totalSample,immune-CD4_T; Homo_714,totalSample,immune-Th2; Homo_714,totalSample,immune-iTreg; Homo_633,totalSample,immune-DC; Homo_633,totalSample,immune-Th1; Homo_633,totalSample,immune-NKT; Homo_633,totalSample,immune-CD4_naive; Homo_633,totalSample,immune-Bcell; Homo_633,totalSample,immune-CD4_T; Homo_633,totalSample,immune-Th2; MCSA,totalSample,immune-DC; MCSA,totalSample,immune-Monocyte; MCSA,totalSample,immune-Macrophage; MCSA,totalSample,immune-CD4_T; MCSA,totalSample,immune-CD8_T; MCSA,totalSample,immune-CD4_naive; MCSA,totalSample,immune-Tr1; MCSA,totalSample,immune-nTreg; MCSA,totalSample,immune-iTreg; MCSA,totalSample,immune-Th2; MCSA,totalSample,immune-Tfh; MCSA,totalSample,immune-Cytotoxic;
TPGS2	Emory,control,male.vs.female; Homo_723,female,AD.vs.control; Homo_723,control,male.vs.female; Homo_714,female,AD.vs.control; Homo_714,male,AD.vs.control; Homo_714,female,AD.vs.MCI; Homo_714,control,male.vs.female; Homo_633,female,AD.vs.control; Homo_633,female,AD.vs.MCI; Homo_633,female,MCI.vs.control; Homo_633,male,MCI.vs.control; Homo_633,control,male.vs.female; Homo_633,MCI,male.vs.female; ROSMAP,female,AD.vs.control; ROSMAP,female,MCI.vs.control; Emory,MCI,age; ROSMAP,AD,MMSE; Emory,totalSample,immune-Neutrophil; Emory,totalSample,immune-CD8_T; Emory,totalSample,immune-Gamma_delta; Emory,totalSample,immune-Th17; Emory,totalSample,immune-CD8_naive; Emory,totalSample,immune-Cytotoxic; Homo_723,totalSample,immune-Bcell; Homo_723,totalSample,immune-Neutrophil; Homo_633,totalSample,immune-Th2; MCSA,totalSample,immune-CD4_T; MCSA,totalSample,immune-CD4_naive; MCSA,totalSample,immune-Tr1; MCSA,totalSample,immune-nTreg; MCSA,totalSample,immune-Tfh; MCSA,totalSample,immune-CD8_naive; ROSMAP,totalSample,immune-Effector_memory; SRP223445,totalSample,immune-InfiltrationScore
PPM1A	Emory,control,male.vs.female; Homo_633,male,MCI.vs.control; ROSMAP,female,AD.vs.control; Emory,totalSample,age; Emory,MCI,age; MCSA,MCI,age; Emory,totalSample,immune-DC; Emory,totalSample,immune-Monocyte; Homo_723,totalSample,immune-Monocyte; Homo_723,totalSample,immune-Bcell; Homo_723,totalSample,immune-DC; Homo_723,totalSample,immune-Gamma_delta; Homo_714,totalSample,immune-Monocyte; Homo_633,totalSample,immune-Monocyte; Homo_633,totalSample,immune-DC; Homo_633,totalSample,immune-Bcell; MCSA,totalSample,immune-DC; MCSA,totalSample,immune-Monocyte; MCSA,totalSample,immune-Neutrophil; MCSA,totalSample,immune-CD8_T; MCSA,totalSample,immune-Tr1; MCSA,totalSample,immune-iTreg; MCSA,totalSample,immune-CD8_naive; MCSA,totalSample,immune-Cytotoxic; MCSA,totalSample,immune-Exhausted; MCSA,totalSample,immune-Effector_memory; MCSA,totalSample,immune-InfiltrationScore; SRP223445,totalSample,immune-CD4_naive; SRP223445,totalSample,immune-Exhausted

FKBP1B	Emory,control,male.vs.female; Homo_723,female,AD.vs.control; Homo_714,female,AD.vs.control; ROSMAP,male,AD.vs.control; SRP223445,female,AD.vs.control; Emory,MCI,age; Emory,totalSample,immune-Gamma_delta; Emory,totalSample,immune-Tr1; Emory,totalSample,immune-iTreg; Emory,totalSample,immune-Th17; Emory,totalSample,immune-CD8_naive; Emory,totalSample,immune-Cytotoxic; Emory,totalSample,immune-Central_memory; Homo_723,totalSample,immune-Bcell; Homo_723,totalSample,immune-CD4_naive; Homo_723,totalSample,immune-Th2; Homo_723,totalSample,immune-iTreg; Homo_723,totalSample,immune-CD4_T; Homo_723,totalSample,immune-Tr1; Homo_714,totalSample,immune-Bcell; Homo_714,totalSample,immune-CD4_naive; Homo_714,totalSample,immune-Th2; Homo_714,totalSample,immune-iTreg; Homo_714,totalSample,immune-CD4_T; Homo_714,totalSample,immune-Tfh; Homo_714,totalSample,immune-Effector_memory; Homo_633,totalSample,immune-Bcell; Homo_633,totalSample,immune-Th2; Homo_633,totalSample,immune-CD4_naive; Homo_633,totalSample,immune-CD4_T; Homo_633,totalSample,immune-iTreg; Homo_633,totalSample,immune-NKT; Homo_633,totalSample,immune-Tr1; Homo_633,totalSample,immune-Tfh; Homo_633,totalSample,immune-DC; Homo_633,totalSample,immune-Effector_memory; Homo_633,totalSample,immune-nTreg; Homo_633,totalSample,immune-Cytotoxic; MCSA,totalSample,immune-CD4_T; MCSA,totalSample,immune-Gamma_delta; MCSA,totalSample,immune-CD4_naive; MCSA,totalSample,immune-Tr1; MCSA,totalSample,immune-nTreg; MCSA,totalSample,immune-iTreg; MCSA,totalSample,immune-Th17; MCSA,totalSample,immune-Tfh; MCSA,totalSample,immune-Central_memory; ROSMAP,totalSample,immune-InfiltrationScore; SRP223445,totalSample,immune-CD4_naive; SRP223445,totalSample,immune-Exhausted; ROSMAP,male-excludeAge90+,survival; ROSMAP,male and female-includeAge90+,survival
CA6	Emory,control,male.vs.female; Homo_723,female,AD.vs.control; Homo_723,male,AD.vs.control; Homo_723,female,AD.vs.MCI; Homo_723,male,AD.vs.MCI; Homo_723,female,MCI.vs.control; Homo_714,female,AD.vs.control; Homo_714,male,AD.vs.control; Homo_714,female,AD.vs.MCI; Homo_714,male,AD.vs.MCI; Homo_714,female,MCI.vs.control; Homo_633,female,AD.vs.control; Homo_633,female,AD.vs.MCI; Homo_633,female,MCI.vs.control; Emory,MCI,age; MCSA,totalSample,age; ROSMAP,MCI,last.visit.age; Emory,totalSample,immune-Neutrophil; Emory,totalSample,immune-CD4_T; Emory,totalSample,immune-CD8_T; Emory,totalSample,immune-CD4_naive; Emory,totalSample,immune-Tr1; Emory,totalSample,immune-nTreg; Emory,totalSample,immune-iTreg; Emory,totalSample,immune-Tfh; Emory,totalSample,immune-CD8_naive; Emory,totalSample,immune-Cytotoxic; Emory,totalSample,immune-MAIT; Emory,totalSample,immune-Central_memory; Homo_723,totalSample,immune-Tfh; Homo_723,totalSample,immune-iTreg; Homo_723,totalSample,immune-Th2; Homo_723,totalSample,immune-Tr1; Homo_723,totalSample,immune-CD4_T; Homo_723,totalSample,immune-Central_memory; Homo_723,totalSample,immune-CD4_naive; Homo_723,totalSample,immune-CD8_T; Homo_723,totalSample,immune-nTreg; Homo_723,totalSample,immune-Gamma_delta; Homo_723,totalSample,immune-DC; Homo_723,totalSample,immune-Effector_memory; Homo_723,totalSample,immune-Macrophage; Homo_723,totalSample,immune-CD8_naive; Homo_714,totalSample,immune-Tfh; Homo_714,totalSample,immune-Th2; Homo_714,totalSample,immune-CD4_T; Homo_714,totalSample,immune-Tr1; Homo_714,totalSample,immune-Central_memory; Homo_714,totalSample,immune-nTreg; Homo_714,totalSample,immune-iTreg; Homo_714,totalSample,immune-CD4_naive; Homo_714,totalSample,immune-Effector_memory; Homo_714,totalSample,immune-Gamma_delta; Homo_714,totalSample,immune-CD8_T; Homo_714,totalSample,immune-CD8_naive; Homo_633,totalSample,immune-Tfh; Homo_633,totalSample,immune-Central_memory; Homo_633,totalSample,immune-CD4_T; Homo_633,totalSample,immune-Tr1; Homo_633,totalSample,immune-nTreg; Homo_633,totalSample,immune-CD4_naive; Homo_633,totalSample,immune-Th2; Homo_633,totalSample,immune-iTreg; Homo_633,totalSample,immune-Gamma_delta; Homo_633,totalSample,immune-Effector_memory; Homo_633,totalSample,immune-Th17; MCSA,totalSample,immune-Monocyte; MCSA,totalSample,immune-CD4_T; MCSA,totalSample,immune-Gamma_delta; MCSA,totalSample,immune-CD4_naive; MCSA,totalSample,immune-Tr1; MCSA,totalSample,immune-nTreg; MCSA,totalSample,immune-iTreg; MCSA,totalSample,immune-Th17; [illegible]
SIAH2	Emory,control,male.vs.female; ACOM,totalSample,age; Emory,totalSample,age; Emory,MCI,age; Emory,totalSample,immune-Gamma_delta; Emory,totalSample,immune-iTreg; Emory,totalSample,immune-CD8_naive; Emory,totalSample,immune-Cytotoxic; Homo_723,totalSample,immune-Bcell; Homo_714,totalSample,immune-Bcell; Homo_633,totalSample,immune-Bcell; SRP223445,totalSample,immune-Exhausted
HBB	Emory,control,male.vs.female; ROSMAP,female,AD.vs.control; ROSMAP,control,male.vs.female; Emory,totalSample,age; Emory,MCI,age; ROSMAP,AD,last.visit.age; ROSMAP,AD,sample.age; ROSMAP,totalSample,age; ROSMAP,MCI,sample.age; Emory,totalSample,immune-CD8_T; Emory,totalSample,immune-Gamma_delta; Emory,totalSample,immune-CD8_naive; Homo_723,totalSample,immune-Bcell; Homo_714,totalSample,immune-Bcell; Homo_633,totalSample,immune-Bcell; ROSMAP,totalSample,immune-InfiltrationScore
GBGT1	Emory,control,male.vs.female; ROSMAP,male,AD.vs.control; MCSA,MCI,age; ROSMAP,MCI,last.visit.age; ROSMAP,MCI,sample.age; Emory,totalSample,immune-CD4_T; Emory,totalSample,immune-Gamma_delta; Emory,totalSample,immune-Tr1; Emory,totalSample,immune-iTreg; Emory,totalSample,immune-Central_memory; Homo_723,totalSample,immune-DC; Homo_723,totalSample,immune-Th1; Homo_723,totalSample,immune-Tr1; Homo_723,totalSample,immune-Cytotoxic; Homo_723,totalSample,immune-Th2; Homo_723,totalSample,immune-CD4_T; Homo_723,totalSample,immune-iTreg; Homo_723,totalSample,immune-CD4_naive; Homo_723,totalSample,immune-Bcell; Homo_714,totalSample,immune-DC; Homo_714,totalSample,immune-Th1; Homo_714,totalSample,immune-CD4_T; Homo_714,totalSample,immune-CD4_naive; Homo_714,totalSample,immune-Th2; Homo_714,totalSample,immune-iTreg; Homo_714,totalSample,immune-Tr1; Homo_714,totalSample,immune-Bcell; Homo_714,totalSample,immune-Tfh; Homo_714,totalSample,immune-NKT; Homo_633,totalSample,immune-DC; Homo_633,totalSample,immune-Th1; Homo_633,totalSample,immune-CD4_naive; Homo_633,totalSample,immune-CD4_T; Homo_633,totalSample,immune-Th2; Homo_633,totalSample,immune-NKT; Homo_633,totalSample,immune-Bcell; Homo_633,totalSample,immune-Tfh; Homo_633,totalSample,immune-Tr1; Homo_633,totalSample,immune-iTreg; Homo_633,totalSample,immune-Cytotoxic; MCSA,totalSample,immune-DC; MCSA,totalSample,immune-Monocyte; MCSA,totalSample,immune-Macrophage; MCSA,totalSample,immune-CD4_T; MCSA,totalSample,immune-CD8_T; MCSA,totalSample,immune-NKT; MCSA,totalSample,immune-Tr1; MCSA,totalSample,immune-iTreg; MCSA,totalSample,immune-Th2; MCSA,totalSample,immune-Cytotoxic; MCSA,totalSample,immune-Effector_memory; MCSA,totalSample,immune-InfiltrationScore; ROSMAP,totalSample,immune-CD4_T; ROSMAP,totalSample,immune-Th2; ROSMAP,totalSample,immune-Th17; ROSMAP,totalSample,immune-Effector_memory; SRP223445,totalSample,immune-[illegible]
WDR45	Emory,control,male.vs.female; Homo_723,female,AD.vs.MCI; Homo_723,female,MCI.vs.control; Homo_714,female,AD.vs.control; Homo_714,female,AD.vs.MCI; Homo_714,female,MCI.vs.control; Homo_633,female,AD.vs.control; Homo_633,female,AD.vs.MCI; Homo_633,female,MCI.vs.control; ROSMAP,female,AD.vs.control; Emory,MCI,age; MCSA,MCI,age; ROSMAP,MCI,last.visit.age; Emory,totalSample,immune-CD4_T; Emory,totalSample,immune-Gamma_delta; Emory,totalSample,immune-Tr1; Emory,totalSample,immune-iTreg; Emory,totalSample,immune-Cytotoxic; Homo_723,totalSample,immune-DC; Homo_723,totalSample,immune-Cytotoxic; Homo_723,totalSample,immune-Tr1; Homo_723,totalSample,immune-Th1; Homo_723,totalSample,immune-Th2; Homo_723,totalSample,immune-iTreg; Homo_723,totalSample,immune-CD4_T; Homo_723,totalSample,immune-CD4_naive; Homo_723,totalSample,immune-Bcell; Homo_714,totalSample,immune-DC; Homo_714,totalSample,immune-Th2; Homo_714,totalSample,immune-Th1; Homo_714,totalSample,immune-Tr1; Homo_714,totalSample,immune-CD4_T; Homo_714,totalSample,immune-Cytotoxic; Homo_714,totalSample,immune-iTreg; Homo_714,totalSample,immune-CD4_naive; Homo_714,totalSample,immune-NKT; Homo_714,totalSample,immune-Bcell; Homo_633,totalSample,immune-DC; Homo_633,totalSample,immune-NKT; Homo_633,totalSample,immune-Th1; Homo_633,totalSample,immune-Th2; Homo_633,totalSample,immune-CD4_T; Homo_633,totalSample,immune-Cytotoxic; Homo_633,totalSample,immune-CD4_naive; Homo_633,totalSample,immune-Bcell; Homo_633,totalSample,immune-Tr1; Homo_633,totalSample,immune-iTreg; MCSA,totalSample,immune-DC; MCSA,totalSample,immune-Monocyte; MCSA,totalSample,immune-Macrophage; MCSA,totalSample,immune-CD8_T; MCSA,totalSample,immune-NKT; MCSA,totalSample,immune-iTreg; MCSA,totalSample,immune-Th2; MCSA,totalSample,immune-Cytotoxic; MCSA,totalSample,immune-Effector_memory; ROSMAP,totalSample,immune-Cytotoxic; ROSMAP,totalSample,immune-Effector_memory;
RNF175	Emory,control,male.vs.female; ROSMAP,female,AD.vs.control; Emory,MCI,age; ROSMAP,AD,last.visit.age; Emory,totalSample,immune-CD8_naive; Emory,totalSample,immune-InfiltrationScore; Homo_723,totalSample,immune-Bcell; Homo_633,totalSample,immune-Th17; Homo_633,totalSample,immune-Gamma_delta; MCSA,totalSample,immune-CD4_T; MCSA,totalSample,immune-CD4_naive; MCSA,totalSample,immune-Tr1; MCSA,totalSample,immune-iTreg; MCSA,totalSample,immune-Th17; MCSA,totalSample,immune-Cytotoxic; MCSA,totalSample,immune-Exhausted
COCH	Emory,control,male.vs.female; Homo_723,male,AD.vs.control; Homo_723,male,AD.vs.MCI; Homo_714,female,AD.vs.control; Homo_714,male,AD.vs.control; Homo_714,female,AD.vs.MCI; Homo_714,male,AD.vs.MCI; Homo_633,female,AD.vs.control; Homo_633,female,AD.vs.MCI; MCSA,MCI,age; ROSMAP,MCI,last.visit.age; Emory,totalSample,immune-B_cell; Emory,totalSample,immune-Macrophage; Emory,totalSample,immune-CD4_T; Emory,totalSample,immune-Tr1; Emory,totalSample,immune-iTreg; Emory,totalSample,immune-CD8_naive; Emory,totalSample,immune-InfiltrationScore; Homo_723,totalSample,immune-Th2; Homo_723,totalSample,immune-iTreg; Homo_723,totalSample,immune-Tfh; Homo_723,totalSample,immune-CD4_T; Homo_723,totalSample,immune-Tr1; Homo_723,totalSample,immune-CD4_naive; Homo_723,totalSample,immune-Macrophage; Homo_723,totalSample,immune-nTreg; Homo_723,totalSample,immune-Central_memory; Homo_714,totalSample,immune-Tfh; Homo_714,totalSample,immune-Th2; Homo_714,totalSample,immune-CD4_T; Homo_714,totalSample,immune-Tr1; Homo_714,totalSample,immune-iTreg; Homo_714,totalSample,immune-CD4_naive; Homo_714,totalSample,immune-Central_memory; Homo_714,totalSample,immune-Macrophage; Homo_714,totalSample,immune-nTreg; Homo_633,totalSample,immune-Tfh; Homo_633,totalSample,immune-CD4_T; Homo_633,totalSample,immune-CD4_naive; Homo_633,totalSample,immune-Th2; Homo_633,totalSample,immune-Central_memory; Homo_633,totalSample,immune-Tr1; Homo_633,totalSample,immune-NKT; Homo_633,totalSample,immune-InfiltrationScore; Homo_633,totalSample,immune-nTreg; Homo_633,totalSample,immune-iTreg; Homo_633,totalSample,immune-Effector_memory; MCSA,totalSample,immune-DC; MCSA,totalSample,immune-Bcell; MCSA,totalSample,immune-Macrophage; MCSA,totalSample,immune-CD4_T; MCSA,totalSample,immune-CD4_naive; MCSA,totalSample,immune-Tr1; MCSA,totalSample,immune-nTreg; MCSA,totalSample,immune-iTreg; MCSA,totalSample,immune-Tfh; MCSA,totalSample,immune-[illegible]
MXI1	Emory,control,male.vs.female; ROSMAP,female,AD.vs.control; Emory,MCI,age; Emory,totalSample,immune-Gamma_delta; Emory,totalSample,immune-CD8_naive; Emory,totalSample,immune-Cytotoxic; Homo_723,totalSample,immune-Bcell; Homo_714,totalSample,immune-Bcell; Homo_633,totalSample,immune-Bcell; Homo_633,totalSample,immune-Monocyte; MCSA,totalSample,immune-CD8_T; MCSA,totalSample,immune-CD8_naive; MCSA,totalSample,immune-Cytotoxic; MCSA,totalSample,immune-Exhausted; SRP223445,totalSample,immune-InfiltrationScore
SELENBP1	Emory,control,male.vs.female; ACOM,totalSample,age; Emory,totalSample,age; Emory,MCI,age; MCSA,MCI,age; ROSMAP,MCI,last.visit.age; Emory,totalSample,immune-Gamma_delta; Emory,totalSample,immune-Tr1; Emory,totalSample,immune-iTreg; Emory,totalSample,immune-CD8_naive; Emory,totalSample,immune-Cytotoxic; Homo_723,totalSample,immune-Bcell; Homo_714,totalSample,immune-Bcell; Homo_633,totalSample,immune-Bcell; Homo_633,totalSample,immune-DC; MCSA,totalSample,immune-DC; MCSA,totalSample,immune-Macrophage; MCSA,totalSample,immune-iTreg; MCSA,totalSample,immune-Th2; MCSA,totalSample,immune-MAIT; ROSMAP,totalSample,immune-InfiltrationScore; SRP223445,totalSample,immune-CD4_naive; ROSMAP,male-excludeAge90+,survival; ROSMAP,male-includeAge90+,survival
RNF123	Emory,control,male.vs.female; ROSMAP,female,AD.vs.control; Emory,totalSample,age; Emory,MCI,age; Emory,totalSample,immune-Gamma_delta; Emory,totalSample,immune-CD8_naive; Homo_723,totalSample,immune-Bcell; Homo_723,totalSample,immune-DC; Homo_714,totalSample,immune-DC; Homo_714,totalSample,immune-Bcell; Homo_633,totalSample,immune-DC; Homo_633,totalSample,immune-Bcell; Homo_633,totalSample,immune-Th1; MCSA,totalSample,immune-DC; MCSA,totalSample,immune-Monocyte; MCSA,totalSample,immune-Macrophage; MCSA,totalSample,immune-CD8_T; MCSA,totalSample,immune-NKT; MCSA,totalSample,immune-Th2; MCSA,totalSample,immune-Cytotoxic; MCSA,totalSample,immune-Central_memory; MCSA,totalSample,immune-Effector_memory; ROSMAP,totalSample,immune-Effector_memory; ROSMAP,male-excludeAge90+,survival
ALDH5A1	Emory,control,male.vs.female; Homo_723,male,AD.vs.control; Homo_723,male,AD.vs.MCI; Homo_723,AD,male.vs.female; Homo_723,control,male.vs.female; Homo_714,female,AD.vs.control; Homo_714,male,AD.vs.control; Homo_714,female,AD.vs.MCI; Homo_714,male,AD.vs.MCI; Homo_714,control,male.vs.female; Homo_633,control,male.vs.female; Emory,MCI,age; Emory,totalSample,immune-Neutrophil; Emory,totalSample,immune-CD8_naive; Homo_723,totalSample,immune-Th1; Homo_723,totalSample,immune-Monocyte; Homo_714,totalSample,immune-Th1; Homo_714,totalSample,immune-NKT; Homo_633,totalSample,immune-Th1; Homo_633,totalSample,immune-NKT; MCSA,totalSample,immune-DC; MCSA,totalSample,immune-Bcell; MCSA,totalSample,immune-Macrophage; MCSA,totalSample,immune-CD4_T; MCSA,totalSample,immune-CD4_naive; MCSA,totalSample,immune-Tr1; MCSA,totalSample,immune-nTreg; MCSA,totalSample,immune-iTreg; MCSA,totalSample,immune-Th17; MCSA,totalSample,immune-Tfh; MCSA,totalSample,immune-CD8_naive; MCSA,totalSample,immune-Cytotoxic; MCSA,totalSample,immune-Exhausted; MCSA,totalSample,immune-Central_memory; ROSMAP,totalSample,immune-InfiltrationScore; SRP223445,totalSample,immune-Th17; SRP223445,totalSample,immune-Exhausted; SRP223445,totalSample,immune-InfiltrationScore; ROSMAP,female-includeAge90+,survival
RUNDC3A	Emory,control,male.vs.female; Homo_723,female,AD.vs.control; Homo_723,female,AD.vs.MCI; Homo_723,male,AD.vs.MCI; Homo_723,female,MCI.vs.control; Homo_723,male,MCI.vs.control; Homo_723,AD,male.vs.female; Homo_723,control,male.vs.female; Homo_714,female,AD.vs.control; Homo_714,female,AD.vs.MCI; Homo_714,male,AD.vs.MCI; Homo_714,female,MCI.vs.control; Homo_714,male,MCI.vs.control; Homo_714,AD,male.vs.female; Homo_714,control,male.vs.female; Homo_714,MCI,male.vs.female; Homo_633,female,AD.vs.control; Homo_633,female,AD.vs.MCI; Homo_633,female,MCI.vs.control; Homo_633,male,MCI.vs.control; Homo_633,control,male.vs.female; Homo_633,MCI,male.vs.female; ROSMAP,female,AD.vs.control; ROSMAP,female,MCI.vs.control; ROSMAP,control,male.vs.female; Emory,MCI,age; MCSA,MCI,age; Emory,totalSample,immune-Monocyte; Emory,totalSample,immune-Gamma_delta; Emory,totalSample,immune-Tr1; Emory,totalSample,immune-iTreg; Emory,totalSample,immune-Cytotoxic; Homo_723,totalSample,immune-DC; Homo_723,totalSample,immune-Th2; Homo_723,totalSample,immune-Monocyte; Homo_723,totalSample,immune-Exhausted; Homo_714,totalSample,immune-Th2; Homo_714,totalSample,immune-DC; Homo_633,totalSample,immune-Th2; Homo_633,totalSample,immune-Monocyte; MCSA,totalSample,immune-DC; MCSA,totalSample,immune-Monocyte; MCSA,totalSample,immune-Macrophage; MCSA,totalSample,immune-Neutrophil; MCSA,totalSample,immune-CD8_T; MCSA,totalSample,immune-NKT; MCSA,totalSample,immune-iTreg; MCSA,totalSample,immune-Th2; MCSA,totalSample,immune-Cytotoxic; MCSA,totalSample,immune-Effector_memory
RNF10	Emory,control,male.vs.female; Homo_723,male,AD.vs.control; Homo_714,male,AD.vs.control; Emory,totalSample,age; Emory,MCI,age; Emory,totalSample,immune-Gamma_delta; Emory,totalSample,immune-Tr1; Emory,totalSample,immune-iTreg; Emory,totalSample,immune-CD8_naive; Homo_723,totalSample,immune-Bcell; Homo_714,totalSample,immune-Bcell; Homo_714,totalSample,immune-CD4_naive; Homo_633,totalSample,immune-Bcell; Homo_633,totalSample,immune-CD4_naive; MCSA,totalSample,immune-Macrophage; MCSA,totalSample,immune-MAIT; MCSA,totalSample,immune-Central_memory; SRP223445,totalSample,immune-InfiltrationScore
TSPO2	Emory,control,male.vs.female; Homo_723,female,AD.vs.control; Homo_723,male,AD.vs.control; Homo_723,female,AD.vs.MCI; Homo_723,male,AD.vs.MCI; Homo_723,female,MCI.vs.control; Homo_723,control,male.vs.female; Homo_714,female,AD.vs.control; Homo_714,male,AD.vs.control; Homo_714,female,AD.vs.MCI; Homo_714,male,AD.vs.MCI; Homo_714,female,MCI.vs.control; Homo_714,control,male.vs.female; Homo_633,control,male.vs.female; ROSMAP,MCI,male.vs.female; ROSMAP,MCI,last.visit.age; Emory,totalSample,immune-Gamma_delta; Emory,totalSample,immune-Tr1; Emory,totalSample,immune-iTreg; Emory,totalSample,immune-CD8_naive; Emory,totalSample,immune-Cytotoxic; Homo_723,totalSample,immune-DC; Homo_723,totalSample,immune-Bcell; Homo_714,totalSample,immune-Th2; MCSA,totalSample,immune-DC; MCSA,totalSample,immune-Macrophage; MCSA,totalSample,immune-Th2; ROSMAP,totalSample,immune-InfiltrationScore; SRP223445,totalSample,immune-InfiltrationScore
HBM	Emory,control,male.vs.female; Homo_723,female,AD.vs.control; Homo_723,female,AD.vs.MCI; Homo_723,male,AD.vs.MCI; Homo_723,female,MCI.vs.control; Homo_723,male,MCI.vs.control; Homo_714,female,AD.vs.control; Homo_714,male,AD.vs.control; Homo_714,female,MCI.vs.control; Homo_714,male,MCI.vs.control; Homo_714,control,male.vs.female; Homo_714,MCI,male.vs.female; Homo_633,male,MCI.vs.control; Homo_633,control,male.vs.female; ROSMAP,female,AD.vs.control; Emory,MCI,age; MCSA,MCI,age; ROSMAP,totalSample,age; ROSMAP,AD,MMSE; ROSMAP,totalSample,MMSE; Emory,totalSample,immune-Gamma_delta; Emory,totalSample,immune-Tr1; Emory,totalSample,immune-iTreg; Emory,totalSample,immune-CD8_naive; Homo_723,totalSample,immune-DC; Homo_723,totalSample,immune-Th2; Homo_723,totalSample,immune-Exhausted; Homo_723,totalSample,immune-Monocyte; Homo_714,totalSample,immune-Th2; Homo_714,totalSample,immune-DC; Homo_633,totalSample,immune-Th2; Homo_633,totalSample,immune-Monocyte; MCSA,totalSample,immune-DC; MCSA,totalSample,immune-Monocyte; MCSA,totalSample,immune-Macrophage; MCSA,totalSample,immune-CD4_T; MCSA,totalSample,immune-CD8_T; MCSA,totalSample,immune-NKT; MCSA,totalSample,immune-Tr1; MCSA,totalSample,immune-iTreg; MCSA,totalSample,immune-Th2; MCSA,totalSample,immune-Cytotoxic;
KEL	Emory,control,male.vs.female; Homo_723,female,AD.vs.control; Homo_723,male,AD.vs.control; Homo_723,female,AD.vs.MCI; Homo_723,male,AD.vs.MCI; Homo_714,female,AD.vs.control; Homo_714,male,AD.vs.control; Homo_714,female,AD.vs.MCI; Homo_714,male,AD.vs.MCI; Homo_633,female,AD.vs.control; Homo_633,female,AD.vs.MCI; Emory,MCI,age; ROSMAP,AD,sample.age; ROSMAP,MCI,sample.age; Emory,totalSample,immune-Gamma_delta; Emory,totalSample,immune-Tr1; Emory,totalSample,immune-iTreg; Emory,totalSample,immune-Th17; Homo_723,totalSample,immune-Bcell; Homo_714,totalSample,immune-Bcell; Homo_714,totalSample,immune-DC; Homo_633,totalSample,immune-DC; Homo_633,totalSample,immune-Bcell; MCSA,totalSample,immune-DC; MCSA,totalSample,immune-Macrophage; MCSA,totalSample,immune-Th2; ROSMAP,totalSample,immune-InfiltrationScore; SRP223445,totalSample,immune-InfiltrationScore
KLHDC8A	Emory,control,male.vs.female; Homo_723,female,AD.vs.control; Homo_723,male,AD.vs.control; Homo_723,female,AD.vs.MCI; Homo_723,male,AD.vs.MCI; Homo_723,female,MCI.vs.control; Homo_714,female,AD.vs.control; Homo_714,female,MCI.vs.control; Homo_714,male,MCI.vs.control; Homo_633,female,AD.vs.control; Homo_633,female,AD.vs.MCI; Homo_633,female,MCI.vs.control; Homo_633,male,MCI.vs.control; MCSA,MCI,age; Emory,totalSample,immune-Tr1; Emory,totalSample,immune-iTreg; Homo_723,totalSample,immune-DC; Homo_723,totalSample,immune-Monocyte; Homo_723,totalSample,immune-Exhausted; Homo_723,totalSample,immune-Bcell; Homo_714,totalSample,immune-Th2; Homo_714,totalSample,immune-DC; Homo_633,totalSample,immune-Th2; Homo_633,totalSample,immune-DC; Homo_633,totalSample,immune-Monocyte; MCSA,totalSample,immune-iTreg; ROSMAP,totalSample,immune-InfiltrationScore; SRP223445,totalSample,immune-Bcell
PNMA2	Emory,control,male.vs.female; Homo_723,female,AD.vs.control; Homo_723,male,AD.vs.control; Homo_723,female,AD.vs.MCI; Homo_723,male,AD.vs.MCI; Homo_723,control,male.vs.female; Homo_714,male,AD.vs.control; Homo_714,male,AD.vs.MCI; Homo_714,female,MCI.vs.control; Homo_714,control,male.vs.female; Homo_633,female,AD.vs.control; Homo_633,female,AD.vs.MCI; Homo_633,female,MCI.vs.control; Homo_633,control,male.vs.female; Emory,MCI,age; ROSMAP,AD,last.visit.age; Homo_723,totalSample,immune-Bcell; Homo_714,totalSample,immune-Bcell; Homo_633,totalSample,immune-Bcell; MCSA,totalSample,immune-Monocyte; MCSA,totalSample,immune-Central_memory
IGF2BP2	Emory,control,male.vs.female; Homo_723,control,male.vs.female; Homo_714,control,male.vs.female; Homo_633,female,AD.vs.control; Homo_633,female,AD.vs.MCI; Homo_633,female,MCI.vs.control; Homo_633,control,male.vs.female; Homo_633,MCI,male.vs.female; ROSMAP,female,AD.vs.control; ROSMAP,male,AD.vs.control; Emory,MCI,age; MCSA,totalSample,age; MCSA,control,age; Emory,totalSample,immune-Gamma_delta; Emory,totalSample,immune-CD8_naive; Homo_723,totalSample,immune-Bcell; Homo_723,totalSample,immune-Effector_memory; Homo_714,totalSample,immune-Effector_memory; Homo_714,totalSample,immune-Th2; Homo_714,totalSample,immune-iTreg; Homo_633,totalSample,immune-Effector_memory; Homo_633,totalSample,immune-Bcell; MCSA,totalSample,immune-Monocyte; MCSA,totalSample,immune-CD4_T; MCSA,totalSample,immune-CD4_naive; MCSA,totalSample,immune-Tr1; MCSA,totalSample,immune-nTreg; MCSA,totalSample,immune-Tfh; MCSA,totalSample,immune-Central_memory; MCSA,totalSample,immune-Effector_memory;
SLC5A9	Emory,control,male.vs.female; Homo_723,female,AD.vs.control; Homo_723,male,AD.vs.control; Homo_723,female,AD.vs.MCI; Homo_723,male,AD.vs.MCI; Homo_714,female,AD.vs.control; Homo_714,male,AD.vs.control; Homo_714,female,AD.vs.MCI; Homo_714,male,AD.vs.MCI; Homo_633,female,AD.vs.control; Homo_633,female,AD.vs.MCI; ROSMAP,female,AD.vs.control; ROSMAP,male,AD.vs.control; ROSMAP,male,AD.vs.MCI; Emory,totalSample,age; Emory,MCI,age; Emory,totalSample,immune-B_cell; Emory,totalSample,immune-Macrophage; Emory,totalSample,immune-Neutrophil; Emory,totalSample,immune-CD4_T; Emory,totalSample,immune-CD8_T; Emory,totalSample,immune-Gamma_delta; Emory,totalSample,immune-Th1; Emory,totalSample,immune-Tfh; Emory,totalSample,immune-CD8_naive; Emory,totalSample,immune-Exhausted; Homo_723,totalSample,immune-Neutrophil; Homo_723,totalSample,immune-Monocyte; Homo_723,totalSample,immune-Gamma_delta; Homo_714,totalSample,immune-Neutrophil; Homo_714,totalSample,immune-Monocyte; Homo_714,totalSample,immune-Th1; Homo_714,totalSample,immune-Gamma_delta; Homo_714,totalSample,immune-MAIT; Homo_633,totalSample,immune-Monocyte; Homo_633,totalSample,immune-Neutrophil; Homo_633,totalSample,immune-Exhausted; Homo_633,totalSample,immune-MAIT; Homo_633,totalSample,immune-Gamma_delta; Homo_633,totalSample,immune-Macrophage; Homo_633,totalSample,immune-CD8_T; MCSA,totalSample,immune-Macrophage; MCSA,totalSample,immune-Neutrophil; MCSA,totalSample,immune-CD4_T; MCSA,totalSample,immune-CD8_T; MCSA,totalSample,immune-CD4_naive; MCSA,totalSample,immune-Tr1; MCSA,totalSample,immune-nTreg; MCSA,totalSample,immune-Th1; MCSA,totalSample,immune-Tfh; MCSA,totalSample,immune-CD8_naive; MCSA,totalSample,immune-MAIT; MCSA,totalSample,immune-Central_memory; MCSA,totalSample,immune-Effector_memory; ROSMAP,totalSample,immune-InfiltrationScore; ROSMAP,male-includeAge90+,survival
MICALL2	Emory,control,male.vs.female; Homo_723,female,AD.vs.MCI; Homo_723,female,MCI.vs.control; Homo_714,female,AD.vs.MCI; Homo_714,female,MCI.vs.control; Homo_714,MCI,male.vs.female; Homo_633,female,MCI.vs.control; Homo_633,MCI,male.vs.female; ROSMAP,female,AD.vs.control; ROSMAP,male,AD.vs.control; ROSMAP,male,MCI.vs.control; Emory,MCI,age; MCSA,totalSample,age; MCSA,MCI,age; Emory,totalSample,immune-InfiltrationScore; Homo_723,totalSample,immune-DC; Homo_723,totalSample,immune-Cytotoxic; Homo_714,totalSample,immune-DC; Homo_633,totalSample,immune-DC; Homo_633,totalSample,immune-NKT; Homo_633,totalSample,immune-Th2; Homo_633,totalSample,immune-Th1; Homo_633,totalSample,immune-Bcell; MCSA,totalSample,immune-DC; MCSA,totalSample,immune-Monocyte; MCSA,totalSample,immune-Macrophage; MCSA,totalSample,immune-Neutrophil; MCSA,totalSample,immune-CD8_T; MCSA,totalSample,immune-NKT; MCSA,totalSample,immune-iTreg; MCSA,totalSample,immune-Th2; MCSA,totalSample,immune-Cytotoxic;

NPRL3	Emory,control,male.vs.female; Homo_723,male,AD.vs.control; Homo_714,male,AD.vs.control; ROSMAP,male,AD.vs.control; ROSMAP,female,AD.vs.MCI; Emory,MCI,age; MCSA,MCI,age; ROSMAP,MCI,last.visit.age; ROSMAP,MCI,sample.age; Emory,totalSample,immune-Gamma_delta; Emory,totalSample,immune-Tr1; Emory,totalSample,immune-iTreg; Homo_723,totalSample,immune-DC; Homo_723,totalSample,immune-Bcell; Homo_723,totalSample,immune-Cytotoxic; Homo_723,totalSample,immune-Tr1; Homo_714,totalSample,immune-DC; Homo_714,totalSample,immune-Bcell; Homo_714,totalSample,immune-Th2; Homo_714,totalSample,immune-iTreg; Homo_714,totalSample,immune-Th1; Homo_714,totalSample,immune-CD4_naive; Homo_633,totalSample,immune-DC; Homo_633,totalSample,immune-NKT; Homo_633,totalSample,immune-Bcell; Homo_633,totalSample,immune-Th1; Homo_633,totalSample,immune-Th2; Homo_633,totalSample,immune-CD4_naive; Homo_633,totalSample,immune-CD4_T; MCSA,totalSample,immune-DC; MCSA,totalSample,immune-Monocyte; MCSA,totalSample,immune-Macrophage; MCSA,totalSample,immune-Neutrophil; MCSA,totalSample,immune-CD8_T; MCSA,totalSample,immune-iTreg; MCSA,totalSample,immune-Th2; MCSA,totalSample,immune-Cytotoxic; MCSA,totalSample,immune-Effector_memory; ROSMAP,totalSample,immune-Effector_memory;
FUNDC2	Emory,control,male.vs.female; Homo_723,male,AD.vs.control; Homo_723,male,MCI.vs.control; Homo_723,control,male.vs.female; Homo_714,male,AD.vs.control; Homo_714,female,AD.vs.MCI; Homo_714,male,MCI.vs.control; Homo_714,control,male.vs.female; Homo_714,MCI,male.vs.female; Homo_633,male,MCI.vs.control; Homo_633,control,male.vs.female; Homo_633,MCI,male.vs.female; ROSMAP,female,AD.vs.control; Emory,totalSample,age; Emory,MCI,age; MCSA,totalSample,age; MCSA,control,age; Emory,totalSample,immune-Neutrophil; Emory,totalSample,immune-CD8_T; Emory,totalSample,immune-Gamma_delta; Emory,totalSample,immune-CD4_naive; Emory,totalSample,immune-Th17; Emory,totalSample,immune-Tfh; Emory,totalSample,immune-CD8_naive; Emory,totalSample,immune-Cytotoxic; Emory,totalSample,immune-Exhausted; Homo_723,totalSample,immune-Exhausted; Homo_723,totalSample,immune-Bcell; Homo_723,totalSample,immune-Th2; Homo_723,totalSample,immune-Neutrophil; Homo_714,totalSample,immune-Th2; Homo_633,totalSample,immune-Th2; MCSA,totalSample,immune-DC; MCSA,totalSample,immune-Monocyte; MCSA,totalSample,immune-CD4_T; MCSA,totalSample,immune-Gamma_delta; MCSA,totalSample,immune-CD4_naive; MCSA,totalSample,immune-nTreg; MCSA,totalSample,immune-Th1; MCSA,totalSample,immune-Th2; MCSA,totalSample,immune-Th17; MCSA,totalSample,immune-Central_memory; SRP223445,totalSample,immune-Neutrophil; SRP223445,totalSample,immune-InfiltrationScore; ROSMAP,male-excludeAge90+,survival
TMEM86B	Emory,control,male.vs.female; ACOM,AD,age; Emory,MCI,age; MCSA,totalSample,age; MCSA,MCI,age; ROSMAP,MCI,last.visit.age; Emory,totalSample,immune-Gamma_delta; Emory,totalSample,immune-Tr1; Emory,totalSample,immune-iTreg; Emory,totalSample,immune-CD8_naive; Emory,totalSample,immune-Cytotoxic; Homo_723,totalSample,immune-DC; Homo_723,totalSample,immune-Cytotoxic; Homo_723,totalSample,immune-Bcell; Homo_723,totalSample,immune-Th1; Homo_723,totalSample,immune-CD8_T; Homo_714,totalSample,immune-DC; Homo_714,totalSample,immune-Bcell; Homo_714,totalSample,immune-NKT; Homo_714,totalSample,immune-Th1; Homo_714,totalSample,immune-Cytotoxic; Homo_714,totalSample,immune-Th2; Homo_633,totalSample,immune-DC; Homo_633,totalSample,immune-NKT; Homo_633,totalSample,immune-Bcell; Homo_633,totalSample,immune-Th2; Homo_633,totalSample,immune-Th1; Homo_633,totalSample,immune-Cytotoxic; MCSA,totalSample,immune-DC; MCSA,totalSample,immune-Monocyte; MCSA,totalSample,immune-Macrophage; MCSA,totalSample,immune-CD8_T; MCSA,totalSample,immune-iTreg; MCSA,totalSample,immune-Th2; MCSA,totalSample,immune-Cytotoxic; MCSA,totalSample,immune-Effector_memory; ROSMAP,totalSample,immune-Th17; ROSMAP,totalSample,immune-InfiltrationScore
MYL4	Emory,control,male.vs.female; ROSMAP,male,AD.vs.control; Emory,totalSample,age; Emory,MCI,age; MCSA,MCI,age; ROSMAP,AD,last.visit.age; Emory,totalSample,immune-Gamma_delta; Emory,totalSample,immune-Tr1; Emory,totalSample,immune-iTreg; Emory,totalSample,immune-CD8_naive; Emory,totalSample,immune-Cytotoxic; Homo_723,totalSample,immune-Bcell; Homo_723,totalSample,immune-CD4_naive; Homo_723,totalSample,immune-Tr1; Homo_723,totalSample,immune-CD4_T; Homo_723,totalSample,immune-Cytotoxic; Homo_723,totalSample,immune-Th2; Homo_723,totalSample,immune-iTreg; Homo_714,totalSample,immune-Bcell; Homo_714,totalSample,immune-DC; Homo_714,totalSample,immune-CD4_naive; Homo_633,totalSample,immune-Bcell; Homo_633,totalSample,immune-DC; Homo_633,totalSample,immune-CD4_naive; Homo_633,totalSample,immune-Effector_memory; Homo_633,totalSample,immune-CD4_T; Homo_633,totalSample,immune-Th2; Homo_633,totalSample,immune-NKT; MCSA,totalSample,immune-DC; MCSA,totalSample,immune-Monocyte; MCSA,totalSample,immune-Macrophage; MCSA,totalSample,immune-CD4_T; MCSA,totalSample,immune-Tr1; MCSA,totalSample,immune-iTreg; MCSA,totalSample,immune-Cytotoxic
SLC4A1	Emory,control,male.vs.female; ROSMAP,female,AD.vs.control; Emory,MCI,age; MCSA,MCI,age; ROSMAP,AD,last.visit.age; Emory,totalSample,immune-Gamma_delta; Emory,totalSample,immune-CD8_naive; Homo_723,totalSample,immune-Bcell; Homo_714,totalSample,immune-Bcell; Homo_714,totalSample,immune-CD4_naive; Homo_633,totalSample,immune-Bcell; Homo_633,totalSample,immune-CD4_naive; Homo_633,totalSample,immune-Effector_memory; MCSA,totalSample,immune-iTreg; MCSA,totalSample,immune-MAIT; MCSA,totalSample,immune-Central_memory; SRP223445,totalSample,immune-CD8_T; SRP223445,totalSample,immune-MAIT
STRADB	Emory,control,male.vs.female; ROSMAP,female,AD.vs.control; Emory,MCI,age; ROSMAP,AD,sample.age; Emory,totalSample,immune-Gamma_delta; Emory,totalSample,immune-Th17; Emory,totalSample,immune-CD8_naive; Homo_723,totalSample,immune-Bcell; Homo_714,totalSample,immune-Bcell; Homo_633,totalSample,immune-Bcell; Homo_633,totalSample,immune-Monocyte; MCSA,totalSample,immune-NK; ROSMAP,totalSample,immune-iTreg; ROSMAP,totalSample,immune-CD8_naive; ROSMAP,totalSample,immune-Effector_memory; SRP223445,totalSample,immune-InfiltrationScore
PSMF1	Emory,control,male.vs.female; ROSMAP,female,AD.vs.control; ACOM,totalSample,age; Emory,totalSample,age; Emory,MCI,age; MCSA,MCI,age; Emory,totalSample,immune-Gamma_delta; Emory,totalSample,immune-Tr1; Emory,totalSample,immune-CD8_naive; Emory,totalSample,immune-Cytotoxic; Homo_723,totalSample,immune-Bcell; Homo_714,totalSample,immune-Bcell; Homo_633,totalSample,immune-Bcell; Homo_633,totalSample,immune-Effector_memory; Homo_633,totalSample,immune-CD4_naive; MCSA,totalSample,immune-NKT; MCSA,totalSample,immune-Exhausted; MCSA,totalSample,immune-MAIT; MCSA,totalSample,immune-Central_memory; ROSMAP,totalSample,immune-Central_memory; ROSMAP,totalSample,immune-Effector_memory; SRP223445,totalSample,immune-NK; SRP223445,totalSample,immune-Neutrophil; SRP223445,totalSample,immune-CD4_naive; SRP223445,totalSample,immune-InfiltrationScore
AHSP	Emory,control,male.vs.female; Homo_723,male,AD.vs.control; Homo_723,female,AD.vs.MCI; Homo_714,female,AD.vs.MCI; Emory,totalSample,age; Emory,MCI,age; MCSA,MCI,age; ROSMAP,AD,last.visit.age; ROSMAP,AD,sample.age; ROSMAP,MCI,sample.age; Emory,totalSample,immune-Gamma_delta; Emory,totalSample,immune-iTreg; Emory,totalSample,immune-CD8_naive; Emory,totalSample,immune-Cytotoxic; Homo_723,totalSample,immune-Bcell; Homo_723,totalSample,immune-Th2; Homo_723,totalSample,immune-Monocyte; Homo_714,totalSample,immune-Bcell; Homo_714,totalSample,immune-CD4_naive; Homo_714,totalSample,immune-iTreg; Homo_633,totalSample,immune-Bcell; Homo_633,totalSample,immune-CD4_naive; Homo_633,totalSample,immune-iTreg; Homo_633,totalSample,immune-CD4_T; Homo_633,totalSample,immune-Monocyte; Homo_633,totalSample,immune-Effector_memory; Homo_633,totalSample,immune-NKT; Homo_633,totalSample,immune-Th2; MCSA,totalSample,immune-NK; MCSA,totalSample,immune-Central_memory;
SERPINE2	Emory,control,male.vs.female; Homo_723,female,AD.vs.control; Homo_723,male,AD.vs.control; Homo_723,female,AD.vs.MCI; Homo_723,male,AD.vs.MCI; Homo_723,female,MCI.vs.control; Homo_723,male,MCI.vs.control; Homo_714,female,AD.vs.control; Homo_714,male,AD.vs.control; Homo_714,female,AD.vs.MCI; Homo_714,male,AD.vs.MCI; Homo_714,female,MCI.vs.control; Homo_633,female,AD.vs.control; Homo_633,female,AD.vs.MCI; ACOM,totalSample,age; MCSA,totalSample,age; MCSA,control,age; Emory,totalSample,immune-Macrophage; Emory,totalSample,immune-Neutrophil; Emory,totalSample,immune-CD4_T; Emory,totalSample,immune-CD8_T; Emory,totalSample,immune-Tr1; Emory,totalSample,immune-iTreg; Emory,totalSample,immune-Th17; Emory,totalSample,immune-Tfh; Emory,totalSample,immune-CD8_naive; Emory,totalSample,immune-Central_memory; Emory,totalSample,immune-InfiltrationScore; Homo_723,totalSample,immune-Central_memory; Homo_723,totalSample,immune-Th17; Homo_723,totalSample,immune-CD4_naive; Homo_723,totalSample,immune-CD4_T; Homo_723,totalSample,immune-nTreg; Homo_723,totalSample,immune-Cytotoxic; Homo_723,totalSample,immune-Th1; Homo_723,totalSample,immune-Tr1; Homo_723,totalSample,immune-Tfh; Homo_723,totalSample,immune-DC; Homo_723,totalSample,immune-Neutrophil; Homo_714,totalSample,immune-CD4_naive; Homo_714,totalSample,immune-Th17; Homo_714,totalSample,immune-Th1; Homo_714,totalSample,immune-CD4_T; Homo_714,totalSample,immune-Central_memory; Homo_714,totalSample,immune-iTreg; Homo_714,totalSample,immune-nTreg; Homo_714,totalSample,immune-Exhausted; Homo_714,totalSample,immune-Cytotoxic; Homo_714,totalSample,immune-Tfh; Homo_714,totalSample,immune-NKT; Homo_714,totalSample,immune-NK; Homo_633,totalSample,immune-Tfh; Homo_633,totalSample,immune-CD4_T; Homo_633,totalSample,immune-iTreg; Homo_633,totalSample,immune-CD4_naive; Homo_633,totalSample,immune-Tr1; Homo_633,totalSample,immune-Central_memory; Homo_633,totalSample,immune-nTreg; Homo_633,totalSample,immune-Th17; Homo_633,totalSample,immune-NKT; Homo_633,totalSample,immune-Th2; Homo_633,totalSample,immune-Gamma_delta; Homo_633,totalSample,immune-Effector_memory; MCSA,totalSample,immune-CD4_T; MCSA,totalSample,immune-Gamma_delta; MCSA,totalSample,immune-CD4_naive; MCSA,totalSample,immune-Tr1; MCSA,totalSample,immune-nTreg; MCSA,totalSample,immune-iTreg; MCSA,totalSample,immune-Th1; MCSA,totalSample,immune-Th17; MCSA,totalSample,immune-Tfh; MCSA,totalSample,immune-CD8_naive; MCSA,totalSample,immune-Cytotoxic; MCSA,totalSample,immune-Exhausted; MCSA,totalSample,immune-Central_memory; ROSMAP,totalSample,immune-[illegible]
CARM1	Emory,control,male.vs.female; ROSMAP,female,AD.vs.control; ROSMAP,male,AD.vs.control; ROSMAP,female,AD.vs.MCI; ROSMAP,male,AD.vs.MCI; Emory,MCI,age; Emory,totalSample,immune-Gamma_delta; Homo_723,totalSample,immune-DC; Homo_723,totalSample,immune-InfiltrationScore; Homo_723,totalSample,immune-Bcell; Homo_714,totalSample,immune-DC; Homo_714,totalSample,immune-Bcell; Homo_714,totalSample,immune-InfiltrationScore; Homo_633,totalSample,immune-DC; Homo_633,totalSample,immune-Bcell; MCSA,totalSample,immune-DC; MCSA,totalSample,immune-Monocyte; MCSA,totalSample,immune-Macrophage; MCSA,totalSample,immune-CD8_T; MCSA,totalSample,immune-NKT; MCSA,totalSample,immune-Th2; MCSA,totalSample,immune-Cytotoxic; MCSA,totalSample,immune-Effector_memory; SRP223445,totalSample,immune-Neutrophil; SRP223445,totalSample,immune-CD4_naive; SRP223445,totalSample,immune-InfiltrationScore
PIP5KL1	Emory,control,male.vs.female; Homo_723,male,AD.vs.control; Homo_723,female,AD.vs.MCI; Homo_723,male,AD.vs.MCI; Homo_723,female,MCI.vs.control; Homo_714,female,AD.vs.control; Homo_714,male,AD.vs.control; Homo_714,female,AD.vs.MCI; Homo_714,male,AD.vs.MCI; Homo_714,female,MCI.vs.control; Homo_633,female,AD.vs.control; Homo_633,female,AD.vs.MCI; Homo_633,female,MCI.vs.control; Emory,MCI,age; MCSA,MCI,age; Emory,totalSample,immune-Gamma_delta; Emory,totalSample,immune-CD8_naive; Homo_723,totalSample,immune-Exhausted; Homo_723,totalSample,immune-CD8_T; Homo_723,totalSample,immune-Cytotoxic; Homo_723,totalSample,immune-CD8_naive; Homo_723,totalSample,immune-DC; Homo_723,totalSample,immune-Monocyte; Homo_714,totalSample,immune-DC; Homo_714,totalSample,immune-Monocyte; Homo_633,totalSample,immune-DC; Homo_633,totalSample,immune-Th2; MCSA,totalSample,immune-DC; MCSA,totalSample,immune-Monocyte; MCSA,totalSample,immune-Neutrophil; MCSA,totalSample,immune-CD8_T; MCSA,totalSample,immune-iTreg; MCSA,totalSample,immune-Th2; MCSA,totalSample,immune-Cytotoxic; MCSA,totalSample,immune-Effector_memory; ROSMAP,female-excludeAge90+,survival; ROSMAP,female-includeAge90+,survival; ROSMAP,male and female-excludeAge90+,survival; ROSMAP,male and female-
ADIPOR1	Emory,control,male.vs.female; Emory,MCI,age; Emory,totalSample,immune-Gamma_delta; Emory,totalSample,immune-Tr1; Emory,totalSample,immune-iTreg; Homo_723,totalSample,immune-Bcell; Homo_723,totalSample,immune-Gamma_delta; Homo_714,totalSample,immune-Bcell; Homo_633,totalSample,immune-Bcell; SRP223445,totalSample,immune-CD4_naive; SRP223445,totalSample,immune-Exhausted
PLPP7	Emory,control,male.vs.female; Homo_633,female,AD.vs.control; Homo_633,female,AD.vs.MCI; ROSMAP,MCI,last.visit.age; Emory,totalSample,immune-DC; Homo_723,totalSample,immune-DC; Homo_723,totalSample,immune-Monocyte; Homo_714,totalSample,immune-DC; Homo_714,totalSample,immune-Th2; Homo_633,totalSample,immune-DC; Homo_633,totalSample,immune-Th2; MCSA,totalSample,immune-DC; MCSA,totalSample,immune-Monocyte; MCSA,totalSample,immune-Neutrophil; MCSA,totalSample,immune-CD8_T; MCSA,totalSample,immune-iTreg; MCSA,totalSample,immune-Th2; MCSA,totalSample,immune-Cytotoxic; MCSA,totalSample,immune-Effector_memory; MCSA,totalSample,immune-
FAM161A	Emory,control,male.vs.female; Homo_723,female,AD.vs.control; Homo_723,male,AD.vs.control; Homo_723,female,AD.vs.MCI; Homo_723,male,AD.vs.MCI; Homo_714,male,AD.vs.control; Homo_633,female,AD.vs.control; Homo_633,female,AD.vs.MCI; Homo_633,male,MCI.vs.control; ACOM,AD,age; MCSA,MCI,age; Emory,totalSample,immune-Macrophage; Emory,totalSample,immune-Neutrophil; Emory,totalSample,immune-CD4_T; Emory,totalSample,immune-CD8_T; Emory,totalSample,immune-Tr1; Emory,totalSample,immune-iTreg; Emory,totalSample,immune-Tfh; Emory,totalSample,immune-InfiltrationScore; Homo_723,totalSample,immune-iTreg; Homo_723,totalSample,immune-Central_memory; Homo_723,totalSample,immune-CD4_T; Homo_723,totalSample,immune-Tr1; Homo_723,totalSample,immune-CD4_naive; Homo_723,totalSample,immune-Th1; Homo_723,totalSample,immune-Tfh; Homo_723,totalSample,immune-nTreg; Homo_723,totalSample,immune-Th2; Homo_723,totalSample,immune-Monocyte; Homo_723,totalSample,immune-CD8_naive; Homo_723,totalSample,immune-NKT; Homo_723,totalSample,immune-Macrophage; Homo_714,totalSample,immune-iTreg; Homo_714,totalSample,immune-Tfh; Homo_714,totalSample,immune-Tr1; Homo_714,totalSample,immune-CD4_naive; Homo_714,totalSample,immune-CD4_T; Homo_714,totalSample,immune-Th1; Homo_714,totalSample,immune-NKT; Homo_714,totalSample,immune-Central_memory; Homo_714,totalSample,immune-nTreg; Homo_714,totalSample,immune-InfiltrationScore; Homo_714,totalSample,immune-Macrophage; Homo_633,totalSample,immune-Tfh; Homo_633,totalSample,immune-CD4_naive; Homo_633,totalSample,immune-NKT; Homo_633,totalSample,immune-Tr1; Homo_633,totalSample,immune-CD4_T; Homo_633,totalSample,immune-iTreg; Homo_633,totalSample,immune-Th1; Homo_633,totalSample,immune-Central_memory; Homo_633,totalSample,immune-nTreg; Homo_633,totalSample,immune-InfiltrationScore; Homo_633,totalSample,immune-MAIT; MCSA,totalSample,immune-DC; MCSA,totalSample,immune-Bcell; MCSA,totalSample,immune-Monocyte; MCSA,totalSample,immune-Macrophage; MCSA,totalSample,immune-CD4_T; MCSA,totalSample,immune-CD4_naive; MCSA,totalSample,immune-Tr1; MCSA,totalSample,immune-nTreg; MCSA,totalSample,immune-iTreg; MCSA,totalSample,immune-Tfh; MCSA,totalSample,immune-CD8_naive; MCSA,totalSample,immune-[illegible]
SLC6A9	Emory,control,male.vs.female; Homo_723,female,AD.vs.control; Homo_723,female,MCI.vs.control; Homo_714,female,MCI.vs.control; Homo_633,female,AD.vs.control; Homo_633,female,AD.vs.MCI; Homo_633,female,MCI.vs.control; Homo_633,male,MCI.vs.control; ROSMAP,female,AD.vs.control; ROSMAP,female,MCI.vs.control; Emory,totalSample,age; Emory,MCI,age; MCSA,MCI,age; Emory,totalSample,immune-Gamma_delta; Emory,totalSample,immune-Th17; Emory,totalSample,immune-CD8_naive; Emory,totalSample,immune-Cytotoxic; Homo_723,totalSample,immune-DC; Homo_723,totalSample,immune-Monocyte; Homo_723,totalSample,immune-Th2; Homo_723,totalSample,immune-Bcell; Homo_723,totalSample,immune-Neutrophil; Homo_723,totalSample,immune-Exhausted; Homo_714,totalSample,immune-Th2; Homo_714,totalSample,immune-DC; Homo_633,totalSample,immune-Th2; Homo_633,totalSample,immune-DC; Homo_633,totalSample,immune-Monocyte; Homo_633,totalSample,immune-Bcell; MCSA,totalSample,immune-DC; MCSA,totalSample,immune-Monocyte; MCSA,totalSample,immune-Macrophage; MCSA,totalSample,immune-NKT; MCSA,totalSample,immune-Th2; ROSMAP,totalSample,immune-InfiltrationScore; SRP223445,totalSample,immune-CD8_T; ROSMAP,female-excludeAge90+,survival
CAMP	Emory,control,male.vs.female; Homo_723,female,AD.vs.control; Homo_723,male,AD.vs.control; Homo_723,female,AD.vs.MCI; Homo_723,male,AD.vs.MCI; Homo_714,female,AD.vs.MCI; Homo_714,control,male.vs.female; MCSA,MCI,age; Emory,totalSample,immune-DC; Emory,totalSample,immune-Macrophage; Emory,totalSample,immune-NK; Emory,totalSample,immune-Neutrophil; Emory,totalSample,immune-CD4_T; Emory,totalSample,immune-NKT; Emory,totalSample,immune-Tr1; Emory,totalSample,immune-nTreg; Emory,totalSample,immune-iTreg; Emory,totalSample,immune-Tfh; Emory,totalSample,immune-Central_memory; Emory,totalSample,immune-InfiltrationScore; Homo_723,totalSample,immune-Monocyte; Homo_723,totalSample,immune-Th1; Homo_723,totalSample,immune-Cytotoxic; Homo_723,totalSample,immune-Macrophage; Homo_723,totalSample,immune-CD4_naive; Homo_714,totalSample,immune-Macrophage; Homo_714,totalSample,immune-Th2; Homo_714,totalSample,immune-CD4_T; Homo_714,totalSample,immune-Tr1; Homo_714,totalSample,immune-Tfh; Homo_714,totalSample,immune-iTreg; Homo_714,totalSample,immune-CD4_naive; Homo_714,totalSample,immune-Central_memory; Homo_714,totalSample,immune-nTreg; Homo_633,totalSample,immune-Macrophage; Homo_633,totalSample,immune-Th2; Homo_633,totalSample,immune-CD4_T; MCSA,totalSample,immune-Macrophage; MCSA,totalSample,immune-CD4_T; MCSA,totalSample,immune-NKT; MCSA,totalSample,immune-CD4_naive; MCSA,totalSample,immune-Tr1; MCSA,totalSample,immune-nTreg; MCSA,totalSample,immune-iTreg; MCSA,totalSample,immune-Th2; MCSA,totalSample,immune-Th17; MCSA,totalSample,immune-Tfh; MCSA,totalSample,immune-MAIT; MCSA,totalSample,immune-Central_memory; MCSA,totalSample,immune-InfiltrationScore; ROSMAP,totalSample,immune-InfiltrationScore; SRP223445,totalSample,immune-Macrophage; SRP223445,totalSample,immune-CD4_T; SRP223445,totalSample,immune-nTreg; SRP223445,totalSample,immune-Th17; SRP223445,totalSample,immune-Exhausted
MKRN1	Emory,control,male.vs.female; ROSMAP,female,AD.vs.control; Emory,MCI,age; Emory,totalSample,immune-Gamma_delta; Emory,totalSample,immune-CD8_naive; Homo_723,totalSample,immune-Bcell; Homo_723,totalSample,immune-Gamma_delta; Homo_714,totalSample,immune-Bcell; Homo_633,totalSample,immune-Bcell; Homo_633,totalSample,immune-Monocyte; Homo_633,totalSample,immune-Gamma_delta; MCSA,totalSample,immune-Monocyte; MCSA,totalSample,immune-NK; MCSA,totalSample,immune-Neutrophil; MCSA,totalSample,immune-CD8_T; MCSA,totalSample,immune-Cytotoxic; MCSA,totalSample,immune-Exhausted; MCSA,totalSample,immune-Effector_memory; SRP223445,totalSample,immune-Neutrophil;
TFR2	Emory,control,male.vs.female; Homo_723,female,AD.vs.control; Homo_723,male,AD.vs.control; Homo_723,female,AD.vs.MCI; Homo_723,male,AD.vs.MCI; Homo_714,female,AD.vs.control; Homo_714,male,AD.vs.control; Homo_714,female,AD.vs.MCI; Homo_714,male,AD.vs.MCI; Homo_633,female,AD.vs.control; Homo_633,female,AD.vs.MCI; Homo_633,control,male.vs.female; ROSMAP,female,MCI.vs.control; ROSMAP,MCI,male.vs.female; Emory,MCI,age; MCSA,MCI,age; Emory,totalSample,immune-Gamma_delta; Emory,totalSample,immune-iTreg; Emory,totalSample,immune-Th17; Emory,totalSample,immune-CD8_naive; Emory,totalSample,immune-Cytotoxic; Homo_723,totalSample,immune-DC; Homo_723,totalSample,immune-Cytotoxic; Homo_723,totalSample,immune-Neutrophil; Homo_723,totalSample,immune-Bcell; Homo_723,totalSample,immune-Exhausted; Homo_723,totalSample,immune-CD8_T; Homo_723,totalSample,immune-Th2; Homo_714,totalSample,immune-DC; Homo_714,totalSample,immune-Th2; Homo_714,totalSample,immune-Cytotoxic; Homo_714,totalSample,immune-Neutrophil; Homo_633,totalSample,immune-DC; Homo_633,totalSample,immune-Th2; Homo_633,totalSample,immune-Cytotoxic; Homo_633,totalSample,immune-Bcell; MCSA,totalSample,immune-DC; MCSA,totalSample,immune-Monocyte; MCSA,totalSample,immune-Macrophage; MCSA,totalSample,immune-Neutrophil; MCSA,totalSample,immune-CD8_T; MCSA,totalSample,immune-iTreg; MCSA,totalSample,immune-Th2; MCSA,totalSample,immune-Cytotoxic; MCSA,totalSample,immune-Effector_memory; ROSMAP,totalSample,immune-InfiltrationScore; SRP223445,totalSample,immune-InfiltrationScore
IFITM10	Emory,control,male.vs.female; Homo_633,control,male.vs.female; ROSMAP,AD,male.vs.female; Emory,totalSample,immune-B_cell; Emory,totalSample,immune-Macrophage; Emory,totalSample,immune-Neutrophil; Emory,totalSample,immune-CD8_T; Emory,totalSample,immune-Tfh; Emory,totalSample,immune-CD8_naive; Emory,totalSample,immune-Cytotoxic; Emory,totalSample,immune-Exhausted; Homo_723,totalSample,immune-DC; Homo_723,totalSample,immune-iTreg; Homo_714,totalSample,immune-DC; Homo_714,totalSample,immune-CD4_T; Homo_714,totalSample,immune-Th1; Homo_714,totalSample,immune-Th2; Homo_633,totalSample,immune-DC; Homo_633,totalSample,immune-Th1; MCSA,totalSample,immune-DC; MCSA,totalSample,immune-Monocyte; MCSA,totalSample,immune-Macrophage; MCSA,totalSample,immune-iTreg; MCSA,totalSample,immune-Th2; ROSMAP,totalSample,immune-MAIT; ROSMAP,totalSample,immune-Effector_memory; SRP223445,totalSample,immune-CD4_naive; ROSMAP,female-excludeAge90+,survival
KCNH2	Emory,control,male.vs.female; Homo_723,female,AD.vs.control; Homo_723,male,AD.vs.control; Homo_723,female,AD.vs.MCI; Homo_723,male,AD.vs.MCI; Homo_723,female,MCI.vs.control; Homo_714,female,AD.vs.control; Homo_714,male,AD.vs.control; Homo_714,female,AD.vs.MCI; Homo_714,female,MCI.vs.control; Homo_633,female,AD.vs.control; Homo_633,female,AD.vs.MCI; Homo_633,female,MCI.vs.control; Homo_633,male,MCI.vs.control; ROSMAP,MCI,last.visit.age; Homo_723,totalSample,immune-DC; Homo_723,totalSample,immune-Neutrophil; Homo_723,totalSample,immune-Monocyte; Homo_723,totalSample,immune-Bcell; Homo_723,totalSample,immune-Th2; Homo_714,totalSample,immune-Th2; Homo_714,totalSample,immune-DC; Homo_633,totalSample,immune-Th2; Homo_633,totalSample,immune-DC; Homo_633,totalSample,immune-Monocyte; MCSA,totalSample,immune-DC; MCSA,totalSample,immune-Monocyte; MCSA,totalSample,immune-NKT; MCSA,totalSample,immune-Th2; MCSA,totalSample,immune-Effector_memory; ROSMAP,male and female-
NBEA	Emory,control,male.vs.female; Homo_723,female,AD.vs.MCI; Homo_723,female,MCI.vs.control; Homo_723,AD,male.vs.female; Homo_723,control,male.vs.female; Homo_723,MCI,male.vs.female; Homo_714,female,MCI.vs.control; Homo_714,male,MCI.vs.control; Homo_714,AD,male.vs.female; Homo_714,control,male.vs.female; Homo_714,MCI,male.vs.female; Homo_633,female,MCI.vs.control; Homo_633,control,male.vs.female; MCSA,totalSample,age; MCSA,control,age; Emory,totalSample,immune-CD4_T; Emory,totalSample,immune-Gamma_delta; Emory,totalSample,immune-CD4_naive; Emory,totalSample,immune-Tr1; Emory,totalSample,immune-iTreg; Emory,totalSample,immune-Th17; Emory,totalSample,immune-Tfh; Emory,totalSample,immune-Central_memory; Emory,totalSample,immune-InfiltrationScore; Homo_723,totalSample,immune-Central_memory; Homo_723,totalSample,immune-CD4_naive; Homo_723,totalSample,immune-Tr1; Homo_723,totalSample,immune-CD4_T; Homo_723,totalSample,immune-Th1; Homo_723,totalSample,immune-iTreg; Homo_723,totalSample,immune-Tfh; Homo_723,totalSample,immune-nTreg; Homo_723,totalSample,immune-Cytotoxic; Homo_723,totalSample,immune-Gamma_delta; Homo_714,totalSample,immune-CD4_naive; Homo_714,totalSample,immune-Th1; Homo_714,totalSample,immune-iTreg; Homo_714,totalSample,immune-CD4_T; Homo_714,totalSample,immune-Tr1; Homo_714,totalSample,immune-NKT; Homo_714,totalSample,immune-Central_memory; Homo_714,totalSample,immune-Tfh; Homo_714,totalSample,immune-nTreg; Homo_714,totalSample,immune-Cytotoxic; Homo_633,totalSample,immune-Tfh; Homo_633,totalSample,immune-iTreg; Homo_633,totalSample,immune-CD4_naive; Homo_633,totalSample,immune-Tr1; Homo_633,totalSample,immune-CD4_T; Homo_633,totalSample,immune-Th1; Homo_633,totalSample,immune-NKT; Homo_633,totalSample,immune-Central_memory; Homo_633,totalSample,immune-nTreg; MCSA,totalSample,immune-DC; MCSA,totalSample,immune-Bcell; MCSA,totalSample,immune-Macrophage; MCSA,totalSample,immune-CD4_T; MCSA,totalSample,immune-Gamma_delta; MCSA,totalSample,immune-CD4_naive; MCSA,totalSample,immune-Tr1; MCSA,totalSample,immune-nTreg; MCSA,totalSample,immune-iTreg; MCSA,totalSample,immune-Th17; MCSA,totalSample,immune-Tfh; MCSA,totalSample,immune-CD8_naive; MCSA,totalSample,immune-Cytotoxic; MCSA,totalSample,immune-[illegible]
FECH	Emory,control,male.vs.female; Homo_714,female,AD.vs.MCI; Emory,totalSample,age; Emory,MCI,age; Emory,totalSample,immune-Gamma_delta; Emory,totalSample,immune-CD8_naive; Homo_723,totalSample,immune-Bcell; Homo_714,totalSample,immune-Bcell; Homo_633,totalSample,immune-Bcell; Homo_633,totalSample,immune-Monocyte; ROSMAP,totalSample,immune-Effector_memory; SRP223445,totalSample,immune-Neutrophil; SRP223445,totalSample,immune-InfiltrationScore; ROSMAP,male-excludeAge90+,survival
OR2T33	Emory,control,male.vs.female; MCSA,MCI,age; Emory,totalSample,immune-Tr1; Emory,totalSample,immune-Central_memory; Homo_723,totalSample,immune-Bcell; Homo_714,totalSample,immune-Bcell; Homo_633,totalSample,immune-Bcell

MRC2	Emory,control,male.vs.female; Homo_723,female,AD.vs.control; Homo_714,female,AD.vs.control; Homo_714,male,AD.vs.control; Homo_714,female,AD.vs.MCI; Homo_714,male,AD.vs.MCI; Homo_714,female,MCI.vs.control; Homo_633,female,AD.vs.control; Homo_633,female,AD.vs.MCI; Homo_633,female,MCI.vs.control; Emory,MCI,age; MCSA,MCI,age; Emory,totalSample,immune-Gamma_delta; Emory,totalSample,immune-CD8_naive; Emory,totalSample,immune-Cytotoxic; Homo_723,totalSample,immune-DC; Homo_723,totalSample,immune-Bcell; Homo_723,totalSample,immune-Neutrophil; Homo_723,totalSample,immune-Cytotoxic; Homo_723,totalSample,immune-CD8_T; Homo_714,totalSample,immune-DC; Homo_714,totalSample,immune-Bcell; Homo_633,totalSample,immune-DC; Homo_633,totalSample,immune-Bcell; Homo_633,totalSample,immune-Th2; MCSA,totalSample,immune-DC; MCSA,totalSample,immune-Monocyte; MCSA,totalSample,immune-Macrophage; MCSA,totalSample,immune-Neutrophil; MCSA,totalSample,immune-CD8_T; MCSA,totalSample,immune-NKT; MCSA,totalSample,immune-iTreg; MCSA,totalSample,immune-Th2; MCSA,totalSample,immune-Cytotoxic; MCSA,totalSample,immune-Effector_memory; SRP223445,totalSample,immune-InfiltrationScore
SDK2	Emory,control,male.vs.female; Homo_723,female,MCI.vs.control; Homo_723,male,MCI.vs.control; Homo_723,control,male.vs.female; Homo_714,female,MCI.vs.control; Homo_714,male,MCI.vs.control; Homo_714,control,male.vs.female; Homo_633,female,MCI.vs.control; Homo_633,male,MCI.vs.control; Homo_633,control,male.vs.female; ROSMAP,female,AD.vs.control; ROSMAP,female,AD.vs.MCI; ROSMAP,control,male.vs.female; MCSA,MCI,age; Emory,totalSample,immune-B_cell; Emory,totalSample,immune-Monocyte; Emory,totalSample,immune-Macrophage; Emory,totalSample,immune-Neutrophil; Emory,totalSample,immune-CD4_T; Emory,totalSample,immune-Gamma_delta; Emory,totalSample,immune-CD4_naive; Emory,totalSample,immune-Tr1; Emory,totalSample,immune-nTreg; Emory,totalSample,immune-iTreg; Emory,totalSample,immune-Th17; Emory,totalSample,immune-Tfh; Emory,totalSample,immune-CD8_naive; Emory,totalSample,immune-Cytotoxic; Emory,totalSample,immune-Central_memory; Emory,totalSample,immune-InfiltrationScore; Homo_723,totalSample,immune-Monocyte; Homo_723,totalSample,immune-DC; Homo_723,totalSample,immune-Central_memory; Homo_714,totalSample,immune-Monocyte; Homo_633,totalSample,immune-Monocyte; MCSA,totalSample,immune-Monocyte; MCSA,totalSample,immune-Neutrophil; MCSA,totalSample,immune-CD8_T; MCSA,totalSample,immune-CD4_naive; MCSA,totalSample,immune-nTreg; MCSA,totalSample,immune-Tfh; MCSA,totalSample,immune-Central_memory; MCSA,totalSample,immune-Effector_memory; MCSA,totalSample,immune-InfiltrationScore
UBE2O	Emory,control,male.vs.female; Homo_723,male,AD.vs.control; Homo_723,male,AD.vs.MCI; Homo_723,control,male.vs.female; Homo_714,male,AD.vs.control; Homo_714,male,AD.vs.MCI; Homo_714,female,MCI.vs.control; Homo_714,control,male.vs.female; Homo_633,female,MCI.vs.control; Homo_633,control,male.vs.female; ROSMAP,female,AD.vs.control; Emory,totalSample,age; Emory,MCI,age; MCSA,MCI,age; Emory,totalSample,immune-Gamma_delta; Emory,totalSample,immune-CD8_naive; Homo_723,totalSample,immune-Bcell; Homo_723,totalSample,immune-DC; Homo_723,totalSample,immune-Cytotoxic; Homo_723,totalSample,immune-Neutrophil; Homo_723,totalSample,immune-Exhausted; Homo_714,totalSample,immune-DC; Homo_714,totalSample,immune-Bcell; Homo_633,totalSample,immune-DC; Homo_633,totalSample,immune-Bcell; Homo_633,totalSample,immune-NKT; MCSA,totalSample,immune-DC; MCSA,totalSample,immune-Monocyte; MCSA,totalSample,immune-Neutrophil; MCSA,totalSample,immune-CD8_T; MCSA,totalSample,immune-iTreg; MCSA,totalSample,immune-Th2; MCSA,totalSample,immune-Cytotoxic; MCSA,totalSample,immune-Effector_memory; MCSA,totalSample,immune-InfiltrationScore; SRP223445,totalSample,immune-Neutrophil; SRP223445,totalSample,immune-InfiltrationScore; ROSMAP,male and female-excludeAge90+,survival
C17orf99	Emory,control,male.vs.female; Homo_723,female,AD.vs.MCI; Homo_723,female,MCI.vs.control; Homo_714,female,AD.vs.control; Homo_714,male,AD.vs.control; Homo_714,female,AD.vs.MCI; Homo_714,male,MCI.vs.control; ROSMAP,female,AD.vs.control; MCSA,MCI,age; Emory,totalSample,immune-Gamma_delta; Emory,totalSample,immune-Tr1; Emory,totalSample,immune-iTreg; Emory,totalSample,immune-Cytotoxic; Homo_723,totalSample,immune-Bcell; Homo_723,totalSample,immune-Cytotoxic; Homo_723,totalSample,immune-Th2; Homo_723,totalSample,immune-DC; Homo_633,totalSample,immune-Th2; MCSA,totalSample,immune-DC; MCSA,totalSample,immune-Monocyte; MCSA,totalSample,immune-Macrophage; MCSA,totalSample,immune-CD8_T; MCSA,totalSample,immune-NKT; MCSA,totalSample,immune-iTreg; MCSA,totalSample,immune-Th2; MCSA,totalSample,immune-Cytotoxic; SRP223445,totalSample,immune-InfiltrationScore
CACNB4	Emory,control,male.vs.female; Homo_723,control,male.vs.female; Homo_714,control,male.vs.female; Homo_633,control,male.vs.female; Emory,MCI,age; MCSA,MCI,age; Emory,totalSample,immune-Neutrophil; Emory,totalSample,immune-CD8_T; Emory,totalSample,immune-Gamma_delta; Emory,totalSample,immune-Th17; Emory,totalSample,immune-Cytotoxic; Emory,totalSample,immune-Exhausted; Homo_723,totalSample,immune-Cytotoxic; Homo_723,totalSample,immune-CD8_T; Homo_723,totalSample,immune-Gamma_delta; Homo_723,totalSample,immune-NKT; Homo_723,totalSample,immune-Exhausted; Homo_723,totalSample,immune-Neutrophil; Homo_714,totalSample,immune-CD8_T; Homo_714,totalSample,immune-Cytotoxic; Homo_714,totalSample,immune-Exhausted; Homo_714,totalSample,immune-NKT; Homo_714,totalSample,immune-Gamma_delta; Homo_714,totalSample,immune-Neutrophil; Homo_714,totalSample,immune-Th17; Homo_633,totalSample,immune-NKT; Homo_633,totalSample,immune-DC; Homo_633,totalSample,immune-CD8_T; Homo_633,totalSample,immune-Cytotoxic; Homo_633,totalSample,immune-Exhausted; Homo_633,totalSample,immune-Gamma_delta; Homo_633,totalSample,immune-Neutrophil; Homo_633,totalSample,immune-Th17; MCSA,totalSample,immune-DC; MCSA,totalSample,immune-Monocyte; MCSA,totalSample,immune-CD4_T; MCSA,totalSample,immune-CD8_T; MCSA,totalSample,immune-Tr1; MCSA,totalSample,immune-iTreg; MCSA,totalSample,immune-Th1; MCSA,totalSample,immune-Th17; MCSA,totalSample,immune-Cytotoxic; MCSA,totalSample,immune-Exhausted; ROSMAP,totalSample,immune-InfiltrationScore
PILRB	Emory,control,male.vs.female; Homo_723,female,AD.vs.control; Homo_723,male,AD.vs.control; Homo_723,female,AD.vs.MCI; Homo_723,male,AD.vs.MCI; Homo_714,female,AD.vs.control; Homo_714,male,AD.vs.control; Homo_714,female,AD.vs.MCI; Homo_714,male,AD.vs.MCI; Homo_633,female,AD.vs.control; Homo_633,female,AD.vs.MCI; ROSMAP,female,AD.vs.control; ROSMAP,male,AD.vs.control; MCSA,MCI,age; Emory,totalSample,immune-Macrophage; Emory,totalSample,immune-Neutrophil; Emory,totalSample,immune-CD4_T; Emory,totalSample,immune-CD8_T; Emory,totalSample,immune-Tr1; Emory,totalSample,immune-iTreg; Emory,totalSample,immune-Tfh; Emory,totalSample,immune-CD8_naive; Homo_723,totalSample,immune-Cytotoxic; Homo_723,totalSample,immune-Th1; Homo_723,totalSample,immune-CD8_T; Homo_723,totalSample,immune-Exhausted; Homo_714,totalSample,immune-Th1; Homo_633,totalSample,immune-DC; Homo_633,totalSample,immune-Th1; Homo_633,totalSample,immune-NKT; MCSA,totalSample,immune-DC; MCSA,totalSample,immune-Monocyte; MCSA,totalSample,immune-CD8_T; MCSA,totalSample,immune-iTreg; MCSA,totalSample,immune-Th2; MCSA,totalSample,immune-Cytotoxic; MCSA,totalSample,immune-Exhausted; MCSA,totalSample,immune-InfiltrationScore; ROSMAP,male-excludeAge90+,survival
BNIP3L	Emory,control,male.vs.female; ROSMAP,female,AD.vs.control; ROSMAP,female,MCI.vs.control; Emory,totalSample,age; Emory,MCI,age; Emory,totalSample,immune-Gamma_delta; Emory,totalSample,immune-CD8_naive; Emory,totalSample,immune-Cytotoxic; Homo_723,totalSample,immune-Bcell; Homo_723,totalSample,immune-Monocyte; Homo_714,totalSample,immune-Monocyte; Homo_714,totalSample,immune-Bcell; Homo_633,totalSample,immune-Monocyte; Homo_633,totalSample,immune-Bcell; MCSA,totalSample,immune-DC; MCSA,totalSample,immune-Monocyte; MCSA,totalSample,immune-CD8_T; MCSA,totalSample,immune-iTreg; MCSA,totalSample,immune-CD8_naive; MCSA,totalSample,immune-Cytotoxic; MCSA,totalSample,immune-Exhausted; MCSA,totalSample,immune-Effector_memory; MCSA,totalSample,immune-InfiltrationScore; SRP223445,totalSample,immune-Exhausted; SRP223445,totalSample,immune-InfiltrationScore; ROSMAP,male-excludeAge90+,survival
RBM38	Emory,control,male.vs.female; Emory,MCI,age; MCSA,MCI,age; Emory,totalSample,immune-Gamma_delta; Emory,totalSample,immune-CD8_naive; Emory,totalSample,immune-Cytotoxic; Homo_723,totalSample,immune-Bcell; Homo_723,totalSample,immune-DC; Homo_714,totalSample,immune-DC; Homo_714,totalSample,immune-Bcell; Homo_633,totalSample,immune-DC; Homo_633,totalSample,immune-Bcell; Homo_633,totalSample,immune-NKT; Homo_633,totalSample,immune-Th2; Homo_633,totalSample,immune-Th1; MCSA,totalSample,immune-DC; MCSA,totalSample,immune-Monocyte; MCSA,totalSample,immune-Macrophage; MCSA,totalSample,immune-Neutrophil; MCSA,totalSample,immune-CD8_T; MCSA,totalSample,immune-NKT; MCSA,totalSample,immune-iTreg; MCSA,totalSample,immune-Th2; MCSA,totalSample,immune-Cytotoxic; MCSA,totalSample,immune-Effector_memory
ARHGEF12	Emory,control,male.vs.female; Homo_723,female,AD.vs.control; Homo_723,male,AD.vs.control; Homo_723,male,AD.vs.MCI; Homo_723,female,MCI.vs.control; Homo_723,AD,male.vs.female; Homo_714,male,AD.vs.control; Homo_714,male,AD.vs.MCI; Homo_714,AD,male.vs.female; Homo_633,male,MCI.vs.control; Emory,MCI,age; Emory,totalSample,immune-Gamma_delta; Emory,totalSample,immune-iTreg; Emory,totalSample,immune-CD8_naive; Homo_723,totalSample,immune-Bcell; MCSA,totalSample,immune-CD4_T; MCSA,totalSample,immune-CD4_naive; MCSA,totalSample,immune-Tr1; MCSA,totalSample,immune-nTreg; MCSA,totalSample,immune-iTreg; MCSA,totalSample,immune-Th17; MCSA,totalSample,immune-CD8_naive; MCSA,totalSample,immune-Exhausted; ROSMAP,totalSample,immune-InfiltrationScore; SRP223445,totalSample,immune-CD4_naive; SRP223445,totalSample,immune-Th1; SRP223445,totalSample,immune-Exhausted
TLDC2	Emory,control,male.vs.female; Homo_714,male,AD.vs.control; ROSMAP,female,AD.vs.control; ROSMAP,female,MCI.vs.control; ACOM,AD,age; MCSA,totalSample,age; MCSA,control,age; ROSMAP,MCI,last.visit.age; ROSMAP,MCI,sample.age; Emory,totalSample,immune-DC; Emory,totalSample,immune-Tr1; Emory,totalSample,immune-iTreg; Emory,totalSample,immune-InfiltrationScore; Homo_723,totalSample,immune-iTreg; Homo_723,totalSample,immune-nTreg; Homo_723,totalSample,immune-Tfh; Homo_723,totalSample,immune-CD4_T; Homo_723,totalSample,immune-Tr1; Homo_723,totalSample,immune-Th2; Homo_723,totalSample,immune-CD4_naive; Homo_714,totalSample,immune-nTreg; Homo_714,totalSample,immune-Tr1; Homo_714,totalSample,immune-Tfh; Homo_714,totalSample,immune-CD4_T; Homo_714,totalSample,immune-iTreg; Homo_714,totalSample,immune-Th2; Homo_714,totalSample,immune-CD4_naive; Homo_714,totalSample,immune-Effector_memory; Homo_633,totalSample,immune-nTreg; Homo_633,totalSample,immune-Tr1; Homo_633,totalSample,immune-CD4_T; Homo_633,totalSample,immune-Tfh; Homo_633,totalSample,immune-Effector_memory; Homo_633,totalSample,immune-CD4_naive; Homo_633,totalSample,immune-iTreg; Homo_633,totalSample,immune-InfiltrationScore; Homo_633,totalSample,immune-Central_memory; MCSA,totalSample,immune-Monocyte; MCSA,totalSample,immune-CD4_T; MCSA,totalSample,immune-Gamma_delta; MCSA,totalSample,immune-CD4_naive; MCSA,totalSample,immune-Tr1; MCSA,totalSample,immune-nTreg; MCSA,totalSample,immune-iTreg; MCSA,totalSample,immune-Th17; MCSA,totalSample,immune-Tfh; MCSA,totalSample,immune-CD8_naive; MCSA,totalSample,immune-Central_memory; MCSA,totalSample,immune-Effector_memory; MCSA,totalSample,immune-InfiltrationScore; ROSMAP,totalSample,immune-Th17; ROSMAP,totalSample,immune-CD8_naive; ROSMAP,totalSample,immune-Cytotoxic; ROSMAP,totalSample,immune-MAIT; ROSMAP,totalSample,immune-Effector_memory; ROSMAP,female-excludeAge90+,survival;
TSPAN5	Emory,control,male.vs.female; Homo_723,female,AD.vs.control; Homo_723,female,AD.vs.MCI; Homo_714,female,AD.vs.control; Homo_714,female,AD.vs.MCI; Homo_714,male,MCI.vs.control; Homo_633,female,AD.vs.control; Homo_633,female,AD.vs.MCI; Homo_633,male,MCI.vs.control; ROSMAP,AD,male.vs.female; Emory,totalSample,age; Emory,MCI,age; Emory,totalSample,immune-Gamma_delta; Emory,totalSample,immune-iTreg; Emory,totalSample,immune-Th17; Emory,totalSample,immune-CD8_naive; Emory,totalSample,immune-Cytotoxic; Homo_723,totalSample,immune-Bcell; Homo_714,totalSample,immune-Bcell; Homo_633,totalSample,immune-Bcell; ROSMAP,totalSample,immune-InfiltrationScore;
MFSD2B	Emory,control,male.vs.female; Homo_723,male,AD.vs.control; Homo_723,male,AD.vs.MCI; Homo_714,male,AD.vs.control; Homo_714,male,AD.vs.MCI; ROSMAP,female,AD.vs.control; ROSMAP,male,AD.vs.control; ROSMAP,female,AD.vs.MCI; ROSMAP,control,male.vs.female; ROSMAP,MCI,male.vs.female; Emory,MCI,age; MCSA,MCI,age; ROSMAP,AD,last.visit.age; Emory,totalSample,immune-Gamma_delta; Emory,totalSample,immune-Tr1; Emory,totalSample,immune-iTreg; Emory,totalSample,immune-InfiltrationScore; Homo_723,totalSample,immune-Bcell; Homo_723,totalSample,immune-DC; Homo_723,totalSample,immune-Th2; Homo_714,totalSample,immune-DC; Homo_714,totalSample,immune-Th2; Homo_714,totalSample,immune-Bcell; Homo_633,totalSample,immune-Bcell; Homo_633,totalSample,immune-Th2; Homo_633,totalSample,immune-DC; MCSA,totalSample,immune-DC; MCSA,totalSample,immune-Macrophage; MCSA,totalSample,immune-iTreg; MCSA,totalSample,immune-MAIT; MCSA,totalSample,immune-Central_memory;
SLC25A39	Emory,control,male.vs.female; ROSMAP,male,AD.vs.control; Emory,MCI,age; MCSA,MCI,age; Emory,totalSample,immune-Gamma_delta; Emory,totalSample,immune-Tr1; Emory,totalSample,immune-iTreg; Emory,totalSample,immune-CD8_naive; Homo_723,totalSample,immune-Bcell; Homo_723,totalSample,immune-DC; Homo_723,totalSample,immune-Th1; Homo_723,totalSample,immune-Cytotoxic; Homo_714,totalSample,immune-DC; Homo_714,totalSample,immune-Bcell; Homo_714,totalSample,immune-Th1; Homo_633,totalSample,immune-DC; Homo_633,totalSample,immune-Bcell; Homo_633,totalSample,immune-Th1; Homo_633,totalSample,immune-Th2; Homo_633,totalSample,immune-NKT; Homo_633,totalSample,immune-CD4_naive; MCSA,totalSample,immune-DC; MCSA,totalSample,immune-Monocyte; MCSA,totalSample,immune-Macrophage; MCSA,totalSample,immune-CD4_T; MCSA,totalSample,immune-CD8_T; MCSA,totalSample,immune-NKT; MCSA,totalSample,immune-Tr1; MCSA,totalSample,immune-iTreg; MCSA,totalSample,immune-Th2; MCSA,totalSample,immune-Cytotoxic; MCSA,totalSample,immune-Effector_memory; SRP223445,totalSample,immune-Neutrophil; SRP223445,totalSample,immune-CD4_naive; SRP223445,totalSample,immune-InfiltrationScore; ROSMAP,male-includeAge90+,survival
ARHGEF37	Emory,control,male.vs.female; Homo_723,female,AD.vs.control; Homo_723,male,AD.vs.control; Homo_723,female,AD.vs.MCI; Homo_723,male,AD.vs.MCI; Homo_714,female,AD.vs.control; Homo_714,male,AD.vs.control; Homo_714,female,AD.vs.MCI; Homo_714,male,AD.vs.MCI; Homo_633,female,AD.vs.control; Homo_633,female,AD.vs.MCI; Emory,MCI,age; ROSMAP,MCI,last.visit.age; Emory,totalSample,immune-Gamma_delta; Emory,totalSample,immune-Tr1; Emory,totalSample,immune-iTreg; Emory,totalSample,immune-CD8_naive; Emory,totalSample,immune-Cytotoxic; Homo_723,totalSample,immune-Bcell; Homo_723,totalSample,immune-DC; Homo_723,totalSample,immune-Neutrophil; Homo_714,totalSample,immune-Th2; Homo_714,totalSample,immune-DC; Homo_714,totalSample,immune-Bcell; Homo_633,totalSample,immune-Bcell; Homo_633,totalSample,immune-DC; MCSA,totalSample,immune-Macrophage; ROSMAP,totalSample,immune-InfiltrationScore; SRP223445,totalSample,immune-CD4_naive; SRP223445,totalSample,immune-InfiltrationScore; ROSMAP,male-
CR1L	Emory,control,male.vs.female; Homo_723,female,AD.vs.control; Homo_723,male,AD.vs.control; Homo_723,female,AD.vs.MCI; Homo_723,male,AD.vs.MCI; Homo_714,female,AD.vs.control; Homo_714,male,AD.vs.control; Homo_714,female,AD.vs.MCI; Homo_714,male,AD.vs.MCI; Homo_633,female,AD.vs.control; Homo_633,female,AD.vs.MCI; Homo_633,female,MCI.vs.control; SRP223445,female,AD.vs.control; ACOM,AD,age; Emory,totalSample,age; Emory,MCI,age; Emory,totalSample,immune-Gamma_delta; Emory,totalSample,immune-iTreg; Emory,totalSample,immune-Th17; Emory,totalSample,immune-CD8_naive; Homo_723,totalSample,immune-Bcell; Homo_723,totalSample,immune-DC; Homo_714,totalSample,immune-Bcell; Homo_714,totalSample,immune-Th2; Homo_633,totalSample,immune-Bcell; ROSMAP,female-excludeAge90+,survival
CTSG	Emory,control,male.vs.female; Homo_723,female,AD.vs.control; Homo_723,control,male.vs.female; Homo_714,female,AD.vs.control; Homo_714,control,male.vs.female; Homo_633,female,AD.vs.control; Homo_633,female,AD.vs.MCI; Homo_633,control,male.vs.female; ROSMAP,male,AD.vs.control; Emory,totalSample,immune-B_cell; Emory,totalSample,immune-Macrophage; Emory,totalSample,immune-CD4_T; Emory,totalSample,immune-nTreg; Emory,totalSample,immune-Tfh; Homo_723,totalSample,immune-Macrophage; Homo_723,totalSample,immune-Th2; Homo_723,totalSample,immune-CD4_T; Homo_723,totalSample,immune-iTreg; Homo_714,totalSample,immune-Macrophage; Homo_714,totalSample,immune-Th2; Homo_714,totalSample,immune-CD4_T; Homo_633,totalSample,immune-Macrophage; Homo_633,totalSample,immune-CD4_T; MCSA,totalSample,immune-Macrophage; MCSA,totalSample,immune-CD4_T; MCSA,totalSample,immune-NKT; MCSA,totalSample,immune-CD4_naive; MCSA,totalSample,immune-Tr1; MCSA,totalSample,immune-nTreg; MCSA,totalSample,immune-iTreg; MCSA,totalSample,immune-Tfh; MCSA,totalSample,immune-Central_memory; ROSMAP,totalSample,immune-InfiltrationScore; SRP223445,totalSample,immune-NK; SRP223445,totalSample,immune-CD4_T; SRP223445,totalSample,immune-Th17; SRP223445,totalSample,immune-InfiltrationScore
XKRX	Emory,control,male.vs.female; MCSA,totalSample,age; MCSA,control,age; Emory,totalSample,immune-B_cell; Emory,totalSample,immune-Macrophage; Emory,totalSample,immune-CD4_T; Emory,totalSample,immune-Tr1; Emory,totalSample,immune-iTreg; Emory,totalSample,immune-CD8_naive; Emory,totalSample,immune-InfiltrationScore; Homo_723,totalSample,immune-iTreg; Homo_723,totalSample,immune-Tr1; Homo_723,totalSample,immune-CD4_T; Homo_723,totalSample,immune-CD4_naive; Homo_723,totalSample,immune-Central_memory; Homo_723,totalSample,immune-Tfh; Homo_723,totalSample,immune-nTreg; Homo_723,totalSample,immune-Th2; Homo_723,totalSample,immune-Th1; Homo_723,totalSample,immune-Macrophage; Homo_714,totalSample,immune-Tr1; Homo_714,totalSample,immune-CD4_T; Homo_714,totalSample,immune-iTreg; Homo_714,totalSample,immune-CD4_naive; Homo_714,totalSample,immune-Central_memory; Homo_714,totalSample,immune-Tfh; Homo_714,totalSample,immune-nTreg; Homo_714,totalSample,immune-Th2; Homo_714,totalSample,immune-Th1; Homo_714,totalSample,immune-Effector_memory; Homo_714,totalSample,immune-Macrophage; Homo_714,totalSample,immune-NKT; Homo_633,totalSample,immune-CD4_T; Homo_633,totalSample,immune-Tr1; Homo_633,totalSample,immune-CD4_naive; Homo_633,totalSample,immune-Tfh; Homo_633,totalSample,immune-Central_memory; Homo_633,totalSample,immune-nTreg; Homo_633,totalSample,immune-iTreg; Homo_633,totalSample,immune-Effector_memory; Homo_633,totalSample,immune-NKT; Homo_633,totalSample,immune-Th1; Homo_633,totalSample,immune-Th2; Homo_633,totalSample,immune-Macrophage; Homo_633,totalSample,immune-Gamma_delta; MCSA,totalSample,immune-DC; MCSA,totalSample,immune-Bcell; MCSA,totalSample,immune-Macrophage; MCSA,totalSample,immune-Neutrophil; MCSA,totalSample,immune-CD4_T; MCSA,totalSample,immune-Gamma_delta; MCSA,totalSample,immune-CD4_naive; MCSA,totalSample,immune-Tr1; MCSA,totalSample,immune-nTreg; MCSA,totalSample,immune-iTreg; MCSA,totalSample,immune-Th17; MCSA,totalSample,immune-Tfh; MCSA,totalSample,immune-CD8_naive; MCSA,totalSample,immune-Central_memory; MCSA,totalSample,immune-Effector_memory; ROSMAP,female-excludeAge90+,survival; ROSMAP,male-excludeAge90+,survival
ANK1	Emory,control,male.vs.female; Homo_714,male,AD.vs.control; ROSMAP,female,AD.vs.control; ROSMAP,male,AD.vs.control; ROSMAP,female,AD.vs.MCI; ROSMAP,female,MCI.vs.control; ROSMAP,MCI,male.vs.female; Emory,totalSample,age; Emory,MCI,age; MCSA,MCI,age; Emory,totalSample,immune-Gamma_delta; Emory,totalSample,immune-CD8_naive; Homo_723,totalSample,immune-Bcell; Homo_723,totalSample,immune-DC; Homo_714,totalSample,immune-Bcell; Homo_714,totalSample,immune-DC; Homo_633,totalSample,immune-Bcell; Homo_633,totalSample,immune-DC; MCSA,totalSample,immune-DC; MCSA,totalSample,immune-Monocyte; MCSA,totalSample,immune-Macrophage; MCSA,totalSample,immune-iTreg; MCSA,totalSample,immune-Th2; ROSMAP,totalSample,immune-InfiltrationScore; SRP223445,totalSample,immune-InfiltrationScore; ROSMAP,female-includeAge90+,survival
GMPR	Emory,control,male.vs.female; Emory,totalSample,age; Emory,MCI,age; MCSA,MCI,age; Emory,totalSample,immune-Gamma_delta; Emory,totalSample,immune-Tr1; Emory,totalSample,immune-iTreg; Emory,totalSample,immune-CD8_naive; Homo_723,totalSample,immune-Bcell; Homo_723,totalSample,immune-iTreg; Homo_723,totalSample,immune-CD4_naive; Homo_723,totalSample,immune-Tr1; Homo_714,totalSample,immune-Bcell; Homo_714,totalSample,immune-iTreg; Homo_714,totalSample,immune-CD4_naive; Homo_714,totalSample,immune-DC; Homo_633,totalSample,immune-Bcell; Homo_633,totalSample,immune-DC; Homo_633,totalSample,immune-CD4_naive; Homo_633,totalSample,immune-NKT; Homo_633,totalSample,immune-iTreg; Homo_633,totalSample,immune-CD4_T; Homo_633,totalSample,immune-Effector_memory; MCSA,totalSample,immune-DC; MCSA,totalSample,immune-Macrophage; MCSA,totalSample,immune-Tr1; MCSA,totalSample,immune-iTreg; MCSA,totalSample,immune-Th2; MCSA,totalSample,immune-MAIT;
BPGM	Emory,control,male.vs.female; Emory,totalSample,age; Emory,MCI,age; ROSMAP,MCI,last.visit.age; ROSMAP,MCI,sample.age; Emory,totalSample,immune-Gamma_delta; Emory,totalSample,immune-CD8_naive; Homo_723,totalSample,immune-Bcell; Homo_723,totalSample,immune-Monocyte; Homo_714,totalSample,immune-Bcell; Homo_714,totalSample,immune-Monocyte; Homo_633,totalSample,immune-Bcell; Homo_633,totalSample,immune-Monocyte; MCSA,totalSample,immune-Monocyte; MCSA,totalSample,immune-Cytotoxic; SRP223445,totalSample,immune-InfiltrationScore
SULT1A2	Emory,control,male.vs.female; Homo_723,male,AD.vs.control; Homo_723,male,AD.vs.MCI; Homo_714,male,AD.vs.control; Homo_714,male,AD.vs.MCI; ACOM,totalSample,age; Emory,MCI,age; MCSA,MCI,age; ROSMAP,MCI,last.visit.age; Emory,totalSample,immune-Neutrophil; Emory,totalSample,immune-CD8_T; Emory,totalSample,immune-Gamma_delta; Emory,totalSample,immune-Cytotoxic; Homo_723,totalSample,immune-DC; Homo_633,totalSample,immune-DC; MCSA,totalSample,immune-DC; MCSA,totalSample,immune-Monocyte; MCSA,totalSample,immune-Macrophage; MCSA,totalSample,immune-iTreg; MCSA,totalSample,immune-Th2; MCSA,totalSample,immune-Cytotoxic; ROSMAP,totalSample,immune-Effector_memory; SRP223445,totalSample,immune-CD4_T; SRP223445,totalSample,immune-InfiltrationScore; ROSMAP,male-excludeAge90+,survival
OR2W3	Emory,control,male.vs.female; Emory,totalSample,age; Emory,MCI,age; ROSMAP,AD,MMSE; Emory,totalSample,immune-Gamma_delta; Emory,totalSample,immune-CD8_naive; Emory,totalSample,immune-Cytotoxic; Homo_723,totalSample,immune-Bcell; Homo_714,totalSample,immune-Bcell; Homo_633,totalSample,immune-Bcell; MCSA,totalSample,immune-DC; MCSA,totalSample,immune-Macrophage; MCSA,totalSample,immune-Th2; ROSMAP,totalSample,immune-InfiltrationScore; SRP223445,totalSample,immune-CD4_naive
RAB3IL1	Emory,control,male.vs.female; Homo_714,male,MCI.vs.control; Homo_633,control,male.vs.female; Homo_633,MCI,male.vs.female; SRP223445,female,AD.vs.control; Emory,MCI,age; ROSMAP,MCI,last.visit.age; Emory,totalSample,immune-Gamma_delta; Emory,totalSample,immune-Tr1; Emory,totalSample,immune-iTreg; Emory,totalSample,immune-CD8_naive; Emory,totalSample,immune-Cytotoxic; Homo_723,totalSample,immune-DC; Homo_723,totalSample,immune-Monocyte; Homo_723,totalSample,immune-Th2; Homo_723,totalSample,immune-Exhausted; Homo_714,totalSample,immune-Th2; Homo_714,totalSample,immune-DC; Homo_714,totalSample,immune-Monocyte; Homo_633,totalSample,immune-Monocyte; Homo_633,totalSample,immune-Th2; Homo_633,totalSample,immune-DC; MCSA,totalSample,immune-DC; MCSA,totalSample,immune-Th2; SRP223445,totalSample,immune-InfiltrationScore
CISD2	Emory,control,male.vs.female; Homo_723,female,AD.vs.control; Homo_723,male,AD.vs.control; Homo_723,female,AD.vs.MCI; Homo_723,male,AD.vs.MCI; Homo_714,female,AD.vs.control; Homo_714,male,AD.vs.control; Homo_714,female,AD.vs.MCI; Homo_714,male,AD.vs.MCI; Emory,totalSample,age; Emory,MCI,age; MCSA,MCI,age; Emory,totalSample,immune-DC; Emory,totalSample,immune-Monocyte; Emory,totalSample,immune-Neutrophil; Emory,totalSample,immune-CD8_T; Emory,totalSample,immune-Gamma_delta; Emory,totalSample,immune-Th17; Emory,totalSample,immune-CD8_naive; Emory,totalSample,immune-Cytotoxic; Homo_723,totalSample,immune-Bcell; Homo_723,totalSample,immune-Th1; Homo_723,totalSample,immune-Neutrophil; Homo_714,totalSample,immune-Th1; Homo_633,totalSample,immune-Th1; MCSA,totalSample,immune-DC; MCSA,totalSample,immune-Monocyte; MCSA,totalSample,immune-Macrophage; MCSA,totalSample,immune-CD8_T; MCSA,totalSample,immune-iTreg; MCSA,totalSample,immune-Th2; MCSA,totalSample,immune-Cytotoxic; MCSA,totalSample,immune-Effector_memory; ROSMAP,totalSample,immune-Effector_memory; SRP223445,totalSample,immune-InfiltrationScore

TENT5C	Emory,control,male.vs.female; Emory,totalSample,age; Emory,MCI,age; ROSMAP,AD,sample.age; ROSMAP,MCI,MMSE; Emory,totalSample,immune-Gamma_delta; Emory,totalSample,immune-CD8_naive; Homo_723,totalSample,immune-Bcell; Homo_714,totalSample,immune-Bcell; Homo_633,totalSample,immune-Bcell; MCSA,totalSample,immune-Cytotoxic; ROSMAP,totalSample,immune-CD4_T; ROSMAP,totalSample,immune-Th17; ROSMAP,totalSample,immune-InfiltrationScore; SRP223445,totalSample,immune-InfiltrationScore
NFIX	Emory,control,male.vs.female; Homo_723,female,AD.vs.control; Homo_723,male,AD.vs.control; Homo_723,female,AD.vs.MCI; Homo_723,male,AD.vs.MCI; Homo_723,male,MCI.vs.control; Homo_714,female,AD.vs.control; Homo_714,male,AD.vs.control; Homo_714,female,AD.vs.MCI; Homo_714,male,MCI.vs.control; Homo_633,male,MCI.vs.control; Homo_633,MCI,male.vs.female; ROSMAP,female,AD.vs.control; ROSMAP,female,MCI.vs.control; ROSMAP,control,male.vs.female; ACOM,totalSample,age; Emory,MCI,age; MCSA,MCI,age; ROSMAP,MCI,last.visit.age; ROSMAP,MCI,sample.age; Emory,totalSample,immune-Monocyte; Emory,totalSample,immune-Gamma_delta; Emory,totalSample,immune-Tr1; Emory,totalSample,immune-iTreg; Emory,totalSample,immune-CD8_naive; Emory,totalSample,immune-InfiltrationScore; Homo_723,totalSample,immune-DC; Homo_723,totalSample,immune-Th2; Homo_723,totalSample,immune-Bcell; Homo_714,totalSample,immune-Th2; Homo_714,totalSample,immune-DC; Homo_633,totalSample,immune-Th2; MCSA,totalSample,immune-DC; MCSA,totalSample,immune-Monocyte; MCSA,totalSample,immune-Th2; MCSA,totalSample,immune-MAIT; SRP223445,totalSample,immune-Neutrophil; SRP223445,totalSample,immune-CD4_naive; SRP223445,totalSample,immune-InfiltrationScore; ROSMAP,female-excludeAge90+,survival
CYP4F12	Emory,control,male.vs.female; Homo_723,control,male.vs.female; Homo_714,male,AD.vs.control; Homo_714,control,male.vs.female; MCSA,MCI,age; ROSMAP,AD,last.visit.age; ROSMAP,MCI,last.visit.age; MCSA,totalSample,immune-Monocyte; MCSA,totalSample,immune-Gamma_delta; MCSA,totalSample,immune-Th17; ROSMAP,totalSample,immune-InfiltrationScore; ROSMAP,male-excludeAge90+,survival
TRIM58	Emory,control,male.vs.female; ROSMAP,female,AD.vs.control; ACOM,totalSample,age; Emory,totalSample,age; Emory,MCI,age; Emory,totalSample,immune-Gamma_delta; Emory,totalSample,immune-CD8_naive; Homo_723,totalSample,immune-Bcell; Homo_723,totalSample,immune-Gamma_delta; Homo_714,totalSample,immune-Bcell; Homo_633,totalSample,immune-Bcell; MCSA,totalSample,immune-Macrophage; SRP223445,totalSample,immune-Exhausted
TMOD1	Emory,control,male.vs.female; Homo_723,male,AD.vs.control; Homo_723,male,MCI.vs.control; Homo_714,female,AD.vs.control; Homo_714,male,AD.vs.control; Homo_714,female,AD.vs.MCI; Homo_714,male,MCI.vs.control; Homo_633,male,MCI.vs.control; Emory,MCI,age; Emory,totalSample,immune-Gamma_delta; Emory,totalSample,immune-CD8_naive; Homo_723,totalSample,immune-Bcell; Homo_714,totalSample,immune-Bcell; Homo_633,totalSample,immune-Bcell; Homo_633,totalSample,immune-Effector_memory; MCSA,totalSample,immune-MAIT; MCSA,totalSample,immune-Central_memory
SGIP1	Emory,control,male.vs.female; Emory,MCI,age; Emory,totalSample,immune-CD4_T; Emory,totalSample,immune-Tr1; Emory,totalSample,immune-iTreg; Emory,totalSample,immune-Central_memory; Homo_723,totalSample,immune-Bcell; Homo_714,totalSample,immune-Bcell; Homo_633,totalSample,immune-Bcell; SRP223445,totalSample,immune-InfiltrationScore
RNF182	Emory,control,male.vs.female; Emory,totalSample,immune-Gamma_delta; Emory,totalSample,immune-iTreg; Emory,totalSample,immune-Cytotoxic
DES	Emory,control,male.vs.female; Homo_723,female,AD.vs.control; Homo_723,male,AD.vs.control; Homo_723,female,AD.vs.MCI; Homo_723,male,AD.vs.MCI; Homo_723,control,male.vs.female; Homo_714,female,AD.vs.control; Homo_714,male,AD.vs.control; Homo_714,female,AD.vs.MCI; Homo_714,male,AD.vs.MCI; Homo_714,control,male.vs.female; Homo_633,female,AD.vs.control; Homo_633,female,AD.vs.MCI; Homo_633,female,MCI.vs.control; Homo_633,male,MCI.vs.control; Homo_633,control,male.vs.female; ACOM,totalSample,age; ROSMAP,AD,MMSE; Homo_723,totalSample,immune-DC; Homo_714,totalSample,immune-Th2; Homo_714,totalSample,immune-DC; ROSMAP,totalSample,immune-
FBXO7	Emory,control,male.vs.female; Emory,MCI,age; ROSMAP,MCI,last.visit.age; Emory,totalSample,immune-Gamma_delta; Emory,totalSample,immune-CD8_naive; Emory,totalSample,immune-Cytotoxic; Homo_723,totalSample,immune-Bcell; Homo_714,totalSample,immune-Bcell; Homo_633,totalSample,immune-Bcell; SRP223445,totalSample,immune-Neutrophil; SRP223445,totalSample,immune-nTreg; SRP223445,totalSample,immune-InfiltrationScore
CXCL5	Emory,control,male.vs.female; Homo_723,female,AD.vs.control; Homo_723,male,AD.vs.control; Homo_723,female,AD.vs.MCI; Homo_723,male,AD.vs.MCI; Homo_723,female,MCI.vs.control; Homo_723,AD,male.vs.female; Homo_723,control,male.vs.female; Homo_714,female,AD.vs.control; Homo_714,male,AD.vs.control; Homo_714,female,AD.vs.MCI; Homo_714,male,AD.vs.MCI; Homo_714,female,MCI.vs.control; Homo_714,AD,male.vs.female; Homo_714,control,male.vs.female; Homo_633,control,male.vs.female; Homo_633,MCI,male.vs.female; ROSMAP,female,AD.vs.control; SRP223445,female,AD.vs.control; SRP223445,AD,male.vs.female; Emory,MCI,age; Homo_723,totalSample,immune-Central_memory; Homo_723,totalSample,immune-Monocyte; Homo_723,totalSample,immune-CD4_naive; Homo_714,totalSample,immune-NKT; Homo_714,totalSample,immune-Th1; Homo_633,totalSample,immune-NKT; Homo_633,totalSample,immune-Th1; MCSA,totalSample,immune-DC; MCSA,totalSample,immune-CD4_T; MCSA,totalSample,immune-CD4_naive; MCSA,totalSample,immune-Tr1; MCSA,totalSample,immune-nTreg; MCSA,totalSample,immune-iTreg; MCSA,totalSample,immune-Th17; MCSA,totalSample,immune-Tfh; MCSA,totalSample,immune-CD8_naive; MCSA,totalSample,immune-Cytotoxic; MCSA,totalSample,immune-Exhausted; MCSA,totalSample,immune-Central_memory; SRP223445,totalSample,immune-
RYR2	Emory,control,male.vs.female; Homo_723,female,AD.vs.control; Homo_723,male,AD.vs.control; Homo_723,female,AD.vs.MCI; Homo_723,male,AD.vs.MCI; Homo_714,female,AD.vs.control; Homo_714,male,AD.vs.control; Homo_714,female,AD.vs.MCI; Homo_714,male,AD.vs.MCI; Homo_633,female,AD.vs.control; Homo_633,female,AD.vs.MCI; Homo_633,female,MCI.vs.control; ROSMAP,male,AD.vs.control; ROSMAP,MCI,last.visit.age; Homo_723,totalSample,immune-DC; Homo_723,totalSample,immune-Th17; ROSMAP,totalSample,immune-InfiltrationScore; SRP223445,totalSample,immune-Exhausted; SRP223445,totalSample,immune-InfiltrationScore; ROSMAP,male and female-excludeAge90+,survival
YOD1	Emory,control,male.vs.female; ROSMAP,female,AD.vs.control; Emory,totalSample,age; Emory,MCI,age; MCSA,MCI,age; Emory,totalSample,immune-CD8_naive; Homo_723,totalSample,immune-Bcell; Homo_723,totalSample,immune-Gamma_delta; Homo_723,totalSample,immune-Cytotoxic; Homo_723,totalSample,immune-Monocyte; Homo_723,totalSample,immune-NKT; Homo_714,totalSample,immune-Monocyte; Homo_633,totalSample,immune-Monocyte; Homo_633,totalSample,immune-NKT; Homo_633,totalSample,immune-Gamma_delta; Homo_633,totalSample,immune-DC; Homo_633,totalSample,immune-Th17; MCSA,totalSample,immune-DC; MCSA,totalSample,immune-Monocyte; MCSA,totalSample,immune-CD4_T; MCSA,totalSample,immune-CD8_T; MCSA,totalSample,immune-Tr1; MCSA,totalSample,immune-iTreg; MCSA,totalSample,immune-CD8_naive; MCSA,totalSample,immune-Cytotoxic; MCSA,totalSample,immune-Exhausted; MCSA,totalSample,immune-Effector_memory; MCSA,totalSample,immune-InfiltrationScore; ROSMAP,totalSample,immune-Monocyte;
PRTN3	Emory,control,male.vs.female; Homo_723,female,AD.vs.control; Homo_723,female,AD.vs.MCI; Homo_714,female,AD.vs.control; Homo_714,female,AD.vs.MCI; ROSMAP,female,AD.vs.control; ROSMAP,totalSample,age; Emory,totalSample,immune-Macrophage; Emory,totalSample,immune-CD4_T; Emory,totalSample,immune-CD8_T; Emory,totalSample,immune-Tfh; Homo_723,totalSample,immune-Th2; Homo_723,totalSample,immune-DC; Homo_723,totalSample,immune-Macrophage; Homo_723,totalSample,immune-Monocyte; Homo_714,totalSample,immune-Th2; Homo_714,totalSample,immune-Tfh; Homo_714,totalSample,immune-Macrophage; Homo_633,totalSample,immune-Th2; Homo_633,totalSample,immune-Monocyte; MCSA,totalSample,immune-Macrophage; MCSA,totalSample,immune-Th2; SRP223445,totalSample,immune-InfiltrationScore
GPR146	Emory,control,male.vs.female; Homo_723,female,AD.vs.control; Homo_723,male,AD.vs.control; Homo_723,male,AD.vs.MCI; Homo_723,female,MCI.vs.control; Homo_723,AD,male.vs.female; Homo_714,male,AD.vs.control; Homo_714,female,AD.vs.MCI; Homo_714,male,AD.vs.MCI; Homo_714,female,MCI.vs.control; Homo_714,male,MCI.vs.control; Homo_714,AD,male.vs.female; Homo_633,male,MCI.vs.control; Emory,MCI,age; MCSA,MCI,age; Emory,totalSample,immune-Gamma_delta; Emory,totalSample,immune-Tr1; Emory,totalSample,immune-iTreg; Emory,totalSample,immune-CD8_naive; Homo_723,totalSample,immune-DC; Homo_723,totalSample,immune-Monocyte; Homo_723,totalSample,immune-Th2; Homo_714,totalSample,immune-Th2; Homo_714,totalSample,immune-DC; Homo_633,totalSample,immune-Th2; Homo_633,totalSample,immune-DC; MCSA,totalSample,immune-DC; MCSA,totalSample,immune-Monocyte; MCSA,totalSample,immune-Macrophage; MCSA,totalSample,immune-Neutrophil; MCSA,totalSample,immune-CD8_T; MCSA,totalSample,immune-NKT; MCSA,totalSample,immune-iTreg; MCSA,totalSample,immune-Th2; MCSA,totalSample,immune-Cytotoxic; MCSA,totalSample,immune-Effector_memory; ROSMAP,totalSample,immune-Neutrophil
MMP28	Emory,control,male.vs.female; Homo_723,male,AD.vs.MCI; Homo_723,male,MCI.vs.control; Homo_723,MCI,male.vs.female; Homo_714,male,AD.vs.MCI; Homo_714,male,MCI.vs.control; Homo_714,MCI,male.vs.female; Homo_633,female,AD.vs.control; Homo_633,female,AD.vs.MCI; Homo_633,control,male.vs.female; Homo_633,MCI,male.vs.female; MCSA,totalSample,age; MCSA,control,age; MCSA,MCI,age; Emory,totalSample,immune-Monocyte; Emory,totalSample,immune-Macrophage; Emory,totalSample,immune-CD4_T; Emory,totalSample,immune-CD8_T; Emory,totalSample,immune-CD4_naive; Emory,totalSample,immune-Tr1; Emory,totalSample,immune-iTreg; Emory,totalSample,immune-Tfh; Emory,totalSample,immune-CD8_naive; Emory,totalSample,immune-Cytotoxic; Emory,totalSample,immune-MAIT; Emory,totalSample,immune-Central_memory; Homo_723,totalSample,immune-Central_memory; Homo_723,totalSample,immune-Tfh; Homo_723,totalSample,immune-CD4_naive; Homo_723,totalSample,immune-nTreg; Homo_723,totalSample,immune-CD4_T; Homo_723,totalSample,immune-Tr1; Homo_723,totalSample,immune-iTreg; Homo_723,totalSample,immune-Th2; Homo_723,totalSample,immune-CD8_T; Homo_723,totalSample,immune-Effector_memory; Homo_723,totalSample,immune-Th17; Homo_714,totalSample,immune-nTreg; Homo_714,totalSample,immune-Tfh; Homo_714,totalSample,immune-Central_memory; Homo_714,totalSample,immune-CD4_naive; Homo_714,totalSample,immune-CD4_T; Homo_714,totalSample,immune-Tr1; Homo_714,totalSample,immune-Th2; Homo_714,totalSample,immune-Effector_memory; Homo_714,totalSample,immune-iTreg; Homo_714,totalSample,immune-Th17; Homo_633,totalSample,immune-Tfh; Homo_633,totalSample,immune-Central_memory; Homo_633,totalSample,immune-nTreg; Homo_633,totalSample,immune-CD4_T; Homo_633,totalSample,immune-Tr1; Homo_633,totalSample,immune-CD4_naive; Homo_633,totalSample,immune-Th2; Homo_633,totalSample,immune-iTreg; Homo_633,totalSample,immune-Effector_memory; Homo_633,totalSample,immune-Gamma_delta; Homo_633,totalSample,immune-Th17; MCSA,totalSample,immune-Monocyte; MCSA,totalSample,immune-Neutrophil; MCSA,totalSample,immune-CD4_T; MCSA,totalSample,immune-CD8_T; MCSA,totalSample,immune-CD4_naive; MCSA,totalSample,immune-Tr1; MCSA,totalSample,immune-nTreg; MCSA,totalSample,immune-iTreg; MCSA,totalSample,immune-Th17; [illegible]
PFDN5	Emory,control,male.vs.female; ROSMAP,female,AD.vs.control; Emory,MCI,age; MCSA,totalSample,age; MCSA,control,age; ROSMAP,MCI,last.visit.age; ROSMAP,MCI,sample.age; Emory,totalSample,immune-DC; Emory,totalSample,immune-Monocyte; Emory,totalSample,immune-Neutrophil; Emory,totalSample,immune-CD8_T; Emory,totalSample,immune-CD4_naive; Emory,totalSample,immune-Tr1; Emory,totalSample,immune-nTreg; Emory,totalSample,immune-Th17; Emory,totalSample,immune-Tfh; Emory,totalSample,immune-Cytotoxic; Emory,totalSample,immune-Effector_memory; Homo_723,totalSample,immune-Neutrophil; Homo_723,totalSample,immune-Monocyte; Homo_723,totalSample,immune-Bcell; Homo_714,totalSample,immune-Monocyte; Homo_714,totalSample,immune-Neutrophil; Homo_714,totalSample,immune-NK; Homo_633,totalSample,immune-Monocyte; Homo_633,totalSample,immune-Cytotoxic; Homo_633,totalSample,immune-nTreg; MCSA,totalSample,immune-DC; MCSA,totalSample,immune-Monocyte; MCSA,totalSample,immune-CD4_T; MCSA,totalSample,immune-Gamma_delta; MCSA,totalSample,immune-CD4_naive; MCSA,totalSample,immune-Tr1; MCSA,totalSample,immune-nTreg; MCSA,totalSample,immune-iTreg; MCSA,totalSample,immune-Th1; MCSA,totalSample,immune-Th2; MCSA,totalSample,immune-Th17; MCSA,totalSample,immune-Tfh; MCSA,totalSample,immune-Exhausted; MCSA,totalSample,immune-Central_memory; MCSA,totalSample,immune-Effector_memory; MCSA,totalSample,immune-InfiltrationScore; SRP223445,totalSample,immune-NK; SRP223445,totalSample,immune-CD4_naive; SRP223445,totalSample,immune-Th17; SRP223445,totalSample,immune-Exhausted; SRP223445,totalSample,immune-InfiltrationScore
TLCD4-RWDD3	Emory,control,male.vs.female; ROSMAP,female,AD.vs.control; ACOM,AD,age; Emory,MCI,age; Emory,totalSample,immune-Th17; Emory,totalSample,immune-Cytotoxic; Homo_723,totalSample,immune-Bcell; Homo_714,totalSample,immune-Bcell; Homo_633,totalSample,immune-Bcell; Homo_633,totalSample,immune-NKT; MCSA,totalSample,immune-DC; MCSA,totalSample,immune-Bcell; MCSA,totalSample,immune-Monocyte; MCSA,totalSample,immune-CD4_T; MCSA,totalSample,immune-CD8_T; MCSA,totalSample,immune-CD4_naive; MCSA,totalSample,immune-Tr1; MCSA,totalSample,immune-nTreg; MCSA,totalSample,immune-iTreg; MCSA,totalSample,immune-CD8_naive; MCSA,totalSample,immune-Cytotoxic; MCSA,totalSample,immune-Exhausted; ROSMAP,totalSample,immune-Th17; ROSMAP,totalSample,immune-InfiltrationScore
DNAJC6	Emory,control,male.vs.female; Homo_723,control,male.vs.female; Homo_714,control,male.vs.female; Homo_633,female,MCI.vs.control; Homo_633,male,MCI.vs.control; Homo_633,control,male.vs.female; ROSMAP,male,AD.vs.control; Emory,MCI,age; Emory,totalSample,immune-CD8_naive; Homo_723,totalSample,immune-Central_memory; Homo_723,totalSample,immune-CD4_T; Homo_723,totalSample,immune-Bcell; Homo_714,totalSample,immune-Central_memory; Homo_714,totalSample,immune-Tfh; Homo_633,totalSample,immune-Tfh; Homo_633,totalSample,immune-Central_memory; MCSA,totalSample,immune-DC; MCSA,totalSample,immune-CD4_T; MCSA,totalSample,immune-CD4_naive; MCSA,totalSample,immune-Tr1; MCSA,totalSample,immune-nTreg; MCSA,totalSample,immune-iTreg; MCSA,totalSample,immune-Th17; MCSA,totalSample,immune-Tfh; MCSA,totalSample,immune-CD8_naive; MCSA,totalSample,immune-Cytotoxic; MCSA,totalSample,immune-Exhausted; MCSA,totalSample,immune-Central_memory; ROSMAP,totalSample,immune-InfiltrationScore; SRP223445,totalSample,immune-CD4_naive; SRP223445,totalSample,immune-Th17; SRP223445,totalSample,immune-Exhausted; SRP223445,totalSample,immune-InfiltrationScore
GLRX5	Emory,control,male.vs.female; Emory,totalSample,age; Emory,MCI,age; Emory,totalSample,immune-Gamma_delta; Emory,totalSample,immune-CD8_naive; Emory,totalSample,immune-Cytotoxic; Homo_723,totalSample,immune-Bcell; Homo_714,totalSample,immune-Bcell; Homo_633,totalSample,immune-Bcell; Homo_633,totalSample,immune-Monocyte; MCSA,totalSample,immune-Monocyte; SRP223445,totalSample,immune-Neutrophil; SRP223445,totalSample,immune-InfiltrationScore
IFI44L	Emory,control,male.vs.female; Homo_723,female,AD.vs.control; Homo_723,AD,male.vs.female; Homo_714,female,AD.vs.control; Homo_723,totalSample,immune-Th1; Homo_714,totalSample,immune-Th1; Homo_633,totalSample,immune-Th1; MCSA,totalSample,immune-CD8_T; MCSA,totalSample,immune-Cytotoxic; SRP223445,totalSample,immune-CD4_naive; SRP223445,totalSample,immune-Exhausted; SRP223445,totalSample,immune-InfiltrationScore
CLC	Emory,control,male.vs.female; Homo_723,female,AD.vs.control; Homo_723,male,AD.vs.control; Homo_723,female,AD.vs.MCI; Homo_723,male,AD.vs.MCI; Homo_723,female,MCI.vs.control; Homo_723,male,MCI.vs.control; Homo_633,female,MCI.vs.control; MCSA,totalSample,age; MCSA,control,age; ROSMAP,MCI,last.visit.age; Emory,totalSample,immune-Monocyte; Emory,totalSample,immune-CD4_naive; Emory,totalSample,immune-Cytotoxic; Emory,totalSample,immune-Central_memory; Homo_723,totalSample,immune-Monocyte; Homo_723,totalSample,immune-DC; Homo_714,totalSample,immune-Central_memory; Homo_633,totalSample,immune-Effector_memory; MCSA,totalSample,immune-Monocyte; MCSA,totalSample,immune-CD4_T; MCSA,totalSample,immune-CD4_naive; MCSA,totalSample,immune-Tr1; MCSA,totalSample,immune-nTreg; MCSA,totalSample,immune-Th2; MCSA,totalSample,immune-Th17; MCSA,totalSample,immune-Tfh; MCSA,totalSample,immune-Central_memory; MCSA,totalSample,immune-Effector_memory;
DEFA1B	Emory,control,male.vs.female; Homo_723,female,AD.vs.control; Homo_723,male,AD.vs.control; Homo_723,female,AD.vs.MCI; Homo_723,male,AD.vs.MCI; Homo_723,control,male.vs.female; Homo_714,control,male.vs.female; Homo_633,control,male.vs.female; ROSMAP,MCI,last.visit.age; ROSMAP,MCI,sample.age; Emory,totalSample,immune-Macrophage; Emory,totalSample,immune-NK; Emory,totalSample,immune-nTreg; Homo_723,totalSample,immune-Th1; Homo_723,totalSample,immune-Macrophage; Homo_723,totalSample,immune-Cytotoxic; Homo_723,totalSample,immune-Monocyte; Homo_714,totalSample,immune-Macrophage; Homo_714,totalSample,immune-Th2; Homo_714,totalSample,immune-CD4_T; Homo_714,totalSample,immune-Tr1; Homo_714,totalSample,immune-iTreg; Homo_633,totalSample,immune-Macrophage; Homo_633,totalSample,immune-Th2; Homo_633,totalSample,immune-CD4_T; MCSA,totalSample,immune-Macrophage; MCSA,totalSample,immune-CD4_T; MCSA,totalSample,immune-NKT; MCSA,totalSample,immune-CD4_naive; MCSA,totalSample,immune-Tr1; MCSA,totalSample,immune-nTreg; MCSA,totalSample,immune-iTreg; MCSA,totalSample,immune-Th2; MCSA,totalSample,immune-Tfh; MCSA,totalSample,immune-Central_memory; SRP223445,totalSample,immune-Macrophage; SRP223445,totalSample,immune-CD4_T; SRP223445,totalSample,immune-CD4_naive
DCAF12	Emory,control,male.vs.female; ACOM,totalSample,age; Emory,totalSample,age; Emory,MCI,age; Emory,totalSample,immune-Gamma_delta; Emory,totalSample,immune-CD8_naive; Homo_723,totalSample,immune-Bcell; Homo_714,totalSample,immune-Bcell; Homo_633,totalSample,immune-Bcell; ROSMAP,totalSample,immune-iTreg; ROSMAP,totalSample,immune-Th2; ROSMAP,totalSample,immune-Th17; ROSMAP,totalSample,immune-Effector_memory; SRP223445,totalSample,immune-InfiltrationScore; ROSMAP,female-excludeAge90+,survival
GPRC5B	Emory,control,male.vs.female; Homo_723,female,AD.vs.control; Homo_723,male,AD.vs.control; Homo_723,female,AD.vs.MCI; Homo_723,male,AD.vs.MCI; Homo_723,female,MCI.vs.control; Homo_723,male,MCI.vs.control; Homo_714,female,AD.vs.control; Homo_714,male,AD.vs.control; Homo_714,female,AD.vs.MCI; Homo_714,male,AD.vs.MCI; Homo_633,female,AD.vs.control; Homo_633,female,AD.vs.MCI; Homo_633,male,MCI.vs.control; Homo_633,control,male.vs.female; Emory,totalSample,immune-CD4_T; Emory,totalSample,immune-Gamma_delta; Emory,totalSample,immune-CD4_naive; Emory,totalSample,immune-Tr1; Emory,totalSample,immune-iTreg; Emory,totalSample,immune-Tfh; Emory,totalSample,immune-Cytotoxic; Emory,totalSample,immune-Central_memory; Homo_723,totalSample,immune-Central_memory; Homo_723,totalSample,immune-Th1; Homo_723,totalSample,immune-nTreg; Homo_723,totalSample,immune-DC; Homo_723,totalSample,immune-Cytotoxic; Homo_723,totalSample,immune-CD4_naive; Homo_723,totalSample,immune-Th17; Homo_714,totalSample,immune-Th1; Homo_714,totalSample,immune-Cytotoxic; Homo_714,totalSample,immune-CD4_naive; Homo_714,totalSample,immune-nTreg; Homo_633,totalSample,immune-Tr1; Homo_633,totalSample,immune-Tfh; Homo_633,totalSample,immune-nTreg; Homo_633,totalSample,immune-Gamma_delta; Homo_633,totalSample,immune-iTreg; Homo_633,totalSample,immune-Th1; Homo_633,totalSample,immune-Central_memory; Homo_633,totalSample,immune-CD4_T; Homo_633,totalSample,immune-CD4_naive; Homo_633,totalSample,immune-Cytotoxic; MCSA,totalSample,immune-CD4_T; MCSA,totalSample,immune-Gamma_delta; MCSA,totalSample,immune-CD4_naive; MCSA,totalSample,immune-Tr1; MCSA,totalSample,immune-nTreg; MCSA,totalSample,immune-iTreg; MCSA,totalSample,immune-Th17; MCSA,totalSample,immune-Tfh; MCSA,totalSample,immune-Cytotoxic; MCSA,totalSample,immune-Central_memory; ROSMAP,female-includeAge90+,survival; ROSMAP,male and female-excludeAge90+,survival
PAGE2B	Emory,control,male.vs.female; Homo_723,male,AD.vs.control; Homo_723,male,AD.vs.MCI; Homo_714,male,AD.vs.control; Homo_714,male,AD.vs.MCI; Homo_633,male,MCI.vs.control; Emory,totalSample,age; Emory,MCI,age; MCSA,totalSample,age; MCSA,control,age; Emory,totalSample,immune-Gamma_delta; Emory,totalSample,immune-CD8_naive; Emory,totalSample,immune-Cytotoxic; Homo_723,totalSample,immune-Bcell; Homo_723,totalSample,immune-Exhausted; Homo_723,totalSample,immune-Monocyte; Homo_633,totalSample,immune-Monocyte; Homo_633,totalSample,immune-Effector_memory; Homo_633,totalSample,immune-Th2; Homo_633,totalSample,immune-nTreg; Homo_633,totalSample,immune-Bcell; Homo_633,totalSample,immune-CD4_T; Homo_633,totalSample,immune-iTreg; Homo_633,totalSample,immune-CD4_naive; MCSA,totalSample,immune-Monocyte; MCSA,totalSample,immune-CD4_T; MCSA,totalSample,immune-Gamma_delta; MCSA,totalSample,immune-CD4_naive; MCSA,totalSample,immune-Tr1; MCSA,totalSample,immune-nTreg; MCSA,totalSample,immune-iTreg; MCSA,totalSample,immune-Th17; MCSA,totalSample,immune-Tfh; MCSA,totalSample,immune-Central_memory; MCSA,totalSample,immune-Effector_memory; MCSA,totalSample,immune-InfiltrationScore
MKRN3	Emory,control,male.vs.female; Homo_714,male,AD.vs.MCI; Emory,totalSample,immune-CD4_T; Emory,totalSample,immune-Tr1; Emory,totalSample,immune-iTreg; Emory,totalSample,immune-Central_memory; Homo_723,totalSample,immune-iTreg; Homo_723,totalSample,immune-CD4_naive; Homo_723,totalSample,immune-CD4_T; Homo_723,totalSample,immune-Tr1; Homo_723,totalSample,immune-nTreg; Homo_723,totalSample,immune-Tfh; Homo_723,totalSample,immune-Central_memory; Homo_723,totalSample,immune-NKT; Homo_723,totalSample,immune-Effector_memory; Homo_723,totalSample,immune-Monocyte; Homo_714,totalSample,immune-iTreg; Homo_714,totalSample,immune-CD4_naive; Homo_714,totalSample,immune-Effector_memory; Homo_714,totalSample,immune-CD4_T; Homo_714,totalSample,immune-nTreg; Homo_714,totalSample,immune-Tfh; Homo_714,totalSample,immune-Tr1; Homo_714,totalSample,immune-NKT; Homo_714,totalSample,immune-Central_memory; Homo_633,totalSample,immune-CD4_naive; Homo_633,totalSample,immune-iTreg; Homo_633,totalSample,immune-Effector_memory; Homo_633,totalSample,immune-CD4_T; Homo_633,totalSample,immune-Tr1; Homo_633,totalSample,immune-NKT; Homo_633,totalSample,immune-nTreg; Homo_633,totalSample,immune-Tfh; Homo_633,totalSample,immune-Central_memory; Homo_633,totalSample,immune-InfiltrationScore; MCSA,totalSample,immune-CD4_T; MCSA,totalSample,immune-CD4_naive; MCSA,totalSample,immune-Tr1; MCSA,totalSample,immune-nTreg; MCSA,totalSample,immune-iTreg; MCSA,totalSample,immune-Tfh; MCSA,totalSample,immune-CD8_naive; MCSA,totalSample,immune-Exhausted; MCSA,totalSample,immune-MAIT; MCSA,totalSample,immune-Central_memory; MCSA,totalSample,immune-Effector_memory; ROSMAP,female-excludeAge90+,survival; ROSMAP,female-includeAge90+,survival; ROSMAP,male and female-excludeAge90+,survival; ROSMAP,male and female-includeAge90+,survival
NUP210L	Emory,control,male.vs.female; Homo_723,male,AD.vs.control; Homo_723,female,AD.vs.MCI; Homo_723,female,MCI.vs.control; Homo_723,male,MCI.vs.control; Homo_714,female,AD.vs.control; Homo_714,male,AD.vs.control; Homo_714,female,MCI.vs.control; Homo_714,male,MCI.vs.control; Homo_633,female,AD.vs.control; Homo_633,female,AD.vs.MCI; Homo_633,female,MCI.vs.control; Homo_633,male,MCI.vs.control; Homo_633,control,male.vs.female; MCSA,female,MCI.vs.control; MCSA,male,MCI.vs.control; ROSMAP,AD,last.visit.age; MCSA,totalSample,immune-CD4_T; MCSA,totalSample,immune-Gamma_delta; MCSA,totalSample,immune-CD4_naive; MCSA,totalSample,immune-Tr1; MCSA,totalSample,immune-nTreg; MCSA,totalSample,immune-iTreg; MCSA,totalSample,immune-Th17; MCSA,totalSample,immune-Tfh; MCSA,totalSample,immune-CD8_naive; MCSA,totalSample,immune-Cytotoxic; MCSA,totalSample,immune-Exhausted; MCSA,totalSample,immune-Central_memory; ROSMAP,totalSample,immune-InfiltrationScore;

ACAD11	Emory,control,male.vs.female; Homo_723,female,AD.vs.MCI; Homo_723,female,MCI.vs.control; Homo_723,male,MCI.vs.control; Homo_714,female,AD.vs.control; Homo_714,male,AD.vs.control; Homo_714,female,AD.vs.MCI; Homo_714,male,AD.vs.MCI; Homo_714,female,MCI.vs.control; Homo_714,male,MCI.vs.control; Homo_633,female,AD.vs.control; Homo_633,female,AD.vs.MCI; Homo_633,female,MCI.vs.control; Homo_633,male,MCI.vs.control; MCSA,MCI,age; Emory,totalSample,immune-Neutrophil; Emory,totalSample,immune-CD4_T; Emory,totalSample,immune-Tr1; Emory,totalSample,immune-iTreg; Emory,totalSample,immune-InfiltrationScore; Homo_723,totalSample,immune-Th2; Homo_723,totalSample,immune-Tfh; Homo_723,totalSample,immune-iTreg; Homo_723,totalSample,immune-CD4_T; Homo_723,totalSample,immune-Tr1; Homo_723,totalSample,immune-Effector_memory; Homo_723,totalSample,immune-CD4_naive; Homo_723,totalSample,immune-Monocyte; Homo_723,totalSample,immune-DC; Homo_723,totalSample,immune-Macrophage; Homo_723,totalSample,immune-nTreg; Homo_714,totalSample,immune-Th2; Homo_714,totalSample,immune-Tfh; Homo_714,totalSample,immune-CD4_T; Homo_714,totalSample,immune-Tr1; Homo_714,totalSample,immune-Effector_memory; Homo_714,totalSample,immune-Central_memory; Homo_714,totalSample,immune-CD4_naive; Homo_714,totalSample,immune-iTreg; Homo_714,totalSample,immune-Monocyte; Homo_633,totalSample,immune-Th2; Homo_633,totalSample,immune-Tfh; Homo_633,totalSample,immune-CD4_T; Homo_633,totalSample,immune-Central_memory; Homo_633,totalSample,immune-Effector_memory; Homo_633,totalSample,immune-CD4_naive; Homo_633,totalSample,immune-Monocyte; Homo_633,totalSample,immune-Tr1; Homo_633,totalSample,immune-Th17; Homo_633,totalSample,immune-Bcell; Homo_633,totalSample,immune-InfiltrationScore; MCSA,totalSample,immune-DC; MCSA,totalSample,immune-Macrophage; MCSA,totalSample,immune-CD4_T; MCSA,totalSample,immune-CD4_naive; MCSA,totalSample,immune-Tr1; MCSA,totalSample,immune-nTreg; MCSA,totalSample,immune-iTreg; MCSA,totalSample,immune-Th2; MCSA,totalSample,immune-Th17; MCSA,totalSample,immune-Tfh; MCSA,totalSample,immune-CD8_naive; MCSA,totalSample,immune-Cytotoxic; MCSA,totalSample,immune-MAIT; MCSA,totalSample,immune-Central_memory; [illegible]
EPB42	Emory,control,male.vs.female; Homo_723,female,AD.vs.control; Homo_723,male,AD.vs.control; Homo_723,female,AD.vs.MCI; Homo_723,male,AD.vs.MCI; Homo_723,male,MCI.vs.control; Homo_723,AD,male.vs.female; Homo_714,female,AD.vs.control; Homo_714,male,AD.vs.control; Homo_714,female,AD.vs.MCI; Homo_714,male,AD.vs.MCI; Homo_714,male,MCI.vs.control; Homo_714,AD,male.vs.female; Homo_633,female,AD.vs.control; Homo_633,female,AD.vs.MCI; Homo_633,male,MCI.vs.control; Homo_633,MCI,male.vs.female; Emory,totalSample,age; Emory,MCI,age; Emory,totalSample,immune-Gamma_delta; Emory,totalSample,immune-Tr1; Emory,totalSample,immune-iTreg; Emory,totalSample,immune-CD8_naive; Emory,totalSample,immune-Cytotoxic; Homo_723,totalSample,immune-Bcell; Homo_723,totalSample,immune-Th2; Homo_714,totalSample,immune-Bcell; Homo_714,totalSample,immune-Th2; Homo_633,totalSample,immune-Bcell; Homo_633,totalSample,immune-Th2; MCSA,totalSample,immune-Macrophage;
TNS1	Emory,control,male.vs.female; Homo_723,male,AD.vs.control; Homo_714,male,AD.vs.control; Homo_714,male,AD.vs.MCI; SRP223445,male,AD.vs.control; Emory,totalSample,age; Emory,MCI,age; MCSA,MCI,age; Emory,totalSample,immune-Gamma_delta; Emory,totalSample,immune-Tr1; Emory,totalSample,immune-iTreg; Emory,totalSample,immune-CD8_naive; Homo_723,totalSample,immune-Bcell; Homo_714,totalSample,immune-Bcell; Homo_714,totalSample,immune-DC; Homo_633,totalSample,immune-Bcell; Homo_633,totalSample,immune-DC; MCSA,totalSample,immune-DC; MCSA,totalSample,immune-Macrophage; MCSA,totalSample,immune-iTreg; MCSA,totalSample,immune-MAIT; ROSMAP,totalSample,immune-Effector_memory; SRP223445,totalSample,immune-Neutrophil; SRP223445,totalSample,immune-CD4_naive; SRP223445,totalSample,immune-nTreg; SRP223445,totalSample,immune-InfiltrationScore; ROSMAP,male-excludeAge90+,survival
LAMP3	Emory,control,male.vs.female; Homo_723,female,AD.vs.control; Homo_723,female,AD.vs.MCI; Homo_723,male,MCI.vs.control; Homo_714,female,AD.vs.control; Homo_714,male,MCI.vs.control; Homo_633,female,AD.vs.control; Homo_633,female,AD.vs.MCI; Homo_633,female,MCI.vs.control; ROSMAP,female,AD.vs.control; ROSMAP,female,AD.vs.MCI; ROSMAP,control,male.vs.female; ROSMAP,MCI,male.vs.female; Emory,totalSample,immune-Cytotoxic; Emory,totalSample,immune-Central_memory; Homo_723,totalSample,immune-Tfh; Homo_723,totalSample,immune-Th2; Homo_723,totalSample,immune-iTreg; Homo_723,totalSample,immune-InfiltrationScore; Homo_723,totalSample,immune-Tr1; Homo_723,totalSample,immune-CD4_T; Homo_723,totalSample,immune-CD4_naive; Homo_723,totalSample,immune-Gamma_delta; Homo_723,totalSample,immune-Central_memory; Homo_714,totalSample,immune-Tfh; Homo_714,totalSample,immune-Th2; Homo_714,totalSample,immune-CD4_T; Homo_714,totalSample,immune-Tr1; Homo_714,totalSample,immune-Gamma_delta; Homo_714,totalSample,immune-Central_memory; Homo_714,totalSample,immune-CD4_naive; Homo_633,totalSample,immune-Tfh; Homo_633,totalSample,immune-Th2; Homo_633,totalSample,immune-CD4_T; Homo_633,totalSample,immune-CD4_naive; Homo_633,totalSample,immune-Tr1; Homo_633,totalSample,immune-Gamma_delta; Homo_633,totalSample,immune-Central_memory; Homo_633,totalSample,immune-Th17; Homo_633,totalSample,immune-nTreg; Homo_633,totalSample,immune-Th1; Homo_633,totalSample,immune-InfiltrationScore; Homo_633,totalSample,immune-Bcell; MCSA,totalSample,immune-CD4_T; MCSA,totalSample,immune-Gamma_delta; MCSA,totalSample,immune-CD4_naive; MCSA,totalSample,immune-Tr1; MCSA,totalSample,immune-nTreg; MCSA,totalSample,immune-iTreg; MCSA,totalSample,immune-Th17; MCSA,totalSample,immune-Tfh; MCSA,totalSample,immune-Cytotoxic
TSPAN6	Emory,control,male.vs.female; Homo_723,female,AD.vs.control; Homo_723,male,AD.vs.control; Homo_723,female,AD.vs.MCI; Homo_723,male,AD.vs.MCI; Homo_723,female,MCI.vs.control; Homo_723,male,MCI.vs.control; Homo_714,female,AD.vs.control; Homo_714,male,AD.vs.control; Homo_714,female,AD.vs.MCI; Homo_714,male,AD.vs.MCI; Homo_633,female,AD.vs.control; Homo_633,female,AD.vs.MCI; Emory,totalSample,immune-DC; Emory,totalSample,immune-Macrophage; Emory,totalSample,immune-CD4_T; Emory,totalSample,immune-CD8_T; Emory,totalSample,immune-Tr1; Emory,totalSample,immune-nTreg; Emory,totalSample,immune-iTreg; Emory,totalSample,immune-Tfh; Emory,totalSample,immune-CD8_naive; Emory,totalSample,immune-Central_memory; Homo_723,totalSample,immune-CD4_T; Homo_723,totalSample,immune-Tfh; Homo_723,totalSample,immune-CD4_naive; Homo_723,totalSample,immune-Tr1; Homo_723,totalSample,immune-Central_memory; Homo_723,totalSample,immune-nTreg; Homo_723,totalSample,immune-Th2; Homo_723,totalSample,immune-iTreg; Homo_723,totalSample,immune-Th1; Homo_723,totalSample,immune-Th17; Homo_723,totalSample,immune-Cytotoxic; Homo_714,totalSample,immune-Tfh; Homo_714,totalSample,immune-CD4_naive; Homo_714,totalSample,immune-CD4_T; Homo_714,totalSample,immune-Central_memory; Homo_714,totalSample,immune-Tr1; Homo_714,totalSample,immune-nTreg; Homo_714,totalSample,immune-iTreg; Homo_714,totalSample,immune-Th2; Homo_714,totalSample,immune-Effector_memory; Homo_714,totalSample,immune-Th1; Homo_714,totalSample,immune-Th17; Homo_633,totalSample,immune-Tfh; Homo_633,totalSample,immune-CD4_T; Homo_633,totalSample,immune-Central_memory; Homo_633,totalSample,immune-CD4_naive; Homo_633,totalSample,immune-Tr1; Homo_633,totalSample,immune-iTreg; Homo_633,totalSample,immune-nTreg; Homo_633,totalSample,immune-Th2; Homo_633,totalSample,immune-Effector_memory; Homo_633,totalSample,immune-Macrophage; Homo_633,totalSample,immune-Th17; Homo_633,totalSample,immune-Gamma_delta; MCSA,totalSample,immune-DC; MCSA,totalSample,immune-Macrophage; MCSA,totalSample,immune-CD4_T; MCSA,totalSample,immune-CD4_naive; MCSA,totalSample,immune-Tr1; MCSA,totalSample,immune-nTreg; MCSA,totalSample,immune-iTreg; MCSA,totalSample,immune-Tfh; MCSA,totalSample,immune-[illegible]
CTNNAL1	Emory,control,male.vs.female; Homo_723,female,AD.vs.control; Homo_723,male,AD.vs.control; Homo_723,female,AD.vs.MCI; Homo_723,male,AD.vs.MCI; Homo_714,female,AD.vs.control; Homo_714,male,AD.vs.control; Homo_714,female,AD.vs.MCI; Homo_714,male,AD.vs.MCI; Homo_633,female,AD.vs.control; Homo_633,female,AD.vs.MCI; Emory,totalSample,age; Emory,MCI,age; MCSA,totalSample,age; MCSA,control,age; Emory,totalSample,immune-Gamma_delta; Emory,totalSample,immune-CD8_naive; Homo_723,totalSample,immune-Bcell; Homo_723,totalSample,immune-Th17; Homo_723,totalSample,immune-Monocyte; Homo_723,totalSample,immune-Neutrophil; Homo_723,totalSample,immune-CD8_T; Homo_714,totalSample,immune-Monocyte; Homo_633,totalSample,immune-Monocyte; MCSA,totalSample,immune-DC; MCSA,totalSample,immune-Monocyte; MCSA,totalSample,immune-CD8_T; MCSA,totalSample,immune-Th2; MCSA,totalSample,immune-Cytotoxic; MCSA,totalSample,immune-Effector_memory; MCSA,totalSample,immune-InfiltrationScore;
MTRNR2L10	Emory,control,male.vs.female; Homo_723,female,AD.vs.control; Homo_723,male,AD.vs.control; Homo_723,female,MCI.vs.control; Homo_723,male,MCI.vs.control; Homo_714,female,AD.vs.control; Homo_714,male,AD.vs.control; Homo_714,female,MCI.vs.control; Homo_714,male,MCI.vs.control; Homo_633,female,MCI.vs.control; Homo_633,male,MCI.vs.control; ROSMAP,female,AD.vs.control; Homo_723,totalSample,immune-Monocyte; Homo_723,totalSample,immune-DC; Homo_723,totalSample,immune-Exhausted; Homo_723,totalSample,immune-Th2; Homo_723,totalSample,immune-Effector_memory; Homo_714,totalSample,immune-Monocyte; Homo_714,totalSample,immune-Th2; Homo_633,totalSample,immune-Monocyte; Homo_633,totalSample,immune-Th2; Homo_633,totalSample,immune-Th1; ROSMAP,totalSample,immune-Neutrophil; ROSMAP,female-excludeAge90+,survival
HEMGN	Emory,control,male.vs.female; Emory,MCI,age; ROSMAP,MCI,last.visit.age; ROSMAP,MCI,sample.age; Emory,totalSample,immune-Gamma_delta; Emory,totalSample,immune-CD8_naive; Homo_723,totalSample,immune-Bcell; Homo_723,totalSample,immune-Monocyte; Homo_714,totalSample,immune-Bcell; Homo_633,totalSample,immune-Bcell; Homo_633,totalSample,immune-Monocyte; MCSA,totalSample,immune-CD8_naive; MCSA,totalSample,immune-Cytotoxic; MCSA,totalSample,immune-Exhausted; ROSMAP,totalSample,immune-InfiltrationScore
FCRL5	Emory,control,male.vs.female; Homo_723,female,AD.vs.control; Homo_723,male,AD.vs.control; Homo_723,female,AD.vs.MCI; Homo_723,male,AD.vs.MCI; Homo_723,male,MCI.vs.control; Homo_714,female,AD.vs.control; Homo_714,male,AD.vs.control; Homo_714,female,AD.vs.MCI; Homo_714,male,AD.vs.MCI; Homo_714,male,MCI.vs.control; Homo_633,female,AD.vs.control; Homo_633,female,AD.vs.MCI; Homo_633,male,MCI.vs.control; Emory,totalSample,immune-B_cell; Emory,totalSample,immune-Macrophage; Emory,totalSample,immune-InfiltrationScore; Homo_723,totalSample,immune-iTreg; Homo_723,totalSample,immune-Th2; Homo_723,totalSample,immune-Tfh; Homo_723,totalSample,immune-Macrophage; Homo_723,totalSample,immune-CD4_T; Homo_723,totalSample,immune-InfiltrationScore; Homo_723,totalSample,immune-Tr1; Homo_723,totalSample,immune-DC; Homo_723,totalSample,immune-Effector_memory; Homo_723,totalSample,immune-Gamma_delta; Homo_714,totalSample,immune-Th2; Homo_714,totalSample,immune-Tfh; Homo_714,totalSample,immune-CD4_T; Homo_714,totalSample,immune-Tr1; Homo_714,totalSample,immune-nTreg; Homo_714,totalSample,immune-Effector_memory; Homo_714,totalSample,immune-iTreg; Homo_714,totalSample,immune-Macrophage; Homo_714,totalSample,immune-Central_memory; Homo_633,totalSample,immune-Th2; Homo_633,totalSample,immune-Tfh; Homo_633,totalSample,immune-CD4_T; Homo_633,totalSample,immune-nTreg; Homo_633,totalSample,immune-Tr1; Homo_633,totalSample,immune-Central_memory; Homo_633,totalSample,immune-Gamma_delta; MCSA,totalSample,immune-Bcell; MCSA,totalSample,immune-Monocyte; MCSA,totalSample,immune-CD4_T; MCSA,totalSample,immune-Gamma_delta; MCSA,totalSample,immune-CD4_naive; MCSA,totalSample,immune-Tr1; MCSA,totalSample,immune-nTreg; MCSA,totalSample,immune-iTreg; MCSA,totalSample,immune-Th17; MCSA,totalSample,immune-Tfh; MCSA,totalSample,immune-CD8_naive; ROSMAP,totalSample,immune-InfiltrationScore;
TMPRSS9	Emory,control,male.vs.female; Homo_723,female,AD.vs.control; Homo_723,male,AD.vs.control; Homo_723,female,AD.vs.MCI; Homo_723,male,AD.vs.MCI; Homo_723,female,MCI.vs.control; Homo_723,male,MCI.vs.control; Homo_714,female,AD.vs.control; Homo_714,male,AD.vs.control; Homo_714,female,AD.vs.MCI; Homo_714,male,AD.vs.MCI; Homo_714,female,MCI.vs.control; Homo_714,male,MCI.vs.control; Homo_633,female,AD.vs.control; Homo_633,female,AD.vs.MCI; Homo_633,female,MCI.vs.control; Homo_633,male,MCI.vs.control; ROSMAP,male,AD.vs.control; ROSMAP,male,AD.vs.MCI; ROSMAP,AD,male.vs.female; Emory,totalSample,age; Emory,MCI,age; MCSA,MCI,age; ROSMAP,AD,last.visit.age; ROSMAP,MCI,last.visit.age; Emory,totalSample,immune-Neutrophil; Emory,totalSample,immune-Gamma_delta; Emory,totalSample,immune-iTreg; Emory,totalSample,immune-CD8_naive; Emory,totalSample,immune-Cytotoxic; Emory,totalSample,immune-Exhausted; Homo_723,totalSample,immune-Exhausted; Homo_723,totalSample,immune-Monocyte; Homo_723,totalSample,immune-DC; Homo_723,totalSample,immune-CD8_T; Homo_723,totalSample,immune-Cytotoxic; Homo_723,totalSample,immune-Th2; Homo_723,totalSample,immune-Th17; Homo_714,totalSample,immune-DC; Homo_714,totalSample,immune-Monocyte; Homo_714,totalSample,immune-Th2; Homo_714,totalSample,immune-Th17; Homo_633,totalSample,immune-Th2; Homo_633,totalSample,immune-DC; Homo_633,totalSample,immune-Monocyte; Homo_633,totalSample,immune-Th17; MCSA,totalSample,immune-DC; MCSA,totalSample,immune-Monocyte; MCSA,totalSample,immune-Macrophage; MCSA,totalSample,immune-Neutrophil; MCSA,totalSample,immune-CD8_T; MCSA,totalSample,immune-NKT; MCSA,totalSample,immune-iTreg; MCSA,totalSample,immune-Th2; MCSA,totalSample,immune-Cytotoxic; MCSA,totalSample,immune-Effector_memory; SRP223445,totalSample,immune-InfiltrationScore
BMX	Emory,control,male.vs.female; Homo_723,female,AD.vs.control; Homo_723,male,AD.vs.control; Homo_723,female,AD.vs.MCI; Homo_723,male,AD.vs.MCI; Homo_723,MCI,male.vs.female; Homo_714,female,AD.vs.control; Homo_714,male,AD.vs.control; Homo_714,female,AD.vs.MCI; Homo_714,male,AD.vs.MCI; Homo_714,MCI,male.vs.female; Homo_633,female,AD.vs.control; Homo_633,female,AD.vs.MCI; Homo_633,MCI,male.vs.female; ROSMAP,male,AD.vs.control; ROSMAP,control,male.vs.female; Emory,totalSample,age; Emory,MCI,age; MCSA,MCI,age; ROSMAP,MCI,sample.age; Emory,totalSample,immune-B_cell; Emory,totalSample,immune-Monocyte; Emory,totalSample,immune-Macrophage; Emory,totalSample,immune-NK; Emory,totalSample,immune-Neutrophil; Emory,totalSample,immune-CD4_T; Emory,totalSample,immune-CD8_T; Emory,totalSample,immune-Gamma_delta; Emory,totalSample,immune-Tr1; Emory,totalSample,immune-Th1; Emory,totalSample,immune-Tfh; Emory,totalSample,immune-CD8_naive; Emory,totalSample,immune-Cytotoxic; Emory,totalSample,immune-Exhausted; Emory,totalSample,immune-MAIT; Emory,totalSample,immune-InfiltrationScore; Homo_723,totalSample,immune-Monocyte; Homo_723,totalSample,immune-Neutrophil; Homo_723,totalSample,immune-Exhausted; Homo_723,totalSample,immune-DC; Homo_723,totalSample,immune-Gamma_delta; Homo_723,totalSample,immune-CD8_T; Homo_723,totalSample,immune-Th1; Homo_723,totalSample,immune-Bcell; Homo_714,totalSample,immune-Monocyte; Homo_714,totalSample,immune-Neutrophil; Homo_714,totalSample,immune-Exhausted; Homo_714,totalSample,immune-Gamma_delta; Homo_714,totalSample,immune-Th1; Homo_714,totalSample,immune-MAIT; Homo_714,totalSample,immune-CD8_T; Homo_714,totalSample,immune-DC; Homo_633,totalSample,immune-Monocyte; Homo_633,totalSample,immune-Neutrophil; Homo_633,totalSample,immune-Exhausted; Homo_633,totalSample,immune-Th2; Homo_633,totalSample,immune-Gamma_delta; Homo_633,totalSample,immune-CD8_T; Homo_633,totalSample,immune-Th17; Homo_633,totalSample,immune-MAIT; Homo_633,totalSample,immune-Th1; Homo_633,totalSample,immune-Macrophage; Homo_633,totalSample,immune-DC; MCSA,totalSample,immune-Monocyte; MCSA,totalSample,immune-Neutrophil; MCSA,totalSample,immune-CD4_T; MCSA,totalSample,immune-CD8_T; MCSA,totalSample,immune-Gamma_delta; MCSA,totalSample,immune-CD4_naive; MCSA,totalSample,immune-nTreg; MCSA,totalSample,immune-Th1; MCSA,totalSample,immune-Tfh; MCSA,totalSample,immune-Cytotoxic; MCSA,totalSample,immune-Exhausted; MCSA,totalSample,immune-Central_memory; MCSA,totalSample,immune-Effector_memory; MCSA,totalSample,immune-InfiltrationScore; [illegible]
SLC2A4	Emory,control,male.vs.female; Homo_633,female,AD.vs.control; Homo_633,female,AD.vs.MCI; Emory,MCI,age; ROSMAP,AD,sample.age; Emory,totalSample,immune-Monocyte; Emory,totalSample,immune-Gamma_delta; Emory,totalSample,immune-Tr1; Emory,totalSample,immune-iTreg; Homo_723,totalSample,immune-DC; Homo_714,totalSample,immune-DC;
DEFA3	Emory,control,male.vs.female; Homo_723,female,AD.vs.control; Homo_723,male,AD.vs.control; Homo_723,female,AD.vs.MCI; Homo_723,male,AD.vs.MCI; Homo_723,control,male.vs.female; Homo_714,female,AD.vs.control; Homo_714,male,AD.vs.control; Homo_714,female,AD.vs.MCI; Homo_714,male,AD.vs.MCI; Homo_714,control,male.vs.female; Homo_633,female,AD.vs.control; Homo_633,female,AD.vs.MCI; Homo_633,control,male.vs.female; Emory,totalSample,immune-Macrophage; Emory,totalSample,immune-NK; Emory,totalSample,immune-CD4_T; Emory,totalSample,immune-NKT; Emory,totalSample,immune-nTreg; Emory,totalSample,immune-Tfh; Emory,totalSample,immune-InfiltrationScore; Homo_723,totalSample,immune-Th1; Homo_723,totalSample,immune-Monocyte; Homo_723,totalSample,immune-Macrophage; Homo_723,totalSample,immune-Cytotoxic; Homo_714,totalSample,immune-Macrophage; Homo_714,totalSample,immune-Th2; Homo_633,totalSample,immune-Macrophage; Homo_633,totalSample,immune-Th2; MCSA,totalSample,immune-Macrophage; MCSA,totalSample,immune-CD4_T; MCSA,totalSample,immune-NKT; MCSA,totalSample,immune-CD4_naive; MCSA,totalSample,immune-Tr1; MCSA,totalSample,immune-nTreg; MCSA,totalSample,immune-iTreg; MCSA,totalSample,immune-Th2; MCSA,totalSample,immune-Tfh; MCSA,totalSample,immune-Central_memory;
PRR5-ARHGAP8	Emory,control,male.vs.female; Homo_723,female,AD.vs.control; Homo_723,female,MCI.vs.control; Homo_723,male,MCI.vs.control; Homo_714,female,AD.vs.control; Homo_714,female,MCI.vs.control; Homo_714,male,MCI.vs.control; Homo_633,female,AD.vs.control; Homo_633,female,AD.vs.MCI; Homo_633,female,MCI.vs.control; Homo_633,male,MCI.vs.control; Homo_633,control,male.vs.female; ROSMAP,female,AD.vs.control; ROSMAP,male,AD.vs.control; ROSMAP,female,AD.vs.MCI; ROSMAP,female,MCI.vs.control; MCSA,MCI,age; ROSMAP,AD,last.visit.age; Emory,totalSample,immune-Gamma_delta; Emory,totalSample,immune-Th17; Emory,totalSample,immune-Cytotoxic; Homo_723,totalSample,immune-DC; Homo_723,totalSample,immune-Monocyte; Homo_723,totalSample,immune-Neutrophil; Homo_723,totalSample,immune-Exhausted; Homo_714,totalSample,immune-DC; Homo_714,totalSample,immune-Th2; Homo_714,totalSample,immune-InfiltrationScore; Homo_633,totalSample,immune-DC; Homo_633,totalSample,immune-Th2; Homo_633,totalSample,immune-Monocyte; Homo_633,totalSample,immune-InfiltrationScore; MCSA,totalSample,immune-DC; MCSA,totalSample,immune-Monocyte; MCSA,totalSample,immune-Neutrophil; MCSA,totalSample,immune-CD8_T; MCSA,totalSample,immune-Th2; MCSA,totalSample,immune-Cytotoxic; MCSA,totalSample,immune-Effector_memory; MCSA,totalSample,immune-[illegible]
RPL21	Emory,control,male.vs.female; ROSMAP,female,AD.vs.control; Emory,totalSample,age; Emory,MCI,age; MCSA,totalSample,age; MCSA,control,age; MCSA,MCI,age; Emory,totalSample,immune-DC; Emory,totalSample,immune-Monocyte; Emory,totalSample,immune-Macrophage; Emory,totalSample,immune-Neutrophil; Emory,totalSample,immune-CD4_T; Emory,totalSample,immune-CD8_T; Emory,totalSample,immune-Gamma_delta; Emory,totalSample,immune-Tr1; Emory,totalSample,immune-Th1; Emory,totalSample,immune-Th17; Emory,totalSample,immune-Tfh; Emory,totalSample,immune-CD8_naive; Emory,totalSample,immune-Cytotoxic; Emory,totalSample,immune-Exhausted; Homo_723,totalSample,immune-Neutrophil; Homo_723,totalSample,immune-Bcell; Homo_714,totalSample,immune-Neutrophil; Homo_714,totalSample,immune-Monocyte; Homo_633,totalSample,immune-Neutrophil; Homo_633,totalSample,immune-Monocyte; Homo_633,totalSample,immune-Cytotoxic; Homo_633,totalSample,immune-MAIT; MCSA,totalSample,immune-DC; MCSA,totalSample,immune-Monocyte; MCSA,totalSample,immune-Macrophage; MCSA,totalSample,immune-Gamma_delta; MCSA,totalSample,immune-nTreg; MCSA,totalSample,immune-Th1; MCSA,totalSample,immune-Th2; MCSA,totalSample,immune-Th17; MCSA,totalSample,immune-Exhausted; ROSMAP,totalSample,immune-Monocyte; SRP223445,totalSample,immune-NK; SRP223445,totalSample,immune-Neutrophil; SRP223445,totalSample,immune-CD4_naive; SRP223445,totalSample,immune-Th17; SRP223445,totalSample,immune-Exhausted; SRP223445,totalSample,immune-InfiltrationScore
OR2T8	Emory,control,male.vs.female; ROSMAP,MCI,last.visit.age; Homo_723,totalSample,immune-Bcell; Homo_714,totalSample,immune-Bcell; Homo_633,totalSample,immune-Bcell; ROSMAP,totalSample,immune-InfiltrationScore
SHISA4	Emory,control,male.vs.female; Homo_723,male,AD.vs.control; Homo_714,male,AD.vs.control; ROSMAP,female,AD.vs.control; Emory,MCI,age; MCSA,MCI,age; Emory,totalSample,immune-Monocyte; Emory,totalSample,immune-Neutrophil; Emory,totalSample,immune-CD8_T; Emory,totalSample,immune-CD8_naive; Homo_723,totalSample,immune-DC; Homo_714,totalSample,immune-DC; Homo_633,totalSample,immune-DC; MCSA,totalSample,immune-DC; MCSA,totalSample,immune-Monocyte; MCSA,totalSample,immune-Macrophage; MCSA,totalSample,immune-CD8_T; MCSA,totalSample,immune-iTreg; MCSA,totalSample,immune-Th2; MCSA,totalSample,immune-Cytotoxic
LOC101929601	Emory,control,male.vs.female; Homo_723,female,AD.vs.control; Homo_723,male,AD.vs.control; Homo_723,female,MCI.vs.control; Homo_723,male,MCI.vs.control; Homo_714,female,AD.vs.control; Homo_714,male,AD.vs.control; Homo_714,female,MCI.vs.control; Homo_714,male,MCI.vs.control; Homo_633,female,MCI.vs.control; Homo_633,male,MCI.vs.control; Emory,totalSample,immune-Neutrophil; Emory,totalSample,immune-CD8_T; Emory,totalSample,immune-Gamma_delta; Emory,totalSample,immune-Tfh; Homo_723,totalSample,immune-Monocyte; Homo_723,totalSample,immune-DC; Homo_723,totalSample,immune-Th2; Homo_714,totalSample,immune-Th2; Homo_714,totalSample,immune-Monocyte;
PPFIBP1	Emory,control,male.vs.female; Homo_723,female,AD.vs.control; Homo_723,male,AD.vs.control; Homo_723,female,AD.vs.MCI; Homo_723,male,AD.vs.MCI; Homo_723,female,MCI.vs.control; Homo_723,male,MCI.vs.control; Homo_723,control,male.vs.female; Homo_714,female,AD.vs.control; Homo_714,male,AD.vs.control; Homo_714,female,AD.vs.MCI; Homo_714,male,AD.vs.MCI; Homo_714,female,MCI.vs.control; Homo_714,male,MCI.vs.control; Homo_714,control,male.vs.female; Homo_633,female,AD.vs.control; Homo_633,female,AD.vs.MCI; ROSMAP,AD,male.vs.female; ACOM,AD,age; MCSA,totalSample,age; MCSA,control,age; ROSMAP,MCI,last.visit.age; ROSMAP,MCI,sample.age; Emory,totalSample,immune-Macrophage; Emory,totalSample,immune-CD4_T; Emory,totalSample,immune-Tr1; Emory,totalSample,immune-nTreg; Emory,totalSample,immune-iTreg; Emory,totalSample,immune-Central_memory; Emory,totalSample,immune-InfiltrationScore; Homo_723,totalSample,immune-CD4_naive; Homo_723,totalSample,immune-Central_memory; Homo_723,totalSample,immune-CD4_T; Homo_723,totalSample,immune-Th17; Homo_723,totalSample,immune-nTreg; Homo_723,totalSample,immune-Tr1; Homo_723,totalSample,immune-Cytotoxic; Homo_723,totalSample,immune-Th1; Homo_723,totalSample,immune-Tfh; Homo_723,totalSample,immune-iTreg; Homo_723,totalSample,immune-InfiltrationScore; Homo_723,totalSample,immune-Exhausted; Homo_723,totalSample,immune-Neutrophil; Homo_723,totalSample,immune-Th2; Homo_714,totalSample,immune-CD4_naive; Homo_714,totalSample,immune-CD4_T; Homo_714,totalSample,immune-Th1; Homo_714,totalSample,immune-Th17; Homo_714,totalSample,immune-nTreg; Homo_714,totalSample,immune-Central_memory; Homo_714,totalSample,immune-Tfh; Homo_714,totalSample,immune-iTreg; Homo_714,totalSample,immune-Tr1; Homo_714,totalSample,immune-NKT; Homo_714,totalSample,immune-InfiltrationScore; Homo_714,totalSample,immune-Exhausted; Homo_714,totalSample,immune-Cytotoxic; Homo_633,totalSample,immune-Tfh; Homo_633,totalSample,immune-CD4_T; Homo_633,totalSample,immune-Tr1; Homo_633,totalSample,immune-CD4_naive; Homo_633,totalSample,immune-iTreg; Homo_633,totalSample,immune-nTreg; Homo_633,totalSample,immune-Central_memory; Homo_633,totalSample,immune-NKT; Homo_633,totalSample,immune-Effector_memory; Homo_633,totalSample,immune-Th2; Homo_633,totalSample,immune-InfiltrationScore; Homo_633,totalSample,immune-Th1; Homo_633,totalSample,immune-Gamma_delta; Homo_633,totalSample,immune-Th17; Homo_633,totalSample,immune-Exhausted; MCSA,totalSample,immune-DC; MCSA,totalSample,immune-Bcell; MCSA,totalSample,immune-Macrophage; MCSA,totalSample,immune-CD4_T; MCSA,totalSample,immune-Gamma_delta; MCSA,totalSample,immune-CD4_naive; MCSA,totalSample,immune-Tr1; MCSA,totalSample,immune-nTreg; MCSA,totalSample,immune-iTreg; MCSA,totalSample,immune-Th17; MCSA,totalSample,immune-Tfh; MCSA,totalSample,immune-CD8_naive; MCSA,totalSample,immune-Cytotoxic; [illegible]
MYZAP	Emory,control,male.vs.female; Homo_723,female,AD.vs.control; Homo_723,male,AD.vs.control; Homo_723,female,AD.vs.MCI; Homo_723,male,AD.vs.MCI; Homo_723,female,MCI.vs.control; Homo_723,male,MCI.vs.control; Homo_714,female,AD.vs.control; Homo_714,male,AD.vs.control; Homo_714,female,AD.vs.MCI; Homo_714,male,AD.vs.MCI; Homo_714,female,MCI.vs.control; Homo_714,male,MCI.vs.control; Homo_633,female,AD.vs.control; Homo_633,female,AD.vs.MCI; Homo_633,female,MCI.vs.control; ROSMAP,female,AD.vs.control; ROSMAP,AD,last.visit.age; ROSMAP,MCI,last.visit.age; Homo_723,totalSample,immune-Exhausted; Homo_723,totalSample,immune-Monocyte; Homo_723,totalSample,immune-Th17; Homo_723,totalSample,immune-CD8_T; Homo_723,totalSample,immune-CD8_naive; Homo_714,totalSample,immune-Monocyte; Homo_714,totalSample,immune-Th17; Homo_714,totalSample,immune-Exhausted; Homo_633,totalSample,immune-Th2; Homo_633,totalSample,immune-Monocyte; Homo_633,totalSample,immune-Tfh; Homo_633,totalSample,immune-Gamma_delta; ROSMAP,totalSample,immune-Th17; ROSMAP,totalSample,immune-InfiltrationScore; SRP223445,totalSample,immune-CD4_naive; SRP223445,totalSample,immune-Exhausted

MPO	Emory,control,male.vs.female; Homo_723,female,AD.vs.control; Homo_723,male,AD.vs.control; Homo_723,female,AD.vs.MCI; Homo_723,male,AD.vs.MCI; Homo_723,control,male.vs.female; Homo_714,female,AD.vs.control; Homo_714,male,AD.vs.control; Homo_714,female,AD.vs.MCI; Homo_714,male,AD.vs.MCI; Homo_714,female,MCI.vs.control; Homo_714,control,male.vs.female; Homo_633,female,AD.vs.MCI; Homo_633,female,MCI.vs.control; Homo_633,control,male.vs.female; Emory,totalSample,immune-B_cell; Emory,totalSample,immune-Macrophage; Emory,totalSample,immune-NK; Emory,totalSample,immune-CD8_T; Emory,totalSample,immune-Gamma_delta; Emory,totalSample,immune-Th1; Emory,totalSample,immune-Tfh; Emory,totalSample,immune-Exhausted; Homo_723,totalSample,immune-Macrophage; Homo_723,totalSample,immune-InfiltrationScore; Homo_723,totalSample,immune-Th2; Homo_714,totalSample,immune-Macrophage; Homo_714,totalSample,immune-Th2; MCSA,totalSample,immune-DC; MCSA,totalSample,immune-Macrophage; MCSA,totalSample,immune-NKT; MCSA,totalSample,immune-Gamma_delta; MCSA,totalSample,immune-Th2; SRP223445,totalSample,immune-CD4_T; SRP223445,totalSample,immune-CD4_naive; SRP223445,totalSample,immune-Th17; SRP223445,totalSample,immune-InfiltrationScore
CORO7-PAM16	Emory,MCI,male.vs.female; Homo_723,female,AD.vs.control; Homo_723,male,AD.vs.MCI; Homo_723,female,MCI.vs.control; Homo_723,male,MCI.vs.control; Homo_723,control,male.vs.female; Homo_723,MCI,male.vs.female; Homo_714,female,AD.vs.control; Homo_714,male,AD.vs.MCI; Homo_714,female,MCI.vs.control; Homo_714,male,MCI.vs.control; Homo_714,control,male.vs.female; Homo_714,MCI,male.vs.female; Homo_633,male,MCI.vs.control; Homo_633,control,male.vs.female; Homo_633,MCI,male.vs.female; ROSMAP,male,AD.vs.control; ROSMAP,MCI,sample.age; Homo_723,totalSample,immune-Monocyte; Homo_723,totalSample,immune-DC; Homo_633,totalSample,immune-Monocyte
RNASE1	Emory,MCI,male.vs.female; Homo_723,female,AD.vs.MCI; Homo_723,MCI,male.vs.female; Homo_714,female,AD.vs.MCI; Homo_714,MCI,male.vs.female; Homo_633,MCI,male.vs.female; ROSMAP,control,male.vs.female; MCSA,MCI,age; MCSA,totalSample,immune-DC; MCSA,totalSample,immune-Macrophage; ROSMAP,totalSample,immune-CD4_T; ROSMAP,totalSample,immune-Tr1; ROSMAP,totalSample,immune-nTreg; ROSMAP,totalSample,immune-iTreg; ROSMAP,totalSample,immune-Th2; ROSMAP,totalSample,immune-Th17; ROSMAP,totalSample,immune-Central_memory; ROSMAP,totalSample,immune-InfiltrationScore; SRP223445,totalSample,immune-Neutrophil; SRP223445,totalSample,immune-
LOC112267855	Homo_723,female,AD.vs.control; Homo_723,male,AD.vs.control; Homo_723,female,AD.vs.MCI; Homo_723,male,AD.vs.MCI; Homo_714,female,AD.vs.control; Homo_714,male,AD.vs.control; Homo_714,female,AD.vs.MCI; Homo_714,male,AD.vs.MCI; Homo_714,control,male.vs.female; Homo_633,female,AD.vs.control; Homo_633,female,AD.vs.MCI; Homo_633,control,male.vs.female; Emory,MCI,age; MCSA,MCI,age; Emory,totalSample,immune-Monocyte; Emory,totalSample,immune-Macrophage; Emory,totalSample,immune-Neutrophil; Emory,totalSample,immune-CD4_T; Emory,totalSample,immune-CD8_T; Emory,totalSample,immune-Gamma_delta; Emory,totalSample,immune-Tfh; Emory,totalSample,immune-CD8_naive; Emory,totalSample,immune-Exhausted; Emory,totalSample,immune-MAIT; Homo_723,totalSample,immune-Neutrophil; Homo_723,totalSample,immune-Th1; Homo_723,totalSample,immune-Monocyte; Homo_723,totalSample,immune-Cytotoxic; Homo_723,totalSample,immune-Gamma_delta; Homo_714,totalSample,immune-Th1; Homo_714,totalSample,immune-CD4_naive; Homo_714,totalSample,immune-Neutrophil; Homo_714,totalSample,immune-CD4_T; Homo_714,totalSample,immune-NKT; Homo_714,totalSample,immune-Central_memory; Homo_633,totalSample,immune-CD4_naive; Homo_633,totalSample,immune-CD4_T; Homo_633,totalSample,immune-Neutrophil; Homo_633,totalSample,immune-Tfh; Homo_633,totalSample,immune-Central_memory; Homo_633,totalSample,immune-Th1; Homo_633,totalSample,immune-NKT; Homo_633,totalSample,immune-iTreg; Homo_633,totalSample,immune-Tr1; Homo_633,totalSample,immune-MAIT; Homo_633,totalSample,immune-Effector_memory; Homo_633,totalSample,immune-Macrophage; MCSA,totalSample,immune-DC; MCSA,totalSample,immune-Monocyte; MCSA,totalSample,immune-Macrophage; MCSA,totalSample,immune-Neutrophil; MCSA,totalSample,immune-CD4_T; MCSA,totalSample,immune-CD4_naive; MCSA,totalSample,immune-Tr1; MCSA,totalSample,immune-nTreg; MCSA,totalSample,immune-iTreg;
TPBG	Homo_723,female,AD.vs.control; Homo_723,male,AD.vs.control; Homo_723,female,AD.vs.MCI; Homo_723,male,AD.vs.MCI; Homo_714,male,AD.vs.control; Homo_714,male,AD.vs.MCI; Homo_633,female,AD.vs.control; Homo_633,female,AD.vs.MCI; Homo_633,male,MCI.vs.control; ROSMAP,female,AD.vs.control; ROSMAP,control,male.vs.female; MCSA,totalSample,age; MCSA,control,age; Emory,totalSample,immune-Gamma_delta; Emory,totalSample,immune-Tr1; Emory,totalSample,immune-iTreg; Emory,totalSample,immune-Th1; Emory,totalSample,immune-Th17; Homo_714,totalSample,immune-InfiltrationScore; Homo_633,totalSample,immune-InfiltrationScore; Homo_633,totalSample,immune-NKT; MCSA,totalSample,immune-CD4_T; MCSA,totalSample,immune-CD4_naive; MCSA,totalSample,immune-Tr1; MCSA,totalSample,immune-nTreg; MCSA,totalSample,immune-iTreg; MCSA,totalSample,immune-Th17; MCSA,totalSample,immune-Tfh; ROSMAP,totalSample,immune-InfiltrationScore; ROSMAP,male-includeAge90+,survival
CD300H	Homo_723,female,AD.vs.control; Homo_723,male,AD.vs.control; Homo_723,female,AD.vs.MCI; Homo_723,male,AD.vs.MCI; Homo_714,female,AD.vs.control; Homo_714,male,AD.vs.control; Homo_714,female,AD.vs.MCI; Homo_714,male,AD.vs.MCI; Homo_633,female,AD.vs.control; Homo_633,female,AD.vs.MCI; Emory,totalSample,immune-NK; Emory,totalSample,immune-Neutrophil; Emory,totalSample,immune-InfiltrationScore; Homo_723,totalSample,immune-Cytotoxic; Homo_723,totalSample,immune-InfiltrationScore; Homo_723,totalSample,immune-Th17; Homo_723,totalSample,immune-CD8_T; Homo_633,totalSample,immune-DC; Homo_633,totalSample,immune-NK; MCSA,totalSample,immune-DC
NNAT	Homo_723,female,AD.vs.control; Homo_723,female,AD.vs.MCI; ROSMAP,female,AD.vs.control; ROSMAP,male,MCI.vs.control; Homo_723,totalSample,immune-CD4_T; Homo_723,totalSample,immune-iTreg; Homo_723,totalSample,immune-nTreg; Homo_723,totalSample,immune-Th2; Homo_723,totalSample,immune-CD4_naive; Homo_723,totalSample,immune-Tfh; Homo_723,totalSample,immune-Central_memory; Homo_723,totalSample,immune-Tr1; Homo_723,totalSample,immune-Effector_memory; Homo_714,totalSample,immune-Effector_memory; Homo_714,totalSample,immune-Tfh; Homo_714,totalSample,immune-Central_memory; Homo_714,totalSample,immune-nTreg; Homo_714,totalSample,immune-CD4_T; Homo_714,totalSample,immune-Th2; Homo_714,totalSample,immune-CD4_naive; Homo_714,totalSample,immune-iTreg; Homo_714,totalSample,immune-Tr1; Homo_633,totalSample,immune-Central_memory; Homo_633,totalSample,immune-Effector_memory; Homo_633,totalSample,immune-Tfh; Homo_633,totalSample,immune-nTreg; Homo_633,totalSample,immune-CD4_T; Homo_633,totalSample,immune-CD4_naive; Homo_633,totalSample,immune-Tr1; Homo_633,totalSample,immune-iTreg; Homo_633,totalSample,immune-InfiltrationScore; MCSA,totalSample,immune-Neutrophil; MCSA,totalSample,immune-CD4_T; MCSA,totalSample,immune-NKT; MCSA,totalSample,immune-Gamma_delta; MCSA,totalSample,immune-CD4_naive; MCSA,totalSample,immune-Tr1; MCSA,totalSample,immune-nTreg; MCSA,totalSample,immune-iTreg; MCSA,totalSample,immune-Tfh; MCSA,totalSample,immune-CD8_naive; MCSA,totalSample,immune-Exhausted; MCSA,totalSample,immune-MAIT; MCSA,totalSample,immune-Central_memory; MCSA,totalSample,immune-Effector_memory
MILR1	Homo_723,female,AD.vs.control; Homo_723,male,AD.vs.control; Homo_723,female,AD.vs.MCI; Homo_723,male,AD.vs.MCI; Homo_714,female,AD.vs.control; Homo_714,male,AD.vs.control; Homo_714,female,AD.vs.MCI; Homo_714,male,AD.vs.MCI; Homo_633,female,AD.vs.control; Homo_633,female,AD.vs.MCI; ROSMAP,male,AD.vs.MCI; Emory,totalSample,immune-Gamma_delta; Homo_723,totalSample,immune-Th2; Homo_723,totalSample,immune-Monocyte; Homo_723,totalSample,immune-Gamma_delta; Homo_723,totalSample,immune-Tfh; Homo_723,totalSample,immune-DC; Homo_723,totalSample,immune-InfiltrationScore; Homo_723,totalSample,immune-iTreg; Homo_723,totalSample,immune-Neutrophil; Homo_723,totalSample,immune-Effector_memory; Homo_723,totalSample,immune-Bcell; Homo_714,totalSample,immune-Th2; Homo_714,totalSample,immune-Tfh; Homo_714,totalSample,immune-Gamma_delta; Homo_714,totalSample,immune-Monocyte; Homo_633,totalSample,immune-Th2; Homo_633,totalSample,immune-Monocyte; Homo_633,totalSample,immune-Exhausted; Homo_633,totalSample,immune-Gamma_delta; Homo_633,totalSample,immune-Th17; Homo_633,totalSample,immune-Tfh; Homo_633,totalSample,immune-InfiltrationScore; MCSA,totalSample,immune-CD8_T; MCSA,totalSample,immune-Gamma_delta; MCSA,totalSample,immune-iTreg; MCSA,totalSample,immune-Th17; MCSA,totalSample,immune-Cytotoxic; MCSA,totalSample,immune-Exhausted; ROSMAP,totalSample,immune-Effector_memory; SRP223445,totalSample,immune-NK
C3orf86	Homo_723,female,AD.vs.control; Homo_723,male,AD.vs.control; Homo_723,female,AD.vs.MCI; Homo_723,male,AD.vs.MCI; Homo_723,female,MCI.vs.control; Homo_714,female,AD.vs.control; Homo_714,male,AD.vs.control; Homo_714,female,AD.vs.MCI; Homo_714,male,AD.vs.MCI; Homo_714,female,MCI.vs.control; Homo_633,female,AD.vs.control; Homo_633,female,AD.vs.MCI; Emory,totalSample,age; Emory,MCI,age; MCSA,MCI,age; Emory,totalSample,immune-Macrophage; Emory,totalSample,immune-NK; Emory,totalSample,immune-Neutrophil; Emory,totalSample,immune-CD4_T; Emory,totalSample,immune-CD8_T; Emory,totalSample,immune-Gamma_delta; Emory,totalSample,immune-Tr1; Emory,totalSample,immune-iTreg; Emory,totalSample,immune-Th1; Emory,totalSample,immune-Tfh; Emory,totalSample,immune-CD8_naive; Emory,totalSample,immune-Cytotoxic; Emory,totalSample,immune-Exhausted; Emory,totalSample,immune-MAIT; Emory,totalSample,immune-Effector_memory; Emory,totalSample,immune-InfiltrationScore; Homo_723,totalSample,immune-Neutrophil; Homo_723,totalSample,immune-Th1; Homo_723,totalSample,immune-Gamma_delta; Homo_723,totalSample,immune-CD8_naive; Homo_714,totalSample,immune-Neutrophil; Homo_714,totalSample,immune-Th1; Homo_714,totalSample,immune-CD4_naive; Homo_633,totalSample,immune-Neutrophil; Homo_633,totalSample,immune-MAIT; Homo_633,totalSample,immune-CD4_naive; Homo_633,totalSample,immune-CD8_T; Homo_633,totalSample,immune-Effector_memory; Homo_633,totalSample,immune-CD4_T; Homo_633,totalSample,immune-Central_memory; Homo_633,totalSample,immune-Tfh; Homo_633,totalSample,immune-Th1; Homo_633,totalSample,immune-Exhausted; MCSA,totalSample,immune-DC; MCSA,totalSample,immune-Bcell; MCSA,totalSample,immune-Monocyte; MCSA,totalSample,immune-Macrophage; MCSA,totalSample,immune-Neutrophil; MCSA,totalSample,immune-CD4_T; MCSA,totalSample,immune-Gamma_delta; MCSA,totalSample,immune-CD4_naive; MCSA,totalSample,immune-Tr1; MCSA,totalSample,immune-nTreg; MCSA,totalSample,immune-iTreg; MCSA,totalSample,immune-Th1; MCSA,totalSample,immune-Th2; MCSA,totalSample,immune-Tfh; MCSA,totalSample,immune-CD8_naive; MCSA,totalSample,immune-MAIT; MCSA,totalSample,immune-Central_memory
TLR1	Homo_723,female,AD.vs.control; Homo_723,male,AD.vs.control; Homo_723,female,AD.vs.MCI; Homo_723,male,AD.vs.MCI; Homo_714,female,AD.vs.control; Homo_714,male,AD.vs.control; Homo_714,female,AD.vs.MCI; Homo_714,male,AD.vs.MCI; Homo_633,female,AD.vs.control; Homo_633,female,AD.vs.MCI; ROSMAP,male,AD.vs.MCI; MCSA,MCI,age; Emory,totalSample,immune-DC; Emory,totalSample,immune-Monocyte; Emory,totalSample,immune-NK; Emory,totalSample,immune-Neutrophil; Emory,totalSample,immune-CD8_T; Emory,totalSample,immune-Gamma_delta; Emory,totalSample,immune-nTreg; Emory,totalSample,immune-Th1; Emory,totalSample,immune-Th17; Emory,totalSample,immune-Tfh; Emory,totalSample,immune-CD8_naive; Emory,totalSample,immune-Cytotoxic; Emory,totalSample,immune-Exhausted; Homo_723,totalSample,immune-Neutrophil; Homo_723,totalSample,immune-Gamma_delta; Homo_723,totalSample,immune-DC; Homo_723,totalSample,immune-Th2; Homo_723,totalSample,immune-Monocyte; Homo_723,totalSample,immune-iTreg; Homo_723,totalSample,immune-Tfh; Homo_723,totalSample,immune-Exhausted; Homo_723,totalSample,immune-Bcell; Homo_723,totalSample,immune-Tr1; Homo_714,totalSample,immune-Th2; Homo_714,totalSample,immune-Gamma_delta; Homo_714,totalSample,immune-Neutrophil; Homo_714,totalSample,immune-DC; Homo_714,totalSample,immune-Monocyte; Homo_714,totalSample,immune-Tfh; Homo_714,totalSample,immune-Exhausted; Homo_714,totalSample,immune-Tr1; Homo_633,totalSample,immune-Exhausted; Homo_633,totalSample,immune-Gamma_delta; Homo_633,totalSample,immune-Th2; Homo_633,totalSample,immune-Monocyte; Homo_633,totalSample,immune-CD8_T; Homo_633,totalSample,immune-Th17; Homo_633,totalSample,immune-Neutrophil; Homo_633,totalSample,immune-DC; Homo_633,totalSample,immune-Cytotoxic; Homo_633,totalSample,immune-NKT; Homo_633,totalSample,immune-Tfh; Homo_633,totalSample,immune-CD4_T; MCSA,totalSample,immune-DC; MCSA,totalSample,immune-Monocyte; MCSA,totalSample,immune-Neutrophil; MCSA,totalSample,immune-CD8_T; MCSA,totalSample,immune-Gamma_delta; MCSA,totalSample,immune-Tr1; MCSA,totalSample,immune-iTreg; MCSA,totalSample,immune-Th1; MCSA,totalSample,immune-Th17; MCSA,totalSample,immune-Cytotoxic; MCSA,totalSample,immune-Exhausted; MCSA,totalSample,immune-Effector_memory; MCSA,totalSample,immune-InfiltrationScore; ROSMAP,totalSample,immune-Neutrophil; ROSMAP,totalSample,immune-Central_memory; ROSMAP,totalSample,immune-Effector_memory; SRP223445,totalSample,immune-CD4_T; SRP223445,totalSample,immune-CD4_naive; SRP223445,totalSample,immune-Exhausted
C5orf63	Homo_723,female,AD.vs.control; Homo_723,male,AD.vs.control; Homo_723,female,AD.vs.MCI; Homo_723,male,AD.vs.MCI; Homo_714,female,AD.vs.control; Homo_714,male,AD.vs.control; Homo_714,female,AD.vs.MCI; Homo_714,male,AD.vs.MCI; Homo_633,female,AD.vs.control; Homo_633,female,AD.vs.MCI; ROSMAP,male,AD.vs.control; ROSMAP,AD,male.vs.female; MCSA,control,age; ROSMAP,totalSample,age; ROSMAP,MCI,last.visit.age; ROSMAP,MCI,sample.age; ROSMAP,AD,MMSE; Emory,totalSample,immune-Macrophage; Emory,totalSample,immune-Neutrophil; Emory,totalSample,immune-CD4_T; Emory,totalSample,immune-CD8_T; Emory,totalSample,immune-Tr1; Emory,totalSample,immune-nTreg; Emory,totalSample,immune-iTreg; Emory,totalSample,immune-Tfh; Emory,totalSample,immune-CD8_naive; Emory,totalSample,immune-Exhausted; Emory,totalSample,immune-MAIT; Emory,totalSample,immune-Central_memory; Emory,totalSample,immune-InfiltrationScore; Homo_723,totalSample,immune-iTreg; Homo_723,totalSample,immune-Tfh; Homo_723,totalSample,immune-CD4_T; Homo_723,totalSample,immune-Tr1; Homo_723,totalSample,immune-Th2; Homo_723,totalSample,immune-CD4_naive; Homo_723,totalSample,immune-Central_memory; Homo_723,totalSample,immune-nTreg; Homo_723,totalSample,immune-Macrophage; Homo_723,totalSample,immune-CD8_T; Homo_723,totalSample,immune-InfiltrationScore; Homo_723,totalSample,immune-DC; Homo_723,totalSample,immune-MAIT; Homo_723,totalSample,immune-Exhausted; Homo_723,totalSample,immune-Effector_memory; Homo_723,totalSample,immune-Gamma_delta; Homo_723,totalSample,immune-Th1; Homo_723,totalSample,immune-CD8_naive; Homo_714,totalSample,immune-Tfh; Homo_714,totalSample,immune-CD4_T; Homo_714,totalSample,immune-Th2; Homo_714,totalSample,immune-Tr1; Homo_714,totalSample,immune-iTreg; Homo_714,totalSample,immune-CD4_naive; Homo_714,totalSample,immune-Central_memory; Homo_714,totalSample,immune-nTreg; Homo_714,totalSample,immune-Macrophage; Homo_714,totalSample,immune-Effector_memory; Homo_714,totalSample,immune-Th1; Homo_714,totalSample,immune-MAIT; Homo_714,totalSample,immune-InfiltrationScore; Homo_714,totalSample,immune-CD8_T; Homo_633,totalSample,immune-Tfh; Homo_633,totalSample,immune-CD4_T; Homo_633,totalSample,immune-CD4_naive; Homo_633,totalSample,immune-Tr1; Homo_633,totalSample,immune-Central_memory; Homo_633,totalSample,immune-iTreg; Homo_633,totalSample,immune-nTreg; Homo_633,totalSample,immune-Th1; Homo_633,totalSample,immune-Effector_memory; Homo_633,totalSample,immune-NKT; Homo_633,totalSample,immune-Th2; Homo_633,totalSample,immune-InfiltrationScore; Homo_633,totalSample,immune-Neutrophil; Homo_633,totalSample,immune-Macrophage; Homo_633,totalSample,immune-MAIT; Homo_633,totalSample,immune-Th17; MCSA,totalSample,immune-DC; MCSA,totalSample,immune-Bcell; MCSA,totalSample,immune-Macrophage; MCSA,totalSample,immune-Neutrophil; MCSA,totalSample,immune-CD4_T; MCSA,totalSample,immune-CD4_naive; MCSA,totalSample,immune-Tr1; MCSA,totalSample,immune-nTreg; MCSA,totalSample,immune-iTreg; MCSA,totalSample,immune-Th2; MCSA,totalSample,immune-Th17; MCSA,totalSample,immune-Tfh; MCSA,totalSample,immune-CD8_naive; MCSA,totalSample,immune-MAIT; MCSA,totalSample,immune-Central_memory; [illegible]
FOSB	Homo_723,female,AD.vs.control; Homo_723,male,AD.vs.control; Homo_723,female,AD.vs.MCI; Homo_723,male,AD.vs.MCI; Homo_714,female,AD.vs.control; Homo_714,male,AD.vs.control; Homo_714,female,AD.vs.MCI; Homo_714,male,AD.vs.MCI; Homo_633,female,AD.vs.control; Homo_633,female,AD.vs.MCI; Homo_633,female,MCI.vs.control; Homo_633,male,MCI.vs.control; ROSMAP,female,AD.vs.control; ROSMAP,male,AD.vs.control; SRP223445,female,AD.vs.control; ROSMAP,AD,last.visit.age; Emory,totalSample,immune-CD4_naive; Homo_723,totalSample,immune-DC; Homo_723,totalSample,immune-Monocyte; Homo_723,totalSample,immune-Neutrophil; Homo_714,totalSample,immune-DC; Homo_714,totalSample,immune-Th2; Homo_714,totalSample,immune-InfiltrationScore; Homo_633,totalSample,immune-Monocyte; Homo_633,totalSample,immune-DC; Homo_633,totalSample,immune-InfiltrationScore; MCSA,totalSample,immune-DC; MCSA,totalSample,immune-Monocyte; MCSA,totalSample,immune-Cytotoxic; MCSA,totalSample,immune-
CCR3	Homo_723,female,AD.vs.control; Homo_723,male,AD.vs.control; Homo_723,female,AD.vs.MCI; Homo_723,male,AD.vs.MCI; ROSMAP,female,AD.vs.control; ROSMAP,female,MCI.vs.control; ROSMAP,control,male.vs.female; Emory,totalSample,age; Emory,MCI,age; Emory,totalSample,immune-Neutrophil; Emory,totalSample,immune-Gamma_delta; Emory,totalSample,immune-CD8_naive; Emory,totalSample,immune-Exhausted; Homo_723,totalSample,immune-Th2; Homo_723,totalSample,immune-Gamma_delta; Homo_723,totalSample,immune-Neutrophil; Homo_723,totalSample,immune-Bcell; Homo_723,totalSample,immune-iTreg; Homo_723,totalSample,immune-InfiltrationScore; Homo_723,totalSample,immune-DC; Homo_714,totalSample,immune-iTreg; Homo_633,totalSample,immune-iTreg; MCSA,totalSample,immune-Gamma_delta; MCSA,totalSample,immune-iTreg; MCSA,totalSample,immune-Th17; MCSA,totalSample,immune-Exhausted; ROSMAP,totalSample,immune-InfiltrationScore
HLA-DMB	Homo_723,female,AD.vs.control; Homo_723,male,AD.vs.control; Homo_723,female,AD.vs.MCI; Homo_723,male,AD.vs.MCI; Homo_714,female,AD.vs.control; Homo_714,male,AD.vs.control; Homo_714,female,AD.vs.MCI; Homo_714,male,AD.vs.MCI; Homo_633,female,AD.vs.control; Homo_633,female,AD.vs.MCI; Homo_633,female,MCI.vs.control; Homo_633,male,MCI.vs.control; ROSMAP,female,AD.vs.control; ROSMAP,female,MCI.vs.control; ACOM,totalSample,age; MCSA,MCI,age; Emory,totalSample,immune-B_cell; Emory,totalSample,immune-Effector_memory; Emory,totalSample,immune-InfiltrationScore; Homo_723,totalSample,immune-Th2; Homo_723,totalSample,immune-Monocyte; Homo_723,totalSample,immune-Effector_memory; Homo_723,totalSample,immune-InfiltrationScore; Homo_723,totalSample,immune-Tfh; Homo_723,totalSample,immune-iTreg; Homo_714,totalSample,immune-Th2; Homo_714,totalSample,immune-Tfh; Homo_714,totalSample,immune-Effector_memory; Homo_714,totalSample,immune-Monocyte; Homo_633,totalSample,immune-Th2; Homo_633,totalSample,immune-Monocyte; Homo_633,totalSample,immune-Exhausted; Homo_633,totalSample,immune-Effector_memory; Homo_633,totalSample,immune-InfiltrationScore; Homo_633,totalSample,immune-Tfh; Homo_633,totalSample,immune-Gamma_delta; MCSA,totalSample,immune-DC; MCSA,totalSample,immune-Bcell; MCSA,totalSample,immune-NK; MCSA,totalSample,immune-Neutrophil; MCSA,totalSample,immune-CD4_T; MCSA,totalSample,immune-Gamma_delta; MCSA,totalSample,immune-CD4_naive; MCSA,totalSample,immune-Tr1; MCSA,totalSample,immune-nTreg; MCSA,totalSample,immune-Central_memory; MCSA,totalSample,immune-InfiltrationScore; ROSMAP,totalSample,immune-Effector_memory; SRP223445,totalSample,immune-Exhausted; SRP223445,totalSample,immune-InfiltrationScore; ROSMAP,female-excludeAge90+,survival; ROSMAP,male and female-excludeAge90+,survival
PLBD1	Homo_723,female,AD.vs.control; Homo_723,male,AD.vs.control; Homo_723,female,AD.vs.MCI; Homo_723,male,AD.vs.MCI; Homo_714,female,AD.vs.control; Homo_714,male,AD.vs.control; Homo_714,female,AD.vs.MCI; Homo_714,male,AD.vs.MCI; Homo_633,female,AD.vs.control; Homo_633,female,AD.vs.MCI; ROSMAP,female,AD.vs.control; ROSMAP,female,MCI.vs.control; ROSMAP,control,male.vs.female; Emory,totalSample,age; Emory,MCI,age; MCSA,MCI,age; ROSMAP,MCI,last.visit.age; Emory,totalSample,immune-DC; Emory,totalSample,immune-B_cell; Emory,totalSample,immune-Monocyte; Emory,totalSample,immune-Macrophage; Emory,totalSample,immune-NK; Emory,totalSample,immune-Neutrophil; Emory,totalSample,immune-CD4_T; Emory,totalSample,immune-CD8_T; Emory,totalSample,immune-Gamma_delta; Emory,totalSample,immune-nTreg; Emory,totalSample,immune-Th1; Emory,totalSample,immune-Tfh; Emory,totalSample,immune-CD8_naive; Emory,totalSample,immune-Cytotoxic; Emory,totalSample,immune-Exhausted; Emory,totalSample,immune-MAIT; Homo_723,totalSample,immune-Monocyte; Homo_723,totalSample,immune-Neutrophil; Homo_723,totalSample,immune-Gamma_delta; Homo_723,totalSample,immune-DC; Homo_723,totalSample,immune-Exhausted; Homo_723,totalSample,immune-Th2; Homo_723,totalSample,immune-Bcell; Homo_723,totalSample,immune-Th1; Homo_714,totalSample,immune-Monocyte; Homo_714,totalSample,immune-Neutrophil; Homo_714,totalSample,immune-Gamma_delta; Homo_714,totalSample,immune-Th2; Homo_714,totalSample,immune-Exhausted; Homo_714,totalSample,immune-Th1; Homo_714,totalSample,immune-DC; Homo_633,totalSample,immune-Monocyte; Homo_633,totalSample,immune-Exhausted; Homo_633,totalSample,immune-Gamma_delta; Homo_633,totalSample,immune-Th2; Homo_633,totalSample,immune-Neutrophil; Homo_633,totalSample,immune-CD8_T; Homo_633,totalSample,immune-Macrophage; Homo_633,totalSample,immune-Th17; Homo_633,totalSample,immune-MAIT; Homo_633,totalSample,immune-DC; MCSA,totalSample,immune-Monocyte; MCSA,totalSample,immune-Macrophage; MCSA,totalSample,immune-Neutrophil; MCSA,totalSample,immune-CD8_T; MCSA,totalSample,immune-Gamma_delta; MCSA,totalSample,immune-Th17; MCSA,totalSample,immune-Tfh; MCSA,totalSample,immune-Cytotoxic; MCSA,totalSample,immune-Exhausted; MCSA,totalSample,immune-Central_memory; MCSA,totalSample,immune-Effector_memory; MCSA,totalSample,immune-InfiltrationScore; ROSMAP,totalSample,immune-Effector_memory; SRP223445,totalSample,immune-Neutrophil; SRP223445,totalSample,immune-CD4_T; SRP223445,totalSample,immune-Gamma_delta; SRP223445,totalSample,immune-CD4_naive; SRP223445,totalSample,immune-Th1; [illegible]
ST6GALNAC2	Homo_723,female,AD.vs.control; Homo_723,male,AD.vs.control; Homo_723,female,AD.vs.MCI; Homo_723,male,AD.vs.MCI; Homo_714,female,AD.vs.control; Homo_714,male,AD.vs.control; Homo_714,female,AD.vs.MCI; Homo_714,male,AD.vs.MCI; Homo_633,female,AD.vs.control; Homo_633,female,AD.vs.MCI; Emory,totalSample,age; Emory,MCI,age; ROSMAP,AD,last.visit.age; Emory,totalSample,immune-B_cell; Emory,totalSample,immune-Macrophage; Emory,totalSample,immune-NK; Emory,totalSample,immune-Neutrophil; Emory,totalSample,immune-CD4_T; Emory,totalSample,immune-CD8_T; Emory,totalSample,immune-Gamma_delta; Emory,totalSample,immune-Th1; Emory,totalSample,immune-Th17; Emory,totalSample,immune-Tfh; Emory,totalSample,immune-CD8_naive; Emory,totalSample,immune-Cytotoxic; Emory,totalSample,immune-Exhausted; Emory,totalSample,immune-MAIT; Emory,totalSample,immune-InfiltrationScore; Homo_723,totalSample,immune-Neutrophil; Homo_723,totalSample,immune-Th1; Homo_723,totalSample,immune-Gamma_delta; Homo_723,totalSample,immune-Bcell; Homo_714,totalSample,immune-Neutrophil; Homo_714,totalSample,immune-Th1; Homo_714,totalSample,immune-Gamma_delta; Homo_714,totalSample,immune-Exhausted; Homo_633,totalSample,immune-Neutrophil; Homo_633,totalSample,immune-Exhausted; Homo_633,totalSample,immune-CD8_T; Homo_633,totalSample,immune-MAIT; Homo_633,totalSample,immune-Gamma_delta; Homo_633,totalSample,immune-Th17; Homo_633,totalSample,immune-Th2; Homo_633,totalSample,immune-Th1; MCSA,totalSample,immune-Bcell; MCSA,totalSample,immune-Macrophage; MCSA,totalSample,immune-Neutrophil; MCSA,totalSample,immune-Gamma_delta; MCSA,totalSample,immune-Th1; MCSA,totalSample,immune-Th17; MCSA,totalSample,immune-Exhausted; SRP223445,totalSample,immune-CD4_naive; SRP223445,totalSample,immune-Th17; ROSMAP,male-excludeAge90+,survival; ROSMAP,male-includeAge90+,survival

TNFSF8	Homo_723,female,AD.vs.control; Homo_723,male,AD.vs.control; Homo_723,female,AD.vs.MCI; Homo_723,male,AD.vs.MCI; Homo_714,female,AD.vs.control; Homo_714,male,AD.vs.control; Homo_714,female,AD.vs.MCI; Homo_714,male,AD.vs.MCI; Homo_633,female,AD.vs.control; Homo_633,female,AD.vs.MCI; MCSA,MCI,age; Emory,totalSample,immune-DC; Emory,totalSample,immune-B_cell; Emory,totalSample,immune-Monocyte; Emory,totalSample,immune-CD4_T; Emory,totalSample,immune-Gamma_delta; Emory,totalSample,immune-Tr1; Emory,totalSample,immune-iTreg; Emory,totalSample,immune-Th17; Emory,totalSample,immune-Central_memory; Homo_723,totalSample,immune-Th2; Homo_723,totalSample,immune-Tfh; Homo_723,totalSample,immune-Monocyte; Homo_723,totalSample,immune-iTreg; Homo_723,totalSample,immune-DC; Homo_723,totalSample,immune-Tr1; Homo_723,totalSample,immune-Effector_memory; Homo_723,totalSample,immune-CD4_T; Homo_723,totalSample,immune-Gamma_delta; Homo_723,totalSample,immune-Bcell; Homo_723,totalSample,immune-CD4_naive; Homo_723,totalSample,immune-InfiltrationScore; Homo_714,totalSample,immune-Th2; Homo_714,totalSample,immune-Tfh; Homo_714,totalSample,immune-Monocyte; Homo_714,totalSample,immune-Tr1; Homo_714,totalSample,immune-Effector_memory; Homo_714,totalSample,immune-CD4_T; Homo_714,totalSample,immune-Central_memory; Homo_714,totalSample,immune-Gamma_delta; Homo_714,totalSample,immune-CD4_naive; Homo_714,totalSample,immune-iTreg; Homo_633,totalSample,immune-Th2; Homo_633,totalSample,immune-Monocyte; Homo_633,totalSample,immune-Tfh; Homo_633,totalSample,immune-CD4_T; Homo_633,totalSample,immune-Central_memory; Homo_633,totalSample,immune-Effector_memory; Homo_633,totalSample,immune-Exhausted; Homo_633,totalSample,immune-CD4_naive; Homo_633,totalSample,immune-Tr1; Homo_633,totalSample,immune-Th17; Homo_633,totalSample,immune-Gamma_delta; Homo_633,totalSample,immune-Bcell; Homo_633,totalSample,immune-InfiltrationScore; MCSA,totalSample,immune-DC; MCSA,totalSample,immune-Monocyte; MCSA,totalSample,immune-Macrophage; MCSA,totalSample,immune-CD4_T; MCSA,totalSample,immune-CD4_naive; MCSA,totalSample,immune-Tr1; MCSA,totalSample,immune-nTreg; MCSA,totalSample,immune-iTreg; MCSA,totalSample,immune-Tfh; MCSA,totalSample,immune-CD8_naive; MCSA,totalSample,immune-Cytotoxic; MCSA,totalSample,immune-Exhausted; MCSA,totalSample,immune-MAIT; MCSA,totalSample,immune-Central_memory; SRP223445,totalSample,immune-Th17; SRP223445,totalSample,immune-Exhausted; SRP223445,totalSample,immune-InfiltrationScore
DHRS9	Homo_723,female,AD.vs.control; Homo_723,male,AD.vs.control; Homo_723,female,AD.vs.MCI; Homo_723,male,AD.vs.MCI; Homo_714,female,AD.vs.control; Homo_714,male,AD.vs.control; Homo_714,female,AD.vs.MCI; Homo_714,male,AD.vs.MCI; Homo_633,female,AD.vs.control; Homo_633,female,AD.vs.MCI; ROSMAP,female,AD.vs.control; ROSMAP,AD,last.visit.age; ROSMAP,MCI,last.visit.age; Emory,totalSample,immune-Neutrophil; Emory,totalSample,immune-CD4_T; Emory,totalSample,immune-CD8_T; Emory,totalSample,immune-Gamma_delta; Emory,totalSample,immune-Tfh; Emory,totalSample,immune-CD8_naive; Emory,totalSample,immune-Cytotoxic; Emory,totalSample,immune-Exhausted; Emory,totalSample,immune-MAIT; Homo_723,totalSample,immune-Monocyte; Homo_723,totalSample,immune-Neutrophil; Homo_723,totalSample,immune-InfiltrationScore; Homo_723,totalSample,immune-Gamma_delta; Homo_723,totalSample,immune-Bcell; Homo_714,totalSample,immune-Monocyte; Homo_714,totalSample,immune-Th2; Homo_714,totalSample,immune-Neutrophil; Homo_714,totalSample,immune-Gamma_delta; Homo_633,totalSample,immune-Monocyte; Homo_633,totalSample,immune-Exhausted; Homo_633,totalSample,immune-Th2; Homo_633,totalSample,immune-CD8_T; Homo_633,totalSample,immune-InfiltrationScore; Homo_633,totalSample,immune-Gamma_delta; MCSA,totalSample,immune-Neutrophil; MCSA,totalSample,immune-CD8_T; MCSA,totalSample,immune-Cytotoxic; MCSA,totalSample,immune-Effector_memory; SRP223445,totalSample,immune-CD4_naive; SRP223445,totalSample,immune-Th17; SRP223445,totalSample,immune-Exhausted; SRP223445,totalSample,immune-InfiltrationScore
SLAMF1	Homo_723,female,AD.vs.control; Homo_723,male,AD.vs.control; Homo_723,female,AD.vs.MCI; Homo_723,male,AD.vs.MCI; Homo_714,control,male.vs.female; Homo_633,control,male.vs.female; MCSA,totalSample,age; MCSA,control,age; ROSMAP,MCI,sample.age; Emory,totalSample,immune-Macrophage; Emory,totalSample,immune-Neutrophil; Emory,totalSample,immune-CD4_T; Emory,totalSample,immune-CD8_T; Emory,totalSample,immune-Tr1; Emory,totalSample,immune-nTreg; Emory,totalSample,immune-iTreg; Emory,totalSample,immune-Tfh; Emory,totalSample,immune-CD8_naive; Emory,totalSample,immune-Exhausted; Emory,totalSample,immune-MAIT; Emory,totalSample,immune-Central_memory; Emory,totalSample,immune-InfiltrationScore; Homo_723,totalSample,immune-Tfh; Homo_723,totalSample,immune-iTreg; Homo_723,totalSample,immune-Th2; Homo_723,totalSample,immune-CD4_T; Homo_723,totalSample,immune-Tr1; Homo_723,totalSample,immune-CD4_naive; Homo_723,totalSample,immune-nTreg; Homo_723,totalSample,immune-Macrophage; Homo_723,totalSample,immune-Effector_memory; Homo_723,totalSample,immune-Central_memory; Homo_723,totalSample,immune-InfiltrationScore; Homo_723,totalSample,immune-CD8_T; Homo_723,totalSample,immune-DC; Homo_723,totalSample,immune-MAIT; Homo_723,totalSample,immune-Gamma_delta; Homo_714,totalSample,immune-CD4_T; Homo_714,totalSample,immune-CD4_naive; Homo_714,totalSample,immune-nTreg; Homo_714,totalSample,immune-Th1; Homo_714,totalSample,immune-Tfh; Homo_714,totalSample,immune-iTreg; Homo_714,totalSample,immune-MAIT; Homo_714,totalSample,immune-Tr1; Homo_714,totalSample,immune-Effector_memory; Homo_714,totalSample,immune-Macrophage; Homo_714,totalSample,immune-Neutrophil; Homo_714,totalSample,immune-Th2; Homo_714,totalSample,immune-Central_memory; Homo_714,totalSample,immune-NKT; Homo_714,totalSample,immune-InfiltrationScore; Homo_714,totalSample,immune-Monocyte; Homo_714,totalSample,immune-CD8_T; Homo_633,totalSample,immune-CD4_T; Homo_633,totalSample,immune-CD4_naive; Homo_633,totalSample,immune-nTreg; Homo_633,totalSample,immune-Tfh; Homo_633,totalSample,immune-MAIT; Homo_633,totalSample,immune-Th1; Homo_633,totalSample,immune-Effector_memory; Homo_633,totalSample,immune-iTreg; Homo_633,totalSample,immune-Tr1; Homo_633,totalSample,immune-Central_memory; Homo_633,totalSample,immune-Neutrophil; Homo_633,totalSample,immune-Macrophage; Homo_633,totalSample,immune-NKT; Homo_633,totalSample,immune-Monocyte; Homo_633,totalSample,immune-InfiltrationScore; Homo_633,totalSample,immune-Th2; Homo_633,totalSample,immune-CD8_T; MCSA,totalSample,immune-DC; MCSA,totalSample,immune-Bcell; MCSA,totalSample,immune-Monocyte; MCSA,totalSample,immune-Macrophage; MCSA,totalSample,immune-Neutrophil; MCSA,totalSample,immune-CD4_T; MCSA,totalSample,immune-CD4_naive; MCSA,totalSample,immune-Tr1; MCSA,totalSample,immune-nTreg; MCSA,totalSample,immune-iTreg; MCSA,totalSample,immune-Th2; MCSA,totalSample,immune-Th17; MCSA,totalSample,immune-Tfh; MCSA,totalSample,immune-CD8_naive; MCSA,totalSample,immune-MAIT; MCSA,totalSample,immune-Central_memory; MCSA,totalSample,immune-Effector_memory; MCSA,totalSample,immune-InfiltrationScore; ROSMAP,totalSample,immune-NKT; ROSMAP,totalSample,immune-MAIT; ROSMAP,totalSample,immune-Central_memory; ROSMAP,totalSample,immune-InfiltrationScore; SRP223445,totalSample,immune-Th17; SRP223445,totalSample,immune-Exhausted; SRP223445,totalSample,immune-InfiltrationScore; ROSMAP,female-excludeAge90+,survival; ROSMAP,female-includeAge90+,survival; ROSMAP,male-excludeAge90+,survival; [illegible]
HLA-DOB	Homo_723,female,AD.vs.control; Homo_723,male,AD.vs.control; Homo_723,female,AD.vs.MCI; Homo_723,male,AD.vs.MCI; Homo_723,female,MCI.vs.control; Homo_723,male,MCI.vs.control; Homo_714,female,AD.vs.control; Homo_714,male,AD.vs.control; Homo_714,female,AD.vs.MCI; Homo_714,male,AD.vs.MCI; Homo_714,female,MCI.vs.control; Homo_714,male,MCI.vs.control; Homo_633,female,AD.vs.control; Homo_633,female,AD.vs.MCI; Homo_633,female,MCI.vs.control; Homo_633,male,MCI.vs.control; MCSA,totalSample,age; MCSA,control,age; ROSMAP,AD,sample.age; Emory,totalSample,immune-B_cell; Emory,totalSample,immune-Macrophage; Emory,totalSample,immune-Neutrophil; Emory,totalSample,immune-CD4_T; Emory,totalSample,immune-Tr1; Emory,totalSample,immune-iTreg; Emory,totalSample,immune-Tfh; Emory,totalSample,immune-CD8_naive; Emory,totalSample,immune-InfiltrationScore; Homo_723,totalSample,immune-Th2; Homo_723,totalSample,immune-Tfh; Homo_723,totalSample,immune-iTreg; Homo_723,totalSample,immune-Macrophage; Homo_723,totalSample,immune-CD4_T; Homo_723,totalSample,immune-Tr1; Homo_723,totalSample,immune-Effector_memory; Homo_723,totalSample,immune-DC; Homo_723,totalSample,immune-InfiltrationScore; Homo_723,totalSample,immune-nTreg; Homo_723,totalSample,immune-CD8_T; Homo_714,totalSample,immune-Th2; Homo_714,totalSample,immune-Tfh; Homo_714,totalSample,immune-CD4_T; Homo_714,totalSample,immune-Tr1; Homo_714,totalSample,immune-Effector_memory; Homo_714,totalSample,immune-nTreg; Homo_714,totalSample,immune-Macrophage; Homo_714,totalSample,immune-Central_memory; Homo_714,totalSample,immune-iTreg; Homo_633,totalSample,immune-Th2; Homo_633,totalSample,immune-Tfh; Homo_633,totalSample,immune-CD4_T; Homo_633,totalSample,immune-nTreg; Homo_633,totalSample,immune-Central_memory; Homo_633,totalSample,immune-Effector_memory; Homo_633,totalSample,immune-Th17; Homo_633,totalSample,immune-Tr1; Homo_633,totalSample,immune-CD4_naive; Homo_633,totalSample,immune-Gamma_delta; MCSA,totalSample,immune-Bcell; MCSA,totalSample,immune-Monocyte; MCSA,totalSample,immune-Macrophage; MCSA,totalSample,immune-Neutrophil; MCSA,totalSample,immune-CD4_T; MCSA,totalSample,immune-Gamma_delta; MCSA,totalSample,immune-CD4_naive; MCSA,totalSample,immune-Tr1; MCSA,totalSample,immune-nTreg; MCSA,totalSample,immune-iTreg; MCSA,totalSample,immune-Th17; MCSA,totalSample,immune-Tfh; MCSA,totalSample,immune-CD8_naive; MCSA,totalSample,immune-Central_memory; MCSA,totalSample,immune-InfiltrationScore; SRP223445,totalSample,immune-Neutrophil; SRP223445,totalSample,immune-nTreg; [illegible]
GZMK	Homo_723,female,AD.vs.control; Homo_723,male,AD.vs.control; Homo_723,female,AD.vs.MCI; Homo_723,male,AD.vs.MCI; Homo_723,female,MCI.vs.control; Homo_723,male,MCI.vs.control; Homo_714,female,AD.vs.control; Homo_714,male,AD.vs.control; Homo_714,female,AD.vs.MCI; Homo_714,male,AD.vs.MCI; Homo_714,female,MCI.vs.control; Homo_714,male,MCI.vs.control; Homo_633,female,AD.vs.control; Homo_633,female,AD.vs.MCI; Homo_633,female,MCI.vs.control; Homo_633,male,MCI.vs.control; ROSMAP,female,AD.vs.control; ROSMAP,female,MCI.vs.control; ACOM,AD,age; ROSMAP,MCI,last.visit.age; Emory,totalSample,immune-Monocyte; Emory,totalSample,immune-Macrophage; Emory,totalSample,immune-Neutrophil; Emory,totalSample,immune-CD4_T; Emory,totalSample,immune-CD8_T; Emory,totalSample,immune-Gamma_delta; Emory,totalSample,immune-CD4_naive; Emory,totalSample,immune-Tr1; Emory,totalSample,immune-iTreg; Emory,totalSample,immune-Th2; Emory,totalSample,immune-Tfh; Emory,totalSample,immune-Cytotoxic; Emory,totalSample,immune-Exhausted; Emory,totalSample,immune-Effector_memory; Emory,totalSample,immune-InfiltrationScore; Homo_723,totalSample,immune-Monocyte; Homo_723,totalSample,immune-MAIT; Homo_723,totalSample,immune-DC; Homo_723,totalSample,immune-Tfh; Homo_723,totalSample,immune-Effector_memory; Homo_723,totalSample,immune-Th2; Homo_723,totalSample,immune-Cytotoxic; Homo_723,totalSample,immune-Macrophage; Homo_723,totalSample,immune-InfiltrationScore; Homo_723,totalSample,immune-CD8_T; Homo_723,totalSample,immune-Bcell; Homo_723,totalSample,immune-iTreg; Homo_714,totalSample,immune-Tfh; Homo_714,totalSample,immune-Th2; Homo_714,totalSample,immune-Effector_memory; Homo_714,totalSample,immune-MAIT; Homo_714,totalSample,immune-CD8_T; Homo_714,totalSample,immune-Cytotoxic; Homo_714,totalSample,immune-Monocyte; Homo_714,totalSample,immune-InfiltrationScore; Homo_714,totalSample,immune-Macrophage; Homo_714,totalSample,immune-Bcell; Homo_633,totalSample,immune-Monocyte; Homo_633,totalSample,immune-InfiltrationScore; Homo_633,totalSample,immune-MAIT; Homo_633,totalSample,immune-Effector_memory; Homo_633,totalSample,immune-Cytotoxic; Homo_633,totalSample,immune-Bcell; Homo_633,totalSample,immune-Neutrophil; Homo_633,totalSample,immune-Tfh; Homo_633,totalSample,immune-CD8_T; Homo_633,totalSample,immune-Th2; MCSA,totalSample,immune-DC; MCSA,totalSample,immune-Macrophage; MCSA,totalSample,immune-Neutrophil; MCSA,totalSample,immune-CD8_T; MCSA,totalSample,immune-Gamma_delta; MCSA,totalSample,immune-Th2; MCSA,totalSample,immune-Cytotoxic; MCSA,totalSample,immune-MAIT; MCSA,totalSample,immune- [illegible]
NAAA	Homo_723,female,AD.vs.control; Homo_723,male,AD.vs.control; Homo_723,female,AD.vs.MCI; Homo_723,male,AD.vs.MCI; Homo_714,female,AD.vs.control; Homo_714,male,AD.vs.control; Homo_714,female,AD.vs.MCI; Homo_714,male,AD.vs.MCI; Homo_633,female,AD.vs.control; Homo_633,female,AD.vs.MCI; ROSMAP,female,AD.vs.control; ROSMAP,MCI,last.visit.age; ROSMAP,MCI,sample.age; Emory,totalSample,immune-Tr1; Emory,totalSample,immune-iTreg; Emory,totalSample,immune-InfiltrationScore; Homo_723,totalSample,immune-iTreg; Homo_723,totalSample,immune-Tfh; Homo_723,totalSample,immune-InfiltrationScore; Homo_723,totalSample,immune-Th2; Homo_723,totalSample,immune-Gamma_delta; Homo_723,totalSample,immune-Effector_memory; Homo_723,totalSample,immune-Tr1; Homo_723,totalSample,immune-Bcell; Homo_723,totalSample,immune-CD4_T; Homo_723,totalSample,immune-CD4_naive; Homo_714,totalSample,immune-Th2; Homo_714,totalSample,immune-Tfh; Homo_714,totalSample,immune-Effector_memory; Homo_714,totalSample,immune-Tr1; Homo_714,totalSample,immune-CD4_T; Homo_714,totalSample,immune-iTreg; Homo_714,totalSample,immune-InfiltrationScore; Homo_714,totalSample,immune-Gamma_delta; Homo_714,totalSample,immune-Central_memory; Homo_633,totalSample,immune-Tfh; Homo_633,totalSample,immune-InfiltrationScore; Homo_633,totalSample,immune-Effector_memory; Homo_633,totalSample,immune-CD4_T; Homo_633,totalSample,immune-Central_memory; Homo_633,totalSample,immune-CD4_naive; Homo_633,totalSample,immune-Th2; Homo_633,totalSample,immune-Gamma_delta; Homo_633,totalSample,immune-Tr1; Homo_633,totalSample,immune-iTreg; Homo_633,totalSample,immune-Exhausted; Homo_633,totalSample,immune-nTreg; Homo_633,totalSample,immune-Bcell; MCSA,totalSample,immune-CD4_T; MCSA,totalSample,immune-CD4_naive; MCSA,totalSample,immune-Tr1; MCSA,totalSample,immune-nTreg; MCSA,totalSample,immune-iTreg; MCSA,totalSample,immune-Th17; MCSA,totalSample,immune-Tfh;
MS4A4E	Homo_723,female,AD.vs.control; Homo_723,male,AD.vs.control; Homo_723,female,AD.vs.MCI; Homo_723,male,AD.vs.MCI; Homo_714,female,AD.vs.control; Homo_714,male,AD.vs.control; Homo_714,female,AD.vs.MCI; Homo_714,male,AD.vs.MCI; Homo_633,female,AD.vs.control; Homo_633,female,AD.vs.MCI; Emory,totalSample,immune-Gamma_delta; Emory,totalSample,immune-CD8_naive; Homo_723,totalSample,immune-Th2; Homo_723,totalSample,immune-Monocyte; Homo_723,totalSample,immune-iTreg; Homo_723,totalSample,immune-Tfh; Homo_723,totalSample,immune-InfiltrationScore; Homo_723,totalSample,immune-Effector_memory; Homo_723,totalSample,immune-Gamma_delta; Homo_723,totalSample,immune-Bcell; Homo_714,totalSample,immune-Th2; Homo_714,totalSample,immune-Tfh; Homo_714,totalSample,immune-Effector_memory; Homo_714,totalSample,immune-Gamma_delta; Homo_633,totalSample,immune-Exhausted; Homo_633,totalSample,immune-Th2; Homo_633,totalSample,immune-Monocyte; Homo_633,totalSample,immune-InfiltrationScore; Homo_633,totalSample,immune-Gamma_delta; Homo_633,totalSample,immune-Th17; Homo_633,totalSample,immune-Tfh; Homo_633,totalSample,immune-Effector_memory; MCSA,totalSample,immune-CD4_T; MCSA,totalSample,immune-Gamma_delta; MCSA,totalSample,immune-CD4_naive; MCSA,totalSample,immune-Tr1; MCSA,totalSample,immune-nTreg; MCSA,totalSample,immune-iTreg; MCSA,totalSample,immune-Th17; MCSA,totalSample,immune-Cytotoxic; MCSA,totalSample,immune-Exhausted; ROSMAP,totalSample,immune-Effector_memory
LIPN	Homo_723,female,AD.vs.control; Homo_723,male,AD.vs.control; Homo_723,female,AD.vs.MCI; Homo_723,male,AD.vs.MCI; Homo_723,MCI,male.vs.female; Homo_714,female,AD.vs.control; Homo_714,male,AD.vs.control; Homo_714,female,AD.vs.MCI; Homo_714,male,AD.vs.MCI; Homo_714,MCI,male.vs.female; Homo_633,female,AD.vs.control; Homo_633,female,AD.vs.MCI; Homo_633,female,MCI.vs.control; Homo_633,MCI,male.vs.female; MCSA,MCI,age; Emory,totalSample,immune-Monocyte; Emory,totalSample,immune-Neutrophil; Emory,totalSample,immune-CD8_T; Emory,totalSample,immune-Gamma_delta; Emory,totalSample,immune-nTreg; Emory,totalSample,immune-Th1; Emory,totalSample,immune-Tfh; Emory,totalSample,immune-CD8_naive; Homo_723,totalSample,immune-Monocyte; Homo_723,totalSample,immune-DC; Homo_723,totalSample,immune-Neutrophil; Homo_723,totalSample,immune-Gamma_delta; Homo_723,totalSample,immune-Th2; Homo_723,totalSample,immune-Bcell; Homo_714,totalSample,immune-Monocyte; Homo_714,totalSample,immune-Th2; Homo_714,totalSample,immune-Tfh; Homo_714,totalSample,immune-Gamma_delta; Homo_714,totalSample,immune-DC; Homo_633,totalSample,immune-Monocyte; Homo_633,totalSample,immune-Th2; Homo_633,totalSample,immune-Exhausted; Homo_633,totalSample,immune-Gamma_delta; Homo_633,totalSample,immune-Th17; MCSA,totalSample,immune-DC; MCSA,totalSample,immune-Monocyte; MCSA,totalSample,immune-Neutrophil; MCSA,totalSample,immune-CD8_T; MCSA,totalSample,immune-iTreg; MCSA,totalSample,immune-Th1; MCSA,totalSample,immune-Cytotoxic; MCSA,totalSample,immune-Exhausted; MCSA,totalSample,immune-Effector_memory
DNAJC25-GNG10	Homo_723,female,AD.vs.control; Homo_723,female,AD.vs.MCI; Homo_714,female,AD.vs.control; ROSMAP,female,AD.vs.control; ROSMAP,female,MCI.vs.control; ROSMAP,MCI,last.visit.age; ROSMAP,MCI,sample.age; Homo_723,totalSample,immune-Monocyte; ROSMAP,totalSample,immune-Effector_memory
NSG2	Homo_723,female,AD.vs.control; Homo_723,female,AD.vs.MCI; Homo_723,male,MCI.vs.control; Homo_723,AD,male.vs.female; Homo_714,female,AD.vs.MCI; Homo_714,male,MCI.vs.control; Homo_633,male,MCI.vs.control; MCSA,MCI,age; ROSMAP,MCI,last.visit.age; ROSMAP,MCI,sample.age; Homo_723,totalSample,immune-Monocyte; Homo_723,totalSample,immune-Exhausted; Homo_723,totalSample,immune-Th2; Homo_723,totalSample,immune-Th17; Homo_714,totalSample,immune-Monocyte; Homo_714,totalSample,immune-Th2; Homo_633,totalSample,immune-Th2; Homo_633,totalSample,immune-Monocyte; Homo_633,totalSample,immune-Th17; Homo_633,totalSample,immune-Exhausted; MCSA,totalSample,immune-Neutrophil; MCSA,totalSample,immune-Th17; ROSMAP,totalSample,immune-Neutrophil; ROSMAP,totalSample,immune-InfiltrationScore
CD8B2	Homo_723,female,AD.vs.control; Homo_723,male,AD.vs.control; Homo_723,female,AD.vs.MCI; Homo_723,male,AD.vs.MCI; Homo_714,female,AD.vs.control; Homo_714,male,AD.vs.control; Homo_714,female,AD.vs.MCI; Homo_714,male,AD.vs.MCI; Homo_633,female,AD.vs.control; Homo_633,female,AD.vs.MCI; ACOM,AD,age; ACOM,totalSample,age; Emory,totalSample,age; Emory,control,age; Emory,totalSample,immune-Macrophage; Emory,totalSample,immune-Neutrophil; Emory,totalSample,immune-CD4_T; Emory,totalSample,immune-CD8_T; Emory,totalSample,immune-Gamma_delta; Emory,totalSample,immune-Tr1; Emory,totalSample,immune-iTreg; Emory,totalSample,immune-Tfh; Emory,totalSample,immune-CD8_naive; Emory,totalSample,immune-Cytotoxic; Emory,totalSample,immune-Exhausted; Emory,totalSample,immune-MAIT; Emory,totalSample,immune-Effector_memory; Emory,totalSample,immune-InfiltrationScore; Homo_723,totalSample,immune-MAIT; Homo_723,totalSample,immune-CD8_T; Homo_723,totalSample,immune-Exhausted; Homo_723,totalSample,immune-Cytotoxic; Homo_723,totalSample,immune-InfiltrationScore; Homo_723,totalSample,immune-Macrophage; Homo_723,totalSample,immune-Tfh; Homo_723,totalSample,immune-Effector_memory; Homo_723,totalSample,immune-Th17; Homo_723,totalSample,immune-iTreg; Homo_723,totalSample,immune-Monocyte; Homo_723,totalSample,immune-CD4_naive; Homo_723,totalSample,immune-Bcell; Homo_723,totalSample,immune-CD8_naive; Homo_723,totalSample,immune-CD4_T; Homo_714,totalSample,immune-CD8_T; Homo_714,totalSample,immune-MAIT; Homo_714,totalSample,immune-Cytotoxic; Homo_714,totalSample,immune-Exhausted; Homo_714,totalSample,immune-InfiltrationScore; Homo_714,totalSample,immune-Macrophage; Homo_714,totalSample,immune-Effector_memory; Homo_714,totalSample,immune-Tfh; Homo_714,totalSample,immune-Th17; Homo_714,totalSample,immune-Neutrophil; Homo_714,totalSample,immune-CD8_naive; Homo_714,totalSample,immune-Monocyte; Homo_714,totalSample,immune-Bcell; Homo_714,totalSample,immune-Gamma_delta; Homo_714,totalSample,immune-CD4_naive; Homo_714,totalSample,immune-CD4_T; Homo_633,totalSample,immune-InfiltrationScore; Homo_633,totalSample,immune-MAIT; Homo_633,totalSample,immune-CD8_T; Homo_633,totalSample,immune-Cytotoxic; Homo_633,totalSample,immune-Neutrophil; Homo_633,totalSample,immune-Effector_memory; Homo_633,totalSample,immune-Macrophage; Homo_633,totalSample,immune-Exhausted; Homo_633,totalSample,immune-Gamma_delta; Homo_633,totalSample,immune-CD4_naive; Homo_633,totalSample,immune-Bcell; Homo_633,totalSample,immune-Tfh; Homo_633,totalSample,immune-Th17; Homo_633,totalSample,immune-Monocyte; Homo_633,totalSample,immune-CD8_naive; Homo_633,totalSample,immune-nTreg; MCSA,totalSample,immune-Monocyte; MCSA,totalSample,immune-Macrophage; MCSA,totalSample,immune-Neutrophil; MCSA,totalSample,immune-CD4_T; MCSA,totalSample,immune-CD8_T; MCSA,totalSample,immune-NKT; MCSA,totalSample,immune-Gamma_delta; MCSA,totalSample,immune-Tr1; MCSA,totalSample,immune-Tfh; MCSA,totalSample,immune-CD8_naive; MCSA,totalSample,immune-Cytotoxic; MCSA,totalSample,immune-MAIT; MCSA,totalSample,immune-Central_memory; MCSA,totalSample,immune-Effector_memory; ROSMAP,totalSample,immune-MAIT; [illegible]
FCRLA	Homo_723,female,AD.vs.control; Homo_723,male,AD.vs.control; Homo_723,female,AD.vs.MCI; Homo_723,male,AD.vs.MCI; Homo_714,female,AD.vs.control; Homo_714,male,AD.vs.control; Homo_714,female,AD.vs.MCI; Homo_714,male,AD.vs.MCI; Homo_633,female,AD.vs.control; Homo_633,female,AD.vs.MCI; ROSMAP,female,AD.vs.control; MCSA,totalSample,age; MCSA,control,age; ROSMAP,AD,MMSE; Emory,totalSample,immune-B_cell; Emory,totalSample,immune-Macrophage; Emory,totalSample,immune-Neutrophil; Emory,totalSample,immune-CD4_T; Emory,totalSample,immune-Tr1; Emory,totalSample,immune-iTreg; Emory,totalSample,immune-Tfh; Emory,totalSample,immune-CD8_naive; Emory,totalSample,immune-InfiltrationScore; Homo_723,totalSample,immune-iTreg; Homo_723,totalSample,immune-Tfh; Homo_723,totalSample,immune-Th2; Homo_723,totalSample,immune-CD4_T; Homo_723,totalSample,immune-Tr1; Homo_723,totalSample,immune-Macrophage; Homo_723,totalSample,immune-nTreg; Homo_723,totalSample,immune-CD4_naive; Homo_723,totalSample,immune-Central_memory; Homo_723,totalSample,immune-CD8_T; Homo_723,totalSample,immune-InfiltrationScore; Homo_723,totalSample,immune-Gamma_delta; Homo_723,totalSample,immune-Effector_memory; Homo_714,totalSample,immune-Tfh; Homo_714,totalSample,immune-Th2; Homo_714,totalSample,immune-CD4_T; Homo_714,totalSample,immune-iTreg; Homo_714,totalSample,immune-Tr1; Homo_714,totalSample,immune-nTreg; Homo_714,totalSample,immune-Central_memory; Homo_714,totalSample,immune-Effector_memory; Homo_714,totalSample,immune-Macrophage; Homo_714,totalSample,immune-CD4_naive; Homo_714,totalSample,immune-Gamma_delta; Homo_633,totalSample,immune-CD4_T; Homo_633,totalSample,immune-Tfh; Homo_633,totalSample,immune-nTreg; Homo_633,totalSample,immune-Central_memory; Homo_633,totalSample,immune-CD4_naive; Homo_633,totalSample,immune-Tr1; Homo_633,totalSample,immune-iTreg; Homo_633,totalSample,immune-Effector_memory; Homo_633,totalSample,immune-Th2; Homo_633,totalSample,immune-NKT; Homo_633,totalSample,immune-Gamma_delta; Homo_633,totalSample,immune-Th1; Homo_633,totalSample,immune-InfiltrationScore; Homo_633,totalSample,immune-Neutrophil; Homo_633,totalSample,immune-Macrophage; MCSA,totalSample,immune-Bcell; MCSA,totalSample,immune-Monocyte; MCSA,totalSample,immune-Macrophage; MCSA,totalSample,immune-Neutrophil; MCSA,totalSample,immune-CD4_T; MCSA,totalSample,immune-Gamma_delta; MCSA,totalSample,immune-CD4_naive; MCSA,totalSample,immune-Tr1; MCSA,totalSample,immune-nTreg; MCSA,totalSample,immune-iTreg; MCSA,totalSample,immune-Th17; MCSA,totalSample,immune-Tfh; [illegible]
IL9R	Homo_723,female,AD.vs.control; Homo_723,male,AD.vs.control; Homo_723,female,AD.vs.MCI; Homo_723,male,AD.vs.MCI; Homo_714,female,AD.vs.control; Homo_714,male,AD.vs.control; Homo_714,female,AD.vs.MCI; Homo_714,male,AD.vs.MCI; Homo_633,female,AD.vs.control; Homo_633,female,AD.vs.MCI; MCSA,MCI,age; ROSMAP,AD,last.visit.age; Emory,totalSample,immune-Gamma_delta; Emory,totalSample,immune-iTreg; Homo_723,totalSample,immune-Th2; Homo_723,totalSample,immune-iTreg; Homo_723,totalSample,immune-Tfh; Homo_723,totalSample,immune-InfiltrationScore; Homo_723,totalSample,immune-Effector_memory; Homo_723,totalSample,immune-Gamma_delta; Homo_723,totalSample,immune-CD4_T; Homo_723,totalSample,immune-Tr1; Homo_714,totalSample,immune-Th2; Homo_714,totalSample,immune-Tfh; Homo_714,totalSample,immune-Effector_memory; Homo_714,totalSample,immune-Tr1; Homo_714,totalSample,immune-nTreg; Homo_714,totalSample,immune-CD4_T; Homo_714,totalSample,immune-Gamma_delta; Homo_633,totalSample,immune-Th2; Homo_633,totalSample,immune-Exhausted; Homo_633,totalSample,immune-nTreg; Homo_633,totalSample,immune-Tfh; Homo_633,totalSample,immune-Gamma_delta; Homo_633,totalSample,immune-InfiltrationScore; MCSA,totalSample,immune-Monocyte; MCSA,totalSample,immune-CD4_T; MCSA,totalSample,immune-CD4_naive; MCSA,totalSample,immune-Tr1; MCSA,totalSample,immune-nTreg; MCSA,totalSample,immune-Tfh; MCSA,totalSample,immune-Central_memory; ROSMAP,male-includeAge90+,survival

SLC31A2	Homo_723,female,AD.vs.control; Homo_723,male,AD.vs.control; Homo_723,female,AD.vs.MCI; Homo_723,male,AD.vs.MCI; Homo_714,female,AD.vs.control; Homo_714,male,AD.vs.control; Homo_714,female,AD.vs.MCI; Homo_714,male,AD.vs.MCI; Homo_633,female,AD.vs.control; Homo_633,female,AD.vs.MCI; ROSMAP,female,AD.vs.control; MCSA,MCI,age; Emory,totalSample,immune-Monocyte; Emory,totalSample,immune-Neutrophil; Emory,totalSample,immune-CD8_T; Emory,totalSample,immune-Gamma_delta; Emory,totalSample,immune-nTreg; Emory,totalSample,immune-Th1; Emory,totalSample,immune-Tfh; Emory,totalSample,immune-CD8_naive; Emory,totalSample,immune-Cytotoxic; Emory,totalSample,immune-Exhausted; Homo_723,totalSample,immune-Neutrophil; Homo_723,totalSample,immune-Gamma_delta; Homo_723,totalSample,immune-InfiltrationScore; Homo_723,totalSample,immune-CD8_naive; Homo_723,totalSample,immune-Bcell; Homo_714,totalSample,immune-Neutrophil; Homo_714,totalSample,immune-Gamma_delta; Homo_633,totalSample,immune-Exhausted; Homo_633,totalSample,immune-CD8_T; Homo_633,totalSample,immune-Gamma_delta; Homo_633,totalSample,immune-Neutrophil; Homo_633,totalSample,immune-Macrophage; MCSA,totalSample,immune-Monocyte; MCSA,totalSample,immune-Neutrophil; MCSA,totalSample,immune-CD8_T; MCSA,totalSample,immune-Cytotoxic; MCSA,totalSample,immune-
NAGPA	Homo_723,female,AD.vs.control; Homo_723,male,AD.vs.control; Homo_723,female,AD.vs.MCI; Homo_723,male,AD.vs.MCI; Homo_714,female,AD.vs.control; Homo_714,male,AD.vs.control; Homo_714,female,AD.vs.MCI; Homo_714,male,AD.vs.MCI; Homo_633,female,AD.vs.control; Homo_633,female,AD.vs.MCI; ACOM,AD,age; Emory,MCI,age; MCSA,totalSample,age; MCSA,MCI,age; ROSMAP,MCI,sample.age; Emory,totalSample,immune-NK; Emory,totalSample,immune-Neutrophil; Emory,totalSample,immune-CD4_T; Emory,totalSample,immune-Tr1; Emory,totalSample,immune-iTreg; Emory,totalSample,immune-Effector_memory; Emory,totalSample,immune-InfiltrationScore; Homo_723,totalSample,immune-CD8_T; Homo_723,totalSample,immune-Exhausted; Homo_723,totalSample,immune-Cytotoxic; Homo_723,totalSample,immune-InfiltrationScore; Homo_723,totalSample,immune-Monocyte; Homo_723,totalSample,immune-Th17; Homo_723,totalSample,immune-DC; Homo_723,totalSample,immune-iTreg; Homo_723,totalSample,immune-Effector_memory; Homo_723,totalSample,immune-Central_memory; Homo_723,totalSample,immune-MAIT; Homo_714,totalSample,immune-DC; Homo_714,totalSample,immune-Monocyte; Homo_714,totalSample,immune-InfiltrationScore; Homo_714,totalSample,immune-Effector_memory; Homo_714,totalSample,immune-CD8_T; Homo_714,totalSample,immune-nTreg; Homo_714,totalSample,immune-Th17; Homo_633,totalSample,immune-DC; Homo_633,totalSample,immune-InfiltrationScore; Homo_633,totalSample,immune-Monocyte; Homo_633,totalSample,immune-nTreg; Homo_633,totalSample,immune-Th2; Homo_633,totalSample,immune-Effector_memory; Homo_633,totalSample,immune-Neutrophil; MCSA,totalSample,immune-DC; MCSA,totalSample,immune-Monocyte; MCSA,totalSample,immune-Neutrophil; MCSA,totalSample,immune-CD8_T; MCSA,totalSample,immune-NKT; MCSA,totalSample,immune-Th2; MCSA,totalSample,immune-Tfh; MCSA,totalSample,immune-Cytotoxic; MCSA,totalSample,immune-Central_memory; MCSA,totalSample,immune-Effector_memory; MCSA,totalSample,immune-InfiltrationScore; ROSMAP,totalSample,immune-Th17; ROSMAP,totalSample,immune-Effector_memory; ROSMAP,totalSample,immune-InfiltrationScore; SRP223445,totalSample,immune-InfiltrationScore; ROSMAP,male-excludeAge90+,survival
VSIG4	Homo_723,female,AD.vs.control; Homo_723,male,AD.vs.control; Homo_723,female,AD.vs.MCI; Homo_723,male,AD.vs.MCI; Homo_714,female,AD.vs.control; Homo_714,male,AD.vs.control; Homo_714,female,AD.vs.MCI; Homo_714,male,AD.vs.MCI; Homo_633,female,AD.vs.control; Homo_633,female,AD.vs.MCI; ROSMAP,female,AD.vs.control; MCSA,MCI,age; ROSMAP,AD,sample.age; Emory,totalSample,immune-DC; Emory,totalSample,immune-NK; Emory,totalSample,immune-Tfh; Emory,totalSample,immune-InfiltrationScore; Homo_723,totalSample,immune-InfiltrationScore; Homo_723,totalSample,immune-Bcell; Homo_723,totalSample,immune-Effector_memory; Homo_714,totalSample,immune-InfiltrationScore; Homo_714,totalSample,immune-Th2; Homo_714,totalSample,immune-Effector_memory; Homo_714,totalSample,immune-Bcell; Homo_714,totalSample,immune-Tfh; Homo_633,totalSample,immune-InfiltrationScore; Homo_633,totalSample,immune-Bcell; Homo_633,totalSample,immune-NK; Homo_633,totalSample,immune-Exhausted; MCSA,totalSample,immune-NK;
FOLR2	Homo_723,female,AD.vs.control; Homo_723,male,AD.vs.control; Homo_723,female,AD.vs.MCI; Homo_723,male,AD.vs.MCI; Homo_714,female,AD.vs.control; Homo_714,male,AD.vs.control; Homo_714,female,AD.vs.MCI; Homo_714,male,AD.vs.MCI; Homo_633,female,AD.vs.control; Homo_633,female,AD.vs.MCI; ROSMAP,female,AD.vs.control; Emory,totalSample,age; Emory,control,age; Emory,MCI,age; ROSMAP,AD,MMSE; Emory,totalSample,immune-DC; Emory,totalSample,immune-Tfh; Emory,totalSample,immune-CD8_naive; Homo_723,totalSample,immune-InfiltrationScore; Homo_723,totalSample,immune-Bcell; Homo_723,totalSample,immune-Effector_memory; Homo_723,totalSample,immune-Th1; Homo_723,totalSample,immune-Cytotoxic; Homo_714,totalSample,immune-InfiltrationScore; Homo_714,totalSample,immune-Th1; Homo_714,totalSample,immune-DC; Homo_714,totalSample,immune-Effector_memory; Homo_633,totalSample,immune-DC; Homo_633,totalSample,immune-InfiltrationScore; Homo_633,totalSample,immune-Monocyte; MCSA,totalSample,immune-DC; MCSA,totalSample,immune-Macrophage; MCSA,totalSample,immune-NK; MCSA,totalSample,immune-iTreg; MCSA,totalSample,immune-Th2; MCSA,totalSample,immune-CD8_naive; MCSA,totalSample,immune-InfiltrationScore; ROSMAP,totalSample,immune-Monocyte; ROSMAP,totalSample,immune-CD4_T;
MEI1	Homo_723,female,AD.vs.control; Homo_723,male,AD.vs.control; Homo_723,female,AD.vs.MCI; Homo_723,male,AD.vs.MCI; Homo_714,female,AD.vs.control; Homo_714,male,AD.vs.control; Homo_714,female,AD.vs.MCI; Homo_714,male,AD.vs.MCI; Homo_633,female,AD.vs.control; Homo_633,female,AD.vs.MCI; ROSMAP,male,AD.vs.control; ROSMAP,male,AD.vs.MCI; ROSMAP,control,male.vs.female; MCSA,totalSample,age; MCSA,control,age; ROSMAP,MCI,last.visit.age; Emory,totalSample,immune-Macrophage; Emory,totalSample,immune-Neutrophil; Emory,totalSample,immune-CD4_T; Emory,totalSample,immune-CD8_T; Emory,totalSample,immune-Tr1; Emory,totalSample,immune-iTreg; Emory,totalSample,immune-Th1; Emory,totalSample,immune-Tfh; Emory,totalSample,immune-Exhausted; Emory,totalSample,immune-Effector_memory; Emory,totalSample,immune-InfiltrationScore; Homo_723,totalSample,immune-CD8_T; Homo_723,totalSample,immune-Exhausted; Homo_723,totalSample,immune-Monocyte; Homo_723,totalSample,immune-Cytotoxic; Homo_723,totalSample,immune-iTreg; Homo_723,totalSample,immune-InfiltrationScore; Homo_723,totalSample,immune-MAIT; Homo_723,totalSample,immune-Tfh; Homo_723,totalSample,immune-Macrophage; Homo_723,totalSample,immune-Central_memory; Homo_723,totalSample,immune-Th17; Homo_723,totalSample,immune-nTreg; Homo_723,totalSample,immune-CD4_naive; Homo_723,totalSample,immune-Effector_memory; Homo_714,totalSample,immune-Monocyte; Homo_714,totalSample,immune-Macrophage; Homo_714,totalSample,immune-CD8_T; Homo_714,totalSample,immune-Tfh; Homo_714,totalSample,immune-InfiltrationScore; Homo_714,totalSample,immune-nTreg; Homo_714,totalSample,immune-Effector_memory; Homo_714,totalSample,immune-Exhausted; Homo_714,totalSample,immune-iTreg; Homo_714,totalSample,immune-MAIT; Homo_714,totalSample,immune-Tr1; Homo_714,totalSample,immune-CD4_T; Homo_714,totalSample,immune-Th17; Homo_714,totalSample,immune-Central_memory; Homo_714,totalSample,immune-Cytotoxic; Homo_633,totalSample,immune-Monocyte; Homo_633,totalSample,immune-InfiltrationScore; Homo_633,totalSample,immune-nTreg; Homo_633,totalSample,immune-Neutrophil; Homo_633,totalSample,immune-Macrophage; Homo_633,totalSample,immune-CD4_naive; Homo_633,totalSample,immune-Effector_memory; Homo_633,totalSample,immune-iTreg; Homo_633,totalSample,immune-Tfh; Homo_633,totalSample,immune-DC; Homo_633,totalSample,immune-CD8_T; Homo_633,totalSample,immune-Central_memory; Homo_633,totalSample,immune-MAIT; Homo_633,totalSample,immune-Tr1; MCSA,totalSample,immune-DC; MCSA,totalSample,immune-Monocyte; MCSA,totalSample,immune-Neutrophil; MCSA,totalSample,immune-CD4_T; MCSA,totalSample,immune-CD8_T; MCSA,totalSample,immune-NKT; MCSA,totalSample,immune-CD4_naive; MCSA,totalSample,immune-Tr1; MCSA,totalSample,immune-nTreg; MCSA,totalSample,immune-Th2; MCSA,totalSample,immune-Tfh; MCSA,totalSample,immune-Cytotoxic; [illegible]
LOC107984832	Homo_723,female,AD.vs.control; Homo_723,male,AD.vs.control; Homo_723,female,AD.vs.MCI; Homo_723,male,AD.vs.MCI; Homo_714,female,AD.vs.control; Homo_714,male,AD.vs.control; Homo_714,female,AD.vs.MCI; Homo_714,male,AD.vs.MCI; Homo_633,female,AD.vs.control; Homo_633,female,AD.vs.MCI; Emory,totalSample,immune-Effector_memory; Emory,totalSample,immune-InfiltrationScore; Homo_723,totalSample,immune-Cytotoxic; Homo_723,totalSample,immune-Th1; Homo_723,totalSample,immune-InfiltrationScore; Homo_723,totalSample,immune-Monocyte; Homo_723,totalSample,immune-Bcell; Homo_723,totalSample,immune-Effector_memory; Homo_714,totalSample,immune-Th1; Homo_714,totalSample,immune-Th2; Homo_714,totalSample,immune-Cytotoxic; Homo_714,totalSample,immune-NKT; Homo_633,totalSample,immune-Th1; Homo_633,totalSample,immune-InfiltrationScore; Homo_633,totalSample,immune-Monocyte; Homo_633,totalSample,immune-DC; MCSA,totalSample,immune-DC; MCSA,totalSample,immune-Monocyte; MCSA,totalSample,immune-CD8_T; MCSA,totalSample,immune-iTreg; MCSA,totalSample,immune-Th2; MCSA,totalSample,immune-Cytotoxic; MCSA,totalSample,immune-InfiltrationScore
CD8B	Homo_723,female,AD.vs.control; Homo_723,male,AD.vs.control; Homo_723,female,AD.vs.MCI; Homo_723,male,AD.vs.MCI; Homo_714,female,AD.vs.control; Homo_714,male,AD.vs.control; Homo_714,female,AD.vs.MCI; Homo_714,male,AD.vs.MCI; Homo_633,female,AD.vs.control; Homo_633,female,AD.vs.MCI; ROSMAP,control,male.vs.female; Emory,control,age; Emory,totalSample,immune-Macrophage; Emory,totalSample,immune-Neutrophil; Emory,totalSample,immune-CD4_T; Emory,totalSample,immune-CD8_T; Emory,totalSample,immune-Gamma_delta; Emory,totalSample,immune-Tr1; Emory,totalSample,immune-iTreg; Emory,totalSample,immune-Tfh; Emory,totalSample,immune-CD8_naive; Emory,totalSample,immune-Cytotoxic; Emory,totalSample,immune-Exhausted; Emory,totalSample,immune-MAIT; Emory,totalSample,immune-Effector_memory; Emory,totalSample,immune-InfiltrationScore; Homo_723,totalSample,immune-MAIT; Homo_723,totalSample,immune-CD8_T; Homo_723,totalSample,immune-Cytotoxic; Homo_723,totalSample,immune-Tfh; Homo_723,totalSample,immune-InfiltrationScore; Homo_723,totalSample,immune-Effector_memory; Homo_723,totalSample,immune-Macrophage; Homo_723,totalSample,immune-Exhausted; Homo_723,totalSample,immune-Bcell; Homo_723,totalSample,immune-iTreg; Homo_723,totalSample,immune-CD4_naive; Homo_723,totalSample,immune-CD4_T; Homo_723,totalSample,immune-Th17; Homo_723,totalSample,immune-Th2; Homo_714,totalSample,immune-CD8_T; Homo_714,totalSample,immune-MAIT; Homo_714,totalSample,immune-Cytotoxic; Homo_714,totalSample,immune-InfiltrationScore; Homo_714,totalSample,immune-Effector_memory; Homo_714,totalSample,immune-Exhausted; Homo_714,totalSample,immune-Macrophage; Homo_714,totalSample,immune-Tfh; Homo_714,totalSample,immune-Neutrophil; Homo_714,totalSample,immune-CD8_naive; Homo_714,totalSample,immune-Bcell; Homo_714,totalSample,immune-CD4_T; Homo_714,totalSample,immune-Th17; Homo_714,totalSample,immune-Th2; Homo_714,totalSample,immune-CD4_naive; Homo_714,totalSample,immune-Central_memory; Homo_633,totalSample,immune-InfiltrationScore; Homo_633,totalSample,immune-CD8_T; Homo_633,totalSample,immune-MAIT; Homo_633,totalSample,immune-Cytotoxic; Homo_633,totalSample,immune-Effector_memory; Homo_633,totalSample,immune-Neutrophil; Homo_633,totalSample,immune-Macrophage; Homo_633,totalSample,immune-Bcell; Homo_633,totalSample,immune-Tfh; Homo_633,totalSample,immune-CD4_naive; Homo_633,totalSample,immune-Gamma_delta; Homo_633,totalSample,immune-Exhausted; Homo_633,totalSample,immune-Central_memory; Homo_633,totalSample,immune-CD8_naive; MCSA,totalSample,immune-Monocyte; MCSA,totalSample,immune-Macrophage; MCSA,totalSample,immune-Neutrophil; MCSA,totalSample,immune-CD4_T; MCSA,totalSample,immune-CD8_T; MCSA,totalSample,immune-NKT; MCSA,totalSample,immune-Gamma_delta; MCSA,totalSample,immune-Tfh; MCSA,totalSample,immune-CD8_naive; MCSA,totalSample,immune-Cytotoxic; MCSA,totalSample,immune-MAIT; MCSA,totalSample,immune-Central_memory; MCSA,totalSample,immune-Effector_memory; MCSA,totalSample,immune-InfiltrationScore; ROSMAP,totalSample,immune-Cytotoxic; ROSMAP,totalSample,immune-Exhausted; ROSMAP,totalSample,immune-MAIT; ROSMAP,totalSample,immune-InfiltrationScore; SRP223445,totalSample,immune-CD8_T; SRP223445,totalSample,immune-CD4_naive; [illegible]
OTULINL	Homo_723,female,AD.vs.control; Homo_723,male,AD.vs.control; Homo_723,female,AD.vs.MCI; Homo_723,male,AD.vs.MCI; Homo_714,female,AD.vs.control; Homo_714,male,AD.vs.control; Homo_714,female,AD.vs.MCI; Homo_714,male,AD.vs.MCI; Homo_633,female,AD.vs.control; Homo_633,female,AD.vs.MCI; ROSMAP,female,AD.vs.control; ROSMAP,female,MCI.vs.control; MCSA,MCI,age; Emory,totalSample,immune-DC; Emory,totalSample,immune-Monocyte; Emory,totalSample,immune-Gamma_delta; Emory,totalSample,immune-Tr1; Emory,totalSample,immune-iTreg; Emory,totalSample,immune-Effector_memory; Homo_723,totalSample,immune-Monocyte; Homo_723,totalSample,immune-DC; Homo_723,totalSample,immune-Th2; Homo_723,totalSample,immune-Tfh; Homo_723,totalSample,immune-iTreg; Homo_723,totalSample,immune-Gamma_delta; Homo_723,totalSample,immune-Bcell; Homo_723,totalSample,immune-Tr1; Homo_723,totalSample,immune-InfiltrationScore; Homo_723,totalSample,immune-Effector_memory; Homo_723,totalSample,immune-CD4_T; Homo_714,totalSample,immune-Th2; Homo_714,totalSample,immune-Monocyte; Homo_714,totalSample,immune-Tfh; Homo_714,totalSample,immune-Tr1; Homo_714,totalSample,immune-Gamma_delta; Homo_714,totalSample,immune-Effector_memory; Homo_714,totalSample,immune-CD4_T; Homo_714,totalSample,immune-DC; Homo_633,totalSample,immune-Monocyte; Homo_633,totalSample,immune-Th2; Homo_633,totalSample,immune-Exhausted; Homo_633,totalSample,immune-Tfh; Homo_633,totalSample,immune-Gamma_delta; Homo_633,totalSample,immune-Bcell; Homo_633,totalSample,immune-CD4_T; Homo_633,totalSample,immune-Effector_memory; Homo_633,totalSample,immune-Central_memory; Homo_633,totalSample,immune-InfiltrationScore; Homo_633,totalSample,immune-Th17; Homo_633,totalSample,immune-CD4_naive; Homo_633,totalSample,immune-DC; MCSA,totalSample,immune-DC; MCSA,totalSample,immune-Monocyte; MCSA,totalSample,immune-CD4_T; MCSA,totalSample,immune-CD8_T; MCSA,totalSample,immune-Tr1; MCSA,totalSample,immune-iTreg; MCSA,totalSample,immune-CD8_naive; MCSA,totalSample,immune-Cytotoxic; MCSA,totalSample,immune-Exhausted; MCSA,totalSample,immune-MAIT; MCSA,totalSample,immune-Effector_memory; MCSA,totalSample,immune-InfiltrationScore; ROSMAP,totalSample,immune-Th17; [illegible]
SLC37A2	Homo_723,female,AD.vs.control; Homo_723,male,AD.vs.control; Homo_723,female,AD.vs.MCI; Homo_723,male,AD.vs.MCI; Homo_714,female,AD.vs.control; Homo_714,male,AD.vs.control; Homo_714,female,AD.vs.MCI; Homo_714,male,AD.vs.MCI; Homo_633,female,AD.vs.control; Homo_633,female,AD.vs.MCI; ROSMAP,female,AD.vs.control; Emory,MCI,age; MCSA,totalSample,age; MCSA,control,age; ROSMAP,AD,last.visit.age; Emory,totalSample,immune-DC; Emory,totalSample,immune-CD8_T; Emory,totalSample,immune-Gamma_delta; Emory,totalSample,immune-Tfh; Emory,totalSample,immune-CD8_naive; Emory,totalSample,immune-InfiltrationScore; Homo_723,totalSample,immune-InfiltrationScore; Homo_723,totalSample,immune-Gamma_delta; Homo_723,totalSample,immune-Bcell; Homo_723,totalSample,immune-Effector_memory; Homo_723,totalSample,immune-iTreg; Homo_714,totalSample,immune-InfiltrationScore; Homo_714,totalSample,immune-Effector_memory; Homo_714,totalSample,immune-Gamma_delta; Homo_633,totalSample,immune-InfiltrationScore; Homo_633,totalSample,immune-DC; Homo_633,totalSample,immune-Exhausted; Homo_633,totalSample,immune-Gamma_delta; Homo_633,totalSample,immune-Effector_memory; MCSA,totalSample,immune-DC; MCSA,totalSample,immune-Monocyte; MCSA,totalSample,immune-Macrophage; MCSA,totalSample,immune-NKT; MCSA,totalSample,immune-Gamma_delta; MCSA,totalSample,immune-Th2; MCSA,totalSample,immune-Th17; MCSA,totalSample,immune-Exhausted; MCSA,totalSample,immune-InfiltrationScore; ROSMAP,totalSample,immune-Effector_memory; SRP223445,totalSample,immune-Neutrophil; SRP223445,totalSample,immune-InfiltrationScore
C5orf58	Homo_723,female,AD.vs.control; Homo_723,male,AD.vs.control; Homo_723,female,AD.vs.MCI; Homo_723,male,AD.vs.MCI; Homo_714,female,AD.vs.control; Homo_714,male,AD.vs.control; Homo_714,female,AD.vs.MCI; Homo_714,male,AD.vs.MCI; Homo_633,female,AD.vs.control; Homo_633,female,AD.vs.MCI; ACOM,AD,age; MCSA,MCI,age; Emory,totalSample,immune-Gamma_delta; Emory,totalSample,immune-Tr1; Emory,totalSample,immune-iTreg; Emory,totalSample,immune-Th17; Homo_723,totalSample,immune-iTreg; Homo_723,totalSample,immune-Tfh; Homo_723,totalSample,immune-Th2; Homo_723,totalSample,immune-Gamma_delta; Homo_723,totalSample,immune-Tr1; Homo_723,totalSample,immune-CD4_T; Homo_723,totalSample,immune-DC; Homo_723,totalSample,immune-CD4_naive; Homo_723,totalSample,immune-InfiltrationScore; Homo_723,totalSample,immune-Central_memory; Homo_723,totalSample,immune-CD8_naive; Homo_723,totalSample,immune-Neutrophil; Homo_714,totalSample,immune-Tfh; Homo_714,totalSample,immune-Th2; Homo_714,totalSample,immune-CD4_T; Homo_714,totalSample,immune-iTreg; Homo_714,totalSample,immune-Tr1; Homo_714,totalSample,immune-Gamma_delta; Homo_714,totalSample,immune-CD4_naive; Homo_714,totalSample,immune-Effector_memory; Homo_714,totalSample,immune-Central_memory; Homo_714,totalSample,immune-DC; Homo_633,totalSample,immune-Tfh; Homo_633,totalSample,immune-CD4_T; Homo_633,totalSample,immune-NKT; Homo_633,totalSample,immune-CD4_naive; Homo_633,totalSample,immune-InfiltrationScore; Homo_633,totalSample,immune-Tr1; Homo_633,totalSample,immune-iTreg; Homo_633,totalSample,immune-Th17; Homo_633,totalSample,immune-Gamma_delta; Homo_633,totalSample,immune-Th2; Homo_633,totalSample,immune-Central_memory; Homo_633,totalSample,immune-Effector_memory; Homo_633,totalSample,immune-Exhausted; Homo_633,totalSample,immune-DC; Homo_633,totalSample,immune-Cytotoxic; MCSA,totalSample,immune-DC; MCSA,totalSample,immune-CD4_T; MCSA,totalSample,immune-CD8_T; MCSA,totalSample,immune-Gamma_delta; MCSA,totalSample,immune-CD4_naive; MCSA,totalSample,immune-Tr1; MCSA,totalSample,immune-nTreg; MCSA,totalSample,immune-iTreg; MCSA,totalSample,immune-Th17; MCSA,totalSample,immune-Tfh; MCSA,totalSample,immune-CD8_naive; MCSA,totalSample,immune- [illegible]
SMIM35	Homo_723,female,AD.vs.control; Homo_723,male,AD.vs.control; Homo_723,female,AD.vs.MCI; Homo_723,male,AD.vs.MCI; Homo_714,female,AD.vs.control; Homo_714,male,AD.vs.control; Homo_714,female,AD.vs.MCI; Homo_714,male,AD.vs.MCI; Homo_633,female,AD.vs.control; Homo_633,female,AD.vs.MCI; ROSMAP,female,AD.vs.control; ROSMAP,female,AD.vs.MCI; Emory,totalSample,immune-Tr1; Emory,totalSample,immune-InfiltrationScore; Homo_723,totalSample,immune-Th2; Homo_723,totalSample,immune-Tfh; Homo_723,totalSample,immune-iTreg; Homo_723,totalSample,immune-Effector_memory; Homo_723,totalSample,immune-InfiltrationScore; Homo_723,totalSample,immune-Bcell; Homo_723,totalSample,immune-Macrophage; Homo_723,totalSample,immune-CD4_T; Homo_723,totalSample,immune-DC; Homo_723,totalSample,immune-Tr1; Homo_714,totalSample,immune-Th2; Homo_714,totalSample,immune-Tfh; Homo_714,totalSample,immune-Effector_memory; Homo_714,totalSample,immune-InfiltrationScore; Homo_714,totalSample,immune-CD4_T; Homo_714,totalSample,immune-Macrophage; Homo_714,totalSample,immune-Tr1; Homo_633,totalSample,immune-InfiltrationScore; Homo_633,totalSample,immune-Th2; Homo_633,totalSample,immune-Effector_memory; Homo_633,totalSample,immune-Tfh; Homo_633,totalSample,immune-CD4_T; Homo_633,totalSample,immune-Bcell; Homo_633,totalSample,immune-CD4_naive; MCSA,totalSample,immune-Macrophage; MCSA,totalSample,immune-CD4_T; MCSA,totalSample,immune-CD4_naive; MCSA,totalSample,immune-Tr1; MCSA,totalSample,immune-nTreg; MCSA,totalSample,immune-iTreg; MCSA,totalSample,immune-Th17; MCSA,totalSample,immune-Tfh; MCSA,totalSample,immune-MAIT;
CD101	Homo_723,female,AD.vs.control; Homo_723,male,AD.vs.control; Homo_723,female,AD.vs.MCI; Homo_723,male,AD.vs.MCI; Homo_714,female,AD.vs.control; Homo_714,male,AD.vs.control; Homo_714,female,AD.vs.MCI; Homo_714,male,AD.vs.MCI; Homo_633,female,AD.vs.control; Homo_633,female,AD.vs.MCI; Emory,MCI,age; Emory,totalSample,immune-DC; Emory,totalSample,immune-CD8_naive; Emory,totalSample,immune-InfiltrationScore; Homo_723,totalSample,immune-Th2; Homo_723,totalSample,immune-Tfh; Homo_723,totalSample,immune-iTreg; Homo_723,totalSample,immune-Effector_memory; Homo_723,totalSample,immune-InfiltrationScore; Homo_723,totalSample,immune-Gamma_delta; Homo_723,totalSample,immune-Bcell; Homo_723,totalSample,immune-CD4_T; Homo_723,totalSample,immune-Tr1; Homo_723,totalSample,immune-CD4_naive; Homo_714,totalSample,immune-Th2; Homo_714,totalSample,immune-Tfh; Homo_714,totalSample,immune-Effector_memory; Homo_714,totalSample,immune-CD4_T; Homo_714,totalSample,immune-Tr1; Homo_714,totalSample,immune-InfiltrationScore; Homo_714,totalSample,immune-Gamma_delta; Homo_714,totalSample,immune-iTreg; Homo_714,totalSample,immune-Central_memory; Homo_633,totalSample,immune-Th2; Homo_633,totalSample,immune-Effector_memory; Homo_633,totalSample,immune-Tfh; Homo_633,totalSample,immune-InfiltrationScore; Homo_633,totalSample,immune-CD4_T; Homo_633,totalSample,immune-Gamma_delta; Homo_633,totalSample,immune-Exhausted; Homo_633,totalSample,immune-Central_memory; Homo_633,totalSample,immune-CD4_naive; Homo_633,totalSample,immune-Th17; Homo_633,totalSample,immune-nTreg; MCSA,totalSample,immune-Monocyte; MCSA,totalSample,immune-CD4_T; MCSA,totalSample,immune-Gamma_delta; MCSA,totalSample,immune-CD4_naive; MCSA,totalSample,immune-Tr1; MCSA,totalSample,immune-nTreg; MCSA,totalSample,immune-iTreg; MCSA,totalSample,immune-Th17; MCSA,totalSample,immune-Tfh; MCSA,totalSample,immune-Exhausted; MCSA,totalSample,immune-
SDHD	Homo_723,female,AD.vs.control; Homo_723,male,AD.vs.control; Homo_723,female,AD.vs.MCI; Homo_723,male,AD.vs.MCI; Homo_714,female,AD.vs.control; Homo_714,male,AD.vs.control; Homo_714,female,AD.vs.MCI; Homo_714,male,AD.vs.MCI; Homo_633,female,AD.vs.control; Homo_633,female,AD.vs.MCI; ROSMAP,female,AD.vs.control; ROSMAP,female,AD.vs.MCI; MCSA,MCI,age; Emory,totalSample,immune-DC; Emory,totalSample,immune-Monocyte; Emory,totalSample,immune-CD4_T; Emory,totalSample,immune-Gamma_delta; Emory,totalSample,immune-Tr1; Emory,totalSample,immune-iTreg; Emory,totalSample,immune-Effector_memory; Homo_723,totalSample,immune-Th2; Homo_723,totalSample,immune-Tfh; Homo_723,totalSample,immune-iTreg; Homo_723,totalSample,immune-DC; Homo_723,totalSample,immune-Monocyte; Homo_723,totalSample,immune-Tr1; Homo_723,totalSample,immune-Effector_memory; Homo_723,totalSample,immune-InfiltrationScore; Homo_723,totalSample,immune-CD4_T; Homo_723,totalSample,immune-Gamma_delta; Homo_723,totalSample,immune-CD4_naive; Homo_723,totalSample,immune-Bcell; Homo_723,totalSample,immune-MAIT; Homo_714,totalSample,immune-Th2; Homo_714,totalSample,immune-Tfh; Homo_714,totalSample,immune-Tr1; Homo_714,totalSample,immune-Effector_memory; Homo_714,totalSample,immune-CD4_T; Homo_714,totalSample,immune-Monocyte; Homo_714,totalSample,immune-Gamma_delta; Homo_714,totalSample,immune-Central_memory; Homo_714,totalSample,immune-iTreg; Homo_714,totalSample,immune-DC; Homo_714,totalSample,immune-CD4_naive; Homo_714,totalSample,immune-InfiltrationScore; Homo_633,totalSample,immune-Tfh; Homo_633,totalSample,immune-Th2; Homo_633,totalSample,immune-Monocyte; Homo_633,totalSample,immune-CD4_T; Homo_633,totalSample,immune-Effector_memory; Homo_633,totalSample,immune-Exhausted; Homo_633,totalSample,immune-InfiltrationScore; Homo_633,totalSample,immune-Central_memory; Homo_633,totalSample,immune-CD4_naive; Homo_633,totalSample,immune-Gamma_delta; Homo_633,totalSample,immune-Th17; Homo_633,totalSample,immune-Tr1; Homo_633,totalSample,immune-NKT; Homo_633,totalSample,immune-DC; Homo_633,totalSample,immune-Bcell; MCSA,totalSample,immune-DC; MCSA,totalSample,immune-Monocyte; MCSA,totalSample,immune-Macrophage; MCSA,totalSample,immune-CD4_T; MCSA,totalSample,immune-CD8_T; MCSA,totalSample,immune-CD4_naive; MCSA,totalSample,immune-Tr1; MCSA,totalSample,immune-nTreg; MCSA,totalSample,immune-iTreg; MCSA,totalSample,immune-Th17; MCSA,totalSample,immune-Tfh; MCSA,totalSample,immune-CD8_naive; MCSA,totalSample,immune-Cytotoxic; MCSA,totalSample,immune-Exhausted; MCSA,totalSample,immune-MAIT; MCSA,totalSample,immune-Central_memory; ROSMAP,totalSample,immune-Effector_memory; SRP223445,totalSample,immune-NK; SRP223445,totalSample,immune-CD4_naive; SRP223445,totalSample,immune-Th17; SRP223445,totalSample,immune-Exhausted; [illegible]
EDEM2	Homo_723,female,AD.vs.control; Homo_723,male,AD.vs.control; Homo_723,female,AD.vs.MCI; Homo_723,male,AD.vs.MCI; Homo_714,female,AD.vs.control; Homo_714,male,AD.vs.control; Homo_714,female,AD.vs.MCI; Homo_714,male,AD.vs.MCI; Homo_633,female,AD.vs.control; Homo_633,female,AD.vs.MCI; ROSMAP,female,AD.vs.control; ROSMAP,female,AD.vs.MCI; Emory,MCI,age; MCSA,totalSample,age; MCSA,control,age; ROSMAP,MCI,last.visit.age; ROSMAP,MCI,sample.age; Emory,totalSample,immune-Monocyte; Emory,totalSample,immune-Neutrophil; Emory,totalSample,immune-CD8_T; Emory,totalSample,immune-Gamma_delta; Emory,totalSample,immune-Tfh; Emory,totalSample,immune-CD8_naive; Emory,totalSample,immune-Exhausted; Homo_723,totalSample,immune-InfiltrationScore; Homo_723,totalSample,immune-Effector_memory; Homo_723,totalSample,immune-Bcell; Homo_723,totalSample,immune-Th2; Homo_723,totalSample,immune-Monocyte; Homo_723,totalSample,immune-Th1; Homo_723,totalSample,immune-Tfh; Homo_723,totalSample,immune-Gamma_delta; Homo_723,totalSample,immune-Neutrophil; Homo_714,totalSample,immune-Th2; Homo_714,totalSample,immune-InfiltrationScore; Homo_714,totalSample,immune-Th1; Homo_714,totalSample,immune-Effector_memory; Homo_714,totalSample,immune-Tfh; Homo_714,totalSample,immune-Gamma_delta; Homo_633,totalSample,immune-InfiltrationScore; Homo_633,totalSample,immune-Exhausted; Homo_633,totalSample,immune-Th2; Homo_633,totalSample,immune-Monocyte; Homo_633,totalSample,immune-Gamma_delta; MCSA,totalSample,immune-DC; MCSA,totalSample,immune-Bcell; MCSA,totalSample,immune-Monocyte; MCSA,totalSample,immune-Macrophage;

SLC46A3	Homo_723,female,AD.vs.control; Homo_723,male,AD.vs.control; Homo_723,female,AD.vs.MCI; Homo_723,male,AD.vs.MCI; Homo_714,female,AD.vs.control; Homo_714,male,AD.vs.control; Homo_714,female,AD.vs.MCI; Homo_714,male,AD.vs.MCI; Homo_633,female,AD.vs.control; Homo_633,female,AD.vs.MCI; MCSA,MCI,age; Emory,totalSample,immune-Macrophage; Emory,totalSample,immune-CD4_T; Emory,totalSample,immune-Tr1; Emory,totalSample,immune-iTreg; Emory,totalSample,immune-Central_memory; Emory,totalSample,immune-InfiltrationScore; Homo_723,totalSample,immune-Th2; Homo_723,totalSample,immune-Tfh; Homo_723,totalSample,immune-iTreg; Homo_723,totalSample,immune-Tr1; Homo_723,totalSample,immune-DC; Homo_723,totalSample,immune-CD4_T; Homo_723,totalSample,immune-CD4_naive; Homo_723,totalSample,immune-Effector_memory; Homo_723,totalSample,immune-InfiltrationScore; Homo_723,totalSample,immune-Gamma_delta; Homo_723,totalSample,immune-Central_memory; Homo_723,totalSample,immune-Monocyte; Homo_723,totalSample,immune-Bcell; Homo_723,totalSample,immune-Macrophage; Homo_714,totalSample,immune-Th2; Homo_714,totalSample,immune-Tfh; Homo_714,totalSample,immune-CD4_T; Homo_714,totalSample,immune-Tr1; Homo_714,totalSample,immune-Effector_memory; Homo_714,totalSample,immune-iTreg; Homo_714,totalSample,immune-Central_memory; Homo_714,totalSample,immune-CD4_naive; Homo_714,totalSample,immune-DC; Homo_714,totalSample,immune-Gamma_delta; Homo_714,totalSample,immune-InfiltrationScore; Homo_714,totalSample,immune-nTreg; Homo_633,totalSample,immune-Tfh; Homo_633,totalSample,immune-Th2; Homo_633,totalSample,immune-CD4_T; Homo_633,totalSample,immune-CD4_naive; Homo_633,totalSample,immune-Effector_memory; Homo_633,totalSample,immune-Central_memory; Homo_633,totalSample,immune-Tr1; Homo_633,totalSample,immune-InfiltrationScore; Homo_633,totalSample,immune-Th17; Homo_633,totalSample,immune-NKT; Homo_633,totalSample,immune-Exhausted; Homo_633,totalSample,immune-iTreg; Homo_633,totalSample,immune-Monocyte; Homo_633,totalSample,immune-Gamma_delta; Homo_633,totalSample,immune-DC; Homo_633,totalSample,immune-Bcell; Homo_633,totalSample,immune-nTreg; MCSA,totalSample,immune-DC; MCSA,totalSample,immune-Macrophage; MCSA,totalSample,immune-CD4_T; MCSA,totalSample,immune-CD8_T; MCSA,totalSample,immune-Gamma_delta; MCSA,totalSample,immune-CD4_naive; MCSA,totalSample,immune-Tr1; MCSA,totalSample,immune-nTreg; MCSA,totalSample,immune-iTreg; MCSA,totalSample,immune-Th17; MCSA,totalSample,immune-Tfh; MCSA,totalSample,immune-CD8_naive; MCSA,totalSample,immune-Cytotoxic; MCSA,totalSample,immune-Exhausted; MCSA,totalSample,immune-MAIT; MCSA,totalSample,immune-Central_memory; ROSMAP,totalSample,immune-Th17; ROSMAP,totalSample,immune-Cytotoxic; ROSMAP,totalSample,immune-Effector_memory; ROSMAP,totalSample,immune-InfiltrationScore
C10orf143	Homo_723,female,AD.vs.control; Homo_723,male,AD.vs.control; Homo_723,female,AD.vs.MCI; Homo_723,male,AD.vs.MCI; Homo_714,female,AD.vs.control; Homo_714,male,AD.vs.control; Homo_714,female,AD.vs.MCI; Homo_714,male,AD.vs.MCI; Homo_633,female,AD.vs.control; Homo_633,female,AD.vs.MCI; ROSMAP,control,male.vs.female; Emory,totalSample,age; Emory,control,age; ROSMAP,MCI,last.visit.age; Emory,totalSample,immune-Macrophage; Emory,totalSample,immune-Neutrophil; Emory,totalSample,immune-CD4_T; Emory,totalSample,immune-CD8_T; Emory,totalSample,immune-Tr1; Emory,totalSample,immune-iTreg; Emory,totalSample,immune-Tfh; Emory,totalSample,immune-CD8_naive; Emory,totalSample,immune-Exhausted; Emory,totalSample,immune-Effector_memory; Emory,totalSample,immune-InfiltrationScore; Homo_723,totalSample,immune-Tfh; Homo_723,totalSample,immune-iTreg; Homo_723,totalSample,immune-CD8_T; Homo_723,totalSample,immune-Macrophage; Homo_723,totalSample,immune-Tr1; Homo_723,totalSample,immune-Th2; Homo_723,totalSample,immune-CD4_T; Homo_723,totalSample,immune-nTreg; Homo_723,totalSample,immune-CD4_naive; Homo_723,totalSample,immune-Central_memory; Homo_723,totalSample,immune-MAIT; Homo_723,totalSample,immune-Effector_memory; Homo_723,totalSample,immune-InfiltrationScore; Homo_723,totalSample,immune-Exhausted; Homo_723,totalSample,immune-Cytotoxic; Homo_714,totalSample,immune-Tfh; Homo_714,totalSample,immune-Effector_memory; Homo_714,totalSample,immune-CD4_T; Homo_714,totalSample,immune-Th2; Homo_714,totalSample,immune-Tr1; Homo_714,totalSample,immune-nTreg; Homo_714,totalSample,immune-CD8_T; Homo_714,totalSample,immune-Macrophage; Homo_714,totalSample,immune-Central_memory; Homo_714,totalSample,immune-iTreg; Homo_714,totalSample,immune-MAIT; Homo_714,totalSample,immune-CD4_naive; Homo_714,totalSample,immune-InfiltrationScore; Homo_714,totalSample,immune-Neutrophil; Homo_714,totalSample,immune-Exhausted; Homo_714,totalSample,immune-CD8_naive; Homo_633,totalSample,immune-Tfh; Homo_633,totalSample,immune-nTreg; Homo_633,totalSample,immune-Neutrophil; Homo_633,totalSample,immune-InfiltrationScore; Homo_633,totalSample,immune-Effector_memory; Homo_633,totalSample,immune-CD4_naive; Homo_633,totalSample,immune-Central_memory; Homo_633,totalSample,immune-CD4_T; Homo_633,totalSample,immune-Tr1; Homo_633,totalSample,immune-Macrophage; Homo_633,totalSample,immune-iTreg; Homo_633,totalSample,immune-MAIT; Homo_633,totalSample,immune-CD8_T; MCSA,totalSample,immune-Monocyte; MCSA,totalSample,immune-Macrophage; MCSA,totalSample,immune-Neutrophil; MCSA,totalSample,immune-CD4_T; MCSA,totalSample,immune-CD8_T; MCSA,totalSample,immune-NKT; MCSA,totalSample,immune-CD4_naive; MCSA,totalSample,immune-Tr1; MCSA,totalSample,immune-nTreg; MCSA,totalSample,immune-iTreg; MCSA,totalSample,immune-Tfh; MCSA,totalSample,immune-CD8_naive; MCSA,totalSample,immune-Cytotoxic; MCSA,totalSample,immune-MAIT; [illegible]
DPEP3	Homo_723,female,AD.vs.control; Homo_723,male,AD.vs.control; Homo_723,female,AD.vs.MCI; Homo_723,male,AD.vs.MCI; Homo_714,female,AD.vs.control; Homo_714,male,AD.vs.control; Homo_714,female,AD.vs.MCI; Homo_714,male,AD.vs.MCI; Homo_633,female,AD.vs.control; Homo_633,female,AD.vs.MCI; ROSMAP,female,AD.vs.MCI; ROSMAP,control,male.vs.female; ROSMAP,MCI,male.vs.female; Emory,MCI,age; MCSA,totalSample,age; MCSA,control,age; MCSA,MCI,age; Emory,totalSample,immune-Neutrophil; Emory,totalSample,immune-CD8_T; Emory,totalSample,immune-Gamma_delta; Emory,totalSample,immune-Th1; Emory,totalSample,immune-Th17; Emory,totalSample,immune-Tfh; Emory,totalSample,immune-CD8_naive; Emory,totalSample,immune-Cytotoxic; Emory,totalSample,immune-Exhausted; Homo_723,totalSample,immune-Th1; Homo_723,totalSample,immune-Neutrophil; Homo_723,totalSample,immune-Bcell; Homo_723,totalSample,immune-Cytotoxic; Homo_714,totalSample,immune-Th1; Homo_714,totalSample,immune-Neutrophil; Homo_714,totalSample,immune-Th2; Homo_714,totalSample,immune-NKT; Homo_633,totalSample,immune-Th1; Homo_633,totalSample,immune-Exhausted; Homo_633,totalSample,immune-Neutrophil; Homo_633,totalSample,immune-Th2; Homo_633,totalSample,immune-MAIT; Homo_633,totalSample,immune-Th17; Homo_633,totalSample,immune-NKT; MCSA,totalSample,immune-DC; MCSA,totalSample,immune-Monocyte; MCSA,totalSample,immune-Macrophage; MCSA,totalSample,immune-Neutrophil; MCSA,totalSample,immune-Gamma_delta; MCSA,totalSample,immune-iTreg; MCSA,totalSample,immune-Th1; MCSA,totalSample,immune-Th2; MCSA,totalSample,immune-Th17; MCSA,totalSample,immune- [illegible]
KY	Homo_723,female,AD.vs.control; Homo_723,male,AD.vs.control; Homo_723,female,AD.vs.MCI; Homo_723,male,AD.vs.MCI; Homo_714,female,AD.vs.control; Homo_714,male,AD.vs.control; Homo_714,female,AD.vs.MCI; Homo_714,male,AD.vs.MCI; Homo_633,female,AD.vs.control; Homo_633,female,AD.vs.MCI; ROSMAP,MCI,last.visit.age; Emory,totalSample,immune-Neutrophil; Emory,totalSample,immune-CD8_T; Emory,totalSample,immune-Gamma_delta; Emory,totalSample,immune-Th1; Emory,totalSample,immune-Th17; Emory,totalSample,immune-Tfh; Emory,totalSample,immune-CD8_naive; Emory,totalSample,immune-Cytotoxic; Emory,totalSample,immune-Exhausted; Homo_723,totalSample,immune-Neutrophil; Homo_723,totalSample,immune-Gamma_delta; Homo_723,totalSample,immune-CD8_naive; Homo_723,totalSample,immune-Bcell; Homo_714,totalSample,immune-Neutrophil; Homo_714,totalSample,immune-Gamma_delta; Homo_714,totalSample,immune-Th2; Homo_633,totalSample,immune-Neutrophil; Homo_633,totalSample,immune-Exhausted; Homo_633,totalSample,immune-CD8_T; Homo_633,totalSample,immune-Gamma_delta; Homo_633,totalSample,immune-Th17; Homo_633,totalSample,immune-Th2; Homo_633,totalSample,immune-MAIT; Homo_633,totalSample,immune-Cytotoxic; MCSA,totalSample,immune-Neutrophil; MCSA,totalSample,immune-CD8_T; MCSA,totalSample,immune-Gamma_delta; MCSA,totalSample,immune-Th17; MCSA,totalSample,immune-Cytotoxic; MCSA,totalSample,immune-Exhausted; ROSMAP,totalSample,immune-InfiltrationScore
SLC8B1	Homo_723,female,AD.vs.control; Homo_723,male,AD.vs.control; Homo_723,female,AD.vs.MCI; Homo_723,male,AD.vs.MCI; Homo_714,female,AD.vs.control; Homo_714,male,AD.vs.control; Homo_714,female,AD.vs.MCI; Homo_714,male,AD.vs.MCI; Homo_633,female,AD.vs.control; Homo_633,female,AD.vs.MCI; MCSA,totalSample,age; MCSA,control,age; MCSA,MCI,age; Emory,totalSample,immune-CD4_T; Emory,totalSample,immune-Gamma_delta; Emory,totalSample,immune-CD4_naive; Emory,totalSample,immune-Tr1; Emory,totalSample,immune-iTreg; Emory,totalSample,immune-Th17; Emory,totalSample,immune-Cytotoxic; Emory,totalSample,immune-Exhausted; Emory,totalSample,immune-Central_memory; Homo_723,totalSample,immune-CD8_T; Homo_723,totalSample,immune-Exhausted; Homo_723,totalSample,immune-InfiltrationScore; Homo_723,totalSample,immune-Cytotoxic; Homo_723,totalSample,immune-iTreg; Homo_723,totalSample,immune-Monocyte; Homo_723,totalSample,immune-Central_memory; Homo_723,totalSample,immune-nTreg; Homo_714,totalSample,immune-Monocyte; Homo_714,totalSample,immune-nTreg; Homo_714,totalSample,immune-Tfh; Homo_714,totalSample,immune-InfiltrationScore; Homo_714,totalSample,immune-Effector_memory; Homo_633,totalSample,immune-DC; Homo_633,totalSample,immune-nTreg; Homo_633,totalSample,immune-Monocyte; Homo_633,totalSample,immune-InfiltrationScore; MCSA,totalSample,immune-DC; MCSA,totalSample,immune-Monocyte; MCSA,totalSample,immune-Macrophage; MCSA,totalSample,immune-CD8_T; MCSA,totalSample,immune-Gamma_delta; MCSA,totalSample,immune-Th2; MCSA,totalSample,immune-Th17; MCSA,totalSample,immune-Cytotoxic; MCSA,totalSample,immune-Central_memory; MCSA,totalSample,immune-Effector_memory; MCSA,totalSample,immune-InfiltrationScore; SRP223445,totalSample,immune-Exhausted
TCN1	Homo_723,female,AD.vs.control; Homo_723,male,AD.vs.control; Homo_723,female,AD.vs.MCI; Homo_723,male,AD.vs.MCI; Homo_714,female,AD.vs.control; Homo_714,male,AD.vs.control; Homo_714,female,AD.vs.MCI; Homo_714,male,AD.vs.MCI; Homo_633,female,AD.vs.control; Homo_633,female,AD.vs.MCI; ROSMAP,AD,last.visit.age; Emory,totalSample,immune-Macrophage; Emory,totalSample,immune-CD8_T; Emory,totalSample,immune-Gamma_delta; Emory,totalSample,immune-Th1; Emory,totalSample,immune-Tfh; Emory,totalSample,immune-Exhausted; Homo_723,totalSample,immune-Monocyte; Homo_723,totalSample,immune-DC; Homo_723,totalSample,immune-Neutrophil; Homo_723,totalSample,immune-Gamma_delta; Homo_723,totalSample,immune-Th1; Homo_723,totalSample,immune-Th2; Homo_714,totalSample,immune-Monocyte; Homo_714,totalSample,immune-Th2; Homo_714,totalSample,immune-Th1; Homo_714,totalSample,immune-Gamma_delta; Homo_633,totalSample,immune-Monocyte; Homo_633,totalSample,immune-Macrophage; Homo_633,totalSample,immune-Th2; Homo_633,totalSample,immune-Exhausted; Homo_633,totalSample,immune-Gamma_delta; MCSA,totalSample,immune-DC; MCSA,totalSample,immune-Macrophage; MCSA,totalSample,immune-Neutrophil; MCSA,totalSample,immune-CD8_T; MCSA,totalSample,immune-CD4_naive; MCSA,totalSample,immune-Th1; MCSA,totalSample,immune-Tfh; MCSA,totalSample,immune-Cytotoxic; MCSA,totalSample,immune-Exhausted; MCSA,totalSample,immune-Central_memory; MCSA,totalSample,immune-Effector_memory; MCSA,totalSample,immune-InfiltrationScore; ROSMAP,totalSample,immune-InfiltrationScore; SRP223445,totalSample,immune-CD4_T
CYB561D2	Homo_723,female,AD.vs.control; Homo_723,male,AD.vs.control; Homo_723,female,AD.vs.MCI; Homo_723,male,AD.vs.MCI; Homo_714,female,AD.vs.control; Homo_714,male,AD.vs.control; Homo_714,female,AD.vs.MCI; Homo_714,male,AD.vs.MCI; Homo_633,female,AD.vs.control; Homo_633,female,AD.vs.MCI; ROSMAP,female,AD.vs.control; MCSA,totalSample,age; MCSA,control,age; MCSA,MCI,age; Emory,totalSample,immune-NK; Emory,totalSample,immune-Neutrophil; Emory,totalSample,immune-CD4_T; Emory,totalSample,immune-Tr1; Emory,totalSample,immune-iTreg; Emory,totalSample,immune-Exhausted; Emory,totalSample,immune-Effector_memory; Emory,totalSample,immune-InfiltrationScore; Homo_723,totalSample,immune-CD8_T; Homo_723,totalSample,immune-InfiltrationScore; Homo_723,totalSample,immune-Cytotoxic; Homo_723,totalSample,immune-Exhausted; Homo_723,totalSample,immune-Effector_memory; Homo_723,totalSample,immune-Monocyte; Homo_723,totalSample,immune-iTreg; Homo_723,totalSample,immune-MAIT; Homo_723,totalSample,immune-Tfh; Homo_723,totalSample,immune-Macrophage; Homo_723,totalSample,immune-Th17; Homo_723,totalSample,immune-Bcell; Homo_714,totalSample,immune-InfiltrationScore; Homo_714,totalSample,immune-Effector_memory; Homo_714,totalSample,immune-CD8_T; Homo_714,totalSample,immune-Monocyte; Homo_714,totalSample,immune-Cytotoxic; Homo_714,totalSample,immune-DC; Homo_714,totalSample,immune-Macrophage; Homo_714,totalSample,immune-Tfh; Homo_714,totalSample,immune-nTreg; Homo_633,totalSample,immune-InfiltrationScore; Homo_633,totalSample,immune-DC; Homo_633,totalSample,immune-Effector_memory; Homo_633,totalSample,immune-Monocyte; Homo_633,totalSample,immune-nTreg; Homo_633,totalSample,immune-Neutrophil; Homo_633,totalSample,immune-NK; Homo_633,totalSample,immune-CD8_T; MCSA,totalSample,immune-DC; MCSA,totalSample,immune-Monocyte; MCSA,totalSample,immune-Neutrophil; MCSA,totalSample,immune-CD8_T; MCSA,totalSample,immune-NKT; MCSA,totalSample,immune-Th2; MCSA,totalSample,immune-Cytotoxic; MCSA,totalSample,immune-Central_memory; MCSA,totalSample,immune-Effector_memory; MCSA,totalSample,immune-InfiltrationScore; ROSMAP,totalSample,immune-Effector_memory; SRP223445,totalSample,immune-Exhausted
HLA-DOA	Homo_723,female,AD.vs.control; Homo_723,male,AD.vs.control; Homo_723,female,AD.vs.MCI; Homo_723,male,AD.vs.MCI; Homo_714,female,AD.vs.control; Homo_714,male,AD.vs.control; Homo_714,female,AD.vs.MCI; Homo_714,male,AD.vs.MCI; Homo_633,female,AD.vs.control; Homo_633,female,AD.vs.MCI; Homo_633,female,MCI.vs.control; Homo_633,male,MCI.vs.control; ROSMAP,female,AD.vs.control; ACOM,totalSample,age; MCSA,totalSample,age; MCSA,control,age; Emory,totalSample,immune-B_cell; Emory,totalSample,immune-Macrophage; Emory,totalSample,immune-Neutrophil; Emory,totalSample,immune-CD4_T; Emory,totalSample,immune-Tr1; Emory,totalSample,immune-iTreg; Emory,totalSample,immune-CD8_naive; Emory,totalSample,immune-InfiltrationScore; Homo_723,totalSample,immune-Tfh; Homo_723,totalSample,immune-Th2; Homo_723,totalSample,immune-iTreg; Homo_723,totalSample,immune-Effector_memory; Homo_723,totalSample,immune-InfiltrationScore; Homo_723,totalSample,immune-CD4_T; Homo_723,totalSample,immune-Tr1; Homo_723,totalSample,immune-CD4_naive; Homo_723,totalSample,immune-Macrophage; Homo_723,totalSample,immune-nTreg; Homo_723,totalSample,immune-DC; Homo_723,totalSample,immune-Gamma_delta; Homo_723,totalSample,immune-Central_memory; Homo_723,totalSample,immune-CD8_T; Homo_714,totalSample,immune-Th2; Homo_714,totalSample,immune-Tfh; Homo_714,totalSample,immune-CD4_T; Homo_714,totalSample,immune-Effector_memory; Homo_714,totalSample,immune-Tr1; Homo_714,totalSample,immune-Central_memory; Homo_714,totalSample,immune-nTreg; Homo_714,totalSample,immune-iTreg; Homo_714,totalSample,immune-CD4_naive; Homo_714,totalSample,immune-InfiltrationScore; Homo_714,totalSample,immune-Macrophage; Homo_714,totalSample,immune-Gamma_delta; Homo_633,totalSample,immune-Tfh; Homo_633,totalSample,immune-Th2; Homo_633,totalSample,immune-CD4_T; Homo_633,totalSample,immune-Effector_memory; Homo_633,totalSample,immune-Central_memory; Homo_633,totalSample,immune-nTreg; Homo_633,totalSample,immune-CD4_naive; Homo_633,totalSample,immune-InfiltrationScore; Homo_633,totalSample,immune-Tr1; Homo_633,totalSample,immune-Gamma_delta; Homo_633,totalSample,immune-Neutrophil; Homo_633,totalSample,immune-Th17; MCSA,totalSample,immune-Bcell; MCSA,totalSample,immune-Monocyte; MCSA,totalSample,immune-Macrophage; MCSA,totalSample,immune-Neutrophil; MCSA,totalSample,immune-CD4_T; MCSA,totalSample,immune-Gamma_delta; MCSA,totalSample,immune-CD4_naive; MCSA,totalSample,immune-Tr1; MCSA,totalSample,immune-nTreg; MCSA,totalSample,immune-iTreg; MCSA,totalSample,immune-Th17; MCSA,totalSample,immune-Tfh; MCSA,totalSample,immune-CD8_naive; MCSA,totalSample,immune-Central_memory; MCSA,totalSample,immune-Effector_memory; MCSA,totalSample,immune-InfiltrationScore; SRP223445,totalSample,immune-CD4_naive; SRP223445,totalSample,immune-Th17; SRP223445,totalSample,immune-Exhausted; [illegible]
HAVCR2	Homo_723,female,AD.vs.control; Homo_723,male,AD.vs.control; Homo_723,female,AD.vs.MCI; Homo_723,male,AD.vs.MCI; Homo_714,female,AD.vs.control; Homo_714,male,AD.vs.control; Homo_714,female,AD.vs.MCI; Homo_714,male,AD.vs.MCI; Homo_633,female,AD.vs.control; Homo_633,female,AD.vs.MCI; ROSMAP,female,AD.vs.control; ROSMAP,female,MCI.vs.control; Emory,MCI,age; MCSA,MCI,age; ROSMAP,MCI,sample.age; Emory,totalSample,immune-NK; Emory,totalSample,immune-iTreg; Emory,totalSample,immune-CD8_naive; Emory,totalSample,immune-Effector_memory; Emory,totalSample,immune-InfiltrationScore; Homo_723,totalSample,immune-InfiltrationScore; Homo_723,totalSample,immune-Th2; Homo_723,totalSample,immune-Effector_memory; Homo_723,totalSample,immune-Monocyte; Homo_723,totalSample,immune-Tfh; Homo_723,totalSample,immune-iTreg; Homo_723,totalSample,immune-Bcell; Homo_723,totalSample,immune-DC; Homo_723,totalSample,immune-Tr1; Homo_714,totalSample,immune-Th2; Homo_714,totalSample,immune-Tfh; Homo_714,totalSample,immune-Effector_memory; Homo_714,totalSample,immune-InfiltrationScore; Homo_714,totalSample,immune-Monocyte; Homo_714,totalSample,immune-Tr1; Homo_714,totalSample,immune-CD4_T; Homo_714,totalSample,immune-Bcell; Homo_633,totalSample,immune-Th2; Homo_633,totalSample,immune-Monocyte; Homo_633,totalSample,immune-InfiltrationScore; Homo_633,totalSample,immune-Effector_memory; Homo_633,totalSample,immune-Exhausted; Homo_633,totalSample,immune-Tfh; Homo_633,totalSample,immune-NK; Homo_633,totalSample,immune-Bcell; Homo_633,totalSample,immune-CD4_T; Homo_633,totalSample,immune-Th17; MCSA,totalSample,immune-NK; MCSA,totalSample,immune-CD4_T; MCSA,totalSample,immune-CD8_T; MCSA,totalSample,immune-CD4_naive; MCSA,totalSample,immune-Tr1; MCSA,totalSample,immune-nTreg; MCSA,totalSample,immune-iTreg; MCSA,totalSample,immune-Cytotoxic; MCSA,totalSample,immune-Exhausted; ROSMAP,totalSample,immune- [illegible]
IL2RA	Homo_723,female,AD.vs.control; Homo_723,male,AD.vs.control; Homo_723,female,AD.vs.MCI; Homo_723,male,AD.vs.MCI; Homo_714,female,AD.vs.control; Homo_714,male,AD.vs.control; Homo_714,female,AD.vs.MCI; Homo_714,male,AD.vs.MCI; Homo_633,female,AD.vs.control; Homo_633,female,AD.vs.MCI; ROSMAP,female,AD.vs.control; ROSMAP,female,MCI.vs.control; ROSMAP,control,male.vs.female; ROSMAP,MCI,last.visit.age; ROSMAP,MCI,sample.age; Emory,totalSample,immune-Macrophage; Emory,totalSample,immune-Neutrophil; Emory,totalSample,immune-CD4_T; Emory,totalSample,immune-Gamma_delta; Emory,totalSample,immune-Tr1; Emory,totalSample,immune-nTreg; Emory,totalSample,immune-iTreg; Emory,totalSample,immune-Tfh; Emory,totalSample,immune-Central_memory; Emory,totalSample,immune-InfiltrationScore; Homo_723,totalSample,immune-iTreg; Homo_723,totalSample,immune-Tfh; Homo_723,totalSample,immune-Th2; Homo_723,totalSample,immune-Tr1; Homo_723,totalSample,immune-CD4_T; Homo_723,totalSample,immune-nTreg; Homo_723,totalSample,immune-Central_memory; Homo_723,totalSample,immune-CD4_naive; Homo_723,totalSample,immune-Gamma_delta; Homo_723,totalSample,immune-Effector_memory; Homo_723,totalSample,immune-InfiltrationScore; Homo_723,totalSample,immune-DC; Homo_723,totalSample,immune-Macrophage; Homo_723,totalSample,immune-CD8_T; Homo_714,totalSample,immune-Tfh; Homo_714,totalSample,immune-Th2; Homo_714,totalSample,immune-Tr1; Homo_714,totalSample,immune-CD4_T; Homo_714,totalSample,immune-iTreg; Homo_714,totalSample,immune-nTreg; Homo_714,totalSample,immune-Central_memory; Homo_714,totalSample,immune-CD4_naive; Homo_714,totalSample,immune-Effector_memory; Homo_714,totalSample,immune-Gamma_delta; Homo_714,totalSample,immune-Macrophage; Homo_633,totalSample,immune-Tfh; Homo_633,totalSample,immune-CD4_T; Homo_633,totalSample,immune-Tr1; Homo_633,totalSample,immune-Central_memory; Homo_633,totalSample,immune-nTreg; Homo_633,totalSample,immune-CD4_naive; Homo_633,totalSample,immune-Th2; Homo_633,totalSample,immune-iTreg; Homo_633,totalSample,immune-Gamma_delta; Homo_633,totalSample,immune-Effector_memory; Homo_633,totalSample,immune-Th17; Homo_633,totalSample,immune-Exhausted; Homo_633,totalSample,immune-NKT; Homo_633,totalSample,immune-Cytotoxic; Homo_633,totalSample,immune-Th1; MCSA,totalSample,immune-Bcell; MCSA,totalSample,immune-Macrophage; MCSA,totalSample,immune-Neutrophil; MCSA,totalSample,immune-CD4_T; MCSA,totalSample,immune-Gamma_delta; MCSA,totalSample,immune-CD4_naive; MCSA,totalSample,immune-Tr1; MCSA,totalSample,immune-nTreg; MCSA,totalSample,immune-iTreg; MCSA,totalSample,immune-Th17; MCSA,totalSample,immune-Tfh; MCSA,totalSample,immune-CD8_naive; MCSA,totalSample,immune-Cytotoxic; MCSA,totalSample,immune-Exhausted; MCSA,totalSample,immune-Central_memory; MCSA,totalSample,immune-Effector_memory; [illegible]
ASTE1	Homo_723,female,AD.vs.control; Homo_723,male,AD.vs.control; Homo_723,female,AD.vs.MCI; Homo_723,male,AD.vs.MCI; Homo_714,female,AD.vs.control; Homo_714,male,AD.vs.control; Homo_714,female,AD.vs.MCI; Homo_714,male,AD.vs.MCI; Homo_633,female,AD.vs.control; Homo_633,female,AD.vs.MCI; ROSMAP,female,AD.vs.control; MCSA,MCI,age; Emory,totalSample,immune-Macrophage; Emory,totalSample,immune-Neutrophil; Emory,totalSample,immune-CD4_T; Emory,totalSample,immune-CD8_T; Emory,totalSample,immune-Tr1; Emory,totalSample,immune-iTreg; Emory,totalSample,immune-Tfh; Emory,totalSample,immune-MAIT; Emory,totalSample,immune-Central_memory; Emory,totalSample,immune-Effector_memory; Emory,totalSample,immune-InfiltrationScore; Homo_723,totalSample,immune-Tfh; Homo_723,totalSample,immune-iTreg; Homo_723,totalSample,immune-Th2; Homo_723,totalSample,immune-CD4_T; Homo_723,totalSample,immune-Tr1; Homo_723,totalSample,immune-CD4_naive; Homo_723,totalSample,immune-Effector_memory; Homo_723,totalSample,immune-InfiltrationScore; Homo_723,totalSample,immune-DC; Homo_723,totalSample,immune-Central_memory; Homo_723,totalSample,immune-Macrophage; Homo_723,totalSample,immune-nTreg; Homo_723,totalSample,immune-Bcell; Homo_723,totalSample,immune-MAIT; Homo_723,totalSample,immune-Gamma_delta; Homo_714,totalSample,immune-Tfh; Homo_714,totalSample,immune-Th2; Homo_714,totalSample,immune-CD4_T; Homo_714,totalSample,immune-Effector_memory; Homo_714,totalSample,immune-Tr1; Homo_714,totalSample,immune-CD4_naive; Homo_714,totalSample,immune-Central_memory; Homo_714,totalSample,immune-iTreg; Homo_714,totalSample,immune-InfiltrationScore; Homo_714,totalSample,immune-nTreg; Homo_714,totalSample,immune-Macrophage; Homo_714,totalSample,immune-Bcell; Homo_714,totalSample,immune-MAIT; Homo_633,totalSample,immune-Tfh; Homo_633,totalSample,immune-CD4_T; Homo_633,totalSample,immune-CD4_naive; Homo_633,totalSample,immune-Effector_memory; Homo_633,totalSample,immune-Central_memory; Homo_633,totalSample,immune-Th2; Homo_633,totalSample,immune-InfiltrationScore; Homo_633,totalSample,immune-Tr1; Homo_633,totalSample,immune-iTreg; Homo_633,totalSample,immune-nTreg; Homo_633,totalSample,immune-Bcell; Homo_633,totalSample,immune-NKT; Homo_633,totalSample,immune-Th17; Homo_633,totalSample,immune-Exhausted; Homo_633,totalSample,immune-Neutrophil; Homo_633,totalSample,immune-Gamma_delta; MCSA,totalSample,immune-DC; MCSA,totalSample,immune-Macrophage; MCSA,totalSample,immune-CD4_T; MCSA,totalSample,immune-CD4_naive; MCSA,totalSample,immune-Tr1; MCSA,totalSample,immune-nTreg; MCSA,totalSample,immune-iTreg; MCSA,totalSample,immune-Th17; MCSA,totalSample,immune-Tfh; MCSA,totalSample,immune-CD8_naive; MCSA,totalSample,immune-Cytotoxic; MCSA,totalSample,immune-Exhausted; MCSA,totalSample,immune-MAIT; MCSA,totalSample,immune-Central_memory; MCSA,totalSample,immune-Effector_memory; [illegible]
SULT1A3	Homo_723,female,AD.vs.control; Homo_723,male,AD.vs.control; Homo_723,female,AD.vs.MCI; Homo_723,male,AD.vs.MCI; Homo_714,female,AD.vs.control; Homo_714,male,AD.vs.control; Homo_714,female,AD.vs.MCI; Homo_714,male,AD.vs.MCI; Homo_633,female,AD.vs.control; Homo_633,female,AD.vs.MCI; MCSA,MCI,age; Homo_723,totalSample,immune-InfiltrationScore; Homo_723,totalSample,immune-Effector_memory; Homo_723,totalSample,immune-Monocyte; Homo_723,totalSample,immune-Th1; Homo_723,totalSample,immune-Bcell; Homo_723,totalSample,immune-Cytotoxic; Homo_714,totalSample,immune-Effector_memory; Homo_714,totalSample,immune-Th1; Homo_714,totalSample,immune-Th2; Homo_714,totalSample,immune-InfiltrationScore; Homo_633,totalSample,immune-InfiltrationScore; Homo_633,totalSample,immune-DC; Homo_633,totalSample,immune-Monocyte; Homo_633,totalSample,immune-Exhausted; MCSA,totalSample,immune-DC; MCSA,totalSample,immune-Monocyte; MCSA,totalSample,immune-Macrophage; MCSA,totalSample,immune-iTreg; MCSA,totalSample,immune-Th2; MCSA,totalSample,immune-CD8_naive; MCSA,totalSample,immune-Cytotoxic; MCSA,totalSample,immune-InfiltrationScore; ROSMAP,totalSample,immune-iTreg; ROSMAP,totalSample,immune-Th17; ROSMAP,totalSample,immune-Cytotoxic; ROSMAP,totalSample,immune-Effector_memory; SRP223445,totalSample,immune-NK;

SUMF1	Homo_723,female,AD.vs.control; Homo_723,male,AD.vs.control; Homo_723,female,AD.vs.MCI; Homo_723,male,AD.vs.MCI; Homo_714,female,AD.vs.control; Homo_714,male,AD.vs.control; Homo_714,female,AD.vs.MCI; Homo_714,male,AD.vs.MCI; Homo_633,female,AD.vs.control; Homo_633,female,AD.vs.MCI; ROSMAP,female,AD.vs.control; ROSMAP,male,AD.vs.control; ROSMAP,female,AD.vs.MCI; ROSMAP,male,AD.vs.MCI; Emory,totalSample,immune-Monocyte; Emory,totalSample,immune-Gamma_delta; Emory,totalSample,immune-Tr1; Emory,totalSample,immune-iTreg; Emory,totalSample,immune-Effector_memory; Emory,totalSample,immune-InfiltrationScore; Homo_723,totalSample,immune-Th2; Homo_723,totalSample,immune-InfiltrationScore; Homo_723,totalSample,immune-iTreg; Homo_723,totalSample,immune-Tfh; Homo_723,totalSample,immune-Effector_memory; Homo_723,totalSample,immune-Monocyte; Homo_723,totalSample,immune-Bcell; Homo_723,totalSample,immune-Gamma_delta; Homo_723,totalSample,immune-DC; Homo_723,totalSample,immune-Tr1; Homo_723,totalSample,immune-CD4_T; Homo_714,totalSample,immune-Th2; Homo_714,totalSample,immune-Tfh; Homo_714,totalSample,immune-Effector_memory; Homo_714,totalSample,immune-Tr1; Homo_714,totalSample,immune-InfiltrationScore; Homo_714,totalSample,immune-CD4_T; Homo_714,totalSample,immune-Gamma_delta; Homo_714,totalSample,immune-Monocyte; Homo_633,totalSample,immune-InfiltrationScore; Homo_633,totalSample,immune-Th2; Homo_633,totalSample,immune-Monocyte; Homo_633,totalSample,immune-Exhausted; Homo_633,totalSample,immune-Tfh; Homo_633,totalSample,immune-Effector_memory; Homo_633,totalSample,immune-Gamma_delta; Homo_633,totalSample,immune-CD4_T; Homo_633,totalSample,immune-CD4_naive; Homo_633,totalSample,immune-Central_memory; Homo_633,totalSample,immune-Bcell; MCSA,totalSample,immune-CD4_T; MCSA,totalSample,immune-CD8_T; MCSA,totalSample,immune-CD4_naive; MCSA,totalSample,immune-Tr1; MCSA,totalSample,immune-nTreg; MCSA,totalSample,immune-iTreg; MCSA,totalSample,immune-Th17; MCSA,totalSample,immune-Cytotoxic; MCSA,totalSample,immune-Exhausted; MCSA,totalSample,immune-Central_memory; ROSMAP,totalSample,immune-Effector_memory; ROSMAP,female-excludeAge90+,survival; ROSMAP,female-includeAge90+,survival; ROSMAP,male-excludeAge90+,survival; [illegible]
LINS1	Homo_723,female,AD.vs.control; Homo_723,male,AD.vs.control; Homo_723,female,AD.vs.MCI; Homo_723,male,AD.vs.MCI; Homo_714,female,AD.vs.control; Homo_714,male,AD.vs.control; Homo_714,female,AD.vs.MCI; Homo_714,male,AD.vs.MCI; Homo_633,female,AD.vs.control; Homo_633,female,AD.vs.MCI; MCSA,totalSample,age; MCSA,control,age; MCSA,MCI,age; ROSMAP,MCI,last.visit.age; ROSMAP,MCI,sample.age; Emory,totalSample,immune-DC; Emory,totalSample,immune-Macrophage; Emory,totalSample,immune-Neutrophil; Emory,totalSample,immune-CD4_T; Emory,totalSample,immune-CD8_T; Emory,totalSample,immune-Tr1; Emory,totalSample,immune-iTreg; Emory,totalSample,immune-Tfh; Emory,totalSample,immune-MAIT; Emory,totalSample,immune-Central_memory; Emory,totalSample,immune-InfiltrationScore; Homo_723,totalSample,immune-Tfh; Homo_723,totalSample,immune-iTreg; Homo_723,totalSample,immune-Th2; Homo_723,totalSample,immune-CD4_T; Homo_723,totalSample,immune-Tr1; Homo_723,totalSample,immune-CD4_naive; Homo_723,totalSample,immune-Central_memory; Homo_723,totalSample,immune-Effector_memory; Homo_723,totalSample,immune-InfiltrationScore; Homo_723,totalSample,immune-nTreg; Homo_723,totalSample,immune-DC; Homo_723,totalSample,immune-Macrophage; Homo_723,totalSample,immune-Gamma_delta; Homo_723,totalSample,immune-MAIT; Homo_723,totalSample,immune-Bcell; Homo_723,totalSample,immune-CD8_T; Homo_714,totalSample,immune-Tfh; Homo_714,totalSample,immune-Th2; Homo_714,totalSample,immune-CD4_T; Homo_714,totalSample,immune-Tr1; Homo_714,totalSample,immune-Central_memory; Homo_714,totalSample,immune-CD4_naive; Homo_714,totalSample,immune-Effector_memory; Homo_714,totalSample,immune-iTreg; Homo_714,totalSample,immune-nTreg; Homo_714,totalSample,immune-InfiltrationScore; Homo_714,totalSample,immune-Macrophage; Homo_714,totalSample,immune-Gamma_delta; Homo_714,totalSample,immune-MAIT; Homo_714,totalSample,immune-DC; Homo_714,totalSample,immune-Bcell; Homo_633,totalSample,immune-Tfh; Homo_633,totalSample,immune-CD4_T; Homo_633,totalSample,immune-CD4_naive; Homo_633,totalSample,immune-Central_memory; Homo_633,totalSample,immune-Tr1; Homo_633,totalSample,immune-Effector_memory; Homo_633,totalSample,immune-Th2; Homo_633,totalSample,immune-iTreg; Homo_633,totalSample,immune-nTreg; Homo_633,totalSample,immune-InfiltrationScore; Homo_633,totalSample,immune-Th17; Homo_633,totalSample,immune-NKT; Homo_633,totalSample,immune-Bcell; Homo_633,totalSample,immune-Gamma_delta; Homo_633,totalSample,immune-MAIT; Homo_633,totalSample,immune-Th1; MCSA,totalSample,immune-DC; MCSA,totalSample,immune-Macrophage; MCSA,totalSample,immune-CD4_T; MCSA,totalSample,immune-CD4_naive; MCSA,totalSample,immune-Tr1; MCSA,totalSample,immune-nTreg; MCSA,totalSample,immune-iTreg; MCSA,totalSample,immune-Th17; MCSA,totalSample,immune-Tfh; MCSA,totalSample,immune-CD8_naive; MCSA,totalSample,immune-Cytotoxic; MCSA,totalSample,immune-Exhausted; MCSA,totalSample,immune-MAIT; MCSA,totalSample,immune-Central_memory; MCSA,totalSample,immune-Effector_memory; ROSMAP,totalSample,immune-Effector_memory; SRP223445,totalSample,immune-nTreg; ROSMAP,male-excludeAge90+,survival
CTSO	Homo_723,female,AD.vs.control; Homo_723,male,AD.vs.control; Homo_723,female,AD.vs.MCI; Homo_723,male,AD.vs.MCI; Homo_714,female,AD.vs.control; Homo_714,male,AD.vs.control; Homo_714,female,AD.vs.MCI; Homo_714,male,AD.vs.MCI; Homo_633,female,AD.vs.control; Homo_633,female,AD.vs.MCI; MCSA,MCI,age; Emory,totalSample,immune-NK; Emory,totalSample,immune-Neutrophil; Emory,totalSample,immune-CD4_T; Emory,totalSample,immune-Tr1; Emory,totalSample,immune-iTreg; Emory,totalSample,immune-Effector_memory; Emory,totalSample,immune-InfiltrationScore; Homo_723,totalSample,immune-Tfh; Homo_723,totalSample,immune-iTreg; Homo_723,totalSample,immune-Th2; Homo_723,totalSample,immune-Tr1; Homo_723,totalSample,immune-CD4_T; Homo_723,totalSample,immune-Effector_memory; Homo_723,totalSample,immune-InfiltrationScore; Homo_723,totalSample,immune-CD4_naive; Homo_723,totalSample,immune-DC; Homo_723,totalSample,immune-Gamma_delta; Homo_723,totalSample,immune-Monocyte; Homo_723,totalSample,immune-Central_memory; Homo_723,totalSample,immune-Bcell; Homo_723,totalSample,immune-MAIT; Homo_714,totalSample,immune-Tfh; Homo_714,totalSample,immune-Th2; Homo_714,totalSample,immune-CD4_T; Homo_714,totalSample,immune-Effector_memory; Homo_714,totalSample,immune-Tr1; Homo_714,totalSample,immune-CD4_naive; Homo_714,totalSample,immune-iTreg; Homo_714,totalSample,immune-Central_memory; Homo_714,totalSample,immune-InfiltrationScore; Homo_714,totalSample,immune-Gamma_delta; Homo_714,totalSample,immune-Bcell; Homo_633,totalSample,immune-Tfh; Homo_633,totalSample,immune-CD4_T; Homo_633,totalSample,immune-CD4_naive; Homo_633,totalSample,immune-Effector_memory; Homo_633,totalSample,immune-Th2; Homo_633,totalSample,immune-Central_memory; Homo_633,totalSample,immune-InfiltrationScore; Homo_633,totalSample,immune-Tr1; Homo_633,totalSample,immune-NKT; Homo_633,totalSample,immune-Monocyte; Homo_633,totalSample,immune-iTreg; Homo_633,totalSample,immune-Exhausted; Homo_633,totalSample,immune-Gamma_delta; Homo_633,totalSample,immune-Bcell; Homo_633,totalSample,immune-Th17; Homo_633,totalSample,immune-nTreg; MCSA,totalSample,immune-DC; MCSA,totalSample,immune-Monocyte; MCSA,totalSample,immune-Macrophage; MCSA,totalSample,immune-CD4_T; MCSA,totalSample,immune-CD8_T; MCSA,totalSample,immune-CD4_naive; MCSA,totalSample,immune-Tr1; MCSA,totalSample,immune-nTreg; MCSA,totalSample,immune-iTreg; MCSA,totalSample,immune-Th17; MCSA,totalSample,immune-Tfh; MCSA,totalSample,immune-CD8_naive; MCSA,totalSample,immune-Cytotoxic; MCSA,totalSample,immune-Exhausted; [illegible]
RPGRIP1	Homo_723,female,AD.vs.control; Homo_723,male,AD.vs.control; Homo_723,female,AD.vs.MCI; Homo_723,male,AD.vs.MCI; Homo_723,female,MCI.vs.control; Homo_714,female,AD.vs.control; Homo_714,male,AD.vs.control; Homo_714,female,AD.vs.MCI; Homo_714,male,AD.vs.MCI; Homo_714,female,MCI.vs.control; Homo_633,female,AD.vs.control; Homo_633,female,AD.vs.MCI; Homo_633,female,MCI.vs.control; Homo_633,male,MCI.vs.control; ROSMAP,female,AD.vs.control; ROSMAP,male,AD.vs.control; ROSMAP,female,AD.vs.MCI; Emory,totalSample,age; Emory,MCI,age; MCSA,MCI,age; ROSMAP,AD,MMSE; Emory,totalSample,immune-Monocyte; Emory,totalSample,immune-Neutrophil; Emory,totalSample,immune-CD8_T; Emory,totalSample,immune-Gamma_delta; Emory,totalSample,immune-Th1; Emory,totalSample,immune-Th17; Emory,totalSample,immune-Tfh; Emory,totalSample,immune-CD8_naive; Emory,totalSample,immune-Cytotoxic; Emory,totalSample,immune-Exhausted; Homo_723,totalSample,immune-Monocyte; Homo_723,totalSample,immune-DC; Homo_723,totalSample,immune-Th2; Homo_723,totalSample,immune-Bcell; Homo_723,totalSample,immune-Neutrophil; Homo_723,totalSample,immune-Exhausted; Homo_723,totalSample,immune-Gamma_delta; Homo_714,totalSample,immune-Monocyte; Homo_714,totalSample,immune-Th2; Homo_714,totalSample,immune-Tfh; Homo_714,totalSample,immune-Th1; Homo_714,totalSample,immune-Gamma_delta; Homo_633,totalSample,immune-Monocyte; Homo_633,totalSample,immune-Th2; Homo_633,totalSample,immune-Exhausted; Homo_633,totalSample,immune-Th17; Homo_633,totalSample,immune-Gamma_delta; MCSA,totalSample,immune-DC; MCSA,totalSample,immune-Monocyte; MCSA,totalSample,immune-Neutrophil; MCSA,totalSample,immune-CD8_T; MCSA,totalSample,immune-Th1; MCSA,totalSample,immune-Th17; MCSA,totalSample,immune-Cytotoxic; MCSA,totalSample,immune-Exhausted; MCSA,totalSample,immune-Effector_memory; MCSA,totalSample,immune-InfiltrationScore; ROSMAP,totalSample,immune-Effector_memory;
LOC107987423	Homo_723,female,AD.vs.control; Homo_723,male,AD.vs.control; Homo_723,female,AD.vs.MCI; Homo_723,male,AD.vs.MCI; Homo_714,female,AD.vs.control; Homo_714,male,AD.vs.control; Homo_714,female,AD.vs.MCI; Homo_714,male,AD.vs.MCI; Homo_633,female,AD.vs.control; Homo_633,female,AD.vs.MCI; Emory,totalSample,immune-Macrophage; Emory,totalSample,immune-CD8_T; Emory,totalSample,immune-Tfh; Emory,totalSample,immune-Exhausted; Homo_723,totalSample,immune-Monocyte; Homo_723,totalSample,immune-Bcell; Homo_714,totalSample,immune-Monocyte; Homo_714,totalSample,immune-Th2; Homo_714,totalSample,immune-Th1; Homo_633,totalSample,immune-Monocyte; Homo_633,totalSample,immune-Exhausted; Homo_633,totalSample,immune-Macrophage; MCSA,totalSample,immune-Macrophage; SRP223445,totalSample,immune-CD4_naive
NR4A2	Homo_723,female,AD.vs.control; Homo_723,male,AD.vs.control; Homo_723,female,AD.vs.MCI; Homo_723,male,AD.vs.MCI; Homo_714,female,AD.vs.control; Homo_714,male,AD.vs.control; Homo_714,female,AD.vs.MCI; Homo_714,male,AD.vs.MCI; Homo_714,male,MCI.vs.control; Homo_633,female,AD.vs.control; Homo_633,female,AD.vs.MCI; Homo_633,female,MCI.vs.control; Homo_633,male,MCI.vs.control; ROSMAP,female,AD.vs.control; ROSMAP,male,AD.vs.control; SRP223445,female,AD.vs.control; MCSA,MCI,age; Emory,totalSample,immune-Th17; Emory,totalSample,immune-Tfh; Homo_723,totalSample,immune-Monocyte; Homo_723,totalSample,immune-DC; Homo_723,totalSample,immune-NK; Homo_714,totalSample,immune-NKT; Homo_633,totalSample,immune-NKT; Homo_633,totalSample,immune-InfiltrationScore; Homo_633,totalSample,immune-Monocyte; Homo_633,totalSample,immune-NK; MCSA,totalSample,immune-DC; MCSA,totalSample,immune-Monocyte; MCSA,totalSample,immune-Tr1; MCSA,totalSample,immune-iTreg;
COLEC12	Homo_723,female,AD.vs.control; Homo_723,male,AD.vs.control; Homo_723,female,AD.vs.MCI; Homo_723,male,AD.vs.MCI; Homo_714,female,AD.vs.control; Homo_714,female,AD.vs.MCI; Homo_633,female,AD.vs.control; Homo_633,female,AD.vs.MCI; ROSMAP,female,AD.vs.control; ROSMAP,male,AD.vs.control; ROSMAP,female,AD.vs.MCI; ROSMAP,male,AD.vs.MCI; Emory,totalSample,immune-Th1; Emory,totalSample,immune-Tfh; MCSA,totalSample,immune-Tr1; MCSA,totalSample,immune-iTreg; MCSA,totalSample,immune-Th17; MCSA,totalSample,immune-Exhausted; SRP223445,totalSample,immune-InfiltrationScore; ROSMAP,male-excludeAge90+,survival
CCDC28A	Homo_723,female,AD.vs.control; Homo_723,male,AD.vs.control; Homo_723,female,AD.vs.MCI; Homo_723,male,AD.vs.MCI; Homo_714,female,AD.vs.control; Homo_714,male,AD.vs.control; Homo_714,female,AD.vs.MCI; Homo_714,male,AD.vs.MCI; Homo_633,female,AD.vs.control; Homo_633,female,AD.vs.MCI; ACOM,AD,age; Emory,MCI,age; MCSA,MCI,age; Emory,totalSample,immune-DC; Emory,totalSample,immune-Monocyte; Emory,totalSample,immune-Gamma_delta; Emory,totalSample,immune-Tr1; Emory,totalSample,immune-iTreg; Homo_723,totalSample,immune-Monocyte; Homo_723,totalSample,immune-DC; Homo_723,totalSample,immune-Th2; Homo_723,totalSample,immune-Tfh; Homo_723,totalSample,immune-iTreg; Homo_723,totalSample,immune-InfiltrationScore; Homo_723,totalSample,immune-Bcell; Homo_723,totalSample,immune-Gamma_delta; Homo_723,totalSample,immune-Neutrophil; Homo_723,totalSample,immune-Tr1; Homo_723,totalSample,immune-Effector_memory; Homo_714,totalSample,immune-Th2; Homo_714,totalSample,immune-Monocyte; Homo_714,totalSample,immune-Tfh; Homo_714,totalSample,immune-DC; Homo_714,totalSample,immune-Tr1; Homo_714,totalSample,immune-Gamma_delta; Homo_633,totalSample,immune-Monocyte; Homo_633,totalSample,immune-Th2; Homo_633,totalSample,immune-Exhausted; Homo_633,totalSample,immune-Tfh; Homo_633,totalSample,immune-Th17; Homo_633,totalSample,immune-InfiltrationScore; Homo_633,totalSample,immune-Gamma_delta; Homo_633,totalSample,immune-Bcell; Homo_633,totalSample,immune-DC; MCSA,totalSample,immune-DC; MCSA,totalSample,immune-Monocyte; MCSA,totalSample,immune-Macrophage; MCSA,totalSample,immune-Neutrophil; MCSA,totalSample,immune-CD8_T; MCSA,totalSample,immune-Tr1; MCSA,totalSample,immune-iTreg; MCSA,totalSample,immune-Th2; MCSA,totalSample,immune-Th17; MCSA,totalSample,immune-Cytotoxic; MCSA,totalSample,immune-Exhausted; MCSA,totalSample,immune-Effector_memory; SRP223445,totalSample,immune-Th17; SRP223445,totalSample,immune-Exhausted; [illegible]
ACOT4	Homo_723,female,AD.vs.control; Homo_723,male,AD.vs.control; Homo_723,female,AD.vs.MCI; Homo_723,male,AD.vs.MCI; Homo_714,female,AD.vs.control; Homo_714,male,AD.vs.control; Homo_714,female,AD.vs.MCI; Homo_714,male,AD.vs.MCI; Homo_633,female,AD.vs.control; Homo_633,female,AD.vs.MCI; ROSMAP,female,AD.vs.control; MCSA,MCI,age; ROSMAP,AD,sample.age; Emory,totalSample,immune-Neutrophil; Emory,totalSample,immune-CD4_T; Emory,totalSample,immune-CD8_T; Emory,totalSample,immune-Gamma_delta; Emory,totalSample,immune-Cytotoxic; Emory,totalSample,immune-Exhausted; Emory,totalSample,immune-Effector_memory; Emory,totalSample,immune-InfiltrationScore; Homo_723,totalSample,immune-InfiltrationScore; Homo_723,totalSample,immune-Tfh; Homo_723,totalSample,immune-Macrophage; Homo_723,totalSample,immune-CD8_T; Homo_723,totalSample,immune-Cytotoxic; Homo_723,totalSample,immune-Effector_memory; Homo_723,totalSample,immune-iTreg; Homo_723,totalSample,immune-MAIT; Homo_723,totalSample,immune-Exhausted; Homo_723,totalSample,immune-Th2; Homo_723,totalSample,immune-Bcell; Homo_723,totalSample,immune-CD4_T; Homo_723,totalSample,immune-CD4_naive; Homo_714,totalSample,immune-InfiltrationScore; Homo_714,totalSample,immune-Macrophage; Homo_714,totalSample,immune-Tfh; Homo_714,totalSample,immune-Effector_memory; Homo_714,totalSample,immune-CD8_T; Homo_714,totalSample,immune-Cytotoxic; Homo_714,totalSample,immune-MAIT; Homo_714,totalSample,immune-Th2; Homo_714,totalSample,immune-Exhausted; Homo_714,totalSample,immune-Neutrophil; Homo_714,totalSample,immune-CD4_T; Homo_714,totalSample,immune-Bcell; Homo_633,totalSample,immune-InfiltrationScore; Homo_633,totalSample,immune-Effector_memory; Homo_633,totalSample,immune-Neutrophil; Homo_633,totalSample,immune-Macrophage; Homo_633,totalSample,immune-NK; Homo_633,totalSample,immune-MAIT; Homo_633,totalSample,immune-Bcell; Homo_633,totalSample,immune-Tfh; Homo_633,totalSample,immune-Cytotoxic; Homo_633,totalSample,immune-CD8_T; Homo_633,totalSample,immune-CD4_naive; Homo_633,totalSample,immune-Gamma_delta; MCSA,totalSample,immune-Macrophage; MCSA,totalSample,immune-Neutrophil; MCSA,totalSample,immune-CD4_T; MCSA,totalSample,immune-Gamma_delta; MCSA,totalSample,immune-CD4_naive; MCSA,totalSample,immune-Tr1; MCSA,totalSample,immune-nTreg; MCSA,totalSample,immune-iTreg; MCSA,totalSample,immune-Tfh; MCSA,totalSample,immune-MAIT; MCSA,totalSample,immune-Effector_memory; SRP223445,totalSample,immune-InfiltrationScore
LAX1	Homo_723,female,AD.vs.control; Homo_723,male,AD.vs.control; Homo_723,female,AD.vs.MCI; Homo_723,male,AD.vs.MCI; Homo_714,female,AD.vs.control; Homo_714,male,AD.vs.control; Homo_714,female,AD.vs.MCI; Homo_714,male,AD.vs.MCI; Homo_633,female,AD.vs.control; Homo_633,female,AD.vs.MCI; Emory,totalSample,immune-Macrophage; Emory,totalSample,immune-NK; Emory,totalSample,immune-Neutrophil; Emory,totalSample,immune-CD4_T; Emory,totalSample,immune-CD8_T; Emory,totalSample,immune-Tr1; Emory,totalSample,immune-iTreg; Emory,totalSample,immune-Tfh; Emory,totalSample,immune-Exhausted; Emory,totalSample,immune-MAIT; Emory,totalSample,immune-Effector_memory; Emory,totalSample,immune-InfiltrationScore; Homo_723,totalSample,immune-Tfh; Homo_723,totalSample,immune-iTreg; Homo_723,totalSample,immune-Th2; Homo_723,totalSample,immune-CD4_T; Homo_723,totalSample,immune-Effector_memory; Homo_723,totalSample,immune-InfiltrationScore; Homo_723,totalSample,immune-CD4_naive; Homo_723,totalSample,immune-Tr1; Homo_723,totalSample,immune-Macrophage; Homo_723,totalSample,immune-Central_memory; Homo_723,totalSample,immune-nTreg; Homo_723,totalSample,immune-MAIT; Homo_723,totalSample,immune-DC; Homo_723,totalSample,immune-CD8_T; Homo_723,totalSample,immune-Bcell; Homo_714,totalSample,immune-Tfh; Homo_714,totalSample,immune-Th2; Homo_714,totalSample,immune-Effector_memory; Homo_714,totalSample,immune-CD4_T; Homo_714,totalSample,immune-CD4_naive; Homo_714,totalSample,immune-InfiltrationScore; Homo_714,totalSample,immune-Central_memory; Homo_714,totalSample,immune-Tr1; Homo_714,totalSample,immune-iTreg; Homo_714,totalSample,immune-Macrophage; Homo_714,totalSample,immune-nTreg; Homo_714,totalSample,immune-MAIT; Homo_714,totalSample,immune-CD8_T; Homo_714,totalSample,immune-Neutrophil; Homo_714,totalSample,immune-Bcell; Homo_633,totalSample,immune-Tfh; Homo_633,totalSample,immune-Effector_memory; Homo_633,totalSample,immune-CD4_naive; Homo_633,totalSample,immune-CD4_T; Homo_633,totalSample,immune-InfiltrationScore; Homo_633,totalSample,immune-Central_memory; Homo_633,totalSample,immune-Tr1; Homo_633,totalSample,immune-nTreg; Homo_633,totalSample,immune-Th2; Homo_633,totalSample,immune-Neutrophil; Homo_633,totalSample,immune-iTreg; Homo_633,totalSample,immune-MAIT; Homo_633,totalSample,immune-Macrophage; Homo_633,totalSample,immune-Bcell; Homo_633,totalSample,immune-NKT; Homo_633,totalSample,immune-CD8_T; MCSA,totalSample,immune-DC; MCSA,totalSample,immune-Monocyte; MCSA,totalSample,immune-Macrophage; MCSA,totalSample,immune-Neutrophil; MCSA,totalSample,immune-CD4_T; MCSA,totalSample,immune-CD4_naive; MCSA,totalSample,immune-Tr1; MCSA,totalSample,immune-nTreg; MCSA,totalSample,immune-iTreg; MCSA,totalSample,immune-Th17; MCSA,totalSample,immune-Tfh; MCSA,totalSample,immune-CD8_naive; MCSA,totalSample,immune-MAIT; MCSA,totalSample,immune-Central_memory; MCSA,totalSample,immune-Effector_memory; MCSA,totalSample,immune-InfiltrationScore; ROSMAP,totalSample,immune-NK; ROSMAP,totalSample,immune-InfiltrationScore; SRP223445,totalSample,immune-Neutrophil; SRP223445,totalSample,immune-CD8_T; SRP223445,totalSample,immune-nTreg; SRP223445,totalSample,immune-MAIT; [illegible]
SLC4A5	Homo_723,female,AD.vs.control; Homo_723,male,AD.vs.control; Homo_723,female,AD.vs.MCI; Homo_723,male,AD.vs.MCI; Homo_633,female,AD.vs.control; Homo_633,female,AD.vs.MCI; ROSMAP,control,male.vs.female; MCSA,totalSample,age; MCSA,control,age; Emory,totalSample,immune-CD4_T; Emory,totalSample,immune-Gamma_delta; Emory,totalSample,immune-CD4_naive; Emory,totalSample,immune-Tr1; Emory,totalSample,immune-nTreg; Emory,totalSample,immune-iTreg; Emory,totalSample,immune-Th17; Emory,totalSample,immune-Tfh; Emory,totalSample,immune-Cytotoxic; Emory,totalSample,immune-Central_memory; Homo_723,totalSample,immune-nTreg; Homo_723,totalSample,immune-CD4_T; Homo_723,totalSample,immune-CD4_naive; Homo_723,totalSample,immune-Tfh; Homo_723,totalSample,immune-iTreg; Homo_723,totalSample,immune-Central_memory; Homo_723,totalSample,immune-Th2; Homo_723,totalSample,immune-Tr1; Homo_723,totalSample,immune-InfiltrationScore; Homo_723,totalSample,immune-Th17; Homo_723,totalSample,immune-Th1; Homo_723,totalSample,immune-Effector_memory; Homo_723,totalSample,immune-Gamma_delta; Homo_714,totalSample,immune-Tfh; Homo_714,totalSample,immune-CD4_T; Homo_714,totalSample,immune-CD4_naive; Homo_714,totalSample,immune-nTreg; Homo_714,totalSample,immune-iTreg; Homo_714,totalSample,immune-Central_memory; Homo_714,totalSample,immune-Tr1; Homo_714,totalSample,immune-Th2; Homo_714,totalSample,immune-InfiltrationScore; Homo_714,totalSample,immune-Effector_memory; Homo_714,totalSample,immune-Gamma_delta; Homo_714,totalSample,immune-Th17; Homo_714,totalSample,immune-Th1; Homo_633,totalSample,immune-Tfh; Homo_633,totalSample,immune-CD4_T; Homo_633,totalSample,immune-nTreg; Homo_633,totalSample,immune-Tr1; Homo_633,totalSample,immune-CD4_naive; Homo_633,totalSample,immune-Central_memory; Homo_633,totalSample,immune-iTreg; Homo_633,totalSample,immune-Th2; Homo_633,totalSample,immune-Effector_memory; Homo_633,totalSample,immune-InfiltrationScore; Homo_633,totalSample,immune-Gamma_delta; Homo_633,totalSample,immune-Th17; Homo_633,totalSample,immune-NKT; Homo_633,totalSample,immune-Exhausted; MCSA,totalSample,immune-Monocyte; MCSA,totalSample,immune-Macrophage; MCSA,totalSample,immune-CD4_T; MCSA,totalSample,immune-Gamma_delta; MCSA,totalSample,immune-CD4_naive; MCSA,totalSample,immune-Tr1; MCSA,totalSample,immune-nTreg; MCSA,totalSample,immune-iTreg; MCSA,totalSample,immune-Th17; MCSA,totalSample,immune-Tfh; MCSA,totalSample,immune-CD8_naive; MCSA,totalSample,immune-Exhausted; MCSA,totalSample,immune-Central_memory; [illegible]
KRT8	Homo_723,female,AD.vs.control; Homo_723,male,AD.vs.control; Homo_723,female,AD.vs.MCI; Homo_723,male,AD.vs.MCI; Homo_723,male,MCI.vs.control; Homo_714,female,AD.vs.control; Homo_714,male,AD.vs.control; Homo_714,female,AD.vs.MCI; Homo_714,male,AD.vs.MCI; Homo_714,male,MCI.vs.control; Homo_633,female,AD.vs.MCI; Homo_633,female,MCI.vs.control; Homo_633,male,MCI.vs.control; ROSMAP,female,AD.vs.control; Emory,totalSample,immune-Th17; Homo_723,totalSample,immune-DC; Homo_714,totalSample,immune-DC; Homo_714,totalSample,immune-Th2; Homo_633,totalSample,immune-DC; MCSA,totalSample,immune-DC; ROSMAP,male and female-excludeAge90+,survival;
INAFM2	Homo_723,female,AD.vs.control; Homo_723,male,AD.vs.control; Homo_723,female,AD.vs.MCI; Homo_723,male,AD.vs.MCI; Homo_714,female,AD.vs.control; Homo_714,male,AD.vs.control; Homo_714,female,AD.vs.MCI; Homo_714,male,AD.vs.MCI; Homo_633,female,AD.vs.control; Homo_633,female,AD.vs.MCI; Emory,totalSample,immune-Neutrophil; Emory,totalSample,immune-CD8_T; Emory,totalSample,immune-Gamma_delta; Emory,totalSample,immune-Th1; Emory,totalSample,immune-Tfh; Emory,totalSample,immune-CD8_naive; Emory,totalSample,immune-Cytotoxic; Emory,totalSample,immune-Exhausted; Homo_723,totalSample,immune-Monocyte; Homo_723,totalSample,immune-Th2; Homo_723,totalSample,immune-Gamma_delta; Homo_723,totalSample,immune-Tfh; Homo_723,totalSample,immune-iTreg; Homo_723,totalSample,immune-DC; Homo_723,totalSample,immune-InfiltrationScore; Homo_723,totalSample,immune-Bcell; Homo_723,totalSample,immune-Neutrophil; Homo_723,totalSample,immune-Tr1; Homo_714,totalSample,immune-Th2; Homo_714,totalSample,immune-Tfh; Homo_714,totalSample,immune-Monocyte; Homo_714,totalSample,immune-Gamma_delta; Homo_714,totalSample,immune-Tr1; Homo_714,totalSample,immune-CD4_T; Homo_633,totalSample,immune-Th2; Homo_633,totalSample,immune-Monocyte; Homo_633,totalSample,immune-Exhausted; Homo_633,totalSample,immune-Gamma_delta; Homo_633,totalSample,immune-Th17; Homo_633,totalSample,immune-Tfh; Homo_633,totalSample,immune-InfiltrationScore; Homo_633,totalSample,immune-CD4_T; MCSA,totalSample,immune-Monocyte; MCSA,totalSample,immune-Neutrophil; MCSA,totalSample,immune-CD8_T; MCSA,totalSample,immune-Gamma_delta; MCSA,totalSample,immune-Tr1; MCSA,totalSample,immune-iTreg; MCSA,totalSample,immune-Th17; MCSA,totalSample,immune-Cytotoxic; MCSA,totalSample,immune-Exhausted; MCSA,totalSample,immune-Effector_memory; SRP223445,totalSample,immune-CD4_naive; SRP223445,totalSample,immune-Exhausted
TNFAIP6	Homo_723,female,AD.vs.control; Homo_723,male,AD.vs.control; Homo_723,female,AD.vs.MCI; Homo_723,male,AD.vs.MCI; Homo_714,female,AD.vs.control; Homo_714,male,AD.vs.control; Homo_714,female,AD.vs.MCI; Homo_714,male,AD.vs.MCI; Homo_633,female,AD.vs.control; Homo_633,female,AD.vs.MCI; SRP223445,female,AD.vs.control; MCSA,totalSample,age; MCSA,MCI,age; Emory,totalSample,immune-B_cell; Emory,totalSample,immune-Monocyte; Emory,totalSample,immune-Macrophage; Emory,totalSample,immune-Neutrophil; Emory,totalSample,immune-CD4_T; Emory,totalSample,immune-CD8_T; Emory,totalSample,immune-Gamma_delta; Emory,totalSample,immune-Tr1; Emory,totalSample,immune-nTreg; Emory,totalSample,immune-iTreg; Emory,totalSample,immune-Tfh; Emory,totalSample,immune-CD8_naive; Emory,totalSample,immune-Exhausted; Emory,totalSample,immune-MAIT; Homo_723,totalSample,immune-Monocyte; Homo_723,totalSample,immune-Neutrophil; Homo_723,totalSample,immune-DC; Homo_723,totalSample,immune-Gamma_delta; Homo_723,totalSample,immune-Bcell; Homo_714,totalSample,immune-Monocyte; Homo_714,totalSample,immune-Neutrophil; Homo_714,totalSample,immune-Th2; Homo_714,totalSample,immune-Gamma_delta; Homo_714,totalSample,immune-DC; Homo_633,totalSample,immune-Monocyte; Homo_633,totalSample,immune-Exhausted; Homo_633,totalSample,immune-Neutrophil; Homo_633,totalSample,immune-CD8_T; Homo_633,totalSample,immune-Gamma_delta; Homo_633,totalSample,immune-Th2; Homo_633,totalSample,immune-Macrophage; MCSA,totalSample,immune-DC; MCSA,totalSample,immune-Monocyte; MCSA,totalSample,immune-Neutrophil; MCSA,totalSample,immune-CD8_T; MCSA,totalSample,immune-CD4_naive; MCSA,totalSample,immune-nTreg; MCSA,totalSample,immune-Tfh; MCSA,totalSample,immune-Cytotoxic; MCSA,totalSample,immune-Exhausted; MCSA,totalSample,immune-Central_memory; MCSA,totalSample,immune-Effector_memory; MCSA,totalSample,immune-InfiltrationScore; SRP223445,totalSample,immune-

DPP4	Homo_723,female,AD.vs.control; Homo_723,male,AD.vs.control; Homo_723,female,AD.vs.MCI; Homo_723,male,AD.vs.MCI; Homo_723,female,MCI.vs.control; Homo_714,female,AD.vs.control; Homo_714,male,AD.vs.control; Homo_714,female,AD.vs.MCI; Homo_714,male,AD.vs.MCI; Homo_714,female,MCI.vs.control; Homo_633,female,AD.vs.control; Homo_633,female,AD.vs.MCI; Homo_633,female,MCI.vs.control; ROSMAP,male,AD.vs.control; MCSA,totalSample,age; MCSA,control,age; Emory,totalSample,immune-DC; Emory,totalSample,immune-Macrophage; Emory,totalSample,immune-Neutrophil; Emory,totalSample,immune-CD4_T; Emory,totalSample,immune-CD8_T; Emory,totalSample,immune-Tr1; Emory,totalSample,immune-nTreg; Emory,totalSample,immune-iTreg; Emory,totalSample,immune-Th2; Emory,totalSample,immune-Th17; Emory,totalSample,immune-Tfh; Emory,totalSample,immune-Cytotoxic; Emory,totalSample,immune-MAIT; Emory,totalSample,immune-Central_memory; Emory,totalSample,immune-InfiltrationScore; Homo_723,totalSample,immune-Th2; Homo_723,totalSample,immune-Tfh; Homo_723,totalSample,immune-iTreg; Homo_723,totalSample,immune-CD4_T; Homo_723,totalSample,immune-Tr1; Homo_723,totalSample,immune-CD4_naive; Homo_723,totalSample,immune-DC; Homo_723,totalSample,immune-Effector_memory; Homo_723,totalSample,immune-Central_memory; Homo_723,totalSample,immune-nTreg; Homo_723,totalSample,immune-Gamma_delta; Homo_723,totalSample,immune-Monocyte; Homo_723,totalSample,immune-Macrophage; Homo_723,totalSample,immune-InfiltrationScore; Homo_723,totalSample,immune-Bcell; Homo_714,totalSample,immune-Th2; Homo_714,totalSample,immune-Tfh; Homo_714,totalSample,immune-CD4_T; Homo_714,totalSample,immune-Tr1; Homo_714,totalSample,immune-Central_memory; Homo_714,totalSample,immune-iTreg; Homo_714,totalSample,immune-Effector_memory; Homo_714,totalSample,immune-CD4_naive; Homo_714,totalSample,immune-nTreg; Homo_714,totalSample,immune-Gamma_delta; Homo_714,totalSample,immune-Th17; Homo_633,totalSample,immune-Tfh; Homo_633,totalSample,immune-Th2; Homo_633,totalSample,immune-CD4_T; Homo_633,totalSample,immune-Central_memory; Homo_633,totalSample,immune-CD4_naive; Homo_633,totalSample,immune-Tr1; Homo_633,totalSample,immune-Th17; Homo_633,totalSample,immune-nTreg; Homo_633,totalSample,immune-Effector_memory; Homo_633,totalSample,immune-iTreg; Homo_633,totalSample,immune-Gamma_delta; Homo_633,totalSample,immune-Exhausted; Homo_633,totalSample,immune-InfiltrationScore; Homo_633,totalSample,immune-Monocyte; MCSA,totalSample,immune-DC; MCSA,totalSample,immune-Macrophage; MCSA,totalSample,immune-CD4_T; MCSA,totalSample,immune-Gamma_delta; MCSA,totalSample,immune-CD4_naive; MCSA,totalSample,immune-Tr1; MCSA,totalSample,immune-nTreg; MCSA,totalSample,immune-iTreg; MCSA,totalSample,immune-Th17; MCSA,totalSample,immune-Tfh; MCSA,totalSample,immune-CD8_naive; MCSA,totalSample,immune-Cytotoxic; MCSA,totalSample,immune-[illegible]
LDHA	Homo_723,female,AD.vs.control; Homo_723,male,AD.vs.control; Homo_723,female,AD.vs.MCI; Homo_723,male,AD.vs.MCI; Homo_714,female,AD.vs.control; Homo_714,male,AD.vs.control; Homo_714,female,AD.vs.MCI; Homo_714,male,AD.vs.MCI; Homo_633,female,AD.vs.control; Homo_633,female,AD.vs.MCI; ROSMAP,female,AD.vs.control; ROSMAP,female,MCI.vs.control; MCSA,MCI,age; ROSMAP,MCI,last.visit.age; Emory,totalSample,immune-Neutrophil; Emory,totalSample,immune-CD4_T; Emory,totalSample,immune-Tr1; Emory,totalSample,immune-iTreg; Emory,totalSample,immune-Tfh; Emory,totalSample,immune-Effector_memory; Emory,totalSample,immune-InfiltrationScore; Homo_723,totalSample,immune-Tfh; Homo_723,totalSample,immune-iTreg; Homo_723,totalSample,immune-Th2; Homo_723,totalSample,immune-InfiltrationScore; Homo_723,totalSample,immune-Tr1; Homo_723,totalSample,immune-CD4_T; Homo_723,totalSample,immune-CD4_naive; Homo_723,totalSample,immune-DC; Homo_723,totalSample,immune-Effector_memory; Homo_723,totalSample,immune-Gamma_delta; Homo_723,totalSample,immune-Bcell; Homo_723,totalSample,immune-Central_memory; Homo_714,totalSample,immune-Tfh; Homo_714,totalSample,immune-Th2; Homo_714,totalSample,immune-Tr1; Homo_714,totalSample,immune-CD4_T; Homo_714,totalSample,immune-Effector_memory; Homo_714,totalSample,immune-InfiltrationScore; Homo_714,totalSample,immune-iTreg; Homo_714,totalSample,immune-CD4_naive; Homo_714,totalSample,immune-Central_memory; Homo_633,totalSample,immune-Tfh; Homo_633,totalSample,immune-InfiltrationScore; Homo_633,totalSample,immune-CD4_T; Homo_633,totalSample,immune-Th2; Homo_633,totalSample,immune-CD4_naive; Homo_633,totalSample,immune-Effector_memory; Homo_633,totalSample,immune-Tr1; Homo_633,totalSample,immune-NKT; Homo_633,totalSample,immune-Central_memory; Homo_633,totalSample,immune-Bcell; Homo_633,totalSample,immune-Monocyte; Homo_633,totalSample,immune-Gamma_delta; MCSA,totalSample,immune-DC; MCSA,totalSample,immune-Monocyte; MCSA,totalSample,immune-CD4_T; MCSA,totalSample,immune-CD8_T; MCSA,totalSample,immune-CD4_naive; MCSA,totalSample,immune-Tr1; MCSA,totalSample,immune-nTreg; MCSA,totalSample,immune-iTreg; MCSA,totalSample,immune-Th2; MCSA,totalSample,immune-Th17; MCSA,totalSample,immune-Tfh; MCSA,totalSample,immune-Cytotoxic; MCSA,totalSample,immune-Exhausted; SRP223445,totalSample,immune-NK; SRP223445,totalSample,immune-CD4_naive; SRP223445,totalSample,immune-Th17; SRP223445,totalSample,immune-Exhausted; SRP223445,totalSample,immune-InfiltrationScore
STAT4	Homo_723,female,AD.vs.control; Homo_723,male,AD.vs.control; Homo_723,female,AD.vs.MCI; Homo_723,male,AD.vs.MCI; Homo_714,female,AD.vs.control; Homo_714,male,AD.vs.control; Homo_714,female,AD.vs.MCI; Homo_714,male,AD.vs.MCI; Homo_633,female,AD.vs.control; Homo_633,female,AD.vs.MCI; ROSMAP,female,AD.vs.control; ROSMAP,control,male.vs.female; MCSA,MCI,age; Emory,totalSample,immune-Macrophage; Emory,totalSample,immune-NK; Emory,totalSample,immune-Neutrophil; Emory,totalSample,immune-CD4_T; Emory,totalSample,immune-CD8_T; Emory,totalSample,immune-Gamma_delta; Emory,totalSample,immune-Tr1; Emory,totalSample,immune-iTreg; Emory,totalSample,immune-Tfh; Emory,totalSample,immune-Cytotoxic; Emory,totalSample,immune-Exhausted; Emory,totalSample,immune-MAIT; Emory,totalSample,immune-Effector_memory; Emory,totalSample,immune-InfiltrationScore; Homo_723,totalSample,immune-Tfh; Homo_723,totalSample,immune-Th2; Homo_723,totalSample,immune-Effector_memory; Homo_723,totalSample,immune-iTreg; Homo_723,totalSample,immune-InfiltrationScore; Homo_723,totalSample,immune-CD4_T; Homo_723,totalSample,immune-CD4_naive; Homo_723,totalSample,immune-Tr1; Homo_723,totalSample,immune-Macrophage; Homo_723,totalSample,immune-DC; Homo_723,totalSample,immune-Bcell; Homo_723,totalSample,immune-MAIT; Homo_723,totalSample,immune-Central_memory; Homo_714,totalSample,immune-Tfh; Homo_714,totalSample,immune-Th2; Homo_714,totalSample,immune-Effector_memory; Homo_714,totalSample,immune-CD4_T; Homo_714,totalSample,immune-InfiltrationScore; Homo_714,totalSample,immune-CD4_naive; Homo_714,totalSample,immune-Tr1; Homo_714,totalSample,immune-Central_memory; Homo_714,totalSample,immune-MAIT; Homo_714,totalSample,immune-Macrophage; Homo_714,totalSample,immune-Bcell; Homo_714,totalSample,immune-iTreg; Homo_714,totalSample,immune-CD8_T; Homo_714,totalSample,immune-nTreg; Homo_633,totalSample,immune-Tfh; Homo_633,totalSample,immune-Effector_memory; Homo_633,totalSample,immune-InfiltrationScore; Homo_633,totalSample,immune-CD4_T; Homo_633,totalSample,immune-CD4_naive; Homo_633,totalSample,immune-Th2; Homo_633,totalSample,immune-Central_memory; Homo_633,totalSample,immune-Bcell; Homo_633,totalSample,immune-Tr1; Homo_633,totalSample,immune-Neutrophil; Homo_633,totalSample,immune-MAIT; Homo_633,totalSample,immune-nTreg; Homo_633,totalSample,immune-iTreg; Homo_633,totalSample,immune-Monocyte; MCSA,totalSample,immune-DC; MCSA,totalSample,immune-Macrophage; MCSA,totalSample,immune-Neutrophil; MCSA,totalSample,immune-CD4_T; MCSA,totalSample,immune-Gamma_delta; MCSA,totalSample,immune-CD4_naive; MCSA,totalSample,immune-Tr1; MCSA,totalSample,immune-nTreg; MCSA,totalSample,immune-iTreg; MCSA,totalSample,immune-Th17; MCSA,totalSample,immune-Tfh; MCSA,totalSample,immune-CD8_naive; MCSA,totalSample,immune-MAIT; MCSA,totalSample,immune-Central_memory; MCSA,totalSample,immune-[illegible]
GALNT3	Homo_723,female,AD.vs.control; Homo_723,male,AD.vs.control; Homo_723,female,AD.vs.MCI; Homo_723,male,AD.vs.MCI; Homo_714,female,AD.vs.control; Homo_714,male,AD.vs.control; Homo_714,female,AD.vs.MCI; Homo_714,male,AD.vs.MCI; Homo_633,female,AD.vs.control; Homo_633,female,AD.vs.MCI; ROSMAP,female,AD.vs.control; Emory,MCI,age; MCSA,MCI,age; ROSMAP,MCI,sample.age; Emory,totalSample,immune-Monocyte; Emory,totalSample,immune-Neutrophil; Emory,totalSample,immune-CD8_T; Emory,totalSample,immune-Gamma_delta; Emory,totalSample,immune-nTreg; Emory,totalSample,immune-Tfh; Emory,totalSample,immune-CD8_naive; Emory,totalSample,immune-Exhausted; Homo_723,totalSample,immune-Monocyte; Homo_723,totalSample,immune-DC; Homo_723,totalSample,immune-Th2; Homo_723,totalSample,immune-Gamma_delta; Homo_723,totalSample,immune-Tfh; Homo_723,totalSample,immune-iTreg; Homo_723,totalSample,immune-InfiltrationScore; Homo_723,totalSample,immune-Neutrophil; Homo_723,totalSample,immune-Bcell; Homo_723,totalSample,immune-Tr1; Homo_723,totalSample,immune-Effector_memory; Homo_723,totalSample,immune-Exhausted; Homo_714,totalSample,immune-Th2; Homo_714,totalSample,immune-Monocyte; Homo_714,totalSample,immune-Tfh; Homo_714,totalSample,immune-DC; Homo_714,totalSample,immune-Gamma_delta; Homo_714,totalSample,immune-Tr1; Homo_714,totalSample,immune-Effector_memory; Homo_714,totalSample,immune-CD4_T; Homo_633,totalSample,immune-Monocyte; Homo_633,totalSample,immune-Th2; Homo_633,totalSample,immune-Exhausted; Homo_633,totalSample,immune-Tfh; Homo_633,totalSample,immune-Gamma_delta; Homo_633,totalSample,immune-InfiltrationScore; Homo_633,totalSample,immune-DC; Homo_633,totalSample,immune-Th17; Homo_633,totalSample,immune-CD4_T; MCSA,totalSample,immune-DC; MCSA,totalSample,immune-Monocyte; MCSA,totalSample,immune-Neutrophil; MCSA,totalSample,immune-CD4_T; MCSA,totalSample,immune-CD8_T; MCSA,totalSample,immune-Tr1; MCSA,totalSample,immune-iTreg; MCSA,totalSample,immune-CD8_naive; MCSA,totalSample,immune-Cytotoxic; MCSA,totalSample,immune-Exhausted; MCSA,totalSample,immune-Effector_memory; MCSA,totalSample,immune-InfiltrationScore; SRP223445,totalSample,immune-CD4_T; SRP223445,totalSample,immune-CD4_naive; SRP223445,totalSample,immune-[illegible]
OR2H2	Homo_723,female,AD.vs.control; Homo_723,female,AD.vs.MCI; Homo_723,male,AD.vs.MCI; MCSA,totalSample,age; MCSA,control,age; Homo_723,totalSample,immune-Th2; Homo_723,totalSample,immune-CD4_T; Homo_723,totalSample,immune-Tfh; Homo_723,totalSample,immune-iTreg; Homo_723,totalSample,immune-nTreg; Homo_723,totalSample,immune-Tr1; Homo_723,totalSample,immune-Th17; Homo_723,totalSample,immune-CD4_naive; Homo_714,totalSample,immune-Th2; Homo_714,totalSample,immune-Tfh; Homo_714,totalSample,immune-CD4_T; Homo_714,totalSample,immune-Tr1; Homo_714,totalSample,immune-Gamma_delta; Homo_633,totalSample,immune-Th2; Homo_633,totalSample,immune-Th17; Homo_633,totalSample,immune-Tfh; Homo_633,totalSample,immune-CD4_T; Homo_633,totalSample,immune-Gamma_delta; Homo_633,totalSample,immune-nTreg; Homo_633,totalSample,immune-Tr1; MCSA,totalSample,immune-Monocyte; MCSA,totalSample,immune-CD4_T; MCSA,totalSample,immune-Gamma_delta; MCSA,totalSample,immune-CD4_naive; MCSA,totalSample,immune-Tr1; MCSA,totalSample,immune-nTreg; MCSA,totalSample,immune-iTreg; MCSA,totalSample,immune-Th17; MCSA,totalSample,immune-Tfh; MCSA,totalSample,immune-Central_memory; ROSMAP,totalSample,immune-InfiltrationScore; ROSMAP,female-excludeAge90+,survival; ROSMAP,female-includeAge90+,survival; ROSMAP,male and
CES1	Homo_723,female,AD.vs.control; Homo_723,male,AD.vs.control; Homo_723,female,AD.vs.MCI; Homo_723,male,AD.vs.MCI; Homo_714,female,AD.vs.control; Homo_714,male,AD.vs.control; Homo_714,female,AD.vs.MCI; Homo_714,male,AD.vs.MCI; Homo_633,female,AD.vs.control; Homo_633,female,AD.vs.MCI; ROSMAP,female,AD.vs.control; Emory,MCI,age; Emory,totalSample,immune-Macrophage; Emory,totalSample,immune-CD8_T; Emory,totalSample,immune-Tfh; Emory,totalSample,immune-Exhausted; Homo_723,totalSample,immune-Monocyte; Homo_723,totalSample,immune-Th1; Homo_723,totalSample,immune-Bcell; Homo_723,totalSample,immune-InfiltrationScore; Homo_714,totalSample,immune-Monocyte; Homo_714,totalSample,immune-Th1; Homo_714,totalSample,immune-Th2; Homo_633,totalSample,immune-Monocyte; Homo_633,totalSample,immune-Exhausted; Homo_633,totalSample,immune-Macrophage; MCSA,totalSample,immune-DC; MCSA,totalSample,immune-Macrophage; MCSA,totalSample,immune-Th2; SRP223445,totalSample,immune-CD4_naive
IL18R1	Homo_723,female,AD.vs.control; Homo_723,male,AD.vs.control; Homo_723,female,AD.vs.MCI; Homo_723,male,AD.vs.MCI; Homo_723,female,MCI.vs.control; Homo_714,female,AD.vs.control; Homo_714,male,AD.vs.control; Homo_714,female,AD.vs.MCI; Homo_714,male,AD.vs.MCI; Homo_714,female,MCI.vs.control; Homo_633,female,AD.vs.control; Homo_633,female,AD.vs.MCI; Homo_633,female,MCI.vs.control; MCSA,MCI,age; ROSMAP,AD,MMSE; Emory,totalSample,immune-DC; Emory,totalSample,immune-B_cell; Emory,totalSample,immune-Monocyte; Emory,totalSample,immune-Neutrophil; Emory,totalSample,immune-nTreg; Emory,totalSample,immune-Th1; Emory,totalSample,immune-CD8_naive; Homo_723,totalSample,immune-DC; Homo_723,totalSample,immune-Monocyte; Homo_723,totalSample,immune-Th2; Homo_723,totalSample,immune-Tfh; Homo_723,totalSample,immune-InfiltrationScore; Homo_723,totalSample,immune-Bcell; Homo_723,totalSample,immune-iTreg; Homo_723,totalSample,immune-Effector_memory; Homo_723,totalSample,immune-Neutrophil; Homo_723,totalSample,immune-Gamma_delta; Homo_723,totalSample,immune-Exhausted; Homo_723,totalSample,immune-Tr1; Homo_714,totalSample,immune-Th2; Homo_714,totalSample,immune-Tfh; Homo_714,totalSample,immune-Monocyte; Homo_714,totalSample,immune-Effector_memory; Homo_714,totalSample,immune-DC; Homo_714,totalSample,immune-CD4_T; Homo_714,totalSample,immune-InfiltrationScore; Homo_714,totalSample,immune-Gamma_delta; Homo_633,totalSample,immune-Th2; Homo_633,totalSample,immune-Monocyte; Homo_633,totalSample,immune-Exhausted; Homo_633,totalSample,immune-Tfh; Homo_633,totalSample,immune-Th17; Homo_633,totalSample,immune-InfiltrationScore; Homo_633,totalSample,immune-Gamma_delta; Homo_633,totalSample,immune-CD4_T; Homo_633,totalSample,immune-Effector_memory; Homo_633,totalSample,immune-DC; Homo_633,totalSample,immune-Bcell; MCSA,totalSample,immune-DC; MCSA,totalSample,immune-Monocyte; MCSA,totalSample,immune-Neutrophil; MCSA,totalSample,immune-CD4_T; MCSA,totalSample,immune-CD8_T; MCSA,totalSample,immune-Tr1; MCSA,totalSample,immune-iTreg; MCSA,totalSample,immune-Th1; MCSA,totalSample,immune-Th17; MCSA,totalSample,immune-Cytotoxic; MCSA,totalSample,immune-Exhausted; ROSMAP,totalSample,immune-Th17; ROSMAP,totalSample,immune-[illegible]
REELD1	Homo_723,female,AD.vs.control; Homo_723,male,AD.vs.control; Homo_723,female,AD.vs.MCI; Homo_723,male,AD.vs.MCI; Homo_723,female,MCI.vs.control; Homo_723,male,MCI.vs.control; Homo_714,female,AD.vs.control; Homo_714,male,AD.vs.control; Homo_714,female,AD.vs.MCI; Homo_714,male,AD.vs.MCI; Homo_714,female,MCI.vs.control; Homo_714,male,MCI.vs.control; Homo_633,female,AD.vs.control; Homo_633,female,AD.vs.MCI; Homo_633,female,MCI.vs.control; Homo_633,male,MCI.vs.control; ROSMAP,female,AD.vs.control; MCSA,totalSample,age; ROSMAP,AD,MMSE; Emory,totalSample,immune-Gamma_delta; Emory,totalSample,immune-CD4_naive; Emory,totalSample,immune-Central_memory; Homo_723,totalSample,immune-Th2; Homo_723,totalSample,immune-Tfh; Homo_723,totalSample,immune-iTreg; Homo_723,totalSample,immune-Monocyte; Homo_723,totalSample,immune-DC; Homo_723,totalSample,immune-Effector_memory; Homo_723,totalSample,immune-Macrophage; Homo_723,totalSample,immune-CD4_T; Homo_714,totalSample,immune-Th2; Homo_714,totalSample,immune-Tfh; Homo_714,totalSample,immune-CD4_T; Homo_714,totalSample,immune-Effector_memory; Homo_714,totalSample,immune-Central_memory; Homo_714,totalSample,immune-Tr1; Homo_633,totalSample,immune-Th2; Homo_633,totalSample,immune-Tfh; Homo_633,totalSample,immune-Th17; Homo_633,totalSample,immune-Central_memory; Homo_633,totalSample,immune-Exhausted; Homo_633,totalSample,immune-CD4_T; Homo_633,totalSample,immune-Monocyte; Homo_633,totalSample,immune-nTreg; Homo_633,totalSample,immune-Gamma_delta; MCSA,totalSample,immune-Monocyte; MCSA,totalSample,immune-Macrophage; MCSA,totalSample,immune-CD4_T; MCSA,totalSample,immune-CD4_naive; MCSA,totalSample,immune-Tr1; MCSA,totalSample,immune-nTreg; MCSA,totalSample,immune-iTreg; MCSA,totalSample,immune-Th17; MCSA,totalSample,immune-Tfh; MCSA,totalSample,immune-CD8_naive; MCSA,totalSample,immune-Central_memory; MCSA,totalSample,immune-Effector_memory; ROSMAP,totalSample,immune-Th17; ROSMAP,totalSample,immune-InfiltrationScore; SRP223445,totalSample,immune-NK; SRP223445,totalSample,immune-CD4_naive; ROSMAP,male-excludeAge90+,survival
CPA3	Homo_723,female,AD.vs.control; Homo_723,male,AD.vs.control; Homo_723,female,AD.vs.MCI; Homo_723,male,AD.vs.MCI; Homo_723,male,MCI.vs.control; Homo_714,female,AD.vs.control; Homo_714,male,AD.vs.control; Homo_714,female,AD.vs.MCI; Homo_714,male,AD.vs.MCI; Homo_714,female,MCI.vs.control; Homo_714,male,MCI.vs.control; Homo_633,female,AD.vs.control; Homo_633,female,AD.vs.MCI; Homo_633,female,MCI.vs.control; Homo_633,male,MCI.vs.control; ROSMAP,control,male.vs.female; MCSA,MCI,age; Emory,totalSample,immune-Gamma_delta; Emory,totalSample,immune-Tfh; Homo_723,totalSample,immune-Monocyte; Homo_723,totalSample,immune-Neutrophil; Homo_723,totalSample,immune-Th2; Homo_723,totalSample,immune-DC; Homo_723,totalSample,immune-Bcell; Homo_723,totalSample,immune-Gamma_delta; Homo_714,totalSample,immune-Th2; Homo_714,totalSample,immune-Monocyte; Homo_633,totalSample,immune-Th2; Homo_633,totalSample,immune-Monocyte; Homo_633,totalSample,immune-Exhausted; Homo_633,totalSample,immune-Gamma_delta; MCSA,totalSample,immune-Neutrophil; MCSA,totalSample,immune-CD8_T; MCSA,totalSample,immune-Th2; MCSA,totalSample,immune-Effector_memory; ROSMAP,totalSample,immune-Th17; ROSMAP,totalSample,immune-InfiltrationScore
LOC105371932	Homo_723,female,AD.vs.control; Homo_723,male,AD.vs.control; Homo_723,female,AD.vs.MCI; Homo_723,male,AD.vs.MCI; Homo_714,female,AD.vs.control; Homo_714,male,AD.vs.control; Homo_714,female,AD.vs.MCI; Homo_714,male,AD.vs.MCI; Homo_633,female,AD.vs.control; Homo_633,female,AD.vs.MCI; MCSA,totalSample,age; MCSA,control,age; Emory,totalSample,immune-DC; Emory,totalSample,immune-Macrophage; Emory,totalSample,immune-Neutrophil; Emory,totalSample,immune-CD4_T; Emory,totalSample,immune-CD8_T; Emory,totalSample,immune-CD4_naive; Emory,totalSample,immune-Tr1; Emory,totalSample,immune-nTreg; Emory,totalSample,immune-iTreg; Emory,totalSample,immune-Tfh; Emory,totalSample,immune-CD8_naive; Emory,totalSample,immune-MAIT; Emory,totalSample,immune-Central_memory; Emory,totalSample,immune-InfiltrationScore; Homo_723,totalSample,immune-iTreg; Homo_723,totalSample,immune-Tfh; Homo_723,totalSample,immune-CD8_T; Homo_723,totalSample,immune-Central_memory; Homo_723,totalSample,immune-nTreg; Homo_723,totalSample,immune-Tr1; Homo_723,totalSample,immune-CD4_T; Homo_723,totalSample,immune-CD4_naive; Homo_723,totalSample,immune-Exhausted; Homo_723,totalSample,immune-Th2; Homo_723,totalSample,immune-Macrophage; Homo_723,totalSample,immune-MAIT; Homo_723,totalSample,immune-InfiltrationScore; Homo_723,totalSample,immune-Effector_memory; Homo_723,totalSample,immune-Monocyte; Homo_723,totalSample,immune-CD8_naive; Homo_714,totalSample,immune-Tfh; Homo_714,totalSample,immune-nTreg; Homo_714,totalSample,immune-Tr1; Homo_714,totalSample,immune-iTreg; Homo_714,totalSample,immune-Central_memory; Homo_714,totalSample,immune-CD4_T; Homo_714,totalSample,immune-Effector_memory; Homo_714,totalSample,immune-CD4_naive; Homo_714,totalSample,immune-Th2; Homo_714,totalSample,immune-Macrophage; Homo_714,totalSample,immune-Monocyte; Homo_714,totalSample,immune-CD8_T; Homo_714,totalSample,immune-InfiltrationScore; Homo_714,totalSample,immune-MAIT; Homo_633,totalSample,immune-nTreg; Homo_633,totalSample,immune-Central_memory; Homo_633,totalSample,immune-Tfh; Homo_633,totalSample,immune-Tr1; Homo_633,totalSample,immune-CD4_naive; Homo_633,totalSample,immune-CD4_T; Homo_633,totalSample,immune-iTreg; Homo_633,totalSample,immune-Effector_memory; Homo_633,totalSample,immune-InfiltrationScore; Homo_633,totalSample,immune-Monocyte; Homo_633,totalSample,immune-Neutrophil; Homo_633,totalSample,immune-Macrophage; MCSA,totalSample,immune-Monocyte; MCSA,totalSample,immune-Neutrophil; MCSA,totalSample,immune-CD4_T; MCSA,totalSample,immune-CD8_T; MCSA,totalSample,immune-NKT; MCSA,totalSample,immune-CD4_naive; MCSA,totalSample,immune-Tr1; MCSA,totalSample,immune-nTreg; MCSA,totalSample,immune-iTreg; MCSA,totalSample,immune-Th2; MCSA,totalSample,immune-Th17; MCSA,totalSample,immune-Tfh; MCSA,totalSample,immune-CD8_naive; MCSA,totalSample,immune-MAIT; [illegible]
FOLR3	Homo_723,female,AD.vs.control; Homo_723,male,AD.vs.control; Homo_723,female,AD.vs.MCI; Homo_723,male,AD.vs.MCI; Homo_714,female,AD.vs.MCI; Homo_714,female,MCI.vs.control; Homo_714,MCI,male.vs.female; Homo_633,MCI,male.vs.female; ROSMAP,female,AD.vs.control; ROSMAP,female,AD.vs.MCI; MCSA,MCI,age; ROSMAP,MCI,last.visit.age; ROSMAP,MCI,sample.age; Emory,totalSample,immune-Monocyte; Emory,totalSample,immune-Neutrophil; Homo_723,totalSample,immune-Cytotoxic; Homo_723,totalSample,immune-Th1; Homo_723,totalSample,immune-Neutrophil; Homo_723,totalSample,immune-Th17; MCSA,totalSample,immune-DC; MCSA,totalSample,immune-Macrophage; MCSA,totalSample,immune-CD4_T; MCSA,totalSample,immune-CD4_naive; MCSA,totalSample,immune-Tr1; MCSA,totalSample,immune-iTreg; MCSA,totalSample,immune-Th2; MCSA,totalSample,immune-Tfh; SRP223445,totalSample,immune-CD4_T
ARSG	Homo_723,female,AD.vs.control; Homo_723,male,AD.vs.control; Homo_723,female,AD.vs.MCI; Homo_723,male,AD.vs.MCI; Homo_714,female,AD.vs.control; Homo_714,male,AD.vs.control; Homo_714,female,AD.vs.MCI; Homo_714,male,AD.vs.MCI; Homo_633,female,AD.vs.control; Homo_633,female,AD.vs.MCI; ACOM,totalSample,age; Emory,totalSample,age; Emory,MCI,age; MCSA,control,age; Emory,totalSample,immune-Neutrophil; Emory,totalSample,immune-Gamma_delta; Emory,totalSample,immune-iTreg; Emory,totalSample,immune-Th17; Emory,totalSample,immune-CD8_naive; Homo_723,totalSample,immune-Th2; Homo_723,totalSample,immune-InfiltrationScore; Homo_723,totalSample,immune-Tfh; Homo_723,totalSample,immune-iTreg; Homo_723,totalSample,immune-Bcell; Homo_723,totalSample,immune-Effector_memory; Homo_723,totalSample,immune-Gamma_delta; Homo_723,totalSample,immune-Neutrophil; Homo_723,totalSample,immune-Tr1; Homo_723,totalSample,immune-CD4_T; Homo_723,totalSample,immune-DC; Homo_714,totalSample,immune-Th2; Homo_714,totalSample,immune-Tfh; Homo_714,totalSample,immune-Effector_memory; Homo_714,totalSample,immune-InfiltrationScore; Homo_714,totalSample,immune-Tr1; Homo_714,totalSample,immune-CD4_T; Homo_714,totalSample,immune-Gamma_delta; Homo_633,totalSample,immune-InfiltrationScore; Homo_633,totalSample,immune-Exhausted; Homo_633,totalSample,immune-Th2; Homo_633,totalSample,immune-Effector_memory; Homo_633,totalSample,immune-Tfh; Homo_633,totalSample,immune-Th17; Homo_633,totalSample,immune-CD4_T; Homo_633,totalSample,immune-Gamma_delta; MCSA,totalSample,immune-Monocyte; MCSA,totalSample,immune-Neutrophil; MCSA,totalSample,immune-CD4_T; MCSA,totalSample,immune-Gamma_delta; MCSA,totalSample,immune-CD4_naive; MCSA,totalSample,immune-Tr1; MCSA,totalSample,immune-nTreg; MCSA,totalSample,immune-iTreg; MCSA,totalSample,immune-Th1; MCSA,totalSample,immune-Th2; MCSA,totalSample,immune-Th17; MCSA,totalSample,immune-Tfh; MCSA,totalSample,immune-Exhausted;
FGFBP3	Homo_723,female,AD.vs.control; Homo_723,male,AD.vs.control; Homo_723,female,AD.vs.MCI; Homo_723,male,AD.vs.MCI; Homo_633,female,AD.vs.control; Homo_633,female,AD.vs.MCI; ROSMAP,AD,sample.age; ROSMAP,MCI,last.visit.age; Emory,totalSample,immune-Macrophage; Emory,totalSample,immune-Neutrophil; Emory,totalSample,immune-CD4_T; Emory,totalSample,immune-CD8_T; Emory,totalSample,immune-Tr1; Emory,totalSample,immune-iTreg; Emory,totalSample,immune-Tfh; Emory,totalSample,immune-Exhausted; Emory,totalSample,immune-InfiltrationScore; Homo_723,totalSample,immune-InfiltrationScore; Homo_723,totalSample,immune-Monocyte; Homo_723,totalSample,immune-CD4_naive; Homo_723,totalSample,immune-nTreg; Homo_723,totalSample,immune-Central_memory; Homo_723,totalSample,immune-Tfh; Homo_723,totalSample,immune-CD4_T; Homo_723,totalSample,immune-iTreg; Homo_723,totalSample,immune-Tr1; Homo_723,totalSample,immune-Neutrophil; Homo_723,totalSample,immune-Th1; Homo_723,totalSample,immune-NKT; Homo_723,totalSample,immune-Macrophage; Homo_714,totalSample,immune-InfiltrationScore; Homo_714,totalSample,immune-CD4_naive; Homo_714,totalSample,immune-Tfh; Homo_714,totalSample,immune-NKT; Homo_714,totalSample,immune-Effector_memory; Homo_714,totalSample,immune-nTreg; Homo_714,totalSample,immune-Neutrophil; Homo_714,totalSample,immune-iTreg; Homo_714,totalSample,immune-Tr1; Homo_714,totalSample,immune-Monocyte; Homo_714,totalSample,immune-CD4_T; Homo_714,totalSample,immune-Macrophage; Homo_714,totalSample,immune-Th1; Homo_714,totalSample,immune-Central_memory; Homo_714,totalSample,immune-MAIT; Homo_633,totalSample,immune-InfiltrationScore; Homo_633,totalSample,immune-CD4_naive; Homo_633,totalSample,immune-Tfh; Homo_633,totalSample,immune-Tr1; Homo_633,totalSample,immune-CD4_T; Homo_633,totalSample,immune-iTreg; Homo_633,totalSample,immune-nTreg; Homo_633,totalSample,immune-Effector_memory; Homo_633,totalSample,immune-Monocyte; Homo_633,totalSample,immune-NKT; Homo_633,totalSample,immune-Central_memory; Homo_633,totalSample,immune-Th1; Homo_633,totalSample,immune-Neutrophil; Homo_633,totalSample,immune-Macrophage; Homo_633,totalSample,immune-MAIT; MCSA,totalSample,immune-DC; MCSA,totalSample,immune-Macrophage; MCSA,totalSample,immune-Neutrophil; MCSA,totalSample,immune-CD4_T; MCSA,totalSample,immune-CD4_naive; MCSA,totalSample,immune-Tr1; MCSA,totalSample,immune-nTreg; MCSA,totalSample,immune-iTreg; MCSA,totalSample,immune-Th17; MCSA,totalSample,immune-Tfh; MCSA,totalSample,immune-CD8_naive; MCSA,totalSample,immune-Exhausted; MCSA,totalSample,immune-MAIT; [illegible]

PSENEN	Homo_723,female,AD.vs.control; Homo_723,male,AD.vs.control; Homo_723,female,AD.vs.MCI; Homo_723,male,AD.vs.MCI; Homo_714,female,AD.vs.control; Homo_714,male,AD.vs.control; Homo_714,female,AD.vs.MCI; Homo_714,male,AD.vs.MCI; Homo_633,female,AD.vs.control; Homo_633,female,AD.vs.MCI; Emory,totalSample,age; Emory,MCI,age; MCSA,MCI,age; Emory,totalSample,immune-Macrophage; Emory,totalSample,immune-Neutrophil; Emory,totalSample,immune-CD4_T; Emory,totalSample,immune-CD8_T; Emory,totalSample,immune-Gamma_delta; Emory,totalSample,immune-Th17; Emory,totalSample,immune-Tfh; Emory,totalSample,immune-CD8_naive; Emory,totalSample,immune-Cytotoxic; Emory,totalSample,immune-Exhausted; Emory,totalSample,immune-MAIT; Homo_723,totalSample,immune-Th1; Homo_723,totalSample,immune-Monocyte; Homo_723,totalSample,immune-Cytotoxic; Homo_723,totalSample,immune-Neutrophil; Homo_723,totalSample,immune-Bcell; Homo_723,totalSample,immune-InfiltrationScore; Homo_714,totalSample,immune-Th1; Homo_714,totalSample,immune-NKT; Homo_633,totalSample,immune-Th1; Homo_633,totalSample,immune-Monocyte; Homo_633,totalSample,immune-Exhausted; Homo_633,totalSample,immune-NKT; Homo_633,totalSample,immune-InfiltrationScore; MCSA,totalSample,immune-DC; MCSA,totalSample,immune-Bcell; MCSA,totalSample,immune-Monocyte; MCSA,totalSample,immune-Macrophage; MCSA,totalSample,immune-Neutrophil; MCSA,totalSample,immune-CD4_T; MCSA,totalSample,immune-CD4_naive; MCSA,totalSample,immune-Tr1; MCSA,totalSample,immune-nTreg; MCSA,totalSample,immune-iTreg; MCSA,totalSample,immune-Th2; MCSA,totalSample,immune-Tfh; MCSA,totalSample,immune-CD8_naive; MCSA,totalSample,immune-Cytotoxic; MCSA,totalSample,immune-MAIT; MCSA,totalSample,immune-Central_memory; ROSMAP,totalSample,immune-Effector_memory; SRP223445,totalSample,immune-CD4_T; SRP223445,totalSample,immune-Th17; ROSMAP,female-excludeAge90+,survival
KRT72	Homo_723,female,AD.vs.control; Homo_723,male,AD.vs.control; Homo_723,female,AD.vs.MCI; Homo_723,male,AD.vs.MCI; Homo_714,female,AD.vs.control; Homo_714,male,AD.vs.control; Homo_714,female,AD.vs.MCI; Homo_714,male,AD.vs.MCI; Homo_714,female,MCI.vs.control; Homo_714,control,male.vs.female; Homo_633,female,AD.vs.control; Homo_633,female,AD.vs.MCI; Homo_633,female,MCI.vs.control; Homo_633,control,male.vs.female; MCSA,control,age; Emory,totalSample,immune-Monocyte; Emory,totalSample,immune-CD4_T; Emory,totalSample,immune-CD8_T; Emory,totalSample,immune-CD4_naive; Emory,totalSample,immune-Tr1; Emory,totalSample,immune-nTreg; Emory,totalSample,immune-iTreg; Emory,totalSample,immune-Tfh; Emory,totalSample,immune-CD8_naive; Emory,totalSample,immune-Cytotoxic; Emory,totalSample,immune-Central_memory; Homo_723,totalSample,immune-iTreg; Homo_723,totalSample,immune-CD8_T; Homo_723,totalSample,immune-Tfh; Homo_723,totalSample,immune-Th2; Homo_723,totalSample,immune-Tr1; Homo_723,totalSample,immune-Macrophage; Homo_723,totalSample,immune-CD4_T; Homo_723,totalSample,immune-nTreg; Homo_723,totalSample,immune-Central_memory; Homo_723,totalSample,immune-CD4_naive; Homo_723,totalSample,immune-Effector_memory; Homo_714,totalSample,immune-Monocyte; Homo_714,totalSample,immune-nTreg; Homo_714,totalSample,immune-Tfh; Homo_714,totalSample,immune-Central_memory; Homo_714,totalSample,immune-iTreg; Homo_714,totalSample,immune-CD4_T; Homo_714,totalSample,immune-Macrophage; Homo_714,totalSample,immune-Tr1; Homo_714,totalSample,immune-CD4_naive; Homo_633,totalSample,immune-Monocyte; Homo_633,totalSample,immune-nTreg; Homo_633,totalSample,immune-Tfh; Homo_633,totalSample,immune-iTreg; Homo_633,totalSample,immune-CD4_naive; Homo_633,totalSample,immune-Tr1; Homo_633,totalSample,immune-CD4_T; MCSA,totalSample,immune-Monocyte; MCSA,totalSample,immune-CD4_T; MCSA,totalSample,immune-CD8_T; MCSA,totalSample,immune-CD4_naive; MCSA,totalSample,immune-Tr1; MCSA,totalSample,immune-nTreg; MCSA,totalSample,immune-Th17; MCSA,totalSample,immune-Tfh; MCSA,totalSample,immune-Central_memory; MCSA,totalSample,immune-Effector_memory
RILP	Homo_723,female,AD.vs.control; Homo_723,male,AD.vs.control; Homo_723,female,AD.vs.MCI; Homo_723,male,AD.vs.MCI; Homo_714,female,AD.vs.control; Homo_714,male,AD.vs.control; Homo_714,female,AD.vs.MCI; Homo_714,male,AD.vs.MCI; Homo_633,female,AD.vs.control; Homo_633,female,AD.vs.MCI; ROSMAP,female,AD.vs.control; ROSMAP,male,AD.vs.control; Emory,MCI,age; MCSA,MCI,age; Emory,totalSample,immune-Gamma_delta; Emory,totalSample,immune-Tr1; Emory,totalSample,immune-iTreg; Homo_723,totalSample,immune-Cytotoxic; Homo_723,totalSample,immune-Exhausted; Homo_723,totalSample,immune-CD8_T; Homo_723,totalSample,immune-Th1; Homo_723,totalSample,immune-Th17; Homo_714,totalSample,immune-DC; Homo_714,totalSample,immune-Th1; Homo_714,totalSample,immune-CD8_naive; Homo_633,totalSample,immune-DC; Homo_633,totalSample,immune-Th2; Homo_633,totalSample,immune-Bcell; Homo_633,totalSample,immune-CD4_naive; MCSA,totalSample,immune-DC; MCSA,totalSample,immune-Monocyte; MCSA,totalSample,immune-Macrophage; MCSA,totalSample,immune-CD4_T; MCSA,totalSample,immune-CD8_T; MCSA,totalSample,immune-NKT; MCSA,totalSample,immune-Tr1; MCSA,totalSample,immune-iTreg; MCSA,totalSample,immune-Th2; MCSA,totalSample,immune-Cytotoxic
IFNGR1	Homo_723,female,AD.vs.control; Homo_723,male,AD.vs.control; Homo_723,female,AD.vs.MCI; Homo_723,male,AD.vs.MCI; Homo_714,female,AD.vs.control; Homo_714,male,AD.vs.control; Homo_714,female,AD.vs.MCI; Homo_714,male,AD.vs.MCI; Homo_633,female,AD.vs.control; Homo_633,female,AD.vs.MCI; SRP223445,female,AD.vs.control; MCSA,totalSample,age; MCSA,MCI,age; Emory,totalSample,immune-DC; Emory,totalSample,immune-Monocyte; Emory,totalSample,immune-Neutrophil; Emory,totalSample,immune-CD8_T; Emory,totalSample,immune-Gamma_delta; Emory,totalSample,immune-nTreg; Emory,totalSample,immune-Th1; Emory,totalSample,immune-Tfh; Emory,totalSample,immune-CD8_naive; Emory,totalSample,immune-Exhausted; Homo_723,totalSample,immune-Monocyte; Homo_723,totalSample,immune-DC; Homo_723,totalSample,immune-Th2; Homo_723,totalSample,immune-Gamma_delta; Homo_723,totalSample,immune-Neutrophil; Homo_723,totalSample,immune-Tfh; Homo_723,totalSample,immune-Exhausted; Homo_723,totalSample,immune-Bcell; Homo_723,totalSample,immune-iTreg; Homo_714,totalSample,immune-Monocyte; Homo_714,totalSample,immune-Th2; Homo_714,totalSample,immune-Tfh; Homo_714,totalSample,immune-Gamma_delta; Homo_714,totalSample,immune-DC; Homo_633,totalSample,immune-Monocyte; Homo_633,totalSample,immune-Exhausted; Homo_633,totalSample,immune-Th2; Homo_633,totalSample,immune-Gamma_delta; Homo_633,totalSample,immune-Th17; Homo_633,totalSample,immune-DC; Homo_633,totalSample,immune-Tfh; MCSA,totalSample,immune-DC; MCSA,totalSample,immune-Monocyte; MCSA,totalSample,immune-Neutrophil; MCSA,totalSample,immune-CD8_T; MCSA,totalSample,immune-iTreg; MCSA,totalSample,immune-Cytotoxic; MCSA,totalSample,immune-Exhausted; MCSA,totalSample,immune-Effector_memory; MCSA,totalSample,immune-InfiltrationScore; ROSMAP,totalSample,immune-Neutrophil; SRP223445,totalSample,immune-Neutrophil
RPP25L	Homo_723,female,AD.vs.control; Homo_723,male,AD.vs.control; Homo_723,female,AD.vs.MCI; Homo_723,male,AD.vs.MCI; Homo_714,female,AD.vs.control; Homo_714,male,AD.vs.control; Homo_714,female,AD.vs.MCI; Homo_714,male,AD.vs.MCI; Homo_633,female,AD.vs.MCI; Emory,MCI,age; MCSA,totalSample,age; MCSA,MCI,age; ROSMAP,control,sample.age; ROSMAP,AD,MMSE; Emory,totalSample,immune-CD4_T; Emory,totalSample,immune-Tr1; Emory,totalSample,immune-iTreg; Emory,totalSample,immune-InfiltrationScore; Homo_723,totalSample,immune-Cytotoxic; Homo_723,totalSample,immune-CD8_T; Homo_723,totalSample,immune-Effector_memory; Homo_723,totalSample,immune-InfiltrationScore; Homo_723,totalSample,immune-Th1; Homo_723,totalSample,immune-Tfh; Homo_723,totalSample,immune-Bcell; Homo_714,totalSample,immune-CD8_T; Homo_714,totalSample,immune-Effector_memory; Homo_714,totalSample,immune-InfiltrationScore; Homo_714,totalSample,immune-Th1; Homo_714,totalSample,immune-Cytotoxic; Homo_714,totalSample,immune-DC; Homo_714,totalSample,immune-Tfh; Homo_714,totalSample,immune-nTreg; Homo_633,totalSample,immune-DC; Homo_633,totalSample,immune-InfiltrationScore; Homo_633,totalSample,immune-Th1; Homo_633,totalSample,immune-Effector_memory; Homo_633,totalSample,immune-nTreg; Homo_633,totalSample,immune-CD8_T; Homo_633,totalSample,immune-NKT; Homo_633,totalSample,immune-Cytotoxic; MCSA,totalSample,immune-DC; MCSA,totalSample,immune-Monocyte; MCSA,totalSample,immune-Neutrophil; MCSA,totalSample,immune-CD8_T; MCSA,totalSample,immune-NKT; MCSA,totalSample,immune-iTreg; MCSA,totalSample,immune-Th2; MCSA,totalSample,immune-Cytotoxic; MCSA,totalSample,immune-Central_memory; MCSA,totalSample,immune-Effector_memory; MCSA,totalSample,immune-InfiltrationScore; ROSMAP,totalSample,immune-CD4_T; ROSMAP,totalSample,immune-nTreg; ROSMAP,totalSample,immune-iTreg; ROSMAP,totalSample,immune-Th2; ROSMAP,totalSample,immune-Th17; ROSMAP,totalSample,immune-Tfh; ROSMAP,totalSample,immune-InfiltrationScore; SRP223445,totalSample,immune-CD4_naive; SRP223445,totalSample,immune-Th17; SRP223445,totalSample,immune-Exhausted; SRP223445,totalSample,immune-InfiltrationScore; ROSMAP,female-excludeAge90+,survival
RHEBL1	Homo_723,female,AD.vs.control; Homo_723,male,AD.vs.control; Homo_723,female,AD.vs.MCI; Homo_723,male,AD.vs.MCI; Homo_714,female,AD.vs.control; Homo_714,male,AD.vs.control; Homo_714,female,AD.vs.MCI; Homo_714,male,AD.vs.MCI; Homo_633,female,AD.vs.control; Homo_633,female,AD.vs.MCI; ROSMAP,control,male.vs.female; ACOM,AD,age; Emory,totalSample,immune-Macrophage; Emory,totalSample,immune-NK; Emory,totalSample,immune-Neutrophil; Emory,totalSample,immune-CD4_T; Emory,totalSample,immune-CD8_T; Emory,totalSample,immune-Gamma_delta; Emory,totalSample,immune-CD4_naive; Emory,totalSample,immune-Tr1; Emory,totalSample,immune-iTreg; Emory,totalSample,immune-Tfh; Emory,totalSample,immune-Cytotoxic; Emory,totalSample,immune-Exhausted; Emory,totalSample,immune-Effector_memory; Emory,totalSample,immune-InfiltrationScore; Homo_723,totalSample,immune-InfiltrationScore; Homo_723,totalSample,immune-Effector_memory; Homo_723,totalSample,immune-Tfh; Homo_723,totalSample,immune-Macrophage; Homo_723,totalSample,immune-Th2; Homo_723,totalSample,immune-iTreg; Homo_723,totalSample,immune-Cytotoxic; Homo_723,totalSample,immune-CD8_T; Homo_723,totalSample,immune-CD4_T; Homo_723,totalSample,immune-MAIT; Homo_723,totalSample,immune-CD4_naive; Homo_723,totalSample,immune-Tr1; Homo_723,totalSample,immune-Bcell; Homo_723,totalSample,immune-nTreg; Homo_714,totalSample,immune-InfiltrationScore; Homo_714,totalSample,immune-Effector_memory; Homo_714,totalSample,immune-Tfh; Homo_714,totalSample,immune-Macrophage; Homo_714,totalSample,immune-CD8_T; Homo_714,totalSample,immune-Th2; Homo_714,totalSample,immune-Cytotoxic; Homo_714,totalSample,immune-MAIT; Homo_714,totalSample,immune-Neutrophil; Homo_714,totalSample,immune-CD4_T; Homo_714,totalSample,immune-Tr1; Homo_714,totalSample,immune-nTreg; Homo_633,totalSample,immune-InfiltrationScore; Homo_633,totalSample,immune-Effector_memory; Homo_633,totalSample,immune-Neutrophil; Homo_633,totalSample,immune-Tfh; Homo_633,totalSample,immune-Macrophage; Homo_633,totalSample,immune-Cytotoxic; Homo_633,totalSample,immune-CD8_T; Homo_633,totalSample,immune-MAIT; Homo_633,totalSample,immune-CD4_naive; Homo_633,totalSample,immune-NK; Homo_633,totalSample,immune-Bcell; Homo_633,totalSample,immune-CD4_T; Homo_633,totalSample,immune-nTreg; MCSA,totalSample,immune-Monocyte; MCSA,totalSample,immune-Macrophage; MCSA,totalSample,immune-NK; MCSA,totalSample,immune-Neutrophil; MCSA,totalSample,immune-CD4_T; MCSA,totalSample,immune-CD8_T; MCSA,totalSample,immune-Gamma_delta; MCSA,totalSample,immune-CD4_naive; MCSA,totalSample,immune-Tr1; MCSA,totalSample,immune-nTreg; MCSA,totalSample,immune-iTreg; MCSA,totalSample,immune-Tfh; MCSA,totalSample,immune-Cytotoxic; MCSA,totalSample,immune-MAIT; MCSA,totalSample,immune-Central_memory; MCSA,totalSample,immune-Effector_memory; MCSA,totalSample,immune-InfiltrationScore; [illegible]
YIPF1	Homo_723,female,AD.vs.control; Homo_723,male,AD.vs.control; Homo_723,female,AD.vs.MCI; Homo_723,male,AD.vs.MCI; Homo_714,female,AD.vs.control; Homo_714,male,AD.vs.control; Homo_714,female,AD.vs.MCI; Homo_714,male,AD.vs.MCI; Homo_633,female,AD.vs.control; Homo_633,female,AD.vs.MCI; Emory,MCI,age; ROSMAP,MCI,last.visit.age; ROSMAP,MCI,sample.age; Emory,totalSample,immune-B_cell; Emory,totalSample,immune-Neutrophil; Emory,totalSample,immune-CD8_T; Emory,totalSample,immune-Gamma_delta; Emory,totalSample,immune-Th17; Emory,totalSample,immune-Tfh; Emory,totalSample,immune-CD8_naive; Emory,totalSample,immune-Cytotoxic; Emory,totalSample,immune-Exhausted; Homo_723,totalSample,immune-Monocyte; Homo_723,totalSample,immune-Neutrophil; Homo_723,totalSample,immune-Th2; Homo_723,totalSample,immune-DC; Homo_723,totalSample,immune-Bcell; Homo_723,totalSample,immune-Gamma_delta; Homo_723,totalSample,immune-Th1; Homo_714,totalSample,immune-Th2; Homo_714,totalSample,immune-Monocyte; Homo_714,totalSample,immune-Neutrophil; Homo_714,totalSample,immune-Th1; Homo_714,totalSample,immune-Gamma_delta; Homo_633,totalSample,immune-Th2; Homo_633,totalSample,immune-Monocyte; Homo_633,totalSample,immune-Exhausted; Homo_633,totalSample,immune-Th17; Homo_633,totalSample,immune-Gamma_delta; Homo_633,totalSample,immune-Neutrophil; MCSA,totalSample,immune-Neutrophil; MCSA,totalSample,immune-CD8_T; MCSA,totalSample,immune-NKT; MCSA,totalSample,immune-Gamma_delta; MCSA,totalSample,immune-CD4_naive; MCSA,totalSample,immune-Th17; MCSA,totalSample,immune-Tfh; MCSA,totalSample,immune-CD8_naive;
PRCP	Homo_723,female,AD.vs.control; Homo_723,male,AD.vs.control; Homo_723,female,AD.vs.MCI; Homo_723,male,AD.vs.MCI; Homo_714,female,AD.vs.control; Homo_714,male,AD.vs.control; Homo_714,female,AD.vs.MCI; Homo_714,male,AD.vs.MCI; Homo_633,female,AD.vs.control; Homo_633,female,AD.vs.MCI; ROSMAP,female,AD.vs.control; ACOM,totalSample,age; Emory,MCI,age; MCSA,MCI,age; Emory,totalSample,immune-Monocyte; Emory,totalSample,immune-Neutrophil; Emory,totalSample,immune-CD8_T; Emory,totalSample,immune-Gamma_delta; Emory,totalSample,immune-Th1; Emory,totalSample,immune-Th17; Emory,totalSample,immune-Tfh; Emory,totalSample,immune-CD8_naive; Emory,totalSample,immune-Cytotoxic; Emory,totalSample,immune-Exhausted; Homo_723,totalSample,immune-Monocyte; Homo_723,totalSample,immune-Th2; Homo_723,totalSample,immune-DC; Homo_723,totalSample,immune-Gamma_delta; Homo_723,totalSample,immune-Neutrophil; Homo_723,totalSample,immune-Tfh; Homo_723,totalSample,immune-InfiltrationScore; Homo_723,totalSample,immune-iTreg; Homo_723,totalSample,immune-Bcell; Homo_723,totalSample,immune-Exhausted; Homo_723,totalSample,immune-Effector_memory; Homo_714,totalSample,immune-Th2; Homo_714,totalSample,immune-Monocyte; Homo_714,totalSample,immune-Tfh; Homo_714,totalSample,immune-Gamma_delta; Homo_633,totalSample,immune-Th2; Homo_633,totalSample,immune-Monocyte; Homo_633,totalSample,immune-Exhausted; Homo_633,totalSample,immune-Th17; Homo_633,totalSample,immune-Gamma_delta; Homo_633,totalSample,immune-Tfh; Homo_633,totalSample,immune-InfiltrationScore; Homo_633,totalSample,immune-CD8_T; MCSA,totalSample,immune-Neutrophil; MCSA,totalSample,immune-CD8_T; MCSA,totalSample,immune-Gamma_delta; MCSA,totalSample,immune-iTreg; MCSA,totalSample,immune-Th17; MCSA,totalSample,immune-Cytotoxic; MCSA,totalSample,immune-Exhausted; MCSA,totalSample,immune-Effector_memory; ROSMAP,totalSample,immune-Central_memory; ROSMAP,totalSample,immune-
GHRL	Homo_723,female,AD.vs.control; Homo_723,male,AD.vs.control; Homo_723,female,AD.vs.MCI; Homo_723,male,AD.vs.MCI; Homo_723,female,MCI.vs.control; Homo_714,female,AD.vs.control; Homo_714,male,AD.vs.control; Homo_714,female,AD.vs.MCI; Homo_714,male,AD.vs.MCI; Homo_714,female,MCI.vs.control; Homo_633,female,AD.vs.control; Homo_633,female,AD.vs.MCI; ROSMAP,male,AD.vs.control; ROSMAP,female,MCI.vs.control; Emory,MCI,age; MCSA,MCI,age; Emory,totalSample,immune-Neutrophil; Emory,totalSample,immune-CD8_T; Emory,totalSample,immune-Gamma_delta; Emory,totalSample,immune-Th17; Emory,totalSample,immune-Tfh; Emory,totalSample,immune-CD8_naive; Emory,totalSample,immune-Cytotoxic; Emory,totalSample,immune-Exhausted; Homo_723,totalSample,immune-Exhausted; Homo_723,totalSample,immune-Cytotoxic; Homo_723,totalSample,immune-CD8_T; Homo_723,totalSample,immune-Th17; Homo_723,totalSample,immune-Th1; Homo_723,totalSample,immune-Neutrophil; Homo_633,totalSample,immune-DC; Homo_633,totalSample,immune-Th2; MCSA,totalSample,immune-DC; MCSA,totalSample,immune-Bcell; MCSA,totalSample,immune-Monocyte; MCSA,totalSample,immune-Macrophage; MCSA,totalSample,immune-CD4_T; MCSA,totalSample,immune-CD4_naive; MCSA,totalSample,immune-Tr1; MCSA,totalSample,immune-nTreg; MCSA,totalSample,immune-iTreg; MCSA,totalSample,immune-Th2; MCSA,totalSample,immune-Tfh; MCSA,totalSample,immune-CD8_naive; MCSA,totalSample,immune-Cytotoxic; MCSA,totalSample,immune-MAIT
ADPRS	Homo_723,female,AD.vs.control; Homo_723,male,AD.vs.control; Homo_723,female,AD.vs.MCI; Homo_723,male,AD.vs.MCI; Homo_714,female,AD.vs.control; Homo_714,male,AD.vs.control; Homo_714,female,AD.vs.MCI; Homo_714,male,AD.vs.MCI; Homo_633,female,AD.vs.control; Homo_633,female,AD.vs.MCI; ROSMAP,female,AD.vs.control; Emory,totalSample,age; Emory,MCI,age; MCSA,totalSample,age; MCSA,control,age; MCSA,MCI,age; ROSMAP,MCI,last.visit.age; Emory,totalSample,immune-Gamma_delta; Emory,totalSample,immune-CD8_naive; Emory,totalSample,immune-InfiltrationScore; Homo_723,totalSample,immune-InfiltrationScore; Homo_723,totalSample,immune-Effector_memory; Homo_723,totalSample,immune-Bcell; Homo_723,totalSample,immune-Th1; Homo_723,totalSample,immune-Cytotoxic; Homo_723,totalSample,immune-Th2; Homo_723,totalSample,immune-Tfh; Homo_714,totalSample,immune-InfiltrationScore; Homo_714,totalSample,immune-Effector_memory; Homo_714,totalSample,immune-Th2; Homo_714,totalSample,immune-Th1; Homo_714,totalSample,immune-Tfh; Homo_633,totalSample,immune-InfiltrationScore; Homo_633,totalSample,immune-DC; Homo_633,totalSample,immune-Effector_memory; Homo_633,totalSample,immune-Exhausted; Homo_633,totalSample,immune-Th1; MCSA,totalSample,immune-DC; MCSA,totalSample,immune-Monocyte; MCSA,totalSample,immune-Macrophage; MCSA,totalSample,immune-CD8_T; MCSA,totalSample,immune-NKT; MCSA,totalSample,immune-Th2; MCSA,totalSample,immune-Cytotoxic; MCSA,totalSample,immune-Effector_memory; MCSA,totalSample,immune-InfiltrationScore; ROSMAP,totalSample,immune-Th2; ROSMAP,totalSample,immune-Th17; ROSMAP,totalSample,immune-Effector_memory;
MTARC1	Homo_723,female,AD.vs.control; Homo_723,male,AD.vs.control; Homo_723,female,AD.vs.MCI; Homo_723,male,AD.vs.MCI; Homo_714,female,AD.vs.control; Homo_714,male,AD.vs.control; Homo_714,female,AD.vs.MCI; Homo_714,male,AD.vs.MCI; Homo_633,female,AD.vs.control; Homo_633,female,AD.vs.MCI; Emory,totalSample,age; Emory,MCI,age; Emory,totalSample,immune-B_cell; Emory,totalSample,immune-Monocyte; Emory,totalSample,immune-Macrophage; Emory,totalSample,immune-Neutrophil; Emory,totalSample,immune-CD4_T; Emory,totalSample,immune-CD8_T; Emory,totalSample,immune-Gamma_delta; Emory,totalSample,immune-nTreg; Emory,totalSample,immune-Th1; Emory,totalSample,immune-Tfh; Emory,totalSample,immune-CD8_naive; Emory,totalSample,immune-Exhausted; Emory,totalSample,immune-MAIT; Homo_723,totalSample,immune-Neutrophil; Homo_723,totalSample,immune-Monocyte; Homo_723,totalSample,immune-DC; Homo_723,totalSample,immune-Gamma_delta; Homo_723,totalSample,immune-Bcell; Homo_723,totalSample,immune-Th1; Homo_723,totalSample,immune-Exhausted; Homo_714,totalSample,immune-Neutrophil; Homo_714,totalSample,immune-Monocyte; Homo_714,totalSample,immune-Gamma_delta; Homo_714,totalSample,immune-Th1; Homo_714,totalSample,immune-Th2; Homo_714,totalSample,immune-Exhausted; Homo_714,totalSample,immune-DC; Homo_633,totalSample,immune-Monocyte; Homo_633,totalSample,immune-Neutrophil; Homo_633,totalSample,immune-Exhausted; Homo_633,totalSample,immune-CD8_T; Homo_633,totalSample,immune-Gamma_delta; Homo_633,totalSample,immune-Macrophage; Homo_633,totalSample,immune-Th2; Homo_633,totalSample,immune-MAIT; Homo_633,totalSample,immune-Th17; MCSA,totalSample,immune-Monocyte; MCSA,totalSample,immune-Macrophage; MCSA,totalSample,immune-Neutrophil; MCSA,totalSample,immune-CD8_T; MCSA,totalSample,immune-Gamma_delta; MCSA,totalSample,immune-CD4_naive; MCSA,totalSample,immune-nTreg; MCSA,totalSample,immune-Th1; MCSA,totalSample,immune-Th17; MCSA,totalSample,immune-Tfh; MCSA,totalSample,immune-Cytotoxic; MCSA,totalSample,immune-Exhausted; MCSA,totalSample,immune-Central_memory; MCSA,totalSample,immune-Effector_memory; MCSA,totalSample,immune-InfiltrationScore; ROSMAP,totalSample,immune-Cytotoxic; ROSMAP,totalSample,immune-Effector_memory; [illegible]
DSG2	Homo_723,female,AD.vs.control; Homo_723,male,AD.vs.control; Homo_723,female,AD.vs.MCI; Homo_723,male,AD.vs.MCI; Homo_714,female,AD.vs.control; Homo_714,male,AD.vs.control; Homo_714,female,AD.vs.MCI; Homo_714,male,AD.vs.MCI; Homo_633,female,AD.vs.MCI; Homo_633,female,MCI.vs.control; Emory,totalSample,immune-Neutrophil; Emory,totalSample,immune-Gamma_delta; Emory,totalSample,immune-Th17; Emory,totalSample,immune-Cytotoxic; Homo_723,totalSample,immune-Cytotoxic; Homo_723,totalSample,immune-Exhausted; Homo_723,totalSample,immune-Th17; Homo_714,totalSample,immune-Exhausted; Homo_633,totalSample,immune-Tfh; Homo_633,totalSample,immune-Exhausted; Homo_633,totalSample,immune-NKT; Homo_633,totalSample,immune-Gamma_delta; Homo_633,totalSample,immune-Th2; MCSA,totalSample,immune-CD4_T; MCSA,totalSample,immune-CD4_naive; MCSA,totalSample,immune-Tr1; MCSA,totalSample,immune-nTreg; MCSA,totalSample,immune-iTreg; MCSA,totalSample,immune-Th1; MCSA,totalSample,immune-Tfh; MCSA,totalSample,immune-Cytotoxic; MCSA,totalSample,immune-Exhausted; MCSA,totalSample,immune-Central_memory; ROSMAP,totalSample,immune-InfiltrationScore
RHEX	Homo_723,female,AD.vs.control; Homo_723,male,AD.vs.control; Homo_723,female,AD.vs.MCI; Homo_723,male,AD.vs.MCI; Homo_714,female,AD.vs.control; Homo_714,male,AD.vs.control; Homo_714,female,AD.vs.MCI; Homo_714,male,AD.vs.MCI; Homo_633,female,AD.vs.control; Homo_633,female,AD.vs.MCI; ROSMAP,MCI,sample.age; ACOM,totalSample,immune-Effector_memory; Emory,totalSample,immune-Gamma_delta; Emory,totalSample,immune-iTreg; Homo_723,totalSample,immune-Neutrophil; Homo_723,totalSample,immune-Gamma_delta; Homo_723,totalSample,immune-Th2; Homo_723,totalSample,immune-Bcell; Homo_714,totalSample,immune-Th2; Homo_633,totalSample,immune-Th2; MCSA,totalSample,immune-Neutrophil; MCSA,totalSample,immune-Th2; ROSMAP,totalSample,immune-InfiltrationScore
TNFSF13	Homo_723,female,AD.vs.control; Homo_723,male,AD.vs.control; Homo_723,female,AD.vs.MCI; Homo_723,male,AD.vs.MCI; Homo_714,female,AD.vs.control; Homo_714,male,AD.vs.control; Homo_714,female,AD.vs.MCI; Homo_714,male,AD.vs.MCI; Homo_633,female,AD.vs.control; Homo_633,female,AD.vs.MCI; Emory,MCI,age; ROSMAP,MCI,sample.age; Emory,totalSample,immune-DC; Emory,totalSample,immune-Macrophage; Emory,totalSample,immune-Neutrophil; Emory,totalSample,immune-CD4_T; Emory,totalSample,immune-CD8_T; Emory,totalSample,immune-Gamma_delta; Emory,totalSample,immune-Tfh; Emory,totalSample,immune-CD8_naive; Emory,totalSample,immune-Exhausted; Homo_723,totalSample,immune-InfiltrationScore; Homo_723,totalSample,immune-Th1; Homo_723,totalSample,immune-Gamma_delta; Homo_723,totalSample,immune-Bcell; Homo_714,totalSample,immune-Th1; Homo_714,totalSample,immune-DC; Homo_633,totalSample,immune-DC; Homo_633,totalSample,immune-Macrophage; Homo_633,totalSample,immune-InfiltrationScore; Homo_633,totalSample,immune-Exhausted; Homo_633,totalSample,immune-MAIT; Homo_633,totalSample,immune-Gamma_delta; MCSA,totalSample,immune-DC; MCSA,totalSample,immune-Monocyte; MCSA,totalSample,immune-Macrophage; MCSA,totalSample,immune-NK; MCSA,totalSample,immune-NKT; MCSA,totalSample,immune-Gamma_delta; MCSA,totalSample,immune-iTreg; MCSA,totalSample,immune-Th2; MCSA,totalSample,immune-InfiltrationScore; ROSMAP,totalSample,immune-Th17; ROSMAP,totalSample,immune-Effector_memory; ROSMAP,female-excludeAge90+,survival
ARMH2	Homo_723,female,AD.vs.control; Homo_723,male,AD.vs.control; Homo_723,female,AD.vs.MCI; Homo_723,male,AD.vs.MCI; Homo_723,female,MCI.vs.control; Homo_714,female,AD.vs.control; Homo_714,male,AD.vs.control; Homo_714,female,AD.vs.MCI; Homo_714,male,AD.vs.MCI; Homo_714,female,MCI.vs.control; Homo_633,female,AD.vs.control; Homo_633,female,AD.vs.MCI; Homo_633,female,MCI.vs.control; ROSMAP,male,AD.vs.control; ROSMAP,MCI,last.visit.age; Homo_723,totalSample,immune-Monocyte; Homo_723,totalSample,immune-DC; Homo_723,totalSample,immune-Th2; Homo_723,totalSample,immune-Neutrophil; Homo_723,totalSample,immune-Gamma_delta; Homo_723,totalSample,immune-Tfh; Homo_714,totalSample,immune-Th2; Homo_714,totalSample,immune-Monocyte; Homo_714,totalSample,immune-Tfh; Homo_714,totalSample,immune-Gamma_delta; Homo_714,totalSample,immune-DC; Homo_633,totalSample,immune-Th2; Homo_633,totalSample,immune-Monocyte; Homo_633,totalSample,immune-Th17; Homo_633,totalSample,immune-Exhausted; Homo_633,totalSample,immune-Gamma_delta; MCSA,totalSample,immune-DC; MCSA,totalSample,immune-Neutrophil; MCSA,totalSample,immune-CD8_T; MCSA,totalSample,immune-NKT; MCSA,totalSample,immune-Gamma_delta; MCSA,totalSample,immune-Th2; MCSA,totalSample,immune-Th17; MCSA,totalSample,immune-Cytotoxic; MCSA,totalSample,immune-Effector_memory; ROSMAP,totalSample,immune-CD4_T; ROSMAP,totalSample,immune-Th17; ROSMAP,totalSample,immune-InfiltrationScore; ROSMAP,male-excludeAge90+,survival; ROSMAP,male and female-excludeAge90+,survival

DHRS12	Homo_723,female,AD.vs.control; Homo_723,male,AD.vs.control; Homo_723,female,AD.vs.MCI; Homo_723,male,AD.vs.MCI; Homo_714,female,AD.vs.control; Homo_714,male,AD.vs.control; Homo_714,female,AD.vs.MCI; Homo_714,male,AD.vs.MCI; Homo_633,female,AD.vs.control; Homo_633,female,AD.vs.MCI; ROSMAP,male,AD.vs.control; ROSMAP,male,AD.vs.MCI; ACOM,totalSample,age; Emory,MCI,age; Emory,totalSample,immune-Neutrophil; Emory,totalSample,immune-CD8_T; Emory,totalSample,immune-Gamma_delta; Emory,totalSample,immune-Tfh; Emory,totalSample,immune-CD8_naive; Emory,totalSample,immune-Cytotoxic; Emory,totalSample,immune-Exhausted; Homo_723,totalSample,immune-Neutrophil; Homo_723,totalSample,immune-Gamma_delta; Homo_723,totalSample,immune-Th1; Homo_723,totalSample,immune-Bcell; Homo_714,totalSample,immune-Neutrophil; Homo_714,totalSample,immune-Th2; Homo_714,totalSample,immune-Gamma_delta; Homo_633,totalSample,immune-Neutrophil; Homo_633,totalSample,immune-MAIT; Homo_633,totalSample,immune-Exhausted; Homo_633,totalSample,immune-Gamma_delta; Homo_633,totalSample,immune-Th17; Homo_633,totalSample,immune-CD8_T; MCSA,totalSample,immune-DC; MCSA,totalSample,immune-Neutrophil; MCSA,totalSample,immune-NKT; MCSA,totalSample,immune-Gamma_delta; MCSA,totalSample,immune-Th17; MCSA,totalSample,immune-CD8_naive; MCSA,totalSample,immune-MAIT; ROSMAP,totalSample,immune-Effector_memory; SRP223445,totalSample,immune-CD4_naive; SRP223445,totalSample,immune-Th17; ROSMAP,female-includeAge90+,survival; ROSMAP,male and female-includeAge90+,survival
CYSLTR2	Homo_723,female,AD.vs.control; Homo_723,male,AD.vs.control; Homo_723,female,AD.vs.MCI; Homo_723,male,AD.vs.MCI; Homo_723,female,MCI.vs.control; Homo_714,female,AD.vs.control; Homo_714,male,AD.vs.control; Homo_714,female,AD.vs.MCI; Homo_714,male,AD.vs.MCI; Homo_714,female,MCI.vs.control; Homo_633,female,AD.vs.control; Homo_633,female,AD.vs.MCI; Homo_633,female,MCI.vs.control; ROSMAP,female,AD.vs.control; ROSMAP,female,AD.vs.MCI; MCSA,MCI,age; Emory,totalSample,immune-Gamma_delta; Emory,totalSample,immune-Tr1; Emory,totalSample,immune-iTreg; Homo_723,totalSample,immune-Monocyte; Homo_723,totalSample,immune-Th2; Homo_723,totalSample,immune-Tfh; Homo_723,totalSample,immune-DC; Homo_723,totalSample,immune-iTreg; Homo_723,totalSample,immune-InfiltrationScore; Homo_723,totalSample,immune-Tr1; Homo_723,totalSample,immune-Gamma_delta; Homo_723,totalSample,immune-Effector_memory; Homo_723,totalSample,immune-CD4_T; Homo_723,totalSample,immune-Bcell; Homo_714,totalSample,immune-Th2; Homo_714,totalSample,immune-Tfh; Homo_714,totalSample,immune-Monocyte; Homo_714,totalSample,immune-Tr1; Homo_714,totalSample,immune-CD4_T; Homo_714,totalSample,immune-Effector_memory; Homo_714,totalSample,immune-Gamma_delta; Homo_714,totalSample,immune-iTreg; Homo_633,totalSample,immune-Monocyte; Homo_633,totalSample,immune-Th2; Homo_633,totalSample,immune-Tfh; Homo_633,totalSample,immune-Exhausted; Homo_633,totalSample,immune-Gamma_delta; Homo_633,totalSample,immune-CD4_T; Homo_633,totalSample,immune-Effector_memory; Homo_633,totalSample,immune-Central_memory; Homo_633,totalSample,immune-Th17; Homo_633,totalSample,immune-InfiltrationScore; Homo_633,totalSample,immune-Tr1; Homo_633,totalSample,immune-CD4_naive; MCSA,totalSample,immune-DC; MCSA,totalSample,immune-Monocyte; MCSA,totalSample,immune-CD4_T; MCSA,totalSample,immune-CD8_T; MCSA,totalSample,immune-CD4_naive; MCSA,totalSample,immune-Tr1; MCSA,totalSample,immune-nTreg; MCSA,totalSample,immune-iTreg; MCSA,totalSample,immune-Tfh; MCSA,totalSample,immune-Cytotoxic; MCSA,totalSample,immune-Exhausted; MCSA,totalSample,immune-Central_memory; SRP223445,totalSample,immune-Neutrophil; SRP223445,totalSample,immune-InfiltrationScore
TMEM45B	Homo_723,female,AD.vs.control; Homo_723,male,AD.vs.control; Homo_723,female,AD.vs.MCI; Homo_723,male,AD.vs.MCI; Homo_714,female,AD.vs.control; Homo_714,male,AD.vs.control; Homo_714,female,AD.vs.MCI; Homo_714,male,AD.vs.MCI; Homo_633,female,AD.vs.control; Homo_633,female,AD.vs.MCI; Emory,MCI,age; ROSMAP,MCI,last.visit.age; ROSMAP,MCI,sample.age; Emory,totalSample,immune-DC; Emory,totalSample,immune-B_cell; Emory,totalSample,immune-Monocyte; Emory,totalSample,immune-NK; Emory,totalSample,immune-Neutrophil; Emory,totalSample,immune-CD8_T; Emory,totalSample,immune-Gamma_delta; Emory,totalSample,immune-Th17; Emory,totalSample,immune-Tfh; Emory,totalSample,immune-CD8_naive; Emory,totalSample,immune-Cytotoxic; Emory,totalSample,immune-Exhausted; Emory,totalSample,immune-InfiltrationScore; Homo_723,totalSample,immune-Neutrophil; Homo_723,totalSample,immune-Gamma_delta; Homo_723,totalSample,immune-DC; Homo_723,totalSample,immune-Th2; Homo_723,totalSample,immune-iTreg; Homo_723,totalSample,immune-Monocyte; Homo_723,totalSample,immune-Tfh; Homo_723,totalSample,immune-Bcell; Homo_714,totalSample,immune-Th2; Homo_714,totalSample,immune-Neutrophil; Homo_714,totalSample,immune-Gamma_delta; Homo_714,totalSample,immune-Tfh; Homo_714,totalSample,immune-DC; Homo_633,totalSample,immune-Exhausted; Homo_633,totalSample,immune-Th2; Homo_633,totalSample,immune-Gamma_delta; Homo_633,totalSample,immune-Th17; Homo_633,totalSample,immune-Neutrophil; Homo_633,totalSample,immune-Monocyte; Homo_633,totalSample,immune-CD8_T; Homo_633,totalSample,immune-Cytotoxic; Homo_633,totalSample,immune-DC; MCSA,totalSample,immune-DC; MCSA,totalSample,immune-Neutrophil; MCSA,totalSample,immune-CD8_T; MCSA,totalSample,immune-Gamma_delta; MCSA,totalSample,immune-Tr1; MCSA,totalSample,immune-iTreg; MCSA,totalSample,immune-Th17; MCSA,totalSample,immune-Cytotoxic; MCSA,totalSample,immune-Exhausted; MCSA,totalSample,immune-InfiltrationScore
CLP1	Homo_723,female,AD.vs.control; Homo_723,male,AD.vs.control; Homo_723,female,AD.vs.MCI; Homo_723,male,AD.vs.MCI; Homo_714,female,AD.vs.control; Homo_714,female,AD.vs.MCI; Homo_714,male,AD.vs.MCI; Homo_633,female,AD.vs.control; Homo_633,female,AD.vs.MCI; ROSMAP,female,AD.vs.control; Emory,MCI,age; ROSMAP,MCI,last.visit.age; Emory,totalSample,immune-Gamma_delta; Emory,totalSample,immune-Tr1; Emory,totalSample,immune-iTreg; Emory,totalSample,immune-CD8_naive; Emory,totalSample,immune-InfiltrationScore; Homo_723,totalSample,immune-Th2; Homo_723,totalSample,immune-Tfh; Homo_723,totalSample,immune-InfiltrationScore; Homo_723,totalSample,immune-iTreg; Homo_723,totalSample,immune-Effector_memory; Homo_723,totalSample,immune-Bcell; Homo_723,totalSample,immune-Monocyte; Homo_723,totalSample,immune-Tr1; Homo_723,totalSample,immune-DC; Homo_723,totalSample,immune-CD4_T; Homo_723,totalSample,immune-Gamma_delta; Homo_723,totalSample,immune-CD4_naive; Homo_714,totalSample,immune-Th2; Homo_714,totalSample,immune-Tfh; Homo_714,totalSample,immune-Effector_memory; Homo_714,totalSample,immune-InfiltrationScore; Homo_714,totalSample,immune-Tr1; Homo_714,totalSample,immune-CD4_T; Homo_714,totalSample,immune-Gamma_delta; Homo_714,totalSample,immune-Monocyte; Homo_714,totalSample,immune-Central_memory; Homo_714,totalSample,immune-iTreg; Homo_633,totalSample,immune-Th2; Homo_633,totalSample,immune-InfiltrationScore; Homo_633,totalSample,immune-Tfh; Homo_633,totalSample,immune-Exhausted; Homo_633,totalSample,immune-Effector_memory; Homo_633,totalSample,immune-CD4_T; Homo_633,totalSample,immune-Monocyte; Homo_633,totalSample,immune-Th17; Homo_633,totalSample,immune-Gamma_delta; Homo_633,totalSample,immune-CD4_naive; Homo_633,totalSample,immune-Central_memory; Homo_633,totalSample,immune-Tr1; Homo_633,totalSample,immune-Bcell; MCSA,totalSample,immune-CD4_T; MCSA,totalSample,immune-CD8_T; MCSA,totalSample,immune-Gamma_delta; MCSA,totalSample,immune-CD4_naive; MCSA,totalSample,immune-Tr1; MCSA,totalSample,immune-nTreg; MCSA,totalSample,immune-iTreg; MCSA,totalSample,immune-Th17; MCSA,totalSample,immune-Tfh; MCSA,totalSample,immune-Cytotoxic; MCSA,totalSample,immune-Exhausted; MCSA,totalSample,immune-[illegible]
SDC4	Homo_723,female,AD.vs.control; Homo_723,male,AD.vs.control; Homo_723,female,AD.vs.MCI; Homo_723,male,AD.vs.MCI; Homo_714,female,AD.vs.control; Homo_714,male,AD.vs.control; Homo_714,female,AD.vs.MCI; Homo_714,male,AD.vs.MCI; Homo_633,female,AD.vs.control; Homo_633,female,AD.vs.MCI; ROSMAP,female,AD.vs.control; ACOM,totalSample,age; Emory,totalSample,immune-Tr1; Emory,totalSample,immune-iTreg; Homo_723,totalSample,immune-Th17; Homo_723,totalSample,immune-Exhausted; Homo_723,totalSample,immune-DC; Homo_723,totalSample,immune-Cytotoxic; Homo_723,totalSample,immune-CD8_T; Homo_714,totalSample,immune-Th17; Homo_714,totalSample,immune-Exhausted; Homo_633,totalSample,immune-InfiltrationScore; Homo_633,totalSample,immune-Th2; Homo_633,totalSample,immune-Tfh; Homo_633,totalSample,immune-Th17; Homo_633,totalSample,immune-Gamma_delta; MCSA,totalSample,immune-Th17; ROSMAP,totalSample,immune-CD8_T; ROSMAP,totalSample,immune-Cytotoxic; ROSMAP,totalSample,immune-Central_memory; ROSMAP,totalSample,immune-Effector_memory; SRP223445,totalSample,immune-CD4_naive; SRP223445,totalSample,immune-Exhausted
MTLN	Homo_723,female,AD.vs.control; Homo_723,male,AD.vs.control; Homo_723,female,AD.vs.MCI; Homo_723,male,AD.vs.MCI; Homo_714,female,AD.vs.control; Homo_714,male,AD.vs.control; Homo_714,female,AD.vs.MCI; Homo_714,male,AD.vs.MCI; Homo_633,female,AD.vs.control; Homo_633,female,AD.vs.MCI; ROSMAP,female,AD.vs.control; ROSMAP,control,male.vs.female; ACOM,totalSample,age; Emory,MCI,age; MCSA,MCI,age; Emory,totalSample,immune-Macrophage; Emory,totalSample,immune-Neutrophil; Emory,totalSample,immune-CD4_T; Emory,totalSample,immune-CD8_T; Emory,totalSample,immune-Tr1; Emory,totalSample,immune-iTreg; Emory,totalSample,immune-Tfh; Emory,totalSample,immune-Exhausted; Emory,totalSample,immune-Effector_memory; Homo_723,totalSample,immune-CD8_T; Homo_723,totalSample,immune-Cytotoxic; Homo_723,totalSample,immune-Exhausted; Homo_723,totalSample,immune-Th17; Homo_723,totalSample,immune-MAIT; Homo_723,totalSample,immune-InfiltrationScore; Homo_723,totalSample,immune-Macrophage; Homo_723,totalSample,immune-Monocyte; Homo_723,totalSample,immune-Central_memory; Homo_723,totalSample,immune-nTreg; Homo_723,totalSample,immune-iTreg; Homo_723,totalSample,immune-Effector_memory; Homo_714,totalSample,immune-CD8_T; Homo_714,totalSample,immune-Exhausted; Homo_714,totalSample,immune-Cytotoxic; Homo_714,totalSample,immune-Monocyte; Homo_714,totalSample,immune-Th17; Homo_714,totalSample,immune-Effector_memory; Homo_714,totalSample,immune-DC; Homo_714,totalSample,immune-nTreg; Homo_714,totalSample,immune-Macrophage; Homo_714,totalSample,immune-MAIT; Homo_714,totalSample,immune-Neutrophil; Homo_714,totalSample,immune-InfiltrationScore; Homo_714,totalSample,immune-CD8_naive; Homo_633,totalSample,immune-DC; Homo_633,totalSample,immune-CD8_T; Homo_633,totalSample,immune-Neutrophil; Homo_633,totalSample,immune-InfiltrationScore; Homo_633,totalSample,immune-nTreg; Homo_633,totalSample,immune-Monocyte; Homo_633,totalSample,immune-Th2; Homo_633,totalSample,immune-Cytotoxic; Homo_633,totalSample,immune-Effector_memory; MCSA,totalSample,immune-DC; MCSA,totalSample,immune-Monocyte; MCSA,totalSample,immune-Neutrophil; MCSA,totalSample,immune-CD8_T; MCSA,totalSample,immune-NKT; MCSA,totalSample,immune-iTreg; MCSA,totalSample,immune-Th2; MCSA,totalSample,immune-Cytotoxic; MCSA,totalSample,immune-Central_memory; MCSA,totalSample,immune-Effector_memory; MCSA,totalSample,immune-InfiltrationScore; ROSMAP,totalSample,immune-Effector_memory; ROSMAP,totalSample,immune-InfiltrationScore
DEGS1	Homo_723,female,AD.vs.control; Homo_723,male,AD.vs.control; Homo_723,female,AD.vs.MCI; Homo_723,male,AD.vs.MCI; Homo_714,female,AD.vs.control; Homo_714,male,AD.vs.control; Homo_714,female,AD.vs.MCI; Homo_714,male,AD.vs.MCI; Homo_633,female,AD.vs.control; Homo_633,female,AD.vs.MCI; ROSMAP,female,AD.vs.control; ROSMAP,female,MCI.vs.control; Emory,totalSample,immune-DC; Emory,totalSample,immune-Monocyte; Emory,totalSample,immune-Neutrophil; Emory,totalSample,immune-Gamma_delta; Emory,totalSample,immune-Tr1; Emory,totalSample,immune-iTreg; Emory,totalSample,immune-Th1; Emory,totalSample,immune-CD8_naive; Emory,totalSample,immune-Cytotoxic; Emory,totalSample,immune-Exhausted; Homo_723,totalSample,immune-Th2; Homo_723,totalSample,immune-iTreg; Homo_723,totalSample,immune-Gamma_delta; Homo_723,totalSample,immune-Tfh; Homo_723,totalSample,immune-DC; Homo_723,totalSample,immune-Tr1; Homo_723,totalSample,immune-CD4_T; Homo_723,totalSample,immune-InfiltrationScore; Homo_723,totalSample,immune-Neutrophil; Homo_723,totalSample,immune-CD4_naive; Homo_723,totalSample,immune-Bcell; Homo_723,totalSample,immune-Monocyte; Homo_723,totalSample,immune-Effector_memory; Homo_714,totalSample,immune-Th2; Homo_714,totalSample,immune-Tfh; Homo_714,totalSample,immune-Gamma_delta; Homo_714,totalSample,immune-CD4_T; Homo_714,totalSample,immune-Tr1; Homo_714,totalSample,immune-iTreg; Homo_714,totalSample,immune-Effector_memory; Homo_714,totalSample,immune-DC; Homo_714,totalSample,immune-Central_memory; Homo_714,totalSample,immune-CD4_naive; Homo_714,totalSample,immune-InfiltrationScore; Homo_633,totalSample,immune-Th2; Homo_633,totalSample,immune-Tfh; Homo_633,totalSample,immune-Gamma_delta; Homo_633,totalSample,immune-Exhausted; Homo_633,totalSample,immune-CD4_T; Homo_633,totalSample,immune-Th17; Homo_633,totalSample,immune-InfiltrationScore; Homo_633,totalSample,immune-NKT; Homo_633,totalSample,immune-CD4_naive; Homo_633,totalSample,immune-Tr1; Homo_633,totalSample,immune-Central_memory; Homo_633,totalSample,immune-Monocyte; Homo_633,totalSample,immune-Effector_memory; Homo_633,totalSample,immune-iTreg; Homo_633,totalSample,immune-DC; Homo_633,totalSample,immune-Cytotoxic; Homo_633,totalSample,immune-CD8_T; MCSA,totalSample,immune-DC; MCSA,totalSample,immune-Neutrophil; MCSA,totalSample,immune-CD4_T; MCSA,totalSample,immune-CD8_T; MCSA,totalSample,immune-Gamma_delta; MCSA,totalSample,immune-CD4_naive; MCSA,totalSample,immune-Tr1; MCSA,totalSample,immune-nTreg; MCSA,totalSample,immune-iTreg; MCSA,totalSample,immune-Th1; [illegible]
TRIM34	Homo_723,female,AD.vs.control; Homo_723,male,AD.vs.control; Homo_723,female,AD.vs.MCI; Homo_723,male,AD.vs.MCI; Homo_714,female,AD.vs.control; Homo_714,male,AD.vs.control; Homo_714,female,AD.vs.MCI; Homo_714,male,AD.vs.MCI; Homo_633,female,AD.vs.control; Homo_633,female,AD.vs.MCI; Homo_633,female,MCI.vs.control; ROSMAP,female,AD.vs.control; MCSA,MCI,age; Emory,totalSample,immune-CD8_T; Emory,totalSample,immune-Gamma_delta; Emory,totalSample,immune-Tr1; Emory,totalSample,immune-iTreg; Emory,totalSample,immune-CD8_naive; Emory,totalSample,immune-Cytotoxic; Emory,totalSample,immune-Exhausted; Homo_723,totalSample,immune-Monocyte; Homo_723,totalSample,immune-Th2; Homo_723,totalSample,immune-DC; Homo_723,totalSample,immune-Tfh; Homo_723,totalSample,immune-iTreg; Homo_723,totalSample,immune-Gamma_delta; Homo_723,totalSample,immune-InfiltrationScore; Homo_723,totalSample,immune-Effector_memory; Homo_723,totalSample,immune-Tr1; Homo_723,totalSample,immune-Exhausted; Homo_723,totalSample,immune-Bcell; Homo_723,totalSample,immune-CD4_T; Homo_723,totalSample,immune-Neutrophil; Homo_714,totalSample,immune-Th2; Homo_714,totalSample,immune-Tfh; Homo_714,totalSample,immune-Monocyte; Homo_714,totalSample,immune-Gamma_delta; Homo_714,totalSample,immune-Tr1; Homo_714,totalSample,immune-Effector_memory; Homo_714,totalSample,immune-CD4_T; Homo_633,totalSample,immune-Th2; Homo_633,totalSample,immune-Monocyte; Homo_633,totalSample,immune-Exhausted; Homo_633,totalSample,immune-Tfh; Homo_633,totalSample,immune-Gamma_delta; Homo_633,totalSample,immune-Th17; Homo_633,totalSample,immune-CD4_T; Homo_633,totalSample,immune-Effector_memory; Homo_633,totalSample,immune-InfiltrationScore; Homo_633,totalSample,immune-Central_memory; Homo_633,totalSample,immune-Tr1; MCSA,totalSample,immune-DC; MCSA,totalSample,immune-Monocyte; MCSA,totalSample,immune-Neutrophil; MCSA,totalSample,immune-CD4_T; MCSA,totalSample,immune-CD8_T; MCSA,totalSample,immune-Gamma_delta; MCSA,totalSample,immune-CD4_naive; MCSA,totalSample,immune-Tr1; MCSA,totalSample,immune-nTreg; MCSA,totalSample,immune-iTreg; MCSA,totalSample,immune-Th17; MCSA,totalSample,immune-Cytotoxic; MCSA,totalSample,immune-Exhausted; MCSA,totalSample,immune-Effector_memory; ROSMAP,totalSample,immune-Th17; ROSMAP,totalSample,immune-CD8_naive; ROSMAP,totalSample,immune-Effector_memory; ROSMAP,totalSample,immune-InfiltrationScore; SRP223445,totalSample,immune-Exhausted; SRP223445,totalSample,immune-InfiltrationScore; ROSMAP,female-excludeAge90+,survival; ROSMAP,male-excludeAge90+,survival
PNPLA1	Homo_723,female,AD.vs.control; Homo_723,male,AD.vs.control; Homo_723,female,AD.vs.MCI; Homo_723,male,AD.vs.MCI; Homo_714,female,AD.vs.control; Homo_714,male,AD.vs.control; Homo_714,female,AD.vs.MCI; Homo_714,male,AD.vs.MCI; Homo_633,female,AD.vs.control; Homo_633,female,AD.vs.MCI; ROSMAP,female,AD.vs.control; ROSMAP,male,AD.vs.MCI; ROSMAP,female,MCI.vs.control; Emory,MCI,age; Emory,totalSample,immune-B_cell; Emory,totalSample,immune-Macrophage; Emory,totalSample,immune-NK; Emory,totalSample,immune-Neutrophil; Emory,totalSample,immune-CD4_T; Emory,totalSample,immune-CD8_T; Emory,totalSample,immune-Gamma_delta; Emory,totalSample,immune-Th1; Emory,totalSample,immune-Th17; Emory,totalSample,immune-Tfh; Emory,totalSample,immune-CD8_naive; Emory,totalSample,immune-Cytotoxic; Emory,totalSample,immune-Exhausted; Emory,totalSample,immune-InfiltrationScore; Homo_723,totalSample,immune-Neutrophil; Homo_723,totalSample,immune-Gamma_delta; Homo_723,totalSample,immune-Th1; Homo_723,totalSample,immune-Bcell; Homo_714,totalSample,immune-Neutrophil; Homo_714,totalSample,immune-Th1; Homo_714,totalSample,immune-Gamma_delta; Homo_714,totalSample,immune-Exhausted; Homo_714,totalSample,immune-MAIT; Homo_633,totalSample,immune-Neutrophil; Homo_633,totalSample,immune-Exhausted; Homo_633,totalSample,immune-MAIT; Homo_633,totalSample,immune-CD8_T; Homo_633,totalSample,immune-Gamma_delta; Homo_633,totalSample,immune-Macrophage; Homo_633,totalSample,immune-Th1; MCSA,totalSample,immune-Macrophage; MCSA,totalSample,immune-Neutrophil; MCSA,totalSample,immune-Gamma_delta;
DACH1	Homo_723,female,AD.vs.control; Homo_723,male,AD.vs.control; Homo_723,female,AD.vs.MCI; Homo_723,male,AD.vs.MCI; Homo_714,female,AD.vs.control; Homo_714,male,AD.vs.control; Homo_714,female,AD.vs.MCI; Homo_714,male,AD.vs.MCI; Homo_633,female,AD.vs.control; Homo_633,female,AD.vs.MCI; Emory,totalSample,age; Emory,MCI,age; ROSMAP,AD,last.visit.age; Emory,totalSample,immune-B_cell; Emory,totalSample,immune-Macrophage; Emory,totalSample,immune-Neutrophil; Emory,totalSample,immune-CD4_T; Emory,totalSample,immune-CD8_T; Emory,totalSample,immune-Gamma_delta; Emory,totalSample,immune-Th1; Emory,totalSample,immune-Tfh; Emory,totalSample,immune-CD8_naive; Emory,totalSample,immune-Exhausted; Emory,totalSample,immune-MAIT; Emory,totalSample,immune-Effector_memory; Homo_723,totalSample,immune-Cytotoxic; Homo_723,totalSample,immune-CD8_T; Homo_723,totalSample,immune-Th17; Homo_723,totalSample,immune-Exhausted; Homo_723,totalSample,immune-Gamma_delta; Homo_723,totalSample,immune-NKT; Homo_723,totalSample,immune-Macrophage; Homo_714,totalSample,immune-CD8_T; Homo_714,totalSample,immune-Exhausted; Homo_714,totalSample,immune-Cytotoxic; Homo_714,totalSample,immune-NKT; Homo_714,totalSample,immune-Gamma_delta; Homo_714,totalSample,immune-Th17; Homo_633,totalSample,immune-Gamma_delta; Homo_633,totalSample,immune-CD8_T; Homo_633,totalSample,immune-NKT; Homo_633,totalSample,immune-Cytotoxic; Homo_633,totalSample,immune-Exhausted; Homo_633,totalSample,immune-Neutrophil; MCSA,totalSample,immune-DC; MCSA,totalSample,immune-Monocyte; MCSA,totalSample,immune-Neutrophil; MCSA,totalSample,immune-CD4_T; MCSA,totalSample,immune-CD8_T; MCSA,totalSample,immune-Gamma_delta; MCSA,totalSample,immune-Tr1; MCSA,totalSample,immune-nTreg; MCSA,totalSample,immune-iTreg; MCSA,totalSample,immune-Th1; MCSA,totalSample,immune-Th17; MCSA,totalSample,immune-Cytotoxic; MCSA,totalSample,immune-Exhausted; MCSA,totalSample,immune-[illegible]
GRHL1	Homo_723,female,AD.vs.control; Homo_723,male,AD.vs.control; Homo_723,female,AD.vs.MCI; Homo_723,male,AD.vs.MCI; Homo_723,female,MCI.vs.control; Homo_723,male,MCI.vs.control; Homo_714,female,AD.vs.control; Homo_714,male,AD.vs.control; Homo_714,female,AD.vs.MCI; Homo_714,male,AD.vs.MCI; Homo_714,female,MCI.vs.control; Homo_714,male,MCI.vs.control; Homo_633,female,AD.vs.control; Homo_633,female,AD.vs.MCI; SRP223445,female,AD.vs.control; ROSMAP,MCI,last.visit.age; Emory,totalSample,immune-Monocyte; Emory,totalSample,immune-Neutrophil; Emory,totalSample,immune-CD8_T; Emory,totalSample,immune-Exhausted; Homo_723,totalSample,immune-Exhausted; Homo_723,totalSample,immune-CD8_T; Homo_723,totalSample,immune-Cytotoxic; Homo_723,totalSample,immune-Th17; Homo_714,totalSample,immune-Exhausted; Homo_714,totalSample,immune-CD8_T; Homo_714,totalSample,immune-Th17; Homo_714,totalSample,immune-Monocyte; Homo_714,totalSample,immune-Cytotoxic; Homo_633,totalSample,immune-Gamma_delta; Homo_633,totalSample,immune-Exhausted; Homo_633,totalSample,immune-Tfh; Homo_633,totalSample,immune-NKT; Homo_633,totalSample,immune-Monocyte; MCSA,totalSample,immune-DC; MCSA,totalSample,immune-Monocyte; MCSA,totalSample,immune-CD4_T; MCSA,totalSample,immune-CD8_T; MCSA,totalSample,immune-Tr1; MCSA,totalSample,immune-iTreg; MCSA,totalSample,immune-Cytotoxic; MCSA,totalSample,immune-Exhausted; ROSMAP,totalSample,immune-InfiltrationScore; SRP223445,totalSample,immune-CD4_naive
VEGFA	Homo_723,female,AD.vs.control; Homo_723,male,AD.vs.control; Homo_723,female,AD.vs.MCI; Homo_723,male,AD.vs.MCI; Homo_714,female,AD.vs.control; Homo_633,female,AD.vs.control; Homo_633,female,AD.vs.MCI; ROSMAP,female,AD.vs.control; ACOM,totalSample,age; Emory,totalSample,age; Emory,MCI,age; MCSA,totalSample,age; MCSA,control,age; Emory,totalSample,immune-Monocyte; Emory,totalSample,immune-Macrophage; Emory,totalSample,immune-Neutrophil; Emory,totalSample,immune-CD8_T; Emory,totalSample,immune-Gamma_delta; Emory,totalSample,immune-Th1; Emory,totalSample,immune-Th17; Emory,totalSample,immune-Tfh; Emory,totalSample,immune-CD8_naive; Emory,totalSample,immune-Cytotoxic; Emory,totalSample,immune-Exhausted; Homo_723,totalSample,immune-Gamma_delta; Homo_723,totalSample,immune-InfiltrationScore; Homo_723,totalSample,immune-Cytotoxic; Homo_714,totalSample,immune-Exhausted; Homo_714,totalSample,immune-Gamma_delta; Homo_714,totalSample,immune-CD8_T; Homo_714,totalSample,immune-InfiltrationScore; Homo_714,totalSample,immune-NKT; Homo_714,totalSample,immune-Cytotoxic; Homo_633,totalSample,immune-Gamma_delta; Homo_633,totalSample,immune-InfiltrationScore; Homo_633,totalSample,immune-Exhausted; Homo_633,totalSample,immune-NKT; Homo_633,totalSample,immune-CD8_T; Homo_633,totalSample,immune-Cytotoxic; Homo_633,totalSample,immune-iTreg; MCSA,totalSample,immune-CD4_T; MCSA,totalSample,immune-NKT; MCSA,totalSample,immune-Gamma_delta; MCSA,totalSample,immune-CD4_naive; MCSA,totalSample,immune-Tr1; MCSA,totalSample,immune-nTreg; MCSA,totalSample,immune-iTreg; MCSA,totalSample,immune-Th1; MCSA,totalSample,immune-Th2; MCSA,totalSample,immune-Th17; MCSA,totalSample,immune-Tfh; MCSA,totalSample,immune-Cytotoxic; MCSA,totalSample,immune-Exhausted; MCSA,totalSample,immune-Central_memory; ROSMAP,totalSample,immune-Exhausted; ROSMAP,totalSample,immune-Effector_memory
ICOS	Homo_723,female,AD.vs.control; Homo_723,male,AD.vs.control; Homo_723,female,AD.vs.MCI; Homo_723,male,AD.vs.MCI; Homo_723,female,MCI.vs.control; Homo_714,female,AD.vs.control; Homo_714,male,AD.vs.control; Homo_714,female,AD.vs.MCI; Homo_714,male,AD.vs.MCI; Homo_714,female,MCI.vs.control; Homo_633,female,AD.vs.control; Homo_633,female,AD.vs.MCI; Homo_633,female,MCI.vs.control; Homo_633,male,MCI.vs.control; ROSMAP,female,AD.vs.control; ROSMAP,female,MCI.vs.control; Emory,totalSample,age; Emory,control,age; ROSMAP,AD,sample.age; ROSMAP,totalSample,last.visit.age; ROSMAP,totalSample,age; ROSMAP,control,sample.age; ROSMAP,AD,MMSE; Emory,totalSample,immune-DC; Emory,totalSample,immune-Macrophage; Emory,totalSample,immune-Neutrophil; Emory,totalSample,immune-CD4_T; Emory,totalSample,immune-CD8_T; Emory,totalSample,immune-Tr1; Emory,totalSample,immune-iTreg; Emory,totalSample,immune-Tfh; Emory,totalSample,immune-CD8_naive; Emory,totalSample,immune-Central_memory; Emory,totalSample,immune-InfiltrationScore; Homo_723,totalSample,immune-Tfh; Homo_723,totalSample,immune-iTreg; Homo_723,totalSample,immune-Th2; Homo_723,totalSample,immune-Tr1; Homo_723,totalSample,immune-CD4_T; Homo_723,totalSample,immune-DC; Homo_723,totalSample,immune-CD4_naive; Homo_723,totalSample,immune-Central_memory; Homo_723,totalSample,immune-Effector_memory; Homo_723,totalSample,immune-Monocyte; Homo_723,totalSample,immune-nTreg; Homo_723,totalSample,immune-Gamma_delta; Homo_723,totalSample,immune-Macrophage; Homo_723,totalSample,immune-InfiltrationScore; Homo_723,totalSample,immune-MAIT; Homo_723,totalSample,immune-Bcell; Homo_714,totalSample,immune-Tfh; Homo_714,totalSample,immune-Th2; Homo_714,totalSample,immune-CD4_T; Homo_714,totalSample,immune-Tr1; Homo_714,totalSample,immune-Central_memory; Homo_714,totalSample,immune-iTreg; Homo_714,totalSample,immune-CD4_naive; Homo_714,totalSample,immune-Effector_memory; Homo_714,totalSample,immune-nTreg; Homo_714,totalSample,immune-DC; Homo_714,totalSample,immune-Gamma_delta; Homo_714,totalSample,immune-MAIT; Homo_714,totalSample,immune-Monocyte; Homo_714,totalSample,immune-Macrophage; Homo_633,totalSample,immune-Tfh; Homo_633,totalSample,immune-CD4_T; Homo_633,totalSample,immune-Central_memory; Homo_633,totalSample,immune-Th2; Homo_633,totalSample,immune-CD4_naive; Homo_633,totalSample,immune-Tr1; Homo_633,totalSample,immune-Effector_memory; Homo_633,totalSample,immune-iTreg; Homo_633,totalSample,immune-nTreg; Homo_633,totalSample,immune-Monocyte; Homo_633,totalSample,immune-Gamma_delta; Homo_633,totalSample,immune-Th17; Homo_633,totalSample,immune-DC; Homo_633,totalSample,immune-NKT; Homo_633,totalSample,immune-InfiltrationScore; Homo_633,totalSample,immune-Bcell; Homo_633,totalSample,immune-Exhausted; Homo_633,totalSample,immune-MAIT; MCSA,totalSample,immune-DC; MCSA,totalSample,immune-Bcell; MCSA,totalSample,immune-Macrophage; MCSA,totalSample,immune-Neutrophil; MCSA,totalSample,immune-CD4_T; MCSA,totalSample,immune-CD4_naive; MCSA,totalSample,immune-Tr1; MCSA,totalSample,immune-nTreg; MCSA,totalSample,immune-iTreg; MCSA,totalSample,immune-Th17; MCSA,totalSample,immune-Tfh; MCSA,totalSample,immune-CD8_naive; [illegible]

DHRS7B	Homo_723,female,AD.vs.control; Homo_723,male,AD.vs.control; Homo_723,female,AD.vs.MCI; Homo_723,male,AD.vs.MCI; Homo_714,female,AD.vs.control; Homo_714,male,AD.vs.control; Homo_714,female,AD.vs.MCI; Homo_714,male,AD.vs.MCI; Homo_633,female,AD.vs.control; Homo_633,female,AD.vs.MCI; Emory,MCI,age; ROSMAP,AD,last.visit.age; ROSMAP,AD,sample.age; ROSMAP,MCI,last.visit.age; ROSMAP,MCI,sample.age; Emory,totalSample,immune-Neutrophil; Emory,totalSample,immune-CD8_T; Emory,totalSample,immune-Gamma_delta; Emory,totalSample,immune-Th17; Emory,totalSample,immune-Tfh; Emory,totalSample,immune-CD8_naive; Emory,totalSample,immune-Cytotoxic; Emory,totalSample,immune-Exhausted; Homo_723,totalSample,immune-Monocyte; Homo_723,totalSample,immune-InfiltrationScore; Homo_723,totalSample,immune-Bcell; Homo_723,totalSample,immune-Th1; Homo_723,totalSample,immune-Effector_memory; Homo_723,totalSample,immune-Neutrophil; Homo_723,totalSample,immune-Th2; Homo_723,totalSample,immune-Gamma_delta; Homo_714,totalSample,immune-Th1; Homo_714,totalSample,immune-Th2; Homo_714,totalSample,immune-Monocyte; Homo_714,totalSample,immune-InfiltrationScore; Homo_633,totalSample,immune-Exhausted; Homo_633,totalSample,immune-Monocyte; Homo_633,totalSample,immune-InfiltrationScore; Homo_633,totalSample,immune-Th2; Homo_633,totalSample,immune-Th1; MCSA,totalSample,immune-DC; MCSA,totalSample,immune-Bcell; MCSA,totalSample,immune-Monocyte; MCSA,totalSample,immune-Macrophage; MCSA,totalSample,immune-Neutrophil; MCSA,totalSample,immune-Gamma_delta; MCSA,totalSample,immune-Th2; MCSA,totalSample,immune- [illegible]
TPX2	Homo_723,female,AD.vs.control; Homo_723,male,AD.vs.control; Homo_723,female,AD.vs.MCI; Homo_723,male,AD.vs.MCI; Homo_723,female,MCI.vs.control; Homo_723,male,MCI.vs.control; Homo_714,female,AD.vs.control; Homo_714,male,AD.vs.control; Homo_714,female,AD.vs.MCI; Homo_714,male,AD.vs.MCI; Homo_714,female,MCI.vs.control; Homo_633,female,AD.vs.control; Homo_633,female,AD.vs.MCI; ACOM,AD,age; Emory,totalSample,immune-DC; Emory,totalSample,immune-NK; Emory,totalSample,immune-Neutrophil; Emory,totalSample,immune-InfiltrationScore; Homo_723,totalSample,immune-InfiltrationScore; Homo_723,totalSample,immune-Th17; Homo_723,totalSample,immune-CD4_naive; Homo_723,totalSample,immune-Exhausted; Homo_723,totalSample,immune-Neutrophil; Homo_723,totalSample,immune-CD4_T; Homo_723,totalSample,immune-nTreg; Homo_723,totalSample,immune-Tfh; Homo_723,totalSample,immune-Cytotoxic; Homo_723,totalSample,immune-DC; Homo_723,totalSample,immune-Tr1; Homo_723,totalSample,immune-CD8_T; Homo_723,totalSample,immune-Central_memory; Homo_723,totalSample,immune-Th1; Homo_723,totalSample,immune-CD8_naive; Homo_714,totalSample,immune-InfiltrationScore; Homo_714,totalSample,immune-CD4_naive; Homo_633,totalSample,immune-InfiltrationScore; Homo_633,totalSample,immune-Tfh; Homo_633,totalSample,immune-Effector_memory; Homo_633,totalSample,immune-CD4_T; Homo_633,totalSample,immune-CD4_naive; Homo_633,totalSample,immune-Tr1; Homo_633,totalSample,immune-Th2; Homo_633,totalSample,immune-Central_memory; Homo_633,totalSample,immune-nTreg; MCSA,totalSample,immune-Monocyte; MCSA,totalSample,immune-CD4_T; MCSA,totalSample,immune-CD4_naive; MCSA,totalSample,immune-Tr1; MCSA,totalSample,immune-nTreg; MCSA,totalSample,immune-iTreg; MCSA,totalSample,immune-Th17; MCSA,totalSample,immune-Tfh; MCSA,totalSample,immune-Central_memory; MCSA,totalSample,immune-InfiltrationScore; ROSMAP,totalSample,immune-InfiltrationScore; SRP223445,totalSample,immune-NK;
EZH2	Homo_723,female,AD.vs.control; Homo_723,male,AD.vs.control; Homo_723,female,AD.vs.MCI; Homo_723,male,AD.vs.MCI; Homo_723,female,MCI.vs.control; Homo_714,female,AD.vs.control; Homo_714,male,AD.vs.control; Homo_714,female,AD.vs.MCI; Homo_714,male,AD.vs.MCI; Homo_633,female,AD.vs.control; Homo_633,female,AD.vs.MCI; MCSA,totalSample,age; MCSA,control,age; ROSMAP,MCI,sample.age; Emory,totalSample,immune-Macrophage; Emory,totalSample,immune-NK; Emory,totalSample,immune-Neutrophil; Emory,totalSample,immune-CD4_T; Emory,totalSample,immune-CD8_T; Emory,totalSample,immune-Tr1; Emory,totalSample,immune-iTreg; Emory,totalSample,immune-Tfh; Emory,totalSample,immune-Exhausted; Emory,totalSample,immune-Central_memory; Emory,totalSample,immune-Effector_memory; Emory,totalSample,immune-InfiltrationScore; Homo_723,totalSample,immune-CD4_naive; Homo_723,totalSample,immune-CD4_T; Homo_723,totalSample,immune-nTreg; Homo_723,totalSample,immune-Th17; Homo_723,totalSample,immune-Central_memory; Homo_723,totalSample,immune-Tr1; Homo_723,totalSample,immune-Tfh; Homo_723,totalSample,immune-InfiltrationScore; Homo_723,totalSample,immune-Th1; Homo_723,totalSample,immune-Cytotoxic; Homo_723,totalSample,immune-Neutrophil; Homo_723,totalSample,immune-DC; Homo_723,totalSample,immune-iTreg; Homo_723,totalSample,immune-Th2; Homo_714,totalSample,immune-CD4_naive; Homo_714,totalSample,immune-InfiltrationScore; Homo_714,totalSample,immune-CD4_T; Homo_714,totalSample,immune-Th1; Homo_714,totalSample,immune-nTreg; Homo_714,totalSample,immune-Tfh; Homo_714,totalSample,immune-Th17; Homo_714,totalSample,immune-Tr1; Homo_714,totalSample,immune-Central_memory; Homo_714,totalSample,immune-iTreg; Homo_714,totalSample,immune-NKT; Homo_714,totalSample,immune-Effector_memory; Homo_633,totalSample,immune-Tfh; Homo_633,totalSample,immune-CD4_T; Homo_633,totalSample,immune-Tr1; Homo_633,totalSample,immune-InfiltrationScore; Homo_633,totalSample,immune-CD4_naive; Homo_633,totalSample,immune-Effector_memory; Homo_633,totalSample,immune-nTreg; Homo_633,totalSample,immune-iTreg; Homo_633,totalSample,immune-Central_memory; Homo_633,totalSample,immune-Th2; Homo_633,totalSample,immune-Th1; Homo_633,totalSample,immune-NKT; MCSA,totalSample,immune-Monocyte; MCSA,totalSample,immune-CD4_T; MCSA,totalSample,immune-Gamma_delta; MCSA,totalSample,immune-CD4_naive; MCSA,totalSample,immune-Tr1; MCSA,totalSample,immune-nTreg; MCSA,totalSample,immune-iTreg; MCSA,totalSample,immune-Th17; MCSA,totalSample,immune-Tfh; MCSA,totalSample,immune-CD8_naive; MCSA,totalSample,immune- [illegible]
ATP9A	Homo_723,female,AD.vs.control; Homo_723,male,AD.vs.control; Homo_723,female,AD.vs.MCI; Homo_723,male,AD.vs.MCI; Homo_723,female,MCI.vs.control; Homo_723,male,MCI.vs.control; Homo_714,female,AD.vs.control; Homo_714,male,AD.vs.control; Homo_714,female,AD.vs.MCI; Homo_714,male,AD.vs.MCI; Homo_714,female,MCI.vs.control; Homo_714,male,MCI.vs.control; Homo_633,female,AD.vs.control; Homo_633,female,AD.vs.MCI; ROSMAP,female,MCI.vs.control; Emory,totalSample,immune-B_cell; Emory,totalSample,immune-Tr1; Emory,totalSample,immune-iTreg; Emory,totalSample,immune-Cytotoxic; Homo_723,totalSample,immune-Th17; Homo_723,totalSample,immune-Exhausted; Homo_723,totalSample,immune-CD8_T; Homo_723,totalSample,immune-Cytotoxic; Homo_723,totalSample,immune-CD4_naive; Homo_714,totalSample,immune-Th17; Homo_714,totalSample,immune-Exhausted; Homo_714,totalSample,immune-InfiltrationScore; Homo_714,totalSample,immune-CD8_T; Homo_633,totalSample,immune-InfiltrationScore; Homo_633,totalSample,immune-Tfh; Homo_633,totalSample,immune-Th2; Homo_633,totalSample,immune-Th17; MCSA,totalSample,immune-CD4_T; MCSA,totalSample,immune-Tr1; MCSA,totalSample,immune-iTreg; MCSA,totalSample,immune-Th1; MCSA,totalSample,immune-Th17; MCSA,totalSample,immune-Exhausted; ROSMAP,totalSample,immune-
PHF11	Homo_723,female,AD.vs.control; Homo_723,male,AD.vs.control; Homo_723,female,AD.vs.MCI; Homo_723,male,AD.vs.MCI; Homo_714,female,AD.vs.control; Homo_714,male,AD.vs.control; Homo_714,female,AD.vs.MCI; Homo_714,male,AD.vs.MCI; Homo_633,female,AD.vs.control; Homo_633,female,AD.vs.MCI; Homo_633,female,MCI.vs.control; Emory,totalSample,immune-Macrophage; Emory,totalSample,immune-NK; Emory,totalSample,immune-CD4_T; Emory,totalSample,immune-Tr1; Emory,totalSample,immune-iTreg; Emory,totalSample,immune-Effector_memory; Emory,totalSample,immune-InfiltrationScore; Homo_723,totalSample,immune-Th2; Homo_723,totalSample,immune-Monocyte; Homo_723,totalSample,immune-Tfh; Homo_723,totalSample,immune-DC; Homo_723,totalSample,immune-iTreg; Homo_723,totalSample,immune-InfiltrationScore; Homo_723,totalSample,immune-Effector_memory; Homo_723,totalSample,immune-Tr1; Homo_723,totalSample,immune-CD4_T; Homo_723,totalSample,immune-Bcell; Homo_714,totalSample,immune-Th2; Homo_714,totalSample,immune-Tfh; Homo_714,totalSample,immune-Monocyte; Homo_714,totalSample,immune-CD4_T; Homo_714,totalSample,immune-Effector_memory; Homo_714,totalSample,immune-Tr1; Homo_633,totalSample,immune-Th2; Homo_633,totalSample,immune-Monocyte; Homo_633,totalSample,immune-Tfh; Homo_633,totalSample,immune-Th17; Homo_633,totalSample,immune-CD4_T; Homo_633,totalSample,immune-InfiltrationScore; Homo_633,totalSample,immune-Bcell; Homo_633,totalSample,immune-Exhausted; MCSA,totalSample,immune-DC; MCSA,totalSample,immune-Macrophage; MCSA,totalSample,immune-CD4_T; MCSA,totalSample,immune-CD8_T; MCSA,totalSample,immune-NKT; MCSA,totalSample,immune-CD4_naive; MCSA,totalSample,immune-Tr1; MCSA,totalSample,immune-nTreg; MCSA,totalSample,immune-iTreg; MCSA,totalSample,immune-Th2; MCSA,totalSample,immune-Th17; MCSA,totalSample,immune-Cytotoxic; SRP223445,totalSample,immune-InfiltrationScore
NAPSA	Homo_723,female,AD.vs.control; Homo_723,male,AD.vs.control; Homo_723,female,AD.vs.MCI; Homo_723,male,AD.vs.MCI; Homo_714,female,AD.vs.control; Homo_714,male,AD.vs.control; Homo_714,female,AD.vs.MCI; Homo_714,male,AD.vs.MCI; Homo_633,female,AD.vs.control; Homo_633,female,AD.vs.MCI; ROSMAP,female,AD.vs.control; ROSMAP,male,AD.vs.control; MCSA,totalSample,age; MCSA,control,age; Emory,totalSample,immune-NK; Emory,totalSample,immune-Neutrophil; Emory,totalSample,immune-Tr1; Emory,totalSample,immune-iTreg; Emory,totalSample,immune-InfiltrationScore; Homo_723,totalSample,immune-Th2; Homo_723,totalSample,immune-Tfh; Homo_723,totalSample,immune-iTreg; Homo_723,totalSample,immune-Effector_memory; Homo_723,totalSample,immune-CD8_T; Homo_723,totalSample,immune-InfiltrationScore; Homo_723,totalSample,immune-Macrophage; Homo_723,totalSample,immune-nTreg; Homo_723,totalSample,immune-CD4_T; Homo_714,totalSample,immune-Th2; Homo_714,totalSample,immune-Tfh; Homo_714,totalSample,immune-Effector_memory; Homo_714,totalSample,immune-nTreg; Homo_714,totalSample,immune-CD4_T; Homo_714,totalSample,immune-Central_memory; Homo_714,totalSample,immune-CD8_T; Homo_633,totalSample,immune-nTreg; Homo_633,totalSample,immune-Effector_memory; Homo_633,totalSample,immune-Th2; Homo_633,totalSample,immune-Tfh; Homo_633,totalSample,immune-Central_memory; Homo_633,totalSample,immune-InfiltrationScore; Homo_633,totalSample,immune-CD4_T; MCSA,totalSample,immune-DC; MCSA,totalSample,immune-Monocyte; MCSA,totalSample,immune-Neutrophil; MCSA,totalSample,immune-CD4_T; MCSA,totalSample,immune-CD8_T; MCSA,totalSample,immune-CD4_naive; MCSA,totalSample,immune-nTreg; MCSA,totalSample,immune-Tfh; MCSA,totalSample,immune-Central_memory; MCSA,totalSample,immune-Effector_memory; MCSA,totalSample,immune-InfiltrationScore; ROSMAP,totalSample,immune-Effector_memory; SRP223445,totalSample,immune-InfiltrationScore; ROSMAP,female-
GNAT2	Homo_723,female,AD.vs.control; Homo_723,male,AD.vs.control; Homo_723,female,AD.vs.MCI; Homo_723,male,AD.vs.MCI; Homo_714,female,AD.vs.control; Homo_714,male,AD.vs.control; Homo_714,female,AD.vs.MCI; Homo_714,male,AD.vs.MCI; Homo_633,female,AD.vs.control; Homo_633,female,AD.vs.MCI; ROSMAP,male,MCI.vs.control; MCSA,MCI,age; ROSMAP,MCI,last.visit.age; ROSMAP,MCI,sample.age; Emory,totalSample,immune-DC; Emory,totalSample,immune-Monocyte; Emory,totalSample,immune-Neutrophil; Emory,totalSample,immune-CD8_T; Emory,totalSample,immune-Gamma_delta; Emory,totalSample,immune-Tr1; Emory,totalSample,immune-iTreg; Emory,totalSample,immune-Th1; Emory,totalSample,immune-Th17; Emory,totalSample,immune-Cytotoxic; Emory,totalSample,immune-Exhausted; Homo_723,totalSample,immune-Neutrophil; Homo_723,totalSample,immune-Gamma_delta; Homo_723,totalSample,immune-DC; Homo_723,totalSample,immune-iTreg; Homo_723,totalSample,immune-Th2; Homo_723,totalSample,immune-InfiltrationScore; Homo_723,totalSample,immune-CD8_naive; Homo_723,totalSample,immune-Tfh; Homo_723,totalSample,immune-Bcell; Homo_714,totalSample,immune-Th2; Homo_714,totalSample,immune-Gamma_delta; Homo_714,totalSample,immune-Tfh; Homo_714,totalSample,immune-Neutrophil; Homo_714,totalSample,immune-DC; Homo_714,totalSample,immune-iTreg; Homo_633,totalSample,immune-Exhausted; Homo_633,totalSample,immune-Gamma_delta; Homo_633,totalSample,immune-NKT; Homo_633,totalSample,immune-Th2; Homo_633,totalSample,immune-Neutrophil; Homo_633,totalSample,immune-CD8_T; Homo_633,totalSample,immune-DC; Homo_633,totalSample,immune-Th17; Homo_633,totalSample,immune-InfiltrationScore; Homo_633,totalSample,immune-Tfh; Homo_633,totalSample,immune-Cytotoxic; MCSA,totalSample,immune-DC; MCSA,totalSample,immune-Neutrophil; MCSA,totalSample,immune-CD4_T; MCSA,totalSample,immune-CD8_T; MCSA,totalSample,immune-Gamma_delta; MCSA,totalSample,immune-Tr1; MCSA,totalSample,immune-iTreg;
HAPLN3	Homo_723,female,AD.vs.control; Homo_723,male,AD.vs.control; Homo_723,female,AD.vs.MCI; Homo_723,male,AD.vs.MCI; Homo_714,female,AD.vs.control; Homo_714,male,AD.vs.control; Homo_714,female,AD.vs.MCI; Homo_714,male,AD.vs.MCI; Homo_633,female,AD.vs.control; Homo_633,female,AD.vs.MCI; MCSA,totalSample,age; MCSA,control,age; MCSA,MCI,age; ROSMAP,AD,last.visit.age; Emory,totalSample,immune-Macrophage; Emory,totalSample,immune-Neutrophil; Emory,totalSample,immune-CD4_T; Emory,totalSample,immune-CD8_T; Emory,totalSample,immune-CD4_naive; Emory,totalSample,immune-Tr1; Emory,totalSample,immune-nTreg; Emory,totalSample,immune-iTreg; Emory,totalSample,immune-Th17; Emory,totalSample,immune-Tfh; Emory,totalSample,immune-CD8_naive; Emory,totalSample,immune-Cytotoxic; Emory,totalSample,immune-MAIT; Emory,totalSample,immune-Central_memory; Homo_723,totalSample,immune-CD8_T; Homo_723,totalSample,immune-iTreg; Homo_723,totalSample,immune-Tfh; Homo_723,totalSample,immune-nTreg; Homo_723,totalSample,immune-Central_memory; Homo_723,totalSample,immune-CD4_T; Homo_723,totalSample,immune-CD4_naive; Homo_723,totalSample,immune-Tr1; Homo_723,totalSample,immune-Th2; Homo_723,totalSample,immune-Exhausted; Homo_723,totalSample,immune-Macrophage; Homo_723,totalSample,immune-Effector_memory; Homo_723,totalSample,immune-InfiltrationScore; Homo_723,totalSample,immune-Monocyte; Homo_723,totalSample,immune-Gamma_delta; Homo_723,totalSample,immune-MAIT; Homo_714,totalSample,immune-MAIT; Homo_714,totalSample,immune-nTreg; Homo_714,totalSample,immune-Tfh; Homo_714,totalSample,immune-Central_memory; Homo_714,totalSample,immune-iTreg; Homo_714,totalSample,immune-Tr1; Homo_714,totalSample,immune-CD4_T; Homo_714,totalSample,immune-Effector_memory; Homo_714,totalSample,immune-Th2; Homo_714,totalSample,immune-CD4_naive; Homo_714,totalSample,immune-Monocyte; Homo_714,totalSample,immune-CD8_T; Homo_714,totalSample,immune-Macrophage; Homo_633,totalSample,immune-nTreg; Homo_633,totalSample,immune-Central_memory; Homo_633,totalSample,immune-Tfh; Homo_633,totalSample,immune-Tr1; Homo_633,totalSample,immune-iTreg; Homo_633,totalSample,immune-CD4_T; Homo_633,totalSample,immune-CD4_naive; Homo_633,totalSample,immune-Effector_memory; Homo_633,totalSample,immune-Monocyte; Homo_633,totalSample,immune-DC; Homo_633,totalSample,immune-InfiltrationScore; Homo_633,totalSample,immune-Neutrophil; MCSA,totalSample,immune-DC; MCSA,totalSample,immune-Monocyte; MCSA,totalSample,immune-Neutrophil; MCSA,totalSample,immune-CD4_T; MCSA,totalSample,immune-CD8_T; MCSA,totalSample,immune-NKT; MCSA,totalSample,immune-Gamma_delta; MCSA,totalSample,immune-CD4_naive; MCSA,totalSample,immune-Tr1; MCSA,totalSample,immune-nTreg; MCSA,totalSample,immune-Th2; MCSA,totalSample,immune-Th17; MCSA,totalSample,immune-Tfh; MCSA,totalSample,immune-CD8_naive; MCSA,totalSample,immune-Cytotoxic; MCSA,totalSample,immune- [illegible]
HERC5	Homo_723,female,AD.vs.control; Homo_723,male,AD.vs.control; Homo_723,female,AD.vs.MCI; Homo_723,male,AD.vs.MCI; Homo_723,female,MCI.vs.control; Homo_723,male,MCI.vs.control; Homo_714,female,AD.vs.control; Homo_714,male,AD.vs.control; Homo_714,female,AD.vs.MCI; Homo_714,male,AD.vs.MCI; Homo_714,female,MCI.vs.control; Homo_714,male,MCI.vs.control; Homo_633,female,AD.vs.control; Homo_633,female,AD.vs.MCI; Homo_633,female,MCI.vs.control; Homo_633,male,MCI.vs.control; ROSMAP,MCI,last.visit.age; Emory,totalSample,immune-NKT; Emory,totalSample,immune-InfiltrationScore; Homo_723,totalSample,immune-Monocyte; Homo_723,totalSample,immune-Th2; Homo_723,totalSample,immune-Tfh; Homo_723,totalSample,immune-InfiltrationScore; Homo_723,totalSample,immune-Bcell; Homo_723,totalSample,immune-DC; Homo_723,totalSample,immune-iTreg; Homo_723,totalSample,immune-Gamma_delta; Homo_714,totalSample,immune-Th2; Homo_714,totalSample,immune-Tfh; Homo_714,totalSample,immune-Monocyte; Homo_633,totalSample,immune-Th2; Homo_633,totalSample,immune-Monocyte; Homo_633,totalSample,immune-Tfh; Homo_633,totalSample,immune-Th17; Homo_633,totalSample,immune-Bcell; MCSA,totalSample,immune-CD8_T; MCSA,totalSample,immune-NKT; MCSA,totalSample,immune-Cytotoxic; ROSMAP,totalSample,immune-Effector_memory;
LY9	Homo_723,female,AD.vs.control; Homo_723,male,AD.vs.control; Homo_723,female,AD.vs.MCI; Homo_723,male,AD.vs.MCI; Homo_714,female,AD.vs.control; Homo_714,male,AD.vs.MCI; Homo_714,female,MCI.vs.control; Homo_714,male,MCI.vs.control; Homo_714,AD,male.vs.female; Homo_714,control,male.vs.female; Homo_714,MCI,male.vs.female; Homo_633,male,MCI.vs.control; Homo_633,control,male.vs.female; Homo_633,MCI,male.vs.female; ROSMAP,female,AD.vs.control; ROSMAP,male,AD.vs.control; ROSMAP,female,MCI.vs.control; MCSA,totalSample,age; MCSA,control,age; MCSA,MCI,age; ROSMAP,MCI,last.visit.age; Emory,totalSample,immune-Monocyte; Emory,totalSample,immune-Macrophage; Emory,totalSample,immune-NK; Emory,totalSample,immune-Neutrophil; Emory,totalSample,immune-CD4_T; Emory,totalSample,immune-CD8_T; Emory,totalSample,immune-Tr1; Emory,totalSample,immune-nTreg; Emory,totalSample,immune-iTreg; Emory,totalSample,immune-Tfh; Emory,totalSample,immune-CD8_naive; Emory,totalSample,immune-Exhausted; Emory,totalSample,immune-MAIT; Emory,totalSample,immune-Central_memory; Emory,totalSample,immune-Effector_memory; Emory,totalSample,immune-InfiltrationScore; Homo_723,totalSample,immune-CD8_T; Homo_723,totalSample,immune-Exhausted; Homo_723,totalSample,immune-iTreg; Homo_723,totalSample,immune-Tfh; Homo_723,totalSample,immune-Monocyte; Homo_723,totalSample,immune-Central_memory; Homo_723,totalSample,immune-CD4_naive; Homo_723,totalSample,immune-nTreg; Homo_723,totalSample,immune-CD4_T; Homo_723,totalSample,immune-Macrophage; Homo_723,totalSample,immune-InfiltrationScore; Homo_723,totalSample,immune-Tr1; Homo_723,totalSample,immune-MAIT; Homo_723,totalSample,immune-Th2; Homo_723,totalSample,immune-Cytotoxic; Homo_723,totalSample,immune-Effector_memory; Homo_723,totalSample,immune-CD8_naive; Homo_714,totalSample,immune-Monocyte; Homo_714,totalSample,immune-CD4_naive; Homo_714,totalSample,immune-nTreg; Homo_714,totalSample,immune-Neutrophil; Homo_714,totalSample,immune-Th1; Homo_714,totalSample,immune-Central_memory; Homo_714,totalSample,immune-CD4_T; Homo_714,totalSample,immune-Tfh; Homo_714,totalSample,immune-iTreg; Homo_714,totalSample,immune-NKT; Homo_714,totalSample,immune-Macrophage; Homo_633,totalSample,immune-Monocyte; Homo_633,totalSample,immune-CD4_naive; Homo_633,totalSample,immune-nTreg; Homo_633,totalSample,immune-Tfh; Homo_633,totalSample,immune-iTreg; Homo_633,totalSample,immune-CD4_T; Homo_633,totalSample,immune-Central_memory; Homo_633,totalSample,immune-Th1; Homo_633,totalSample,immune-NKT; Homo_633,totalSample,immune-Exhausted; Homo_633,totalSample,immune-Macrophage; MCSA,totalSample,immune-Monocyte; MCSA,totalSample,immune-Macrophage; MCSA,totalSample,immune-Neutrophil; MCSA,totalSample,immune-CD4_T; MCSA,totalSample,immune-CD8_T; MCSA,totalSample,immune-CD4_naive; MCSA,totalSample,immune-Tr1; MCSA,totalSample,immune-nTreg; MCSA,totalSample,immune-iTreg; MCSA,totalSample,immune-Th17; MCSA,totalSample,immune-Tfh; MCSA,totalSample,immune-CD8_naive; MCSA,totalSample,immune-Cytotoxic; MCSA,totalSample,immune-MAIT; MCSA,totalSample,immune-Central_memory; MCSA,totalSample,immune-Effector_memory; MCSA,totalSample,immune-InfiltrationScore; ROSMAP,totalSample,immune-CD4_naive; [illegible]
GZMM	Homo_723,female,AD.vs.control; Homo_723,male,AD.vs.control; Homo_723,female,AD.vs.MCI; Homo_723,male,AD.vs.MCI; Homo_714,female,AD.vs.control; Homo_714,male,AD.vs.control; Homo_714,female,AD.vs.MCI; Homo_714,male,AD.vs.MCI; Homo_633,female,AD.vs.control; Homo_633,female,AD.vs.MCI; ROSMAP,female,AD.vs.control; ROSMAP,male,AD.vs.control; MCSA,MCI,age; Emory,totalSample,immune-Macrophage; Emory,totalSample,immune-NK; Emory,totalSample,immune-Neutrophil; Emory,totalSample,immune-CD4_T; Emory,totalSample,immune-CD8_T; Emory,totalSample,immune-Gamma_delta; Emory,totalSample,immune-Tr1; Emory,totalSample,immune-iTreg; Emory,totalSample,immune-Tfh; Emory,totalSample,immune-Cytotoxic; Emory,totalSample,immune-Exhausted; Emory,totalSample,immune-MAIT; Emory,totalSample,immune-Effector_memory; Emory,totalSample,immune-InfiltrationScore; Homo_723,totalSample,immune-Cytotoxic; Homo_723,totalSample,immune-CD8_T; Homo_723,totalSample,immune-InfiltrationScore; Homo_723,totalSample,immune-Exhausted; Homo_723,totalSample,immune-Effector_memory; Homo_723,totalSample,immune-Th17; Homo_723,totalSample,immune-MAIT; Homo_723,totalSample,immune-Macrophage; Homo_723,totalSample,immune-DC; Homo_723,totalSample,immune-Th1; Homo_723,totalSample,immune-Neutrophil; Homo_723,totalSample,immune-Monocyte; Homo_723,totalSample,immune-Bcell; Homo_714,totalSample,immune-CD8_T; Homo_714,totalSample,immune-Cytotoxic; Homo_714,totalSample,immune-InfiltrationScore; Homo_714,totalSample,immune-DC; Homo_714,totalSample,immune-Effector_memory; Homo_714,totalSample,immune-Exhausted; Homo_714,totalSample,immune-Macrophage; Homo_714,totalSample,immune-Neutrophil; Homo_714,totalSample,immune-Th17; Homo_714,totalSample,immune-Monocyte; Homo_714,totalSample,immune-MAIT; Homo_714,totalSample,immune-Th1; Homo_714,totalSample,immune-Gamma_delta; Homo_714,totalSample,immune-nTreg; Homo_633,totalSample,immune-DC; Homo_633,totalSample,immune-CD8_T; Homo_633,totalSample,immune-Cytotoxic; Homo_633,totalSample,immune-InfiltrationScore; Homo_633,totalSample,immune-Neutrophil; Homo_633,totalSample,immune-Effector_memory; Homo_633,totalSample,immune-Gamma_delta; Homo_633,totalSample,immune-Macrophage; Homo_633,totalSample,immune-Th2; Homo_633,totalSample,immune-Th1; Homo_633,totalSample,immune-Monocyte; Homo_633,totalSample,immune-Th17; Homo_633,totalSample,immune-NK; Homo_633,totalSample,immune-NKT; Homo_633,totalSample,immune-MAIT; Homo_633,totalSample,immune-nTreg; MCSA,totalSample,immune-DC; MCSA,totalSample,immune-Monocyte; MCSA,totalSample,immune-Neutrophil; MCSA,totalSample,immune-CD8_T; MCSA,totalSample,immune-NKT; MCSA,totalSample,immune-Gamma_delta; MCSA,totalSample,immune-iTreg; MCSA,totalSample,immune-Th2; MCSA,totalSample,immune-Th17; MCSA,totalSample,immune-Cytotoxic; MCSA,totalSample,immune-Exhausted; MCSA,totalSample,immune-Central_memory; MCSA,totalSample,immune- [illegible]
MYDGF	Homo_723,female,AD.vs.control; Homo_723,male,AD.vs.control; Homo_723,female,AD.vs.MCI; Homo_723,male,AD.vs.MCI; Homo_714,female,AD.vs.control; Homo_714,male,AD.vs.control; Homo_714,female,AD.vs.MCI; Homo_714,male,AD.vs.MCI; Homo_633,female,AD.vs.control; Homo_633,female,AD.vs.MCI; ROSMAP,female,AD.vs.control; ROSMAP,female,MCI.vs.control; MCSA,MCI,age; ROSMAP,MCI,last.visit.age; ROSMAP,MCI,sample.age; Emory,totalSample,immune-NK; Emory,totalSample,immune-Neutrophil; Emory,totalSample,immune-CD4_T; Emory,totalSample,immune-CD8_T; Emory,totalSample,immune-Tr1; Emory,totalSample,immune-iTreg; Emory,totalSample,immune-Tfh; Emory,totalSample,immune-Exhausted; Emory,totalSample,immune-Effector_memory; Emory,totalSample,immune-InfiltrationScore; Homo_723,totalSample,immune-InfiltrationScore; Homo_723,totalSample,immune-CD8_T; Homo_723,totalSample,immune-Cytotoxic; Homo_723,totalSample,immune-Effector_memory; Homo_723,totalSample,immune-Exhausted; Homo_723,totalSample,immune-MAIT; Homo_723,totalSample,immune-Th17; Homo_723,totalSample,immune-Tfh; Homo_723,totalSample,immune-Bcell; Homo_723,totalSample,immune-iTreg; Homo_714,totalSample,immune-InfiltrationScore; Homo_714,totalSample,immune-Effector_memory; Homo_714,totalSample,immune-CD8_T; Homo_714,totalSample,immune-DC; Homo_714,totalSample,immune-Cytotoxic; Homo_714,totalSample,immune-Th17; Homo_714,totalSample,immune-Neutrophil; Homo_714,totalSample,immune-Tfh; Homo_714,totalSample,immune-MAIT; Homo_714,totalSample,immune-Exhausted; Homo_633,totalSample,immune-InfiltrationScore; Homo_633,totalSample,immune-DC; Homo_633,totalSample,immune-Neutrophil; Homo_633,totalSample,immune-Effector_memory; Homo_633,totalSample,immune-NK; Homo_633,totalSample,immune-CD8_T; Homo_633,totalSample,immune-Cytotoxic; Homo_633,totalSample,immune-Bcell; Homo_633,totalSample,immune-nTreg; MCSA,totalSample,immune-DC; MCSA,totalSample,immune-Monocyte; MCSA,totalSample,immune-NK; MCSA,totalSample,immune-Neutrophil; MCSA,totalSample,immune-CD8_T; MCSA,totalSample,immune-NKT; MCSA,totalSample,immune-iTreg; MCSA,totalSample,immune-Th2; MCSA,totalSample,immune-Cytotoxic; MCSA,totalSample,immune-Exhausted; MCSA,totalSample,immune-Effector_memory; MCSA,totalSample,immune-InfiltrationScore; ROSMAP,totalSample,immune-CD8_naive; ROSMAP,totalSample,immune-Effector_memory; SRP223445,totalSample,immune-CD4_naive; SRP223445,totalSample,immune-Exhausted; SRP223445,totalSample,immune-InfiltrationScore

LMLN	Homo_723,female,AD.vs.control; Homo_723,male,AD.vs.control; Homo_723,female,AD.vs.MCI; Homo_723,male,AD.vs.MCI; Homo_714,female,AD.vs.control; Homo_714,male,AD.vs.control; Homo_714,female,AD.vs.MCI; Homo_714,male,AD.vs.MCI; Homo_633,female,AD.vs.control; Homo_633,female,AD.vs.MCI; ROSMAP,male,AD.vs.control; ROSMAP,female,AD.vs.MCI; ROSMAP,male,AD.vs.MCI; ROSMAP,AD,male.vs.female; ROSMAP,control,male.vs.female; ROSMAP,MCI,male.vs.female; MCSA,totalSample,age; MCSA,control,age; ROSMAP,MCI,last.visit.age; ROSMAP,MCI,sample.age; Emory,totalSample,immune-CD4_T; Emory,totalSample,immune-CD8_T; Emory,totalSample,immune-Tr1; Emory,totalSample,immune-iTreg; Emory,totalSample,immune-MAIT; Emory,totalSample,immune-Central_memory; Emory,totalSample,immune-InfiltrationScore; Homo_723,totalSample,immune-CD4_naive; Homo_723,totalSample,immune-Central_memory; Homo_723,totalSample,immune-CD4_T; Homo_723,totalSample,immune-nTreg; Homo_723,totalSample,immune-Tr1; Homo_723,totalSample,immune-iTreg; Homo_723,totalSample,immune-Tfh; Homo_723,totalSample,immune-Th1; Homo_723,totalSample,immune-Th17; Homo_723,totalSample,immune-Cytotoxic; Homo_723,totalSample,immune-Th2; Homo_723,totalSample,immune-InfiltrationScore; Homo_723,totalSample,immune-Neutrophil; Homo_723,totalSample,immune-NKT; Homo_723,totalSample,immune-Gamma_delta; Homo_714,totalSample,immune-CD4_naive; Homo_714,totalSample,immune-CD4_T; Homo_714,totalSample,immune-Central_memory; Homo_714,totalSample,immune-nTreg; Homo_714,totalSample,immune-Tr1; Homo_714,totalSample,immune-iTreg; Homo_714,totalSample,immune-Tfh; Homo_714,totalSample,immune-Th1; Homo_714,totalSample,immune-NKT; Homo_714,totalSample,immune-InfiltrationScore; Homo_714,totalSample,immune-Th17; Homo_714,totalSample,immune-Effector_memory; Homo_714,totalSample,immune-Cytotoxic; Homo_714,totalSample,immune-Gamma_delta; Homo_633,totalSample,immune-Tfh; Homo_633,totalSample,immune-CD4_T; Homo_633,totalSample,immune-CD4_naive; Homo_633,totalSample,immune-Tr1; Homo_633,totalSample,immune-iTreg; Homo_633,totalSample,immune-Central_memory; Homo_633,totalSample,immune-nTreg; Homo_633,totalSample,immune-Effector_memory; Homo_633,totalSample,immune-NKT; Homo_633,totalSample,immune-InfiltrationScore; Homo_633,totalSample,immune-Gamma_delta; Homo_633,totalSample,immune-Th2; Homo_633,totalSample,immune-Th1; MCSA,totalSample,immune-Neutrophil; MCSA,totalSample,immune-CD4_T; MCSA,totalSample,immune-Gamma_delta; MCSA,totalSample,immune-CD4_naive; MCSA,totalSample,immune-Tr1; MCSA,totalSample,immune-nTreg; MCSA,totalSample,immune-iTreg; MCSA,totalSample,immune-Th17; MCSA,totalSample,immune-Tfh; MCSA,totalSample,immune-CD8_naive; MCSA,totalSample,immune-Exhausted; MCSA,totalSample,immune-Central_memory; MCSA,totalSample,immune-Effector_memory; SRP223445,totalSample,immune-CD4_naive; SRP223445,totalSample,immune-Exhausted
RETREG3	Homo_723,female,AD.vs.control; Homo_723,male,AD.vs.control; Homo_723,female,AD.vs.MCI; Homo_723,male,AD.vs.MCI; Homo_714,female,AD.vs.control; Homo_714,male,AD.vs.control; Homo_714,female,AD.vs.MCI; Homo_714,male,AD.vs.MCI; Homo_633,female,AD.vs.control; Homo_633,female,AD.vs.MCI; MCSA,totalSample,age; MCSA,control,age; Emory,totalSample,immune-Macrophage; Emory,totalSample,immune-NK; Emory,totalSample,immune-Neutrophil; Emory,totalSample,immune-CD4_T; Emory,totalSample,immune-CD8_T; Emory,totalSample,immune-Tr1; Emory,totalSample,immune-iTreg; Emory,totalSample,immune-Central_memory; Emory,totalSample,immune-Effector_memory; Emory,totalSample,immune-InfiltrationScore; Homo_723,totalSample,immune-Th2; Homo_723,totalSample,immune-Tfh; Homo_723,totalSample,immune-iTreg; Homo_723,totalSample,immune-InfiltrationScore; Homo_723,totalSample,immune-CD4_T; Homo_723,totalSample,immune-Macrophage; Homo_723,totalSample,immune-Effector_memory; Homo_723,totalSample,immune-CD4_naive; Homo_723,totalSample,immune-Tr1; Homo_723,totalSample,immune-Bcell; Homo_723,totalSample,immune-nTreg; Homo_723,totalSample,immune-CD8_T; Homo_714,totalSample,immune-Th2; Homo_714,totalSample,immune-Tfh; Homo_714,totalSample,immune-CD4_T; Homo_714,totalSample,immune-Effector_memory; Homo_714,totalSample,immune-InfiltrationScore; Homo_714,totalSample,immune-Tr1; Homo_714,totalSample,immune-Macrophage; Homo_714,totalSample,immune-nTreg; Homo_714,totalSample,immune-CD4_naive; Homo_714,totalSample,immune-iTreg; Homo_714,totalSample,immune-Central_memory; Homo_714,totalSample,immune-Bcell; Homo_714,totalSample,immune-CD8_T; Homo_633,totalSample,immune-InfiltrationScore; Homo_633,totalSample,immune-Th2; Homo_633,totalSample,immune-Tfh; Homo_633,totalSample,immune-CD4_T; Homo_633,totalSample,immune-Effector_memory; Homo_633,totalSample,immune-CD4_naive; Homo_633,totalSample,immune-nTreg; Homo_633,totalSample,immune-Tr1; Homo_633,totalSample,immune-Bcell; Homo_633,totalSample,immune-Central_memory; Homo_633,totalSample,immune-Macrophage; Homo_633,totalSample,immune-iTreg; Homo_633,totalSample,immune-Th17; Homo_633,totalSample,immune-NK; MCSA,totalSample,immune-Monocyte; MCSA,totalSample,immune-Macrophage; MCSA,totalSample,immune-CD4_T; MCSA,totalSample,immune-NKT; MCSA,totalSample,immune-CD4_naive; MCSA,totalSample,immune-Tr1; MCSA,totalSample,immune-nTreg; MCSA,totalSample,immune-iTreg; MCSA,totalSample,immune-Th17; MCSA,totalSample,immune-Tfh; MCSA,totalSample,immune-Effector_memory; MCSA,totalSample,immune- [illegible]
KRT73	Homo_723,female,AD.vs.control; Homo_723,male,AD.vs.control; Homo_723,female,AD.vs.MCI; Homo_723,male,AD.vs.MCI; Homo_714,female,AD.vs.control; Homo_714,male,AD.vs.control; Homo_714,female,AD.vs.MCI; Homo_714,male,AD.vs.MCI; Homo_633,female,AD.vs.control; Homo_633,female,AD.vs.MCI; Emory,totalSample,immune-CD4_T; Emory,totalSample,immune-CD4_naive; Emory,totalSample,immune-Tr1; Emory,totalSample,immune-iTreg; Emory,totalSample,immune-Cytotoxic; Emory,totalSample,immune-Central_memory; Homo_723,totalSample,immune-Tfh; Homo_723,totalSample,immune-iTreg; Homo_723,totalSample,immune-Th2; Homo_723,totalSample,immune-Tr1; Homo_723,totalSample,immune-CD4_T; Homo_723,totalSample,immune-CD8_T; Homo_723,totalSample,immune-CD4_naive; Homo_723,totalSample,immune-Central_memory; Homo_723,totalSample,immune-nTreg; Homo_723,totalSample,immune-Macrophage; Homo_723,totalSample,immune-Effector_memory; Homo_723,totalSample,immune-InfiltrationScore; Homo_723,totalSample,immune-Bcell; Homo_714,totalSample,immune-Tfh; Homo_714,totalSample,immune-Th2; Homo_714,totalSample,immune-Tr1; Homo_714,totalSample,immune-CD4_T; Homo_714,totalSample,immune-nTreg; Homo_714,totalSample,immune-Central_memory; Homo_714,totalSample,immune-Effector_memory; Homo_714,totalSample,immune-iTreg; Homo_714,totalSample,immune-CD4_naive; Homo_714,totalSample,immune-Macrophage; Homo_714,totalSample,immune-CD8_T; Homo_633,totalSample,immune-Tfh; Homo_633,totalSample,immune-nTreg; Homo_633,totalSample,immune-Tr1; Homo_633,totalSample,immune-CD4_T; Homo_633,totalSample,immune-CD4_naive; Homo_633,totalSample,immune-Central_memory; Homo_633,totalSample,immune-Effector_memory; Homo_633,totalSample,immune-Th2; Homo_633,totalSample,immune-iTreg; Homo_633,totalSample,immune-InfiltrationScore; MCSA,totalSample,immune-Monocyte; MCSA,totalSample,immune-CD4_T; MCSA,totalSample,immune-CD4_naive; MCSA,totalSample,immune-Tr1; MCSA,totalSample,immune-nTreg; MCSA,totalSample,immune-iTreg; MCSA,totalSample,immune-Th17; MCSA,totalSample,immune-Tfh; MCSA,totalSample,immune-Central_memory; MCSA,totalSample,immune-Effector_memory
CCNI2	Homo_723,female,AD.vs.control; Homo_723,male,AD.vs.control; Homo_723,female,AD.vs.MCI; Homo_723,male,AD.vs.MCI; Homo_714,female,AD.vs.control; Homo_714,male,AD.vs.control; Homo_714,female,AD.vs.MCI; Homo_714,male,AD.vs.MCI; Homo_633,female,AD.vs.control; Homo_633,female,AD.vs.MCI; ROSMAP,male,AD.vs.control; MCSA,totalSample,age; MCSA,control,age; MCSA,MCI,age; Emory,totalSample,immune-Macrophage; Emory,totalSample,immune-Neutrophil; Emory,totalSample,immune-CD4_T; Emory,totalSample,immune-CD8_T; Emory,totalSample,immune-Tr1; Emory,totalSample,immune-nTreg; Emory,totalSample,immune-iTreg; Emory,totalSample,immune-Th17; Emory,totalSample,immune-Tfh; Emory,totalSample,immune-CD8_naive; Emory,totalSample,immune-Central_memory; Emory,totalSample,immune-InfiltrationScore; Homo_723,totalSample,immune-Tfh; Homo_723,totalSample,immune-iTreg; Homo_723,totalSample,immune-Th2; Homo_723,totalSample,immune-CD4_T; Homo_723,totalSample,immune-Tr1; Homo_723,totalSample,immune-CD4_naive; Homo_723,totalSample,immune-nTreg; Homo_723,totalSample,immune-Central_memory; Homo_723,totalSample,immune-Effector_memory; Homo_723,totalSample,immune-CD8_T; Homo_723,totalSample,immune-Macrophage; Homo_723,totalSample,immune-InfiltrationScore; Homo_723,totalSample,immune-MAIT; Homo_723,totalSample,immune-Gamma_delta; Homo_714,totalSample,immune-Tfh; Homo_714,totalSample,immune-Th2; Homo_714,totalSample,immune-CD4_T; Homo_714,totalSample,immune-nTreg; Homo_714,totalSample,immune-Central_memory; Homo_714,totalSample,immune-Tr1; Homo_714,totalSample,immune-iTreg; Homo_714,totalSample,immune-CD4_naive; Homo_714,totalSample,immune-Effector_memory; Homo_714,totalSample,immune-Macrophage; Homo_714,totalSample,immune-CD8_T; Homo_714,totalSample,immune-MAIT; Homo_714,totalSample,immune-InfiltrationScore; Homo_633,totalSample,immune-Tfh; Homo_633,totalSample,immune-CD4_T; Homo_633,totalSample,immune-nTreg; Homo_633,totalSample,immune-Central_memory; Homo_633,totalSample,immune-CD4_naive; Homo_633,totalSample,immune-Tr1; Homo_633,totalSample,immune-iTreg; Homo_633,totalSample,immune-Effector_memory; Homo_633,totalSample,immune-Th2; Homo_633,totalSample,immune-InfiltrationScore; Homo_633,totalSample,immune-Th17; Homo_633,totalSample,immune-Neutrophil; Homo_633,totalSample,immune-Macrophage; Homo_633,totalSample,immune-Gamma_delta; Homo_633,totalSample,immune-MAIT; MCSA,totalSample,immune-Monocyte; MCSA,totalSample,immune-Macrophage; MCSA,totalSample,immune-Neutrophil; MCSA,totalSample,immune-CD4_T; MCSA,totalSample,immune-CD8_T; MCSA,totalSample,immune-Gamma_delta; MCSA,totalSample,immune-CD4_naive; MCSA,totalSample,immune-Tr1; MCSA,totalSample,immune-nTreg; MCSA,totalSample,immune-iTreg; MCSA,totalSample,immune-Th17; MCSA,totalSample,immune-Tfh; MCSA,totalSample,immune-CD8_naive; MCSA,totalSample,immune-MAIT; MCSA,totalSample,immune- [illegible]
ARG2	Homo_723,female,AD.vs.control; Homo_723,male,AD.vs.control; Homo_723,female,AD.vs.MCI; Homo_723,male,AD.vs.MCI; Homo_723,female,MCI.vs.control; Homo_714,female,AD.vs.control; Homo_714,male,AD.vs.control; Homo_714,female,AD.vs.MCI; Homo_714,male,AD.vs.MCI; Homo_633,female,AD.vs.control; Homo_633,female,AD.vs.MCI; ROSMAP,female,AD.vs.control; ROSMAP,MCI,last.visit.age; ROSMAP,MCI,sample.age; Homo_723,totalSample,immune-Th17; Homo_723,totalSample,immune-DC; Homo_723,totalSample,immune-CD8_T; Homo_633,totalSample,immune-InfiltrationScore; ROSMAP,totalSample,immune-Effector_memory; SRP223445,totalSample,immune-CD4_naive;
CXCR1	Homo_723,female,AD.vs.control; Homo_723,male,AD.vs.control; Homo_723,female,AD.vs.MCI; Homo_723,male,AD.vs.MCI; SRP223445,female,AD.vs.control; SRP223445,male,AD.vs.control; Emory,MCI,age; MCSA,control,age; Emory,totalSample,immune-B_cell; Emory,totalSample,immune-Macrophage; Emory,totalSample,immune-NK; Emory,totalSample,immune-Neutrophil; Emory,totalSample,immune-CD4_T; Emory,totalSample,immune-CD8_T; Emory,totalSample,immune-Gamma_delta; Emory,totalSample,immune-Tr1; Emory,totalSample,immune-Th1; Emory,totalSample,immune-Th17; Emory,totalSample,immune-Tfh; Emory,totalSample,immune-CD8_naive; Emory,totalSample,immune-Cytotoxic; Emory,totalSample,immune-Exhausted; Emory,totalSample,immune-MAIT; Emory,totalSample,immune-Effector_memory; Emory,totalSample,immune-InfiltrationScore; Homo_723,totalSample,immune-Neutrophil; Homo_723,totalSample,immune-Th1; Homo_723,totalSample,immune-Gamma_delta; Homo_723,totalSample,immune-Bcell; Homo_723,totalSample,immune-DC; Homo_714,totalSample,immune-Neutrophil; Homo_714,totalSample,immune-Exhausted; Homo_714,totalSample,immune-MAIT; Homo_714,totalSample,immune-CD8_T; Homo_714,totalSample,immune-Th1; Homo_714,totalSample,immune-Gamma_delta; Homo_714,totalSample,immune-CD4_naive; Homo_714,totalSample,immune-Th17; Homo_714,totalSample,immune-Central_memory; Homo_633,totalSample,immune-Neutrophil; Homo_633,totalSample,immune-CD8_T; Homo_633,totalSample,immune-Exhausted; Homo_633,totalSample,immune-MAIT; Homo_633,totalSample,immune-Th17; Homo_633,totalSample,immune-Gamma_delta; Homo_633,totalSample,immune-Cytotoxic; Homo_633,totalSample,immune-Th1; MCSA,totalSample,immune-DC; MCSA,totalSample,immune-Bcell; MCSA,totalSample,immune-Monocyte; MCSA,totalSample,immune-Macrophage; MCSA,totalSample,immune-Neutrophil; MCSA,totalSample,immune-CD8_T; MCSA,totalSample,immune-Gamma_delta; MCSA,totalSample,immune-Th1; MCSA,totalSample,immune-Th2; MCSA,totalSample,immune-Th17; MCSA,totalSample,immune-CD8_naive; MCSA,totalSample,immune-Exhausted; MCSA,totalSample,immune-MAIT; SRP223445,totalSample,immune-Neutrophil; SRP223445,totalSample,immune-CD4_naive; ROSMAP,male-excludeAge90+,survival
TMCO4	Homo_723,female,AD.vs.control; Homo_723,male,AD.vs.control; Homo_723,female,AD.vs.MCI; Homo_723,male,AD.vs.MCI; Homo_714,female,AD.vs.control; Homo_714,male,AD.vs.control; Homo_714,female,AD.vs.MCI; Homo_714,male,AD.vs.MCI; Homo_633,female,AD.vs.control; Homo_633,female,AD.vs.MCI; ROSMAP,female,AD.vs.control; ROSMAP,male,MCI.vs.control; ROSMAP,MCI,male.vs.female; MCSA,totalSample,age; ROSMAP,AD,last.visit.age; ROSMAP,MCI,sample.age; Emory,totalSample,immune-Monocyte; Emory,totalSample,immune-Macrophage; Emory,totalSample,immune-NK; Emory,totalSample,immune-Neutrophil; Emory,totalSample,immune-CD4_T; Emory,totalSample,immune-CD8_T; Emory,totalSample,immune-Tr1; Emory,totalSample,immune-nTreg; Emory,totalSample,immune-iTreg; Emory,totalSample,immune-Exhausted; Emory,totalSample,immune-Effector_memory; Emory,totalSample,immune-InfiltrationScore; Homo_723,totalSample,immune-Effector_memory; Homo_723,totalSample,immune-InfiltrationScore; Homo_723,totalSample,immune-Tfh; Homo_723,totalSample,immune-CD8_T; Homo_723,totalSample,immune-Cytotoxic; Homo_723,totalSample,immune-nTreg; Homo_723,totalSample,immune-iTreg; Homo_723,totalSample,immune-Th2; Homo_723,totalSample,immune-Macrophage; Homo_723,totalSample,immune-CD4_naive; Homo_723,totalSample,immune-CD4_T; Homo_723,totalSample,immune-Bcell; Homo_723,totalSample,immune-Central_memory; Homo_723,totalSample,immune-MAIT; Homo_714,totalSample,immune-Effector_memory; Homo_714,totalSample,immune-InfiltrationScore; Homo_714,totalSample,immune-CD8_T; Homo_714,totalSample,immune-Tfh; Homo_714,totalSample,immune-nTreg; Homo_714,totalSample,immune-Central_memory; Homo_714,totalSample,immune-Th2; Homo_714,totalSample,immune-Cytotoxic; Homo_714,totalSample,immune-Neutrophil; Homo_714,totalSample,immune-Macrophage; Homo_714,totalSample,immune-CD4_T; Homo_714,totalSample,immune-MAIT; Homo_714,totalSample,immune-CD4_naive; Homo_633,totalSample,immune-InfiltrationScore; Homo_633,totalSample,immune-Effector_memory; Homo_633,totalSample,immune-nTreg; Homo_633,totalSample,immune-Central_memory; Homo_633,totalSample,immune-Neutrophil; Homo_633,totalSample,immune-CD8_T; Homo_633,totalSample,immune-DC; Homo_633,totalSample,immune-Tfh; Homo_633,totalSample,immune-CD4_naive; Homo_633,totalSample,immune-CD4_T; Homo_633,totalSample,immune-Cytotoxic; MCSA,totalSample,immune-DC; MCSA,totalSample,immune-Monocyte; MCSA,totalSample,immune-Neutrophil; MCSA,totalSample,immune-CD4_T; MCSA,totalSample,immune-CD8_T; MCSA,totalSample,immune-NKT; MCSA,totalSample,immune-CD4_naive; MCSA,totalSample,immune-nTreg; MCSA,totalSample,immune-Th2; MCSA,totalSample,immune-Tfh; MCSA,totalSample,immune-Cytotoxic; MCSA,totalSample,immune-MAIT; MCSA,totalSample,immune-Central_memory; MCSA,totalSample,immune-Effector_memory; MCSA,totalSample,immune-InfiltrationScore; ROSMAP,totalSample,immune-Effector_memory; [illegible]
PCDHGB4	Homo_723,female,AD.vs.control; Homo_723,male,AD.vs.control; Homo_723,female,AD.vs.MCI; Homo_723,female,MCI.vs.control; Homo_723,AD,male.vs.female; Homo_714,female,AD.vs.control; Homo_714,male,AD.vs.control; Homo_714,female,MCI.vs.control; Homo_633,female,MCI.vs.control; Homo_633,male,MCI.vs.control; ROSMAP,female,AD.vs.MCI; ROSMAP,MCI,male.vs.female; ROSMAP,MCI,last.visit.age; ROSMAP,MCI,sample.age; Homo_723,totalSample,immune-Monocyte; MCSA,totalSample,immune-CD4_T; MCSA,totalSample,immune-Tr1; MCSA,totalSample,immune-nTreg; MCSA,totalSample,immune-iTreg
DCBLD2	Homo_723,female,AD.vs.control; Homo_723,male,AD.vs.control; Homo_723,female,AD.vs.MCI; Homo_723,male,AD.vs.MCI; Homo_723,female,MCI.vs.control; Homo_723,male,MCI.vs.control; Homo_714,female,AD.vs.control; Homo_714,male,AD.vs.control; Homo_714,female,AD.vs.MCI; Homo_714,male,AD.vs.MCI; Homo_714,male,MCI.vs.control; Homo_633,female,AD.vs.control; Homo_633,female,AD.vs.MCI; MCSA,MCI,age; Emory,totalSample,immune-Gamma_delta; Homo_723,totalSample,immune-Exhausted; Homo_723,totalSample,immune-Th17; Homo_723,totalSample,immune-Cytotoxic; Homo_723,totalSample,immune-CD8_T; Homo_723,totalSample,immune-Monocyte; Homo_723,totalSample,immune-CD4_naive; Homo_723,totalSample,immune-CD4_T; Homo_723,totalSample,immune-Gamma_delta; Homo_723,totalSample,immune-Th2; Homo_723,totalSample,immune-Tr1; Homo_723,totalSample,immune-Th1; Homo_723,totalSample,immune-Tfh; Homo_714,totalSample,immune-Th17; Homo_714,totalSample,immune-Monocyte; Homo_714,totalSample,immune-Exhausted; Homo_714,totalSample,immune-Gamma_delta; Homo_714,totalSample,immune-Cytotoxic; Homo_714,totalSample,immune-CD8_T; Homo_714,totalSample,immune-CD4_naive; Homo_714,totalSample,immune-CD4_T; Homo_633,totalSample,immune-Th2; Homo_633,totalSample,immune-Tfh; Homo_633,totalSample,immune-Monocyte; Homo_633,totalSample,immune-Gamma_delta; Homo_633,totalSample,immune-Th17; Homo_633,totalSample,immune-CD4_T; Homo_633,totalSample,immune-Exhausted; Homo_633,totalSample,immune-DC; Homo_633,totalSample,immune-Central_memory; Homo_633,totalSample,immune-CD4_naive; Homo_633,totalSample,immune-Tr1; MCSA,totalSample,immune-DC; MCSA,totalSample,immune-Monocyte; MCSA,totalSample,immune-CD4_T; MCSA,totalSample,immune-CD8_T; MCSA,totalSample,immune-CD4_naive; MCSA,totalSample,immune-Tr1; MCSA,totalSample,immune-nTreg; MCSA,totalSample,immune-iTreg; MCSA,totalSample,immune-Th2; MCSA,totalSample,immune-Th17; MCSA,totalSample,immune-Tfh; MCSA,totalSample,immune-CD8_naive; MCSA,totalSample,immune-Cytotoxic; MCSA,totalSample,immune-Exhausted; MCSA,totalSample,immune-MAIT; MCSA,totalSample,immune-Central_memory; MCSA,totalSample,immune-Effector_memory; SRP223445,totalSample,immune-CD4_naive
SPOCK1	Homo_723,female,AD.vs.control; Homo_723,male,AD.vs.control; Homo_723,female,AD.vs.MCI; Homo_723,male,AD.vs.MCI; Homo_633,female,AD.vs.control; Homo_633,female,AD.vs.MCI; ROSMAP,female,MCI.vs.control; MCSA,MCI,age; ROSMAP,MCI,sample.age; Emory,totalSample,immune-Neutrophil; Emory,totalSample,immune-CD8_T; Emory,totalSample,immune-Gamma_delta; Emory,totalSample,immune-Tfh; Emory,totalSample,immune-Cytotoxic; Emory,totalSample,immune-Exhausted; Homo_723,totalSample,immune-Gamma_delta; Homo_714,totalSample,immune-Gamma_delta; Homo_714,totalSample,immune-Exhausted; Homo_633,totalSample,immune-Gamma_delta; Homo_633,totalSample,immune-Exhausted; Homo_633,totalSample,immune-CD8_T; Homo_633,totalSample,immune-NKT; Homo_633,totalSample,immune-Cytotoxic; Homo_633,totalSample,immune-InfiltrationScore; MCSA,totalSample,immune-CD4_T; MCSA,totalSample,immune-CD8_T; MCSA,totalSample,immune-Gamma_delta; MCSA,totalSample,immune-Tr1; MCSA,totalSample,immune-iTreg; MCSA,totalSample,immune-Th17; MCSA,totalSample,immune-Cytotoxic; MCSA,totalSample,immune-Exhausted
WNT4	Homo_723,female,AD.vs.control; Homo_723,female,AD.vs.MCI; Homo_714,female,AD.vs.control; Homo_714,male,AD.vs.control; Homo_714,female,AD.vs.MCI; Homo_714,male,AD.vs.MCI; Homo_633,female,AD.vs.control; Homo_633,female,AD.vs.MCI; ROSMAP,female,AD.vs.control; ROSMAP,male,AD.vs.control; ROSMAP,female,MCI.vs.control; ROSMAP,male,MCI.vs.control; ROSMAP,MCI,male.vs.female; MCSA,totalSample,age; MCSA,control,age; MCSA,MCI,age; Homo_723,totalSample,immune-Th2; Homo_714,totalSample,immune-Th2; Homo_633,totalSample,immune-Th2; Homo_633,totalSample,immune-Th17; MCSA,totalSample,immune-DC; MCSA,totalSample,immune-Monocyte;
FAM177B	Homo_723,female,AD.vs.control; Homo_723,male,AD.vs.control; Homo_723,female,AD.vs.MCI; Homo_723,male,AD.vs.MCI; Homo_714,female,AD.vs.control; Homo_714,male,AD.vs.control; Homo_714,female,AD.vs.MCI; Homo_714,male,AD.vs.MCI; Homo_633,female,AD.vs.control; Homo_633,female,AD.vs.MCI; ROSMAP,female,AD.vs.control; ROSMAP,control,male.vs.female; ROSMAP,MCI,last.visit.age; ROSMAP,MCI,sample.age; Emory,totalSample,immune-B_cell; Emory,totalSample,immune-Gamma_delta; Emory,totalSample,immune-Tr1; Emory,totalSample,immune-iTreg; Homo_723,totalSample,immune-iTreg; Homo_723,totalSample,immune-Th2; Homo_723,totalSample,immune-Tfh; Homo_723,totalSample,immune-CD4_T; Homo_723,totalSample,immune-Tr1; Homo_723,totalSample,immune-Gamma_delta; Homo_723,totalSample,immune-DC; Homo_723,totalSample,immune-CD4_naive; Homo_723,totalSample,immune-Central_memory; Homo_723,totalSample,immune-Macrophage; Homo_714,totalSample,immune-Th2; Homo_714,totalSample,immune-Tfh; Homo_714,totalSample,immune-CD4_T; Homo_714,totalSample,immune-Tr1; Homo_714,totalSample,immune-iTreg; Homo_714,totalSample,immune-Gamma_delta; Homo_714,totalSample,immune-Central_memory; Homo_714,totalSample,immune-CD4_naive; Homo_714,totalSample,immune-nTreg; Homo_714,totalSample,immune-Effector_memory; Homo_633,totalSample,immune-Tfh; Homo_633,totalSample,immune-CD4_T; Homo_633,totalSample,immune-Th2; Homo_633,totalSample,immune-CD4_naive; Homo_633,totalSample,immune-Gamma_delta; Homo_633,totalSample,immune-Tr1; Homo_633,totalSample,immune-Central_memory; Homo_633,totalSample,immune-NKT; Homo_633,totalSample,immune-Exhausted; Homo_633,totalSample,immune-iTreg; Homo_633,totalSample,immune-nTreg; Homo_633,totalSample,immune-Th1; Homo_633,totalSample,immune-Th17; MCSA,totalSample,immune-DC; MCSA,totalSample,immune-Bcell; MCSA,totalSample,immune-Macrophage; MCSA,totalSample,immune-CD4_T; MCSA,totalSample,immune-Gamma_delta; MCSA,totalSample,immune-CD4_naive; MCSA,totalSample,immune-Tr1; MCSA,totalSample,immune-nTreg; MCSA,totalSample,immune-iTreg; MCSA,totalSample,immune-Th2; MCSA,totalSample,immune-Th17; MCSA,totalSample,immune-Tfh; MCSA,totalSample,immune-CD8_naive; MCSA,totalSample,immune-Cytotoxic; MCSA,totalSample,immune-Exhausted; MCSA,totalSample,immune-Central_memory; ROSMAP,totalSample,immune-nTreg; ROSMAP,totalSample,immune-Th17; ROSMAP,totalSample,immune-Central_memory; ROSMAP,totalSample,immune-InfiltrationScore
GIMAP5	Homo_723,female,AD.vs.control; Homo_723,male,AD.vs.control; Homo_723,female,AD.vs.MCI; Homo_723,male,AD.vs.MCI; Homo_723,female,MCI.vs.control; Homo_723,control,male.vs.female; Homo_723,MCI,male.vs.female; Homo_714,female,AD.vs.control; Homo_714,male,AD.vs.control; Homo_714,female,AD.vs.MCI; Homo_714,male,AD.vs.MCI; Homo_714,female,MCI.vs.control; Homo_714,control,male.vs.female; Homo_714,MCI,male.vs.female; Homo_633,male,MCI.vs.control; Homo_633,control,male.vs.female; Homo_633,MCI,male.vs.female; ROSMAP,female,AD.vs.control; MCSA,totalSample,age; MCSA,control,age; ROSMAP,AD,last.visit.age; Emory,totalSample,immune-Macrophage; Emory,totalSample,immune-Neutrophil; Emory,totalSample,immune-CD4_T; Emory,totalSample,immune-CD8_T; Emory,totalSample,immune-Tr1; Emory,totalSample,immune-nTreg; Emory,totalSample,immune-iTreg; Emory,totalSample,immune-Tfh; Emory,totalSample,immune-CD8_naive; Emory,totalSample,immune-Exhausted; Emory,totalSample,immune-MAIT; Emory,totalSample,immune-Central_memory; Emory,totalSample,immune-InfiltrationScore; Homo_723,totalSample,immune-Monocyte; Homo_723,totalSample,immune-CD4_naive; Homo_723,totalSample,immune-Central_memory; Homo_723,totalSample,immune-nTreg; Homo_723,totalSample,immune-Tfh; Homo_723,totalSample,immune-Exhausted; Homo_723,totalSample,immune-Neutrophil; Homo_723,totalSample,immune-CD4_T; Homo_723,totalSample,immune-CD8_T; Homo_723,totalSample,immune-iTreg; Homo_723,totalSample,immune-Tr1; Homo_723,totalSample,immune-Th1; Homo_723,totalSample,immune-DC; Homo_723,totalSample,immune-InfiltrationScore; Homo_714,totalSample,immune-CD4_naive; Homo_714,totalSample,immune-Monocyte; Homo_714,totalSample,immune-nTreg; Homo_714,totalSample,immune-Tfh; Homo_714,totalSample,immune-Central_memory; Homo_714,totalSample,immune-Th1; Homo_714,totalSample,immune-Neutrophil; Homo_714,totalSample,immune-CD4_T; Homo_714,totalSample,immune-iTreg; Homo_714,totalSample,immune-Macrophage; Homo_633,totalSample,immune-Monocyte; Homo_633,totalSample,immune-CD4_naive; Homo_633,totalSample,immune-Tfh; Homo_633,totalSample,immune-nTreg; Homo_633,totalSample,immune-iTreg; Homo_633,totalSample,immune-CD4_T; Homo_633,totalSample,immune-Central_memory; Homo_633,totalSample,immune-Th1; Homo_633,totalSample,immune-Tr1; Homo_633,totalSample,immune-InfiltrationScore; Homo_633,totalSample,immune-Effector_memory; Homo_633,totalSample,immune-Neutrophil; Homo_633,totalSample,immune-Macrophage; MCSA,totalSample,immune-Monocyte; MCSA,totalSample,immune-Macrophage; MCSA,totalSample,immune-Neutrophil; MCSA,totalSample,immune-CD4_T; MCSA,totalSample,immune-CD8_T; MCSA,totalSample,immune-NKT; MCSA,totalSample,immune-CD4_naive; MCSA,totalSample,immune-Tr1; MCSA,totalSample,immune-nTreg; MCSA,totalSample,immune-iTreg; MCSA,totalSample,immune-Th2; MCSA,totalSample,immune-Th17; MCSA,totalSample,immune-Tfh; MCSA,totalSample,immune-CD8_naive; MCSA,totalSample,immune-Cytotoxic; MCSA,totalSample,immune-MAIT; MCSA,totalSample,immune-Central_memory; MCSA,totalSample,immune-Effector_memory; MCSA,totalSample,immune-InfiltrationScore; ROSMAP,totalSample,immune-Th17; ROSMAP,totalSample,immune-Effector_memory; [illegible]

TGFA	Homo_723,female,AD.vs.control; Homo_723,male,AD.vs.control; Homo_723,female,AD.vs.MCI; Homo_723,male,AD.vs.MCI; Homo_714,female,AD.vs.control; Homo_714,male,AD.vs.control; Homo_714,female,AD.vs.MCI; Homo_714,male,AD.vs.MCI; Homo_633,female,AD.vs.control; Homo_633,female,AD.vs.MCI; ROSMAP,female,AD.vs.control; ROSMAP,female,AD.vs.MCI; ROSMAP,MCI,last.visit.age; ROSMAP,MCI,sample.age; Emory,totalSample,immune-DC; Emory,totalSample,immune-Monocyte; Emory,totalSample,immune-Neutrophil; Emory,totalSample,immune-CD8_T; Emory,totalSample,immune-Gamma_delta; Emory,totalSample,immune-nTreg; Emory,totalSample,immune-Th1; Emory,totalSample,immune-Tfh; Emory,totalSample,immune-CD8_naive; Emory,totalSample,immune-Cytotoxic; Emory,totalSample,immune-Exhausted; Homo_723,totalSample,immune-Monocyte; Homo_723,totalSample,immune-DC; Homo_723,totalSample,immune-Neutrophil; Homo_723,totalSample,immune-Gamma_delta; Homo_723,totalSample,immune-Th2; Homo_723,totalSample,immune-Exhausted; Homo_723,totalSample,immune-Bcell; Homo_723,totalSample,immune-iTreg; Homo_714,totalSample,immune-Monocyte; Homo_714,totalSample,immune-Th2; Homo_714,totalSample,immune-Neutrophil; Homo_714,totalSample,immune-Gamma_delta; Homo_714,totalSample,immune-DC; Homo_714,totalSample,immune-Tfh; Homo_714,totalSample,immune-Th1; Homo_714,totalSample,immune-Exhausted; Homo_633,totalSample,immune-Monocyte; Homo_633,totalSample,immune-Th2; Homo_633,totalSample,immune-Exhausted; Homo_633,totalSample,immune-Gamma_delta; Homo_633,totalSample,immune-Th17; Homo_633,totalSample,immune-Neutrophil; Homo_633,totalSample,immune-CD8_T; Homo_633,totalSample,immune-DC; MCSA,totalSample,immune-DC; MCSA,totalSample,immune-Monocyte; MCSA,totalSample,immune-Neutrophil; MCSA,totalSample,immune-CD8_T; MCSA,totalSample,immune-Gamma_delta; MCSA,totalSample,immune-iTreg; MCSA,totalSample,immune-Th1; MCSA,totalSample,immune-Th17; MCSA,totalSample,immune-Cytotoxic; MCSA,totalSample,immune-Exhausted; MCSA,totalSample,immune-Effector_memory; MCSA,totalSample,immune-InfiltrationScore; ROSMAP,totalSample,immune-Th17; ROSMAP,totalSample,immune-InfiltrationScore; SRP223445,totalSample,immune-Neutrophil
DOK6	Homo_723,female,AD.vs.control; Homo_723,male,AD.vs.control; Homo_723,female,AD.vs.MCI; Homo_723,male,AD.vs.MCI; Homo_714,female,AD.vs.control; Homo_714,male,AD.vs.control; Homo_714,female,AD.vs.MCI; Homo_714,male,AD.vs.MCI; Homo_633,female,AD.vs.control; Homo_633,female,AD.vs.MCI; ACOM,totalSample,age; MCSA,MCI,age; Emory,totalSample,immune-Tr1; Emory,totalSample,immune-Exhausted; Emory,totalSample,immune-InfiltrationScore; Homo_723,totalSample,immune-CD4_naive; Homo_723,totalSample,immune-CD4_T; Homo_723,totalSample,immune-Th17; Homo_723,totalSample,immune-Central_memory; Homo_723,totalSample,immune-Tfh; Homo_723,totalSample,immune-Th1; Homo_723,totalSample,immune-Tr1; Homo_723,totalSample,immune-InfiltrationScore; Homo_723,totalSample,immune-Neutrophil; Homo_723,totalSample,immune-nTreg; Homo_714,totalSample,immune-CD4_naive; Homo_714,totalSample,immune-InfiltrationScore; Homo_714,totalSample,immune-Tfh; Homo_714,totalSample,immune-CD4_T; Homo_714,totalSample,immune-Th1; Homo_714,totalSample,immune-Central_memory; Homo_714,totalSample,immune-NKT; Homo_714,totalSample,immune-Th17; Homo_633,totalSample,immune-Tfh; Homo_633,totalSample,immune-InfiltrationScore; Homo_633,totalSample,immune-CD4_naive; Homo_633,totalSample,immune-CD4_T; Homo_633,totalSample,immune-Effector_memory; Homo_633,totalSample,immune-Central_memory; Homo_633,totalSample,immune-Tr1; Homo_633,totalSample,immune-NKT; Homo_633,totalSample,immune-iTreg; Homo_633,totalSample,immune-MAIT; Homo_633,totalSample,immune-Bcell; Homo_633,totalSample,immune-Th2; MCSA,totalSample,immune-DC; MCSA,totalSample,immune-Macrophage; MCSA,totalSample,immune-CD4_T; MCSA,totalSample,immune-CD4_naive; MCSA,totalSample,immune-Tr1; MCSA,totalSample,immune-nTreg; MCSA,totalSample,immune-iTreg; MCSA,totalSample,immune-Th17; MCSA,totalSample,immune-Tfh; MCSA,totalSample,immune-CD8_naive; MCSA,totalSample,immune-Exhausted; MCSA,totalSample,immune-
TEX264	Homo_723,female,AD.vs.control; Homo_723,male,AD.vs.control; Homo_723,female,AD.vs.MCI; Homo_723,male,AD.vs.MCI; Homo_714,female,AD.vs.control; Homo_714,male,AD.vs.control; Homo_714,female,AD.vs.MCI; Homo_714,male,AD.vs.MCI; Homo_633,female,AD.vs.control; Homo_633,female,AD.vs.MCI; MCSA,totalSample,age; MCSA,control,age; MCSA,MCI,age; ROSMAP,MCI,last.visit.age; ROSMAP,MCI,sample.age; Emory,totalSample,immune-Macrophage; Emory,totalSample,immune-NK; Emory,totalSample,immune-Neutrophil; Emory,totalSample,immune-CD4_T; Emory,totalSample,immune-CD8_T; Emory,totalSample,immune-Gamma_delta; Emory,totalSample,immune-Tr1; Emory,totalSample,immune-iTreg; Emory,totalSample,immune-Th1; Emory,totalSample,immune-Tfh; Emory,totalSample,immune-Exhausted; Emory,totalSample,immune-MAIT; Emory,totalSample,immune-Effector_memory; Emory,totalSample,immune-InfiltrationScore; Homo_723,totalSample,immune-Effector_memory; Homo_723,totalSample,immune-CD8_T; Homo_723,totalSample,immune-Cytotoxic; Homo_723,totalSample,immune-InfiltrationScore; Homo_723,totalSample,immune-Tfh; Homo_723,totalSample,immune-iTreg; Homo_723,totalSample,immune-Th2; Homo_723,totalSample,immune-Bcell; Homo_723,totalSample,immune-Macrophage; Homo_723,totalSample,immune-nTreg; Homo_723,totalSample,immune-Th1; Homo_723,totalSample,immune-MAIT; Homo_714,totalSample,immune-Effector_memory; Homo_714,totalSample,immune-CD8_T; Homo_714,totalSample,immune-InfiltrationScore; Homo_714,totalSample,immune-Tfh; Homo_714,totalSample,immune-Cytotoxic; Homo_714,totalSample,immune-Th2; Homo_714,totalSample,immune-nTreg; Homo_714,totalSample,immune-Central_memory; Homo_714,totalSample,immune-Th1; Homo_714,totalSample,immune-DC; Homo_714,totalSample,immune-Macrophage; Homo_633,totalSample,immune-InfiltrationScore; Homo_633,totalSample,immune-DC; Homo_633,totalSample,immune-Effector_memory; Homo_633,totalSample,immune-Neutrophil; Homo_633,totalSample,immune-CD8_T; Homo_633,totalSample,immune-nTreg; Homo_633,totalSample,immune-Central_memory; Homo_633,totalSample,immune-Cytotoxic; Homo_633,totalSample,immune-Th1; MCSA,totalSample,immune-DC; MCSA,totalSample,immune-Monocyte; MCSA,totalSample,immune-Neutrophil; MCSA,totalSample,immune-CD8_T; MCSA,totalSample,immune-NKT; MCSA,totalSample,immune-Th2; MCSA,totalSample,immune-Tfh; MCSA,totalSample,immune-Cytotoxic; MCSA,totalSample,immune-Exhausted; MCSA,totalSample,immune-Central_memory; MCSA,totalSample,immune-Effector_memory; MCSA,totalSample,immune-InfiltrationScore; ROSMAP,totalSample,immune-Effector_memory; SRP223445,totalSample,immune- [illegible]
ZMYND12	Homo_723,female,AD.vs.control; Homo_723,male,AD.vs.control; Homo_723,female,AD.vs.MCI; Homo_723,male,AD.vs.MCI; Homo_723,female,MCI.vs.control; Homo_723,male,MCI.vs.control; Homo_714,female,AD.vs.control; Homo_714,male,AD.vs.control; Homo_714,female,AD.vs.MCI; Homo_714,male,AD.vs.MCI; Homo_714,female,MCI.vs.control; Homo_714,male,MCI.vs.control; Homo_633,female,AD.vs.control; Homo_633,female,AD.vs.MCI; ROSMAP,female,AD.vs.control; ROSMAP,male,AD.vs.control; ROSMAP,female,MCI.vs.control; SRP223445,female,AD.vs.control; Homo_723,totalSample,immune-Th17; Homo_723,totalSample,immune-Exhausted; Homo_723,totalSample,immune-Cytotoxic; Homo_723,totalSample,immune-CD8_T; Homo_714,totalSample,immune-Exhausted; Homo_714,totalSample,immune-CD8_T; Homo_714,totalSample,immune-Th17; Homo_633,totalSample,immune-Gamma_delta; ROSMAP,totalSample,immune-Th17; ROSMAP,totalSample,immune-InfiltrationScore; SRP223445,totalSample,immune-CD4_naive
PDGFC	Homo_723,female,AD.vs.control; Homo_723,male,AD.vs.control; Homo_723,female,AD.vs.MCI; Homo_723,male,AD.vs.MCI; Homo_723,female,MCI.vs.control; Homo_723,male,MCI.vs.control; Homo_714,female,AD.vs.control; Homo_714,male,AD.vs.control; Homo_714,female,AD.vs.MCI; Homo_714,male,AD.vs.MCI; Homo_714,female,MCI.vs.control; Homo_714,male,MCI.vs.control; Homo_633,female,AD.vs.control; Homo_633,female,AD.vs.MCI; MCSA,MCI,age; ROSMAP,MCI,last.visit.age; ROSMAP,MCI,sample.age; Emory,totalSample,immune-Monocyte; Emory,totalSample,immune-Gamma_delta; Emory,totalSample,immune-Th1; Homo_723,totalSample,immune-Exhausted; Homo_723,totalSample,immune-Th17; Homo_723,totalSample,immune-CD8_T; Homo_723,totalSample,immune-Cytotoxic; Homo_723,totalSample,immune-Monocyte; Homo_723,totalSample,immune-CD4_naive; Homo_723,totalSample,immune-Gamma_delta; Homo_714,totalSample,immune-Monocyte; Homo_714,totalSample,immune-Exhausted; Homo_714,totalSample,immune-Th17; Homo_714,totalSample,immune-CD8_T; Homo_714,totalSample,immune-Gamma_delta; Homo_714,totalSample,immune-Cytotoxic; Homo_633,totalSample,immune-Monocyte; Homo_633,totalSample,immune-Tfh; Homo_633,totalSample,immune-Exhausted; Homo_633,totalSample,immune-Gamma_delta; Homo_633,totalSample,immune-Th2; Homo_633,totalSample,immune-NKT; Homo_633,totalSample,immune-Th17; Homo_633,totalSample,immune-CD4_T; Homo_633,totalSample,immune-Effector_memory; Homo_633,totalSample,immune-CD4_naive; MCSA,totalSample,immune-DC; MCSA,totalSample,immune-Monocyte; MCSA,totalSample,immune-CD4_T; MCSA,totalSample,immune-CD8_T; MCSA,totalSample,immune-CD4_naive; MCSA,totalSample,immune-Tr1; MCSA,totalSample,immune-nTreg; MCSA,totalSample,immune-iTreg; MCSA,totalSample,immune-CD8_naive; MCSA,totalSample,immune-Cytotoxic; MCSA,totalSample,immune-Exhausted; SRP223445,totalSample,immune-CD4_naive; SRP223445,totalSample,immune-Exhausted
EIF4A1	Homo_723,female,AD.vs.control; Homo_723,male,AD.vs.control; Homo_723,female,AD.vs.MCI; Homo_723,male,AD.vs.MCI; Homo_723,female,MCI.vs.control; Homo_723,male,MCI.vs.control; Homo_714,female,AD.vs.control; Homo_714,male,AD.vs.control; Homo_714,female,AD.vs.MCI; Homo_714,male,AD.vs.MCI; Homo_714,female,MCI.vs.control; Homo_714,male,MCI.vs.control; Homo_633,female,AD.vs.control; Homo_633,female,AD.vs.MCI; Homo_633,female,MCI.vs.control; Homo_633,male,MCI.vs.control; ROSMAP,female,AD.vs.control; Emory,MCI,age; ROSMAP,MCI,last.visit.age; ROSMAP,MCI,sample.age; Emory,totalSample,immune-Neutrophil; Emory,totalSample,immune-Tr1; Emory,totalSample,immune-iTreg; Emory,totalSample,immune-Effector_memory; Emory,totalSample,immune-InfiltrationScore; Homo_723,totalSample,immune-Th2; Homo_723,totalSample,immune-Monocyte; Homo_723,totalSample,immune-Tfh; Homo_723,totalSample,immune-Effector_memory; Homo_723,totalSample,immune-iTreg; Homo_723,totalSample,immune-InfiltrationScore; Homo_723,totalSample,immune-Bcell; Homo_714,totalSample,immune-Th2; Homo_714,totalSample,immune-Tfh; Homo_714,totalSample,immune-Effector_memory; Homo_714,totalSample,immune-Monocyte; Homo_633,totalSample,immune-Monocyte; Homo_633,totalSample,immune-Th2; Homo_633,totalSample,immune-Effector_memory; Homo_633,totalSample,immune-Tfh; Homo_633,totalSample,immune-InfiltrationScore; Homo_633,totalSample,immune-Exhausted; Homo_633,totalSample,immune-Bcell; Homo_633,totalSample,immune-Th17; MCSA,totalSample,immune-DC; MCSA,totalSample,immune-NK; MCSA,totalSample,immune-Neutrophil; MCSA,totalSample,immune-Th17; MCSA,totalSample,immune-InfiltrationScore; SRP223445,totalSample,immune-CD4_naive; SRP223445,totalSample,immune-Th17; SRP223445,totalSample,immune-Exhausted; SRP223445,totalSample,immune-InfiltrationScore
SLIT1	Homo_723,female,AD.vs.control; Homo_723,female,AD.vs.MCI; Homo_723,female,MCI.vs.control; Homo_633,female,AD.vs.control; Homo_633,female,AD.vs.MCI; ROSMAP,female,AD.vs.control; ROSMAP,male,AD.vs.control; ROSMAP,female,MCI.vs.control; Emory,totalSample,immune-CD8_T; Emory,totalSample,immune-Gamma_delta; Emory,totalSample,immune-Tfh; Emory,totalSample,immune-CD8_naive; Homo_723,totalSample,immune-DC; Homo_714,totalSample,immune-DC; Homo_633,totalSample,immune-DC; Homo_633,totalSample,immune-InfiltrationScore; MCSA,totalSample,immune-DC; MCSA,totalSample,immune-Monocyte; MCSA,totalSample,immune-Macrophage; MCSA,totalSample,immune-NKT;
CHKB	Homo_723,female,AD.vs.control; Homo_723,male,AD.vs.control; Homo_723,female,AD.vs.MCI; Homo_723,male,AD.vs.MCI; Homo_714,female,AD.vs.control; Homo_714,male,AD.vs.control; Homo_714,female,AD.vs.MCI; Homo_714,male,AD.vs.MCI; Homo_633,female,AD.vs.control; Homo_633,female,AD.vs.MCI; ROSMAP,male,AD.vs.control; ROSMAP,female,MCI.vs.control; ROSMAP,male,MCI.vs.control; MCSA,totalSample,age; MCSA,control,age; ROSMAP,MCI,sample.age; Emory,totalSample,immune-Macrophage; Emory,totalSample,immune-CD4_T; Emory,totalSample,immune-Tr1; Emory,totalSample,immune-iTreg; Emory,totalSample,immune-Effector_memory; Emory,totalSample,immune-InfiltrationScore; Homo_723,totalSample,immune-Th2; Homo_723,totalSample,immune-Tfh; Homo_723,totalSample,immune-iTreg; Homo_723,totalSample,immune-CD8_T; Homo_723,totalSample,immune-InfiltrationScore; Homo_723,totalSample,immune-Effector_memory; Homo_723,totalSample,immune-CD4_T; Homo_723,totalSample,immune-Macrophage; Homo_723,totalSample,immune-nTreg; Homo_723,totalSample,immune-CD4_naive; Homo_723,totalSample,immune-Tr1; Homo_723,totalSample,immune-Cytotoxic; Homo_723,totalSample,immune-Bcell; Homo_714,totalSample,immune-Th2; Homo_714,totalSample,immune-Tfh; Homo_714,totalSample,immune-Effector_memory; Homo_714,totalSample,immune-CD4_T; Homo_714,totalSample,immune-nTreg; Homo_714,totalSample,immune-Macrophage; Homo_714,totalSample,immune-Tr1; Homo_714,totalSample,immune-InfiltrationScore; Homo_714,totalSample,immune-iTreg; Homo_714,totalSample,immune-Central_memory; Homo_714,totalSample,immune-CD8_T; Homo_633,totalSample,immune-InfiltrationScore; Homo_633,totalSample,immune-nTreg; Homo_633,totalSample,immune-Tfh; Homo_633,totalSample,immune-Effector_memory; Homo_633,totalSample,immune-CD4_T; Homo_633,totalSample,immune-Th2; Homo_633,totalSample,immune-CD4_naive; Homo_633,totalSample,immune-Tr1; Homo_633,totalSample,immune-Central_memory; MCSA,totalSample,immune-DC; MCSA,totalSample,immune-Monocyte; MCSA,totalSample,immune-CD4_T; MCSA,totalSample,immune-CD4_naive; MCSA,totalSample,immune-Tr1; MCSA,totalSample,immune-nTreg; MCSA,totalSample,immune-Th17; MCSA,totalSample,immune-Tfh; MCSA,totalSample,immune-Effector_memory; MCSA,totalSample,immune-InfiltrationScore; [illegible]
DHRS7	Homo_723,female,AD.vs.control; Homo_723,male,AD.vs.control; Homo_723,female,AD.vs.MCI; Homo_723,male,AD.vs.MCI; Homo_714,female,AD.vs.control; Homo_714,male,AD.vs.control; Homo_714,female,AD.vs.MCI; Homo_714,male,AD.vs.MCI; Homo_633,female,AD.vs.control; Homo_633,female,AD.vs.MCI; ROSMAP,female,AD.vs.control; ROSMAP,female,MCI.vs.control; Emory,MCI,age; MCSA,totalSample,age; MCSA,MCI,age; Emory,totalSample,immune-DC; Emory,totalSample,immune-Monocyte; Emory,totalSample,immune-Neutrophil; Emory,totalSample,immune-CD8_T; Emory,totalSample,immune-Gamma_delta; Emory,totalSample,immune-nTreg; Emory,totalSample,immune-Tfh; Emory,totalSample,immune-CD8_naive; Emory,totalSample,immune-Exhausted; Homo_723,totalSample,immune-Monocyte; Homo_723,totalSample,immune-DC; Homo_723,totalSample,immune-Neutrophil; Homo_723,totalSample,immune-Th2; Homo_723,totalSample,immune-Bcell; Homo_723,totalSample,immune-Gamma_delta; Homo_723,totalSample,immune-InfiltrationScore; Homo_714,totalSample,immune-Monocyte; Homo_714,totalSample,immune-Th2; Homo_714,totalSample,immune-DC; Homo_714,totalSample,immune-Neutrophil; Homo_714,totalSample,immune-Tfh; Homo_714,totalSample,immune-Gamma_delta; Homo_714,totalSample,immune-Th1; Homo_633,totalSample,immune-Monocyte; Homo_633,totalSample,immune-Th2; Homo_633,totalSample,immune-Exhausted; Homo_633,totalSample,immune-Th17; Homo_633,totalSample,immune-DC; Homo_633,totalSample,immune-Neutrophil; Homo_633,totalSample,immune-Gamma_delta; Homo_633,totalSample,immune-InfiltrationScore; MCSA,totalSample,immune-DC; MCSA,totalSample,immune-Monocyte; MCSA,totalSample,immune-Neutrophil; MCSA,totalSample,immune-CD8_T; MCSA,totalSample,immune-NKT; MCSA,totalSample,immune-Th2; MCSA,totalSample,immune-Th17; MCSA,totalSample,immune-Tfh; MCSA,totalSample,immune-Cytotoxic; MCSA,totalSample,immune-Exhausted; MCSA,totalSample,immune-Central_memory; MCSA,totalSample,immune-
DUSP4	Homo_723,female,AD.vs.control; Homo_723,male,AD.vs.control; Homo_723,female,AD.vs.MCI; Homo_723,male,AD.vs.MCI; Homo_723,female,MCI.vs.control; Homo_723,male,MCI.vs.control; Homo_714,female,AD.vs.control; Homo_714,male,AD.vs.control; Homo_714,female,AD.vs.MCI; Homo_714,male,AD.vs.MCI; Homo_633,female,AD.vs.control; Homo_633,female,AD.vs.MCI; ROSMAP,MCI,male.vs.female; Emory,totalSample,immune-iTreg; Homo_723,totalSample,immune-Th17; Homo_723,totalSample,immune-nTreg; Homo_723,totalSample,immune-CD4_T; Homo_723,totalSample,immune-CD4_naive; Homo_723,totalSample,immune-Tr1; Homo_723,totalSample,immune-InfiltrationScore; Homo_723,totalSample,immune-Exhausted; Homo_723,totalSample,immune-Th1; Homo_723,totalSample,immune-Tfh; Homo_723,totalSample,immune-Cytotoxic; Homo_714,totalSample,immune-InfiltrationScore; Homo_714,totalSample,immune-Th17; Homo_714,totalSample,immune-nTreg; Homo_714,totalSample,immune-CD4_naive; Homo_714,totalSample,immune-CD4_T; Homo_633,totalSample,immune-Tfh; Homo_633,totalSample,immune-Tr1; Homo_633,totalSample,immune-Th2; Homo_633,totalSample,immune-InfiltrationScore; Homo_633,totalSample,immune-CD4_T; Homo_633,totalSample,immune-nTreg; Homo_633,totalSample,immune-iTreg; MCSA,totalSample,immune-Macrophage; MCSA,totalSample,immune-CD4_T; MCSA,totalSample,immune-CD4_naive; MCSA,totalSample,immune-Tr1; MCSA,totalSample,immune-nTreg; MCSA,totalSample,immune-iTreg; MCSA,totalSample,immune-Th17; MCSA,totalSample,immune-Tfh; MCSA,totalSample,immune-Central_memory; ROSMAP,totalSample,immune-Central_memory; ROSMAP,totalSample,immune-Effector_memory;
TRIM38	Homo_723,female,AD.vs.control; Homo_723,male,AD.vs.control; Homo_723,female,AD.vs.MCI; Homo_723,male,AD.vs.MCI; Homo_714,female,AD.vs.control; Homo_714,male,AD.vs.control; Homo_714,female,AD.vs.MCI; Homo_714,male,AD.vs.MCI; Homo_633,female,AD.vs.control; Homo_633,female,AD.vs.MCI; ROSMAP,female,AD.vs.control; ROSMAP,female,MCI.vs.control; Emory,MCI,age; MCSA,MCI,age; ROSMAP,AD,last.visit.age; Emory,totalSample,immune-Gamma_delta; Emory,totalSample,immune-Tr1; Emory,totalSample,immune-iTreg; Emory,totalSample,immune-CD8_naive; Emory,totalSample,immune-Cytotoxic; Emory,totalSample,immune-InfiltrationScore; Homo_723,totalSample,immune-Th2; Homo_723,totalSample,immune-Monocyte; Homo_723,totalSample,immune-Tfh; Homo_723,totalSample,immune-InfiltrationScore; Homo_723,totalSample,immune-DC; Homo_723,totalSample,immune-iTreg; Homo_723,totalSample,immune-Gamma_delta; Homo_723,totalSample,immune-Effector_memory; Homo_723,totalSample,immune-Bcell; Homo_723,totalSample,immune-CD4_T; Homo_723,totalSample,immune-Tr1; Homo_714,totalSample,immune-Th2; Homo_714,totalSample,immune-Tfh; Homo_714,totalSample,immune-Monocyte; Homo_714,totalSample,immune-Gamma_delta; Homo_714,totalSample,immune-CD4_T; Homo_714,totalSample,immune-Effector_memory; Homo_714,totalSample,immune-Tr1; Homo_633,totalSample,immune-Th2; Homo_633,totalSample,immune-Monocyte; Homo_633,totalSample,immune-Tfh; Homo_633,totalSample,immune-Exhausted; Homo_633,totalSample,immune-Gamma_delta; Homo_633,totalSample,immune-InfiltrationScore; Homo_633,totalSample,immune-Th17; Homo_633,totalSample,immune-CD4_T; MCSA,totalSample,immune-Neutrophil; MCSA,totalSample,immune-CD4_T; MCSA,totalSample,immune-CD8_T; MCSA,totalSample,immune-Gamma_delta; MCSA,totalSample,immune-Tr1; MCSA,totalSample,immune-nTreg; MCSA,totalSample,immune-iTreg; MCSA,totalSample,immune-Th17; MCSA,totalSample,immune-Cytotoxic; MCSA,totalSample,immune-Exhausted;
LYPD1	Homo_723,female,AD.vs.control; Homo_723,male,AD.vs.control; Homo_723,female,AD.vs.MCI; Homo_723,male,AD.vs.MCI; MCSA,MCI,age; Emory,totalSample,immune-NK; Emory,totalSample,immune-Neutrophil; Emory,totalSample,immune-Gamma_delta; Emory,totalSample,immune-Exhausted; Emory,totalSample,immune-MAIT; ROSMAP,totalSample,immune-
DTNA	Homo_723,female,AD.vs.control; Homo_723,male,AD.vs.control; Homo_723,female,AD.vs.MCI; Homo_723,male,AD.vs.MCI; Homo_723,female,MCI.vs.control; Homo_723,male,MCI.vs.control; Homo_714,female,AD.vs.control; Homo_714,male,AD.vs.control; Homo_714,female,AD.vs.MCI; Homo_714,male,AD.vs.MCI; Homo_633,female,AD.vs.control; Homo_633,female,AD.vs.MCI; ACOM,AD,age; MCSA,MCI,age; Emory,totalSample,immune-CD8_T; Emory,totalSample,immune-Gamma_delta; Emory,totalSample,immune-Tfh; Homo_723,totalSample,immune-Exhausted; Homo_723,totalSample,immune-CD8_T; Homo_723,totalSample,immune-Cytotoxic; Homo_723,totalSample,immune-Th17; Homo_714,totalSample,immune-Exhausted; Homo_714,totalSample,immune-CD8_T; Homo_633,totalSample,immune-InfiltrationScore; Homo_633,totalSample,immune-Exhausted; Homo_633,totalSample,immune-Gamma_delta; MCSA,totalSample,immune-Monocyte; MCSA,totalSample,immune-CD4_T; MCSA,totalSample,immune-CD8_T; MCSA,totalSample,immune-CD4_naive; MCSA,totalSample,immune-Tr1; MCSA,totalSample,immune-nTreg; MCSA,totalSample,immune-iTreg; MCSA,totalSample,immune-Cytotoxic; MCSA,totalSample,immune-Exhausted; ROSMAP,totalSample,immune-InfiltrationScore; SRP223445,totalSample,immune-InfiltrationScore; ROSMAP,female-includeAge90+,survival
MAL	Homo_723,female,AD.vs.control; Homo_723,male,AD.vs.control; Homo_723,female,AD.vs.MCI; Homo_723,male,AD.vs.MCI; Homo_714,female,AD.vs.control; Homo_714,male,AD.vs.control; Homo_714,female,AD.vs.MCI; Homo_714,male,AD.vs.MCI; Homo_633,female,AD.vs.control; Homo_633,female,AD.vs.MCI; SRP223445,male,AD.vs.control; Emory,MCI,age; MCSA,totalSample,age; MCSA,control,age; Emory,totalSample,immune-Monocyte; Emory,totalSample,immune-Macrophage; Emory,totalSample,immune-Neutrophil; Emory,totalSample,immune-CD4_T; Emory,totalSample,immune-CD8_T; Emory,totalSample,immune-CD4_naive; Emory,totalSample,immune-Tr1; Emory,totalSample,immune-nTreg; Emory,totalSample,immune-iTreg; Emory,totalSample,immune-Th1; Emory,totalSample,immune-Tfh; Emory,totalSample,immune-CD8_naive; Emory,totalSample,immune-MAIT; Emory,totalSample,immune-Central_memory; Homo_723,totalSample,immune-Tfh; Homo_723,totalSample,immune-iTreg; Homo_723,totalSample,immune-Th2; Homo_723,totalSample,immune-Central_memory; Homo_723,totalSample,immune-nTreg; Homo_723,totalSample,immune-CD4_T; Homo_723,totalSample,immune-Tr1; Homo_723,totalSample,immune-CD8_T; Homo_723,totalSample,immune-CD4_naive; Homo_723,totalSample,immune-Macrophage; Homo_723,totalSample,immune-Gamma_delta; Homo_723,totalSample,immune-Effector_memory; Homo_714,totalSample,immune-Tfh; Homo_714,totalSample,immune-Th2; Homo_714,totalSample,immune-nTreg; Homo_714,totalSample,immune-Central_memory; Homo_714,totalSample,immune-CD4_T; Homo_714,totalSample,immune-Tr1; Homo_714,totalSample,immune-iTreg; Homo_714,totalSample,immune-CD4_naive; Homo_714,totalSample,immune-Effector_memory; Homo_714,totalSample,immune-Macrophage; Homo_714,totalSample,immune-Gamma_delta; Homo_714,totalSample,immune-CD8_T; Homo_633,totalSample,immune-nTreg; Homo_633,totalSample,immune-Tfh; Homo_633,totalSample,immune-Central_memory; Homo_633,totalSample,immune-CD4_T; Homo_633,totalSample,immune-Tr1; Homo_633,totalSample,immune-iTreg; Homo_633,totalSample,immune-CD4_naive; Homo_633,totalSample,immune-Th2; Homo_633,totalSample,immune-Gamma_delta; Homo_633,totalSample,immune-Th17; Homo_633,totalSample,immune-Effector_memory; Homo_633,totalSample,immune-Neutrophil; Homo_633,totalSample,immune-Macrophage; Homo_633,totalSample,immune-Th1; MCSA,totalSample,immune-Monocyte; MCSA,totalSample,immune-Neutrophil; MCSA,totalSample,immune-CD4_T; MCSA,totalSample,immune-CD8_T; MCSA,totalSample,immune-Gamma_delta; MCSA,totalSample,immune-CD4_naive; MCSA,totalSample,immune-Tr1; MCSA,totalSample,immune-nTreg; MCSA,totalSample,immune-iTreg; MCSA,totalSample,immune-Th17; MCSA,totalSample,immune-Tfh; MCSA,totalSample,immune-CD8_naive; MCSA,totalSample,immune-Central_memory; MCSA,totalSample,immune-Effector_memory; MCSA,totalSample,immune-InfiltrationScore; ROSMAP,totalSample,immune-InfiltrationScore
KRT1	Homo_723,female,AD.vs.control; Homo_723,male,AD.vs.control; Homo_723,female,AD.vs.MCI; Homo_723,male,AD.vs.MCI; Homo_723,female,MCI.vs.control; Homo_633,female,AD.vs.control; Homo_633,female,AD.vs.MCI; ROSMAP,female,AD.vs.control; ROSMAP,female,MCI.vs.control; Emory,MCI,age; ROSMAP,MCI,sample.age; Emory,totalSample,immune-Gamma_delta; Emory,totalSample,immune-CD8_naive; Homo_723,totalSample,immune-Exhausted; Homo_723,totalSample,immune-CD8_T; Homo_723,totalSample,immune-Cytotoxic; Homo_723,totalSample,immune-Bcell; Homo_723,totalSample,immune-CD8_naive; Homo_714,totalSample,immune-Bcell; Homo_633,totalSample,immune-Bcell; Homo_633,totalSample,immune-Monocyte; Homo_633,totalSample,immune-CD8_naive; MCSA,totalSample,immune-CD8_naive; SRP223445,totalSample,immune-InfiltrationScore
LOC400499	Homo_723,female,AD.vs.control; Homo_723,male,AD.vs.control; Homo_723,female,AD.vs.MCI; Homo_723,male,AD.vs.MCI; Homo_714,female,AD.vs.control; Homo_714,male,AD.vs.control; Homo_714,female,AD.vs.MCI; Homo_714,male,AD.vs.MCI; Homo_633,female,AD.vs.control; Homo_633,female,AD.vs.MCI; MCSA,totalSample,age; MCSA,control,age; MCSA,MCI,age; Emory,totalSample,immune-Neutrophil; Emory,totalSample,immune-CD8_T; Emory,totalSample,immune-Gamma_delta; Emory,totalSample,immune-Th1; Emory,totalSample,immune-Th17; Emory,totalSample,immune-Tfh; Emory,totalSample,immune-Cytotoxic; Emory,totalSample,immune-Exhausted; Emory,totalSample,immune-MAIT; Emory,totalSample,immune-Effector_memory; Homo_723,totalSample,immune-Neutrophil; Homo_723,totalSample,immune-Th1; Homo_723,totalSample,immune-Cytotoxic; Homo_723,totalSample,immune-Gamma_delta; Homo_714,totalSample,immune-Neutrophil; Homo_714,totalSample,immune-Th1; Homo_633,totalSample,immune-Neutrophil; Homo_633,totalSample,immune-Th1; Homo_633,totalSample,immune-MAIT; Homo_633,totalSample,immune-Exhausted; MCSA,totalSample,immune-DC; MCSA,totalSample,immune-Bcell; MCSA,totalSample,immune-Monocyte; MCSA,totalSample,immune-Macrophage; MCSA,totalSample,immune-Neutrophil; MCSA,totalSample,immune-Gamma_delta; MCSA,totalSample,immune-Tr1; MCSA,totalSample,immune-iTreg; MCSA,totalSample,immune-Th1; MCSA,totalSample,immune-Th2; MCSA,totalSample,immune-Th17; MCSA,totalSample,immune-CD8_naive; MCSA,totalSample,immune-MAIT

STRIP2	Homo_723,female,AD.vs.control; Homo_723,male,AD.vs.control; Homo_723,female,AD.vs.MCI; Homo_723,male,AD.vs.MCI; Homo_723,male,MCI.vs.control; Homo_714,female,AD.vs.control; Homo_714,male,AD.vs.control; Homo_714,female,AD.vs.MCI; Homo_714,male,AD.vs.MCI; Homo_633,female,AD.vs.control; Homo_633,female,AD.vs.MCI; ROSMAP,MCI,last.visit.age; Emory,totalSample,immune-Neutrophil; Emory,totalSample,immune-CD8_T; Emory,totalSample,immune-Gamma_delta; Emory,totalSample,immune-Th1; Homo_723,totalSample,immune-Th17; Homo_723,totalSample,immune-CD8_T; Homo_723,totalSample,immune-Exhausted; Homo_723,totalSample,immune-Cytotoxic; Homo_714,totalSample,immune-Th17; Homo_714,totalSample,immune-Exhausted; Homo_714,totalSample,immune-CD8_T; Homo_633,totalSample,immune-InfiltrationScore; MCSA,totalSample,immune-Tr1; MCSA,totalSample,immune-iTreg; MCSA,totalSample,immune-Th17; SRP223445,totalSample,immune-CD4_naive; SRP223445,totalSample,immune-Exhausted
METTL23	Homo_723,female,AD.vs.control; Homo_723,male,AD.vs.control; Homo_723,female,AD.vs.MCI; Homo_723,male,AD.vs.MCI; Homo_714,female,AD.vs.control; Homo_714,male,AD.vs.control; Homo_714,female,AD.vs.MCI; Homo_714,male,AD.vs.MCI; Homo_633,female,AD.vs.control; Homo_633,female,AD.vs.MCI; ROSMAP,female,AD.vs.control; Emory,totalSample,immune-Macrophage; Emory,totalSample,immune-NK; Emory,totalSample,immune-Neutrophil; Emory,totalSample,immune-CD4_T; Emory,totalSample,immune-CD8_T; Emory,totalSample,immune-Tr1; Emory,totalSample,immune-iTreg; Emory,totalSample,immune-Tfh; Emory,totalSample,immune-Exhausted; Emory,totalSample,immune-MAIT; Emory,totalSample,immune-Central_memory; Emory,totalSample,immune-Effector_memory; Emory,totalSample,immune-InfiltrationScore; Homo_723,totalSample,immune-Tfh; Homo_723,totalSample,immune-iTreg; Homo_723,totalSample,immune-CD4_T; Homo_723,totalSample,immune-Tr1; Homo_723,totalSample,immune-Th2; Homo_723,totalSample,immune-CD4_naive; Homo_723,totalSample,immune-InfiltrationScore; Homo_723,totalSample,immune-MAIT; Homo_723,totalSample,immune-Macrophage; Homo_723,totalSample,immune-Central_memory; Homo_723,totalSample,immune-CD8_T; Homo_723,totalSample,immune-Effector_memory; Homo_723,totalSample,immune-nTreg; Homo_723,totalSample,immune-Exhausted; Homo_723,totalSample,immune-DC; Homo_714,totalSample,immune-Tfh; Homo_714,totalSample,immune-CD4_T; Homo_714,totalSample,immune-Tr1; Homo_714,totalSample,immune-Th2; Homo_714,totalSample,immune-Effector_memory; Homo_714,totalSample,immune-iTreg; Homo_714,totalSample,immune-CD4_naive; Homo_714,totalSample,immune-InfiltrationScore; Homo_714,totalSample,immune-Central_memory; Homo_714,totalSample,immune-Macrophage; Homo_714,totalSample,immune-MAIT; Homo_714,totalSample,immune-nTreg; Homo_714,totalSample,immune-CD8_T; Homo_714,totalSample,immune-Neutrophil; Homo_633,totalSample,immune-Tfh; Homo_633,totalSample,immune-CD4_T; Homo_633,totalSample,immune-CD4_naive; Homo_633,totalSample,immune-InfiltrationScore; Homo_633,totalSample,immune-Effector_memory; Homo_633,totalSample,immune-Tr1; Homo_633,totalSample,immune-Central_memory; Homo_633,totalSample,immune-Neutrophil; Homo_633,totalSample,immune-iTreg; Homo_633,totalSample,immune-MAIT; Homo_633,totalSample,immune-NKT; Homo_633,totalSample,immune-nTreg; Homo_633,totalSample,immune-Th1; Homo_633,totalSample,immune-Macrophage; Homo_633,totalSample,immune-Bcell; Homo_633,totalSample,immune-Th2; MCSA,totalSample,immune-DC; MCSA,totalSample,immune-Macrophage; MCSA,totalSample,immune-Neutrophil; MCSA,totalSample,immune-CD4_T; MCSA,totalSample,immune-CD4_naive; MCSA,totalSample,immune-Tr1; MCSA,totalSample,immune-nTreg; MCSA,totalSample,immune-iTreg; MCSA,totalSample,immune-Th2; MCSA,totalSample,immune-Th17; MCSA,totalSample,immune-Tfh; MCSA,totalSample,immune-CD8_naive; MCSA,totalSample,immune-MAIT; [illegible]
ZSCAN16	Homo_723,female,AD.vs.control; Homo_723,male,AD.vs.control; Homo_723,female,AD.vs.MCI; Homo_723,male,AD.vs.MCI; Homo_714,female,AD.vs.control; Homo_714,male,AD.vs.control; Homo_714,female,AD.vs.MCI; Homo_714,male,AD.vs.MCI; Homo_633,female,AD.vs.control; Homo_633,female,AD.vs.MCI; MCSA,totalSample,age; MCSA,control,age; MCSA,MCI,age; ROSMAP,MCI,last.visit.age; Emory,totalSample,immune-Monocyte; Emory,totalSample,immune-Macrophage; Emory,totalSample,immune-CD4_T; Emory,totalSample,immune-Gamma_delta; Emory,totalSample,immune-Tr1; Emory,totalSample,immune-iTreg; Emory,totalSample,immune-InfiltrationScore; Homo_723,totalSample,immune-Monocyte; Homo_723,totalSample,immune-Th2; Homo_723,totalSample,immune-DC; Homo_723,totalSample,immune-Tfh; Homo_723,totalSample,immune-iTreg; Homo_723,totalSample,immune-InfiltrationScore; Homo_723,totalSample,immune-Effector_memory; Homo_723,totalSample,immune-Tr1; Homo_723,totalSample,immune-Bcell; Homo_723,totalSample,immune-CD4_T; Homo_714,totalSample,immune-Th2; Homo_714,totalSample,immune-Monocyte; Homo_714,totalSample,immune-Tfh; Homo_714,totalSample,immune-Tr1; Homo_714,totalSample,immune-Effector_memory; Homo_714,totalSample,immune-DC; Homo_633,totalSample,immune-Monocyte; Homo_633,totalSample,immune-Th2; Homo_633,totalSample,immune-Tfh; Homo_633,totalSample,immune-InfiltrationScore; Homo_633,totalSample,immune-Exhausted; Homo_633,totalSample,immune-Bcell; MCSA,totalSample,immune-DC; MCSA,totalSample,immune-Monocyte; MCSA,totalSample,immune-Macrophage; MCSA,totalSample,immune-CD8_T; MCSA,totalSample,immune-iTreg; MCSA,totalSample,immune-Th2; MCSA,totalSample,immune-Cytotoxic; MCSA,totalSample,immune-Exhausted; MCSA,totalSample,immune-MAIT; MCSA,totalSample,immune-Effector_memory; ROSMAP,totalSample,immune-CD4_T; ROSMAP,totalSample,immune-Th17; ROSMAP,totalSample,immune-InfiltrationScore; SRP223445,totalSample,immune-NK; SRP223445,totalSample,immune-CD4_naive;
UPP1	Homo_723,female,AD.vs.control; Homo_723,male,AD.vs.control; Homo_723,female,AD.vs.MCI; Homo_723,male,AD.vs.MCI; Homo_714,female,AD.vs.control; Homo_714,male,AD.vs.control; Homo_714,female,AD.vs.MCI; Homo_714,male,AD.vs.MCI; Homo_633,female,AD.vs.control; Homo_633,female,AD.vs.MCI; MCSA,MCI,age; Homo_723,totalSample,immune-Exhausted; Homo_723,totalSample,immune-CD8_T; Homo_723,totalSample,immune-Cytotoxic; Homo_723,totalSample,immune-Th17; Homo_723,totalSample,immune-InfiltrationScore; Homo_723,totalSample,immune-Monocyte; Homo_714,totalSample,immune-DC; Homo_714,totalSample,immune-Monocyte; Homo_633,totalSample,immune-DC; Homo_633,totalSample,immune-Th2; Homo_633,totalSample,immune-Monocyte; Homo_633,totalSample,immune-InfiltrationScore; MCSA,totalSample,immune-DC; MCSA,totalSample,immune-Monocyte; MCSA,totalSample,immune-Macrophage; MCSA,totalSample,immune-CD4_T; MCSA,totalSample,immune-CD8_T; MCSA,totalSample,immune-Tr1; MCSA,totalSample,immune-iTreg; MCSA,totalSample,immune-Th2; MCSA,totalSample,immune-CD8_naive; MCSA,totalSample,immune-Cytotoxic; MCSA,totalSample,immune-Exhausted; MCSA,totalSample,immune-MAIT; MCSA,totalSample,immune-Effector_memory; MCSA,totalSample,immune-InfiltrationScore; ROSMAP,totalSample,immune-Macrophage; ROSMAP,totalSample,immune-MAIT; ROSMAP,totalSample,immune-Effector_memory; SRP223445,totalSample,immune-CD4_naive; SRP223445,totalSample,immune-InfiltrationScore
SCAMP5	Homo_723,female,AD.vs.control; Homo_723,male,AD.vs.control; Homo_723,female,AD.vs.MCI; Homo_723,male,AD.vs.MCI; Homo_714,female,AD.vs.control; Homo_714,male,AD.vs.control; Homo_714,female,AD.vs.MCI; Homo_714,male,AD.vs.MCI; Homo_633,female,AD.vs.control; Homo_633,female,AD.vs.MCI; Homo_633,female,MCI.vs.control; ROSMAP,female,AD.vs.control; ROSMAP,male,AD.vs.control; ROSMAP,female,MCI.vs.control; ACOM,AD,age; MCSA,totalSample,age; MCSA,control,age; Homo_723,totalSample,immune-DC; Homo_723,totalSample,immune-Monocyte; Homo_723,totalSample,immune-InfiltrationScore; Homo_723,totalSample,immune-Neutrophil; Homo_723,totalSample,immune-NK; Homo_714,totalSample,immune-DC; Homo_714,totalSample,immune-InfiltrationScore; Homo_714,totalSample,immune-NK; Homo_633,totalSample,immune-DC; Homo_633,totalSample,immune-InfiltrationScore; Homo_633,totalSample,immune-NK; MCSA,totalSample,immune-DC; MCSA,totalSample,immune-Monocyte; MCSA,totalSample,immune-Cytotoxic; MCSA,totalSample,immune-InfiltrationScore; ROSMAP,totalSample,immune-InfiltrationScore; SRP223445,totalSample,immune-CD4_naive; SRP223445,totalSample,immune-Exhausted; SRP223445,totalSample,immune-InfiltrationScore
FBXL2	Homo_723,female,AD.vs.control; Homo_723,male,AD.vs.control; Homo_723,female,AD.vs.MCI; Homo_723,male,AD.vs.MCI; Homo_714,female,AD.vs.control; Homo_714,male,AD.vs.control; Homo_714,female,AD.vs.MCI; Homo_714,male,AD.vs.MCI; Homo_633,female,AD.vs.control; Homo_633,female,AD.vs.MCI; Homo_633,female,MCI.vs.control; MCSA,totalSample,age; MCSA,control,age; Emory,totalSample,immune-CD4_naive; Homo_723,totalSample,immune-CD4_T; Homo_723,totalSample,immune-Monocyte; Homo_723,totalSample,immune-CD4_naive; Homo_723,totalSample,immune-nTreg; Homo_723,totalSample,immune-Central_memory; Homo_633,totalSample,immune-CD4_T; Homo_633,totalSample,immune-Tfh; Homo_633,totalSample,immune-Monocyte; Homo_633,totalSample,immune-CD4_naive; Homo_633,totalSample,immune-Th1; Homo_633,totalSample,immune-nTreg; Homo_633,totalSample,immune-Tr1; Homo_633,totalSample,immune-iTreg; MCSA,totalSample,immune-Monocyte; MCSA,totalSample,immune-CD4_T; MCSA,totalSample,immune-NKT; MCSA,totalSample,immune-CD4_naive; MCSA,totalSample,immune-Tr1; MCSA,totalSample,immune-nTreg; MCSA,totalSample,immune-iTreg; MCSA,totalSample,immune-Th17; MCSA,totalSample,immune-Tfh; MCSA,totalSample,immune-InfiltrationScore
CADM1	Homo_723,female,AD.vs.control; Homo_723,male,AD.vs.control; Homo_723,female,AD.vs.MCI; Homo_723,male,AD.vs.MCI; Homo_714,male,AD.vs.control; Homo_714,female,AD.vs.MCI; Homo_714,male,AD.vs.MCI; Homo_633,female,AD.vs.control; Homo_633,female,AD.vs.MCI; ROSMAP,male,MCI.vs.control; ROSMAP,control,male.vs.female; MCSA,MCI,age; ROSMAP,MCI,last.visit.age; ROSMAP,MCI,sample.age; Emory,totalSample,immune-CD4_T; Emory,totalSample,immune-Tr1; Emory,totalSample,immune-iTreg; Emory,totalSample,immune-InfiltrationScore; Homo_723,totalSample,immune-InfiltrationScore; Homo_723,totalSample,immune-Tfh; Homo_723,totalSample,immune-CD4_T; Homo_723,totalSample,immune-CD4_naive; Homo_723,totalSample,immune-MAIT; Homo_723,totalSample,immune-iTreg; Homo_723,totalSample,immune-Tr1; Homo_723,totalSample,immune-Macrophage; Homo_723,totalSample,immune-NKT; Homo_714,totalSample,immune-InfiltrationScore; Homo_714,totalSample,immune-Tfh; Homo_714,totalSample,immune-MAIT; Homo_714,totalSample,immune-CD4_naive; Homo_714,totalSample,immune-Effector_memory; Homo_714,totalSample,immune-CD4_T; Homo_714,totalSample,immune-NKT; Homo_714,totalSample,immune-iTreg; Homo_714,totalSample,immune-Macrophage; Homo_714,totalSample,immune-Bcell; Homo_633,totalSample,immune-InfiltrationScore; Homo_633,totalSample,immune-CD4_naive; Homo_633,totalSample,immune-Tfh; Homo_633,totalSample,immune-MAIT; Homo_633,totalSample,immune-Bcell; Homo_633,totalSample,immune-NKT; Homo_633,totalSample,immune-Effector_memory; Homo_633,totalSample,immune-CD4_T; MCSA,totalSample,immune-DC; MCSA,totalSample,immune-Macrophage; MCSA,totalSample,immune-CD4_T; MCSA,totalSample,immune-Gamma_delta; MCSA,totalSample,immune-CD4_naive; MCSA,totalSample,immune-Tr1; MCSA,totalSample,immune-nTreg; MCSA,totalSample,immune-iTreg; MCSA,totalSample,immune-Tfh; MCSA,totalSample,immune-CD8_naive; MCSA,totalSample,immune-MAIT; MCSA,totalSample,immune-Central_memory;
C4BPA	Homo_723,female,AD.vs.control; Homo_723,male,AD.vs.control; Homo_723,female,AD.vs.MCI; Homo_723,male,AD.vs.MCI; Homo_714,female,AD.vs.control; Homo_714,male,AD.vs.control; Homo_714,female,AD.vs.MCI; Homo_714,male,AD.vs.MCI; Homo_633,female,AD.vs.control; Homo_633,female,AD.vs.MCI; MCSA,female,MCI.vs.control; ROSMAP,female,MCI.vs.control; ROSMAP,AD,sample.age; ROSMAP,control,last.visit.age; ROSMAP,control,sample.age; Homo_723,totalSample,immune-Monocyte; Homo_723,totalSample,immune-Gamma_delta; Homo_723,totalSample,immune-Neutrophil; Homo_723,totalSample,immune-DC; Homo_714,totalSample,immune-Th2; Homo_714,totalSample,immune-Gamma_delta; Homo_714,totalSample,immune-Monocyte; Homo_633,totalSample,immune-Monocyte; Homo_633,totalSample,immune-Gamma_delta; Homo_633,totalSample,immune-Exhausted; Homo_633,totalSample,immune-Th2; MCSA,totalSample,immune-Monocyte; MCSA,totalSample,immune-Neutrophil; MCSA,totalSample,immune-CD8_T; MCSA,totalSample,immune-Gamma_delta; MCSA,totalSample,immune-Cytotoxic; MCSA,totalSample,immune-Exhausted; MCSA,totalSample,immune-Effector_memory
PROS1	Homo_723,female,AD.vs.control; Homo_723,male,AD.vs.control; Homo_723,female,AD.vs.MCI; Homo_723,male,AD.vs.MCI; Homo_723,female,MCI.vs.control; Homo_723,male,MCI.vs.control; Homo_714,female,AD.vs.control; Homo_714,male,AD.vs.control; Homo_714,female,AD.vs.MCI; Homo_714,male,AD.vs.MCI; Homo_714,female,MCI.vs.control; Homo_714,male,MCI.vs.control; Homo_633,female,AD.vs.control; Homo_633,female,AD.vs.MCI; ROSMAP,control,male.vs.female; SRP223445,female,AD.vs.control; Emory,totalSample,immune-Th1; Homo_723,totalSample,immune-Exhausted; Homo_723,totalSample,immune-CD8_T; Homo_723,totalSample,immune-Th17; Homo_723,totalSample,immune-Cytotoxic; Homo_723,totalSample,immune-Monocyte; Homo_714,totalSample,immune-Exhausted; Homo_714,totalSample,immune-Monocyte; Homo_714,totalSample,immune-CD8_T; Homo_714,totalSample,immune-Th17; Homo_633,totalSample,immune-Monocyte; Homo_633,totalSample,immune-Gamma_delta; Homo_633,totalSample,immune-Exhausted; MCSA,totalSample,immune-Monocyte; MCSA,totalSample,immune-CD8_T; MCSA,totalSample,immune-iTreg; MCSA,totalSample,immune-Cytotoxic; MCSA,totalSample,immune-Exhausted; ROSMAP,totalSample,immune-InfiltrationScore; SRP223445,totalSample,immune-CD4_naive; SRP223445,totalSample,immune-Exhausted
LRRC8B	Homo_723,female,AD.vs.control; Homo_723,male,AD.vs.control; Homo_723,female,AD.vs.MCI; Homo_723,male,AD.vs.MCI; Homo_714,female,AD.vs.control; Homo_714,male,AD.vs.control; Homo_714,female,AD.vs.MCI; Homo_714,male,AD.vs.MCI; Homo_633,female,AD.vs.control; Homo_633,female,AD.vs.MCI; ROSMAP,male,AD.vs.control; ROSMAP,male,MCI.vs.control; SRP223445,female,AD.vs.control; MCSA,MCI,age; Emory,totalSample,immune-CD4_T; Emory,totalSample,immune-Gamma_delta; Emory,totalSample,immune-CD4_naive; Emory,totalSample,immune-Tr1; Emory,totalSample,immune-iTreg; Emory,totalSample,immune-Th17; Emory,totalSample,immune-Cytotoxic; Emory,totalSample,immune-Central_memory; Emory,totalSample,immune-InfiltrationScore; Homo_723,totalSample,immune-Cytotoxic; Homo_723,totalSample,immune-CD4_naive; Homo_723,totalSample,immune-Central_memory; Homo_723,totalSample,immune-CD4_T; Homo_723,totalSample,immune-Th17; Homo_723,totalSample,immune-Th1; Homo_723,totalSample,immune-Tr1; Homo_723,totalSample,immune-Gamma_delta; Homo_723,totalSample,immune-nTreg; Homo_723,totalSample,immune-iTreg; Homo_723,totalSample,immune-Tfh; Homo_723,totalSample,immune-NKT; Homo_714,totalSample,immune-CD4_naive; Homo_714,totalSample,immune-NKT; Homo_714,totalSample,immune-Th1; Homo_714,totalSample,immune-Cytotoxic; Homo_714,totalSample,immune-CD4_T; Homo_714,totalSample,immune-Central_memory; Homo_714,totalSample,immune-iTreg; Homo_714,totalSample,immune-Th17; Homo_714,totalSample,immune-Tr1; Homo_714,totalSample,immune-Exhausted; Homo_714,totalSample,immune-Gamma_delta; Homo_714,totalSample,immune-Tfh; Homo_714,totalSample,immune-CD8_T; Homo_714,totalSample,immune-nTreg; Homo_633,totalSample,immune-Tfh; Homo_633,totalSample,immune-NKT; Homo_633,totalSample,immune-CD4_T; Homo_633,totalSample,immune-Tr1; Homo_633,totalSample,immune-CD4_naive; Homo_633,totalSample,immune-iTreg; Homo_633,totalSample,immune-Central_memory; Homo_633,totalSample,immune-Gamma_delta; Homo_633,totalSample,immune-Th1; Homo_633,totalSample,immune-nTreg; Homo_633,totalSample,immune-Cytotoxic; Homo_633,totalSample,immune-Effector_memory; Homo_633,totalSample,immune-InfiltrationScore; Homo_633,totalSample,immune-Th2; Homo_633,totalSample,immune-Th17; MCSA,totalSample,immune-DC; MCSA,totalSample,immune-CD4_T; MCSA,totalSample,immune-CD8_T; MCSA,totalSample,immune-Gamma_delta; MCSA,totalSample,immune-CD4_naive; MCSA,totalSample,immune-Tr1; MCSA,totalSample,immune-nTreg; MCSA,totalSample,immune-iTreg; MCSA,totalSample,immune-Th17; MCSA,totalSample,immune-Tfh; MCSA,totalSample,immune-CD8_naive; MCSA,totalSample,immune-Cytotoxic; MCSA,totalSample,immune-Exhausted; MCSA,totalSample,immune-MAIT; MCSA,totalSample,immune-Central_memory; SRP223445,totalSample,immune-CD4_naive; SRP223445,totalSample,immune-Exhausted; SRP223445,totalSample,immune-InfiltrationScore; ROSMAP,male-excludeAge90+,survival; ROSMAP,male-[illegible]
PAK3	Homo_723,female,AD.vs.control; Homo_723,female,AD.vs.MCI; Homo_723,female,MCI.vs.control; Homo_633,female,AD.vs.control; Homo_633,female,AD.vs.MCI; Emory,totalSample,immune-CD4_T; Emory,totalSample,immune-Tr1; Emory,totalSample,immune-iTreg; Emory,totalSample,immune-Th2; Emory,totalSample,immune-Th17; Homo_723,totalSample,immune-Tfh; Homo_723,totalSample,immune-Th2; Homo_723,totalSample,immune-CD4_T; Homo_723,totalSample,immune-iTreg; Homo_723,totalSample,immune-Tr1; Homo_723,totalSample,immune-CD4_naive; Homo_723,totalSample,immune-nTreg; Homo_723,totalSample,immune-Central_memory; Homo_723,totalSample,immune-DC; Homo_723,totalSample,immune-Effector_memory; Homo_723,totalSample,immune-Th17; Homo_714,totalSample,immune-Tfh; Homo_714,totalSample,immune-Th2; Homo_714,totalSample,immune-CD4_T; Homo_714,totalSample,immune-Tr1; Homo_714,totalSample,immune-iTreg; Homo_714,totalSample,immune-CD4_naive; Homo_714,totalSample,immune-Central_memory; Homo_714,totalSample,immune-Effector_memory; Homo_714,totalSample,immune-DC; Homo_714,totalSample,immune-nTreg; Homo_714,totalSample,immune-InfiltrationScore; Homo_633,totalSample,immune-Tfh; Homo_633,totalSample,immune-CD4_T; Homo_633,totalSample,immune-CD4_naive; Homo_633,totalSample,immune-Central_memory; Homo_633,totalSample,immune-Th2; Homo_633,totalSample,immune-Tr1; Homo_633,totalSample,immune-Effector_memory; Homo_633,totalSample,immune-iTreg; Homo_633,totalSample,immune-Th17; Homo_633,totalSample,immune-NKT; Homo_633,totalSample,immune-InfiltrationScore; Homo_633,totalSample,immune-DC; Homo_633,totalSample,immune-nTreg; Homo_633,totalSample,immune-MAIT; Homo_633,totalSample,immune-Gamma_delta; MCSA,totalSample,immune-DC; MCSA,totalSample,immune-Macrophage; MCSA,totalSample,immune-CD4_T; MCSA,totalSample,immune-CD4_naive; MCSA,totalSample,immune-Tr1; MCSA,totalSample,immune-nTreg; MCSA,totalSample,immune-iTreg; MCSA,totalSample,immune-Th17; MCSA,totalSample,immune-Tfh; MCSA,totalSample,immune-CD8_naive; MCSA,totalSample,immune-Cytotoxic; MCSA,totalSample,immune-Exhausted; MCSA,totalSample,immune-MAIT; MCSA,totalSample,immune-Central_memory; ROSMAP,totalSample,immune-InfiltrationScore
CARS2	Homo_723,female,AD.vs.control; Homo_723,male,AD.vs.control; Homo_723,female,AD.vs.MCI; Homo_723,male,AD.vs.MCI; Homo_714,female,AD.vs.control; Homo_714,male,AD.vs.control; Homo_714,female,AD.vs.MCI; Homo_714,male,AD.vs.MCI; Homo_633,female,AD.vs.control; Homo_633,female,AD.vs.MCI; Emory,totalSample,age; Emory,MCI,age; MCSA,totalSample,age; MCSA,control,age; MCSA,MCI,age; ROSMAP,AD,last.visit.age; ROSMAP,AD,sample.age; Emory,totalSample,immune-Monocyte; Emory,totalSample,immune-Neutrophil; Emory,totalSample,immune-CD8_T; Emory,totalSample,immune-Gamma_delta; Emory,totalSample,immune-CD8_naive; Emory,totalSample,immune-Cytotoxic; Emory,totalSample,immune-Exhausted; Homo_723,totalSample,immune-InfiltrationScore; Homo_723,totalSample,immune-Effector_memory; Homo_723,totalSample,immune-Th1; Homo_723,totalSample,immune-Cytotoxic; Homo_723,totalSample,immune-CD8_T; Homo_723,totalSample,immune-Bcell; Homo_714,totalSample,immune-Th1; Homo_714,totalSample,immune-Effector_memory; Homo_714,totalSample,immune-Th2; Homo_714,totalSample,immune-InfiltrationScore; Homo_633,totalSample,immune-InfiltrationScore; Homo_633,totalSample,immune-Exhausted; Homo_633,totalSample,immune-DC; Homo_633,totalSample,immune-Th1; Homo_633,totalSample,immune-nTreg; MCSA,totalSample,immune-DC; MCSA,totalSample,immune-Monocyte; MCSA,totalSample,immune-Macrophage; MCSA,totalSample,immune-CD8_T; MCSA,totalSample,immune-NKT; MCSA,totalSample,immune-Gamma_delta; MCSA,totalSample,immune-iTreg; MCSA,totalSample,immune-Th2; MCSA,totalSample,immune-Cytotoxic; MCSA,totalSample,immune-Effector_memory; MCSA,totalSample,immune-InfiltrationScore; ROSMAP,totalSample,immune-Effector_memory; SRP223445,totalSample,immune-CD4_naive; SRP223445,totalSample,immune-Th17; SRP223445,totalSample,immune-Exhausted; SRP223445,totalSample,immune-InfiltrationScore
SLC2A9	Homo_723,female,AD.vs.control; Homo_723,male,AD.vs.control; Homo_723,female,AD.vs.MCI; Homo_723,male,AD.vs.MCI; Homo_714,female,AD.vs.control; Homo_714,male,AD.vs.control; Homo_714,female,AD.vs.MCI; Homo_714,male,AD.vs.MCI; Homo_633,female,AD.vs.control; Homo_633,female,AD.vs.MCI; ROSMAP,female,AD.vs.control; Emory,MCI,age; Emory,totalSample,immune-Monocyte; Emory,totalSample,immune-CD8_T; Emory,totalSample,immune-Gamma_delta; Emory,totalSample,immune-Tfh; Emory,totalSample,immune-CD8_naive; Homo_723,totalSample,immune-Gamma_delta; Homo_723,totalSample,immune-InfiltrationScore; Homo_723,totalSample,immune-Bcell; Homo_633,totalSample,immune-InfiltrationScore; Homo_633,totalSample,immune-NKT; Homo_633,totalSample,immune-CD8_T; Homo_633,totalSample,immune-Gamma_delta; MCSA,totalSample,immune-CD8_T; MCSA,totalSample,immune-Gamma_delta; MCSA,totalSample,immune-iTreg; MCSA,totalSample,immune-Th17; MCSA,totalSample,immune-Cytotoxic; MCSA,totalSample,immune-Exhausted; ROSMAP,totalSample,immune-Cytotoxic; ROSMAP,totalSample,immune-Effector_memory
NEXN	Homo_723,female,AD.vs.control; Homo_723,male,AD.vs.control; Homo_723,female,AD.vs.MCI; Homo_723,male,AD.vs.MCI; Homo_723,female,MCI.vs.control; Homo_723,male,MCI.vs.control; Homo_714,female,AD.vs.control; Homo_714,male,AD.vs.control; Homo_714,female,AD.vs.MCI; Homo_714,male,AD.vs.MCI; Homo_714,female,MCI.vs.control; Homo_714,male,MCI.vs.control; Homo_633,female,AD.vs.control; Homo_633,female,AD.vs.MCI; SRP223445,female,AD.vs.control; MCSA,totalSample,age; MCSA,MCI,age; Emory,totalSample,immune-Th17; Homo_723,totalSample,immune-Exhausted; Homo_723,totalSample,immune-CD8_T; Homo_723,totalSample,immune-Cytotoxic; Homo_723,totalSample,immune-Th17; Homo_723,totalSample,immune-Th1; Homo_723,totalSample,immune-Monocyte; Homo_723,totalSample,immune-NK; Homo_714,totalSample,immune-Monocyte; Homo_714,totalSample,immune-Exhausted; Homo_714,totalSample,immune-CD8_T; Homo_714,totalSample,immune-Th17; Homo_714,totalSample,immune-NK; Homo_714,totalSample,immune-Th1; Homo_633,totalSample,immune-Monocyte; Homo_633,totalSample,immune-Tfh; Homo_633,totalSample,immune-Th2; Homo_633,totalSample,immune-Gamma_delta; MCSA,totalSample,immune-DC; MCSA,totalSample,immune-Monocyte; MCSA,totalSample,immune-CD8_T; MCSA,totalSample,immune-Tr1; MCSA,totalSample,immune-iTreg; MCSA,totalSample,immune-Th2; MCSA,totalSample,immune-Cytotoxic; MCSA,totalSample,immune-Exhausted; MCSA,totalSample,immune-Effector_memory; ROSMAP,totalSample,immune-Effector_memory; ROSMAP,totalSample,immune-InfiltrationScore; SRP223445,totalSample,immune-CD4_naive
PGAP1	Homo_723,female,AD.vs.control; Homo_723,male,AD.vs.control; Homo_723,female,AD.vs.MCI; Homo_723,male,AD.vs.MCI; Homo_723,female,MCI.vs.control; Homo_714,female,AD.vs.control; Homo_714,male,AD.vs.control; Homo_714,female,AD.vs.MCI; Homo_714,male,AD.vs.MCI; Homo_633,female,AD.vs.control; Homo_633,female,AD.vs.MCI; ROSMAP,female,AD.vs.control; MCSA,MCI,age; ROSMAP,MCI,last.visit.age; Emory,totalSample,immune-CD4_T; Emory,totalSample,immune-Tr1; Emory,totalSample,immune-iTreg; Emory,totalSample,immune-Tfh; Emory,totalSample,immune-Central_memory; Emory,totalSample,immune-InfiltrationScore; Homo_723,totalSample,immune-CD4_T; Homo_723,totalSample,immune-CD4_naive; Homo_723,totalSample,immune-Tr1; Homo_723,totalSample,immune-Tfh; Homo_723,totalSample,immune-Th2; Homo_723,totalSample,immune-iTreg; Homo_723,totalSample,immune-Cytotoxic; Homo_723,totalSample,immune-Th1; Homo_723,totalSample,immune-Central_memory; Homo_723,totalSample,immune-Th17; Homo_723,totalSample,immune-nTreg; Homo_723,totalSample,immune-Exhausted; Homo_723,totalSample,immune-Gamma_delta; Homo_723,totalSample,immune-InfiltrationScore; Homo_714,totalSample,immune-Tfh; Homo_714,totalSample,immune-CD4_T; Homo_714,totalSample,immune-CD4_naive; Homo_714,totalSample,immune-Tr1; Homo_714,totalSample,immune-iTreg; Homo_714,totalSample,immune-Central_memory; Homo_714,totalSample,immune-Th2; Homo_714,totalSample,immune-Th1; Homo_714,totalSample,immune-Cytotoxic; Homo_714,totalSample,immune-Th17; Homo_714,totalSample,immune-nTreg; Homo_714,totalSample,immune-NKT; Homo_714,totalSample,immune-Gamma_delta; Homo_714,totalSample,immune-Exhausted; Homo_714,totalSample,immune-InfiltrationScore; Homo_633,totalSample,immune-Tfh; Homo_633,totalSample,immune-CD4_T; Homo_633,totalSample,immune-CD4_naive; Homo_633,totalSample,immune-Tr1; Homo_633,totalSample,immune-Central_memory; Homo_633,totalSample,immune-Th2; Homo_633,totalSample,immune-iTreg; Homo_633,totalSample,immune-Effector_memory; Homo_633,totalSample,immune-NKT; Homo_633,totalSample,immune-Gamma_delta; Homo_633,totalSample,immune-Th17; Homo_633,totalSample,immune-nTreg; Homo_633,totalSample,immune-Th1; Homo_633,totalSample,immune-Exhausted; Homo_633,totalSample,immune-InfiltrationScore; Homo_633,totalSample,immune-DC; Homo_633,totalSample,immune-Cytotoxic; MCSA,totalSample,immune-DC; MCSA,totalSample,immune-Monocyte; MCSA,totalSample,immune-Macrophage; MCSA,totalSample,immune-CD4_T; MCSA,totalSample,immune-CD8_T; MCSA,totalSample,immune-Gamma_delta; MCSA,totalSample,immune-CD4_naive; MCSA,totalSample,immune-Tr1; MCSA,totalSample,immune-nTreg; MCSA,totalSample,immune-iTreg; MCSA,totalSample,immune-Th17; MCSA,totalSample,immune-Tfh; MCSA,totalSample,immune-CD8_naive; MCSA,totalSample,immune-Cytotoxic; MCSA,totalSample,immune-Exhausted; MCSA,totalSample,immune-MAIT; MCSA,totalSample,immune-Central_memory

CTDP1	Homo_723,female,AD.vs.control; Homo_723,male,AD.vs.control; Homo_723,female,MCI.vs.control; Homo_723,male,MCI.vs.control; Homo_723,AD,male.vs.female; Homo_723,control,male.vs.female; Homo_723,MCI,male.vs.female; Homo_714,female,AD.vs.control; Homo_714,male,AD.vs.control; Homo_714,female,MCI.vs.control; Homo_714,male,MCI.vs.control; Homo_714,control,male.vs.female; Homo_714,MCI,male.vs.female; Homo_633,female,AD.vs.control; Homo_633,female,AD.vs.MCI; Homo_633,female,MCI.vs.control; Homo_633,male,MCI.vs.control; Homo_633,control,male.vs.female; ROSMAP,female,AD.vs.control; Emory,totalSample,age; Emory,MCI,age; MCSA,MCI,age; Emory,totalSample,immune-CD8_T; Emory,totalSample,immune-Gamma_delta; Emory,totalSample,immune-Th17; Emory,totalSample,immune-Tfh; Emory,totalSample,immune-CD8_naive; Emory,totalSample,immune-Cytotoxic; Homo_723,totalSample,immune-Monocyte; Homo_723,totalSample,immune-DC; Homo_723,totalSample,immune-Central_memory; Homo_723,totalSample,immune-InfiltrationScore; Homo_723,totalSample,immune-nTreg; Homo_714,totalSample,immune-Monocyte; Homo_714,totalSample,immune-DC; Homo_633,totalSample,immune-Monocyte; Homo_633,totalSample,immune-InfiltrationScore; Homo_633,totalSample,immune-nTreg; MCSA,totalSample,immune-DC; MCSA,totalSample,immune-Monocyte; MCSA,totalSample,immune-NKT; MCSA,totalSample,immune-Gamma_delta; MCSA,totalSample,immune-CD4_naive; MCSA,totalSample,immune-nTreg; MCSA,totalSample,immune-Th2; MCSA,totalSample,immune-Tfh; MCSA,totalSample,immune-Central_memory; MCSA,totalSample,immune-Effector_memory; SRP223445,totalSample,immune-InfiltrationScore
MBOAT1	Homo_723,female,AD.vs.control; Homo_723,male,AD.vs.control; Homo_723,female,AD.vs.MCI; Homo_723,male,AD.vs.MCI; Homo_723,female,MCI.vs.control; Homo_714,female,AD.vs.control; Homo_714,male,AD.vs.control; Homo_714,female,AD.vs.MCI; Homo_714,male,AD.vs.MCI; Homo_714,female,MCI.vs.control; Homo_633,female,AD.vs.control; Homo_633,female,AD.vs.MCI; Homo_633,female,MCI.vs.control; Emory,MCI,age; ROSMAP,AD,last.visit.age; Emory,totalSample,immune-Monocyte; Emory,totalSample,immune-Neutrophil; Emory,totalSample,immune-CD8_T; Emory,totalSample,immune-Gamma_delta; Emory,totalSample,immune-Th1; Emory,totalSample,immune-Th17; Emory,totalSample,immune-Tfh; Emory,totalSample,immune-CD8_naive; Emory,totalSample,immune-Exhausted; Homo_723,totalSample,immune-Monocyte; Homo_723,totalSample,immune-Th2; Homo_723,totalSample,immune-DC; Homo_723,totalSample,immune-Gamma_delta; Homo_723,totalSample,immune-Tfh; Homo_723,totalSample,immune-iTreg; Homo_723,totalSample,immune-Neutrophil; Homo_723,totalSample,immune-Exhausted; Homo_723,totalSample,immune-Bcell; Homo_723,totalSample,immune-InfiltrationScore; Homo_723,totalSample,immune-Tr1; Homo_723,totalSample,immune-Effector_memory; Homo_714,totalSample,immune-Th2; Homo_714,totalSample,immune-Monocyte; Homo_714,totalSample,immune-Tfh; Homo_714,totalSample,immune-Gamma_delta; Homo_714,totalSample,immune-CD4_T; Homo_714,totalSample,immune-DC; Homo_714,totalSample,immune-Tr1; Homo_633,totalSample,immune-Th2; Homo_633,totalSample,immune-Monocyte; Homo_633,totalSample,immune-Exhausted; Homo_633,totalSample,immune-Th17; Homo_633,totalSample,immune-Gamma_delta; Homo_633,totalSample,immune-Tfh; Homo_633,totalSample,immune-CD4_T; MCSA,totalSample,immune-DC; MCSA,totalSample,immune-Monocyte; MCSA,totalSample,immune-Neutrophil; MCSA,totalSample,immune-CD4_T; MCSA,totalSample,immune-CD8_T; MCSA,totalSample,immune-Gamma_delta; MCSA,totalSample,immune-Tr1; MCSA,totalSample,immune-nTreg; MCSA,totalSample,immune-iTreg; MCSA,totalSample,immune-Th17; MCSA,totalSample,immune-Cytotoxic; MCSA,totalSample,immune-Exhausted; MCSA,totalSample,immune-Effector_memory; ROSMAP,totalSample,immune-Effector_memory
TRIM39	Homo_723,female,AD.vs.control; Homo_723,male,AD.vs.control; Homo_723,female,AD.vs.MCI; Homo_723,male,AD.vs.MCI; Homo_723,female,MCI.vs.control; Homo_714,female,AD.vs.control; Homo_714,male,AD.vs.control; Homo_714,female,AD.vs.MCI; Homo_714,male,AD.vs.MCI; Homo_714,female,MCI.vs.control; Homo_633,female,AD.vs.control; Homo_633,female,AD.vs.MCI; Homo_633,female,MCI.vs.control; ROSMAP,female,AD.vs.control; Emory,totalSample,immune-Gamma_delta; Emory,totalSample,immune-Tr1; Emory,totalSample,immune-iTreg; Emory,totalSample,immune-Th17; Emory,totalSample,immune-Cytotoxic; Emory,totalSample,immune-InfiltrationScore; Homo_723,totalSample,immune-Th2; Homo_723,totalSample,immune-Tfh; Homo_723,totalSample,immune-Monocyte; Homo_723,totalSample,immune-DC; Homo_723,totalSample,immune-InfiltrationScore; Homo_723,totalSample,immune-iTreg; Homo_723,totalSample,immune-Effector_memory; Homo_723,totalSample,immune-Bcell; Homo_723,totalSample,immune-CD4_T; Homo_723,totalSample,immune-Tr1; Homo_723,totalSample,immune-Gamma_delta; Homo_714,totalSample,immune-Th2; Homo_714,totalSample,immune-Tfh; Homo_714,totalSample,immune-Effector_memory; Homo_714,totalSample,immune-CD4_T; Homo_714,totalSample,immune-Monocyte; Homo_714,totalSample,immune-InfiltrationScore; Homo_714,totalSample,immune-Tr1; Homo_714,totalSample,immune-Gamma_delta; Homo_633,totalSample,immune-Th2; Homo_633,totalSample,immune-Monocyte; Homo_633,totalSample,immune-Tfh; Homo_633,totalSample,immune-InfiltrationScore; Homo_633,totalSample,immune-Exhausted; Homo_633,totalSample,immune-Th17; Homo_633,totalSample,immune-CD4_T; Homo_633,totalSample,immune-Effector_memory; Homo_633,totalSample,immune-Gamma_delta; MCSA,totalSample,immune-DC; MCSA,totalSample,immune-Neutrophil; MCSA,totalSample,immune-CD4_T; MCSA,totalSample,immune-Gamma_delta; MCSA,totalSample,immune-CD4_naive; MCSA,totalSample,immune-Tr1; MCSA,totalSample,immune-nTreg; MCSA,totalSample,immune-iTreg; MCSA,totalSample,immune-Th1; MCSA,totalSample,immune-Th17; MCSA,totalSample,immune-Tfh; MCSA,totalSample,immune-Cytotoxic; MCSA,totalSample,immune-Exhausted; MCSA,totalSample,immune-Central_memory; SRP223445,totalSample,immune-CD4_naive; SRP223445,totalSample,immune-Th17; SRP223445,totalSample,immune-Exhausted; [illegible]
ATL1	Homo_723,female,AD.vs.control; Homo_723,male,AD.vs.control; Homo_723,female,AD.vs.MCI; Homo_723,male,AD.vs.MCI; Homo_714,female,AD.vs.control; Homo_714,male,AD.vs.control; Homo_714,female,AD.vs.MCI; Homo_714,male,AD.vs.MCI; Homo_633,female,AD.vs.control; Homo_633,female,AD.vs.MCI; ROSMAP,male,AD.vs.control; MCSA,MCI,age; Emory,totalSample,immune-Neutrophil; Emory,totalSample,immune-InfiltrationScore; Homo_723,totalSample,immune-CD4_naive; Homo_723,totalSample,immune-InfiltrationScore; Homo_723,totalSample,immune-CD4_T; Homo_723,totalSample,immune-NKT; Homo_723,totalSample,immune-Neutrophil; Homo_723,totalSample,immune-Central_memory; Homo_723,totalSample,immune-Tfh; Homo_723,totalSample,immune-Th1; Homo_723,totalSample,immune-Th17; Homo_723,totalSample,immune-Tr1; Homo_723,totalSample,immune-nTreg; Homo_723,totalSample,immune-Cytotoxic; Homo_714,totalSample,immune-InfiltrationScore; Homo_714,totalSample,immune-CD4_naive; Homo_714,totalSample,immune-NKT; Homo_714,totalSample,immune-Neutrophil; Homo_714,totalSample,immune-CD4_T; Homo_714,totalSample,immune-Central_memory; Homo_714,totalSample,immune-Tfh; Homo_633,totalSample,immune-InfiltrationScore; Homo_633,totalSample,immune-Tfh; Homo_633,totalSample,immune-CD4_naive; Homo_633,totalSample,immune-CD4_T; Homo_633,totalSample,immune-NKT; Homo_633,totalSample,immune-Effector_memory; Homo_633,totalSample,immune-Central_memory; Homo_633,totalSample,immune-Tr1; Homo_633,totalSample,immune-Neutrophil; Homo_633,totalSample,immune-iTreg; MCSA,totalSample,immune-DC; MCSA,totalSample,immune-Macrophage; MCSA,totalSample,immune-CD4_T; MCSA,totalSample,immune-CD4_naive; MCSA,totalSample,immune-Tr1; MCSA,totalSample,immune-nTreg; MCSA,totalSample,immune-iTreg; MCSA,totalSample,immune-Tfh; MCSA,totalSample,immune-CD8_naive; MCSA,totalSample,immune-Exhausted; MCSA,totalSample,immune-MAIT; MCSA,totalSample,immune-Central_memory; ROSMAP,totalSample,immune-[illegible]
ARRDC5	Homo_723,female,AD.vs.control; Homo_723,male,AD.vs.control; Homo_723,female,AD.vs.MCI; Homo_723,male,AD.vs.MCI; Homo_714,female,AD.vs.control; Homo_714,male,AD.vs.control; Homo_714,female,AD.vs.MCI; Homo_714,male,AD.vs.MCI; Homo_633,female,AD.vs.control; Homo_633,female,AD.vs.MCI; MCSA,totalSample,age; MCSA,control,age; MCSA,MCI,age; ROSMAP,AD,last.visit.age; Emory,totalSample,immune-Monocyte; Emory,totalSample,immune-Macrophage; Emory,totalSample,immune-Neutrophil; Emory,totalSample,immune-CD4_T; Emory,totalSample,immune-CD8_T; Emory,totalSample,immune-CD4_naive; Emory,totalSample,immune-Tr1; Emory,totalSample,immune-iTreg; Emory,totalSample,immune-Tfh; Emory,totalSample,immune-CD8_naive; Emory,totalSample,immune-Exhausted; Emory,totalSample,immune-Central_memory; Emory,totalSample,immune-InfiltrationScore; Homo_723,totalSample,immune-CD8_T; Homo_723,totalSample,immune-Exhausted; Homo_723,totalSample,immune-Monocyte; Homo_723,totalSample,immune-iTreg; Homo_723,totalSample,immune-Tfh; Homo_723,totalSample,immune-Cytotoxic; Homo_723,totalSample,immune-InfiltrationScore; Homo_723,totalSample,immune-Central_memory; Homo_723,totalSample,immune-nTreg; Homo_723,totalSample,immune-Macrophage; Homo_723,totalSample,immune-CD4_naive; Homo_723,totalSample,immune-CD4_T; Homo_723,totalSample,immune-CD8_naive; Homo_723,totalSample,immune-MAIT; Homo_714,totalSample,immune-Monocyte; Homo_714,totalSample,immune-nTreg; Homo_714,totalSample,immune-Tfh; Homo_714,totalSample,immune-Macrophage; Homo_714,totalSample,immune-CD8_T; Homo_714,totalSample,immune-Effector_memory; Homo_714,totalSample,immune-iTreg; Homo_714,totalSample,immune-InfiltrationScore; Homo_714,totalSample,immune-Central_memory; Homo_714,totalSample,immune-CD4_T; Homo_714,totalSample,immune-Tr1; Homo_714,totalSample,immune-CD4_naive; Homo_633,totalSample,immune-Monocyte; Homo_633,totalSample,immune-nTreg; Homo_633,totalSample,immune-InfiltrationScore; Homo_633,totalSample,immune-CD4_naive; Homo_633,totalSample,immune-iTreg; Homo_633,totalSample,immune-Tfh; Homo_633,totalSample,immune-Central_memory; Homo_633,totalSample,immune-Tr1; Homo_633,totalSample,immune-DC; Homo_633,totalSample,immune-Effector_memory; Homo_633,totalSample,immune-Macrophage; Homo_633,totalSample,immune-CD4_T; Homo_633,totalSample,immune-Neutrophil; MCSA,totalSample,immune-DC; MCSA,totalSample,immune-Monocyte; MCSA,totalSample,immune-Neutrophil; MCSA,totalSample,immune-CD4_T; MCSA,totalSample,immune-CD8_T; MCSA,totalSample,immune-CD4_naive; MCSA,totalSample,immune-Tr1; MCSA,totalSample,immune-nTreg; MCSA,totalSample,immune-Th2; MCSA,totalSample,immune-Th17; MCSA,totalSample,immune-Tfh; MCSA,totalSample,immune-Cytotoxic; MCSA,totalSample,immune-Central_memory; MCSA,totalSample,immune-Effector_memory; MCSA,totalSample,immune-InfiltrationScore; [illegible]
CRIP3	Homo_723,female,AD.vs.control; Homo_723,male,AD.vs.control; Homo_723,female,AD.vs.MCI; Homo_723,male,AD.vs.MCI; Homo_714,female,AD.vs.control; Homo_714,male,AD.vs.control; Homo_714,female,AD.vs.MCI; Homo_714,male,AD.vs.MCI; Homo_633,female,AD.vs.control; Homo_633,female,AD.vs.MCI; ROSMAP,female,AD.vs.control; MCSA,totalSample,age; MCSA,control,age; Emory,totalSample,immune-B_cell; Emory,totalSample,immune-Neutrophil; Homo_723,totalSample,immune-CD8_T; Homo_723,totalSample,immune-Th2; Homo_723,totalSample,immune-Tfh; Homo_723,totalSample,immune-iTreg; Homo_723,totalSample,immune-Macrophage; Homo_723,totalSample,immune-nTreg; Homo_723,totalSample,immune-Effector_memory; Homo_723,totalSample,immune-Cytotoxic; Homo_723,totalSample,immune-CD4_T; Homo_714,totalSample,immune-Th2; Homo_714,totalSample,immune-Tfh; Homo_714,totalSample,immune-nTreg; Homo_714,totalSample,immune-Effector_memory; Homo_714,totalSample,immune-CD8_T; Homo_714,totalSample,immune-CD4_T; Homo_714,totalSample,immune-Macrophage; Homo_714,totalSample,immune-Central_memory; Homo_633,totalSample,immune-nTreg; Homo_633,totalSample,immune-Effector_memory; Homo_633,totalSample,immune-Tfh; Homo_633,totalSample,immune-Neutrophil; Homo_633,totalSample,immune-Th2; Homo_633,totalSample,immune-Central_memory; Homo_633,totalSample,immune-CD4_T; Homo_633,totalSample,immune-DC; MCSA,totalSample,immune-DC; MCSA,totalSample,immune-Monocyte; MCSA,totalSample,immune-Neutrophil; MCSA,totalSample,immune-CD4_T; MCSA,totalSample,immune-CD8_T; MCSA,totalSample,immune-CD4_naive; MCSA,totalSample,immune-Tr1; MCSA,totalSample,immune-nTreg; MCSA,totalSample,immune-Th17; MCSA,totalSample,immune-Tfh; MCSA,totalSample,immune-Cytotoxic; MCSA,totalSample,immune-Central_memory; MCSA,totalSample,immune-Effector_memory; MCSA,totalSample,immune-InfiltrationScore; ROSMAP,totalSample,immune-Effector_memory
FN1	Homo_723,female,AD.vs.control; Homo_723,male,AD.vs.control; Homo_723,female,AD.vs.MCI; Homo_723,male,AD.vs.MCI; Homo_723,female,MCI.vs.control; Homo_723,male,MCI.vs.control; Homo_714,female,AD.vs.control; Homo_714,male,AD.vs.control; Homo_714,female,AD.vs.MCI; Homo_714,male,AD.vs.MCI; Homo_714,female,MCI.vs.control; Homo_714,male,MCI.vs.control; Homo_633,female,AD.vs.control; Homo_633,female,AD.vs.MCI; ROSMAP,male,AD.vs.MCI; ROSMAP,control,male.vs.female; ROSMAP,MCI,male.vs.female; ROSMAP,MCI,last.visit.age; Emory,totalSample,immune-Tr1; Emory,totalSample,immune-iTreg; Emory,totalSample,immune-InfiltrationScore; Homo_723,totalSample,immune-Th17; Homo_723,totalSample,immune-CD4_naive; Homo_723,totalSample,immune-Th1; Homo_723,totalSample,immune-Cytotoxic; Homo_723,totalSample,immune-CD4_T; Homo_723,totalSample,immune-nTreg; Homo_723,totalSample,immune-Central_memory; Homo_723,totalSample,immune-Neutrophil; Homo_723,totalSample,immune-Exhausted; Homo_723,totalSample,immune-InfiltrationScore; Homo_723,totalSample,immune-Tr1; Homo_714,totalSample,immune-CD4_naive; Homo_714,totalSample,immune-NKT; Homo_714,totalSample,immune-InfiltrationScore; Homo_714,totalSample,immune-Th1; Homo_714,totalSample,immune-Exhausted; Homo_714,totalSample,immune-Th17; Homo_633,totalSample,immune-Tfh; Homo_633,totalSample,immune-CD4_T; Homo_633,totalSample,immune-Tr1; Homo_633,totalSample,immune-CD4_naive; Homo_633,totalSample,immune-NKT; Homo_633,totalSample,immune-InfiltrationScore; Homo_633,totalSample,immune-Effector_memory; Homo_633,totalSample,immune-iTreg; Homo_633,totalSample,immune-nTreg; Homo_633,totalSample,immune-Gamma_delta; Homo_633,totalSample,immune-Central_memory; MCSA,totalSample,immune-CD4_T; MCSA,totalSample,immune-CD4_naive; MCSA,totalSample,immune-Tr1; MCSA,totalSample,immune-nTreg; MCSA,totalSample,immune-iTreg; MCSA,totalSample,immune-Th17; MCSA,totalSample,immune-Tfh; MCSA,totalSample,immune-CD8_naive; MCSA,totalSample,immune-Cytotoxic; MCSA,totalSample,immune-Exhausted; MCSA,totalSample,immune-Central_memory; SRP223445,totalSample,immune-CD4_naive; SRP223445,totalSample,immune-Exhausted; SRP223445,totalSample,immune-InfiltrationScore
LOC105372412	Homo_723,female,AD.vs.control; Homo_723,male,AD.vs.control; Homo_723,female,AD.vs.MCI; Homo_723,male,AD.vs.MCI; Homo_723,female,MCI.vs.control; Homo_714,female,AD.vs.control; Homo_714,male,AD.vs.control; Homo_714,female,AD.vs.MCI; Homo_714,male,AD.vs.MCI; Homo_714,female,MCI.vs.control; Homo_633,female,AD.vs.control; Homo_633,female,AD.vs.MCI; MCSA,MCI,age; Emory,totalSample,immune-Macrophage; Emory,totalSample,immune-NK; Emory,totalSample,immune-Neutrophil; Emory,totalSample,immune-CD8_T; Emory,totalSample,immune-Gamma_delta; Emory,totalSample,immune-Th17; Emory,totalSample,immune-Tfh; Emory,totalSample,immune-CD8_naive; Emory,totalSample,immune-Cytotoxic; Emory,totalSample,immune-Exhausted; Emory,totalSample,immune-Effector_memory; Homo_723,totalSample,immune-Neutrophil; Homo_723,totalSample,immune-Th1; Homo_723,totalSample,immune-Gamma_delta; Homo_714,totalSample,immune-Neutrophil; Homo_714,totalSample,immune-Th1; Homo_633,totalSample,immune-Neutrophil; Homo_633,totalSample,immune-MAIT; Homo_633,totalSample,immune-CD8_T; Homo_633,totalSample,immune-CD4_naive; Homo_633,totalSample,immune-Effector_memory; MCSA,totalSample,immune-DC; MCSA,totalSample,immune-Bcell; MCSA,totalSample,immune-Macrophage; MCSA,totalSample,immune-Neutrophil; MCSA,totalSample,immune-CD4_T; MCSA,totalSample,immune-CD4_naive; MCSA,totalSample,immune-Tr1; MCSA,totalSample,immune-nTreg; MCSA,totalSample,immune-iTreg; MCSA,totalSample,immune-Tfh; MCSA,totalSample,immune-CD8_naive; MCSA,totalSample,immune-MAIT; MCSA,totalSample,immune-Central_memory
CLEC12B	Homo_723,female,AD.vs.control; Homo_723,male,AD.vs.control; Homo_723,female,AD.vs.MCI; Homo_723,male,AD.vs.MCI; Homo_723,female,MCI.vs.control; Homo_723,male,MCI.vs.control; Homo_714,female,AD.vs.control; Homo_714,male,AD.vs.control; Homo_714,female,AD.vs.MCI; Homo_714,male,AD.vs.MCI; Homo_714,female,MCI.vs.control; Homo_714,male,MCI.vs.control; Homo_633,female,AD.vs.control; Homo_633,female,AD.vs.MCI; Homo_633,female,MCI.vs.control; Homo_633,male,MCI.vs.control; ROSMAP,female,MCI.vs.control; ROSMAP,AD,last.visit.age; Emory,totalSample,immune-Monocyte; Emory,totalSample,immune-Gamma_delta; Homo_723,totalSample,immune-Monocyte; Homo_723,totalSample,immune-DC; Homo_723,totalSample,immune-Th2; Homo_723,totalSample,immune-Tfh; Homo_723,totalSample,immune-Effector_memory; Homo_723,totalSample,immune-Gamma_delta; Homo_723,totalSample,immune-Bcell; Homo_723,totalSample,immune-iTreg; Homo_714,totalSample,immune-Th2; Homo_714,totalSample,immune-Monocyte; Homo_714,totalSample,immune-Tfh; Homo_714,totalSample,immune-Gamma_delta; Homo_714,totalSample,immune-Effector_memory; Homo_633,totalSample,immune-Monocyte; Homo_633,totalSample,immune-Th2; Homo_633,totalSample,immune-Exhausted; Homo_633,totalSample,immune-Th17; Homo_633,totalSample,immune-Gamma_delta; Homo_633,totalSample,immune-Tfh; MCSA,totalSample,immune-CD8_T; MCSA,totalSample,immune-Tr1; MCSA,totalSample,immune-iTreg; MCSA,totalSample,immune-Cytotoxic; MCSA,totalSample,immune-Exhausted; ROSMAP,totalSample,immune-Effector_memory
HEBP2	Homo_723,female,AD.vs.control; Homo_723,male,AD.vs.control; Homo_723,female,AD.vs.MCI; Homo_723,male,AD.vs.MCI; Homo_714,female,AD.vs.control; Homo_714,male,AD.vs.control; Homo_714,female,AD.vs.MCI; Homo_714,male,AD.vs.MCI; Homo_633,female,AD.vs.control; Homo_633,female,AD.vs.MCI; Emory,totalSample,age; Emory,MCI,age; MCSA,MCI,age; Emory,totalSample,immune-DC; Emory,totalSample,immune-Monocyte; Emory,totalSample,immune-Neutrophil; Emory,totalSample,immune-CD8_T; Emory,totalSample,immune-Gamma_delta; Emory,totalSample,immune-CD8_naive; Emory,totalSample,immune-Exhausted; Homo_723,totalSample,immune-Monocyte; Homo_723,totalSample,immune-DC; Homo_723,totalSample,immune-Th2; Homo_723,totalSample,immune-Gamma_delta; Homo_723,totalSample,immune-Neutrophil; Homo_723,totalSample,immune-Tfh; Homo_723,totalSample,immune-Exhausted; Homo_723,totalSample,immune-iTreg; Homo_723,totalSample,immune-Bcell; Homo_714,totalSample,immune-Monocyte; Homo_714,totalSample,immune-Th2; Homo_714,totalSample,immune-Tfh; Homo_714,totalSample,immune-Gamma_delta; Homo_714,totalSample,immune-DC; Homo_633,totalSample,immune-Monocyte; Homo_633,totalSample,immune-Th2; Homo_633,totalSample,immune-Exhausted; Homo_633,totalSample,immune-Gamma_delta; Homo_633,totalSample,immune-Th17; Homo_633,totalSample,immune-Tfh; Homo_633,totalSample,immune-DC; MCSA,totalSample,immune-DC; MCSA,totalSample,immune-Monocyte; MCSA,totalSample,immune-Neutrophil; MCSA,totalSample,immune-CD8_T; MCSA,totalSample,immune-Gamma_delta; MCSA,totalSample,immune-iTreg; MCSA,totalSample,immune-Th1; MCSA,totalSample,immune-Cytotoxic; MCSA,totalSample,immune-Exhausted; MCSA,totalSample,immune-Effector_memory; MCSA,totalSample,immune-InfiltrationScore; ROSMAP,totalSample,immune-Effector_memory; SRP223445,totalSample,immune-CD4_T; SRP223445,totalSample,immune-CD4_naive; SRP223445,totalSample,immune-Th17; SRP223445,totalSample,immune-Exhausted
ANKRD22	Homo_723,female,AD.vs.control; Homo_723,male,AD.vs.control; Homo_723,female,AD.vs.MCI; Homo_723,male,AD.vs.MCI; Homo_714,female,AD.vs.control; Homo_714,male,AD.vs.control; Homo_714,female,AD.vs.MCI; Homo_714,male,AD.vs.MCI; Homo_633,female,AD.vs.control; Homo_633,female,AD.vs.MCI; ACOM,AD,age; MCSA,totalSample,age; MCSA,control,age; ROSMAP,control,sample.age; ROSMAP,MCI,last.visit.age; ROSMAP,MCI,sample.age; ROSMAP,AD,MMSE; Emory,totalSample,immune-B_cell; Emory,totalSample,immune-Monocyte; Emory,totalSample,immune-Macrophage; Emory,totalSample,immune-Neutrophil; Emory,totalSample,immune-CD4_T; Emory,totalSample,immune-CD8_T; Emory,totalSample,immune-Gamma_delta; Emory,totalSample,immune-nTreg; Emory,totalSample,immune-Tfh; Emory,totalSample,immune-CD8_naive; Emory,totalSample,immune-Exhausted; Emory,totalSample,immune-MAIT; Homo_723,totalSample,immune-Monocyte; Homo_723,totalSample,immune-Neutrophil; Homo_723,totalSample,immune-DC; Homo_723,totalSample,immune-Gamma_delta; Homo_723,totalSample,immune-Bcell; Homo_714,totalSample,immune-Monocyte; Homo_714,totalSample,immune-Neutrophil; Homo_633,totalSample,immune-Monocyte; Homo_633,totalSample,immune-Exhausted; Homo_633,totalSample,immune-Macrophage; Homo_633,totalSample,immune-iTreg; Homo_633,totalSample,immune-CD8_T; Homo_633,totalSample,immune-Gamma_delta; Homo_633,totalSample,immune-MAIT; MCSA,totalSample,immune-Monocyte; MCSA,totalSample,immune-Macrophage; MCSA,totalSample,immune-Neutrophil; MCSA,totalSample,immune-CD4_T; MCSA,totalSample,immune-CD8_T; MCSA,totalSample,immune-CD4_naive; MCSA,totalSample,immune-Tr1; MCSA,totalSample,immune-nTreg; MCSA,totalSample,immune-Tfh; MCSA,totalSample,immune-Cytotoxic; MCSA,totalSample,immune-Central_memory; MCSA,totalSample,immune-Effector_memory; ROSMAP,totalSample,immune-CD4_T; ROSMAP,totalSample,immune-nTreg; ROSMAP,totalSample,immune-Th2;
CPQ	Homo_723,female,AD.vs.control; Homo_723,male,AD.vs.control; Homo_723,female,AD.vs.MCI; Homo_723,male,AD.vs.MCI; Homo_714,female,AD.vs.control; Homo_714,male,AD.vs.control; Homo_714,female,AD.vs.MCI; Homo_714,male,AD.vs.MCI; Homo_633,female,AD.vs.control; Homo_633,female,AD.vs.MCI; SRP223445,female,AD.vs.control; Emory,MCI,age; MCSA,MCI,age; Emory,totalSample,immune-Neutrophil; Emory,totalSample,immune-CD8_T; Emory,totalSample,immune-Gamma_delta; Emory,totalSample,immune-Th1; Emory,totalSample,immune-Th17; Emory,totalSample,immune-Tfh; Emory,totalSample,immune-CD8_naive; Emory,totalSample,immune-Cytotoxic; Emory,totalSample,immune-Exhausted; Homo_723,totalSample,immune-Neutrophil; Homo_723,totalSample,immune-Th2; Homo_723,totalSample,immune-Gamma_delta; Homo_723,totalSample,immune-DC; Homo_723,totalSample,immune-Monocyte; Homo_723,totalSample,immune-Bcell; Homo_723,totalSample,immune-InfiltrationScore; Homo_723,totalSample,immune-iTreg; Homo_723,totalSample,immune-Tfh; Homo_723,totalSample,immune-Exhausted; Homo_723,totalSample,immune-Th1; Homo_714,totalSample,immune-Th2; Homo_714,totalSample,immune-Gamma_delta; Homo_714,totalSample,immune-Neutrophil; Homo_714,totalSample,immune-Tfh; Homo_714,totalSample,immune-Monocyte; Homo_714,totalSample,immune-Th1; Homo_633,totalSample,immune-Th2; Homo_633,totalSample,immune-Exhausted; Homo_633,totalSample,immune-Th17; Homo_633,totalSample,immune-Monocyte; Homo_633,totalSample,immune-Gamma_delta; Homo_633,totalSample,immune-InfiltrationScore; Homo_633,totalSample,immune-Neutrophil; Homo_633,totalSample,immune-CD8_T; Homo_633,totalSample,immune-Tfh; MCSA,totalSample,immune-Neutrophil; MCSA,totalSample,immune-CD8_T; MCSA,totalSample,immune-Gamma_delta; MCSA,totalSample,immune-iTreg; MCSA,totalSample,immune-Th1; MCSA,totalSample,immune-Th17; MCSA,totalSample,immune-Cytotoxic; MCSA,totalSample,immune-Exhausted; MCSA,totalSample,immune-Effector_memory;
PTK7	Homo_723,female,AD.vs.control; Homo_723,male,AD.vs.control; Homo_723,female,AD.vs.MCI; Homo_723,male,AD.vs.MCI; Homo_723,female,MCI.vs.control; Homo_723,male,MCI.vs.control; Homo_714,female,AD.vs.control; Homo_714,male,AD.vs.control; Homo_714,female,AD.vs.MCI; Homo_714,male,AD.vs.MCI; Homo_633,female,AD.vs.control; Homo_633,female,AD.vs.MCI; Emory,MCI,age; MCSA,totalSample,age; MCSA,control,age; MCSA,MCI,age; Homo_723,totalSample,immune-DC; Homo_723,totalSample,immune-nTreg; Homo_723,totalSample,immune-Central_memory; Homo_723,totalSample,immune-Th17; Homo_723,totalSample,immune-CD4_naive; Homo_723,totalSample,immune-InfiltrationScore; Homo_714,totalSample,immune-DC; Homo_714,totalSample,immune-InfiltrationScore; Homo_714,totalSample,immune-nTreg; Homo_633,totalSample,immune-DC; Homo_633,totalSample,immune-InfiltrationScore; Homo_633,totalSample,immune-nTreg; Homo_633,totalSample,immune-Tfh; Homo_633,totalSample,immune-Effector_memory; Homo_633,totalSample,immune-Tr1; Homo_633,totalSample,immune-Central_memory; MCSA,totalSample,immune-DC; MCSA,totalSample,immune-Monocyte; MCSA,totalSample,immune-CD4_T; MCSA,totalSample,immune-CD8_T; MCSA,totalSample,immune-NKT; MCSA,totalSample,immune-CD4_naive; MCSA,totalSample,immune-Tr1; MCSA,totalSample,immune-nTreg; MCSA,totalSample,immune-Th1; MCSA,totalSample,immune-Th2; MCSA,totalSample,immune-Th17; MCSA,totalSample,immune-Tfh; MCSA,totalSample,immune-Cytotoxic; MCSA,totalSample,immune-Central_memory; MCSA,totalSample,immune-Effector_memory; MCSA,totalSample,immune-InfiltrationScore; ROSMAP,totalSample,immune-InfiltrationScore;

PIGC	Homo_723,female,AD.vs.control; Homo_723,male,AD.vs.control; Homo_723,female,AD.vs.MCI; Homo_723,male,AD.vs.MCI; Homo_714,female,AD.vs.control; Homo_714,male,AD.vs.control; Homo_714,female,AD.vs.MCI; Homo_714,male,AD.vs.MCI; Homo_633,female,AD.vs.control; Homo_633,female,AD.vs.MCI; ROSMAP,female,AD.vs.control; ROSMAP,male,AD.vs.control; ACOM,totalSample,age; MCSA,MCI,age; ROSMAP,MCI,last.visit.age; Emory,totalSample,immune-Macrophage; Emory,totalSample,immune-NK; Emory,totalSample,immune-Neutrophil; Emory,totalSample,immune-CD4_T; Emory,totalSample,immune-CD8_T; Emory,totalSample,immune-Tr1; Emory,totalSample,immune-iTreg; Emory,totalSample,immune-Tfh; Emory,totalSample,immune-MAIT; Emory,totalSample,immune-Central_memory; Emory,totalSample,immune-InfiltrationScore; Homo_723,totalSample,immune-Th2; Homo_723,totalSample,immune-Tfh; Homo_723,totalSample,immune-iTreg; Homo_723,totalSample,immune-CD4_T; Homo_723,totalSample,immune-Tr1; Homo_723,totalSample,immune-CD4_naive; Homo_723,totalSample,immune-Effector_memory; Homo_723,totalSample,immune-DC; Homo_723,totalSample,immune-InfiltrationScore; Homo_723,totalSample,immune-Central_memory; Homo_723,totalSample,immune-Macrophage; Homo_723,totalSample,immune-nTreg; Homo_723,totalSample,immune-Gamma_delta; Homo_723,totalSample,immune-Bcell; Homo_723,totalSample,immune-Monocyte; Homo_723,totalSample,immune-MAIT; Homo_714,totalSample,immune-Th2; Homo_714,totalSample,immune-Tfh; Homo_714,totalSample,immune-CD4_T; Homo_714,totalSample,immune-Tr1; Homo_714,totalSample,immune-Effector_memory; Homo_714,totalSample,immune-Central_memory; Homo_714,totalSample,immune-iTreg; Homo_714,totalSample,immune-CD4_naive; Homo_714,totalSample,immune-nTreg; Homo_714,totalSample,immune-InfiltrationScore; Homo_714,totalSample,immune-Gamma_delta; Homo_714,totalSample,immune-DC; Homo_714,totalSample,immune-Macrophage; Homo_633,totalSample,immune-Tfh; Homo_633,totalSample,immune-CD4_T; Homo_633,totalSample,immune-Th2; Homo_633,totalSample,immune-CD4_naive; Homo_633,totalSample,immune-Effector_memory; Homo_633,totalSample,immune-Central_memory; Homo_633,totalSample,immune-Tr1; Homo_633,totalSample,immune-InfiltrationScore; Homo_633,totalSample,immune-nTreg; Homo_633,totalSample,immune-iTreg; Homo_633,totalSample,immune-Th17; Homo_633,totalSample,immune-Bcell; Homo_633,totalSample,immune-Monocyte; Homo_633,totalSample,immune-Gamma_delta; Homo_633,totalSample,immune-NKT; MCSA,totalSample,immune-DC; MCSA,totalSample,immune-Macrophage; MCSA,totalSample,immune-CD4_T; MCSA,totalSample,immune-CD4_naive; MCSA,totalSample,immune-Tr1; MCSA,totalSample,immune-nTreg; MCSA,totalSample,immune-iTreg; MCSA,totalSample,immune-Th2; MCSA,totalSample,immune-Th17; MCSA,totalSample,immune-Tfh; MCSA,totalSample,immune-CD8_naive; MCSA,totalSample,immune-Cytotoxic; MCSA,totalSample,immune-MAIT; [illegible]
VGLL4	Homo_723,female,AD.vs.control; Homo_723,male,AD.vs.control; Homo_723,female,AD.vs.MCI; Homo_723,male,AD.vs.MCI; Homo_714,female,AD.vs.control; Homo_714,male,AD.vs.control; Homo_714,female,AD.vs.MCI; Homo_714,male,AD.vs.MCI; Homo_633,female,AD.vs.control; Homo_633,female,AD.vs.MCI; ROSMAP,MCI,last.visit.age; Emory,totalSample,immune-Macrophage; Emory,totalSample,immune-NK; Emory,totalSample,immune-Neutrophil; Emory,totalSample,immune-CD4_T; Emory,totalSample,immune-CD8_T; Emory,totalSample,immune-Tr1; Emory,totalSample,immune-iTreg; Emory,totalSample,immune-Tfh; Emory,totalSample,immune-Cytotoxic; Emory,totalSample,immune-Exhausted; Emory,totalSample,immune-MAIT; Emory,totalSample,immune-Effector_memory; Emory,totalSample,immune-InfiltrationScore; Homo_723,totalSample,immune-CD4_naive; Homo_723,totalSample,immune-Monocyte; Homo_723,totalSample,immune-DC; Homo_723,totalSample,immune-Central_memory; Homo_723,totalSample,immune-Neutrophil; Homo_723,totalSample,immune-InfiltrationScore; Homo_723,totalSample,immune-CD4_T; Homo_723,totalSample,immune-Th1; Homo_723,totalSample,immune-nTreg; Homo_723,totalSample,immune-NK; Homo_723,totalSample,immune-NKT; Homo_723,totalSample,immune-Tfh; Homo_714,totalSample,immune-InfiltrationScore; Homo_714,totalSample,immune-CD4_naive; Homo_714,totalSample,immune-NKT; Homo_714,totalSample,immune-Th1; Homo_714,totalSample,immune-Neutrophil; Homo_714,totalSample,immune-NK; Homo_714,totalSample,immune-Central_memory; Homo_714,totalSample,immune-Monocyte; Homo_633,totalSample,immune-InfiltrationScore; Homo_633,totalSample,immune-CD4_naive; Homo_633,totalSample,immune-Tfh; Homo_633,totalSample,immune-NKT; Homo_633,totalSample,immune-CD4_T; Homo_633,totalSample,immune-iTreg; Homo_633,totalSample,immune-Monocyte; Homo_633,totalSample,immune-Central_memory; Homo_633,totalSample,immune-Effector_memory; Homo_633,totalSample,immune-Tr1; Homo_633,totalSample,immune-nTreg; Homo_633,totalSample,immune-Neutrophil; Homo_633,totalSample,immune-MAIT; Homo_633,totalSample,immune-Macrophage; MCSA,totalSample,immune-DC; MCSA,totalSample,immune-Macrophage; MCSA,totalSample,immune-Neutrophil; MCSA,totalSample,immune-CD4_T; MCSA,totalSample,immune-CD4_naive; MCSA,totalSample,immune-Tr1; MCSA,totalSample,immune-nTreg; MCSA,totalSample,immune-iTreg; MCSA,totalSample,immune-Th17; MCSA,totalSample,immune-Tfh; MCSA,totalSample,immune-CD8_naive; MCSA,totalSample,immune-Exhausted; MCSA,totalSample,immune-MAIT; MCSA,totalSample,immune-Central_memory; MCSA,totalSample,immune-Effector_memory; [illegible]
DCT	Homo_723,female,AD.vs.control; Homo_723,male,AD.vs.control; Homo_723,female,AD.vs.MCI; Homo_723,male,AD.vs.MCI; Homo_723,male,MCI.vs.control; Homo_714,female,AD.vs.control; Homo_714,female,AD.vs.MCI; Homo_714,male,AD.vs.MCI; Homo_714,female,MCI.vs.control; Homo_633,female,AD.vs.control; MCSA,MCI,age; Homo_723,totalSample,immune-Th2; Homo_723,totalSample,immune-CD4_T; Homo_723,totalSample,immune-Tfh; Homo_723,totalSample,immune-CD4_naive; Homo_723,totalSample,immune-Tr1; Homo_723,totalSample,immune-Central_memory; Homo_723,totalSample,immune-iTreg; Homo_723,totalSample,immune-Th17; Homo_723,totalSample,immune-nTreg; Homo_723,totalSample,immune-Exhausted; Homo_714,totalSample,immune-Tfh; Homo_714,totalSample,immune-CD4_T; Homo_714,totalSample,immune-CD4_naive; Homo_714,totalSample,immune-Central_memory; Homo_714,totalSample,immune-Th2; Homo_714,totalSample,immune-Tr1; Homo_714,totalSample,immune-iTreg; Homo_714,totalSample,immune-Gamma_delta; Homo_633,totalSample,immune-Tfh; Homo_633,totalSample,immune-CD4_T; Homo_633,totalSample,immune-CD4_naive; Homo_633,totalSample,immune-Central_memory; Homo_633,totalSample,immune-Th2; Homo_633,totalSample,immune-Effector_memory; Homo_633,totalSample,immune-Tr1; Homo_633,totalSample,immune-iTreg; Homo_633,totalSample,immune-nTreg; Homo_633,totalSample,immune-Gamma_delta; MCSA,totalSample,immune-DC; MCSA,totalSample,immune-Macrophage; MCSA,totalSample,immune-CD4_T; MCSA,totalSample,immune-CD4_naive; MCSA,totalSample,immune-Tr1; MCSA,totalSample,immune-nTreg; MCSA,totalSample,immune-iTreg; MCSA,totalSample,immune-Th17; MCSA,totalSample,immune-Tfh; MCSA,totalSample,immune-CD8_naive; MCSA,totalSample,immune-Cytotoxic; MCSA,totalSample,immune-Exhausted; MCSA,totalSample,immune-MAIT; MCSA,totalSample,immune-Central_memory; ROSMAP,totalSample,immune-InfiltrationScore
ATP1B1	Homo_723,female,AD.vs.control; Homo_723,male,AD.vs.control; Homo_723,female,AD.vs.MCI; Homo_723,male,AD.vs.MCI; Homo_723,female,MCI.vs.control; Homo_714,female,AD.vs.control; Homo_714,female,AD.vs.MCI; Homo_633,female,AD.vs.control; Homo_633,female,AD.vs.MCI; ROSMAP,female,AD.vs.MCI; ROSMAP,female,MCI.vs.control; MCSA,MCI,age; Emory,totalSample,immune-NK; Emory,totalSample,immune-Neutrophil; Emory,totalSample,immune-InfiltrationScore; Homo_723,totalSample,immune-Th2; Homo_723,totalSample,immune-CD4_T; Homo_723,totalSample,immune-CD4_naive; Homo_723,totalSample,immune-InfiltrationScore; Homo_723,totalSample,immune-Exhausted; Homo_723,totalSample,immune-Tfh; Homo_723,totalSample,immune-Tr1; Homo_723,totalSample,immune-Th1; Homo_723,totalSample,immune-NKT; Homo_723,totalSample,immune-Th17; Homo_714,totalSample,immune-InfiltrationScore; Homo_714,totalSample,immune-CD4_naive; Homo_714,totalSample,immune-CD4_T; Homo_714,totalSample,immune-Tfh; Homo_714,totalSample,immune-NKT; Homo_714,totalSample,immune-Th2; Homo_633,totalSample,immune-Tfh; Homo_633,totalSample,immune-InfiltrationScore; Homo_633,totalSample,immune-CD4_T; Homo_633,totalSample,immune-CD4_naive; Homo_633,totalSample,immune-Effector_memory; Homo_633,totalSample,immune-Th2; Homo_633,totalSample,immune-NKT; Homo_633,totalSample,immune-Central_memory; Homo_633,totalSample,immune-Tr1; Homo_633,totalSample,immune-Monocyte; MCSA,totalSample,immune-DC; MCSA,totalSample,immune-Macrophage; MCSA,totalSample,immune-CD4_T; MCSA,totalSample,immune-CD4_naive; MCSA,totalSample,immune-Tr1; MCSA,totalSample,immune-nTreg; MCSA,totalSample,immune-iTreg; MCSA,totalSample,immune-Th2; MCSA,totalSample,immune-Tfh; MCSA,totalSample,immune-CD8_naive; MCSA,totalSample,immune-Cytotoxic; MCSA,totalSample,immune-Exhausted; MCSA,totalSample,immune-MAIT; MCSA,totalSample,immune-Central_memory
DNAH12	Homo_723,female,AD.vs.control; Homo_723,male,AD.vs.control; Homo_723,female,AD.vs.MCI; Homo_723,male,AD.vs.MCI; Homo_723,male,MCI.vs.control; Homo_714,female,AD.vs.control; Homo_714,male,AD.vs.control; Homo_714,female,AD.vs.MCI; Homo_714,male,AD.vs.MCI; Homo_714,male,MCI.vs.control; Homo_633,female,AD.vs.control; Homo_633,female,AD.vs.MCI; ROSMAP,female,AD.vs.control; ROSMAP,female,AD.vs.MCI; Homo_723,totalSample,immune-Th17; Homo_723,totalSample,immune-Th1; Homo_723,totalSample,immune-Cytotoxic; Homo_723,totalSample,immune-CD4_naive; Homo_723,totalSample,immune-CD4_T; Homo_723,totalSample,immune-nTreg; Homo_723,totalSample,immune-Central_memory; Homo_714,totalSample,immune-Th1; Homo_714,totalSample,immune-CD4_naive; Homo_714,totalSample,immune-Th17; Homo_714,totalSample,immune-Exhausted; Homo_633,totalSample,immune-Tfh; Homo_633,totalSample,immune-CD4_T; Homo_633,totalSample,immune-Tr1; Homo_633,totalSample,immune-CD4_naive; Homo_633,totalSample,immune-InfiltrationScore; Homo_633,totalSample,immune-iTreg; MCSA,totalSample,immune-DC; MCSA,totalSample,immune-CD4_T; MCSA,totalSample,immune-CD4_naive; MCSA,totalSample,immune-Tr1; MCSA,totalSample,immune-nTreg; MCSA,totalSample,immune-iTreg; MCSA,totalSample,immune-Th17; MCSA,totalSample,immune-Tfh; MCSA,totalSample,immune-CD8_naive; MCSA,totalSample,immune-Cytotoxic; MCSA,totalSample,immune-Central_memory; ROSMAP,totalSample,immune-InfiltrationScore; SRP223445,totalSample,immune-CD4_naive; SRP223445,totalSample,immune-InfiltrationScore; ROSMAP,female-excludeAge90+,survival
KIFAP3	Homo_723,female,AD.vs.control; Homo_723,male,AD.vs.control; Homo_723,female,AD.vs.MCI; Homo_723,male,AD.vs.MCI; Homo_714,female,AD.vs.control; Homo_714,male,AD.vs.control; Homo_714,female,AD.vs.MCI; Homo_714,male,AD.vs.MCI; Homo_633,female,AD.vs.control; Homo_633,female,AD.vs.MCI; MCSA,MCI,age; ROSMAP,AD,sample.age; Emory,totalSample,immune-Macrophage; Emory,totalSample,immune-NK; Emory,totalSample,immune-Neutrophil; Emory,totalSample,immune-CD4_T; Emory,totalSample,immune-CD8_T; Emory,totalSample,immune-Tr1; Emory,totalSample,immune-iTreg; Emory,totalSample,immune-Tfh; Emory,totalSample,immune-Exhausted; Emory,totalSample,immune-MAIT; Emory,totalSample,immune-Central_memory; Emory,totalSample,immune-Effector_memory; Emory,totalSample,immune-InfiltrationScore; Homo_723,totalSample,immune-CD4_naive; Homo_723,totalSample,immune-CD4_T; Homo_723,totalSample,immune-InfiltrationScore; Homo_723,totalSample,immune-Tfh; Homo_723,totalSample,immune-Th1; Homo_723,totalSample,immune-Neutrophil; Homo_723,totalSample,immune-Central_memory; Homo_723,totalSample,immune-Tr1; Homo_723,totalSample,immune-NK; Homo_723,totalSample,immune-Th17; Homo_723,totalSample,immune-Exhausted; Homo_723,totalSample,immune-Cytotoxic; Homo_723,totalSample,immune-nTreg; Homo_723,totalSample,immune-Th2; Homo_723,totalSample,immune-iTreg; Homo_723,totalSample,immune-NKT; Homo_723,totalSample,immune-Effector_memory; Homo_714,totalSample,immune-CD4_naive; Homo_714,totalSample,immune-InfiltrationScore; Homo_714,totalSample,immune-NK; Homo_714,totalSample,immune-Tfh; Homo_714,totalSample,immune-CD4_T; Homo_714,totalSample,immune-Central_memory; Homo_714,totalSample,immune-Neutrophil; Homo_714,totalSample,immune-Th1; Homo_714,totalSample,immune-NKT; Homo_714,totalSample,immune-Bcell; Homo_714,totalSample,immune-Effector_memory; Homo_714,totalSample,immune-Tr1; Homo_633,totalSample,immune-Tfh; Homo_633,totalSample,immune-InfiltrationScore; Homo_633,totalSample,immune-CD4_naive; Homo_633,totalSample,immune-CD4_T; Homo_633,totalSample,immune-Effector_memory; Homo_633,totalSample,immune-Central_memory; Homo_633,totalSample,immune-Tr1; Homo_633,totalSample,immune-Bcell; Homo_633,totalSample,immune-NKT; Homo_633,totalSample,immune-Neutrophil; Homo_633,totalSample,immune-iTreg; Homo_633,totalSample,immune-Th2; Homo_633,totalSample,immune-NK; Homo_633,totalSample,immune-MAIT; Homo_633,totalSample,immune-Macrophage; MCSA,totalSample,immune-DC; MCSA,totalSample,immune-Monocyte; MCSA,totalSample,immune-Macrophage; MCSA,totalSample,immune-CD4_T; MCSA,totalSample,immune-CD4_naive; MCSA,totalSample,immune-Tr1; MCSA,totalSample,immune-nTreg; MCSA,totalSample,immune-iTreg; MCSA,totalSample,immune-Th2; MCSA,totalSample,immune-Tfh; MCSA,totalSample,immune-CD8_naive; MCSA,totalSample,immune-Cytotoxic; MCSA,totalSample,immune-Exhausted; MCSA,totalSample,immune-MAIT; [illegible]
MICB	Homo_723,female,AD.vs.control; Homo_723,male,AD.vs.control; Homo_723,female,AD.vs.MCI; Homo_723,male,AD.vs.MCI; Homo_714,female,AD.vs.control; Homo_714,male,AD.vs.control; Homo_714,female,AD.vs.MCI; Homo_714,male,AD.vs.MCI; Homo_633,female,AD.vs.control; Homo_633,female,AD.vs.MCI; ROSMAP,female,AD.vs.control; Emory,MCI,age; MCSA,MCI,age; Emory,totalSample,immune-CD8_naive; Emory,totalSample,immune-Effector_memory; Homo_723,totalSample,immune-InfiltrationScore; Homo_723,totalSample,immune-Th2; Homo_723,totalSample,immune-Tfh; Homo_723,totalSample,immune-Monocyte; Homo_723,totalSample,immune-Effector_memory; Homo_723,totalSample,immune-iTreg; Homo_723,totalSample,immune-Gamma_delta; Homo_723,totalSample,immune-Bcell; Homo_723,totalSample,immune-DC; Homo_723,totalSample,immune-Exhausted; Homo_723,totalSample,immune-CD4_T; Homo_723,totalSample,immune-Tr1; Homo_714,totalSample,immune-Th2; Homo_714,totalSample,immune-Tfh; Homo_714,totalSample,immune-Effector_memory; Homo_714,totalSample,immune-InfiltrationScore; Homo_714,totalSample,immune-Monocyte; Homo_714,totalSample,immune-CD4_T; Homo_714,totalSample,immune-Gamma_delta; Homo_714,totalSample,immune-Central_memory; Homo_633,totalSample,immune-Th2; Homo_633,totalSample,immune-InfiltrationScore; Homo_633,totalSample,immune-Exhausted; Homo_633,totalSample,immune-Monocyte; Homo_633,totalSample,immune-Tfh; Homo_633,totalSample,immune-Effector_memory; Homo_633,totalSample,immune-Gamma_delta; Homo_633,totalSample,immune-CD4_T; Homo_633,totalSample,immune-Th17; Homo_633,totalSample,immune-Central_memory; Homo_633,totalSample,immune-CD4_naive; MCSA,totalSample,immune-CD4_T; MCSA,totalSample,immune-Gamma_delta; MCSA,totalSample,immune-CD4_naive; MCSA,totalSample,immune-Tr1; MCSA,totalSample,immune-nTreg; MCSA,totalSample,immune-iTreg; MCSA,totalSample,immune-Th17; MCSA,totalSample,immune-Tfh; MCSA,totalSample,immune-Cytotoxic; MCSA,totalSample,immune-Exhausted;
FCRL1	Homo_723,female,AD.vs.control; Homo_723,male,AD.vs.control; Homo_723,female,AD.vs.MCI; Homo_723,male,AD.vs.MCI; Homo_723,male,MCI.vs.control; ROSMAP,control,male.vs.female; MCSA,totalSample,age; MCSA,control,age; Emory,totalSample,immune-B_cell; Emory,totalSample,immune-Macrophage; Emory,totalSample,immune-Neutrophil; Emory,totalSample,immune-CD4_T; Emory,totalSample,immune-Tr1; Emory,totalSample,immune-iTreg; Emory,totalSample,immune-Tfh; Emory,totalSample,immune-CD8_naive; Emory,totalSample,immune-InfiltrationScore; Homo_723,totalSample,immune-iTreg; Homo_723,totalSample,immune-Th2; Homo_723,totalSample,immune-Tfh; Homo_723,totalSample,immune-Tr1; Homo_723,totalSample,immune-CD4_T; Homo_723,totalSample,immune-DC; Homo_723,totalSample,immune-CD4_naive; Homo_723,totalSample,immune-Gamma_delta; Homo_723,totalSample,immune-Effector_memory; Homo_723,totalSample,immune-nTreg; Homo_723,totalSample,immune-Central_memory; Homo_723,totalSample,immune-Macrophage; Homo_723,totalSample,immune-InfiltrationScore; Homo_714,totalSample,immune-iTreg; Homo_714,totalSample,immune-CD4_T; Homo_714,totalSample,immune-Tfh; Homo_714,totalSample,immune-CD4_naive; Homo_714,totalSample,immune-Tr1; Homo_714,totalSample,immune-nTreg; Homo_714,totalSample,immune-Th1; Homo_714,totalSample,immune-NKT; Homo_714,totalSample,immune-Central_memory; Homo_714,totalSample,immune-Th2; Homo_714,totalSample,immune-Effector_memory; Homo_714,totalSample,immune-Cytotoxic; Homo_714,totalSample,immune-Gamma_delta; Homo_714,totalSample,immune-Bcell; Homo_633,totalSample,immune-CD4_T; Homo_633,totalSample,immune-Tfh; Homo_633,totalSample,immune-Tr1; Homo_633,totalSample,immune-CD4_naive; Homo_633,totalSample,immune-iTreg; Homo_633,totalSample,immune-NKT; Homo_633,totalSample,immune-Central_memory; Homo_633,totalSample,immune-nTreg; Homo_633,totalSample,immune-Th1; Homo_633,totalSample,immune-Effector_memory; Homo_633,totalSample,immune-Gamma_delta; Homo_633,totalSample,immune-Cytotoxic; Homo_633,totalSample,immune-Th2; Homo_633,totalSample,immune-Exhausted; Homo_633,totalSample,immune-Th17; MCSA,totalSample,immune-DC; MCSA,totalSample,immune-Bcell; MCSA,totalSample,immune-Macrophage; MCSA,totalSample,immune-CD4_T; MCSA,totalSample,immune-Gamma_delta; MCSA,totalSample,immune-CD4_naive; MCSA,totalSample,immune-Tr1; MCSA,totalSample,immune-nTreg; MCSA,totalSample,immune-iTreg; MCSA,totalSample,immune-Th17; MCSA,totalSample,immune-Tfh; MCSA,totalSample,immune-CD8_naive; MCSA,totalSample,immune-Cytotoxic; MCSA,totalSample,immune-Exhausted; MCSA,totalSample,immune- [illegible]
MRPL16	Homo_723,female,AD.vs.control; Homo_723,male,AD.vs.control; Homo_723,female,AD.vs.MCI; Homo_723,male,AD.vs.MCI; Homo_714,female,AD.vs.control; Homo_714,male,AD.vs.control; Homo_714,female,AD.vs.MCI; Homo_714,male,AD.vs.MCI; Homo_633,female,AD.vs.control; Homo_633,female,AD.vs.MCI; MCSA,totalSample,age; MCSA,control,age; Emory,totalSample,immune-Macrophage; Emory,totalSample,immune-NK; Emory,totalSample,immune-Neutrophil; Emory,totalSample,immune-CD4_T; Emory,totalSample,immune-CD8_T; Emory,totalSample,immune-Tr1; Emory,totalSample,immune-iTreg; Emory,totalSample,immune-Tfh; Emory,totalSample,immune-Exhausted; Emory,totalSample,immune-MAIT; Emory,totalSample,immune-Central_memory; Emory,totalSample,immune-Effector_memory; Emory,totalSample,immune-InfiltrationScore; Homo_723,totalSample,immune-Tfh; Homo_723,totalSample,immune-InfiltrationScore; Homo_723,totalSample,immune-iTreg; Homo_723,totalSample,immune-Effector_memory; Homo_723,totalSample,immune-Th2; Homo_723,totalSample,immune-CD4_T; Homo_723,totalSample,immune-CD4_naive; Homo_723,totalSample,immune-Tr1; Homo_723,totalSample,immune-nTreg; Homo_723,totalSample,immune-Central_memory; Homo_723,totalSample,immune-Bcell; Homo_723,totalSample,immune-Macrophage; Homo_723,totalSample,immune-CD8_T; Homo_723,totalSample,immune-MAIT; Homo_714,totalSample,immune-Tfh; Homo_714,totalSample,immune-Effector_memory; Homo_714,totalSample,immune-Th2; Homo_714,totalSample,immune-InfiltrationScore; Homo_714,totalSample,immune-CD4_T; Homo_714,totalSample,immune-Tr1; Homo_714,totalSample,immune-CD4_naive; Homo_714,totalSample,immune-Central_memory; Homo_714,totalSample,immune-nTreg; Homo_714,totalSample,immune-iTreg; Homo_714,totalSample,immune-MAIT; Homo_633,totalSample,immune-InfiltrationScore; Homo_633,totalSample,immune-Tfh; Homo_633,totalSample,immune-Effector_memory; Homo_633,totalSample,immune-CD4_T; Homo_633,totalSample,immune-CD4_naive; Homo_633,totalSample,immune-Central_memory; Homo_633,totalSample,immune-Tr1; Homo_633,totalSample,immune-nTreg; Homo_633,totalSample,immune-Th2; Homo_633,totalSample,immune-Neutrophil; Homo_633,totalSample,immune-Bcell; Homo_633,totalSample,immune-iTreg; MCSA,totalSample,immune-Monocyte; MCSA,totalSample,immune-NK; MCSA,totalSample,immune-Neutrophil; MCSA,totalSample,immune-CD4_T; MCSA,totalSample,immune-CD4_naive; MCSA,totalSample,immune-Tr1; MCSA,totalSample,immune-nTreg; MCSA,totalSample,immune-iTreg; MCSA,totalSample,immune-Th17; MCSA,totalSample,immune-Tfh; MCSA,totalSample,immune-Central_memory; MCSA,totalSample,immune-Effector_memory; MCSA,totalSample,immune-InfiltrationScore; ROSMAP,totalSample,immune-Cytotoxic; ROSMAP,totalSample,immune-Effector_memory; SRP223445,totalSample,immune-CD4_naive; SRP223445,totalSample,immune-Th17; SRP223445,totalSample,immune-Exhausted; SRP223445,totalSample,immune-InfiltrationScore
GNAI1	Homo_723,female,AD.vs.control; Homo_723,male,AD.vs.control; Homo_723,female,AD.vs.MCI; Homo_723,male,AD.vs.MCI; Homo_723,female,MCI.vs.control; Homo_723,male,MCI.vs.control; Homo_714,female,AD.vs.control; Homo_714,male,AD.vs.control; Homo_714,female,AD.vs.MCI; Homo_714,male,AD.vs.MCI; Homo_714,female,MCI.vs.control; Homo_633,female,AD.vs.control; Homo_633,female,AD.vs.MCI; MCSA,MCI,age; ROSMAP,MCI,last.visit.age; ROSMAP,MCI,sample.age; Emory,totalSample,immune-Gamma_delta; Emory,totalSample,immune-Tr1; Emory,totalSample,immune-Th17; Emory,totalSample,immune-Cytotoxic; Homo_723,totalSample,immune-Cytotoxic; Homo_723,totalSample,immune-CD4_naive; Homo_723,totalSample,immune-Th1; Homo_723,totalSample,immune-Th17; Homo_723,totalSample,immune-Central_memory; Homo_723,totalSample,immune-CD4_T; Homo_723,totalSample,immune-Exhausted; Homo_723,totalSample,immune-Tr1; Homo_723,totalSample,immune-NKT; Homo_723,totalSample,immune-CD8_T; Homo_714,totalSample,immune-CD4_naive; Homo_714,totalSample,immune-NKT; Homo_714,totalSample,immune-Exhausted; Homo_714,totalSample,immune-Cytotoxic; Homo_714,totalSample,immune-Th1; Homo_714,totalSample,immune-Th17; Homo_714,totalSample,immune-CD8_T; Homo_633,totalSample,immune-Tfh; Homo_633,totalSample,immune-CD4_naive; Homo_633,totalSample,immune-NKT; Homo_633,totalSample,immune-CD4_T; Homo_633,totalSample,immune-Tr1; Homo_633,totalSample,immune-Central_memory; Homo_633,totalSample,immune-Gamma_delta; Homo_633,totalSample,immune-iTreg; Homo_633,totalSample,immune-Effector_memory; MCSA,totalSample,immune-DC; MCSA,totalSample,immune-Monocyte; MCSA,totalSample,immune-CD4_T; MCSA,totalSample,immune-CD4_naive; MCSA,totalSample,immune-Tr1; MCSA,totalSample,immune-nTreg; MCSA,totalSample,immune-iTreg; MCSA,totalSample,immune-Tfh; MCSA,totalSample,immune-CD8_naive; MCSA,totalSample,immune-Cytotoxic; MCSA,totalSample,immune-Exhausted;
CRTAM	Homo_723,female,AD.vs.control; Homo_723,male,AD.vs.control; Homo_723,female,AD.vs.MCI; Homo_723,male,AD.vs.MCI; Homo_714,female,AD.vs.control; Homo_714,male,AD.vs.control; Homo_714,female,AD.vs.MCI; Homo_714,male,AD.vs.MCI; Homo_633,female,AD.vs.control; Homo_633,female,AD.vs.MCI; ROSMAP,female,AD.vs.control; ROSMAP,female,MCI.vs.control; Emory,totalSample,age; Emory,control,age; Emory,MCI,age; ROSMAP,AD,last.visit.age; ROSMAP,AD,sample.age; Emory,totalSample,immune-Macrophage; Emory,totalSample,immune-NK; Emory,totalSample,immune-Neutrophil; Emory,totalSample,immune-CD4_T; Emory,totalSample,immune-CD8_T; Emory,totalSample,immune-Gamma_delta; Emory,totalSample,immune-Tr1; Emory,totalSample,immune-iTreg; Emory,totalSample,immune-Tfh; Emory,totalSample,immune-CD8_naive; Emory,totalSample,immune-Cytotoxic; Emory,totalSample,immune-Exhausted; Emory,totalSample,immune-MAIT; Emory,totalSample,immune-Effector_memory; Emory,totalSample,immune-InfiltrationScore; Homo_723,totalSample,immune-Tfh; Homo_723,totalSample,immune-Effector_memory; Homo_723,totalSample,immune-MAIT; Homo_723,totalSample,immune-InfiltrationScore; Homo_723,totalSample,immune-iTreg; Homo_723,totalSample,immune-Th2; Homo_723,totalSample,immune-DC; Homo_723,totalSample,immune-CD8_T; Homo_723,totalSample,immune-Macrophage; Homo_723,totalSample,immune-CD4_naive; Homo_723,totalSample,immune-CD4_T; Homo_723,totalSample,immune-Cytotoxic; Homo_723,totalSample,immune-Monocyte; Homo_723,totalSample,immune-Bcell; Homo_723,totalSample,immune-Tr1; Homo_714,totalSample,immune-Tfh; Homo_714,totalSample,immune-Effector_memory; Homo_714,totalSample,immune-InfiltrationScore; Homo_714,totalSample,immune-MAIT; Homo_714,totalSample,immune-CD8_T; Homo_714,totalSample,immune-Th2; Homo_714,totalSample,immune-Cytotoxic; Homo_714,totalSample,immune-Neutrophil; Homo_714,totalSample,immune-CD4_T; Homo_714,totalSample,immune-Bcell; Homo_714,totalSample,immune-Macrophage; Homo_714,totalSample,immune-CD4_naive; Homo_714,totalSample,immune-Central_memory; Homo_714,totalSample,immune-Tr1; Homo_714,totalSample,immune-Exhausted; Homo_633,totalSample,immune-InfiltrationScore; Homo_633,totalSample,immune-Effector_memory; Homo_633,totalSample,immune-Tfh; Homo_633,totalSample,immune-Neutrophil; Homo_633,totalSample,immune-MAIT; Homo_633,totalSample,immune-CD8_T; Homo_633,totalSample,immune-CD4_naive; Homo_633,totalSample,immune-Bcell; Homo_633,totalSample,immune-Monocyte; Homo_633,totalSample,immune-Cytotoxic; Homo_633,totalSample,immune-Central_memory; Homo_633,totalSample,immune-CD4_T; MCSA,totalSample,immune-DC; MCSA,totalSample,immune-Monocyte; MCSA,totalSample,immune-Macrophage; MCSA,totalSample,immune-Neutrophil; MCSA,totalSample,immune-CD4_T; MCSA,totalSample,immune-CD8_T; MCSA,totalSample,immune-Gamma_delta; MCSA,totalSample,immune-CD4_naive; MCSA,totalSample,immune-Tr1; MCSA,totalSample,immune-nTreg; MCSA,totalSample,immune-iTreg; MCSA,totalSample,immune-Tfh; MCSA,totalSample,immune-CD8_naive; MCSA,totalSample,immune-MAIT; MCSA,totalSample,immune-Central_memory; MCSA,totalSample,immune-Effector_memory; ROSMAP,totalSample,immune-NK; ROSMAP,totalSample,immune-Th17; ROSMAP,totalSample,immune-InfiltrationScore; [illegible]

FLT3	Homo_723,female,AD.vs.control; Homo_723,male,AD.vs.control; Homo_723,female,AD.vs.MCI; Homo_723,male,AD.vs.MCI; Homo_714,female,AD.vs.control; Homo_714,male,AD.vs.control; Homo_714,female,AD.vs.MCI; Homo_714,male,AD.vs.MCI; Homo_633,female,AD.vs.control; Homo_633,female,AD.vs.MCI; Homo_633,female,MCI.vs.control; Emory,MCI,age; MCSA,MCI,age; ROSMAP,control,sample.age; Emory,totalSample,immune-Th1; Emory,totalSample,immune-Tfh; Emory,totalSample,immune-CD8_naive; Emory,totalSample,immune-InfiltrationScore; Homo_723,totalSample,immune-Monocyte; Homo_723,totalSample,immune-Th2; Homo_723,totalSample,immune-InfiltrationScore; Homo_723,totalSample,immune-Bcell; Homo_723,totalSample,immune-Effector_memory; Homo_723,totalSample,immune-Tfh; Homo_723,totalSample,immune-DC; Homo_723,totalSample,immune-iTreg; Homo_723,totalSample,immune-Gamma_delta; Homo_714,totalSample,immune-Th2; Homo_714,totalSample,immune-Monocyte; Homo_714,totalSample,immune-Tfh; Homo_714,totalSample,immune-Effector_memory; Homo_714,totalSample,immune-Gamma_delta; Homo_633,totalSample,immune-Th2; Homo_633,totalSample,immune-Monocyte; Homo_633,totalSample,immune-Exhausted; Homo_633,totalSample,immune-Tfh; Homo_633,totalSample,immune-InfiltrationScore; Homo_633,totalSample,immune-Effector_memory; Homo_633,totalSample,immune-Th17; Homo_633,totalSample,immune-Gamma_delta; Homo_633,totalSample,immune-Bcell; MCSA,totalSample,immune-CD4_T; MCSA,totalSample,immune-CD8_T; MCSA,totalSample,immune-Tr1; MCSA,totalSample,immune-iTreg; MCSA,totalSample,immune-Cytotoxic; MCSA,totalSample,immune-Exhausted; ROSMAP,totalSample,immune-Effector_memory;
DAG1	Homo_723,female,AD.vs.control; Homo_723,male,AD.vs.control; Homo_723,female,AD.vs.MCI; Homo_723,male,AD.vs.MCI; Homo_714,female,AD.vs.control; Homo_714,male,AD.vs.control; Homo_714,female,AD.vs.MCI; Homo_714,male,AD.vs.MCI; Homo_633,female,AD.vs.control; Homo_633,female,AD.vs.MCI; MCSA,totalSample,age; MCSA,control,age; MCSA,MCI,age; ROSMAP,MCI,last.visit.age; Emory,totalSample,immune-CD4_T; Emory,totalSample,immune-Tr1; Emory,totalSample,immune-iTreg; Emory,totalSample,immune-InfiltrationScore; Homo_723,totalSample,immune-DC; Homo_723,totalSample,immune-nTreg; Homo_723,totalSample,immune-CD4_naive; Homo_723,totalSample,immune-InfiltrationScore; Homo_723,totalSample,immune-Th17; Homo_723,totalSample,immune-Central_memory; Homo_723,totalSample,immune-CD4_T; Homo_723,totalSample,immune-Monocyte; Homo_723,totalSample,immune-Neutrophil; Homo_714,totalSample,immune-InfiltrationScore; Homo_714,totalSample,immune-DC; Homo_714,totalSample,immune-nTreg; Homo_714,totalSample,immune-CD4_naive; Homo_633,totalSample,immune-InfiltrationScore; Homo_633,totalSample,immune-nTreg; Homo_633,totalSample,immune-Effector_memory; Homo_633,totalSample,immune-CD4_T; Homo_633,totalSample,immune-Tfh; Homo_633,totalSample,immune-CD4_naive; Homo_633,totalSample,immune-DC; Homo_633,totalSample,immune-Tr1; Homo_633,totalSample,immune-Monocyte; Homo_633,totalSample,immune-Central_memory; MCSA,totalSample,immune-DC; MCSA,totalSample,immune-Monocyte; MCSA,totalSample,immune-Neutrophil; MCSA,totalSample,immune-CD4_T; MCSA,totalSample,immune-CD8_T; MCSA,totalSample,immune-CD4_naive; MCSA,totalSample,immune-Tr1; MCSA,totalSample,immune-nTreg; MCSA,totalSample,immune-Th2; MCSA,totalSample,immune-Th17; MCSA,totalSample,immune-Tfh; MCSA,totalSample,immune-Cytotoxic; MCSA,totalSample,immune-Central_memory; MCSA,totalSample,immune-Effector_memory; MCSA,totalSample,immune-InfiltrationScore;
DUSP19	Homo_723,female,AD.vs.control; Homo_723,male,AD.vs.control; Homo_723,female,AD.vs.MCI; Homo_723,male,AD.vs.MCI; Homo_723,female,MCI.vs.control; Homo_723,male,MCI.vs.control; Homo_714,female,AD.vs.control; Homo_714,male,AD.vs.control; Homo_714,female,AD.vs.MCI; Homo_714,male,AD.vs.MCI; Homo_714,female,MCI.vs.control; Homo_714,male,MCI.vs.control; Homo_633,female,AD.vs.control; Homo_633,female,AD.vs.MCI; MCSA,MCI,age; ROSMAP,MCI,last.visit.age; ROSMAP,MCI,sample.age; Emory,totalSample,immune-Tr1; Emory,totalSample,immune-iTreg; Emory,totalSample,immune-InfiltrationScore; Homo_723,totalSample,immune-Th17; Homo_723,totalSample,immune-Exhausted; Homo_723,totalSample,immune-CD4_naive; Homo_723,totalSample,immune-Cytotoxic; Homo_723,totalSample,immune-CD4_T; Homo_723,totalSample,immune-Tfh; Homo_723,totalSample,immune-CD8_T; Homo_723,totalSample,immune-Th1; Homo_723,totalSample,immune-Tr1; Homo_723,totalSample,immune-Th2; Homo_723,totalSample,immune-InfiltrationScore; Homo_723,totalSample,immune-Central_memory; Homo_714,totalSample,immune-CD4_naive; Homo_714,totalSample,immune-Th17; Homo_714,totalSample,immune-CD4_T; Homo_714,totalSample,immune-InfiltrationScore; Homo_714,totalSample,immune-Tfh; Homo_714,totalSample,immune-Monocyte; Homo_714,totalSample,immune-NKT; Homo_714,totalSample,immune-Central_memory; Homo_633,totalSample,immune-Tfh; Homo_633,totalSample,immune-Th2; Homo_633,totalSample,immune-CD4_T; Homo_633,totalSample,immune-CD4_naive; Homo_633,totalSample,immune-InfiltrationScore; Homo_633,totalSample,immune-Effector_memory; Homo_633,totalSample,immune-Central_memory; Homo_633,totalSample,immune-Monocyte; Homo_633,totalSample,immune-Th17; Homo_633,totalSample,immune-Tr1; Homo_633,totalSample,immune-NKT; Homo_633,totalSample,immune-DC; Homo_633,totalSample,immune-iTreg; Homo_633,totalSample,immune-Gamma_delta; MCSA,totalSample,immune-DC; MCSA,totalSample,immune-Macrophage; MCSA,totalSample,immune-CD4_T; MCSA,totalSample,immune-CD4_naive; MCSA,totalSample,immune-Tr1; MCSA,totalSample,immune-nTreg; MCSA,totalSample,immune-iTreg; MCSA,totalSample,immune-Th17; MCSA,totalSample,immune-Tfh; MCSA,totalSample,immune-CD8_naive; MCSA,totalSample,immune-Cytotoxic; MCSA,totalSample,immune-Exhausted; [illegible]
FER	Homo_723,female,AD.vs.control; Homo_723,male,AD.vs.control; Homo_723,female,AD.vs.MCI; Homo_723,male,AD.vs.MCI; Homo_723,female,MCI.vs.control; Homo_723,male,MCI.vs.control; Homo_714,female,AD.vs.control; Homo_714,male,AD.vs.control; Homo_714,female,AD.vs.MCI; Homo_714,male,AD.vs.MCI; Homo_714,female,MCI.vs.control; Homo_714,male,MCI.vs.control; Homo_633,female,AD.vs.control; Homo_633,female,AD.vs.MCI; ROSMAP,female,AD.vs.control; MCSA,MCI,age; Emory,totalSample,immune-Macrophage; Emory,totalSample,immune-Neutrophil; Emory,totalSample,immune-CD4_T; Emory,totalSample,immune-Tr1; Emory,totalSample,immune-iTreg; Emory,totalSample,immune-InfiltrationScore; Homo_723,totalSample,immune-CD4_naive; Homo_723,totalSample,immune-CD4_T; Homo_723,totalSample,immune-Cytotoxic; Homo_723,totalSample,immune-Central_memory; Homo_723,totalSample,immune-Th17; Homo_723,totalSample,immune-Th1; Homo_723,totalSample,immune-Tr1; Homo_723,totalSample,immune-Tfh; Homo_723,totalSample,immune-Exhausted; Homo_723,totalSample,immune-nTreg; Homo_723,totalSample,immune-NKT; Homo_723,totalSample,immune-InfiltrationScore; Homo_723,totalSample,immune-iTreg; Homo_723,totalSample,immune-CD8_T; Homo_723,totalSample,immune-Gamma_delta; Homo_723,totalSample,immune-Th2; Homo_723,totalSample,immune-Neutrophil; Homo_714,totalSample,immune-CD4_naive; Homo_714,totalSample,immune-NKT; Homo_714,totalSample,immune-CD4_T; Homo_714,totalSample,immune-Central_memory; Homo_714,totalSample,immune-Tfh; Homo_714,totalSample,immune-Th1; Homo_714,totalSample,immune-Th17; Homo_714,totalSample,immune-InfiltrationScore; Homo_714,totalSample,immune-iTreg; Homo_714,totalSample,immune-Exhausted; Homo_714,totalSample,immune-Cytotoxic; Homo_714,totalSample,immune-Tr1; Homo_714,totalSample,immune-Gamma_delta; Homo_633,totalSample,immune-Tfh; Homo_633,totalSample,immune-CD4_naive; Homo_633,totalSample,immune-CD4_T; Homo_633,totalSample,immune-NKT; Homo_633,totalSample,immune-Central_memory; Homo_633,totalSample,immune-Effector_memory; Homo_633,totalSample,immune-Tr1; Homo_633,totalSample,immune-InfiltrationScore; Homo_633,totalSample,immune-iTreg; Homo_633,totalSample,immune-Gamma_delta; Homo_633,totalSample,immune-Th2; Homo_633,totalSample,immune-nTreg; Homo_633,totalSample,immune-Th1; Homo_633,totalSample,immune-Th17; MCSA,totalSample,immune-DC; MCSA,totalSample,immune-Macrophage; MCSA,totalSample,immune-CD4_T; MCSA,totalSample,immune-CD8_T; MCSA,totalSample,immune-CD4_naive; MCSA,totalSample,immune-Tr1; MCSA,totalSample,immune-nTreg; MCSA,totalSample,immune-iTreg; MCSA,totalSample,immune-Th17; MCSA,totalSample,immune-Tfh; MCSA,totalSample,immune-CD8_naive; MCSA,totalSample,immune-Cytotoxic; MCSA,totalSample,immune-Exhausted; MCSA,totalSample,immune-MAIT; MCSA,totalSample,immune-Central_memory; ROSMAP,totalSample,immune-Th17; ROSMAP,totalSample,immune-Effector_memory; [illegible]
IDH3B	Homo_723,female,AD.vs.control; Homo_723,male,AD.vs.control; Homo_723,female,AD.vs.MCI; Homo_723,male,AD.vs.MCI; Homo_714,female,AD.vs.control; Homo_714,male,AD.vs.control; Homo_714,female,AD.vs.MCI; Homo_714,male,AD.vs.MCI; Homo_633,female,AD.vs.control; Homo_633,female,AD.vs.MCI; Emory,MCI,age; MCSA,totalSample,age; MCSA,control,age; ROSMAP,AD,last.visit.age; Emory,totalSample,immune-Monocyte; Emory,totalSample,immune-Macrophage; Emory,totalSample,immune-NK; Emory,totalSample,immune-Neutrophil; Emory,totalSample,immune-CD4_T; Emory,totalSample,immune-Tr1; Emory,totalSample,immune-nTreg; Emory,totalSample,immune-iTreg; Emory,totalSample,immune-Exhausted; Emory,totalSample,immune-Effector_memory; Emory,totalSample,immune-InfiltrationScore; Homo_723,totalSample,immune-Effector_memory; Homo_723,totalSample,immune-Tfh; Homo_723,totalSample,immune-InfiltrationScore; Homo_723,totalSample,immune-Th2; Homo_723,totalSample,immune-iTreg; Homo_723,totalSample,immune-CD4_T; Homo_723,totalSample,immune-Bcell; Homo_723,totalSample,immune-CD4_naive; Homo_723,totalSample,immune-nTreg; Homo_723,totalSample,immune-Tr1; Homo_723,totalSample,immune-Macrophage; Homo_723,totalSample,immune-CD8_T; Homo_714,totalSample,immune-Th2; Homo_714,totalSample,immune-Tfh; Homo_714,totalSample,immune-Effector_memory; Homo_714,totalSample,immune-InfiltrationScore; Homo_714,totalSample,immune-CD4_T; Homo_714,totalSample,immune-nTreg; Homo_714,totalSample,immune-Tr1; Homo_714,totalSample,immune-Central_memory; Homo_714,totalSample,immune-CD8_T; Homo_714,totalSample,immune-CD4_naive; Homo_633,totalSample,immune-InfiltrationScore; Homo_633,totalSample,immune-Effector_memory; Homo_633,totalSample,immune-Tfh; Homo_633,totalSample,immune-Th2; Homo_633,totalSample,immune-nTreg; Homo_633,totalSample,immune-CD4_T; Homo_633,totalSample,immune-CD4_naive; Homo_633,totalSample,immune-Central_memory; Homo_633,totalSample,immune-Bcell; Homo_633,totalSample,immune-Tr1; MCSA,totalSample,immune-DC; MCSA,totalSample,immune-Monocyte; MCSA,totalSample,immune-Neutrophil; MCSA,totalSample,immune-CD4_T; MCSA,totalSample,immune-CD8_T; MCSA,totalSample,immune-CD4_naive; MCSA,totalSample,immune-Tr1; MCSA,totalSample,immune-nTreg; MCSA,totalSample,immune-Th17; MCSA,totalSample,immune-Tfh; MCSA,totalSample,immune-Cytotoxic; MCSA,totalSample,immune-Central_memory; MCSA,totalSample,immune-Effector_memory; MCSA,totalSample,immune-InfiltrationScore; ROSMAP,totalSample,immune-Effector_memory; SRP223445,totalSample,immune-NK; SRP223445,totalSample,immune-Neutrophil; SRP223445,totalSample,immune-CD4_naive; [illegible]
EFNA5	Homo_723,female,AD.vs.control; Homo_723,male,AD.vs.control; Homo_723,female,AD.vs.MCI; Homo_723,male,AD.vs.MCI; Homo_714,female,AD.vs.control; Homo_714,male,AD.vs.control; Homo_714,female,AD.vs.MCI; Homo_714,male,AD.vs.MCI; Homo_633,female,AD.vs.control; Homo_633,female,AD.vs.MCI; MCSA,MCI,age; Emory,totalSample,immune-nTreg; Emory,totalSample,immune-Cytotoxic; Emory,totalSample,immune-Central_memory; Homo_723,totalSample,immune-InfiltrationScore; Homo_723,totalSample,immune-NK; Homo_723,totalSample,immune-Neutrophil; Homo_714,totalSample,immune-InfiltrationScore; Homo_714,totalSample,immune-NK; Homo_714,totalSample,immune-Bcell; Homo_633,totalSample,immune-InfiltrationScore; Homo_633,totalSample,immune-Cytotoxic; Homo_633,totalSample,immune-Bcell; Homo_633,totalSample,immune-Gamma_delta; Homo_633,totalSample,immune-NK; Homo_633,totalSample,immune-Effector_memory; Homo_633,totalSample,immune-MAIT; MCSA,totalSample,immune-DC; MCSA,totalSample,immune-Gamma_delta; MCSA,totalSample,immune-iTreg; MCSA,totalSample,immune-MAIT; ROSMAP,totalSample,immune-Central_memory; SRP223445,totalSample,immune-Exhausted; SRP223445,totalSample,immune-InfiltrationScore
MMRN1	Homo_723,female,AD.vs.control; Homo_723,male,AD.vs.control; Homo_723,female,AD.vs.MCI; Homo_723,male,AD.vs.MCI; Homo_723,female,MCI.vs.control; Homo_723,male,MCI.vs.control; Homo_714,female,AD.vs.control; Homo_714,male,AD.vs.control; Homo_714,female,AD.vs.MCI; Homo_714,male,AD.vs.MCI; Homo_714,female,MCI.vs.control; Homo_714,male,MCI.vs.control; Homo_633,female,AD.vs.control; Homo_633,female,AD.vs.MCI; ROSMAP,female,AD.vs.control; ACOM,totalSample,age; MCSA,control,age; ROSMAP,totalSample,age; ROSMAP,control,sample.age; Emory,totalSample,immune-Th1; Emory,totalSample,immune-Th17; Emory,totalSample,immune-Exhausted; Homo_723,totalSample,immune-Th17; Homo_723,totalSample,immune-Exhausted; Homo_723,totalSample,immune-Cytotoxic; Homo_723,totalSample,immune-CD8_T; Homo_723,totalSample,immune-CD4_naive; Homo_723,totalSample,immune-Gamma_delta; Homo_723,totalSample,immune-CD4_T; Homo_723,totalSample,immune-Central_memory; Homo_714,totalSample,immune-Th17; Homo_714,totalSample,immune-Exhausted; Homo_714,totalSample,immune-Cytotoxic; Homo_714,totalSample,immune-Gamma_delta; Homo_714,totalSample,immune-CD4_naive; Homo_714,totalSample,immune-CD8_T; Homo_714,totalSample,immune-Central_memory; Homo_633,totalSample,immune-Tfh; Homo_633,totalSample,immune-Gamma_delta; Homo_633,totalSample,immune-Th2; Homo_633,totalSample,immune-Th17; Homo_633,totalSample,immune-CD4_T; Homo_633,totalSample,immune-Central_memory; Homo_633,totalSample,immune-Exhausted; Homo_633,totalSample,immune-CD4_naive; Homo_633,totalSample,immune-Tr1; Homo_633,totalSample,immune-Effector_memory; Homo_633,totalSample,immune-iTreg; Homo_633,totalSample,immune-InfiltrationScore; Homo_633,totalSample,immune-Cytotoxic; MCSA,totalSample,immune-CD4_T; MCSA,totalSample,immune-CD8_T; MCSA,totalSample,immune-Gamma_delta; MCSA,totalSample,immune-CD4_naive; MCSA,totalSample,immune-Tr1; MCSA,totalSample,immune-nTreg; MCSA,totalSample,immune-iTreg; MCSA,totalSample,immune-Th17; MCSA,totalSample,immune-Tfh; MCSA,totalSample,immune-Cytotoxic; MCSA,totalSample,immune-Exhausted; MCSA,totalSample,immune-Central_memory; ROSMAP,totalSample,immune-Th17; ROSMAP,totalSample,immune-InfiltrationScore; SRP223445,totalSample,immune-CD4_naive; [illegible]
CLCN3	Homo_723,female,AD.vs.control; Homo_723,male,AD.vs.control; Homo_723,female,AD.vs.MCI; Homo_723,male,AD.vs.MCI; Homo_723,female,MCI.vs.control; Homo_723,male,MCI.vs.control; Homo_714,female,AD.vs.control; Homo_714,male,AD.vs.control; Homo_714,female,AD.vs.MCI; Homo_714,male,AD.vs.MCI; Homo_714,female,MCI.vs.control; Homo_714,male,MCI.vs.control; Homo_633,female,AD.vs.control; Homo_633,female,AD.vs.MCI; Homo_633,female,MCI.vs.control; SRP223445,female,AD.vs.control; MCSA,MCI,age; Emory,totalSample,immune-CD4_T; Emory,totalSample,immune-Tr1; Emory,totalSample,immune-iTreg; Emory,totalSample,immune-Th17; Emory,totalSample,immune-MAIT; Emory,totalSample,immune-Central_memory; Emory,totalSample,immune-InfiltrationScore; Homo_723,totalSample,immune-Exhausted; Homo_723,totalSample,immune-Th17; Homo_723,totalSample,immune-Cytotoxic; Homo_723,totalSample,immune-CD4_naive; Homo_723,totalSample,immune-CD4_T; Homo_723,totalSample,immune-CD8_T; Homo_723,totalSample,immune-Tfh; Homo_723,totalSample,immune-Th2; Homo_723,totalSample,immune-Tr1; Homo_723,totalSample,immune-Central_memory; Homo_723,totalSample,immune-Monocyte; Homo_723,totalSample,immune-Th1; Homo_723,totalSample,immune-nTreg; Homo_723,totalSample,immune-InfiltrationScore; Homo_723,totalSample,immune-Gamma_delta; Homo_714,totalSample,immune-Th17; Homo_714,totalSample,immune-CD4_naive; Homo_714,totalSample,immune-Exhausted; Homo_714,totalSample,immune-Monocyte; Homo_714,totalSample,immune-CD4_T; Homo_714,totalSample,immune-Central_memory; Homo_714,totalSample,immune-Tfh; Homo_714,totalSample,immune-Gamma_delta; Homo_714,totalSample,immune-Th2; Homo_714,totalSample,immune-InfiltrationScore; Homo_714,totalSample,immune-Cytotoxic; Homo_633,totalSample,immune-Tfh; Homo_633,totalSample,immune-Th2; Homo_633,totalSample,immune-CD4_T; Homo_633,totalSample,immune-Central_memory; Homo_633,totalSample,immune-CD4_naive; Homo_633,totalSample,immune-Effector_memory; Homo_633,totalSample,immune-Monocyte; Homo_633,totalSample,immune-Th17; Homo_633,totalSample,immune-Gamma_delta; Homo_633,totalSample,immune-Tr1; Homo_633,totalSample,immune-Exhausted; Homo_633,totalSample,immune-InfiltrationScore; Homo_633,totalSample,immune-iTreg; Homo_633,totalSample,immune-NKT; Homo_633,totalSample,immune-Bcell; MCSA,totalSample,immune-DC; MCSA,totalSample,immune-Macrophage; MCSA,totalSample,immune-CD4_T; MCSA,totalSample,immune-CD8_T; MCSA,totalSample,immune-Gamma_delta; MCSA,totalSample,immune-CD4_naive; MCSA,totalSample,immune-Tr1; MCSA,totalSample,immune-nTreg; MCSA,totalSample,immune-iTreg; MCSA,totalSample,immune-Th17; MCSA,totalSample,immune-Tfh; MCSA,totalSample,immune-CD8_naive; MCSA,totalSample,immune-Cytotoxic; MCSA,totalSample,immune-Exhausted; MCSA,totalSample,immune-MAIT; MCSA,totalSample,immune-Central_memory; ROSMAP,totalSample,immune-iTreg; [illegible]
CD96	Homo_723,female,AD.vs.control; Homo_723,male,AD.vs.control; Homo_723,female,AD.vs.MCI; Homo_723,male,AD.vs.MCI; Homo_714,female,AD.vs.control; Homo_714,male,AD.vs.control; Homo_714,female,AD.vs.MCI; Homo_714,male,AD.vs.MCI; Homo_633,female,AD.vs.control; Homo_633,female,AD.vs.MCI; ROSMAP,female,MCI.vs.control; SRP223445,female,AD.vs.control; MCSA,totalSample,age; MCSA,control,age; Emory,totalSample,immune-Macrophage; Emory,totalSample,immune-NK; Emory,totalSample,immune-Neutrophil; Emory,totalSample,immune-CD4_T; Emory,totalSample,immune-CD8_T; Emory,totalSample,immune-Tr1; Emory,totalSample,immune-iTreg; Emory,totalSample,immune-Tfh; Emory,totalSample,immune-CD8_naive; Emory,totalSample,immune-Exhausted; Emory,totalSample,immune-MAIT; Emory,totalSample,immune-Central_memory; Emory,totalSample,immune-Effector_memory; Emory,totalSample,immune-InfiltrationScore; Homo_723,totalSample,immune-Tfh; Homo_723,totalSample,immune-iTreg; Homo_723,totalSample,immune-Th2; Homo_723,totalSample,immune-CD4_T; Homo_723,totalSample,immune-CD4_naive; Homo_723,totalSample,immune-Tr1; Homo_723,totalSample,immune-Effector_memory; Homo_723,totalSample,immune-InfiltrationScore; Homo_723,totalSample,immune-Central_memory; Homo_723,totalSample,immune-nTreg; Homo_723,totalSample,immune-Macrophage; Homo_723,totalSample,immune-MAIT; Homo_723,totalSample,immune-CD8_T; Homo_723,totalSample,immune-Bcell; Homo_723,totalSample,immune-DC; Homo_714,totalSample,immune-Tfh; Homo_714,totalSample,immune-Th2; Homo_714,totalSample,immune-CD4_T; Homo_714,totalSample,immune-Effector_memory; Homo_714,totalSample,immune-CD4_naive; Homo_714,totalSample,immune-Central_memory; Homo_714,totalSample,immune-Tr1; Homo_714,totalSample,immune-iTreg; Homo_714,totalSample,immune-nTreg; Homo_714,totalSample,immune-InfiltrationScore; Homo_714,totalSample,immune-Macrophage; Homo_714,totalSample,immune-MAIT; Homo_714,totalSample,immune-CD8_T; Homo_714,totalSample,immune-Bcell; Homo_714,totalSample,immune-Neutrophil; Homo_633,totalSample,immune-Tfh; Homo_633,totalSample,immune-CD4_T; Homo_633,totalSample,immune-CD4_naive; Homo_633,totalSample,immune-Central_memory; Homo_633,totalSample,immune-Effector_memory; Homo_633,totalSample,immune-InfiltrationScore; Homo_633,totalSample,immune-Tr1; Homo_633,totalSample,immune-nTreg; Homo_633,totalSample,immune-iTreg; Homo_633,totalSample,immune-Th2; Homo_633,totalSample,immune-Neutrophil; Homo_633,totalSample,immune-MAIT; Homo_633,totalSample,immune-Bcell; Homo_633,totalSample,immune-Macrophage; Homo_633,totalSample,immune-NKT; Homo_633,totalSample,immune-Th17; Homo_633,totalSample,immune-CD8_T; MCSA,totalSample,immune-DC; MCSA,totalSample,immune-Macrophage; MCSA,totalSample,immune-Neutrophil; MCSA,totalSample,immune-CD4_T; MCSA,totalSample,immune-CD4_naive; MCSA,totalSample,immune-Tr1; MCSA,totalSample,immune-nTreg; MCSA,totalSample,immune-iTreg; MCSA,totalSample,immune-Th17; MCSA,totalSample,immune-Tfh; MCSA,totalSample,immune-CD8_naive; MCSA,totalSample,immune-Exhausted; MCSA,totalSample,immune-MAIT; MCSA,totalSample,immune-Central_memory; MCSA,totalSample,immune-Effector_memory; MCSA,totalSample,immune-InfiltrationScore; ROSMAP,totalSample,immune-[illegible]
PAFAH2	Homo_723,female,AD.vs.control; Homo_723,male,AD.vs.control; Homo_723,female,AD.vs.MCI; Homo_723,male,AD.vs.MCI; Homo_714,female,AD.vs.control; Homo_714,male,AD.vs.control; Homo_714,female,AD.vs.MCI; Homo_714,male,AD.vs.MCI; Homo_633,female,AD.vs.control; Homo_633,female,AD.vs.MCI; MCSA,MCI,age; Emory,totalSample,immune-Macrophage; Emory,totalSample,immune-NK; Emory,totalSample,immune-Neutrophil; Emory,totalSample,immune-CD4_T; Emory,totalSample,immune-CD8_T; Emory,totalSample,immune-Gamma_delta; Emory,totalSample,immune-Tr1; Emory,totalSample,immune-iTreg; Emory,totalSample,immune-Cytotoxic; Emory,totalSample,immune-Exhausted; Emory,totalSample,immune-Effector_memory; Emory,totalSample,immune-InfiltrationScore; Homo_723,totalSample,immune-InfiltrationScore; Homo_723,totalSample,immune-Tfh; Homo_723,totalSample,immune-Effector_memory; Homo_723,totalSample,immune-iTreg; Homo_723,totalSample,immune-Th2; Homo_723,totalSample,immune-CD4_T; Homo_723,totalSample,immune-CD4_naive; Homo_723,totalSample,immune-Tr1; Homo_723,totalSample,immune-Bcell; Homo_723,totalSample,immune-Macrophage; Homo_723,totalSample,immune-MAIT; Homo_723,totalSample,immune-DC; Homo_714,totalSample,immune-Tfh; Homo_714,totalSample,immune-Effector_memory; Homo_714,totalSample,immune-InfiltrationScore; Homo_714,totalSample,immune-Th2; Homo_714,totalSample,immune-CD4_T; Homo_714,totalSample,immune-Tr1; Homo_714,totalSample,immune-CD4_naive; Homo_714,totalSample,immune-iTreg; Homo_714,totalSample,immune-Central_memory; Homo_714,totalSample,immune-Macrophage; Homo_714,totalSample,immune-Bcell; Homo_714,totalSample,immune-MAIT; Homo_633,totalSample,immune-InfiltrationScore; Homo_633,totalSample,immune-Effector_memory; Homo_633,totalSample,immune-Tfh; Homo_633,totalSample,immune-CD4_naive; Homo_633,totalSample,immune-CD4_T; Homo_633,totalSample,immune-Th2; Homo_633,totalSample,immune-Central_memory; Homo_633,totalSample,immune-Bcell; Homo_633,totalSample,immune-Tr1; Homo_633,totalSample,immune-Neutrophil; Homo_633,totalSample,immune-iTreg; Homo_633,totalSample,immune-NK; Homo_633,totalSample,immune-MAIT; MCSA,totalSample,immune-DC; MCSA,totalSample,immune-Macrophage; MCSA,totalSample,immune-CD4_T; MCSA,totalSample,immune-CD4_naive; MCSA,totalSample,immune-Tr1; MCSA,totalSample,immune-nTreg; MCSA,totalSample,immune-iTreg; MCSA,totalSample,immune-Th17; MCSA,totalSample,immune-Tfh; MCSA,totalSample,immune-CD8_naive; MCSA,totalSample,immune-Exhausted; MCSA,totalSample,immune-MAIT; MCSA,totalSample,immune-[illegible]
EFHC2	Homo_723,female,AD.vs.control; Homo_723,male,AD.vs.control; Homo_723,female,AD.vs.MCI; Homo_723,male,AD.vs.MCI; Homo_723,female,MCI.vs.control; Homo_723,male,MCI.vs.control; Homo_714,female,AD.vs.control; Homo_714,male,AD.vs.control; Homo_714,female,AD.vs.MCI; Homo_714,male,AD.vs.MCI; Homo_714,female,MCI.vs.control; Homo_714,male,MCI.vs.control; Homo_633,female,AD.vs.control; Homo_633,female,AD.vs.MCI; SRP223445,AD,male.vs.female; MCSA,MCI,age; ROSMAP,MCI,last.visit.age; Emory,totalSample,immune-DC; Emory,totalSample,immune-Neutrophil; Emory,totalSample,immune-Gamma_delta; Emory,totalSample,immune-Tr1; Emory,totalSample,immune-iTreg; Emory,totalSample,immune-Th1; Emory,totalSample,immune-Th17; Emory,totalSample,immune-Cytotoxic; Emory,totalSample,immune-Exhausted; Homo_723,totalSample,immune-Exhausted; Homo_723,totalSample,immune-Th17; Homo_723,totalSample,immune-Cytotoxic; Homo_723,totalSample,immune-CD8_T; Homo_723,totalSample,immune-Monocyte; Homo_723,totalSample,immune-Gamma_delta; Homo_723,totalSample,immune-CD4_naive; Homo_723,totalSample,immune-Tr1; Homo_714,totalSample,immune-Exhausted; Homo_714,totalSample,immune-Th17; Homo_714,totalSample,immune-Monocyte; Homo_714,totalSample,immune-CD8_T; Homo_714,totalSample,immune-Gamma_delta; Homo_714,totalSample,immune-Cytotoxic; Homo_633,totalSample,immune-Th2; Homo_633,totalSample,immune-Exhausted; Homo_633,totalSample,immune-Tfh; Homo_633,totalSample,immune-Monocyte; Homo_633,totalSample,immune-Gamma_delta; Homo_633,totalSample,immune-Th17; Homo_633,totalSample,immune-CD4_T; Homo_633,totalSample,immune-Tr1; Homo_633,totalSample,immune-NKT; Homo_633,totalSample,immune-DC; Homo_633,totalSample,immune-Central_memory; MCSA,totalSample,immune-DC; MCSA,totalSample,immune-Monocyte; MCSA,totalSample,immune-Neutrophil; MCSA,totalSample,immune-CD4_T; MCSA,totalSample,immune-CD8_T; MCSA,totalSample,immune-CD4_naive; MCSA,totalSample,immune-Tr1; MCSA,totalSample,immune-nTreg; MCSA,totalSample,immune-iTreg; MCSA,totalSample,immune-Th1; MCSA,totalSample,immune-Th17; MCSA,totalSample,immune-Tfh; MCSA,totalSample,immune-CD8_naive; MCSA,totalSample,immune-Cytotoxic; MCSA,totalSample,immune-Exhausted; MCSA,totalSample,immune-MAIT; [illegible]
GSTA4	Homo_723,female,AD.vs.control; Homo_723,male,AD.vs.control; Homo_723,female,AD.vs.MCI; Homo_723,male,AD.vs.MCI; Homo_723,female,MCI.vs.control; Homo_723,male,MCI.vs.control; Homo_714,female,AD.vs.MCI; Homo_714,male,AD.vs.MCI; Homo_633,female,AD.vs.control; Homo_633,female,AD.vs.MCI; Emory,control,age; MCSA,MCI,age; Emory,totalSample,immune-Macrophage; Emory,totalSample,immune-NK; Emory,totalSample,immune-Neutrophil; Emory,totalSample,immune-CD8_T; Emory,totalSample,immune-Tr1; Emory,totalSample,immune-Exhausted; Emory,totalSample,immune-InfiltrationScore; Homo_723,totalSample,immune-InfiltrationScore; Homo_723,totalSample,immune-Neutrophil; Homo_723,totalSample,immune-Th2; Homo_723,totalSample,immune-Exhausted; Homo_723,totalSample,immune-NK; Homo_714,totalSample,immune-InfiltrationScore; Homo_714,totalSample,immune-Neutrophil; Homo_714,totalSample,immune-NK; Homo_714,totalSample,immune-Monocyte; Homo_633,totalSample,immune-InfiltrationScore; Homo_633,totalSample,immune-Neutrophil; Homo_633,totalSample,immune-Tfh; Homo_633,totalSample,immune-Th2; Homo_633,totalSample,immune-Effector_memory; Homo_633,totalSample,immune-CD4_T; Homo_633,totalSample,immune-NK; Homo_633,totalSample,immune-CD4_naive; MCSA,totalSample,immune-DC; MCSA,totalSample,immune-Macrophage; MCSA,totalSample,immune-NK; MCSA,totalSample,immune-CD4_T; MCSA,totalSample,immune-CD4_naive; MCSA,totalSample,immune-Tr1; MCSA,totalSample,immune-nTreg; MCSA,totalSample,immune-iTreg; MCSA,totalSample,immune-Th2; MCSA,totalSample,immune-MAIT; ROSMAP,totalSample,immune-InfiltrationScore; SRP223445,totalSample,immune-

C1orf198	Homo_723,female,AD.vs.control; Homo_723,male,AD.vs.control; Homo_723,female,AD.vs.MCI; Homo_723,male,AD.vs.MCI; Homo_723,female,MCI.vs.control; Homo_723,male,MCI.vs.control; Homo_714,female,AD.vs.control; Homo_714,male,AD.vs.control; Homo_714,female,AD.vs.MCI; Homo_714,male,AD.vs.MCI; Homo_714,female,MCI.vs.control; Homo_714,male,MCI.vs.control; Homo_633,female,AD.vs.control; Homo_633,female,AD.vs.MCI; ROSMAP,female,AD.vs.control; SRP223445,female,AD.vs.control; ACOM,totalSample,age; MCSA,totalSample,age; MCSA,control,age; ROSMAP,AD,sample.age; ROSMAP,MCI,last.visit.age; Emory,totalSample,immune-Th1; Homo_723,totalSample,immune-DC; Homo_723,totalSample,immune-Monocyte; Homo_723,totalSample,immune-Central_memory; Homo_723,totalSample,immune-Th17; Homo_714,totalSample,immune-DC; Homo_714,totalSample,immune-InfiltrationScore; Homo_633,totalSample,immune-InfiltrationScore; Homo_633,totalSample,immune-DC; Homo_633,totalSample,immune-Monocyte; MCSA,totalSample,immune-DC; MCSA,totalSample,immune-Monocyte; MCSA,totalSample,immune-CD4_T; MCSA,totalSample,immune-Gamma_delta; MCSA,totalSample,immune-CD4_naive; MCSA,totalSample,immune-Tr1; MCSA,totalSample,immune-nTreg; MCSA,totalSample,immune-Th17; MCSA,totalSample,immune-Tfh; MCSA,totalSample,immune-Central_memory; MCSA,totalSample,immune-Effector_memory; MCSA,totalSample,immune-InfiltrationScore; ROSMAP,totalSample,immune-Neutrophil; ROSMAP,totalSample,immune-InfiltrationScore; SRP223445,totalSample,immune-CD4_naive; SRP223445,totalSample,immune-Exhausted
GBP6	Homo_723,female,AD.vs.control; Homo_723,male,AD.vs.control; Homo_723,female,AD.vs.MCI; Homo_723,male,AD.vs.MCI; Homo_714,female,AD.vs.control; Homo_714,male,AD.vs.control; Homo_714,female,AD.vs.MCI; Homo_714,male,AD.vs.MCI; Homo_633,female,AD.vs.control; Homo_633,female,AD.vs.MCI; ROSMAP,AD,male.vs.female; MCSA,MCI,age; Emory,totalSample,immune-B_cell; Emory,totalSample,immune-Neutrophil; Emory,totalSample,immune-CD4_T; Emory,totalSample,immune-CD8_T; Emory,totalSample,immune-Gamma_delta; Emory,totalSample,immune-Tfh; Emory,totalSample,immune-CD8_naive; Homo_723,totalSample,immune-Monocyte; Homo_723,totalSample,immune-Neutrophil; Homo_723,totalSample,immune-Th2; Homo_723,totalSample,immune-Bcell; Homo_723,totalSample,immune-InfiltrationScore; Homo_723,totalSample,immune-DC; Homo_723,totalSample,immune-Gamma_delta; Homo_714,totalSample,immune-Th2; Homo_714,totalSample,immune-Monocyte; Homo_633,totalSample,immune-Monocyte; Homo_633,totalSample,immune-Th2; Homo_633,totalSample,immune-Exhausted; Homo_633,totalSample,immune-InfiltrationScore; Homo_633,totalSample,immune-Gamma_delta; Homo_633,totalSample,immune-Th17; MCSA,totalSample,immune-Neutrophil; MCSA,totalSample,immune-CD8_T; MCSA,totalSample,immune-Cytotoxic; MCSA,totalSample,immune-Effector_memory; ROSMAP,male and
LRP11	Homo_723,female,AD.vs.control; Homo_723,male,AD.vs.control; Homo_723,female,AD.vs.MCI; Homo_723,male,AD.vs.MCI; Homo_714,male,AD.vs.MCI; Homo_633,female,AD.vs.control; Homo_633,female,AD.vs.MCI; MCSA,MCI,age; ROSMAP,MCI,sample.age; Emory,totalSample,immune-CD4_T; Emory,totalSample,immune-Tr1; Emory,totalSample,immune-iTreg; Emory,totalSample,immune-InfiltrationScore; Homo_723,totalSample,immune-CD4_T; Homo_723,totalSample,immune-Tfh; Homo_723,totalSample,immune-CD4_naive; Homo_723,totalSample,immune-iTreg; Homo_723,totalSample,immune-Tr1; Homo_723,totalSample,immune-Th2; Homo_723,totalSample,immune-Central_memory; Homo_723,totalSample,immune-InfiltrationScore; Homo_723,totalSample,immune-nTreg; Homo_723,totalSample,immune-Th1; Homo_723,totalSample,immune-Cytotoxic; Homo_714,totalSample,immune-Tfh; Homo_714,totalSample,immune-CD4_naive; Homo_714,totalSample,immune-CD4_T; Homo_714,totalSample,immune-Tr1; Homo_714,totalSample,immune-iTreg; Homo_714,totalSample,immune-InfiltrationScore; Homo_714,totalSample,immune-Th2; Homo_714,totalSample,immune-Central_memory; Homo_714,totalSample,immune-NKT; Homo_714,totalSample,immune-Effector_memory; Homo_633,totalSample,immune-Tfh; Homo_633,totalSample,immune-NKT; Homo_633,totalSample,immune-CD4_T; Homo_633,totalSample,immune-CD4_naive; Homo_633,totalSample,immune-InfiltrationScore; Homo_633,totalSample,immune-Tr1; Homo_633,totalSample,immune-Central_memory; Homo_633,totalSample,immune-iTreg; Homo_633,totalSample,immune-Effector_memory; Homo_633,totalSample,immune-Th2; MCSA,totalSample,immune-DC; MCSA,totalSample,immune-Monocyte; MCSA,totalSample,immune-Macrophage; MCSA,totalSample,immune-CD4_T; MCSA,totalSample,immune-CD4_naive; MCSA,totalSample,immune-Tr1; MCSA,totalSample,immune-nTreg; MCSA,totalSample,immune-iTreg; MCSA,totalSample,immune-Th17; MCSA,totalSample,immune-Tfh; MCSA,totalSample,immune-CD8_naive; MCSA,totalSample,immune-Cytotoxic; MCSA,totalSample,immune-
POGLUT1	Homo_723,female,AD.vs.control; Homo_723,male,AD.vs.control; Homo_723,female,AD.vs.MCI; Homo_723,male,AD.vs.MCI; Homo_714,female,AD.vs.control; Homo_714,male,AD.vs.control; Homo_714,female,AD.vs.MCI; Homo_714,male,AD.vs.MCI; Homo_633,female,AD.vs.control; Homo_633,female,AD.vs.MCI; ROSMAP,female,AD.vs.control; MCSA,MCI,age; Emory,totalSample,immune-Macrophage; Emory,totalSample,immune-NK; Emory,totalSample,immune-Neutrophil; Emory,totalSample,immune-CD4_T; Emory,totalSample,immune-CD8_T; Emory,totalSample,immune-Tr1; Emory,totalSample,immune-iTreg; Emory,totalSample,immune-Tfh; Emory,totalSample,immune-Central_memory; Emory,totalSample,immune-Effector_memory; Emory,totalSample,immune-InfiltrationScore; Homo_723,totalSample,immune-iTreg; Homo_723,totalSample,immune-Tfh; Homo_723,totalSample,immune-Th2; Homo_723,totalSample,immune-CD4_T; Homo_723,totalSample,immune-Tr1; Homo_723,totalSample,immune-CD4_naive; Homo_723,totalSample,immune-Effector_memory; Homo_723,totalSample,immune-InfiltrationScore; Homo_723,totalSample,immune-DC; Homo_723,totalSample,immune-Central_memory; Homo_723,totalSample,immune-Macrophage; Homo_723,totalSample,immune-MAIT; Homo_723,totalSample,immune-nTreg; Homo_723,totalSample,immune-Gamma_delta; Homo_723,totalSample,immune-Bcell; Homo_723,totalSample,immune-NKT; Homo_714,totalSample,immune-Tfh; Homo_714,totalSample,immune-Th2; Homo_714,totalSample,immune-CD4_T; Homo_714,totalSample,immune-Tr1; Homo_714,totalSample,immune-Effector_memory; Homo_714,totalSample,immune-CD4_naive; Homo_714,totalSample,immune-iTreg; Homo_714,totalSample,immune-Central_memory; Homo_714,totalSample,immune-InfiltrationScore; Homo_714,totalSample,immune-nTreg; Homo_714,totalSample,immune-DC; Homo_714,totalSample,immune-MAIT; Homo_714,totalSample,immune-Macrophage; Homo_714,totalSample,immune-NKT; Homo_714,totalSample,immune-Bcell; Homo_714,totalSample,immune-Gamma_delta; Homo_633,totalSample,immune-Tfh; Homo_633,totalSample,immune-CD4_T; Homo_633,totalSample,immune-CD4_naive; Homo_633,totalSample,immune-Effector_memory; Homo_633,totalSample,immune-Central_memory; Homo_633,totalSample,immune-Tr1; Homo_633,totalSample,immune-InfiltrationScore; Homo_633,totalSample,immune-Th2; Homo_633,totalSample,immune-NKT; Homo_633,totalSample,immune-iTreg; Homo_633,totalSample,immune-Bcell; Homo_633,totalSample,immune-nTreg; Homo_633,totalSample,immune-Th1; Homo_633,totalSample,immune-DC; Homo_633,totalSample,immune-Th17; Homo_633,totalSample,immune-MAIT; Homo_633,totalSample,immune-Neutrophil; Homo_633,totalSample,immune-Gamma_delta; MCSA,totalSample,immune-DC; MCSA,totalSample,immune-Monocyte; MCSA,totalSample,immune-Macrophage; MCSA,totalSample,immune-CD4_T; MCSA,totalSample,immune-CD8_T; MCSA,totalSample,immune-CD4_naive; MCSA,totalSample,immune-Tr1; MCSA,totalSample,immune-nTreg; MCSA,totalSample,immune-iTreg; MCSA,totalSample,immune-Th2; MCSA,totalSample,immune-Th17; MCSA,totalSample,immune-Tfh; MCSA,totalSample,immune-CD8_naive; MCSA,totalSample,immune-Cytotoxic; MCSA,totalSample,immune-Exhausted; MCSA,totalSample,immune-MAIT; MCSA,totalSample,immune-Central_memory; ROSMAP,totalSample,immune-[illegible]
USP31	Homo_723,female,AD.vs.control; Homo_723,male,AD.vs.control; Homo_723,female,AD.vs.MCI; Homo_723,male,AD.vs.MCI; Homo_723,female,MCI.vs.control; Homo_723,male,MCI.vs.control; Homo_714,female,AD.vs.control; Homo_714,male,AD.vs.control; Homo_714,female,AD.vs.MCI; Homo_714,male,AD.vs.MCI; Homo_714,female,MCI.vs.control; Homo_714,male,MCI.vs.control; Homo_633,female,AD.vs.control; Homo_633,female,AD.vs.MCI; ROSMAP,AD,last.visit.age; Emory,totalSample,immune-Tr1; Emory,totalSample,immune-InfiltrationScore; Homo_723,totalSample,immune-Th17; Homo_723,totalSample,immune-Exhausted; Homo_723,totalSample,immune-CD4_naive; Homo_723,totalSample,immune-CD4_T; Homo_723,totalSample,immune-InfiltrationScore; Homo_723,totalSample,immune-Cytotoxic; Homo_723,totalSample,immune-CD8_T; Homo_723,totalSample,immune-Tfh; Homo_723,totalSample,immune-Central_memory; Homo_723,totalSample,immune-Tr1; Homo_723,totalSample,immune-nTreg; Homo_723,totalSample,immune-Th2; Homo_723,totalSample,immune-Effector_memory; Homo_714,totalSample,immune-CD4_naive; Homo_714,totalSample,immune-InfiltrationScore; Homo_714,totalSample,immune-Th17; Homo_714,totalSample,immune-Exhausted; Homo_714,totalSample,immune-CD4_T; Homo_714,totalSample,immune-Tfh; Homo_714,totalSample,immune-Central_memory; Homo_714,totalSample,immune-NKT; Homo_714,totalSample,immune-Effector_memory; Homo_633,totalSample,immune-Tfh; Homo_633,totalSample,immune-InfiltrationScore; Homo_633,totalSample,immune-CD4_T; Homo_633,totalSample,immune-Effector_memory; Homo_633,totalSample,immune-CD4_naive; Homo_633,totalSample,immune-Th2; Homo_633,totalSample,immune-Central_memory; Homo_633,totalSample,immune-Tr1; Homo_633,totalSample,immune-Th17; Homo_633,totalSample,immune-NKT; Homo_633,totalSample,immune-Exhausted; Homo_633,totalSample,immune-iTreg; Homo_633,totalSample,immune-Gamma_delta; Homo_633,totalSample,immune-Monocyte; Homo_633,totalSample,immune-Bcell; MCSA,totalSample,immune-DC; MCSA,totalSample,immune-CD4_T; MCSA,totalSample,immune-CD4_naive; MCSA,totalSample,immune-Tr1; MCSA,totalSample,immune-nTreg; MCSA,totalSample,immune-iTreg; MCSA,totalSample,immune-Th17; MCSA,totalSample,immune-Tfh; MCSA,totalSample,immune-CD8_naive; MCSA,totalSample,immune-Cytotoxic; MCSA,totalSample,immune-Exhausted; MCSA,totalSample,immune-MAIT; MCSA,totalSample,immune-Central_memory; ROSMAP,totalSample,immune-InfiltrationScore; SRP223445,totalSample,immune-CD4_naive; SRP223445,totalSample,immune-Exhausted
CNTN2	Homo_723,female,AD.vs.control; Homo_723,female,AD.vs.MCI; Homo_633,female,MCI.vs.control; ROSMAP,female,AD.vs.control; ROSMAP,male,AD.vs.control; Emory,totalSample,immune-CD8_T; Emory,totalSample,immune-Gamma_delta; ROSMAP,totalSample,immune-InfiltrationScore; SRP223445,totalSample,immune-Exhausted; ROSMAP,male and female-
SRPRB	Homo_723,female,AD.vs.control; Homo_723,male,AD.vs.control; Homo_723,female,AD.vs.MCI; Homo_723,male,AD.vs.MCI; Homo_714,female,AD.vs.control; Homo_714,male,AD.vs.control; Homo_714,female,AD.vs.MCI; Homo_714,male,AD.vs.MCI; Homo_633,female,AD.vs.control; Homo_633,female,AD.vs.MCI; Homo_633,female,MCI.vs.control; ROSMAP,female,AD.vs.control; Emory,totalSample,immune-Macrophage; Emory,totalSample,immune-NK; Emory,totalSample,immune-Neutrophil; Emory,totalSample,immune-CD4_T; Emory,totalSample,immune-CD8_T; Emory,totalSample,immune-Tr1; Emory,totalSample,immune-iTreg; Emory,totalSample,immune-Tfh; Emory,totalSample,immune-Exhausted; Emory,totalSample,immune-MAIT; Emory,totalSample,immune-Effector_memory; Emory,totalSample,immune-InfiltrationScore; Homo_723,totalSample,immune-Th2; Homo_723,totalSample,immune-Tfh; Homo_723,totalSample,immune-iTreg; Homo_723,totalSample,immune-CD4_T; Homo_723,totalSample,immune-Effector_memory; Homo_723,totalSample,immune-Tr1; Homo_723,totalSample,immune-DC; Homo_723,totalSample,immune-CD4_naive; Homo_723,totalSample,immune-Macrophage; Homo_723,totalSample,immune-InfiltrationScore; Homo_723,totalSample,immune-Monocyte; Homo_723,totalSample,immune-Bcell; Homo_723,totalSample,immune-MAIT; Homo_714,totalSample,immune-Th2; Homo_714,totalSample,immune-Tfh; Homo_714,totalSample,immune-Effector_memory; Homo_714,totalSample,immune-CD4_T; Homo_714,totalSample,immune-Tr1; Homo_714,totalSample,immune-Central_memory; Homo_714,totalSample,immune-CD4_naive; Homo_714,totalSample,immune-iTreg; Homo_714,totalSample,immune-InfiltrationScore; Homo_714,totalSample,immune-Macrophage; Homo_714,totalSample,immune-Bcell; Homo_714,totalSample,immune-MAIT; Homo_714,totalSample,immune-CD8_T; Homo_633,totalSample,immune-Tfh; Homo_633,totalSample,immune-Th2; Homo_633,totalSample,immune-CD4_T; Homo_633,totalSample,immune-Effector_memory; Homo_633,totalSample,immune-CD4_naive; Homo_633,totalSample,immune-Central_memory; Homo_633,totalSample,immune-InfiltrationScore; Homo_633,totalSample,immune-Monocyte; Homo_633,totalSample,immune-Bcell; Homo_633,totalSample,immune-Tr1; Homo_633,totalSample,immune-Neutrophil; MCSA,totalSample,immune-DC; MCSA,totalSample,immune-Macrophage; MCSA,totalSample,immune-Neutrophil; MCSA,totalSample,immune-CD4_T; MCSA,totalSample,immune-CD4_naive; MCSA,totalSample,immune-Tr1; MCSA,totalSample,immune-nTreg; MCSA,totalSample,immune-iTreg; MCSA,totalSample,immune-Th2; MCSA,totalSample,immune-Th17; MCSA,totalSample,immune-Tfh; MCSA,totalSample,immune-CD8_naive; MCSA,totalSample,immune-MAIT; MCSA,totalSample,immune-Central_memory; MCSA,totalSample,immune-Effector_memory; ROSMAP,totalSample,immune-Th17; ROSMAP,totalSample,immune-Effector_memory; ROSMAP,totalSample,immune-InfiltrationScore; ROSMAP,female-excludeAge90+,survival; ROSMAP,female-includeAge90+,survival; ROSMAP,male and female-includeAge90+,survival
LIMS3	Homo_723,female,AD.vs.control; Homo_723,male,AD.vs.control; Homo_723,female,AD.vs.MCI; Homo_723,male,AD.vs.MCI; Homo_723,AD,male.vs.female; Homo_714,female,AD.vs.control; Homo_714,male,AD.vs.control; Homo_714,female,AD.vs.MCI; Homo_714,male,AD.vs.MCI; Homo_633,female,AD.vs.control; Homo_633,female,AD.vs.MCI; SRP223445,female,AD.vs.control; Homo_723,totalSample,immune-Th2; Homo_723,totalSample,immune-Monocyte; Homo_723,totalSample,immune-Gamma_delta; Homo_723,totalSample,immune-CD4_T; Homo_723,totalSample,immune-Th17; Homo_723,totalSample,immune-Tfh; Homo_723,totalSample,immune-Tr1; Homo_714,totalSample,immune-Monocyte; Homo_714,totalSample,immune-Th2; Homo_714,totalSample,immune-Tfh; Homo_714,totalSample,immune-Gamma_delta; Homo_714,totalSample,immune-Th17; Homo_714,totalSample,immune-CD4_T; Homo_633,totalSample,immune-Th2; Homo_633,totalSample,immune-Monocyte; Homo_633,totalSample,immune-Tfh; Homo_633,totalSample,immune-Th17; Homo_633,totalSample,immune-Gamma_delta; Homo_633,totalSample,immune-CD4_T; Homo_633,totalSample,immune-Tr1; MCSA,totalSample,immune-DC; MCSA,totalSample,immune-Monocyte; MCSA,totalSample,immune-CD4_T; MCSA,totalSample,immune-CD8_T; MCSA,totalSample,immune-Tr1; MCSA,totalSample,immune-iTreg; MCSA,totalSample,immune-Th2; MCSA,totalSample,immune-Th17; MCSA,totalSample,immune-Cytotoxic; MCSA,totalSample,immune-Effector_memory; ROSMAP,totalSample,immune-Effector_memory; ROSMAP,male-excludeAge90+,survival
RGS16	Homo_723,female,AD.vs.control; Homo_723,male,AD.vs.control; Homo_723,female,AD.vs.MCI; Homo_723,male,AD.vs.MCI; Homo_723,control,male.vs.female; Homo_714,female,AD.vs.control; Homo_714,male,AD.vs.control; Homo_714,female,AD.vs.MCI; Homo_714,male,AD.vs.MCI; Homo_633,female,AD.vs.control; Homo_633,female,AD.vs.MCI; Homo_633,female,MCI.vs.control; Homo_633,male,MCI.vs.control; MCSA,MCI,age; ROSMAP,AD,last.visit.age; Emory,totalSample,immune-CD4_T; Emory,totalSample,immune-Tr1; Emory,totalSample,immune-nTreg; Emory,totalSample,immune-iTreg; Emory,totalSample,immune-Tfh; Emory,totalSample,immune-CD8_naive; Emory,totalSample,immune-Central_memory; Homo_723,totalSample,immune-DC; Homo_723,totalSample,immune-Monocyte; Homo_723,totalSample,immune-Th1; Homo_723,totalSample,immune-Central_memory; Homo_723,totalSample,immune-Neutrophil; Homo_714,totalSample,immune-DC; Homo_714,totalSample,immune-Th1; Homo_633,totalSample,immune-Monocyte; Homo_633,totalSample,immune-DC; Homo_633,totalSample,immune-Th1; MCSA,totalSample,immune-DC; MCSA,totalSample,immune-Gamma_delta; ROSMAP,totalSample,immune-InfiltrationScore
HEXB	Homo_723,female,AD.vs.control; Homo_723,male,AD.vs.control; Homo_723,female,AD.vs.MCI; Homo_723,male,AD.vs.MCI; Homo_714,female,AD.vs.control; Homo_714,male,AD.vs.control; Homo_714,female,AD.vs.MCI; Homo_714,male,AD.vs.MCI; Homo_633,female,AD.vs.control; Homo_633,female,AD.vs.MCI; ROSMAP,male,AD.vs.control; Emory,MCI,age; MCSA,totalSample,age; MCSA,control,age; MCSA,MCI,age; ROSMAP,MCI,last.visit.age; ROSMAP,MCI,sample.age; Emory,totalSample,immune-Monocyte; Emory,totalSample,immune-Gamma_delta; Emory,totalSample,immune-Tr1; Emory,totalSample,immune-iTreg; Emory,totalSample,immune-Th1; Emory,totalSample,immune-CD8_naive; Homo_723,totalSample,immune-Monocyte; Homo_723,totalSample,immune-DC; Homo_723,totalSample,immune-Gamma_delta; Homo_723,totalSample,immune-Neutrophil; Homo_723,totalSample,immune-Bcell; Homo_723,totalSample,immune-Th2; Homo_723,totalSample,immune-InfiltrationScore; Homo_723,totalSample,immune-iTreg; Homo_714,totalSample,immune-Monocyte; Homo_714,totalSample,immune-Th2; Homo_714,totalSample,immune-Tfh; Homo_714,totalSample,immune-Gamma_delta; Homo_633,totalSample,immune-Monocyte; Homo_633,totalSample,immune-Th2; Homo_633,totalSample,immune-Bcell; Homo_633,totalSample,immune-Exhausted; Homo_633,totalSample,immune-Gamma_delta; Homo_633,totalSample,immune-CD8_T; MCSA,totalSample,immune-DC; MCSA,totalSample,immune-Monocyte; MCSA,totalSample,immune-Neutrophil; MCSA,totalSample,immune-CD8_T; MCSA,totalSample,immune-iTreg; MCSA,totalSample,immune-Th2; MCSA,totalSample,immune-Cytotoxic; MCSA,totalSample,immune-Exhausted; MCSA,totalSample,immune-Effector_memory; MCSA,totalSample,immune-InfiltrationScore; SRP223445,totalSample,immune-Neutrophil; SRP223445,totalSample,immune-CD4_naive; ROSMAP,female-includeAge90+,survival; ROSMAP,male-excludeAge90+,survival
TRAPPC3L	Homo_723,female,AD.vs.control; Homo_723,male,AD.vs.control; Homo_723,female,AD.vs.MCI; Homo_723,male,AD.vs.MCI; Homo_723,female,MCI.vs.control; Homo_723,male,MCI.vs.control; Homo_714,female,AD.vs.control; Homo_714,male,AD.vs.control; Homo_714,female,AD.vs.MCI; Homo_714,male,AD.vs.MCI; Homo_714,female,MCI.vs.control; Homo_714,male,MCI.vs.control; Homo_633,female,AD.vs.control; Homo_633,female,AD.vs.MCI; ROSMAP,female,AD.vs.control; MCSA,totalSample,age; MCSA,control,age; MCSA,MCI,age; ROSMAP,totalSample,age; ROSMAP,MCI,sample.age; ROSMAP,totalSample,MMSE; Emory,totalSample,immune-Th17; Emory,totalSample,immune-Exhausted; Homo_723,totalSample,immune-CD8_T; Homo_723,totalSample,immune-Monocyte; Homo_723,totalSample,immune-Exhausted; Homo_723,totalSample,immune-Macrophage; Homo_723,totalSample,immune-NK; Homo_714,totalSample,immune-Monocyte; Homo_714,totalSample,immune-Exhausted; Homo_714,totalSample,immune-CD8_T; Homo_633,totalSample,immune-Monocyte; MCSA,totalSample,immune-DC; MCSA,totalSample,immune-Monocyte; MCSA,totalSample,immune-Th2; MCSA,totalSample,immune-Th17; MCSA,totalSample,immune-Effector_memory; MCSA,totalSample,immune-InfiltrationScore; ROSMAP,totalSample,immune-CD4_T; ROSMAP,totalSample,immune-Th17;
ARHGAP18	Homo_723,female,AD.vs.control; Homo_723,male,AD.vs.control; Homo_723,female,AD.vs.MCI; Homo_723,male,AD.vs.MCI; Homo_723,female,MCI.vs.control; Homo_723,male,MCI.vs.control; Homo_714,female,AD.vs.control; Homo_714,male,AD.vs.control; Homo_714,female,AD.vs.MCI; Homo_714,male,AD.vs.MCI; Homo_714,female,MCI.vs.control; Homo_714,male,MCI.vs.control; Homo_633,female,AD.vs.control; Homo_633,female,AD.vs.MCI; MCSA,MCI,age; ROSMAP,MCI,last.visit.age; Homo_723,totalSample,immune-Exhausted; Homo_723,totalSample,immune-CD8_T; Homo_723,totalSample,immune-Cytotoxic; Homo_723,totalSample,immune-Th17; Homo_723,totalSample,immune-InfiltrationScore; Homo_723,totalSample,immune-CD4_naive; Homo_723,totalSample,immune-NK; Homo_723,totalSample,immune-Tfh; Homo_723,totalSample,immune-Central_memory; Homo_723,totalSample,immune-Gamma_delta; Homo_714,totalSample,immune-Exhausted; Homo_714,totalSample,immune-Monocyte; Homo_714,totalSample,immune-Th17; Homo_714,totalSample,immune-CD4_naive; Homo_714,totalSample,immune-InfiltrationScore; Homo_714,totalSample,immune-NKT; Homo_714,totalSample,immune-CD8_T; Homo_714,totalSample,immune-NK; Homo_633,totalSample,immune-Tfh; Homo_633,totalSample,immune-InfiltrationScore; Homo_633,totalSample,immune-Monocyte; Homo_633,totalSample,immune-CD4_T; Homo_633,totalSample,immune-CD4_naive; Homo_633,totalSample,immune-NKT; Homo_633,totalSample,immune-Th2; Homo_633,totalSample,immune-Effector_memory; Homo_633,totalSample,immune-Central_memory; Homo_633,totalSample,immune-Exhausted; Homo_633,totalSample,immune-Gamma_delta; Homo_633,totalSample,immune-Tr1; MCSA,totalSample,immune-DC; MCSA,totalSample,immune-Monocyte; MCSA,totalSample,immune-CD4_T; MCSA,totalSample,immune-CD8_T; MCSA,totalSample,immune-CD4_naive; MCSA,totalSample,immune-Tr1; MCSA,totalSample,immune-nTreg; MCSA,totalSample,immune-iTreg; MCSA,totalSample,immune-Tfh; MCSA,totalSample,immune-CD8_naive; MCSA,totalSample,immune-Cytotoxic; MCSA,totalSample,immune-Exhausted; MCSA,totalSample,immune-MAIT; MCSA,totalSample,immune-Central_memory; SRP223445,totalSample,immune-CD4_naive; SRP223445,totalSample,immune-Exhausted
BTBD3	Homo_723,female,AD.vs.control; Homo_723,male,AD.vs.control; Homo_723,female,AD.vs.MCI; Homo_723,male,AD.vs.MCI; Homo_723,female,MCI.vs.control; Homo_723,male,MCI.vs.control; Homo_714,female,AD.vs.control; Homo_714,male,AD.vs.control; Homo_714,female,AD.vs.MCI; Homo_714,male,AD.vs.MCI; Homo_714,female,MCI.vs.control; Homo_714,male,MCI.vs.control; Homo_633,female,AD.vs.control; Homo_633,female,AD.vs.MCI; ACOM,AD,age; Emory,control,age; MCSA,MCI,age; ROSMAP,AD,last.visit.age; ROSMAP,MCI,last.visit.age; ROSMAP,AD,MMSE; Emory,totalSample,immune-Gamma_delta; Emory,totalSample,immune-Tr1; Emory,totalSample,immune-iTreg; Emory,totalSample,immune-InfiltrationScore; Homo_723,totalSample,immune-Exhausted; Homo_723,totalSample,immune-Th17; Homo_723,totalSample,immune-Cytotoxic; Homo_723,totalSample,immune-CD8_T; Homo_723,totalSample,immune-CD4_naive; Homo_723,totalSample,immune-CD4_T; Homo_723,totalSample,immune-InfiltrationScore; Homo_723,totalSample,immune-Central_memory; Homo_723,totalSample,immune-Tfh; Homo_723,totalSample,immune-Tr1; Homo_723,totalSample,immune-Monocyte; Homo_723,totalSample,immune-Gamma_delta; Homo_714,totalSample,immune-Exhausted; Homo_714,totalSample,immune-Th17; Homo_714,totalSample,immune-Monocyte; Homo_714,totalSample,immune-CD4_naive; Homo_714,totalSample,immune-InfiltrationScore; Homo_714,totalSample,immune-CD8_T; Homo_714,totalSample,immune-Gamma_delta; Homo_714,totalSample,immune-Cytotoxic; Homo_714,totalSample,immune-Central_memory; Homo_714,totalSample,immune-NKT; Homo_633,totalSample,immune-Tfh; Homo_633,totalSample,immune-Exhausted; Homo_633,totalSample,immune-Th2; Homo_633,totalSample,immune-Gamma_delta; Homo_633,totalSample,immune-Monocyte; Homo_633,totalSample,immune-InfiltrationScore; Homo_633,totalSample,immune-CD4_T; Homo_633,totalSample,immune-Effector_memory; Homo_633,totalSample,immune-CD4_naive; Homo_633,totalSample,immune-Central_memory; Homo_633,totalSample,immune-NKT; Homo_633,totalSample,immune-Tr1; Homo_633,totalSample,immune-Th17; MCSA,totalSample,immune-Monocyte; MCSA,totalSample,immune-CD4_T; MCSA,totalSample,immune-CD8_T; MCSA,totalSample,immune-Gamma_delta; MCSA,totalSample,immune-CD4_naive; MCSA,totalSample,immune-Tr1; MCSA,totalSample,immune-nTreg; MCSA,totalSample,immune-iTreg; MCSA,totalSample,immune-Th17; MCSA,totalSample,immune-Tfh; MCSA,totalSample,immune-CD8_naive; MCSA,totalSample,immune-Cytotoxic; MCSA,totalSample,immune-Exhausted; MCSA,totalSample,immune-Central_memory; SRP223445,totalSample,immune-Neutrophil
ESRRA	Homo_723,female,AD.vs.control; Homo_723,male,AD.vs.control; Homo_723,female,AD.vs.MCI; Homo_723,male,AD.vs.MCI; Homo_714,female,AD.vs.control; Homo_714,male,AD.vs.control; Homo_714,female,AD.vs.MCI; Homo_714,male,AD.vs.MCI; Homo_633,female,AD.vs.control; Homo_633,female,AD.vs.MCI; ACOM,AD,age; Emory,totalSample,age; Emory,MCI,age; MCSA,totalSample,age; MCSA,control,age; MCSA,MCI,age; ROSMAP,MCI,last.visit.age; Emory,totalSample,immune-B_cell; Emory,totalSample,immune-Neutrophil; Emory,totalSample,immune-CD8_T; Emory,totalSample,immune-Gamma_delta; Emory,totalSample,immune-Tfh; Emory,totalSample,immune-CD8_naive; Emory,totalSample,immune-Exhausted; Homo_723,totalSample,immune-InfiltrationScore; Homo_723,totalSample,immune-Effector_memory; Homo_723,totalSample,immune-Th1; Homo_723,totalSample,immune-Cytotoxic; Homo_723,totalSample,immune-CD8_T; Homo_723,totalSample,immune-Bcell; Homo_714,totalSample,immune-Th1; Homo_714,totalSample,immune-Effector_memory; Homo_714,totalSample,immune-InfiltrationScore; Homo_714,totalSample,immune-DC; Homo_633,totalSample,immune-DC; Homo_633,totalSample,immune-InfiltrationScore; Homo_633,totalSample,immune-Th1; Homo_633,totalSample,immune-Effector_memory; Homo_633,totalSample,immune-Exhausted; Homo_633,totalSample,immune-nTreg; MCSA,totalSample,immune-DC; MCSA,totalSample,immune-Monocyte; MCSA,totalSample,immune-Macrophage; MCSA,totalSample,immune-Neutrophil; MCSA,totalSample,immune-CD8_T; MCSA,totalSample,immune-NKT; MCSA,totalSample,immune-Th2; MCSA,totalSample,immune-Cytotoxic; MCSA,totalSample,immune-Central_memory; MCSA,totalSample,immune-Effector_memory; MCSA,totalSample,immune-InfiltrationScore; SRP223445,totalSample,immune-CD4_naive; SRP223445,totalSample,immune-Exhausted; SRP223445,totalSample,immune-InfiltrationScore

SEPTIN11	Homo_723,female,AD.vs.control; Homo_723,male,AD.vs.control; Homo_723,female,AD.vs.MCI; Homo_723,male,AD.vs.MCI; Homo_723,female,MCI.vs.control; Homo_723,male,MCI.vs.control; Homo_714,female,AD.vs.control; Homo_714,male,AD.vs.control; Homo_714,female,AD.vs.MCI; Homo_714,male,AD.vs.MCI; Homo_714,female,MCI.vs.control; Homo_714,male,MCI.vs.control; Homo_633,female,AD.vs.control; Homo_633,female,AD.vs.MCI; ROSMAP,female,AD.vs.control; ROSMAP,female,AD.vs.MCI; MCSA,MCI,age; Emory,totalSample,immune-Macrophage; Emory,totalSample,immune-NK; Emory,totalSample,immune-Neutrophil; Emory,totalSample,immune-CD4_T; Emory,totalSample,immune-CD8_T; Emory,totalSample,immune-Tr1; Emory,totalSample,immune-iTreg; Emory,totalSample,immune-Tfh; Emory,totalSample,immune-Cytotoxic; Emory,totalSample,immune-Exhausted; Emory,totalSample,immune-Effector_memory; Emory,totalSample,immune-InfiltrationScore; Homo_723,totalSample,immune-CD4_naive; Homo_723,totalSample,immune-Th17; Homo_723,totalSample,immune-CD4_T; Homo_723,totalSample,immune-Exhausted; Homo_723,totalSample,immune-Tfh; Homo_723,totalSample,immune-InfiltrationScore; Homo_723,totalSample,immune-Neutrophil; Homo_723,totalSample,immune-Tr1; Homo_723,totalSample,immune-Central_memory; Homo_723,totalSample,immune-Cytotoxic; Homo_723,totalSample,immune-Th2; Homo_723,totalSample,immune-Th1; Homo_723,totalSample,immune-nTreg; Homo_723,totalSample,immune-CD8_T; Homo_723,totalSample,immune-NK; Homo_723,totalSample,immune-Effector_memory; Homo_714,totalSample,immune-CD4_naive; Homo_714,totalSample,immune-InfiltrationScore; Homo_714,totalSample,immune-Tfh; Homo_714,totalSample,immune-CD4_T; Homo_714,totalSample,immune-Central_memory; Homo_714,totalSample,immune-Th17; Homo_714,totalSample,immune-Neutrophil; Homo_714,totalSample,immune-NK; Homo_714,totalSample,immune-NKT; Homo_714,totalSample,immune-Effector_memory; Homo_633,totalSample,immune-Tfh; Homo_633,totalSample,immune-InfiltrationScore; Homo_633,totalSample,immune-CD4_T; Homo_633,totalSample,immune-CD4_naive; Homo_633,totalSample,immune-Effector_memory; Homo_633,totalSample,immune-Central_memory; Homo_633,totalSample,immune-Th2; Homo_633,totalSample,immune-Tr1; Homo_633,totalSample,immune-Bcell; Homo_633,totalSample,immune-iTreg; Homo_633,totalSample,immune-NKT; Homo_633,totalSample,immune-Neutrophil; Homo_633,totalSample,immune-MAIT; MCSA,totalSample,immune-DC; MCSA,totalSample,immune-Macrophage; MCSA,totalSample,immune-CD4_T; MCSA,totalSample,immune-CD4_naive; MCSA,totalSample,immune-Tr1; MCSA,totalSample,immune-nTreg; MCSA,totalSample,immune-iTreg; MCSA,totalSample,immune-Th2; MCSA,totalSample,immune-Th17; MCSA,totalSample,immune-Tfh; MCSA,totalSample,immune-CD8_naive; MCSA,totalSample,immune-Cytotoxic; MCSA,totalSample,immune-Exhausted; MCSA,totalSample,immune-MAIT; MCSA,totalSample,immune-[illegible]
DKK3	Homo_723,female,AD.vs.control; Homo_723,male,AD.vs.control; Homo_723,female,AD.vs.MCI; Homo_723,male,AD.vs.MCI; Homo_723,female,MCI.vs.control; Homo_714,female,AD.vs.control; Homo_714,male,AD.vs.control; Homo_714,female,AD.vs.MCI; Homo_714,male,AD.vs.MCI; Homo_633,female,AD.vs.control; Homo_633,female,AD.vs.MCI; ROSMAP,female,AD.vs.control; Emory,totalSample,age; Emory,control,age; Emory,MCI,age; MCSA,totalSample,age; MCSA,control,age; ROSMAP,MCI,last.visit.age; ROSMAP,MCI,sample.age; Emory,totalSample,immune-Neutrophil; Emory,totalSample,immune-CD4_T; Emory,totalSample,immune-CD8_T; Emory,totalSample,immune-CD8_naive; Emory,totalSample,immune-Exhausted; Emory,totalSample,immune-MAIT; Emory,totalSample,immune-InfiltrationScore; Homo_723,totalSample,immune-Neutrophil; Homo_723,totalSample,immune-CD4_naive; Homo_723,totalSample,immune-nTreg; Homo_723,totalSample,immune-CD4_T; Homo_723,totalSample,immune-Central_memory; Homo_723,totalSample,immune-Tfh; Homo_723,totalSample,immune-MAIT; Homo_723,totalSample,immune-Tr1; Homo_723,totalSample,immune-InfiltrationScore; Homo_714,totalSample,immune-InfiltrationScore; Homo_714,totalSample,immune-CD4_naive; Homo_714,totalSample,immune-Neutrophil; Homo_714,totalSample,immune-MAIT; Homo_714,totalSample,immune-Tfh; Homo_714,totalSample,immune-CD4_T; Homo_714,totalSample,immune-nTreg; Homo_633,totalSample,immune-InfiltrationScore; Homo_633,totalSample,immune-MAIT; Homo_633,totalSample,immune-Tfh; Homo_633,totalSample,immune-CD4_T; Homo_633,totalSample,immune-CD4_naive; Homo_633,totalSample,immune-nTreg; Homo_633,totalSample,immune-Neutrophil; Homo_633,totalSample,immune-Effector_memory; Homo_633,totalSample,immune-Central_memory; MCSA,totalSample,immune-Macrophage; MCSA,totalSample,immune-Neutrophil; MCSA,totalSample,immune-CD4_T; MCSA,totalSample,immune-CD8_T; MCSA,totalSample,immune-CD4_naive; MCSA,totalSample,immune-Tr1; MCSA,totalSample,immune-nTreg; MCSA,totalSample,immune-iTreg; MCSA,totalSample,immune-Th17; MCSA,totalSample,immune-Tfh; MCSA,totalSample,immune-CD8_naive; MCSA,totalSample,immune-MAIT; MCSA,totalSample,immune-Central_memory; MCSA,totalSample,immune-Effector_memory; ROSMAP,totalSample,immune-MAIT
WWC1	Homo_723,female,AD.vs.control; Homo_723,male,AD.vs.control; Homo_723,female,AD.vs.MCI; Homo_723,male,AD.vs.MCI; Homo_714,female,AD.vs.control; Homo_714,male,AD.vs.control; Homo_714,female,AD.vs.MCI; Homo_714,male,AD.vs.MCI; Homo_633,female,AD.vs.control; Homo_633,female,AD.vs.MCI; Emory,MCI,age; MCSA,totalSample,age; MCSA,control,age; MCSA,MCI,age; Emory,totalSample,immune-CD8_T; Emory,totalSample,immune-Gamma_delta; Emory,totalSample,immune-Th17; Homo_723,totalSample,immune-Th17; Homo_723,totalSample,immune-Gamma_delta; Homo_723,totalSample,immune-nTreg; Homo_714,totalSample,immune-Gamma_delta; Homo_714,totalSample,immune-Th17; Homo_633,totalSample,immune-Gamma_delta; Homo_633,totalSample,immune-Tfh; Homo_633,totalSample,immune-nTreg; MCSA,totalSample,immune-Monocyte; MCSA,totalSample,immune-CD4_T; MCSA,totalSample,immune-Gamma_delta; MCSA,totalSample,immune-CD4_naive; MCSA,totalSample,immune-Tr1; MCSA,totalSample,immune-nTreg; MCSA,totalSample,immune-iTreg; MCSA,totalSample,immune-Th17; MCSA,totalSample,immune-Tfh; MCSA,totalSample,immune-Central_memory; ROSMAP,totalSample,immune-InfiltrationScore
KLRG1	Homo_723,female,AD.vs.control; Homo_723,male,AD.vs.control; Homo_723,female,AD.vs.MCI; Homo_723,male,AD.vs.MCI; Homo_714,female,AD.vs.control; Homo_714,male,AD.vs.control; Homo_714,female,AD.vs.MCI; Homo_714,male,AD.vs.MCI; Homo_633,female,AD.vs.control; Homo_633,female,AD.vs.MCI; ROSMAP,male,AD.vs.MCI; ROSMAP,MCI,male.vs.female; Emory,totalSample,immune-Macrophage; Emory,totalSample,immune-NK; Emory,totalSample,immune-Neutrophil; Emory,totalSample,immune-CD4_T; Emory,totalSample,immune-CD8_T; Emory,totalSample,immune-Gamma_delta; Emory,totalSample,immune-CD4_naive; Emory,totalSample,immune-Tr1; Emory,totalSample,immune-iTreg; Emory,totalSample,immune-Cytotoxic; Emory,totalSample,immune-Exhausted; Emory,totalSample,immune-Effector_memory; Emory,totalSample,immune-InfiltrationScore; Homo_723,totalSample,immune-MAIT; Homo_723,totalSample,immune-InfiltrationScore; Homo_723,totalSample,immune-Effector_memory; Homo_723,totalSample,immune-Cytotoxic; Homo_723,totalSample,immune-Tfh; Homo_723,totalSample,immune-CD8_T; Homo_723,totalSample,immune-Macrophage; Homo_723,totalSample,immune-Exhausted; Homo_723,totalSample,immune-Bcell; Homo_723,totalSample,immune-iTreg; Homo_723,totalSample,immune-CD4_naive; Homo_723,totalSample,immune-Th17; Homo_723,totalSample,immune-CD4_T; Homo_714,totalSample,immune-InfiltrationScore; Homo_714,totalSample,immune-MAIT; Homo_714,totalSample,immune-Cytotoxic; Homo_714,totalSample,immune-Effector_memory; Homo_714,totalSample,immune-CD8_T; Homo_714,totalSample,immune-Tfh; Homo_714,totalSample,immune-Macrophage; Homo_714,totalSample,immune-Exhausted; Homo_714,totalSample,immune-Bcell; Homo_714,totalSample,immune-Neutrophil; Homo_714,totalSample,immune-Gamma_delta; Homo_714,totalSample,immune-CD4_naive; Homo_714,totalSample,immune-Th17; Homo_714,totalSample,immune-CD8_naive; Homo_633,totalSample,immune-InfiltrationScore; Homo_633,totalSample,immune-MAIT; Homo_633,totalSample,immune-Cytotoxic; Homo_633,totalSample,immune-Effector_memory; Homo_633,totalSample,immune-CD8_T; Homo_633,totalSample,immune-Neutrophil; Homo_633,totalSample,immune-Bcell; Homo_633,totalSample,immune-Gamma_delta; Homo_633,totalSample,immune-Macrophage; Homo_633,totalSample,immune-CD4_naive; Homo_633,totalSample,immune-Tfh; MCSA,totalSample,immune-DC; MCSA,totalSample,immune-Macrophage; MCSA,totalSample,immune-CD4_T; MCSA,totalSample,immune-Gamma_delta; MCSA,totalSample,immune-Tr1; MCSA,totalSample,immune-iTreg; MCSA,totalSample,immune-Tfh; MCSA,totalSample,immune-CD8_naive; MCSA,totalSample,immune-MAIT; MCSA,totalSample,immune-Effector_memory; ROSMAP,totalSample,immune-Bcell; ROSMAP,totalSample,immune-CD4_T; ROSMAP,totalSample,immune-Gamma_delta; ROSMAP,totalSample,immune-Tr1; ROSMAP,totalSample,immune-nTreg; ROSMAP,totalSample,immune-iTreg; ROSMAP,totalSample,immune-Th1; ROSMAP,totalSample,immune-Th2; ROSMAP,totalSample,immune-Th17; ROSMAP,totalSample,immune-Tfh; ROSMAP,totalSample,immune-InfiltrationScore; SRP223445,totalSample,immune-Neutrophil; SRP223445,totalSample,immune-CD8_T; SRP223445,totalSample,immune-Gamma_delta; SRP223445,totalSample,immune-Cytotoxic; SRP223445,totalSample,immune-MAIT
NCR3	Homo_723,female,AD.vs.control; Homo_723,male,AD.vs.control; Homo_723,female,AD.vs.MCI; Homo_723,male,AD.vs.MCI; Homo_714,female,AD.vs.control; Homo_714,male,AD.vs.control; Homo_714,female,AD.vs.MCI; Homo_714,male,AD.vs.MCI; Homo_633,female,AD.vs.control; Homo_633,female,AD.vs.MCI; ROSMAP,female,AD.vs.control; ROSMAP,female,MCI.vs.control; MCSA,totalSample,age; MCSA,control,age; ROSMAP,MCI,last.visit.age; Emory,totalSample,immune-Macrophage; Emory,totalSample,immune-NK; Emory,totalSample,immune-Neutrophil; Emory,totalSample,immune-CD4_T; Emory,totalSample,immune-CD8_T; Emory,totalSample,immune-Gamma_delta; Emory,totalSample,immune-CD4_naive; Emory,totalSample,immune-Tr1; Emory,totalSample,immune-iTreg; Emory,totalSample,immune-Tfh; Emory,totalSample,immune-Cytotoxic; Emory,totalSample,immune-Exhausted; Emory,totalSample,immune-Effector_memory; Emory,totalSample,immune-InfiltrationScore; Homo_723,totalSample,immune-InfiltrationScore; Homo_723,totalSample,immune-Cytotoxic; Homo_723,totalSample,immune-CD8_T; Homo_723,totalSample,immune-Effector_memory; Homo_723,totalSample,immune-Macrophage; Homo_723,totalSample,immune-Exhausted; Homo_723,totalSample,immune-Tfh; Homo_723,totalSample,immune-Monocyte; Homo_723,totalSample,immune-MAIT; Homo_723,totalSample,immune-iTreg; Homo_723,totalSample,immune-nTreg; Homo_723,totalSample,immune-Neutrophil; Homo_714,totalSample,immune-InfiltrationScore; Homo_714,totalSample,immune-Effector_memory; Homo_714,totalSample,immune-CD8_T; Homo_714,totalSample,immune-Macrophage; Homo_714,totalSample,immune-Cytotoxic; Homo_714,totalSample,immune-Neutrophil; Homo_714,totalSample,immune-Monocyte; Homo_714,totalSample,immune-MAIT; Homo_714,totalSample,immune-Tfh; Homo_714,totalSample,immune-nTreg; Homo_714,totalSample,immune-Exhausted; Homo_633,totalSample,immune-InfiltrationScore; Homo_633,totalSample,immune-Effector_memory; Homo_633,totalSample,immune-Neutrophil; Homo_633,totalSample,immune-Macrophage; Homo_633,totalSample,immune-CD8_T; Homo_633,totalSample,immune-NK; Homo_633,totalSample,immune-Cytotoxic; Homo_633,totalSample,immune-nTreg; Homo_633,totalSample,immune-Monocyte; Homo_633,totalSample,immune-DC; Homo_633,totalSample,immune-Gamma_delta; Homo_633,totalSample,immune-MAIT; MCSA,totalSample,immune-DC; MCSA,totalSample,immune-Monocyte; MCSA,totalSample,immune-Neutrophil; MCSA,totalSample,immune-CD8_T; MCSA,totalSample,immune-Gamma_delta; MCSA,totalSample,immune-nTreg; MCSA,totalSample,immune-Tfh; MCSA,totalSample,immune-Cytotoxic; MCSA,totalSample,immune-Exhausted; MCSA,totalSample,immune-MAIT; MCSA,totalSample,immune-Effector_memory; MCSA,totalSample,immune-InfiltrationScore; ROSMAP,totalSample,immune-NK; ROSMAP,totalSample,immune-CD4_T; ROSMAP,totalSample,immune-Th17; ROSMAP,totalSample,immune-InfiltrationScore; SRP223445,totalSample,immune-Neutrophil; SRP223445,totalSample,immune-[illegible]
CHRNA10	Homo_723,female,AD.vs.control; Homo_723,male,AD.vs.control; Homo_723,female,AD.vs.MCI; Homo_723,male,AD.vs.MCI; Homo_714,female,AD.vs.control; Homo_714,male,AD.vs.control; Homo_714,female,AD.vs.MCI; Homo_714,male,AD.vs.MCI; Homo_633,female,AD.vs.control; Homo_633,female,AD.vs.MCI; MCSA,totalSample,age; MCSA,control,age; MCSA,MCI,age; Emory,totalSample,immune-Neutrophil; Emory,totalSample,immune-Gamma_delta; Emory,totalSample,immune-Th17; Homo_723,totalSample,immune-Th2; Homo_723,totalSample,immune-Th1; Homo_723,totalSample,immune-InfiltrationScore; Homo_723,totalSample,immune-Tfh; Homo_723,totalSample,immune-Effector_memory; Homo_723,totalSample,immune-Monocyte; Homo_723,totalSample,immune-Bcell; Homo_723,totalSample,immune-Neutrophil; Homo_723,totalSample,immune-iTreg; Homo_723,totalSample,immune-Gamma_delta; Homo_714,totalSample,immune-Th2; Homo_714,totalSample,immune-Th1; Homo_714,totalSample,immune-Tfh; Homo_714,totalSample,immune-Gamma_delta; Homo_633,totalSample,immune-Th2; Homo_633,totalSample,immune-Exhausted; Homo_633,totalSample,immune-Th17; Homo_633,totalSample,immune-Th1; Homo_633,totalSample,immune-Gamma_delta; Homo_633,totalSample,immune-InfiltrationScore; MCSA,totalSample,immune-DC; MCSA,totalSample,immune-Monocyte; MCSA,totalSample,immune-Gamma_delta;
AFAP1	Homo_723,female,AD.vs.control; Homo_723,male,AD.vs.control; Homo_723,female,AD.vs.MCI; Homo_723,male,AD.vs.MCI; Homo_723,female,MCI.vs.control; Homo_714,female,AD.vs.control; Homo_714,male,AD.vs.control; Homo_714,female,AD.vs.MCI; Homo_714,male,AD.vs.MCI; Homo_714,female,MCI.vs.control; Homo_633,female,AD.vs.control; Homo_633,female,AD.vs.MCI; ROSMAP,female,AD.vs.MCI; ROSMAP,female,MCI.vs.control; Emory,MCI,age; ROSMAP,control,last.visit.age; Emory,totalSample,immune-B_cell; Emory,totalSample,immune-Tr1; Emory,totalSample,immune-iTreg; Homo_723,totalSample,immune-DC; Homo_723,totalSample,immune-Th17; Homo_723,totalSample,immune-CD4_naive; Homo_723,totalSample,immune-nTreg; Homo_723,totalSample,immune-Central_memory; Homo_714,totalSample,immune-InfiltrationScore; Homo_714,totalSample,immune-DC; Homo_714,totalSample,immune-CD4_naive; Homo_714,totalSample,immune-Th17; Homo_633,totalSample,immune-InfiltrationScore; Homo_633,totalSample,immune-Tfh; Homo_633,totalSample,immune-nTreg; Homo_633,totalSample,immune-Central_memory; Homo_633,totalSample,immune-CD4_T; Homo_633,totalSample,immune-CD4_naive; Homo_633,totalSample,immune-Tr1; MCSA,totalSample,immune-Monocyte; MCSA,totalSample,immune-CD4_T; MCSA,totalSample,immune-CD4_naive; MCSA,totalSample,immune-Tr1; MCSA,totalSample,immune-nTreg; MCSA,totalSample,immune-Th17; MCSA,totalSample,immune-Tfh; SRP223445,totalSample,immune-Exhausted
DEFA1	Homo_723,female,AD.vs.control; Homo_723,male,AD.vs.control; Homo_723,female,AD.vs.MCI; Homo_723,male,AD.vs.MCI; Homo_723,control,male.vs.female; Homo_714,female,AD.vs.MCI; Homo_714,control,male.vs.female; Homo_633,control,male.vs.female; ROSMAP,MCI,last.visit.age; ROSMAP,MCI,sample.age; Emory,totalSample,immune-Macrophage; Emory,totalSample,immune-NK; Emory,totalSample,immune-CD4_T; Emory,totalSample,immune-NKT; Emory,totalSample,immune-nTreg; Emory,totalSample,immune-Tfh; Homo_723,totalSample,immune-Th1; Homo_723,totalSample,immune-Monocyte; Homo_723,totalSample,immune-Macrophage; Homo_723,totalSample,immune-Cytotoxic; Homo_714,totalSample,immune-Macrophage; Homo_714,totalSample,immune-Th2; Homo_714,totalSample,immune-CD4_T; Homo_714,totalSample,immune-Central_memory; Homo_714,totalSample,immune-Tr1; Homo_633,totalSample,immune-Macrophage; Homo_633,totalSample,immune-Th2; Homo_633,totalSample,immune-CD4_T; MCSA,totalSample,immune-Macrophage; MCSA,totalSample,immune-CD4_T; MCSA,totalSample,immune-NKT; MCSA,totalSample,immune-CD4_naive; MCSA,totalSample,immune-Tr1; MCSA,totalSample,immune-nTreg; MCSA,totalSample,immune-iTreg; MCSA,totalSample,immune-Th2; MCSA,totalSample,immune-Tfh; MCSA,totalSample,immune-Central_memory;
MEIS1	Homo_723,female,AD.vs.control; Homo_723,male,AD.vs.control; Homo_723,female,AD.vs.MCI; Homo_723,male,AD.vs.MCI; Homo_723,female,MCI.vs.control; Homo_723,male,MCI.vs.control; Homo_714,female,AD.vs.control; Homo_714,male,AD.vs.control; Homo_714,female,AD.vs.MCI; Homo_714,male,AD.vs.MCI; Homo_714,female,MCI.vs.control; Homo_714,male,MCI.vs.control; Homo_633,female,AD.vs.control; Homo_633,female,AD.vs.MCI; ROSMAP,AD,last.visit.age; Homo_723,totalSample,immune-Th17; Homo_723,totalSample,immune-Exhausted; Homo_723,totalSample,immune-CD8_T; Homo_723,totalSample,immune-Cytotoxic; Homo_723,totalSample,immune-DC; Homo_723,totalSample,immune-CD4_naive; Homo_714,totalSample,immune-Th17; Homo_714,totalSample,immune-Exhausted; Homo_714,totalSample,immune-CD8_T; Homo_714,totalSample,immune-InfiltrationScore; Homo_633,totalSample,immune-Tfh; Homo_633,totalSample,immune-InfiltrationScore; Homo_633,totalSample,immune-Gamma_delta; Homo_633,totalSample,immune-Th17; Homo_633,totalSample,immune-Th2; Homo_633,totalSample,immune-CD4_T; Homo_633,totalSample,immune-Tr1; Homo_633,totalSample,immune-NKT; MCSA,totalSample,immune-CD4_T; MCSA,totalSample,immune-Tr1; MCSA,totalSample,immune-iTreg; MCSA,totalSample,immune-Th17; MCSA,totalSample,immune-Cytotoxic; MCSA,totalSample,immune-Exhausted; ROSMAP,totalSample,immune-InfiltrationScore; SRP223445,totalSample,immune-CD4_naive; SRP223445,totalSample,immune-Exhausted
TRO	Homo_723,female,AD.vs.control; Homo_723,male,AD.vs.control; Homo_723,female,AD.vs.MCI; Homo_723,female,MCI.vs.control; Homo_714,female,AD.vs.control; Homo_714,male,AD.vs.control; Homo_714,male,AD.vs.MCI; Homo_633,female,AD.vs.control; Homo_633,female,AD.vs.MCI; MCSA,totalSample,age; MCSA,control,age; Homo_723,totalSample,immune-Tfh; Homo_723,totalSample,immune-Effector_memory; Homo_723,totalSample,immune-InfiltrationScore; Homo_723,totalSample,immune-nTreg; Homo_723,totalSample,immune-CD4_T; Homo_723,totalSample,immune-CD4_naive; Homo_723,totalSample,immune-Th2; Homo_723,totalSample,immune-iTreg; Homo_723,totalSample,immune-Tr1; Homo_723,totalSample,immune-Central_memory; Homo_723,totalSample,immune-Macrophage; Homo_714,totalSample,immune-Effector_memory; Homo_714,totalSample,immune-InfiltrationScore; Homo_714,totalSample,immune-Tfh; Homo_714,totalSample,immune-nTreg; Homo_714,totalSample,immune-CD4_naive; Homo_714,totalSample,immune-CD4_T; Homo_714,totalSample,immune-Central_memory; Homo_714,totalSample,immune-Th2; Homo_714,totalSample,immune-Tr1; Homo_714,totalSample,immune-Macrophage; Homo_714,totalSample,immune-iTreg; Homo_633,totalSample,immune-InfiltrationScore; Homo_633,totalSample,immune-Effector_memory; Homo_633,totalSample,immune-Tfh; Homo_633,totalSample,immune-nTreg; Homo_633,totalSample,immune-CD4_T; Homo_633,totalSample,immune-CD4_naive; Homo_633,totalSample,immune-Central_memory; Homo_633,totalSample,immune-Tr1; Homo_633,totalSample,immune-Th2; Homo_633,totalSample,immune-Neutrophil; MCSA,totalSample,immune-Monocyte; MCSA,totalSample,immune-Neutrophil; MCSA,totalSample,immune-CD4_T; MCSA,totalSample,immune-CD8_T; MCSA,totalSample,immune-NKT; MCSA,totalSample,immune-CD4_naive; MCSA,totalSample,immune-Tr1; MCSA,totalSample,immune-nTreg; MCSA,totalSample,immune-iTreg; MCSA,totalSample,immune-Th2; MCSA,totalSample,immune-Th17; MCSA,totalSample,immune-Tfh;
CDK2AP1	Homo_723,female,AD.vs.control; Homo_723,male,AD.vs.control; Homo_723,female,AD.vs.MCI; Homo_723,male,AD.vs.MCI; Homo_714,female,AD.vs.control; Homo_714,male,AD.vs.control; Homo_714,female,AD.vs.MCI; Homo_714,male,AD.vs.MCI; Homo_633,female,AD.vs.control; Homo_633,female,AD.vs.MCI; ROSMAP,MCI,last.visit.age; ROSMAP,MCI,sample.age; Emory,totalSample,immune-Monocyte; Homo_723,totalSample,immune-DC; Homo_723,totalSample,immune-InfiltrationScore; Homo_723,totalSample,immune-NK; Homo_723,totalSample,immune-CD8_T; Homo_714,totalSample,immune-DC; Homo_714,totalSample,immune-Exhausted; Homo_714,totalSample,immune-NK; Homo_714,totalSample,immune-InfiltrationScore; Homo_714,totalSample,immune-CD8_T; Homo_714,totalSample,immune-NKT; Homo_714,totalSample,immune-Th2; Homo_633,totalSample,immune-InfiltrationScore; Homo_633,totalSample,immune-NKT; Homo_633,totalSample,immune-NK; MCSA,totalSample,immune-Monocyte; MCSA,totalSample,immune-NK; MCSA,totalSample,immune-iTreg; MCSA,totalSample,immune-Exhausted; ROSMAP,totalSample,immune-InfiltrationScore; SRP223445,totalSample,immune-CD4_naive; SRP223445,totalSample,immune-Exhausted
SURF1	Homo_723,female,AD.vs.control; Homo_723,male,AD.vs.control; Homo_723,female,AD.vs.MCI; Homo_723,male,AD.vs.MCI; Homo_714,female,AD.vs.control; Homo_714,male,AD.vs.control; Homo_714,female,AD.vs.MCI; Homo_714,male,AD.vs.MCI; Homo_633,female,AD.vs.control; Homo_633,female,AD.vs.MCI; ROSMAP,female,AD.vs.control; MCSA,totalSample,age; MCSA,control,age; ROSMAP,MCI,sample.age; Emory,totalSample,immune-Tr1; Emory,totalSample,immune-Effector_memory; Homo_723,totalSample,immune-CD8_T; Homo_723,totalSample,immune-InfiltrationScore; Homo_723,totalSample,immune-Exhausted; Homo_723,totalSample,immune-Cytotoxic; Homo_723,totalSample,immune-iTreg; Homo_714,totalSample,immune-InfiltrationScore; Homo_633,totalSample,immune-InfiltrationScore; Homo_633,totalSample,immune-NK; MCSA,totalSample,immune-DC; MCSA,totalSample,immune-Monocyte; MCSA,totalSample,immune-NKT; MCSA,totalSample,immune-CD8_naive; MCSA,totalSample,immune-InfiltrationScore; ROSMAP,totalSample,immune-[illegible]
NRXN1	Homo_723,female,AD.vs.control; Homo_723,male,AD.vs.control; Homo_723,female,AD.vs.MCI; Homo_723,male,AD.vs.MCI; Homo_714,female,AD.vs.control; Homo_714,male,AD.vs.control; Homo_714,female,AD.vs.MCI; Homo_633,female,AD.vs.control; Homo_633,female,AD.vs.MCI; Homo_633,female,MCI.vs.control; ROSMAP,male,AD.vs.control; ACOM,totalSample,age; Emory,MCI,age; Emory,totalSample,immune-Neutrophil; Emory,totalSample,immune-CD8_T; Emory,totalSample,immune-Gamma_delta; Emory,totalSample,immune-Th17; Emory,totalSample,immune-Tfh; Emory,totalSample,immune-Cytotoxic; Emory,totalSample,immune-Exhausted; Homo_723,totalSample,immune-Cytotoxic; Homo_714,totalSample,immune-Exhausted; Homo_714,totalSample,immune-CD8_T; Homo_633,totalSample,immune-Neutrophil; Homo_633,totalSample,immune-CD8_T; MCSA,totalSample,immune-DC; MCSA,totalSample,immune-Neutrophil; MCSA,totalSample,immune-CD8_T; MCSA,totalSample,immune-iTreg; MCSA,totalSample,immune-Th17; MCSA,totalSample,immune-Cytotoxic; MCSA,totalSample,immune-Exhausted; MCSA,totalSample,immune-InfiltrationScore; ROSMAP,totalSample,immune-InfiltrationScore; ROSMAP,male and female-excludeAge90+,survival; ROSMAP,male and female-includeAge90+,survival
ARHGEF18	Homo_723,female,AD.vs.control; Homo_723,male,AD.vs.control; Homo_723,male,AD.vs.MCI; Homo_723,female,MCI.vs.control; Homo_723,MCI,male.vs.female; Homo_714,female,AD.vs.control; Homo_714,male,AD.vs.control; Homo_714,male,AD.vs.MCI; Homo_714,female,MCI.vs.control; Homo_714,control,male.vs.female; Homo_714,MCI,male.vs.female; Homo_633,female,AD.vs.control; Homo_633,female,AD.vs.MCI; Homo_633,female,MCI.vs.control; Homo_633,control,male.vs.female; Homo_633,MCI,male.vs.female; MCSA,totalSample,age; MCSA,control,age; MCSA,MCI,age; Emory,totalSample,immune-Monocyte; Emory,totalSample,immune-CD4_T; Emory,totalSample,immune-Gamma_delta; Emory,totalSample,immune-CD4_naive; Emory,totalSample,immune-Tr1; Emory,totalSample,immune-nTreg; Emory,totalSample,immune-iTreg; Emory,totalSample,immune-Th17; Emory,totalSample,immune-Cytotoxic; Emory,totalSample,immune-Central_memory; Emory,totalSample,immune-InfiltrationScore; Homo_723,totalSample,immune-Monocyte; Homo_723,totalSample,immune-Central_memory; Homo_723,totalSample,immune-nTreg; Homo_723,totalSample,immune-DC; Homo_723,totalSample,immune-CD4_naive; Homo_723,totalSample,immune-CD4_T; Homo_723,totalSample,immune-Tfh; Homo_714,totalSample,immune-Monocyte; Homo_714,totalSample,immune-nTreg; Homo_714,totalSample,immune-CD4_naive; Homo_714,totalSample,immune-DC; Homo_714,totalSample,immune-Central_memory; Homo_633,totalSample,immune-Monocyte; Homo_633,totalSample,immune-nTreg; Homo_633,totalSample,immune-Tfh; Homo_633,totalSample,immune-CD4_naive; Homo_633,totalSample,immune-iTreg; Homo_633,totalSample,immune-Central_memory; Homo_633,totalSample,immune-CD4_T; Homo_633,totalSample,immune-Tr1; MCSA,totalSample,immune-DC; MCSA,totalSample,immune-Monocyte; MCSA,totalSample,immune-Neutrophil; MCSA,totalSample,immune-CD4_T; MCSA,totalSample,immune-CD8_T; MCSA,totalSample,immune-NKT; MCSA,totalSample,immune-Gamma_delta; MCSA,totalSample,immune-CD4_naive; MCSA,totalSample,immune-Tr1; MCSA,totalSample,immune-nTreg; MCSA,totalSample,immune-Th2; MCSA,totalSample,immune-Th17; MCSA,totalSample,immune-Tfh; MCSA,totalSample,immune-Cytotoxic; MCSA,totalSample,immune-Central_memory; MCSA,totalSample,immune-Effector_memory; MCSA,totalSample,immune-InfiltrationScore; [illegible]
FXYD6	Homo_723,female,AD.vs.control; Homo_723,male,AD.vs.control; Homo_723,female,AD.vs.MCI; Homo_723,male,AD.vs.MCI; Homo_714,female,AD.vs.control; Homo_714,male,AD.vs.control; Homo_633,female,AD.vs.control; Homo_633,female,AD.vs.MCI; Homo_633,female,MCI.vs.control; ACOM,totalSample,age; Emory,MCI,age; MCSA,MCI,age; ROSMAP,MCI,last.visit.age; ROSMAP,MCI,sample.age; Emory,totalSample,immune-Macrophage; Emory,totalSample,immune-CD8_T; Emory,totalSample,immune-Gamma_delta; Emory,totalSample,immune-Tfh; Emory,totalSample,immune-CD8_naive; Emory,totalSample,immune-Cytotoxic; Homo_723,totalSample,immune-DC; Homo_723,totalSample,immune-Th2; Homo_723,totalSample,immune-Macrophage; Homo_723,totalSample,immune-Monocyte; Homo_714,totalSample,immune-Th2; Homo_714,totalSample,immune-DC; Homo_633,totalSample,immune-DC; Homo_633,totalSample,immune-Th2; MCSA,totalSample,immune-DC; MCSA,totalSample,immune-Macrophage; MCSA,totalSample,immune-CD4_T; MCSA,totalSample,immune-Tr1; MCSA,totalSample,immune-iTreg; MCSA,totalSample,immune-CD8_naive; SRP223445,totalSample,immune-CD4_naive; SRP223445,totalSample,immune-Exhausted; SRP223445,totalSample,immune-InfiltrationScore

RAMAC	Homo_723,female,AD.vs.control; Homo_723,male,AD.vs.control; Homo_723,female,AD.vs.MCI; Homo_723,male,AD.vs.MCI; Homo_714,female,AD.vs.control; Homo_714,male,AD.vs.control; Homo_714,female,AD.vs.MCI; Homo_714,male,AD.vs.MCI; Homo_633,female,AD.vs.control; Homo_633,female,AD.vs.MCI; ROSMAP,female,AD.vs.control; Emory,MCI,age; MCSA,totalSample,age; MCSA,control,age; MCSA,MCI,age; ROSMAP,AD,sample.age; ROSMAP,MCI,last.visit.age; Emory,totalSample,immune-DC; Emory,totalSample,immune-Monocyte; Emory,totalSample,immune-Gamma_delta; Emory,totalSample,immune-Tr1; Emory,totalSample,immune-nTreg; Homo_723,totalSample,immune-Monocyte; Homo_723,totalSample,immune-DC; Homo_723,totalSample,immune-Th2; Homo_723,totalSample,immune-Tfh; Homo_723,totalSample,immune-InfiltrationScore; Homo_723,totalSample,immune-iTreg; Homo_723,totalSample,immune-Effector_memory; Homo_723,totalSample,immune-Bcell; Homo_714,totalSample,immune-Monocyte; Homo_714,totalSample,immune-Th2; Homo_714,totalSample,immune-Tfh; Homo_633,totalSample,immune-Monocyte; Homo_633,totalSample,immune-Th2; Homo_633,totalSample,immune-Exhausted; Homo_633,totalSample,immune-Tfh; Homo_633,totalSample,immune-InfiltrationScore; Homo_633,totalSample,immune-Th17; Homo_633,totalSample,immune-Gamma_delta; MCSA,totalSample,immune-DC; MCSA,totalSample,immune-Monocyte; MCSA,totalSample,immune-Macrophage; MCSA,totalSample,immune-Neutrophil; MCSA,totalSample,immune-CD8_T; MCSA,totalSample,immune-iTreg; MCSA,totalSample,immune-Th2; MCSA,totalSample,immune-Cytotoxic; MCSA,totalSample,immune-Exhausted; MCSA,totalSample,immune-MAIT; MCSA,totalSample,immune-[illegible]
ANKRD28	Homo_723,female,AD.vs.control; Homo_723,male,AD.vs.control; Homo_723,female,AD.vs.MCI; Homo_723,male,AD.vs.MCI; Homo_723,female,MCI.vs.control; Homo_723,male,MCI.vs.control; Homo_714,female,AD.vs.control; Homo_714,male,AD.vs.control; Homo_714,female,AD.vs.MCI; Homo_714,male,AD.vs.MCI; Homo_714,female,MCI.vs.control; Homo_714,male,MCI.vs.control; Homo_633,female,AD.vs.control; Homo_633,female,AD.vs.MCI; MCSA,MCI,age; Emory,totalSample,immune-DC; Emory,totalSample,immune-Macrophage; Emory,totalSample,immune-CD4_T; Emory,totalSample,immune-Tr1; Emory,totalSample,immune-iTreg; Emory,totalSample,immune-Central_memory; Emory,totalSample,immune-InfiltrationScore; Homo_723,totalSample,immune-Exhausted; Homo_723,totalSample,immune-Th17; Homo_723,totalSample,immune-CD4_naive; Homo_723,totalSample,immune-Cytotoxic; Homo_723,totalSample,immune-CD4_T; Homo_723,totalSample,immune-Tfh; Homo_723,totalSample,immune-CD8_T; Homo_723,totalSample,immune-Tr1; Homo_723,totalSample,immune-Central_memory; Homo_723,totalSample,immune-Th2; Homo_723,totalSample,immune-Th1; Homo_723,totalSample,immune-nTreg; Homo_723,totalSample,immune-InfiltrationScore; Homo_714,totalSample,immune-CD4_naive; Homo_714,totalSample,immune-Th17; Homo_714,totalSample,immune-CD4_T; Homo_714,totalSample,immune-Exhausted; Homo_714,totalSample,immune-Tfh; Homo_714,totalSample,immune-Central_memory; Homo_714,totalSample,immune-Monocyte; Homo_714,totalSample,immune-NKT; Homo_714,totalSample,immune-InfiltrationScore; Homo_714,totalSample,immune-Tr1; Homo_714,totalSample,immune-Gamma_delta; Homo_714,totalSample,immune-Th2; Homo_714,totalSample,immune-Cytotoxic; Homo_633,totalSample,immune-Tfh; Homo_633,totalSample,immune-Th2; Homo_633,totalSample,immune-CD4_T; Homo_633,totalSample,immune-CD4_naive; Homo_633,totalSample,immune-Central_memory; Homo_633,totalSample,immune-Effector_memory; Homo_633,totalSample,immune-Tr1; Homo_633,totalSample,immune-NKT; Homo_633,totalSample,immune-InfiltrationScore; Homo_633,totalSample,immune-Gamma_delta; Homo_633,totalSample,immune-Th17; Homo_633,totalSample,immune-Monocyte; Homo_633,totalSample,immune-iTreg; Homo_633,totalSample,immune-Exhausted; Homo_633,totalSample,immune-DC; Homo_633,totalSample,immune-Bcell; MCSA,totalSample,immune-DC; MCSA,totalSample,immune-Monocyte; MCSA,totalSample,immune-Macrophage; MCSA,totalSample,immune-CD4_T; MCSA,totalSample,immune-CD8_T; MCSA,totalSample,immune-CD4_naive; MCSA,totalSample,immune-Tr1; MCSA,totalSample,immune-nTreg; MCSA,totalSample,immune-iTreg; MCSA,totalSample,immune-Th17; MCSA,totalSample,immune-Tfh; MCSA,totalSample,immune-CD8_naive; MCSA,totalSample,immune-Cytotoxic; MCSA,totalSample,immune-Exhausted; MCSA,totalSample,immune-MAIT; MCSA,totalSample,immune-Central_memory; SRP223445,totalSample,immune-CD4_naive; SRP223445,totalSample,immune-Exhausted
LAMB1	Homo_723,female,AD.vs.control; Homo_723,male,AD.vs.control; Homo_723,female,AD.vs.MCI; Homo_723,male,AD.vs.MCI; Homo_723,female,MCI.vs.control; Homo_723,male,MCI.vs.control; Homo_714,female,AD.vs.control; Homo_714,male,AD.vs.control; Homo_714,female,AD.vs.MCI; Homo_714,male,AD.vs.MCI; Homo_714,female,MCI.vs.control; Homo_714,male,MCI.vs.control; Homo_714,control,male.vs.female; Homo_633,female,AD.vs.control; Homo_633,female,AD.vs.MCI; Homo_633,female,MCI.vs.control; ROSMAP,female,AD.vs.control; ROSMAP,male,AD.vs.control; Emory,totalSample,immune-Gamma_delta; Emory,totalSample,immune-Th17; Emory,totalSample,immune-Cytotoxic; Emory,totalSample,immune-Exhausted; Homo_723,totalSample,immune-Th17; Homo_723,totalSample,immune-Cytotoxic; Homo_723,totalSample,immune-CD8_T; Homo_723,totalSample,immune-Exhausted; Homo_714,totalSample,immune-Exhausted; Homo_714,totalSample,immune-CD8_T; Homo_714,totalSample,immune-Th17; Homo_633,totalSample,immune-NKT; Homo_633,totalSample,immune-Gamma_delta; MCSA,totalSample,immune-Neutrophil; MCSA,totalSample,immune-CD4_T; MCSA,totalSample,immune-CD8_T; MCSA,totalSample,immune-Gamma_delta; MCSA,totalSample,immune-Tr1; MCSA,totalSample,immune-iTreg; MCSA,totalSample,immune-Th17; MCSA,totalSample,immune-Cytotoxic; MCSA,totalSample,immune-Exhausted; SRP223445,totalSample,immune-Neutrophil; SRP223445,totalSample,immune-CD4_naive; SRP223445,totalSample,immune-nTreg; SRP223445,totalSample,immune-InfiltrationScore
ATE1	Homo_723,female,AD.vs.control; Homo_723,male,AD.vs.control; Homo_723,female,AD.vs.MCI; Homo_723,male,AD.vs.MCI; Homo_723,female,MCI.vs.control; Homo_714,female,AD.vs.control; Homo_714,male,AD.vs.control; Homo_714,female,AD.vs.MCI; Homo_714,male,AD.vs.MCI; Homo_714,female,MCI.vs.control; Homo_633,female,AD.vs.control; Homo_633,female,AD.vs.MCI; MCSA,MCI,age; Emory,totalSample,immune-Macrophage; Emory,totalSample,immune-NK; Emory,totalSample,immune-Neutrophil; Emory,totalSample,immune-CD4_T; Emory,totalSample,immune-CD8_T; Emory,totalSample,immune-Tr1; Emory,totalSample,immune-iTreg; Emory,totalSample,immune-Tfh; Emory,totalSample,immune-Central_memory; Emory,totalSample,immune-Effector_memory; Emory,totalSample,immune-InfiltrationScore; Homo_723,totalSample,immune-CD4_naive; Homo_723,totalSample,immune-Central_memory; Homo_723,totalSample,immune-CD4_T; Homo_723,totalSample,immune-Cytotoxic; Homo_723,totalSample,immune-Tr1; Homo_723,totalSample,immune-Tfh; Homo_723,totalSample,immune-Th1; Homo_723,totalSample,immune-InfiltrationScore; Homo_723,totalSample,immune-Th17; Homo_723,totalSample,immune-NKT; Homo_723,totalSample,immune-Exhausted; Homo_723,totalSample,immune-nTreg; Homo_723,totalSample,immune-iTreg; Homo_723,totalSample,immune-Neutrophil; Homo_723,totalSample,immune-CD8_T; Homo_723,totalSample,immune-NK; Homo_714,totalSample,immune-CD4_naive; Homo_714,totalSample,immune-NKT; Homo_714,totalSample,immune-InfiltrationScore; Homo_714,totalSample,immune-CD4_T; Homo_714,totalSample,immune-Central_memory; Homo_714,totalSample,immune-Tfh; Homo_714,totalSample,immune-Th1; Homo_714,totalSample,immune-Tr1; Homo_714,totalSample,immune-iTreg; Homo_714,totalSample,immune-Exhausted; Homo_633,totalSample,immune-Tfh; Homo_633,totalSample,immune-CD4_naive; Homo_633,totalSample,immune-CD4_T; Homo_633,totalSample,immune-NKT; Homo_633,totalSample,immune-Tr1; Homo_633,totalSample,immune-InfiltrationScore; Homo_633,totalSample,immune-Central_memory; Homo_633,totalSample,immune-Effector_memory; Homo_633,totalSample,immune-iTreg; Homo_633,totalSample,immune-Th2; Homo_633,totalSample,immune-Th1; Homo_633,totalSample,immune-Bcell; Homo_633,totalSample,immune-nTreg; MCSA,totalSample,immune-DC; MCSA,totalSample,immune-Monocyte; MCSA,totalSample,immune-Macrophage; MCSA,totalSample,immune-CD4_T; MCSA,totalSample,immune-CD8_T; MCSA,totalSample,immune-CD4_naive; MCSA,totalSample,immune-Tr1; MCSA,totalSample,immune-nTreg; MCSA,totalSample,immune-iTreg; MCSA,totalSample,immune-Th17; MCSA,totalSample,immune-Tfh; MCSA,totalSample,immune-CD8_naive; MCSA,totalSample,immune-Cytotoxic; MCSA,totalSample,immune-Exhausted; MCSA,totalSample,immune-MAIT; MCSA,totalSample,immune-Central_memory; ROSMAP,totalSample,immune-Effector_memory; SRP223445,totalSample,immune-CD4_naive; SRP223445,totalSample,immune-Exhausted
ANKZF1	Homo_723,female,AD.vs.control; Homo_723,male,AD.vs.control; Homo_723,female,AD.vs.MCI; Homo_723,male,AD.vs.MCI; Homo_723,female,MCI.vs.control; Homo_723,male,MCI.vs.control; Homo_723,control,male.vs.female; Homo_714,female,AD.vs.control; Homo_714,male,AD.vs.control; Homo_714,female,MCI.vs.control; Homo_714,male,MCI.vs.control; Homo_714,control,male.vs.female; Homo_633,female,AD.vs.control; Homo_633,female,AD.vs.MCI; Homo_633,female,MCI.vs.control; Homo_633,male,MCI.vs.control; Homo_633,control,male.vs.female; ACOM,totalSample,age; MCSA,totalSample,age; MCSA,control,age; ROSMAP,MCI,last.visit.age; Emory,totalSample,immune-Macrophage; Emory,totalSample,immune-CD4_T; Emory,totalSample,immune-Tr1; Emory,totalSample,immune-iTreg; Emory,totalSample,immune-Th17; Emory,totalSample,immune-MAIT; Emory,totalSample,immune-Central_memory; Emory,totalSample,immune-InfiltrationScore; Homo_723,totalSample,immune-Monocyte; Homo_723,totalSample,immune-Central_memory; Homo_723,totalSample,immune-CD4_naive; Homo_723,totalSample,immune-nTreg; Homo_723,totalSample,immune-CD4_T; Homo_723,totalSample,immune-Tfh; Homo_723,totalSample,immune-DC; Homo_723,totalSample,immune-iTreg; Homo_723,totalSample,immune-Tr1; Homo_714,totalSample,immune-Monocyte; Homo_714,totalSample,immune-CD4_naive; Homo_714,totalSample,immune-nTreg; Homo_714,totalSample,immune-CD4_T; Homo_714,totalSample,immune-iTreg; Homo_714,totalSample,immune-Central_memory; Homo_714,totalSample,immune-Th1; Homo_714,totalSample,immune-Tfh; Homo_633,totalSample,immune-Monocyte; Homo_633,totalSample,immune-nTreg; Homo_633,totalSample,immune-CD4_naive; Homo_633,totalSample,immune-iTreg; Homo_633,totalSample,immune-CD4_T; Homo_633,totalSample,immune-Tfh; Homo_633,totalSample,immune-Central_memory; Homo_633,totalSample,immune-InfiltrationScore; Homo_633,totalSample,immune-Tr1; Homo_633,totalSample,immune-Th1; MCSA,totalSample,immune-Monocyte; MCSA,totalSample,immune-Neutrophil; MCSA,totalSample,immune-CD4_T; MCSA,totalSample,immune-CD8_T; MCSA,totalSample,immune-Gamma_delta; MCSA,totalSample,immune-CD4_naive; MCSA,totalSample,immune-Tr1; MCSA,totalSample,immune-nTreg; MCSA,totalSample,immune-iTreg; MCSA,totalSample,immune-Th2; MCSA,totalSample,immune-Th17; MCSA,totalSample,immune-Tfh; MCSA,totalSample,immune-CD8_naive; MCSA,totalSample,immune-Central_memory; MCSA,totalSample,immune-Effector_memory; MCSA,totalSample,immune-InfiltrationScore; ROSMAP,totalSample,immune-Effector_memory; ROSMAP,totalSample,immune-InfiltrationScore; SRP223445,totalSample,immune-CD4_naive; SRP223445,totalSample,immune-Exhausted; SRP223445,totalSample,immune-[illegible]
FXYD2	Homo_723,female,AD.vs.control; Homo_723,male,AD.vs.control; Homo_723,female,AD.vs.MCI; Homo_723,male,AD.vs.MCI; Homo_714,female,AD.vs.control; Homo_714,male,AD.vs.control; Homo_714,female,AD.vs.MCI; Homo_714,male,AD.vs.MCI; Homo_633,female,AD.vs.control; Homo_633,female,AD.vs.MCI; ROSMAP,female,AD.vs.control; ROSMAP,male,AD.vs.control; ACOM,totalSample,age; MCSA,totalSample,age; MCSA,control,age; MCSA,MCI,age; Emory,totalSample,immune-Neutrophil; Emory,totalSample,immune-CD4_T; Emory,totalSample,immune-CD8_T; Emory,totalSample,immune-Tr1; Emory,totalSample,immune-iTreg; Emory,totalSample,immune-Tfh; Emory,totalSample,immune-MAIT; Homo_723,totalSample,immune-CD8_T; Homo_723,totalSample,immune-Cytotoxic; Homo_723,totalSample,immune-Exhausted; Homo_723,totalSample,immune-Tfh; Homo_723,totalSample,immune-MAIT; Homo_723,totalSample,immune-iTreg; Homo_723,totalSample,immune-Macrophage; Homo_723,totalSample,immune-Effector_memory; Homo_723,totalSample,immune-nTreg; Homo_723,totalSample,immune-Central_memory; Homo_714,totalSample,immune-CD8_T; Homo_714,totalSample,immune-Tfh; Homo_714,totalSample,immune-Effector_memory; Homo_714,totalSample,immune-nTreg; Homo_714,totalSample,immune-Macrophage; Homo_714,totalSample,immune-MAIT; Homo_714,totalSample,immune-Exhausted; Homo_714,totalSample,immune-Cytotoxic; Homo_633,totalSample,immune-nTreg; Homo_633,totalSample,immune-Neutrophil; Homo_633,totalSample,immune-CD8_T; Homo_633,totalSample,immune-DC; Homo_633,totalSample,immune-InfiltrationScore; Homo_633,totalSample,immune-Monocyte; Homo_633,totalSample,immune-Effector_memory; Homo_633,totalSample,immune-Macrophage; MCSA,totalSample,immune-DC; MCSA,totalSample,immune-Monocyte; MCSA,totalSample,immune-Neutrophil; MCSA,totalSample,immune-CD8_T; MCSA,totalSample,immune-Th2; MCSA,totalSample,immune-Tfh; MCSA,totalSample,immune-Cytotoxic; [illegible]
SELENOI	Homo_723,female,AD.vs.control; Homo_723,male,AD.vs.control; Homo_723,female,AD.vs.MCI; Homo_723,male,AD.vs.MCI; Homo_723,female,MCI.vs.control; Homo_723,male,MCI.vs.control; Homo_714,female,AD.vs.control; Homo_714,male,AD.vs.control; Homo_714,female,AD.vs.MCI; Homo_714,male,AD.vs.MCI; Homo_714,female,MCI.vs.control; Homo_714,male,MCI.vs.control; Homo_633,female,AD.vs.control; Homo_633,female,AD.vs.MCI; Emory,totalSample,immune-Macrophage; Emory,totalSample,immune-Neutrophil; Emory,totalSample,immune-CD4_T; Emory,totalSample,immune-CD8_T; Emory,totalSample,immune-Tr1; Emory,totalSample,immune-iTreg; Emory,totalSample,immune-Tfh; Emory,totalSample,immune-Central_memory; Emory,totalSample,immune-InfiltrationScore; Homo_723,totalSample,immune-CD4_naive; Homo_723,totalSample,immune-Th17; Homo_723,totalSample,immune-Exhausted; Homo_723,totalSample,immune-CD4_T; Homo_723,totalSample,immune-Cytotoxic; Homo_723,totalSample,immune-Tfh; Homo_723,totalSample,immune-Tr1; Homo_723,totalSample,immune-Th1; Homo_723,totalSample,immune-Central_memory; Homo_723,totalSample,immune-Th2; Homo_723,totalSample,immune-nTreg; Homo_723,totalSample,immune-Neutrophil; Homo_723,totalSample,immune-InfiltrationScore; Homo_723,totalSample,immune-CD8_T; Homo_714,totalSample,immune-CD4_naive; Homo_714,totalSample,immune-CD4_T; Homo_714,totalSample,immune-Th17; Homo_714,totalSample,immune-Central_memory; Homo_714,totalSample,immune-Tfh; Homo_714,totalSample,immune-InfiltrationScore; Homo_714,totalSample,immune-Th1; Homo_714,totalSample,immune-Monocyte; Homo_714,totalSample,immune-Tr1; Homo_714,totalSample,immune-Exhausted; Homo_633,totalSample,immune-Tfh; Homo_633,totalSample,immune-CD4_T; Homo_633,totalSample,immune-CD4_naive; Homo_633,totalSample,immune-Th2; Homo_633,totalSample,immune-Effector_memory; Homo_633,totalSample,immune-Central_memory; Homo_633,totalSample,immune-Tr1; Homo_633,totalSample,immune-InfiltrationScore; Homo_633,totalSample,immune-Monocyte; Homo_633,totalSample,immune-Bcell; Homo_633,totalSample,immune-iTreg; Homo_633,totalSample,immune-NKT; Homo_633,totalSample,immune-Th17; Homo_633,totalSample,immune-nTreg; MCSA,totalSample,immune-DC; MCSA,totalSample,immune-Macrophage; MCSA,totalSample,immune-CD4_T; MCSA,totalSample,immune-CD4_naive; MCSA,totalSample,immune-Tr1; MCSA,totalSample,immune-nTreg; MCSA,totalSample,immune-iTreg; MCSA,totalSample,immune-Th17; MCSA,totalSample,immune-Tfh; MCSA,totalSample,immune-CD8_naive; MCSA,totalSample,immune-Cytotoxic; MCSA,totalSample,immune-Exhausted; [illegible]
FLRT1	Homo_723,female,AD.vs.control; Homo_723,female,AD.vs.MCI; Homo_723,male,MCI.vs.control; Homo_714,female,AD.vs.MCI; Homo_714,female,MCI.vs.control; Homo_714,male,MCI.vs.control; Homo_633,female,AD.vs.control; Homo_633,female,AD.vs.MCI; Homo_633,female,MCI.vs.control; Homo_633,male,MCI.vs.control; MCSA,totalSample,age; MCSA,control,age; ROSMAP,AD,last.visit.age; Homo_723,totalSample,immune-Monocyte; Homo_723,totalSample,immune-DC; Homo_723,totalSample,immune-Central_memory; Homo_723,totalSample,immune-nTreg; Homo_723,totalSample,immune-CD8_T; Homo_723,totalSample,immune-CD8_naive; Homo_723,totalSample,immune-Exhausted; Homo_714,totalSample,immune-Monocyte; Homo_714,totalSample,immune-nTreg; Homo_714,totalSample,immune-DC; Homo_633,totalSample,immune-Monocyte; Homo_633,totalSample,immune-nTreg; Homo_633,totalSample,immune-DC; Homo_633,totalSample,immune-InfiltrationScore; MCSA,totalSample,immune-DC; MCSA,totalSample,immune-Monocyte; MCSA,totalSample,immune-Neutrophil; MCSA,totalSample,immune-CD4_T; MCSA,totalSample,immune-CD8_T; MCSA,totalSample,immune-NKT; MCSA,totalSample,immune-CD4_naive; MCSA,totalSample,immune-Tr1; MCSA,totalSample,immune-nTreg; MCSA,totalSample,immune-Th2; MCSA,totalSample,immune-Tfh; MCSA,totalSample,immune-Cytotoxic;
ABT1	Homo_723,female,AD.vs.control; Homo_723,male,AD.vs.control; Homo_723,female,AD.vs.MCI; Homo_723,male,AD.vs.MCI; Homo_714,female,AD.vs.control; Homo_714,male,AD.vs.control; Homo_714,female,AD.vs.MCI; Homo_714,male,AD.vs.MCI; Homo_633,female,AD.vs.control; Homo_633,female,AD.vs.MCI; MCSA,totalSample,age; MCSA,control,age; MCSA,MCI,age; ROSMAP,MCI,last.visit.age; ROSMAP,MCI,sample.age; Emory,totalSample,immune-Macrophage; Emory,totalSample,immune-NK; Emory,totalSample,immune-Neutrophil; Emory,totalSample,immune-CD4_T; Emory,totalSample,immune-CD8_T; Emory,totalSample,immune-Tr1; Emory,totalSample,immune-iTreg; Emory,totalSample,immune-Tfh; Emory,totalSample,immune-Exhausted; Emory,totalSample,immune-Effector_memory; Emory,totalSample,immune-InfiltrationScore; Homo_723,totalSample,immune-InfiltrationScore; Homo_723,totalSample,immune-CD8_T; Homo_723,totalSample,immune-Effector_memory; Homo_723,totalSample,immune-Tfh; Homo_723,totalSample,immune-iTreg; Homo_723,totalSample,immune-Cytotoxic; Homo_723,totalSample,immune-nTreg; Homo_723,totalSample,immune-MAIT; Homo_723,totalSample,immune-Central_memory; Homo_723,totalSample,immune-Macrophage; Homo_723,totalSample,immune-Exhausted; Homo_723,totalSample,immune-CD4_naive; Homo_723,totalSample,immune-CD4_T; Homo_723,totalSample,immune-Monocyte; Homo_714,totalSample,immune-Effector_memory; Homo_714,totalSample,immune-InfiltrationScore; Homo_714,totalSample,immune-Tfh; Homo_714,totalSample,immune-CD8_T; Homo_714,totalSample,immune-nTreg; Homo_714,totalSample,immune-Central_memory; Homo_714,totalSample,immune-Macrophage; Homo_714,totalSample,immune-Monocyte; Homo_714,totalSample,immune-MAIT; Homo_714,totalSample,immune-DC; Homo_714,totalSample,immune-iTreg; Homo_714,totalSample,immune-Cytotoxic; Homo_714,totalSample,immune-CD4_T; Homo_714,totalSample,immune-Neutrophil; Homo_633,totalSample,immune-InfiltrationScore; Homo_633,totalSample,immune-Effector_memory; Homo_633,totalSample,immune-nTreg; Homo_633,totalSample,immune-DC; Homo_633,totalSample,immune-Central_memory; Homo_633,totalSample,immune-Neutrophil; Homo_633,totalSample,immune-CD8_T; Homo_633,totalSample,immune-Monocyte; Homo_633,totalSample,immune-Tfh; Homo_633,totalSample,immune-CD4_naive; Homo_633,totalSample,immune-MAIT; MCSA,totalSample,immune-DC; MCSA,totalSample,immune-Monocyte; MCSA,totalSample,immune-Neutrophil; MCSA,totalSample,immune-CD4_T; MCSA,totalSample,immune-CD8_T; MCSA,totalSample,immune-NKT; MCSA,totalSample,immune-CD4_naive; MCSA,totalSample,immune-nTreg; MCSA,totalSample,immune-Th2; MCSA,totalSample,immune-Tfh; MCSA,totalSample,immune-Cytotoxic; MCSA,totalSample,immune-Central_memory; MCSA,totalSample,immune-Effector_memory; MCSA,totalSample,immune-InfiltrationScore; SRP223445,totalSample,immune-NK; SRP223445,totalSample,immune-CD4_naive; SRP223445,totalSample,immune-Th17; SRP223445,totalSample,immune-[illegible]
LGR4	Homo_723,female,AD.vs.control; Homo_723,male,AD.vs.control; Homo_723,female,AD.vs.MCI; Homo_723,male,AD.vs.MCI; Homo_723,female,MCI.vs.control; Homo_714,female,AD.vs.control; Homo_714,female,AD.vs.MCI; Homo_714,male,AD.vs.MCI; Homo_714,female,MCI.vs.control; Homo_633,female,AD.vs.control; Homo_633,female,AD.vs.MCI; ROSMAP,female,AD.vs.control; ROSMAP,AD,MMSE; Emory,totalSample,immune-Gamma_delta; Homo_723,totalSample,immune-Exhausted; Homo_723,totalSample,immune-Cytotoxic; Homo_723,totalSample,immune-Th17; Homo_723,totalSample,immune-CD4_naive; Homo_723,totalSample,immune-CD4_T; Homo_723,totalSample,immune-Th2; Homo_723,totalSample,immune-Central_memory; Homo_723,totalSample,immune-Tr1; Homo_723,totalSample,immune-Tfh; Homo_723,totalSample,immune-iTreg; Homo_723,totalSample,immune-Gamma_delta; Homo_723,totalSample,immune-Th1; Homo_714,totalSample,immune-CD4_naive; Homo_714,totalSample,immune-Exhausted; Homo_714,totalSample,immune-Gamma_delta; Homo_714,totalSample,immune-Central_memory; Homo_714,totalSample,immune-CD4_T; Homo_714,totalSample,immune-Th17; Homo_714,totalSample,immune-Tfh; Homo_714,totalSample,immune-Th2; Homo_714,totalSample,immune-Tr1; Homo_714,totalSample,immune-Cytotoxic; Homo_714,totalSample,immune-NKT; Homo_633,totalSample,immune-Tfh; Homo_633,totalSample,immune-CD4_T; Homo_633,totalSample,immune-Th2; Homo_633,totalSample,immune-Gamma_delta; Homo_633,totalSample,immune-CD4_naive; Homo_633,totalSample,immune-Central_memory; Homo_633,totalSample,immune-Exhausted; Homo_633,totalSample,immune-Effector_memory; Homo_633,totalSample,immune-NKT; Homo_633,totalSample,immune-Tr1; Homo_633,totalSample,immune-Th17; Homo_633,totalSample,immune-iTreg; Homo_633,totalSample,immune-Monocyte; MCSA,totalSample,immune-DC; MCSA,totalSample,immune-Bcell; MCSA,totalSample,immune-CD4_T; MCSA,totalSample,immune-CD8_T; MCSA,totalSample,immune-Gamma_delta; MCSA,totalSample,immune-CD4_naive; MCSA,totalSample,immune-Tr1; MCSA,totalSample,immune-nTreg; MCSA,totalSample,immune-iTreg; MCSA,totalSample,immune-Th17; MCSA,totalSample,immune-Tfh; MCSA,totalSample,immune-CD8_naive; MCSA,totalSample,immune-Cytotoxic; MCSA,totalSample,immune-Exhausted; MCSA,totalSample,immune-MAIT; MCSA,totalSample,immune-[illegible]
IL13RA1	Homo_723,female,AD.vs.control; Homo_723,male,AD.vs.control; Homo_723,female,AD.vs.MCI; Homo_723,male,AD.vs.MCI; Homo_714,female,AD.vs.control; Homo_714,male,AD.vs.control; Homo_714,female,AD.vs.MCI; Homo_714,male,AD.vs.MCI; Homo_633,female,AD.vs.control; Homo_633,female,AD.vs.MCI; Emory,MCI,age; MCSA,MCI,age; Emory,totalSample,immune-Monocyte; Emory,totalSample,immune-Neutrophil; Emory,totalSample,immune-CD8_T; Emory,totalSample,immune-Gamma_delta; Emory,totalSample,immune-Th1; Emory,totalSample,immune-Th17; Emory,totalSample,immune-Tfh; Emory,totalSample,immune-CD8_naive; Emory,totalSample,immune-Cytotoxic; Emory,totalSample,immune-Exhausted; Homo_723,totalSample,immune-Monocyte; Homo_723,totalSample,immune-Neutrophil; Homo_723,totalSample,immune-DC; Homo_723,totalSample,immune-Gamma_delta; Homo_723,totalSample,immune-Bcell; Homo_723,totalSample,immune-Th2; Homo_723,totalSample,immune-Exhausted; Homo_714,totalSample,immune-Monocyte; Homo_714,totalSample,immune-Th2; Homo_714,totalSample,immune-Neutrophil; Homo_714,totalSample,immune-Gamma_delta; Homo_714,totalSample,immune-Exhausted; Homo_714,totalSample,immune-DC; Homo_633,totalSample,immune-Monocyte; Homo_633,totalSample,immune-Exhausted; Homo_633,totalSample,immune-Th2; Homo_633,totalSample,immune-CD8_T; Homo_633,totalSample,immune-Gamma_delta; Homo_633,totalSample,immune-Neutrophil; Homo_633,totalSample,immune-Th17; Homo_633,totalSample,immune-Macrophage; MCSA,totalSample,immune-DC; MCSA,totalSample,immune-Monocyte; MCSA,totalSample,immune-Neutrophil; MCSA,totalSample,immune-CD8_T; MCSA,totalSample,immune-NKT; MCSA,totalSample,immune-iTreg; MCSA,totalSample,immune-Th1; MCSA,totalSample,immune-Th2; MCSA,totalSample,immune-Cytotoxic; MCSA,totalSample,immune-Exhausted; MCSA,totalSample,immune-Central_memory; MCSA,totalSample,immune-Effector_memory; MCSA,totalSample,immune-InfiltrationScore; ROSMAP,totalSample,immune-Effector_memory; [illegible]
KIF2C	Homo_723,female,AD.vs.control; Homo_723,male,AD.vs.control; Homo_723,female,AD.vs.MCI; Homo_723,male,AD.vs.MCI; Homo_723,female,MCI.vs.control; Homo_723,male,MCI.vs.control; Homo_714,female,AD.vs.control; Homo_714,male,AD.vs.control; Homo_714,female,AD.vs.MCI; Homo_714,male,AD.vs.MCI; Homo_633,female,AD.vs.control; Homo_633,female,AD.vs.MCI; ROSMAP,AD,MMSE; Emory,totalSample,immune-Neutrophil; Emory,totalSample,immune-Tr1; Emory,totalSample,immune-iTreg; Emory,totalSample,immune-InfiltrationScore; Homo_723,totalSample,immune-Th17; Homo_723,totalSample,immune-nTreg; Homo_723,totalSample,immune-Neutrophil; Homo_723,totalSample,immune-InfiltrationScore; Homo_723,totalSample,immune-CD4_naive; Homo_723,totalSample,immune-CD4_T; Homo_723,totalSample,immune-DC; Homo_723,totalSample,immune-Exhausted; Homo_714,totalSample,immune-InfiltrationScore; Homo_633,totalSample,immune-InfiltrationScore; Homo_633,totalSample,immune-Tfh; Homo_633,totalSample,immune-Tr1; Homo_633,totalSample,immune-nTreg; Homo_633,totalSample,immune-CD4_T; Homo_633,totalSample,immune-Effector_memory; Homo_633,totalSample,immune-Th2; MCSA,totalSample,immune-Monocyte; MCSA,totalSample,immune-Neutrophil; MCSA,totalSample,immune-CD4_T; MCSA,totalSample,immune-CD4_naive; MCSA,totalSample,immune-Tr1; MCSA,totalSample,immune-nTreg; MCSA,totalSample,immune-Tfh; MCSA,totalSample,immune-InfiltrationScore; ROSMAP,totalSample,immune-InfiltrationScore

DNAJC17	Homo_723,female,AD.vs.control; Homo_723,male,AD.vs.control; Homo_723,female,AD.vs.MCI; Homo_723,male,AD.vs.MCI; Homo_714,female,AD.vs.control; Homo_714,male,AD.vs.control; Homo_714,female,AD.vs.MCI; Homo_714,male,AD.vs.MCI; Homo_633,female,AD.vs.control; Homo_633,female,AD.vs.MCI; SRP223445,female,AD.vs.control; MCSA,MCI,age; ROSMAP,MCI,last.visit.age; Emory,totalSample,immune-Macrophage; Emory,totalSample,immune-NK; Emory,totalSample,immune-Neutrophil; Emory,totalSample,immune-CD4_T; Emory,totalSample,immune-CD8_T; Emory,totalSample,immune-Tr1; Emory,totalSample,immune-iTreg; Emory,totalSample,immune-Tfh; Emory,totalSample,immune-Exhausted; Emory,totalSample,immune-MAIT; Emory,totalSample,immune-Effector_memory; Emory,totalSample,immune-InfiltrationScore; Homo_723,totalSample,immune-CD8_T; Homo_723,totalSample,immune-Cytotoxic; Homo_723,totalSample,immune-InfiltrationScore; Homo_723,totalSample,immune-Exhausted; Homo_723,totalSample,immune-Effector_memory; Homo_723,totalSample,immune-Tfh; Homo_723,totalSample,immune-Macrophage; Homo_723,totalSample,immune-MAIT; Homo_723,totalSample,immune-iTreg; Homo_723,totalSample,immune-nTreg; Homo_723,totalSample,immune-Th17; Homo_714,totalSample,immune-CD8_T; Homo_714,totalSample,immune-InfiltrationScore; Homo_714,totalSample,immune-Cytotoxic; Homo_714,totalSample,immune-Effector_memory; Homo_714,totalSample,immune-Macrophage; Homo_714,totalSample,immune-Tfh; Homo_714,totalSample,immune-Neutrophil; Homo_714,totalSample,immune-nTreg; Homo_714,totalSample,immune-Exhausted; Homo_714,totalSample,immune-DC; Homo_714,totalSample,immune-MAIT; Homo_633,totalSample,immune-InfiltrationScore; Homo_633,totalSample,immune-DC; Homo_633,totalSample,immune-Neutrophil; Homo_633,totalSample,immune-CD8_T; Homo_633,totalSample,immune-Cytotoxic; Homo_633,totalSample,immune-nTreg; Homo_633,totalSample,immune-NK; Homo_633,totalSample,immune-Effector_memory; MCSA,totalSample,immune-DC; MCSA,totalSample,immune-Monocyte; MCSA,totalSample,immune-Neutrophil; MCSA,totalSample,immune-CD8_T; MCSA,totalSample,immune-NKT; MCSA,totalSample,immune-Gamma_delta; MCSA,totalSample,immune-iTreg; MCSA,totalSample,immune-Th2; MCSA,totalSample,immune-Th17; MCSA,totalSample,immune-Cytotoxic; MCSA,totalSample,immune-Exhausted; MCSA,totalSample,immune-Effector_memory; MCSA,totalSample,immune-InfiltrationScore; ROSMAP,totalSample,immune-Th17; ROSMAP,totalSample,immune-Effector_memory; SRP223445,totalSample,immune-NK; SRP223445,totalSample,immune-Neutrophil; SRP223445,totalSample,immune-CD4_naive; SRP223445,totalSample,immune-InfiltrationScore
TNFRSF11A	Homo_723,female,AD.vs.control; Homo_723,male,AD.vs.control; Homo_723,female,AD.vs.MCI; Homo_723,male,AD.vs.MCI; Homo_714,female,AD.vs.control; Homo_714,male,AD.vs.control; Homo_714,female,AD.vs.MCI; Homo_714,male,AD.vs.MCI; Homo_633,female,AD.vs.control; Homo_633,female,AD.vs.MCI; Emory,totalSample,immune-iTreg; Homo_723,totalSample,immune-Th17; Homo_723,totalSample,immune-DC; Homo_723,totalSample,immune-Th1; Homo_723,totalSample,immune-NK; Homo_714,totalSample,immune-CD8_T; Homo_714,totalSample,immune-Th1; Homo_714,totalSample,immune-NK; Homo_633,totalSample,immune-InfiltrationScore; Homo_633,totalSample,immune-Tr1; Homo_633,totalSample,immune-iTreg; Homo_633,totalSample,immune-Tfh; Homo_633,totalSample,immune-NK; MCSA,totalSample,immune-CD4_T; MCSA,totalSample,immune-CD4_naive; MCSA,totalSample,immune-Tr1; MCSA,totalSample,immune-nTreg; MCSA,totalSample,immune-iTreg; MCSA,totalSample,immune-Th17; MCSA,totalSample,immune-Tfh;
DHX58	Homo_723,female,AD.vs.control; Homo_723,male,AD.vs.control; Homo_723,female,AD.vs.MCI; Homo_723,male,AD.vs.MCI; Homo_723,female,MCI.vs.control; Homo_714,female,AD.vs.control; Homo_714,male,AD.vs.control; Homo_714,female,AD.vs.MCI; Homo_714,male,AD.vs.MCI; Homo_714,female,MCI.vs.control; Homo_714,male,MCI.vs.control; Homo_633,female,AD.vs.control; Homo_633,female,AD.vs.MCI; Homo_633,female,MCI.vs.control; Homo_633,male,MCI.vs.control; ROSMAP,male,AD.vs.control; ROSMAP,AD,male.vs.female; Emory,MCI,age; MCSA,totalSample,age; MCSA,control,age; MCSA,MCI,age; ROSMAP,MCI,last.visit.age; Emory,totalSample,immune-Gamma_delta; Emory,totalSample,immune-CD8_naive; Emory,totalSample,immune-Cytotoxic; Emory,totalSample,immune-InfiltrationScore; Homo_723,totalSample,immune-Effector_memory; Homo_723,totalSample,immune-InfiltrationScore; Homo_723,totalSample,immune-Tfh; Homo_723,totalSample,immune-Bcell; Homo_723,totalSample,immune-Cytotoxic; Homo_723,totalSample,immune-Th2; Homo_723,totalSample,immune-CD8_T; Homo_723,totalSample,immune-Th1; Homo_714,totalSample,immune-Effector_memory; Homo_714,totalSample,immune-Th2; Homo_714,totalSample,immune-Tfh; Homo_714,totalSample,immune-InfiltrationScore; Homo_714,totalSample,immune-nTreg; Homo_714,totalSample,immune-DC; Homo_633,totalSample,immune-DC; Homo_633,totalSample,immune-InfiltrationScore; Homo_633,totalSample,immune-Effector_memory; Homo_633,totalSample,immune-nTreg; Homo_633,totalSample,immune-Central_memory; MCSA,totalSample,immune-DC; MCSA,totalSample,immune-Monocyte; MCSA,totalSample,immune-Neutrophil; MCSA,totalSample,immune-CD8_T; MCSA,totalSample,immune-NKT; MCSA,totalSample,immune-Th2; MCSA,totalSample,immune-Tfh; MCSA,totalSample,immune-Cytotoxic; MCSA,totalSample,immune-Central_memory; MCSA,totalSample,immune-Effector_memory; MCSA,totalSample,immune-InfiltrationScore; ROSMAP,totalSample,immune-Effector_memory; ROSMAP,totalSample,immune-InfiltrationScore; SRP223445,totalSample,immune-NK; SRP223445,totalSample,immune-Th17; SRP223445,totalSample,immune-Exhausted; SRP223445,totalSample,immune-InfiltrationScore; ROSMAP,female-excludeAge90+,survival; ROSMAP,female-includeAge90+,survival
LRRN1	Homo_723,female,AD.vs.control; Homo_723,male,AD.vs.control; Homo_723,female,AD.vs.MCI; Homo_723,male,AD.vs.MCI; Homo_723,female,MCI.vs.control; Homo_714,female,AD.vs.control; Homo_714,male,AD.vs.control; Homo_714,female,AD.vs.MCI; Homo_714,male,AD.vs.MCI; Homo_633,female,AD.vs.control; Homo_633,female,AD.vs.MCI; ROSMAP,MCI,sample.age; Emory,totalSample,immune-Macrophage; Emory,totalSample,immune-Neutrophil; Emory,totalSample,immune-CD8_T; Emory,totalSample,immune-Gamma_delta; Emory,totalSample,immune-Th1; Emory,totalSample,immune-Tfh; Emory,totalSample,immune-CD8_naive; Emory,totalSample,immune-Cytotoxic; Emory,totalSample,immune-Exhausted; Homo_723,totalSample,immune-Gamma_delta; Homo_723,totalSample,immune-Neutrophil; Homo_723,totalSample,immune-Th2; Homo_723,totalSample,immune-CD8_naive; Homo_714,totalSample,immune-Gamma_delta; Homo_714,totalSample,immune-Neutrophil; Homo_714,totalSample,immune-Th2; Homo_714,totalSample,immune-DC; Homo_633,totalSample,immune-Gamma_delta; Homo_633,totalSample,immune-Neutrophil; Homo_633,totalSample,immune-Exhausted; Homo_633,totalSample,immune-CD8_T; Homo_633,totalSample,immune-Th17; Homo_633,totalSample,immune-Th2; Homo_633,totalSample,immune-Cytotoxic; Homo_633,totalSample,immune-Monocyte; Homo_633,totalSample,immune-DC; MCSA,totalSample,immune-Neutrophil; MCSA,totalSample,immune-CD8_T; MCSA,totalSample,immune-Gamma_delta; MCSA,totalSample,immune-iTreg; MCSA,totalSample,immune-Th1; MCSA,totalSample,immune-Th17; MCSA,totalSample,immune-Cytotoxic; MCSA,totalSample,immune-Exhausted; MCSA,totalSample,immune-Effector_memory;
DCLRE1A	Homo_723,female,AD.vs.control; Homo_723,male,AD.vs.control; Homo_723,female,AD.vs.MCI; Homo_723,male,AD.vs.MCI; Homo_723,female,MCI.vs.control; Homo_723,male,MCI.vs.control; Homo_714,female,AD.vs.control; Homo_714,male,AD.vs.control; Homo_714,female,AD.vs.MCI; Homo_714,male,AD.vs.MCI; Homo_714,female,MCI.vs.control; Homo_714,male,MCI.vs.control; Homo_633,female,AD.vs.control; Homo_633,female,AD.vs.MCI; SRP223445,female,AD.vs.control; Emory,MCI,age; MCSA,MCI,age; ROSMAP,AD,MMSE; Emory,totalSample,immune-Macrophage; Emory,totalSample,immune-NK; Emory,totalSample,immune-Neutrophil; Emory,totalSample,immune-CD4_T; Emory,totalSample,immune-CD8_T; Emory,totalSample,immune-Tr1; Emory,totalSample,immune-iTreg; Emory,totalSample,immune-Tfh; Emory,totalSample,immune-Exhausted; Emory,totalSample,immune-MAIT; Emory,totalSample,immune-Effector_memory; Emory,totalSample,immune-InfiltrationScore; Homo_723,totalSample,immune-Exhausted; Homo_723,totalSample,immune-CD4_naive; Homo_723,totalSample,immune-Th17; Homo_723,totalSample,immune-CD4_T; Homo_723,totalSample,immune-Tfh; Homo_723,totalSample,immune-Cytotoxic; Homo_723,totalSample,immune-InfiltrationScore; Homo_723,totalSample,immune-CD8_T; Homo_723,totalSample,immune-Tr1; Homo_723,totalSample,immune-Neutrophil; Homo_723,totalSample,immune-Central_memory; Homo_723,totalSample,immune-Monocyte; Homo_723,totalSample,immune-Effector_memory; Homo_723,totalSample,immune-Th1; Homo_723,totalSample,immune-NK; Homo_714,totalSample,immune-CD4_naive; Homo_714,totalSample,immune-Monocyte; Homo_714,totalSample,immune-InfiltrationScore; Homo_714,totalSample,immune-Tfh; Homo_714,totalSample,immune-Central_memory; Homo_714,totalSample,immune-CD4_T; Homo_714,totalSample,immune-Th17; Homo_714,totalSample,immune-Neutrophil; Homo_714,totalSample,immune-Exhausted; Homo_633,totalSample,immune-Tfh; Homo_633,totalSample,immune-Effector_memory; Homo_633,totalSample,immune-InfiltrationScore; Homo_633,totalSample,immune-CD4_T; Homo_633,totalSample,immune-Monocyte; Homo_633,totalSample,immune-CD4_naive; Homo_633,totalSample,immune-Th2; Homo_633,totalSample,immune-Central_memory; Homo_633,totalSample,immune-Tr1; Homo_633,totalSample,immune-Bcell; Homo_633,totalSample,immune-Neutrophil; Homo_633,totalSample,immune-NKT; MCSA,totalSample,immune-DC; MCSA,totalSample,immune-Monocyte; MCSA,totalSample,immune-Macrophage; MCSA,totalSample,immune-CD4_T; MCSA,totalSample,immune-CD4_naive; MCSA,totalSample,immune-Tr1; MCSA,totalSample,immune-nTreg; MCSA,totalSample,immune-iTreg; MCSA,totalSample,immune-Tfh; MCSA,totalSample,immune-CD8_naive; MCSA,totalSample,immune-Cytotoxic; MCSA,totalSample,immune-Exhausted; MCSA,totalSample,immune-MAIT; MCSA,totalSample,immune-Central_memory; ROSMAP,totalSample,immune-Th17; ROSMAP,totalSample,immune-InfiltrationScore; SRP223445,totalSample,immune-CD4_naive; SRP223445,totalSample,immune-Th17
NECTIN1	Homo_723,female,AD.vs.control; Homo_723,male,AD.vs.control; Homo_723,female,AD.vs.MCI; Homo_723,male,AD.vs.MCI; Homo_714,female,AD.vs.control; Homo_633,female,AD.vs.control; Homo_633,female,AD.vs.MCI; Homo_633,female,MCI.vs.control; ROSMAP,female,AD.vs.control; Emory,totalSample,age; Emory,MCI,age; MCSA,MCI,age; Emory,totalSample,immune-Macrophage; Emory,totalSample,immune-Neutrophil; Emory,totalSample,immune-CD4_T; Emory,totalSample,immune-CD8_T; Emory,totalSample,immune-Gamma_delta; Emory,totalSample,immune-Tfh; Emory,totalSample,immune-CD8_naive; Emory,totalSample,immune-Exhausted; Homo_723,totalSample,immune-DC; Homo_723,totalSample,immune-Th2; Homo_723,totalSample,immune-Monocyte; Homo_723,totalSample,immune-Tr1; Homo_723,totalSample,immune-iTreg; Homo_714,totalSample,immune-Th2; Homo_714,totalSample,immune-DC; Homo_714,totalSample,immune-CD4_T; Homo_714,totalSample,immune-Tfh; Homo_714,totalSample,immune-Tr1; Homo_714,totalSample,immune-Central_memory; Homo_633,totalSample,immune-DC; Homo_633,totalSample,immune-Th2; Homo_633,totalSample,immune-CD4_T; MCSA,totalSample,immune-DC; MCSA,totalSample,immune-Bcell; MCSA,totalSample,immune-Monocyte; MCSA,totalSample,immune-Macrophage; MCSA,totalSample,immune-CD4_T; MCSA,totalSample,immune-CD4_naive; MCSA,totalSample,immune-Tr1; MCSA,totalSample,immune-nTreg; MCSA,totalSample,immune-iTreg; MCSA,totalSample,immune-Th1; MCSA,totalSample,immune-Th2; MCSA,totalSample,immune-Tfh; MCSA,totalSample,immune-CD8_naive; MCSA,totalSample,immune-Cytotoxic; MCSA,totalSample,immune-MAIT;
SIRPD	Homo_723,female,AD.vs.control; Homo_723,male,AD.vs.control; Homo_723,female,AD.vs.MCI; Homo_723,male,AD.vs.MCI; Homo_723,female,MCI.vs.control; Homo_714,female,AD.vs.control; Homo_714,male,AD.vs.control; Homo_714,female,AD.vs.MCI; Homo_714,male,AD.vs.MCI; Homo_714,female,MCI.vs.control; Homo_633,female,AD.vs.control; Homo_633,female,AD.vs.MCI; Homo_633,female,MCI.vs.control; ROSMAP,female,AD.vs.control; Emory,MCI,age; Emory,totalSample,immune-DC; Emory,totalSample,immune-Monocyte; Emory,totalSample,immune-Neutrophil; Emory,totalSample,immune-CD8_T; Emory,totalSample,immune-Gamma_delta; Emory,totalSample,immune-nTreg; Emory,totalSample,immune-Th1; Emory,totalSample,immune-Th17; Emory,totalSample,immune-Tfh; Emory,totalSample,immune-CD8_naive; Emory,totalSample,immune-Cytotoxic; Emory,totalSample,immune-Exhausted; Homo_723,totalSample,immune-Neutrophil; Homo_723,totalSample,immune-Gamma_delta; Homo_723,totalSample,immune-CD8_naive; Homo_714,totalSample,immune-Neutrophil; Homo_714,totalSample,immune-Gamma_delta; Homo_633,totalSample,immune-CD8_T; Homo_633,totalSample,immune-Neutrophil; Homo_633,totalSample,immune-Gamma_delta; Homo_633,totalSample,immune-NKT; Homo_633,totalSample,immune-InfiltrationScore; Homo_633,totalSample,immune-Cytotoxic; MCSA,totalSample,immune-Macrophage; MCSA,totalSample,immune-Neutrophil; MCSA,totalSample,immune-CD8_T; MCSA,totalSample,immune-Gamma_delta; MCSA,totalSample,immune-Th1; MCSA,totalSample,immune-Th2; MCSA,totalSample,immune-Th17; MCSA,totalSample,immune-Cytotoxic; MCSA,totalSample,immune-Exhausted; ROSMAP,totalSample,immune-Effector_memory
LOC102723728	Homo_723,female,AD.vs.control; Homo_723,male,AD.vs.control; Homo_723,female,AD.vs.MCI; Homo_723,male,AD.vs.MCI; Homo_714,female,AD.vs.control; Homo_714,male,AD.vs.control; Homo_714,female,AD.vs.MCI; Homo_714,male,AD.vs.MCI; Homo_633,female,AD.vs.control; Homo_633,female,AD.vs.MCI; Emory,totalSample,age; Emory,MCI,age; MCSA,totalSample,age; MCSA,control,age; Emory,totalSample,immune-Gamma_delta; Emory,totalSample,immune-Th17; Emory,totalSample,immune-InfiltrationScore; Homo_723,totalSample,immune-DC; Homo_723,totalSample,immune-Monocyte; Homo_723,totalSample,immune-Th17; Homo_714,totalSample,immune-DC; Homo_714,totalSample,immune-Monocyte; Homo_714,totalSample,immune-InfiltrationScore; Homo_633,totalSample,immune-InfiltrationScore; Homo_633,totalSample,immune-Monocyte; Homo_633,totalSample,immune-DC; Homo_633,totalSample,immune-nTreg; MCSA,totalSample,immune-DC; MCSA,totalSample,immune-Monocyte; MCSA,totalSample,immune-Th17; MCSA,totalSample,immune-InfiltrationScore; SRP223445,totalSample,immune-nTreg; SRP223445,totalSample,immune-Exhausted; SRP223445,totalSample,immune-InfiltrationScore
MNDA	Homo_723,female,AD.vs.control; Homo_723,male,AD.vs.control; Homo_723,female,AD.vs.MCI; Homo_723,male,AD.vs.MCI; Homo_723,female,MCI.vs.control; SRP223445,male,AD.vs.control; Emory,MCI,age; MCSA,totalSample,age; MCSA,control,age; MCSA,MCI,age; Emory,totalSample,immune-DC; Emory,totalSample,immune-Monocyte; Emory,totalSample,immune-NK; Emory,totalSample,immune-Neutrophil; Emory,totalSample,immune-CD4_T; Emory,totalSample,immune-CD8_T; Emory,totalSample,immune-Gamma_delta; Emory,totalSample,immune-Th17; Emory,totalSample,immune-Tfh; Emory,totalSample,immune-CD8_naive; Emory,totalSample,immune-Cytotoxic; Emory,totalSample,immune-Exhausted; Emory,totalSample,immune-MAIT; Emory,totalSample,immune-InfiltrationScore; Homo_723,totalSample,immune-Monocyte; Homo_723,totalSample,immune-DC; Homo_723,totalSample,immune-Neutrophil; Homo_723,totalSample,immune-Exhausted; Homo_723,totalSample,immune-Th2; Homo_723,totalSample,immune-Gamma_delta; Homo_723,totalSample,immune-Th1; Homo_714,totalSample,immune-Neutrophil; Homo_714,totalSample,immune-CD8_T; Homo_714,totalSample,immune-Exhausted; Homo_714,totalSample,immune-Monocyte; Homo_714,totalSample,immune-Macrophage; Homo_714,totalSample,immune-nTreg; Homo_714,totalSample,immune-DC; Homo_714,totalSample,immune-Central_memory; Homo_714,totalSample,immune-MAIT; Homo_714,totalSample,immune-CD4_naive; Homo_714,totalSample,immune-Cytotoxic; Homo_714,totalSample,immune-Effector_memory; Homo_714,totalSample,immune-Gamma_delta; Homo_714,totalSample,immune-CD4_T; Homo_633,totalSample,immune-Neutrophil; Homo_633,totalSample,immune-CD8_T; Homo_633,totalSample,immune-Monocyte; Homo_633,totalSample,immune-Exhausted; Homo_633,totalSample,immune-Macrophage; Homo_633,totalSample,immune-nTreg; Homo_633,totalSample,immune-Gamma_delta; Homo_633,totalSample,immune-Effector_memory; Homo_633,totalSample,immune-MAIT; Homo_633,totalSample,immune-DC; Homo_633,totalSample,immune-Cytotoxic; Homo_633,totalSample,immune-Central_memory; Homo_633,totalSample,immune-Th17; Homo_633,totalSample,immune-CD4_naive; MCSA,totalSample,immune-DC; MCSA,totalSample,immune-Monocyte; MCSA,totalSample,immune-Neutrophil; MCSA,totalSample,immune-CD4_T; MCSA,totalSample,immune-CD8_T; MCSA,totalSample,immune-NKT; MCSA,totalSample,immune-CD4_naive; MCSA,totalSample,immune-nTreg; MCSA,totalSample,immune-Th2; MCSA,totalSample,immune-Tfh; MCSA,totalSample,immune-Cytotoxic; MCSA,totalSample,immune-Exhausted; MCSA,totalSample,immune-Central_memory; MCSA,totalSample,immune-Effector_memory; MCSA,totalSample,immune-InfiltrationScore; ROSMAP,totalSample,immune-Th17; ROSMAP,totalSample,immune-Cytotoxic; ROSMAP,totalSample,immune-MAIT; ROSMAP,totalSample,immune-Effector_memory; SRP223445,totalSample,immune-Monocyte; SRP223445,totalSample,immune-Neutrophil; SRP223445,totalSample,immune-CD4_T; [illegible]
PFN2	Homo_723,female,AD.vs.control; Homo_723,male,AD.vs.control; Homo_723,female,AD.vs.MCI; Homo_723,male,AD.vs.MCI; Homo_723,female,MCI.vs.control; Homo_714,male,AD.vs.control; Homo_714,female,AD.vs.MCI; Homo_714,male,AD.vs.MCI; Homo_633,female,AD.vs.control; Homo_633,female,AD.vs.MCI; Emory,MCI,age; ROSMAP,MCI,last.visit.age; Emory,totalSample,immune-Macrophage; Emory,totalSample,immune-CD4_T; Emory,totalSample,immune-Gamma_delta; Emory,totalSample,immune-Tr1; Emory,totalSample,immune-nTreg; Emory,totalSample,immune-iTreg; Emory,totalSample,immune-Th17; Emory,totalSample,immune-Tfh; Emory,totalSample,immune-Central_memory; Homo_723,totalSample,immune-CD4_T; Homo_723,totalSample,immune-CD4_naive; Homo_723,totalSample,immune-Th2; Homo_723,totalSample,immune-Tfh; Homo_723,totalSample,immune-Tr1; Homo_723,totalSample,immune-nTreg; Homo_723,totalSample,immune-iTreg; Homo_723,totalSample,immune-Central_memory; Homo_723,totalSample,immune-Th17; Homo_723,totalSample,immune-Th1; Homo_723,totalSample,immune-Cytotoxic; Homo_723,totalSample,immune-Exhausted; Homo_714,totalSample,immune-Tfh; Homo_714,totalSample,immune-CD4_T; Homo_714,totalSample,immune-CD4_naive; Homo_714,totalSample,immune-Tr1; Homo_714,totalSample,immune-Th2; Homo_714,totalSample,immune-iTreg; Homo_714,totalSample,immune-Central_memory; Homo_714,totalSample,immune-Th1; Homo_714,totalSample,immune-nTreg; Homo_714,totalSample,immune-Th17; Homo_714,totalSample,immune-NKT; Homo_714,totalSample,immune-Gamma_delta; Homo_633,totalSample,immune-Tfh; Homo_633,totalSample,immune-CD4_T; Homo_633,totalSample,immune-Th2; Homo_633,totalSample,immune-CD4_naive; Homo_633,totalSample,immune-Central_memory; Homo_633,totalSample,immune-Tr1; Homo_633,totalSample,immune-iTreg; Homo_633,totalSample,immune-nTreg; Homo_633,totalSample,immune-NKT; Homo_633,totalSample,immune-Th17; Homo_633,totalSample,immune-Gamma_delta; Homo_633,totalSample,immune-Effector_memory; Homo_633,totalSample,immune-Th1; Homo_633,totalSample,immune-DC; MCSA,totalSample,immune-DC; MCSA,totalSample,immune-Macrophage; MCSA,totalSample,immune-CD4_T; MCSA,totalSample,immune-CD4_naive; MCSA,totalSample,immune-Tr1; MCSA,totalSample,immune-nTreg; MCSA,totalSample,immune-iTreg; MCSA,totalSample,immune-Th2; MCSA,totalSample,immune-Th17; MCSA,totalSample,immune-Tfh; MCSA,totalSample,immune-CD8_naive; MCSA,totalSample,immune-Cytotoxic; MCSA,totalSample,immune-Exhausted; MCSA,totalSample,immune-MAIT; MCSA,totalSample,immune-Central_memory; ROSMAP,totalSample,immune-InfiltrationScore; SRP223445,totalSample,immune-CD4_naive; [illegible]
PARD6G	Homo_723,female,AD.vs.control; Homo_723,male,AD.vs.control; Homo_723,female,AD.vs.MCI; Homo_723,male,AD.vs.MCI; Homo_723,female,MCI.vs.control; Homo_723,male,MCI.vs.control; Homo_714,female,AD.vs.control; Homo_714,male,AD.vs.control; Homo_714,female,AD.vs.MCI; Homo_714,male,AD.vs.MCI; Homo_714,female,MCI.vs.control; Homo_714,male,MCI.vs.control; Homo_633,female,AD.vs.control; Homo_633,female,AD.vs.MCI; ROSMAP,female,AD.vs.control; ROSMAP,female,AD.vs.MCI; MCSA,MCI,age; Emory,totalSample,immune-CD8_naive; Homo_723,totalSample,immune-Th17; Homo_723,totalSample,immune-Exhausted; Homo_723,totalSample,immune-CD4_naive; Homo_723,totalSample,immune-CD4_T; Homo_723,totalSample,immune-Tfh; Homo_723,totalSample,immune-InfiltrationScore; Homo_723,totalSample,immune-nTreg; Homo_723,totalSample,immune-CD8_T; Homo_723,totalSample,immune-Central_memory; Homo_723,totalSample,immune-Cytotoxic; Homo_714,totalSample,immune-InfiltrationScore; Homo_714,totalSample,immune-CD4_naive; Homo_714,totalSample,immune-Th17; Homo_714,totalSample,immune-Exhausted; Homo_633,totalSample,immune-Tfh; Homo_633,totalSample,immune-InfiltrationScore; Homo_633,totalSample,immune-CD4_T; Homo_633,totalSample,immune-Effector_memory; Homo_633,totalSample,immune-CD4_naive; Homo_633,totalSample,immune-Th2; Homo_633,totalSample,immune-Central_memory; MCSA,totalSample,immune-DC; MCSA,totalSample,immune-CD4_T; MCSA,totalSample,immune-CD4_naive; MCSA,totalSample,immune-Tr1; MCSA,totalSample,immune-nTreg; MCSA,totalSample,immune-iTreg; MCSA,totalSample,immune-Th17; MCSA,totalSample,immune-Tfh;
ZNF711	Homo_723,female,AD.vs.control; Homo_723,male,AD.vs.control; Homo_723,female,AD.vs.MCI; Homo_723,male,AD.vs.MCI; Homo_723,female,MCI.vs.control; Homo_723,male,MCI.vs.control; Homo_714,male,AD.vs.control; Homo_714,female,AD.vs.MCI; Homo_714,male,AD.vs.MCI; Homo_714,male,MCI.vs.control; Homo_633,female,AD.vs.control; Homo_633,female,AD.vs.MCI; ROSMAP,female,AD.vs.control; ROSMAP,AD,MMSE; Emory,totalSample,immune-Macrophage; Emory,totalSample,immune-CD4_T; Emory,totalSample,immune-Tr1; Emory,totalSample,immune-iTreg; Emory,totalSample,immune-InfiltrationScore; Homo_723,totalSample,immune-CD4_naive; Homo_723,totalSample,immune-CD4_T; Homo_723,totalSample,immune-Tr1; Homo_723,totalSample,immune-Tfh; Homo_723,totalSample,immune-Th2; Homo_723,totalSample,immune-Central_memory; Homo_723,totalSample,immune-iTreg; Homo_723,totalSample,immune-Th17; Homo_723,totalSample,immune-Th1; Homo_723,totalSample,immune-nTreg; Homo_723,totalSample,immune-Exhausted; Homo_723,totalSample,immune-Cytotoxic; Homo_723,totalSample,immune-InfiltrationScore; Homo_723,totalSample,immune-Neutrophil; Homo_723,totalSample,immune-Effector_memory; Homo_723,totalSample,immune-NKT; Homo_714,totalSample,immune-CD4_naive; Homo_714,totalSample,immune-Tfh; Homo_714,totalSample,immune-CD4_T; Homo_714,totalSample,immune-Tr1; Homo_714,totalSample,immune-Central_memory; Homo_714,totalSample,immune-Th2; Homo_714,totalSample,immune-iTreg; Homo_714,totalSample,immune-Effector_memory; Homo_714,totalSample,immune-InfiltrationScore; Homo_714,totalSample,immune-NKT; Homo_714,totalSample,immune-Th1; Homo_714,totalSample,immune-Th17; Homo_633,totalSample,immune-Tfh; Homo_633,totalSample,immune-CD4_T; Homo_633,totalSample,immune-CD4_naive; Homo_633,totalSample,immune-Central_memory; Homo_633,totalSample,immune-Effector_memory; Homo_633,totalSample,immune-Tr1; Homo_633,totalSample,immune-Th2; Homo_633,totalSample,immune-NKT; Homo_633,totalSample,immune-iTreg; Homo_633,totalSample,immune-InfiltrationScore; Homo_633,totalSample,immune-DC; Homo_633,totalSample,immune-Exhausted; Homo_633,totalSample,immune-Th17; Homo_633,totalSample,immune-nTreg; Homo_633,totalSample,immune-Gamma_delta; MCSA,totalSample,immune-DC; MCSA,totalSample,immune-Bcell; MCSA,totalSample,immune-Monocyte; MCSA,totalSample,immune-Macrophage; MCSA,totalSample,immune-CD4_T; MCSA,totalSample,immune-CD4_naive; MCSA,totalSample,immune-Tr1; MCSA,totalSample,immune-nTreg; MCSA,totalSample,immune-iTreg; MCSA,totalSample,immune-Tfh; MCSA,totalSample,immune-CD8_naive; MCSA,totalSample,immune-Cytotoxic; MCSA,totalSample,immune-Exhausted; MCSA,totalSample,immune-MAIT; MCSA,totalSample,immune-Central_memory; ROSMAP,totalSample,immune-InfiltrationScore; SRP223445,totalSample,immune-Neutrophil; ROSMAP,male-includeAge90+,survival
C14orf132	Homo_723,female,AD.vs.control; Homo_723,male,AD.vs.control; Homo_723,female,AD.vs.MCI; Homo_723,male,AD.vs.MCI; Homo_714,female,AD.vs.control; Homo_714,male,AD.vs.control; Homo_714,female,AD.vs.MCI; Homo_714,male,AD.vs.MCI; Homo_633,female,AD.vs.control; Homo_633,female,AD.vs.MCI; MCSA,totalSample,age; MCSA,control,age; Emory,totalSample,immune-Macrophage; Emory,totalSample,immune-CD4_T; Emory,totalSample,immune-Gamma_delta; Emory,totalSample,immune-CD4_naive; Emory,totalSample,immune-Tr1; Emory,totalSample,immune-nTreg; Emory,totalSample,immune-iTreg; Emory,totalSample,immune-Th17; Emory,totalSample,immune-Tfh; Emory,totalSample,immune-CD8_naive; Emory,totalSample,immune-Cytotoxic; Emory,totalSample,immune-Central_memory; Homo_723,totalSample,immune-Central_memory; Homo_723,totalSample,immune-nTreg; Homo_723,totalSample,immune-CD4_naive; Homo_723,totalSample,immune-CD4_T; Homo_723,totalSample,immune-Tfh; Homo_723,totalSample,immune-Tr1; Homo_723,totalSample,immune-iTreg; Homo_723,totalSample,immune-Th2; Homo_723,totalSample,immune-Th17; Homo_723,totalSample,immune-Th1; Homo_723,totalSample,immune-Neutrophil; Homo_723,totalSample,immune-Cytotoxic; Homo_714,totalSample,immune-CD4_naive; Homo_714,totalSample,immune-nTreg; Homo_714,totalSample,immune-Central_memory; Homo_714,totalSample,immune-Tfh; Homo_714,totalSample,immune-CD4_T; Homo_714,totalSample,immune-Tr1; Homo_714,totalSample,immune-iTreg; Homo_714,totalSample,immune-Th1; Homo_714,totalSample,immune-Th17; Homo_714,totalSample,immune-InfiltrationScore; Homo_714,totalSample,immune-Th2; Homo_714,totalSample,immune-Effector_memory; Homo_633,totalSample,immune-Tfh; Homo_633,totalSample,immune-CD4_T; Homo_633,totalSample,immune-Tr1; Homo_633,totalSample,immune-nTreg; Homo_633,totalSample,immune-Central_memory; Homo_633,totalSample,immune-CD4_naive; Homo_633,totalSample,immune-iTreg; Homo_633,totalSample,immune-Th2; Homo_633,totalSample,immune-Effector_memory; Homo_633,totalSample,immune-Gamma_delta; Homo_633,totalSample,immune-NKT; Homo_633,totalSample,immune-InfiltrationScore; MCSA,totalSample,immune-Macrophage; MCSA,totalSample,immune-CD4_T; MCSA,totalSample,immune-Gamma_delta; MCSA,totalSample,immune-CD4_naive; MCSA,totalSample,immune-Tr1; MCSA,totalSample,immune-nTreg; MCSA,totalSample,immune-iTreg; MCSA,totalSample,immune-Th17; MCSA,totalSample,immune-Tfh; MCSA,totalSample,immune-CD8_naive; MCSA,totalSample,immune-Exhausted; MCSA,totalSample,immune-MAIT; MCSA,totalSample,immune-Central_memory; MCSA,totalSample,immune-Effector_memory; [illegible]

LAPTM4B	Homo_723,female,AD.vs.control; Homo_723,male,AD.vs.control; Homo_723,female,AD.vs.MCI; Homo_723,male,AD.vs.MCI; Homo_714,female,AD.vs.control; Homo_714,male,AD.vs.control; Homo_714,female,AD.vs.MCI; Homo_714,male,AD.vs.MCI; Homo_633,female,AD.vs.control; Homo_633,female,AD.vs.MCI; ROSMAP,female,AD.vs.control; ROSMAP,MCI,male.vs.female; MCSA,totalSample,age; MCSA,control,age; ROSMAP,AD,last.visit.age; ROSMAP,AD,sample.age; Emory,totalSample,immune-CD4_T; Emory,totalSample,immune-Tr1; Emory,totalSample,immune-iTreg; Emory,totalSample,immune-Tfh; Emory,totalSample,immune-MAIT; Emory,totalSample,immune-Central_memory; Homo_723,totalSample,immune-CD4_T; Homo_723,totalSample,immune-Th17; Homo_723,totalSample,immune-Central_memory; Homo_723,totalSample,immune-CD4_naive; Homo_723,totalSample,immune-nTreg; Homo_723,totalSample,immune-Tr1; Homo_723,totalSample,immune-Th2; Homo_723,totalSample,immune-Tfh; Homo_723,totalSample,immune-iTreg; Homo_723,totalSample,immune-Cytotoxic; Homo_723,totalSample,immune-Gamma_delta; Homo_723,totalSample,immune-Th1; Homo_723,totalSample,immune-Exhausted; Homo_714,totalSample,immune-Central_memory; Homo_714,totalSample,immune-Tfh; Homo_714,totalSample,immune-CD4_T; Homo_714,totalSample,immune-CD4_naive; Homo_714,totalSample,immune-Th17; Homo_714,totalSample,immune-Tr1; Homo_714,totalSample,immune-nTreg; Homo_714,totalSample,immune-Th2; Homo_714,totalSample,immune-Gamma_delta; Homo_714,totalSample,immune-iTreg; Homo_714,totalSample,immune-Cytotoxic; Homo_714,totalSample,immune-Exhausted; Homo_714,totalSample,immune-Th1; Homo_633,totalSample,immune-Tfh; Homo_633,totalSample,immune-CD4_T; Homo_633,totalSample,immune-Central_memory; Homo_633,totalSample,immune-Tr1; Homo_633,totalSample,immune-Th2; Homo_633,totalSample,immune-CD4_naive; Homo_633,totalSample,immune-nTreg; Homo_633,totalSample,immune-iTreg; Homo_633,totalSample,immune-Gamma_delta; Homo_633,totalSample,immune-Th17; Homo_633,totalSample,immune-Effector_memory; Homo_633,totalSample,immune-Exhausted; Homo_633,totalSample,immune-InfiltrationScore; MCSA,totalSample,immune-CD4_T; MCSA,totalSample,immune-Gamma_delta; MCSA,totalSample,immune-CD4_naive; MCSA,totalSample,immune-Tr1; MCSA,totalSample,immune-nTreg; MCSA,totalSample,immune-iTreg; MCSA,totalSample,immune-Th17; MCSA,totalSample,immune-Tfh; MCSA,totalSample,immune-CD8_naive; MCSA,totalSample,immune-Cytotoxic; MCSA,totalSample,immune-Exhausted; MCSA,totalSample,immune-Central_memory; SRP223445,totalSample,immune-CD4_naive; [illegible]
SERPINB2	Homo_723,female,AD.vs.control; Homo_723,male,AD.vs.control; Homo_723,female,AD.vs.MCI; Homo_723,male,AD.vs.MCI; Homo_714,female,AD.vs.control; Homo_714,male,AD.vs.control; Homo_714,female,AD.vs.MCI; Homo_714,male,AD.vs.MCI; Homo_633,female,AD.vs.control; Homo_633,female,AD.vs.MCI; ROSMAP,male,AD.vs.control; ROSMAP,AD,male.vs.female; Emory,MCI,age; MCSA,MCI,age; Emory,totalSample,immune-Monocyte; Emory,totalSample,immune-Neutrophil; Emory,totalSample,immune-CD8_T; Emory,totalSample,immune-Gamma_delta; Emory,totalSample,immune-Tfh; Emory,totalSample,immune-CD8_naive; Emory,totalSample,immune-Exhausted; Homo_723,totalSample,immune-Monocyte; Homo_723,totalSample,immune-DC; Homo_723,totalSample,immune-Gamma_delta; Homo_723,totalSample,immune-Neutrophil; Homo_723,totalSample,immune-Th2; Homo_723,totalSample,immune-InfiltrationScore; Homo_723,totalSample,immune-Bcell; Homo_714,totalSample,immune-Monocyte; Homo_714,totalSample,immune-Th2; Homo_714,totalSample,immune-Gamma_delta; Homo_714,totalSample,immune-Tfh; Homo_633,totalSample,immune-Monocyte; Homo_633,totalSample,immune-Exhausted; Homo_633,totalSample,immune-Gamma_delta; Homo_633,totalSample,immune-Th2; Homo_633,totalSample,immune-Macrophage; Homo_633,totalSample,immune-CD8_T; MCSA,totalSample,immune-DC; MCSA,totalSample,immune-Monocyte; MCSA,totalSample,immune-Neutrophil; MCSA,totalSample,immune-CD8_T; MCSA,totalSample,immune-iTreg; MCSA,totalSample,immune-Cytotoxic; MCSA,totalSample,immune-Exhausted; MCSA,totalSample,immune-Effector_memory; MCSA,totalSample,immune-InfiltrationScore; ROSMAP,totalSample,immune-Th17;
HGD	Homo_723,female,AD.vs.control; Homo_723,male,AD.vs.control; Homo_723,female,AD.vs.MCI; Homo_723,male,AD.vs.MCI; Homo_723,female,MCI.vs.control; Homo_723,male,MCI.vs.control; Homo_714,female,AD.vs.control; Homo_714,male,AD.vs.control; Homo_714,female,AD.vs.MCI; Homo_714,male,AD.vs.MCI; Homo_714,female,MCI.vs.control; Homo_714,male,MCI.vs.control; Homo_633,female,AD.vs.control; Homo_633,female,AD.vs.MCI; ROSMAP,female,AD.vs.control; ROSMAP,male,AD.vs.control; ROSMAP,totalSample,age; ROSMAP,AD,MMSE; ROSMAP,totalSample,MMSE; Homo_723,totalSample,immune-Th17; Homo_723,totalSample,immune-Exhausted; Homo_723,totalSample,immune-Monocyte; Homo_723,totalSample,immune-CD8_T; Homo_723,totalSample,immune-CD8_naive; Homo_714,totalSample,immune-Th17; Homo_714,totalSample,immune-Monocyte; Homo_714,totalSample,immune-Exhausted; Homo_633,totalSample,immune-Monocyte; Homo_633,totalSample,immune-Th2; Homo_633,totalSample,immune-Th17; ROSMAP,totalSample,immune-CD4_T; ROSMAP,totalSample,immune-nTreg; ROSMAP,totalSample,immune-Th17; ROSMAP,totalSample,immune-Central_memory; ROSMAP,totalSample,immune-InfiltrationScore; SRP223445,totalSample,immune-CD4_naive; SRP223445,totalSample,immune-Exhausted; SRP223445,totalSample,immune-InfiltrationScore; ROSMAP,male-includeAge90+,survival
CAPN3	Homo_723,female,AD.vs.control; Homo_723,male,AD.vs.control; Homo_723,female,AD.vs.MCI; Homo_723,male,AD.vs.MCI; Homo_723,female,MCI.vs.control; Homo_723,male,MCI.vs.control; Homo_714,female,AD.vs.control; Homo_714,male,AD.vs.control; Homo_714,female,AD.vs.MCI; Homo_714,male,AD.vs.MCI; Homo_714,female,MCI.vs.control; Homo_714,male,MCI.vs.control; Homo_633,female,AD.vs.control; Homo_633,female,AD.vs.MCI; Homo_633,female,MCI.vs.control; Homo_633,male,MCI.vs.control; MCSA,totalSample,age; MCSA,control,age; Emory,totalSample,immune-CD4_T; Emory,totalSample,immune-Gamma_delta; Emory,totalSample,immune-Tr1; Emory,totalSample,immune-iTreg; Emory,totalSample,immune-InfiltrationScore; Homo_723,totalSample,immune-Th2; Homo_723,totalSample,immune-Effector_memory; Homo_723,totalSample,immune-Tfh; Homo_723,totalSample,immune-Monocyte; Homo_723,totalSample,immune-InfiltrationScore; Homo_723,totalSample,immune-iTreg; Homo_723,totalSample,immune-CD4_T; Homo_714,totalSample,immune-Th2; Homo_714,totalSample,immune-Tfh; Homo_714,totalSample,immune-Effector_memory; Homo_714,totalSample,immune-CD4_T; Homo_714,totalSample,immune-nTreg; Homo_714,totalSample,immune-Central_memory; Homo_633,totalSample,immune-Th2; Homo_633,totalSample,immune-Effector_memory; Homo_633,totalSample,immune-Tfh; Homo_633,totalSample,immune-Th17; Homo_633,totalSample,immune-nTreg; Homo_633,totalSample,immune-CD4_T; Homo_633,totalSample,immune-Central_memory; Homo_633,totalSample,immune-InfiltrationScore; Homo_633,totalSample,immune-Exhausted; MCSA,totalSample,immune-DC; MCSA,totalSample,immune-Monocyte; MCSA,totalSample,immune-Neutrophil; MCSA,totalSample,immune-CD4_T; MCSA,totalSample,immune-Gamma_delta; MCSA,totalSample,immune-CD4_naive; MCSA,totalSample,immune-Tr1; MCSA,totalSample,immune-nTreg; MCSA,totalSample,immune-Th17; MCSA,totalSample,immune-Tfh; MCSA,totalSample,immune-Central_memory; MCSA,totalSample,immune-[illegible]
VSIG2	Homo_723,female,AD.vs.control; Homo_723,male,AD.vs.control; Homo_723,female,AD.vs.MCI; Homo_723,male,AD.vs.MCI; Homo_714,female,AD.vs.control; Homo_714,male,AD.vs.control; Homo_714,female,AD.vs.MCI; Homo_714,male,AD.vs.MCI; Homo_633,female,AD.vs.control; Homo_633,female,AD.vs.MCI; ROSMAP,male,AD.vs.control; MCSA,MCI,age; ROSMAP,AD,last.visit.age; ROSMAP,MCI,MMSE; Homo_723,totalSample,immune-DC; Homo_714,totalSample,immune-DC; Homo_714,totalSample,immune-Th2; Homo_714,totalSample,immune-Effector_memory; Homo_633,totalSample,immune-DC; MCSA,totalSample,immune-DC; MCSA,totalSample,immune-Monocyte; MCSA,totalSample,immune-iTreg; MCSA,totalSample,immune-Cytotoxic; MCSA,totalSample,immune-Exhausted; SRP223445,totalSample,immune-CD4_naive; SRP223445,totalSample,immune-Exhausted
HMGA2	Homo_723,female,AD.vs.control; Homo_723,female,AD.vs.MCI; Homo_723,male,AD.vs.MCI; Homo_723,AD,male.vs.female; Homo_714,female,AD.vs.control; Homo_714,female,AD.vs.MCI; Homo_714,male,AD.vs.MCI; ROSMAP,female,AD.vs.control; ROSMAP,male,AD.vs.control; ROSMAP,female,AD.vs.MCI; ROSMAP,male,AD.vs.MCI; ROSMAP,MCI,last.visit.age; Homo_723,totalSample,immune-Exhausted; ROSMAP,female-excludeAge90+,survival; ROSMAP,female-includeAge90+,survival; ROSMAP,male and female-excludeAge90+,survival
BMP6	Homo_723,female,AD.vs.control; Homo_723,male,AD.vs.control; Homo_723,female,AD.vs.MCI; Homo_723,male,AD.vs.MCI; Homo_714,female,AD.vs.control; Homo_714,male,AD.vs.control; Homo_714,female,AD.vs.MCI; Homo_714,male,AD.vs.MCI; Homo_633,female,AD.vs.control; Homo_633,female,AD.vs.MCI; ROSMAP,female,MCI.vs.control; Emory,MCI,age; Emory,totalSample,immune-NKT; Emory,totalSample,immune-Th1; Homo_723,totalSample,immune-Gamma_delta; Homo_723,totalSample,immune-DC; Homo_723,totalSample,immune-Th17; Homo_723,totalSample,immune-CD8_T; Homo_714,totalSample,immune-CD8_T; Homo_714,totalSample,immune-Exhausted; Homo_714,totalSample,immune-NKT; Homo_714,totalSample,immune-Gamma_delta; Homo_714,totalSample,immune-Cytotoxic; Homo_714,totalSample,immune-Th17; Homo_633,totalSample,immune-NKT; Homo_633,totalSample,immune-Gamma_delta; Homo_633,totalSample,immune-InfiltrationScore; MCSA,totalSample,immune-Gamma_delta; MCSA,totalSample,immune-iTreg; MCSA,totalSample,immune-Th17; MCSA,totalSample,immune-Cytotoxic; MCSA,totalSample,immune-Exhausted; ROSMAP,totalSample,immune-InfiltrationScore; SRP223445,totalSample,immune-CD4_naive; SRP223445,totalSample,immune-Exhausted
TTC16	Homo_723,female,AD.vs.control; Homo_723,male,AD.vs.control; Homo_723,female,AD.vs.MCI; Homo_723,male,AD.vs.MCI; Homo_714,female,AD.vs.control; Homo_714,male,AD.vs.control; Homo_714,female,AD.vs.MCI; Homo_714,male,AD.vs.MCI; Homo_633,female,AD.vs.control; Homo_633,female,AD.vs.MCI; ROSMAP,male,AD.vs.MCI; ROSMAP,control,male.vs.female; ROSMAP,MCI,last.visit.age; Emory,totalSample,immune-Monocyte; Emory,totalSample,immune-Macrophage; Emory,totalSample,immune-Neutrophil; Emory,totalSample,immune-CD4_T; Emory,totalSample,immune-CD8_T; Emory,totalSample,immune-Gamma_delta; Emory,totalSample,immune-Tr1; Emory,totalSample,immune-Cytotoxic; Emory,totalSample,immune-Exhausted; Emory,totalSample,immune-Effector_memory; Emory,totalSample,immune-InfiltrationScore; Homo_723,totalSample,immune-Cytotoxic; Homo_723,totalSample,immune-CD8_T; Homo_723,totalSample,immune-InfiltrationScore; Homo_723,totalSample,immune-MAIT; Homo_723,totalSample,immune-Effector_memory; Homo_723,totalSample,immune-Exhausted; Homo_723,totalSample,immune-Macrophage; Homo_723,totalSample,immune-Th17; Homo_723,totalSample,immune-Tfh; Homo_723,totalSample,immune-Monocyte; Homo_723,totalSample,immune-Bcell; Homo_723,totalSample,immune-Gamma_delta; Homo_723,totalSample,immune-Th1; Homo_714,totalSample,immune-Cytotoxic; Homo_714,totalSample,immune-CD8_T; Homo_714,totalSample,immune-InfiltrationScore; Homo_714,totalSample,immune-Effector_memory; Homo_714,totalSample,immune-MAIT; Homo_714,totalSample,immune-Exhausted; Homo_714,totalSample,immune-Macrophage; Homo_714,totalSample,immune-Gamma_delta; Homo_714,totalSample,immune-Neutrophil; Homo_714,totalSample,immune-Th17; Homo_714,totalSample,immune-Monocyte; Homo_714,totalSample,immune-Bcell; Homo_714,totalSample,immune-Th1; Homo_714,totalSample,immune-Tfh; Homo_714,totalSample,immune-CD8_naive; Homo_633,totalSample,immune-InfiltrationScore; Homo_633,totalSample,immune-Cytotoxic; Homo_633,totalSample,immune-CD8_T; Homo_633,totalSample,immune-Gamma_delta; Homo_633,totalSample,immune-MAIT; Homo_633,totalSample,immune-Neutrophil; Homo_633,totalSample,immune-Effector_memory; Homo_633,totalSample,immune-Macrophage; Homo_633,totalSample,immune-Th17; Homo_633,totalSample,immune-Bcell; Homo_633,totalSample,immune-Exhausted; Homo_633,totalSample,immune-Th2; Homo_633,totalSample,immune-DC; Homo_633,totalSample,immune-Monocyte; Homo_633,totalSample,immune-Th1; MCSA,totalSample,immune-DC; MCSA,totalSample,immune-Monocyte; MCSA,totalSample,immune-Neutrophil; MCSA,totalSample,immune-CD8_T; MCSA,totalSample,immune-NKT; MCSA,totalSample,immune-Gamma_delta; MCSA,totalSample,immune-Th2; MCSA,totalSample,immune-Tfh; MCSA,totalSample,immune-Cytotoxic; MCSA,totalSample,immune-MAIT; MCSA,totalSample,immune-Central_memory; MCSA,totalSample,immune-Effector_memory; MCSA,totalSample,immune-InfiltrationScore; [illegible]
IFT81	Homo_723,female,AD.vs.control; Homo_723,male,AD.vs.control; Homo_723,female,AD.vs.MCI; Homo_723,male,AD.vs.MCI; Homo_723,female,MCI.vs.control; Homo_714,female,AD.vs.control; Homo_714,male,AD.vs.control; Homo_714,female,AD.vs.MCI; Homo_714,male,AD.vs.MCI; Homo_714,female,MCI.vs.control; Homo_633,female,AD.vs.control; Homo_633,female,AD.vs.MCI; MCSA,MCI,age; Homo_723,totalSample,immune-Exhausted; Homo_723,totalSample,immune-Cytotoxic; Homo_723,totalSample,immune-CD8_T; Homo_723,totalSample,immune-Th17; Homo_723,totalSample,immune-Th1; Homo_714,totalSample,immune-Monocyte; Homo_714,totalSample,immune-Exhausted; Homo_714,totalSample,immune-CD8_T; Homo_633,totalSample,immune-Monocyte; Homo_633,totalSample,immune-Tfh; Homo_633,totalSample,immune-Th2; Homo_633,totalSample,immune-Exhausted; Homo_633,totalSample,immune-InfiltrationScore; Homo_633,totalSample,immune-Gamma_delta; MCSA,totalSample,immune-DC; MCSA,totalSample,immune-Monocyte; MCSA,totalSample,immune-Macrophage; MCSA,totalSample,immune-CD4_T; MCSA,totalSample,immune-CD8_T; MCSA,totalSample,immune-Tr1; MCSA,totalSample,immune-nTreg; MCSA,totalSample,immune-iTreg; MCSA,totalSample,immune-Th2; MCSA,totalSample,immune-CD8_naive; MCSA,totalSample,immune-Cytotoxic; MCSA,totalSample,immune-Exhausted;
MYEF2	Homo_723,female,AD.vs.control; Homo_723,male,AD.vs.control; Homo_723,female,AD.vs.MCI; Homo_723,male,AD.vs.MCI; Homo_723,female,MCI.vs.control; Homo_723,male,MCI.vs.control; Homo_714,female,AD.vs.control; Homo_714,male,AD.vs.control; Homo_714,female,AD.vs.MCI; Homo_714,male,AD.vs.MCI; Homo_714,female,MCI.vs.control; Homo_714,male,MCI.vs.control; Homo_633,female,AD.vs.control; Homo_633,female,AD.vs.MCI; Homo_633,female,MCI.vs.control; Homo_633,male,MCI.vs.control; ROSMAP,male,AD.vs.control; Emory,totalSample,immune-CD4_T; Emory,totalSample,immune-Gamma_delta; Emory,totalSample,immune-Tr1; Emory,totalSample,immune-iTreg; Homo_723,totalSample,immune-Exhausted; Homo_723,totalSample,immune-Th17; Homo_723,totalSample,immune-CD4_naive; Homo_723,totalSample,immune-CD4_T; Homo_723,totalSample,immune-Tfh; Homo_723,totalSample,immune-Th2; Homo_723,totalSample,immune-Tr1; Homo_723,totalSample,immune-Cytotoxic; Homo_723,totalSample,immune-Central_memory; Homo_723,totalSample,immune-nTreg; Homo_723,totalSample,immune-Monocyte; Homo_723,totalSample,immune-iTreg; Homo_723,totalSample,immune-Effector_memory; Homo_723,totalSample,immune-InfiltrationScore; Homo_723,totalSample,immune-Th1; Homo_714,totalSample,immune-CD4_naive; Homo_714,totalSample,immune-Tfh; Homo_714,totalSample,immune-Th17; Homo_714,totalSample,immune-CD4_T; Homo_714,totalSample,immune-Central_memory; Homo_714,totalSample,immune-Monocyte; Homo_714,totalSample,immune-Th2; Homo_714,totalSample,immune-Tr1; Homo_714,totalSample,immune-Exhausted; Homo_714,totalSample,immune-InfiltrationScore; Homo_714,totalSample,immune-Effector_memory; Homo_714,totalSample,immune-iTreg; Homo_633,totalSample,immune-Tfh; Homo_633,totalSample,immune-Th2; Homo_633,totalSample,immune-CD4_T; Homo_633,totalSample,immune-Central_memory; Homo_633,totalSample,immune-CD4_naive; Homo_633,totalSample,immune-Effector_memory; Homo_633,totalSample,immune-Tr1; Homo_633,totalSample,immune-Monocyte; Homo_633,totalSample,immune-Th17; Homo_633,totalSample,immune-Gamma_delta; Homo_633,totalSample,immune-iTreg; Homo_633,totalSample,immune-Exhausted; Homo_633,totalSample,immune-InfiltrationScore; Homo_633,totalSample,immune-nTreg; Homo_633,totalSample,immune-DC; MCSA,totalSample,immune-DC; MCSA,totalSample,immune-Bcell; MCSA,totalSample,immune-Macrophage; MCSA,totalSample,immune-CD4_T; MCSA,totalSample,immune-CD4_naive; MCSA,totalSample,immune-Tr1; MCSA,totalSample,immune-nTreg; MCSA,totalSample,immune-iTreg; MCSA,totalSample,immune-Th17; MCSA,totalSample,immune-Tfh; MCSA,totalSample,immune-CD8_naive; MCSA,totalSample,immune-Cytotoxic; MCSA,totalSample,immune-Exhausted; MCSA,totalSample,immune-MAIT; MCSA,totalSample,immune-Central_memory; ROSMAP,totalSample,immune-InfiltrationScore; SRP223445,totalSample,immune-NK; SRP223445,totalSample,immune-Exhausted; SRP223445,totalSample,immune-[illegible]
FNBP1L	Homo_723,female,AD.vs.control; Homo_723,male,AD.vs.control; Homo_723,female,AD.vs.MCI; Homo_723,male,AD.vs.MCI; Homo_723,female,MCI.vs.control; Homo_723,male,MCI.vs.control; Homo_714,female,AD.vs.control; Homo_714,male,AD.vs.control; Homo_714,female,AD.vs.MCI; Homo_714,male,AD.vs.MCI; Homo_714,female,MCI.vs.control; Homo_714,male,MCI.vs.control; Homo_633,female,AD.vs.control; Homo_633,female,AD.vs.MCI; ROSMAP,female,AD.vs.control; ROSMAP,female,MCI.vs.control; ROSMAP,control,male.vs.female; Emory,totalSample,age; Emory,MCI,age; Emory,totalSample,immune-Macrophage; Emory,totalSample,immune-CD4_T; Emory,totalSample,immune-Tr1; Emory,totalSample,immune-iTreg; Emory,totalSample,immune-CD8_naive; Homo_723,totalSample,immune-Th17; Homo_723,totalSample,immune-Cytotoxic; Homo_723,totalSample,immune-CD4_naive; Homo_723,totalSample,immune-CD4_T; Homo_723,totalSample,immune-Central_memory; Homo_723,totalSample,immune-Exhausted; Homo_723,totalSample,immune-Th1; Homo_723,totalSample,immune-Tr1; Homo_723,totalSample,immune-CD8_T; Homo_723,totalSample,immune-Tfh; Homo_723,totalSample,immune-Gamma_delta; Homo_723,totalSample,immune-nTreg; Homo_723,totalSample,immune-NKT; Homo_723,totalSample,immune-iTreg; Homo_723,totalSample,immune-Th2; Homo_714,totalSample,immune-Th17; Homo_714,totalSample,immune-CD4_naive; Homo_714,totalSample,immune-Exhausted; Homo_714,totalSample,immune-NKT; Homo_714,totalSample,immune-Cytotoxic; Homo_714,totalSample,immune-CD4_T; Homo_714,totalSample,immune-Central_memory; Homo_714,totalSample,immune-Tfh; Homo_714,totalSample,immune-Th1; Homo_714,totalSample,immune-Gamma_delta; Homo_714,totalSample,immune-CD8_T; Homo_714,totalSample,immune-Tr1; Homo_714,totalSample,immune-iTreg; Homo_633,totalSample,immune-Tfh; Homo_633,totalSample,immune-CD4_T; Homo_633,totalSample,immune-NKT; Homo_633,totalSample,immune-Tr1; Homo_633,totalSample,immune-Central_memory; Homo_633,totalSample,immune-CD4_naive; Homo_633,totalSample,immune-iTreg; Homo_633,totalSample,immune-Gamma_delta; Homo_633,totalSample,immune-Th2; Homo_633,totalSample,immune-Th17; Homo_633,totalSample,immune-Cytotoxic; Homo_633,totalSample,immune-Exhausted; Homo_633,totalSample,immune-InfiltrationScore; Homo_633,totalSample,immune-nTreg; Homo_633,totalSample,immune-Th1; MCSA,totalSample,immune-DC; MCSA,totalSample,immune-CD4_T; MCSA,totalSample,immune-CD8_T; MCSA,totalSample,immune-Gamma_delta; MCSA,totalSample,immune-CD4_naive; MCSA,totalSample,immune-Tr1; MCSA,totalSample,immune-nTreg; MCSA,totalSample,immune-iTreg; MCSA,totalSample,immune-Th1; MCSA,totalSample,immune-Th17; MCSA,totalSample,immune-Tfh; MCSA,totalSample,immune-CD8_naive; MCSA,totalSample,immune-Cytotoxic; MCSA,totalSample,immune-Exhausted; MCSA,totalSample,immune-Central_memory; ROSMAP,totalSample,immune-InfiltrationScore
UBE2C	Homo_723,female,AD.vs.control; Homo_723,male,AD.vs.control; Homo_723,female,AD.vs.MCI; Homo_723,male,AD.vs.MCI; Homo_723,female,MCI.vs.control; Homo_714,female,AD.vs.control; Homo_714,male,AD.vs.control; Homo_714,female,AD.vs.MCI; Homo_714,male,AD.vs.MCI; Homo_633,female,AD.vs.control; Homo_633,female,AD.vs.MCI; ROSMAP,female,AD.vs.control; MCSA,totalSample,age; Homo_723,totalSample,immune-DC; Homo_723,totalSample,immune-Th17; Homo_723,totalSample,immune-CD8_T; Homo_723,totalSample,immune-Macrophage; Homo_723,totalSample,immune-MAIT; Homo_714,totalSample,immune-DC; Homo_714,totalSample,immune-CD8_T; Homo_714,totalSample,immune-Exhausted; Homo_714,totalSample,immune-CD8_naive; Homo_633,totalSample,immune-InfiltrationScore; MCSA,totalSample,immune-DC; MCSA,totalSample,immune-Monocyte; MCSA,totalSample,immune-Macrophage; MCSA,totalSample,immune-Gamma_delta; MCSA,totalSample,immune-Th17; ROSMAP,totalSample,immune-InfiltrationScore;
CDK5RAP1	Homo_723,female,AD.vs.control; Homo_723,male,AD.vs.control; Homo_723,female,AD.vs.MCI; Homo_723,male,AD.vs.MCI; Homo_714,female,AD.vs.control; Homo_714,male,AD.vs.control; Homo_714,female,AD.vs.MCI; Homo_714,male,AD.vs.MCI; Homo_633,female,AD.vs.control; Homo_633,female,AD.vs.MCI; MCSA,totalSample,age; MCSA,control,age; Emory,totalSample,immune-Macrophage; Emory,totalSample,immune-NK; Emory,totalSample,immune-Neutrophil; Emory,totalSample,immune-CD4_T; Emory,totalSample,immune-CD8_T; Emory,totalSample,immune-Tr1; Emory,totalSample,immune-iTreg; Emory,totalSample,immune-Tfh; Emory,totalSample,immune-Exhausted; Emory,totalSample,immune-MAIT; Emory,totalSample,immune-Central_memory; Emory,totalSample,immune-Effector_memory; Emory,totalSample,immune-InfiltrationScore; Homo_723,totalSample,immune-Th2; Homo_723,totalSample,immune-Tfh; Homo_723,totalSample,immune-iTreg; Homo_723,totalSample,immune-Effector_memory; Homo_723,totalSample,immune-CD4_T; Homo_723,totalSample,immune-InfiltrationScore; Homo_723,totalSample,immune-Tr1; Homo_723,totalSample,immune-CD4_naive; Homo_723,totalSample,immune-Macrophage; Homo_723,totalSample,immune-nTreg; Homo_723,totalSample,immune-Bcell; Homo_723,totalSample,immune-Central_memory; Homo_723,totalSample,immune-DC; Homo_723,totalSample,immune-CD8_T; Homo_714,totalSample,immune-Th2; Homo_714,totalSample,immune-Tfh; Homo_714,totalSample,immune-Effector_memory; Homo_714,totalSample,immune-CD4_T; Homo_714,totalSample,immune-Tr1; Homo_714,totalSample,immune-Central_memory; Homo_714,totalSample,immune-InfiltrationScore; Homo_714,totalSample,immune-CD4_naive; Homo_714,totalSample,immune-nTreg; Homo_714,totalSample,immune-iTreg; Homo_714,totalSample,immune-Macrophage; Homo_714,totalSample,immune-CD8_T; Homo_633,totalSample,immune-Tfh; Homo_633,totalSample,immune-Effector_memory; Homo_633,totalSample,immune-Th2; Homo_633,totalSample,immune-CD4_T; Homo_633,totalSample,immune-InfiltrationScore; Homo_633,totalSample,immune-CD4_naive; Homo_633,totalSample,immune-Central_memory; Homo_633,totalSample,immune-Tr1; Homo_633,totalSample,immune-nTreg; Homo_633,totalSample,immune-Bcell; Homo_633,totalSample,immune-Neutrophil; Homo_633,totalSample,immune-Th17; Homo_633,totalSample,immune-iTreg; MCSA,totalSample,immune-Monocyte; MCSA,totalSample,immune-Macrophage; MCSA,totalSample,immune-Neutrophil; MCSA,totalSample,immune-CD4_T; MCSA,totalSample,immune-CD4_naive; MCSA,totalSample,immune-Tr1; MCSA,totalSample,immune-nTreg; MCSA,totalSample,immune-iTreg; MCSA,totalSample,immune-[illegible]
ERMN	Homo_723,female,AD.vs.control; Homo_723,male,AD.vs.control; Homo_723,female,AD.vs.MCI; Homo_723,male,AD.vs.MCI; Homo_723,female,MCI.vs.control; Homo_723,male,MCI.vs.control; Homo_714,female,AD.vs.control; Homo_714,male,AD.vs.control; Homo_714,female,AD.vs.MCI; Homo_714,male,AD.vs.MCI; Homo_714,female,MCI.vs.control; Homo_714,male,MCI.vs.control; Homo_633,female,AD.vs.control; Homo_633,female,AD.vs.MCI; Homo_633,female,MCI.vs.control; Homo_633,male,MCI.vs.control; ROSMAP,female,AD.vs.control; MCSA,totalSample,age; MCSA,control,age; Emory,totalSample,immune-InfiltrationScore; Homo_723,totalSample,immune-Th2; Homo_723,totalSample,immune-Tfh; Homo_723,totalSample,immune-iTreg; Homo_723,totalSample,immune-DC; Homo_723,totalSample,immune-Monocyte; Homo_723,totalSample,immune-CD4_T; Homo_723,totalSample,immune-Effector_memory; Homo_723,totalSample,immune-Tr1; Homo_723,totalSample,immune-CD4_naive; Homo_723,totalSample,immune-Macrophage; Homo_723,totalSample,immune-Gamma_delta; Homo_723,totalSample,immune-InfiltrationScore; Homo_723,totalSample,immune-Bcell; Homo_714,totalSample,immune-Th2; Homo_714,totalSample,immune-Tfh; Homo_714,totalSample,immune-CD4_T; Homo_714,totalSample,immune-Effector_memory; Homo_714,totalSample,immune-Tr1; Homo_714,totalSample,immune-Central_memory; Homo_714,totalSample,immune-Monocyte; Homo_714,totalSample,immune-iTreg; Homo_714,totalSample,immune-CD4_naive; Homo_714,totalSample,immune-Gamma_delta; Homo_714,totalSample,immune-DC; Homo_633,totalSample,immune-Th2; Homo_633,totalSample,immune-Tfh; Homo_633,totalSample,immune-CD4_T; Homo_633,totalSample,immune-Monocyte; Homo_633,totalSample,immune-Th17; Homo_633,totalSample,immune-Effector_memory; Homo_633,totalSample,immune-Central_memory; Homo_633,totalSample,immune-CD4_naive; Homo_633,totalSample,immune-Exhausted; Homo_633,totalSample,immune-Gamma_delta; Homo_633,totalSample,immune-Tr1; Homo_633,totalSample,immune-InfiltrationScore; MCSA,totalSample,immune-DC; MCSA,totalSample,immune-Macrophage; MCSA,totalSample,immune-CD4_T; MCSA,totalSample,immune-CD8_T; MCSA,totalSample,immune-Gamma_delta; MCSA,totalSample,immune-CD4_naive; MCSA,totalSample,immune-Tr1; MCSA,totalSample,immune-nTreg; MCSA,totalSample,immune-iTreg; MCSA,totalSample,immune-Th17; MCSA,totalSample,immune-Tfh; MCSA,totalSample,immune-CD8_naive; MCSA,totalSample,immune-Cytotoxic; MCSA,totalSample,immune-Exhausted; MCSA,totalSample,immune-MAIT; MCSA,totalSample,immune-Central_memory; ROSMAP,male and female-excludeAge90+,survival; ROSMAP,male and female-includeAge90+,survival

TPRG1	Homo_723,female,AD.vs.control; Homo_723,male,AD.vs.control; Homo_723,female,AD.vs.MCI; Homo_723,male,AD.vs.MCI; Homo_714,female,AD.vs.control; Homo_714,male,AD.vs.control; Homo_714,female,AD.vs.MCI; Homo_714,male,AD.vs.MCI; Homo_633,female,AD.vs.control; Homo_633,female,AD.vs.MCI; ROSMAP,AD,last.visit.age; Emory,totalSample,immune-CD8_T; Emory,totalSample,immune-Gamma_delta; Emory,totalSample,immune-CD4_naive; Emory,totalSample,immune-nTreg; Emory,totalSample,immune-Cytotoxic; Emory,totalSample,immune-Exhausted; Emory,totalSample,immune-Effector_memory; Emory,totalSample,immune-InfiltrationScore; Homo_723,totalSample,immune-MAIT; Homo_723,totalSample,immune-InfiltrationScore; Homo_723,totalSample,immune-Cytotoxic; Homo_723,totalSample,immune-Effector_memory; Homo_723,totalSample,immune-Tfh; Homo_723,totalSample,immune-Bcell; Homo_723,totalSample,immune-Macrophage; Homo_723,totalSample,immune-DC; Homo_723,totalSample,immune-CD8_T; Homo_714,totalSample,immune-InfiltrationScore; Homo_714,totalSample,immune-MAIT; Homo_714,totalSample,immune-Cytotoxic; Homo_714,totalSample,immune-CD8_T; Homo_714,totalSample,immune-Effector_memory; Homo_714,totalSample,immune-Bcell; Homo_714,totalSample,immune-Tfh; Homo_714,totalSample,immune-Exhausted; Homo_714,totalSample,immune-Macrophage; Homo_633,totalSample,immune-InfiltrationScore; Homo_633,totalSample,immune-MAIT; Homo_633,totalSample,immune-Cytotoxic; Homo_633,totalSample,immune-CD8_T; Homo_633,totalSample,immune-Bcell; Homo_633,totalSample,immune-Monocyte; Homo_633,totalSample,immune-Effector_memory; Homo_633,totalSample,immune-Neutrophil; Homo_633,totalSample,immune-Gamma_delta; Homo_633,totalSample,immune-Macrophage; MCSA,totalSample,immune-DC; MCSA,totalSample,immune-Macrophage; MCSA,totalSample,immune-CD8_T; MCSA,totalSample,immune-Gamma_delta; MCSA,totalSample,immune-Cytotoxic; MCSA,totalSample,immune-MAIT; [illegible]
ICA1	Homo_723,female,AD.vs.control; Homo_723,male,AD.vs.control; Homo_723,female,AD.vs.MCI; Homo_723,male,AD.vs.MCI; Homo_723,female,MCI.vs.control; Homo_723,male,MCI.vs.control; Homo_714,female,AD.vs.control; Homo_714,male,AD.vs.control; Homo_714,female,AD.vs.MCI; Homo_714,male,AD.vs.MCI; Homo_714,female,MCI.vs.control; Homo_714,male,MCI.vs.control; Homo_633,female,AD.vs.control; Homo_633,female,AD.vs.MCI; ROSMAP,AD,MMSE; Homo_723,totalSample,immune-Th17; Homo_723,totalSample,immune-Exhausted; Homo_723,totalSample,immune-CD8_T; Homo_723,totalSample,immune-InfiltrationScore; Homo_723,totalSample,immune-Cytotoxic; Homo_723,totalSample,immune-CD4_naive; Homo_714,totalSample,immune-Th17; Homo_714,totalSample,immune-Exhausted; Homo_714,totalSample,immune-InfiltrationScore; Homo_714,totalSample,immune-Monocyte; Homo_633,totalSample,immune-InfiltrationScore; Homo_633,totalSample,immune-Tfh; Homo_633,totalSample,immune-Th2; Homo_633,totalSample,immune-Monocyte; Homo_633,totalSample,immune-Gamma_delta; Homo_633,totalSample,immune-Exhausted; MCSA,totalSample,immune-Th17; ROSMAP,totalSample,immune-InfiltrationScore
TFIP11	Homo_723,female,AD.vs.control; Homo_723,male,AD.vs.control; Homo_723,female,AD.vs.MCI; Homo_723,male,AD.vs.MCI; Homo_714,female,AD.vs.control; Homo_714,male,AD.vs.control; Homo_714,female,AD.vs.MCI; Homo_714,male,AD.vs.MCI; Homo_633,female,AD.vs.control; Homo_633,female,AD.vs.MCI; Emory,MCI,age; MCSA,totalSample,age; MCSA,control,age; ROSMAP,MCI,sample.age; Emory,totalSample,immune-CD8_naive; Emory,totalSample,immune-InfiltrationScore; Homo_723,totalSample,immune-InfiltrationScore; Homo_723,totalSample,immune-Effector_memory; Homo_723,totalSample,immune-Th2; Homo_723,totalSample,immune-Tfh; Homo_723,totalSample,immune-iTreg; Homo_723,totalSample,immune-Bcell; Homo_723,totalSample,immune-CD4_T; Homo_723,totalSample,immune-Tr1; Homo_723,totalSample,immune-CD4_naive; Homo_723,totalSample,immune-Monocyte; Homo_714,totalSample,immune-Th2; Homo_714,totalSample,immune-Tfh; Homo_714,totalSample,immune-Effector_memory; Homo_714,totalSample,immune-InfiltrationScore; Homo_714,totalSample,immune-CD4_T; Homo_714,totalSample,immune-Central_memory; Homo_714,totalSample,immune-nTreg; Homo_714,totalSample,immune-Tr1; Homo_633,totalSample,immune-InfiltrationScore; Homo_633,totalSample,immune-Th2; Homo_633,totalSample,immune-Effector_memory; Homo_633,totalSample,immune-Tfh; Homo_633,totalSample,immune-Exhausted; Homo_633,totalSample,immune-CD4_T; Homo_633,totalSample,immune-Central_memory; Homo_633,totalSample,immune-nTreg; Homo_633,totalSample,immune-Th17; Homo_633,totalSample,immune-CD4_naive; MCSA,totalSample,immune-Monocyte; MCSA,totalSample,immune-CD4_T; MCSA,totalSample,immune-Gamma_delta; MCSA,totalSample,immune-CD4_naive; MCSA,totalSample,immune-Tr1; MCSA,totalSample,immune-nTreg; MCSA,totalSample,immune-iTreg; MCSA,totalSample,immune-Th2; MCSA,totalSample,immune-Th17; MCSA,totalSample,immune-Tfh; MCSA,totalSample,immune-Exhausted; MCSA,totalSample,immune-Central_memory; MCSA,totalSample,immune-Effector_memory; MCSA,totalSample,immune-InfiltrationScore; SRP223445,totalSample,immune-NK; SRP223445,totalSample,immune-CD4_naive; SRP223445,totalSample,immune-Exhausted; SRP223445,totalSample,immune-InfiltrationScore
EIF4G3	Homo_723,female,AD.vs.control; Homo_723,male,AD.vs.control; Homo_723,female,AD.vs.MCI; Homo_723,male,AD.vs.MCI; Homo_723,female,MCI.vs.control; Homo_723,male,MCI.vs.control; Homo_714,female,AD.vs.control; Homo_714,male,AD.vs.control; Homo_714,female,AD.vs.MCI; Homo_714,male,AD.vs.MCI; Homo_714,female,MCI.vs.control; Homo_714,male,MCI.vs.control; Homo_633,female,AD.vs.control; Homo_633,female,AD.vs.MCI; ROSMAP,male,AD.vs.control; ACOM,totalSample,age; Emory,totalSample,age; Emory,MCI,age; Emory,totalSample,immune-CD8_T; Emory,totalSample,immune-Gamma_delta; Emory,totalSample,immune-Th1; Emory,totalSample,immune-Tfh; Emory,totalSample,immune-CD8_naive; Emory,totalSample,immune-InfiltrationScore; Homo_723,totalSample,immune-Th17; Homo_723,totalSample,immune-CD8_T; Homo_723,totalSample,immune-Exhausted; Homo_723,totalSample,immune-Cytotoxic; Homo_723,totalSample,immune-CD4_naive; Homo_723,totalSample,immune-InfiltrationScore; Homo_723,totalSample,immune-CD4_T; Homo_723,totalSample,immune-Central_memory; Homo_723,totalSample,immune-NK; Homo_714,totalSample,immune-Exhausted; Homo_714,totalSample,immune-InfiltrationScore; Homo_714,totalSample,immune-Th17; Homo_714,totalSample,immune-CD4_naive; Homo_714,totalSample,immune-CD8_T; Homo_714,totalSample,immune-NKT; Homo_714,totalSample,immune-Cytotoxic; Homo_633,totalSample,immune-InfiltrationScore; Homo_633,totalSample,immune-Tfh; Homo_633,totalSample,immune-CD4_T; Homo_633,totalSample,immune-Effector_memory; Homo_633,totalSample,immune-CD4_naive; Homo_633,totalSample,immune-Th2; Homo_633,totalSample,immune-NKT; Homo_633,totalSample,immune-Tr1; Homo_633,totalSample,immune-Gamma_delta; Homo_633,totalSample,immune-Exhausted; Homo_633,totalSample,immune-iTreg; Homo_633,totalSample,immune-Th17; Homo_633,totalSample,immune-Central_memory; MCSA,totalSample,immune-CD4_T; MCSA,totalSample,immune-CD8_T; MCSA,totalSample,immune-Gamma_delta; MCSA,totalSample,immune-CD4_naive; MCSA,totalSample,immune-Tr1; MCSA,totalSample,immune-nTreg; MCSA,totalSample,immune-iTreg; MCSA,totalSample,immune-Th17; MCSA,totalSample,immune-Tfh; MCSA,totalSample,immune-Cytotoxic; MCSA,totalSample,immune-Exhausted; MCSA,totalSample,immune-Central_memory; [illegible]
PLEKHA5	Homo_723,female,AD.vs.control; Homo_723,male,AD.vs.control; Homo_723,female,AD.vs.MCI; Homo_723,male,AD.vs.MCI; Homo_723,female,MCI.vs.control; Homo_723,male,MCI.vs.control; Homo_714,female,AD.vs.control; Homo_714,male,AD.vs.control; Homo_714,female,AD.vs.MCI; Homo_714,male,AD.vs.MCI; Homo_714,male,MCI.vs.control; Homo_633,female,AD.vs.control; Homo_633,female,AD.vs.MCI; MCSA,MCI,age; ROSMAP,MCI,sample.age; Emory,totalSample,immune-Cytotoxic; Emory,totalSample,immune-Effector_memory; Emory,totalSample,immune-InfiltrationScore; Homo_723,totalSample,immune-CD4_naive; Homo_723,totalSample,immune-InfiltrationScore; Homo_723,totalSample,immune-Neutrophil; Homo_723,totalSample,immune-Th17; Homo_723,totalSample,immune-CD4_T; Homo_723,totalSample,immune-Tfh; Homo_723,totalSample,immune-Exhausted; Homo_723,totalSample,immune-NK; Homo_723,totalSample,immune-NKT; Homo_714,totalSample,immune-InfiltrationScore; Homo_714,totalSample,immune-CD4_naive; Homo_714,totalSample,immune-NKT; Homo_714,totalSample,immune-Neutrophil; Homo_714,totalSample,immune-Effector_memory; Homo_714,totalSample,immune-Bcell; Homo_633,totalSample,immune-InfiltrationScore; Homo_633,totalSample,immune-Effector_memory; Homo_633,totalSample,immune-Tfh; Homo_633,totalSample,immune-CD4_naive; Homo_633,totalSample,immune-MAIT; Homo_633,totalSample,immune-NKT; Homo_633,totalSample,immune-CD4_T; Homo_633,totalSample,immune-Bcell; Homo_633,totalSample,immune-Neutrophil; Homo_633,totalSample,immune-Central_memory; MCSA,totalSample,immune-DC; MCSA,totalSample,immune-Macrophage; MCSA,totalSample,immune-CD4_T; MCSA,totalSample,immune-NKT; MCSA,totalSample,immune-Gamma_delta; MCSA,totalSample,immune-CD4_naive; MCSA,totalSample,immune-Tr1; MCSA,totalSample,immune-nTreg; MCSA,totalSample,immune-iTreg; MCSA,totalSample,immune-Tfh; MCSA,totalSample,immune-CD8_naive; MCSA,totalSample,immune-Exhausted; MCSA,totalSample,immune-MAIT; MCSA,totalSample,immune-Central_memory; MCSA,totalSample,immune-Effector_memory; ROSMAP,totalSample,immune-InfiltrationScore; SRP223445,totalSample,immune-CD4_naive; ROSMAP,male and female-excludeAge90+,survival
ACSS1	Homo_723,female,AD.vs.control; Homo_723,male,AD.vs.control; Homo_723,female,AD.vs.MCI; Homo_723,male,AD.vs.MCI; Homo_714,female,AD.vs.control; Homo_714,male,AD.vs.control; Homo_714,female,AD.vs.MCI; Homo_714,male,AD.vs.MCI; Homo_633,female,AD.vs.control; Homo_633,female,AD.vs.MCI; MCSA,totalSample,age; MCSA,control,age; Emory,totalSample,immune-Macrophage; Emory,totalSample,immune-NK; Emory,totalSample,immune-Neutrophil; Emory,totalSample,immune-CD4_T; Emory,totalSample,immune-CD8_T; Emory,totalSample,immune-Tr1; Emory,totalSample,immune-iTreg; Emory,totalSample,immune-Exhausted; Emory,totalSample,immune-Effector_memory; Emory,totalSample,immune-InfiltrationScore; Homo_723,totalSample,immune-InfiltrationScore; Homo_723,totalSample,immune-Tfh; Homo_723,totalSample,immune-Effector_memory; Homo_723,totalSample,immune-iTreg; Homo_723,totalSample,immune-CD4_naive; Homo_723,totalSample,immune-nTreg; Homo_723,totalSample,immune-CD4_T; Homo_723,totalSample,immune-CD8_T; Homo_723,totalSample,immune-Th2; Homo_723,totalSample,immune-Tr1; Homo_723,totalSample,immune-Central_memory; Homo_723,totalSample,immune-Macrophage; Homo_723,totalSample,immune-MAIT; Homo_723,totalSample,immune-Bcell; Homo_723,totalSample,immune-Cytotoxic; Homo_714,totalSample,immune-Effector_memory; Homo_714,totalSample,immune-InfiltrationScore; Homo_714,totalSample,immune-Tfh; Homo_714,totalSample,immune-nTreg; Homo_714,totalSample,immune-CD4_T; Homo_714,totalSample,immune-CD4_naive; Homo_714,totalSample,immune-Th2; Homo_714,totalSample,immune-Tr1; Homo_714,totalSample,immune-iTreg; Homo_714,totalSample,immune-Central_memory; Homo_714,totalSample,immune-Macrophage; Homo_714,totalSample,immune-CD8_T; Homo_714,totalSample,immune-Monocyte; Homo_714,totalSample,immune-Neutrophil; Homo_714,totalSample,immune-MAIT; Homo_633,totalSample,immune-InfiltrationScore; Homo_633,totalSample,immune-Effector_memory; Homo_633,totalSample,immune-nTreg; Homo_633,totalSample,immune-Tfh; Homo_633,totalSample,immune-CD4_naive; Homo_633,totalSample,immune-CD4_T; Homo_633,totalSample,immune-Central_memory; Homo_633,totalSample,immune-Tr1; Homo_633,totalSample,immune-Neutrophil; Homo_633,totalSample,immune-iTreg; Homo_633,totalSample,immune-Monocyte; Homo_633,totalSample,immune-Macrophage; MCSA,totalSample,immune-DC; MCSA,totalSample,immune-Monocyte; MCSA,totalSample,immune-Neutrophil; MCSA,totalSample,immune-CD4_T; MCSA,totalSample,immune-CD8_T; MCSA,totalSample,immune-NKT; MCSA,totalSample,immune-CD4_naive; MCSA,totalSample,immune-Tr1; MCSA,totalSample,immune-nTreg; MCSA,totalSample,immune-iTreg; MCSA,totalSample,immune-Th2; MCSA,totalSample,immune-Th17; MCSA,totalSample,immune-Tfh; MCSA,totalSample,immune-Cytotoxic; MCSA,totalSample,immune-MAIT; MCSA,totalSample,immune-Central_memory; MCSA,totalSample,immune-Effector_memory; MCSA,totalSample,immune-InfiltrationScore; ROSMAP,totalSample,immune-Th17; ROSMAP,totalSample,immune-Cytotoxic; ROSMAP,totalSample,immune-Effector_memory; SRP223445,totalSample,immune-NK; SRP223445,totalSample,immune-Neutrophil; SRP223445,totalSample,immune-CD4_naive; SRP223445,totalSample,immune-nTreg; SRP223445,totalSample,immune-InfiltrationScore; ROSMAP,female-excludeAge90+,survival
WNT10A	Homo_723,female,AD.vs.control; Homo_723,male,AD.vs.control; Homo_723,female,AD.vs.MCI; Homo_723,male,AD.vs.MCI; Homo_723,male,MCI.vs.control; Homo_714,female,AD.vs.control; Homo_714,male,AD.vs.control; Homo_714,female,AD.vs.MCI; Homo_714,male,AD.vs.MCI; Homo_714,female,MCI.vs.control; Homo_714,male,MCI.vs.control; Homo_633,female,AD.vs.control; Homo_633,female,AD.vs.MCI; Homo_633,male,MCI.vs.control; ROSMAP,female,AD.vs.control; ROSMAP,female,MCI.vs.control; MCSA,totalSample,age; MCSA,control,age; Emory,totalSample,immune-B_cell; Emory,totalSample,immune-Macrophage; Emory,totalSample,immune-Neutrophil; Emory,totalSample,immune-CD4_T; Emory,totalSample,immune-Tr1; Emory,totalSample,immune-nTreg; Emory,totalSample,immune-iTreg; Emory,totalSample,immune-Tfh; Emory,totalSample,immune-CD8_naive; Homo_723,totalSample,immune-CD8_T; Homo_723,totalSample,immune-Exhausted; Homo_723,totalSample,immune-iTreg; Homo_723,totalSample,immune-Monocyte; Homo_723,totalSample,immune-nTreg; Homo_723,totalSample,immune-Central_memory; Homo_723,totalSample,immune-Tfh; Homo_723,totalSample,immune-Macrophage; Homo_723,totalSample,immune-Tr1; Homo_723,totalSample,immune-CD4_T; Homo_723,totalSample,immune-CD4_naive; Homo_723,totalSample,immune-InfiltrationScore; Homo_723,totalSample,immune-Cytotoxic; Homo_723,totalSample,immune-CD8_naive; Homo_714,totalSample,immune-nTreg; Homo_714,totalSample,immune-Monocyte; Homo_714,totalSample,immune-iTreg; Homo_714,totalSample,immune-Tfh; Homo_714,totalSample,immune-Tr1; Homo_714,totalSample,immune-Macrophage; Homo_714,totalSample,immune-Central_memory; Homo_714,totalSample,immune-CD4_T; Homo_633,totalSample,immune-nTreg; Homo_633,totalSample,immune-Monocyte; Homo_633,totalSample,immune-iTreg; Homo_633,totalSample,immune-Tr1; Homo_633,totalSample,immune-Central_memory; Homo_633,totalSample,immune-DC; Homo_633,totalSample,immune-CD4_T; Homo_633,totalSample,immune-Th1; Homo_633,totalSample,immune-Tfh; Homo_633,totalSample,immune-CD4_naive; Homo_633,totalSample,immune-Macrophage; MCSA,totalSample,immune-DC; MCSA,totalSample,immune-Monocyte; MCSA,totalSample,immune-Neutrophil; MCSA,totalSample,immune-CD4_T; MCSA,totalSample,immune-CD8_T; MCSA,totalSample,immune-NKT; MCSA,totalSample,immune-Gamma_delta; MCSA,totalSample,immune-CD4_naive; MCSA,totalSample,immune-Tr1; MCSA,totalSample,immune-nTreg; MCSA,totalSample,immune-Th2; MCSA,totalSample,immune-Tfh; MCSA,totalSample,immune-CD8_naive; MCSA,totalSample,immune-Cytotoxic; MCSA,totalSample,immune-[illegible]
SLC25A19	Homo_723,female,AD.vs.control; Homo_723,male,AD.vs.control; Homo_723,female,AD.vs.MCI; Homo_723,male,AD.vs.MCI; Homo_714,female,AD.vs.control; Homo_714,male,AD.vs.control; Homo_714,female,AD.vs.MCI; Homo_714,male,AD.vs.MCI; Homo_633,female,AD.vs.control; Homo_633,female,AD.vs.MCI; MCSA,totalSample,age; MCSA,control,age; MCSA,MCI,age; ROSMAP,MCI,last.visit.age; ROSMAP,MCI,sample.age; Emory,totalSample,immune-B_cell; Emory,totalSample,immune-Macrophage; Emory,totalSample,immune-Neutrophil; Emory,totalSample,immune-CD4_T; Emory,totalSample,immune-Tr1; Emory,totalSample,immune-iTreg; Emory,totalSample,immune-Exhausted; Emory,totalSample,immune-InfiltrationScore; Homo_723,totalSample,immune-CD8_T; Homo_723,totalSample,immune-InfiltrationScore; Homo_723,totalSample,immune-Exhausted; Homo_723,totalSample,immune-Monocyte; Homo_723,totalSample,immune-Cytotoxic; Homo_723,totalSample,immune-iTreg; Homo_723,totalSample,immune-nTreg; Homo_723,totalSample,immune-Tfh; Homo_723,totalSample,immune-Effector_memory; Homo_723,totalSample,immune-Central_memory; Homo_723,totalSample,immune-Macrophage; Homo_723,totalSample,immune-DC; Homo_723,totalSample,immune-CD4_naive; Homo_714,totalSample,immune-InfiltrationScore; Homo_714,totalSample,immune-Monocyte; Homo_714,totalSample,immune-nTreg; Homo_714,totalSample,immune-Effector_memory; Homo_714,totalSample,immune-Tfh; Homo_714,totalSample,immune-CD8_T; Homo_714,totalSample,immune-DC; Homo_714,totalSample,immune-Neutrophil; Homo_714,totalSample,immune-Macrophage; Homo_633,totalSample,immune-InfiltrationScore; Homo_633,totalSample,immune-nTreg; Homo_633,totalSample,immune-DC; Homo_633,totalSample,immune-Monocyte; Homo_633,totalSample,immune-Neutrophil; Homo_633,totalSample,immune-Effector_memory; Homo_633,totalSample,immune-CD4_naive; Homo_633,totalSample,immune-Central_memory; Homo_633,totalSample,immune-Tfh; MCSA,totalSample,immune-DC; MCSA,totalSample,immune-Monocyte; MCSA,totalSample,immune-NK; MCSA,totalSample,immune-Neutrophil; MCSA,totalSample,immune-CD4_T; MCSA,totalSample,immune-CD8_T; MCSA,totalSample,immune-NKT; MCSA,totalSample,immune-CD4_naive; MCSA,totalSample,immune-nTreg; MCSA,totalSample,immune-Th2; MCSA,totalSample,immune-Tfh; MCSA,totalSample,immune-Cytotoxic; MCSA,totalSample,immune-Central_memory; MCSA,totalSample,immune-Effector_memory; MCSA,totalSample,immune-InfiltrationScore; ROSMAP,totalSample,immune-Effector_memory; SRP223445,totalSample,immune-NK; SRP223445,totalSample,immune-Neutrophil; SRP223445,totalSample,immune-InfiltrationScore; ROSMAP,male-[illegible]
SCG2	Homo_723,female,AD.vs.control; Homo_723,female,AD.vs.MCI; Homo_714,female,AD.vs.control; Homo_714,female,AD.vs.MCI; ROSMAP,AD,sample.age; Homo_633,totalSample,immune-Th2
KIFC1	Homo_723,female,AD.vs.control; Homo_723,male,AD.vs.control; Homo_723,female,AD.vs.MCI; Homo_723,male,AD.vs.MCI; Homo_714,female,AD.vs.control; Homo_714,male,AD.vs.control; Homo_714,female,AD.vs.MCI; Homo_714,male,AD.vs.MCI; Homo_633,female,AD.vs.control; Homo_633,female,AD.vs.MCI; ROSMAP,female,AD.vs.control; ROSMAP,male,AD.vs.control; ROSMAP,male,MCI.vs.control; ROSMAP,AD,sample.age; Emory,totalSample,immune-DC; Emory,totalSample,immune-InfiltrationScore; Homo_723,totalSample,immune-DC; Homo_723,totalSample,immune-InfiltrationScore; Homo_723,totalSample,immune-Neutrophil; Homo_723,totalSample,immune-nTreg; Homo_723,totalSample,immune-Monocyte; Homo_714,totalSample,immune-DC; Homo_714,totalSample,immune-InfiltrationScore; Homo_633,totalSample,immune-InfiltrationScore; Homo_633,totalSample,immune-DC; MCSA,totalSample,immune-DC; MCSA,totalSample,immune-Monocyte; MCSA,totalSample,immune-Macrophage; MCSA,totalSample,immune-Neutrophil; MCSA,totalSample,immune-NKT; MCSA,totalSample,immune-Th2; MCSA,totalSample,immune-Effector_memory; MCSA,totalSample,immune-InfiltrationScore
ENKUR	Homo_723,female,AD.vs.control; Homo_723,male,AD.vs.control; Homo_723,female,AD.vs.MCI; Homo_723,male,AD.vs.MCI; Homo_723,female,MCI.vs.control; Homo_723,male,MCI.vs.control; Homo_714,female,AD.vs.control; Homo_714,male,AD.vs.control; Homo_714,female,AD.vs.MCI; Homo_714,male,AD.vs.MCI; Homo_714,female,MCI.vs.control; Homo_714,male,MCI.vs.control; Homo_633,female,AD.vs.control; Homo_633,female,AD.vs.MCI; ROSMAP,male,AD.vs.control; SRP223445,female,AD.vs.control; MCSA,MCI,age; ROSMAP,AD,MMSE; Emory,totalSample,immune-Th17; Emory,totalSample,immune-Effector_memory; Homo_723,totalSample,immune-Exhausted; Homo_723,totalSample,immune-Th17; Homo_723,totalSample,immune-CD8_T; Homo_723,totalSample,immune-Cytotoxic; Homo_723,totalSample,immune-Monocyte; Homo_714,totalSample,immune-Monocyte; Homo_714,totalSample,immune-Th17; Homo_714,totalSample,immune-Exhausted; Homo_633,totalSample,immune-Th2; Homo_633,totalSample,immune-Monocyte; Homo_633,totalSample,immune-Tfh; Homo_633,totalSample,immune-Gamma_delta; Homo_633,totalSample,immune-Exhausted; Homo_633,totalSample,immune-Th17; MCSA,totalSample,immune-DC; MCSA,totalSample,immune-Monocyte; MCSA,totalSample,immune-CD8_T; MCSA,totalSample,immune-iTreg; MCSA,totalSample,immune-Th2; MCSA,totalSample,immune-Cytotoxic; MCSA,totalSample,immune-Exhausted; ROSMAP,totalSample,immune-InfiltrationScore; SRP223445,totalSample,immune-CD4_naive; SRP223445,totalSample,immune-Exhausted
ATP5PB	Homo_723,female,AD.vs.control; Homo_723,male,AD.vs.control; Homo_723,female,AD.vs.MCI; Homo_723,male,AD.vs.MCI; Homo_714,female,AD.vs.control; Homo_714,male,AD.vs.control; Homo_714,female,AD.vs.MCI; Homo_714,male,AD.vs.MCI; Homo_633,female,AD.vs.control; Homo_633,female,AD.vs.MCI; ROSMAP,female,AD.vs.control; ROSMAP,female,MCI.vs.control; ACOM,AD,age; MCSA,totalSample,age; MCSA,control,age; MCSA,MCI,age; ROSMAP,AD,last.visit.age; ROSMAP,MCI,last.visit.age; ROSMAP,MCI,sample.age; Emory,totalSample,immune-Monocyte; Emory,totalSample,immune-Macrophage; Emory,totalSample,immune-NK; Emory,totalSample,immune-Neutrophil; Emory,totalSample,immune-CD4_T; Emory,totalSample,immune-Tr1; Emory,totalSample,immune-iTreg; Emory,totalSample,immune-Tfh; Emory,totalSample,immune-Effector_memory; Emory,totalSample,immune-InfiltrationScore; Homo_723,totalSample,immune-Monocyte; Homo_723,totalSample,immune-DC; Homo_723,totalSample,immune-Th2; Homo_723,totalSample,immune-Tfh; Homo_723,totalSample,immune-iTreg; Homo_723,totalSample,immune-InfiltrationScore; Homo_723,totalSample,immune-MAIT; Homo_714,totalSample,immune-Monocyte; Homo_714,totalSample,immune-Th2; Homo_714,totalSample,immune-Tfh; Homo_633,totalSample,immune-Monocyte; Homo_633,totalSample,immune-Th2; Homo_633,totalSample,immune-Bcell; Homo_633,totalSample,immune-Tfh; MCSA,totalSample,immune-DC; MCSA,totalSample,immune-Monocyte; MCSA,totalSample,immune-Macrophage; MCSA,totalSample,immune-Gamma_delta; MCSA,totalSample,immune-Th2; MCSA,totalSample,immune-Th17; MCSA,totalSample,immune-MAIT; ROSMAP,totalSample,immune-[illegible]
RARB	Homo_723,female,AD.vs.control; Homo_723,male,AD.vs.control; Homo_723,female,AD.vs.MCI; Homo_723,male,AD.vs.MCI; Homo_714,female,AD.vs.control; Homo_714,male,AD.vs.control; Homo_714,female,AD.vs.MCI; Homo_714,male,AD.vs.MCI; Homo_633,female,AD.vs.control; Homo_633,female,AD.vs.MCI; ROSMAP,AD,last.visit.age; Emory,totalSample,immune-Tr1; Homo_723,totalSample,immune-Th1; Homo_723,totalSample,immune-CD4_naive; Homo_723,totalSample,immune-CD4_T; Homo_723,totalSample,immune-Cytotoxic; Homo_723,totalSample,immune-Tr1; Homo_723,totalSample,immune-Th17; Homo_723,totalSample,immune-Tfh; Homo_723,totalSample,immune-InfiltrationScore; Homo_723,totalSample,immune-nTreg; Homo_723,totalSample,immune-Central_memory; Homo_723,totalSample,immune-Neutrophil; Homo_723,totalSample,immune-Exhausted; Homo_714,totalSample,immune-Th1; Homo_714,totalSample,immune-NKT; Homo_714,totalSample,immune-InfiltrationScore; Homo_714,totalSample,immune-CD4_naive; Homo_714,totalSample,immune-CD4_T; Homo_714,totalSample,immune-Tfh; Homo_714,totalSample,immune-Tr1; Homo_633,totalSample,immune-Tfh; Homo_633,totalSample,immune-CD4_T; Homo_633,totalSample,immune-Tr1; Homo_633,totalSample,immune-CD4_naive; Homo_633,totalSample,immune-NKT; Homo_633,totalSample,immune-Central_memory; Homo_633,totalSample,immune-iTreg; Homo_633,totalSample,immune-InfiltrationScore; Homo_633,totalSample,immune-Th2; Homo_633,totalSample,immune-Effector_memory; Homo_633,totalSample,immune-Th1; Homo_633,totalSample,immune-nTreg; MCSA,totalSample,immune-DC; MCSA,totalSample,immune-Macrophage; MCSA,totalSample,immune-CD4_T; MCSA,totalSample,immune-CD4_naive; MCSA,totalSample,immune-Tr1; MCSA,totalSample,immune-nTreg; MCSA,totalSample,immune-iTreg; MCSA,totalSample,immune-Th17; MCSA,totalSample,immune-Tfh; MCSA,totalSample,immune-CD8_naive; MCSA,totalSample,immune-Cytotoxic; MCSA,totalSample,immune-Exhausted; MCSA,totalSample,immune-MAIT;
PSMD9	Homo_723,female,AD.vs.control; Homo_723,male,AD.vs.control; Homo_723,female,AD.vs.MCI; Homo_723,male,AD.vs.MCI; Homo_714,female,AD.vs.control; Homo_714,male,AD.vs.control; Homo_714,female,AD.vs.MCI; Homo_714,male,AD.vs.MCI; Homo_633,female,AD.vs.control; Homo_633,female,AD.vs.MCI; ROSMAP,female,AD.vs.control; Emory,totalSample,immune-DC; Emory,totalSample,immune-Monocyte; Emory,totalSample,immune-NK; Emory,totalSample,immune-CD4_naive; Emory,totalSample,immune-Tr1; Emory,totalSample,immune-iTreg; Emory,totalSample,immune-Effector_memory; Homo_723,totalSample,immune-InfiltrationScore; Homo_723,totalSample,immune-Cytotoxic; Homo_723,totalSample,immune-Effector_memory; Homo_723,totalSample,immune-Monocyte; Homo_723,totalSample,immune-CD8_T; Homo_723,totalSample,immune-Th1; Homo_723,totalSample,immune-MAIT; Homo_714,totalSample,immune-InfiltrationScore; Homo_714,totalSample,immune-Th1; Homo_714,totalSample,immune-Effector_memory; Homo_714,totalSample,immune-Cytotoxic; Homo_714,totalSample,immune-CD8_T; Homo_633,totalSample,immune-InfiltrationScore; Homo_633,totalSample,immune-Monocyte; Homo_633,totalSample,immune-DC; MCSA,totalSample,immune-DC; MCSA,totalSample,immune-Neutrophil; MCSA,totalSample,immune-CD8_T; MCSA,totalSample,immune-NKT; MCSA,totalSample,immune-iTreg; MCSA,totalSample,immune-Th2; MCSA,totalSample,immune-Th17; MCSA,totalSample,immune-Cytotoxic; MCSA,totalSample,immune-Effector_memory; ROSMAP,totalSample,immune-Th17; ROSMAP,totalSample,immune-Effector_memory; SRP223445,totalSample,immune-NK; SRP223445,totalSample,immune-CD4_naive;

NUBP1	Homo_723,female,AD.vs.control; Homo_723,male,AD.vs.control; Homo_723,female,AD.vs.MCI; Homo_723,male,AD.vs.MCI; Homo_714,female,AD.vs.control; Homo_714,male,AD.vs.control; Homo_714,female,AD.vs.MCI; Homo_714,male,AD.vs.MCI; Homo_633,female,AD.vs.control; Homo_633,female,AD.vs.MCI; ROSMAP,female,AD.vs.control; ROSMAP,AD,sample.age; ROSMAP,MCI,last.visit.age; Emory,totalSample,immune-Macrophage; Emory,totalSample,immune-NK; Emory,totalSample,immune-Neutrophil; Emory,totalSample,immune-CD4_T; Emory,totalSample,immune-Tr1; Emory,totalSample,immune-iTreg; Emory,totalSample,immune-Exhausted; Emory,totalSample,immune-Effector_memory; Emory,totalSample,immune-InfiltrationScore; Homo_723,totalSample,immune-Effector_memory; Homo_723,totalSample,immune-InfiltrationScore; Homo_723,totalSample,immune-Tfh; Homo_723,totalSample,immune-Th2; Homo_723,totalSample,immune-Monocyte; Homo_723,totalSample,immune-Bcell; Homo_723,totalSample,immune-iTreg; Homo_723,totalSample,immune-Cytotoxic; Homo_714,totalSample,immune-Effector_memory; Homo_714,totalSample,immune-Tfh; Homo_714,totalSample,immune-InfiltrationScore; Homo_714,totalSample,immune-Th2; Homo_633,totalSample,immune-InfiltrationScore; Homo_633,totalSample,immune-Effector_memory; Homo_633,totalSample,immune-Monocyte; Homo_633,totalSample,immune-Tfh; Homo_633,totalSample,immune-Th2; Homo_633,totalSample,immune-DC; MCSA,totalSample,immune-DC; MCSA,totalSample,immune-Monocyte; MCSA,totalSample,immune-NK; MCSA,totalSample,immune-Neutrophil; MCSA,totalSample,immune-CD8_T; MCSA,totalSample,immune-NKT; MCSA,totalSample,immune-CD4_naive; MCSA,totalSample,immune-nTreg; MCSA,totalSample,immune-Th2; MCSA,totalSample,immune-Tfh; MCSA,totalSample,immune-Cytotoxic; MCSA,totalSample,immune-Central_memory; MCSA,totalSample,immune-Effector_memory; MCSA,totalSample,immune-InfiltrationScore; SRP223445,totalSample,immune-NK; SRP223445,totalSample,immune-Exhausted; [illegible]
SLC12A2	Homo_723,female,AD.vs.control; Homo_723,male,AD.vs.control; Homo_723,female,AD.vs.MCI; Homo_723,male,AD.vs.MCI; Homo_723,female,MCI.vs.control; Homo_723,male,MCI.vs.control; Homo_714,female,AD.vs.control; Homo_714,male,AD.vs.control; Homo_714,female,AD.vs.MCI; Homo_714,male,AD.vs.MCI; Homo_714,female,MCI.vs.control; Homo_714,male,MCI.vs.control; Homo_633,female,AD.vs.control; Homo_633,female,AD.vs.MCI; MCSA,MCI,age; ROSMAP,AD,sample.age; Emory,totalSample,immune-Macrophage; Emory,totalSample,immune-Neutrophil; Emory,totalSample,immune-CD4_T; Emory,totalSample,immune-Tr1; Emory,totalSample,immune-iTreg; Emory,totalSample,immune-Tfh; Emory,totalSample,immune-InfiltrationScore; Homo_723,totalSample,immune-CD4_naive; Homo_723,totalSample,immune-CD4_T; Homo_723,totalSample,immune-Th17; Homo_723,totalSample,immune-Cytotoxic; Homo_723,totalSample,immune-Tr1; Homo_723,totalSample,immune-Tfh; Homo_723,totalSample,immune-Central_memory; Homo_723,totalSample,immune-Th1; Homo_723,totalSample,immune-Exhausted; Homo_723,totalSample,immune-InfiltrationScore; Homo_723,totalSample,immune-nTreg; Homo_723,totalSample,immune-iTreg; Homo_723,totalSample,immune-Th2; Homo_723,totalSample,immune-NKT; Homo_723,totalSample,immune-Neutrophil; Homo_723,totalSample,immune-CD8_T; Homo_714,totalSample,immune-CD4_naive; Homo_714,totalSample,immune-CD4_T; Homo_714,totalSample,immune-NKT; Homo_714,totalSample,immune-Tfh; Homo_714,totalSample,immune-Central_memory; Homo_714,totalSample,immune-Th1; Homo_714,totalSample,immune-Th17; Homo_714,totalSample,immune-InfiltrationScore; Homo_714,totalSample,immune-Tr1; Homo_714,totalSample,immune-iTreg; Homo_714,totalSample,immune-Exhausted; Homo_633,totalSample,immune-Tfh; Homo_633,totalSample,immune-CD4_T; Homo_633,totalSample,immune-CD4_naive; Homo_633,totalSample,immune-Tr1; Homo_633,totalSample,immune-Central_memory; Homo_633,totalSample,immune-NKT; Homo_633,totalSample,immune-Effector_memory; Homo_633,totalSample,immune-iTreg; Homo_633,totalSample,immune-InfiltrationScore; Homo_633,totalSample,immune-Th2; Homo_633,totalSample,immune-nTreg; Homo_633,totalSample,immune-Gamma_delta; Homo_633,totalSample,immune-Th1; Homo_633,totalSample,immune-DC; Homo_633,totalSample,immune-Th17; MCSA,totalSample,immune-DC; MCSA,totalSample,immune-Bcell; MCSA,totalSample,immune-Monocyte; MCSA,totalSample,immune-Macrophage; MCSA,totalSample,immune-CD4_T; MCSA,totalSample,immune-CD8_T; MCSA,totalSample,immune-CD4_naive; MCSA,totalSample,immune-Tr1; MCSA,totalSample,immune-nTreg; MCSA,totalSample,immune-iTreg; MCSA,totalSample,immune-Th2; MCSA,totalSample,immune-Th17; MCSA,totalSample,immune-Tfh; MCSA,totalSample,immune-CD8_naive; MCSA,totalSample,immune-Cytotoxic; MCSA,totalSample,immune-Exhausted; MCSA,totalSample,immune-MAIT; MCSA,totalSample,immune-Central_memory; ROSMAP,totalSample,immune-InfiltrationScore; [illegible]
CPLANE1	Homo_723,female,AD.vs.control; Homo_723,male,AD.vs.control; Homo_723,female,AD.vs.MCI; Homo_723,male,AD.vs.MCI; Homo_723,female,MCI.vs.control; Homo_714,female,AD.vs.control; Homo_714,male,AD.vs.control; Homo_714,female,AD.vs.MCI; Homo_714,male,AD.vs.MCI; Homo_633,female,AD.vs.control; Homo_633,female,AD.vs.MCI; MCSA,MCI,age; ROSMAP,MCI,last.visit.age; ROSMAP,MCI,sample.age; ROSMAP,MCI,MMSE; Emory,totalSample,immune-Monocyte; Emory,totalSample,immune-Macrophage; Emory,totalSample,immune-Neutrophil; Emory,totalSample,immune-CD4_T; Emory,totalSample,immune-CD8_T; Emory,totalSample,immune-Tr1; Emory,totalSample,immune-iTreg; Emory,totalSample,immune-Tfh; Emory,totalSample,immune-Exhausted; Emory,totalSample,immune-Central_memory; Emory,totalSample,immune-Effector_memory; Emory,totalSample,immune-InfiltrationScore; Homo_723,totalSample,immune-CD4_naive; Homo_723,totalSample,immune-CD4_T; Homo_723,totalSample,immune-Tfh; Homo_723,totalSample,immune-Central_memory; Homo_723,totalSample,immune-Th17; Homo_723,totalSample,immune-nTreg; Homo_723,totalSample,immune-Tr1; Homo_723,totalSample,immune-InfiltrationScore; Homo_723,totalSample,immune-Th1; Homo_723,totalSample,immune-Exhausted; Homo_723,totalSample,immune-Th2; Homo_723,totalSample,immune-Neutrophil; Homo_723,totalSample,immune-iTreg; Homo_723,totalSample,immune-Cytotoxic; Homo_723,totalSample,immune-Effector_memory; Homo_723,totalSample,immune-NKT; Homo_714,totalSample,immune-CD4_naive; Homo_714,totalSample,immune-CD4_T; Homo_714,totalSample,immune-Tfh; Homo_714,totalSample,immune-Central_memory; Homo_714,totalSample,immune-InfiltrationScore; Homo_714,totalSample,immune-Effector_memory; Homo_714,totalSample,immune-Th17; Homo_714,totalSample,immune-Th1; Homo_714,totalSample,immune-NKT; Homo_714,totalSample,immune-nTreg; Homo_714,totalSample,immune-iTreg; Homo_714,totalSample,immune-Tr1; Homo_714,totalSample,immune-Neutrophil; Homo_633,totalSample,immune-Tfh; Homo_633,totalSample,immune-CD4_naive; Homo_633,totalSample,immune-CD4_T; Homo_633,totalSample,immune-Effector_memory; Homo_633,totalSample,immune-Central_memory; Homo_633,totalSample,immune-InfiltrationScore; Homo_633,totalSample,immune-Tr1; Homo_633,totalSample,immune-Th2; Homo_633,totalSample,immune-iTreg; Homo_633,totalSample,immune-nTreg; Homo_633,totalSample,immune-NKT; Homo_633,totalSample,immune-Bcell; Homo_633,totalSample,immune-Neutrophil; Homo_633,totalSample,immune-MAIT; Homo_633,totalSample,immune-Th17; MCSA,totalSample,immune-DC; MCSA,totalSample,immune-Macrophage; MCSA,totalSample,immune-CD4_T; MCSA,totalSample,immune-CD4_naive; MCSA,totalSample,immune-Tr1; MCSA,totalSample,immune-nTreg; MCSA,totalSample,immune-iTreg; MCSA,totalSample,immune-Th17; MCSA,totalSample,immune-Tfh; MCSA,totalSample,immune-CD8_naive; MCSA,totalSample,immune-Cytotoxic; MCSA,totalSample,immune-Exhausted; MCSA,totalSample,immune-MAIT; [illegible]
ALKBH1	Homo_723,female,AD.vs.control; Homo_723,male,AD.vs.control; Homo_723,female,AD.vs.MCI; Homo_723,male,AD.vs.MCI; Homo_714,female,AD.vs.control; Homo_714,male,AD.vs.control; Homo_714,female,AD.vs.MCI; Homo_714,male,AD.vs.MCI; Homo_633,female,AD.vs.control; Homo_633,female,AD.vs.MCI; MCSA,totalSample,age; MCSA,control,age; MCSA,MCI,age; ROSMAP,MCI,last.visit.age; Emory,totalSample,immune-Macrophage; Emory,totalSample,immune-Neutrophil; Emory,totalSample,immune-CD4_T; Emory,totalSample,immune-Tr1; Emory,totalSample,immune-iTreg; Emory,totalSample,immune-Central_memory; Emory,totalSample,immune-Effector_memory; Emory,totalSample,immune-InfiltrationScore; Homo_723,totalSample,immune-Th2; Homo_723,totalSample,immune-Tfh; Homo_723,totalSample,immune-iTreg; Homo_723,totalSample,immune-Effector_memory; Homo_723,totalSample,immune-CD4_T; Homo_723,totalSample,immune-InfiltrationScore; Homo_723,totalSample,immune-Tr1; Homo_723,totalSample,immune-CD4_naive; Homo_723,totalSample,immune-nTreg; Homo_723,totalSample,immune-Central_memory; Homo_723,totalSample,immune-DC; Homo_723,totalSample,immune-Bcell; Homo_723,totalSample,immune-Macrophage; Homo_723,totalSample,immune-Monocyte; Homo_723,totalSample,immune-Gamma_delta; Homo_714,totalSample,immune-Th2; Homo_714,totalSample,immune-Tfh; Homo_714,totalSample,immune-Effector_memory; Homo_714,totalSample,immune-CD4_T; Homo_714,totalSample,immune-Tr1; Homo_714,totalSample,immune-Central_memory; Homo_714,totalSample,immune-CD4_naive; Homo_714,totalSample,immune-InfiltrationScore; Homo_714,totalSample,immune-iTreg; Homo_714,totalSample,immune-nTreg; Homo_633,totalSample,immune-Tfh; Homo_633,totalSample,immune-Th2; Homo_633,totalSample,immune-Effector_memory; Homo_633,totalSample,immune-CD4_T; Homo_633,totalSample,immune-CD4_naive; Homo_633,totalSample,immune-InfiltrationScore; Homo_633,totalSample,immune-Central_memory; Homo_633,totalSample,immune-Tr1; Homo_633,totalSample,immune-nTreg; Homo_633,totalSample,immune-Exhausted; Homo_633,totalSample,immune-Th17; Homo_633,totalSample,immune-Bcell; Homo_633,totalSample,immune-iTreg; Homo_633,totalSample,immune-Monocyte; Homo_633,totalSample,immune-Gamma_delta; MCSA,totalSample,immune-Monocyte; MCSA,totalSample,immune-Macrophage; MCSA,totalSample,immune-Neutrophil; MCSA,totalSample,immune-CD4_T; MCSA,totalSample,immune-CD4_naive; MCSA,totalSample,immune-Tr1; MCSA,totalSample,immune-nTreg; MCSA,totalSample,immune-iTreg; MCSA,totalSample,immune-Th17; MCSA,totalSample,immune-Tfh; MCSA,totalSample,immune-CD8_naive; MCSA,totalSample,immune-Exhausted; MCSA,totalSample,immune-MAIT; MCSA,totalSample,immune-Central_memory; MCSA,totalSample,immune-Effector_memory; MCSA,totalSample,immune-InfiltrationScore; SRP223445,totalSample,immune-NK; SRP223445,totalSample,immune-CD4_naive; SRP223445,totalSample,immune-Exhausted; SRP223445,totalSample,immune-InfiltrationScore
ZNRF3	Homo_723,female,AD.vs.control; Homo_723,male,AD.vs.control; Homo_723,female,AD.vs.MCI; Homo_723,male,AD.vs.MCI; Homo_714,female,AD.vs.control; Homo_714,male,AD.vs.control; Homo_714,female,AD.vs.MCI; Homo_714,male,AD.vs.MCI; Homo_633,female,AD.vs.control; Homo_633,female,AD.vs.MCI; Emory,totalSample,immune-Tr1; Emory,totalSample,immune-iTreg; Homo_723,totalSample,immune-CD4_naive; Homo_723,totalSample,immune-Central_memory; Homo_723,totalSample,immune-nTreg; Homo_723,totalSample,immune-CD4_T; Homo_723,totalSample,immune-Tfh; Homo_723,totalSample,immune-Tr1; Homo_723,totalSample,immune-iTreg; Homo_723,totalSample,immune-Neutrophil; Homo_714,totalSample,immune-CD4_naive; Homo_714,totalSample,immune-Tfh; Homo_714,totalSample,immune-InfiltrationScore; Homo_714,totalSample,immune-nTreg; Homo_714,totalSample,immune-Central_memory; Homo_714,totalSample,immune-CD4_T; Homo_633,totalSample,immune-Tfh; Homo_633,totalSample,immune-InfiltrationScore; Homo_633,totalSample,immune-CD4_naive; Homo_633,totalSample,immune-nTreg; Homo_633,totalSample,immune-CD4_T; Homo_633,totalSample,immune-Tr1; Homo_633,totalSample,immune-iTreg; Homo_633,totalSample,immune-Effector_memory; Homo_633,totalSample,immune-Central_memory; Homo_633,totalSample,immune-Neutrophil; MCSA,totalSample,immune-Monocyte; MCSA,totalSample,immune-Macrophage; MCSA,totalSample,immune-Neutrophil; MCSA,totalSample,immune-CD4_T; MCSA,totalSample,immune-CD8_T; MCSA,totalSample,immune-CD4_naive; MCSA,totalSample,immune-Tr1; MCSA,totalSample,immune-nTreg; MCSA,totalSample,immune-iTreg; MCSA,totalSample,immune-Th17; MCSA,totalSample,immune-Tfh; MCSA,totalSample,immune-CD8_naive; MCSA,totalSample,immune-MAIT; MCSA,totalSample,immune-Central_memory; MCSA,totalSample,immune-Effector_memory; MCSA,totalSample,immune-InfiltrationScore; ROSMAP,totalSample,immune-InfiltrationScore; SRP223445,totalSample,immune-CD4_naive
THRB	Homo_723,female,AD.vs.control; Homo_723,male,AD.vs.control; Homo_723,female,AD.vs.MCI; Homo_723,male,AD.vs.MCI; Homo_723,female,MCI.vs.control; Homo_723,male,MCI.vs.control; Homo_723,control,male.vs.female; Homo_723,MCI,male.vs.female; Homo_714,female,AD.vs.control; Homo_714,male,AD.vs.control; Homo_714,female,AD.vs.MCI; Homo_714,male,AD.vs.MCI; Homo_714,female,MCI.vs.control; Homo_714,male,MCI.vs.control; Homo_714,control,male.vs.female; Homo_714,MCI,male.vs.female; Homo_633,female,AD.vs.control; Homo_633,female,AD.vs.MCI; Homo_633,female,MCI.vs.control; Homo_633,MCI,male.vs.female; ROSMAP,control,male.vs.female; SRP223445,female,AD.vs.control; MCSA,MCI,age; Homo_723,totalSample,immune-DC; Homo_714,totalSample,immune-DC; Homo_714,totalSample,immune-Th2; ROSMAP,totalSample,immune-InfiltrationScore; SRP223445,totalSample,immune-CD4_naive; SRP223445,totalSample,immune-Exhausted
FKBP15	Homo_723,female,AD.vs.control; Homo_723,male,AD.vs.control; Homo_723,female,AD.vs.MCI; Homo_723,male,AD.vs.MCI; Homo_714,female,AD.vs.control; Homo_714,male,AD.vs.control; Homo_714,female,AD.vs.MCI; Homo_714,male,AD.vs.MCI; Homo_633,female,AD.vs.control; Homo_633,female,AD.vs.MCI; Emory,MCI,age; ROSMAP,MCI,last.visit.age; Emory,totalSample,immune-Neutrophil; Emory,totalSample,immune-CD8_T; Emory,totalSample,immune-Gamma_delta; Emory,totalSample,immune-Th1; Emory,totalSample,immune-Th17; Emory,totalSample,immune-Tfh; Emory,totalSample,immune-CD8_naive; Emory,totalSample,immune-Cytotoxic; Emory,totalSample,immune-Exhausted; Homo_723,totalSample,immune-Monocyte; Homo_723,totalSample,immune-Th2; Homo_723,totalSample,immune-InfiltrationScore; Homo_723,totalSample,immune-Gamma_delta; Homo_723,totalSample,immune-Effector_memory; Homo_723,totalSample,immune-Bcell; Homo_723,totalSample,immune-Tfh; Homo_723,totalSample,immune-Exhausted; Homo_723,totalSample,immune-Neutrophil; Homo_723,totalSample,immune-DC; Homo_723,totalSample,immune-iTreg; Homo_714,totalSample,immune-Th2; Homo_714,totalSample,immune-Monocyte; Homo_714,totalSample,immune-Tfh; Homo_714,totalSample,immune-Gamma_delta; Homo_714,totalSample,immune-Effector_memory; Homo_714,totalSample,immune-InfiltrationScore; Homo_714,totalSample,immune-Th1; Homo_633,totalSample,immune-Th2; Homo_633,totalSample,immune-Exhausted; Homo_633,totalSample,immune-Monocyte; Homo_633,totalSample,immune-Gamma_delta; Homo_633,totalSample,immune-Th17; Homo_633,totalSample,immune-InfiltrationScore; Homo_633,totalSample,immune-Tfh; Homo_633,totalSample,immune-Effector_memory; Homo_633,totalSample,immune-Bcell; MCSA,totalSample,immune-Neutrophil; MCSA,totalSample,immune-CD8_T; MCSA,totalSample,immune-Gamma_delta; MCSA,totalSample,immune-Tr1; MCSA,totalSample,immune-iTreg; MCSA,totalSample,immune-Th17; MCSA,totalSample,immune-Cytotoxic; MCSA,totalSample,immune-Exhausted; ROSMAP,totalSample,immune-Effector_memory; SRP223445,totalSample,immune-NK; SRP223445,totalSample,immune-CD4_naive; SRP223445,totalSample,immune-Exhausted; SRP223445,totalSample,immune-InfiltrationScore
CRYGS	Homo_723,female,AD.vs.control; Homo_723,male,AD.vs.control; Homo_723,female,AD.vs.MCI; Homo_723,male,AD.vs.MCI; Homo_723,female,MCI.vs.control; Homo_723,male,MCI.vs.control; Homo_714,female,AD.vs.control; Homo_714,male,AD.vs.control; Homo_714,female,AD.vs.MCI; Homo_714,male,AD.vs.MCI; Homo_714,female,MCI.vs.control; Homo_714,male,MCI.vs.control; Homo_633,female,AD.vs.control; Homo_633,female,AD.vs.MCI; Homo_633,female,MCI.vs.control; Homo_633,male,MCI.vs.control; ROSMAP,control,male.vs.female; MCSA,totalSample,age; MCSA,control,age; ROSMAP,MCI,MMSE; Emory,totalSample,immune-Neutrophil; Emory,totalSample,immune-CD4_T; Emory,totalSample,immune-CD8_T; Emory,totalSample,immune-Tr1; Emory,totalSample,immune-iTreg; Emory,totalSample,immune-Tfh; Homo_723,totalSample,immune-Th2; Homo_723,totalSample,immune-Tfh; Homo_723,totalSample,immune-iTreg; Homo_723,totalSample,immune-Effector_memory; Homo_723,totalSample,immune-CD4_T; Homo_723,totalSample,immune-Macrophage; Homo_723,totalSample,immune-DC; Homo_723,totalSample,immune-Tr1; Homo_723,totalSample,immune-CD4_naive; Homo_723,totalSample,immune-Monocyte; Homo_723,totalSample,immune-nTreg; Homo_723,totalSample,immune-Bcell; Homo_714,totalSample,immune-Th2; Homo_714,totalSample,immune-Tfh; Homo_714,totalSample,immune-CD4_T; Homo_714,totalSample,immune-Effector_memory; Homo_714,totalSample,immune-Central_memory; Homo_714,totalSample,immune-Tr1; Homo_714,totalSample,immune-Macrophage; Homo_714,totalSample,immune-nTreg; Homo_714,totalSample,immune-CD4_naive; Homo_714,totalSample,immune-CD8_T; Homo_633,totalSample,immune-Th2; Homo_633,totalSample,immune-Tfh; Homo_633,totalSample,immune-CD4_T; Homo_633,totalSample,immune-Effector_memory; Homo_633,totalSample,immune-Central_memory; Homo_633,totalSample,immune-CD4_naive; Homo_633,totalSample,immune-Th17; Homo_633,totalSample,immune-nTreg; Homo_633,totalSample,immune-InfiltrationScore; Homo_633,totalSample,immune-Tr1; Homo_633,totalSample,immune-Bcell; MCSA,totalSample,immune-Monocyte; MCSA,totalSample,immune-Macrophage; MCSA,totalSample,immune-Neutrophil; MCSA,totalSample,immune-CD4_T; MCSA,totalSample,immune-CD8_T; MCSA,totalSample,immune-CD4_naive; MCSA,totalSample,immune-Tr1; MCSA,totalSample,immune-nTreg; MCSA,totalSample,immune-iTreg; MCSA,totalSample,immune-Th17; MCSA,totalSample,immune-Tfh; MCSA,totalSample,immune-Central_memory; MCSA,totalSample,immune-Effector_memory; MCSA,totalSample,immune-InfiltrationScore; ROSMAP,totalSample,immune-Effector_memory; [illegible]
RGL4	Homo_723,female,AD.vs.control; Homo_723,male,AD.vs.control; Homo_723,female,AD.vs.MCI; Homo_723,male,AD.vs.MCI; Homo_714,female,AD.vs.control; Homo_714,male,AD.vs.control; Homo_714,female,AD.vs.MCI; Homo_714,male,AD.vs.MCI; Homo_633,female,AD.vs.control; Homo_633,female,AD.vs.MCI; ROSMAP,female,AD.vs.MCI; Emory,MCI,age; MCSA,MCI,age; Emory,totalSample,immune-DC; Emory,totalSample,immune-B_cell; Emory,totalSample,immune-Monocyte; Emory,totalSample,immune-Macrophage; Emory,totalSample,immune-NK; Emory,totalSample,immune-Neutrophil; Emory,totalSample,immune-CD8_T; Emory,totalSample,immune-Gamma_delta; Emory,totalSample,immune-Th17; Emory,totalSample,immune-CD8_naive; Emory,totalSample,immune-Exhausted; Homo_723,totalSample,immune-Monocyte; Homo_723,totalSample,immune-Th1; Homo_723,totalSample,immune-Neutrophil; Homo_723,totalSample,immune-Bcell; Homo_723,totalSample,immune-DC; Homo_723,totalSample,immune-Th2; Homo_723,totalSample,immune-Gamma_delta; Homo_714,totalSample,immune-Th1; Homo_714,totalSample,immune-Th2; Homo_714,totalSample,immune-Monocyte; Homo_714,totalSample,immune-NKT; Homo_714,totalSample,immune-Neutrophil; Homo_714,totalSample,immune-Gamma_delta; Homo_633,totalSample,immune-Exhausted; Homo_633,totalSample,immune-Monocyte; Homo_633,totalSample,immune-Th1; Homo_633,totalSample,immune-Th2; Homo_633,totalSample,immune-Gamma_delta; MCSA,totalSample,immune-DC; MCSA,totalSample,immune-Bcell; MCSA,totalSample,immune-Monocyte; MCSA,totalSample,immune-Macrophage; MCSA,totalSample,immune-Neutrophil; MCSA,totalSample,immune-Gamma_delta; MCSA,totalSample,immune-Th1; MCSA,totalSample,immune-Th2; SRP223445,totalSample,immune-Monocyte; SRP223445,totalSample,immune-CD4_T; SRP223445,totalSample,immune-CD4_naive; SRP223445,totalSample,immune-Th1
SEMA6A	Homo_723,female,AD.vs.control; Homo_723,male,AD.vs.control; Homo_723,female,AD.vs.MCI; Homo_723,male,AD.vs.MCI; Homo_723,female,MCI.vs.control; Homo_714,female,AD.vs.control; Homo_714,male,AD.vs.control; Homo_714,female,AD.vs.MCI; Homo_714,male,AD.vs.MCI; Homo_633,female,AD.vs.control; Homo_633,female,AD.vs.MCI; ROSMAP,female,AD.vs.control; MCSA,totalSample,age; MCSA,control,age; Emory,totalSample,immune-CD4_T; Emory,totalSample,immune-Tr1; Emory,totalSample,immune-iTreg; Emory,totalSample,immune-Central_memory; Homo_723,totalSample,immune-DC; Homo_723,totalSample,immune-Central_memory; Homo_723,totalSample,immune-Monocyte; Homo_723,totalSample,immune-nTreg; Homo_723,totalSample,immune-CD4_naive; Homo_723,totalSample,immune-Neutrophil; Homo_723,totalSample,immune-Th1; Homo_723,totalSample,immune-CD4_T; Homo_723,totalSample,immune-InfiltrationScore; Homo_714,totalSample,immune-CD4_naive; Homo_714,totalSample,immune-InfiltrationScore; Homo_714,totalSample,immune-nTreg; Homo_714,totalSample,immune-Monocyte; Homo_714,totalSample,immune-Th1; Homo_714,totalSample,immune-DC; Homo_714,totalSample,immune-Central_memory; Homo_714,totalSample,immune-NKT; Homo_714,totalSample,immune-Neutrophil; Homo_633,totalSample,immune-nTreg; Homo_633,totalSample,immune-Monocyte; Homo_633,totalSample,immune-InfiltrationScore; Homo_633,totalSample,immune-CD4_naive; Homo_633,totalSample,immune-Central_memory; Homo_633,totalSample,immune-Tfh; Homo_633,totalSample,immune-Effector_memory; Homo_633,totalSample,immune-iTreg; Homo_633,totalSample,immune-CD4_T; Homo_633,totalSample,immune-Tr1; MCSA,totalSample,immune-Monocyte; MCSA,totalSample,immune-Neutrophil; MCSA,totalSample,immune-CD4_T; MCSA,totalSample,immune-CD8_T; MCSA,totalSample,immune-NKT; MCSA,totalSample,immune-Gamma_delta; MCSA,totalSample,immune-CD4_naive; MCSA,totalSample,immune-Tr1; MCSA,totalSample,immune-nTreg; MCSA,totalSample,immune-iTreg; MCSA,totalSample,immune-Th2; MCSA,totalSample,immune-Th17; MCSA,totalSample,immune-Tfh; MCSA,totalSample,immune-CD8_naive; MCSA,totalSample,immune-Central_memory; MCSA,totalSample,immune-Effector_memory; MCSA,totalSample,immune-InfiltrationScore; ROSMAP,totalSample,immune-InfiltrationScore; SRP223445,totalSample,immune-[illegible]
DENND2C	Homo_723,female,AD.vs.control; Homo_723,male,AD.vs.control; Homo_723,female,AD.vs.MCI; Homo_723,male,AD.vs.MCI; Homo_723,female,MCI.vs.control; Homo_723,male,MCI.vs.control; Homo_714,female,AD.vs.control; Homo_714,male,AD.vs.control; Homo_714,female,AD.vs.MCI; Homo_714,male,AD.vs.MCI; Homo_714,female,MCI.vs.control; Homo_714,male,MCI.vs.control; Homo_633,female,AD.vs.control; Homo_633,female,AD.vs.MCI; SRP223445,female,AD.vs.control; ROSMAP,MCI,last.visit.age; Homo_723,totalSample,immune-Th17; Homo_723,totalSample,immune-Exhausted; Homo_723,totalSample,immune-CD4_naive; Homo_723,totalSample,immune-CD8_T; Homo_723,totalSample,immune-Cytotoxic; Homo_723,totalSample,immune-CD4_T; Homo_723,totalSample,immune-Tfh; Homo_723,totalSample,immune-Central_memory; Homo_723,totalSample,immune-InfiltrationScore; Homo_714,totalSample,immune-Th17; Homo_714,totalSample,immune-CD4_naive; Homo_714,totalSample,immune-Exhausted; Homo_714,totalSample,immune-CD4_T; Homo_714,totalSample,immune-InfiltrationScore; Homo_714,totalSample,immune-Tfh; Homo_714,totalSample,immune-Central_memory; Homo_633,totalSample,immune-Tfh; Homo_633,totalSample,immune-Th2; Homo_633,totalSample,immune-CD4_T; Homo_633,totalSample,immune-CD4_naive; Homo_633,totalSample,immune-InfiltrationScore; Homo_633,totalSample,immune-Central_memory; Homo_633,totalSample,immune-Th17; Homo_633,totalSample,immune-Effector_memory; Homo_633,totalSample,immune-Gamma_delta; Homo_633,totalSample,immune-Exhausted; Homo_633,totalSample,immune-Tr1; Homo_633,totalSample,immune-iTreg; MCSA,totalSample,immune-DC; MCSA,totalSample,immune-CD4_T; MCSA,totalSample,immune-CD4_naive; MCSA,totalSample,immune-Tr1; MCSA,totalSample,immune-nTreg; MCSA,totalSample,immune-iTreg; MCSA,totalSample,immune-Th17; MCSA,totalSample,immune-Tfh; MCSA,totalSample,immune-CD8_naive; MCSA,totalSample,immune-Cytotoxic; MCSA,totalSample,immune-Exhausted; MCSA,totalSample,immune-[illegible]
TM9SF1	Homo_723,female,AD.vs.control; Homo_723,male,AD.vs.control; Homo_723,female,AD.vs.MCI; Homo_723,male,AD.vs.MCI; Homo_714,female,AD.vs.control; Homo_714,male,AD.vs.control; Homo_714,female,AD.vs.MCI; Homo_714,male,AD.vs.MCI; Homo_633,female,AD.vs.control; Homo_633,female,AD.vs.MCI; Emory,MCI,age; MCSA,totalSample,age; MCSA,control,age; ROSMAP,AD,last.visit.age; Emory,totalSample,immune-NKT; Emory,totalSample,immune-Gamma_delta; Emory,totalSample,immune-Th17; Emory,totalSample,immune-Tfh; Emory,totalSample,immune-CD8_naive; Emory,totalSample,immune-Cytotoxic; Emory,totalSample,immune-InfiltrationScore; Homo_723,totalSample,immune-InfiltrationScore; Homo_723,totalSample,immune-iTreg; Homo_723,totalSample,immune-Th2; Homo_723,totalSample,immune-Tfh; Homo_723,totalSample,immune-Gamma_delta; Homo_723,totalSample,immune-Effector_memory; Homo_723,totalSample,immune-Bcell; Homo_723,totalSample,immune-CD4_T; Homo_723,totalSample,immune-nTreg; Homo_723,totalSample,immune-CD4_naive; Homo_714,totalSample,immune-Th2; Homo_714,totalSample,immune-InfiltrationScore; Homo_714,totalSample,immune-Tfh; Homo_714,totalSample,immune-Effector_memory; Homo_714,totalSample,immune-nTreg; Homo_714,totalSample,immune-Gamma_delta; Homo_714,totalSample,immune-Tr1; Homo_714,totalSample,immune-iTreg; Homo_714,totalSample,immune-CD4_T; Homo_633,totalSample,immune-InfiltrationScore; Homo_633,totalSample,immune-nTreg; Homo_633,totalSample,immune-Exhausted; Homo_633,totalSample,immune-Effector_memory; Homo_633,totalSample,immune-Tfh; Homo_633,totalSample,immune-Gamma_delta; Homo_633,totalSample,immune-CD4_T; Homo_633,totalSample,immune-Th2; Homo_633,totalSample,immune-Tr1; Homo_633,totalSample,immune-CD4_naive; Homo_633,totalSample,immune-Central_memory; MCSA,totalSample,immune-DC; MCSA,totalSample,immune-Monocyte; MCSA,totalSample,immune-CD4_T; MCSA,totalSample,immune-Gamma_delta; MCSA,totalSample,immune-CD4_naive; MCSA,totalSample,immune-Tr1; MCSA,totalSample,immune-nTreg; MCSA,totalSample,immune-iTreg; MCSA,totalSample,immune-Th1; MCSA,totalSample,immune-Th2; MCSA,totalSample,immune-Th17; MCSA,totalSample,immune-Tfh; MCSA,totalSample,immune-Exhausted; MCSA,totalSample,immune-Central_memory; MCSA,totalSample,immune-Effector_memory; [illegible]

ZDHHC6	Homo_723,female,AD.vs.control; Homo_723,male,AD.vs.control; Homo_723,female,AD.vs.MCI; Homo_723,male,AD.vs.MCI; Homo_714,female,AD.vs.control; Homo_714,male,AD.vs.control; Homo_714,female,AD.vs.MCI; Homo_714,male,AD.vs.MCI; Homo_633,female,AD.vs.control; Homo_633,female,AD.vs.MCI; MCSA,MCI,age; ROSMAP,MCI,last.visit.age; ROSMAP,MCI,sample.age; Emory,totalSample,immune-DC; Emory,totalSample,immune-Monocyte; Emory,totalSample,immune-Macrophage; Emory,totalSample,immune-CD4_T; Emory,totalSample,immune-Gamma_delta; Emory,totalSample,immune-Tr1; Emory,totalSample,immune-iTreg; Emory,totalSample,immune-Central_memory; Emory,totalSample,immune-Effector_memory; Emory,totalSample,immune-InfiltrationScore; Homo_723,totalSample,immune-Th2; Homo_723,totalSample,immune-Tfh; Homo_723,totalSample,immune-iTreg; Homo_723,totalSample,immune-CD4_T; Homo_723,totalSample,immune-Tr1; Homo_723,totalSample,immune-DC; Homo_723,totalSample,immune-Effector_memory; Homo_723,totalSample,immune-CD4_naive; Homo_723,totalSample,immune-InfiltrationScore; Homo_723,totalSample,immune-Monocyte; Homo_723,totalSample,immune-Gamma_delta; Homo_723,totalSample,immune-Central_memory; Homo_723,totalSample,immune-Macrophage; Homo_723,totalSample,immune-nTreg; Homo_723,totalSample,immune-Bcell; Homo_723,totalSample,immune-MAIT; Homo_714,totalSample,immune-Th2; Homo_714,totalSample,immune-Tfh; Homo_714,totalSample,immune-CD4_T; Homo_714,totalSample,immune-Tr1; Homo_714,totalSample,immune-Effector_memory; Homo_714,totalSample,immune-Central_memory; Homo_714,totalSample,immune-iTreg; Homo_714,totalSample,immune-CD4_naive; Homo_714,totalSample,immune-InfiltrationScore; Homo_714,totalSample,immune-DC; Homo_714,totalSample,immune-Gamma_delta; Homo_714,totalSample,immune-Monocyte; Homo_714,totalSample,immune-nTreg; Homo_633,totalSample,immune-Tfh; Homo_633,totalSample,immune-Th2; Homo_633,totalSample,immune-CD4_T; Homo_633,totalSample,immune-CD4_naive; Homo_633,totalSample,immune-Effector_memory; Homo_633,totalSample,immune-Central_memory; Homo_633,totalSample,immune-Tr1; Homo_633,totalSample,immune-InfiltrationScore; Homo_633,totalSample,immune-Monocyte; Homo_633,totalSample,immune-Th17; Homo_633,totalSample,immune-Exhausted; Homo_633,totalSample,immune-Gamma_delta; Homo_633,totalSample,immune-iTreg; Homo_633,totalSample,immune-NKT; Homo_633,totalSample,immune-nTreg; Homo_633,totalSample,immune-DC; Homo_633,totalSample,immune-Bcell; MCSA,totalSample,immune-DC; MCSA,totalSample,immune-Macrophage; MCSA,totalSample,immune-CD4_T; MCSA,totalSample,immune-CD8_T; MCSA,totalSample,immune-CD4_naive; MCSA,totalSample,immune-Tr1; MCSA,totalSample,immune-nTreg; MCSA,totalSample,immune-iTreg; MCSA,totalSample,immune-Th17; MCSA,totalSample,immune-Tfh; MCSA,totalSample,immune-CD8_naive; MCSA,totalSample,immune-Cytotoxic; MCSA,totalSample,immune-Exhausted; [illegible]
CDON	Homo_723,female,AD.vs.control; Homo_723,male,AD.vs.control; Homo_723,female,AD.vs.MCI; Homo_723,male,AD.vs.MCI; Homo_723,female,MCI.vs.control; Homo_714,female,AD.vs.control; Homo_714,male,AD.vs.control; Homo_714,female,AD.vs.MCI; Homo_714,male,AD.vs.MCI; Homo_633,female,AD.vs.control; Homo_633,female,AD.vs.MCI; Emory,MCI,age; ROSMAP,MCI,last.visit.age; ROSMAP,MCI,sample.age; Homo_723,totalSample,immune-CD4_naive; Homo_723,totalSample,immune-CD4_T; Homo_723,totalSample,immune-Tfh; Homo_723,totalSample,immune-nTreg; Homo_723,totalSample,immune-Central_memory; Homo_723,totalSample,immune-Tr1; Homo_723,totalSample,immune-Th2; Homo_723,totalSample,immune-iTreg; Homo_723,totalSample,immune-Th17; Homo_723,totalSample,immune-Th1; Homo_714,totalSample,immune-Tfh; Homo_714,totalSample,immune-CD4_naive; Homo_714,totalSample,immune-CD4_T; Homo_714,totalSample,immune-Central_memory; Homo_714,totalSample,immune-nTreg; Homo_714,totalSample,immune-Tr1; Homo_714,totalSample,immune-iTreg; Homo_714,totalSample,immune-Th2; Homo_714,totalSample,immune-Th17; Homo_633,totalSample,immune-Tfh; Homo_633,totalSample,immune-CD4_T; Homo_633,totalSample,immune-Central_memory; Homo_633,totalSample,immune-CD4_naive; Homo_633,totalSample,immune-nTreg; Homo_633,totalSample,immune-Tr1; Homo_633,totalSample,immune-iTreg; Homo_633,totalSample,immune-Th2; Homo_633,totalSample,immune-Effector_memory; Homo_633,totalSample,immune-Gamma_delta; Homo_633,totalSample,immune-Th17; MCSA,totalSample,immune-Macrophage; MCSA,totalSample,immune-Neutrophil; MCSA,totalSample,immune-CD4_T; MCSA,totalSample,immune-CD8_T; MCSA,totalSample,immune-CD4_naive; MCSA,totalSample,immune-Tr1; MCSA,totalSample,immune-nTreg; MCSA,totalSample,immune-iTreg; MCSA,totalSample,immune-Th17; MCSA,totalSample,immune-Tfh; MCSA,totalSample,immune-CD8_naive; MCSA,totalSample,immune-MAIT; MCSA,totalSample,immune-Central_memory; MCSA,totalSample,immune- [illegible]
TLK1	Homo_723,female,AD.vs.control; Homo_723,male,AD.vs.control; Homo_723,female,AD.vs.MCI; Homo_723,male,AD.vs.MCI; Homo_723,female,MCI.vs.control; Homo_723,male,MCI.vs.control; Homo_714,female,AD.vs.control; Homo_714,male,AD.vs.control; Homo_714,female,AD.vs.MCI; Homo_714,male,AD.vs.MCI; Homo_714,female,MCI.vs.control; Homo_714,male,MCI.vs.control; Homo_633,female,AD.vs.control; Homo_633,female,AD.vs.MCI; MCSA,MCI,age; ROSMAP,MCI,sample.age; Emory,totalSample,immune-Macrophage; Emory,totalSample,immune-Neutrophil; Emory,totalSample,immune-CD4_T; Emory,totalSample,immune-CD8_T; Emory,totalSample,immune-Gamma_delta; Emory,totalSample,immune-Tr1; Emory,totalSample,immune-iTreg; Emory,totalSample,immune-Tfh; Emory,totalSample,immune-Central_memory; Emory,totalSample,immune-InfiltrationScore; Homo_723,totalSample,immune-Cytotoxic; Homo_723,totalSample,immune-Th17; Homo_723,totalSample,immune-CD4_naive; Homo_723,totalSample,immune-Th1; Homo_723,totalSample,immune-Central_memory; Homo_723,totalSample,immune-CD4_T; Homo_723,totalSample,immune-Tr1; Homo_723,totalSample,immune-Exhausted; Homo_723,totalSample,immune-Tfh; Homo_723,totalSample,immune-CD8_T; Homo_723,totalSample,immune-Gamma_delta; Homo_723,totalSample,immune-Th2; Homo_723,totalSample,immune-NKT; Homo_714,totalSample,immune-CD4_naive; Homo_714,totalSample,immune-Th1; Homo_714,totalSample,immune-NKT; Homo_714,totalSample,immune-Th17; Homo_714,totalSample,immune-CD4_T; Homo_714,totalSample,immune-Central_memory; Homo_714,totalSample,immune-Tfh; Homo_714,totalSample,immune-Monocyte; Homo_714,totalSample,immune-Tr1; Homo_714,totalSample,immune-Exhausted; Homo_714,totalSample,immune-Cytotoxic; Homo_714,totalSample,immune-Gamma_delta; Homo_633,totalSample,immune-Tfh; Homo_633,totalSample,immune-CD4_T; Homo_633,totalSample,immune-Th2; Homo_633,totalSample,immune-NKT; Homo_633,totalSample,immune-Tr1; Homo_633,totalSample,immune-CD4_naive; Homo_633,totalSample,immune-Central_memory; Homo_633,totalSample,immune-Gamma_delta; Homo_633,totalSample,immune-Th1; Homo_633,totalSample,immune-iTreg; Homo_633,totalSample,immune-DC; Homo_633,totalSample,immune-Monocyte; Homo_633,totalSample,immune-Th17; MCSA,totalSample,immune-DC; MCSA,totalSample,immune-Bcell; MCSA,totalSample,immune-Monocyte; MCSA,totalSample,immune-Macrophage; MCSA,totalSample,immune-CD4_T; MCSA,totalSample,immune-CD8_T; MCSA,totalSample,immune-CD4_naive; MCSA,totalSample,immune-Tr1; MCSA,totalSample,immune-nTreg; MCSA,totalSample,immune-iTreg; MCSA,totalSample,immune-Th2; MCSA,totalSample,immune-Tfh; MCSA,totalSample,immune-CD8_naive; MCSA,totalSample,immune-Cytotoxic; MCSA,totalSample,immune-Exhausted; MCSA,totalSample,immune-MAIT; MCSA,totalSample,immune-Central_memory; ROSMAP,totalSample,immune-Effector_memory; SRP223445,totalSample,immune-CD4_naive; SRP223445,totalSample,immune-Exhausted
ABCC4	Homo_723,female,AD.vs.control; Homo_723,male,AD.vs.control; Homo_723,female,AD.vs.MCI; Homo_723,male,AD.vs.MCI; Homo_723,female,MCI.vs.control; Homo_723,male,MCI.vs.control; Homo_714,female,AD.vs.control; Homo_714,male,AD.vs.control; Homo_714,female,AD.vs.MCI; Homo_714,male,AD.vs.MCI; Homo_714,female,MCI.vs.control; Homo_714,male,MCI.vs.control; Homo_633,female,AD.vs.control; Homo_633,female,AD.vs.MCI; ROSMAP,female,AD.vs.control; Emory,MCI,age; Homo_723,totalSample,immune-Th17; Homo_723,totalSample,immune-Exhausted; Homo_723,totalSample,immune-Cytotoxic; Homo_723,totalSample,immune-CD4_naive; Homo_723,totalSample,immune-CD8_T; Homo_723,totalSample,immune-CD4_T; Homo_723,totalSample,immune-Central_memory; Homo_723,totalSample,immune-Tfh; Homo_723,totalSample,immune-Tr1; Homo_723,totalSample,immune-Gamma_delta; Homo_714,totalSample,immune-Th17; Homo_714,totalSample,immune-Exhausted; Homo_714,totalSample,immune-CD4_naive; Homo_714,totalSample,immune-Monocyte; Homo_714,totalSample,immune-Central_memory; Homo_714,totalSample,immune-Tfh; Homo_714,totalSample,immune-CD4_T; Homo_714,totalSample,immune-Gamma_delta; Homo_633,totalSample,immune-Tfh; Homo_633,totalSample,immune-CD4_T; Homo_633,totalSample,immune-Th2; Homo_633,totalSample,immune-Central_memory; Homo_633,totalSample,immune-CD4_naive; Homo_633,totalSample,immune-Gamma_delta; Homo_633,totalSample,immune-Tr1; Homo_633,totalSample,immune-Effector_memory; Homo_633,totalSample,immune-Th17; Homo_633,totalSample,immune-InfiltrationScore; Homo_633,totalSample,immune-Monocyte; Homo_633,totalSample,immune-NKT; Homo_633,totalSample,immune-Exhausted; Homo_633,totalSample,immune-iTreg; MCSA,totalSample,immune-DC; MCSA,totalSample,immune-CD4_T; MCSA,totalSample,immune-CD8_T; MCSA,totalSample,immune-CD4_naive; MCSA,totalSample,immune-Tr1; MCSA,totalSample,immune-nTreg; MCSA,totalSample,immune-iTreg; MCSA,totalSample,immune-Th17; MCSA,totalSample,immune-Tfh; MCSA,totalSample,immune-CD8_naive; MCSA,totalSample,immune-Cytotoxic; MCSA,totalSample,immune-Exhausted; MCSA,totalSample,immune-MAIT; MCSA,totalSample,immune-Central_memory; ROSMAP,totalSample,immune-InfiltrationScore; SRP223445,totalSample,immune-CD4_naive; SRP223445,totalSample,immune-Exhausted
LAMB2	Homo_723,female,AD.vs.control; Homo_723,male,AD.vs.control; Homo_723,female,AD.vs.MCI; Homo_723,male,AD.vs.MCI; Homo_723,female,MCI.vs.control; Homo_714,female,AD.vs.control; Homo_714,female,AD.vs.MCI; Homo_633,female,AD.vs.control; Homo_633,female,AD.vs.MCI; Emory,MCI,age; MCSA,totalSample,age; MCSA,control,age; MCSA,MCI,age; Emory,totalSample,immune-Macrophage; Emory,totalSample,immune-CD8_T; Emory,totalSample,immune-Tfh; Homo_723,totalSample,immune-DC; Homo_714,totalSample,immune-DC; Homo_633,totalSample,immune-DC; Homo_633,totalSample,immune-InfiltrationScore; MCSA,totalSample,immune-DC; MCSA,totalSample,immune-Monocyte; MCSA,totalSample,immune-Macrophage; MCSA,totalSample,immune-Gamma_delta; MCSA,totalSample,immune-Th2; MCSA,totalSample,immune-Cytotoxic; MCSA,totalSample,immune-Effector_memory; MCSA,totalSample,immune-InfiltrationScore; ROSMAP,totalSample,immune-Effector_memory; ROSMAP,totalSample,immune-InfiltrationScore
MAP9	Homo_723,female,AD.vs.control; Homo_723,male,AD.vs.control; Homo_723,female,AD.vs.MCI; Homo_723,male,AD.vs.MCI; Homo_714,female,AD.vs.control; Homo_714,male,AD.vs.control; Homo_714,female,AD.vs.MCI; Homo_714,male,AD.vs.MCI; Homo_633,female,AD.vs.control; Homo_633,female,AD.vs.MCI; ROSMAP,female,AD.vs.control; ROSMAP,female,MCI.vs.control; Emory,totalSample,age; Emory,control,age; MCSA,MCI,age; ROSMAP,AD,sample.age; Emory,totalSample,immune-B_cell; Emory,totalSample,immune-Macrophage; Emory,totalSample,immune-NK; Emory,totalSample,immune-Neutrophil; Emory,totalSample,immune-CD4_T; Emory,totalSample,immune-CD8_T; Emory,totalSample,immune-Tr1; Emory,totalSample,immune-iTreg; Emory,totalSample,immune-Tfh; Emory,totalSample,immune-CD8_naive; Emory,totalSample,immune-Exhausted; Emory,totalSample,immune-InfiltrationScore; Homo_723,totalSample,immune-CD4_naive; Homo_723,totalSample,immune-CD4_T; Homo_723,totalSample,immune-Tfh; Homo_723,totalSample,immune-iTreg; Homo_723,totalSample,immune-Tr1; Homo_723,totalSample,immune-Central_memory; Homo_723,totalSample,immune-nTreg; Homo_723,totalSample,immune-Th1; Homo_723,totalSample,immune-Th2; Homo_723,totalSample,immune-InfiltrationScore; Homo_723,totalSample,immune-NKT; Homo_723,totalSample,immune-Cytotoxic; Homo_723,totalSample,immune-Neutrophil; Homo_723,totalSample,immune-Effector_memory; Homo_714,totalSample,immune-CD4_naive; Homo_714,totalSample,immune-Tfh; Homo_714,totalSample,immune-iTreg; Homo_714,totalSample,immune-CD4_T; Homo_714,totalSample,immune-Tr1; Homo_714,totalSample,immune-NKT; Homo_714,totalSample,immune-Central_memory; Homo_714,totalSample,immune-InfiltrationScore; Homo_714,totalSample,immune-Effector_memory; Homo_714,totalSample,immune-Th1; Homo_714,totalSample,immune-nTreg; Homo_714,totalSample,immune-Neutrophil; Homo_714,totalSample,immune-Th2; Homo_633,totalSample,immune-Tfh; Homo_633,totalSample,immune-CD4_naive; Homo_633,totalSample,immune-CD4_T; Homo_633,totalSample,immune-Central_memory; Homo_633,totalSample,immune-NKT; Homo_633,totalSample,immune-iTreg; Homo_633,totalSample,immune-Tr1; Homo_633,totalSample,immune-Effector_memory; Homo_633,totalSample,immune-InfiltrationScore; Homo_633,totalSample,immune-nTreg; Homo_633,totalSample,immune-MAIT; Homo_633,totalSample,immune-Th1; Homo_633,totalSample,immune-Neutrophil; Homo_633,totalSample,immune-Macrophage; Homo_633,totalSample,immune-Th2; Homo_633,totalSample,immune-DC; MCSA,totalSample,immune-DC; MCSA,totalSample,immune-Bcell; MCSA,totalSample,immune-Macrophage; MCSA,totalSample,immune-CD4_T; MCSA,totalSample,immune-CD4_naive; MCSA,totalSample,immune-Tr1; MCSA,totalSample,immune-nTreg; MCSA,totalSample,immune-iTreg; MCSA,totalSample,immune-Th17; MCSA,totalSample,immune-Tfh; MCSA,totalSample,immune-CD8_naive; MCSA,totalSample,immune-Cytotoxic; MCSA,totalSample,immune-Exhausted; [illegible]
MAGED4	Homo_723,female,AD.vs.control; Homo_723,female,AD.vs.MCI; Homo_723,male,AD.vs.MCI; Homo_714,male,AD.vs.control; Homo_714,male,AD.vs.MCI; Homo_633,female,AD.vs.control; Homo_633,female,AD.vs.MCI; ROSMAP,control,male.vs.female; ROSMAP,MCI,male.vs.female; MCSA,totalSample,age; MCSA,control,age; MCSA,MCI,age; ROSMAP,AD,last.visit.age; ROSMAP,control,last.visit.age; Emory,totalSample,immune-B_cell; Emory,totalSample,immune-Monocyte; Emory,totalSample,immune-Macrophage; Emory,totalSample,immune-Neutrophil; Emory,totalSample,immune-CD4_T; Emory,totalSample,immune-CD8_T; Emory,totalSample,immune-Tr1; Emory,totalSample,immune-iTreg; Emory,totalSample,immune-Tfh; Emory,totalSample,immune-CD8_naive; Emory,totalSample,immune-Exhausted; Homo_723,totalSample,immune-Monocyte; Homo_723,totalSample,immune-nTreg; Homo_723,totalSample,immune-DC; Homo_723,totalSample,immune-CD8_T; Homo_723,totalSample,immune-Central_memory; Homo_723,totalSample,immune-CD4_T; Homo_723,totalSample,immune-Neutrophil; Homo_723,totalSample,immune-Exhausted; Homo_723,totalSample,immune-Tfh; Homo_723,totalSample,immune-CD4_naive; Homo_714,totalSample,immune-Monocyte; Homo_714,totalSample,immune-nTreg; Homo_714,totalSample,immune-InfiltrationScore; Homo_714,totalSample,immune-Macrophage; Homo_633,totalSample,immune-Monocyte; Homo_633,totalSample,immune-nTreg; Homo_633,totalSample,immune-InfiltrationScore; Homo_633,totalSample,immune-DC; Homo_633,totalSample,immune-iTreg; MCSA,totalSample,immune-DC; MCSA,totalSample,immune-Monocyte; MCSA,totalSample,immune-Neutrophil; MCSA,totalSample,immune-CD4_T; MCSA,totalSample,immune-CD8_T; MCSA,totalSample,immune-CD4_naive; MCSA,totalSample,immune-Tr1; MCSA,totalSample,immune-nTreg; MCSA,totalSample,immune-Th17; MCSA,totalSample,immune-Tfh; MCSA,totalSample,immune-Cytotoxic; MCSA,totalSample,immune-Central_memory; MCSA,totalSample,immune-Effector_memory; [illegible]
ANKUB1	Homo_723,female,AD.vs.control; Homo_723,male,AD.vs.control; Homo_723,female,AD.vs.MCI; Homo_723,male,AD.vs.MCI; Homo_714,female,AD.vs.control; Homo_714,male,AD.vs.control; Homo_714,female,AD.vs.MCI; Homo_714,male,AD.vs.MCI; Homo_633,female,AD.vs.control; Homo_633,female,AD.vs.MCI; MCSA,MCI,age; Emory,totalSample,immune-Neutrophil; Emory,totalSample,immune-Gamma_delta; Emory,totalSample,immune-Exhausted; Homo_723,totalSample,immune-Gamma_delta; Homo_723,totalSample,immune-InfiltrationScore; Homo_723,totalSample,immune-Neutrophil; Homo_723,totalSample,immune-Bcell; Homo_723,totalSample,immune-DC; Homo_723,totalSample,immune-iTreg; Homo_723,totalSample,immune-CD8_naive; Homo_714,totalSample,immune-Th2; Homo_714,totalSample,immune-Tfh; Homo_633,totalSample,immune-NKT; Homo_633,totalSample,immune-Exhausted; Homo_633,totalSample,immune-Gamma_delta; MCSA,totalSample,immune-DC; MCSA,totalSample,immune-Monocyte; MCSA,totalSample,immune-Neutrophil; MCSA,totalSample,immune-CD8_T; MCSA,totalSample,immune-iTreg; MCSA,totalSample,immune-Cytotoxic; MCSA,totalSample,immune-Exhausted; ROSMAP,totalSample,immune-Effector_memory
STK17A	Homo_723,female,AD.vs.control; Homo_723,male,AD.vs.control; Homo_723,female,AD.vs.MCI; Homo_723,male,AD.vs.MCI; Homo_714,female,AD.vs.control; Homo_714,male,AD.vs.control; Homo_714,female,AD.vs.MCI; Homo_714,male,AD.vs.MCI; Homo_633,female,AD.vs.control; Homo_633,female,AD.vs.MCI; MCSA,MCI,age; Emory,totalSample,immune-DC; Emory,totalSample,immune-Monocyte; Emory,totalSample,immune-Macrophage; Emory,totalSample,immune-CD4_T; Emory,totalSample,immune-CD8_T; Emory,totalSample,immune-Tr1; Emory,totalSample,immune-iTreg; Emory,totalSample,immune-Tfh; Emory,totalSample,immune-Central_memory; Emory,totalSample,immune-Effector_memory; Emory,totalSample,immune-InfiltrationScore; Homo_723,totalSample,immune-Tfh; Homo_723,totalSample,immune-Th2; Homo_723,totalSample,immune-DC; Homo_723,totalSample,immune-iTreg; Homo_723,totalSample,immune-Monocyte; Homo_723,totalSample,immune-Tr1; Homo_723,totalSample,immune-CD4_T; Homo_723,totalSample,immune-InfiltrationScore; Homo_723,totalSample,immune-Effector_memory; Homo_723,totalSample,immune-CD4_naive; Homo_723,totalSample,immune-Macrophage; Homo_723,totalSample,immune-Gamma_delta; Homo_723,totalSample,immune-MAIT; Homo_723,totalSample,immune-Bcell; Homo_714,totalSample,immune-Th2; Homo_714,totalSample,immune-Tfh; Homo_714,totalSample,immune-CD4_T; Homo_714,totalSample,immune-Tr1; Homo_714,totalSample,immune-Effector_memory; Homo_714,totalSample,immune-Monocyte; Homo_714,totalSample,immune-DC; Homo_714,totalSample,immune-Central_memory; Homo_714,totalSample,immune-InfiltrationScore; Homo_714,totalSample,immune-iTreg; Homo_714,totalSample,immune-CD4_naive; Homo_633,totalSample,immune-Th2; Homo_633,totalSample,immune-Tfh; Homo_633,totalSample,immune-Monocyte; Homo_633,totalSample,immune-CD4_T; Homo_633,totalSample,immune-InfiltrationScore; Homo_633,totalSample,immune-Effector_memory; Homo_633,totalSample,immune-CD4_naive; Homo_633,totalSample,immune-Central_memory; Homo_633,totalSample,immune-DC; Homo_633,totalSample,immune-Tr1; Homo_633,totalSample,immune-Th17; Homo_633,totalSample,immune-Exhausted; Homo_633,totalSample,immune-Bcell; MCSA,totalSample,immune-DC; MCSA,totalSample,immune-Monocyte; MCSA,totalSample,immune-Macrophage; MCSA,totalSample,immune-CD4_T; MCSA,totalSample,immune-CD8_T; MCSA,totalSample,immune-CD4_naive; MCSA,totalSample,immune-Tr1; MCSA,totalSample,immune-nTreg; MCSA,totalSample,immune-iTreg; MCSA,totalSample,immune-Th2; MCSA,totalSample,immune-Th17; MCSA,totalSample,immune-Tfh; MCSA,totalSample,immune-CD8_naive; MCSA,totalSample,immune-Cytotoxic; MCSA,totalSample,immune-Exhausted; MCSA,totalSample,immune-MAIT; MCSA,totalSample,immune-Central_memory; SRP223445,totalSample,immune-NK; SRP223445,totalSample,immune-Neutrophil; SRP223445,totalSample,immune-Th17; SRP223445,totalSample,immune-Exhausted; SRP223445,totalSample,immune- [illegible]
MCF2L	Homo_723,female,AD.vs.control; Homo_723,male,AD.vs.control; Homo_723,female,AD.vs.MCI; Homo_723,male,AD.vs.MCI; Homo_723,female,MCI.vs.control; Homo_723,male,MCI.vs.control; Homo_714,female,AD.vs.control; Homo_714,male,AD.vs.control; Homo_714,female,AD.vs.MCI; Homo_714,male,AD.vs.MCI; Homo_714,female,MCI.vs.control; Homo_714,male,MCI.vs.control; Homo_633,female,AD.vs.control; Homo_633,female,AD.vs.MCI; ROSMAP,female,AD.vs.control; SRP223445,male,AD.vs.control; MCSA,totalSample,age; MCSA,control,age; ROSMAP,AD,last.visit.age; ROSMAP,AD,sample.age; Emory,totalSample,immune-Monocyte; Emory,totalSample,immune-CD4_T; Emory,totalSample,immune-Gamma_delta; Emory,totalSample,immune-CD4_naive; Emory,totalSample,immune-Tr1; Emory,totalSample,immune-nTreg; Emory,totalSample,immune-iTreg; Emory,totalSample,immune-Th17; Emory,totalSample,immune-Tfh; Emory,totalSample,immune-CD8_naive; Emory,totalSample,immune-Cytotoxic; Emory,totalSample,immune-Central_memory; Emory,totalSample,immune-InfiltrationScore; Homo_723,totalSample,immune-nTreg; Homo_723,totalSample,immune-CD4_naive; Homo_723,totalSample,immune-Central_memory; Homo_723,totalSample,immune-CD4_T; Homo_723,totalSample,immune-Th17; Homo_723,totalSample,immune-Tfh; Homo_723,totalSample,immune-Tr1; Homo_723,totalSample,immune-Neutrophil; Homo_723,totalSample,immune-DC; Homo_723,totalSample,immune-iTreg; Homo_723,totalSample,immune-InfiltrationScore; Homo_723,totalSample,immune-Th2; Homo_723,totalSample,immune-Th1; Homo_723,totalSample,immune-Effector_memory; Homo_714,totalSample,immune-nTreg; Homo_714,totalSample,immune-CD4_naive; Homo_714,totalSample,immune-Central_memory; Homo_714,totalSample,immune-CD4_T; Homo_714,totalSample,immune-Tfh; Homo_714,totalSample,immune-iTreg; Homo_714,totalSample,immune-Th17; Homo_714,totalSample,immune-InfiltrationScore; Homo_714,totalSample,immune-Tr1; Homo_714,totalSample,immune-Effector_memory; Homo_714,totalSample,immune-Neutrophil; Homo_714,totalSample,immune-Th1; Homo_633,totalSample,immune-nTreg; Homo_633,totalSample,immune-Tfh; Homo_633,totalSample,immune-Central_memory; Homo_633,totalSample,immune-CD4_T; Homo_633,totalSample,immune-Tr1; Homo_633,totalSample,immune-CD4_naive; Homo_633,totalSample,immune-Effector_memory; Homo_633,totalSample,immune-iTreg; Homo_633,totalSample,immune-InfiltrationScore; Homo_633,totalSample,immune-Th2; Homo_633,totalSample,immune-Gamma_delta; Homo_633,totalSample,immune-Monocyte; Homo_633,totalSample,immune-Th17; Homo_633,totalSample,immune-Neutrophil; MCSA,totalSample,immune-Monocyte; MCSA,totalSample,immune-Neutrophil; MCSA,totalSample,immune-CD4_T; MCSA,totalSample,immune-CD8_T; MCSA,totalSample,immune-NKT; MCSA,totalSample,immune-Gamma_delta; MCSA,totalSample,immune-CD4_naive; MCSA,totalSample,immune-Tr1; MCSA,totalSample,immune-nTreg; MCSA,totalSample,immune-iTreg; MCSA,totalSample,immune-Th2; MCSA,totalSample,immune-Th17; MCSA,totalSample,immune-Tfh; MCSA,totalSample,immune-CD8_naive; MCSA,totalSample,immune-Exhausted; MCSA,totalSample,immune-Central_memory; MCSA,totalSample,immune-Effector_memory; MCSA,totalSample,immune-InfiltrationScore; ROSMAP,totalSample,immune-InfiltrationScore; SRP223445,totalSample,immune- [illegible]
ASAP3	Homo_723,female,AD.vs.control; Homo_723,male,AD.vs.control; Homo_723,female,AD.vs.MCI; Homo_723,male,AD.vs.MCI; Homo_723,female,MCI.vs.control; Homo_714,female,AD.vs.control; Homo_714,female,AD.vs.MCI; Homo_714,male,AD.vs.MCI; Homo_633,female,AD.vs.control; Homo_633,female,AD.vs.MCI; ROSMAP,female,MCI.vs.control; MCSA,totalSample,age; MCSA,control,age; MCSA,MCI,age; Homo_723,totalSample,immune-nTreg; Homo_723,totalSample,immune-Th17; Homo_723,totalSample,immune-CD4_naive; Homo_723,totalSample,immune-CD4_T; Homo_723,totalSample,immune-Central_memory; Homo_723,totalSample,immune-Neutrophil; Homo_723,totalSample,immune-Tfh; Homo_723,totalSample,immune-DC; Homo_723,totalSample,immune-Th2; Homo_723,totalSample,immune-InfiltrationScore; Homo_714,totalSample,immune-nTreg; Homo_714,totalSample,immune-Th17; Homo_714,totalSample,immune-CD4_naive; Homo_714,totalSample,immune-Central_memory; Homo_714,totalSample,immune-CD4_T; Homo_714,totalSample,immune-Tfh; Homo_714,totalSample,immune-InfiltrationScore; Homo_714,totalSample,immune-Neutrophil; Homo_633,totalSample,immune-Tfh; Homo_633,totalSample,immune-nTreg; Homo_633,totalSample,immune-Central_memory; Homo_633,totalSample,immune-CD4_T; Homo_633,totalSample,immune-Th2; Homo_633,totalSample,immune-Effector_memory; Homo_633,totalSample,immune-Tr1; Homo_633,totalSample,immune-CD4_naive; Homo_633,totalSample,immune-InfiltrationScore; Homo_633,totalSample,immune-Th17; Homo_633,totalSample,immune-iTreg; Homo_633,totalSample,immune-Gamma_delta; MCSA,totalSample,immune-DC; MCSA,totalSample,immune-Monocyte; MCSA,totalSample,immune-Neutrophil; MCSA,totalSample,immune-CD4_T; MCSA,totalSample,immune-Gamma_delta; MCSA,totalSample,immune-CD4_naive; MCSA,totalSample,immune-Tr1; MCSA,totalSample,immune-nTreg; MCSA,totalSample,immune-iTreg; MCSA,totalSample,immune-Th17; MCSA,totalSample,immune-Tfh; MCSA,totalSample,immune-Central_memory; MCSA,totalSample,immune- [illegible]
CFAP161	Homo_723,female,AD.vs.control; Homo_723,male,AD.vs.control; Homo_723,female,AD.vs.MCI; Homo_723,male,AD.vs.MCI; Homo_723,female,MCI.vs.control; Homo_723,male,MCI.vs.control; Homo_723,control,male.vs.female; Homo_714,female,AD.vs.control; Homo_714,male,AD.vs.control; Homo_714,female,AD.vs.MCI; Homo_714,male,AD.vs.MCI; Homo_714,female,MCI.vs.control; Homo_714,male,MCI.vs.control; Homo_714,control,male.vs.female; Homo_633,female,AD.vs.control; Homo_633,female,AD.vs.MCI; ROSMAP,female,AD.vs.control; ROSMAP,MCI,last.visit.age; ROSMAP,MCI,sample.age; ROSMAP,AD,MMSE; Emory,totalSample,immune-Exhausted; Homo_723,totalSample,immune-Th17; Homo_723,totalSample,immune-Exhausted; Homo_723,totalSample,immune-CD8_T; Homo_723,totalSample,immune-Gamma_delta; Homo_723,totalSample,immune-MAIT; Homo_714,totalSample,immune-Exhausted; Homo_714,totalSample,immune-Th17; Homo_714,totalSample,immune-CD8_T; Homo_714,totalSample,immune-Gamma_delta; Homo_633,totalSample,immune-Gamma_delta; Homo_633,totalSample,immune-Th17; Homo_633,totalSample,immune-Exhausted; MCSA,totalSample,immune-Th17; ROSMAP,totalSample,immune-Th17; ROSMAP,totalSample,immune-InfiltrationScore; SRP223445,totalSample,immune-CD4_naive; SRP223445,totalSample,immune-Exhausted; ROSMAP,female-

PSMA5	Homo_723,female,AD.vs.control; Homo_723,male,AD.vs.control; Homo_723,female,AD.vs.MCI; Homo_723,male,AD.vs.MCI; Homo_714,female,AD.vs.control; Homo_714,male,AD.vs.control; Homo_714,female,AD.vs.MCI; Homo_714,male,AD.vs.MCI; Homo_633,female,AD.vs.control; Homo_633,female,AD.vs.MCI; ROSMAP,female,AD.vs.control; ROSMAP,MCI,last.visit.age; Emory,totalSample,immune-Macrophage; Emory,totalSample,immune-NK; Emory,totalSample,immune-Neutrophil; Emory,totalSample,immune-CD4_T; Emory,totalSample,immune-CD8_T; Emory,totalSample,immune-Tr1; Emory,totalSample,immune-iTreg; Emory,totalSample,immune-Th1; Emory,totalSample,immune-Tfh; Emory,totalSample,immune-Exhausted; Emory,totalSample,immune-Effector_memory; Emory,totalSample,immune-InfiltrationScore; Homo_723,totalSample,immune-Th2; Homo_723,totalSample,immune-Tfh; Homo_723,totalSample,immune-Monocyte; Homo_723,totalSample,immune-Effector_memory; Homo_723,totalSample,immune-InfiltrationScore; Homo_723,totalSample,immune-iTreg; Homo_723,totalSample,immune-DC; Homo_723,totalSample,immune-CD4_T; Homo_723,totalSample,immune-Bcell; Homo_723,totalSample,immune-Tr1; Homo_723,totalSample,immune-CD4_naive; Homo_723,totalSample,immune-Macrophage; Homo_714,totalSample,immune-Th2; Homo_714,totalSample,immune-Tfh; Homo_714,totalSample,immune-Effector_memory; Homo_714,totalSample,immune-Monocyte; Homo_714,totalSample,immune-CD4_T; Homo_714,totalSample,immune-InfiltrationScore; Homo_714,totalSample,immune-Bcell; Homo_714,totalSample,immune-Tr1; Homo_633,totalSample,immune-Th2; Homo_633,totalSample,immune-Monocyte; Homo_633,totalSample,immune-Tfh; Homo_633,totalSample,immune-InfiltrationScore; Homo_633,totalSample,immune-Bcell; Homo_633,totalSample,immune-Effector_memory; Homo_633,totalSample,immune-CD4_T; Homo_633,totalSample,immune-CD4_naive; MCSA,totalSample,immune-DC; MCSA,totalSample,immune-Macrophage; MCSA,totalSample,immune-CD4_T; MCSA,totalSample,immune-NKT; MCSA,totalSample,immune-Gamma_delta; MCSA,totalSample,immune-CD4_naive; MCSA,totalSample,immune-Tr1; MCSA,totalSample,immune-nTreg; MCSA,totalSample,immune-iTreg; MCSA,totalSample,immune-Th1; MCSA,totalSample,immune-Th2; MCSA,totalSample,immune-Th17; MCSA,totalSample,immune-Tfh; MCSA,totalSample,immune-MAIT; MCSA,totalSample,immune-InfiltrationScore; ROSMAP,totalSample,immune-iTreg; ROSMAP,totalSample,immune-Th17; ROSMAP,totalSample,immune-Effector_memory; ROSMAP,totalSample,immune-InfiltrationScore; SRP223445,totalSample,immune-NK; SRP223445,totalSample,immune-CD4_naive; [illegible]
CLIC6	Homo_723,female,AD.vs.control; Homo_723,female,AD.vs.MCI; Homo_723,AD,male.vs.female; Homo_714,male,AD.vs.control; Homo_714,male,AD.vs.MCI; Homo_633,female,AD.vs.control; Homo_633,female,AD.vs.MCI; Homo_633,control,male.vs.female; Homo_723,totalSample,immune-Th2; Homo_633,totalSample,immune-Tfh; Homo_633,totalSample,immune-Th2; MCSA,totalSample,immune-CD4_T; MCSA,totalSample,immune-CD4_naive; MCSA,totalSample,immune-Tr1; MCSA,totalSample,immune-nTreg; MCSA,totalSample,immune-iTreg; MCSA,totalSample,immune-Th17; MCSA,totalSample,immune-Tfh; MCSA,totalSample,immune-CD8_naive; MCSA,totalSample,immune-Central_memory; SRP223445,totalSample,immune-
TNFRSF10D	Homo_723,female,AD.vs.control; Homo_723,male,AD.vs.control; Homo_723,female,AD.vs.MCI; Homo_723,male,AD.vs.MCI; Homo_723,female,MCI.vs.control; Homo_723,male,MCI.vs.control; Homo_714,female,AD.vs.control; Homo_714,male,AD.vs.control; Homo_714,female,AD.vs.MCI; Homo_714,male,AD.vs.MCI; Homo_714,female,MCI.vs.control; Homo_714,male,MCI.vs.control; Homo_633,female,AD.vs.control; Homo_633,female,AD.vs.MCI; Homo_633,female,MCI.vs.control; Homo_633,male,MCI.vs.control; MCSA,totalSample,age; MCSA,control,age; Emory,totalSample,immune-Macrophage; Emory,totalSample,immune-CD4_T; Emory,totalSample,immune-Gamma_delta; Emory,totalSample,immune-CD4_naive; Emory,totalSample,immune-Tr1; Emory,totalSample,immune-iTreg; Emory,totalSample,immune-Th17; Emory,totalSample,immune-Tfh; Emory,totalSample,immune-Cytotoxic; Emory,totalSample,immune-MAIT; Emory,totalSample,immune-Central_memory; Homo_723,totalSample,immune-Th2; Homo_723,totalSample,immune-Tfh; Homo_723,totalSample,immune-iTreg; Homo_723,totalSample,immune-CD4_T; Homo_723,totalSample,immune-Tr1; Homo_723,totalSample,immune-CD4_naive; Homo_723,totalSample,immune-Effector_memory; Homo_723,totalSample,immune-nTreg; Homo_723,totalSample,immune-Monocyte; Homo_723,totalSample,immune-DC; Homo_723,totalSample,immune-Central_memory; Homo_723,totalSample,immune-Gamma_delta; Homo_723,totalSample,immune-Macrophage; Homo_723,totalSample,immune-InfiltrationScore; Homo_723,totalSample,immune-Bcell; Homo_714,totalSample,immune-Th2; Homo_714,totalSample,immune-Tfh; Homo_714,totalSample,immune-CD4_T; Homo_714,totalSample,immune-Tr1; Homo_714,totalSample,immune-Central_memory; Homo_714,totalSample,immune-Effector_memory; Homo_714,totalSample,immune-nTreg; Homo_714,totalSample,immune-CD4_naive; Homo_714,totalSample,immune-iTreg; Homo_714,totalSample,immune-Gamma_delta; Homo_714,totalSample,immune-Th17; Homo_633,totalSample,immune-Th2; Homo_633,totalSample,immune-Tfh; Homo_633,totalSample,immune-CD4_T; Homo_633,totalSample,immune-Central_memory; Homo_633,totalSample,immune-Tr1; Homo_633,totalSample,immune-Th17; Homo_633,totalSample,immune-nTreg; Homo_633,totalSample,immune-CD4_naive; Homo_633,totalSample,immune-Effector_memory; Homo_633,totalSample,immune-Gamma_delta; Homo_633,totalSample,immune-Exhausted; Homo_633,totalSample,immune-iTreg; Homo_633,totalSample,immune-Monocyte; MCSA,totalSample,immune-Monocyte; MCSA,totalSample,immune-CD4_T; MCSA,totalSample,immune-Gamma_delta; MCSA,totalSample,immune-CD4_naive; MCSA,totalSample,immune-Tr1; MCSA,totalSample,immune-nTreg; MCSA,totalSample,immune-iTreg; MCSA,totalSample,immune-Th17; MCSA,totalSample,immune-Tfh; MCSA,totalSample,immune-CD8_naive; MCSA,totalSample,immune-Cytotoxic; MCSA,totalSample,immune-Exhausted; MCSA,totalSample,immune-Central_memory; MCSA,totalSample,immune-Effector_memory
SLC13A4	Homo_723,female,AD.vs.control; Homo_723,female,AD.vs.MCI; Homo_723,male,AD.vs.MCI; Homo_714,female,AD.vs.control; Homo_714,male,AD.vs.control; Homo_714,female,AD.vs.MCI; Homo_714,male,AD.vs.MCI; Homo_633,female,AD.vs.control; Homo_633,female,AD.vs.MCI; ROSMAP,female,AD.vs.control; ROSMAP,male,AD.vs.control; MCSA,totalSample,age; MCSA,control,age; Emory,totalSample,immune-Gamma_delta; Emory,totalSample,immune-nTreg; Emory,totalSample,immune-iTreg; Emory,totalSample,immune-Th17; Emory,totalSample,immune-Cytotoxic; Homo_723,totalSample,immune-iTreg; Homo_723,totalSample,immune-Th2; Homo_723,totalSample,immune-nTreg; Homo_723,totalSample,immune-CD4_T; Homo_723,totalSample,immune-Tfh; Homo_723,totalSample,immune-CD8_naive; Homo_723,totalSample,immune-Tr1; Homo_723,totalSample,immune-CD8_T; Homo_723,totalSample,immune-Central_memory; Homo_723,totalSample,immune-CD4_naive; Homo_723,totalSample,immune-Monocyte; Homo_723,totalSample,immune-Macrophage; Homo_723,totalSample,immune-InfiltrationScore; Homo_714,totalSample,immune-Tfh; Homo_714,totalSample,immune-nTreg; Homo_714,totalSample,immune-Th2; Homo_714,totalSample,immune-Tr1; Homo_714,totalSample,immune-CD4_T; Homo_714,totalSample,immune-iTreg; Homo_714,totalSample,immune-Effector_memory; Homo_714,totalSample,immune-InfiltrationScore; Homo_714,totalSample,immune-Monocyte; Homo_714,totalSample,immune-Macrophage; Homo_633,totalSample,immune-nTreg; Homo_633,totalSample,immune-InfiltrationScore; Homo_633,totalSample,immune-Tfh; Homo_633,totalSample,immune-CD4_T; Homo_633,totalSample,immune-Tr1; Homo_633,totalSample,immune-iTreg; Homo_633,totalSample,immune-CD4_naive; Homo_633,totalSample,immune-Monocyte; Homo_633,totalSample,immune-Effector_memory; Homo_633,totalSample,immune-Central_memory; MCSA,totalSample,immune-Monocyte; MCSA,totalSample,immune-CD4_T; MCSA,totalSample,immune-Gamma_delta; MCSA,totalSample,immune-CD4_naive; MCSA,totalSample,immune-Tr1; MCSA,totalSample,immune-nTreg; MCSA,totalSample,immune-iTreg; MCSA,totalSample,immune-Th17; MCSA,totalSample,immune-Tfh; MCSA,totalSample,immune-CD8_naive; MCSA,totalSample,immune-Central_memory; MCSA,totalSample,immune-Effector_memory; [illegible]
ADGRE3	Homo_723,female,AD.vs.control; Homo_723,male,AD.vs.control; Homo_723,female,AD.vs.MCI; Homo_723,male,AD.vs.MCI; Homo_723,female,MCI.vs.control; Homo_723,male,MCI.vs.control; Homo_714,male,AD.vs.control; Homo_714,female,AD.vs.MCI; Homo_714,female,MCI.vs.control; Homo_714,male,MCI.vs.control; Homo_714,control,male.vs.female; Homo_714,MCI,male.vs.female; Homo_633,female,AD.vs.control; Homo_633,female,AD.vs.MCI; Homo_633,female,MCI.vs.control; Homo_633,male,MCI.vs.control; Homo_633,control,male.vs.female; Homo_633,MCI,male.vs.female; ROSMAP,female,AD.vs.control; ROSMAP,male,AD.vs.MCI; ROSMAP,control,male.vs.female; SRP223445,female,AD.vs.control; Emory,MCI,age; Emory,totalSample,immune-Monocyte; Emory,totalSample,immune-NK; Emory,totalSample,immune-Neutrophil; Emory,totalSample,immune-CD8_T; Emory,totalSample,immune-Gamma_delta; Emory,totalSample,immune-Th1; Emory,totalSample,immune-Th17; Emory,totalSample,immune-Tfh; Emory,totalSample,immune-CD8_naive; Emory,totalSample,immune-Cytotoxic; Emory,totalSample,immune-Exhausted; Emory,totalSample,immune-InfiltrationScore; Homo_723,totalSample,immune-Neutrophil; Homo_723,totalSample,immune-Gamma_delta; Homo_723,totalSample,immune-CD8_naive; Homo_723,totalSample,immune-Exhausted; Homo_714,totalSample,immune-CD8_T; Homo_714,totalSample,immune-Neutrophil; Homo_633,totalSample,immune-Neutrophil; MCSA,totalSample,immune-NK; MCSA,totalSample,immune-Neutrophil; MCSA,totalSample,immune-CD8_T; MCSA,totalSample,immune-Gamma_delta; MCSA,totalSample,immune-Th1; MCSA,totalSample,immune-Th17; MCSA,totalSample,immune-Cytotoxic; MCSA,totalSample,immune-Exhausted; MCSA,totalSample,immune-MAIT; MCSA,totalSample,immune-Effector_memory; ROSMAP,totalSample,immune-Effector_memory; SRP223445,totalSample,immune-Neutrophil; SRP223445,totalSample,immune-CD4_naive
TDRP	Homo_723,female,AD.vs.control; Homo_723,male,AD.vs.control; Homo_723,female,AD.vs.MCI; Homo_723,male,AD.vs.MCI; Homo_723,female,MCI.vs.control; Homo_723,male,MCI.vs.control; Homo_714,female,AD.vs.control; Homo_714,male,AD.vs.control; Homo_714,female,AD.vs.MCI; Homo_714,male,AD.vs.MCI; Homo_714,female,MCI.vs.control; Homo_714,male,MCI.vs.control; Homo_633,female,AD.vs.control; Homo_633,female,AD.vs.MCI; Emory,totalSample,immune-NK; Emory,totalSample,immune-Neutrophil; Emory,totalSample,immune-Gamma_delta; Emory,totalSample,immune-Th1; Emory,totalSample,immune-Exhausted; Emory,totalSample,immune-InfiltrationScore; Homo_723,totalSample,immune-Exhausted; Homo_723,totalSample,immune-Th17; Homo_723,totalSample,immune-CD8_T; Homo_723,totalSample,immune-Cytotoxic; Homo_723,totalSample,immune-Monocyte; Homo_714,totalSample,immune-Exhausted; Homo_714,totalSample,immune-Th17; Homo_714,totalSample,immune-Monocyte; Homo_714,totalSample,immune-CD8_T; Homo_633,totalSample,immune-Monocyte; Homo_633,totalSample,immune-Th2; Homo_633,totalSample,immune-Exhausted; Homo_633,totalSample,immune-Gamma_delta; Homo_633,totalSample,immune-Th17; Homo_633,totalSample,immune-Tfh; MCSA,totalSample,immune-Monocyte; MCSA,totalSample,immune-Tr1; MCSA,totalSample,immune-iTreg; MCSA,totalSample,immune-Cytotoxic; MCSA,totalSample,immune-Exhausted; SRP223445,totalSample,immune-CD4_naive; SRP223445,totalSample,immune-Exhausted; ROSMAP,female-excludeAge90+,survival; ROSMAP,male and female-excludeAge90+,survival
PARP12	Homo_723,female,AD.vs.control; Homo_723,female,AD.vs.MCI; Homo_723,male,AD.vs.MCI; Homo_723,female,MCI.vs.control; Homo_723,male,MCI.vs.control; Homo_723,AD,male.vs.female; Homo_723,control,male.vs.female; Homo_714,female,AD.vs.control; Homo_714,male,AD.vs.MCI; Homo_714,female,MCI.vs.control; Homo_714,male,MCI.vs.control; Homo_714,AD,male.vs.female; Homo_714,control,male.vs.female; Homo_633,female,AD.vs.control; Homo_633,female,AD.vs.MCI; Homo_633,female,MCI.vs.control; Homo_633,male,MCI.vs.control; Homo_633,control,male.vs.female; MCSA,totalSample,age; MCSA,control,age; MCSA,MCI,age; Emory,totalSample,immune-NKT; Emory,totalSample,immune-Tr1; Emory,totalSample,immune-iTreg; Emory,totalSample,immune-InfiltrationScore; Homo_723,totalSample,immune-Monocyte; Homo_723,totalSample,immune-DC; Homo_714,totalSample,immune-Monocyte; Homo_714,totalSample,immune-DC; Homo_714,totalSample,immune-Th1; Homo_633,totalSample,immune-Monocyte; Homo_633,totalSample,immune-Th1; Homo_633,totalSample,immune-InfiltrationScore; MCSA,totalSample,immune-DC; MCSA,totalSample,immune-Monocyte; MCSA,totalSample,immune-Neutrophil; MCSA,totalSample,immune-CD4_T; MCSA,totalSample,immune-Gamma_delta; MCSA,totalSample,immune-CD4_naive; MCSA,totalSample,immune-Tr1; MCSA,totalSample,immune-nTreg; MCSA,totalSample,immune-iTreg; MCSA,totalSample,immune-Th2; MCSA,totalSample,immune-Th17; MCSA,totalSample,immune-Tfh; MCSA,totalSample,immune-Central_memory; MCSA,totalSample,immune-Effector_memory; MCSA,totalSample,immune-InfiltrationScore; ROSMAP,totalSample,immune-Effector_memory; SRP223445,totalSample,immune-CD4_naive;
PBX1	Homo_723,female,AD.vs.control; Homo_723,male,AD.vs.control; Homo_723,female,AD.vs.MCI; Homo_723,male,AD.vs.MCI; Homo_723,female,MCI.vs.control; Homo_723,male,MCI.vs.control; Homo_714,female,AD.vs.control; Homo_714,male,AD.vs.control; Homo_714,male,AD.vs.MCI; Homo_714,female,MCI.vs.control; Homo_714,male,MCI.vs.control; Homo_633,female,AD.vs.control; Homo_633,female,AD.vs.MCI; ACOM,totalSample,age; Emory,MCI,age; ROSMAP,AD,last.visit.age; Emory,totalSample,immune-iTreg; Homo_723,totalSample,immune-Th17; Homo_723,totalSample,immune-DC; Homo_723,totalSample,immune-Bcell; Homo_723,totalSample,immune-CD8_T; Homo_714,totalSample,immune-Th17; Homo_714,totalSample,immune-DC; Homo_633,totalSample,immune-Bcell; Homo_633,totalSample,immune-Gamma_delta; MCSA,totalSample,immune-Macrophage; ROSMAP,totalSample,immune-InfiltrationScore; SRP223445,totalSample,immune-CD4_naive; SRP223445,totalSample,immune-Th1; SRP223445,totalSample,immune-
VPS16	Homo_723,female,AD.vs.control; Homo_723,male,AD.vs.control; Homo_723,female,AD.vs.MCI; Homo_714,female,AD.vs.control; Homo_714,male,AD.vs.control; Homo_714,female,AD.vs.MCI; Homo_714,male,AD.vs.MCI; Homo_633,female,AD.vs.control; Homo_633,female,AD.vs.MCI; ROSMAP,female,AD.vs.control; Emory,MCI,age; MCSA,totalSample,age; MCSA,control,age; ROSMAP,MCI,last.visit.age; ROSMAP,MCI,sample.age; Emory,totalSample,immune-NK; Emory,totalSample,immune-Neutrophil; Emory,totalSample,immune-CD4_T; Emory,totalSample,immune-Tr1; Emory,totalSample,immune-iTreg; Emory,totalSample,immune-Effector_memory; Emory,totalSample,immune-InfiltrationScore; Homo_723,totalSample,immune-Effector_memory; Homo_723,totalSample,immune-InfiltrationScore; Homo_723,totalSample,immune-Tfh; Homo_723,totalSample,immune-Th2; Homo_723,totalSample,immune-iTreg; Homo_723,totalSample,immune-Bcell; Homo_723,totalSample,immune-nTreg; Homo_723,totalSample,immune-Cytotoxic; Homo_723,totalSample,immune-CD8_T; Homo_723,totalSample,immune-CD4_T; Homo_723,totalSample,immune-CD4_naive; Homo_723,totalSample,immune-Th1; Homo_723,totalSample,immune-Macrophage; Homo_714,totalSample,immune-Effector_memory; Homo_714,totalSample,immune-InfiltrationScore; Homo_714,totalSample,immune-Tfh; Homo_714,totalSample,immune-Th2; Homo_714,totalSample,immune-nTreg; Homo_714,totalSample,immune-CD8_T; Homo_714,totalSample,immune-Central_memory; Homo_714,totalSample,immune-CD4_T; Homo_714,totalSample,immune-Th1; Homo_633,totalSample,immune-InfiltrationScore; Homo_633,totalSample,immune-Effector_memory; Homo_633,totalSample,immune-nTreg; Homo_633,totalSample,immune-Tfh; Homo_633,totalSample,immune-Central_memory; Homo_633,totalSample,immune-DC; Homo_633,totalSample,immune-Th2; Homo_633,totalSample,immune-CD4_T; Homo_633,totalSample,immune-Bcell; MCSA,totalSample,immune-DC; MCSA,totalSample,immune-Monocyte; MCSA,totalSample,immune-Neutrophil; MCSA,totalSample,immune-CD4_T; MCSA,totalSample,immune-CD8_T; MCSA,totalSample,immune-NKT; MCSA,totalSample,immune-CD4_naive; MCSA,totalSample,immune-Tr1; MCSA,totalSample,immune-nTreg; MCSA,totalSample,immune-Th2; MCSA,totalSample,immune-Th17; MCSA,totalSample,immune-Tfh; MCSA,totalSample,immune-Cytotoxic; MCSA,totalSample,immune-Central_memory; MCSA,totalSample,immune-Effector_memory; MCSA,totalSample,immune-InfiltrationScore; ROSMAP,totalSample,immune-iTreg; ROSMAP,totalSample,immune-Th17; ROSMAP,totalSample,immune-CD8_naive; ROSMAP,totalSample,immune-Effector_memory; ROSMAP,totalSample,immune-InfiltrationScore; [illegible]
RBM10	Homo_723,female,AD.vs.control; Homo_723,female,MCI.vs.control; Homo_723,AD,male.vs.female; Homo_723,control,male.vs.female; Homo_723,MCI,male.vs.female; Homo_714,female,AD.vs.control; Homo_714,male,AD.vs.control; Homo_714,male,AD.vs.MCI; Homo_714,female,MCI.vs.control; Homo_714,control,male.vs.female; Homo_714,MCI,male.vs.female; Homo_633,female,AD.vs.control; Homo_633,female,AD.vs.MCI; Homo_633,female,MCI.vs.control; Homo_633,control,male.vs.female; Homo_633,MCI,male.vs.female; ROSMAP,female,AD.vs.control; ROSMAP,male,AD.vs.control; Emory,totalSample,age; Emory,MCI,age; MCSA,totalSample,age; MCSA,control,age; MCSA,MCI,age; ROSMAP,MCI,last.visit.age; Emory,totalSample,immune-Monocyte; Emory,totalSample,immune-Tr1; Emory,totalSample,immune-nTreg; Emory,totalSample,immune-iTreg; Emory,totalSample,immune-Th17; Emory,totalSample,immune-CD8_naive; Emory,totalSample,immune-InfiltrationScore; Homo_723,totalSample,immune-Monocyte; Homo_723,totalSample,immune-DC; Homo_723,totalSample,immune-Central_memory; Homo_714,totalSample,immune-Monocyte; Homo_714,totalSample,immune-DC; Homo_633,totalSample,immune-Monocyte; Homo_633,totalSample,immune-nTreg; MCSA,totalSample,immune-DC; MCSA,totalSample,immune-Monocyte; MCSA,totalSample,immune-Macrophage; MCSA,totalSample,immune-Neutrophil; MCSA,totalSample,immune-CD8_T; MCSA,totalSample,immune-NKT; MCSA,totalSample,immune-Th2; MCSA,totalSample,immune-Cytotoxic; MCSA,totalSample,immune-Central_memory; MCSA,totalSample,immune-Effector_memory; MCSA,totalSample,immune-InfiltrationScore; ROSMAP,totalSample,immune-Effector_memory; SRP223445,totalSample,immune-
PRPF6	Homo_723,female,AD.vs.control; Homo_723,male,AD.vs.MCI; Homo_723,female,MCI.vs.control; Homo_723,male,MCI.vs.control; Homo_723,control,male.vs.female; Homo_723,MCI,male.vs.female; Homo_714,female,AD.vs.control; Homo_714,male,AD.vs.control; Homo_714,male,AD.vs.MCI; Homo_714,female,MCI.vs.control; Homo_714,male,MCI.vs.control; Homo_714,control,male.vs.female; Homo_714,MCI,male.vs.female; Homo_633,female,AD.vs.control; Homo_633,female,AD.vs.MCI; Homo_633,female,MCI.vs.control; Homo_633,male,MCI.vs.control; Homo_633,control,male.vs.female; Homo_633,MCI,male.vs.female; ROSMAP,female,AD.vs.control; Emory,MCI,age; MCSA,totalSample,age; MCSA,control,age; MCSA,MCI,age; ROSMAP,MCI,last.visit.age; Emory,totalSample,immune-DC; Emory,totalSample,immune-Monocyte; Emory,totalSample,immune-Neutrophil; Emory,totalSample,immune-CD4_T; Emory,totalSample,immune-CD4_naive; Emory,totalSample,immune-Tr1; Emory,totalSample,immune-nTreg; Emory,totalSample,immune-iTreg; Emory,totalSample,immune-Th17; Emory,totalSample,immune-Central_memory; Emory,totalSample,immune-InfiltrationScore; Homo_723,totalSample,immune-Monocyte; Homo_723,totalSample,immune-DC; Homo_723,totalSample,immune-Central_memory; Homo_723,totalSample,immune-Neutrophil; Homo_723,totalSample,immune-nTreg; Homo_723,totalSample,immune-CD8_T; Homo_723,totalSample,immune-CD4_naive; Homo_723,totalSample,immune-InfiltrationScore; Homo_723,totalSample,immune-Exhausted; Homo_714,totalSample,immune-Monocyte; Homo_714,totalSample,immune-DC; Homo_714,totalSample,immune-InfiltrationScore; Homo_714,totalSample,immune-nTreg; Homo_633,totalSample,immune-Monocyte; Homo_633,totalSample,immune-nTreg; Homo_633,totalSample,immune-InfiltrationScore; Homo_633,totalSample,immune-CD4_naive; MCSA,totalSample,immune-DC; MCSA,totalSample,immune-Monocyte; MCSA,totalSample,immune-Neutrophil; MCSA,totalSample,immune-CD8_T; MCSA,totalSample,immune-NKT; MCSA,totalSample,immune-CD4_naive; MCSA,totalSample,immune-nTreg; MCSA,totalSample,immune-Th2; MCSA,totalSample,immune-Tfh; MCSA,totalSample,immune-Cytotoxic; MCSA,totalSample,immune-Central_memory; MCSA,totalSample,immune-Effector_memory; MCSA,totalSample,immune-InfiltrationScore; ROSMAP,totalSample,immune-Th2; ROSMAP,totalSample,immune-Th17; ROSMAP,totalSample,immune-Effector_memory; SRP223445,totalSample,immune-CD4_naive; SRP223445,totalSample,immune-Th17; SRP223445,totalSample,immune-Exhausted; SRP223445,totalSample,immune-InfiltrationScore; ROSMAP,female-excludeAge90+,survival; ROSMAP,female-includeAge90+,survival
PDE4D	Homo_723,female,AD.vs.control; Homo_723,male,AD.vs.control; Homo_723,female,AD.vs.MCI; Homo_723,male,AD.vs.MCI; Homo_723,female,MCI.vs.control; Homo_723,male,MCI.vs.control; Homo_714,female,AD.vs.control; Homo_714,male,AD.vs.control; Homo_714,female,AD.vs.MCI; Homo_714,male,AD.vs.MCI; Homo_714,female,MCI.vs.control; Homo_714,male,MCI.vs.control; Homo_633,female,AD.vs.control; Homo_633,female,AD.vs.MCI; Homo_633,female,MCI.vs.control; SRP223445,female,AD.vs.control; Emory,totalSample,immune-Macrophage; Emory,totalSample,immune-CD4_T; Emory,totalSample,immune-Tr1; Emory,totalSample,immune-iTreg; Emory,totalSample,immune-Tfh; Emory,totalSample,immune-InfiltrationScore; Homo_723,totalSample,immune-Th17; Homo_723,totalSample,immune-Exhausted; Homo_723,totalSample,immune-CD4_naive; Homo_723,totalSample,immune-Cytotoxic; Homo_723,totalSample,immune-CD4_T; Homo_723,totalSample,immune-CD8_T; Homo_723,totalSample,immune-Tfh; Homo_723,totalSample,immune-Tr1; Homo_723,totalSample,immune-Th2; Homo_723,totalSample,immune-InfiltrationScore; Homo_723,totalSample,immune-Central_memory; Homo_723,totalSample,immune-Monocyte; Homo_723,totalSample,immune-Th1; Homo_723,totalSample,immune-NK; Homo_714,totalSample,immune-Th17; Homo_714,totalSample,immune-CD4_naive; Homo_714,totalSample,immune-CD4_T; Homo_714,totalSample,immune-Exhausted; Homo_714,totalSample,immune-Tfh; Homo_714,totalSample,immune-Monocyte; Homo_714,totalSample,immune-InfiltrationScore; Homo_714,totalSample,immune-Central_memory; Homo_714,totalSample,immune-NK; Homo_633,totalSample,immune-Tfh; Homo_633,totalSample,immune-Th2; Homo_633,totalSample,immune-CD4_T; Homo_633,totalSample,immune-CD4_naive; Homo_633,totalSample,immune-InfiltrationScore; Homo_633,totalSample,immune-Central_memory; Homo_633,totalSample,immune-Effector_memory; Homo_633,totalSample,immune-Th17; Homo_633,totalSample,immune-Tr1; Homo_633,totalSample,immune-Monocyte; Homo_633,totalSample,immune-iTreg; Homo_633,totalSample,immune-NKT; Homo_633,totalSample,immune-Exhausted; Homo_633,totalSample,immune-Bcell; Homo_633,totalSample,immune-Gamma_delta; Homo_633,totalSample,immune-DC; MCSA,totalSample,immune-DC; MCSA,totalSample,immune-Monocyte; MCSA,totalSample,immune-Macrophage; MCSA,totalSample,immune-CD4_T; MCSA,totalSample,immune-CD8_T; MCSA,totalSample,immune-CD4_naive; MCSA,totalSample,immune-Tr1; MCSA,totalSample,immune-nTreg; MCSA,totalSample,immune-iTreg; MCSA,totalSample,immune-Th17; MCSA,totalSample,immune-Tfh; MCSA,totalSample,immune-CD8_naive; MCSA,totalSample,immune-Cytotoxic; [illegible]
TMEM230	Homo_723,female,AD.vs.control; Homo_723,male,AD.vs.control; Homo_723,female,AD.vs.MCI; Homo_723,male,AD.vs.MCI; Homo_714,female,AD.vs.control; Homo_714,male,AD.vs.control; Homo_714,female,AD.vs.MCI; Homo_714,male,AD.vs.MCI; Homo_633,female,AD.vs.control; Homo_633,female,AD.vs.MCI; ROSMAP,female,AD.vs.control; ROSMAP,female,MCI.vs.control; ACOM,AD,age; ACOM,totalSample,age; MCSA,MCI,age; Emory,totalSample,immune-Monocyte; Emory,totalSample,immune-Macrophage; Emory,totalSample,immune-Neutrophil; Emory,totalSample,immune-CD4_T; Emory,totalSample,immune-CD8_T; Emory,totalSample,immune-Tr1; Emory,totalSample,immune-iTreg; Emory,totalSample,immune-Tfh; Emory,totalSample,immune-Central_memory; Emory,totalSample,immune-Effector_memory; Emory,totalSample,immune-InfiltrationScore; Homo_723,totalSample,immune-iTreg; Homo_723,totalSample,immune-Tfh; Homo_723,totalSample,immune-Th2; Homo_723,totalSample,immune-CD4_T; Homo_723,totalSample,immune-Tr1; Homo_723,totalSample,immune-CD4_naive; Homo_723,totalSample,immune-InfiltrationScore; Homo_723,totalSample,immune-Central_memory; Homo_723,totalSample,immune-MAIT; Homo_723,totalSample,immune-DC; Homo_723,totalSample,immune-Macrophage; Homo_723,totalSample,immune-Effector_memory; Homo_723,totalSample,immune-Gamma_delta; Homo_723,totalSample,immune-NKT; Homo_723,totalSample,immune-nTreg; Homo_714,totalSample,immune-Tfh; Homo_714,totalSample,immune-Th2; Homo_714,totalSample,immune-Tr1; Homo_714,totalSample,immune-CD4_T; Homo_714,totalSample,immune-Effector_memory; Homo_714,totalSample,immune-iTreg; Homo_714,totalSample,immune-Central_memory; Homo_714,totalSample,immune-CD4_naive; Homo_714,totalSample,immune-InfiltrationScore; Homo_714,totalSample,immune-MAIT; Homo_714,totalSample,immune-Macrophage; Homo_714,totalSample,immune-DC; Homo_633,totalSample,immune-Tfh; Homo_633,totalSample,immune-CD4_T; Homo_633,totalSample,immune-CD4_naive; Homo_633,totalSample,immune-InfiltrationScore; Homo_633,totalSample,immune-Effector_memory; Homo_633,totalSample,immune-Central_memory; Homo_633,totalSample,immune-NKT; Homo_633,totalSample,immune-Tr1; Homo_633,totalSample,immune-Th2; Homo_633,totalSample,immune-iTreg; Homo_633,totalSample,immune-MAIT; Homo_633,totalSample,immune-Th1; Homo_633,totalSample,immune-Bcell; Homo_633,totalSample,immune-Neutrophil; Homo_633,totalSample,immune-nTreg; MCSA,totalSample,immune-DC; MCSA,totalSample,immune-Monocyte; MCSA,totalSample,immune-Macrophage; MCSA,totalSample,immune-CD4_T; MCSA,totalSample,immune-CD8_T; MCSA,totalSample,immune-CD4_naive; MCSA,totalSample,immune-Tr1; MCSA,totalSample,immune-nTreg; MCSA,totalSample,immune-iTreg; MCSA,totalSample,immune-Th2; MCSA,totalSample,immune-Th17; MCSA,totalSample,immune-Tfh; MCSA,totalSample,immune-CD8_naive; MCSA,totalSample,immune-Cytotoxic; MCSA,totalSample,immune-Exhausted; [illegible]
BIRC5	Homo_723,female,AD.vs.control; Homo_723,male,AD.vs.control; Homo_723,female,AD.vs.MCI; Homo_723,male,AD.vs.MCI; Homo_714,female,AD.vs.control; Homo_714,male,AD.vs.control; Homo_714,female,AD.vs.MCI; Homo_714,male,AD.vs.MCI; Homo_633,female,AD.vs.control; Homo_633,female,AD.vs.MCI; ROSMAP,female,AD.vs.control; Emory,MCI,age; Homo_723,totalSample,immune-DC; Homo_723,totalSample,immune-Neutrophil; Homo_723,totalSample,immune-InfiltrationScore; Homo_714,totalSample,immune-DC; Homo_714,totalSample,immune-InfiltrationScore; Homo_714,totalSample,immune-Neutrophil; Homo_714,totalSample,immune-NK; Homo_633,totalSample,immune-DC; Homo_633,totalSample,immune-InfiltrationScore; MCSA,totalSample,immune-DC; MCSA,totalSample,immune-Monocyte; MCSA,totalSample,immune-Neutrophil; MCSA,totalSample,immune-InfiltrationScore; SRP223445,totalSample,immune-CD4_naive; SRP223445,totalSample,immune-InfiltrationScore; ROSMAP,male-excludeAge90+,survival

CEP128	Homo_723,female,AD.vs.control; Homo_723,male,AD.vs.control; Homo_723,female,AD.vs.MCI; Homo_723,male,AD.vs.MCI; Homo_714,female,AD.vs.control; Homo_714,male,AD.vs.control; Homo_714,female,AD.vs.MCI; Homo_714,male,AD.vs.MCI; Homo_633,female,AD.vs.control; Homo_633,female,AD.vs.MCI; Emory,totalSample,immune-Macrophage; Emory,totalSample,immune-NK; Emory,totalSample,immune-Neutrophil; Emory,totalSample,immune-CD4_T; Emory,totalSample,immune-CD8_T; Emory,totalSample,immune-Tr1; Emory,totalSample,immune-iTreg; Emory,totalSample,immune-Tfh; Emory,totalSample,immune-Exhausted; Emory,totalSample,immune-Effector_memory; Emory,totalSample,immune-InfiltrationScore; Homo_723,totalSample,immune-CD4_naive; Homo_723,totalSample,immune-CD4_T; Homo_723,totalSample,immune-Central_memory; Homo_723,totalSample,immune-Tr1; Homo_723,totalSample,immune-Tfh; Homo_723,totalSample,immune-InfiltrationScore; Homo_723,totalSample,immune-Th1; Homo_723,totalSample,immune-nTreg; Homo_723,totalSample,immune-Neutrophil; Homo_723,totalSample,immune-Th17; Homo_723,totalSample,immune-Cytotoxic; Homo_723,totalSample,immune-iTreg; Homo_723,totalSample,immune-NK; Homo_723,totalSample,immune-NKT; Homo_714,totalSample,immune-CD4_naive; Homo_714,totalSample,immune-InfiltrationScore; Homo_714,totalSample,immune-CD4_T; Homo_714,totalSample,immune-Tfh; Homo_714,totalSample,immune-Th1; Homo_714,totalSample,immune-Central_memory; Homo_714,totalSample,immune-NKT; Homo_714,totalSample,immune-Tr1; Homo_714,totalSample,immune-iTreg; Homo_714,totalSample,immune-Neutrophil; Homo_714,totalSample,immune-Effector_memory; Homo_714,totalSample,immune-nTreg; Homo_714,totalSample,immune-Bcell; Homo_633,totalSample,immune-Tfh; Homo_633,totalSample,immune-CD4_naive; Homo_633,totalSample,immune-CD4_T; Homo_633,totalSample,immune-InfiltrationScore; Homo_633,totalSample,immune-Effector_memory; Homo_633,totalSample,immune-Tr1; Homo_633,totalSample,immune-Central_memory; Homo_633,totalSample,immune-iTreg; Homo_633,totalSample,immune-NKT; Homo_633,totalSample,immune-nTreg; Homo_633,totalSample,immune-Bcell; Homo_633,totalSample,immune-Th1; Homo_633,totalSample,immune-MAIT; Homo_633,totalSample,immune-Neutrophil; Homo_633,totalSample,immune-Th2; MCSA,totalSample,immune-DC; MCSA,totalSample,immune-Macrophage; MCSA,totalSample,immune-CD4_T; MCSA,totalSample,immune-CD4_naive; MCSA,totalSample,immune-Tr1; MCSA,totalSample,immune-nTreg; MCSA,totalSample,immune-iTreg; MCSA,totalSample,immune-Tfh; MCSA,totalSample,immune-CD8_naive; MCSA,totalSample,immune-Cytotoxic; MCSA,totalSample,immune-[illegible]
FNDC5	Homo_723,female,AD.vs.control; Homo_723,male,AD.vs.control; Homo_723,female,AD.vs.MCI; Homo_723,male,AD.vs.MCI; Homo_714,male,AD.vs.control; Homo_714,male,AD.vs.MCI; Homo_633,female,AD.vs.control; Homo_633,female,AD.vs.MCI; ROSMAP,female,AD.vs.control; ROSMAP,female,AD.vs.MCI; MCSA,totalSample,age; MCSA,control,age; Homo_723,totalSample,immune-Th2; Homo_723,totalSample,immune-CD4_T; Homo_723,totalSample,immune-Tfh; Homo_723,totalSample,immune-nTreg; Homo_723,totalSample,immune-CD4_naive; Homo_723,totalSample,immune-iTreg; Homo_723,totalSample,immune-Tr1; Homo_723,totalSample,immune-Effector_memory; Homo_723,totalSample,immune-Central_memory; Homo_714,totalSample,immune-Tfh; Homo_714,totalSample,immune-Th2; Homo_714,totalSample,immune-CD4_T; Homo_714,totalSample,immune-nTreg; Homo_714,totalSample,immune-CD4_naive; Homo_714,totalSample,immune-Effector_memory; Homo_633,totalSample,immune-Tfh; Homo_633,totalSample,immune-CD4_T; Homo_633,totalSample,immune-nTreg; Homo_633,totalSample,immune-Effector_memory; Homo_633,totalSample,immune-InfiltrationScore; Homo_633,totalSample,immune-CD4_naive; Homo_633,totalSample,immune-Central_memory; Homo_633,totalSample,immune-Tr1; Homo_633,totalSample,immune-Th2; MCSA,totalSample,immune-Monocyte; MCSA,totalSample,immune-CD4_T; MCSA,totalSample,immune-Gamma_delta; MCSA,totalSample,immune-CD4_naive; MCSA,totalSample,immune-Tr1; MCSA,totalSample,immune-nTreg; MCSA,totalSample,immune-iTreg; MCSA,totalSample,immune-Th17; MCSA,totalSample,immune-Tfh; MCSA,totalSample,immune-Central_memory; MCSA,totalSample,immune-[illegible]
FZD3	Homo_723,female,AD.vs.control; Homo_723,female,AD.vs.MCI; Homo_723,female,MCI.vs.control; Homo_714,female,AD.vs.control; Homo_714,female,AD.vs.MCI; Homo_633,female,AD.vs.control; Homo_633,female,AD.vs.MCI; Emory,control,age; MCSA,control,age; MCSA,MCI,age; ROSMAP,MCI,last.visit.age; Emory,totalSample,immune-Macrophage; Emory,totalSample,immune-Neutrophil; Emory,totalSample,immune-CD4_T; Emory,totalSample,immune-CD8_T; Emory,totalSample,immune-Tr1; Emory,totalSample,immune-iTreg; Emory,totalSample,immune-Tfh; Emory,totalSample,immune-CD8_naive; Emory,totalSample,immune-Exhausted; Emory,totalSample,immune-InfiltrationScore; Homo_723,totalSample,immune-Tfh; Homo_723,totalSample,immune-CD4_T; Homo_723,totalSample,immune-CD4_naive; Homo_723,totalSample,immune-iTreg; Homo_723,totalSample,immune-Tr1; Homo_723,totalSample,immune-Th2; Homo_723,totalSample,immune-Central_memory; Homo_723,totalSample,immune-nTreg; Homo_723,totalSample,immune-Macrophage; Homo_723,totalSample,immune-Effector_memory; Homo_723,totalSample,immune-InfiltrationScore; Homo_723,totalSample,immune-MAIT; Homo_723,totalSample,immune-Th1; Homo_723,totalSample,immune-Neutrophil; Homo_714,totalSample,immune-Tfh; Homo_714,totalSample,immune-CD4_T; Homo_714,totalSample,immune-CD4_naive; Homo_714,totalSample,immune-Tr1; Homo_714,totalSample,immune-iTreg; Homo_714,totalSample,immune-Central_memory; Homo_714,totalSample,immune-Effector_memory; Homo_714,totalSample,immune-Th2; Homo_714,totalSample,immune-nTreg; Homo_714,totalSample,immune-InfiltrationScore; Homo_714,totalSample,immune-Macrophage; Homo_714,totalSample,immune-MAIT; Homo_714,totalSample,immune-NKT; Homo_714,totalSample,immune-Neutrophil; Homo_714,totalSample,immune-Th1; Homo_633,totalSample,immune-Tfh; Homo_633,totalSample,immune-CD4_T; Homo_633,totalSample,immune-CD4_naive; Homo_633,totalSample,immune-Central_memory; Homo_633,totalSample,immune-Tr1; Homo_633,totalSample,immune-Effector_memory; Homo_633,totalSample,immune-iTreg; Homo_633,totalSample,immune-NKT; Homo_633,totalSample,immune-InfiltrationScore; Homo_633,totalSample,immune-nTreg; Homo_633,totalSample,immune-Th2; Homo_633,totalSample,immune-MAIT; Homo_633,totalSample,immune-Neutrophil; Homo_633,totalSample,immune-Macrophage; Homo_633,totalSample,immune-DC; Homo_633,totalSample,immune-Th1; MCSA,totalSample,immune-DC; MCSA,totalSample,immune-Macrophage; MCSA,totalSample,immune-Neutrophil; MCSA,totalSample,immune-CD4_T; MCSA,totalSample,immune-CD4_naive; MCSA,totalSample,immune-Tr1; MCSA,totalSample,immune-nTreg; MCSA,totalSample,immune-iTreg; MCSA,totalSample,immune-Th17; MCSA,totalSample,immune-Tfh; MCSA,totalSample,immune-CD8_naive; MCSA,totalSample,immune-Cytotoxic; MCSA,totalSample,immune-Exhausted; MCSA,totalSample,immune-MAIT; MCSA,totalSample,immune-Central_memory; MCSA,totalSample,immune-Effector_memory; ROSMAP,male-excludeAge90+,survival
RYR3	Homo_723,female,AD.vs.control; Homo_723,male,AD.vs.control; Homo_723,female,AD.vs.MCI; Homo_723,male,AD.vs.MCI; Homo_714,female,AD.vs.control; Homo_714,male,AD.vs.control; Homo_714,female,AD.vs.MCI; Homo_714,male,AD.vs.MCI; Homo_633,female,AD.vs.control; Homo_633,female,AD.vs.MCI; MCSA,MCI,age; Emory,totalSample,immune-Gamma_delta; Emory,totalSample,immune-Th17; Emory,totalSample,immune-Cytotoxic; Homo_723,totalSample,immune-Th17; Homo_723,totalSample,immune-Cytotoxic; Homo_723,totalSample,immune-CD4_naive; Homo_723,totalSample,immune-Exhausted; Homo_723,totalSample,immune-CD8_T; Homo_714,totalSample,immune-Exhausted; Homo_714,totalSample,immune-Th17; Homo_714,totalSample,immune-NKT; Homo_714,totalSample,immune-CD8_T; Homo_633,totalSample,immune-NKT; Homo_633,totalSample,immune-Tfh; Homo_633,totalSample,immune-InfiltrationScore; Homo_633,totalSample,immune-CD4_T; Homo_633,totalSample,immune-Tr1; Homo_633,totalSample,immune-Gamma_delta; MCSA,totalSample,immune-DC; MCSA,totalSample,immune-CD4_T; MCSA,totalSample,immune-CD4_naive; MCSA,totalSample,immune-Tr1; MCSA,totalSample,immune-nTreg; MCSA,totalSample,immune-iTreg; MCSA,totalSample,immune-Th2; MCSA,totalSample,immune-Tfh; MCSA,totalSample,immune-Cytotoxic; MCSA,totalSample,immune-Exhausted;
SESTD1	Homo_723,female,AD.vs.control; Homo_723,male,AD.vs.control; Homo_723,female,AD.vs.MCI; Homo_723,male,AD.vs.MCI; Homo_714,female,AD.vs.control; Homo_714,male,AD.vs.control; Homo_714,female,AD.vs.MCI; Homo_714,male,AD.vs.MCI; Homo_633,female,AD.vs.control; Homo_633,female,AD.vs.MCI; ROSMAP,male,AD.vs.control; SRP223445,female,AD.vs.control; MCSA,MCI,age; Emory,totalSample,immune-NKT; Emory,totalSample,immune-Gamma_delta; Emory,totalSample,immune-InfiltrationScore; Homo_723,totalSample,immune-Exhausted; Homo_723,totalSample,immune-Th17; Homo_723,totalSample,immune-Cytotoxic; Homo_723,totalSample,immune-CD8_T; Homo_723,totalSample,immune-Th1; Homo_723,totalSample,immune-Gamma_delta; Homo_723,totalSample,immune-CD4_naive; Homo_723,totalSample,immune-Monocyte; Homo_723,totalSample,immune-CD4_T; Homo_714,totalSample,immune-Exhausted; Homo_714,totalSample,immune-Th17; Homo_714,totalSample,immune-Monocyte; Homo_714,totalSample,immune-CD8_T; Homo_714,totalSample,immune-Cytotoxic; Homo_714,totalSample,immune-Gamma_delta; Homo_714,totalSample,immune-Th1; Homo_714,totalSample,immune-NKT; Homo_633,totalSample,immune-Th2; Homo_633,totalSample,immune-Gamma_delta; Homo_633,totalSample,immune-Monocyte; Homo_633,totalSample,immune-Tfh; Homo_633,totalSample,immune-Exhausted; Homo_633,totalSample,immune-CD4_T; Homo_633,totalSample,immune-Th17; Homo_633,totalSample,immune-NKT; Homo_633,totalSample,immune-Cytotoxic; Homo_633,totalSample,immune-Tr1; Homo_633,totalSample,immune-InfiltrationScore; Homo_633,totalSample,immune-CD4_naive; Homo_633,totalSample,immune-CD8_T; MCSA,totalSample,immune-DC; MCSA,totalSample,immune-Monocyte; MCSA,totalSample,immune-CD4_T; MCSA,totalSample,immune-CD8_T; MCSA,totalSample,immune-Gamma_delta; MCSA,totalSample,immune-CD4_naive; MCSA,totalSample,immune-Tr1; MCSA,totalSample,immune-nTreg; MCSA,totalSample,immune-[illegible]
HAUS4	Homo_723,female,AD.vs.control; Homo_723,male,AD.vs.control; Homo_723,female,AD.vs.MCI; Homo_723,male,AD.vs.MCI; Homo_714,female,AD.vs.control; Homo_714,male,AD.vs.control; Homo_714,female,AD.vs.MCI; Homo_714,male,AD.vs.MCI; Homo_633,female,AD.vs.control; Homo_633,female,AD.vs.MCI; Homo_633,female,MCI.vs.control; ROSMAP,female,AD.vs.control; ROSMAP,MCI,last.visit.age; ROSMAP,MCI,sample.age; Emory,totalSample,immune-Monocyte; Emory,totalSample,immune-Neutrophil; Emory,totalSample,immune-CD8_T; Emory,totalSample,immune-Gamma_delta; Emory,totalSample,immune-Th1; Emory,totalSample,immune-Tfh; Emory,totalSample,immune-CD8_naive; Emory,totalSample,immune-Cytotoxic; Emory,totalSample,immune-Exhausted; Homo_723,totalSample,immune-Monocyte; Homo_723,totalSample,immune-Neutrophil; Homo_723,totalSample,immune-DC; Homo_723,totalSample,immune-Th2; Homo_723,totalSample,immune-Gamma_delta; Homo_723,totalSample,immune-Exhausted; Homo_723,totalSample,immune-Bcell; Homo_723,totalSample,immune-Th1; Homo_714,totalSample,immune-Monocyte; Homo_714,totalSample,immune-Th2; Homo_714,totalSample,immune-Gamma_delta; Homo_714,totalSample,immune-Th1; Homo_714,totalSample,immune-Tfh; Homo_714,totalSample,immune-Neutrophil; Homo_633,totalSample,immune-Monocyte; Homo_633,totalSample,immune-Th2; Homo_633,totalSample,immune-Exhausted; Homo_633,totalSample,immune-Th17; Homo_633,totalSample,immune-Gamma_delta; MCSA,totalSample,immune-Neutrophil; MCSA,totalSample,immune-CD8_T; MCSA,totalSample,immune-Gamma_delta; MCSA,totalSample,immune-Th17; MCSA,totalSample,immune-Cytotoxic; MCSA,totalSample,immune-Exhausted; MCSA,totalSample,immune-MAIT; MCSA,totalSample,immune-Effector_memory; ROSMAP,totalSample,immune-iTreg; ROSMAP,totalSample,immune-Th2; ROSMAP,totalSample,immune-Th17; ROSMAP,totalSample,immune-Cytotoxic; ROSMAP,totalSample,immune-Effector_memory; ROSMAP,totalSample,immune-InfiltrationScore; SRP223445,totalSample,immune-[illegible]
NEK1	Homo_723,female,AD.vs.control; Homo_723,male,AD.vs.control; Homo_723,female,AD.vs.MCI; Homo_723,male,AD.vs.MCI; Homo_714,female,AD.vs.control; Homo_714,male,AD.vs.control; Homo_714,female,AD.vs.MCI; Homo_714,male,AD.vs.MCI; Homo_633,female,AD.vs.control; Homo_633,female,AD.vs.MCI; MCSA,MCI,age; ROSMAP,AD,last.visit.age; ROSMAP,MCI,sample.age; Emory,totalSample,immune-Macrophage; Emory,totalSample,immune-Neutrophil; Emory,totalSample,immune-CD4_T; Emory,totalSample,immune-CD8_T; Emory,totalSample,immune-Tr1; Emory,totalSample,immune-iTreg; Emory,totalSample,immune-Tfh; Emory,totalSample,immune-Central_memory; Emory,totalSample,immune-Effector_memory; Emory,totalSample,immune-InfiltrationScore; Homo_723,totalSample,immune-Th1; Homo_723,totalSample,immune-Central_memory; Homo_723,totalSample,immune-CD4_naive; Homo_723,totalSample,immune-Neutrophil; Homo_723,totalSample,immune-CD4_T; Homo_723,totalSample,immune-Cytotoxic; Homo_723,totalSample,immune-Tr1; Homo_723,totalSample,immune-NKT; Homo_714,totalSample,immune-Th1; Homo_714,totalSample,immune-NKT; Homo_714,totalSample,immune-CD4_naive; Homo_714,totalSample,immune-InfiltrationScore; Homo_714,totalSample,immune-Central_memory; Homo_714,totalSample,immune-Neutrophil; Homo_714,totalSample,immune-NK; Homo_633,totalSample,immune-NKT; Homo_633,totalSample,immune-Tfh; Homo_633,totalSample,immune-Th1; Homo_633,totalSample,immune-CD4_naive; Homo_633,totalSample,immune-Tr1; Homo_633,totalSample,immune-CD4_T; Homo_633,totalSample,immune-iTreg; Homo_633,totalSample,immune-InfiltrationScore; Homo_633,totalSample,immune-Central_memory; Homo_633,totalSample,immune-MAIT; Homo_633,totalSample,immune-Effector_memory; MCSA,totalSample,immune-DC; MCSA,totalSample,immune-Bcell; MCSA,totalSample,immune-Monocyte; MCSA,totalSample,immune-Macrophage; MCSA,totalSample,immune-CD4_T; MCSA,totalSample,immune-CD4_naive; MCSA,totalSample,immune-Tr1; MCSA,totalSample,immune-nTreg; MCSA,totalSample,immune-iTreg; MCSA,totalSample,immune-Th2; MCSA,totalSample,immune-Tfh; MCSA,totalSample,immune-CD8_naive; MCSA,totalSample,immune-Cytotoxic; MCSA,totalSample,immune-Exhausted; MCSA,totalSample,immune-MAIT; MCSA,totalSample,immune-Central_memory; ROSMAP,totalSample,immune-[illegible]
CA14	Homo_723,female,AD.vs.control; Homo_723,male,AD.vs.control; Homo_723,female,AD.vs.MCI; Homo_723,male,AD.vs.MCI; Homo_714,female,AD.vs.control; Homo_714,male,AD.vs.control; Homo_714,female,AD.vs.MCI; Homo_714,male,AD.vs.MCI; Homo_633,female,AD.vs.control; Homo_633,female,AD.vs.MCI; ROSMAP,female,AD.vs.MCI; Homo_723,totalSample,immune-Th2; Homo_723,totalSample,immune-Tfh; Homo_723,totalSample,immune-CD4_T; Homo_723,totalSample,immune-iTreg; Homo_723,totalSample,immune-Monocyte; Homo_723,totalSample,immune-DC; Homo_714,totalSample,immune-Th2; Homo_714,totalSample,immune-Tfh; Homo_714,totalSample,immune-CD4_T; Homo_714,totalSample,immune-Gamma_delta; Homo_633,totalSample,immune-Th2; Homo_633,totalSample,immune-Th17; Homo_633,totalSample,immune-Monocyte; Homo_633,totalSample,immune-Tfh; Homo_633,totalSample,immune-Gamma_delta; Homo_633,totalSample,immune-CD4_T; Homo_633,totalSample,immune-Exhausted; MCSA,totalSample,immune-CD4_T; MCSA,totalSample,immune-CD8_T; MCSA,totalSample,immune-NKT; MCSA,totalSample,immune-Gamma_delta; MCSA,totalSample,immune-Tr1; MCSA,totalSample,immune-nTreg; MCSA,totalSample,immune-iTreg; MCSA,totalSample,immune-Th17; MCSA,totalSample,immune-Cytotoxic
HMCES	Homo_723,female,AD.vs.control; Homo_723,male,AD.vs.control; Homo_723,female,AD.vs.MCI; Homo_723,male,AD.vs.MCI; Homo_723,female,MCI.vs.control; Homo_723,control,male.vs.female; Homo_723,MCI,male.vs.female; Homo_714,female,AD.vs.control; Homo_714,male,AD.vs.control; Homo_714,male,AD.vs.MCI; Homo_714,female,MCI.vs.control; Homo_714,male,MCI.vs.control; Homo_714,control,male.vs.female; Homo_714,MCI,male.vs.female; Homo_633,female,AD.vs.control; Homo_633,female,AD.vs.MCI; Homo_633,female,MCI.vs.control; Homo_633,male,MCI.vs.control; Homo_633,control,male.vs.female; Homo_633,MCI,male.vs.female; ROSMAP,female,AD.vs.control; ROSMAP,male,AD.vs.control; ROSMAP,female,MCI.vs.control; MCSA,totalSample,age; MCSA,control,age; ROSMAP,MCI,last.visit.age; Emory,totalSample,immune-Monocyte; Emory,totalSample,immune-Macrophage; Emory,totalSample,immune-Neutrophil; Emory,totalSample,immune-CD4_T; Emory,totalSample,immune-CD8_T; Emory,totalSample,immune-Tr1; Emory,totalSample,immune-nTreg; Emory,totalSample,immune-iTreg; Emory,totalSample,immune-Tfh; Emory,totalSample,immune-Exhausted; Emory,totalSample,immune-MAIT; Emory,totalSample,immune-Central_memory; Emory,totalSample,immune-InfiltrationScore; Homo_723,totalSample,immune-Monocyte; Homo_723,totalSample,immune-CD4_naive; Homo_723,totalSample,immune-nTreg; Homo_723,totalSample,immune-Central_memory; Homo_723,totalSample,immune-CD4_T; Homo_723,totalSample,immune-Tfh; Homo_723,totalSample,immune-iTreg; Homo_723,totalSample,immune-DC; Homo_723,totalSample,immune-Tr1; Homo_723,totalSample,immune-InfiltrationScore; Homo_723,totalSample,immune-Th1; Homo_723,totalSample,immune-Neutrophil; Homo_723,totalSample,immune-Exhausted; Homo_714,totalSample,immune-Monocyte; Homo_714,totalSample,immune-CD4_naive; Homo_714,totalSample,immune-nTreg; Homo_714,totalSample,immune-CD4_T; Homo_714,totalSample,immune-iTreg; Homo_714,totalSample,immune-Th1; Homo_714,totalSample,immune-Tfh; Homo_714,totalSample,immune-Central_memory; Homo_714,totalSample,immune-InfiltrationScore; Homo_714,totalSample,immune-Neutrophil; Homo_633,totalSample,immune-Monocyte; Homo_633,totalSample,immune-nTreg; Homo_633,totalSample,immune-CD4_naive; Homo_633,totalSample,immune-Tfh; Homo_633,totalSample,immune-CD4_T; Homo_633,totalSample,immune-iTreg; Homo_633,totalSample,immune-Th1; Homo_633,totalSample,immune-Central_memory; Homo_633,totalSample,immune-InfiltrationScore; Homo_633,totalSample,immune-Tr1; Homo_633,totalSample,immune-Macrophage; MCSA,totalSample,immune-Monocyte; MCSA,totalSample,immune-Macrophage; MCSA,totalSample,immune-Neutrophil; MCSA,totalSample,immune-CD4_T; MCSA,totalSample,immune-Gamma_delta; MCSA,totalSample,immune-CD4_naive; MCSA,totalSample,immune-Tr1; MCSA,totalSample,immune-nTreg; MCSA,totalSample,immune-iTreg; MCSA,totalSample,immune-Th17; MCSA,totalSample,immune-Tfh; MCSA,totalSample,immune-CD8_naive; MCSA,totalSample,immune-Central_memory; [illegible]
PLEKHG5	Homo_723,female,AD.vs.control; Homo_723,male,AD.vs.control; Homo_723,female,AD.vs.MCI; Homo_723,male,AD.vs.MCI; Homo_714,female,AD.vs.control; Homo_714,male,AD.vs.control; Homo_714,female,AD.vs.MCI; Homo_714,male,AD.vs.MCI; Homo_633,female,AD.vs.control; Homo_633,female,AD.vs.MCI; Homo_633,male,MCI.vs.control; ROSMAP,female,AD.vs.control; ROSMAP,male,AD.vs.control; ROSMAP,female,AD.vs.MCI; MCSA,totalSample,age; MCSA,control,age; Emory,totalSample,immune-CD4_T; Emory,totalSample,immune-Gamma_delta; Emory,totalSample,immune-CD4_naive; Emory,totalSample,immune-Tr1; Emory,totalSample,immune-iTreg; Emory,totalSample,immune-Th17; Emory,totalSample,immune-Tfh; Emory,totalSample,immune-Cytotoxic; Emory,totalSample,immune-Central_memory; Homo_723,totalSample,immune-DC; Homo_723,totalSample,immune-Central_memory; Homo_723,totalSample,immune-Monocyte; Homo_723,totalSample,immune-nTreg; Homo_723,totalSample,immune-CD4_naive; Homo_723,totalSample,immune-Neutrophil; Homo_723,totalSample,immune-CD4_T; Homo_714,totalSample,immune-nTreg; Homo_714,totalSample,immune-Monocyte; Homo_714,totalSample,immune-CD4_naive; Homo_714,totalSample,immune-InfiltrationScore; Homo_714,totalSample,immune-Central_memory; Homo_714,totalSample,immune-DC; Homo_633,totalSample,immune-nTreg; Homo_633,totalSample,immune-Monocyte; Homo_633,totalSample,immune-Central_memory; Homo_633,totalSample,immune-iTreg; Homo_633,totalSample,immune-Tfh; Homo_633,totalSample,immune-CD4_naive; Homo_633,totalSample,immune-Tr1; Homo_633,totalSample,immune-InfiltrationScore; Homo_633,totalSample,immune-CD4_T; Homo_633,totalSample,immune-Effector_memory; MCSA,totalSample,immune-DC; MCSA,totalSample,immune-Monocyte; MCSA,totalSample,immune-Neutrophil; MCSA,totalSample,immune-CD4_T; MCSA,totalSample,immune-CD8_T; MCSA,totalSample,immune-NKT; MCSA,totalSample,immune-CD4_naive; MCSA,totalSample,immune-Tr1; MCSA,totalSample,immune-nTreg; MCSA,totalSample,immune-Th2; MCSA,totalSample,immune-Th17; MCSA,totalSample,immune-Tfh; MCSA,totalSample,immune-CD8_naive; MCSA,totalSample,immune-Cytotoxic; MCSA,totalSample,immune-Central_memory; MCSA,totalSample,immune-Effector_memory; MCSA,totalSample,immune-InfiltrationScore
CTDSPL	Homo_723,female,AD.vs.control; Homo_723,male,AD.vs.control; Homo_723,female,AD.vs.MCI; Homo_723,male,AD.vs.MCI; Homo_723,female,MCI.vs.control; Homo_723,male,MCI.vs.control; Homo_714,female,AD.vs.control; Homo_714,male,AD.vs.control; Homo_714,female,AD.vs.MCI; Homo_714,male,AD.vs.MCI; Homo_714,female,MCI.vs.control; Homo_714,male,MCI.vs.control; Homo_633,female,AD.vs.control; Homo_633,female,AD.vs.MCI; ROSMAP,MCI,male.vs.female; ROSMAP,MCI,last.visit.age; ROSMAP,MCI,sample.age; Emory,totalSample,immune-Th17; Homo_723,totalSample,immune-DC; Homo_723,totalSample,immune-Th17; Homo_723,totalSample,immune-MAIT; Homo_723,totalSample,immune-CD8_T; Homo_723,totalSample,immune-Exhausted; Homo_714,totalSample,immune-DC; Homo_714,totalSample,immune-Th17; Homo_714,totalSample,immune-Exhausted; Homo_714,totalSample,immune-CD8_T; Homo_633,totalSample,immune-DC; Homo_633,totalSample,immune-Gamma_delta; Homo_633,totalSample,immune-Th17; MCSA,totalSample,immune-Monocyte; MCSA,totalSample,immune-Th17; SRP223445,totalSample,immune-CD4_naive; SRP223445,totalSample,immune-Exhausted; ROSMAP,female-excludeAge90+,survival; ROSMAP,female-includeAge90+,survival
AGBL5	Homo_723,female,AD.vs.control; Homo_723,male,AD.vs.control; Homo_723,female,AD.vs.MCI; Homo_723,male,AD.vs.MCI; Homo_714,female,AD.vs.control; Homo_714,male,AD.vs.control; Homo_714,female,AD.vs.MCI; Homo_714,male,AD.vs.MCI; Homo_633,female,AD.vs.control; Homo_633,female,AD.vs.MCI; Homo_633,female,MCI.vs.control; Homo_633,male,MCI.vs.control; ROSMAP,female,AD.vs.control; ACOM,totalSample,age; MCSA,totalSample,age; MCSA,control,age; Emory,totalSample,immune-Neutrophil; Emory,totalSample,immune-CD4_T; Emory,totalSample,immune-Tr1; Emory,totalSample,immune-nTreg; Emory,totalSample,immune-iTreg; Emory,totalSample,immune-InfiltrationScore; Homo_723,totalSample,immune-DC; Homo_723,totalSample,immune-Monocyte; Homo_723,totalSample,immune-Central_memory; Homo_723,totalSample,immune-Neutrophil; Homo_723,totalSample,immune-nTreg; Homo_723,totalSample,immune-CD4_naive; Homo_723,totalSample,immune-Th1; Homo_714,totalSample,immune-Monocyte; Homo_714,totalSample,immune-Th1; Homo_714,totalSample,immune-NKT; Homo_714,totalSample,immune-CD4_naive; Homo_714,totalSample,immune-InfiltrationScore; Homo_714,totalSample,immune-DC; Homo_633,totalSample,immune-Monocyte; Homo_633,totalSample,immune-InfiltrationScore; Homo_633,totalSample,immune-nTreg; Homo_633,totalSample,immune-iTreg; Homo_633,totalSample,immune-CD4_naive; Homo_633,totalSample,immune-Tfh; Homo_633,totalSample,immune-NKT; MCSA,totalSample,immune-Monocyte; MCSA,totalSample,immune-Neutrophil; MCSA,totalSample,immune-CD4_T; MCSA,totalSample,immune-CD8_T; MCSA,totalSample,immune-NKT; MCSA,totalSample,immune-CD4_naive; MCSA,totalSample,immune-Tr1; MCSA,totalSample,immune-nTreg; MCSA,totalSample,immune-iTreg; MCSA,totalSample,immune-Th2; MCSA,totalSample,immune-Th17; MCSA,totalSample,immune-Tfh; MCSA,totalSample,immune-CD8_naive; MCSA,totalSample,immune-MAIT; MCSA,totalSample,immune-Central_memory;
INAVA	Homo_723,female,AD.vs.control; Homo_723,female,AD.vs.MCI; ROSMAP,female,AD.vs.control; ACOM,totalSample,age; Emory,totalSample,immune-Macrophage; Emory,totalSample,immune-CD8_T; MCSA,totalSample,immune-Macrophage
CNTNAP1	Homo_723,female,AD.vs.control; Homo_723,male,AD.vs.control; Homo_723,female,AD.vs.MCI; Homo_723,male,AD.vs.MCI; Homo_723,male,MCI.vs.control; Homo_714,female,AD.vs.control; Homo_714,female,MCI.vs.control; Homo_714,male,MCI.vs.control; Homo_633,female,AD.vs.control; Homo_633,female,AD.vs.MCI; Homo_633,female,MCI.vs.control; Homo_633,male,MCI.vs.control; MCSA,totalSample,age; MCSA,control,age; MCSA,MCI,age; ROSMAP,MCI,MMSE; Emory,totalSample,immune-CD4_T; Emory,totalSample,immune-Gamma_delta; Emory,totalSample,immune-Tr1; Emory,totalSample,immune-nTreg; Emory,totalSample,immune-iTreg; Emory,totalSample,immune-Th17; Emory,totalSample,immune-Tfh; Emory,totalSample,immune-Cytotoxic; Emory,totalSample,immune-Central_memory; Emory,totalSample,immune-InfiltrationScore; Homo_723,totalSample,immune-Monocyte; Homo_723,totalSample,immune-nTreg; Homo_723,totalSample,immune-DC; Homo_723,totalSample,immune-Central_memory; Homo_723,totalSample,immune-CD8_T; Homo_723,totalSample,immune-Exhausted; Homo_714,totalSample,immune-Monocyte; Homo_714,totalSample,immune-nTreg; Homo_633,totalSample,immune-Monocyte; Homo_633,totalSample,immune-nTreg; Homo_633,totalSample,immune-DC; Homo_633,totalSample,immune-InfiltrationScore; MCSA,totalSample,immune-DC; MCSA,totalSample,immune-Monocyte; MCSA,totalSample,immune-CD4_T; MCSA,totalSample,immune-CD8_T; MCSA,totalSample,immune-CD4_naive; MCSA,totalSample,immune-nTreg; MCSA,totalSample,immune-Th17; MCSA,totalSample,immune-Tfh; MCSA,totalSample,immune-Cytotoxic; MCSA,totalSample,immune-Central_memory; MCSA,totalSample,immune-Effector_memory;
CEMIP	Homo_723,female,AD.vs.control; Homo_723,male,AD.vs.control; Homo_723,female,AD.vs.MCI; Homo_723,male,AD.vs.MCI; Homo_714,female,AD.vs.control; Homo_714,male,AD.vs.control; Homo_714,female,AD.vs.MCI; Homo_714,male,AD.vs.MCI; Homo_633,female,AD.vs.control; Homo_633,female,AD.vs.MCI; Homo_633,female,MCI.vs.control; Homo_633,male,MCI.vs.control; ACOM,totalSample,age; ROSMAP,AD,last.visit.age; ROSMAP,MCI,last.visit.age; Emory,totalSample,immune-CD4_T; Emory,totalSample,immune-Tr1; Homo_723,totalSample,immune-Monocyte; Homo_723,totalSample,immune-DC; Homo_723,totalSample,immune-Central_memory; Homo_723,totalSample,immune-nTreg; Homo_723,totalSample,immune-CD4_naive; Homo_723,totalSample,immune-Th1; Homo_723,totalSample,immune-Neutrophil; Homo_714,totalSample,immune-Th1; Homo_714,totalSample,immune-CD4_naive; Homo_714,totalSample,immune-Monocyte; Homo_633,totalSample,immune-nTreg; Homo_633,totalSample,immune-Monocyte; Homo_633,totalSample,immune-Tfh; Homo_633,totalSample,immune-CD4_naive; Homo_633,totalSample,immune-CD4_T; Homo_633,totalSample,immune-InfiltrationScore; Homo_633,totalSample,immune-Tr1; MCSA,totalSample,immune-Monocyte; MCSA,totalSample,immune-Neutrophil; MCSA,totalSample,immune-CD4_T; MCSA,totalSample,immune-CD8_T; MCSA,totalSample,immune-CD4_naive; MCSA,totalSample,immune-Tr1; MCSA,totalSample,immune-nTreg; MCSA,totalSample,immune-iTreg; MCSA,totalSample,immune-Th17; MCSA,totalSample,immune-Tfh; MCSA,totalSample,immune-Central_memory; MCSA,totalSample,immune-Effector_memory; MCSA,totalSample,immune-InfiltrationScore; ROSMAP,totalSample,immune-Effector_memory;

LIN54	Homo_723,female,AD.vs.control; Homo_723,male,AD.vs.control; Homo_723,female,AD.vs.MCI; Homo_723,male,AD.vs.MCI; Homo_723,female,MCI.vs.control; Homo_723,male,MCI.vs.control; Homo_714,female,AD.vs.control; Homo_714,male,AD.vs.control; Homo_714,female,AD.vs.MCI; Homo_714,male,AD.vs.MCI; Homo_714,female,MCI.vs.control; Homo_714,male,MCI.vs.control; Homo_633,female,AD.vs.control; Homo_633,female,AD.vs.MCI; MCSA,MCI,age; ROSMAP,MCI,last.visit.age; ROSMAP,MCI,sample.age; Emory,totalSample,immune-CD4_T; Emory,totalSample,immune-Gamma_delta; Emory,totalSample,immune-Tr1; Emory,totalSample,immune-iTreg; Emory,totalSample,immune-Th1; Emory,totalSample,immune-Central_memory; Emory,totalSample,immune-InfiltrationScore; Homo_723,totalSample,immune-Exhausted; Homo_723,totalSample,immune-Th17; Homo_723,totalSample,immune-Cytotoxic; Homo_723,totalSample,immune-CD4_naive; Homo_723,totalSample,immune-CD8_T; Homo_723,totalSample,immune-CD4_T; Homo_723,totalSample,immune-Tfh; Homo_723,totalSample,immune-Tr1; Homo_723,totalSample,immune-Central_memory; Homo_723,totalSample,immune-Th2; Homo_723,totalSample,immune-InfiltrationScore; Homo_723,totalSample,immune-Monocyte; Homo_723,totalSample,immune-Gamma_delta; Homo_714,totalSample,immune-CD4_naive; Homo_714,totalSample,immune-Th17; Homo_714,totalSample,immune-Exhausted; Homo_714,totalSample,immune-Central_memory; Homo_714,totalSample,immune-CD4_T; Homo_714,totalSample,immune-Monocyte; Homo_714,totalSample,immune-Tfh; Homo_714,totalSample,immune-NKT; Homo_714,totalSample,immune-InfiltrationScore; Homo_714,totalSample,immune-Gamma_delta; Homo_714,totalSample,immune-Cytotoxic; Homo_633,totalSample,immune-Tfh; Homo_633,totalSample,immune-Th2; Homo_633,totalSample,immune-CD4_T; Homo_633,totalSample,immune-CD4_naive; Homo_633,totalSample,immune-Central_memory; Homo_633,totalSample,immune-Effector_memory; Homo_633,totalSample,immune-InfiltrationScore; Homo_633,totalSample,immune-Tr1; Homo_633,totalSample,immune-Exhausted; Homo_633,totalSample,immune-Gamma_delta; Homo_633,totalSample,immune-NKT; Homo_633,totalSample,immune-Monocyte; Homo_633,totalSample,immune-Th17; Homo_633,totalSample,immune-iTreg; Homo_633,totalSample,immune-DC; Homo_633,totalSample,immune-Bcell; MCSA,totalSample,immune-DC; MCSA,totalSample,immune-Monocyte; MCSA,totalSample,immune-Macrophage; MCSA,totalSample,immune-CD4_T; MCSA,totalSample,immune-CD8_T; MCSA,totalSample,immune-CD4_naive; MCSA,totalSample,immune-Tr1; MCSA,totalSample,immune-nTreg; MCSA,totalSample,immune-iTreg; MCSA,totalSample,immune-Th17; MCSA,totalSample,immune-Tfh; MCSA,totalSample,immune-CD8_naive; MCSA,totalSample,immune-Cytotoxic; MCSA,totalSample,immune-Exhausted; MCSA,totalSample,immune-MAIT; MCSA,totalSample,immune-Central_memory; SRP223445,totalSample,immune-Exhausted; SRP223445,totalSample,immune-InfiltrationScore
WNT11	Homo_723,female,AD.vs.control; Homo_723,male,AD.vs.control; Homo_723,female,AD.vs.MCI; Homo_723,male,AD.vs.MCI; Homo_714,female,AD.vs.control; Homo_714,male,AD.vs.control; Homo_714,female,AD.vs.MCI; Homo_714,male,AD.vs.MCI; Homo_633,female,AD.vs.control; Homo_633,female,AD.vs.MCI; ROSMAP,control,male.vs.female; Emory,control,age; MCSA,totalSample,age; MCSA,MCI,age; ROSMAP,AD,last.visit.age; ROSMAP,control,last.visit.age; Emory,totalSample,immune-B_cell; Emory,totalSample,immune-Neutrophil; Emory,totalSample,immune-CD8_T; Emory,totalSample,immune-Th1; Emory,totalSample,immune-Exhausted; Homo_723,totalSample,immune-DC; Homo_714,totalSample,immune-DC; Homo_714,totalSample,immune-Th2; Homo_633,totalSample,immune-DC; Homo_633,totalSample,immune-InfiltrationScore; MCSA,totalSample,immune-DC; MCSA,totalSample,immune-Monocyte; MCSA,totalSample,immune-CD8_T; MCSA,totalSample,immune-NKT; MCSA,totalSample,immune-Th2; MCSA,totalSample,immune-Cytotoxic; MCSA,totalSample,immune-Effector_memory; MCSA,totalSample,immune-InfiltrationScore; SRP223445,totalSample,immune-CD4_naive
ARMCX1	Homo_723,female,AD.vs.control; Homo_723,male,AD.vs.control; Homo_723,female,AD.vs.MCI; Homo_723,male,AD.vs.MCI; Homo_723,female,MCI.vs.control; Homo_723,male,MCI.vs.control; Homo_714,female,AD.vs.control; Homo_714,male,AD.vs.control; Homo_714,female,AD.vs.MCI; Homo_714,male,AD.vs.MCI; Homo_714,female,MCI.vs.control; Homo_633,female,AD.vs.control; Homo_633,female,AD.vs.MCI; ROSMAP,female,AD.vs.control; MCSA,MCI,age; ROSMAP,totalSample,age; ROSMAP,control,sample.age; Emory,totalSample,immune-Macrophage; Emory,totalSample,immune-CD4_T; Emory,totalSample,immune-Tr1; Emory,totalSample,immune-iTreg; Emory,totalSample,immune-InfiltrationScore; Homo_723,totalSample,immune-Exhausted; Homo_723,totalSample,immune-CD4_naive; Homo_723,totalSample,immune-InfiltrationScore; Homo_723,totalSample,immune-Th17; Homo_723,totalSample,immune-Cytotoxic; Homo_723,totalSample,immune-Monocyte; Homo_723,totalSample,immune-Tfh; Homo_723,totalSample,immune-CD4_T; Homo_723,totalSample,immune-Effector_memory; Homo_723,totalSample,immune-Tr1; Homo_723,totalSample,immune-CD8_T; Homo_723,totalSample,immune-Th2; Homo_723,totalSample,immune-Central_memory; Homo_723,totalSample,immune-nTreg; Homo_714,totalSample,immune-Monocyte; Homo_714,totalSample,immune-Exhausted; Homo_714,totalSample,immune-InfiltrationScore; Homo_714,totalSample,immune-CD4_naive; Homo_714,totalSample,immune-Tfh; Homo_714,totalSample,immune-Effector_memory; Homo_714,totalSample,immune-Central_memory; Homo_714,totalSample,immune-Th17; Homo_714,totalSample,immune-NK; Homo_633,totalSample,immune-Tfh; Homo_633,totalSample,immune-Effector_memory; Homo_633,totalSample,immune-Monocyte; Homo_633,totalSample,immune-Th2; Homo_633,totalSample,immune-Exhausted; Homo_633,totalSample,immune-Central_memory; Homo_633,totalSample,immune-CD4_T; Homo_633,totalSample,immune-InfiltrationScore; Homo_633,totalSample,immune-CD4_naive; Homo_633,totalSample,immune-Gamma_delta; Homo_633,totalSample,immune-Tr1; MCSA,totalSample,immune-Monocyte; MCSA,totalSample,immune-CD4_T; MCSA,totalSample,immune-NKT; MCSA,totalSample,immune-CD4_naive; MCSA,totalSample,immune-Tr1; MCSA,totalSample,immune-nTreg; MCSA,totalSample,immune-iTreg; MCSA,totalSample,immune-Tfh; MCSA,totalSample,immune-CD8_naive; MCSA,totalSample,immune-Cytotoxic; MCSA,totalSample,immune-Exhausted; MCSA,totalSample,immune-MAIT; MCSA,totalSample,immune-Central_memory; ROSMAP,totalSample,immune-Monocyte; ROSMAP,totalSample,immune-CD4_T; ROSMAP,totalSample,immune-Th17; ROSMAP,totalSample,immune-InfiltrationScore; [illegible]
TMEM64	Homo_723,female,AD.vs.control; Homo_723,male,AD.vs.control; Homo_723,female,AD.vs.MCI; Homo_723,male,AD.vs.MCI; Homo_714,female,AD.vs.control; Homo_714,male,AD.vs.control; Homo_714,female,AD.vs.MCI; Homo_714,male,AD.vs.MCI; Homo_633,female,AD.vs.control; Homo_633,female,AD.vs.MCI; ROSMAP,female,MCI.vs.control; ROSMAP,male,MCI.vs.control; ROSMAP,AD,male.vs.female; MCSA,MCI,age; Emory,totalSample,immune-DC; Emory,totalSample,immune-Monocyte; Emory,totalSample,immune-Macrophage; Emory,totalSample,immune-Neutrophil; Emory,totalSample,immune-CD4_T; Emory,totalSample,immune-CD8_T; Emory,totalSample,immune-Tr1; Emory,totalSample,immune-iTreg; Emory,totalSample,immune-Effector_memory; Emory,totalSample,immune-InfiltrationScore; Homo_723,totalSample,immune-CD4_naive; Homo_723,totalSample,immune-CD4_T; Homo_723,totalSample,immune-Th17; Homo_723,totalSample,immune-Cytotoxic; Homo_723,totalSample,immune-Central_memory; Homo_723,totalSample,immune-Tr1; Homo_723,totalSample,immune-Tfh; Homo_723,totalSample,immune-Th1; Homo_723,totalSample,immune-NKT; Homo_723,totalSample,immune-Exhausted; Homo_723,totalSample,immune-Neutrophil; Homo_723,totalSample,immune-InfiltrationScore; Homo_723,totalSample,immune-nTreg; Homo_723,totalSample,immune-iTreg; Homo_723,totalSample,immune-Th2; Homo_723,totalSample,immune-CD8_T; Homo_714,totalSample,immune-CD4_naive; Homo_714,totalSample,immune-NKT; Homo_714,totalSample,immune-CD4_T; Homo_714,totalSample,immune-Tfh; Homo_714,totalSample,immune-Th1; Homo_714,totalSample,immune-Central_memory; Homo_714,totalSample,immune-InfiltrationScore; Homo_714,totalSample,immune-Th17; Homo_714,totalSample,immune-Tr1; Homo_714,totalSample,immune-Effector_memory; Homo_714,totalSample,immune-iTreg; Homo_714,totalSample,immune-Neutrophil; Homo_714,totalSample,immune-Exhausted; Homo_633,totalSample,immune-Tfh; Homo_633,totalSample,immune-NKT; Homo_633,totalSample,immune-CD4_T; Homo_633,totalSample,immune-CD4_naive; Homo_633,totalSample,immune-Effector_memory; Homo_633,totalSample,immune-Central_memory; Homo_633,totalSample,immune-InfiltrationScore; Homo_633,totalSample,immune-Tr1; Homo_633,totalSample,immune-iTreg; Homo_633,totalSample,immune-Th2; Homo_633,totalSample,immune-MAIT; Homo_633,totalSample,immune-Th1; Homo_633,totalSample,immune-DC; Homo_633,totalSample,immune-nTreg; Homo_633,totalSample,immune-Gamma_delta; Homo_633,totalSample,immune-Neutrophil; MCSA,totalSample,immune-DC; MCSA,totalSample,immune-Bcell; MCSA,totalSample,immune-Monocyte; MCSA,totalSample,immune-Macrophage; MCSA,totalSample,immune-CD4_T; MCSA,totalSample,immune-CD8_T; MCSA,totalSample,immune-CD4_naive; MCSA,totalSample,immune-Tr1; MCSA,totalSample,immune-nTreg; MCSA,totalSample,immune-iTreg; MCSA,totalSample,immune-Th17; MCSA,totalSample,immune-Tfh; MCSA,totalSample,immune-CD8_naive; MCSA,totalSample,immune-Cytotoxic; [illegible]
ISCA2	Homo_723,female,AD.vs.control; Homo_723,male,AD.vs.control; Homo_723,female,AD.vs.MCI; Homo_723,male,AD.vs.MCI; Homo_714,female,AD.vs.control; Homo_714,male,AD.vs.control; Homo_714,female,AD.vs.MCI; Homo_714,male,AD.vs.MCI; Homo_633,female,AD.vs.control; Homo_633,female,AD.vs.MCI; ROSMAP,female,AD.vs.control; MCSA,MCI,age; ROSMAP,MCI,last.visit.age; ROSMAP,MCI,sample.age; Emory,totalSample,immune-Macrophage; Emory,totalSample,immune-Neutrophil; Emory,totalSample,immune-CD4_T; Emory,totalSample,immune-CD8_T; Emory,totalSample,immune-Tr1; Emory,totalSample,immune-iTreg; Emory,totalSample,immune-Th1; Emory,totalSample,immune-Tfh; Emory,totalSample,immune-Central_memory; Emory,totalSample,immune-InfiltrationScore; Homo_723,totalSample,immune-iTreg; Homo_723,totalSample,immune-Tfh; Homo_723,totalSample,immune-Th2; Homo_723,totalSample,immune-Tr1; Homo_723,totalSample,immune-CD4_T; Homo_723,totalSample,immune-InfiltrationScore; Homo_723,totalSample,immune-CD4_naive; Homo_723,totalSample,immune-Central_memory; Homo_723,totalSample,immune-CD8_T; Homo_723,totalSample,immune-DC; Homo_723,totalSample,immune-MAIT; Homo_723,totalSample,immune-Macrophage; Homo_723,totalSample,immune-nTreg; Homo_714,totalSample,immune-Tfh; Homo_714,totalSample,immune-Th2; Homo_714,totalSample,immune-Tr1; Homo_714,totalSample,immune-CD4_T; Homo_714,totalSample,immune-iTreg; Homo_714,totalSample,immune-Effector_memory; Homo_714,totalSample,immune-InfiltrationScore; Homo_714,totalSample,immune-CD4_naive; Homo_714,totalSample,immune-Central_memory; Homo_633,totalSample,immune-Tfh; Homo_633,totalSample,immune-Th1; Homo_633,totalSample,immune-CD4_T; Homo_633,totalSample,immune-Tr1; Homo_633,totalSample,immune-InfiltrationScore; Homo_633,totalSample,immune-CD4_naive; Homo_633,totalSample,immune-NKT; Homo_633,totalSample,immune-iTreg; Homo_633,totalSample,immune-Central_memory; Homo_633,totalSample,immune-nTreg; Homo_633,totalSample,immune-Effector_memory; MCSA,totalSample,immune-DC; MCSA,totalSample,immune-Monocyte; MCSA,totalSample,immune-Macrophage; MCSA,totalSample,immune-CD4_T; MCSA,totalSample,immune-CD8_T; MCSA,totalSample,immune-NKT; MCSA,totalSample,immune-CD4_naive; MCSA,totalSample,immune-Tr1; MCSA,totalSample,immune-nTreg; MCSA,totalSample,immune-iTreg; MCSA,totalSample,immune-Th2; MCSA,totalSample,immune-Tfh; MCSA,totalSample,immune-Cytotoxic; ROSMAP,totalSample,immune-Th17; ROSMAP,totalSample,immune-Effector_memory; SRP223445,totalSample,immune-InfiltrationScore
DPY19L2	Homo_723,female,AD.vs.control; Homo_723,male,AD.vs.control; Homo_723,female,AD.vs.MCI; Homo_723,male,AD.vs.MCI; Homo_723,female,MCI.vs.control; Homo_723,male,MCI.vs.control; Homo_714,female,AD.vs.control; Homo_714,male,AD.vs.control; Homo_714,female,AD.vs.MCI; Homo_714,male,AD.vs.MCI; Homo_714,female,MCI.vs.control; Homo_714,male,MCI.vs.control; Homo_633,female,AD.vs.control; Homo_633,female,AD.vs.MCI; Homo_723,totalSample,immune-Th17; Homo_723,totalSample,immune-Exhausted; Homo_723,totalSample,immune-CD4_naive; Homo_723,totalSample,immune-CD4_T; Homo_723,totalSample,immune-Tr1; Homo_723,totalSample,immune-Cytotoxic; Homo_723,totalSample,immune-Tfh; Homo_723,totalSample,immune-Central_memory; Homo_723,totalSample,immune-Th2; Homo_714,totalSample,immune-Th17; Homo_714,totalSample,immune-CD4_naive; Homo_714,totalSample,immune-CD4_T; Homo_714,totalSample,immune-Central_memory; Homo_714,totalSample,immune-Exhausted; Homo_714,totalSample,immune-Gamma_delta; Homo_714,totalSample,immune-Monocyte; Homo_633,totalSample,immune-Tfh; Homo_633,totalSample,immune-CD4_T; Homo_633,totalSample,immune-Effector_memory; Homo_633,totalSample,immune-CD4_naive; Homo_633,totalSample,immune-Th2; Homo_633,totalSample,immune-Central_memory; Homo_633,totalSample,immune-Gamma_delta; Homo_633,totalSample,immune-InfiltrationScore; Homo_633,totalSample,immune-Th17; Homo_633,totalSample,immune-Tr1; Homo_633,totalSample,immune-Monocyte; Homo_633,totalSample,immune-Exhausted; Homo_633,totalSample,immune-iTreg; Homo_633,totalSample,immune-NKT; MCSA,totalSample,immune-DC; MCSA,totalSample,immune-Macrophage; MCSA,totalSample,immune-CD4_T; MCSA,totalSample,immune-CD4_naive; MCSA,totalSample,immune-Tr1; MCSA,totalSample,immune-nTreg; MCSA,totalSample,immune-iTreg; MCSA,totalSample,immune-Th17; MCSA,totalSample,immune-Tfh; MCSA,totalSample,immune-CD8_naive; MCSA,totalSample,immune-Cytotoxic; MCSA,totalSample,immune-Exhausted; MCSA,totalSample,immune-MAIT; MCSA,totalSample,immune-Central_memory; ROSMAP,totalSample,immune-InfiltrationScore; SRP223445,totalSample,immune-CD4_naive; SRP223445,totalSample,immune-Exhausted; SRP223445,totalSample,immune-InfiltrationScore; ROSMAP,female-excludeAge90+,survival; ROSMAP,male [illegible]
TMEM17	Homo_723,female,AD.vs.control; Homo_723,male,AD.vs.control; Homo_723,female,AD.vs.MCI; Homo_723,male,AD.vs.MCI; Homo_723,male,MCI.vs.control; Homo_714,female,AD.vs.control; Homo_714,male,AD.vs.control; Homo_714,female,AD.vs.MCI; Homo_714,male,AD.vs.MCI; Homo_714,male,MCI.vs.control; Homo_633,female,AD.vs.control; Homo_633,female,AD.vs.MCI; MCSA,MCI,age; Homo_714,totalSample,immune-NK; Homo_633,totalSample,immune-InfiltrationScore; MCSA,totalSample,immune-CD4_T; MCSA,totalSample,immune-Tr1; MCSA,totalSample,immune-iTreg; ROSMAP,totalSample,immune-InfiltrationScore; SRP223445,totalSample,immune-CD4_naive
TWSG1	Homo_723,female,AD.vs.control; Homo_723,male,AD.vs.control; Homo_723,female,AD.vs.MCI; Homo_723,male,AD.vs.MCI; Homo_723,female,MCI.vs.control; Homo_723,male,MCI.vs.control; Homo_714,female,AD.vs.control; Homo_714,male,AD.vs.control; Homo_714,female,AD.vs.MCI; Homo_714,male,AD.vs.MCI; Homo_714,female,MCI.vs.control; Homo_714,male,MCI.vs.control; Homo_633,female,AD.vs.control; Homo_633,female,AD.vs.MCI; MCSA,MCI,age; Emory,totalSample,immune-DC; Emory,totalSample,immune-Monocyte; Emory,totalSample,immune-CD4_T; Emory,totalSample,immune-CD8_T; Emory,totalSample,immune-Tr1; Emory,totalSample,immune-iTreg; Emory,totalSample,immune-Central_memory; Emory,totalSample,immune-Effector_memory; Homo_723,totalSample,immune-Exhausted; Homo_723,totalSample,immune-CD4_naive; Homo_723,totalSample,immune-Cytotoxic; Homo_723,totalSample,immune-Th17; Homo_723,totalSample,immune-CD4_T; Homo_723,totalSample,immune-Tfh; Homo_723,totalSample,immune-Central_memory; Homo_723,totalSample,immune-Tr1; Homo_723,totalSample,immune-CD8_T; Homo_723,totalSample,immune-InfiltrationScore; Homo_723,totalSample,immune-Th1; Homo_723,totalSample,immune-NKT; Homo_723,totalSample,immune-Th2; Homo_723,totalSample,immune-nTreg; Homo_714,totalSample,immune-CD4_naive; Homo_714,totalSample,immune-Central_memory; Homo_714,totalSample,immune-NKT; Homo_714,totalSample,immune-Tfh; Homo_714,totalSample,immune-CD4_T; Homo_714,totalSample,immune-Exhausted; Homo_714,totalSample,immune-Th17; Homo_714,totalSample,immune-InfiltrationScore; Homo_714,totalSample,immune-Monocyte; Homo_633,totalSample,immune-Tfh; Homo_633,totalSample,immune-CD4_T; Homo_633,totalSample,immune-CD4_naive; Homo_633,totalSample,immune-Effector_memory; Homo_633,totalSample,immune-Central_memory; Homo_633,totalSample,immune-InfiltrationScore; Homo_633,totalSample,immune-NKT; Homo_633,totalSample,immune-Th2; Homo_633,totalSample,immune-Tr1; Homo_633,totalSample,immune-Monocyte; Homo_633,totalSample,immune-DC; Homo_633,totalSample,immune-iTreg; Homo_633,totalSample,immune-Exhausted; Homo_633,totalSample,immune-Gamma_delta; Homo_633,totalSample,immune-Bcell; MCSA,totalSample,immune-DC; MCSA,totalSample,immune-Monocyte; MCSA,totalSample,immune-Macrophage; MCSA,totalSample,immune-CD4_T; MCSA,totalSample,immune-CD8_T; MCSA,totalSample,immune-CD4_naive; MCSA,totalSample,immune-Tr1; MCSA,totalSample,immune-nTreg; MCSA,totalSample,immune-iTreg; MCSA,totalSample,immune-Th17; MCSA,totalSample,immune-Tfh; MCSA,totalSample,immune-CD8_naive; MCSA,totalSample,immune-Cytotoxic; [illegible]
TRADD	Homo_723,female,AD.vs.control; Homo_723,male,AD.vs.control; Homo_723,female,AD.vs.MCI; Homo_723,male,AD.vs.MCI; Homo_714,female,AD.vs.control; Homo_714,male,AD.vs.control; Homo_714,female,AD.vs.MCI; Homo_714,male,AD.vs.MCI; Homo_633,female,AD.vs.control; Homo_633,female,AD.vs.MCI; ROSMAP,female,AD.vs.control; ROSMAP,male,AD.vs.control; MCSA,totalSample,age; MCSA,control,age; MCSA,MCI,age; Emory,totalSample,immune-Macrophage; Emory,totalSample,immune-Neutrophil; Emory,totalSample,immune-CD4_T; Emory,totalSample,immune-Tr1; Emory,totalSample,immune-nTreg; Emory,totalSample,immune-iTreg; Emory,totalSample,immune-Th17; Emory,totalSample,immune-Tfh; Emory,totalSample,immune-InfiltrationScore; Homo_723,totalSample,immune-CD8_T; Homo_723,totalSample,immune-InfiltrationScore; Homo_723,totalSample,immune-Effector_memory; Homo_723,totalSample,immune-Tfh; Homo_723,totalSample,immune-nTreg; Homo_723,totalSample,immune-iTreg; Homo_723,totalSample,immune-Cytotoxic; Homo_723,totalSample,immune-Th2; Homo_723,totalSample,immune-Central_memory; Homo_723,totalSample,immune-Macrophage; Homo_723,totalSample,immune-CD4_T; Homo_723,totalSample,immune-MAIT; Homo_723,totalSample,immune-CD4_naive; Homo_723,totalSample,immune-Bcell; Homo_714,totalSample,immune-nTreg; Homo_714,totalSample,immune-Effector_memory; Homo_714,totalSample,immune-Tfh; Homo_714,totalSample,immune-InfiltrationScore; Homo_714,totalSample,immune-CD8_T; Homo_714,totalSample,immune-Th2; Homo_714,totalSample,immune-Central_memory; Homo_714,totalSample,immune-Macrophage; Homo_714,totalSample,immune-CD4_T; Homo_714,totalSample,immune-DC; Homo_714,totalSample,immune-Tr1; Homo_714,totalSample,immune-iTreg; Homo_633,totalSample,immune-nTreg; Homo_633,totalSample,immune-InfiltrationScore; Homo_633,totalSample,immune-DC; Homo_633,totalSample,immune-Effector_memory; Homo_633,totalSample,immune-Central_memory; Homo_633,totalSample,immune-Tfh; Homo_633,totalSample,immune-CD4_T; Homo_633,totalSample,immune-Tr1; Homo_633,totalSample,immune-CD8_T; Homo_633,totalSample,immune-Neutrophil; MCSA,totalSample,immune-DC; MCSA,totalSample,immune-Monocyte; MCSA,totalSample,immune-Neutrophil; MCSA,totalSample,immune-CD8_T; MCSA,totalSample,immune-NKT; MCSA,totalSample,immune-CD4_naive; MCSA,totalSample,immune-nTreg; MCSA,totalSample,immune-Th2; MCSA,totalSample,immune-Tfh; MCSA,totalSample,immune-Cytotoxic; MCSA,totalSample,immune-Central_memory; MCSA,totalSample,immune-Effector_memory; MCSA,totalSample,immune-InfiltrationScore; ROSMAP,totalSample,immune-CD4_T; ROSMAP,totalSample,immune-iTreg; ROSMAP,totalSample,immune-Th2; ROSMAP,totalSample,immune-Th17; ROSMAP,totalSample,immune-Cytotoxic; ROSMAP,totalSample,immune-MAIT; ROSMAP,totalSample,immune-Effector_memory; ROSMAP,totalSample,immune-InfiltrationScore; SRP223445,totalSample,immune- [illegible]
HJURP	Homo_723,female,AD.vs.control; Homo_723,male,AD.vs.control; Homo_723,female,AD.vs.MCI; Homo_723,male,AD.vs.MCI; Homo_723,female,MCI.vs.control; Homo_723,male,MCI.vs.control; Homo_714,female,AD.vs.control; Homo_714,male,AD.vs.control; Homo_714,female,AD.vs.MCI; Homo_714,male,AD.vs.MCI; Homo_714,female,MCI.vs.control; Homo_633,female,AD.vs.control; Homo_633,female,AD.vs.MCI; Homo_723,totalSample,immune-Exhausted; Homo_723,totalSample,immune-Th17; Homo_723,totalSample,immune-CD8_T; Homo_723,totalSample,immune-InfiltrationScore; Homo_723,totalSample,immune-Neutrophil; Homo_714,totalSample,immune-InfiltrationScore; Homo_714,totalSample,immune-Exhausted; Homo_633,totalSample,immune-InfiltrationScore; MCSA,totalSample,immune-Macrophage; MCSA,totalSample,immune-NKT
TNFRSF21	Homo_723,female,AD.vs.control; Homo_723,male,AD.vs.control; Homo_723,female,AD.vs.MCI; Homo_723,male,AD.vs.MCI; Homo_723,female,MCI.vs.control; Homo_714,female,AD.vs.control; Homo_714,male,AD.vs.control; Homo_714,female,AD.vs.MCI; Homo_714,male,AD.vs.MCI; Homo_714,female,MCI.vs.control; Homo_633,female,AD.vs.control; Homo_633,female,AD.vs.MCI; ROSMAP,AD,last.visit.age; Emory,totalSample,immune-CD4_naive; Emory,totalSample,immune-Th17; Homo_723,totalSample,immune-DC; Homo_723,totalSample,immune-Th17; Homo_723,totalSample,immune-NK; Homo_723,totalSample,immune-Cytotoxic; Homo_714,totalSample,immune-NK; Homo_714,totalSample,immune-InfiltrationScore; Homo_714,totalSample,immune-CD8_T; Homo_714,totalSample,immune-Th17; Homo_714,totalSample,immune-Exhausted; Homo_633,totalSample,immune-InfiltrationScore; Homo_633,totalSample,immune-NK; Homo_633,totalSample,immune-Tr1; MCSA,totalSample,immune-CD4_T; MCSA,totalSample,immune-Tr1; MCSA,totalSample,immune-iTreg; MCSA,totalSample,immune-Th17; ROSMAP,totalSample,immune-Effector_memory; SRP223445,totalSample,immune-nTreg
ZC3H12B	Homo_723,female,AD.vs.control; Homo_723,male,AD.vs.control; Homo_723,female,AD.vs.MCI; Homo_723,male,AD.vs.MCI; Homo_714,female,AD.vs.control; Homo_714,male,AD.vs.control; Homo_714,female,AD.vs.MCI; Homo_714,male,AD.vs.MCI; Homo_633,female,AD.vs.control; Homo_633,female,AD.vs.MCI; ROSMAP,female,AD.vs.control; ROSMAP,control,male.vs.female; MCSA,MCI,age; ROSMAP,MCI,last.visit.age; ROSMAP,MCI,sample.age; Emory,totalSample,immune-Monocyte; Emory,totalSample,immune-Macrophage; Emory,totalSample,immune-Neutrophil; Emory,totalSample,immune-CD4_T; Emory,totalSample,immune-CD8_T; Emory,totalSample,immune-CD4_naive; Emory,totalSample,immune-Tr1; Emory,totalSample,immune-nTreg; Emory,totalSample,immune-iTreg; Emory,totalSample,immune-Tfh; Emory,totalSample,immune-Central_memory; Emory,totalSample,immune-InfiltrationScore; Homo_723,totalSample,immune-Central_memory; Homo_723,totalSample,immune-CD4_naive; Homo_723,totalSample,immune-CD4_T; Homo_723,totalSample,immune-Tr1; Homo_723,totalSample,immune-Th1; Homo_723,totalSample,immune-nTreg; Homo_723,totalSample,immune-iTreg; Homo_723,totalSample,immune-Tfh; Homo_723,totalSample,immune-Monocyte; Homo_723,totalSample,immune-Neutrophil; Homo_723,totalSample,immune-Cytotoxic; Homo_723,totalSample,immune-NKT; Homo_723,totalSample,immune-Th2; Homo_714,totalSample,immune-CD4_naive; Homo_714,totalSample,immune-Central_memory; Homo_714,totalSample,immune-Th1; Homo_714,totalSample,immune-CD4_T; Homo_714,totalSample,immune-Tr1; Homo_714,totalSample,immune-iTreg; Homo_714,totalSample,immune-Tfh; Homo_714,totalSample,immune-NKT; Homo_714,totalSample,immune-nTreg; Homo_714,totalSample,immune-Cytotoxic; Homo_633,totalSample,immune-Tfh; Homo_633,totalSample,immune-CD4_naive; Homo_633,totalSample,immune-CD4_T; Homo_633,totalSample,immune-Tr1; Homo_633,totalSample,immune-iTreg; Homo_633,totalSample,immune-Central_memory; Homo_633,totalSample,immune-nTreg; Homo_633,totalSample,immune-NKT; Homo_633,totalSample,immune-Th1; Homo_633,totalSample,immune-Effector_memory; Homo_633,totalSample,immune-InfiltrationScore; Homo_633,totalSample,immune-Monocyte; Homo_633,totalSample,immune-Neutrophil; Homo_633,totalSample,immune-Macrophage; MCSA,totalSample,immune-DC; MCSA,totalSample,immune-Bcell; MCSA,totalSample,immune-Macrophage; MCSA,totalSample,immune-Neutrophil; MCSA,totalSample,immune-CD4_T; MCSA,totalSample,immune-CD4_naive; MCSA,totalSample,immune-Tr1; MCSA,totalSample,immune-nTreg; MCSA,totalSample,immune-iTreg; MCSA,totalSample,immune-Th17; MCSA,totalSample,immune-Tfh; MCSA,totalSample,immune-CD8_naive; MCSA,totalSample,immune-Cytotoxic; MCSA,totalSample,immune-Exhausted; MCSA,totalSample,immune-MAIT; MCSA,totalSample,immune-Central_memory; MCSA,totalSample,immune-Effector_memory; ROSMAP,totalSample,immune-InfiltrationScore; SRP223445,totalSample,immune-CD4_naive
FHL1	Homo_723,female,AD.vs.control; Homo_723,male,AD.vs.control; Homo_723,female,AD.vs.MCI; Homo_723,male,AD.vs.MCI; Homo_714,female,AD.vs.control; Homo_714,male,AD.vs.control; Homo_714,female,AD.vs.MCI; Homo_714,male,AD.vs.MCI; Homo_633,female,AD.vs.control; Homo_633,female,AD.vs.MCI; MCSA,totalSample,age; MCSA,control,age; Emory,totalSample,immune-nTreg; Emory,totalSample,immune-Th17; Emory,totalSample,immune-Cytotoxic; Homo_723,totalSample,immune-Th17; Homo_723,totalSample,immune-Central_memory; Homo_723,totalSample,immune-DC; Homo_723,totalSample,immune-CD4_naive; Homo_723,totalSample,immune-nTreg; Homo_723,totalSample,immune-CD4_T; Homo_723,totalSample,immune-Cytotoxic; Homo_723,totalSample,immune-Th1; Homo_714,totalSample,immune-Th17; Homo_714,totalSample,immune-CD4_naive; Homo_714,totalSample,immune-Exhausted; Homo_714,totalSample,immune-Th1; Homo_714,totalSample,immune-NKT; Homo_714,totalSample,immune-Central_memory; Homo_714,totalSample,immune-nTreg; Homo_714,totalSample,immune-DC; Homo_714,totalSample,immune-Cytotoxic; Homo_633,totalSample,immune-Tfh; Homo_633,totalSample,immune-CD4_T; Homo_633,totalSample,immune-iTreg; Homo_633,totalSample,immune-nTreg; Homo_633,totalSample,immune-Tr1; Homo_633,totalSample,immune-Central_memory; Homo_633,totalSample,immune-CD4_naive; Homo_633,totalSample,immune-InfiltrationScore; Homo_633,totalSample,immune-NKT; Homo_633,totalSample,immune-Gamma_delta; Homo_633,totalSample,immune-Th17; MCSA,totalSample,immune-CD4_T; MCSA,totalSample,immune-Gamma_delta; MCSA,totalSample,immune-CD4_naive; MCSA,totalSample,immune-Tr1; MCSA,totalSample,immune-nTreg; MCSA,totalSample,immune-iTreg; MCSA,totalSample,immune-Th1; MCSA,totalSample,immune-Th17; MCSA,totalSample,immune-Tfh; MCSA,totalSample,immune-CD8_naive; MCSA,totalSample,immune-Exhausted; MCSA,totalSample,immune-MAIT; MCSA,totalSample,immune-Central_memory; MCSA,totalSample,immune-Effector_memory;

FAM185A	Homo_723,female,AD.vs.control; Homo_723,male,AD.vs.control; Homo_723,female,AD.vs.MCI; Homo_723,male,AD.vs.MCI; Homo_714,female,AD.vs.control; Homo_714,male,AD.vs.control; Homo_714,female,AD.vs.MCI; Homo_714,male,AD.vs.MCI; Homo_633,female,AD.vs.control; Homo_633,female,AD.vs.MCI; ROSMAP,female,AD.vs.control; ROSMAP,control,male.vs.female; MCSA,MCI,age; ROSMAP,MCI,last.visit.age; ROSMAP,MCI,sample.age; Emory,totalSample,immune-Macrophage; Emory,totalSample,immune-CD4_T; Emory,totalSample,immune-Tr1; Emory,totalSample,immune-iTreg; Emory,totalSample,immune-Th17; Emory,totalSample,immune-InfiltrationScore; Homo_723,totalSample,immune-CD4_naive; Homo_723,totalSample,immune-Central_memory; Homo_723,totalSample,immune-Cytotoxic; Homo_723,totalSample,immune-Th1; Homo_723,totalSample,immune-CD4_T; Homo_723,totalSample,immune-Tr1; Homo_723,totalSample,immune-Th17; Homo_723,totalSample,immune-InfiltrationScore; Homo_723,totalSample,immune-nTreg; Homo_723,totalSample,immune-Tfh; Homo_723,totalSample,immune-NKT; Homo_723,totalSample,immune-Neutrophil; Homo_714,totalSample,immune-CD4_naive; Homo_714,totalSample,immune-NKT; Homo_714,totalSample,immune-Th1; Homo_714,totalSample,immune-InfiltrationScore; Homo_714,totalSample,immune-CD4_T; Homo_714,totalSample,immune-Central_memory; Homo_714,totalSample,immune-Tr1; Homo_714,totalSample,immune-Exhausted; Homo_714,totalSample,immune-Tfh; Homo_714,totalSample,immune-Cytotoxic; Homo_714,totalSample,immune-Th17; Homo_633,totalSample,immune-Tfh; Homo_633,totalSample,immune-NKT; Homo_633,totalSample,immune-Tr1; Homo_633,totalSample,immune-CD4_T; Homo_633,totalSample,immune-CD4_naive; Homo_633,totalSample,immune-InfiltrationScore; Homo_633,totalSample,immune-iTreg; Homo_633,totalSample,immune-Central_memory; Homo_633,totalSample,immune-Effector_memory; Homo_633,totalSample,immune-Th1; Homo_633,totalSample,immune-nTreg; MCSA,totalSample,immune-DC; MCSA,totalSample,immune-Monocyte; MCSA,totalSample,immune-Macrophage; MCSA,totalSample,immune-CD4_T; MCSA,totalSample,immune-CD4_naive; MCSA,totalSample,immune-Tr1; MCSA,totalSample,immune-nTreg; MCSA,totalSample,immune-iTreg; MCSA,totalSample,immune-Th17; MCSA,totalSample,immune-Tfh; MCSA,totalSample,immune-CD8_naive;
FRMD3	Homo_723,female,AD.vs.control; Homo_723,male,AD.vs.control; Homo_723,female,AD.vs.MCI; Homo_723,male,AD.vs.MCI; Homo_723,female,MCI.vs.control; Homo_723,male,MCI.vs.control; Homo_714,female,AD.vs.control; Homo_714,male,AD.vs.control; Homo_714,female,AD.vs.MCI; Homo_714,male,AD.vs.MCI; Homo_714,female,MCI.vs.control; Homo_714,male,MCI.vs.control; Homo_633,female,AD.vs.control; Homo_633,female,AD.vs.MCI; ROSMAP,female,AD.vs.control; ROSMAP,female,MCI.vs.control; MCSA,MCI,age; Emory,totalSample,immune-DC; Emory,totalSample,immune-CD8_T; Emory,totalSample,immune-Tfh; Homo_723,totalSample,immune-CD8_T; Homo_723,totalSample,immune-Cytotoxic; Homo_723,totalSample,immune-DC; Homo_723,totalSample,immune-Th17; Homo_723,totalSample,immune-Th1; Homo_723,totalSample,immune-NK; Homo_714,totalSample,immune-CD8_T; Homo_714,totalSample,immune-Exhausted; Homo_714,totalSample,immune-Cytotoxic; Homo_714,totalSample,immune-NK; Homo_633,totalSample,immune-CD8_T; Homo_633,totalSample,immune-NKT; Homo_633,totalSample,immune-Gamma_delta; MCSA,totalSample,immune-DC; MCSA,totalSample,immune-Monocyte; MCSA,totalSample,immune-Neutrophil; MCSA,totalSample,immune-CD8_T; MCSA,totalSample,immune-iTreg; MCSA,totalSample,immune-Cytotoxic; MCSA,totalSample,immune-Exhausted; MCSA,totalSample,immune-Effector_memory; ROSMAP,totalSample,immune-Th17; ROSMAP,totalSample,immune-Effector_memory; SRP223445,totalSample,immune-Exhausted
TSPAN9	Homo_723,female,AD.vs.control; Homo_723,male,AD.vs.control; Homo_723,female,AD.vs.MCI; Homo_723,male,AD.vs.MCI; Homo_723,female,MCI.vs.control; Homo_723,male,MCI.vs.control; Homo_714,female,AD.vs.control; Homo_714,male,AD.vs.control; Homo_714,female,AD.vs.MCI; Homo_714,male,AD.vs.MCI; Homo_714,female,MCI.vs.control; Homo_714,male,MCI.vs.control; Homo_633,female,AD.vs.control; Homo_633,female,AD.vs.MCI; MCSA,totalSample,age; MCSA,MCI,age; ROSMAP,MCI,last.visit.age; Emory,totalSample,immune-Th17; Emory,totalSample,immune-Cytotoxic; Emory,totalSample,immune-Effector_memory; Homo_723,totalSample,immune-DC; Homo_723,totalSample,immune-Th17; Homo_723,totalSample,immune-Exhausted; Homo_723,totalSample,immune-MAIT; Homo_714,totalSample,immune-DC; Homo_714,totalSample,immune-Th17; Homo_714,totalSample,immune-Exhausted; Homo_633,totalSample,immune-DC; Homo_633,totalSample,immune-InfiltrationScore; MCSA,totalSample,immune-DC; MCSA,totalSample,immune-Monocyte; MCSA,totalSample,immune-CD8_T; MCSA,totalSample,immune-Th2; MCSA,totalSample,immune-Cytotoxic; MCSA,totalSample,immune-Effector_memory; ROSMAP,totalSample,immune-InfiltrationScore; SRP223445,totalSample,immune-CD4_naive; SRP223445,totalSample,immune-Exhausted
NCAPH2	Homo_723,female,AD.vs.control; Homo_723,male,AD.vs.control; Homo_723,female,AD.vs.MCI; Homo_723,male,AD.vs.MCI; Homo_714,female,AD.vs.control; Homo_714,male,AD.vs.control; Homo_714,female,AD.vs.MCI; Homo_714,male,AD.vs.MCI; Homo_633,female,AD.vs.control; Homo_633,female,AD.vs.MCI; ROSMAP,female,AD.vs.control; Emory,MCI,age; MCSA,totalSample,age; MCSA,control,age; MCSA,MCI,age; Emory,totalSample,immune-DC; Emory,totalSample,immune-Tr1; Emory,totalSample,immune-iTreg; Emory,totalSample,immune-InfiltrationScore; Homo_723,totalSample,immune-Cytotoxic; Homo_723,totalSample,immune-CD8_T; Homo_723,totalSample,immune-InfiltrationScore; Homo_723,totalSample,immune-Effector_memory; Homo_723,totalSample,immune-Th1; Homo_723,totalSample,immune-Tfh; Homo_723,totalSample,immune-nTreg; Homo_723,totalSample,immune-Bcell; Homo_714,totalSample,immune-DC; Homo_714,totalSample,immune-Effector_memory; Homo_714,totalSample,immune-CD8_T; Homo_714,totalSample,immune-InfiltrationScore; Homo_714,totalSample,immune-Th1; Homo_714,totalSample,immune-Cytotoxic; Homo_714,totalSample,immune-nTreg; Homo_633,totalSample,immune-DC; Homo_633,totalSample,immune-InfiltrationScore; Homo_633,totalSample,immune-nTreg; Homo_633,totalSample,immune-Effector_memory; Homo_633,totalSample,immune-Th1; Homo_633,totalSample,immune-NKT; Homo_633,totalSample,immune-CD8_T; MCSA,totalSample,immune-DC; MCSA,totalSample,immune-Monocyte; MCSA,totalSample,immune-Macrophage; MCSA,totalSample,immune-Neutrophil; MCSA,totalSample,immune-CD8_T; MCSA,totalSample,immune-NKT; MCSA,totalSample,immune-iTreg; MCSA,totalSample,immune-
GPA33	Homo_723,female,AD.vs.control; Homo_723,male,AD.vs.control; Homo_723,female,AD.vs.MCI; Homo_723,male,AD.vs.MCI; Homo_723,female,MCI.vs.control; Homo_723,male,MCI.vs.control; Homo_714,female,AD.vs.control; Homo_714,male,AD.vs.control; Homo_714,female,AD.vs.MCI; Homo_714,male,AD.vs.MCI; Homo_714,female,MCI.vs.control; Homo_714,male,MCI.vs.control; Homo_633,female,AD.vs.control; Homo_633,female,AD.vs.MCI; Homo_633,male,MCI.vs.control; MCSA,totalSample,age; MCSA,control,age; MCSA,MCI,age; ROSMAP,MCI,last.visit.age; ROSMAP,MCI,sample.age; Emory,totalSample,immune-Macrophage; Emory,totalSample,immune-Neutrophil; Emory,totalSample,immune-CD4_T; Emory,totalSample,immune-CD8_T; Emory,totalSample,immune-CD4_naive; Emory,totalSample,immune-Tr1; Emory,totalSample,immune-nTreg; Emory,totalSample,immune-iTreg; Emory,totalSample,immune-Th17; Emory,totalSample,immune-Tfh; Emory,totalSample,immune-MAIT; Emory,totalSample,immune-Central_memory; Emory,totalSample,immune-InfiltrationScore; Homo_723,totalSample,immune-iTreg; Homo_723,totalSample,immune-CD8_T; Homo_723,totalSample,immune-Tfh; Homo_723,totalSample,immune-Central_memory; Homo_723,totalSample,immune-Exhausted; Homo_723,totalSample,immune-Monocyte; Homo_723,totalSample,immune-nTreg; Homo_723,totalSample,immune-CD4_naive; Homo_723,totalSample,immune-CD4_T; Homo_723,totalSample,immune-Tr1; Homo_723,totalSample,immune-InfiltrationScore; Homo_723,totalSample,immune-Macrophage; Homo_723,totalSample,immune-MAIT; Homo_723,totalSample,immune-Th2; Homo_723,totalSample,immune-CD8_naive; Homo_714,totalSample,immune-Tfh; Homo_714,totalSample,immune-nTreg; Homo_714,totalSample,immune-Monocyte; Homo_714,totalSample,immune-iTreg; Homo_714,totalSample,immune-CD4_T; Homo_714,totalSample,immune-Tr1; Homo_714,totalSample,immune-Central_memory; Homo_714,totalSample,immune-CD4_naive; Homo_714,totalSample,immune-Effector_memory; Homo_714,totalSample,immune-Macrophage; Homo_714,totalSample,immune-InfiltrationScore; Homo_714,totalSample,immune-Th2; Homo_714,totalSample,immune-CD8_T; Homo_633,totalSample,immune-nTreg; Homo_633,totalSample,immune-Monocyte; Homo_633,totalSample,immune-CD4_naive; Homo_633,totalSample,immune-Tfh; Homo_633,totalSample,immune-iTreg; Homo_633,totalSample,immune-Tr1; Homo_633,totalSample,immune-Central_memory; Homo_633,totalSample,immune-CD4_T; Homo_633,totalSample,immune-InfiltrationScore; Homo_633,totalSample,immune-Effector_memory; Homo_633,totalSample,immune-NKT; Homo_633,totalSample,immune-Macrophage; Homo_633,totalSample,immune-Th1; MCSA,totalSample,immune-Monocyte; MCSA,totalSample,immune-Neutrophil; MCSA,totalSample,immune-CD4_T; MCSA,totalSample,immune-CD8_T; MCSA,totalSample,immune-NKT; MCSA,totalSample,immune-Gamma_delta; MCSA,totalSample,immune-CD4_naive; MCSA,totalSample,immune-Tr1; MCSA,totalSample,immune-nTreg; MCSA,totalSample,immune-iTreg; MCSA,totalSample,immune-Th2; MCSA,totalSample,immune-Th17; MCSA,totalSample,immune-Tfh; MCSA,totalSample,immune-CD8_naive; MCSA,totalSample,immune-MAIT; MCSA,totalSample,immune-Central_memory; MCSA,totalSample,immune-Effector_memory; MCSA,totalSample,immune-InfiltrationScore; ROSMAP,totalSample,immune-InfiltrationScore
AASS	Homo_723,female,AD.vs.control; Homo_723,male,AD.vs.control; Homo_723,female,AD.vs.MCI; Homo_723,male,AD.vs.MCI; Homo_723,female,MCI.vs.control; Homo_723,male,MCI.vs.control; Homo_714,female,AD.vs.control; Homo_714,male,AD.vs.control; Homo_714,female,AD.vs.MCI; Homo_714,male,AD.vs.MCI; Homo_714,female,MCI.vs.control; Homo_714,male,MCI.vs.control; Homo_633,female,AD.vs.control; Homo_633,female,AD.vs.MCI; MCSA,MCI,age; ROSMAP,MCI,last.visit.age; Emory,totalSample,immune-CD4_T; Emory,totalSample,immune-Tr1; Emory,totalSample,immune-iTreg; Homo_723,totalSample,immune-CD4_naive; Homo_723,totalSample,immune-CD4_T; Homo_723,totalSample,immune-Tfh; Homo_723,totalSample,immune-Th17; Homo_723,totalSample,immune-Exhausted; Homo_723,totalSample,immune-Th2; Homo_723,totalSample,immune-Tr1; Homo_723,totalSample,immune-Central_memory; Homo_723,totalSample,immune-nTreg; Homo_723,totalSample,immune-Cytotoxic; Homo_723,totalSample,immune-iTreg; Homo_723,totalSample,immune-InfiltrationScore; Homo_723,totalSample,immune-Effector_memory; Homo_723,totalSample,immune-Th1; Homo_714,totalSample,immune-CD4_naive; Homo_714,totalSample,immune-Tfh; Homo_714,totalSample,immune-CD4_T; Homo_714,totalSample,immune-Central_memory; Homo_714,totalSample,immune-InfiltrationScore; Homo_714,totalSample,immune-Th17; Homo_714,totalSample,immune-Effector_memory; Homo_714,totalSample,immune-Th2; Homo_714,totalSample,immune-Tr1; Homo_714,totalSample,immune-iTreg; Homo_714,totalSample,immune-Exhausted; Homo_633,totalSample,immune-Tfh; Homo_633,totalSample,immune-CD4_T; Homo_633,totalSample,immune-CD4_naive; Homo_633,totalSample,immune-Th2; Homo_633,totalSample,immune-Central_memory; Homo_633,totalSample,immune-Effector_memory; Homo_633,totalSample,immune-Tr1; Homo_633,totalSample,immune-InfiltrationScore; Homo_633,totalSample,immune-iTreg; Homo_633,totalSample,immune-Th17; Homo_633,totalSample,immune-Exhausted; Homo_633,totalSample,immune-nTreg; Homo_633,totalSample,immune-Gamma_delta; Homo_633,totalSample,immune-Bcell; Homo_633,totalSample,immune-NKT; MCSA,totalSample,immune-DC; MCSA,totalSample,immune-Macrophage; MCSA,totalSample,immune-CD4_T; MCSA,totalSample,immune-CD4_naive; MCSA,totalSample,immune-Tr1; MCSA,totalSample,immune-nTreg; MCSA,totalSample,immune-iTreg; MCSA,totalSample,immune-Th17; MCSA,totalSample,immune-Tfh; MCSA,totalSample,immune-CD8_naive; MCSA,totalSample,immune-Cytotoxic; MCSA,totalSample,immune-Exhausted; MCSA,totalSample,immune-MAIT; MCSA,totalSample,immune-Central_memory; ROSMAP,totalSample,immune-InfiltrationScore
SEZ6L	Homo_723,female,AD.vs.control; Homo_723,male,AD.vs.control; Homo_723,female,AD.vs.MCI; Homo_723,male,AD.vs.MCI; Homo_714,male,AD.vs.control; Homo_714,female,AD.vs.MCI; Homo_714,male,AD.vs.MCI; Homo_633,female,AD.vs.control; Homo_633,female,AD.vs.MCI; ROSMAP,female,AD.vs.control; ROSMAP,male,AD.vs.control; Emory,MCI,age; Emory,totalSample,immune-DC; Emory,totalSample,immune-InfiltrationScore; Homo_723,totalSample,immune-InfiltrationScore; Homo_714,totalSample,immune-InfiltrationScore; Homo_633,totalSample,immune-InfiltrationScore; MCSA,totalSample,immune-CD4_T; MCSA,totalSample,immune-CD4_naive; MCSA,totalSample,immune-Tr1; MCSA,totalSample,immune-nTreg; MCSA,totalSample,immune-iTreg; MCSA,totalSample,immune-Th17; MCSA,totalSample,immune-Exhausted; SRP223445,totalSample,immune-InfiltrationScore; ROSMAP,male and female-includeAge90+,survival
MMD	Homo_723,female,AD.vs.control; Homo_723,male,AD.vs.control; Homo_723,female,AD.vs.MCI; Homo_723,male,AD.vs.MCI; Homo_723,female,MCI.vs.control; Homo_723,male,MCI.vs.control; Homo_714,female,AD.vs.control; Homo_714,male,AD.vs.control; Homo_714,female,AD.vs.MCI; Homo_714,male,AD.vs.MCI; Homo_714,female,MCI.vs.control; Homo_714,male,MCI.vs.control; Homo_633,female,AD.vs.control; Homo_633,female,AD.vs.MCI; ROSMAP,female,AD.vs.control; Emory,MCI,age; Homo_723,totalSample,immune-Cytotoxic; Homo_723,totalSample,immune-Th17; Homo_723,totalSample,immune-Central_memory; Homo_723,totalSample,immune-DC; Homo_723,totalSample,immune-CD8_T; Homo_723,totalSample,immune-Th1; Homo_723,totalSample,immune-Gamma_delta; Homo_723,totalSample,immune-NKT; Homo_723,totalSample,immune-CD4_naive; Homo_714,totalSample,immune-NKT; Homo_714,totalSample,immune-Exhausted; Homo_714,totalSample,immune-CD8_T; Homo_714,totalSample,immune-Th17; Homo_714,totalSample,immune-Cytotoxic; Homo_633,totalSample,immune-NKT; Homo_633,totalSample,immune-Tfh; Homo_633,totalSample,immune-Gamma_delta; Homo_633,totalSample,immune-iTreg; Homo_633,totalSample,immune-Tr1; Homo_633,totalSample,immune-CD4_T; Homo_633,totalSample,immune-InfiltrationScore; MCSA,totalSample,immune-DC; MCSA,totalSample,immune-Monocyte; MCSA,totalSample,immune-CD4_T; MCSA,totalSample,immune-CD8_T; MCSA,totalSample,immune-CD4_naive; MCSA,totalSample,immune-Tr1; MCSA,totalSample,immune-nTreg; MCSA,totalSample,immune-iTreg; MCSA,totalSample,immune-Th1; MCSA,totalSample,immune-Th17; MCSA,totalSample,immune-Tfh; MCSA,totalSample,immune-CD8_naive; MCSA,totalSample,immune-Cytotoxic; MCSA,totalSample,immune-Exhausted; MCSA,totalSample,immune-Central_memory; SRP223445,totalSample,immune-CD4_naive; SRP223445,totalSample,immune-Exhausted
NAT14	Homo_723,female,AD.vs.control; Homo_723,male,AD.vs.control; Homo_723,female,AD.vs.MCI; Homo_723,male,AD.vs.MCI; Homo_714,male,AD.vs.control; Homo_714,female,AD.vs.MCI; Homo_714,male,AD.vs.MCI; Homo_714,female,MCI.vs.control; Homo_633,female,AD.vs.control; Homo_633,female,AD.vs.MCI; Homo_633,female,MCI.vs.control; ROSMAP,female,AD.vs.control; MCSA,MCI,age; Emory,totalSample,immune-CD4_T; Emory,totalSample,immune-Tr1; Emory,totalSample,immune-iTreg; Emory,totalSample,immune-Tfh; Homo_723,totalSample,immune-DC; Homo_723,totalSample,immune-Monocyte; Homo_723,totalSample,immune-CD8_T; Homo_723,totalSample,immune-Exhausted; Homo_723,totalSample,immune-Cytotoxic; Homo_714,totalSample,immune-DC; Homo_714,totalSample,immune-Monocyte; Homo_633,totalSample,immune-DC; Homo_633,totalSample,immune-Monocyte; Homo_633,totalSample,immune-Th2; MCSA,totalSample,immune-DC; MCSA,totalSample,immune-Monocyte; MCSA,totalSample,immune-Neutrophil; MCSA,totalSample,immune-CD8_T; MCSA,totalSample,immune-iTreg; MCSA,totalSample,immune-Th2; MCSA,totalSample,immune-Cytotoxic; MCSA,totalSample,immune-Effector_memory; MCSA,totalSample,immune-InfiltrationScore; ROSMAP,male and female-includeAge90+,survival
TTI2	Homo_723,female,AD.vs.control; Homo_723,male,AD.vs.control; Homo_723,female,AD.vs.MCI; Homo_723,male,AD.vs.MCI; Homo_714,female,AD.vs.control; Homo_714,male,AD.vs.control; Homo_714,female,AD.vs.MCI; Homo_714,male,AD.vs.MCI; Homo_633,female,AD.vs.control; Homo_633,female,AD.vs.MCI; ROSMAP,female,MCI.vs.control; ROSMAP,control,male.vs.female; Emory,MCI,age; MCSA,control,age; ROSMAP,MCI,last.visit.age; ROSMAP,MCI,sample.age; Emory,totalSample,immune-Gamma_delta; Emory,totalSample,immune-Tr1; Emory,totalSample,immune-iTreg; Emory,totalSample,immune-CD8_naive; Emory,totalSample,immune-InfiltrationScore; Homo_723,totalSample,immune-Th2; Homo_723,totalSample,immune-InfiltrationScore; Homo_723,totalSample,immune-Tfh; Homo_723,totalSample,immune-Effector_memory; Homo_723,totalSample,immune-iTreg; Homo_723,totalSample,immune-CD4_T; Homo_723,totalSample,immune-Bcell; Homo_723,totalSample,immune-Tr1; Homo_723,totalSample,immune-CD4_naive; Homo_723,totalSample,immune-Monocyte; Homo_723,totalSample,immune-Gamma_delta; Homo_714,totalSample,immune-Th2; Homo_714,totalSample,immune-Tfh; Homo_714,totalSample,immune-Effector_memory; Homo_714,totalSample,immune-InfiltrationScore; Homo_714,totalSample,immune-CD4_T; Homo_714,totalSample,immune-Tr1; Homo_714,totalSample,immune-Central_memory; Homo_714,totalSample,immune-nTreg; Homo_633,totalSample,immune-InfiltrationScore; Homo_633,totalSample,immune-Th2; Homo_633,totalSample,immune-Tfh; Homo_633,totalSample,immune-Effector_memory; Homo_633,totalSample,immune-CD4_T; Homo_633,totalSample,immune-Exhausted; Homo_633,totalSample,immune-CD4_naive; Homo_633,totalSample,immune-Central_memory; Homo_633,totalSample,immune-nTreg; Homo_633,totalSample,immune-Th17; Homo_633,totalSample,immune-Tr1; Homo_633,totalSample,immune-Bcell; Homo_633,totalSample,immune-Gamma_delta; MCSA,totalSample,immune-Monocyte; MCSA,totalSample,immune-CD4_T; MCSA,totalSample,immune-Gamma_delta; MCSA,totalSample,immune-CD4_naive; MCSA,totalSample,immune-Tr1; MCSA,totalSample,immune-nTreg; MCSA,totalSample,immune-iTreg; MCSA,totalSample,immune-Th17; MCSA,totalSample,immune-Tfh; MCSA,totalSample,immune-Exhausted; MCSA,totalSample,immune-Central_memory; MCSA,totalSample,immune-Effector_memory;
ADTRP	Homo_723,female,AD.vs.control; Homo_723,male,AD.vs.control; Homo_723,female,AD.vs.MCI; Homo_723,male,AD.vs.MCI; Homo_723,female,MCI.vs.control; Homo_723,male,MCI.vs.control; Homo_714,female,AD.vs.control; Homo_714,male,AD.vs.control; Homo_714,female,AD.vs.MCI; Homo_714,male,AD.vs.MCI; Homo_714,female,MCI.vs.control; Homo_714,male,MCI.vs.control; Homo_633,female,AD.vs.control; Homo_633,female,AD.vs.MCI; Homo_633,female,MCI.vs.control; Homo_633,male,MCI.vs.control; ROSMAP,female,AD.vs.control; MCSA,MCI,age; Emory,totalSample,immune-DC; Emory,totalSample,immune-CD4_T; Emory,totalSample,immune-Gamma_delta; Emory,totalSample,immune-CD4_naive; Emory,totalSample,immune-Tr1; Emory,totalSample,immune-nTreg; Emory,totalSample,immune-iTreg; Emory,totalSample,immune-Tfh; Emory,totalSample,immune-Cytotoxic; Emory,totalSample,immune-Central_memory; Homo_723,totalSample,immune-Th2; Homo_723,totalSample,immune-Tfh; Homo_723,totalSample,immune-iTreg; Homo_723,totalSample,immune-Tr1; Homo_723,totalSample,immune-DC; Homo_723,totalSample,immune-Monocyte; Homo_723,totalSample,immune-CD4_T; Homo_723,totalSample,immune-Gamma_delta; Homo_723,totalSample,immune-CD4_naive; Homo_723,totalSample,immune-Central_memory; Homo_723,totalSample,immune-Effector_memory; Homo_723,totalSample,immune-nTreg; Homo_714,totalSample,immune-Th2; Homo_714,totalSample,immune-Tfh; Homo_714,totalSample,immune-Tr1; Homo_714,totalSample,immune-CD4_T; Homo_714,totalSample,immune-Central_memory; Homo_714,totalSample,immune-iTreg; Homo_714,totalSample,immune-Gamma_delta; Homo_714,totalSample,immune-CD4_naive; Homo_714,totalSample,immune-nTreg; Homo_714,totalSample,immune-Monocyte; Homo_714,totalSample,immune-Effector_memory; Homo_714,totalSample,immune-DC; Homo_633,totalSample,immune-Th2; Homo_633,totalSample,immune-Tfh; Homo_633,totalSample,immune-Central_memory; Homo_633,totalSample,immune-CD4_T; Homo_633,totalSample,immune-Tr1; Homo_633,totalSample,immune-Gamma_delta; Homo_633,totalSample,immune-Monocyte; Homo_633,totalSample,immune-CD4_naive; Homo_633,totalSample,immune-nTreg; Homo_633,totalSample,immune-Th17; Homo_633,totalSample,immune-iTreg; Homo_633,totalSample,immune-Exhausted; Homo_633,totalSample,immune-Effector_memory; MCSA,totalSample,immune-DC; MCSA,totalSample,immune-Bcell; MCSA,totalSample,immune-Monocyte; MCSA,totalSample,immune-Macrophage; MCSA,totalSample,immune-CD4_T; MCSA,totalSample,immune-CD4_naive; MCSA,totalSample,immune-Tr1; MCSA,totalSample,immune-nTreg; MCSA,totalSample,immune-iTreg; MCSA,totalSample,immune-Th2; MCSA,totalSample,immune-Tfh; MCSA,totalSample,immune-CD8_naive; MCSA,totalSample,immune-Cytotoxic; MCSA,totalSample,immune-Exhausted; MCSA,totalSample,immune-Central_memory; SRP223445,totalSample,immune-Th17; SRP223445,totalSample,immune-InfiltrationScore
STON1	Homo_723,female,AD.vs.control; Homo_723,male,AD.vs.control; Homo_723,female,AD.vs.MCI; Homo_723,male,AD.vs.MCI; Homo_723,female,MCI.vs.control; Homo_723,male,MCI.vs.control; Homo_714,female,AD.vs.control; Homo_714,male,AD.vs.control; Homo_714,female,AD.vs.MCI; Homo_714,male,AD.vs.MCI; Homo_714,female,MCI.vs.control; Homo_714,male,MCI.vs.control; Homo_633,female,AD.vs.control; Homo_633,female,AD.vs.MCI; ROSMAP,female,AD.vs.control; ROSMAP,male,AD.vs.control; ROSMAP,male,MCI.vs.control; SRP223445,female,AD.vs.control; ROSMAP,AD,last.visit.age; Homo_723,totalSample,immune-Th17; Homo_723,totalSample,immune-Exhausted; Homo_723,totalSample,immune-Cytotoxic; Homo_723,totalSample,immune-CD8_T; Homo_723,totalSample,immune-CD4_naive; Homo_723,totalSample,immune-CD4_T; Homo_723,totalSample,immune-Tfh; Homo_714,totalSample,immune-Th17; Homo_714,totalSample,immune-Exhausted; Homo_714,totalSample,immune-CD4_naive; Homo_714,totalSample,immune-Monocyte; Homo_714,totalSample,immune-Gamma_delta; Homo_633,totalSample,immune-Th2; Homo_633,totalSample,immune-Tfh; Homo_633,totalSample,immune-CD4_T; Homo_633,totalSample,immune-Th17; Homo_633,totalSample,immune-Gamma_delta; Homo_633,totalSample,immune-InfiltrationScore; Homo_633,totalSample,immune-Central_memory; Homo_633,totalSample,immune-CD4_naive; Homo_633,totalSample,immune-Monocyte; Homo_633,totalSample,immune-Exhausted; Homo_633,totalSample,immune-Tr1; Homo_633,totalSample,immune-Effector_memory; MCSA,totalSample,immune-DC; MCSA,totalSample,immune-CD4_T; MCSA,totalSample,immune-CD8_T; MCSA,totalSample,immune-CD4_naive; MCSA,totalSample,immune-Tr1; MCSA,totalSample,immune-nTreg; MCSA,totalSample,immune-iTreg; MCSA,totalSample,immune-Th17; MCSA,totalSample,immune-Tfh; MCSA,totalSample,immune-CD8_naive; MCSA,totalSample,immune-Cytotoxic; MCSA,totalSample,immune-Exhausted; MCSA,totalSample,immune-Central_memory; SRP223445,totalSample,immune-
AQP9	Homo_723,female,AD.vs.control; Homo_723,male,AD.vs.control; Homo_723,female,AD.vs.MCI; Homo_723,male,AD.vs.MCI; Homo_714,female,AD.vs.control; SRP223445,female,AD.vs.control; SRP223445,male,AD.vs.control; Emory,MCI,age; MCSA,MCI,age; Emory,totalSample,immune-DC; Emory,totalSample,immune-B_cell; Emory,totalSample,immune-Monocyte; Emory,totalSample,immune-NK; Emory,totalSample,immune-Neutrophil; Emory,totalSample,immune-CD4_T; Emory,totalSample,immune-CD8_T; Emory,totalSample,immune-Gamma_delta; Emory,totalSample,immune-Th1; Emory,totalSample,immune-Th17; Emory,totalSample,immune-Tfh; Emory,totalSample,immune-CD8_naive; Emory,totalSample,immune-Cytotoxic; Emory,totalSample,immune-Exhausted; Emory,totalSample,immune-MAIT; Emory,totalSample,immune-Effector_memory; Emory,totalSample,immune-InfiltrationScore; Homo_723,totalSample,immune-Neutrophil; Homo_723,totalSample,immune-Monocyte; Homo_723,totalSample,immune-DC; Homo_723,totalSample,immune-Gamma_delta; Homo_723,totalSample,immune-Exhausted; Homo_723,totalSample,immune-Th2; Homo_723,totalSample,immune-Th1; Homo_723,totalSample,immune-Bcell; Homo_714,totalSample,immune-Neutrophil; Homo_714,totalSample,immune-CD8_T; Homo_714,totalSample,immune-Exhausted; Homo_714,totalSample,immune-DC; Homo_714,totalSample,immune-MAIT; Homo_714,totalSample,immune-Monocyte; Homo_714,totalSample,immune-Cytotoxic; Homo_714,totalSample,immune-Gamma_delta; Homo_714,totalSample,immune-Effector_memory; Homo_714,totalSample,immune-Macrophage; Homo_714,totalSample,immune-Central_memory; Homo_714,totalSample,immune-CD4_naive; Homo_714,totalSample,immune-Th1; Homo_714,totalSample,immune-NK; Homo_714,totalSample,immune-Th17; Homo_633,totalSample,immune-Neutrophil; Homo_633,totalSample,immune-CD8_T; Homo_633,totalSample,immune-Exhausted; Homo_633,totalSample,immune-MAIT; Homo_633,totalSample,immune-DC; Homo_633,totalSample,immune-Cytotoxic; Homo_633,totalSample,immune-Gamma_delta; Homo_633,totalSample,immune-Macrophage; Homo_633,totalSample,immune-Monocyte; Homo_633,totalSample,immune-Th2; Homo_633,totalSample,immune-Effector_memory; Homo_633,totalSample,immune-Th17; Homo_633,totalSample,immune-Central_memory; Homo_633,totalSample,immune-CD4_naive; Homo_633,totalSample,immune-nTreg; MCSA,totalSample,immune-DC; MCSA,totalSample,immune-Monocyte; MCSA,totalSample,immune-Neutrophil; MCSA,totalSample,immune-CD8_T; MCSA,totalSample,immune-NKT; MCSA,totalSample,immune-Gamma_delta; MCSA,totalSample,immune-Th1; MCSA,totalSample,immune-Th17; MCSA,totalSample,immune-Tfh; MCSA,totalSample,immune-Cytotoxic; MCSA,totalSample,immune-Exhausted; MCSA,totalSample,immune-Central_memory; MCSA,totalSample,immune-Effector_memory; MCSA,totalSample,immune-InfiltrationScore; ROSMAP,totalSample,immune-Neutrophil; ROSMAP,totalSample,immune-Effector_memory; SRP223445,totalSample,immune-Monocyte; SRP223445,totalSample,immune-Neutrophil; SRP223445,totalSample,immune-

TXNRD2	Homo_723,female,AD.vs.control; Homo_723,male,AD.vs.control; Homo_723,female,AD.vs.MCI; Homo_723,male,AD.vs.MCI; Homo_714,female,AD.vs.control; Homo_714,male,AD.vs.control; Homo_714,female,AD.vs.MCI; Homo_714,male,AD.vs.MCI; Homo_633,female,AD.vs.control; Homo_633,female,AD.vs.MCI; ROSMAP,male,AD.vs.control; MCSA,totalSample,age; MCSA,MCI,age; ROSMAP,AD,last.visit.age; Emory,totalSample,immune-Neutrophil; Emory,totalSample,immune-Gamma_delta; Emory,totalSample,immune-CD8_naive; Emory,totalSample,immune-Cytotoxic; Homo_723,totalSample,immune-CD8_T; Homo_723,totalSample,immune-Cytotoxic; Homo_723,totalSample,immune-Exhausted; Homo_723,totalSample,immune-DC; Homo_723,totalSample,immune-InfiltrationScore; Homo_723,totalSample,immune-Th17; Homo_723,totalSample,immune-Monocyte; Homo_714,totalSample,immune-DC; Homo_714,totalSample,immune-Monocyte; Homo_714,totalSample,immune-CD8_T; Homo_714,totalSample,immune-InfiltrationScore; Homo_714,totalSample,immune-Cytotoxic; Homo_633,totalSample,immune-DC; Homo_633,totalSample,immune-Th2; Homo_633,totalSample,immune-Monocyte; Homo_633,totalSample,immune-InfiltrationScore; MCSA,totalSample,immune-DC; MCSA,totalSample,immune-Monocyte; MCSA,totalSample,immune-Macrophage; MCSA,totalSample,immune-Neutrophil; MCSA,totalSample,immune-CD8_T; MCSA,totalSample,immune-NKT; MCSA,totalSample,immune-iTreg; MCSA,totalSample,immune-Th2; MCSA,totalSample,immune-Cytotoxic; MCSA,totalSample,immune-Central_memory; MCSA,totalSample,immune-Effector_memory; MCSA,totalSample,immune-InfiltrationScore; ROSMAP,totalSample,immune-Effector_memory;
SLC24A3	Homo_723,female,AD.vs.control; Homo_723,male,AD.vs.control; Homo_723,female,AD.vs.MCI; Homo_723,male,AD.vs.MCI; Homo_723,female,MCI.vs.control; Homo_723,male,MCI.vs.control; Homo_714,female,AD.vs.control; Homo_714,male,AD.vs.control; Homo_714,female,AD.vs.MCI; Homo_714,male,AD.vs.MCI; Homo_714,female,MCI.vs.control; Homo_714,male,MCI.vs.control; Homo_633,female,AD.vs.control; Homo_633,female,AD.vs.MCI; Homo_633,male,MCI.vs.control; ROSMAP,MCI,sample.age; Emory,totalSample,immune-Neutrophil; Emory,totalSample,immune-CD8_T; Emory,totalSample,immune-Gamma_delta; Emory,totalSample,immune-Th1; Emory,totalSample,immune-Th17; Emory,totalSample,immune-Cytotoxic; Emory,totalSample,immune-Exhausted; Emory,totalSample,immune-Effector_memory; Homo_723,totalSample,immune-DC; Homo_723,totalSample,immune-Th17; Homo_723,totalSample,immune-CD8_T; Homo_723,totalSample,immune-MAIT; Homo_723,totalSample,immune-Macrophage; Homo_714,totalSample,immune-CD8_T; Homo_714,totalSample,immune-Exhausted; Homo_714,totalSample,immune-DC; Homo_714,totalSample,immune-MAIT; Homo_633,totalSample,immune-DC; Homo_633,totalSample,immune-MAIT; MCSA,totalSample,immune-Neutrophil; MCSA,totalSample,immune-Gamma_delta; MCSA,totalSample,immune-Th1; ROSMAP,totalSample,immune-InfiltrationScore;
ARHGAP32	Homo_723,female,AD.vs.control; Homo_723,male,AD.vs.control; Homo_723,female,AD.vs.MCI; Homo_723,male,AD.vs.MCI; Homo_723,female,MCI.vs.control; Homo_723,male,MCI.vs.control; Homo_714,female,AD.vs.control; Homo_714,male,AD.vs.control; Homo_714,female,AD.vs.MCI; Homo_714,male,AD.vs.MCI; Homo_714,female,MCI.vs.control; Homo_714,male,MCI.vs.control; Homo_633,female,AD.vs.control; Homo_633,female,AD.vs.MCI; ROSMAP,female,AD.vs.control; MCSA,totalSample,age; MCSA,control,age; Emory,totalSample,immune-Macrophage; Emory,totalSample,immune-CD4_T; Emory,totalSample,immune-Tr1; Emory,totalSample,immune-nTreg; Emory,totalSample,immune-iTreg; Emory,totalSample,immune-Th1; Emory,totalSample,immune-Th17; Emory,totalSample,immune-Cytotoxic; Emory,totalSample,immune-Central_memory; Emory,totalSample,immune-InfiltrationScore; Homo_723,totalSample,immune-CD4_naive; Homo_723,totalSample,immune-Central_memory; Homo_723,totalSample,immune-Th17; Homo_723,totalSample,immune-nTreg; Homo_723,totalSample,immune-CD4_T; Homo_723,totalSample,immune-Cytotoxic; Homo_723,totalSample,immune-DC; Homo_723,totalSample,immune-Tr1; Homo_723,totalSample,immune-Th1; Homo_723,totalSample,immune-Monocyte; Homo_723,totalSample,immune-Tfh; Homo_723,totalSample,immune-InfiltrationScore; Homo_723,totalSample,immune-iTreg; Homo_714,totalSample,immune-CD4_naive; Homo_714,totalSample,immune-NKT; Homo_714,totalSample,immune-Th17; Homo_714,totalSample,immune-CD4_T; Homo_714,totalSample,immune-iTreg; Homo_714,totalSample,immune-Cytotoxic; Homo_714,totalSample,immune-Th1; Homo_714,totalSample,immune-InfiltrationScore; Homo_714,totalSample,immune-Central_memory; Homo_714,totalSample,immune-nTreg; Homo_714,totalSample,immune-Exhausted; Homo_714,totalSample,immune-Tr1; Homo_714,totalSample,immune-Tfh; Homo_633,totalSample,immune-Tfh; Homo_633,totalSample,immune-iTreg; Homo_633,totalSample,immune-CD4_T; Homo_633,totalSample,immune-Tr1; Homo_633,totalSample,immune-CD4_naive; Homo_633,totalSample,immune-NKT; Homo_633,totalSample,immune-nTreg; Homo_633,totalSample,immune-InfiltrationScore; Homo_633,totalSample,immune-Central_memory; Homo_633,totalSample,immune-Effector_memory; Homo_633,totalSample,immune-Gamma_delta; Homo_633,totalSample,immune-Monocyte; Homo_633,totalSample,immune-Cytotoxic; Homo_633,totalSample,immune-Th1; MCSA,totalSample,immune-Monocyte; MCSA,totalSample,immune-CD4_T; MCSA,totalSample,immune-Gamma_delta; MCSA,totalSample,immune-CD4_naive; MCSA,totalSample,immune-Tr1; MCSA,totalSample,immune-nTreg; MCSA,totalSample,immune-iTreg; MCSA,totalSample,immune-Th1; MCSA,totalSample,immune-Th17; MCSA,totalSample,immune-Tfh; MCSA,totalSample,immune-CD8_naive; MCSA,totalSample,immune-Cytotoxic; MCSA,totalSample,immune-Exhausted; MCSA,totalSample,immune-Central_memory; MCSA,totalSample,immune-Effector_memory; MCSA,totalSample,immune-InfiltrationScore; ROSMAP,totalSample,immune- [illegible]
SUCNR1	Homo_723,female,AD.vs.control; Homo_723,male,AD.vs.control; Homo_723,female,AD.vs.MCI; Homo_723,male,AD.vs.MCI; Homo_723,female,MCI.vs.control; Homo_714,female,AD.vs.control; Homo_714,male,AD.vs.control; Homo_714,female,AD.vs.MCI; Homo_714,male,AD.vs.MCI; Homo_714,female,MCI.vs.control; Homo_714,AD,male.vs.female; Homo_633,female,AD.vs.control; Homo_633,female,AD.vs.MCI; ROSMAP,female,AD.vs.control; ROSMAP,female,MCI.vs.control; ROSMAP,control,male.vs.female; SRP223445,AD,male.vs.female; ACOM,totalSample,age; MCSA,MCI,age; ROSMAP,AD,last.visit.age; ROSMAP,AD,sample.age; Emory,totalSample,immune-Neutrophil; Emory,totalSample,immune-CD8_T; Emory,totalSample,immune-Gamma_delta; Emory,totalSample,immune-Th1; Emory,totalSample,immune-Tfh; Emory,totalSample,immune-Exhausted; Homo_723,totalSample,immune-CD8_T; Homo_723,totalSample,immune-Gamma_delta; Homo_723,totalSample,immune-Cytotoxic; Homo_723,totalSample,immune-Macrophage; Homo_723,totalSample,immune-Exhausted; Homo_723,totalSample,immune-NK; Homo_714,totalSample,immune-CD8_T; Homo_714,totalSample,immune-Exhausted; Homo_714,totalSample,immune-Cytotoxic; Homo_714,totalSample,immune-NKT; Homo_714,totalSample,immune-Macrophage; Homo_714,totalSample,immune-Gamma_delta; Homo_633,totalSample,immune-NKT; Homo_633,totalSample,immune-Gamma_delta; Homo_633,totalSample,immune-CD8_T; Homo_633,totalSample,immune-Cytotoxic; Homo_633,totalSample,immune-InfiltrationScore; MCSA,totalSample,immune-DC; MCSA,totalSample,immune-Monocyte; MCSA,totalSample,immune-CD4_T; MCSA,totalSample,immune-CD8_T; MCSA,totalSample,immune-Gamma_delta; MCSA,totalSample,immune-Tr1; MCSA,totalSample,immune-iTreg; MCSA,totalSample,immune-Th17; MCSA,totalSample,immune-Cytotoxic; MCSA,totalSample,immune-Exhausted; ROSMAP,totalSample,immune-Th17; ROSMAP,totalSample,immune-InfiltrationScore; SRP223445,totalSample,immune-CD4_naive
ABHD5	Homo_723,female,AD.vs.control; Homo_723,male,AD.vs.control; Homo_723,female,AD.vs.MCI; Homo_723,male,AD.vs.MCI; Homo_723,female,MCI.vs.control; Homo_714,female,AD.vs.control; Homo_714,male,AD.vs.control; Homo_714,female,AD.vs.MCI; Homo_714,male,AD.vs.MCI; Homo_714,female,MCI.vs.control; Homo_633,female,AD.vs.control; Homo_633,female,AD.vs.MCI; Homo_633,female,MCI.vs.control; Emory,totalSample,age; Emory,MCI,age; MCSA,MCI,age; ROSMAP,AD,last.visit.age; Emory,totalSample,immune-Monocyte; Emory,totalSample,immune-Neutrophil; Emory,totalSample,immune-CD4_T; Emory,totalSample,immune-CD8_T; Emory,totalSample,immune-Gamma_delta; Emory,totalSample,immune-nTreg; Emory,totalSample,immune-Th1; Emory,totalSample,immune-Th17; Emory,totalSample,immune-Tfh; Emory,totalSample,immune-CD8_naive; Emory,totalSample,immune-Cytotoxic; Emory,totalSample,immune-Exhausted; Homo_723,totalSample,immune-Monocyte; Homo_723,totalSample,immune-DC; Homo_723,totalSample,immune-Neutrophil; Homo_723,totalSample,immune-Th2; Homo_723,totalSample,immune-Gamma_delta; Homo_723,totalSample,immune-Exhausted; Homo_723,totalSample,immune-Bcell; Homo_714,totalSample,immune-Monocyte; Homo_714,totalSample,immune-Th2; Homo_714,totalSample,immune-Gamma_delta; Homo_714,totalSample,immune-Neutrophil; Homo_714,totalSample,immune-DC; Homo_714,totalSample,immune-Tfh; Homo_633,totalSample,immune-Monocyte; Homo_633,totalSample,immune-Th2; Homo_633,totalSample,immune-Exhausted; Homo_633,totalSample,immune-Th17; Homo_633,totalSample,immune-Gamma_delta; Homo_633,totalSample,immune-Neutrophil; Homo_633,totalSample,immune-DC; Homo_633,totalSample,immune-CD8_T; MCSA,totalSample,immune-DC; MCSA,totalSample,immune-Monocyte; MCSA,totalSample,immune-Neutrophil; MCSA,totalSample,immune-CD8_T; MCSA,totalSample,immune-Gamma_delta; MCSA,totalSample,immune-iTreg; MCSA,totalSample,immune-Th1; MCSA,totalSample,immune-Th17; MCSA,totalSample,immune- [illegible]
ACSBG1	Homo_723,female,AD.vs.control; Homo_723,male,AD.vs.control; Homo_723,female,AD.vs.MCI; Homo_723,male,AD.vs.MCI; Homo_723,female,MCI.vs.control; Homo_723,male,MCI.vs.control; Homo_714,female,AD.vs.control; Homo_714,male,AD.vs.control; Homo_714,female,AD.vs.MCI; Homo_714,male,AD.vs.MCI; Homo_714,female,MCI.vs.control; Homo_714,male,MCI.vs.control; Homo_633,female,AD.vs.control; Homo_633,female,AD.vs.MCI; Emory,totalSample,immune-Neutrophil; Emory,totalSample,immune-CD8_T; Emory,totalSample,immune-Gamma_delta; Emory,totalSample,immune-Exhausted; Homo_723,totalSample,immune-Th17; Homo_723,totalSample,immune-DC; Homo_723,totalSample,immune-Exhausted; Homo_723,totalSample,immune-Gamma_delta; Homo_714,totalSample,immune-Th17; Homo_714,totalSample,immune-Exhausted; Homo_714,totalSample,immune-DC; Homo_714,totalSample,immune-Gamma_delta; Homo_633,totalSample,immune-Gamma_delta; Homo_633,totalSample,immune-DC; Homo_633,totalSample,immune-Exhausted;
NCKAP1	Homo_723,female,AD.vs.control; Homo_723,male,AD.vs.control; Homo_723,female,AD.vs.MCI; Homo_723,male,AD.vs.MCI; Homo_723,female,MCI.vs.control; Homo_723,male,MCI.vs.control; Homo_714,female,AD.vs.control; Homo_714,male,AD.vs.control; Homo_714,female,AD.vs.MCI; Homo_714,male,AD.vs.MCI; Homo_714,female,MCI.vs.control; Homo_714,male,MCI.vs.control; Homo_633,female,AD.vs.control; Homo_633,female,AD.vs.MCI; Emory,totalSample,age; Emory,MCI,age; MCSA,MCI,age; Emory,totalSample,immune-CD4_T; Emory,totalSample,immune-Tr1; Emory,totalSample,immune-iTreg; Homo_723,totalSample,immune-Th17; Homo_723,totalSample,immune-Exhausted; Homo_723,totalSample,immune-CD4_naive; Homo_723,totalSample,immune-Cytotoxic; Homo_723,totalSample,immune-CD4_T; Homo_723,totalSample,immune-CD8_T; Homo_723,totalSample,immune-Tfh; Homo_723,totalSample,immune-InfiltrationScore; Homo_723,totalSample,immune-Central_memory; Homo_723,totalSample,immune-Tr1; Homo_723,totalSample,immune-Th1; Homo_723,totalSample,immune-Th2; Homo_723,totalSample,immune-NK; Homo_714,totalSample,immune-Th17; Homo_714,totalSample,immune-CD4_naive; Homo_714,totalSample,immune-InfiltrationScore; Homo_714,totalSample,immune-Exhausted; Homo_714,totalSample,immune-Monocyte; Homo_714,totalSample,immune-CD4_T; Homo_714,totalSample,immune-Tfh; Homo_714,totalSample,immune-Central_memory; Homo_714,totalSample,immune-NKT; Homo_633,totalSample,immune-Tfh; Homo_633,totalSample,immune-CD4_T; Homo_633,totalSample,immune-InfiltrationScore; Homo_633,totalSample,immune-Effector_memory; Homo_633,totalSample,immune-Th2; Homo_633,totalSample,immune-CD4_naive; Homo_633,totalSample,immune-Central_memory; Homo_633,totalSample,immune-Monocyte; Homo_633,totalSample,immune-Th17; Homo_633,totalSample,immune-Tr1; Homo_633,totalSample,immune-NKT; Homo_633,totalSample,immune-Exhausted; Homo_633,totalSample,immune-Bcell; MCSA,totalSample,immune-DC; MCSA,totalSample,immune-Monocyte; MCSA,totalSample,immune-Macrophage; MCSA,totalSample,immune-CD4_T; MCSA,totalSample,immune-CD4_naive; MCSA,totalSample,immune-Tr1; MCSA,totalSample,immune-nTreg; MCSA,totalSample,immune-iTreg; MCSA,totalSample,immune-Th17; MCSA,totalSample,immune-Tfh; MCSA,totalSample,immune-CD8_naive; [illegible]
ARHGEF26	Homo_723,female,AD.vs.control; Homo_723,male,AD.vs.control; Homo_723,female,AD.vs.MCI; Homo_723,male,AD.vs.MCI; Homo_714,female,AD.vs.control; Homo_714,male,AD.vs.control; Homo_714,female,AD.vs.MCI; Homo_714,male,AD.vs.MCI; Homo_714,female,MCI.vs.control; Homo_633,female,AD.vs.MCI; Homo_633,female,MCI.vs.control; Homo_633,male,MCI.vs.control; ROSMAP,male,AD.vs.control; ROSMAP,female,MCI.vs.control; ROSMAP,male,MCI.vs.control; ROSMAP,AD,male.vs.female; MCSA,MCI,age; ROSMAP,AD,MMSE; Homo_723,totalSample,immune-Monocyte; Homo_723,totalSample,immune-DC; Homo_714,totalSample,immune-InfiltrationScore; Homo_633,totalSample,immune-Monocyte; Homo_633,totalSample,immune-InfiltrationScore; MCSA,totalSample,immune-CD4_T; MCSA,totalSample,immune-iTreg; MCSA,totalSample,immune-Th17
HES1	Homo_723,female,AD.vs.control; Homo_723,male,AD.vs.control; Homo_723,female,AD.vs.MCI; Homo_723,male,AD.vs.MCI; Homo_723,female,MCI.vs.control; Homo_723,male,MCI.vs.control; Homo_714,female,AD.vs.control; Homo_714,male,AD.vs.control; Homo_714,female,AD.vs.MCI; Homo_714,male,AD.vs.MCI; Homo_714,female,MCI.vs.control; Homo_714,male,MCI.vs.control; Homo_633,female,AD.vs.control; Homo_633,female,AD.vs.MCI; ROSMAP,female,AD.vs.control; ROSMAP,female,MCI.vs.control; MCSA,MCI,age; Emory,totalSample,immune-Th1; Homo_723,totalSample,immune-Exhausted; Homo_723,totalSample,immune-Th17; Homo_723,totalSample,immune-DC; Homo_723,totalSample,immune-CD8_T; Homo_723,totalSample,immune-InfiltrationScore; Homo_714,totalSample,immune-Exhausted; Homo_714,totalSample,immune-InfiltrationScore; Homo_714,totalSample,immune-Th17; Homo_633,totalSample,immune-InfiltrationScore; Homo_633,totalSample,immune-Gamma_delta; Homo_633,totalSample,immune-Exhausted; Homo_633,totalSample,immune-Th2; MCSA,totalSample,immune-DC; MCSA,totalSample,immune-Monocyte; MCSA,totalSample,immune-Gamma_delta; MCSA,totalSample,immune-iTreg; MCSA,totalSample,immune-Exhausted; MCSA,totalSample,immune-InfiltrationScore; SRP223445,totalSample,immune-Exhausted; SRP223445,totalSample,immune-InfiltrationScore
DIRAS1	Homo_723,female,AD.vs.control; Homo_723,male,AD.vs.control; Homo_723,female,AD.vs.MCI; Homo_723,male,AD.vs.MCI; Homo_723,female,MCI.vs.control; Homo_723,male,MCI.vs.control; Homo_714,female,AD.vs.control; Homo_714,male,AD.vs.control; Homo_714,female,AD.vs.MCI; Homo_714,male,AD.vs.MCI; Homo_714,female,MCI.vs.control; Homo_714,male,MCI.vs.control; Homo_633,female,AD.vs.control; Homo_633,female,AD.vs.MCI; Homo_633,male,MCI.vs.control; ROSMAP,female,AD.vs.control; MCSA,totalSample,age; MCSA,control,age; ROSMAP,AD,last.visit.age; ROSMAP,AD,sample.age; ROSMAP,totalSample,age; ROSMAP,MCI,sample.age; ROSMAP,AD,MMSE; ROSMAP,totalSample,MMSE; Homo_723,totalSample,immune-Th17; Homo_723,totalSample,immune-Neutrophil; Homo_723,totalSample,immune-nTreg; Homo_723,totalSample,immune-DC; Homo_714,totalSample,immune-Th17; Homo_633,totalSample,immune-nTreg; MCSA,totalSample,immune-Bcell; MCSA,totalSample,immune-Monocyte; MCSA,totalSample,immune-Neutrophil; MCSA,totalSample,immune-nTreg; MCSA,totalSample,immune-Th17; MCSA,totalSample,immune-Tfh; MCSA,totalSample,immune-InfiltrationScore; ROSMAP,totalSample,immune-CD4_T; ROSMAP,totalSample,immune-Tr1; ROSMAP,totalSample,immune-iTreg; ROSMAP,totalSample,immune-CD8_naive; SRP223445,totalSample,immune-CD4_naive
RHOBTB1	Homo_723,female,AD.vs.control; Homo_723,male,AD.vs.control; Homo_723,female,AD.vs.MCI; Homo_723,male,AD.vs.MCI; Homo_723,female,MCI.vs.control; Homo_723,male,MCI.vs.control; Homo_714,female,AD.vs.control; Homo_714,male,AD.vs.control; Homo_714,female,AD.vs.MCI; Homo_714,male,AD.vs.MCI; Homo_714,female,MCI.vs.control; Homo_714,male,MCI.vs.control; Homo_633,female,AD.vs.control; Homo_633,female,AD.vs.MCI; ROSMAP,male,MCI.vs.control; ROSMAP,control,male.vs.female; Emory,MCI,age; ROSMAP,MCI,last.visit.age; Emory,totalSample,immune-Th1; Homo_723,totalSample,immune-Exhausted; Homo_723,totalSample,immune-Th17; Homo_723,totalSample,immune-CD8_T; Homo_723,totalSample,immune-Cytotoxic; Homo_723,totalSample,immune-DC; Homo_723,totalSample,immune-Gamma_delta; Homo_723,totalSample,immune-MAIT; Homo_714,totalSample,immune-Exhausted; Homo_714,totalSample,immune-Th17; Homo_714,totalSample,immune-CD8_T; Homo_714,totalSample,immune-Monocyte; Homo_714,totalSample,immune-Gamma_delta; Homo_633,totalSample,immune-Gamma_delta; Homo_633,totalSample,immune-Exhausted; Homo_633,totalSample,immune-Monocyte; Homo_633,totalSample,immune-Th2; MCSA,totalSample,immune-CD8_T; MCSA,totalSample,immune-iTreg; MCSA,totalSample,immune-Cytotoxic; MCSA,totalSample,immune-Exhausted; SRP223445,totalSample,immune- [illegible]
MPZL2	Homo_723,female,AD.vs.control; Homo_723,male,AD.vs.control; Homo_723,female,AD.vs.MCI; Homo_723,male,AD.vs.MCI; Homo_714,female,AD.vs.control; Homo_714,male,AD.vs.control; Homo_714,female,AD.vs.MCI; Homo_714,male,AD.vs.MCI; Homo_633,female,AD.vs.control; Homo_633,female,AD.vs.MCI; ROSMAP,female,AD.vs.control; Emory,totalSample,age; Emory,MCI,age; Emory,totalSample,immune-B_cell; Emory,totalSample,immune-Monocyte; Emory,totalSample,immune-Macrophage; Emory,totalSample,immune-NK; Emory,totalSample,immune-Neutrophil; Emory,totalSample,immune-CD8_T; Emory,totalSample,immune-Gamma_delta; Emory,totalSample,immune-Tfh; Emory,totalSample,immune-CD8_naive; Emory,totalSample,immune-Cytotoxic; Emory,totalSample,immune-Exhausted; Homo_723,totalSample,immune-Monocyte; Homo_723,totalSample,immune-DC; Homo_723,totalSample,immune-Gamma_delta; Homo_723,totalSample,immune-Neutrophil; Homo_723,totalSample,immune-Th2; Homo_723,totalSample,immune-iTreg; Homo_723,totalSample,immune-Tfh; Homo_723,totalSample,immune-Bcell; Homo_723,totalSample,immune-Exhausted; Homo_723,totalSample,immune-Tr1; Homo_714,totalSample,immune-Th2; Homo_714,totalSample,immune-Monocyte; Homo_714,totalSample,immune-Gamma_delta; Homo_714,totalSample,immune-Tfh; Homo_714,totalSample,immune-Tr1; Homo_714,totalSample,immune-Neutrophil; Homo_714,totalSample,immune-DC; Homo_714,totalSample,immune-Exhausted; Homo_633,totalSample,immune-Monocyte; Homo_633,totalSample,immune-Exhausted; Homo_633,totalSample,immune-Th2; Homo_633,totalSample,immune-Gamma_delta; Homo_633,totalSample,immune-Th17; Homo_633,totalSample,immune-CD8_T; Homo_633,totalSample,immune-Tfh; Homo_633,totalSample,immune-Neutrophil; Homo_633,totalSample,immune-DC; Homo_633,totalSample,immune-Cytotoxic; MCSA,totalSample,immune-DC; MCSA,totalSample,immune-Monocyte; MCSA,totalSample,immune-Neutrophil; MCSA,totalSample,immune-CD8_T; MCSA,totalSample,immune-Gamma_delta; MCSA,totalSample,immune-Tr1; MCSA,totalSample,immune-iTreg; MCSA,totalSample,immune-Th1; MCSA,totalSample,immune-Th17; MCSA,totalSample,immune-Cytotoxic; MCSA,totalSample,immune-Exhausted; MCSA,totalSample,immune-Effector_memory; ROSMAP,totalSample,immune-CD4_T; ROSMAP,totalSample,immune-Th17; ROSMAP,totalSample,immune-Effector_memory; [illegible]
ITGA2	Homo_723,female,AD.vs.control; Homo_723,male,AD.vs.control; Homo_723,female,AD.vs.MCI; Homo_723,male,AD.vs.MCI; Homo_723,female,MCI.vs.control; Homo_723,male,MCI.vs.control; Homo_714,female,AD.vs.control; Homo_714,male,AD.vs.control; Homo_714,female,AD.vs.MCI; Homo_714,male,AD.vs.MCI; Homo_714,female,MCI.vs.control; Homo_714,male,MCI.vs.control; Homo_633,female,AD.vs.control; Homo_633,female,AD.vs.MCI; ROSMAP,MCI,last.visit.age; Homo_723,totalSample,immune-Cytotoxic; Homo_723,totalSample,immune-Th17; Homo_723,totalSample,immune-Th1; Homo_723,totalSample,immune-CD4_naive; Homo_723,totalSample,immune-Exhausted; Homo_723,totalSample,immune-CD8_T; Homo_723,totalSample,immune-Central_memory; Homo_723,totalSample,immune-CD4_T; Homo_723,totalSample,immune-Gamma_delta; Homo_714,totalSample,immune-NKT; Homo_714,totalSample,immune-Th17; Homo_714,totalSample,immune-Exhausted; Homo_714,totalSample,immune-CD4_naive; Homo_714,totalSample,immune-Th1; Homo_714,totalSample,immune-Cytotoxic; Homo_714,totalSample,immune-CD8_T; Homo_714,totalSample,immune-Gamma_delta; Homo_633,totalSample,immune-Tfh; Homo_633,totalSample,immune-NKT; Homo_633,totalSample,immune-Gamma_delta; Homo_633,totalSample,immune-CD4_T; Homo_633,totalSample,immune-iTreg; Homo_633,totalSample,immune-CD4_naive; Homo_633,totalSample,immune-Tr1; Homo_633,totalSample,immune-Central_memory; MCSA,totalSample,immune-CD4_T; MCSA,totalSample,immune-CD8_T; MCSA,totalSample,immune-Gamma_delta; MCSA,totalSample,immune-CD4_naive; MCSA,totalSample,immune-Tr1; MCSA,totalSample,immune-nTreg; MCSA,totalSample,immune-iTreg; MCSA,totalSample,immune-Th17; MCSA,totalSample,immune-Tfh; MCSA,totalSample,immune-CD8_naive; MCSA,totalSample,immune-Cytotoxic; MCSA,totalSample,immune-Exhausted; MCSA,totalSample,immune-Central_memory; ROSMAP,totalSample,immune-InfiltrationScore; SRP223445,totalSample,immune-CD4_naive
RIMS3	Homo_723,female,AD.vs.control; Homo_723,female,AD.vs.MCI; Homo_633,female,AD.vs.control; Homo_633,female,AD.vs.MCI; MCSA,totalSample,age; MCSA,control,age; ROSMAP,MCI,last.visit.age; ROSMAP,MCI,sample.age; Emory,totalSample,immune-Macrophage; Emory,totalSample,immune-Neutrophil; Emory,totalSample,immune-CD4_T; Emory,totalSample,immune-Tr1; Emory,totalSample,immune-iTreg; Emory,totalSample,immune-InfiltrationScore; Homo_723,totalSample,immune-InfiltrationScore; Homo_723,totalSample,immune-CD4_naive; Homo_723,totalSample,immune-nTreg; Homo_723,totalSample,immune-CD4_T; Homo_723,totalSample,immune-Tfh; Homo_723,totalSample,immune-Monocyte; Homo_723,totalSample,immune-Central_memory; Homo_723,totalSample,immune-DC; Homo_723,totalSample,immune-iTreg; Homo_723,totalSample,immune-Neutrophil; Homo_723,totalSample,immune-Th2; Homo_723,totalSample,immune-Tr1; Homo_723,totalSample,immune-Effector_memory; Homo_723,totalSample,immune-Th17; Homo_714,totalSample,immune-InfiltrationScore; Homo_714,totalSample,immune-CD4_naive; Homo_714,totalSample,immune-Effector_memory; Homo_714,totalSample,immune-nTreg; Homo_714,totalSample,immune-CD4_T; Homo_714,totalSample,immune-Tfh; Homo_714,totalSample,immune-Monocyte; Homo_714,totalSample,immune-Neutrophil; Homo_714,totalSample,immune-NKT; Homo_714,totalSample,immune-Macrophage; Homo_714,totalSample,immune-iTreg; Homo_633,totalSample,immune-InfiltrationScore; Homo_633,totalSample,immune-nTreg; Homo_633,totalSample,immune-Tfh; Homo_633,totalSample,immune-CD4_T; Homo_633,totalSample,immune-CD4_naive; Homo_633,totalSample,immune-Effector_memory; Homo_633,totalSample,immune-Tr1; Homo_633,totalSample,immune-iTreg; Homo_633,totalSample,immune-Central_memory; Homo_633,totalSample,immune-Monocyte; Homo_633,totalSample,immune-NKT; Homo_633,totalSample,immune-Macrophage; MCSA,totalSample,immune-Monocyte; MCSA,totalSample,immune-Neutrophil; MCSA,totalSample,immune-CD4_T; MCSA,totalSample,immune-CD4_naive; MCSA,totalSample,immune-Tr1; MCSA,totalSample,immune-nTreg; MCSA,totalSample,immune-iTreg; MCSA,totalSample,immune-Th17; MCSA,totalSample,immune-Tfh; MCSA,totalSample,immune-Central_memory; MCSA,totalSample,immune-Effector_memory; MCSA,totalSample,immune-InfiltrationScore; [illegible]
SPACA9	Homo_723,female,AD.vs.control; Homo_723,male,AD.vs.control; Homo_723,female,AD.vs.MCI; Homo_723,male,AD.vs.MCI; Homo_723,male,MCI.vs.control; Homo_714,female,AD.vs.control; Homo_714,male,AD.vs.control; Homo_714,female,AD.vs.MCI; Homo_714,male,AD.vs.MCI; Homo_714,male,MCI.vs.control; Homo_633,female,AD.vs.control; Homo_633,female,AD.vs.MCI; Homo_633,female,MCI.vs.control; Homo_633,male,MCI.vs.control; MCSA,totalSample,age; MCSA,control,age; ROSMAP,AD,sample.age; Emory,totalSample,immune-Macrophage; Emory,totalSample,immune-CD4_T; Emory,totalSample,immune-Tr1; Emory,totalSample,immune-iTreg; Emory,totalSample,immune-InfiltrationScore; Homo_723,totalSample,immune-DC; Homo_723,totalSample,immune-Monocyte; Homo_714,totalSample,immune-Th2; Homo_714,totalSample,immune-DC; Homo_714,totalSample,immune-Monocyte; Homo_714,totalSample,immune-InfiltrationScore; Homo_633,totalSample,immune-Monocyte; Homo_633,totalSample,immune-Th2; Homo_633,totalSample,immune-DC; Homo_633,totalSample,immune-InfiltrationScore; MCSA,totalSample,immune-DC; MCSA,totalSample,immune-Monocyte; MCSA,totalSample,immune-CD8_T; MCSA,totalSample,immune-Cytotoxic; MCSA,totalSample,immune-Effector_memory; ROSMAP,totalSample,immune-InfiltrationScore; SRP223445,totalSample,immune-Exhausted
CALB1	Homo_723,female,AD.vs.control; Homo_723,male,AD.vs.control; Homo_723,female,AD.vs.MCI; Homo_723,male,AD.vs.MCI; Homo_723,female,MCI.vs.control; Homo_714,female,AD.vs.control; Homo_714,male,AD.vs.control; Homo_714,male,AD.vs.MCI; Homo_633,female,AD.vs.control; Homo_633,female,AD.vs.MCI; MCSA,MCI,age; Emory,totalSample,immune-Monocyte; Emory,totalSample,immune-Gamma_delta; Homo_723,totalSample,immune-Th2; Homo_723,totalSample,immune-Gamma_delta; Homo_723,totalSample,immune-DC; Homo_723,totalSample,immune-iTreg; Homo_723,totalSample,immune-CD4_T; Homo_723,totalSample,immune-Monocyte; Homo_714,totalSample,immune-Th2; Homo_714,totalSample,immune-Gamma_delta; Homo_714,totalSample,immune-Tfh; Homo_714,totalSample,immune-Monocyte; Homo_714,totalSample,immune-DC; Homo_633,totalSample,immune-Exhausted; Homo_633,totalSample,immune-NKT; Homo_633,totalSample,immune-Gamma_delta; Homo_633,totalSample,immune-Th2; Homo_633,totalSample,immune-DC; Homo_633,totalSample,immune-Monocyte; Homo_633,totalSample,immune-Tfh; Homo_633,totalSample,immune-Th17; MCSA,totalSample,immune-DC; MCSA,totalSample,immune-Monocyte; MCSA,totalSample,immune-CD4_T; MCSA,totalSample,immune-CD8_T; MCSA,totalSample,immune-Tr1; MCSA,totalSample,immune-iTreg; MCSA,totalSample,immune-Th17; MCSA,totalSample,immune-CD8_naive; MCSA,totalSample,immune-Cytotoxic; MCSA,totalSample,immune-Exhausted; MCSA,totalSample,immune-Effector_memory

ANGPT1	Homo_723,female,AD.vs.control; Homo_723,male,AD.vs.control; Homo_723,female,AD.vs.MCI; Homo_723,male,AD.vs.MCI; Homo_723,female,MCI.vs.control; Homo_723,male,MCI.vs.control; Homo_714,female,AD.vs.control; Homo_714,male,AD.vs.control; Homo_714,female,AD.vs.MCI; Homo_714,male,AD.vs.MCI; Homo_714,female,MCI.vs.control; Homo_714,male,MCI.vs.control; Homo_633,female,AD.vs.control; Homo_633,female,AD.vs.MCI; ROSMAP,female,AD.vs.control; ROSMAP,male,AD.vs.control; MCSA,MCI,age; Emory,totalSample,immune-DC; Emory,totalSample,immune-Monocyte; Emory,totalSample,immune-Neutrophil; Emory,totalSample,immune-Gamma_delta; Emory,totalSample,immune-Tr1; Emory,totalSample,immune-iTreg; Emory,totalSample,immune-Th2; Emory,totalSample,immune-Th17; Emory,totalSample,immune-Exhausted; Homo_723,totalSample,immune-Exhausted; Homo_723,totalSample,immune-Th17; Homo_723,totalSample,immune-CD8_T; Homo_723,totalSample,immune-Cytotoxic; Homo_723,totalSample,immune-Monocyte; Homo_723,totalSample,immune-CD4_naive; Homo_723,totalSample,immune-NKT; Homo_714,totalSample,immune-Exhausted; Homo_714,totalSample,immune-Th17; Homo_714,totalSample,immune-Monocyte; Homo_714,totalSample,immune-CD8_T; Homo_714,totalSample,immune-NKT; Homo_714,totalSample,immune-Cytotoxic; Homo_633,totalSample,immune-Exhausted; Homo_633,totalSample,immune-Tfh; Homo_633,totalSample,immune-Monocyte; Homo_633,totalSample,immune-Th2; Homo_633,totalSample,immune-NKT; Homo_633,totalSample,immune-Gamma_delta; Homo_633,totalSample,immune-InfiltrationScore; Homo_633,totalSample,immune-Th17; Homo_633,totalSample,immune-DC; Homo_633,totalSample,immune-CD4_T; Homo_633,totalSample,immune-Tr1; MCSA,totalSample,immune-DC; MCSA,totalSample,immune-Monocyte; MCSA,totalSample,immune-CD4_T; MCSA,totalSample,immune-CD8_T; MCSA,totalSample,immune-CD4_naive; MCSA,totalSample,immune-Tr1; MCSA,totalSample,immune-nTreg; MCSA,totalSample,immune-iTreg; MCSA,totalSample,immune-Th1; MCSA,totalSample,immune-Th17; MCSA,totalSample,immune-CD8_naive; MCSA,totalSample,immune-Cytotoxic; MCSA,totalSample,immune-Exhausted; MCSA,totalSample,immune-MAIT; MCSA,totalSample,immune-Central_memory; ROSMAP,totalSample,immune-Th17; ROSMAP,totalSample,immune-InfiltrationScore; [illegible]
SIK1	Homo_723,female,AD.vs.control; Homo_723,male,AD.vs.control; Homo_723,female,AD.vs.MCI; Homo_723,male,AD.vs.MCI; Homo_714,female,AD.vs.control; Homo_714,male,AD.vs.control; Homo_714,female,AD.vs.MCI; Homo_714,male,AD.vs.MCI; Homo_633,female,AD.vs.control; Homo_633,female,AD.vs.MCI; ROSMAP,female,AD.vs.control; ROSMAP,female,MCI.vs.control; Emory,totalSample,immune-B_cell; Emory,totalSample,immune-NK; Emory,totalSample,immune-Effector_memory; Emory,totalSample,immune-InfiltrationScore; Homo_723,totalSample,immune-DC; Homo_723,totalSample,immune-Monocyte; Homo_723,totalSample,immune-NK; Homo_723,totalSample,immune-InfiltrationScore; Homo_723,totalSample,immune-Neutrophil; Homo_714,totalSample,immune-InfiltrationScore; Homo_714,totalSample,immune-NK; Homo_714,totalSample,immune-DC; Homo_714,totalSample,immune-NKT; Homo_633,totalSample,immune-InfiltrationScore; Homo_633,totalSample,immune-NKT; Homo_633,totalSample,immune-Monocyte; Homo_633,totalSample,immune-NK; MCSA,totalSample,immune-NK; MCSA,totalSample,immune-Tr1; MCSA,totalSample,immune-nTreg; MCSA,totalSample,immune-iTreg; MCSA,totalSample,immune-Tfh; MCSA,totalSample,immune-Central_memory; SRP223445,totalSample,immune-Exhausted
TMEM163	Homo_723,female,AD.vs.control; Homo_723,male,AD.vs.control; Homo_723,female,AD.vs.MCI; Homo_723,male,AD.vs.MCI; Homo_723,male,MCI.vs.control; Homo_714,female,AD.vs.control; Homo_714,male,AD.vs.control; Homo_714,female,AD.vs.MCI; Homo_714,male,AD.vs.MCI; Homo_633,female,AD.vs.control; Homo_633,female,AD.vs.MCI; ROSMAP,female,AD.vs.control; ROSMAP,female,MCI.vs.control; ROSMAP,male,MCI.vs.control; ROSMAP,control,male.vs.female; ROSMAP,MCI,male.vs.female; Emory,totalSample,immune-InfiltrationScore; Homo_723,totalSample,immune-DC; Homo_723,totalSample,immune-Th17; Homo_723,totalSample,immune-NK; Homo_723,totalSample,immune-InfiltrationScore; Homo_714,totalSample,immune-NK; Homo_714,totalSample,immune-InfiltrationScore; Homo_633,totalSample,immune-InfiltrationScore; Homo_633,totalSample,immune-NK; MCSA,totalSample,immune-Th17; ROSMAP,totalSample,immune-InfiltrationScore; SRP223445,totalSample,immune-CD4_naive; SRP223445,totalSample,immune-Exhausted
STAU2	Homo_723,female,AD.vs.control; Homo_723,male,AD.vs.control; Homo_723,female,AD.vs.MCI; Homo_723,male,AD.vs.MCI; Homo_723,female,MCI.vs.control; Homo_714,female,AD.vs.control; Homo_714,male,AD.vs.control; Homo_714,female,AD.vs.MCI; Homo_714,male,AD.vs.MCI; Homo_714,female,MCI.vs.control; Homo_633,female,AD.vs.control; Homo_633,female,AD.vs.MCI; MCSA,MCI,age; Emory,totalSample,immune-CD4_T; Emory,totalSample,immune-Tr1; Emory,totalSample,immune-iTreg; Emory,totalSample,immune-Effector_memory; Emory,totalSample,immune-InfiltrationScore; Homo_723,totalSample,immune-Exhausted; Homo_723,totalSample,immune-Cytotoxic; Homo_723,totalSample,immune-Th17; Homo_723,totalSample,immune-CD4_naive; Homo_723,totalSample,immune-CD8_T; Homo_723,totalSample,immune-CD4_T; Homo_723,totalSample,immune-Tr1; Homo_723,totalSample,immune-Central_memory; Homo_723,totalSample,immune-Tfh; Homo_723,totalSample,immune-InfiltrationScore; Homo_723,totalSample,immune-Th1; Homo_723,totalSample,immune-NKT; Homo_723,totalSample,immune-Th2; Homo_714,totalSample,immune-CD4_naive; Homo_714,totalSample,immune-Exhausted; Homo_714,totalSample,immune-Th17; Homo_714,totalSample,immune-InfiltrationScore; Homo_714,totalSample,immune-NKT; Homo_714,totalSample,immune-Tfh; Homo_714,totalSample,immune-CD4_T; Homo_714,totalSample,immune-Central_memory; Homo_714,totalSample,immune-Monocyte; Homo_714,totalSample,immune-Tr1; Homo_714,totalSample,immune-CD8_T; Homo_714,totalSample,immune-Cytotoxic; Homo_633,totalSample,immune-Tfh; Homo_633,totalSample,immune-CD4_T; Homo_633,totalSample,immune-CD4_naive; Homo_633,totalSample,immune-InfiltrationScore; Homo_633,totalSample,immune-Effector_memory; Homo_633,totalSample,immune-Th2; Homo_633,totalSample,immune-Central_memory; Homo_633,totalSample,immune-NKT; Homo_633,totalSample,immune-Tr1; Homo_633,totalSample,immune-Exhausted; Homo_633,totalSample,immune-iTreg; Homo_633,totalSample,immune-Monocyte; Homo_633,totalSample,immune-DC; Homo_633,totalSample,immune-Gamma_delta; Homo_633,totalSample,immune-Bcell; Homo_633,totalSample,immune-Th17; MCSA,totalSample,immune-DC; MCSA,totalSample,immune-Monocyte; MCSA,totalSample,immune-Macrophage; MCSA,totalSample,immune-CD4_T; MCSA,totalSample,immune-CD8_T; MCSA,totalSample,immune-CD4_naive; MCSA,totalSample,immune-Tr1; MCSA,totalSample,immune-nTreg; MCSA,totalSample,immune-iTreg; MCSA,totalSample,immune-Th17; MCSA,totalSample,immune-Tfh; MCSA,totalSample,immune-CD8_naive; MCSA,totalSample,immune-Cytotoxic; MCSA,totalSample,immune-Exhausted; MCSA,totalSample,immune-MAIT; [illegible]
CARMIL1	Homo_723,female,AD.vs.control; Homo_723,male,AD.vs.control; Homo_723,female,AD.vs.MCI; Homo_723,male,AD.vs.MCI; Homo_723,female,MCI.vs.control; Homo_723,male,MCI.vs.control; Homo_714,female,AD.vs.control; Homo_714,male,AD.vs.control; Homo_714,female,AD.vs.MCI; Homo_714,male,AD.vs.MCI; Homo_714,female,MCI.vs.control; Homo_714,male,MCI.vs.control; Homo_633,female,AD.vs.control; Homo_633,female,AD.vs.MCI; ROSMAP,female,AD.vs.control; ROSMAP,control,male.vs.female; Emory,totalSample,immune-CD4_T; Emory,totalSample,immune-Gamma_delta; Emory,totalSample,immune-CD4_naive; Emory,totalSample,immune-Tr1; Emory,totalSample,immune-iTreg; Emory,totalSample,immune-Th17; Emory,totalSample,immune-Central_memory; Emory,totalSample,immune-InfiltrationScore; Homo_723,totalSample,immune-CD4_naive; Homo_723,totalSample,immune-Central_memory; Homo_723,totalSample,immune-CD4_T; Homo_723,totalSample,immune-Cytotoxic; Homo_723,totalSample,immune-nTreg; Homo_723,totalSample,immune-Th17; Homo_723,totalSample,immune-Th1; Homo_723,totalSample,immune-Tr1; Homo_723,totalSample,immune-Tfh; Homo_723,totalSample,immune-iTreg; Homo_723,totalSample,immune-Gamma_delta; Homo_723,totalSample,immune-Neutrophil; Homo_723,totalSample,immune-NKT; Homo_714,totalSample,immune-CD4_naive; Homo_714,totalSample,immune-Central_memory; Homo_714,totalSample,immune-Th1; Homo_714,totalSample,immune-NKT; Homo_714,totalSample,immune-CD4_T; Homo_714,totalSample,immune-nTreg; Homo_714,totalSample,immune-Tr1; Homo_714,totalSample,immune-Cytotoxic; Homo_714,totalSample,immune-Tfh; Homo_714,totalSample,immune-iTreg; Homo_714,totalSample,immune-Th17; Homo_714,totalSample,immune-Gamma_delta; Homo_714,totalSample,immune-Exhausted; Homo_633,totalSample,immune-Tfh; Homo_633,totalSample,immune-CD4_T; Homo_633,totalSample,immune-Tr1; Homo_633,totalSample,immune-CD4_naive; Homo_633,totalSample,immune-Central_memory; Homo_633,totalSample,immune-iTreg; Homo_633,totalSample,immune-NKT; Homo_633,totalSample,immune-nTreg; Homo_633,totalSample,immune-Gamma_delta; Homo_633,totalSample,immune-Effector_memory; Homo_633,totalSample,immune-Th2; Homo_633,totalSample,immune-Th1; Homo_633,totalSample,immune-Cytotoxic; Homo_633,totalSample,immune-InfiltrationScore; MCSA,totalSample,immune-Bcell; MCSA,totalSample,immune-Macrophage; MCSA,totalSample,immune-CD4_T; MCSA,totalSample,immune-Gamma_delta; MCSA,totalSample,immune-CD4_naive; MCSA,totalSample,immune-Tr1; MCSA,totalSample,immune-nTreg; MCSA,totalSample,immune-iTreg; MCSA,totalSample,immune-Th17; [illegible]
SNRNP40	Homo_723,female,AD.vs.control; Homo_723,male,AD.vs.control; Homo_723,female,AD.vs.MCI; Homo_723,male,AD.vs.MCI; Homo_714,female,AD.vs.control; Homo_714,male,AD.vs.control; Homo_714,female,AD.vs.MCI; Homo_714,male,AD.vs.MCI; Homo_633,female,AD.vs.control; Homo_633,female,AD.vs.MCI; ROSMAP,male,AD.vs.control; ROSMAP,AD,sample.age; Emory,totalSample,immune-Macrophage; Emory,totalSample,immune-NK; Emory,totalSample,immune-Neutrophil; Emory,totalSample,immune-CD4_T; Emory,totalSample,immune-CD8_T; Emory,totalSample,immune-Tr1; Emory,totalSample,immune-iTreg; Emory,totalSample,immune-Tfh; Emory,totalSample,immune-Exhausted; Emory,totalSample,immune-MAIT; Emory,totalSample,immune-Central_memory; Emory,totalSample,immune-Effector_memory; Emory,totalSample,immune-InfiltrationScore; Homo_723,totalSample,immune-Tfh; Homo_723,totalSample,immune-Th2; Homo_723,totalSample,immune-iTreg; Homo_723,totalSample,immune-Effector_memory; Homo_723,totalSample,immune-InfiltrationScore; Homo_723,totalSample,immune-CD4_T; Homo_723,totalSample,immune-CD4_naive; Homo_723,totalSample,immune-Tr1; Homo_723,totalSample,immune-Macrophage; Homo_723,totalSample,immune-nTreg; Homo_723,totalSample,immune-MAIT; Homo_723,totalSample,immune-Bcell; Homo_723,totalSample,immune-Central_memory; Homo_723,totalSample,immune-CD8_T; Homo_723,totalSample,immune-DC; Homo_714,totalSample,immune-Tfh; Homo_714,totalSample,immune-Th2; Homo_714,totalSample,immune-Effector_memory; Homo_714,totalSample,immune-CD4_T; Homo_714,totalSample,immune-InfiltrationScore; Homo_714,totalSample,immune-Tr1; Homo_714,totalSample,immune-CD4_naive; Homo_714,totalSample,immune-Central_memory; Homo_714,totalSample,immune-iTreg; Homo_714,totalSample,immune-nTreg; Homo_714,totalSample,immune-MAIT; Homo_714,totalSample,immune-Macrophage; Homo_714,totalSample,immune-CD8_T; Homo_714,totalSample,immune-Bcell; Homo_714,totalSample,immune-Neutrophil; Homo_633,totalSample,immune-Tfh; Homo_633,totalSample,immune-Effector_memory; Homo_633,totalSample,immune-InfiltrationScore; Homo_633,totalSample,immune-CD4_T; Homo_633,totalSample,immune-CD4_naive; Homo_633,totalSample,immune-Th2; Homo_633,totalSample,immune-Central_memory; Homo_633,totalSample,immune-Neutrophil; Homo_633,totalSample,immune-Tr1; Homo_633,totalSample,immune-Bcell; Homo_633,totalSample,immune-nTreg; Homo_633,totalSample,immune-iTreg; Homo_633,totalSample,immune-MAIT; MCSA,totalSample,immune-DC; MCSA,totalSample,immune-Macrophage; MCSA,totalSample,immune-Neutrophil; MCSA,totalSample,immune-CD4_T; MCSA,totalSample,immune-CD4_naive; MCSA,totalSample,immune-Tr1; MCSA,totalSample,immune-nTreg; MCSA,totalSample,immune-iTreg; MCSA,totalSample,immune-Th2; MCSA,totalSample,immune-Th17; MCSA,totalSample,immune-Tfh; MCSA,totalSample,immune-CD8_naive; MCSA,totalSample,immune-MAIT; MCSA,totalSample,immune-Central_memory; [illegible]
KIF23	Homo_723,female,AD.vs.control; Homo_723,male,AD.vs.control; Homo_723,female,AD.vs.MCI; Homo_723,male,AD.vs.MCI; Homo_723,female,MCI.vs.control; Homo_723,male,MCI.vs.control; Homo_714,female,AD.vs.control; Homo_714,male,AD.vs.control; Homo_714,female,AD.vs.MCI; Homo_714,male,AD.vs.MCI; Homo_714,female,MCI.vs.control; Homo_714,male,MCI.vs.control; Homo_633,female,AD.vs.control; Homo_633,female,AD.vs.MCI; MCSA,MCI,age; Emory,totalSample,immune-Macrophage; Emory,totalSample,immune-Gamma_delta; Emory,totalSample,immune-Cytotoxic; Homo_723,totalSample,immune-Exhausted; Homo_723,totalSample,immune-CD8_T; Homo_723,totalSample,immune-Cytotoxic; Homo_723,totalSample,immune-Th17; Homo_723,totalSample,immune-Monocyte; Homo_723,totalSample,immune-Macrophage; Homo_714,totalSample,immune-Monocyte; Homo_714,totalSample,immune-Exhausted; Homo_714,totalSample,immune-Th17; Homo_714,totalSample,immune-CD8_T; Homo_714,totalSample,immune-Macrophage; Homo_633,totalSample,immune-Monocyte; Homo_633,totalSample,immune-Th2; Homo_633,totalSample,immune-Exhausted; Homo_633,totalSample,immune-Gamma_delta; Homo_633,totalSample,immune-Tfh; Homo_633,totalSample,immune-InfiltrationScore; Homo_633,totalSample,immune-Th17; MCSA,totalSample,immune-DC; MCSA,totalSample,immune-Monocyte; MCSA,totalSample,immune-CD8_T; MCSA,totalSample,immune-Tr1; MCSA,totalSample,immune-iTreg; MCSA,totalSample,immune-Cytotoxic; MCSA,totalSample,immune-Exhausted; ROSMAP,totalSample,immune-InfiltrationScore; ROSMAP,female-excludeAge90+,survival
POLDIP2	Homo_723,female,AD.vs.control; Homo_723,male,AD.vs.control; Homo_723,female,AD.vs.MCI; Homo_723,male,AD.vs.MCI; Homo_714,female,AD.vs.control; Homo_714,male,AD.vs.control; Homo_714,female,AD.vs.MCI; Homo_714,male,AD.vs.MCI; Homo_633,female,AD.vs.control; Homo_633,female,AD.vs.MCI; MCSA,totalSample,age; MCSA,control,age; Emory,totalSample,immune-Monocyte; Emory,totalSample,immune-NK; Emory,totalSample,immune-Neutrophil; Emory,totalSample,immune-CD4_T; Emory,totalSample,immune-CD8_T; Emory,totalSample,immune-Tr1; Emory,totalSample,immune-iTreg; Emory,totalSample,immune-Exhausted; Emory,totalSample,immune-MAIT; Emory,totalSample,immune-Effector_memory; Emory,totalSample,immune-InfiltrationScore; Homo_723,totalSample,immune-InfiltrationScore; Homo_723,totalSample,immune-Effector_memory; Homo_723,totalSample,immune-Tfh; Homo_723,totalSample,immune-Th2; Homo_723,totalSample,immune-iTreg; Homo_723,totalSample,immune-nTreg; Homo_723,totalSample,immune-CD4_T; Homo_723,totalSample,immune-CD8_T; Homo_723,totalSample,immune-CD4_naive; Homo_723,totalSample,immune-Cytotoxic; Homo_723,totalSample,immune-Tr1; Homo_723,totalSample,immune-Macrophage; Homo_723,totalSample,immune-Bcell; Homo_714,totalSample,immune-Effector_memory; Homo_714,totalSample,immune-Tfh; Homo_714,totalSample,immune-InfiltrationScore; Homo_714,totalSample,immune-Th2; Homo_714,totalSample,immune-nTreg; Homo_714,totalSample,immune-Central_memory; Homo_714,totalSample,immune-CD4_T; Homo_714,totalSample,immune-Tr1; Homo_714,totalSample,immune-CD8_T; Homo_633,totalSample,immune-InfiltrationScore; Homo_633,totalSample,immune-Effector_memory; Homo_633,totalSample,immune-nTreg; Homo_633,totalSample,immune-Tfh; Homo_633,totalSample,immune-Central_memory; Homo_633,totalSample,immune-CD4_T; Homo_633,totalSample,immune-Th2; Homo_633,totalSample,immune-DC; Homo_633,totalSample,immune-CD4_naive; MCSA,totalSample,immune-DC; MCSA,totalSample,immune-Monocyte; MCSA,totalSample,immune-Neutrophil; MCSA,totalSample,immune-CD4_T; MCSA,totalSample,immune-CD8_T; MCSA,totalSample,immune-CD4_naive; MCSA,totalSample,immune-nTreg; MCSA,totalSample,immune-Th2; MCSA,totalSample,immune-Th17; MCSA,totalSample,immune-Tfh; MCSA,totalSample,immune-Cytotoxic; MCSA,totalSample,immune-Central_memory; MCSA,totalSample,immune-Effector_memory; MCSA,totalSample,immune-InfiltrationScore; ROSMAP,totalSample,immune-Effector_memory; SRP223445,totalSample,immune-CD4_naive; SRP223445,totalSample,immune-Exhausted; SRP223445,totalSample,immune-InfiltrationScore
TTLL7	Homo_723,female,AD.vs.control; Homo_723,male,AD.vs.control; Homo_723,female,AD.vs.MCI; Homo_723,male,AD.vs.MCI; Homo_723,female,MCI.vs.control; Homo_723,male,MCI.vs.control; Homo_714,female,AD.vs.control; Homo_714,male,AD.vs.control; Homo_714,female,AD.vs.MCI; Homo_714,male,AD.vs.MCI; Homo_714,female,MCI.vs.control; Homo_714,male,MCI.vs.control; Homo_633,female,AD.vs.control; Homo_633,female,AD.vs.MCI; MCSA,MCI,age; ROSMAP,AD,last.visit.age; Homo_723,totalSample,immune-CD8_T; Homo_723,totalSample,immune-Cytotoxic; Homo_723,totalSample,immune-NKT; Homo_723,totalSample,immune-Exhausted; Homo_723,totalSample,immune-Th17; Homo_723,totalSample,immune-NK; Homo_714,totalSample,immune-NKT; Homo_714,totalSample,immune-CD8_T; Homo_714,totalSample,immune-Exhausted; Homo_633,totalSample,immune-NKT; Homo_633,totalSample,immune-InfiltrationScore; MCSA,totalSample,immune-DC; MCSA,totalSample,immune-Monocyte; MCSA,totalSample,immune-CD4_T; MCSA,totalSample,immune-CD8_T; MCSA,totalSample,immune-Tr1; MCSA,totalSample,immune-iTreg; MCSA,totalSample,immune-Cytotoxic; MCSA,totalSample,immune-Exhausted; ROSMAP,totalSample,immune-InfiltrationScore; SRP223445,totalSample,immune-CD4_naive; SRP223445,totalSample,immune-Exhausted
RUFY2	Homo_723,female,AD.vs.control; Homo_723,male,AD.vs.control; Homo_723,female,AD.vs.MCI; Homo_723,male,AD.vs.MCI; Homo_714,female,AD.vs.control; Homo_714,male,AD.vs.control; Homo_714,female,AD.vs.MCI; Homo_714,male,AD.vs.MCI; Homo_633,female,AD.vs.control; Homo_633,female,AD.vs.MCI; MCSA,MCI,age; Emory,totalSample,immune-Macrophage; Emory,totalSample,immune-Neutrophil; Emory,totalSample,immune-CD4_T; Emory,totalSample,immune-CD8_T; Emory,totalSample,immune-Tr1; Emory,totalSample,immune-iTreg; Emory,totalSample,immune-Tfh; Emory,totalSample,immune-Central_memory; Emory,totalSample,immune-Effector_memory; Emory,totalSample,immune-InfiltrationScore; Homo_723,totalSample,immune-CD4_naive; Homo_723,totalSample,immune-CD4_T; Homo_723,totalSample,immune-Th17; Homo_723,totalSample,immune-Central_memory; Homo_723,totalSample,immune-Cytotoxic; Homo_723,totalSample,immune-Tr1; Homo_723,totalSample,immune-Tfh; Homo_723,totalSample,immune-Exhausted; Homo_723,totalSample,immune-Th1; Homo_723,totalSample,immune-nTreg; Homo_723,totalSample,immune-Th2; Homo_723,totalSample,immune-iTreg; Homo_723,totalSample,immune-InfiltrationScore; Homo_723,totalSample,immune-Neutrophil; Homo_723,totalSample,immune-CD8_T; Homo_723,totalSample,immune-NKT; Homo_714,totalSample,immune-CD4_naive; Homo_714,totalSample,immune-CD4_T; Homo_714,totalSample,immune-Tfh; Homo_714,totalSample,immune-Central_memory; Homo_714,totalSample,immune-Th17; Homo_714,totalSample,immune-NKT; Homo_714,totalSample,immune-Tr1; Homo_714,totalSample,immune-InfiltrationScore; Homo_714,totalSample,immune-Th1; Homo_714,totalSample,immune-iTreg; Homo_714,totalSample,immune-Exhausted; Homo_714,totalSample,immune-Effector_memory; Homo_714,totalSample,immune-nTreg; Homo_714,totalSample,immune-Cytotoxic; Homo_633,totalSample,immune-Tfh; Homo_633,totalSample,immune-CD4_T; Homo_633,totalSample,immune-CD4_naive; Homo_633,totalSample,immune-Central_memory; Homo_633,totalSample,immune-Tr1; Homo_633,totalSample,immune-Effector_memory; Homo_633,totalSample,immune-Th2; Homo_633,totalSample,immune-NKT; Homo_633,totalSample,immune-InfiltrationScore; Homo_633,totalSample,immune-iTreg; Homo_633,totalSample,immune-nTreg; Homo_633,totalSample,immune-Gamma_delta; Homo_633,totalSample,immune-DC; Homo_633,totalSample,immune-Th17; Homo_633,totalSample,immune-Exhausted; Homo_633,totalSample,immune-Th1; Homo_633,totalSample,immune-MAIT; Homo_633,totalSample,immune-Bcell; MCSA,totalSample,immune-DC; MCSA,totalSample,immune-Monocyte; MCSA,totalSample,immune-Macrophage; MCSA,totalSample,immune-CD4_T; MCSA,totalSample,immune-CD8_T; MCSA,totalSample,immune-CD4_naive; MCSA,totalSample,immune-Tr1; MCSA,totalSample,immune-nTreg; MCSA,totalSample,immune-iTreg; MCSA,totalSample,immune-Th17; MCSA,totalSample,immune-Tfh; MCSA,totalSample,immune-CD8_naive; MCSA,totalSample,immune-Cytotoxic; MCSA,totalSample,immune-Exhausted; [illegible]
MYLK	Homo_723,female,AD.vs.control; Homo_723,male,AD.vs.control; Homo_723,female,AD.vs.MCI; Homo_723,male,AD.vs.MCI; Homo_723,female,MCI.vs.control; Homo_723,male,MCI.vs.control; Homo_714,female,AD.vs.control; Homo_714,male,AD.vs.control; Homo_714,female,AD.vs.MCI; Homo_714,male,AD.vs.MCI; Homo_714,female,MCI.vs.control; Homo_714,male,MCI.vs.control; Homo_633,female,AD.vs.control; Homo_633,female,AD.vs.MCI; ACOM,totalSample,age; MCSA,totalSample,age; MCSA,control,age; ROSMAP,AD,last.visit.age; Emory,totalSample,immune-Th1; Homo_723,totalSample,immune-DC; Homo_723,totalSample,immune-Th17; Homo_723,totalSample,immune-Central_memory; Homo_723,totalSample,immune-Cytotoxic; Homo_723,totalSample,immune-CD4_naive; Homo_723,totalSample,immune-Gamma_delta; Homo_723,totalSample,immune-CD8_T; Homo_723,totalSample,immune-InfiltrationScore; Homo_714,totalSample,immune-Exhausted; Homo_714,totalSample,immune-CD8_T; Homo_714,totalSample,immune-Th17; Homo_714,totalSample,immune-NKT; Homo_714,totalSample,immune-Cytotoxic; Homo_714,totalSample,immune-DC; Homo_633,totalSample,immune-Tfh; Homo_633,totalSample,immune-InfiltrationScore; Homo_633,totalSample,immune-NKT; Homo_633,totalSample,immune-Gamma_delta; Homo_633,totalSample,immune-iTreg; Homo_633,totalSample,immune-CD4_T; MCSA,totalSample,immune-CD4_T; MCSA,totalSample,immune-Gamma_delta; MCSA,totalSample,immune-CD4_naive; MCSA,totalSample,immune-Tr1; MCSA,totalSample,immune-nTreg; MCSA,totalSample,immune-iTreg; MCSA,totalSample,immune-Th17; MCSA,totalSample,immune-Tfh; MCSA,totalSample,immune-CD8_naive; MCSA,totalSample,immune-Cytotoxic; MCSA,totalSample,immune-Exhausted; MCSA,totalSample,immune-Central_memory; ROSMAP,totalSample,immune-InfiltrationScore; SRP223445,totalSample,immune-CD4_naive; SRP223445,totalSample,immune-Exhausted; ROSMAP,male-includeAge90+,survival
GNS	Homo_723,female,AD.vs.control; Homo_723,male,AD.vs.control; Homo_723,female,AD.vs.MCI; Homo_723,male,AD.vs.MCI; Homo_714,female,AD.vs.control; Homo_714,male,AD.vs.control; Homo_714,female,AD.vs.MCI; Homo_714,male,AD.vs.MCI; Homo_633,female,AD.vs.control; Homo_633,female,AD.vs.MCI; ROSMAP,female,AD.vs.control; ROSMAP,female,MCI.vs.control; ROSMAP,control,male.vs.female; ACOM,AD,age; ACOM,totalSample,age; Emory,totalSample,age; Emory,MCI,age; ROSMAP,MCI,last.visit.age; ROSMAP,MCI,sample.age; Emory,totalSample,immune-B_cell; Emory,totalSample,immune-Macrophage; Emory,totalSample,immune-Neutrophil; Emory,totalSample,immune-CD4_T; Emory,totalSample,immune-CD8_T; Emory,totalSample,immune-Gamma_delta; Emory,totalSample,immune-Th1; Emory,totalSample,immune-Tfh; Emory,totalSample,immune-CD8_naive; Emory,totalSample,immune-Cytotoxic; Emory,totalSample,immune-Exhausted; Homo_723,totalSample,immune-Th2; Homo_723,totalSample,immune-Monocyte; Homo_723,totalSample,immune-Tfh; Homo_723,totalSample,immune-Gamma_delta; Homo_723,totalSample,immune-DC; Homo_723,totalSample,immune-iTreg; Homo_723,totalSample,immune-InfiltrationScore; Homo_723,totalSample,immune-Bcell; Homo_723,totalSample,immune-Effector_memory; Homo_723,totalSample,immune-Neutrophil; Homo_723,totalSample,immune-Exhausted; Homo_723,totalSample,immune-Tr1; Homo_723,totalSample,immune-CD4_T; Homo_714,totalSample,immune-Th2; Homo_714,totalSample,immune-Tfh; Homo_714,totalSample,immune-Monocyte; Homo_714,totalSample,immune-Gamma_delta; Homo_714,totalSample,immune-Effector_memory; Homo_714,totalSample,immune-Tr1; Homo_714,totalSample,immune-CD4_T; Homo_633,totalSample,immune-Th2; Homo_633,totalSample,immune-Monocyte; Homo_633,totalSample,immune-Exhausted; Homo_633,totalSample,immune-Gamma_delta; Homo_633,totalSample,immune-Th17; Homo_633,totalSample,immune-Tfh; Homo_633,totalSample,immune-InfiltrationScore; Homo_633,totalSample,immune-Effector_memory; Homo_633,totalSample,immune-CD4_T; Homo_633,totalSample,immune-Bcell; MCSA,totalSample,immune-Neutrophil; MCSA,totalSample,immune-CD4_T; MCSA,totalSample,immune-CD8_T; MCSA,totalSample,immune-Gamma_delta; MCSA,totalSample,immune-CD4_naive; MCSA,totalSample,immune-Tr1; MCSA,totalSample,immune-nTreg; MCSA,totalSample,immune-iTreg; MCSA,totalSample,immune-Th17; MCSA,totalSample,immune-Cytotoxic; MCSA,totalSample,immune-Exhausted; ROSMAP,totalSample,immune-Effector_memory; ROSMAP,male and female-includeAge90+,survival

SOGA3	Homo_723,female,AD.vs.control; Homo_723,male,AD.vs.control; Homo_723,female,AD.vs.MCI; Homo_723,male,AD.vs.MCI; Homo_723,female,MCI.vs.control; Homo_633,female,AD.vs.control; Homo_633,female,AD.vs.MCI; MCSA,MCI,age; Emory,totalSample,immune-Neutrophil; Emory,totalSample,immune-CD4_T; Emory,totalSample,immune-Tr1; Emory,totalSample,immune-nTreg; Emory,totalSample,immune-iTreg; Emory,totalSample,immune-Th17; Emory,totalSample,immune-Tfh; Emory,totalSample,immune-Central_memory; Emory,totalSample,immune-InfiltrationScore; Homo_723,totalSample,immune-Tfh; Homo_723,totalSample,immune-CD4_T; Homo_723,totalSample,immune-CD4_naive; Homo_723,totalSample,immune-Tr1; Homo_723,totalSample,immune-iTreg; Homo_723,totalSample,immune-nTreg; Homo_723,totalSample,immune-Central_memory; Homo_723,totalSample,immune-Th2; Homo_723,totalSample,immune-Effector_memory; Homo_723,totalSample,immune-Th17; Homo_723,totalSample,immune-Th1; Homo_723,totalSample,immune-InfiltrationScore; Homo_723,totalSample,immune-Cytotoxic; Homo_723,totalSample,immune-Exhausted; Homo_723,totalSample,immune-Neutrophil; Homo_723,totalSample,immune-Gamma_delta; Homo_714,totalSample,immune-Tfh; Homo_714,totalSample,immune-CD4_T; Homo_714,totalSample,immune-CD4_naive; Homo_714,totalSample,immune-Tr1; Homo_714,totalSample,immune-iTreg; Homo_714,totalSample,immune-Central_memory; Homo_714,totalSample,immune-nTreg; Homo_714,totalSample,immune-Effector_memory; Homo_714,totalSample,immune-Th2; Homo_714,totalSample,immune-InfiltrationScore; Homo_714,totalSample,immune-Th1; Homo_714,totalSample,immune-Th17; Homo_714,totalSample,immune-Gamma_delta; Homo_714,totalSample,immune-Neutrophil; Homo_714,totalSample,immune-NKT; Homo_633,totalSample,immune-Tfh; Homo_633,totalSample,immune-CD4_T; Homo_633,totalSample,immune-CD4_naive; Homo_633,totalSample,immune-Tr1; Homo_633,totalSample,immune-Central_memory; Homo_633,totalSample,immune-iTreg; Homo_633,totalSample,immune-Effector_memory; Homo_633,totalSample,immune-nTreg; Homo_633,totalSample,immune-Th2; Homo_633,totalSample,immune-NKT; Homo_633,totalSample,immune-Gamma_delta; Homo_633,totalSample,immune-InfiltrationScore; Homo_633,totalSample,immune-Th17; Homo_633,totalSample,immune-Th1; Homo_633,totalSample,immune-Exhausted; MCSA,totalSample,immune-DC; MCSA,totalSample,immune-Bcell; MCSA,totalSample,immune-Macrophage; MCSA,totalSample,immune-CD4_T; MCSA,totalSample,immune-Gamma_delta; MCSA,totalSample,immune-CD4_naive; MCSA,totalSample,immune-Tr1; MCSA,totalSample,immune-nTreg; MCSA,totalSample,immune-iTreg; MCSA,totalSample,immune-Th17; MCSA,totalSample,immune-Tfh; MCSA,totalSample,immune-CD8_naive; MCSA,totalSample,immune-Cytotoxic; MCSA,totalSample,immune-Exhausted; MCSA,totalSample,immune-MAIT; MCSA,totalSample,immune-Central_memory
WDR74	Homo_723,female,AD.vs.control; Homo_723,male,AD.vs.control; Homo_723,female,AD.vs.MCI; Homo_723,male,AD.vs.MCI; Homo_714,female,AD.vs.control; Homo_714,male,AD.vs.control; Homo_714,female,AD.vs.MCI; Homo_714,male,AD.vs.MCI; Homo_633,female,AD.vs.control; Homo_633,female,AD.vs.MCI; ROSMAP,female,AD.vs.control; ROSMAP,male,AD.vs.control; MCSA,totalSample,age; MCSA,control,age; MCSA,MCI,age; Emory,totalSample,immune-B_cell; Emory,totalSample,immune-Monocyte; Emory,totalSample,immune-Macrophage; Emory,totalSample,immune-NK; Emory,totalSample,immune-Neutrophil; Emory,totalSample,immune-CD4_T; Emory,totalSample,immune-CD8_T; Emory,totalSample,immune-Tr1; Emory,totalSample,immune-nTreg; Emory,totalSample,immune-iTreg; Emory,totalSample,immune-Th1; Emory,totalSample,immune-Tfh; Emory,totalSample,immune-CD8_naive; Emory,totalSample,immune-Exhausted; Emory,totalSample,immune-MAIT; Emory,totalSample,immune-Central_memory; Emory,totalSample,immune-InfiltrationScore; Homo_723,totalSample,immune-CD8_T; Homo_723,totalSample,immune-Tfh; Homo_723,totalSample,immune-nTreg; Homo_723,totalSample,immune-iTreg; Homo_723,totalSample,immune-Effector_memory; Homo_723,totalSample,immune-InfiltrationScore; Homo_723,totalSample,immune-CD4_T; Homo_723,totalSample,immune-CD4_naive; Homo_723,totalSample,immune-Macrophage; Homo_723,totalSample,immune-Th2; Homo_723,totalSample,immune-Central_memory; Homo_723,totalSample,immune-Cytotoxic; Homo_723,totalSample,immune-Tr1; Homo_723,totalSample,immune-Exhausted; Homo_723,totalSample,immune-Neutrophil; Homo_723,totalSample,immune-MAIT; Homo_714,totalSample,immune-Tfh; Homo_714,totalSample,immune-nTreg; Homo_714,totalSample,immune-Effector_memory; Homo_714,totalSample,immune-CD8_T; Homo_714,totalSample,immune-InfiltrationScore; Homo_714,totalSample,immune-Th2; Homo_714,totalSample,immune-Central_memory; Homo_714,totalSample,immune-CD4_T; Homo_714,totalSample,immune-Macrophage; Homo_714,totalSample,immune-CD4_naive; Homo_714,totalSample,immune-Neutrophil; Homo_714,totalSample,immune-Tr1; Homo_714,totalSample,immune-iTreg; Homo_714,totalSample,immune-Monocyte; Homo_714,totalSample,immune-MAIT; Homo_714,totalSample,immune-Exhausted; Homo_714,totalSample,immune-DC; Homo_633,totalSample,immune-nTreg; Homo_633,totalSample,immune-Effector_memory; Homo_633,totalSample,immune-InfiltrationScore; Homo_633,totalSample,immune-Tfh; Homo_633,totalSample,immune-Neutrophil; Homo_633,totalSample,immune-Central_memory; Homo_633,totalSample,immune-CD4_T; Homo_633,totalSample,immune-CD4_naive; Homo_633,totalSample,immune-CD8_T; Homo_633,totalSample,immune-Tr1; Homo_633,totalSample,immune-DC; Homo_633,totalSample,immune-Macrophage; Homo_633,totalSample,immune-iTreg; Homo_633,totalSample,immune-Monocyte; MCSA,totalSample,immune-DC; MCSA,totalSample,immune-Monocyte; MCSA,totalSample,immune-Neutrophil; MCSA,totalSample,immune-CD4_T; MCSA,totalSample,immune-CD8_T; MCSA,totalSample,immune-CD4_naive; MCSA,totalSample,immune-Tr1; MCSA,totalSample,immune-nTreg; MCSA,totalSample,immune-Th2; MCSA,totalSample,immune-Th17; MCSA,totalSample,immune-Tfh; MCSA,totalSample,immune-Cytotoxic; MCSA,totalSample,immune-Central_memory; [illegible]
IGFBP2	Homo_723,female,AD.vs.control; Homo_723,male,AD.vs.control; Homo_723,female,AD.vs.MCI; Homo_723,male,AD.vs.MCI; Homo_714,female,AD.vs.control; Homo_714,male,AD.vs.control; Homo_714,female,AD.vs.MCI; Homo_714,male,AD.vs.MCI; Homo_633,female,AD.vs.control; Homo_633,female,AD.vs.MCI; Homo_633,female,MCI.vs.control; Homo_633,male,MCI.vs.control; ROSMAP,female,AD.vs.control; SRP223445,female,AD.vs.control; Emory,totalSample,immune-Macrophage; Emory,totalSample,immune-Neutrophil; Emory,totalSample,immune-CD8_T; Homo_723,totalSample,immune-DC; Homo_723,totalSample,immune-Macrophage; Homo_714,totalSample,immune-DC; Homo_714,totalSample,immune-Th2; Homo_633,totalSample,immune-DC; MCSA,totalSample,immune-DC; MCSA,totalSample,immune-Macrophage; MCSA,totalSample,immune-Th2; ROSMAP,male-excludeAge90+,survival
C11orf98	Homo_723,female,AD.vs.control; Homo_723,male,AD.vs.control; Homo_723,female,AD.vs.MCI; Homo_723,male,AD.vs.MCI; Homo_714,female,AD.vs.control; Homo_714,male,AD.vs.control; Homo_714,female,AD.vs.MCI; Homo_714,male,AD.vs.MCI; Homo_633,female,AD.vs.control; Homo_633,female,AD.vs.MCI; MCSA,MCI,age; ROSMAP,totalSample,last.visit.age; ROSMAP,totalSample,age; ROSMAP,control,last.visit.age; ROSMAP,control,sample.age; ROSMAP,MCI,last.visit.age; Emory,totalSample,immune-Macrophage; Emory,totalSample,immune-Neutrophil; Emory,totalSample,immune-CD4_T; Emory,totalSample,immune-CD8_T; Emory,totalSample,immune-Tr1; Emory,totalSample,immune-iTreg; Emory,totalSample,immune-Th1; Emory,totalSample,immune-Tfh; Emory,totalSample,immune-Exhausted; Emory,totalSample,immune-InfiltrationScore; Homo_723,totalSample,immune-Cytotoxic; Homo_723,totalSample,immune-Monocyte; Homo_723,totalSample,immune-CD8_T; Homo_723,totalSample,immune-Th1; Homo_714,totalSample,immune-Cytotoxic; Homo_714,totalSample,immune-CD8_T; Homo_714,totalSample,immune-NKT; Homo_714,totalSample,immune-Monocyte; Homo_714,totalSample,immune-Th1; Homo_633,totalSample,immune-Monocyte; Homo_633,totalSample,immune-Cytotoxic; Homo_633,totalSample,immune-NKT; Homo_633,totalSample,immune-Th1; Homo_633,totalSample,immune-DC; Homo_633,totalSample,immune-NK; MCSA,totalSample,immune-DC; MCSA,totalSample,immune-CD4_T; MCSA,totalSample,immune-CD8_T; MCSA,totalSample,immune-Gamma_delta; MCSA,totalSample,immune-CD4_naive; MCSA,totalSample,immune-Tr1; MCSA,totalSample,immune-nTreg; MCSA,totalSample,immune-iTreg; MCSA,totalSample,immune-Th17; MCSA,totalSample,immune-Tfh; MCSA,totalSample,immune-CD8_naive; MCSA,totalSample,immune-Cytotoxic; MCSA,totalSample,immune-Exhausted; MCSA,totalSample,immune-Central_memory; ROSMAP,totalSample,immune-Th17; ROSMAP,totalSample,immune-Effector_memory; ROSMAP,totalSample,immune-InfiltrationScore; SRP223445,totalSample,immune-Monocyte; SRP223445,totalSample,immune-NK; SRP223445,totalSample,immune-CD4_naive; SRP223445,totalSample,immune-Exhausted; SRP223445,totalSample,immune-InfiltrationScore; ROSMAP,female-excludeAge90+,survival; ROSMAP,female-includeAge90+,survival
AGAP1	Homo_723,female,AD.vs.control; Homo_723,male,AD.vs.control; Homo_723,female,AD.vs.MCI; Homo_723,male,AD.vs.MCI; Homo_723,female,MCI.vs.control; Homo_723,male,MCI.vs.control; Homo_714,female,AD.vs.control; Homo_714,male,AD.vs.control; Homo_714,female,AD.vs.MCI; Homo_714,male,AD.vs.MCI; Homo_633,female,AD.vs.control; Homo_633,female,AD.vs.MCI; ROSMAP,female,AD.vs.control; Emory,totalSample,immune-Gamma_delta; Emory,totalSample,immune-Cytotoxic; Emory,totalSample,immune-Exhausted; Emory,totalSample,immune-Effector_memory; Emory,totalSample,immune-InfiltrationScore; Homo_723,totalSample,immune-InfiltrationScore; Homo_723,totalSample,immune-NK; Homo_723,totalSample,immune-Neutrophil; Homo_723,totalSample,immune-DC; Homo_723,totalSample,immune-Monocyte; Homo_723,totalSample,immune-Gamma_delta; Homo_723,totalSample,immune-CD4_naive; Homo_714,totalSample,immune-InfiltrationScore; Homo_714,totalSample,immune-Gamma_delta; Homo_714,totalSample,immune-Neutrophil; Homo_714,totalSample,immune-NK; Homo_714,totalSample,immune-Cytotoxic; Homo_714,totalSample,immune-Th2; Homo_714,totalSample,immune-Effector_memory; Homo_633,totalSample,immune-InfiltrationScore; Homo_633,totalSample,immune-Cytotoxic; Homo_633,totalSample,immune-Effector_memory; Homo_633,totalSample,immune-Gamma_delta; Homo_633,totalSample,immune-Macrophage; Homo_633,totalSample,immune-MAIT; Homo_633,totalSample,immune-Neutrophil; Homo_633,totalSample,immune-CD8_T; Homo_633,totalSample,immune-NK; Homo_633,totalSample,immune-Bcell; MCSA,totalSample,immune-Monocyte; MCSA,totalSample,immune-Neutrophil; MCSA,totalSample,immune-CD4_T; MCSA,totalSample,immune-CD8_T; MCSA,totalSample,immune-NKT; MCSA,totalSample,immune-Gamma_delta; MCSA,totalSample,immune-Tr1; MCSA,totalSample,immune-Th17; MCSA,totalSample,immune-Tfh; MCSA,totalSample,immune-Cytotoxic; MCSA,totalSample,immune-MAIT; MCSA,totalSample,immune-Effector_memory; MCSA,totalSample,immune-InfiltrationScore; ROSMAP,totalSample,immune-InfiltrationScore; SRP223445,totalSample,immune-Neutrophil; SRP223445,totalSample,immune-CD4_naive; SRP223445,totalSample,immune-InfiltrationScore
PUS7	Homo_723,female,AD.vs.control; Homo_723,male,AD.vs.control; Homo_723,female,AD.vs.MCI; Homo_723,male,AD.vs.MCI; Homo_723,female,MCI.vs.control; Homo_723,male,MCI.vs.control; Homo_714,female,AD.vs.control; Homo_714,male,AD.vs.control; Homo_714,female,AD.vs.MCI; Homo_714,male,AD.vs.MCI; Homo_714,female,MCI.vs.control; Homo_714,male,MCI.vs.control; Homo_633,female,AD.vs.control; Homo_633,female,AD.vs.MCI; MCSA,MCI,age; Emory,totalSample,immune-Macrophage; Emory,totalSample,immune-Neutrophil; Emory,totalSample,immune-CD4_T; Emory,totalSample,immune-Tr1; Emory,totalSample,immune-iTreg; Emory,totalSample,immune-Central_memory; Emory,totalSample,immune-InfiltrationScore; Homo_723,totalSample,immune-CD4_naive; Homo_723,totalSample,immune-Th17; Homo_723,totalSample,immune-CD4_T; Homo_723,totalSample,immune-Central_memory; Homo_723,totalSample,immune-Tr1; Homo_723,totalSample,immune-Cytotoxic; Homo_723,totalSample,immune-Tfh; Homo_723,totalSample,immune-Th1; Homo_723,totalSample,immune-nTreg; Homo_723,totalSample,immune-iTreg; Homo_723,totalSample,immune-Exhausted; Homo_723,totalSample,immune-Th2; Homo_723,totalSample,immune-InfiltrationScore; Homo_723,totalSample,immune-NKT; Homo_723,totalSample,immune-Neutrophil; Homo_714,totalSample,immune-CD4_naive; Homo_714,totalSample,immune-CD4_T; Homo_714,totalSample,immune-NKT; Homo_714,totalSample,immune-Th17; Homo_714,totalSample,immune-Tfh; Homo_714,totalSample,immune-Central_memory; Homo_714,totalSample,immune-Th1; Homo_714,totalSample,immune-Tr1; Homo_714,totalSample,immune-iTreg; Homo_714,totalSample,immune-InfiltrationScore; Homo_714,totalSample,immune-Exhausted; Homo_714,totalSample,immune-Cytotoxic; Homo_714,totalSample,immune-nTreg; Homo_633,totalSample,immune-Tfh; Homo_633,totalSample,immune-CD4_T; Homo_633,totalSample,immune-CD4_naive; Homo_633,totalSample,immune-Tr1; Homo_633,totalSample,immune-NKT; Homo_633,totalSample,immune-Central_memory; Homo_633,totalSample,immune-iTreg; Homo_633,totalSample,immune-Effector_memory; Homo_633,totalSample,immune-InfiltrationScore; Homo_633,totalSample,immune-Th2; Homo_633,totalSample,immune-nTreg; Homo_633,totalSample,immune-Th1; Homo_633,totalSample,immune-Th17; Homo_633,totalSample,immune-Gamma_delta; Homo_633,totalSample,immune-Bcell; MCSA,totalSample,immune-DC; MCSA,totalSample,immune-Macrophage; MCSA,totalSample,immune-CD4_T; MCSA,totalSample,immune-CD4_naive; MCSA,totalSample,immune-Tr1; MCSA,totalSample,immune-nTreg; MCSA,totalSample,immune-iTreg; MCSA,totalSample,immune-Th17; MCSA,totalSample,immune-Tfh; [illegible]
FLNB	Homo_723,female,AD.vs.control; Homo_723,male,AD.vs.control; Homo_723,female,AD.vs.MCI; Homo_723,female,MCI.vs.control; Homo_723,male,MCI.vs.control; Homo_723,control,male.vs.female; Homo_723,MCI,male.vs.female; Homo_714,female,AD.vs.control; Homo_714,male,AD.vs.control; Homo_714,female,MCI.vs.control; Homo_714,male,MCI.vs.control; Homo_714,control,male.vs.female; Homo_714,MCI,male.vs.female; Homo_633,female,AD.vs.control; Homo_633,female,AD.vs.MCI; Homo_633,female,MCI.vs.control; Homo_633,male,MCI.vs.control; Homo_633,control,male.vs.female; SRP223445,male,AD.vs.control; ACOM,totalSample,age; MCSA,totalSample,age; MCSA,control,age; MCSA,MCI,age; Emory,totalSample,immune-Monocyte; Emory,totalSample,immune-Neutrophil; Emory,totalSample,immune-CD4_T; Emory,totalSample,immune-CD8_T; Emory,totalSample,immune-CD4_naive; Emory,totalSample,immune-Tr1; Emory,totalSample,immune-nTreg; Emory,totalSample,immune-iTreg; Emory,totalSample,immune-MAIT; Emory,totalSample,immune-Central_memory; Emory,totalSample,immune-InfiltrationScore; Homo_723,totalSample,immune-Monocyte; Homo_723,totalSample,immune-Central_memory; Homo_723,totalSample,immune-CD4_naive; Homo_723,totalSample,immune-nTreg; Homo_723,totalSample,immune-DC; Homo_723,totalSample,immune-CD4_T; Homo_723,totalSample,immune-iTreg; Homo_723,totalSample,immune-Tfh; Homo_723,totalSample,immune-Tr1; Homo_723,totalSample,immune-Neutrophil; Homo_723,totalSample,immune-Th1; Homo_714,totalSample,immune-Monocyte; Homo_714,totalSample,immune-CD4_naive; Homo_714,totalSample,immune-nTreg; Homo_714,totalSample,immune-iTreg; Homo_714,totalSample,immune-Central_memory; Homo_714,totalSample,immune-CD4_T; Homo_714,totalSample,immune-Th1; Homo_714,totalSample,immune-Tfh; Homo_714,totalSample,immune-NKT; Homo_714,totalSample,immune-DC; Homo_633,totalSample,immune-Monocyte; Homo_633,totalSample,immune-iTreg; Homo_633,totalSample,immune-CD4_naive; Homo_633,totalSample,immune-nTreg; Homo_633,totalSample,immune-Tfh; Homo_633,totalSample,immune-CD4_T; Homo_633,totalSample,immune-Central_memory; Homo_633,totalSample,immune-Tr1; Homo_633,totalSample,immune-NKT; Homo_633,totalSample,immune-Th1; Homo_633,totalSample,immune-InfiltrationScore; MCSA,totalSample,immune-Monocyte; MCSA,totalSample,immune-Neutrophil; MCSA,totalSample,immune-CD4_T; MCSA,totalSample,immune-Gamma_delta; MCSA,totalSample,immune-CD4_naive; MCSA,totalSample,immune-Tr1; MCSA,totalSample,immune-nTreg; MCSA,totalSample,immune-iTreg; MCSA,totalSample,immune-Th17; MCSA,totalSample,immune-Tfh; MCSA,totalSample,immune-CD8_naive; [illegible]
LCN10	Homo_723,female,AD.vs.control; Homo_723,male,AD.vs.control; Homo_723,female,AD.vs.MCI; Homo_723,male,AD.vs.MCI; Homo_714,female,AD.vs.control; Homo_714,male,AD.vs.control; Homo_714,female,AD.vs.MCI; Homo_714,male,AD.vs.MCI; Homo_633,female,AD.vs.control; Homo_633,female,AD.vs.MCI; ROSMAP,female,AD.vs.control; MCSA,totalSample,age; MCSA,control,age; Emory,totalSample,immune-B_cell; Emory,totalSample,immune-Macrophage; Emory,totalSample,immune-Neutrophil; Emory,totalSample,immune-CD4_T; Emory,totalSample,immune-Tr1; Emory,totalSample,immune-iTreg; Emory,totalSample,immune-Tfh; Emory,totalSample,immune-CD8_naive; Emory,totalSample,immune-InfiltrationScore; Homo_723,totalSample,immune-Tfh; Homo_723,totalSample,immune-iTreg; Homo_723,totalSample,immune-nTreg; Homo_723,totalSample,immune-CD8_T; Homo_723,totalSample,immune-Central_memory; Homo_723,totalSample,immune-CD4_T; Homo_723,totalSample,immune-Macrophage; Homo_723,totalSample,immune-InfiltrationScore; Homo_723,totalSample,immune-CD4_naive; Homo_723,totalSample,immune-Th2; Homo_723,totalSample,immune-Tr1; Homo_723,totalSample,immune-Effector_memory; Homo_714,totalSample,immune-nTreg; Homo_714,totalSample,immune-Tfh; Homo_714,totalSample,immune-CD4_T; Homo_714,totalSample,immune-Macrophage; Homo_714,totalSample,immune-Effector_memory; Homo_714,totalSample,immune-iTreg; Homo_714,totalSample,immune-Central_memory; Homo_714,totalSample,immune-Tr1; Homo_714,totalSample,immune-Th2; Homo_714,totalSample,immune-InfiltrationScore; Homo_714,totalSample,immune-CD4_naive; Homo_714,totalSample,immune-Monocyte; Homo_714,totalSample,immune-Neutrophil; Homo_714,totalSample,immune-CD8_T; Homo_633,totalSample,immune-nTreg; Homo_633,totalSample,immune-Tfh; Homo_633,totalSample,immune-CD4_T; Homo_633,totalSample,immune-Central_memory; Homo_633,totalSample,immune-Neutrophil; Homo_633,totalSample,immune-Tr1; Homo_633,totalSample,immune-CD4_naive; Homo_633,totalSample,immune-Monocyte; Homo_633,totalSample,immune-iTreg; Homo_633,totalSample,immune-InfiltrationScore; Homo_633,totalSample,immune-Effector_memory; Homo_633,totalSample,immune-Macrophage; MCSA,totalSample,immune-Bcell; MCSA,totalSample,immune-Monocyte; MCSA,totalSample,immune-Neutrophil; MCSA,totalSample,immune-CD4_T; MCSA,totalSample,immune-CD8_T; MCSA,totalSample,immune-Gamma_delta; MCSA,totalSample,immune-CD4_naive; MCSA,totalSample,immune-Tr1; MCSA,totalSample,immune-nTreg; MCSA,totalSample,immune-Th17; MCSA,totalSample,immune-Tfh; MCSA,totalSample,immune-CD8_naive; MCSA,totalSample,immune-Cytotoxic; MCSA,totalSample,immune-[illegible]
SLC7A6OS	Homo_723,female,AD.vs.control; Homo_723,male,AD.vs.control; Homo_723,female,AD.vs.MCI; Homo_723,male,AD.vs.MCI; Homo_714,female,AD.vs.control; Homo_714,male,AD.vs.control; Homo_714,female,AD.vs.MCI; Homo_714,male,AD.vs.MCI; Homo_633,female,AD.vs.control; Homo_633,female,AD.vs.MCI; ROSMAP,MCI,last.visit.age; ROSMAP,MCI,sample.age; Emory,totalSample,immune-Macrophage; Emory,totalSample,immune-CD4_T; Emory,totalSample,immune-Tr1; Emory,totalSample,immune-iTreg; Emory,totalSample,immune-Central_memory; Emory,totalSample,immune-InfiltrationScore; Homo_723,totalSample,immune-Tfh; Homo_723,totalSample,immune-Th2; Homo_723,totalSample,immune-Monocyte; Homo_723,totalSample,immune-Effector_memory; Homo_723,totalSample,immune-iTreg; Homo_723,totalSample,immune-InfiltrationScore; Homo_723,totalSample,immune-DC; Homo_723,totalSample,immune-Macrophage; Homo_723,totalSample,immune-Bcell; Homo_714,totalSample,immune-Th2; Homo_714,totalSample,immune-Tfh; Homo_714,totalSample,immune-Effector_memory; Homo_714,totalSample,immune-CD4_T; Homo_633,totalSample,immune-Th2; Homo_633,totalSample,immune-Tfh; Homo_633,totalSample,immune-Monocyte; Homo_633,totalSample,immune-Effector_memory; Homo_633,totalSample,immune-Central_memory; Homo_633,totalSample,immune-CD4_T; MCSA,totalSample,immune-CD4_T; MCSA,totalSample,immune-CD4_naive; MCSA,totalSample,immune-Tr1; MCSA,totalSample,immune-nTreg; MCSA,totalSample,immune-iTreg; MCSA,totalSample,immune-Th17; MCSA,totalSample,immune-Tfh; ROSMAP,totalSample,immune-Effector_memory; SRP223445,totalSample,immune-NK; SRP223445,totalSample,immune-[illegible]
RANBP9	Homo_723,female,AD.vs.control; Homo_723,male,AD.vs.control; Homo_723,female,AD.vs.MCI; Homo_723,male,AD.vs.MCI; Homo_723,female,MCI.vs.control; Homo_723,male,MCI.vs.control; Homo_714,female,AD.vs.control; Homo_714,male,AD.vs.control; Homo_714,female,AD.vs.MCI; Homo_714,male,AD.vs.MCI; Homo_714,female,MCI.vs.control; Homo_714,male,MCI.vs.control; Homo_633,female,AD.vs.control; Homo_633,female,AD.vs.MCI; Homo_633,female,MCI.vs.control; ROSMAP,female,AD.vs.control; SRP223445,female,AD.vs.control; MCSA,MCI,age; ROSMAP,MCI,last.visit.age; Emory,totalSample,immune-Macrophage; Emory,totalSample,immune-CD4_T; Emory,totalSample,immune-Tr1; Emory,totalSample,immune-iTreg; Emory,totalSample,immune-InfiltrationScore; Homo_723,totalSample,immune-Exhausted; Homo_723,totalSample,immune-Th17; Homo_723,totalSample,immune-Monocyte; Homo_723,totalSample,immune-CD8_T; Homo_723,totalSample,immune-Cytotoxic; Homo_723,totalSample,immune-CD4_naive; Homo_723,totalSample,immune-CD4_T; Homo_723,totalSample,immune-Tfh; Homo_714,totalSample,immune-Monocyte; Homo_714,totalSample,immune-Th17; Homo_714,totalSample,immune-Exhausted; Homo_714,totalSample,immune-CD4_naive; Homo_633,totalSample,immune-Monocyte; Homo_633,totalSample,immune-Th2; Homo_633,totalSample,immune-Tfh; Homo_633,totalSample,immune-CD4_T; Homo_633,totalSample,immune-Central_memory; Homo_633,totalSample,immune-Exhausted; Homo_633,totalSample,immune-CD4_naive; Homo_633,totalSample,immune-Th17; Homo_633,totalSample,immune-Gamma_delta; Homo_633,totalSample,immune-Effector_memory; Homo_633,totalSample,immune-InfiltrationScore; Homo_633,totalSample,immune-Bcell; Homo_633,totalSample,immune-Tr1; MCSA,totalSample,immune-DC; MCSA,totalSample,immune-Monocyte; MCSA,totalSample,immune-Macrophage; MCSA,totalSample,immune-CD4_T; MCSA,totalSample,immune-CD8_T; MCSA,totalSample,immune-CD4_naive; MCSA,totalSample,immune-Tr1; MCSA,totalSample,immune-nTreg; MCSA,totalSample,immune-iTreg; MCSA,totalSample,immune-Th2; MCSA,totalSample,immune-Tfh; MCSA,totalSample,immune-CD8_naive; MCSA,totalSample,immune-Cytotoxic; MCSA,totalSample,immune-Exhausted; MCSA,totalSample,immune-MAIT; MCSA,totalSample,immune-Central_memory; SRP223445,totalSample,immune-CD4_naive; SRP223445,totalSample,immune-Exhausted
NCAPH	Homo_723,female,AD.vs.control; Homo_723,male,AD.vs.control; Homo_723,female,AD.vs.MCI; Homo_723,male,AD.vs.MCI; Homo_714,female,AD.vs.control; Homo_714,male,AD.vs.control; Homo_714,female,AD.vs.MCI; Homo_714,male,AD.vs.MCI; Homo_633,female,AD.vs.control; Homo_633,female,AD.vs.MCI; Emory,totalSample,immune-NK; Emory,totalSample,immune-Neutrophil; Emory,totalSample,immune-CD4_naive; Emory,totalSample,immune-Cytotoxic; Emory,totalSample,immune-Exhausted; Emory,totalSample,immune-Effector_memory; Emory,totalSample,immune-InfiltrationScore; Homo_723,totalSample,immune-InfiltrationScore; Homo_723,totalSample,immune-Neutrophil; Homo_723,totalSample,immune-CD4_naive; Homo_723,totalSample,immune-CD4_T; Homo_723,totalSample,immune-Th17; Homo_723,totalSample,immune-Tfh; Homo_723,totalSample,immune-DC; Homo_723,totalSample,immune-NK; Homo_723,totalSample,immune-nTreg; Homo_723,totalSample,immune-Th1; Homo_714,totalSample,immune-InfiltrationScore; Homo_714,totalSample,immune-CD4_naive; Homo_714,totalSample,immune-Neutrophil; Homo_714,totalSample,immune-NK; Homo_633,totalSample,immune-InfiltrationScore; Homo_633,totalSample,immune-Tfh; Homo_633,totalSample,immune-Effector_memory; Homo_633,totalSample,immune-CD4_naive; Homo_633,totalSample,immune-Neutrophil; Homo_633,totalSample,immune-CD4_T; Homo_633,totalSample,immune-MAIT; MCSA,totalSample,immune-Neutrophil; MCSA,totalSample,immune-CD4_T; MCSA,totalSample,immune-CD4_naive; MCSA,totalSample,immune-Tr1; MCSA,totalSample,immune-nTreg; MCSA,totalSample,immune-iTreg; MCSA,totalSample,immune-Tfh; MCSA,totalSample,immune-MAIT;
FOXJ1	Homo_723,female,AD.vs.control; Homo_723,male,AD.vs.control; Homo_723,female,AD.vs.MCI; Homo_723,male,AD.vs.MCI; Homo_723,female,MCI.vs.control; Homo_723,control,male.vs.female; Homo_714,male,AD.vs.control; Homo_714,female,AD.vs.MCI; Homo_714,male,AD.vs.MCI; Homo_714,control,male.vs.female; Homo_633,female,AD.vs.control; Homo_633,female,AD.vs.MCI; Emory,totalSample,age; Emory,MCI,age; MCSA,totalSample,age; MCSA,control,age; MCSA,MCI,age; Emory,totalSample,immune-Macrophage; Emory,totalSample,immune-CD4_T; Emory,totalSample,immune-Tr1; Emory,totalSample,immune-iTreg; Emory,totalSample,immune-Tfh; Homo_723,totalSample,immune-CD8_T; Homo_723,totalSample,immune-Tfh; Homo_723,totalSample,immune-nTreg; Homo_723,totalSample,immune-Macrophage; Homo_723,totalSample,immune-Central_memory; Homo_723,totalSample,immune-iTreg; Homo_723,totalSample,immune-CD4_T; Homo_723,totalSample,immune-CD4_naive; Homo_723,totalSample,immune-Effector_memory; Homo_714,totalSample,immune-Tfh; Homo_714,totalSample,immune-Effector_memory; Homo_714,totalSample,immune-nTreg; Homo_714,totalSample,immune-CD8_T; Homo_714,totalSample,immune-Central_memory; Homo_633,totalSample,immune-nTreg; Homo_633,totalSample,immune-Tfh; Homo_633,totalSample,immune-Central_memory; Homo_633,totalSample,immune-Effector_memory; Homo_633,totalSample,immune-CD4_naive; Homo_633,totalSample,immune-CD4_T; Homo_633,totalSample,immune-DC; Homo_633,totalSample,immune-Neutrophil; MCSA,totalSample,immune-DC; MCSA,totalSample,immune-Monocyte; MCSA,totalSample,immune-Neutrophil; MCSA,totalSample,immune-CD4_T; MCSA,totalSample,immune-CD8_T; MCSA,totalSample,immune-NKT; MCSA,totalSample,immune-CD4_naive; MCSA,totalSample,immune-Tr1; MCSA,totalSample,immune-nTreg; MCSA,totalSample,immune-Th2; MCSA,totalSample,immune-Tfh; MCSA,totalSample,immune-Cytotoxic; MCSA,totalSample,immune-Central_memory; MCSA,totalSample,immune-[illegible]

LRP6	Homo_723,female,AD.vs.control; Homo_723,male,AD.vs.control; Homo_723,female,AD.vs.MCI; Homo_723,male,AD.vs.MCI; Homo_723,female,MCI.vs.control; Homo_723,male,MCI.vs.control; Homo_714,female,AD.vs.control; Homo_714,male,AD.vs.control; Homo_714,female,AD.vs.MCI; Homo_714,male,AD.vs.MCI; Homo_714,female,MCI.vs.control; Homo_714,male,MCI.vs.control; Homo_633,female,AD.vs.control; Homo_633,female,AD.vs.MCI; MCSA,MCI,age; Emory,totalSample,immune-CD4_T; Emory,totalSample,immune-Gamma_delta; Emory,totalSample,immune-CD4_naive; Emory,totalSample,immune-Tr1; Emory,totalSample,immune-iTreg; Emory,totalSample,immune-Th17; Emory,totalSample,immune-Cytotoxic; Emory,totalSample,immune-Central_memory; Homo_723,totalSample,immune-CD4_naive; Homo_723,totalSample,immune-Th17; Homo_723,totalSample,immune-Cytotoxic; Homo_723,totalSample,immune-CD4_T; Homo_723,totalSample,immune-Central_memory; Homo_723,totalSample,immune-Tr1; Homo_723,totalSample,immune-Tfh; Homo_723,totalSample,immune-nTreg; Homo_723,totalSample,immune-Exhausted; Homo_723,totalSample,immune-Th1; Homo_723,totalSample,immune-Th2; Homo_723,totalSample,immune-iTreg; Homo_723,totalSample,immune-Gamma_delta; Homo_714,totalSample,immune-CD4_naive; Homo_714,totalSample,immune-Th17; Homo_714,totalSample,immune-CD4_T; Homo_714,totalSample,immune-Central_memory; Homo_714,totalSample,immune-Tfh; Homo_714,totalSample,immune-Exhausted; Homo_714,totalSample,immune-Tr1; Homo_714,totalSample,immune-Cytotoxic; Homo_714,totalSample,immune-Th1; Homo_714,totalSample,immune-iTreg; Homo_714,totalSample,immune-Gamma_delta; Homo_714,totalSample,immune-nTreg; Homo_714,totalSample,immune-Th2; Homo_714,totalSample,immune-NKT; Homo_714,totalSample,immune-CD8_T; Homo_633,totalSample,immune-Tfh; Homo_633,totalSample,immune-CD4_T; Homo_633,totalSample,immune-Tr1; Homo_633,totalSample,immune-CD4_naive; Homo_633,totalSample,immune-Central_memory; Homo_633,totalSample,immune-iTreg; Homo_633,totalSample,immune-Th2; Homo_633,totalSample,immune-Gamma_delta; Homo_633,totalSample,immune-nTreg; Homo_633,totalSample,immune-Th17; Homo_633,totalSample,immune-Cytotoxic; Homo_633,totalSample,immune-Exhausted; Homo_633,totalSample,immune-Effector_memory; Homo_633,totalSample,immune-NKT; Homo_633,totalSample,immune-DC; Homo_633,totalSample,immune-Th1; MCSA,totalSample,immune-DC; MCSA,totalSample,immune-Macrophage; MCSA,totalSample,immune-CD4_T; MCSA,totalSample,immune-Gamma_delta; MCSA,totalSample,immune-CD4_naive; MCSA,totalSample,immune-Tr1; MCSA,totalSample,immune-nTreg; MCSA,totalSample,immune-iTreg; MCSA,totalSample,immune-Th17; MCSA,totalSample,immune-Tfh; MCSA,totalSample,immune-CD8_naive; MCSA,totalSample,immune-Cytotoxic; MCSA,totalSample,immune-Exhausted; MCSA,totalSample,immune-MAIT; MCSA,totalSample,immune-Central_memory; ROSMAP,totalSample,immune-InfiltrationScore
FASLG	Homo_723,female,AD.vs.control; Homo_723,male,AD.vs.control; Homo_723,female,AD.vs.MCI; Homo_723,male,AD.vs.MCI; Homo_723,female,MCI.vs.control; Homo_714,female,AD.vs.control; Homo_714,male,AD.vs.control; Homo_714,female,AD.vs.MCI; Homo_714,male,AD.vs.MCI; Homo_714,female,MCI.vs.control; Homo_633,female,AD.vs.control; Homo_633,female,AD.vs.MCI; Homo_633,female,MCI.vs.control; ROSMAP,female,AD.vs.control; SRP223445,female,AD.vs.control; MCSA,MCI,age; ROSMAP,MCI,last.visit.age; Emory,totalSample,immune-Macrophage; Emory,totalSample,immune-NK; Emory,totalSample,immune-Neutrophil; Emory,totalSample,immune-CD8_T; Emory,totalSample,immune-Gamma_delta; Emory,totalSample,immune-CD4_naive; Emory,totalSample,immune-Tr1; Emory,totalSample,immune-nTreg; Emory,totalSample,immune-iTreg; Emory,totalSample,immune-Th17; Emory,totalSample,immune-Cytotoxic; Emory,totalSample,immune-Exhausted; Emory,totalSample,immune-Effector_memory; Emory,totalSample,immune-InfiltrationScore; Homo_723,totalSample,immune-InfiltrationScore; Homo_723,totalSample,immune-Exhausted; Homo_723,totalSample,immune-MAIT; Homo_723,totalSample,immune-Th17; Homo_723,totalSample,immune-Cytotoxic; Homo_723,totalSample,immune-NK; Homo_723,totalSample,immune-Macrophage; Homo_723,totalSample,immune-Effector_memory; Homo_723,totalSample,immune-Monocyte; Homo_723,totalSample,immune-CD8_T; Homo_723,totalSample,immune-Bcell; Homo_723,totalSample,immune-Gamma_delta; Homo_714,totalSample,immune-InfiltrationScore; Homo_714,totalSample,immune-Cytotoxic; Homo_714,totalSample,immune-Th17; Homo_714,totalSample,immune-Effector_memory; Homo_714,totalSample,immune-Gamma_delta; Homo_714,totalSample,immune-MAIT; Homo_714,totalSample,immune-Macrophage; Homo_714,totalSample,immune-NK; Homo_714,totalSample,immune-Neutrophil; Homo_714,totalSample,immune-Exhausted; Homo_714,totalSample,immune-CD8_T; Homo_714,totalSample,immune-Bcell; Homo_633,totalSample,immune-InfiltrationScore; Homo_633,totalSample,immune-NK; Homo_633,totalSample,immune-Gamma_delta; Homo_633,totalSample,immune-Cytotoxic; Homo_633,totalSample,immune-Neutrophil; Homo_633,totalSample,immune-MAIT; Homo_633,totalSample,immune-Th17; Homo_633,totalSample,immune-Bcell; Homo_633,totalSample,immune-Th2; Homo_633,totalSample,immune-Effector_memory; Homo_633,totalSample,immune-Macrophage; Homo_633,totalSample,immune-Exhausted; Homo_633,totalSample,immune-CD8_T; MCSA,totalSample,immune-DC; MCSA,totalSample,immune-Macrophage; MCSA,totalSample,immune-NK; MCSA,totalSample,immune-Gamma_delta; MCSA,totalSample,immune-iTreg; [illegible]
KLKB1	Homo_723,female,AD.vs.control; Homo_723,male,AD.vs.control; Homo_723,female,AD.vs.MCI; Homo_723,male,AD.vs.MCI; Homo_723,female,MCI.vs.control; Homo_723,male,MCI.vs.control; Homo_714,female,AD.vs.control; Homo_714,male,AD.vs.control; Homo_714,female,AD.vs.MCI; Homo_714,male,AD.vs.MCI; Homo_714,female,MCI.vs.control; Homo_714,male,MCI.vs.control; Homo_633,female,AD.vs.control; Homo_633,female,AD.vs.MCI; Homo_633,female,MCI.vs.control; Homo_633,male,MCI.vs.control; ROSMAP,female,AD.vs.control; ROSMAP,control,male.vs.female; MCSA,totalSample,age; MCSA,control,age; Homo_723,totalSample,immune-Th2; Homo_723,totalSample,immune-Tfh; Homo_723,totalSample,immune-iTreg; Homo_723,totalSample,immune-CD4_T; Homo_723,totalSample,immune-DC; Homo_723,totalSample,immune-Monocyte; Homo_723,totalSample,immune-Effector_memory; Homo_723,totalSample,immune-Tr1; Homo_723,totalSample,immune-Macrophage; Homo_723,totalSample,immune-CD4_naive; Homo_714,totalSample,immune-Th2; Homo_714,totalSample,immune-Tfh; Homo_714,totalSample,immune-CD4_T; Homo_714,totalSample,immune-Effector_memory; Homo_714,totalSample,immune-Central_memory; Homo_714,totalSample,immune-Tr1; Homo_714,totalSample,immune-CD4_naive; Homo_714,totalSample,immune-nTreg; Homo_633,totalSample,immune-Th2; Homo_633,totalSample,immune-Tfh; Homo_633,totalSample,immune-CD4_T; Homo_633,totalSample,immune-Central_memory; Homo_633,totalSample,immune-Effector_memory; Homo_633,totalSample,immune-CD4_naive; Homo_633,totalSample,immune-Th17; Homo_633,totalSample,immune-nTreg; Homo_633,totalSample,immune-Monocyte; Homo_633,totalSample,immune-Tr1; MCSA,totalSample,immune-Monocyte; MCSA,totalSample,immune-Macrophage; MCSA,totalSample,immune-Neutrophil; MCSA,totalSample,immune-CD4_T; MCSA,totalSample,immune-CD4_naive; MCSA,totalSample,immune-Tr1; MCSA,totalSample,immune-nTreg; MCSA,totalSample,immune-iTreg; MCSA,totalSample,immune-Th17; MCSA,totalSample,immune-Tfh; MCSA,totalSample,immune-CD8_naive; MCSA,totalSample,immune-MAIT; MCSA,totalSample,immune-Central_memory; MCSA,totalSample,immune-Effector_memory; ROSMAP,totalSample,immune-Th17; ROSMAP,totalSample,immune-InfiltrationScore
BRIP1	Homo_723,female,AD.vs.control; Homo_723,male,AD.vs.control; Homo_723,female,AD.vs.MCI; Homo_723,male,AD.vs.MCI; Homo_723,female,MCI.vs.control; Homo_723,male,MCI.vs.control; Homo_714,female,AD.vs.control; Homo_714,male,AD.vs.control; Homo_714,female,AD.vs.MCI; Homo_714,male,AD.vs.MCI; Homo_714,female,MCI.vs.control; Homo_714,male,MCI.vs.control; Homo_633,female,AD.vs.control; Homo_633,female,AD.vs.MCI; MCSA,MCI,age; ROSMAP,MCI,last.visit.age; Emory,totalSample,immune-Tr1; Homo_723,totalSample,immune-CD4_naive; Homo_723,totalSample,immune-Cytotoxic; Homo_723,totalSample,immune-Th17; Homo_723,totalSample,immune-CD4_T; Homo_723,totalSample,immune-Th1; Homo_723,totalSample,immune-Tr1; Homo_723,totalSample,immune-Exhausted; Homo_723,totalSample,immune-Central_memory; Homo_723,totalSample,immune-Tfh; Homo_723,totalSample,immune-Neutrophil; Homo_723,totalSample,immune-nTreg; Homo_723,totalSample,immune-CD8_T; Homo_723,totalSample,immune-InfiltrationScore; Homo_723,totalSample,immune-Th2; Homo_714,totalSample,immune-CD4_naive; Homo_714,totalSample,immune-CD4_T; Homo_714,totalSample,immune-Tfh; Homo_714,totalSample,immune-Central_memory; Homo_714,totalSample,immune-Th1; Homo_714,totalSample,immune-Th17; Homo_714,totalSample,immune-InfiltrationScore; Homo_714,totalSample,immune-NKT; Homo_714,totalSample,immune-Tr1; Homo_714,totalSample,immune-Exhausted; Homo_633,totalSample,immune-Tfh; Homo_633,totalSample,immune-CD4_naive; Homo_633,totalSample,immune-CD4_T; Homo_633,totalSample,immune-Central_memory; Homo_633,totalSample,immune-Tr1; Homo_633,totalSample,immune-Effector_memory; Homo_633,totalSample,immune-InfiltrationScore; Homo_633,totalSample,immune-Th2; Homo_633,totalSample,immune-iTreg; Homo_633,totalSample,immune-NKT; Homo_633,totalSample,immune-Gamma_delta; MCSA,totalSample,immune-DC; MCSA,totalSample,immune-Macrophage; MCSA,totalSample,immune-CD4_T; MCSA,totalSample,immune-CD4_naive; MCSA,totalSample,immune-Tr1; MCSA,totalSample,immune-nTreg; MCSA,totalSample,immune-iTreg; MCSA,totalSample,immune-Tfh; MCSA,totalSample,immune-CD8_naive; MCSA,totalSample,immune-Cytotoxic; MCSA,totalSample,immune-Exhausted; MCSA,totalSample,immune-MAIT; MCSA,totalSample,immune-Central_memory; [illegible]
PF4	Homo_723,female,AD.vs.control; Homo_723,male,AD.vs.control; Homo_723,female,AD.vs.MCI; Homo_723,male,AD.vs.MCI; Homo_723,female,MCI.vs.control; Homo_723,male,MCI.vs.control; Homo_723,control,male.vs.female; SRP223445,female,AD.vs.control; MCSA,MCI,age; Emory,totalSample,immune-Macrophage; Emory,totalSample,immune-NK; Emory,totalSample,immune-Neutrophil; Emory,totalSample,immune-Exhausted; Emory,totalSample,immune-InfiltrationScore; Homo_723,totalSample,immune-DC; Homo_723,totalSample,immune-Th2; Homo_723,totalSample,immune-Monocyte; Homo_723,totalSample,immune-Macrophage; Homo_723,totalSample,immune-Tfh; Homo_714,totalSample,immune-DC; Homo_633,totalSample,immune-DC; Homo_633,totalSample,immune-Th1; MCSA,totalSample,immune-DC; MCSA,totalSample,immune-Monocyte; MCSA,totalSample,immune-Macrophage; MCSA,totalSample,immune-CD8_T; MCSA,totalSample,immune-NKT; MCSA,totalSample,immune-iTreg; MCSA,totalSample,immune-Th2; MCSA,totalSample,immune-Cytotoxic; MCSA,totalSample,immune-Effector_memory; SRP223445,totalSample,immune-CD4_naive; SRP223445,totalSample,immune-Exhausted; SRP223445,totalSample,immune-InfiltrationScore; ROSMAP,female-excludeAge90+,survival; ROSMAP,male-excludeAge90+,survival
PLXNA4	Homo_723,female,AD.vs.control; Homo_723,male,AD.vs.control; Homo_723,female,AD.vs.MCI; Homo_723,male,AD.vs.MCI; Homo_714,female,AD.vs.control; Homo_714,male,AD.vs.control; Homo_714,female,AD.vs.MCI; Homo_714,male,AD.vs.MCI; Homo_633,female,AD.vs.control; Homo_633,female,AD.vs.MCI; MCSA,totalSample,age; MCSA,control,age; MCSA,MCI,age; Emory,totalSample,immune-CD4_T; Emory,totalSample,immune-Gamma_delta; Emory,totalSample,immune-Tr1; Emory,totalSample,immune-iTreg; Emory,totalSample,immune-Th17; Emory,totalSample,immune-Cytotoxic; Emory,totalSample,immune-InfiltrationScore; Homo_723,totalSample,immune-Th17; Homo_723,totalSample,immune-CD4_naive; Homo_723,totalSample,immune-nTreg; Homo_723,totalSample,immune-Cytotoxic; Homo_723,totalSample,immune-Central_memory; Homo_723,totalSample,immune-CD4_T; Homo_723,totalSample,immune-Exhausted; Homo_723,totalSample,immune-DC; Homo_723,totalSample,immune-Tr1; Homo_723,totalSample,immune-InfiltrationScore; Homo_723,totalSample,immune-MAIT; Homo_714,totalSample,immune-Th17; Homo_714,totalSample,immune-Exhausted; Homo_714,totalSample,immune-CD4_naive; Homo_714,totalSample,immune-Cytotoxic; Homo_714,totalSample,immune-nTreg; Homo_714,totalSample,immune-CD8_T; Homo_714,totalSample,immune-InfiltrationScore; Homo_633,totalSample,immune-Tr1; Homo_633,totalSample,immune-Tfh; Homo_633,totalSample,immune-CD4_T; Homo_633,totalSample,immune-nTreg; Homo_633,totalSample,immune-Th2; Homo_633,totalSample,immune-Gamma_delta; Homo_633,totalSample,immune-iTreg; Homo_633,totalSample,immune-InfiltrationScore; Homo_633,totalSample,immune-Exhausted; Homo_633,totalSample,immune-CD4_naive; Homo_633,totalSample,immune-Th17; Homo_633,totalSample,immune-Cytotoxic; Homo_633,totalSample,immune-Central_memory; Homo_633,totalSample,immune-Effector_memory; MCSA,totalSample,immune-Monocyte; MCSA,totalSample,immune-CD4_T; MCSA,totalSample,immune-Gamma_delta; MCSA,totalSample,immune-CD4_naive; MCSA,totalSample,immune-Tr1; MCSA,totalSample,immune-nTreg; MCSA,totalSample,immune-iTreg; MCSA,totalSample,immune-Th1; MCSA,totalSample,immune-Th17; MCSA,totalSample,immune-Tfh; MCSA,totalSample,immune-CD8_naive; MCSA,totalSample,immune-Exhausted; MCSA,totalSample,immune-Central_memory; [illegible]
RTN2	Homo_723,female,AD.vs.control; Homo_723,male,AD.vs.control; Homo_723,female,AD.vs.MCI; Homo_723,male,AD.vs.MCI; Homo_714,female,AD.vs.control; Homo_714,male,AD.vs.control; Homo_714,female,AD.vs.MCI; Homo_714,male,AD.vs.MCI; Homo_633,female,AD.vs.control; Homo_633,female,AD.vs.MCI; Homo_633,female,MCI.vs.control; ROSMAP,male,AD.vs.control; ROSMAP,AD,male.vs.female; MCSA,MCI,age; ROSMAP,AD,last.visit.age; Emory,totalSample,immune-Neutrophil; Emory,totalSample,immune-CD8_T; Emory,totalSample,immune-Gamma_delta; Emory,totalSample,immune-Th1; Emory,totalSample,immune-Th17; Emory,totalSample,immune-Tfh; Emory,totalSample,immune-Cytotoxic; Emory,totalSample,immune-Exhausted; Emory,totalSample,immune-Effector_memory; Homo_723,totalSample,immune-DC; Homo_723,totalSample,immune-Th2; Homo_723,totalSample,immune-Monocyte; Homo_723,totalSample,immune-Macrophage; Homo_723,totalSample,immune-iTreg; Homo_714,totalSample,immune-Th2; Homo_714,totalSample,immune-DC; Homo_714,totalSample,immune-Tfh; Homo_633,totalSample,immune-DC; Homo_633,totalSample,immune-Th2; MCSA,totalSample,immune-DC; MCSA,totalSample,immune-Monocyte; MCSA,totalSample,immune-Macrophage; MCSA,totalSample,immune-CD4_T; MCSA,totalSample,immune-NKT; MCSA,totalSample,immune-Tr1; MCSA,totalSample,immune-iTreg; MCSA,totalSample,immune-Th2; MCSA,totalSample,immune-Cytotoxic; SRP223445,totalSample,immune-CD4_naive; SRP223445,totalSample,immune-Exhausted; ROSMAP,male and female-excludeAge90+,survival
ROCK2	Homo_723,female,AD.vs.control; Homo_723,male,AD.vs.control; Homo_723,female,AD.vs.MCI; Homo_723,male,AD.vs.MCI; Homo_723,female,MCI.vs.control; Homo_714,female,AD.vs.control; Homo_714,male,AD.vs.control; Homo_714,female,AD.vs.MCI; Homo_714,male,AD.vs.MCI; Homo_714,female,MCI.vs.control; Homo_633,female,AD.vs.control; Homo_633,female,AD.vs.MCI; ROSMAP,female,MCI.vs.control; Emory,MCI,age; MCSA,MCI,age; ROSMAP,AD,last.visit.age; Emory,totalSample,immune-CD4_T; Emory,totalSample,immune-Gamma_delta; Emory,totalSample,immune-Tr1; Emory,totalSample,immune-iTreg; Emory,totalSample,immune-Central_memory; Emory,totalSample,immune-InfiltrationScore; Homo_723,totalSample,immune-Cytotoxic; Homo_723,totalSample,immune-CD4_naive; Homo_723,totalSample,immune-Central_memory; Homo_723,totalSample,immune-Th17; Homo_723,totalSample,immune-Th1; Homo_723,totalSample,immune-Exhausted; Homo_723,totalSample,immune-CD4_T; Homo_723,totalSample,immune-NKT; Homo_723,totalSample,immune-CD8_T; Homo_723,totalSample,immune-Tr1; Homo_723,totalSample,immune-Gamma_delta; Homo_723,totalSample,immune-Tfh; Homo_723,totalSample,immune-InfiltrationScore; Homo_714,totalSample,immune-NKT; Homo_714,totalSample,immune-CD4_naive; Homo_714,totalSample,immune-Exhausted; Homo_714,totalSample,immune-Cytotoxic; Homo_714,totalSample,immune-CD8_T; Homo_714,totalSample,immune-Th1; Homo_714,totalSample,immune-Th17; Homo_714,totalSample,immune-CD4_T; Homo_714,totalSample,immune-Central_memory; Homo_714,totalSample,immune-InfiltrationScore; Homo_714,totalSample,immune-iTreg; Homo_714,totalSample,immune-Tfh; Homo_714,totalSample,immune-Tr1; Homo_714,totalSample,immune-Gamma_delta; Homo_633,totalSample,immune-Tfh; Homo_633,totalSample,immune-NKT; Homo_633,totalSample,immune-CD4_naive; Homo_633,totalSample,immune-CD4_T; Homo_633,totalSample,immune-Tr1; Homo_633,totalSample,immune-iTreg; Homo_633,totalSample,immune-Central_memory; Homo_633,totalSample,immune-InfiltrationScore; Homo_633,totalSample,immune-Gamma_delta; Homo_633,totalSample,immune-Effector_memory; Homo_633,totalSample,immune-Cytotoxic; Homo_633,totalSample,immune-Exhausted; Homo_633,totalSample,immune-Th1; Homo_633,totalSample,immune-DC; MCSA,totalSample,immune-DC; MCSA,totalSample,immune-Monocyte; MCSA,totalSample,immune-CD4_T; MCSA,totalSample,immune-CD8_T; MCSA,totalSample,immune-Gamma_delta; MCSA,totalSample,immune-CD4_naive; MCSA,totalSample,immune-Tr1; MCSA,totalSample,immune-nTreg; MCSA,totalSample,immune-iTreg; MCSA,totalSample,immune-Th17; MCSA,totalSample,immune-Tfh; MCSA,totalSample,immune-CD8_naive; MCSA,totalSample,immune-Cytotoxic; MCSA,totalSample,immune-Exhausted; MCSA,totalSample,immune-MAIT; MCSA,totalSample,immune-Central_memory; ROSMAP,totalSample,immune-nTreg; ROSMAP,totalSample,immune-iTreg; ROSMAP,totalSample,immune-Th1; ROSMAP,totalSample,immune-Th17; ROSMAP,totalSample,immune-Effector_memory; [illegible]
PHGDH	Homo_723,female,AD.vs.control; Homo_723,male,AD.vs.control; Homo_723,female,AD.vs.MCI; Homo_723,male,AD.vs.MCI; Homo_714,female,AD.vs.control; Homo_714,female,AD.vs.MCI; Homo_633,female,AD.vs.control; Homo_633,female,AD.vs.MCI; MCSA,totalSample,age; MCSA,control,age; MCSA,MCI,age; Emory,totalSample,immune-Monocyte; Emory,totalSample,immune-Neutrophil; Emory,totalSample,immune-CD4_T; Emory,totalSample,immune-CD8_T; Emory,totalSample,immune-Tr1; Emory,totalSample,immune-nTreg; Emory,totalSample,immune-iTreg; Emory,totalSample,immune-Tfh; Emory,totalSample,immune-CD8_naive; Emory,totalSample,immune-MAIT; Emory,totalSample,immune-Central_memory; Homo_723,totalSample,immune-Central_memory; Homo_723,totalSample,immune-nTreg; Homo_723,totalSample,immune-CD8_T; Homo_723,totalSample,immune-DC; Homo_723,totalSample,immune-Tfh; Homo_723,totalSample,immune-CD4_naive; Homo_723,totalSample,immune-CD4_T; Homo_723,totalSample,immune-Monocyte; Homo_723,totalSample,immune-iTreg; Homo_723,totalSample,immune-Neutrophil; Homo_723,totalSample,immune-Th2; Homo_714,totalSample,immune-nTreg; Homo_714,totalSample,immune-Central_memory; Homo_714,totalSample,immune-Tfh; Homo_714,totalSample,immune-Monocyte; Homo_714,totalSample,immune-DC; Homo_714,totalSample,immune-CD4_T; Homo_714,totalSample,immune-CD4_naive; Homo_633,totalSample,immune-nTreg; Homo_633,totalSample,immune-Central_memory; Homo_633,totalSample,immune-Tfh; Homo_633,totalSample,immune-DC; Homo_633,totalSample,immune-Monocyte; Homo_633,totalSample,immune-CD4_T; Homo_633,totalSample,immune-CD4_naive; Homo_633,totalSample,immune-Tr1; Homo_633,totalSample,immune-Effector_memory; Homo_633,totalSample,immune-Neutrophil; MCSA,totalSample,immune-DC; MCSA,totalSample,immune-Monocyte; MCSA,totalSample,immune-Neutrophil; MCSA,totalSample,immune-CD4_T; MCSA,totalSample,immune-CD8_T; MCSA,totalSample,immune-CD4_naive; MCSA,totalSample,immune-nTreg; MCSA,totalSample,immune-Th2; MCSA,totalSample,immune-Tfh; MCSA,totalSample,immune-Cytotoxic; MCSA,totalSample,immune-Central_memory; MCSA,totalSample,immune-Effector_memory; MCSA,totalSample,immune-InfiltrationScore; ROSMAP,totalSample,immune-InfiltrationScore; ROSMAP,male-includeAge90+,survival
SLC39A11	Homo_723,female,AD.vs.control; Homo_723,male,AD.vs.control; Homo_723,female,AD.vs.MCI; Homo_723,male,AD.vs.MCI; Homo_714,female,AD.vs.control; Homo_714,male,AD.vs.control; Homo_714,female,AD.vs.MCI; Homo_714,male,AD.vs.MCI; Homo_633,female,AD.vs.control; Homo_633,female,AD.vs.MCI; Emory,MCI,age; MCSA,MCI,age; Emory,totalSample,immune-Neutrophil; Emory,totalSample,immune-Tr1; Emory,totalSample,immune-iTreg; Emory,totalSample,immune-Effector_memory; Emory,totalSample,immune-InfiltrationScore; Homo_723,totalSample,immune-Th2; Homo_723,totalSample,immune-InfiltrationScore; Homo_723,totalSample,immune-Effector_memory; Homo_723,totalSample,immune-Tfh; Homo_723,totalSample,immune-iTreg; Homo_723,totalSample,immune-Monocyte; Homo_723,totalSample,immune-Bcell; Homo_723,totalSample,immune-CD4_T; Homo_723,totalSample,immune-Tr1; Homo_723,totalSample,immune-CD4_naive; Homo_714,totalSample,immune-Th2; Homo_714,totalSample,immune-Tfh; Homo_714,totalSample,immune-Effector_memory; Homo_714,totalSample,immune-InfiltrationScore; Homo_714,totalSample,immune-Tr1; Homo_714,totalSample,immune-CD4_T; Homo_633,totalSample,immune-InfiltrationScore; Homo_633,totalSample,immune-Effector_memory; Homo_633,totalSample,immune-Th2; Homo_633,totalSample,immune-Tfh; Homo_633,totalSample,immune-Exhausted; Homo_633,totalSample,immune-Monocyte; Homo_633,totalSample,immune-CD4_naive; Homo_633,totalSample,immune-CD4_T; Homo_633,totalSample,immune-Central_memory; Homo_633,totalSample,immune-Bcell; Homo_633,totalSample,immune-Gamma_delta; MCSA,totalSample,immune-CD4_T; MCSA,totalSample,immune-NKT; MCSA,totalSample,immune-CD4_naive; MCSA,totalSample,immune-Tr1; MCSA,totalSample,immune-nTreg; MCSA,totalSample,immune-iTreg; MCSA,totalSample,immune-Tfh; MCSA,totalSample,immune-Exhausted; MCSA,totalSample,immune-InfiltrationScore; ROSMAP,totalSample,immune-MAIT; ROSMAP,totalSample,immune-Effector_memory; SRP223445,totalSample,immune-Exhausted
ASL	Homo_723,female,AD.vs.control; Homo_723,male,AD.vs.control; Homo_723,female,AD.vs.MCI; Homo_723,male,AD.vs.MCI; Homo_714,female,AD.vs.control; Homo_714,male,AD.vs.control; Homo_714,female,AD.vs.MCI; Homo_714,male,AD.vs.MCI; Homo_633,female,AD.vs.control; Homo_633,female,AD.vs.MCI; Emory,totalSample,age; Emory,MCI,age; MCSA,MCI,age; ROSMAP,MCI,last.visit.age; Emory,totalSample,immune-CD8_naive; Emory,totalSample,immune-InfiltrationScore; Homo_723,totalSample,immune-Cytotoxic; Homo_723,totalSample,immune-InfiltrationScore; Homo_723,totalSample,immune-CD8_T; Homo_723,totalSample,immune-Effector_memory; Homo_723,totalSample,immune-Th1; Homo_723,totalSample,immune-DC; Homo_723,totalSample,immune-Bcell; Homo_714,totalSample,immune-DC; Homo_714,totalSample,immune-InfiltrationScore; Homo_714,totalSample,immune-Th1; Homo_714,totalSample,immune-Effector_memory; Homo_714,totalSample,immune-CD8_T; Homo_714,totalSample,immune-Cytotoxic; Homo_633,totalSample,immune-DC; Homo_633,totalSample,immune-InfiltrationScore; Homo_633,totalSample,immune-Th1; Homo_633,totalSample,immune-NKT; Homo_633,totalSample,immune-Effector_memory; MCSA,totalSample,immune-DC; MCSA,totalSample,immune-Monocyte; MCSA,totalSample,immune-Macrophage; MCSA,totalSample,immune-Neutrophil; MCSA,totalSample,immune-CD8_T; MCSA,totalSample,immune-NKT; MCSA,totalSample,immune-iTreg; MCSA,totalSample,immune-Th2; MCSA,totalSample,immune-Cytotoxic; MCSA,totalSample,immune-Effector_memory; MCSA,totalSample,immune-InfiltrationScore; ROSMAP,totalSample,immune-Th17; ROSMAP,totalSample,immune-Effector_memory; ROSMAP,totalSample,immune-InfiltrationScore; SRP223445,totalSample,immune-NK; SRP223445,totalSample,immune-CD4_naive; SRP223445,totalSample,immune-Exhausted; SRP223445,totalSample,immune-InfiltrationScore; ROSMAP,male-excludeAge90+,survival
KCNJ13	Homo_723,female,AD.vs.control; Homo_723,male,AD.vs.control; Homo_723,female,AD.vs.MCI; Homo_723,male,AD.vs.MCI; Homo_723,female,MCI.vs.control; Homo_723,male,MCI.vs.control; Homo_714,female,MCI.vs.control; Homo_714,male,MCI.vs.control; Homo_633,female,AD.vs.control; Homo_633,female,AD.vs.MCI; Homo_633,female,MCI.vs.control; Homo_633,male,MCI.vs.control; Emory,totalSample,immune-CD4_T; Emory,totalSample,immune-Tr1; Emory,totalSample,immune-iTreg; Emory,totalSample,immune-Central_memory; Homo_723,totalSample,immune-Monocyte; Homo_723,totalSample,immune-Central_memory; Homo_723,totalSample,immune-Exhausted; Homo_723,totalSample,immune-NKT; Homo_723,totalSample,immune-CD8_naive; Homo_723,totalSample,immune-Th1; Homo_714,totalSample,immune-Monocyte; Homo_714,totalSample,immune-NKT; Homo_633,totalSample,immune-NKT; Homo_633,totalSample,immune-Monocyte; Homo_633,totalSample,immune-Th1; MCSA,totalSample,immune-CD4_T; MCSA,totalSample,immune-Gamma_delta; MCSA,totalSample,immune-CD4_naive; MCSA,totalSample,immune-Tr1; MCSA,totalSample,immune-nTreg; MCSA,totalSample,immune-iTreg; MCSA,totalSample,immune-Th17; MCSA,totalSample,immune-Tfh; MCSA,totalSample,immune-CD8_naive; MCSA,totalSample,immune-Cytotoxic; MCSA,totalSample,immune-Exhausted; MCSA,totalSample,immune-

MS4A1	Homo_723,female,AD.vs.control; Homo_723,male,AD.vs.control; Homo_723,female,AD.vs.MCI; Homo_723,male,AD.vs.MCI; Homo_633,female,AD.vs.control; Homo_633,female,AD.vs.MCI; ROSMAP,female,AD.vs.control; ROSMAP,female,MCI.vs.control; MCSA,totalSample,age; MCSA,control,age; ROSMAP,MCI,last.visit.age; Emory,totalSample,immune-DC; Emory,totalSample,immune-B_cell; Emory,totalSample,immune-Macrophage; Emory,totalSample,immune-Neutrophil; Emory,totalSample,immune-CD4_T; Emory,totalSample,immune-CD8_T; Emory,totalSample,immune-Tr1; Emory,totalSample,immune-iTreg; Emory,totalSample,immune-Tfh; Emory,totalSample,immune-CD8_naive; Emory,totalSample,immune-InfiltrationScore; Homo_723,totalSample,immune-iTreg; Homo_723,totalSample,immune-Tfh; Homo_723,totalSample,immune-Tr1; Homo_723,totalSample,immune-Th2; Homo_723,totalSample,immune-CD4_T; Homo_723,totalSample,immune-CD4_naive; Homo_723,totalSample,immune-DC; Homo_723,totalSample,immune-Central_memory; Homo_723,totalSample,immune-nTreg; Homo_723,totalSample,immune-Macrophage; Homo_723,totalSample,immune-InfiltrationScore; Homo_723,totalSample,immune-Effector_memory; Homo_723,totalSample,immune-Gamma_delta; Homo_714,totalSample,immune-CD4_T; Homo_714,totalSample,immune-Tfh; Homo_714,totalSample,immune-Tr1; Homo_714,totalSample,immune-iTreg; Homo_714,totalSample,immune-CD4_naive; Homo_714,totalSample,immune-nTreg; Homo_714,totalSample,immune-Th1; Homo_714,totalSample,immune-Central_memory; Homo_714,totalSample,immune-NKT; Homo_714,totalSample,immune-Th2; Homo_714,totalSample,immune-Effector_memory; Homo_714,totalSample,immune-Neutrophil; Homo_714,totalSample,immune-InfiltrationScore; Homo_714,totalSample,immune-Macrophage; Homo_714,totalSample,immune-Bcell; Homo_633,totalSample,immune-CD4_T; Homo_633,totalSample,immune-Tfh; Homo_633,totalSample,immune-Tr1; Homo_633,totalSample,immune-CD4_naive; Homo_633,totalSample,immune-NKT; Homo_633,totalSample,immune-Central_memory; Homo_633,totalSample,immune-iTreg; Homo_633,totalSample,immune-Th1; Homo_633,totalSample,immune-nTreg; Homo_633,totalSample,immune-Effector_memory; Homo_633,totalSample,immune-Neutrophil; Homo_633,totalSample,immune-Th2; Homo_633,totalSample,immune-Gamma_delta; Homo_633,totalSample,immune-DC; Homo_633,totalSample,immune-InfiltrationScore; MCSA,totalSample,immune-DC; MCSA,totalSample,immune-Bcell; MCSA,totalSample,immune-Macrophage; MCSA,totalSample,immune-Neutrophil; MCSA,totalSample,immune-CD4_T; MCSA,totalSample,immune-Gamma_delta; MCSA,totalSample,immune-CD4_naive; MCSA,totalSample,immune-Tr1; MCSA,totalSample,immune-nTreg; MCSA,totalSample,immune-iTreg; MCSA,totalSample,immune-Th17; MCSA,totalSample,immune-Tfh; MCSA,totalSample,immune-CD8_naive; MCSA,totalSample,immune-Cytotoxic; MCSA,totalSample,immune-Exhausted; MCSA,totalSample,immune-Central_memory; ROSMAP,totalSample,immune- [illegible]
TUT4	Homo_723,female,AD.vs.control; Homo_723,male,AD.vs.control; Homo_723,female,AD.vs.MCI; Homo_723,male,AD.vs.MCI; Homo_714,female,AD.vs.control; Homo_714,male,AD.vs.control; Homo_714,female,AD.vs.MCI; Homo_714,male,AD.vs.MCI; Homo_633,female,AD.vs.control; Homo_633,female,AD.vs.MCI; ROSMAP,MCI,MMSE; Emory,totalSample,immune-Macrophage; Emory,totalSample,immune-Neutrophil; Emory,totalSample,immune-CD4_T; Emory,totalSample,immune-CD8_T; Emory,totalSample,immune-Tr1; Emory,totalSample,immune-nTreg; Emory,totalSample,immune-iTreg; Emory,totalSample,immune-Tfh; Emory,totalSample,immune-CD8_naive; Emory,totalSample,immune-Central_memory; Emory,totalSample,immune-InfiltrationScore; Homo_723,totalSample,immune-CD4_naive; Homo_723,totalSample,immune-Central_memory; Homo_723,totalSample,immune-CD4_T; Homo_723,totalSample,immune-nTreg; Homo_723,totalSample,immune-Tr1; Homo_723,totalSample,immune-Tfh; Homo_723,totalSample,immune-Th1; Homo_723,totalSample,immune-iTreg; Homo_723,totalSample,immune-Cytotoxic; Homo_723,totalSample,immune-Th17; Homo_723,totalSample,immune-Monocyte; Homo_723,totalSample,immune-InfiltrationScore; Homo_723,totalSample,immune-Neutrophil; Homo_723,totalSample,immune-NKT; Homo_723,totalSample,immune-Th2; Homo_714,totalSample,immune-CD4_naive; Homo_714,totalSample,immune-Central_memory; Homo_714,totalSample,immune-CD4_T; Homo_714,totalSample,immune-NKT; Homo_714,totalSample,immune-iTreg; Homo_714,totalSample,immune-nTreg; Homo_714,totalSample,immune-Tfh; Homo_714,totalSample,immune-Th1; Homo_714,totalSample,immune-InfiltrationScore; Homo_714,totalSample,immune-Tr1; Homo_714,totalSample,immune-Cytotoxic; Homo_714,totalSample,immune-Effector_memory; Homo_714,totalSample,immune-Th17; Homo_633,totalSample,immune-Tfh; Homo_633,totalSample,immune-CD4_naive; Homo_633,totalSample,immune-CD4_T; Homo_633,totalSample,immune-iTreg; Homo_633,totalSample,immune-Tr1; Homo_633,totalSample,immune-Central_memory; Homo_633,totalSample,immune-NKT; Homo_633,totalSample,immune-nTreg; Homo_633,totalSample,immune-InfiltrationScore; Homo_633,totalSample,immune-Effector_memory; Homo_633,totalSample,immune-Th1; Homo_633,totalSample,immune-Monocyte; Homo_633,totalSample,immune-Gamma_delta; Homo_633,totalSample,immune-DC; MCSA,totalSample,immune-Macrophage; MCSA,totalSample,immune-CD4_T; MCSA,totalSample,immune-NKT; MCSA,totalSample,immune-Gamma_delta; MCSA,totalSample,immune-CD4_naive; MCSA,totalSample,immune-Tr1; MCSA,totalSample,immune-nTreg; MCSA,totalSample,immune-iTreg; MCSA,totalSample,immune-Th17; MCSA,totalSample,immune-Tfh; MCSA,totalSample,immune-CD8_naive; MCSA,totalSample,immune-Cytotoxic; MCSA,totalSample,immune-Exhausted; MCSA,totalSample,immune-MAIT; MCSA,totalSample,immune-Central_memory; MCSA,totalSample,immune-Effector_memory; SRP223445,totalSample,immune-NK
TMEM231	Homo_723,female,AD.vs.control; Homo_723,female,AD.vs.MCI; Homo_723,male,AD.vs.MCI; Homo_714,female,AD.vs.control; Homo_714,female,AD.vs.MCI; Homo_714,male,AD.vs.MCI; Homo_714,female,MCI.vs.control; Homo_633,female,AD.vs.control; Homo_633,female,AD.vs.MCI; Homo_633,female,MCI.vs.control; ROSMAP,female,AD.vs.control; ROSMAP,male,AD.vs.control; ROSMAP,female,MCI.vs.control; MCSA,totalSample,age; MCSA,control,age; Emory,totalSample,immune-CD4_T; Emory,totalSample,immune-Tr1; Emory,totalSample,immune-iTreg; Emory,totalSample,immune-InfiltrationScore; Homo_723,totalSample,immune-Monocyte; Homo_723,totalSample,immune-iTreg; Homo_723,totalSample,immune-Exhausted; Homo_723,totalSample,immune-nTreg; Homo_723,totalSample,immune-CD8_T; Homo_723,totalSample,immune-Central_memory; Homo_723,totalSample,immune-CD4_T; Homo_723,totalSample,immune-Tfh; Homo_723,totalSample,immune-InfiltrationScore; Homo_723,totalSample,immune-CD4_naive; Homo_723,totalSample,immune-CD8_naive; Homo_723,totalSample,immune-Macrophage; Homo_723,totalSample,immune-Tr1; Homo_714,totalSample,immune-Monocyte; Homo_714,totalSample,immune-InfiltrationScore; Homo_714,totalSample,immune-Tfh; Homo_714,totalSample,immune-nTreg; Homo_714,totalSample,immune-iTreg; Homo_714,totalSample,immune-Tr1; Homo_714,totalSample,immune-Macrophage; Homo_633,totalSample,immune-Monocyte; Homo_633,totalSample,immune-InfiltrationScore; Homo_633,totalSample,immune-nTreg; Homo_633,totalSample,immune-NKT; Homo_633,totalSample,immune-CD4_naive; Homo_633,totalSample,immune-Tfh; Homo_633,totalSample,immune-iTreg; Homo_633,totalSample,immune-Tr1; Homo_633,totalSample,immune-CD4_T; MCSA,totalSample,immune-Monocyte; MCSA,totalSample,immune-Macrophage; MCSA,totalSample,immune-CD4_T; MCSA,totalSample,immune-CD8_T; MCSA,totalSample,immune-CD4_naive; MCSA,totalSample,immune-Tr1; MCSA,totalSample,immune-nTreg; MCSA,totalSample,immune-iTreg; MCSA,totalSample,immune-Th1; MCSA,totalSample,immune-Th17; MCSA,totalSample,immune-Tfh; MCSA,totalSample,immune-Central_memory; MCSA,totalSample,immune-Effector_memory; MCSA,totalSample,immune-InfiltrationScore; ROSMAP,totalSample,immune-InfiltrationScore
NHP2	Homo_723,female,AD.vs.control; Homo_723,male,AD.vs.control; Homo_723,female,AD.vs.MCI; Homo_723,male,AD.vs.MCI; Homo_714,female,AD.vs.control; Homo_714,male,AD.vs.control; Homo_714,female,AD.vs.MCI; Homo_714,male,AD.vs.MCI; Homo_633,female,AD.vs.control; Homo_633,female,AD.vs.MCI; ACOM,AD,age; MCSA,totalSample,age; MCSA,control,age; MCSA,MCI,age; ROSMAP,AD,last.visit.age; Emory,totalSample,immune-Macrophage; Emory,totalSample,immune-NK; Emory,totalSample,immune-Neutrophil; Emory,totalSample,immune-CD4_T; Emory,totalSample,immune-CD8_T; Emory,totalSample,immune-Tr1; Emory,totalSample,immune-nTreg; Emory,totalSample,immune-iTreg; Emory,totalSample,immune-Th1; Emory,totalSample,immune-Tfh; Emory,totalSample,immune-CD8_naive; Emory,totalSample,immune-Exhausted; Emory,totalSample,immune-InfiltrationScore; Homo_723,totalSample,immune-CD8_T; Homo_723,totalSample,immune-Exhausted; Homo_723,totalSample,immune-Cytotoxic; Homo_723,totalSample,immune-Monocyte; Homo_723,totalSample,immune-nTreg; Homo_723,totalSample,immune-Macrophage; Homo_723,totalSample,immune-iTreg; Homo_723,totalSample,immune-Tfh; Homo_723,totalSample,immune-Central_memory; Homo_723,totalSample,immune-DC; Homo_723,totalSample,immune-InfiltrationScore; Homo_723,totalSample,immune-MAIT; Homo_723,totalSample,immune-Effector_memory; Homo_723,totalSample,immune-Neutrophil; Homo_723,totalSample,immune-Th17; Homo_723,totalSample,immune-CD8_naive; Homo_714,totalSample,immune-CD8_T; Homo_714,totalSample,immune-Monocyte; Homo_714,totalSample,immune-nTreg; Homo_714,totalSample,immune-DC; Homo_714,totalSample,immune-Macrophage; Homo_714,totalSample,immune-Effector_memory; Homo_714,totalSample,immune-Exhausted; Homo_714,totalSample,immune-Tfh; Homo_714,totalSample,immune-InfiltrationScore; Homo_714,totalSample,immune-Neutrophil; Homo_714,totalSample,immune-Cytotoxic; Homo_714,totalSample,immune-Central_memory; Homo_714,totalSample,immune-MAIT; Homo_714,totalSample,immune-Th17; Homo_633,totalSample,immune-DC; Homo_633,totalSample,immune-Neutrophil; Homo_633,totalSample,immune-nTreg; Homo_633,totalSample,immune-Monocyte; Homo_633,totalSample,immune-CD8_T; Homo_633,totalSample,immune-InfiltrationScore; Homo_633,totalSample,immune-Central_memory; Homo_633,totalSample,immune-Macrophage; Homo_633,totalSample,immune-Effector_memory; Homo_633,totalSample,immune-Cytotoxic; MCSA,totalSample,immune-DC; MCSA,totalSample,immune-Monocyte; MCSA,totalSample,immune-Neutrophil; MCSA,totalSample,immune-CD8_T; MCSA,totalSample,immune-NKT; MCSA,totalSample,immune-iTreg; MCSA,totalSample,immune-Th2; MCSA,totalSample,immune-Tfh; MCSA,totalSample,immune-Cytotoxic; MCSA,totalSample,immune-Exhausted; MCSA,totalSample,immune-Central_memory; MCSA,totalSample,immune-Effector_memory; MCSA,totalSample,immune-InfiltrationScore; ROSMAP,totalSample,immune-Effector_memory; SRP223445,totalSample,immune- [illegible]
TRIP12	Homo_723,female,AD.vs.control; Homo_723,male,AD.vs.control; Homo_723,female,AD.vs.MCI; Homo_723,male,AD.vs.MCI; Homo_723,female,MCI.vs.control; Homo_723,male,MCI.vs.control; Homo_723,MCI,male.vs.female; Homo_714,female,AD.vs.control; Homo_714,male,AD.vs.control; Homo_714,male,AD.vs.MCI; Homo_714,female,MCI.vs.control; Homo_714,male,MCI.vs.control; Homo_714,MCI,male.vs.female; Homo_633,female,AD.vs.control; Homo_633,female,AD.vs.MCI; Homo_633,female,MCI.vs.control; Homo_633,male,MCI.vs.control; Homo_633,MCI,male.vs.female; ROSMAP,female,AD.vs.control; ROSMAP,MCI,last.visit.age; Emory,totalSample,immune-NKT; Emory,totalSample,immune-Th1; Emory,totalSample,immune-InfiltrationScore; Homo_723,totalSample,immune-NKT; Homo_723,totalSample,immune-Th1; Homo_723,totalSample,immune-Cytotoxic; Homo_714,totalSample,immune-NKT; Homo_714,totalSample,immune-Th1; Homo_633,totalSample,immune-NKT; Homo_633,totalSample,immune-Th1; MCSA,totalSample,immune-DC; MCSA,totalSample,immune-Monocyte; MCSA,totalSample,immune-CD4_T; MCSA,totalSample,immune-CD8_T; MCSA,totalSample,immune-CD4_naive; MCSA,totalSample,immune-Tr1; MCSA,totalSample,immune-nTreg; MCSA,totalSample,immune-iTreg; MCSA,totalSample,immune-Th17; MCSA,totalSample,immune-Tfh; MCSA,totalSample,immune-CD8_naive;
CNTD1	Homo_723,female,AD.vs.control; Homo_723,male,AD.vs.control; Homo_723,female,AD.vs.MCI; Homo_723,male,AD.vs.MCI; Homo_714,female,AD.vs.control; Homo_714,male,AD.vs.control; Homo_714,female,AD.vs.MCI; Homo_714,male,AD.vs.MCI; Homo_633,female,AD.vs.control; Homo_633,female,AD.vs.MCI; ROSMAP,female,AD.vs.control; Emory,MCI,age; MCSA,totalSample,age; MCSA,control,age; ROSMAP,MCI,last.visit.age; Emory,totalSample,immune-Gamma_delta; Emory,totalSample,immune-Th17; Homo_723,totalSample,immune-Th2; Homo_723,totalSample,immune-Tfh; Homo_723,totalSample,immune-iTreg; Homo_723,totalSample,immune-CD4_T; Homo_723,totalSample,immune-InfiltrationScore; Homo_723,totalSample,immune-Tr1; Homo_723,totalSample,immune-CD4_naive; Homo_723,totalSample,immune-Gamma_delta; Homo_723,totalSample,immune-Effector_memory; Homo_723,totalSample,immune-DC; Homo_723,totalSample,immune-nTreg; Homo_714,totalSample,immune-Th2; Homo_714,totalSample,immune-Tfh; Homo_714,totalSample,immune-CD4_T; Homo_714,totalSample,immune-Tr1; Homo_714,totalSample,immune-Effector_memory; Homo_714,totalSample,immune-iTreg; Homo_714,totalSample,immune-nTreg; Homo_714,totalSample,immune-InfiltrationScore; Homo_714,totalSample,immune-Gamma_delta; Homo_714,totalSample,immune-CD4_naive; Homo_714,totalSample,immune-Central_memory; Homo_633,totalSample,immune-Th2; Homo_633,totalSample,immune-Tfh; Homo_633,totalSample,immune-CD4_T; Homo_633,totalSample,immune-Th17; Homo_633,totalSample,immune-InfiltrationScore; Homo_633,totalSample,immune-CD4_naive; Homo_633,totalSample,immune-nTreg; Homo_633,totalSample,immune-Tr1; Homo_633,totalSample,immune-Effector_memory; Homo_633,totalSample,immune-Exhausted; Homo_633,totalSample,immune-iTreg; Homo_633,totalSample,immune-Gamma_delta; Homo_633,totalSample,immune-Central_memory; MCSA,totalSample,immune-Monocyte; MCSA,totalSample,immune-CD4_T; MCSA,totalSample,immune-Gamma_delta; MCSA,totalSample,immune-CD4_naive; MCSA,totalSample,immune-Tr1; MCSA,totalSample,immune-nTreg; MCSA,totalSample,immune-iTreg; MCSA,totalSample,immune-Th17; MCSA,totalSample,immune-Tfh; MCSA,totalSample,immune-Cytotoxic; MCSA,totalSample,immune-Exhausted; MCSA,totalSample,immune-Central_memory; SRP223445,totalSample,immune-InfiltrationScore; ROSMAP,male- [illegible]
FAM81B	Homo_723,female,AD.vs.control; Homo_723,male,AD.vs.control; Homo_723,female,AD.vs.MCI; Homo_723,male,AD.vs.MCI; Homo_723,female,MCI.vs.control; Homo_723,male,MCI.vs.control; Homo_723,control,male.vs.female; Homo_714,female,AD.vs.control; Homo_714,male,AD.vs.control; Homo_714,female,AD.vs.MCI; Homo_714,male,AD.vs.MCI; Homo_714,female,MCI.vs.control; Homo_714,male,MCI.vs.control; Homo_714,control,male.vs.female; Homo_633,female,AD.vs.control; Homo_633,female,AD.vs.MCI; ROSMAP,female,AD.vs.control; ROSMAP,male,AD.vs.control; SRP223445,female,AD.vs.control; MCSA,totalSample,age; MCSA,control,age; ROSMAP,totalSample,age; ROSMAP,MCI,last.visit.age; ROSMAP,MCI,sample.age; Emory,totalSample,immune-CD4_naive; Emory,totalSample,immune-Cytotoxic; Homo_723,totalSample,immune-Exhausted; Homo_723,totalSample,immune-Monocyte; Homo_723,totalSample,immune-CD8_T; Homo_723,totalSample,immune-Th17; Homo_714,totalSample,immune-Monocyte; Homo_633,totalSample,immune-Monocyte; MCSA,totalSample,immune-DC; MCSA,totalSample,immune-Monocyte; MCSA,totalSample,immune-Gamma_delta; MCSA,totalSample,immune-CD4_naive; MCSA,totalSample,immune-nTreg; MCSA,totalSample,immune-Th2; MCSA,totalSample,immune-Th17; MCSA,totalSample,immune-Effector_memory; ROSMAP,totalSample,immune-Neutrophil; ROSMAP,totalSample,immune-CD4_T; ROSMAP,totalSample,immune-Th17; ROSMAP,totalSample,immune-Central_memory; ROSMAP,totalSample,immune-InfiltrationScore; SRP223445,totalSample,immune-CD4_naive; SRP223445,totalSample,immune-InfiltrationScore
FLVCR2	Homo_723,female,AD.vs.control; Homo_723,male,AD.vs.control; Homo_723,female,AD.vs.MCI; Homo_723,male,AD.vs.MCI; Homo_714,female,AD.vs.control; Homo_714,female,AD.vs.MCI; Homo_714,male,AD.vs.MCI; Homo_633,female,AD.vs.control; Homo_633,female,AD.vs.MCI; MCSA,MCI,age; ROSMAP,MCI,last.visit.age; Emory,totalSample,immune-CD8_T; Emory,totalSample,immune-NKT; Emory,totalSample,immune-Gamma_delta; Emory,totalSample,immune-Tfh; Emory,totalSample,immune-CD8_naive; Emory,totalSample,immune-InfiltrationScore; Homo_723,totalSample,immune-InfiltrationScore; Homo_723,totalSample,immune-Monocyte; Homo_723,totalSample,immune-Gamma_delta; Homo_723,totalSample,immune-Th2; Homo_723,totalSample,immune-Effector_memory; Homo_723,totalSample,immune-Tfh; Homo_723,totalSample,immune-iTreg; Homo_723,totalSample,immune-Bcell; Homo_714,totalSample,immune-Th2; Homo_714,totalSample,immune-Tfh; Homo_714,totalSample,immune-Gamma_delta; Homo_714,totalSample,immune-Monocyte; Homo_714,totalSample,immune-Effector_memory; Homo_714,totalSample,immune-InfiltrationScore; Homo_633,totalSample,immune-Monocyte; Homo_633,totalSample,immune-Exhausted; Homo_633,totalSample,immune-Gamma_delta; Homo_633,totalSample,immune-Th2; Homo_633,totalSample,immune-InfiltrationScore; Homo_633,totalSample,immune-Effector_memory; Homo_633,totalSample,immune-Tfh; Homo_633,totalSample,immune-Th17; MCSA,totalSample,immune-Macrophage; MCSA,totalSample,immune-CD8_T; MCSA,totalSample,immune-Gamma_delta; MCSA,totalSample,immune-Tr1; MCSA,totalSample,immune-iTreg; MCSA,totalSample,immune-Th17; MCSA,totalSample,immune-Cytotoxic;
CLIC4	Homo_723,female,AD.vs.control; Homo_723,male,AD.vs.control; Homo_723,female,AD.vs.MCI; Homo_723,male,AD.vs.MCI; Homo_723,female,MCI.vs.control; Homo_723,male,MCI.vs.control; Homo_714,female,AD.vs.control; Homo_714,male,AD.vs.control; Homo_714,female,AD.vs.MCI; Homo_714,male,AD.vs.MCI; Homo_714,female,MCI.vs.control; Homo_714,male,MCI.vs.control; Homo_633,female,AD.vs.control; Homo_633,female,AD.vs.MCI; ROSMAP,female,AD.vs.control; MCSA,MCI,age; Emory,totalSample,immune-Monocyte; Emory,totalSample,immune-Gamma_delta; Emory,totalSample,immune-Tr1; Emory,totalSample,immune-iTreg; Emory,totalSample,immune-Th1; Homo_723,totalSample,immune-Exhausted; Homo_723,totalSample,immune-Th17; Homo_723,totalSample,immune-Cytotoxic; Homo_723,totalSample,immune-CD8_T; Homo_723,totalSample,immune-Monocyte; Homo_723,totalSample,immune-CD4_naive; Homo_723,totalSample,immune-CD4_T; Homo_723,totalSample,immune-Gamma_delta; Homo_723,totalSample,immune-Th1; Homo_723,totalSample,immune-Tr1; Homo_723,totalSample,immune-Th2; Homo_714,totalSample,immune-Monocyte; Homo_714,totalSample,immune-Exhausted; Homo_714,totalSample,immune-Th17; Homo_714,totalSample,immune-Gamma_delta; Homo_714,totalSample,immune-CD8_T; Homo_714,totalSample,immune-Cytotoxic; Homo_714,totalSample,immune-CD4_naive; Homo_714,totalSample,immune-NKT; Homo_633,totalSample,immune-Th2; Homo_633,totalSample,immune-Monocyte; Homo_633,totalSample,immune-Tfh; Homo_633,totalSample,immune-Gamma_delta; Homo_633,totalSample,immune-CD4_T; Homo_633,totalSample,immune-NKT; Homo_633,totalSample,immune-Exhausted; Homo_633,totalSample,immune-Th17; Homo_633,totalSample,immune-Central_memory; Homo_633,totalSample,immune-CD4_naive; Homo_633,totalSample,immune-Tr1; Homo_633,totalSample,immune-Effector_memory; Homo_633,totalSample,immune-DC; MCSA,totalSample,immune-DC; MCSA,totalSample,immune-Bcell; MCSA,totalSample,immune-Monocyte; MCSA,totalSample,immune-CD4_T; MCSA,totalSample,immune-CD8_T; MCSA,totalSample,immune-Gamma_delta; MCSA,totalSample,immune-CD4_naive; MCSA,totalSample,immune-Tr1; MCSA,totalSample,immune-nTreg; MCSA,totalSample,immune-iTreg; MCSA,totalSample,immune-Th2; MCSA,totalSample,immune-Th17; MCSA,totalSample,immune-Tfh; [illegible]
KLHDC1	Homo_723,female,AD.vs.control; Homo_723,male,AD.vs.control; Homo_723,female,AD.vs.MCI; Homo_723,male,AD.vs.MCI; Homo_723,female,MCI.vs.control; Homo_723,male,MCI.vs.control; Homo_714,female,AD.vs.control; Homo_714,male,AD.vs.control; Homo_714,female,AD.vs.MCI; Homo_714,male,AD.vs.MCI; Homo_714,female,MCI.vs.control; Homo_714,male,MCI.vs.control; Homo_633,female,AD.vs.control; Homo_633,female,AD.vs.MCI; ROSMAP,male,AD.vs.MCI; ROSMAP,control,male.vs.female; MCSA,MCI,age; Emory,totalSample,immune-Macrophage; Emory,totalSample,immune-CD4_T; Emory,totalSample,immune-Gamma_delta; Emory,totalSample,immune-Tr1; Emory,totalSample,immune-iTreg; Emory,totalSample,immune-Tfh; Emory,totalSample,immune-CD8_naive; Emory,totalSample,immune-Central_memory; Emory,totalSample,immune-InfiltrationScore; Homo_723,totalSample,immune-CD4_naive; Homo_723,totalSample,immune-CD4_T; Homo_723,totalSample,immune-Tr1; Homo_723,totalSample,immune-Cytotoxic; Homo_723,totalSample,immune-Th1; Homo_723,totalSample,immune-Tfh; Homo_723,totalSample,immune-Th17; Homo_723,totalSample,immune-nTreg; Homo_723,totalSample,immune-iTreg; Homo_723,totalSample,immune-Exhausted; Homo_723,totalSample,immune-Th2; Homo_723,totalSample,immune-NKT; Homo_723,totalSample,immune-Gamma_delta; Homo_723,totalSample,immune-Neutrophil; Homo_723,totalSample,immune-InfiltrationScore; Homo_714,totalSample,immune-CD4_naive; Homo_714,totalSample,immune-CD4_T; Homo_714,totalSample,immune-Central_memory; Homo_714,totalSample,immune-Th1; Homo_714,totalSample,immune-NKT; Homo_714,totalSample,immune-Tfh; Homo_714,totalSample,immune-Tr1; Homo_714,totalSample,immune-iTreg; Homo_714,totalSample,immune-Th17; Homo_714,totalSample,immune-Cytotoxic; Homo_714,totalSample,immune-nTreg; Homo_714,totalSample,immune-Exhausted; Homo_714,totalSample,immune-Gamma_delta; Homo_633,totalSample,immune-Tfh; Homo_633,totalSample,immune-CD4_T; Homo_633,totalSample,immune-CD4_naive; Homo_633,totalSample,immune-Tr1; Homo_633,totalSample,immune-Central_memory; Homo_633,totalSample,immune-NKT; Homo_633,totalSample,immune-iTreg; Homo_633,totalSample,immune-Effector_memory; Homo_633,totalSample,immune-nTreg; Homo_633,totalSample,immune-Th2; Homo_633,totalSample,immune-Gamma_delta; Homo_633,totalSample,immune-Th1; Homo_633,totalSample,immune-InfiltrationScore; Homo_633,totalSample,immune-DC; Homo_633,totalSample,immune-Th17; Homo_633,totalSample,immune-Cytotoxic; Homo_633,totalSample,immune-Exhausted; MCSA,totalSample,immune-DC; MCSA,totalSample,immune-Bcell; MCSA,totalSample,immune-Monocyte; MCSA,totalSample,immune-Macrophage; MCSA,totalSample,immune-CD4_T; MCSA,totalSample,immune-CD4_naive; MCSA,totalSample,immune-Tr1; MCSA,totalSample,immune-nTreg; MCSA,totalSample,immune-iTreg; MCSA,totalSample,immune-Th17; MCSA,totalSample,immune-Tfh; MCSA,totalSample,immune-CD8_naive; MCSA,totalSample,immune-Cytotoxic; MCSA,totalSample,immune-Exhausted; MCSA,totalSample,immune-MAIT; MCSA,totalSample,immune-Central_memory; ROSMAP,totalSample,immune-Effector_memory; SRP223445,totalSample,immune-CD4_naive; SRP223445,totalSample,immune-Exhausted; SRP223445,totalSample,immune-InfiltrationScore
KCND3	Homo_723,female,AD.vs.control; Homo_723,male,AD.vs.control; Homo_723,female,AD.vs.MCI; Homo_723,male,AD.vs.MCI; Homo_723,female,MCI.vs.control; Homo_723,male,MCI.vs.control; Homo_714,female,AD.vs.control; Homo_714,male,AD.vs.control; Homo_714,female,AD.vs.MCI; Homo_714,male,AD.vs.MCI; Homo_714,female,MCI.vs.control; Homo_714,male,MCI.vs.control; Homo_633,female,AD.vs.control; Homo_633,female,AD.vs.MCI; Emory,totalSample,immune-Th17; Homo_723,totalSample,immune-Th17; Homo_723,totalSample,immune-DC; Homo_723,totalSample,immune-Cytotoxic; Homo_723,totalSample,immune-Exhausted; Homo_723,totalSample,immune-CD8_T; Homo_723,totalSample,immune-MAIT; Homo_714,totalSample,immune-Th17; Homo_714,totalSample,immune-Exhausted; Homo_714,totalSample,immune-CD8_T; Homo_714,totalSample,immune-Cytotoxic; Homo_633,totalSample,immune-Gamma_delta; Homo_633,totalSample,immune-Th17; Homo_633,totalSample,immune-Th2; MCSA,totalSample,immune-CD4_T; MCSA,totalSample,immune-Gamma_delta; MCSA,totalSample,immune-CD4_naive; MCSA,totalSample,immune-Tr1; MCSA,totalSample,immune-nTreg; MCSA,totalSample,immune-iTreg; MCSA,totalSample,immune-Th17; SRP223445,totalSample,immune-CD4_naive; SRP223445,totalSample,immune-Exhausted
C12orf42	Homo_723,female,AD.vs.control; Homo_723,male,AD.vs.control; Homo_723,female,AD.vs.MCI; Homo_723,male,AD.vs.MCI; Homo_714,female,AD.vs.control; Homo_714,male,AD.vs.control; Homo_714,female,AD.vs.MCI; Homo_714,male,AD.vs.MCI; Homo_633,female,AD.vs.control; Homo_633,female,AD.vs.MCI; ROSMAP,male,AD.vs.control; Emory,MCI,age; MCSA,totalSample,age; MCSA,control,age; ROSMAP,MCI,last.visit.age; ROSMAP,MCI,sample.age; Emory,totalSample,immune-B_cell; Emory,totalSample,immune-Macrophage; Emory,totalSample,immune-Neutrophil; Emory,totalSample,immune-CD4_T; Emory,totalSample,immune-CD8_T; Emory,totalSample,immune-Tr1; Emory,totalSample,immune-iTreg; Emory,totalSample,immune-Tfh; Emory,totalSample,immune-CD8_naive; Emory,totalSample,immune-Exhausted; Emory,totalSample,immune-InfiltrationScore; Homo_723,totalSample,immune-iTreg; Homo_723,totalSample,immune-Tfh; Homo_723,totalSample,immune-CD4_T; Homo_723,totalSample,immune-CD4_naive; Homo_723,totalSample,immune-Central_memory; Homo_723,totalSample,immune-Tr1; Homo_723,totalSample,immune-nTreg; Homo_723,totalSample,immune-CD8_T; Homo_723,totalSample,immune-Th2; Homo_723,totalSample,immune-Macrophage; Homo_723,totalSample,immune-InfiltrationScore; Homo_723,totalSample,immune-Effector_memory; Homo_723,totalSample,immune-Exhausted; Homo_723,totalSample,immune-MAIT; Homo_723,totalSample,immune-CD8_naive; Homo_714,totalSample,immune-Tfh; Homo_714,totalSample,immune-CD4_T; Homo_714,totalSample,immune-nTreg; Homo_714,totalSample,immune-Tr1; Homo_714,totalSample,immune-iTreg; Homo_714,totalSample,immune-CD4_naive; Homo_714,totalSample,immune-Central_memory; Homo_714,totalSample,immune-Th2; Homo_714,totalSample,immune-Macrophage; Homo_714,totalSample,immune-Effector_memory; Homo_714,totalSample,immune-Neutrophil; Homo_714,totalSample,immune-MAIT; Homo_714,totalSample,immune-InfiltrationScore; Homo_714,totalSample,immune-CD8_T; Homo_714,totalSample,immune-Th1; Homo_714,totalSample,immune-Monocyte; Homo_633,totalSample,immune-Tfh; Homo_633,totalSample,immune-CD4_T; Homo_633,totalSample,immune-CD4_naive; Homo_633,totalSample,immune-nTreg; Homo_633,totalSample,immune-Tr1; Homo_633,totalSample,immune-Central_memory; Homo_633,totalSample,immune-iTreg; Homo_633,totalSample,immune-Neutrophil; Homo_633,totalSample,immune-Effector_memory; Homo_633,totalSample,immune-Macrophage; Homo_633,totalSample,immune-Th1; Homo_633,totalSample,immune-NKT; Homo_633,totalSample,immune-InfiltrationScore; Homo_633,totalSample,immune-MAIT; Homo_633,totalSample,immune-Monocyte; MCSA,totalSample,immune-DC; MCSA,totalSample,immune-Bcell; MCSA,totalSample,immune-Monocyte; MCSA,totalSample,immune-Macrophage; MCSA,totalSample,immune-Neutrophil; MCSA,totalSample,immune-CD4_T; MCSA,totalSample,immune-CD4_naive; MCSA,totalSample,immune-Tr1; MCSA,totalSample,immune-nTreg; MCSA,totalSample,immune-iTreg; MCSA,totalSample,immune-Th17; MCSA,totalSample,immune-Tfh; MCSA,totalSample,immune-CD8_naive; MCSA,totalSample,immune-MAIT; MCSA,totalSample,immune-Central_memory; MCSA,totalSample,immune-Effector_memory; MCSA,totalSample,immune-InfiltrationScore; ROSMAP,totalSample,immune-InfiltrationScore

TBC1D16	Homo_723,female,AD.vs.control; Homo_723,male,AD.vs.control; Homo_723,female,AD.vs.MCI; Homo_723,male,AD.vs.MCI; Homo_723,female,MCI.vs.control; Homo_723,male,MCI.vs.control; Homo_714,female,AD.vs.control; Homo_714,male,AD.vs.control; Homo_714,female,AD.vs.MCI; Homo_714,male,AD.vs.MCI; Homo_714,female,MCI.vs.control; Homo_714,male,MCI.vs.control; Homo_633,female,AD.vs.control; Homo_633,female,AD.vs.MCI; ROSMAP,AD,male.vs.female; ROSMAP,MCI,male.vs.female; SRP223445,female,AD.vs.control; Emory,totalSample,age; Emory,control,age; MCSA,totalSample,age; MCSA,control,age; MCSA,MCI,age; Emory,totalSample,immune-Neutrophil; Emory,totalSample,immune-InfiltrationScore; Homo_723,totalSample,immune-DC; Homo_723,totalSample,immune-InfiltrationScore; Homo_723,totalSample,immune-nTreg; Homo_723,totalSample,immune-Neutrophil; Homo_723,totalSample,immune-CD4_naive; Homo_723,totalSample,immune-Th17; Homo_723,totalSample,immune-Central_memory; Homo_723,totalSample,immune-Effector_memory; Homo_723,totalSample,immune-CD4_T; Homo_723,totalSample,immune-Tfh; Homo_723,totalSample,immune-Exhausted; Homo_714,totalSample,immune-InfiltrationScore; Homo_714,totalSample,immune-DC; Homo_714,totalSample,immune-nTreg; Homo_714,totalSample,immune-Neutrophil; Homo_714,totalSample,immune-CD4_naive; Homo_714,totalSample,immune-Effector_memory; Homo_633,totalSample,immune-InfiltrationScore; Homo_633,totalSample,immune-nTreg; Homo_633,totalSample,immune-Effector_memory; Homo_633,totalSample,immune-DC; Homo_633,totalSample,immune-Tfh; Homo_633,totalSample,immune-Central_memory; Homo_633,totalSample,immune-CD4_naive; Homo_633,totalSample,immune-CD4_T; Homo_633,totalSample,immune-Neutrophil; MCSA,totalSample,immune-DC; MCSA,totalSample,immune-Monocyte; MCSA,totalSample,immune-Neutrophil; MCSA,totalSample,immune-CD4_T; MCSA,totalSample,immune-CD8_T; MCSA,totalSample,immune-NKT; MCSA,totalSample,immune-CD4_naive; MCSA,totalSample,immune-Tr1; MCSA,totalSample,immune-nTreg; MCSA,totalSample,immune-Th2; MCSA,totalSample,immune-Tfh; MCSA,totalSample,immune-Cytotoxic; MCSA,totalSample,immune-Central_memory; MCSA,totalSample,immune-Effector_memory; MCSA,totalSample,immune-InfiltrationScore; SRP223445,totalSample,immune-Neutrophil; SRP223445,totalSample,immune-nTreg; SRP223445,totalSample,immune-[illegible]
CNTLN	Homo_723,female,AD.vs.control; Homo_723,male,AD.vs.control; Homo_723,female,AD.vs.MCI; Homo_723,male,AD.vs.MCI; Homo_723,female,MCI.vs.control; Homo_723,male,MCI.vs.control; Homo_714,female,AD.vs.control; Homo_714,male,AD.vs.control; Homo_714,female,AD.vs.MCI; Homo_714,male,AD.vs.MCI; Homo_714,female,MCI.vs.control; Homo_714,male,MCI.vs.control; Homo_633,female,AD.vs.control; Homo_633,female,AD.vs.MCI; ROSMAP,female,MCI.vs.control; MCSA,MCI,age; ROSMAP,MCI,last.visit.age; ROSMAP,MCI,sample.age; Emory,totalSample,immune-Macrophage; Emory,totalSample,immune-CD8_T; Emory,totalSample,immune-Gamma_delta; Emory,totalSample,immune-Tfh; Emory,totalSample,immune-CD8_naive; Homo_723,totalSample,immune-Exhausted; Homo_723,totalSample,immune-CD8_T; Homo_723,totalSample,immune-Cytotoxic; Homo_723,totalSample,immune-Th17; Homo_723,totalSample,immune-Macrophage; Homo_723,totalSample,immune-Monocyte; Homo_714,totalSample,immune-Exhausted; Homo_714,totalSample,immune-Monocyte; Homo_714,totalSample,immune-CD8_T; Homo_714,totalSample,immune-Macrophage; Homo_714,totalSample,immune-Th17; Homo_714,totalSample,immune-Cytotoxic; Homo_714,totalSample,immune-Gamma_delta; Homo_633,totalSample,immune-Monocyte; Homo_633,totalSample,immune-Exhausted; Homo_633,totalSample,immune-Gamma_delta; Homo_633,totalSample,immune-Macrophage; Homo_633,totalSample,immune-CD8_T; Homo_633,totalSample,immune-Tfh; Homo_633,totalSample,immune-InfiltrationScore; MCSA,totalSample,immune-DC; MCSA,totalSample,immune-Monocyte; MCSA,totalSample,immune-CD8_T; MCSA,totalSample,immune-Tr1; MCSA,totalSample,immune-iTreg; MCSA,totalSample,immune-Cytotoxic; MCSA,totalSample,immune-Exhausted; MCSA,totalSample,immune-Effector_memory; ROSMAP,totalSample,immune-Th17; ROSMAP,totalSample,immune-MAIT; ROSMAP,totalSample,immune-Effector_memory; ROSMAP,totalSample,immune-InfiltrationScore; SRP223445,totalSample,immune-[illegible]
C16orf74	Homo_723,female,AD.vs.control; Homo_723,male,AD.vs.control; Homo_723,female,AD.vs.MCI; Homo_723,male,AD.vs.MCI; Homo_714,female,AD.vs.control; Homo_714,male,AD.vs.control; Homo_714,female,AD.vs.MCI; Homo_714,male,AD.vs.MCI; Homo_633,female,AD.vs.control; Homo_633,female,AD.vs.MCI; MCSA,totalSample,age; MCSA,control,age; MCSA,MCI,age; ROSMAP,AD,last.visit.age; Emory,totalSample,immune-B_cell; Emory,totalSample,immune-Macrophage; Emory,totalSample,immune-Neutrophil; Emory,totalSample,immune-CD4_T; Emory,totalSample,immune-Tr1; Emory,totalSample,immune-iTreg; Emory,totalSample,immune-Tfh; Emory,totalSample,immune-CD8_naive; Homo_723,totalSample,immune-CD8_T; Homo_723,totalSample,immune-Cytotoxic; Homo_723,totalSample,immune-nTreg; Homo_723,totalSample,immune-Exhausted; Homo_723,totalSample,immune-Central_memory; Homo_723,totalSample,immune-iTreg; Homo_723,totalSample,immune-Tfh; Homo_723,totalSample,immune-Effector_memory; Homo_723,totalSample,immune-Macrophage; Homo_723,totalSample,immune-InfiltrationScore; Homo_723,totalSample,immune-CD8_naive; Homo_714,totalSample,immune-nTreg; Homo_714,totalSample,immune-CD8_T; Homo_714,totalSample,immune-Effector_memory; Homo_714,totalSample,immune-Tfh; Homo_714,totalSample,immune-Central_memory; Homo_714,totalSample,immune-DC; Homo_714,totalSample,immune-Macrophage; Homo_714,totalSample,immune-Neutrophil; Homo_714,totalSample,immune-CD8_naive; Homo_633,totalSample,immune-DC; Homo_633,totalSample,immune-nTreg; Homo_633,totalSample,immune-Neutrophil; Homo_633,totalSample,immune-Central_memory; Homo_633,totalSample,immune-CD8_T; Homo_633,totalSample,immune-Monocyte; MCSA,totalSample,immune-DC; MCSA,totalSample,immune-Monocyte; MCSA,totalSample,immune-Neutrophil; MCSA,totalSample,immune-CD8_T; MCSA,totalSample,immune-NKT; MCSA,totalSample,immune-Th2; MCSA,totalSample,immune-Tfh; MCSA,totalSample,immune-Cytotoxic; MCSA,totalSample,immune-[illegible]
EHBP1	Homo_723,female,AD.vs.control; Homo_723,male,AD.vs.control; Homo_723,female,AD.vs.MCI; Homo_723,male,AD.vs.MCI; Homo_723,female,MCI.vs.control; Homo_723,male,MCI.vs.control; Homo_714,female,AD.vs.control; Homo_714,male,AD.vs.control; Homo_714,female,AD.vs.MCI; Homo_714,male,AD.vs.MCI; Homo_714,female,MCI.vs.control; Homo_714,male,MCI.vs.control; Homo_633,female,AD.vs.control; Homo_633,female,AD.vs.MCI; ROSMAP,female,AD.vs.control; Emory,totalSample,immune-Macrophage; Emory,totalSample,immune-NK; Emory,totalSample,immune-Neutrophil; Emory,totalSample,immune-CD4_T; Emory,totalSample,immune-CD8_T; Emory,totalSample,immune-Tr1; Emory,totalSample,immune-iTreg; Emory,totalSample,immune-Tfh; Emory,totalSample,immune-Exhausted; Emory,totalSample,immune-MAIT; Emory,totalSample,immune-Central_memory; Emory,totalSample,immune-Effector_memory; Emory,totalSample,immune-InfiltrationScore; Homo_723,totalSample,immune-CD4_naive; Homo_723,totalSample,immune-CD4_T; Homo_723,totalSample,immune-Tfh; Homo_723,totalSample,immune-Central_memory; Homo_723,totalSample,immune-Th17; Homo_723,totalSample,immune-Tr1; Homo_723,totalSample,immune-nTreg; Homo_723,totalSample,immune-Exhausted; Homo_723,totalSample,immune-Th2; Homo_723,totalSample,immune-Cytotoxic; Homo_723,totalSample,immune-InfiltrationScore; Homo_723,totalSample,immune-iTreg; Homo_723,totalSample,immune-Neutrophil; Homo_723,totalSample,immune-Th1; Homo_723,totalSample,immune-Effector_memory; Homo_714,totalSample,immune-CD4_naive; Homo_714,totalSample,immune-CD4_T; Homo_714,totalSample,immune-Central_memory; Homo_714,totalSample,immune-Tfh; Homo_714,totalSample,immune-InfiltrationScore; Homo_714,totalSample,immune-Th17; Homo_714,totalSample,immune-Effector_memory; Homo_714,totalSample,immune-Tr1; Homo_714,totalSample,immune-iTreg; Homo_714,totalSample,immune-NKT; Homo_714,totalSample,immune-nTreg; Homo_714,totalSample,immune-Th1; Homo_714,totalSample,immune-Neutrophil; Homo_714,totalSample,immune-Th2; Homo_714,totalSample,immune-Exhausted; Homo_633,totalSample,immune-Tfh; Homo_633,totalSample,immune-CD4_T; Homo_633,totalSample,immune-CD4_naive; Homo_633,totalSample,immune-Effector_memory; Homo_633,totalSample,immune-Central_memory; Homo_633,totalSample,immune-InfiltrationScore; Homo_633,totalSample,immune-Tr1; Homo_633,totalSample,immune-Th2; Homo_633,totalSample,immune-iTreg; Homo_633,totalSample,immune-nTreg; Homo_633,totalSample,immune-NKT; Homo_633,totalSample,immune-Bcell; Homo_633,totalSample,immune-Neutrophil; Homo_633,totalSample,immune-Th17; Homo_633,totalSample,immune-MAIT; Homo_633,totalSample,immune-Gamma_delta; MCSA,totalSample,immune-DC; MCSA,totalSample,immune-Macrophage; MCSA,totalSample,immune-CD4_T; MCSA,totalSample,immune-CD4_naive; MCSA,totalSample,immune-Tr1; MCSA,totalSample,immune-nTreg; MCSA,totalSample,immune-iTreg; MCSA,totalSample,immune-Th17; MCSA,totalSample,immune-Tfh; MCSA,totalSample,immune-CD8_naive; MCSA,totalSample,immune-Cytotoxic; MCSA,totalSample,immune-Exhausted; MCSA,totalSample,immune-MAIT; MCSA,totalSample,immune-Central_memory; MCSA,totalSample,immune-Effector_memory; ROSMAP,totalSample,immune-Effector_memory; SRP223445,totalSample,immune-Th1
LOC102723407	Homo_723,female,AD.vs.control; Homo_723,male,AD.vs.control; Homo_723,female,AD.vs.MCI; Homo_723,male,AD.vs.MCI; Homo_723,female,MCI.vs.control; Homo_723,male,MCI.vs.control; Homo_723,control,male.vs.female; Homo_714,female,AD.vs.control; Homo_714,male,AD.vs.control; Homo_714,female,AD.vs.MCI; Homo_714,male,AD.vs.MCI; Homo_714,female,MCI.vs.control; Homo_714,male,MCI.vs.control; Homo_714,control,male.vs.female; Homo_633,female,AD.vs.control; Homo_633,female,AD.vs.MCI; Homo_633,female,MCI.vs.control; Homo_633,male,MCI.vs.control; Homo_633,control,male.vs.female; Emory,MCI,age; MCSA,MCI,age; Emory,totalSample,immune-B_cell; Emory,totalSample,immune-Macrophage; Emory,totalSample,immune-Neutrophil; Emory,totalSample,immune-CD4_T; Emory,totalSample,immune-CD8_T; Emory,totalSample,immune-Tr1; Emory,totalSample,immune-iTreg; Emory,totalSample,immune-Tfh; Emory,totalSample,immune-CD8_naive; Emory,totalSample,immune-Exhausted; Emory,totalSample,immune-InfiltrationScore; Homo_723,totalSample,immune-Exhausted; Homo_723,totalSample,immune-CD8_T; Homo_723,totalSample,immune-iTreg; Homo_723,totalSample,immune-Tfh; Homo_723,totalSample,immune-InfiltrationScore; Homo_723,totalSample,immune-Central_memory; Homo_714,totalSample,immune-Tfh; Homo_714,totalSample,immune-nTreg; Homo_714,totalSample,immune-Monocyte; Homo_714,totalSample,immune-Tr1; Homo_714,totalSample,immune-iTreg; Homo_714,totalSample,immune-Macrophage; Homo_633,totalSample,immune-Monocyte; Homo_633,totalSample,immune-nTreg; Homo_633,totalSample,immune-Tfh; Homo_633,totalSample,immune-Neutrophil; MCSA,totalSample,immune-Monocyte; MCSA,totalSample,immune-CD4_T; MCSA,totalSample,immune-Gamma_delta; MCSA,totalSample,immune-CD4_naive; MCSA,totalSample,immune-Tr1; MCSA,totalSample,immune-nTreg; MCSA,totalSample,immune-Th17; MCSA,totalSample,immune-Tfh; MCSA,totalSample,immune-Central_memory; MCSA,totalSample,immune-InfiltrationScore; SRP223445,totalSample,immune-InfiltrationScore
ITIH4	Homo_723,female,AD.vs.control; Homo_723,male,AD.vs.control; Homo_723,female,AD.vs.MCI; Homo_723,male,AD.vs.MCI; Homo_723,female,MCI.vs.control; Homo_723,male,MCI.vs.control; Homo_714,female,AD.vs.control; Homo_714,male,AD.vs.control; Homo_714,female,AD.vs.MCI; Homo_714,male,AD.vs.MCI; Homo_714,female,MCI.vs.control; Homo_714,male,MCI.vs.control; Homo_633,female,AD.vs.control; Homo_633,female,AD.vs.MCI; Homo_633,female,MCI.vs.control; Homo_633,male,MCI.vs.control; MCSA,totalSample,age; MCSA,control,age; MCSA,MCI,age; Emory,totalSample,immune-DC; Emory,totalSample,immune-CD4_T; Emory,totalSample,immune-Tr1; Emory,totalSample,immune-iTreg; Emory,totalSample,immune-Th17; Emory,totalSample,immune-Central_memory; Emory,totalSample,immune-InfiltrationScore; Homo_723,totalSample,immune-Th2; Homo_723,totalSample,immune-Effector_memory; Homo_723,totalSample,immune-Tfh; Homo_723,totalSample,immune-iTreg; Homo_723,totalSample,immune-CD4_T; Homo_723,totalSample,immune-InfiltrationScore; Homo_723,totalSample,immune-nTreg; Homo_723,totalSample,immune-CD8_T; Homo_723,totalSample,immune-Macrophage; Homo_723,totalSample,immune-Bcell; Homo_723,totalSample,immune-CD4_naive; Homo_714,totalSample,immune-Th2; Homo_714,totalSample,immune-Tfh; Homo_714,totalSample,immune-Effector_memory; Homo_714,totalSample,immune-nTreg; Homo_714,totalSample,immune-CD4_T; Homo_714,totalSample,immune-Central_memory; Homo_714,totalSample,immune-InfiltrationScore; Homo_714,totalSample,immune-CD8_T; Homo_633,totalSample,immune-Th2; Homo_633,totalSample,immune-Effector_memory; Homo_633,totalSample,immune-nTreg; Homo_633,totalSample,immune-Tfh; Homo_633,totalSample,immune-InfiltrationScore; Homo_633,totalSample,immune-Central_memory; Homo_633,totalSample,immune-CD4_T; Homo_633,totalSample,immune-Th17; MCSA,totalSample,immune-DC; MCSA,totalSample,immune-Monocyte; MCSA,totalSample,immune-Neutrophil; MCSA,totalSample,immune-CD4_T; MCSA,totalSample,immune-CD8_T; MCSA,totalSample,immune-Gamma_delta; MCSA,totalSample,immune-CD4_naive; MCSA,totalSample,immune-Tr1; MCSA,totalSample,immune-nTreg; MCSA,totalSample,immune-Th2; MCSA,totalSample,immune-Th17; MCSA,totalSample,immune-Tfh; MCSA,totalSample,immune-Cytotoxic; MCSA,totalSample,immune-Central_memory; MCSA,totalSample,immune-Effector_memory; MCSA,totalSample,immune-InfiltrationScore; ROSMAP,totalSample,immune-DC; ROSMAP,totalSample,immune-CD4_T; ROSMAP,totalSample,immune-CD4_naive; ROSMAP,totalSample,immune-Tr1; ROSMAP,totalSample,immune-Th2; ROSMAP,totalSample,immune-Effector_memory; [illegible]
IRAG1	Homo_723,female,AD.vs.control; Homo_723,male,AD.vs.control; Homo_723,female,AD.vs.MCI; Homo_723,male,AD.vs.MCI; Homo_714,female,AD.vs.control; Homo_714,male,AD.vs.control; Homo_714,female,AD.vs.MCI; Homo_714,male,AD.vs.MCI; Homo_633,female,AD.vs.control; Homo_633,female,AD.vs.MCI; ROSMAP,female,AD.vs.control; ROSMAP,male,AD.vs.control; ROSMAP,female,MCI.vs.control; ROSMAP,AD,male.vs.female; Emory,MCI,age; Emory,totalSample,immune-Monocyte; Emory,totalSample,immune-Macrophage; Emory,totalSample,immune-NK; Emory,totalSample,immune-Neutrophil; Emory,totalSample,immune-CD4_T; Emory,totalSample,immune-CD8_T; Emory,totalSample,immune-Gamma_delta; Emory,totalSample,immune-Th1; Emory,totalSample,immune-Th17; Emory,totalSample,immune-Tfh; Emory,totalSample,immune-CD8_naive; Emory,totalSample,immune-Cytotoxic; Emory,totalSample,immune-Exhausted; Emory,totalSample,immune-MAIT; Emory,totalSample,immune-InfiltrationScore; Homo_723,totalSample,immune-Neutrophil; Homo_723,totalSample,immune-Monocyte; Homo_723,totalSample,immune-DC; Homo_723,totalSample,immune-Gamma_delta; Homo_723,totalSample,immune-Th1; Homo_723,totalSample,immune-Exhausted; Homo_723,totalSample,immune-Bcell; Homo_723,totalSample,immune-Th2; Homo_714,totalSample,immune-Neutrophil; Homo_714,totalSample,immune-Monocyte; Homo_714,totalSample,immune-Th2; Homo_714,totalSample,immune-Th1; Homo_714,totalSample,immune-Gamma_delta; Homo_714,totalSample,immune-Exhausted; Homo_633,totalSample,immune-Th2; Homo_633,totalSample,immune-Monocyte; Homo_633,totalSample,immune-Exhausted; Homo_633,totalSample,immune-Neutrophil; Homo_633,totalSample,immune-Th17; Homo_633,totalSample,immune-Gamma_delta; Homo_633,totalSample,immune-CD8_T; Homo_633,totalSample,immune-MAIT; Homo_633,totalSample,immune-Th1; MCSA,totalSample,immune-Neutrophil; MCSA,totalSample,immune-CD8_T; MCSA,totalSample,immune-NKT; MCSA,totalSample,immune-Gamma_delta; MCSA,totalSample,immune-Th1; MCSA,totalSample,immune-Th17; MCSA,totalSample,immune-Tfh; MCSA,totalSample,immune-Cytotoxic; MCSA,totalSample,immune-Exhausted; MCSA,totalSample,immune-MAIT; MCSA,totalSample,immune-Central_memory; MCSA,totalSample,immune-Effector_memory; ROSMAP,totalSample,immune-Effector_memory; [illegible]
DAB2	Homo_723,female,AD.vs.control; Homo_723,male,AD.vs.control; Homo_723,female,AD.vs.MCI; Homo_723,male,AD.vs.MCI; Homo_723,female,MCI.vs.control; Homo_723,male,MCI.vs.control; Homo_714,female,AD.vs.control; Homo_714,male,AD.vs.control; Homo_714,female,AD.vs.MCI; Homo_714,male,AD.vs.MCI; Homo_714,female,MCI.vs.control; Homo_714,male,MCI.vs.control; Homo_633,female,AD.vs.control; Homo_633,female,AD.vs.MCI; ROSMAP,MCI,last.visit.age; Emory,totalSample,immune-DC; Emory,totalSample,immune-Th1; Homo_723,totalSample,immune-DC; Homo_723,totalSample,immune-Monocyte; Homo_723,totalSample,immune-NK; Homo_723,totalSample,immune-Th17; Homo_723,totalSample,immune-Cytotoxic; Homo_723,totalSample,immune-InfiltrationScore; Homo_723,totalSample,immune-Central_memory; Homo_714,totalSample,immune-CD8_T; Homo_714,totalSample,immune-NK; Homo_714,totalSample,immune-NKT; Homo_714,totalSample,immune-Exhausted; Homo_714,totalSample,immune-DC; Homo_714,totalSample,immune-InfiltrationScore; Homo_714,totalSample,immune-Th1; Homo_714,totalSample,immune-Cytotoxic; Homo_633,totalSample,immune-InfiltrationScore; Homo_633,totalSample,immune-NKT; Homo_633,totalSample,immune-Monocyte; Homo_633,totalSample,immune-NK; MCSA,totalSample,immune-CD4_T; MCSA,totalSample,immune-CD8_T; MCSA,totalSample,immune-Gamma_delta; MCSA,totalSample,immune-CD4_naive; MCSA,totalSample,immune-Tr1; MCSA,totalSample,immune-nTreg; MCSA,totalSample,immune-iTreg; MCSA,totalSample,immune-Th17; MCSA,totalSample,immune-Tfh; MCSA,totalSample,immune-Cytotoxic; MCSA,totalSample,immune-Exhausted;
ZNF724	Homo_723,female,AD.vs.control; Homo_723,male,AD.vs.control; Homo_723,female,AD.vs.MCI; Homo_723,male,AD.vs.MCI; Homo_714,female,AD.vs.control; Homo_714,male,AD.vs.control; Homo_714,female,AD.vs.MCI; Homo_714,male,AD.vs.MCI; Homo_633,female,AD.vs.control; Homo_633,female,AD.vs.MCI; ROSMAP,AD,last.visit.age; ROSMAP,AD,sample.age; Emory,totalSample,immune-Macrophage; Emory,totalSample,immune-Neutrophil; Emory,totalSample,immune-CD4_T; Emory,totalSample,immune-CD8_T; Emory,totalSample,immune-Tr1; Emory,totalSample,immune-iTreg; Emory,totalSample,immune-Tfh; Emory,totalSample,immune-InfiltrationScore; Homo_723,totalSample,immune-CD4_naive; Homo_723,totalSample,immune-Central_memory; Homo_723,totalSample,immune-CD4_T; Homo_723,totalSample,immune-Th1; Homo_723,totalSample,immune-nTreg; Homo_723,totalSample,immune-Tr1; Homo_723,totalSample,immune-Tfh; Homo_723,totalSample,immune-Neutrophil; Homo_723,totalSample,immune-Cytotoxic; Homo_723,totalSample,immune-InfiltrationScore; Homo_723,totalSample,immune-NKT; Homo_723,totalSample,immune-iTreg; Homo_723,totalSample,immune-Monocyte; Homo_723,totalSample,immune-Th17; Homo_714,totalSample,immune-CD4_naive; Homo_714,totalSample,immune-NKT; Homo_714,totalSample,immune-Th1; Homo_714,totalSample,immune-InfiltrationScore; Homo_714,totalSample,immune-Central_memory; Homo_714,totalSample,immune-CD4_T; Homo_714,totalSample,immune-Tfh; Homo_714,totalSample,immune-iTreg; Homo_714,totalSample,immune-Tr1; Homo_714,totalSample,immune-nTreg; Homo_714,totalSample,immune-Neutrophil; Homo_714,totalSample,immune-Effector_memory; Homo_633,totalSample,immune-Tfh; Homo_633,totalSample,immune-CD4_naive; Homo_633,totalSample,immune-CD4_T; Homo_633,totalSample,immune-NKT; Homo_633,totalSample,immune-Central_memory; Homo_633,totalSample,immune-iTreg; Homo_633,totalSample,immune-Tr1; Homo_633,totalSample,immune-Effector_memory; Homo_633,totalSample,immune-InfiltrationScore; Homo_633,totalSample,immune-nTreg; Homo_633,totalSample,immune-Th1; MCSA,totalSample,immune-DC; MCSA,totalSample,immune-Macrophage; MCSA,totalSample,immune-CD4_T; MCSA,totalSample,immune-Gamma_delta; MCSA,totalSample,immune-CD4_naive; MCSA,totalSample,immune-Tr1; MCSA,totalSample,immune-nTreg; MCSA,totalSample,immune-iTreg; MCSA,totalSample,immune-Th17; MCSA,totalSample,immune-Tfh; MCSA,totalSample,immune-CD8_naive; MCSA,totalSample,immune-Cytotoxic; MCSA,totalSample,immune-Exhausted; MCSA,totalSample,immune-MAIT; MCSA,totalSample,immune-Central_memory; ROSMAP,totalSample,immune-InfiltrationScore; SRP223445,totalSample,immune-NK; [illegible]
PRSS36	Homo_723,female,AD.vs.control; Homo_723,male,AD.vs.control; Homo_723,female,AD.vs.MCI; Homo_723,male,AD.vs.MCI; Homo_714,female,AD.vs.control; Homo_714,male,AD.vs.control; Homo_714,female,AD.vs.MCI; Homo_714,male,AD.vs.MCI; Homo_633,female,AD.vs.control; Homo_633,female,AD.vs.MCI; ROSMAP,female,AD.vs.control; ROSMAP,male,AD.vs.control; ROSMAP,female,AD.vs.MCI; ACOM,totalSample,age; Emory,MCI,age; MCSA,MCI,age; Emory,totalSample,immune-Neutrophil; Emory,totalSample,immune-CD8_T; Emory,totalSample,immune-nTreg; Emory,totalSample,immune-Tfh; Emory,totalSample,immune-CD8_naive; Homo_723,totalSample,immune-Th1; Homo_723,totalSample,immune-Cytotoxic; Homo_723,totalSample,immune-InfiltrationScore; Homo_723,totalSample,immune-CD8_T; Homo_714,totalSample,immune-Th1; Homo_714,totalSample,immune-DC; Homo_714,totalSample,immune-NKT; Homo_714,totalSample,immune-Cytotoxic; Homo_633,totalSample,immune-Th1; Homo_633,totalSample,immune-DC; Homo_633,totalSample,immune-InfiltrationScore; Homo_633,totalSample,immune-NKT; MCSA,totalSample,immune-DC; MCSA,totalSample,immune-Monocyte; MCSA,totalSample,immune-Macrophage; MCSA,totalSample,immune-CD4_T; MCSA,totalSample,immune-CD8_T; MCSA,totalSample,immune-NKT; MCSA,totalSample,immune-Tr1;
PTGFRN	Homo_723,female,AD.vs.control; Homo_723,male,AD.vs.control; Homo_723,female,AD.vs.MCI; Homo_723,male,AD.vs.MCI; Homo_723,female,MCI.vs.control; Homo_633,female,AD.vs.control; Homo_633,female,AD.vs.MCI; Emory,MCI,age; ROSMAP,MCI,last.visit.age; Emory,totalSample,immune-CD8_T; Emory,totalSample,immune-Gamma_delta; Emory,totalSample,immune-Tfh; Emory,totalSample,immune-Cytotoxic; Emory,totalSample,immune-Exhausted; Emory,totalSample,immune-MAIT; Homo_723,totalSample,immune-Gamma_delta; Homo_723,totalSample,immune-Exhausted; Homo_714,totalSample,immune-Gamma_delta; Homo_714,totalSample,immune-Exhausted; Homo_633,totalSample,immune-Gamma_delta; Homo_633,totalSample,immune-Exhausted; Homo_633,totalSample,immune-Th2; Homo_633,totalSample,immune-Tfh; MCSA,totalSample,immune-CD4_T; MCSA,totalSample,immune-Gamma_delta; MCSA,totalSample,immune-CD4_naive; MCSA,totalSample,immune-Tr1; MCSA,totalSample,immune-nTreg; MCSA,totalSample,immune-iTreg; MCSA,totalSample,immune-Th17; MCSA,totalSample,immune-Tfh; MCSA,totalSample,immune-Exhausted; MCSA,totalSample,immune-Central_memory; ROSMAP,totalSample,immune-InfiltrationScore; SRP223445,totalSample,immune-InfiltrationScore
PTPRS	Homo_723,female,AD.vs.control; Homo_723,male,AD.vs.control; Homo_723,female,AD.vs.MCI; Homo_723,male,AD.vs.MCI; Homo_723,female,MCI.vs.control; Homo_723,male,MCI.vs.control; Homo_714,female,AD.vs.control; Homo_714,male,AD.vs.control; Homo_714,female,AD.vs.MCI; Homo_714,male,AD.vs.MCI; Homo_714,female,MCI.vs.control; Homo_714,male,MCI.vs.control; Homo_633,female,AD.vs.control; Homo_633,female,AD.vs.MCI; ROSMAP,female,AD.vs.control; ROSMAP,MCI,male.vs.female; Emory,totalSample,age; Emory,MCI,age; MCSA,totalSample,age; MCSA,control,age; Emory,totalSample,immune-CD8_T; Emory,totalSample,immune-Gamma_delta; Emory,totalSample,immune-Tfh; Homo_723,totalSample,immune-DC; Homo_723,totalSample,immune-Th17; Homo_723,totalSample,immune-MAIT; Homo_714,totalSample,immune-DC; Homo_714,totalSample,immune-Th17; Homo_633,totalSample,immune-DC; Homo_633,totalSample,immune-InfiltrationScore; MCSA,totalSample,immune-DC; MCSA,totalSample,immune-Monocyte; MCSA,totalSample,immune-Th17; MCSA,totalSample,immune-Cytotoxic; MCSA,totalSample,immune-Effector_memory; MCSA,totalSample,immune-InfiltrationScore; SRP223445,totalSample,immune-Exhausted
ARFGAP2	Homo_723,female,AD.vs.control; Homo_723,male,AD.vs.control; Homo_723,male,AD.vs.MCI; Homo_723,female,MCI.vs.control; Homo_723,control,male.vs.female; Homo_723,MCI,male.vs.female; Homo_714,female,AD.vs.control; Homo_714,male,AD.vs.control; Homo_714,female,AD.vs.MCI; Homo_714,male,AD.vs.MCI; Homo_714,female,MCI.vs.control; Homo_714,control,male.vs.female; Homo_633,female,AD.vs.control; Homo_633,female,AD.vs.MCI; Homo_633,female,MCI.vs.control; Homo_633,control,male.vs.female; Emory,MCI,age; MCSA,totalSample,age; MCSA,control,age; MCSA,MCI,age; ROSMAP,MCI,sample.age; Emory,totalSample,immune-Monocyte; Emory,totalSample,immune-NK; Emory,totalSample,immune-CD4_T; Emory,totalSample,immune-Tr1; Emory,totalSample,immune-iTreg; Emory,totalSample,immune-InfiltrationScore; Homo_723,totalSample,immune-Monocyte; Homo_723,totalSample,immune-DC; Homo_723,totalSample,immune-CD4_naive; Homo_723,totalSample,immune-Central_memory; Homo_723,totalSample,immune-nTreg; Homo_723,totalSample,immune-InfiltrationScore; Homo_723,totalSample,immune-Neutrophil; Homo_714,totalSample,immune-Monocyte; Homo_714,totalSample,immune-InfiltrationScore; Homo_714,totalSample,immune-CD4_naive; Homo_714,totalSample,immune-DC; Homo_714,totalSample,immune-nTreg; Homo_633,totalSample,immune-Monocyte; Homo_633,totalSample,immune-InfiltrationScore; Homo_633,totalSample,immune-nTreg; Homo_633,totalSample,immune-CD4_naive; MCSA,totalSample,immune-DC; MCSA,totalSample,immune-Monocyte; MCSA,totalSample,immune-Neutrophil; MCSA,totalSample,immune-CD4_T; MCSA,totalSample,immune-CD8_T; MCSA,totalSample,immune-NKT; MCSA,totalSample,immune-Gamma_delta; MCSA,totalSample,immune-CD4_naive; MCSA,totalSample,immune-Tr1; MCSA,totalSample,immune-nTreg; MCSA,totalSample,immune-Th2; MCSA,totalSample,immune-Th17; MCSA,totalSample,immune-Tfh; MCSA,totalSample,immune-Cytotoxic; MCSA,totalSample,immune-Central_memory; MCSA,totalSample,immune-Effector_memory; MCSA,totalSample,immune-InfiltrationScore; ROSMAP,totalSample,immune-Effector_memory; SRP223445,totalSample,immune-NK; SRP223445,totalSample,immune-Neutrophil; SRP223445,totalSample,immune-CD4_naive; SRP223445,totalSample,immune-Th17; SRP223445,totalSample,immune-Exhausted; SRP223445,totalSample,immune-[illegible]

UNC50	Homo_723,female,AD.vs.control; Homo_723,male,AD.vs.control; Homo_723,female,AD.vs.MCI; Homo_723,male,AD.vs.MCI; Homo_714,female,AD.vs.control; Homo_714,male,AD.vs.control; Homo_714,female,AD.vs.MCI; Homo_714,male,AD.vs.MCI; Homo_633,female,AD.vs.control; Homo_633,female,AD.vs.MCI; MCSA,totalSample,age; MCSA,MCI,age; ROSMAP,MCI,last.visit.age; Emory,totalSample,immune-DC; Emory,totalSample,immune-Monocyte; Emory,totalSample,immune-Macrophage; Emory,totalSample,immune-CD4_T; Emory,totalSample,immune-Tr1; Emory,totalSample,immune-iTreg; Emory,totalSample,immune-Effector_memory; Emory,totalSample,immune-InfiltrationScore; Homo_723,totalSample,immune-Th2; Homo_723,totalSample,immune-Monocyte; Homo_723,totalSample,immune-DC; Homo_723,totalSample,immune-Tfh; Homo_723,totalSample,immune-iTreg; Homo_723,totalSample,immune-Effector_memory; Homo_723,totalSample,immune-CD4_T; Homo_723,totalSample,immune-Tr1; Homo_723,totalSample,immune-InfiltrationScore; Homo_723,totalSample,immune-Bcell; Homo_714,totalSample,immune-Th2; Homo_714,totalSample,immune-Tfh; Homo_714,totalSample,immune-Monocyte; Homo_714,totalSample,immune-Effector_memory; Homo_714,totalSample,immune-CD4_T; Homo_714,totalSample,immune-Tr1; Homo_714,totalSample,immune-DC; Homo_633,totalSample,immune-Monocyte; Homo_633,totalSample,immune-Th2; Homo_633,totalSample,immune-Tfh; Homo_633,totalSample,immune-InfiltrationScore; Homo_633,totalSample,immune-CD4_T; Homo_633,totalSample,immune-Exhausted; Homo_633,totalSample,immune-Effector_memory; Homo_633,totalSample,immune-Bcell; Homo_633,totalSample,immune-Central_memory; Homo_633,totalSample,immune-CD4_naive; MCSA,totalSample,immune-DC; MCSA,totalSample,immune-Monocyte; MCSA,totalSample,immune-Macrophage; MCSA,totalSample,immune-CD4_T; MCSA,totalSample,immune-CD8_T; MCSA,totalSample,immune-Tr1; MCSA,totalSample,immune-iTreg; MCSA,totalSample,immune-Th2; MCSA,totalSample,immune-CD8_naive; MCSA,totalSample,immune-Cytotoxic; MCSA,totalSample,immune-Exhausted; MCSA,totalSample,immune-MAIT; ROSMAP,totalSample,immune-Th17; ROSMAP,totalSample,immune-Effector_memory
PAWR	Homo_723,female,AD.vs.control; Homo_723,male,AD.vs.control; Homo_723,female,AD.vs.MCI; Homo_723,male,AD.vs.MCI; Homo_723,male,MCI.vs.control; Homo_714,female,AD.vs.control; Homo_714,male,AD.vs.control; Homo_714,female,AD.vs.MCI; Homo_714,male,AD.vs.MCI; Homo_633,female,AD.vs.control; Homo_633,female,AD.vs.MCI; Emory,totalSample,immune-B_cell; Emory,totalSample,immune-Macrophage; Emory,totalSample,immune-Neutrophil; Emory,totalSample,immune-CD4_T; Emory,totalSample,immune-Gamma_delta; Emory,totalSample,immune-Tr1; Emory,totalSample,immune-iTreg; Emory,totalSample,immune-CD8_naive; Emory,totalSample,immune-InfiltrationScore; Homo_723,totalSample,immune-CD4_naive; Homo_723,totalSample,immune-CD4_T; Homo_723,totalSample,immune-Th1; Homo_723,totalSample,immune-Th17; Homo_723,totalSample,immune-Central_memory; Homo_723,totalSample,immune-Cytotoxic; Homo_723,totalSample,immune-Tr1; Homo_723,totalSample,immune-nTreg; Homo_723,totalSample,immune-Tfh; Homo_723,totalSample,immune-iTreg; Homo_723,totalSample,immune-Neutrophil; Homo_723,totalSample,immune-Gamma_delta; Homo_723,totalSample,immune-Th2; Homo_723,totalSample,immune-Exhausted; Homo_723,totalSample,immune-NKT; Homo_714,totalSample,immune-Th1; Homo_714,totalSample,immune-NKT; Homo_714,totalSample,immune-CD4_naive; Homo_714,totalSample,immune-CD4_T; Homo_714,totalSample,immune-Central_memory; Homo_714,totalSample,immune-Tfh; Homo_714,totalSample,immune-Th17; Homo_714,totalSample,immune-Tr1; Homo_714,totalSample,immune-Gamma_delta; Homo_714,totalSample,immune-Cytotoxic; Homo_714,totalSample,immune-Exhausted; Homo_714,totalSample,immune-iTreg; Homo_714,totalSample,immune-nTreg; Homo_633,totalSample,immune-Tfh; Homo_633,totalSample,immune-CD4_T; Homo_633,totalSample,immune-NKT; Homo_633,totalSample,immune-Tr1; Homo_633,totalSample,immune-CD4_naive; Homo_633,totalSample,immune-Central_memory; Homo_633,totalSample,immune-Gamma_delta; Homo_633,totalSample,immune-iTreg; Homo_633,totalSample,immune-Th1; Homo_633,totalSample,immune-Th2; Homo_633,totalSample,immune-nTreg; Homo_633,totalSample,immune-Effector_memory; Homo_633,totalSample,immune-Th17; Homo_633,totalSample,immune-Cytotoxic; MCSA,totalSample,immune-DC; MCSA,totalSample,immune-Bcell; MCSA,totalSample,immune-Macrophage; MCSA,totalSample,immune-CD4_T; MCSA,totalSample,immune-Gamma_delta; MCSA,totalSample,immune-CD4_naive; MCSA,totalSample,immune-Tr1; MCSA,totalSample,immune-nTreg; MCSA,totalSample,immune-iTreg; MCSA,totalSample,immune-Th17; MCSA,totalSample,immune-Tfh; MCSA,totalSample,immune-[illegible]
CDKL3	Homo_723,female,AD.vs.control; Homo_723,male,AD.vs.control; Homo_723,female,AD.vs.MCI; Homo_723,male,AD.vs.MCI; Homo_723,female,MCI.vs.control; Homo_723,male,MCI.vs.control; Homo_714,female,AD.vs.control; Homo_714,male,AD.vs.control; Homo_714,female,AD.vs.MCI; Homo_714,male,AD.vs.MCI; Homo_714,female,MCI.vs.control; Homo_714,male,MCI.vs.control; Homo_633,female,AD.vs.control; Homo_633,female,AD.vs.MCI; Homo_633,female,MCI.vs.control; Homo_633,male,MCI.vs.control; ROSMAP,female,AD.vs.control; MCSA,MCI,age; Emory,totalSample,immune-Gamma_delta; Emory,totalSample,immune-Tr1; Emory,totalSample,immune-iTreg; Homo_723,totalSample,immune-Th1; Homo_723,totalSample,immune-Monocyte; Homo_723,totalSample,immune-Central_memory; Homo_723,totalSample,immune-Neutrophil; Homo_714,totalSample,immune-Th1; Homo_714,totalSample,immune-NKT; Homo_633,totalSample,immune-Th1; Homo_633,totalSample,immune-NKT; Homo_633,totalSample,immune-Monocyte; Homo_633,totalSample,immune-InfiltrationScore; MCSA,totalSample,immune-DC; MCSA,totalSample,immune-Macrophage; MCSA,totalSample,immune-Neutrophil; MCSA,totalSample,immune-CD4_T; MCSA,totalSample,immune-CD4_naive; MCSA,totalSample,immune-Tr1; MCSA,totalSample,immune-nTreg; MCSA,totalSample,immune-iTreg; MCSA,totalSample,immune-Th2; MCSA,totalSample,immune-Tfh; MCSA,totalSample,immune-CD8_naive; MCSA,totalSample,immune-MAIT; MCSA,totalSample,immune-Central_memory; MCSA,totalSample,immune-Effector_memory
TMEM216	Homo_723,female,AD.vs.control; Homo_723,male,AD.vs.control; Homo_723,female,AD.vs.MCI; Homo_723,male,AD.vs.MCI; Homo_714,female,AD.vs.control; Homo_714,male,AD.vs.control; Homo_714,female,AD.vs.MCI; Homo_714,male,AD.vs.MCI; Homo_633,female,AD.vs.control; Homo_633,female,AD.vs.MCI; MCSA,totalSample,age; MCSA,control,age; MCSA,MCI,age; ROSMAP,MCI,last.visit.age; ROSMAP,MCI,sample.age; Emory,totalSample,immune-DC; Emory,totalSample,immune-Monocyte; Emory,totalSample,immune-Gamma_delta; Emory,totalSample,immune-Tr1; Emory,totalSample,immune-iTreg; Homo_723,totalSample,immune-iTreg; Homo_723,totalSample,immune-InfiltrationScore; Homo_723,totalSample,immune-Tfh; Homo_723,totalSample,immune-Tr1; Homo_723,totalSample,immune-Th2; Homo_723,totalSample,immune-CD4_T; Homo_723,totalSample,immune-nTreg; Homo_723,totalSample,immune-CD8_T; Homo_723,totalSample,immune-Central_memory; Homo_723,totalSample,immune-CD8_naive; Homo_723,totalSample,immune-Gamma_delta; Homo_723,totalSample,immune-CD4_naive; Homo_714,totalSample,immune-Tfh; Homo_714,totalSample,immune-InfiltrationScore; Homo_714,totalSample,immune-iTreg; Homo_714,totalSample,immune-Tr1; Homo_714,totalSample,immune-Th2; Homo_714,totalSample,immune-Effector_memory; Homo_714,totalSample,immune-CD4_T; Homo_714,totalSample,immune-nTreg; Homo_633,totalSample,immune-InfiltrationScore; Homo_633,totalSample,immune-iTreg; Homo_633,totalSample,immune-Tfh; Homo_633,totalSample,immune-nTreg; Homo_633,totalSample,immune-NKT; Homo_633,totalSample,immune-Tr1; Homo_633,totalSample,immune-CD4_T; Homo_633,totalSample,immune-CD4_naive; Homo_633,totalSample,immune-Effector_memory; MCSA,totalSample,immune-Monocyte; MCSA,totalSample,immune-CD4_T; MCSA,totalSample,immune-Gamma_delta; MCSA,totalSample,immune-CD4_naive; MCSA,totalSample,immune-Tr1; MCSA,totalSample,immune-nTreg; MCSA,totalSample,immune-iTreg; MCSA,totalSample,immune-Th17; MCSA,totalSample,immune-Tfh; MCSA,totalSample,immune-Exhausted; MCSA,totalSample,immune-Central_memory; MCSA,totalSample,immune-Effector_memory; MCSA,totalSample,immune-InfiltrationScore; ROSMAP,totalSample,immune-Th17; ROSMAP,totalSample,immune-Effector_memory; SRP223445,totalSample,immune-CD4_naive; [illegible]
GZMH	Homo_723,female,AD.vs.control; Homo_723,male,AD.vs.control; Homo_723,female,AD.vs.MCI; Homo_723,male,AD.vs.MCI; Homo_714,control,male.vs.female; Homo_633,control,male.vs.female; ROSMAP,female,MCI.vs.control; MCSA,totalSample,age; MCSA,MCI,age; ROSMAP,AD,sample.age; Emory,totalSample,immune-NK; Emory,totalSample,immune-Neutrophil; Emory,totalSample,immune-CD8_T; Emory,totalSample,immune-Gamma_delta; Emory,totalSample,immune-CD4_naive; Emory,totalSample,immune-nTreg; Emory,totalSample,immune-Th17; Emory,totalSample,immune-Cytotoxic; Emory,totalSample,immune-Exhausted; Emory,totalSample,immune-Central_memory; Emory,totalSample,immune-Effector_memory; Emory,totalSample,immune-InfiltrationScore; Homo_723,totalSample,immune-Cytotoxic; Homo_723,totalSample,immune-InfiltrationScore; Homo_723,totalSample,immune-MAIT; Homo_723,totalSample,immune-Effector_memory; Homo_723,totalSample,immune-Th17; Homo_723,totalSample,immune-Gamma_delta; Homo_723,totalSample,immune-Macrophage; Homo_723,totalSample,immune-CD8_T; Homo_723,totalSample,immune-Th1; Homo_723,totalSample,immune-Exhausted; Homo_723,totalSample,immune-Bcell; Homo_714,totalSample,immune-Cytotoxic; Homo_714,totalSample,immune-Gamma_delta; Homo_714,totalSample,immune-InfiltrationScore; Homo_714,totalSample,immune-CD8_T; Homo_714,totalSample,immune-Exhausted; Homo_714,totalSample,immune-MAIT; Homo_714,totalSample,immune-Th17; Homo_714,totalSample,immune-Neutrophil; Homo_714,totalSample,immune-Th2; Homo_714,totalSample,immune-Bcell; Homo_714,totalSample,immune-Macrophage; Homo_714,totalSample,immune-iTreg; Homo_714,totalSample,immune-Tr1; Homo_714,totalSample,immune-Effector_memory; Homo_714,totalSample,immune-nTreg; Homo_633,totalSample,immune-Cytotoxic; Homo_633,totalSample,immune-Gamma_delta; Homo_633,totalSample,immune-InfiltrationScore; Homo_633,totalSample,immune-CD8_T; Homo_633,totalSample,immune-Th17; Homo_633,totalSample,immune-MAIT; Homo_633,totalSample,immune-Th2; Homo_633,totalSample,immune-Exhausted; Homo_633,totalSample,immune-Neutrophil; Homo_633,totalSample,immune-Macrophage; Homo_633,totalSample,immune-iTreg; Homo_633,totalSample,immune-Bcell; Homo_633,totalSample,immune-Tr1; Homo_633,totalSample,immune-nTreg; Homo_633,totalSample,immune-CD4_T; Homo_633,totalSample,immune-Effector_memory; MCSA,totalSample,immune-Neutrophil; MCSA,totalSample,immune-CD8_T; MCSA,totalSample,immune-Gamma_delta; MCSA,totalSample,immune-Th17; MCSA,totalSample,immune-Cytotoxic; MCSA,totalSample,immune-MAIT; MCSA,totalSample,immune-Effector_memory; MCSA,totalSample,immune-InfiltrationScore; ROSMAP,totalSample,immune-Bcell; ROSMAP,totalSample,immune-Monocyte; ROSMAP,totalSample,immune-Macrophage; ROSMAP,totalSample,immune-Neutrophil; ROSMAP,totalSample,immune-CD4_T; ROSMAP,totalSample,immune-CD8_T; ROSMAP,totalSample,immune-Gamma_delta; ROSMAP,totalSample,immune-Tr1; ROSMAP,totalSample,immune-nTreg; ROSMAP,totalSample,immune-iTreg; ROSMAP,totalSample,immune-Th1; ROSMAP,totalSample,immune-Th2; ROSMAP,totalSample,immune-Th17; ROSMAP,totalSample,immune-Cytotoxic; ROSMAP,totalSample,immune-InfiltrationScore; SRP223445,totalSample,immune-Neutrophil; SRP223445,totalSample,immune-CD8_T; [illegible]
IL24	Homo_723,female,AD.vs.control; Homo_723,male,AD.vs.control; Homo_723,female,AD.vs.MCI; Homo_723,male,AD.vs.MCI; Homo_723,female,MCI.vs.control; Homo_723,male,MCI.vs.control; Homo_714,male,MCI.vs.control; Homo_714,MCI,male.vs.female; ROSMAP,female,AD.vs.control; MCSA,totalSample,age; MCSA,control,age; MCSA,MCI,age; Emory,totalSample,immune-Monocyte; Emory,totalSample,immune-Macrophage; Emory,totalSample,immune-Neutrophil; Emory,totalSample,immune-CD4_T; Emory,totalSample,immune-CD8_T; Emory,totalSample,immune-CD4_naive; Emory,totalSample,immune-Tr1; Emory,totalSample,immune-nTreg; Emory,totalSample,immune-iTreg; Emory,totalSample,immune-Central_memory; Emory,totalSample,immune-InfiltrationScore; Homo_723,totalSample,immune-Th2; Homo_723,totalSample,immune-Tfh; Homo_723,totalSample,immune-iTreg; Homo_723,totalSample,immune-DC; Homo_723,totalSample,immune-Macrophage; Homo_723,totalSample,immune-CD4_T; Homo_723,totalSample,immune-Monocyte; Homo_723,totalSample,immune-Effector_memory; Homo_723,totalSample,immune-Tr1; Homo_723,totalSample,immune-CD8_T; Homo_723,totalSample,immune-CD4_naive; Homo_714,totalSample,immune-nTreg; Homo_714,totalSample,immune-CD4_T; Homo_714,totalSample,immune-iTreg; Homo_714,totalSample,immune-CD4_naive; Homo_714,totalSample,immune-Tr1; Homo_714,totalSample,immune-Macrophage; Homo_714,totalSample,immune-Th2; Homo_714,totalSample,immune-Th1; Homo_714,totalSample,immune-Monocyte; Homo_714,totalSample,immune-Tfh; Homo_714,totalSample,immune-Central_memory; Homo_633,totalSample,immune-nTreg; Homo_633,totalSample,immune-CD4_T; Homo_633,totalSample,immune-Tr1; Homo_633,totalSample,immune-iTreg; Homo_633,totalSample,immune-CD4_naive; Homo_633,totalSample,immune-Tfh; Homo_633,totalSample,immune-Central_memory; Homo_633,totalSample,immune-Monocyte; Homo_633,totalSample,immune-Macrophage; Homo_633,totalSample,immune-Th1; Homo_633,totalSample,immune-Effector_memory; MCSA,totalSample,immune-Monocyte; MCSA,totalSample,immune-Macrophage; MCSA,totalSample,immune-Neutrophil; MCSA,totalSample,immune-CD4_T; MCSA,totalSample,immune-CD8_T; MCSA,totalSample,immune-CD4_naive; MCSA,totalSample,immune-Tr1; MCSA,totalSample,immune-nTreg; MCSA,totalSample,immune-iTreg; MCSA,totalSample,immune-[illegible]
ACVR2A	Homo_723,female,AD.vs.control; Homo_723,male,AD.vs.control; Homo_723,female,AD.vs.MCI; Homo_723,male,AD.vs.MCI; Homo_723,female,MCI.vs.control; Homo_723,male,MCI.vs.control; Homo_714,female,AD.vs.control; Homo_714,male,AD.vs.control; Homo_714,female,AD.vs.MCI; Homo_714,male,AD.vs.MCI; Homo_714,female,MCI.vs.control; Homo_714,male,MCI.vs.control; Homo_633,female,AD.vs.control; Homo_633,female,AD.vs.MCI; MCSA,MCI,age; ROSMAP,MCI,sample.age; Emory,totalSample,immune-Monocyte; Emory,totalSample,immune-CD4_T; Emory,totalSample,immune-Tr1; Emory,totalSample,immune-iTreg; Emory,totalSample,immune-Tfh; Emory,totalSample,immune-InfiltrationScore; Homo_723,totalSample,immune-Exhausted; Homo_723,totalSample,immune-Th17; Homo_723,totalSample,immune-CD4_naive; Homo_723,totalSample,immune-CD4_T; Homo_723,totalSample,immune-Cytotoxic; Homo_723,totalSample,immune-Tfh; Homo_723,totalSample,immune-Th2; Homo_723,totalSample,immune-Tr1; Homo_723,totalSample,immune-CD8_T; Homo_723,totalSample,immune-Central_memory; Homo_723,totalSample,immune-Monocyte; Homo_723,totalSample,immune-nTreg; Homo_723,totalSample,immune-Th1; Homo_723,totalSample,immune-Effector_memory; Homo_714,totalSample,immune-Th17; Homo_714,totalSample,immune-CD4_naive; Homo_714,totalSample,immune-CD4_T; Homo_714,totalSample,immune-Exhausted; Homo_714,totalSample,immune-Central_memory; Homo_714,totalSample,immune-Tfh; Homo_714,totalSample,immune-Monocyte; Homo_714,totalSample,immune-Th2; Homo_714,totalSample,immune-Tr1; Homo_633,totalSample,immune-Tfh; Homo_633,totalSample,immune-Th2; Homo_633,totalSample,immune-CD4_T; Homo_633,totalSample,immune-CD4_naive; Homo_633,totalSample,immune-Effector_memory; Homo_633,totalSample,immune-Central_memory; Homo_633,totalSample,immune-Tr1; Homo_633,totalSample,immune-Th17; Homo_633,totalSample,immune-Monocyte; Homo_633,totalSample,immune-Exhausted; Homo_633,totalSample,immune-Gamma_delta; Homo_633,totalSample,immune-iTreg; Homo_633,totalSample,immune-InfiltrationScore; Homo_633,totalSample,immune-nTreg; Homo_633,totalSample,immune-Bcell; Homo_633,totalSample,immune-DC; MCSA,totalSample,immune-DC; MCSA,totalSample,immune-Macrophage; MCSA,totalSample,immune-CD4_T; MCSA,totalSample,immune-CD8_T; MCSA,totalSample,immune-Gamma_delta; MCSA,totalSample,immune-CD4_naive; MCSA,totalSample,immune-Tr1; MCSA,totalSample,immune-nTreg; MCSA,totalSample,immune-iTreg; MCSA,totalSample,immune-Th17; MCSA,totalSample,immune-Tfh; MCSA,totalSample,immune-CD8_naive; MCSA,totalSample,immune-Cytotoxic; MCSA,totalSample,immune-[illegible]
PDCD1	Homo_723,female,AD.vs.control; Homo_723,male,AD.vs.control; Homo_723,female,AD.vs.MCI; Homo_723,male,AD.vs.MCI; Homo_723,female,MCI.vs.control; Homo_723,male,MCI.vs.control; Homo_714,female,AD.vs.control; Homo_714,male,AD.vs.control; Homo_714,female,AD.vs.MCI; Homo_714,male,AD.vs.MCI; Homo_714,female,MCI.vs.control; Homo_714,male,MCI.vs.control; Homo_633,female,AD.vs.control; Homo_633,female,AD.vs.MCI; Homo_633,female,MCI.vs.control; Homo_633,male,MCI.vs.control; MCSA,totalSample,age; MCSA,control,age; MCSA,MCI,age; Emory,totalSample,immune-Neutrophil; Emory,totalSample,immune-CD8_T; Emory,totalSample,immune-Gamma_delta; Emory,totalSample,immune-CD4_naive; Emory,totalSample,immune-Cytotoxic; Emory,totalSample,immune-Exhausted; Emory,totalSample,immune-Effector_memory; Emory,totalSample,immune-InfiltrationScore; Homo_723,totalSample,immune-Exhausted; Homo_723,totalSample,immune-CD8_T; Homo_723,totalSample,immune-Cytotoxic; Homo_723,totalSample,immune-Monocyte; Homo_723,totalSample,immune-Th17; Homo_723,totalSample,immune-MAIT; Homo_723,totalSample,immune-DC; Homo_723,totalSample,immune-InfiltrationScore; Homo_714,totalSample,immune-Monocyte; Homo_714,totalSample,immune-Exhausted; Homo_714,totalSample,immune-CD8_T; Homo_714,totalSample,immune-Th17; Homo_714,totalSample,immune-Cytotoxic; Homo_714,totalSample,immune-DC; Homo_714,totalSample,immune-InfiltrationScore; Homo_714,totalSample,immune-Macrophage; Homo_633,totalSample,immune-Monocyte; Homo_633,totalSample,immune-Th2; Homo_633,totalSample,immune-DC; Homo_633,totalSample,immune-InfiltrationScore; Homo_633,totalSample,immune-Th17; Homo_633,totalSample,immune-Exhausted; Homo_633,totalSample,immune-CD8_T; Homo_633,totalSample,immune-Cytotoxic; Homo_633,totalSample,immune-Neutrophil; MCSA,totalSample,immune-DC; MCSA,totalSample,immune-Monocyte; MCSA,totalSample,immune-Neutrophil; MCSA,totalSample,immune-CD8_T; MCSA,totalSample,immune-NKT; MCSA,totalSample,immune-iTreg; MCSA,totalSample,immune-Th2; MCSA,totalSample,immune-Cytotoxic; MCSA,totalSample,immune-Exhausted; MCSA,totalSample,immune-Central_memory; MCSA,totalSample,immune-Effector_memory; MCSA,totalSample,immune-InfiltrationScore; ROSMAP,totalSample,immune-Exhausted; [illegible]
LAIR2	Homo_723,female,AD.vs.control; Homo_723,male,AD.vs.control; Homo_723,female,AD.vs.MCI; Homo_723,male,AD.vs.MCI; Homo_714,female,AD.vs.control; Homo_714,male,AD.vs.control; Homo_714,female,AD.vs.MCI; Homo_714,male,AD.vs.MCI; Homo_633,female,AD.vs.control; Homo_633,female,AD.vs.MCI; ROSMAP,female,AD.vs.control; Emory,totalSample,immune-CD4_naive; Emory,totalSample,immune-Cytotoxic; Emory,totalSample,immune-Effector_memory; Homo_723,totalSample,immune-Cytotoxic; Homo_723,totalSample,immune-InfiltrationScore; Homo_723,totalSample,immune-CD8_T; Homo_723,totalSample,immune-Th17; Homo_723,totalSample,immune-Effector_memory; Homo_723,totalSample,immune-MAIT; Homo_714,totalSample,immune-InfiltrationScore; Homo_714,totalSample,immune-Cytotoxic; Homo_714,totalSample,immune-CD8_T; Homo_714,totalSample,immune-Effector_memory; Homo_714,totalSample,immune-Th17; Homo_633,totalSample,immune-InfiltrationScore; Homo_633,totalSample,immune-Neutrophil; Homo_633,totalSample,immune-Cytotoxic; Homo_633,totalSample,immune-NK; MCSA,totalSample,immune-Neutrophil; MCSA,totalSample,immune-CD8_T; MCSA,totalSample,immune-Cytotoxic; MCSA,totalSample,immune-Effector_memory; MCSA,totalSample,immune-InfiltrationScore; SRP223445,totalSample,immune-Th17; ROSMAP,male-includeAge90+,survival
HSBP1L1	Homo_723,female,AD.vs.control; Homo_723,male,AD.vs.control; Homo_723,female,AD.vs.MCI; Homo_723,male,AD.vs.MCI; Homo_714,female,AD.vs.control; Homo_714,male,AD.vs.control; Homo_714,female,AD.vs.MCI; Homo_714,male,AD.vs.MCI; Homo_633,female,AD.vs.control; Homo_633,female,AD.vs.MCI; ROSMAP,female,AD.vs.control; MCSA,totalSample,age; MCSA,control,age; Emory,totalSample,immune-CD4_T; Emory,totalSample,immune-Tr1; Emory,totalSample,immune-iTreg; Emory,totalSample,immune-Tfh; Emory,totalSample,immune-MAIT; Emory,totalSample,immune-Central_memory; Homo_723,totalSample,immune-Tfh; Homo_723,totalSample,immune-iTreg; Homo_723,totalSample,immune-Th2; Homo_723,totalSample,immune-CD4_T; Homo_723,totalSample,immune-Central_memory; Homo_723,totalSample,immune-Tr1; Homo_723,totalSample,immune-CD8_T; Homo_723,totalSample,immune-nTreg; Homo_723,totalSample,immune-CD4_naive; Homo_723,totalSample,immune-InfiltrationScore; Homo_723,totalSample,immune-Effector_memory; Homo_723,totalSample,immune-Macrophage; Homo_714,totalSample,immune-Tfh; Homo_714,totalSample,immune-Th2; Homo_714,totalSample,immune-Tr1; Homo_714,totalSample,immune-Central_memory; Homo_714,totalSample,immune-CD4_T; Homo_714,totalSample,immune-nTreg; Homo_714,totalSample,immune-Effector_memory; Homo_714,totalSample,immune-iTreg; Homo_714,totalSample,immune-CD4_naive; Homo_633,totalSample,immune-Tfh; Homo_633,totalSample,immune-Central_memory; Homo_633,totalSample,immune-nTreg; Homo_633,totalSample,immune-CD4_T; Homo_633,totalSample,immune-Tr1; Homo_633,totalSample,immune-CD4_naive; Homo_633,totalSample,immune-iTreg; Homo_633,totalSample,immune-Th2; Homo_633,totalSample,immune-Effector_memory; Homo_633,totalSample,immune-InfiltrationScore; MCSA,totalSample,immune-Monocyte; MCSA,totalSample,immune-Neutrophil; MCSA,totalSample,immune-CD4_T; MCSA,totalSample,immune-CD4_naive; MCSA,totalSample,immune-Tr1; MCSA,totalSample,immune-nTreg; MCSA,totalSample,immune-iTreg; MCSA,totalSample,immune-Th17; MCSA,totalSample,immune-Tfh; MCSA,totalSample,immune-Central_memory; MCSA,totalSample,immune-Effector_memory; SRP223445,totalSample,immune-InfiltrationScore; ROSMAP,male-excludeAge90+,survival
NEO1	Homo_723,female,AD.vs.control; Homo_723,male,AD.vs.control; Homo_723,female,AD.vs.MCI; Homo_723,female,MCI.vs.control; Homo_714,female,AD.vs.control; Homo_714,male,AD.vs.control; Homo_714,female,MCI.vs.control; Homo_633,female,AD.vs.control; Homo_633,female,AD.vs.MCI; Homo_633,female,MCI.vs.control; MCSA,totalSample,age; MCSA,control,age; ROSMAP,MCI,last.visit.age; Emory,totalSample,immune-Monocyte; Emory,totalSample,immune-Macrophage; Emory,totalSample,immune-Neutrophil; Emory,totalSample,immune-CD4_T; Emory,totalSample,immune-CD8_T; Emory,totalSample,immune-Tr1; Emory,totalSample,immune-iTreg; Emory,totalSample,immune-MAIT; Emory,totalSample,immune-InfiltrationScore; Homo_723,totalSample,immune-Th2; Homo_723,totalSample,immune-Tfh; Homo_723,totalSample,immune-CD4_T; Homo_723,totalSample,immune-CD4_naive; Homo_723,totalSample,immune-iTreg; Homo_723,totalSample,immune-Effector_memory; Homo_723,totalSample,immune-nTreg; Homo_723,totalSample,immune-Tr1; Homo_723,totalSample,immune-InfiltrationScore; Homo_723,totalSample,immune-Central_memory; Homo_723,totalSample,immune-Macrophage; Homo_723,totalSample,immune-Th17; Homo_714,totalSample,immune-Tfh; Homo_714,totalSample,immune-Th2; Homo_714,totalSample,immune-Effector_memory; Homo_714,totalSample,immune-CD4_T; Homo_714,totalSample,immune-Central_memory; Homo_714,totalSample,immune-CD4_naive; Homo_714,totalSample,immune-InfiltrationScore; Homo_714,totalSample,immune-nTreg; Homo_714,totalSample,immune-Tr1; Homo_714,totalSample,immune-iTreg; Homo_714,totalSample,immune-Macrophage; Homo_714,totalSample,immune-Th17; Homo_633,totalSample,immune-Tfh; Homo_633,totalSample,immune-InfiltrationScore; Homo_633,totalSample,immune-Effector_memory; Homo_633,totalSample,immune-CD4_T; Homo_633,totalSample,immune-CD4_naive; Homo_633,totalSample,immune-Central_memory; Homo_633,totalSample,immune-Th2; Homo_633,totalSample,immune-nTreg; Homo_633,totalSample,immune-Tr1; Homo_633,totalSample,immune-Th17; Homo_633,totalSample,immune-iTreg; Homo_633,totalSample,immune-Bcell; MCSA,totalSample,immune-Monocyte; MCSA,totalSample,immune-Neutrophil; MCSA,totalSample,immune-CD4_T; MCSA,totalSample,immune-CD8_T; MCSA,totalSample,immune-NKT; MCSA,totalSample,immune-CD4_naive; MCSA,totalSample,immune-Tr1; MCSA,totalSample,immune-nTreg; MCSA,totalSample,immune-iTreg; MCSA,totalSample,immune-Th1; MCSA,totalSample,immune-Th17; MCSA,totalSample,immune-Tfh; MCSA,totalSample,immune-CD8_naive; MCSA,totalSample,immune-MAIT; MCSA,totalSample,immune-Central_memory; MCSA,totalSample,immune-[illegible]
NUBP2	Homo_723,female,AD.vs.control; Homo_723,male,AD.vs.control; Homo_723,female,AD.vs.MCI; Homo_723,male,AD.vs.MCI; Homo_723,female,MCI.vs.control; Homo_723,AD,male.vs.female; Homo_723,control,male.vs.female; Homo_714,female,AD.vs.control; Homo_714,male,AD.vs.control; Homo_714,female,AD.vs.MCI; Homo_714,male,AD.vs.MCI; Homo_714,female,MCI.vs.control; Homo_714,AD,male.vs.female; Homo_714,control,male.vs.female; Homo_633,female,AD.vs.control; Homo_633,female,AD.vs.MCI; Homo_633,female,MCI.vs.control; Homo_633,control,male.vs.female; ROSMAP,female,AD.vs.control; MCSA,MCI,age; Emory,totalSample,immune-Monocyte; Emory,totalSample,immune-Macrophage; Emory,totalSample,immune-NK; Emory,totalSample,immune-Neutrophil; Emory,totalSample,immune-CD4_T; Emory,totalSample,immune-CD8_T; Emory,totalSample,immune-Tr1; Emory,totalSample,immune-iTreg; Emory,totalSample,immune-Th1; Emory,totalSample,immune-Tfh; Emory,totalSample,immune-Exhausted; Emory,totalSample,immune-Effector_memory; Emory,totalSample,immune-InfiltrationScore; Homo_723,totalSample,immune-DC; Homo_723,totalSample,immune-Monocyte; Homo_723,totalSample,immune-Neutrophil; Homo_723,totalSample,immune-CD8_T; Homo_723,totalSample,immune-Central_memory; Homo_723,totalSample,immune-Cytotoxic; Homo_723,totalSample,immune-nTreg; Homo_723,totalSample,immune-Exhausted; Homo_714,totalSample,immune-DC; Homo_714,totalSample,immune-Monocyte; Homo_714,totalSample,immune-Neutrophil; Homo_714,totalSample,immune-nTreg; Homo_633,totalSample,immune-Monocyte; Homo_633,totalSample,immune-DC; Homo_633,totalSample,immune-nTreg; Homo_633,totalSample,immune-Th2; MCSA,totalSample,immune-DC; MCSA,totalSample,immune-Monocyte; MCSA,totalSample,immune-Neutrophil; MCSA,totalSample,immune-CD8_T; MCSA,totalSample,immune-NKT; MCSA,totalSample,immune-iTreg; MCSA,totalSample,immune-Th2; MCSA,totalSample,immune-Cytotoxic; MCSA,totalSample,immune-Central_memory; MCSA,totalSample,immune-Effector_memory; MCSA,totalSample,immune-InfiltrationScore; ROSMAP,totalSample,immune-Cytotoxic; ROSMAP,totalSample,immune-Central_memory; ROSMAP,totalSample,immune-Effector_memory; SRP223445,totalSample,immune-NK; SRP223445,totalSample,immune-CD4_naive; SRP223445,totalSample,immune-Exhausted; SRP223445,totalSample,immune-[illegible]

TXNDC12	Homo_723,female,AD.vs.control; Homo_723,male,AD.vs.control; Homo_723,female,AD.vs.MCI; Homo_723,male,AD.vs.MCI; Homo_714,female,AD.vs.control; Homo_714,male,AD.vs.control; Homo_714,female,AD.vs.MCI; Homo_714,male,AD.vs.MCI; Homo_633,female,AD.vs.control; Homo_633,female,AD.vs.MCI; SRP223445,female,AD.vs.control; MCSA,totalSample,age; MCSA,control,age; MCSA,MCI,age; ROSMAP,MCI,last.visit.age; ROSMAP,MCI,sample.age; Emory,totalSample,immune-DC; Emory,totalSample,immune-Monocyte; Emory,totalSample,immune-Gamma_delta; Emory,totalSample,immune-Tr1; Emory,totalSample,immune-iTreg; Emory,totalSample,immune-CD8_naive; Homo_723,totalSample,immune-Monocyte; Homo_723,totalSample,immune-DC; Homo_723,totalSample,immune-Th2; Homo_723,totalSample,immune-Tfh; Homo_723,totalSample,immune-InfiltrationScore; Homo_723,totalSample,immune-Bcell; Homo_723,totalSample,immune-iTreg; Homo_723,totalSample,immune-Gamma_delta; Homo_714,totalSample,immune-Monocyte; Homo_714,totalSample,immune-Th2; Homo_714,totalSample,immune-Tfh; Homo_633,totalSample,immune-Monocyte; Homo_633,totalSample,immune-Th2; Homo_633,totalSample,immune-Exhausted; Homo_633,totalSample,immune-InfiltrationScore; Homo_633,totalSample,immune-Bcell; Homo_633,totalSample,immune-Tfh; MCSA,totalSample,immune-DC; MCSA,totalSample,immune-Monocyte; MCSA,totalSample,immune-Neutrophil; MCSA,totalSample,immune-CD8_T; MCSA,totalSample,immune-iTreg; MCSA,totalSample,immune-Th2; MCSA,totalSample,immune-Cytotoxic; MCSA,totalSample,immune-Exhausted; MCSA,totalSample,immune-MAIT; MCSA,totalSample,immune-Effector_memory; MCSA,totalSample,immune-InfiltrationScore; ROSMAP,totalSample,immune-Effector_memory; SRP223445,totalSample,immune-Monocyte; SRP223445,totalSample,immune-Neutrophil; ROSMAP,male-excludeAge90+,survival
DMD	Homo_723,female,AD.vs.control; Homo_723,male,AD.vs.control; Homo_723,female,AD.vs.MCI; Homo_723,male,AD.vs.MCI; Homo_723,male,MCI.vs.control; Homo_714,female,AD.vs.control; Homo_714,male,AD.vs.control; Homo_714,female,AD.vs.MCI; Homo_714,male,AD.vs.MCI; Homo_714,male,MCI.vs.control; Homo_633,female,AD.vs.control; Homo_633,female,AD.vs.MCI; Homo_633,female,MCI.vs.control; ROSMAP,MCI,male.vs.female; Emory,totalSample,immune-Gamma_delta; Emory,totalSample,immune-CD4_naive; Emory,totalSample,immune-Cytotoxic; Homo_723,totalSample,immune-Cytotoxic; Homo_723,totalSample,immune-Th1; Homo_723,totalSample,immune-CD4_naive; Homo_723,totalSample,immune-Th17; Homo_723,totalSample,immune-Central_memory; Homo_723,totalSample,immune-CD4_T; Homo_714,totalSample,immune-Th1; Homo_714,totalSample,immune-CD4_naive; Homo_714,totalSample,immune-NKT; Homo_633,totalSample,immune-NKT; Homo_633,totalSample,immune-Tfh; Homo_633,totalSample,immune-CD4_T; Homo_633,totalSample,immune-CD4_naive; Homo_633,totalSample,immune-Tr1; Homo_633,totalSample,immune-Gamma_delta; Homo_633,totalSample,immune-Th1; MCSA,totalSample,immune-DC; MCSA,totalSample,immune-Bcell; MCSA,totalSample,immune-Macrophage; MCSA,totalSample,immune-CD4_T; MCSA,totalSample,immune-CD4_naive; MCSA,totalSample,immune-Tr1; MCSA,totalSample,immune-nTreg; MCSA,totalSample,immune-iTreg; MCSA,totalSample,immune-Tfh; MCSA,totalSample,immune-CD8_naive; MCSA,totalSample,immune-Cytotoxic; MCSA,totalSample,immune-Central_memory; ROSMAP,totalSample,immune-InfiltrationScore
ZDHHC9	Homo_723,female,AD.vs.control; Homo_723,male,AD.vs.control; Homo_723,female,AD.vs.MCI; Homo_723,male,AD.vs.MCI; Homo_714,male,AD.vs.MCI; Homo_633,female,AD.vs.control; Homo_633,female,AD.vs.MCI; ROSMAP,female,AD.vs.control; ROSMAP,control,male.vs.female; MCSA,totalSample,age; MCSA,control,age; Emory,totalSample,immune-Macrophage; Emory,totalSample,immune-Neutrophil; Emory,totalSample,immune-CD4_T; Emory,totalSample,immune-CD8_T; Emory,totalSample,immune-Tr1; Emory,totalSample,immune-nTreg; Emory,totalSample,immune-iTreg; Emory,totalSample,immune-Tfh; Emory,totalSample,immune-InfiltrationScore; Homo_723,totalSample,immune-Monocyte; Homo_723,totalSample,immune-nTreg; Homo_723,totalSample,immune-Central_memory; Homo_723,totalSample,immune-iTreg; Homo_723,totalSample,immune-CD4_naive; Homo_723,totalSample,immune-Tfh; Homo_723,totalSample,immune-CD4_T; Homo_723,totalSample,immune-InfiltrationScore; Homo_723,totalSample,immune-Tr1; Homo_723,totalSample,immune-CD8_T; Homo_723,totalSample,immune-Th2; Homo_723,totalSample,immune-Exhausted; Homo_714,totalSample,immune-InfiltrationScore; Homo_714,totalSample,immune-nTreg; Homo_714,totalSample,immune-Tfh; Homo_714,totalSample,immune-Monocyte; Homo_714,totalSample,immune-iTreg; Homo_714,totalSample,immune-CD4_naive; Homo_714,totalSample,immune-Tr1; Homo_714,totalSample,immune-CD4_T; Homo_714,totalSample,immune-Effector_memory; Homo_714,totalSample,immune-Central_memory; Homo_714,totalSample,immune-Macrophage; Homo_714,totalSample,immune-NKT; Homo_633,totalSample,immune-nTreg; Homo_633,totalSample,immune-Monocyte; Homo_633,totalSample,immune-InfiltrationScore; Homo_633,totalSample,immune-Tr1; Homo_633,totalSample,immune-CD4_naive; Homo_633,totalSample,immune-Tfh; Homo_633,totalSample,immune-iTreg; Homo_633,totalSample,immune-CD4_T; Homo_633,totalSample,immune-Central_memory; Homo_633,totalSample,immune-NKT; Homo_633,totalSample,immune-Effector_memory; MCSA,totalSample,immune-Monocyte; MCSA,totalSample,immune-Neutrophil; MCSA,totalSample,immune-CD4_T; MCSA,totalSample,immune-CD8_T; MCSA,totalSample,immune-Gamma_delta; MCSA,totalSample,immune-CD4_naive; MCSA,totalSample,immune-Tr1; MCSA,totalSample,immune-nTreg; MCSA,totalSample,immune-iTreg; [illegible]
CXorf38	Homo_723,female,AD.vs.control; Homo_723,male,AD.vs.control; Homo_723,female,AD.vs.MCI; Homo_723,male,AD.vs.MCI; Homo_714,female,AD.vs.control; Homo_714,male,AD.vs.control; Homo_714,female,AD.vs.MCI; Homo_714,male,AD.vs.MCI; Homo_633,female,AD.vs.control; Homo_633,female,AD.vs.MCI; ROSMAP,female,AD.vs.control; Emory,MCI,age; MCSA,MCI,age; Emory,totalSample,immune-Gamma_delta; Emory,totalSample,immune-Tr1; Emory,totalSample,immune-iTreg; Emory,totalSample,immune-CD8_naive; Emory,totalSample,immune-Cytotoxic; Emory,totalSample,immune-Exhausted; Homo_723,totalSample,immune-Monocyte; Homo_723,totalSample,immune-Th2; Homo_723,totalSample,immune-Tfh; Homo_723,totalSample,immune-InfiltrationScore; Homo_723,totalSample,immune-DC; Homo_723,totalSample,immune-iTreg; Homo_723,totalSample,immune-Effector_memory; Homo_723,totalSample,immune-Gamma_delta; Homo_723,totalSample,immune-Bcell; Homo_723,totalSample,immune-Tr1; Homo_723,totalSample,immune-Exhausted; Homo_723,totalSample,immune-CD4_T; Homo_714,totalSample,immune-Th2; Homo_714,totalSample,immune-Tfh; Homo_714,totalSample,immune-Monocyte; Homo_714,totalSample,immune-Effector_memory; Homo_714,totalSample,immune-Gamma_delta; Homo_714,totalSample,immune-Tr1; Homo_714,totalSample,immune-CD4_T; Homo_714,totalSample,immune-InfiltrationScore; Homo_633,totalSample,immune-Monocyte; Homo_633,totalSample,immune-Th2; Homo_633,totalSample,immune-Exhausted; Homo_633,totalSample,immune-Tfh; Homo_633,totalSample,immune-InfiltrationScore; Homo_633,totalSample,immune-Gamma_delta; Homo_633,totalSample,immune-Effector_memory; Homo_633,totalSample,immune-Th17; Homo_633,totalSample,immune-CD4_T; Homo_633,totalSample,immune-Central_memory; Homo_633,totalSample,immune-CD4_naive; Homo_633,totalSample,immune-Bcell; MCSA,totalSample,immune-DC; MCSA,totalSample,immune-Monocyte; MCSA,totalSample,immune-CD4_T; MCSA,totalSample,immune-CD8_T; MCSA,totalSample,immune-CD4_naive; MCSA,totalSample,immune-Tr1; MCSA,totalSample,immune-nTreg; MCSA,totalSample,immune-iTreg; MCSA,totalSample,immune-Th17; MCSA,totalSample,immune-Cytotoxic; MCSA,totalSample,immune-Exhausted; MCSA,totalSample,immune-Central_memory; ROSMAP,totalSample,immune-Effector_memory; SRP223445,totalSample,immune-CD4_naive; [illegible]
SPIRE1	Homo_723,female,AD.vs.control; Homo_723,male,AD.vs.control; Homo_723,female,AD.vs.MCI; Homo_723,male,AD.vs.MCI; Homo_723,female,MCI.vs.control; Homo_723,male,MCI.vs.control; Homo_714,female,AD.vs.control; Homo_714,male,AD.vs.control; Homo_714,female,AD.vs.MCI; Homo_714,male,AD.vs.MCI; Homo_714,female,MCI.vs.control; Homo_714,male,MCI.vs.control; Homo_633,female,AD.vs.control; Homo_633,female,AD.vs.MCI; ROSMAP,male,AD.vs.control; MCSA,MCI,age; Emory,totalSample,immune-InfiltrationScore; Homo_723,totalSample,immune-Exhausted; Homo_723,totalSample,immune-Th17; Homo_723,totalSample,immune-CD8_T; Homo_723,totalSample,immune-Cytotoxic; Homo_723,totalSample,immune-InfiltrationScore; Homo_723,totalSample,immune-CD4_naive; Homo_723,totalSample,immune-Monocyte; Homo_723,totalSample,immune-CD4_T; Homo_723,totalSample,immune-Tfh; Homo_723,totalSample,immune-NK; Homo_714,totalSample,immune-Monocyte; Homo_714,totalSample,immune-Exhausted; Homo_714,totalSample,immune-InfiltrationScore; Homo_714,totalSample,immune-Th17; Homo_714,totalSample,immune-CD4_naive; Homo_714,totalSample,immune-NK; Homo_633,totalSample,immune-Tfh; Homo_633,totalSample,immune-Monocyte; Homo_633,totalSample,immune-Th2; Homo_633,totalSample,immune-InfiltrationScore; Homo_633,totalSample,immune-Effector_memory; Homo_633,totalSample,immune-Exhausted; Homo_633,totalSample,immune-CD4_T; Homo_633,totalSample,immune-CD4_naive; Homo_633,totalSample,immune-Central_memory; Homo_633,totalSample,immune-Gamma_delta; Homo_633,totalSample,immune-Bcell; Homo_633,totalSample,immune-Th17; MCSA,totalSample,immune-DC; MCSA,totalSample,immune-Monocyte; MCSA,totalSample,immune-CD4_T; MCSA,totalSample,immune-CD8_T; MCSA,totalSample,immune-CD4_naive; MCSA,totalSample,immune-Tr1; MCSA,totalSample,immune-nTreg; MCSA,totalSample,immune-iTreg; MCSA,totalSample,immune-Tfh; MCSA,totalSample,immune-CD8_naive; MCSA,totalSample,immune-Cytotoxic; MCSA,totalSample,immune-Exhausted; MCSA,totalSample,immune-MAIT; MCSA,totalSample,immune-Central_memory; SRP223445,totalSample,immune-InfiltrationScore; ROSMAP,male-excludeAge90+,survival
ALKBH3	Homo_723,female,AD.vs.control; Homo_723,male,AD.vs.control; Homo_723,female,AD.vs.MCI; Homo_723,male,AD.vs.MCI; Homo_714,female,AD.vs.control; Homo_714,male,AD.vs.control; Homo_714,female,AD.vs.MCI; Homo_714,male,AD.vs.MCI; Homo_633,female,AD.vs.control; Homo_633,female,AD.vs.MCI; ROSMAP,female,AD.vs.control; Emory,control,age; MCSA,MCI,age; Emory,totalSample,immune-Macrophage; Emory,totalSample,immune-NK; Emory,totalSample,immune-Neutrophil; Emory,totalSample,immune-CD4_T; Emory,totalSample,immune-CD8_T; Emory,totalSample,immune-Tr1; Emory,totalSample,immune-iTreg; Emory,totalSample,immune-Tfh; Emory,totalSample,immune-Exhausted; Emory,totalSample,immune-Central_memory; Emory,totalSample,immune-Effector_memory; Emory,totalSample,immune-InfiltrationScore; Homo_723,totalSample,immune-Tfh; Homo_723,totalSample,immune-Th2; Homo_723,totalSample,immune-Monocyte; Homo_723,totalSample,immune-Effector_memory; Homo_723,totalSample,immune-InfiltrationScore; Homo_723,totalSample,immune-iTreg; Homo_723,totalSample,immune-DC; Homo_723,totalSample,immune-Tr1; Homo_723,totalSample,immune-CD4_T; Homo_723,totalSample,immune-Bcell; Homo_723,totalSample,immune-Macrophage; Homo_714,totalSample,immune-Th2; Homo_714,totalSample,immune-Tfh; Homo_714,totalSample,immune-Effector_memory; Homo_714,totalSample,immune-Monocyte; Homo_714,totalSample,immune-InfiltrationScore; Homo_714,totalSample,immune-Tr1; Homo_714,totalSample,immune-CD4_T; Homo_714,totalSample,immune-CD8_T; Homo_714,totalSample,immune-Bcell; Homo_633,totalSample,immune-Monocyte; Homo_633,totalSample,immune-Th2; Homo_633,totalSample,immune-Tfh; Homo_633,totalSample,immune-InfiltrationScore; Homo_633,totalSample,immune-Effector_memory; Homo_633,totalSample,immune-Bcell; Homo_633,totalSample,immune-Neutrophil; Homo_633,totalSample,immune-Central_memory; Homo_633,totalSample,immune-CD4_T; MCSA,totalSample,immune-DC; MCSA,totalSample,immune-Macrophage; MCSA,totalSample,immune-Neutrophil; MCSA,totalSample,immune-CD4_T; MCSA,totalSample,immune-CD8_T; MCSA,totalSample,immune-Gamma_delta; MCSA,totalSample,immune-Tr1; MCSA,totalSample,immune-Th1; MCSA,totalSample,immune-Th2; MCSA,totalSample,immune-Tfh; MCSA,totalSample,immune-MAIT; MCSA,totalSample,immune-Effector_memory; ROSMAP,totalSample,immune-Th17
SYBU	Homo_723,female,AD.vs.control; Homo_723,male,AD.vs.control; Homo_723,female,AD.vs.MCI; Homo_723,male,AD.vs.MCI; Homo_714,female,AD.vs.control; Homo_714,male,AD.vs.control; Homo_714,female,AD.vs.MCI; Homo_714,male,AD.vs.MCI; Homo_633,female,AD.vs.control; Homo_633,female,AD.vs.MCI; MCSA,totalSample,age; MCSA,control,age; Emory,totalSample,immune-B_cell; Emory,totalSample,immune-Macrophage; Emory,totalSample,immune-CD4_T; Emory,totalSample,immune-Gamma_delta; Emory,totalSample,immune-Tr1; Emory,totalSample,immune-iTreg; Emory,totalSample,immune-Th17; Homo_723,totalSample,immune-Central_memory; Homo_723,totalSample,immune-nTreg; Homo_723,totalSample,immune-CD4_naive; Homo_723,totalSample,immune-CD4_T; Homo_723,totalSample,immune-Neutrophil; Homo_723,totalSample,immune-Th1; Homo_723,totalSample,immune-Tr1; Homo_723,totalSample,immune-Monocyte; Homo_723,totalSample,immune-Th17; Homo_714,totalSample,immune-CD4_naive; Homo_714,totalSample,immune-nTreg; Homo_714,totalSample,immune-Th1; Homo_714,totalSample,immune-NKT; Homo_633,totalSample,immune-nTreg; Homo_633,totalSample,immune-CD4_T; Homo_633,totalSample,immune-Tfh; Homo_633,totalSample,immune-CD4_naive; Homo_633,totalSample,immune-Tr1; Homo_633,totalSample,immune-iTreg; Homo_633,totalSample,immune-NKT; Homo_633,totalSample,immune-Central_memory; Homo_633,totalSample,immune-Monocyte; Homo_633,totalSample,immune-Th1; MCSA,totalSample,immune-Bcell; MCSA,totalSample,immune-Macrophage; MCSA,totalSample,immune-Neutrophil; MCSA,totalSample,immune-CD4_T; MCSA,totalSample,immune-CD4_naive; MCSA,totalSample,immune-Tr1; MCSA,totalSample,immune-nTreg; MCSA,totalSample,immune-iTreg; MCSA,totalSample,immune-Th17; MCSA,totalSample,immune-Tfh; MCSA,totalSample,immune-CD8_naive; MCSA,totalSample,immune-Central_memory; MCSA,totalSample,immune-Effector_memory; ROSMAP,totalSample,immune-InfiltrationScore
GPAT2	Homo_723,female,AD.vs.control; Homo_723,male,AD.vs.control; Homo_723,female,AD.vs.MCI; Homo_723,male,AD.vs.MCI; Homo_714,female,AD.vs.control; Homo_714,male,AD.vs.control; Homo_714,female,AD.vs.MCI; Homo_714,male,AD.vs.MCI; Homo_633,female,AD.vs.control; Homo_633,female,AD.vs.MCI; MCSA,totalSample,age; MCSA,control,age; ROSMAP,AD,last.visit.age; Emory,totalSample,immune-InfiltrationScore; Homo_723,totalSample,immune-CD8_T; Homo_723,totalSample,immune-InfiltrationScore; Homo_723,totalSample,immune-Cytotoxic; Homo_723,totalSample,immune-Macrophage; Homo_723,totalSample,immune-iTreg; Homo_723,totalSample,immune-Tfh; Homo_723,totalSample,immune-Exhausted; Homo_723,totalSample,immune-Effector_memory; Homo_714,totalSample,immune-CD8_T; Homo_714,totalSample,immune-Effector_memory; Homo_714,totalSample,immune-InfiltrationScore; Homo_714,totalSample,immune-nTreg; Homo_714,totalSample,immune-Tfh; Homo_714,totalSample,immune-Macrophage; Homo_633,totalSample,immune-InfiltrationScore; Homo_633,totalSample,immune-DC; Homo_633,totalSample,immune-nTreg; Homo_633,totalSample,immune-Effector_memory; MCSA,totalSample,immune-DC; MCSA,totalSample,immune-Monocyte; MCSA,totalSample,immune-Neutrophil; MCSA,totalSample,immune-CD8_T; MCSA,totalSample,immune-Th2; MCSA,totalSample,immune-Cytotoxic; MCSA,totalSample,immune-Central_memory; MCSA,totalSample,immune-Effector_memory; ROSMAP,totalSample,immune-InfiltrationScore
TRIM2	Homo_723,female,AD.vs.control; Homo_723,male,AD.vs.control; Homo_723,female,AD.vs.MCI; Homo_723,male,AD.vs.MCI; Homo_723,female,MCI.vs.control; Homo_723,male,MCI.vs.control; Homo_714,female,AD.vs.control; Homo_714,male,AD.vs.control; Homo_714,female,AD.vs.MCI; Homo_714,male,AD.vs.MCI; Homo_714,female,MCI.vs.control; Homo_714,male,MCI.vs.control; Homo_633,female,AD.vs.control; Homo_633,female,AD.vs.MCI; MCSA,MCI,age; ROSMAP,AD,last.visit.age; Emory,totalSample,immune-Macrophage; Emory,totalSample,immune-CD4_T; Emory,totalSample,immune-Gamma_delta; Emory,totalSample,immune-Tr1; Emory,totalSample,immune-nTreg; Emory,totalSample,immune-iTreg; Emory,totalSample,immune-Th17; Emory,totalSample,immune-Tfh; Emory,totalSample,immune-Central_memory; Homo_723,totalSample,immune-Th17; Homo_723,totalSample,immune-CD4_naive; Homo_723,totalSample,immune-Cytotoxic; Homo_723,totalSample,immune-Central_memory; Homo_723,totalSample,immune-CD4_T; Homo_723,totalSample,immune-Tr1; Homo_723,totalSample,immune-Th1; Homo_723,totalSample,immune-Tfh; Homo_723,totalSample,immune-nTreg; Homo_723,totalSample,immune-Exhausted; Homo_723,totalSample,immune-iTreg; Homo_723,totalSample,immune-Th2; Homo_723,totalSample,immune-Gamma_delta; Homo_723,totalSample,immune-NKT; Homo_714,totalSample,immune-CD4_naive; Homo_714,totalSample,immune-Th17; Homo_714,totalSample,immune-CD4_T; Homo_714,totalSample,immune-Th1; Homo_714,totalSample,immune-Tfh; Homo_714,totalSample,immune-iTreg; Homo_714,totalSample,immune-NKT; Homo_714,totalSample,immune-Exhausted; Homo_714,totalSample,immune-Central_memory; Homo_714,totalSample,immune-Cytotoxic; Homo_714,totalSample,immune-Tr1; Homo_714,totalSample,immune-nTreg; Homo_714,totalSample,immune-Gamma_delta; Homo_633,totalSample,immune-Tfh; Homo_633,totalSample,immune-CD4_T; Homo_633,totalSample,immune-CD4_naive; Homo_633,totalSample,immune-Tr1; Homo_633,totalSample,immune-iTreg; Homo_633,totalSample,immune-Central_memory; Homo_633,totalSample,immune-NKT; Homo_633,totalSample,immune-Th2; Homo_633,totalSample,immune-Gamma_delta; Homo_633,totalSample,immune-nTreg; Homo_633,totalSample,immune-Th17; Homo_633,totalSample,immune-Th1; Homo_633,totalSample,immune-Effector_memory; Homo_633,totalSample,immune-Cytotoxic; Homo_633,totalSample,immune-Exhausted; Homo_633,totalSample,immune-DC; MCSA,totalSample,immune-DC; MCSA,totalSample,immune-Bcell; MCSA,totalSample,immune-Monocyte; MCSA,totalSample,immune-Macrophage; MCSA,totalSample,immune-CD4_T; MCSA,totalSample,immune-CD8_T; MCSA,totalSample,immune-Gamma_delta; MCSA,totalSample,immune-CD4_naive; MCSA,totalSample,immune-Tr1; MCSA,totalSample,immune-nTreg; MCSA,totalSample,immune-iTreg; MCSA,totalSample,immune-Th17; MCSA,totalSample,immune-Tfh; MCSA,totalSample,immune-CD8_naive; MCSA,totalSample,immune-Cytotoxic; MCSA,totalSample,immune-Exhausted; MCSA,totalSample,immune-MAIT; [illegible]
MAGI3	Homo_723,female,AD.vs.control; Homo_723,male,AD.vs.control; Homo_723,female,AD.vs.MCI; Homo_723,male,AD.vs.MCI; Homo_714,female,AD.vs.control; Homo_714,male,AD.vs.control; Homo_714,female,AD.vs.MCI; Homo_714,male,AD.vs.MCI; Homo_633,female,AD.vs.control; Homo_633,female,AD.vs.MCI; ROSMAP,control,male.vs.female; MCSA,totalSample,age; MCSA,control,age; Emory,totalSample,immune-Macrophage; Emory,totalSample,immune-CD4_T; Emory,totalSample,immune-Gamma_delta; Emory,totalSample,immune-CD4_naive; Emory,totalSample,immune-Tr1; Emory,totalSample,immune-nTreg; Emory,totalSample,immune-iTreg; Emory,totalSample,immune-Th17; Emory,totalSample,immune-Tfh; Emory,totalSample,immune-Cytotoxic; Emory,totalSample,immune-Central_memory; Homo_723,totalSample,immune-CD4_naive; Homo_723,totalSample,immune-Th17; Homo_723,totalSample,immune-CD4_T; Homo_723,totalSample,immune-Cytotoxic; Homo_723,totalSample,immune-Central_memory; Homo_723,totalSample,immune-Th1; Homo_723,totalSample,immune-Tr1; Homo_723,totalSample,immune-nTreg; Homo_723,totalSample,immune-Tfh; Homo_723,totalSample,immune-iTreg; Homo_723,totalSample,immune-Th2; Homo_723,totalSample,immune-Gamma_delta; Homo_723,totalSample,immune-NKT; Homo_714,totalSample,immune-CD4_naive; Homo_714,totalSample,immune-Th1; Homo_714,totalSample,immune-CD4_T; Homo_714,totalSample,immune-NKT; Homo_714,totalSample,immune-Th17; Homo_714,totalSample,immune-Cytotoxic; Homo_714,totalSample,immune-Tr1; Homo_714,totalSample,immune-iTreg; Homo_714,totalSample,immune-Tfh; Homo_714,totalSample,immune-nTreg; Homo_714,totalSample,immune-Central_memory; Homo_714,totalSample,immune-Exhausted; Homo_714,totalSample,immune-Gamma_delta; Homo_714,totalSample,immune-CD8_T; Homo_633,totalSample,immune-Tfh; Homo_633,totalSample,immune-CD4_T; Homo_633,totalSample,immune-Tr1; Homo_633,totalSample,immune-iTreg; Homo_633,totalSample,immune-CD4_naive; Homo_633,totalSample,immune-NKT; Homo_633,totalSample,immune-nTreg; Homo_633,totalSample,immune-Th1; Homo_633,totalSample,immune-Central_memory; Homo_633,totalSample,immune-Gamma_delta; Homo_633,totalSample,immune-Cytotoxic; Homo_633,totalSample,immune-Th2; Homo_633,totalSample,immune-Th17; Homo_633,totalSample,immune-InfiltrationScore; Homo_633,totalSample,immune-Effector_memory; MCSA,totalSample,immune-DC; MCSA,totalSample,immune-CD4_T; MCSA,totalSample,immune-CD8_T; MCSA,totalSample,immune-Gamma_delta; MCSA,totalSample,immune-CD4_naive; MCSA,totalSample,immune-Tr1; MCSA,totalSample,immune-nTreg; MCSA,totalSample,immune-iTreg; MCSA,totalSample,immune-Th17; MCSA,totalSample,immune-Tfh; MCSA,totalSample,immune-CD8_naive; MCSA,totalSample,immune-Cytotoxic; MCSA,totalSample,immune-Exhausted; MCSA,totalSample,immune-Central_memory; ROSMAP,totalSample,immune-InfiltrationScore; SRP223445,totalSample,immune-NK; SRP223445,totalSample,immune-Exhausted
ACTR3B	Homo_723,female,AD.vs.control; Homo_723,male,AD.vs.control; Homo_723,female,AD.vs.MCI; Homo_723,male,AD.vs.MCI; Homo_723,female,MCI.vs.control; Homo_723,male,MCI.vs.control; Homo_714,female,AD.vs.control; Homo_714,male,AD.vs.control; Homo_714,female,AD.vs.MCI; Homo_714,male,AD.vs.MCI; Homo_714,female,MCI.vs.control; Homo_714,male,MCI.vs.control; Homo_633,female,AD.vs.control; Homo_633,female,AD.vs.MCI; Homo_633,female,MCI.vs.control; Homo_633,male,MCI.vs.control; MCSA,totalSample,age; MCSA,control,age; ROSMAP,MCI,last.visit.age; ROSMAP,MCI,sample.age; Emory,totalSample,immune-CD4_T; Emory,totalSample,immune-Tr1; Emory,totalSample,immune-iTreg; Emory,totalSample,immune-Central_memory; Emory,totalSample,immune-InfiltrationScore; Homo_723,totalSample,immune-Monocyte; Homo_723,totalSample,immune-DC; Homo_723,totalSample,immune-Th1; Homo_723,totalSample,immune-Central_memory; Homo_723,totalSample,immune-CD4_naive; Homo_714,totalSample,immune-NKT; Homo_714,totalSample,immune-Th1; Homo_714,totalSample,immune-CD4_naive; Homo_633,totalSample,immune-NKT; Homo_633,totalSample,immune-Th1; Homo_633,totalSample,immune-Monocyte; Homo_633,totalSample,immune-InfiltrationScore; Homo_633,totalSample,immune-Tfh; Homo_633,totalSample,immune-iTreg; Homo_633,totalSample,immune-CD4_naive; MCSA,totalSample,immune-DC; MCSA,totalSample,immune-CD4_T; MCSA,totalSample,immune-Gamma_delta; MCSA,totalSample,immune-CD4_naive; MCSA,totalSample,immune-Tr1; MCSA,totalSample,immune-nTreg; MCSA,totalSample,immune-iTreg; MCSA,totalSample,immune-Th17; MCSA,totalSample,immune-Tfh; MCSA,totalSample,immune-CD8_naive; MCSA,totalSample,immune-Cytotoxic; MCSA,totalSample,immune-Exhausted; MCSA,totalSample,immune-Central_memory; SRP223445,totalSample,immune-CD4_naive; SRP223445,totalSample,immune-Exhausted
TCAF1	Homo_723,female,AD.vs.control; Homo_723,male,AD.vs.control; Homo_723,female,AD.vs.MCI; Homo_723,male,AD.vs.MCI; Homo_714,female,AD.vs.control; Homo_714,male,AD.vs.control; Homo_714,female,AD.vs.MCI; Homo_714,male,AD.vs.MCI; Homo_633,female,AD.vs.control; Homo_633,female,AD.vs.MCI; ROSMAP,female,MCI.vs.control; SRP223445,female,AD.vs.control; MCSA,MCI,age; Emory,totalSample,immune-Monocyte; Emory,totalSample,immune-Macrophage; Emory,totalSample,immune-NK; Emory,totalSample,immune-Neutrophil; Emory,totalSample,immune-CD4_T; Emory,totalSample,immune-CD8_T; Emory,totalSample,immune-Tr1; Emory,totalSample,immune-iTreg; Emory,totalSample,immune-Exhausted; Emory,totalSample,immune-Effector_memory; Emory,totalSample,immune-InfiltrationScore; Homo_723,totalSample,immune-CD4_naive; Homo_723,totalSample,immune-InfiltrationScore; Homo_723,totalSample,immune-Central_memory; Homo_723,totalSample,immune-Neutrophil; Homo_723,totalSample,immune-CD4_T; Homo_723,totalSample,immune-Tfh; Homo_723,totalSample,immune-Tr1; Homo_723,totalSample,immune-Th1; Homo_723,totalSample,immune-nTreg; Homo_723,totalSample,immune-NKT; Homo_723,totalSample,immune-NK; Homo_723,totalSample,immune-iTreg; Homo_714,totalSample,immune-InfiltrationScore; Homo_714,totalSample,immune-CD4_naive; Homo_714,totalSample,immune-NKT; Homo_714,totalSample,immune-Neutrophil; Homo_714,totalSample,immune-Central_memory; Homo_714,totalSample,immune-Tfh; Homo_714,totalSample,immune-CD4_T; Homo_714,totalSample,immune-Effector_memory; Homo_714,totalSample,immune-Th1; Homo_714,totalSample,immune-NK; Homo_714,totalSample,immune-Tr1; Homo_714,totalSample,immune-iTreg; Homo_633,totalSample,immune-Tfh; Homo_633,totalSample,immune-InfiltrationScore; Homo_633,totalSample,immune-CD4_naive; Homo_633,totalSample,immune-Effector_memory; Homo_633,totalSample,immune-CD4_T; Homo_633,totalSample,immune-NKT; Homo_633,totalSample,immune-Central_memory; Homo_633,totalSample,immune-Tr1; Homo_633,totalSample,immune-iTreg; Homo_633,totalSample,immune-Neutrophil; Homo_633,totalSample,immune-nTreg; Homo_633,totalSample,immune-Bcell; MCSA,totalSample,immune-DC; MCSA,totalSample,immune-Macrophage; MCSA,totalSample,immune-CD4_T; MCSA,totalSample,immune-CD4_naive; MCSA,totalSample,immune-Tr1; MCSA,totalSample,immune-nTreg; MCSA,totalSample,immune-iTreg; MCSA,totalSample,immune-Tfh; MCSA,totalSample,immune-CD8_naive; MCSA,totalSample,immune-Cytotoxic; MCSA,totalSample,immune-Exhausted; MCSA,totalSample,immune-MAIT; MCSA,totalSample,immune-Central_memory; ROSMAP,totalSample,immune-Th17; ROSMAP,totalSample,immune-Effector_memory; ROSMAP,totalSample,immune-InfiltrationScore; SRP223445,totalSample,immune-NK; SRP223445,totalSample,immune-Neutrophil; SRP223445,totalSample,immune-CD4_naive; SRP223445,totalSample,immune-nTreg; SRP223445,totalSample,immune-InfiltrationScore; ROSMAP,female-excludeAge90+,survival; [illegible]
SEPTIN4	Homo_723,female,AD.vs.control; Homo_723,male,AD.vs.control; Homo_723,female,AD.vs.MCI; Homo_723,male,AD.vs.MCI; Homo_714,female,AD.vs.control; Homo_714,male,AD.vs.control; Homo_714,female,AD.vs.MCI; Homo_714,male,AD.vs.MCI; Homo_633,female,AD.vs.control; Homo_633,female,AD.vs.MCI; Homo_633,female,MCI.vs.control; ROSMAP,female,AD.vs.control; ROSMAP,male,AD.vs.control; ROSMAP,male,AD.vs.MCI; ROSMAP,AD,male.vs.female; ROSMAP,MCI,sample.age; Emory,totalSample,immune-DC; Emory,totalSample,immune-Th17; Homo_723,totalSample,immune-DC; Homo_723,totalSample,immune-Monocyte; Homo_714,totalSample,immune-DC; Homo_714,totalSample,immune-Th2; Homo_714,totalSample,immune-InfiltrationScore; Homo_633,totalSample,immune-DC; Homo_633,totalSample,immune-InfiltrationScore; MCSA,totalSample,immune-DC; MCSA,totalSample,immune-Monocyte; MCSA,totalSample,immune-Macrophage; SRP223445,totalSample,immune-CD4_naive; SRP223445,totalSample,immune-Exhausted

AKAP6	Homo_723,female,AD.vs.control; Homo_723,male,AD.vs.control; Homo_723,female,AD.vs.MCI; Homo_723,male,AD.vs.MCI; Homo_633,female,AD.vs.control; Homo_633,female,AD.vs.MCI; MCSA,totalSample,age; MCSA,control,age; Emory,totalSample,immune-B_cell; Emory,totalSample,immune-Macrophage; Emory,totalSample,immune-CD4_T; Emory,totalSample,immune-Tr1; Emory,totalSample,immune-iTreg; Emory,totalSample,immune-Tfh; Emory,totalSample,immune-Central_memory; Emory,totalSample,immune-InfiltrationScore; Homo_723,totalSample,immune-Central_memory; Homo_723,totalSample,immune-CD4_naive; Homo_723,totalSample,immune-CD4_T; Homo_723,totalSample,immune-nTreg; Homo_723,totalSample,immune-Tfh; Homo_723,totalSample,immune-iTreg; Homo_723,totalSample,immune-Tr1; Homo_723,totalSample,immune-Th1; Homo_723,totalSample,immune-Neutrophil; Homo_723,totalSample,immune-Th2; Homo_723,totalSample,immune-Cytotoxic; Homo_723,totalSample,immune-Gamma_delta; Homo_714,totalSample,immune-Tfh; Homo_714,totalSample,immune-CD4_naive; Homo_714,totalSample,immune-CD4_T; Homo_714,totalSample,immune-iTreg; Homo_714,totalSample,immune-nTreg; Homo_714,totalSample,immune-Tr1; Homo_714,totalSample,immune-Th1; Homo_714,totalSample,immune-Central_memory; Homo_714,totalSample,immune-NKT; Homo_714,totalSample,immune-Effector_memory; Homo_714,totalSample,immune-Neutrophil; Homo_714,totalSample,immune-Gamma_delta; Homo_714,totalSample,immune-Cytotoxic; Homo_633,totalSample,immune-Tfh; Homo_633,totalSample,immune-CD4_T; Homo_633,totalSample,immune-CD4_naive; Homo_633,totalSample,immune-Tr1; Homo_633,totalSample,immune-nTreg; Homo_633,totalSample,immune-iTreg; Homo_633,totalSample,immune-Central_memory; Homo_633,totalSample,immune-NKT; Homo_633,totalSample,immune-Th1; Homo_633,totalSample,immune-Effector_memory; Homo_633,totalSample,immune-Gamma_delta; Homo_633,totalSample,immune-Cytotoxic; MCSA,totalSample,immune-DC; MCSA,totalSample,immune-Bcell; MCSA,totalSample,immune-Macrophage; MCSA,totalSample,immune-Neutrophil; MCSA,totalSample,immune-CD4_T; MCSA,totalSample,immune-Gamma_delta; MCSA,totalSample,immune-CD4_naive; MCSA,totalSample,immune-Tr1; MCSA,totalSample,immune-nTreg; MCSA,totalSample,immune-iTreg; MCSA,totalSample,immune-Th17; MCSA,totalSample,immune-Tfh; MCSA,totalSample,immune-CD8_naive; MCSA,totalSample,immune-Cytotoxic; MCSA,totalSample,immune-Exhausted; MCSA,totalSample,immune-Central_memory; MCSA,totalSample,immune-Effector_memory; ROSMAP,totalSample,immune-InfiltrationScore
RGS6	Homo_723,female,AD.vs.control; Homo_723,male,AD.vs.control; Homo_723,female,AD.vs.MCI; Homo_723,male,AD.vs.MCI; Homo_714,female,AD.vs.control; Homo_714,male,AD.vs.control; Homo_714,female,AD.vs.MCI; Homo_714,male,AD.vs.MCI; Homo_633,female,AD.vs.control; Homo_633,female,AD.vs.MCI; Emory,MCI,age; Emory,totalSample,immune-Tr1; Emory,totalSample,immune-iTreg; Emory,totalSample,immune-Th1; Homo_723,totalSample,immune-DC; Homo_714,totalSample,immune-DC; Homo_633,totalSample,immune-DC; ROSMAP,totalSample,immune-InfiltrationScore; SRP223445,totalSample,immune-CD4_naive; SRP223445,totalSample,immune-Exhausted
C19orf33	Homo_723,female,AD.vs.control; Homo_723,male,AD.vs.control; Homo_723,female,AD.vs.MCI; Homo_723,male,AD.vs.MCI; Homo_723,female,MCI.vs.control; Homo_723,male,MCI.vs.control; Homo_723,control,male.vs.female; Homo_714,female,AD.vs.control; Homo_714,male,AD.vs.control; Homo_714,female,AD.vs.MCI; Homo_714,male,AD.vs.MCI; Homo_714,female,MCI.vs.control; Homo_714,male,MCI.vs.control; Homo_714,control,male.vs.female; Homo_633,female,AD.vs.control; Homo_633,female,AD.vs.MCI; Homo_633,female,MCI.vs.control; ROSMAP,female,AD.vs.control; SRP223445,female,AD.vs.control; Emory,MCI,age; MCSA,MCI,age; ROSMAP,MCI,sample.age; Emory,totalSample,immune-Th17; Homo_723,totalSample,immune-DC; Homo_723,totalSample,immune-Th2; Homo_714,totalSample,immune-DC; Homo_714,totalSample,immune-Th2; Homo_633,totalSample,immune-DC; Homo_633,totalSample,immune-Th1; MCSA,totalSample,immune-DC; MCSA,totalSample,immune-Monocyte; MCSA,totalSample,immune-CD8_T; MCSA,totalSample,immune-iTreg; MCSA,totalSample,immune-Th2; MCSA,totalSample,immune-Cytotoxic; MCSA,totalSample,immune-Effector_memory
SLAMF6	Homo_723,female,AD.vs.control; Homo_723,male,AD.vs.control; Homo_723,female,AD.vs.MCI; Homo_723,male,AD.vs.MCI; Homo_714,female,AD.vs.control; Homo_714,male,AD.vs.control; Homo_714,female,AD.vs.MCI; Homo_714,male,AD.vs.MCI; Homo_714,female,MCI.vs.control; Homo_714,control,male.vs.female; Homo_633,female,AD.vs.control; Homo_633,female,AD.vs.MCI; Homo_633,female,MCI.vs.control; ROSMAP,female,AD.vs.control; ROSMAP,female,MCI.vs.control; ROSMAP,control,male.vs.female; MCSA,MCI,age; Emory,totalSample,immune-Macrophage; Emory,totalSample,immune-NK; Emory,totalSample,immune-Neutrophil; Emory,totalSample,immune-CD4_T; Emory,totalSample,immune-CD8_T; Emory,totalSample,immune-Gamma_delta; Emory,totalSample,immune-Tr1; Emory,totalSample,immune-iTreg; Emory,totalSample,immune-Tfh; Emory,totalSample,immune-Cytotoxic; Emory,totalSample,immune-Exhausted; Emory,totalSample,immune-MAIT; Emory,totalSample,immune-Effector_memory; Emory,totalSample,immune-InfiltrationScore; Homo_723,totalSample,immune-InfiltrationScore; Homo_723,totalSample,immune-iTreg; Homo_723,totalSample,immune-Exhausted; Homo_723,totalSample,immune-Tfh; Homo_723,totalSample,immune-Macrophage; Homo_723,totalSample,immune-Monocyte; Homo_723,totalSample,immune-CD4_naive; Homo_723,totalSample,immune-CD8_T; Homo_723,totalSample,immune-MAIT; Homo_723,totalSample,immune-CD4_T; Homo_723,totalSample,immune-Tr1; Homo_723,totalSample,immune-Effector_memory; Homo_723,totalSample,immune-Central_memory; Homo_723,totalSample,immune-nTreg; Homo_723,totalSample,immune-Th17; Homo_723,totalSample,immune-Th2; Homo_723,totalSample,immune-Cytotoxic; Homo_714,totalSample,immune-CD4_naive; Homo_714,totalSample,immune-Neutrophil; Homo_714,totalSample,immune-InfiltrationScore; Homo_714,totalSample,immune-MAIT; Homo_714,totalSample,immune-Monocyte; Homo_714,totalSample,immune-Macrophage; Homo_714,totalSample,immune-NKT; Homo_714,totalSample,immune-Effector_memory; Homo_714,totalSample,immune-CD4_T; Homo_714,totalSample,immune-Tfh; Homo_714,totalSample,immune-Th1; Homo_714,totalSample,immune-iTreg; Homo_714,totalSample,immune-Gamma_delta; Homo_714,totalSample,immune-Exhausted; Homo_714,totalSample,immune-nTreg; Homo_633,totalSample,immune-InfiltrationScore; Homo_633,totalSample,immune-CD4_naive; Homo_633,totalSample,immune-Monocyte; Homo_633,totalSample,immune-MAIT; Homo_633,totalSample,immune-Neutrophil; Homo_633,totalSample,immune-Effector_memory; Homo_633,totalSample,immune-NKT; Homo_633,totalSample,immune-Macrophage; Homo_633,totalSample,immune-Tfh; Homo_633,totalSample,immune-Exhausted; Homo_633,totalSample,immune-CD4_T; Homo_633,totalSample,immune-Th1; Homo_633,totalSample,immune-iTreg; MCSA,totalSample,immune-DC; MCSA,totalSample,immune-Monocyte; MCSA,totalSample,immune-Macrophage; MCSA,totalSample,immune-Neutrophil; MCSA,totalSample,immune-CD4_T; MCSA,totalSample,immune-CD4_naive; MCSA,totalSample,immune-Tr1; MCSA,totalSample,immune-nTreg; MCSA,totalSample,immune-iTreg; MCSA,totalSample,immune-Th17; MCSA,totalSample,immune-Tfh; MCSA,totalSample,immune-CD8_naive; MCSA,totalSample,immune-MAIT; MCSA,totalSample,immune-Central_memory; MCSA,totalSample,immune-Effector_memory; MCSA,totalSample,immune-[illegible]
ERAP2	Homo_723,female,AD.vs.control; Homo_723,male,AD.vs.control; Homo_723,female,AD.vs.MCI; Homo_723,male,AD.vs.MCI; Homo_714,female,AD.vs.control; Homo_714,male,AD.vs.control; Homo_714,female,AD.vs.MCI; Homo_714,male,AD.vs.MCI; Homo_633,female,AD.vs.control; Homo_633,female,AD.vs.MCI; Homo_633,female,MCI.vs.control; ROSMAP,MCI,last.visit.age; ROSMAP,MCI,sample.age; Emory,totalSample,immune-Tr1; Emory,totalSample,immune-iTreg; Emory,totalSample,immune-Tfh; Homo_723,totalSample,immune-iTreg; Homo_723,totalSample,immune-Th2; Homo_723,totalSample,immune-CD4_T; Homo_723,totalSample,immune-Tr1; Homo_723,totalSample,immune-DC; Homo_723,totalSample,immune-Effector_memory; Homo_723,totalSample,immune-Bcell; Homo_723,totalSample,immune-CD4_naive; Homo_723,totalSample,immune-InfiltrationScore; Homo_714,totalSample,immune-Tfh; Homo_714,totalSample,immune-Th2; Homo_714,totalSample,immune-Effector_memory; Homo_714,totalSample,immune-CD4_T; Homo_714,totalSample,immune-Central_memory; Homo_714,totalSample,immune-Tr1; Homo_714,totalSample,immune-iTreg; Homo_633,totalSample,immune-Tfh; Homo_633,totalSample,immune-CD4_T; Homo_633,totalSample,immune-Central_memory; Homo_633,totalSample,immune-Th2; Homo_633,totalSample,immune-CD4_naive; Homo_633,totalSample,immune-Effector_memory; MCSA,totalSample,immune-CD4_T; MCSA,totalSample,immune-CD4_naive; MCSA,totalSample,immune-Tr1; MCSA,totalSample,immune-nTreg; MCSA,totalSample,immune-iTreg; MCSA,totalSample,immune-Tfh; MCSA,totalSample,immune-Central_memory; SRP223445,totalSample,immune-CD4_naive
PCCA	Homo_723,female,AD.vs.control; Homo_723,male,AD.vs.control; Homo_723,female,AD.vs.MCI; Homo_723,male,AD.vs.MCI; Homo_714,female,AD.vs.control; Homo_714,male,AD.vs.control; Homo_714,female,AD.vs.MCI; Homo_714,male,AD.vs.MCI; Homo_633,female,AD.vs.control; Homo_633,female,AD.vs.MCI; ACOM,AD,age; ROSMAP,MCI,last.visit.age; ROSMAP,MCI,sample.age; Emory,totalSample,immune-Macrophage; Emory,totalSample,immune-NK; Emory,totalSample,immune-Neutrophil; Emory,totalSample,immune-CD4_T; Emory,totalSample,immune-Tr1; Emory,totalSample,immune-iTreg; Emory,totalSample,immune-Effector_memory; Emory,totalSample,immune-InfiltrationScore; Homo_723,totalSample,immune-Th17; Homo_723,totalSample,immune-Exhausted; Homo_723,totalSample,immune-CD4_naive; Homo_723,totalSample,immune-InfiltrationScore; Homo_723,totalSample,immune-CD4_T; Homo_723,totalSample,immune-Tfh; Homo_723,totalSample,immune-Cytotoxic; Homo_723,totalSample,immune-Th2; Homo_723,totalSample,immune-Tr1; Homo_723,totalSample,immune-Effector_memory; Homo_723,totalSample,immune-nTreg; Homo_723,totalSample,immune-CD8_T; Homo_723,totalSample,immune-Neutrophil; Homo_723,totalSample,immune-Central_memory; Homo_714,totalSample,immune-InfiltrationScore; Homo_714,totalSample,immune-CD4_naive; Homo_714,totalSample,immune-Th17; Homo_714,totalSample,immune-CD4_T; Homo_714,totalSample,immune-Tfh; Homo_714,totalSample,immune-Effector_memory; Homo_714,totalSample,immune-Exhausted; Homo_714,totalSample,immune-NK; Homo_714,totalSample,immune-Bcell; Homo_633,totalSample,immune-Tfh; Homo_633,totalSample,immune-Effector_memory; Homo_633,totalSample,immune-InfiltrationScore; Homo_633,totalSample,immune-Th2; Homo_633,totalSample,immune-CD4_T; Homo_633,totalSample,immune-CD4_naive; Homo_633,totalSample,immune-Central_memory; Homo_633,totalSample,immune-Tr1; Homo_633,totalSample,immune-Bcell; Homo_633,totalSample,immune-Th17; Homo_633,totalSample,immune-nTreg; MCSA,totalSample,immune-Macrophage; MCSA,totalSample,immune-CD4_T; MCSA,totalSample,immune-CD4_naive; MCSA,totalSample,immune-Tr1; MCSA,totalSample,immune-nTreg; MCSA,totalSample,immune-iTreg; MCSA,totalSample,immune-Th17; MCSA,totalSample,immune-Tfh; MCSA,totalSample,immune-Cytotoxic; MCSA,totalSample,immune-MAIT; [illegible]
B4GALT2	Homo_723,female,AD.vs.control; Homo_723,male,AD.vs.control; Homo_723,female,AD.vs.MCI; Homo_723,male,AD.vs.MCI; Homo_723,female,MCI.vs.control; Homo_723,male,MCI.vs.control; Homo_714,female,AD.vs.control; Homo_714,male,AD.vs.control; Homo_714,female,AD.vs.MCI; Homo_714,male,AD.vs.MCI; Homo_633,female,AD.vs.control; Homo_633,female,AD.vs.MCI; ROSMAP,female,AD.vs.control; ROSMAP,female,AD.vs.MCI; MCSA,MCI,age; ROSMAP,AD,sample.age; Emory,totalSample,immune-NK; Emory,totalSample,immune-Neutrophil; Emory,totalSample,immune-CD8_T; Emory,totalSample,immune-Gamma_delta; Emory,totalSample,immune-CD4_naive; Emory,totalSample,immune-Cytotoxic; Emory,totalSample,immune-Exhausted; Emory,totalSample,immune-Effector_memory; Emory,totalSample,immune-InfiltrationScore; Homo_723,totalSample,immune-DC; Homo_723,totalSample,immune-Neutrophil; Homo_723,totalSample,immune-InfiltrationScore; Homo_723,totalSample,immune-Monocyte; Homo_723,totalSample,immune-NK; Homo_723,totalSample,immune-Gamma_delta; Homo_723,totalSample,immune-nTreg; Homo_714,totalSample,immune-InfiltrationScore; Homo_714,totalSample,immune-DC; Homo_714,totalSample,immune-Neutrophil; Homo_714,totalSample,immune-Th2; Homo_714,totalSample,immune-NK; Homo_714,totalSample,immune-Cytotoxic; Homo_714,totalSample,immune-Gamma_delta; Homo_633,totalSample,immune-InfiltrationScore; Homo_633,totalSample,immune-Neutrophil; Homo_633,totalSample,immune-DC; Homo_633,totalSample,immune-Cytotoxic; Homo_633,totalSample,immune-CD8_T; Homo_633,totalSample,immune-Effector_memory; Homo_633,totalSample,immune-MAIT; Homo_633,totalSample,immune-Th2; Homo_633,totalSample,immune-Monocyte; Homo_633,totalSample,immune-Macrophage; Homo_633,totalSample,immune-Gamma_delta; Homo_633,totalSample,immune-NK; MCSA,totalSample,immune-DC; MCSA,totalSample,immune-Monocyte; MCSA,totalSample,immune-Neutrophil; MCSA,totalSample,immune-CD8_T; MCSA,totalSample,immune-NKT; MCSA,totalSample,immune-Gamma_delta; MCSA,totalSample,immune-iTreg; MCSA,totalSample,immune-Th2; MCSA,totalSample,immune-Cytotoxic; MCSA,totalSample,immune-Exhausted; MCSA,totalSample,immune-Effector_memory; MCSA,totalSample,immune-InfiltrationScore; SRP223445,totalSample,immune-CD4_naive; SRP223445,totalSample,immune-[illegible]
BMERB1	Homo_723,female,AD.vs.control; Homo_723,male,AD.vs.control; Homo_723,female,AD.vs.MCI; Homo_723,male,AD.vs.MCI; Homo_714,female,AD.vs.control; Homo_714,female,AD.vs.MCI; Homo_714,male,AD.vs.MCI; Homo_633,female,AD.vs.control; Homo_633,female,AD.vs.MCI; Emory,totalSample,immune-Macrophage; Emory,totalSample,immune-CD4_T; Emory,totalSample,immune-CD8_T; Emory,totalSample,immune-Tr1; Emory,totalSample,immune-iTreg; Emory,totalSample,immune-InfiltrationScore; Homo_723,totalSample,immune-InfiltrationScore; Homo_723,totalSample,immune-Th17; Homo_723,totalSample,immune-Exhausted; Homo_723,totalSample,immune-Tfh; Homo_723,totalSample,immune-CD4_naive; Homo_723,totalSample,immune-CD4_T; Homo_723,totalSample,immune-Th2; Homo_723,totalSample,immune-nTreg; Homo_723,totalSample,immune-NK; Homo_714,totalSample,immune-InfiltrationScore; Homo_714,totalSample,immune-Tfh; Homo_714,totalSample,immune-NK; Homo_633,totalSample,immune-InfiltrationScore; Homo_633,totalSample,immune-Tfh; Homo_633,totalSample,immune-Th2; Homo_633,totalSample,immune-Bcell; Homo_633,totalSample,immune-CD4_T; Homo_633,totalSample,immune-Effector_memory; Homo_633,totalSample,immune-Tr1; MCSA,totalSample,immune-Macrophage; MCSA,totalSample,immune-CD4_T; MCSA,totalSample,immune-CD4_naive; MCSA,totalSample,immune-Tr1; MCSA,totalSample,immune-nTreg; MCSA,totalSample,immune-iTreg; MCSA,totalSample,immune-Tfh; MCSA,totalSample,immune-MAIT; SRP223445,totalSample,immune-InfiltrationScore
ATAD2B	Homo_723,female,AD.vs.control; Homo_723,male,AD.vs.control; Homo_723,female,AD.vs.MCI; Homo_723,male,AD.vs.MCI; Homo_714,female,AD.vs.control; Homo_714,male,AD.vs.control; Homo_714,female,AD.vs.MCI; Homo_714,male,AD.vs.MCI; Homo_633,female,AD.vs.control; Homo_633,female,AD.vs.MCI; MCSA,MCI,age; ROSMAP,MCI,last.visit.age; ROSMAP,MCI,sample.age; Emory,totalSample,immune-Macrophage; Emory,totalSample,immune-CD4_T; Emory,totalSample,immune-Tr1; Emory,totalSample,immune-iTreg; Emory,totalSample,immune-Central_memory; Emory,totalSample,immune-InfiltrationScore; Homo_723,totalSample,immune-CD4_naive; Homo_723,totalSample,immune-Cytotoxic; Homo_723,totalSample,immune-Central_memory; Homo_723,totalSample,immune-CD4_T; Homo_723,totalSample,immune-Th17; Homo_723,totalSample,immune-Tr1; Homo_723,totalSample,immune-Th1; Homo_723,totalSample,immune-Exhausted; Homo_723,totalSample,immune-Tfh; Homo_723,totalSample,immune-NKT; Homo_723,totalSample,immune-CD8_T; Homo_723,totalSample,immune-iTreg; Homo_723,totalSample,immune-InfiltrationScore; Homo_723,totalSample,immune-Gamma_delta; Homo_723,totalSample,immune-nTreg; Homo_714,totalSample,immune-CD4_naive; Homo_714,totalSample,immune-NKT; Homo_714,totalSample,immune-CD4_T; Homo_714,totalSample,immune-Exhausted; Homo_714,totalSample,immune-Th1; Homo_714,totalSample,immune-Central_memory; Homo_714,totalSample,immune-Tfh; Homo_714,totalSample,immune-Th17; Homo_714,totalSample,immune-iTreg; Homo_714,totalSample,immune-Cytotoxic; Homo_714,totalSample,immune-Tr1; Homo_714,totalSample,immune-InfiltrationScore; Homo_714,totalSample,immune-CD8_T; Homo_714,totalSample,immune-Gamma_delta; Homo_633,totalSample,immune-Tfh; Homo_633,totalSample,immune-CD4_naive; Homo_633,totalSample,immune-CD4_T; Homo_633,totalSample,immune-NKT; Homo_633,totalSample,immune-Central_memory; Homo_633,totalSample,immune-Tr1; Homo_633,totalSample,immune-iTreg; Homo_633,totalSample,immune-Effector_memory; Homo_633,totalSample,immune-InfiltrationScore; Homo_633,totalSample,immune-Gamma_delta; Homo_633,totalSample,immune-Th2; Homo_633,totalSample,immune-Th1; Homo_633,totalSample,immune-Exhausted; Homo_633,totalSample,immune-DC; Homo_633,totalSample,immune-Th17; Homo_633,totalSample,immune-Cytotoxic; MCSA,totalSample,immune-DC; MCSA,totalSample,immune-Monocyte; MCSA,totalSample,immune-Macrophage; MCSA,totalSample,immune-CD4_T; MCSA,totalSample,immune-CD8_T; MCSA,totalSample,immune-Gamma_delta; MCSA,totalSample,immune-CD4_naive; MCSA,totalSample,immune-Tr1; MCSA,totalSample,immune-nTreg; MCSA,totalSample,immune-iTreg; MCSA,totalSample,immune-Th17; MCSA,totalSample,immune-Tfh; MCSA,totalSample,immune-CD8_naive; MCSA,totalSample,immune-Cytotoxic; MCSA,totalSample,immune-Exhausted; MCSA,totalSample,immune-MAIT; MCSA,totalSample,immune-Central_memory; ROSMAP,male-[illegible]
GAS2L3	Homo_723,female,AD.vs.control; Homo_723,male,AD.vs.control; Homo_723,female,AD.vs.MCI; Homo_723,male,AD.vs.MCI; Homo_723,female,MCI.vs.control; Homo_723,male,MCI.vs.control; Homo_714,female,AD.vs.control; Homo_714,male,AD.vs.control; Homo_714,female,AD.vs.MCI; Homo_714,male,AD.vs.MCI; Homo_714,female,MCI.vs.control; Homo_714,male,MCI.vs.control; Homo_633,female,AD.vs.control; Homo_633,female,AD.vs.MCI; Emory,MCI,age; MCSA,MCI,age; Emory,totalSample,immune-Macrophage; Emory,totalSample,immune-CD8_T; Emory,totalSample,immune-Gamma_delta; Emory,totalSample,immune-Exhausted; Homo_723,totalSample,immune-Th17; Homo_723,totalSample,immune-Exhausted; Homo_723,totalSample,immune-Cytotoxic; Homo_723,totalSample,immune-CD8_T; Homo_723,totalSample,immune-CD4_naive; Homo_723,totalSample,immune-Gamma_delta; Homo_714,totalSample,immune-Exhausted; Homo_714,totalSample,immune-Th17; Homo_714,totalSample,immune-Monocyte; Homo_714,totalSample,immune-CD8_T; Homo_714,totalSample,immune-Cytotoxic; Homo_714,totalSample,immune-Gamma_delta; Homo_633,totalSample,immune-Gamma_delta; Homo_633,totalSample,immune-Tfh; Homo_633,totalSample,immune-Th17; Homo_633,totalSample,immune-Th2; Homo_633,totalSample,immune-Monocyte; Homo_633,totalSample,immune-CD4_T; Homo_633,totalSample,immune-Exhausted; Homo_633,totalSample,immune-NKT; Homo_633,totalSample,immune-CD4_naive; Homo_633,totalSample,immune-Cytotoxic; MCSA,totalSample,immune-DC; MCSA,totalSample,immune-Monocyte; MCSA,totalSample,immune-CD4_T; MCSA,totalSample,immune-CD8_T; MCSA,totalSample,immune-Gamma_delta; MCSA,totalSample,immune-CD4_naive; MCSA,totalSample,immune-Tr1; MCSA,totalSample,immune-nTreg; MCSA,totalSample,immune-iTreg; MCSA,totalSample,immune-Th17; MCSA,totalSample,immune-Tfh; MCSA,totalSample,immune-CD8_naive; MCSA,totalSample,immune-Cytotoxic; MCSA,totalSample,immune-Exhausted; MCSA,totalSample,immune-Central_memory; ROSMAP,totalSample,immune-[illegible]
THEM6	Homo_723,female,AD.vs.control; Homo_723,male,AD.vs.control; Homo_723,female,AD.vs.MCI; Homo_723,male,AD.vs.MCI; Homo_723,female,MCI.vs.control; Homo_714,female,AD.vs.control; Homo_714,male,AD.vs.control; Homo_714,female,AD.vs.MCI; Homo_714,male,AD.vs.MCI; Homo_714,female,MCI.vs.control; Homo_633,female,AD.vs.control; Homo_633,female,AD.vs.MCI; ROSMAP,female,AD.vs.control; MCSA,totalSample,age; MCSA,control,age; MCSA,MCI,age; Emory,totalSample,immune-Monocyte; Emory,totalSample,immune-Macrophage; Emory,totalSample,immune-NK; Emory,totalSample,immune-Neutrophil; Emory,totalSample,immune-CD4_T; Emory,totalSample,immune-CD8_T; Emory,totalSample,immune-Tr1; Emory,totalSample,immune-nTreg; Emory,totalSample,immune-iTreg; Emory,totalSample,immune-Tfh; Emory,totalSample,immune-Exhausted; Emory,totalSample,immune-MAIT; Emory,totalSample,immune-Central_memory; Emory,totalSample,immune-InfiltrationScore; Homo_723,totalSample,immune-CD8_T; Homo_723,totalSample,immune-Exhausted; Homo_723,totalSample,immune-Monocyte; Homo_723,totalSample,immune-Cytotoxic; Homo_723,totalSample,immune-DC; Homo_723,totalSample,immune-Central_memory; Homo_723,totalSample,immune-Th17; Homo_723,totalSample,immune-InfiltrationScore; Homo_723,totalSample,immune-nTreg; Homo_723,totalSample,immune-Macrophage; Homo_723,totalSample,immune-MAIT; Homo_723,totalSample,immune-CD8_naive; Homo_723,totalSample,immune-iTreg; Homo_723,totalSample,immune-Tfh; Homo_714,totalSample,immune-Monocyte; Homo_714,totalSample,immune-CD8_T; Homo_714,totalSample,immune-DC; Homo_714,totalSample,immune-nTreg; Homo_714,totalSample,immune-Macrophage; Homo_714,totalSample,immune-InfiltrationScore; Homo_714,totalSample,immune-Effector_memory; Homo_714,totalSample,immune-Exhausted; Homo_714,totalSample,immune-Th17; Homo_714,totalSample,immune-Cytotoxic; Homo_714,totalSample,immune-Tfh; Homo_633,totalSample,immune-Monocyte; Homo_633,totalSample,immune-DC; Homo_633,totalSample,immune-nTreg; Homo_633,totalSample,immune-InfiltrationScore; Homo_633,totalSample,immune-Th2; Homo_633,totalSample,immune-Neutrophil; Homo_633,totalSample,immune-CD8_T; MCSA,totalSample,immune-DC; MCSA,totalSample,immune-Monocyte; MCSA,totalSample,immune-Neutrophil; MCSA,totalSample,immune-CD8_T; MCSA,totalSample,immune-NKT; MCSA,totalSample,immune-Th2; MCSA,totalSample,immune-Tfh; MCSA,totalSample,immune-Cytotoxic; MCSA,totalSample,immune-Central_memory; MCSA,totalSample,immune-Effector_memory; MCSA,totalSample,immune-InfiltrationScore; ROSMAP,totalSample,immune-Th17; [illegible]
FGFR1	Homo_723,female,AD.vs.control; Homo_723,male,AD.vs.control; Homo_723,female,AD.vs.MCI; Homo_723,male,AD.vs.MCI; Homo_723,female,MCI.vs.control; Homo_723,male,MCI.vs.control; Homo_714,female,AD.vs.control; Homo_714,female,MCI.vs.control; Homo_714,male,MCI.vs.control; Homo_633,female,AD.vs.control; Homo_633,female,AD.vs.MCI; Homo_633,female,MCI.vs.control; Homo_633,male,MCI.vs.control; ROSMAP,female,AD.vs.control; ROSMAP,male,MCI.vs.control; ROSMAP,MCI,male.vs.female; Emory,MCI,age; MCSA,totalSample,age; MCSA,control,age; MCSA,MCI,age; Emory,totalSample,immune-Tr1; Emory,totalSample,immune-iTreg; Homo_723,totalSample,immune-Monocyte; Homo_723,totalSample,immune-DC; Homo_723,totalSample,immune-nTreg; Homo_723,totalSample,immune-Central_memory; Homo_714,totalSample,immune-Monocyte; Homo_714,totalSample,immune-InfiltrationScore; Homo_633,totalSample,immune-Monocyte; Homo_633,totalSample,immune-InfiltrationScore; MCSA,totalSample,immune-Monocyte; MCSA,totalSample,immune-CD4_T; MCSA,totalSample,immune-CD4_naive; MCSA,totalSample,immune-Tr1; MCSA,totalSample,immune-nTreg; MCSA,totalSample,immune-Th17; MCSA,totalSample,immune-Tfh; MCSA,totalSample,immune-Central_memory; MCSA,totalSample,immune-Effector_memory; MCSA,totalSample,immune-InfiltrationScore;
ARHGAP21	Homo_723,female,AD.vs.control; Homo_723,male,AD.vs.control; Homo_723,female,AD.vs.MCI; Homo_723,male,AD.vs.MCI; Homo_723,female,MCI.vs.control; Homo_723,male,MCI.vs.control; Homo_714,female,AD.vs.control; Homo_714,male,AD.vs.control; Homo_714,female,AD.vs.MCI; Homo_714,male,AD.vs.MCI; Homo_714,female,MCI.vs.control; Homo_714,male,MCI.vs.control; Homo_633,female,AD.vs.control; Homo_633,female,AD.vs.MCI; Homo_633,female,MCI.vs.control; Homo_633,male,MCI.vs.control; ACOM,totalSample,age; Emory,totalSample,immune-Tr1; Emory,totalSample,immune-iTreg; Homo_723,totalSample,immune-Exhausted; Homo_723,totalSample,immune-Th17; Homo_723,totalSample,immune-Cytotoxic; Homo_723,totalSample,immune-CD8_T; Homo_723,totalSample,immune-CD4_naive; Homo_723,totalSample,immune-CD4_T; Homo_723,totalSample,immune-Monocyte; Homo_723,totalSample,immune-Tfh; Homo_723,totalSample,immune-Th2; Homo_723,totalSample,immune-Central_memory; Homo_723,totalSample,immune-InfiltrationScore; Homo_723,totalSample,immune-Tr1; Homo_723,totalSample,immune-nTreg; Homo_714,totalSample,immune-Th17; Homo_714,totalSample,immune-Exhausted; Homo_714,totalSample,immune-CD4_naive; Homo_714,totalSample,immune-Monocyte; Homo_714,totalSample,immune-Central_memory; Homo_714,totalSample,immune-InfiltrationScore; Homo_714,totalSample,immune-CD4_T; Homo_714,totalSample,immune-Tfh; Homo_714,totalSample,immune-Gamma_delta; Homo_633,totalSample,immune-Th2; Homo_633,totalSample,immune-Tfh; Homo_633,totalSample,immune-CD4_T; Homo_633,totalSample,immune-Effector_memory; Homo_633,totalSample,immune-Central_memory; Homo_633,totalSample,immune-Exhausted; Homo_633,totalSample,immune-Monocyte; Homo_633,totalSample,immune-CD4_naive; Homo_633,totalSample,immune-InfiltrationScore; Homo_633,totalSample,immune-Gamma_delta; Homo_633,totalSample,immune-Th17; Homo_633,totalSample,immune-Tr1; MCSA,totalSample,immune-CD4_T; MCSA,totalSample,immune-Gamma_delta; MCSA,totalSample,immune-CD4_naive; MCSA,totalSample,immune-Tr1; MCSA,totalSample,immune-nTreg; MCSA,totalSample,immune-iTreg; MCSA,totalSample,immune-Th1; MCSA,totalSample,immune-Th17; MCSA,totalSample,immune-Tfh; MCSA,totalSample,immune-CD8_naive; MCSA,totalSample,immune-Cytotoxic; MCSA,totalSample,immune-Exhausted; MCSA,totalSample,immune-MAIT; MCSA,totalSample,immune-Central_memory; ROSMAP,totalSample,immune-Effector_memory; SRP223445,totalSample,immune-CD4_naive; SRP223445,totalSample,immune-Exhausted

LRRN2	Homo_723,female,AD.vs.control; Homo_723,male,AD.vs.control; Homo_723,female,AD.vs.MCI; Homo_723,male,AD.vs.MCI; Homo_723,female,MCI.vs.control; Homo_714,female,AD.vs.control; Homo_714,male,AD.vs.MCI; Homo_633,female,AD.vs.control; Homo_633,female,AD.vs.MCI; MCSA,totalSample,age; MCSA,control,age; Emory,totalSample,immune-CD8_T; Emory,totalSample,immune-Tfh; Emory,totalSample,immune-MAIT; Homo_723,totalSample,immune-nTreg; Homo_723,totalSample,immune-Effector_memory; Homo_723,totalSample,immune-InfiltrationScore; Homo_714,totalSample,immune-Effector_memory; Homo_714,totalSample,immune-InfiltrationScore; Homo_714,totalSample,immune-nTreg; Homo_714,totalSample,immune-Gamma_delta; Homo_633,totalSample,immune-nTreg; Homo_633,totalSample,immune-Gamma_delta; Homo_633,totalSample,immune-InfiltrationScore; Homo_633,totalSample,immune-Exhausted; Homo_633,totalSample,immune-Central_memory; Homo_633,totalSample,immune-Effector_memory; Homo_633,totalSample,immune-Tfh; MCSA,totalSample,immune-DC; MCSA,totalSample,immune-Monocyte; MCSA,totalSample,immune-Neutrophil; MCSA,totalSample,immune-CD4_T; MCSA,totalSample,immune-CD8_T; MCSA,totalSample,immune-NKT; MCSA,totalSample,immune-Gamma_delta; MCSA,totalSample,immune-CD4_naive; MCSA,totalSample,immune-Tr1; MCSA,totalSample,immune-nTreg; MCSA,totalSample,immune-Th2; MCSA,totalSample,immune-Tfh; MCSA,totalSample,immune-CD8_naive; MCSA,totalSample,immune-Exhausted; MCSA,totalSample,immune-Central_memory; MCSA,totalSample,immune-Effector_memory; ROSMAP,totalSample,immune-InfiltrationScore
IGF2BP3	Homo_723,female,AD.vs.control; Homo_723,male,AD.vs.control; Homo_723,female,AD.vs.MCI; Homo_723,male,AD.vs.MCI; Homo_723,female,MCI.vs.control; Homo_723,male,MCI.vs.control; Homo_714,female,AD.vs.control; Homo_714,male,AD.vs.control; Homo_714,female,AD.vs.MCI; Homo_714,male,AD.vs.MCI; Homo_633,female,AD.vs.control; Homo_633,female,AD.vs.MCI; Homo_633,female,MCI.vs.control; Homo_633,male,MCI.vs.control; ACOM,totalSample,age; MCSA,MCI,age; Emory,totalSample,immune-Th17; Emory,totalSample,immune-Cytotoxic; Homo_723,totalSample,immune-DC; Homo_723,totalSample,immune-Cytotoxic; Homo_723,totalSample,immune-Th1; Homo_714,totalSample,immune-CD8_T; Homo_714,totalSample,immune-NKT; Homo_714,totalSample,immune-Th1; Homo_633,totalSample,immune-NKT; Homo_633,totalSample,immune-Th1; MCSA,totalSample,immune-Monocyte; MCSA,totalSample,immune-CD4_T; MCSA,totalSample,immune-CD8_T; MCSA,totalSample,immune-Gamma_delta; MCSA,totalSample,immune-CD4_naive; MCSA,totalSample,immune-Tr1; MCSA,totalSample,immune-nTreg; MCSA,totalSample,immune-iTreg; MCSA,totalSample,immune-Cytotoxic; MCSA,totalSample,immune-Exhausted; SRP223445,totalSample,immune-CD4_naive; SRP223445,totalSample,immune-Exhausted
ADCY5	Homo_723,female,AD.vs.control; Homo_723,male,AD.vs.control; Homo_723,female,AD.vs.MCI; Homo_723,male,AD.vs.MCI; Homo_714,male,AD.vs.control; Homo_714,female,AD.vs.MCI; Homo_714,male,AD.vs.MCI; Homo_633,female,AD.vs.control; Homo_633,female,AD.vs.MCI; ROSMAP,female,AD.vs.control; ROSMAP,male,AD.vs.control; MCSA,totalSample,age; MCSA,control,age; ROSMAP,MCI,last.visit.age; Emory,totalSample,immune-Th17; Homo_723,totalSample,immune-Tfh; Homo_723,totalSample,immune-CD4_T; Homo_723,totalSample,immune-Th2; Homo_723,totalSample,immune-CD4_naive; Homo_723,totalSample,immune-nTreg; Homo_723,totalSample,immune-iTreg; Homo_723,totalSample,immune-Central_memory; Homo_723,totalSample,immune-Tr1; Homo_723,totalSample,immune-Macrophage; Homo_723,totalSample,immune-Effector_memory; Homo_723,totalSample,immune-Th17; Homo_714,totalSample,immune-Tfh; Homo_714,totalSample,immune-CD4_T; Homo_714,totalSample,immune-CD4_naive; Homo_714,totalSample,immune-Th2; Homo_714,totalSample,immune-nTreg; Homo_714,totalSample,immune-Central_memory; Homo_714,totalSample,immune-Effector_memory; Homo_714,totalSample,immune-Macrophage; Homo_714,totalSample,immune-iTreg; Homo_714,totalSample,immune-InfiltrationScore; Homo_714,totalSample,immune-Tr1; Homo_633,totalSample,immune-Tfh; Homo_633,totalSample,immune-CD4_T; Homo_633,totalSample,immune-nTreg; Homo_633,totalSample,immune-CD4_naive; Homo_633,totalSample,immune-InfiltrationScore; Homo_633,totalSample,immune-Effector_memory; Homo_633,totalSample,immune-Central_memory; Homo_633,totalSample,immune-Th2; Homo_633,totalSample,immune-Tr1; Homo_633,totalSample,immune-iTreg; Homo_633,totalSample,immune-Th17; MCSA,totalSample,immune-Monocyte; MCSA,totalSample,immune-Macrophage; MCSA,totalSample,immune-Neutrophil; MCSA,totalSample,immune-CD4_T; MCSA,totalSample,immune-CD8_T; MCSA,totalSample,immune-CD4_naive; MCSA,totalSample,immune-Tr1; MCSA,totalSample,immune-nTreg; MCSA,totalSample,immune-iTreg; MCSA,totalSample,immune-Th17; MCSA,totalSample,immune-Tfh; MCSA,totalSample,immune-CD8_naive; MCSA,totalSample,immune-Central_memory; MCSA,totalSample,immune-Effector_memory; MCSA,totalSample,immune-InfiltrationScore; ROSMAP,totalSample,immune-InfiltrationScore
CDC14C	Homo_723,female,AD.vs.control; Homo_723,male,AD.vs.control; Homo_723,female,AD.vs.MCI; Homo_723,male,AD.vs.MCI; Homo_714,female,AD.vs.control; Homo_714,male,AD.vs.control; Homo_714,female,AD.vs.MCI; Homo_714,male,AD.vs.MCI; Homo_633,female,MCI.vs.control; Homo_633,male,MCI.vs.control; Homo_723,totalSample,immune-DC; Homo_723,totalSample,immune-Monocyte; Homo_714,totalSample,immune-NKT; Homo_714,totalSample,immune-Th2; Homo_633,totalSample,immune-NKT; Homo_633,totalSample,immune-Monocyte; MCSA,totalSample,immune-CD4_T; MCSA,totalSample,immune-CD4_naive; MCSA,totalSample,immune-Tr1; MCSA,totalSample,immune-nTreg; MCSA,totalSample,immune-iTreg; MCSA,totalSample,immune-Tfh; MCSA,totalSample,immune-CD8_naive; MCSA,totalSample,immune-MAIT; MCSA,totalSample,immune-Central_memory; SRP223445,totalSample,immune-CD4_naive; SRP223445,totalSample,immune-Exhausted; SRP223445,totalSample,immune-InfiltrationScore
FAM83D	Homo_723,female,AD.vs.control; Homo_723,male,AD.vs.control; Homo_723,female,AD.vs.MCI; Homo_723,male,AD.vs.MCI; Homo_723,female,MCI.vs.control; Homo_714,female,AD.vs.control; Homo_714,male,AD.vs.control; Homo_714,female,AD.vs.MCI; Homo_714,male,AD.vs.MCI; Homo_714,female,MCI.vs.control; Homo_633,female,AD.vs.control; Homo_633,female,AD.vs.MCI; ROSMAP,AD,MMSE; Emory,totalSample,immune-Neutrophil; Emory,totalSample,immune-CD4_T; Emory,totalSample,immune-CD8_T; Emory,totalSample,immune-Tr1; Emory,totalSample,immune-iTreg; Emory,totalSample,immune-InfiltrationScore; Homo_723,totalSample,immune-Central_memory; Homo_723,totalSample,immune-CD4_naive; Homo_723,totalSample,immune-nTreg; Homo_723,totalSample,immune-DC; Homo_723,totalSample,immune-CD4_T; Homo_723,totalSample,immune-Th17; Homo_723,totalSample,immune-Th1; Homo_723,totalSample,immune-Neutrophil; Homo_723,totalSample,immune-Tr1; Homo_723,totalSample,immune-Tfh; Homo_723,totalSample,immune-InfiltrationScore; Homo_723,totalSample,immune-Cytotoxic; Homo_714,totalSample,immune-CD4_naive; Homo_714,totalSample,immune-Th1; Homo_714,totalSample,immune-InfiltrationScore; Homo_714,totalSample,immune-nTreg; Homo_714,totalSample,immune-Central_memory; Homo_714,totalSample,immune-NKT; Homo_714,totalSample,immune-CD4_T; Homo_714,totalSample,immune-Tfh; Homo_714,totalSample,immune-Th17; Homo_714,totalSample,immune-Neutrophil; Homo_633,totalSample,immune-Tfh; Homo_633,totalSample,immune-CD4_T; Homo_633,totalSample,immune-Tr1; Homo_633,totalSample,immune-nTreg; Homo_633,totalSample,immune-InfiltrationScore; Homo_633,totalSample,immune-CD4_naive; Homo_633,totalSample,immune-Central_memory; Homo_633,totalSample,immune-iTreg; Homo_633,totalSample,immune-Effector_memory; Homo_633,totalSample,immune-NKT; Homo_633,totalSample,immune-Th1; MCSA,totalSample,immune-Monocyte; MCSA,totalSample,immune-Neutrophil; MCSA,totalSample,immune-CD4_T; MCSA,totalSample,immune-CD4_naive; MCSA,totalSample,immune-Tr1; MCSA,totalSample,immune-nTreg; MCSA,totalSample,immune-iTreg; MCSA,totalSample,immune-Th17; MCSA,totalSample,immune-Tfh; MCSA,totalSample,immune-CD8_naive; MCSA,totalSample,immune-MAIT; MCSA,totalSample,immune-Central_memory; MCSA,totalSample,immune-Effector_memory; [illegible]
STARD4	Homo_723,female,AD.vs.control; Homo_723,male,AD.vs.control; Homo_723,female,AD.vs.MCI; Homo_723,male,AD.vs.MCI; Homo_714,female,AD.vs.control; Homo_714,male,AD.vs.control; Homo_714,female,AD.vs.MCI; Homo_714,male,AD.vs.MCI; Homo_633,female,AD.vs.control; Homo_633,female,AD.vs.MCI; ROSMAP,female,AD.vs.control; SRP223445,female,AD.vs.control; MCSA,MCI,age; ROSMAP,MCI,last.visit.age; ROSMAP,MCI,sample.age; Emory,totalSample,immune-Monocyte; Emory,totalSample,immune-Macrophage; Emory,totalSample,immune-NK; Emory,totalSample,immune-Neutrophil; Emory,totalSample,immune-CD4_T; Emory,totalSample,immune-CD8_T; Emory,totalSample,immune-Tr1; Emory,totalSample,immune-iTreg; Emory,totalSample,immune-Tfh; Emory,totalSample,immune-Effector_memory; Emory,totalSample,immune-InfiltrationScore; Homo_723,totalSample,immune-CD4_naive; Homo_723,totalSample,immune-Exhausted; Homo_723,totalSample,immune-Cytotoxic; Homo_723,totalSample,immune-CD4_T; Homo_723,totalSample,immune-InfiltrationScore; Homo_723,totalSample,immune-Tfh; Homo_723,totalSample,immune-Tr1; Homo_723,totalSample,immune-Th1; Homo_723,totalSample,immune-Th17; Homo_723,totalSample,immune-CD8_T; Homo_723,totalSample,immune-Central_memory; Homo_723,totalSample,immune-NKT; Homo_723,totalSample,immune-Neutrophil; Homo_723,totalSample,immune-NK; Homo_723,totalSample,immune-Th2; Homo_714,totalSample,immune-CD4_naive; Homo_714,totalSample,immune-InfiltrationScore; Homo_714,totalSample,immune-NKT; Homo_714,totalSample,immune-Tfh; Homo_714,totalSample,immune-CD4_T; Homo_714,totalSample,immune-Monocyte; Homo_714,totalSample,immune-Central_memory; Homo_714,totalSample,immune-NK; Homo_714,totalSample,immune-Exhausted; Homo_714,totalSample,immune-Bcell; Homo_633,totalSample,immune-Tfh; Homo_633,totalSample,immune-CD4_naive; Homo_633,totalSample,immune-CD4_T; Homo_633,totalSample,immune-InfiltrationScore; Homo_633,totalSample,immune-Effector_memory; Homo_633,totalSample,immune-NKT; Homo_633,totalSample,immune-Central_memory; Homo_633,totalSample,immune-Th2; Homo_633,totalSample,immune-Tr1; Homo_633,totalSample,immune-Monocyte; Homo_633,totalSample,immune-Bcell; Homo_633,totalSample,immune-iTreg; Homo_633,totalSample,immune-DC; MCSA,totalSample,immune-DC; MCSA,totalSample,immune-Monocyte; MCSA,totalSample,immune-Macrophage; MCSA,totalSample,immune-CD4_T; MCSA,totalSample,immune-CD8_T; MCSA,totalSample,immune-CD4_naive; MCSA,totalSample,immune-Tr1; MCSA,totalSample,immune-nTreg; MCSA,totalSample,immune-iTreg; MCSA,totalSample,immune-Tfh; MCSA,totalSample,immune-CD8_naive; MCSA,totalSample,immune-Cytotoxic; MCSA,totalSample,immune-Exhausted; MCSA,totalSample,immune-MAIT; MCSA,totalSample,immune-Central_memory; ROSMAP,totalSample,immune-Macrophage; ROSMAP,totalSample,immune-Effector_memory; SRP223445,totalSample,immune-NK
CARS1	Homo_723,female,AD.vs.control; Homo_723,male,AD.vs.control; Homo_723,female,AD.vs.MCI; Homo_723,male,AD.vs.MCI; Homo_714,female,AD.vs.control; Homo_714,male,AD.vs.control; Homo_714,female,AD.vs.MCI; Homo_714,male,AD.vs.MCI; Homo_633,female,AD.vs.control; Homo_633,female,AD.vs.MCI; Emory,MCI,age; MCSA,totalSample,age; MCSA,control,age; ROSMAP,MCI,last.visit.age; Emory,totalSample,immune-B_cell; Emory,totalSample,immune-Neutrophil; Emory,totalSample,immune-CD8_T; Emory,totalSample,immune-Gamma_delta; Emory,totalSample,immune-Tfh; Emory,totalSample,immune-CD8_naive; Emory,totalSample,immune-InfiltrationScore; Homo_723,totalSample,immune-InfiltrationScore; Homo_723,totalSample,immune-Effector_memory; Homo_723,totalSample,immune-Th2; Homo_723,totalSample,immune-Tfh; Homo_723,totalSample,immune-Bcell; Homo_723,totalSample,immune-iTreg; Homo_723,totalSample,immune-Th1; Homo_723,totalSample,immune-CD4_T; Homo_714,totalSample,immune-Th2; Homo_714,totalSample,immune-Effector_memory; Homo_714,totalSample,immune-Tfh; Homo_714,totalSample,immune-InfiltrationScore; Homo_714,totalSample,immune-Th1; Homo_633,totalSample,immune-InfiltrationScore; Homo_633,totalSample,immune-Effector_memory; Homo_633,totalSample,immune-Th2; Homo_633,totalSample,immune-Exhausted; Homo_633,totalSample,immune-Tfh; Homo_633,totalSample,immune-nTreg; Homo_633,totalSample,immune-Central_memory; Homo_633,totalSample,immune-Th17; Homo_633,totalSample,immune-CD4_T; MCSA,totalSample,immune-DC; MCSA,totalSample,immune-Monocyte; MCSA,totalSample,immune-CD4_T; MCSA,totalSample,immune-NKT; MCSA,totalSample,immune-Gamma_delta; MCSA,totalSample,immune-CD4_naive; MCSA,totalSample,immune-Tr1; MCSA,totalSample,immune-nTreg; MCSA,totalSample,immune-Th2; MCSA,totalSample,immune-Th17; MCSA,totalSample,immune-Tfh; MCSA,totalSample,immune-Exhausted; MCSA,totalSample,immune-Central_memory; MCSA,totalSample,immune-Effector_memory; MCSA,totalSample,immune-InfiltrationScore; ROSMAP,totalSample,immune-Th17; ROSMAP,totalSample,immune-Effector_memory; SRP223445,totalSample,immune-Th17; SRP223445,totalSample,immune-Exhausted; SRP223445,totalSample,immune-InfiltrationScore; ROSMAP,male-excludeAge90+,survival
C1orf116	Homo_723,female,AD.vs.control; Homo_723,male,AD.vs.control; Homo_723,female,AD.vs.MCI; Homo_723,male,AD.vs.MCI; Homo_714,female,AD.vs.control; Homo_714,male,AD.vs.control; Homo_714,female,AD.vs.MCI; Homo_714,male,AD.vs.MCI; Homo_633,female,AD.vs.control; Homo_633,female,AD.vs.MCI; ROSMAP,female,AD.vs.control; Emory,MCI,age; MCSA,MCI,age; ROSMAP,MCI,sample.age; Emory,totalSample,immune-Th1; Homo_723,totalSample,immune-DC; Homo_714,totalSample,immune-DC; Homo_714,totalSample,immune-Th2; Homo_633,totalSample,immune-DC; MCSA,totalSample,immune-DC; MCSA,totalSample,immune-Monocyte; MCSA,totalSample,immune-Th17; SRP223445,totalSample,immune- [illegible]
LOC107986860	Homo_723,female,AD.vs.control; Homo_723,male,AD.vs.control; Homo_723,female,AD.vs.MCI; Homo_723,male,AD.vs.MCI; Homo_714,female,AD.vs.control; Homo_714,male,AD.vs.control; Homo_714,female,AD.vs.MCI; Homo_714,male,AD.vs.MCI; Homo_633,female,AD.vs.control; Homo_633,female,AD.vs.MCI; Emory,totalSample,immune-CD4_T; Emory,totalSample,immune-CD8_T; Emory,totalSample,immune-Tr1; Emory,totalSample,immune-iTreg; Emory,totalSample,immune-Tfh; Emory,totalSample,immune-Central_memory; Homo_723,totalSample,immune-Tfh; Homo_723,totalSample,immune-iTreg; Homo_723,totalSample,immune-Th2; Homo_723,totalSample,immune-CD8_T; Homo_723,totalSample,immune-Tr1; Homo_723,totalSample,immune-DC; Homo_723,totalSample,immune-CD4_T; Homo_723,totalSample,immune-Macrophage; Homo_723,totalSample,immune-CD4_naive; Homo_714,totalSample,immune-Tfh; Homo_714,totalSample,immune-Th2; Homo_714,totalSample,immune-Tr1; Homo_714,totalSample,immune-CD4_T; Homo_714,totalSample,immune-Central_memory; Homo_714,totalSample,immune-CD8_T; Homo_714,totalSample,immune-iTreg; Homo_633,totalSample,immune-Tfh; Homo_633,totalSample,immune-CD4_naive; Homo_633,totalSample,immune-Tr1; Homo_633,totalSample,immune-Central_memory; Homo_633,totalSample,immune-CD4_T; MCSA,totalSample,immune-Macrophage;
GPSM1	Homo_723,female,AD.vs.control; Homo_723,male,AD.vs.control; Homo_723,female,AD.vs.MCI; Homo_723,male,AD.vs.MCI; Homo_633,female,AD.vs.control; Homo_633,female,AD.vs.MCI; ROSMAP,female,AD.vs.control; ROSMAP,male,AD.vs.control; ROSMAP,AD,male.vs.female; ROSMAP,control,male.vs.female; MCSA,totalSample,age; MCSA,control,age; MCSA,MCI,age; ROSMAP,MCI,last.visit.age; Emory,totalSample,immune-Monocyte; Emory,totalSample,immune-Neutrophil; Emory,totalSample,immune-CD4_T; Emory,totalSample,immune-Tr1; Emory,totalSample,immune-nTreg; Emory,totalSample,immune-iTreg; Emory,totalSample,immune-InfiltrationScore; Homo_723,totalSample,immune-DC; Homo_723,totalSample,immune-Monocyte; Homo_723,totalSample,immune-nTreg; Homo_723,totalSample,immune-Neutrophil; Homo_723,totalSample,immune-Central_memory; Homo_723,totalSample,immune-CD8_T; Homo_723,totalSample,immune-InfiltrationScore; Homo_714,totalSample,immune-DC; Homo_714,totalSample,immune-Monocyte; Homo_714,totalSample,immune-InfiltrationScore; Homo_714,totalSample,immune-nTreg; Homo_633,totalSample,immune-DC; Homo_633,totalSample,immune-Monocyte; Homo_633,totalSample,immune-nTreg; Homo_633,totalSample,immune-InfiltrationScore; MCSA,totalSample,immune-DC; MCSA,totalSample,immune-Monocyte; MCSA,totalSample,immune-Neutrophil; MCSA,totalSample,immune-CD8_T; MCSA,totalSample,immune-NKT; MCSA,totalSample,immune-CD4_naive; MCSA,totalSample,immune-Th2; MCSA,totalSample,immune-Th17; MCSA,totalSample,immune-Tfh; MCSA,totalSample,immune-Cytotoxic; MCSA,totalSample,immune-Central_memory; MCSA,totalSample,immune-Effector_memory; MCSA,totalSample,immune-InfiltrationScore
TRPM6	Homo_723,female,AD.vs.control; Homo_723,male,AD.vs.control; Homo_723,female,AD.vs.MCI; Homo_723,male,AD.vs.MCI; Homo_723,female,MCI.vs.control; Homo_714,female,AD.vs.control; Homo_714,male,AD.vs.control; Homo_714,female,AD.vs.MCI; Homo_714,male,AD.vs.MCI; Homo_714,female,MCI.vs.control; Homo_633,female,AD.vs.control; Homo_633,female,AD.vs.MCI; Homo_633,female,MCI.vs.control; Emory,totalSample,age; Emory,MCI,age; Emory,totalSample,immune-Monocyte; Emory,totalSample,immune-Macrophage; Emory,totalSample,immune-Neutrophil; Emory,totalSample,immune-CD8_T; Emory,totalSample,immune-Gamma_delta; Emory,totalSample,immune-Th1; Emory,totalSample,immune-Th17; Emory,totalSample,immune-Tfh; Emory,totalSample,immune-CD8_naive; Emory,totalSample,immune-Cytotoxic; Emory,totalSample,immune-Exhausted; Emory,totalSample,immune-MAIT; Homo_723,totalSample,immune-Monocyte; Homo_723,totalSample,immune-DC; Homo_723,totalSample,immune-Neutrophil; Homo_723,totalSample,immune-Th2; Homo_723,totalSample,immune-Gamma_delta; Homo_723,totalSample,immune-Exhausted; Homo_723,totalSample,immune-Bcell; Homo_723,totalSample,immune-Tfh; Homo_723,totalSample,immune-Th1; Homo_714,totalSample,immune-Th2; Homo_714,totalSample,immune-Monocyte; Homo_714,totalSample,immune-Neutrophil; Homo_714,totalSample,immune-Gamma_delta; Homo_714,totalSample,immune-Tfh; Homo_714,totalSample,immune-Th1; Homo_633,totalSample,immune-Th2; Homo_633,totalSample,immune-Monocyte; Homo_633,totalSample,immune-Exhausted; Homo_633,totalSample,immune-Th17; Homo_633,totalSample,immune-Gamma_delta; Homo_633,totalSample,immune-Neutrophil; Homo_633,totalSample,immune-CD8_T; MCSA,totalSample,immune-DC; MCSA,totalSample,immune-Neutrophil; MCSA,totalSample,immune-CD8_T; MCSA,totalSample,immune-Gamma_delta; MCSA,totalSample,immune-iTreg; MCSA,totalSample,immune-Th1; MCSA,totalSample,immune-Th17; MCSA,totalSample,immune-Cytotoxic; MCSA,totalSample,immune- [illegible]
TTC22	Homo_723,female,AD.vs.control; Homo_723,male,AD.vs.control; Homo_723,female,AD.vs.MCI; Homo_723,male,AD.vs.MCI; Homo_714,female,AD.vs.control; Homo_714,male,AD.vs.control; Homo_714,female,AD.vs.MCI; Homo_714,male,AD.vs.MCI; Homo_633,female,AD.vs.control; Homo_633,female,AD.vs.MCI; ROSMAP,female,AD.vs.control; ROSMAP,female,AD.vs.MCI; ROSMAP,female,MCI.vs.control; Emory,totalSample,immune-Monocyte; Emory,totalSample,immune-Macrophage; Emory,totalSample,immune-NK; Emory,totalSample,immune-Neutrophil; Emory,totalSample,immune-CD4_T; Emory,totalSample,immune-CD8_T; Emory,totalSample,immune-Gamma_delta; Emory,totalSample,immune-Tr1; Emory,totalSample,immune-iTreg; Emory,totalSample,immune-Cytotoxic; Emory,totalSample,immune-Exhausted; Emory,totalSample,immune-Effector_memory; Emory,totalSample,immune-InfiltrationScore; Homo_723,totalSample,immune-InfiltrationScore; Homo_723,totalSample,immune-Monocyte; Homo_723,totalSample,immune-Exhausted; Homo_723,totalSample,immune-CD8_T; Homo_723,totalSample,immune-Cytotoxic; Homo_723,totalSample,immune-Macrophage; Homo_723,totalSample,immune-MAIT; Homo_723,totalSample,immune-Tfh; Homo_723,totalSample,immune-Effector_memory; Homo_723,totalSample,immune-iTreg; Homo_723,totalSample,immune-CD4_naive; Homo_723,totalSample,immune-Bcell; Homo_723,totalSample,immune-nTreg; Homo_714,totalSample,immune-InfiltrationScore; Homo_714,totalSample,immune-Monocyte; Homo_714,totalSample,immune-Macrophage; Homo_714,totalSample,immune-CD8_T; Homo_714,totalSample,immune-Effector_memory; Homo_714,totalSample,immune-Cytotoxic; Homo_714,totalSample,immune-MAIT; Homo_714,totalSample,immune-Exhausted; Homo_714,totalSample,immune-Tfh; Homo_714,totalSample,immune-nTreg; Homo_633,totalSample,immune-InfiltrationScore; Homo_633,totalSample,immune-Monocyte; Homo_633,totalSample,immune-Macrophage; Homo_633,totalSample,immune-Effector_memory; Homo_633,totalSample,immune-CD8_T; Homo_633,totalSample,immune-Cytotoxic; Homo_633,totalSample,immune-nTreg; Homo_633,totalSample,immune-Neutrophil; Homo_633,totalSample,immune-MAIT; Homo_633,totalSample,immune-Bcell; Homo_633,totalSample,immune-CD4_naive; Homo_633,totalSample,immune-DC; Homo_633,totalSample,immune-Gamma_delta; Homo_633,totalSample,immune-NK; MCSA,totalSample,immune-DC; MCSA,totalSample,immune-Monocyte; MCSA,totalSample,immune-Neutrophil; MCSA,totalSample,immune-CD4_T; MCSA,totalSample,immune-CD8_T; MCSA,totalSample,immune-CD4_naive; MCSA,totalSample,immune-nTreg; MCSA,totalSample,immune-Th2; [illegible]
CDH4	Homo_723,female,AD.vs.control; Homo_723,male,AD.vs.control; Homo_723,female,AD.vs.MCI; Homo_723,male,AD.vs.MCI; Homo_714,female,AD.vs.control; Homo_714,male,AD.vs.control; Homo_714,male,AD.vs.MCI; Homo_633,female,AD.vs.control; Homo_633,female,AD.vs.MCI; MCSA,totalSample,age; MCSA,MCI,age; MCSA,totalSample,immune-DC; MCSA,totalSample,immune-Monocyte; MCSA,totalSample,immune-iTreg; MCSA,totalSample,immune-Th2
SH3BP4	Homo_723,female,AD.vs.control; Homo_723,male,AD.vs.control; Homo_723,female,AD.vs.MCI; Homo_723,male,AD.vs.MCI; Homo_723,female,MCI.vs.control; Homo_723,male,MCI.vs.control; Homo_714,female,AD.vs.control; Homo_714,male,AD.vs.control; Homo_714,female,AD.vs.MCI; Homo_714,male,AD.vs.MCI; Homo_714,female,MCI.vs.control; Homo_633,female,AD.vs.control; Homo_633,female,AD.vs.MCI; ROSMAP,female,AD.vs.control; ROSMAP,male,AD.vs.control; ROSMAP,female,AD.vs.MCI; ROSMAP,control,male.vs.female; Emory,totalSample,immune-InfiltrationScore; Homo_723,totalSample,immune-DC; Homo_723,totalSample,immune-InfiltrationScore; Homo_723,totalSample,immune-Monocyte; Homo_723,totalSample,immune-Neutrophil; Homo_723,totalSample,immune-CD4_naive; Homo_723,totalSample,immune-Th17; Homo_723,totalSample,immune-NK; Homo_714,totalSample,immune-InfiltrationScore; Homo_714,totalSample,immune-DC; Homo_714,totalSample,immune-NKT; Homo_714,totalSample,immune-Exhausted; Homo_633,totalSample,immune-InfiltrationScore; Homo_633,totalSample,immune-NKT; MCSA,totalSample,immune-Neutrophil; MCSA,totalSample,immune-CD4_T; MCSA,totalSample,immune-NKT; MCSA,totalSample,immune-CD4_naive; MCSA,totalSample,immune-Tr1; MCSA,totalSample,immune-nTreg; MCSA,totalSample,immune-iTreg; MCSA,totalSample,immune-Th17; MCSA,totalSample,immune-Tfh; MCSA,totalSample,immune-Exhausted; MCSA,totalSample,immune-Central_memory; MCSA,totalSample,immune-Effector_memory; MCSA,totalSample,immune-InfiltrationScore; SRP223445,totalSample,immune-Neutrophil; SRP223445,totalSample,immune-nTreg; SRP223445,totalSample,immune-InfiltrationScore; ROSMAP,female- [illegible]
AJAP1	Homo_723,female,AD.vs.control; Homo_723,male,AD.vs.control; Homo_723,female,AD.vs.MCI; Homo_723,male,AD.vs.MCI; Homo_714,female,AD.vs.control; Homo_714,male,AD.vs.control; Homo_714,female,AD.vs.MCI; Homo_714,male,AD.vs.MCI; Homo_633,female,AD.vs.control; Homo_633,female,AD.vs.MCI; Emory,MCI,age; Emory,totalSample,immune-CD8_T; Emory,totalSample,immune-Gamma_delta; Emory,totalSample,immune-Exhausted; Homo_714,totalSample,immune-InfiltrationScore; MCSA,totalSample,immune-CD4_T; MCSA,totalSample,immune-CD4_naive; MCSA,totalSample,immune-Tr1; MCSA,totalSample,immune-nTreg; MCSA,totalSample,immune-iTreg; MCSA,totalSample,immune-Th17; MCSA,totalSample,immune-Tfh; MCSA,totalSample,immune-Exhausted; ROSMAP,totalSample,immune-InfiltrationScore; SRP223445,totalSample,immune-InfiltrationScore; ROSMAP,male-includeAge90+,survival
GPM6B	Homo_723,female,AD.vs.control; Homo_723,male,AD.vs.control; Homo_723,female,AD.vs.MCI; Homo_723,male,AD.vs.MCI; Homo_723,male,MCI.vs.control; Homo_714,female,AD.vs.control; Homo_714,male,AD.vs.control; Homo_714,female,AD.vs.MCI; Homo_714,male,AD.vs.MCI; Homo_714,female,MCI.vs.control; Homo_714,male,MCI.vs.control; Homo_633,female,AD.vs.control; Homo_633,female,AD.vs.MCI; Homo_633,female,MCI.vs.control; Homo_633,male,MCI.vs.control; MCSA,control,age; MCSA,MCI,age; Emory,totalSample,immune-Macrophage; Emory,totalSample,immune-Neutrophil; Emory,totalSample,immune-CD4_T; Emory,totalSample,immune-CD8_T; Emory,totalSample,immune-Tr1; Emory,totalSample,immune-iTreg; Emory,totalSample,immune-Tfh; Emory,totalSample,immune-CD8_naive; Emory,totalSample,immune-Exhausted; Emory,totalSample,immune-InfiltrationScore; Homo_723,totalSample,immune-iTreg; Homo_723,totalSample,immune-Exhausted; Homo_723,totalSample,immune-Central_memory; Homo_723,totalSample,immune-Monocyte; Homo_723,totalSample,immune-Tfh; Homo_723,totalSample,immune-CD4_T; Homo_723,totalSample,immune-CD8_naive; Homo_723,totalSample,immune-CD8_T; Homo_723,totalSample,immune-CD4_naive; Homo_723,totalSample,immune-Macrophage; Homo_723,totalSample,immune-Tr1; Homo_723,totalSample,immune-Th2; Homo_723,totalSample,immune-nTreg; Homo_723,totalSample,immune-MAIT; Homo_714,totalSample,immune-Tfh; Homo_714,totalSample,immune-iTreg; Homo_714,totalSample,immune-Tr1; Homo_714,totalSample,immune-CD4_T; Homo_714,totalSample,immune-Monocyte; Homo_714,totalSample,immune-CD4_naive; Homo_714,totalSample,immune-Macrophage; Homo_714,totalSample,immune-Effector_memory; Homo_714,totalSample,immune-Central_memory; Homo_714,totalSample,immune-nTreg; Homo_714,totalSample,immune-NKT; Homo_714,totalSample,immune-Th2; Homo_633,totalSample,immune-NKT; Homo_633,totalSample,immune-CD4_naive; Homo_633,totalSample,immune-Tfh; Homo_633,totalSample,immune-iTreg; Homo_633,totalSample,immune-Th1; Homo_633,totalSample,immune-Monocyte; Homo_633,totalSample,immune-CD4_T; Homo_633,totalSample,immune-Tr1; Homo_633,totalSample,immune-InfiltrationScore; Homo_633,totalSample,immune-Central_memory; Homo_633,totalSample,immune-nTreg; Homo_633,totalSample,immune-Effector_memory; MCSA,totalSample,immune-DC; MCSA,totalSample,immune-Macrophage; MCSA,totalSample,immune-CD4_T; MCSA,totalSample,immune-Gamma_delta; MCSA,totalSample,immune-CD4_naive; MCSA,totalSample,immune-Tr1; MCSA,totalSample,immune-nTreg; MCSA,totalSample,immune-iTreg; MCSA,totalSample,immune-Th17; MCSA,totalSample,immune-Tfh; [illegible]

PRTFDC1	Homo_723,female,AD.vs.control; Homo_723,male,AD.vs.control; Homo_723,female,AD.vs.MCI; Homo_723,male,AD.vs.MCI; Homo_723,female,MCI.vs.control; Homo_723,male,MCI.vs.control; Homo_714,female,AD.vs.control; Homo_714,male,AD.vs.control; Homo_714,female,AD.vs.MCI; Homo_714,male,AD.vs.MCI; Homo_714,female,MCI.vs.control; Homo_714,male,MCI.vs.control; Homo_633,female,AD.vs.control; Homo_633,female,AD.vs.MCI; ROSMAP,MCI,sample.age; Homo_723,totalSample,immune-Exhausted; Homo_723,totalSample,immune-Th17; Homo_723,totalSample,immune-Cytotoxic; Homo_723,totalSample,immune-CD8_T; Homo_723,totalSample,immune-Monocyte; Homo_714,totalSample,immune-Monocyte; Homo_714,totalSample,immune-Exhausted; Homo_714,totalSample,immune-Th17; Homo_633,totalSample,immune-Monocyte; Homo_633,totalSample,immune-Th2; Homo_633,totalSample,immune-Tfh; Homo_633,totalSample,immune-Gamma_delta; Homo_633,totalSample,immune-Central_memory; Homo_633,totalSample,immune-Th17; Homo_633,totalSample,immune-CD4_T; Homo_633,totalSample,immune-Exhausted; MCSA,totalSample,immune-DC; MCSA,totalSample,immune-Monocyte; MCSA,totalSample,immune-CD4_T; MCSA,totalSample,immune-CD4_naive; MCSA,totalSample,immune-Tr1; MCSA,totalSample,immune-nTreg; MCSA,totalSample,immune-iTreg; MCSA,totalSample,immune-Tfh; MCSA,totalSample,immune-CD8_naive; MCSA,totalSample,immune-Cytotoxic; ROSMAP,totalSample,immune-InfiltrationScore; SRP223445,totalSample,immune-CD4_naive; SRP223445,totalSample,immune-Exhausted
OSER1	Homo_723,female,AD.vs.control; Homo_723,male,AD.vs.control; Homo_723,female,AD.vs.MCI; Homo_723,male,AD.vs.MCI; Homo_714,female,AD.vs.control; Homo_714,male,AD.vs.control; Homo_714,female,AD.vs.MCI; Homo_714,male,AD.vs.MCI; Homo_633,female,AD.vs.control; Homo_633,female,AD.vs.MCI; MCSA,totalSample,age; MCSA,control,age; MCSA,MCI,age; ROSMAP,MCI,last.visit.age; Emory,totalSample,immune-DC; Emory,totalSample,immune-Monocyte; Emory,totalSample,immune-Neutrophil; Emory,totalSample,immune-CD8_T; Emory,totalSample,immune-Gamma_delta; Emory,totalSample,immune-nTreg; Emory,totalSample,immune-Th1; Emory,totalSample,immune-Tfh; Emory,totalSample,immune-CD8_naive; Emory,totalSample,immune-Exhausted; Homo_723,totalSample,immune-Monocyte; Homo_723,totalSample,immune-DC; Homo_723,totalSample,immune-Neutrophil; Homo_723,totalSample,immune-Th2; Homo_723,totalSample,immune-Gamma_delta; Homo_723,totalSample,immune-Bcell; Homo_714,totalSample,immune-Monocyte; Homo_714,totalSample,immune-Th2; Homo_714,totalSample,immune-Gamma_delta; Homo_714,totalSample,immune-DC; Homo_633,totalSample,immune-Monocyte; Homo_633,totalSample,immune-Th2; Homo_633,totalSample,immune-Exhausted; Homo_633,totalSample,immune-Th17; Homo_633,totalSample,immune-Gamma_delta; MCSA,totalSample,immune-DC; MCSA,totalSample,immune-Monocyte; MCSA,totalSample,immune-Neutrophil; MCSA,totalSample,immune-CD8_T; MCSA,totalSample,immune-NKT; MCSA,totalSample,immune-CD4_naive; MCSA,totalSample,immune-nTreg; MCSA,totalSample,immune-Th2; MCSA,totalSample,immune-Tfh; MCSA,totalSample,immune-Cytotoxic; MCSA,totalSample,immune-Exhausted; MCSA,totalSample,immune-Central_memory; MCSA,totalSample,immune-Effector_memory; MCSA,totalSample,immune-InfiltrationScore; SRP223445,totalSample,immune-CD4_naive; SRP223445,totalSample,immune-Exhausted
MS4A4A	Homo_723,female,AD.vs.control; Homo_723,male,AD.vs.control; Homo_723,female,AD.vs.MCI; Homo_723,male,AD.vs.MCI; Homo_714,female,AD.vs.control; Homo_714,male,AD.vs.control; Homo_714,female,AD.vs.MCI; Homo_714,male,AD.vs.MCI; Homo_633,female,AD.vs.control; Homo_633,female,AD.vs.MCI; MCSA,totalSample,age; MCSA,control,age; MCSA,MCI,age; ROSMAP,MCI,last.visit.age; Emory,totalSample,immune-Monocyte; Emory,totalSample,immune-NK; Emory,totalSample,immune-Gamma_delta; Emory,totalSample,immune-InfiltrationScore; Homo_723,totalSample,immune-Monocyte; Homo_723,totalSample,immune-InfiltrationScore; Homo_723,totalSample,immune-Gamma_delta; Homo_714,totalSample,immune-Monocyte; Homo_633,totalSample,immune-Monocyte; Homo_633,totalSample,immune-Exhausted; Homo_633,totalSample,immune-Macrophage; Homo_633,totalSample,immune-NK; MCSA,totalSample,immune-Monocyte; MCSA,totalSample,immune-NK; MCSA,totalSample,immune-CD4_T; MCSA,totalSample,immune-CD8_T; MCSA,totalSample,immune-CD4_naive; MCSA,totalSample,immune-nTreg; MCSA,totalSample,immune-Tfh; MCSA,totalSample,immune-Cytotoxic; MCSA,totalSample,immune-Central_memory; MCSA,totalSample,immune-Effector_memory; ROSMAP,totalSample,immune-Effector_memory
SRGAP3	Homo_723,female,AD.vs.control; Homo_723,female,AD.vs.MCI; Homo_633,female,AD.vs.control; Homo_633,female,AD.vs.MCI; Emory,MCI,age; MCSA,totalSample,age; MCSA,control,age; ROSMAP,MCI,sample.age; Homo_723,totalSample,immune-nTreg; Homo_723,totalSample,immune-Monocyte; Homo_723,totalSample,immune-iTreg; Homo_723,totalSample,immune-CD4_T; Homo_714,totalSample,immune-InfiltrationScore; Homo_714,totalSample,immune-nTreg; Homo_714,totalSample,immune-iTreg; Homo_633,totalSample,immune-iTreg; Homo_633,totalSample,immune-nTreg; Homo_633,totalSample,immune-Monocyte; Homo_633,totalSample,immune-InfiltrationScore; Homo_633,totalSample,immune-Tr1; MCSA,totalSample,immune-Monocyte; MCSA,totalSample,immune-CD4_T; MCSA,totalSample,immune-Gamma_delta; MCSA,totalSample,immune-CD4_naive; MCSA,totalSample,immune-Tr1; MCSA,totalSample,immune-nTreg; MCSA,totalSample,immune-Th17; MCSA,totalSample,immune-Tfh; ROSMAP,totalSample,immune- [illegible]
HIP1R	Homo_723,female,AD.vs.control; Homo_723,male,AD.vs.control; Homo_723,female,AD.vs.MCI; Homo_723,female,MCI.vs.control; Homo_723,male,MCI.vs.control; Homo_723,control,male.vs.female; Homo_723,MCI,male.vs.female; Homo_714,female,AD.vs.control; Homo_714,male,AD.vs.control; Homo_714,female,AD.vs.MCI; Homo_714,female,MCI.vs.control; Homo_714,male,MCI.vs.control; Homo_714,control,male.vs.female; Homo_714,MCI,male.vs.female; Homo_633,female,AD.vs.control; Homo_633,female,AD.vs.MCI; Homo_633,female,MCI.vs.control; Homo_633,male,MCI.vs.control; Homo_633,control,male.vs.female; ROSMAP,female,AD.vs.control; ROSMAP,male,AD.vs.control; ROSMAP,male,AD.vs.MCI; ROSMAP,AD,male.vs.female; ACOM,AD,age; ACOM,totalSample,age; Emory,MCI,age; MCSA,totalSample,age; MCSA,control,age; MCSA,MCI,age; Emory,totalSample,immune-B_cell; Emory,totalSample,immune-Monocyte; Emory,totalSample,immune-Macrophage; Emory,totalSample,immune-Neutrophil; Emory,totalSample,immune-CD4_T; Emory,totalSample,immune-CD8_T; Emory,totalSample,immune-Tr1; Emory,totalSample,immune-nTreg; Emory,totalSample,immune-iTreg; Emory,totalSample,immune-Tfh; Emory,totalSample,immune-CD8_naive; Emory,totalSample,immune-Exhausted; Emory,totalSample,immune-Central_memory; Emory,totalSample,immune-InfiltrationScore; Homo_723,totalSample,immune-Monocyte; Homo_723,totalSample,immune-Central_memory; Homo_723,totalSample,immune-DC; Homo_723,totalSample,immune-nTreg; Homo_723,totalSample,immune-CD4_naive; Homo_723,totalSample,immune-Neutrophil; Homo_723,totalSample,immune-CD8_T; Homo_723,totalSample,immune-Tfh; Homo_723,totalSample,immune-CD4_T; Homo_723,totalSample,immune-Exhausted; Homo_714,totalSample,immune-Monocyte; Homo_714,totalSample,immune-nTreg; Homo_714,totalSample,immune-DC; Homo_714,totalSample,immune-CD4_naive; Homo_714,totalSample,immune-Neutrophil; Homo_714,totalSample,immune-Th1; Homo_633,totalSample,immune-Monocyte; Homo_633,totalSample,immune-nTreg; Homo_633,totalSample,immune-Tfh; Homo_633,totalSample,immune-CD4_naive; Homo_633,totalSample,immune-iTreg; Homo_633,totalSample,immune-Central_memory; MCSA,totalSample,immune-DC; MCSA,totalSample,immune-Monocyte; MCSA,totalSample,immune-Neutrophil; MCSA,totalSample,immune-CD4_T; MCSA,totalSample,immune-CD8_T; MCSA,totalSample,immune-NKT; MCSA,totalSample,immune-CD4_naive; MCSA,totalSample,immune-Tr1; MCSA,totalSample,immune-nTreg; MCSA,totalSample,immune-Th2; MCSA,totalSample,immune-Tfh; MCSA,totalSample,immune-CD8_naive; MCSA,totalSample,immune-Cytotoxic; MCSA,totalSample,immune-Central_memory; MCSA,totalSample,immune- [illegible]
SV2A	Homo_723,female,AD.vs.control; Homo_723,female,AD.vs.MCI; Homo_723,male,MCI.vs.control; Homo_714,female,AD.vs.MCI; Homo_714,female,MCI.vs.control; Homo_714,male,MCI.vs.control; Homo_633,female,AD.vs.control; Homo_633,female,AD.vs.MCI; Homo_633,female,MCI.vs.control; Homo_633,male,MCI.vs.control; MCSA,totalSample,age; MCSA,control,age; MCSA,MCI,age; Emory,totalSample,immune-Neutrophil; Emory,totalSample,immune-CD4_T; Emory,totalSample,immune-CD8_T; Emory,totalSample,immune-Tr1; Emory,totalSample,immune-iTreg; Emory,totalSample,immune-Tfh; Emory,totalSample,immune-MAIT; Emory,totalSample,immune-InfiltrationScore; Homo_723,totalSample,immune-Monocyte; Homo_723,totalSample,immune-Exhausted; Homo_723,totalSample,immune-Central_memory; Homo_723,totalSample,immune-DC; Homo_723,totalSample,immune-CD8_T; Homo_723,totalSample,immune-nTreg; Homo_723,totalSample,immune-CD4_naive; Homo_723,totalSample,immune-CD4_T; Homo_714,totalSample,immune-Monocyte; Homo_714,totalSample,immune-nTreg; Homo_633,totalSample,immune-Monocyte; Homo_633,totalSample,immune-InfiltrationScore; Homo_633,totalSample,immune-nTreg; MCSA,totalSample,immune-Monocyte; MCSA,totalSample,immune-Neutrophil; MCSA,totalSample,immune-CD4_T; MCSA,totalSample,immune-CD8_T; MCSA,totalSample,immune-CD4_naive; MCSA,totalSample,immune-Tr1; MCSA,totalSample,immune-nTreg; MCSA,totalSample,immune-Th17; MCSA,totalSample,immune-Tfh; MCSA,totalSample,immune-Central_memory; MCSA,totalSample,immune-Effector_memory; MCSA,totalSample,immune-InfiltrationScore; ROSMAP,male-includeAge90+,survival
C12orf76	Homo_723,female,AD.vs.control; Homo_723,male,AD.vs.control; Homo_723,female,AD.vs.MCI; Homo_723,male,AD.vs.MCI; Homo_723,female,MCI.vs.control; Homo_714,female,AD.vs.control; Homo_714,male,AD.vs.control; Homo_714,female,AD.vs.MCI; Homo_714,male,AD.vs.MCI; Homo_714,female,MCI.vs.control; Homo_714,male,MCI.vs.control; Homo_633,female,AD.vs.control; Homo_633,female,AD.vs.MCI; ACOM,AD,age; Emory,MCI,age; MCSA,MCI,age; Emory,totalSample,immune-Monocyte; Emory,totalSample,immune-CD4_T; Emory,totalSample,immune-Tr1; Emory,totalSample,immune-iTreg; Homo_723,totalSample,immune-Th17; Homo_723,totalSample,immune-Exhausted; Homo_723,totalSample,immune-Th2; Homo_723,totalSample,immune-CD4_T; Homo_723,totalSample,immune-Monocyte; Homo_723,totalSample,immune-CD4_naive; Homo_723,totalSample,immune-Cytotoxic; Homo_723,totalSample,immune-Tfh; Homo_723,totalSample,immune-Tr1; Homo_723,totalSample,immune-Central_memory; Homo_723,totalSample,immune-nTreg; Homo_723,totalSample,immune-Gamma_delta; Homo_723,totalSample,immune-CD8_T; Homo_714,totalSample,immune-Th17; Homo_714,totalSample,immune-Monocyte; Homo_714,totalSample,immune-Central_memory; Homo_714,totalSample,immune-Gamma_delta; Homo_714,totalSample,immune-Exhausted; Homo_714,totalSample,immune-CD4_T; Homo_714,totalSample,immune-Th2; Homo_714,totalSample,immune-Tfh; Homo_714,totalSample,immune-CD4_naive; Homo_633,totalSample,immune-Th2; Homo_633,totalSample,immune-Tfh; Homo_633,totalSample,immune-CD4_T; Homo_633,totalSample,immune-Monocyte; Homo_633,totalSample,immune-Central_memory; Homo_633,totalSample,immune-Th17; Homo_633,totalSample,immune-Gamma_delta; Homo_633,totalSample,immune-Effector_memory; Homo_633,totalSample,immune-Tr1; Homo_633,totalSample,immune-CD4_naive; Homo_633,totalSample,immune-Exhausted; Homo_633,totalSample,immune-InfiltrationScore; Homo_633,totalSample,immune-nTreg; MCSA,totalSample,immune-DC; MCSA,totalSample,immune-Bcell; MCSA,totalSample,immune-Macrophage; MCSA,totalSample,immune-CD4_T; MCSA,totalSample,immune-CD4_naive; MCSA,totalSample,immune-Tr1; MCSA,totalSample,immune-nTreg; MCSA,totalSample,immune-iTreg; MCSA,totalSample,immune-Th17; MCSA,totalSample,immune-Tfh; MCSA,totalSample,immune-CD8_naive; MCSA,totalSample,immune-Cytotoxic; MCSA,totalSample,immune-Exhausted; MCSA,totalSample,immune-MAIT; MCSA,totalSample,immune-Central_memory; ROSMAP,totalSample,immune-Effector_memory; SRP223445,totalSample,immune-CD4_naive; SRP223445,totalSample,immune-Exhausted
DLG5	Homo_723,female,AD.vs.control; Homo_723,male,AD.vs.control; Homo_723,female,AD.vs.MCI; Homo_723,male,AD.vs.MCI; Homo_723,female,MCI.vs.control; Homo_714,female,AD.vs.control; Homo_714,male,AD.vs.control; Homo_714,female,AD.vs.MCI; Homo_714,male,AD.vs.MCI; Homo_633,female,AD.vs.control; Homo_633,female,AD.vs.MCI; ROSMAP,female,AD.vs.control; ROSMAP,male,AD.vs.control; ROSMAP,female,MCI.vs.control; Emory,totalSample,immune-NK; Emory,totalSample,immune-Neutrophil; Emory,totalSample,immune-Tr1; Emory,totalSample,immune-Cytotoxic; Emory,totalSample,immune-Effector_memory; Emory,totalSample,immune-InfiltrationScore; Homo_723,totalSample,immune-NK; Homo_723,totalSample,immune-DC; Homo_723,totalSample,immune-InfiltrationScore; Homo_723,totalSample,immune-Monocyte; Homo_723,totalSample,immune-Neutrophil; Homo_723,totalSample,immune-Gamma_delta; Homo_714,totalSample,immune-InfiltrationScore; Homo_714,totalSample,immune-NK; Homo_714,totalSample,immune-Th2; Homo_714,totalSample,immune-Gamma_delta; Homo_714,totalSample,immune-Neutrophil; Homo_633,totalSample,immune-InfiltrationScore; Homo_633,totalSample,immune-NK; Homo_633,totalSample,immune-Gamma_delta; Homo_633,totalSample,immune-Bcell; Homo_633,totalSample,immune-Macrophage; Homo_633,totalSample,immune-Monocyte; Homo_633,totalSample,immune-Cytotoxic; MCSA,totalSample,immune-Gamma_delta; MCSA,totalSample,immune-Th17; MCSA,totalSample,immune-Effector_memory; MCSA,totalSample,immune-InfiltrationScore; ROSMAP,totalSample,immune-InfiltrationScore; SRP223445,totalSample,immune-NK; SRP223445,totalSample,immune-CD4_naive;
ELOVL7	Homo_723,female,AD.vs.control; Homo_723,male,AD.vs.control; Homo_723,female,AD.vs.MCI; Homo_723,male,AD.vs.MCI; Homo_723,female,MCI.vs.control; Homo_723,male,MCI.vs.control; Homo_714,female,AD.vs.control; Homo_714,male,AD.vs.control; Homo_714,female,AD.vs.MCI; Homo_714,male,AD.vs.MCI; Homo_714,female,MCI.vs.control; Homo_714,male,MCI.vs.control; Homo_633,female,AD.vs.control; Homo_633,female,AD.vs.MCI; ROSMAP,female,AD.vs.control; ROSMAP,male,AD.vs.control; SRP223445,female,AD.vs.control; Emory,totalSample,immune-Th1; Emory,totalSample,immune-Exhausted; Homo_723,totalSample,immune-Th17; Homo_723,totalSample,immune-Exhausted; Homo_723,totalSample,immune-CD8_T; Homo_723,totalSample,immune-Cytotoxic; Homo_723,totalSample,immune-Gamma_delta; Homo_723,totalSample,immune-CD4_naive; Homo_723,totalSample,immune-Monocyte; Homo_714,totalSample,immune-Th17; Homo_714,totalSample,immune-Exhausted; Homo_714,totalSample,immune-CD8_T; Homo_714,totalSample,immune-Monocyte; Homo_714,totalSample,immune-Gamma_delta; Homo_714,totalSample,immune-Cytotoxic; Homo_633,totalSample,immune-Th2; Homo_633,totalSample,immune-Gamma_delta; Homo_633,totalSample,immune-Tfh; Homo_633,totalSample,immune-Th17; Homo_633,totalSample,immune-Monocyte; Homo_633,totalSample,immune-Exhausted; Homo_633,totalSample,immune-CD4_T; Homo_633,totalSample,immune-NKT; Homo_633,totalSample,immune-Central_memory; Homo_633,totalSample,immune-InfiltrationScore; MCSA,totalSample,immune-DC; MCSA,totalSample,immune-CD4_T; MCSA,totalSample,immune-CD8_T; MCSA,totalSample,immune-Gamma_delta; MCSA,totalSample,immune-CD4_naive; MCSA,totalSample,immune-Tr1; MCSA,totalSample,immune-nTreg; MCSA,totalSample,immune-iTreg; MCSA,totalSample,immune-Th17; MCSA,totalSample,immune-Cytotoxic; MCSA,totalSample,immune-Exhausted; SRP223445,totalSample,immune-CD4_naive; SRP223445,totalSample,immune-Exhausted
SRSF7	Homo_723,female,AD.vs.control; Homo_723,male,AD.vs.control; Homo_723,female,AD.vs.MCI; Homo_723,male,AD.vs.MCI; Homo_723,female,MCI.vs.control; Homo_714,female,AD.vs.control; Homo_714,male,AD.vs.control; Homo_714,female,AD.vs.MCI; Homo_714,male,AD.vs.MCI; Homo_714,female,MCI.vs.control; Homo_633,female,AD.vs.control; Homo_633,female,AD.vs.MCI; ROSMAP,female,AD.vs.control; ROSMAP,male,AD.vs.control; ROSMAP,female,MCI.vs.control; MCSA,MCI,age; Emory,totalSample,immune-Macrophage; Emory,totalSample,immune-NK; Emory,totalSample,immune-Neutrophil; Emory,totalSample,immune-CD4_T; Emory,totalSample,immune-CD8_T; Emory,totalSample,immune-Tr1; Emory,totalSample,immune-iTreg; Emory,totalSample,immune-Tfh; Emory,totalSample,immune-Exhausted; Emory,totalSample,immune-Central_memory; Emory,totalSample,immune-Effector_memory; Emory,totalSample,immune-InfiltrationScore; Homo_723,totalSample,immune-Th1; Homo_723,totalSample,immune-CD4_naive; Homo_723,totalSample,immune-Central_memory; Homo_723,totalSample,immune-Tr1; Homo_723,totalSample,immune-CD4_T; Homo_723,totalSample,immune-iTreg; Homo_723,totalSample,immune-Monocyte; Homo_723,totalSample,immune-Tfh; Homo_723,totalSample,immune-NKT; Homo_723,totalSample,immune-Neutrophil; Homo_723,totalSample,immune-nTreg; Homo_714,totalSample,immune-Th1; Homo_714,totalSample,immune-CD4_naive; Homo_714,totalSample,immune-NKT; Homo_714,totalSample,immune-iTreg; Homo_714,totalSample,immune-CD4_T; Homo_714,totalSample,immune-Tr1; Homo_633,totalSample,immune-Th1; Homo_633,totalSample,immune-iTreg; Homo_633,totalSample,immune-NKT; Homo_633,totalSample,immune-CD4_naive; Homo_633,totalSample,immune-Tfh; Homo_633,totalSample,immune-CD4_T; Homo_633,totalSample,immune-Tr1; Homo_633,totalSample,immune-Monocyte; Homo_633,totalSample,immune-Central_memory; MCSA,totalSample,immune-DC; MCSA,totalSample,immune-Bcell; MCSA,totalSample,immune-Macrophage; MCSA,totalSample,immune-Neutrophil; MCSA,totalSample,immune-CD4_T; MCSA,totalSample,immune-CD4_naive; MCSA,totalSample,immune-Tr1; MCSA,totalSample,immune-nTreg; MCSA,totalSample,immune-iTreg; MCSA,totalSample,immune-Th17; MCSA,totalSample,immune-Tfh; MCSA,totalSample,immune-CD8_naive; MCSA,totalSample,immune-Cytotoxic; MCSA,totalSample,immune-Exhausted; MCSA,totalSample,immune-MAIT; MCSA,totalSample,immune-Central_memory; SRP223445,totalSample,immune-CD4_naive; SRP223445,totalSample,immune-Th17; SRP223445,totalSample,immune-Exhausted; SRP223445,totalSample,immune-InfiltrationScore
CBX7	Homo_723,female,AD.vs.control; Homo_723,male,AD.vs.control; Homo_723,female,AD.vs.MCI; Homo_723,male,AD.vs.MCI; Homo_714,female,AD.vs.control; Homo_714,male,AD.vs.control; Homo_714,female,AD.vs.MCI; Homo_714,male,AD.vs.MCI; Homo_633,female,AD.vs.control; Homo_633,female,AD.vs.MCI; ROSMAP,female,AD.vs.control; ROSMAP,control,male.vs.female; Emory,MCI,age; MCSA,totalSample,age; MCSA,control,age; Emory,totalSample,immune-Macrophage; Emory,totalSample,immune-CD4_T; Emory,totalSample,immune-Gamma_delta; Emory,totalSample,immune-CD4_naive; Emory,totalSample,immune-Tr1; Emory,totalSample,immune-iTreg; Emory,totalSample,immune-Th17; Emory,totalSample,immune-Cytotoxic; Emory,totalSample,immune-Central_memory; Emory,totalSample,immune-InfiltrationScore; Homo_723,totalSample,immune-Tfh; Homo_723,totalSample,immune-InfiltrationScore; Homo_723,totalSample,immune-iTreg; Homo_723,totalSample,immune-Th2; Homo_723,totalSample,immune-Effector_memory; Homo_723,totalSample,immune-nTreg; Homo_723,totalSample,immune-CD4_T; Homo_723,totalSample,immune-CD4_naive; Homo_723,totalSample,immune-Tr1; Homo_723,totalSample,immune-Central_memory; Homo_723,totalSample,immune-Macrophage; Homo_723,totalSample,immune-CD8_T; Homo_723,totalSample,immune-Bcell; Homo_723,totalSample,immune-Gamma_delta; Homo_714,totalSample,immune-Tfh; Homo_714,totalSample,immune-Th2; Homo_714,totalSample,immune-nTreg; Homo_714,totalSample,immune-Effector_memory; Homo_714,totalSample,immune-InfiltrationScore; Homo_714,totalSample,immune-Central_memory; Homo_714,totalSample,immune-CD4_T; Homo_714,totalSample,immune-Tr1; Homo_714,totalSample,immune-iTreg; Homo_714,totalSample,immune-Macrophage; Homo_714,totalSample,immune-CD4_naive; Homo_714,totalSample,immune-CD8_T; Homo_714,totalSample,immune-Gamma_delta; Homo_633,totalSample,immune-nTreg; Homo_633,totalSample,immune-InfiltrationScore; Homo_633,totalSample,immune-Tfh; Homo_633,totalSample,immune-Effector_memory; Homo_633,totalSample,immune-Central_memory; Homo_633,totalSample,immune-CD4_T; Homo_633,totalSample,immune-Th2; Homo_633,totalSample,immune-Tr1; Homo_633,totalSample,immune-CD4_naive; Homo_633,totalSample,immune-iTreg; Homo_633,totalSample,immune-Exhausted; Homo_633,totalSample,immune-Th17; Homo_633,totalSample,immune-Gamma_delta; MCSA,totalSample,immune-DC; MCSA,totalSample,immune-Monocyte; MCSA,totalSample,immune-CD4_T; MCSA,totalSample,immune-CD8_T; MCSA,totalSample,immune-NKT; MCSA,totalSample,immune-Gamma_delta; MCSA,totalSample,immune-CD4_naive; MCSA,totalSample,immune-Tr1; MCSA,totalSample,immune-nTreg; MCSA,totalSample,immune-iTreg; MCSA,totalSample,immune-Th2; MCSA,totalSample,immune-Th17; MCSA,totalSample,immune-Tfh; MCSA,totalSample,immune-Central_memory; MCSA,totalSample,immune-Effector_memory; MCSA,totalSample,immune-InfiltrationScore; [illegible]
ZNF185	Homo_723,female,AD.vs.control; Homo_723,male,AD.vs.control; Homo_723,female,AD.vs.MCI; Homo_723,male,AD.vs.MCI; Homo_714,female,AD.vs.control; Homo_714,male,AD.vs.control; Homo_714,female,AD.vs.MCI; Homo_714,male,AD.vs.MCI; Homo_633,female,AD.vs.control; Homo_633,female,AD.vs.MCI; MCSA,control,age; Emory,totalSample,immune-Neutrophil; Emory,totalSample,immune-CD8_T; Emory,totalSample,immune-Gamma_delta; Emory,totalSample,immune-Th1; Emory,totalSample,immune-Th17; Emory,totalSample,immune-Tfh; Emory,totalSample,immune-CD8_naive; Emory,totalSample,immune-Cytotoxic; Emory,totalSample,immune-Exhausted; Homo_723,totalSample,immune-Gamma_delta; Homo_723,totalSample,immune-Th17; Homo_723,totalSample,immune-CD8_T; Homo_723,totalSample,immune-Exhausted; Homo_723,totalSample,immune-Macrophage; Homo_723,totalSample,immune-Cytotoxic; Homo_723,totalSample,immune-InfiltrationScore; Homo_714,totalSample,immune-Exhausted; Homo_714,totalSample,immune-CD8_T; Homo_714,totalSample,immune-Gamma_delta; Homo_714,totalSample,immune-Cytotoxic; Homo_714,totalSample,immune-Th17; Homo_714,totalSample,immune-MAIT; Homo_633,totalSample,immune-Gamma_delta; Homo_633,totalSample,immune-Exhausted; Homo_633,totalSample,immune-InfiltrationScore; Homo_633,totalSample,immune-CD8_T; MCSA,totalSample,immune-Neutrophil; MCSA,totalSample,immune-CD8_T; MCSA,totalSample,immune-Gamma_delta; MCSA,totalSample,immune-Th17; MCSA,totalSample,immune-Cytotoxic; MCSA,totalSample,immune-Exhausted; SRP223445,totalSample,immune-CD4_naive; SRP223445,totalSample,immune-Exhausted
RDH10	Homo_723,female,AD.vs.control; Homo_723,male,AD.vs.control; Homo_723,female,AD.vs.MCI; Homo_723,male,AD.vs.MCI; Homo_714,female,AD.vs.MCI; Homo_633,female,AD.vs.control; Homo_633,female,AD.vs.MCI; ROSMAP,female,AD.vs.control; ROSMAP,control,male.vs.female; MCSA,MCI,age; Emory,totalSample,immune-Tr1; Emory,totalSample,immune-iTreg; Emory,totalSample,immune-Effector_memory; Emory,totalSample,immune-InfiltrationScore; Homo_723,totalSample,immune-Th2; Homo_723,totalSample,immune-Tfh; Homo_723,totalSample,immune-InfiltrationScore; Homo_723,totalSample,immune-Exhausted; Homo_723,totalSample,immune-CD4_T; Homo_723,totalSample,immune-CD4_naive; Homo_723,totalSample,immune-Tr1; Homo_723,totalSample,immune-Effector_memory; Homo_723,totalSample,immune-iTreg; Homo_723,totalSample,immune-NKT; Homo_723,totalSample,immune-Monocyte; Homo_714,totalSample,immune-InfiltrationScore; Homo_714,totalSample,immune-Tfh; Homo_714,totalSample,immune-Monocyte; Homo_714,totalSample,immune-Th2; Homo_714,totalSample,immune-CD4_naive; Homo_714,totalSample,immune-CD4_T; Homo_714,totalSample,immune-Effector_memory; Homo_714,totalSample,immune-NKT; Homo_714,totalSample,immune-Tr1; Homo_714,totalSample,immune-Central_memory; Homo_714,totalSample,immune-Bcell; Homo_633,totalSample,immune-Tfh; Homo_633,totalSample,immune-InfiltrationScore; Homo_633,totalSample,immune-Effector_memory; Homo_633,totalSample,immune-Th2; Homo_633,totalSample,immune-CD4_T; Homo_633,totalSample,immune-CD4_naive; Homo_633,totalSample,immune-Monocyte; Homo_633,totalSample,immune-Central_memory; Homo_633,totalSample,immune-Bcell; Homo_633,totalSample,immune-NKT; Homo_633,totalSample,immune-Tr1; Homo_633,totalSample,immune-Exhausted; Homo_633,totalSample,immune-DC; MCSA,totalSample,immune-DC; MCSA,totalSample,immune-Monocyte; MCSA,totalSample,immune-Macrophage; MCSA,totalSample,immune-CD4_T; MCSA,totalSample,immune-CD8_T; MCSA,totalSample,immune-CD4_naive; MCSA,totalSample,immune-Tr1; MCSA,totalSample,immune-nTreg; MCSA,totalSample,immune-iTreg; MCSA,totalSample,immune-Th17; MCSA,totalSample,immune-Tfh; MCSA,totalSample,immune-CD8_naive; MCSA,totalSample,immune-Cytotoxic; MCSA,totalSample,immune-Exhausted; MCSA,totalSample,immune-MAIT; [illegible]
PLXNB1	Homo_723,female,AD.vs.control; Homo_723,female,AD.vs.MCI; Homo_723,female,MCI.vs.control; Homo_633,female,AD.vs.control; Homo_633,female,AD.vs.MCI; ROSMAP,female,AD.vs.control; ROSMAP,female,MCI.vs.control; MCSA,totalSample,age; MCSA,control,age; MCSA,MCI,age; ROSMAP,MCI,last.visit.age; Homo_723,totalSample,immune-DC; Homo_723,totalSample,immune-InfiltrationScore; Homo_723,totalSample,immune-Neutrophil; Homo_714,totalSample,immune-InfiltrationScore; Homo_714,totalSample,immune-DC; Homo_633,totalSample,immune-InfiltrationScore; Homo_633,totalSample,immune-DC; MCSA,totalSample,immune-DC; MCSA,totalSample,immune-Monocyte; MCSA,totalSample,immune-CD8_T; MCSA,totalSample,immune-Th2; MCSA,totalSample,immune-Cytotoxic; MCSA,totalSample,immune-Effector_memory; MCSA,totalSample,immune-InfiltrationScore
SIK1B	Homo_723,female,AD.vs.control; Homo_723,male,AD.vs.control; Homo_723,female,AD.vs.MCI; Homo_723,male,AD.vs.MCI; Homo_723,female,MCI.vs.control; Homo_714,female,AD.vs.control; Homo_714,male,AD.vs.control; Homo_714,female,AD.vs.MCI; Homo_714,male,AD.vs.MCI; Homo_633,female,AD.vs.control; Homo_633,female,AD.vs.MCI; Emory,totalSample,immune-NK; Emory,totalSample,immune-Gamma_delta; Emory,totalSample,immune-InfiltrationScore; Homo_723,totalSample,immune-DC; Homo_723,totalSample,immune-NK; Homo_723,totalSample,immune-InfiltrationScore; Homo_723,totalSample,immune-Neutrophil; Homo_723,totalSample,immune-Monocyte; Homo_714,totalSample,immune-InfiltrationScore; Homo_714,totalSample,immune-NK; Homo_714,totalSample,immune-DC; Homo_714,totalSample,immune-NKT; Homo_633,totalSample,immune-InfiltrationScore; Homo_633,totalSample,immune-NK; Homo_633,totalSample,immune-DC; MCSA,totalSample,immune-Tr1; MCSA,totalSample,immune-InfiltrationScore; SRP223445,totalSample,immune-

EGF	Homo_723,female,AD.vs.control; Homo_723,male,AD.vs.control; Homo_723,female,AD.vs.MCI; Homo_723,male,AD.vs.MCI; Homo_723,female,MCI.vs.control; Homo_723,male,MCI.vs.control; Homo_714,female,AD.vs.control; Homo_714,male,AD.vs.control; Homo_714,female,AD.vs.MCI; Homo_714,male,AD.vs.MCI; Homo_714,female,MCI.vs.control; Homo_714,male,MCI.vs.control; Homo_633,female,AD.vs.control; Homo_633,female,AD.vs.MCI; Homo_633,female,MCI.vs.control; Emory,totalSample,immune-CD8_T; Emory,totalSample,immune-Th1; Emory,totalSample,immune-Tfh; Homo_723,totalSample,immune-Exhausted; Homo_723,totalSample,immune-Th17; Homo_723,totalSample,immune-CD8_T; Homo_723,totalSample,immune-Cytotoxic; Homo_723,totalSample,immune-CD4_naive; Homo_723,totalSample,immune-Gamma_delta; Homo_723,totalSample,immune-MAIT; Homo_714,totalSample,immune-Th17; Homo_714,totalSample,immune-Exhausted; Homo_714,totalSample,immune-CD8_T; Homo_714,totalSample,immune-Gamma_delta; Homo_714,totalSample,immune-Monocyte; Homo_714,totalSample,immune-Cytotoxic; Homo_633,totalSample,immune-Th2; Homo_633,totalSample,immune-Gamma_delta; Homo_633,totalSample,immune-Tfh; Homo_633,totalSample,immune-Th17; Homo_633,totalSample,immune-Exhausted; Homo_633,totalSample,immune-Monocyte; Homo_633,totalSample,immune-CD4_T; Homo_633,totalSample,immune-Central_memory; Homo_633,totalSample,immune-InfiltrationScore; MCSA,totalSample,immune-CD4_T; MCSA,totalSample,immune-Gamma_delta; MCSA,totalSample,immune-CD4_naive; MCSA,totalSample,immune-Tr1; MCSA,totalSample,immune-nTreg; MCSA,totalSample,immune-iTreg; MCSA,totalSample,immune-Th17; MCSA,totalSample,immune-Tfh; MCSA,totalSample,immune-Cytotoxic; MCSA,totalSample,immune-Exhausted; MCSA,totalSample,immune-Central_memory; ROSMAP,totalSample,immune-InfiltrationScore; SRP223445,totalSample,immune-CD4_naive; SRP223445,totalSample,immune-Exhausted; ROSMAP,female-excludeAge90+,survival; ROSMAP,female- [illegible]
COL9A1	Homo_723,female,AD.vs.control; Homo_723,female,AD.vs.MCI; Homo_723,AD,male.vs.female; ACOM,totalSample,age; Homo_723,totalSample,immune-Th2; Homo_714,totalSample,immune-Th2; Homo_633,totalSample,immune-Th2; MCSA,totalSample,immune-Bcell; MCSA,totalSample,immune-Macrophage; MCSA,totalSample,immune-CD4_T; MCSA,totalSample,immune-CD4_naive; MCSA,totalSample,immune-Tr1; MCSA,totalSample,immune-nTreg; MCSA,totalSample,immune-iTreg; MCSA,totalSample,immune-Tfh; MCSA,totalSample,immune-CD8_naive; MCSA,totalSample,immune-Central_memory; ROSMAP,totalSample,immune-InfiltrationScore
PPBP	Homo_723,female,AD.vs.control; Homo_723,male,AD.vs.control; Homo_723,female,AD.vs.MCI; Homo_723,male,AD.vs.MCI; Homo_723,female,MCI.vs.control; Homo_723,male,MCI.vs.control; SRP223445,female,AD.vs.control; MCSA,totalSample,age; MCSA,control,age; ROSMAP,MCI,last.visit.age; ROSMAP,MCI,sample.age; Emory,totalSample,immune-Monocyte; Emory,totalSample,immune-InfiltrationScore; Homo_723,totalSample,immune-CD8_T; Homo_723,totalSample,immune-Macrophage; Homo_723,totalSample,immune-Exhausted; Homo_723,totalSample,immune-Th2; Homo_723,totalSample,immune-iTreg; Homo_723,totalSample,immune-CD8_naive; Homo_723,totalSample,immune-Monocyte; Homo_723,totalSample,immune-Tfh; Homo_723,totalSample,immune-nTreg; Homo_723,totalSample,immune-CD4_T; Homo_714,totalSample,immune-Effector_memory; Homo_714,totalSample,immune-Monocyte; Homo_714,totalSample,immune-nTreg; Homo_633,totalSample,immune-Monocyte; Homo_633,totalSample,immune-Effector_memory; Homo_633,totalSample,immune-nTreg; MCSA,totalSample,immune-Monocyte; MCSA,totalSample,immune-CD4_T; MCSA,totalSample,immune-Gamma_delta; MCSA,totalSample,immune-CD4_naive; MCSA,totalSample,immune-Tr1; MCSA,totalSample,immune-nTreg; MCSA,totalSample,immune-Th2; MCSA,totalSample,immune-Th17; MCSA,totalSample,immune-Tfh; MCSA,totalSample,immune-Central_memory; MCSA,totalSample,immune-Effector_memory; SRP223445,totalSample,immune-CD4_naive; SRP223445,totalSample,immune-InfiltrationScore; ROSMAP,male-excludeAge90+,survival
MYH10	Homo_723,female,AD.vs.control; Homo_723,male,AD.vs.control; Homo_723,female,AD.vs.MCI; Homo_723,male,AD.vs.MCI; Homo_723,female,MCI.vs.control; Homo_723,male,MCI.vs.control; Homo_714,female,AD.vs.control; Homo_714,male,AD.vs.control; Homo_714,female,AD.vs.MCI; Homo_714,male,AD.vs.MCI; Homo_633,female,AD.vs.control; Homo_633,female,AD.vs.MCI; Homo_633,female,MCI.vs.control; Homo_633,male,MCI.vs.control; ROSMAP,male,AD.vs.control; SRP223445,male,AD.vs.control; Emory,MCI,age; MCSA,totalSample,age; MCSA,control,age; ROSMAP,AD,last.visit.age; ROSMAP,AD,MMSE; Emory,totalSample,immune-Neutrophil; Emory,totalSample,immune-CD8_T; Emory,totalSample,immune-Gamma_delta; Emory,totalSample,immune-Th17; Emory,totalSample,immune-CD8_naive; Emory,totalSample,immune-Cytotoxic; Emory,totalSample,immune-Exhausted; Homo_723,totalSample,immune-Monocyte; Homo_723,totalSample,immune-DC; Homo_723,totalSample,immune-Central_memory; Homo_723,totalSample,immune-Th17; Homo_723,totalSample,immune-nTreg; Homo_714,totalSample,immune-Exhausted; Homo_714,totalSample,immune-NKT; Homo_714,totalSample,immune-CD8_T; Homo_633,totalSample,immune-Monocyte; Homo_633,totalSample,immune-NKT; Homo_633,totalSample,immune-iTreg; Homo_633,totalSample,immune-nTreg; Homo_633,totalSample,immune-InfiltrationScore; Homo_633,totalSample,immune-Tr1; MCSA,totalSample,immune-Monocyte; MCSA,totalSample,immune-CD4_T; MCSA,totalSample,immune-Gamma_delta; MCSA,totalSample,immune-CD4_naive; MCSA,totalSample,immune-Tr1; MCSA,totalSample,immune-nTreg; MCSA,totalSample,immune-iTreg; MCSA,totalSample,immune-Th1; MCSA,totalSample,immune-Th17; MCSA,totalSample,immune-Tfh; MCSA,totalSample,immune-CD8_naive; MCSA,totalSample,immune-Exhausted; MCSA,totalSample,immune-Central_memory; SRP223445,totalSample,immune-Neutrophil; SRP223445,totalSample,immune-CD4_naive; SRP223445,totalSample,immune-Exhausted; SRP223445,totalSample,immune- [illegible]
ADGRA3	Homo_723,female,AD.vs.control; Homo_723,male,AD.vs.control; Homo_723,female,AD.vs.MCI; Homo_723,male,AD.vs.MCI; Homo_723,female,MCI.vs.control; Homo_714,female,AD.vs.control; Homo_714,male,AD.vs.control; Homo_714,female,AD.vs.MCI; Homo_714,male,AD.vs.MCI; Homo_714,female,MCI.vs.control; Homo_633,female,AD.vs.control; Homo_633,female,AD.vs.MCI; Homo_633,female,MCI.vs.control; Emory,totalSample,immune-CD4_T; Emory,totalSample,immune-Gamma_delta; Emory,totalSample,immune-Tr1; Emory,totalSample,immune-iTreg; Homo_723,totalSample,immune-Th2; Homo_723,totalSample,immune-CD4_T; Homo_723,totalSample,immune-CD4_naive; Homo_723,totalSample,immune-Tr1; Homo_723,totalSample,immune-iTreg; Homo_723,totalSample,immune-Exhausted; Homo_723,totalSample,immune-Th17; Homo_723,totalSample,immune-Central_memory; Homo_723,totalSample,immune-Tfh; Homo_723,totalSample,immune-nTreg; Homo_723,totalSample,immune-Cytotoxic; Homo_723,totalSample,immune-Th1; Homo_723,totalSample,immune-Gamma_delta; Homo_714,totalSample,immune-Tr1; Homo_714,totalSample,immune-CD4_naive; Homo_714,totalSample,immune-CD4_T; Homo_714,totalSample,immune-Th2; Homo_714,totalSample,immune-Tfh; Homo_714,totalSample,immune-Central_memory; Homo_714,totalSample,immune-Exhausted; Homo_714,totalSample,immune-iTreg; Homo_714,totalSample,immune-Gamma_delta; Homo_714,totalSample,immune-Th17; Homo_714,totalSample,immune-Cytotoxic; Homo_714,totalSample,immune-nTreg; Homo_714,totalSample,immune-Effector_memory; Homo_714,totalSample,immune-Th1; Homo_633,totalSample,immune-Tfh; Homo_633,totalSample,immune-CD4_T; Homo_633,totalSample,immune-Th2; Homo_633,totalSample,immune-Tr1; Homo_633,totalSample,immune-Central_memory; Homo_633,totalSample,immune-CD4_naive; Homo_633,totalSample,immune-Effector_memory; Homo_633,totalSample,immune-iTreg; Homo_633,totalSample,immune-Exhausted; Homo_633,totalSample,immune-Gamma_delta; Homo_633,totalSample,immune-nTreg; Homo_633,totalSample,immune-Th17; Homo_633,totalSample,immune-InfiltrationScore; Homo_633,totalSample,immune-Cytotoxic; MCSA,totalSample,immune-DC; MCSA,totalSample,immune-CD4_T; MCSA,totalSample,immune-Gamma_delta; MCSA,totalSample,immune-CD4_naive; MCSA,totalSample,immune-Tr1; MCSA,totalSample,immune-nTreg; MCSA,totalSample,immune-iTreg; MCSA,totalSample,immune-Th17; MCSA,totalSample,immune-Tfh; MCSA,totalSample,immune-CD8_naive; MCSA,totalSample,immune-Cytotoxic; MCSA,totalSample,immune-Exhausted; MCSA,totalSample,immune-Central_memory; ROSMAP,totalSample,immune-InfiltrationScore
ALDH7A1	Homo_723,female,AD.vs.control; Homo_723,male,AD.vs.control; Homo_723,female,AD.vs.MCI; Homo_723,male,AD.vs.MCI; Homo_723,female,MCI.vs.control; Homo_714,female,AD.vs.control; Homo_714,male,AD.vs.control; Homo_714,female,AD.vs.MCI; Homo_714,male,AD.vs.MCI; Homo_633,female,AD.vs.control; Homo_633,female,AD.vs.MCI; Emory,totalSample,immune-Gamma_delta; Emory,totalSample,immune-Tr1; Emory,totalSample,immune-iTreg; Emory,totalSample,immune-Cytotoxic; Emory,totalSample,immune-InfiltrationScore; Homo_723,totalSample,immune-CD4_naive; Homo_723,totalSample,immune-CD4_T; Homo_723,totalSample,immune-nTreg; Homo_723,totalSample,immune-Tr1; Homo_723,totalSample,immune-Th1; Homo_723,totalSample,immune-Th2; Homo_723,totalSample,immune-Tfh; Homo_723,totalSample,immune-Th17; Homo_723,totalSample,immune-Central_memory; Homo_723,totalSample,immune-Cytotoxic; Homo_723,totalSample,immune-iTreg; Homo_714,totalSample,immune-CD4_naive; Homo_714,totalSample,immune-Tr1; Homo_714,totalSample,immune-CD4_T; Homo_714,totalSample,immune-Tfh; Homo_714,totalSample,immune-Th1; Homo_633,totalSample,immune-Tfh; Homo_633,totalSample,immune-CD4_T; Homo_633,totalSample,immune-Tr1; Homo_633,totalSample,immune-Th2; Homo_633,totalSample,immune-CD4_naive; Homo_633,totalSample,immune-Central_memory; Homo_633,totalSample,immune-Gamma_delta; Homo_633,totalSample,immune-nTreg; Homo_633,totalSample,immune-iTreg; MCSA,totalSample,immune-DC; MCSA,totalSample,immune-Macrophage; MCSA,totalSample,immune-CD4_T; MCSA,totalSample,immune-CD4_naive; MCSA,totalSample,immune-Tr1; MCSA,totalSample,immune-nTreg; MCSA,totalSample,immune-iTreg; MCSA,totalSample,immune-Th17; MCSA,totalSample,immune-Tfh; MCSA,totalSample,immune-CD8_naive; MCSA,totalSample,immune-Central_memory; ROSMAP,totalSample,immune-InfiltrationScore
PARD3	Homo_723,female,AD.vs.control; Homo_723,male,AD.vs.control; Homo_723,female,AD.vs.MCI; Homo_723,male,AD.vs.MCI; Homo_723,female,MCI.vs.control; Homo_723,male,MCI.vs.control; Homo_714,female,AD.vs.control; Homo_714,male,AD.vs.control; Homo_714,female,AD.vs.MCI; Homo_714,male,AD.vs.MCI; Homo_714,female,MCI.vs.control; Homo_714,male,MCI.vs.control; Homo_633,female,AD.vs.control; Homo_633,female,AD.vs.MCI; ROSMAP,male,AD.vs.control; ROSMAP,MCI,last.visit.age; Emory,totalSample,immune-Neutrophil; Emory,totalSample,immune-Gamma_delta; Emory,totalSample,immune-Th17; Emory,totalSample,immune-Cytotoxic; Emory,totalSample,immune-Effector_memory; Homo_723,totalSample,immune-Th17; Homo_723,totalSample,immune-DC; Homo_723,totalSample,immune-Exhausted; Homo_723,totalSample,immune-CD8_T; Homo_723,totalSample,immune-Cytotoxic; Homo_723,totalSample,immune-MAIT; Homo_714,totalSample,immune-Exhausted; Homo_714,totalSample,immune-Th17; Homo_714,totalSample,immune-CD8_T; Homo_714,totalSample,immune-DC; Homo_714,totalSample,immune-MAIT; Homo_633,totalSample,immune-Gamma_delta; Homo_633,totalSample,immune-InfiltrationScore; Homo_633,totalSample,immune-Tfh; Homo_633,totalSample,immune-Th17; Homo_633,totalSample,immune-Exhausted; MCSA,totalSample,immune-Tr1; MCSA,totalSample,immune-iTreg; MCSA,totalSample,immune-Th17; MCSA,totalSample,immune-Exhausted; ROSMAP,totalSample,immune-Th17; ROSMAP,totalSample,immune-InfiltrationScore; SRP223445,totalSample,immune-CD4_naive; SRP223445,totalSample,immune-Exhausted
ZC3H12C	Homo_723,female,AD.vs.control; Homo_723,male,AD.vs.control; Homo_723,female,AD.vs.MCI; Homo_723,male,AD.vs.MCI; Homo_723,female,MCI.vs.control; Homo_723,male,MCI.vs.control; Homo_714,female,AD.vs.control; Homo_714,male,AD.vs.control; Homo_714,female,AD.vs.MCI; Homo_714,male,AD.vs.MCI; Homo_714,female,MCI.vs.control; Homo_714,male,MCI.vs.control; Homo_633,female,AD.vs.control; Homo_633,female,AD.vs.MCI; MCSA,MCI,age; Emory,totalSample,immune-Neutrophil; Emory,totalSample,immune-Gamma_delta; Emory,totalSample,immune-Tfh; Homo_723,totalSample,immune-Exhausted; Homo_723,totalSample,immune-Th17; Homo_723,totalSample,immune-Cytotoxic; Homo_723,totalSample,immune-CD8_T; Homo_723,totalSample,immune-Monocyte; Homo_723,totalSample,immune-CD4_naive; Homo_723,totalSample,immune-Gamma_delta; Homo_723,totalSample,immune-Macrophage; Homo_714,totalSample,immune-Exhausted; Homo_714,totalSample,immune-Th17; Homo_714,totalSample,immune-Monocyte; Homo_714,totalSample,immune-CD8_T; Homo_714,totalSample,immune-Cytotoxic; Homo_714,totalSample,immune-Gamma_delta; Homo_714,totalSample,immune-CD4_naive; Homo_633,totalSample,immune-Monocyte; Homo_633,totalSample,immune-Gamma_delta; Homo_633,totalSample,immune-Exhausted; Homo_633,totalSample,immune-Th2; Homo_633,totalSample,immune-Tfh; Homo_633,totalSample,immune-Th17; Homo_633,totalSample,immune-CD4_T; Homo_633,totalSample,immune-NKT; Homo_633,totalSample,immune-DC; Homo_633,totalSample,immune-InfiltrationScore; MCSA,totalSample,immune-DC; MCSA,totalSample,immune-Monocyte; MCSA,totalSample,immune-CD4_T; MCSA,totalSample,immune-CD8_T; MCSA,totalSample,immune-Gamma_delta; MCSA,totalSample,immune-CD4_naive; MCSA,totalSample,immune-Tr1; MCSA,totalSample,immune-nTreg; MCSA,totalSample,immune-iTreg; MCSA,totalSample,immune-Th17; MCSA,totalSample,immune-Tfh; MCSA,totalSample,immune-CD8_naive; MCSA,totalSample,immune-Cytotoxic;
FMNL3	Homo_723,female,AD.vs.control; Homo_723,male,AD.vs.control; Homo_723,male,AD.vs.MCI; Homo_723,female,MCI.vs.control; Homo_723,control,male.vs.female; Homo_723,MCI,male.vs.female; Homo_714,female,AD.vs.control; Homo_714,male,AD.vs.control; Homo_714,male,AD.vs.MCI; Homo_714,female,MCI.vs.control; Homo_714,control,male.vs.female; Homo_714,MCI,male.vs.female; Homo_633,female,AD.vs.control; Homo_633,female,AD.vs.MCI; Homo_633,female,MCI.vs.control; Homo_633,control,male.vs.female; Homo_633,MCI,male.vs.female; ROSMAP,male,AD.vs.control; SRP223445,male,AD.vs.control; MCSA,totalSample,age; MCSA,control,age; MCSA,MCI,age; ROSMAP,AD,MMSE; Emory,totalSample,immune-Macrophage; Emory,totalSample,immune-NK; Emory,totalSample,immune-Neutrophil; Emory,totalSample,immune-CD4_T; Emory,totalSample,immune-CD8_T; Emory,totalSample,immune-Tr1; Emory,totalSample,immune-nTreg; Emory,totalSample,immune-iTreg; Emory,totalSample,immune-Tfh; Emory,totalSample,immune-Exhausted; Emory,totalSample,immune-MAIT; Emory,totalSample,immune-Effector_memory; Emory,totalSample,immune-InfiltrationScore; Homo_723,totalSample,immune-Monocyte; Homo_723,totalSample,immune-DC; Homo_723,totalSample,immune-Exhausted; Homo_723,totalSample,immune-Neutrophil; Homo_723,totalSample,immune-CD4_naive; Homo_723,totalSample,immune-Central_memory; Homo_723,totalSample,immune-nTreg; Homo_723,totalSample,immune-CD8_T; Homo_714,totalSample,immune-Monocyte; Homo_714,totalSample,immune-CD4_naive; Homo_714,totalSample,immune-Neutrophil; Homo_714,totalSample,immune-DC; Homo_714,totalSample,immune-nTreg; Homo_633,totalSample,immune-Monocyte; Homo_633,totalSample,immune-CD4_naive; Homo_633,totalSample,immune-nTreg; MCSA,totalSample,immune-DC; MCSA,totalSample,immune-Monocyte; MCSA,totalSample,immune-Macrophage; MCSA,totalSample,immune-Neutrophil; MCSA,totalSample,immune-CD4_T; MCSA,totalSample,immune-CD8_T; MCSA,totalSample,immune-CD4_naive; MCSA,totalSample,immune-Tr1; MCSA,totalSample,immune-nTreg; MCSA,totalSample,immune-iTreg; MCSA,totalSample,immune-Th17; MCSA,totalSample,immune-Tfh; MCSA,totalSample,immune-Cytotoxic; MCSA,totalSample,immune-Central_memory; MCSA,totalSample,immune-Effector_memory; MCSA,totalSample,immune-InfiltrationScore; [illegible]
GRAMD4	Homo_723,female,AD.vs.control; Homo_723,male,AD.vs.control; Homo_723,female,AD.vs.MCI; Homo_723,male,AD.vs.MCI; Homo_723,female,MCI.vs.control; Homo_723,male,MCI.vs.control; Homo_723,AD,male.vs.female; Homo_723,MCI,male.vs.female; Homo_714,female,AD.vs.control; Homo_714,male,AD.vs.control; Homo_714,female,AD.vs.MCI; Homo_714,male,AD.vs.MCI; Homo_714,female,MCI.vs.control; Homo_714,male,MCI.vs.control; Homo_714,MCI,male.vs.female; Homo_633,female,AD.vs.control; Homo_633,female,AD.vs.MCI; Homo_633,female,MCI.vs.control; Homo_633,male,MCI.vs.control; Homo_633,MCI,male.vs.female; ROSMAP,male,AD.vs.control; Emory,MCI,age; Emory,totalSample,immune-Gamma_delta; Emory,totalSample,immune-Tr1; Emory,totalSample,immune-iTreg; Emory,totalSample,immune-InfiltrationScore; Homo_723,totalSample,immune-DC; Homo_723,totalSample,immune-Monocyte; Homo_723,totalSample,immune-InfiltrationScore; Homo_723,totalSample,immune-Central_memory; Homo_714,totalSample,immune-DC; Homo_714,totalSample,immune-Monocyte; Homo_714,totalSample,immune-InfiltrationScore; Homo_633,totalSample,immune-DC; Homo_633,totalSample,immune-Monocyte; Homo_633,totalSample,immune-InfiltrationScore; Homo_633,totalSample,immune-Th2; MCSA,totalSample,immune-DC; MCSA,totalSample,immune-Monocyte; MCSA,totalSample,immune-Neutrophil; MCSA,totalSample,immune-CD8_T; MCSA,totalSample,immune-NKT; MCSA,totalSample,immune-Th2; MCSA,totalSample,immune-Tfh; MCSA,totalSample,immune-Cytotoxic; MCSA,totalSample,immune-Central_memory; MCSA,totalSample,immune-Effector_memory; MCSA,totalSample,immune-InfiltrationScore; ROSMAP,totalSample,immune- [illegible]
KIF18A	Homo_723,female,AD.vs.control; Homo_723,male,AD.vs.control; Homo_723,female,AD.vs.MCI; Homo_723,male,AD.vs.MCI; Homo_723,female,MCI.vs.control; Homo_723,male,MCI.vs.control; Homo_714,female,AD.vs.control; Homo_714,male,AD.vs.control; Homo_714,female,AD.vs.MCI; Homo_714,male,AD.vs.MCI; Homo_714,female,MCI.vs.control; Homo_714,male,MCI.vs.control; Homo_633,female,AD.vs.control; Homo_633,female,AD.vs.MCI; Emory,control,age; ROSMAP,MCI,last.visit.age; Homo_723,totalSample,immune-Exhausted; Homo_723,totalSample,immune-Th17; Homo_723,totalSample,immune-Cytotoxic; Homo_723,totalSample,immune-CD8_T; Homo_723,totalSample,immune-Th1; Homo_723,totalSample,immune-CD4_naive; Homo_723,totalSample,immune-Neutrophil; Homo_714,totalSample,immune-Monocyte; Homo_714,totalSample,immune-Exhausted; Homo_714,totalSample,immune-CD4_naive; Homo_633,totalSample,immune-Tfh; Homo_633,totalSample,immune-Monocyte; Homo_633,totalSample,immune-Effector_memory; Homo_633,totalSample,immune-CD4_T; Homo_633,totalSample,immune-InfiltrationScore; Homo_633,totalSample,immune-Th2; Homo_633,totalSample,immune-CD4_naive; Homo_633,totalSample,immune-Central_memory; Homo_633,totalSample,immune-NKT; MCSA,totalSample,immune-DC; MCSA,totalSample,immune-Monocyte; MCSA,totalSample,immune-Macrophage; MCSA,totalSample,immune-CD4_T; MCSA,totalSample,immune-CD4_naive; MCSA,totalSample,immune-Tr1; MCSA,totalSample,immune-nTreg; MCSA,totalSample,immune-iTreg; MCSA,totalSample,immune-Th2; MCSA,totalSample,immune-Tfh; MCSA,totalSample,immune-CD8_naive; MCSA,totalSample,immune-Cytotoxic; MCSA,totalSample,immune- [illegible]
TOP2A	Homo_723,female,AD.vs.control; Homo_723,male,AD.vs.control; Homo_723,female,AD.vs.MCI; Homo_723,male,AD.vs.MCI; Homo_723,female,MCI.vs.control; Homo_723,male,MCI.vs.control; Homo_714,female,AD.vs.control; Homo_714,male,AD.vs.control; Homo_714,female,AD.vs.MCI; Homo_714,male,AD.vs.MCI; Homo_714,female,MCI.vs.control; Homo_714,male,MCI.vs.control; Homo_633,female,AD.vs.control; Homo_633,female,AD.vs.MCI; Emory,totalSample,immune-Neutrophil; Emory,totalSample,immune-InfiltrationScore; Homo_723,totalSample,immune-Exhausted; Homo_723,totalSample,immune-Th17; Homo_723,totalSample,immune-Cytotoxic; Homo_723,totalSample,immune-CD4_naive; Homo_723,totalSample,immune-CD4_T; Homo_723,totalSample,immune-CD8_T; Homo_723,totalSample,immune-Tr1; Homo_723,totalSample,immune-Tfh; Homo_723,totalSample,immune-Th1; Homo_723,totalSample,immune-Central_memory; Homo_723,totalSample,immune-nTreg; Homo_723,totalSample,immune-InfiltrationScore; Homo_723,totalSample,immune-Neutrophil; Homo_723,totalSample,immune-Th2; Homo_714,totalSample,immune-CD4_naive; Homo_714,totalSample,immune-Th17; Homo_714,totalSample,immune-Exhausted; Homo_714,totalSample,immune-CD4_T; Homo_714,totalSample,immune-InfiltrationScore; Homo_714,totalSample,immune-Tfh; Homo_714,totalSample,immune-Central_memory; Homo_714,totalSample,immune-Monocyte; Homo_633,totalSample,immune-Tfh; Homo_633,totalSample,immune-CD4_T; Homo_633,totalSample,immune-Effector_memory; Homo_633,totalSample,immune-Th2; Homo_633,totalSample,immune-CD4_naive; Homo_633,totalSample,immune-Tr1; Homo_633,totalSample,immune-Central_memory; Homo_633,totalSample,immune-InfiltrationScore; Homo_633,totalSample,immune-Gamma_delta; Homo_633,totalSample,immune-iTreg; Homo_633,totalSample,immune-Monocyte; Homo_633,totalSample,immune-Th17; Homo_633,totalSample,immune-Exhausted; MCSA,totalSample,immune-DC; MCSA,totalSample,immune-CD4_T; MCSA,totalSample,immune-CD8_T; MCSA,totalSample,immune-NKT; MCSA,totalSample,immune-CD4_naive; MCSA,totalSample,immune-Tr1; MCSA,totalSample,immune-nTreg; MCSA,totalSample,immune-iTreg; MCSA,totalSample,immune-Th17; MCSA,totalSample,immune-Tfh; MCSA,totalSample,immune-CD8_naive; MCSA,totalSample,immune-Cytotoxic; MCSA,totalSample,immune-Exhausted; [illegible]
CPLX2	Homo_723,female,AD.vs.control; Homo_723,female,AD.vs.MCI; Homo_723,male,MCI.vs.control; Homo_714,female,MCI.vs.control; Homo_714,male,MCI.vs.control; Homo_633,female,AD.vs.MCI; Homo_633,female,MCI.vs.control; Homo_633,male,MCI.vs.control; ROSMAP,female,AD.vs.control; ROSMAP,female,MCI.vs.control; MCSA,MCI,age; Emory,totalSample,immune-Th17; Homo_723,totalSample,immune-Monocyte; ROSMAP,totalSample,immune-InfiltrationScore
CA13	Homo_723,female,AD.vs.control; Homo_723,male,AD.vs.control; Homo_723,female,AD.vs.MCI; Homo_723,male,AD.vs.MCI; Homo_723,female,MCI.vs.control; Homo_723,male,MCI.vs.control; Homo_714,female,AD.vs.control; Homo_714,male,AD.vs.control; Homo_714,female,AD.vs.MCI; Homo_714,male,AD.vs.MCI; Homo_714,female,MCI.vs.control; Homo_714,male,MCI.vs.control; Homo_633,female,AD.vs.control; Homo_633,female,AD.vs.MCI; ROSMAP,female,AD.vs.control; ROSMAP,male,AD.vs.control; ROSMAP,female,AD.vs.MCI; Emory,totalSample,immune-Gamma_delta; Emory,totalSample,immune-Tr1; Emory,totalSample,immune-iTreg; Emory,totalSample,immune-Cytotoxic; Homo_723,totalSample,immune-Exhausted; Homo_723,totalSample,immune-Th17; Homo_723,totalSample,immune-Cytotoxic; Homo_723,totalSample,immune-CD8_T; Homo_723,totalSample,immune-CD4_naive; Homo_723,totalSample,immune-Th1; Homo_723,totalSample,immune-CD4_T; Homo_723,totalSample,immune-Tr1; Homo_723,totalSample,immune-InfiltrationScore; Homo_723,totalSample,immune-NKT; Homo_714,totalSample,immune-Th17; Homo_714,totalSample,immune-Exhausted; Homo_714,totalSample,immune-Monocyte; Homo_714,totalSample,immune-CD4_naive; Homo_714,totalSample,immune-NKT; Homo_714,totalSample,immune-CD8_T; Homo_714,totalSample,immune-Cytotoxic; Homo_714,totalSample,immune-Th1; Homo_633,totalSample,immune-Tfh; Homo_633,totalSample,immune-NKT; Homo_633,totalSample,immune-Th2; Homo_633,totalSample,immune-CD4_T; Homo_633,totalSample,immune-Monocyte; Homo_633,totalSample,immune-CD4_naive; Homo_633,totalSample,immune-Gamma_delta; Homo_633,totalSample,immune-Tr1; Homo_633,totalSample,immune-InfiltrationScore; Homo_633,totalSample,immune-Th17; Homo_633,totalSample,immune-Exhausted; Homo_633,totalSample,immune-Central_memory; Homo_633,totalSample,immune-Effector_memory; MCSA,totalSample,immune-DC; MCSA,totalSample,immune-Bcell; MCSA,totalSample,immune-Monocyte; MCSA,totalSample,immune-Macrophage; MCSA,totalSample,immune-CD4_T; MCSA,totalSample,immune-CD8_T; MCSA,totalSample,immune-CD4_naive; MCSA,totalSample,immune-Tr1; MCSA,totalSample,immune-nTreg; MCSA,totalSample,immune-iTreg; MCSA,totalSample,immune-Tfh; MCSA,totalSample,immune-CD8_naive; MCSA,totalSample,immune-Cytotoxic; [illegible]
HROB	Homo_723,female,AD.vs.control; Homo_723,male,AD.vs.control; Homo_723,female,AD.vs.MCI; Homo_723,male,AD.vs.MCI; Homo_714,female,AD.vs.control; Homo_714,male,AD.vs.control; Homo_714,female,AD.vs.MCI; Homo_714,male,AD.vs.MCI; Homo_633,female,AD.vs.control; Homo_633,female,AD.vs.MCI; Homo_633,female,MCI.vs.control; ROSMAP,female,AD.vs.control; ROSMAP,male,AD.vs.control; ROSMAP,female,AD.vs.MCI; MCSA,totalSample,age; MCSA,control,age; MCSA,MCI,age; Homo_723,totalSample,immune-DC; Homo_723,totalSample,immune-Monocyte; Homo_723,totalSample,immune-Neutrophil; Homo_714,totalSample,immune-DC; Homo_714,totalSample,immune-Th2; Homo_714,totalSample,immune-Monocyte; Homo_633,totalSample,immune-Monocyte; Homo_633,totalSample,immune-DC; Homo_633,totalSample,immune-InfiltrationScore; MCSA,totalSample,immune-DC; MCSA,totalSample,immune-Monocyte; MCSA,totalSample,immune-Neutrophil; MCSA,totalSample,immune-CD8_T; MCSA,totalSample,immune-Th2; MCSA,totalSample,immune-Th17; MCSA,totalSample,immune-Cytotoxic; MCSA,totalSample,immune-Effector_memory; MCSA,totalSample,immune-InfiltrationScore; SRP223445,totalSample,immune-Exhausted; ROSMAP,male-excludeAge90+,survival

AFF2	Homo_723,female,AD.vs.control; Homo_723,male,AD.vs.control; Homo_723,female,AD.vs.MCI; Homo_723,male,AD.vs.MCI; Homo_723,female,MCI.vs.control; Homo_723,male,MCI.vs.control; Homo_714,female,AD.vs.control; Homo_714,male,AD.vs.control; Homo_714,female,AD.vs.MCI; Homo_714,male,AD.vs.MCI; Homo_714,female,MCI.vs.control; Homo_714,male,MCI.vs.control; Homo_633,female,AD.vs.control; Homo_633,female,AD.vs.MCI; ROSMAP,male,AD.vs.control; ROSMAP,female,AD.vs.MCI; ROSMAP,male,AD.vs.MCI; ROSMAP,AD,male.vs.female; ROSMAP,MCI,male.vs.female; MCSA,MCI,age; Emory,totalSample,immune-Neutrophil; Emory,totalSample,immune-Gamma_delta; Emory,totalSample,immune-Th17; Emory,totalSample,immune-Exhausted; Homo_723,totalSample,immune-Exhausted; Homo_723,totalSample,immune-Th17; Homo_723,totalSample,immune-CD8_T; Homo_723,totalSample,immune-Cytotoxic; Homo_723,totalSample,immune-Monocyte; Homo_723,totalSample,immune-Macrophage; Homo_723,totalSample,immune-MAIT; Homo_723,totalSample,immune-Gamma_delta; Homo_714,totalSample,immune-Exhausted; Homo_714,totalSample,immune-Th17; Homo_714,totalSample,immune-CD8_T; Homo_714,totalSample,immune-Monocyte; Homo_714,totalSample,immune-Macrophage; Homo_714,totalSample,immune-Gamma_delta; Homo_714,totalSample,immune-Cytotoxic; Homo_714,totalSample,immune-MAIT; Homo_633,totalSample,immune-Th2; Homo_633,totalSample,immune-Gamma_delta; Homo_633,totalSample,immune-Monocyte; Homo_633,totalSample,immune-Exhausted; Homo_633,totalSample,immune-Th17; Homo_633,totalSample,immune-CD8_T; Homo_633,totalSample,immune-Macrophage; Homo_633,totalSample,immune-Tfh; MCSA,totalSample,immune-DC; MCSA,totalSample,immune-Monocyte; MCSA,totalSample,immune-Neutrophil; MCSA,totalSample,immune-CD8_T; MCSA,totalSample,immune-Gamma_delta; MCSA,totalSample,immune-iTreg; MCSA,totalSample,immune-Th1; MCSA,totalSample,immune-Th17; MCSA,totalSample,immune-Cytotoxic; MCSA,totalSample,immune-Exhausted; MCSA,totalSample,immune-Effector_memory; MCSA,totalSample,immune-InfiltrationScore; SRP223445,totalSample,immune-Monocyte; SRP223445,totalSample,immune-CD4_T; SRP223445,totalSample,immune-CD4_naive; SRP223445,totalSample,immune-nTreg; SRP223445,totalSample,immune-Th1; SRP223445,totalSample,immune-Central_memory
MPP2	Homo_723,female,AD.vs.control; Homo_723,female,AD.vs.MCI; Homo_633,female,AD.vs.control; Homo_633,female,AD.vs.MCI; ROSMAP,male,MCI.vs.control; ROSMAP,MCI,male.vs.female; MCSA,totalSample,age; MCSA,control,age; Emory,totalSample,immune-CD4_T; Emory,totalSample,immune-CD4_naive; Emory,totalSample,immune-Tr1; Emory,totalSample,immune-nTreg; Emory,totalSample,immune-iTreg; Emory,totalSample,immune-Th17; Emory,totalSample,immune-Cytotoxic; Emory,totalSample,immune-Central_memory; Homo_723,totalSample,immune-nTreg; Homo_723,totalSample,immune-Central_memory; Homo_723,totalSample,immune-CD4_naive; Homo_723,totalSample,immune-Monocyte; Homo_723,totalSample,immune-CD4_T; Homo_723,totalSample,immune-Tfh; Homo_723,totalSample,immune-iTreg; Homo_723,totalSample,immune-CD8_T; Homo_723,totalSample,immune-Tr1; Homo_723,totalSample,immune-DC; Homo_723,totalSample,immune-InfiltrationScore; Homo_714,totalSample,immune-nTreg; Homo_714,totalSample,immune-Tfh; Homo_714,totalSample,immune-CD4_naive; Homo_714,totalSample,immune-InfiltrationScore; Homo_714,totalSample,immune-iTreg; Homo_714,totalSample,immune-Central_memory; Homo_714,totalSample,immune-Monocyte; Homo_714,totalSample,immune-Tr1; Homo_714,totalSample,immune-CD4_T; Homo_633,totalSample,immune-nTreg; Homo_633,totalSample,immune-Tfh; Homo_633,totalSample,immune-Monocyte; Homo_633,totalSample,immune-iTreg; Homo_633,totalSample,immune-CD4_naive; Homo_633,totalSample,immune-Tr1; Homo_633,totalSample,immune-Central_memory; Homo_633,totalSample,immune-CD4_T; Homo_633,totalSample,immune-InfiltrationScore; MCSA,totalSample,immune-Monocyte; MCSA,totalSample,immune-Neutrophil; MCSA,totalSample,immune-CD4_T; MCSA,totalSample,immune-CD8_T; MCSA,totalSample,immune-CD4_naive; MCSA,totalSample,immune-Tr1; MCSA,totalSample,immune-nTreg; MCSA,totalSample,immune-iTreg; MCSA,totalSample,immune-Th17; MCSA,totalSample,immune-Tfh; MCSA,totalSample,immune- [illegible]
IGFLR1	Homo_723,female,AD.vs.control; Homo_723,male,AD.vs.control; Homo_723,female,AD.vs.MCI; Homo_723,male,AD.vs.MCI; Homo_723,female,MCI.vs.control; Homo_723,male,MCI.vs.control; Homo_714,female,AD.vs.control; Homo_714,male,AD.vs.control; Homo_714,female,AD.vs.MCI; Homo_714,male,AD.vs.MCI; Homo_714,female,MCI.vs.control; Homo_714,male,MCI.vs.control; Homo_633,female,AD.vs.control; Homo_633,female,AD.vs.MCI; Homo_633,female,MCI.vs.control; Homo_633,male,MCI.vs.control; Emory,MCI,age; MCSA,totalSample,age; MCSA,control,age; MCSA,MCI,age; Emory,totalSample,immune-CD8_naive; Emory,totalSample,immune-InfiltrationScore; Homo_723,totalSample,immune-Exhausted; Homo_723,totalSample,immune-CD8_T; Homo_723,totalSample,immune-Cytotoxic; Homo_723,totalSample,immune-Monocyte; Homo_723,totalSample,immune-Th17; Homo_723,totalSample,immune-DC; Homo_714,totalSample,immune-Monocyte; Homo_714,totalSample,immune-DC; Homo_714,totalSample,immune-Th17; Homo_633,totalSample,immune-DC; Homo_633,totalSample,immune-Monocyte; Homo_633,totalSample,immune-Th2; Homo_633,totalSample,immune-Th17; MCSA,totalSample,immune-DC; MCSA,totalSample,immune-Monocyte; MCSA,totalSample,immune-Macrophage; MCSA,totalSample,immune-Neutrophil; MCSA,totalSample,immune-CD8_T; MCSA,totalSample,immune-iTreg; MCSA,totalSample,immune-Th2; MCSA,totalSample,immune-Cytotoxic; MCSA,totalSample,immune-Effector_memory; MCSA,totalSample,immune-InfiltrationScore; ROSMAP,totalSample,immune-iTreg; ROSMAP,totalSample,immune-Th17; ROSMAP,totalSample,immune-Cytotoxic; ROSMAP,totalSample,immune-Effector_memory; SRP223445,totalSample,immune-CD4_T; SRP223445,totalSample,immune-Th17; SRP223445,totalSample,immune-InfiltrationScore; ROSMAP,female-excludeAge90+,survival; ROSMAP,female-includeAge90+,survival
SYN3	Homo_723,female,AD.vs.control; Homo_723,male,AD.vs.control; Homo_723,female,AD.vs.MCI; Homo_723,male,AD.vs.MCI; Homo_714,female,MCI.vs.control; Homo_633,female,AD.vs.control; Homo_633,female,AD.vs.MCI; Homo_633,female,MCI.vs.control; Homo_633,male,MCI.vs.control; ROSMAP,male,AD.vs.control; ROSMAP,male,MCI.vs.control; ROSMAP,control,male.vs.female; MCSA,totalSample,age; MCSA,control,age; Emory,totalSample,immune-B_cell; Emory,totalSample,immune-InfiltrationScore; Homo_723,totalSample,immune-DC; Homo_723,totalSample,immune-Monocyte; Homo_723,totalSample,immune-Neutrophil; Homo_633,totalSample,immune-Monocyte; MCSA,totalSample,immune-Bcell; MCSA,totalSample,immune-Monocyte; MCSA,totalSample,immune-CD4_T; MCSA,totalSample,immune-Gamma_delta; MCSA,totalSample,immune-CD4_naive; MCSA,totalSample,immune-Tr1; MCSA,totalSample,immune-nTreg; MCSA,totalSample,immune-iTreg; MCSA,totalSample,immune-Th17; MCSA,totalSample,immune-Tfh; MCSA,totalSample,immune-Central_memory; MCSA,totalSample,immune-InfiltrationScore; ROSMAP,totalSample,immune-InfiltrationScore; SRP223445,totalSample,immune-InfiltrationScore; ROSMAP,male-includeAge90+,survival
PLTP	Homo_723,female,AD.vs.control; Homo_723,male,AD.vs.control; Homo_723,female,AD.vs.MCI; Homo_723,male,AD.vs.MCI; Homo_723,female,MCI.vs.control; Homo_714,female,AD.vs.control; Homo_714,male,AD.vs.control; Homo_714,female,MCI.vs.control; Homo_714,male,MCI.vs.control; Homo_633,female,AD.vs.control; Homo_633,female,AD.vs.MCI; Homo_633,female,MCI.vs.control; Homo_633,male,MCI.vs.control; ROSMAP,female,AD.vs.control; ROSMAP,female,MCI.vs.control; ROSMAP,MCI,last.visit.age; ROSMAP,MCI,sample.age; Homo_723,totalSample,immune-DC; Homo_723,totalSample,immune-Monocyte; Homo_714,totalSample,immune-Th2; Homo_714,totalSample,immune-DC; Homo_714,totalSample,immune-Monocyte; Homo_633,totalSample,immune-Monocyte; Homo_633,totalSample,immune-Th2; Homo_633,totalSample,immune-DC; MCSA,totalSample,immune-DC; MCSA,totalSample,immune-Monocyte; MCSA,totalSample,immune-Th2; MCSA,totalSample,immune-Cytotoxic; MCSA,totalSample,immune-Effector_memory;
MACIR	Homo_723,female,AD.vs.control; Homo_723,male,AD.vs.control; Homo_723,female,AD.vs.MCI; Homo_723,male,AD.vs.MCI; Homo_714,female,AD.vs.control; Homo_714,male,AD.vs.control; Homo_714,female,AD.vs.MCI; Homo_714,male,AD.vs.MCI; Homo_633,female,AD.vs.control; Homo_633,female,AD.vs.MCI; MCSA,MCI,age; ROSMAP,totalSample,age; ROSMAP,control,sample.age; Emory,totalSample,immune-Monocyte; Emory,totalSample,immune-Tr1; Emory,totalSample,immune-iTreg; Emory,totalSample,immune-InfiltrationScore; Homo_723,totalSample,immune-Exhausted; Homo_723,totalSample,immune-Cytotoxic; Homo_723,totalSample,immune-CD8_T; Homo_723,totalSample,immune-Th17; Homo_723,totalSample,immune-CD4_naive; Homo_723,totalSample,immune-CD4_T; Homo_723,totalSample,immune-NKT; Homo_723,totalSample,immune-Th1; Homo_723,totalSample,immune-Tr1; Homo_723,totalSample,immune-InfiltrationScore; Homo_723,totalSample,immune-Central_memory; Homo_714,totalSample,immune-Exhausted; Homo_714,totalSample,immune-NKT; Homo_714,totalSample,immune-CD4_naive; Homo_714,totalSample,immune-Monocyte; Homo_714,totalSample,immune-Th17; Homo_714,totalSample,immune-InfiltrationScore; Homo_714,totalSample,immune-CD8_T; Homo_714,totalSample,immune-CD4_T; Homo_714,totalSample,immune-Cytotoxic; Homo_633,totalSample,immune-Tfh; Homo_633,totalSample,immune-NKT; Homo_633,totalSample,immune-CD4_T; Homo_633,totalSample,immune-CD4_naive; Homo_633,totalSample,immune-Th2; Homo_633,totalSample,immune-InfiltrationScore; Homo_633,totalSample,immune-Monocyte; Homo_633,totalSample,immune-Gamma_delta; Homo_633,totalSample,immune-Central_memory; Homo_633,totalSample,immune-Effector_memory; Homo_633,totalSample,immune-DC; Homo_633,totalSample,immune-Tr1; Homo_633,totalSample,immune-Exhausted; MCSA,totalSample,immune-DC; MCSA,totalSample,immune-Monocyte; MCSA,totalSample,immune-Macrophage; MCSA,totalSample,immune-CD4_T; MCSA,totalSample,immune-CD8_T; MCSA,totalSample,immune-CD4_naive; MCSA,totalSample,immune-Tr1; MCSA,totalSample,immune-nTreg; MCSA,totalSample,immune-iTreg; MCSA,totalSample,immune-Tfh; MCSA,totalSample,immune-CD8_naive; MCSA,totalSample,immune-Cytotoxic; MCSA,totalSample,immune-Exhausted; MCSA,totalSample,immune-MAIT; [illegible]
NAGS	Homo_723,female,AD.vs.control; Homo_723,male,AD.vs.control; Homo_723,female,AD.vs.MCI; Homo_723,male,AD.vs.MCI; Homo_714,female,AD.vs.control; Homo_714,male,AD.vs.control; Homo_714,female,AD.vs.MCI; Homo_714,male,AD.vs.MCI; Homo_633,female,AD.vs.control; Homo_633,female,AD.vs.MCI; ROSMAP,female,AD.vs.control; ROSMAP,male,AD.vs.control; MCSA,totalSample,age; MCSA,control,age; MCSA,MCI,age; Emory,totalSample,immune-InfiltrationScore; Homo_723,totalSample,immune-InfiltrationScore; Homo_723,totalSample,immune-CD8_T; Homo_723,totalSample,immune-Cytotoxic; Homo_723,totalSample,immune-Effector_memory; Homo_723,totalSample,immune-DC; Homo_723,totalSample,immune-Exhausted; Homo_714,totalSample,immune-InfiltrationScore; Homo_714,totalSample,immune-DC; Homo_714,totalSample,immune-Effector_memory; Homo_714,totalSample,immune-CD8_T; Homo_714,totalSample,immune-Cytotoxic; Homo_633,totalSample,immune-DC; Homo_633,totalSample,immune-InfiltrationScore; Homo_633,totalSample,immune-Effector_memory; Homo_633,totalSample,immune-nTreg; Homo_633,totalSample,immune-Neutrophil; MCSA,totalSample,immune-DC; MCSA,totalSample,immune-Monocyte; MCSA,totalSample,immune-Neutrophil; MCSA,totalSample,immune-CD8_T; MCSA,totalSample,immune-NKT; MCSA,totalSample,immune-Th2; MCSA,totalSample,immune-Cytotoxic; MCSA,totalSample,immune-Central_memory; MCSA,totalSample,immune-Effector_memory; MCSA,totalSample,immune-InfiltrationScore; SRP223445,totalSample,immune-Neutrophil; SRP223445,totalSample,immune-Th17; SRP223445,totalSample,immune-InfiltrationScore
LOC102723750	Homo_723,female,AD.vs.control; Homo_723,male,AD.vs.control; Homo_723,female,AD.vs.MCI; Homo_723,male,AD.vs.MCI; Homo_723,female,MCI.vs.control; Homo_714,female,AD.vs.control; Homo_714,male,AD.vs.control; Homo_714,female,AD.vs.MCI; Homo_714,male,AD.vs.MCI; Homo_714,female,MCI.vs.control; Homo_633,female,AD.vs.control; Homo_633,female,AD.vs.MCI; Emory,MCI,age; Emory,totalSample,immune-Gamma_delta; Emory,totalSample,immune-Tr1; Emory,totalSample,immune-iTreg; Homo_723,totalSample,immune-Exhausted; Homo_723,totalSample,immune-CD8_T; Homo_723,totalSample,immune-Cytotoxic; Homo_723,totalSample,immune-Th17; Homo_723,totalSample,immune-CD8_naive; Homo_714,totalSample,immune-CD8_naive; Homo_633,totalSample,immune-DC; Homo_633,totalSample,immune-Th2; Homo_633,totalSample,immune-Bcell; MCSA,totalSample,immune-DC; MCSA,totalSample,immune-Monocyte; MCSA,totalSample,immune-Macrophage; MCSA,totalSample,immune-Neutrophil; MCSA,totalSample,immune-CD8_T; MCSA,totalSample,immune-NKT; MCSA,totalSample,immune-Th2; MCSA,totalSample,immune-Cytotoxic; MCSA,totalSample,immune-Effector_memory
ELAC2	Homo_723,female,AD.vs.control; Homo_723,male,AD.vs.control; Homo_723,female,AD.vs.MCI; Homo_723,male,AD.vs.MCI; Homo_714,female,AD.vs.control; Homo_714,male,AD.vs.control; Homo_714,female,AD.vs.MCI; Homo_714,male,AD.vs.MCI; Homo_633,female,AD.vs.control; Homo_633,female,AD.vs.MCI; MCSA,totalSample,age; MCSA,control,age; ROSMAP,AD,last.visit.age; ROSMAP,MCI,last.visit.age; Emory,totalSample,immune-DC; Emory,totalSample,immune-Monocyte; Emory,totalSample,immune-NK; Emory,totalSample,immune-Neutrophil; Emory,totalSample,immune-CD4_T; Emory,totalSample,immune-CD8_T; Emory,totalSample,immune-Tr1; Emory,totalSample,immune-iTreg; Emory,totalSample,immune-Exhausted; Emory,totalSample,immune-MAIT; Emory,totalSample,immune-InfiltrationScore; Homo_723,totalSample,immune-Effector_memory; Homo_723,totalSample,immune-InfiltrationScore; Homo_723,totalSample,immune-Tfh; Homo_723,totalSample,immune-iTreg; Homo_723,totalSample,immune-Th2; Homo_723,totalSample,immune-nTreg; Homo_723,totalSample,immune-CD4_naive; Homo_723,totalSample,immune-CD4_T; Homo_723,totalSample,immune-CD8_T; Homo_723,totalSample,immune-Central_memory; Homo_723,totalSample,immune-Tr1; Homo_723,totalSample,immune-Macrophage; Homo_723,totalSample,immune-Bcell; Homo_723,totalSample,immune-MAIT; Homo_714,totalSample,immune-Effector_memory; Homo_714,totalSample,immune-Tfh; Homo_714,totalSample,immune-InfiltrationScore; Homo_714,totalSample,immune-nTreg; Homo_714,totalSample,immune-Th2; Homo_714,totalSample,immune-CD4_T; Homo_714,totalSample,immune-Central_memory; Homo_714,totalSample,immune-CD4_naive; Homo_714,totalSample,immune-Tr1; Homo_714,totalSample,immune-iTreg; Homo_714,totalSample,immune-Macrophage; Homo_714,totalSample,immune-CD8_T; Homo_714,totalSample,immune-Monocyte; Homo_714,totalSample,immune-MAIT; Homo_633,totalSample,immune-Effector_memory; Homo_633,totalSample,immune-InfiltrationScore; Homo_633,totalSample,immune-nTreg; Homo_633,totalSample,immune-Tfh; Homo_633,totalSample,immune-CD4_naive; Homo_633,totalSample,immune-Central_memory; Homo_633,totalSample,immune-CD4_T; Homo_633,totalSample,immune-Tr1; Homo_633,totalSample,immune-iTreg; Homo_633,totalSample,immune-Neutrophil; Homo_633,totalSample,immune-DC; Homo_633,totalSample,immune-Monocyte; Homo_633,totalSample,immune-Th2; MCSA,totalSample,immune-DC; MCSA,totalSample,immune-Monocyte; MCSA,totalSample,immune-Neutrophil; MCSA,totalSample,immune-CD4_T; MCSA,totalSample,immune-CD8_T; MCSA,totalSample,immune-NKT; MCSA,totalSample,immune-CD4_naive; MCSA,totalSample,immune-Tr1; MCSA,totalSample,immune-nTreg; MCSA,totalSample,immune-iTreg; MCSA,totalSample,immune-Th2; MCSA,totalSample,immune-Th17; MCSA,totalSample,immune-Tfh; MCSA,totalSample,immune-CD8_naive; MCSA,totalSample,immune-MAIT; MCSA,totalSample,immune-Central_memory; MCSA,totalSample,immune-Effector_memory; MCSA,totalSample,immune-InfiltrationScore; ROSMAP,totalSample,immune-Cytotoxic; ROSMAP,totalSample,immune-MAIT; ROSMAP,totalSample,immune-Effector_memory; SRP223445,totalSample,immune-CD4_naive; SRP223445,totalSample,immune-Th17; SRP223445,totalSample,immune-Exhausted; SRP223445,totalSample,immune-InfiltrationScore; ROSMAP,female-excludeAge90+,survival; ROSMAP,male-[illegible]
CIAPIN1	Homo_723,female,AD.vs.control; Homo_723,male,AD.vs.control; Homo_723,female,AD.vs.MCI; Homo_723,male,AD.vs.MCI; Homo_714,female,AD.vs.control; Homo_714,male,AD.vs.control; Homo_714,female,AD.vs.MCI; Homo_714,male,AD.vs.MCI; Homo_633,female,AD.vs.control; Homo_633,female,AD.vs.MCI; MCSA,totalSample,age; MCSA,control,age; Emory,totalSample,immune-Macrophage; Emory,totalSample,immune-NK; Emory,totalSample,immune-Neutrophil; Emory,totalSample,immune-CD4_T; Emory,totalSample,immune-CD8_T; Emory,totalSample,immune-Tr1; Emory,totalSample,immune-iTreg; Emory,totalSample,immune-Tfh; Emory,totalSample,immune-Exhausted; Emory,totalSample,immune-MAIT; Emory,totalSample,immune-Central_memory; Emory,totalSample,immune-Effector_memory; Emory,totalSample,immune-InfiltrationScore; Homo_723,totalSample,immune-Th2; Homo_723,totalSample,immune-Tfh; Homo_723,totalSample,immune-iTreg; Homo_723,totalSample,immune-CD4_T; Homo_723,totalSample,immune-Effector_memory; Homo_723,totalSample,immune-CD4_naive; Homo_723,totalSample,immune-Tr1; Homo_723,totalSample,immune-InfiltrationScore; Homo_723,totalSample,immune-nTreg; Homo_723,totalSample,immune-Macrophage; Homo_723,totalSample,immune-Central_memory; Homo_723,totalSample,immune-Bcell; Homo_723,totalSample,immune-CD8_T; Homo_714,totalSample,immune-Tfh; Homo_714,totalSample,immune-Th2; Homo_714,totalSample,immune-Effector_memory; Homo_714,totalSample,immune-CD4_T; Homo_714,totalSample,immune-Tr1; Homo_714,totalSample,immune-Central_memory; Homo_714,totalSample,immune-CD4_naive; Homo_714,totalSample,immune-InfiltrationScore; Homo_714,totalSample,immune-iTreg; Homo_714,totalSample,immune-nTreg; Homo_714,totalSample,immune-Macrophage; Homo_714,totalSample,immune-Bcell; Homo_633,totalSample,immune-Tfh; Homo_633,totalSample,immune-Effector_memory; Homo_633,totalSample,immune-CD4_T; Homo_633,totalSample,immune-CD4_naive; Homo_633,totalSample,immune-InfiltrationScore; Homo_633,totalSample,immune-Th2; Homo_633,totalSample,immune-Central_memory; Homo_633,totalSample,immune-Tr1; Homo_633,totalSample,immune-nTreg; Homo_633,totalSample,immune-Bcell; Homo_633,totalSample,immune-iTreg; Homo_633,totalSample,immune-Neutrophil; Homo_633,totalSample,immune-Th17; MCSA,totalSample,immune-Monocyte; MCSA,totalSample,immune-Macrophage; MCSA,totalSample,immune-Neutrophil; MCSA,totalSample,immune-CD4_T; MCSA,totalSample,immune-CD4_naive; MCSA,totalSample,immune-Tr1; MCSA,totalSample,immune-nTreg; MCSA,totalSample,immune-iTreg; MCSA,totalSample,immune-Th17; MCSA,totalSample,immune-Tfh; MCSA,totalSample,immune-CD8_naive; MCSA,totalSample,immune-MAIT; MCSA,totalSample,immune-Central_memory; MCSA,totalSample,immune-Effector_memory; MCSA,totalSample,immune-InfiltrationScore; ROSMAP,totalSample,immune-Effector_memory; SRP223445,totalSample,immune-NK; SRP223445,totalSample,immune-Neutrophil; SRP223445,totalSample,immune-CD4_naive; [illegible]
TRIM45	Homo_723,female,AD.vs.control; Homo_723,female,AD.vs.MCI; Homo_723,male,AD.vs.MCI; Homo_714,male,AD.vs.control; Homo_714,male,AD.vs.MCI; Homo_633,female,AD.vs.control; Homo_633,female,AD.vs.MCI; ROSMAP,female,AD.vs.control; ROSMAP,male,AD.vs.control; ROSMAP,female,MCI.vs.control; ROSMAP,male,MCI.vs.control; ACOM,totalSample,age; MCSA,control,age; MCSA,MCI,age; ROSMAP,MCI,last.visit.age; ROSMAP,MCI,sample.age; Emory,totalSample,immune-Gamma_delta; Emory,totalSample,immune-Th17; Emory,totalSample,immune-Cytotoxic; Homo_723,totalSample,immune-Tfh; Homo_723,totalSample,immune-CD4_T; Homo_723,totalSample,immune-iTreg; Homo_723,totalSample,immune-CD4_naive; Homo_723,totalSample,immune-Central_memory; Homo_723,totalSample,immune-nTreg; Homo_723,totalSample,immune-Th2; Homo_723,totalSample,immune-Tr1; Homo_723,totalSample,immune-CD8_naive; Homo_723,totalSample,immune-InfiltrationScore; Homo_723,totalSample,immune-Effector_memory; Homo_714,totalSample,immune-Tfh; Homo_714,totalSample,immune-CD4_T; Homo_714,totalSample,immune-CD4_naive; Homo_714,totalSample,immune-Central_memory; Homo_714,totalSample,immune-iTreg; Homo_714,totalSample,immune-Effector_memory; Homo_714,totalSample,immune-InfiltrationScore; Homo_714,totalSample,immune-Tr1; Homo_714,totalSample,immune-nTreg; Homo_714,totalSample,immune-Th2; Homo_633,totalSample,immune-Tfh; Homo_633,totalSample,immune-InfiltrationScore; Homo_633,totalSample,immune-CD4_naive; Homo_633,totalSample,immune-CD4_T; Homo_633,totalSample,immune-Central_memory; Homo_633,totalSample,immune-Effector_memory; Homo_633,totalSample,immune-NKT; Homo_633,totalSample,immune-Tr1; Homo_633,totalSample,immune-nTreg; Homo_633,totalSample,immune-iTreg; MCSA,totalSample,immune-Neutrophil; MCSA,totalSample,immune-CD4_T; MCSA,totalSample,immune-CD4_naive; MCSA,totalSample,immune-Tr1; MCSA,totalSample,immune-nTreg; MCSA,totalSample,immune-iTreg; MCSA,totalSample,immune-Th17; MCSA,totalSample,immune-Tfh; MCSA,totalSample,immune-CD8_naive; MCSA,totalSample,immune-MAIT; MCSA,totalSample,immune-Central_memory; MCSA,totalSample,immune-Effector_memory
FAM110B	Homo_723,female,AD.vs.control; Homo_723,male,AD.vs.control; Homo_723,female,AD.vs.MCI; Homo_723,male,AD.vs.MCI; Homo_714,female,AD.vs.control; Homo_714,male,AD.vs.control; Homo_714,female,AD.vs.MCI; Homo_714,male,AD.vs.MCI; Homo_633,female,AD.vs.control; Homo_633,female,AD.vs.MCI; Homo_633,female,MCI.vs.control; Homo_633,male,MCI.vs.control; Emory,MCI,age; Emory,totalSample,immune-CD8_T; Emory,totalSample,immune-Gamma_delta; Emory,totalSample,immune-Th1; Emory,totalSample,immune-Tfh; Emory,totalSample,immune-CD8_naive; Emory,totalSample,immune-Exhausted; Homo_723,totalSample,immune-DC; Homo_723,totalSample,immune-Effector_memory; Homo_723,totalSample,immune-Th2; Homo_723,totalSample,immune-Macrophage; Homo_723,totalSample,immune-iTreg; Homo_714,totalSample,immune-Th2; Homo_714,totalSample,immune-CD8_T; Homo_714,totalSample,immune-Effector_memory; Homo_714,totalSample,immune-Tfh; Homo_714,totalSample,immune-Central_memory; Homo_633,totalSample,immune-Effector_memory; Homo_633,totalSample,immune-Central_memory; MCSA,totalSample,immune-Monocyte; MCSA,totalSample,immune-Neutrophil; MCSA,totalSample,immune-CD4_T; MCSA,totalSample,immune-CD8_T; MCSA,totalSample,immune-NKT; MCSA,totalSample,immune-CD4_naive; MCSA,totalSample,immune-Tr1; MCSA,totalSample,immune-nTreg; MCSA,totalSample,immune-Tfh; MCSA,totalSample,immune-CD8_naive; MCSA,totalSample,immune-MAIT; MCSA,totalSample,immune-Central_memory; MCSA,totalSample,immune-Effector_memory
MAST4	Homo_723,female,AD.vs.control; Homo_723,male,AD.vs.control; Homo_723,female,AD.vs.MCI; Homo_723,male,AD.vs.MCI; Homo_714,female,AD.vs.control; Homo_714,male,AD.vs.control; Homo_714,female,AD.vs.MCI; Homo_714,male,AD.vs.MCI; Homo_633,female,AD.vs.control; Homo_633,female,AD.vs.MCI; Homo_633,female,MCI.vs.control; Homo_633,male,MCI.vs.control; MCSA,totalSample,age; MCSA,control,age; ROSMAP,MCI,sample.age; Emory,totalSample,immune-Monocyte; Emory,totalSample,immune-Macrophage; Emory,totalSample,immune-NK; Emory,totalSample,immune-Neutrophil; Emory,totalSample,immune-CD4_T; Emory,totalSample,immune-CD8_T; Emory,totalSample,immune-Tr1; Emory,totalSample,immune-nTreg; Emory,totalSample,immune-iTreg; Emory,totalSample,immune-Th17; Emory,totalSample,immune-Tfh; Emory,totalSample,immune-CD8_naive; Emory,totalSample,immune-Central_memory; Emory,totalSample,immune-InfiltrationScore; Homo_723,totalSample,immune-Central_memory; Homo_723,totalSample,immune-Monocyte; Homo_723,totalSample,immune-CD4_naive; Homo_723,totalSample,immune-Th1; Homo_723,totalSample,immune-nTreg; Homo_723,totalSample,immune-DC; Homo_723,totalSample,immune-Neutrophil; Homo_723,totalSample,immune-CD4_T; Homo_723,totalSample,immune-Tr1; Homo_723,totalSample,immune-Th17; Homo_714,totalSample,immune-Th1; Homo_714,totalSample,immune-CD4_naive; Homo_714,totalSample,immune-NKT; Homo_714,totalSample,immune-nTreg; Homo_714,totalSample,immune-Monocyte; Homo_714,totalSample,immune-Central_memory; Homo_714,totalSample,immune-iTreg; Homo_714,totalSample,immune-CD4_T; Homo_714,totalSample,immune-InfiltrationScore; Homo_633,totalSample,immune-CD4_naive; Homo_633,totalSample,immune-Tfh; Homo_633,totalSample,immune-iTreg; Homo_633,totalSample,immune-Monocyte; Homo_633,totalSample,immune-Tr1; Homo_633,totalSample,immune-CD4_T; Homo_633,totalSample,immune-nTreg; Homo_633,totalSample,immune-NKT; Homo_633,totalSample,immune-Th1; Homo_633,totalSample,immune-Central_memory; Homo_633,totalSample,immune-InfiltrationScore; Homo_633,totalSample,immune-Effector_memory; MCSA,totalSample,immune-DC; MCSA,totalSample,immune-Macrophage; MCSA,totalSample,immune-Neutrophil; MCSA,totalSample,immune-CD4_T; MCSA,totalSample,immune-Gamma_delta; MCSA,totalSample,immune-CD4_naive; MCSA,totalSample,immune-Tr1; MCSA,totalSample,immune-nTreg; MCSA,totalSample,immune-iTreg; MCSA,totalSample,immune-Th17; MCSA,totalSample,immune-Tfh; MCSA,totalSample,immune-CD8_naive; MCSA,totalSample,immune-Exhausted; MCSA,totalSample,immune-MAIT; MCSA,totalSample,immune-[illegible]

SPINK2	Homo_723,female,AD.vs.control; Homo_723,male,AD.vs.control; Homo_723,female,AD.vs.MCI; Homo_723,male,AD.vs.MCI; Homo_714,female,AD.vs.control; Homo_714,male,AD.vs.control; Homo_714,female,AD.vs.MCI; Homo_714,male,AD.vs.MCI; Homo_633,female,AD.vs.control; Homo_633,female,AD.vs.MCI; Emory,totalSample,age; Emory,control,age; Emory,MCI,age; Emory,totalSample,immune-DC; Emory,totalSample,immune-Macrophage; Emory,totalSample,immune-Neutrophil; Emory,totalSample,immune-CD4_T; Emory,totalSample,immune-CD8_T; Emory,totalSample,immune-Tr1; Emory,totalSample,immune-iTreg; Emory,totalSample,immune-Tfh; Emory,totalSample,immune-CD8_naive; Emory,totalSample,immune-Exhausted; Emory,totalSample,immune-MAIT; Homo_723,totalSample,immune-CD8_T; Homo_723,totalSample,immune-Tfh; Homo_723,totalSample,immune-iTreg; Homo_723,totalSample,immune-Th2; Homo_723,totalSample,immune-MAIT; Homo_723,totalSample,immune-CD4_T; Homo_723,totalSample,immune-Central_memory; Homo_723,totalSample,immune-Macrophage; Homo_723,totalSample,immune-Tr1; Homo_723,totalSample,immune-Exhausted; Homo_723,totalSample,immune-CD4_naive; Homo_723,totalSample,immune-DC; Homo_723,totalSample,immune-Cytotoxic; Homo_723,totalSample,immune-nTreg; Homo_714,totalSample,immune-Tfh; Homo_714,totalSample,immune-Th2; Homo_714,totalSample,immune-CD8_T; Homo_714,totalSample,immune-CD4_T; Homo_714,totalSample,immune-Tr1; Homo_714,totalSample,immune-Central_memory; Homo_714,totalSample,immune-iTreg; Homo_714,totalSample,immune-MAIT; Homo_714,totalSample,immune-nTreg; Homo_714,totalSample,immune-Neutrophil; Homo_714,totalSample,immune-Macrophage; Homo_714,totalSample,immune-CD4_naive; Homo_714,totalSample,immune-Effector_memory; Homo_714,totalSample,immune-Exhausted; Homo_633,totalSample,immune-Tfh; Homo_633,totalSample,immune-Neutrophil; Homo_633,totalSample,immune-Central_memory; Homo_633,totalSample,immune-CD4_T; Homo_633,totalSample,immune-CD4_naive; Homo_633,totalSample,immune-Tr1; Homo_633,totalSample,immune-nTreg; Homo_633,totalSample,immune-MAIT; Homo_633,totalSample,immune-CD8_T; MCSA,totalSample,immune-DC; MCSA,totalSample,immune-Macrophage; MCSA,totalSample,immune-Neutrophil; MCSA,totalSample,immune-CD4_T; MCSA,totalSample,immune-CD8_T; [illegible]
ZNF385C	Homo_723,female,AD.vs.control; Homo_723,male,AD.vs.control; Homo_723,female,AD.vs.MCI; Homo_723,male,AD.vs.MCI; Homo_714,female,AD.vs.control; Homo_714,male,AD.vs.control; Homo_714,female,AD.vs.MCI; Homo_714,male,AD.vs.MCI; Homo_633,female,AD.vs.MCI; Homo_633,female,MCI.vs.control; Emory,MCI,age; MCSA,MCI,age; ROSMAP,MCI,last.visit.age; Homo_723,totalSample,immune-DC; MCSA,totalSample,immune-Monocyte; MCSA,totalSample,immune-Th17
KIF11	Homo_723,female,AD.vs.control; Homo_723,male,AD.vs.control; Homo_723,female,AD.vs.MCI; Homo_723,male,AD.vs.MCI; Homo_723,female,MCI.vs.control; Homo_723,male,MCI.vs.control; Homo_714,female,AD.vs.control; Homo_714,male,AD.vs.control; Homo_714,female,AD.vs.MCI; Homo_714,male,AD.vs.MCI; Homo_714,female,MCI.vs.control; Homo_714,male,MCI.vs.control; Homo_633,female,AD.vs.control; Homo_633,female,AD.vs.MCI; Emory,MCI,age; MCSA,MCI,age; ROSMAP,MCI,last.visit.age; Emory,totalSample,immune-CD4_T; Emory,totalSample,immune-Tr1; Emory,totalSample,immune-iTreg; Emory,totalSample,immune-InfiltrationScore; Homo_723,totalSample,immune-Exhausted; Homo_723,totalSample,immune-Th17; Homo_723,totalSample,immune-Cytotoxic; Homo_723,totalSample,immune-CD4_naive; Homo_723,totalSample,immune-CD8_T; Homo_723,totalSample,immune-CD4_T; Homo_723,totalSample,immune-Monocyte; Homo_723,totalSample,immune-Tr1; Homo_723,totalSample,immune-Tfh; Homo_723,totalSample,immune-InfiltrationScore; Homo_723,totalSample,immune-Th2; Homo_723,totalSample,immune-Th1; Homo_714,totalSample,immune-Monocyte; Homo_714,totalSample,immune-CD4_naive; Homo_714,totalSample,immune-Exhausted; Homo_714,totalSample,immune-InfiltrationScore; Homo_714,totalSample,immune-Th17; Homo_714,totalSample,immune-Tfh; Homo_714,totalSample,immune-Central_memory; Homo_633,totalSample,immune-Tfh; Homo_633,totalSample,immune-Th2; Homo_633,totalSample,immune-Monocyte; Homo_633,totalSample,immune-CD4_T; Homo_633,totalSample,immune-Effector_memory; Homo_633,totalSample,immune-InfiltrationScore; Homo_633,totalSample,immune-CD4_naive; Homo_633,totalSample,immune-Central_memory; Homo_633,totalSample,immune-Tr1; Homo_633,totalSample,immune-Gamma_delta; Homo_633,totalSample,immune-Exhausted; MCSA,totalSample,immune-DC; MCSA,totalSample,immune-Monocyte; MCSA,totalSample,immune-CD4_T; MCSA,totalSample,immune-CD4_naive; MCSA,totalSample,immune-Tr1; MCSA,totalSample,immune-nTreg; MCSA,totalSample,immune-iTreg; MCSA,totalSample,immune-Tfh; MCSA,totalSample,immune-CD8_naive; MCSA,totalSample,immune-Cytotoxic; MCSA,totalSample,immune-Exhausted; MCSA,totalSample,immune-MAIT; MCSA,totalSample,immune-Central_memory; ROSMAP,totalSample,immune-InfiltrationScore
KIAA0895	Homo_723,female,AD.vs.control; Homo_723,female,AD.vs.MCI; Homo_723,female,MCI.vs.control; Homo_714,female,AD.vs.control; Homo_714,female,AD.vs.MCI; Homo_633,female,AD.vs.control; Homo_633,female,AD.vs.MCI; ROSMAP,MCI,last.visit.age; Emory,totalSample,immune-Gamma_delta; Emory,totalSample,immune-Tr1; Emory,totalSample,immune-iTreg; Homo_723,totalSample,immune-Th2; Homo_723,totalSample,immune-CD4_T; Homo_723,totalSample,immune-Tr1; Homo_723,totalSample,immune-iTreg; Homo_723,totalSample,immune-Tfh; Homo_723,totalSample,immune-CD4_naive; Homo_723,totalSample,immune-Cytotoxic; Homo_723,totalSample,immune-Th17; Homo_723,totalSample,immune-Exhausted; Homo_723,totalSample,immune-Gamma_delta; Homo_723,totalSample,immune-Central_memory; Homo_723,totalSample,immune-nTreg; Homo_714,totalSample,immune-Th2; Homo_714,totalSample,immune-Tfh; Homo_714,totalSample,immune-CD4_T; Homo_714,totalSample,immune-Tr1; Homo_714,totalSample,immune-CD4_naive; Homo_714,totalSample,immune-Gamma_delta; Homo_714,totalSample,immune-iTreg; Homo_714,totalSample,immune-Th17; Homo_714,totalSample,immune-Exhausted; Homo_714,totalSample,immune-Central_memory; Homo_633,totalSample,immune-Tfh; Homo_633,totalSample,immune-Th2; Homo_633,totalSample,immune-CD4_T; Homo_633,totalSample,immune-Tr1; Homo_633,totalSample,immune-Gamma_delta; Homo_633,totalSample,immune-NKT; Homo_633,totalSample,immune-Exhausted; Homo_633,totalSample,immune-CD4_naive; Homo_633,totalSample,immune-Th17; Homo_633,totalSample,immune-iTreg; Homo_633,totalSample,immune-DC; Homo_633,totalSample,immune-Central_memory; Homo_633,totalSample,immune-Cytotoxic; MCSA,totalSample,immune-DC; MCSA,totalSample,immune-Macrophage; MCSA,totalSample,immune-CD4_T; MCSA,totalSample,immune-CD8_T; MCSA,totalSample,immune-CD4_naive; MCSA,totalSample,immune-Tr1; MCSA,totalSample,immune-nTreg; MCSA,totalSample,immune-iTreg; MCSA,totalSample,immune-Th17; MCSA,totalSample,immune-Tfh; MCSA,totalSample,immune-CD8_naive; MCSA,totalSample,immune-Cytotoxic; MCSA,totalSample,immune-Exhausted; MCSA,totalSample,immune-Central_memory; ROSMAP,male-includeAge90+,survival
MAPRE2	Homo_723,female,AD.vs.control; Homo_723,male,AD.vs.control; Homo_723,female,AD.vs.MCI; Homo_723,male,AD.vs.MCI; Homo_714,female,AD.vs.control; Homo_714,male,AD.vs.control; Homo_714,female,AD.vs.MCI; Homo_714,male,AD.vs.MCI; Homo_633,female,AD.vs.control; Homo_633,female,AD.vs.MCI; ROSMAP,female,AD.vs.control; ROSMAP,male,AD.vs.control; Emory,totalSample,immune-Macrophage; Emory,totalSample,immune-NK; Emory,totalSample,immune-Neutrophil; Emory,totalSample,immune-CD4_T; Emory,totalSample,immune-CD8_T; Emory,totalSample,immune-Tr1; Emory,totalSample,immune-iTreg; Emory,totalSample,immune-Exhausted; Emory,totalSample,immune-Effector_memory; Emory,totalSample,immune-InfiltrationScore; Homo_723,totalSample,immune-CD4_naive; Homo_723,totalSample,immune-InfiltrationScore; Homo_723,totalSample,immune-Central_memory; Homo_723,totalSample,immune-DC; Homo_723,totalSample,immune-nTreg; Homo_723,totalSample,immune-Monocyte; Homo_723,totalSample,immune-CD4_T; Homo_723,totalSample,immune-Neutrophil; Homo_723,totalSample,immune-Tfh; Homo_723,totalSample,immune-NKT; Homo_723,totalSample,immune-NK; Homo_723,totalSample,immune-Tr1; Homo_723,totalSample,immune-Th1; Homo_723,totalSample,immune-Th17; Homo_714,totalSample,immune-InfiltrationScore; Homo_714,totalSample,immune-CD4_naive; Homo_714,totalSample,immune-NKT; Homo_714,totalSample,immune-Central_memory; Homo_714,totalSample,immune-nTreg; Homo_714,totalSample,immune-CD4_T; Homo_714,totalSample,immune-NK; Homo_633,totalSample,immune-InfiltrationScore; Homo_633,totalSample,immune-Tfh; Homo_633,totalSample,immune-CD4_naive; Homo_633,totalSample,immune-NKT; Homo_633,totalSample,immune-Effector_memory; Homo_633,totalSample,immune-CD4_T; Homo_633,totalSample,immune-Tr1; Homo_633,totalSample,immune-iTreg; Homo_633,totalSample,immune-Central_memory; Homo_633,totalSample,immune-nTreg; Homo_633,totalSample,immune-Monocyte; MCSA,totalSample,immune-CD4_T; MCSA,totalSample,immune-NKT; MCSA,totalSample,immune-CD4_naive; MCSA,totalSample,immune-Tr1; MCSA,totalSample,immune-nTreg; MCSA,totalSample,immune-iTreg; MCSA,totalSample,immune-Th17; MCSA,totalSample,immune-Tfh; MCSA,totalSample,immune-CD8_naive; MCSA,totalSample,immune-Exhausted; MCSA,totalSample,immune-MAIT; MCSA,totalSample,immune-Central_memory; MCSA,totalSample,immune-Effector_memory; MCSA,totalSample,immune-InfiltrationScore; SRP223445,totalSample,immune-CD4_naive; SRP223445,totalSample,immune-Exhausted
SPX	Homo_723,female,AD.vs.control; Homo_723,male,AD.vs.control; Homo_723,female,AD.vs.MCI; Homo_723,male,AD.vs.MCI; Homo_723,female,MCI.vs.control; Homo_723,male,MCI.vs.control; Homo_714,female,AD.vs.control; Homo_714,male,AD.vs.control; Homo_714,female,AD.vs.MCI; Homo_714,male,AD.vs.MCI; Homo_714,female,MCI.vs.control; Homo_714,male,MCI.vs.control; Homo_633,female,AD.vs.control; Homo_633,female,AD.vs.MCI; Emory,MCI,age; Homo_723,totalSample,immune-Cytotoxic; Homo_723,totalSample,immune-Gamma_delta; Homo_723,totalSample,immune-CD8_T; Homo_723,totalSample,immune-Th17; Homo_723,totalSample,immune-DC; Homo_714,totalSample,immune-Exhausted; Homo_714,totalSample,immune-CD8_T; Homo_714,totalSample,immune-NKT; Homo_633,totalSample,immune-Gamma_delta; Homo_633,totalSample,immune-NKT; MCSA,totalSample,immune-Monocyte; MCSA,totalSample,immune-CD8_T; MCSA,totalSample,immune-Tr1; MCSA,totalSample,immune-iTreg; MCSA,totalSample,immune-Cytotoxic; MCSA,totalSample,immune-Exhausted; ROSMAP,totalSample,immune-InfiltrationScore; SRP223445,totalSample,immune-CD4_naive; SRP223445,totalSample,immune-Exhausted
SAP30BP	Homo_723,female,AD.vs.control; Homo_723,male,AD.vs.control; Homo_723,female,AD.vs.MCI; Homo_723,male,AD.vs.MCI; Homo_714,female,AD.vs.control; Homo_714,male,AD.vs.control; Homo_714,female,AD.vs.MCI; Homo_714,male,AD.vs.MCI; Homo_633,female,AD.vs.control; Homo_633,female,AD.vs.MCI; Emory,MCI,age; MCSA,totalSample,age; MCSA,control,age; MCSA,MCI,age; Emory,totalSample,immune-CD4_T; Emory,totalSample,immune-Tr1; Emory,totalSample,immune-iTreg; Emory,totalSample,immune-InfiltrationScore; Homo_723,totalSample,immune-Effector_memory; Homo_723,totalSample,immune-InfiltrationScore; Homo_723,totalSample,immune-Th2; Homo_723,totalSample,immune-Tfh; Homo_723,totalSample,immune-Bcell; Homo_723,totalSample,immune-iTreg; Homo_723,totalSample,immune-Monocyte; Homo_723,totalSample,immune-Th1; Homo_714,totalSample,immune-Th2; Homo_714,totalSample,immune-Effector_memory; Homo_714,totalSample,immune-Tfh; Homo_714,totalSample,immune-InfiltrationScore; Homo_714,totalSample,immune-Th1; Homo_714,totalSample,immune-nTreg; Homo_714,totalSample,immune-Central_memory; Homo_633,totalSample,immune-InfiltrationScore; Homo_633,totalSample,immune-Effector_memory; Homo_633,totalSample,immune-Th2; Homo_633,totalSample,immune-Tfh; Homo_633,totalSample,immune-Exhausted; Homo_633,totalSample,immune-nTreg; Homo_633,totalSample,immune-Central_memory; Homo_633,totalSample,immune-Th17; MCSA,totalSample,immune-DC; MCSA,totalSample,immune-Monocyte; MCSA,totalSample,immune-CD4_T; MCSA,totalSample,immune-CD8_T; MCSA,totalSample,immune-NKT; MCSA,totalSample,immune-Gamma_delta; MCSA,totalSample,immune-CD4_naive; MCSA,totalSample,immune-Tr1; MCSA,totalSample,immune-nTreg; MCSA,totalSample,immune-Th2; MCSA,totalSample,immune-Th17; MCSA,totalSample,immune-Tfh; MCSA,totalSample,immune-Cytotoxic; MCSA,totalSample,immune-Central_memory; MCSA,totalSample,immune-Effector_memory; MCSA,totalSample,immune-InfiltrationScore; SRP223445,totalSample,immune-CD4_naive; SRP223445,totalSample,immune-Th17; SRP223445,totalSample,immune-Exhausted; SRP223445,totalSample,immune-InfiltrationScore; ROSMAP,female-excludeAge90+,survival; ROSMAP,female-includeAge90+,survival; ROSMAP,male and female-excludeAge90+,survival; ROSMAP,male and female-includeAge90+,survival
FAM189B	Homo_723,female,AD.vs.control; Homo_723,male,AD.vs.control; Homo_723,female,AD.vs.MCI; Homo_723,male,AD.vs.MCI; Homo_714,female,AD.vs.control; Homo_714,male,AD.vs.control; Homo_714,female,AD.vs.MCI; Homo_714,male,AD.vs.MCI; Homo_633,female,AD.vs.control; Homo_633,female,MCI.vs.control; ROSMAP,female,AD.vs.control; MCSA,totalSample,age; MCSA,control,age; MCSA,MCI,age; ROSMAP,AD,last.visit.age; Emory,totalSample,immune-InfiltrationScore; Homo_723,totalSample,immune-DC; Homo_723,totalSample,immune-Monocyte; Homo_723,totalSample,immune-Neutrophil; Homo_714,totalSample,immune-DC; Homo_714,totalSample,immune-Th2; Homo_714,totalSample,immune-Monocyte; Homo_633,totalSample,immune-DC; Homo_633,totalSample,immune-Monocyte; MCSA,totalSample,immune-DC; MCSA,totalSample,immune-Monocyte; MCSA,totalSample,immune-CD8_T; MCSA,totalSample,immune-NKT; MCSA,totalSample,immune-iTreg; MCSA,totalSample,immune-Th17; MCSA,totalSample,immune-CD8_naive; MCSA,totalSample,immune-Cytotoxic; MCSA,totalSample,immune-Exhausted; MCSA,totalSample,immune-MAIT; MCSA,totalSample,immune-InfiltrationScore; ROSMAP,totalSample,immune-Effector_memory; ROSMAP,male-includeAge90+,survival
SLC25A20	Homo_723,female,AD.vs.control; Homo_723,male,AD.vs.control; Homo_723,female,AD.vs.MCI; Homo_723,male,AD.vs.MCI; Homo_714,female,AD.vs.control; Homo_714,male,AD.vs.control; Homo_714,female,AD.vs.MCI; Homo_714,male,AD.vs.MCI; Homo_633,female,AD.vs.control; Homo_633,female,AD.vs.MCI; ROSMAP,female,AD.vs.control; MCSA,MCI,age; Emory,totalSample,immune-CD4_T; Emory,totalSample,immune-Gamma_delta; Emory,totalSample,immune-Tr1; Emory,totalSample,immune-iTreg; Homo_723,totalSample,immune-Th2; Homo_723,totalSample,immune-Tfh; Homo_723,totalSample,immune-InfiltrationScore; Homo_723,totalSample,immune-Monocyte; Homo_723,totalSample,immune-Effector_memory; Homo_723,totalSample,immune-iTreg; Homo_723,totalSample,immune-DC; Homo_723,totalSample,immune-Bcell; Homo_723,totalSample,immune-CD4_T; Homo_723,totalSample,immune-Tr1; Homo_723,totalSample,immune-CD4_naive; Homo_714,totalSample,immune-Th2; Homo_714,totalSample,immune-Tfh; Homo_714,totalSample,immune-Effector_memory; Homo_714,totalSample,immune-InfiltrationScore; Homo_714,totalSample,immune-Monocyte; Homo_714,totalSample,immune-CD4_T; Homo_714,totalSample,immune-Tr1; Homo_633,totalSample,immune-Th2; Homo_633,totalSample,immune-Monocyte; Homo_633,totalSample,immune-Tfh; Homo_633,totalSample,immune-InfiltrationScore; Homo_633,totalSample,immune-Effector_memory; Homo_633,totalSample,immune-CD4_T; Homo_633,totalSample,immune-CD4_naive; Homo_633,totalSample,immune-Bcell; Homo_633,totalSample,immune-Central_memory; Homo_633,totalSample,immune-Exhausted; MCSA,totalSample,immune-DC; MCSA,totalSample,immune-CD4_T; MCSA,totalSample,immune-CD4_naive; MCSA,totalSample,immune-Tr1; MCSA,totalSample,immune-nTreg; MCSA,totalSample,immune-iTreg; MCSA,totalSample,immune-Th17; MCSA,totalSample,immune-Tfh; MCSA,totalSample,immune-Cytotoxic; MCSA,totalSample,immune-Exhausted; MCSA,totalSample,immune-MAIT; MCSA,totalSample,immune-Central_memory; ROSMAP,totalSample,immune-Th17;
VNN3	Homo_723,female,AD.vs.control; Homo_723,male,AD.vs.control; Homo_723,female,AD.vs.MCI; Homo_723,male,AD.vs.MCI; Homo_723,female,MCI.vs.control; Homo_714,male,MCI.vs.control; Homo_633,female,AD.vs.control; Homo_633,female,AD.vs.MCI; Homo_633,female,MCI.vs.control; Homo_633,male,MCI.vs.control; Homo_633,control,male.vs.female; Emory,totalSample,age; Emory,MCI,age; MCSA,MCI,age; Emory,totalSample,immune-Monocyte; Emory,totalSample,immune-Neutrophil; Emory,totalSample,immune-CD8_T; Emory,totalSample,immune-Gamma_delta; Emory,totalSample,immune-Th17; Emory,totalSample,immune-Tfh; Emory,totalSample,immune-CD8_naive; Emory,totalSample,immune-Cytotoxic; Emory,totalSample,immune-Exhausted; Homo_723,totalSample,immune-Monocyte; Homo_723,totalSample,immune-Neutrophil; Homo_723,totalSample,immune-DC; Homo_723,totalSample,immune-Th2; Homo_723,totalSample,immune-Bcell; Homo_723,totalSample,immune-Gamma_delta; Homo_723,totalSample,immune-Exhausted; Homo_723,totalSample,immune-Th1; Homo_714,totalSample,immune-Neutrophil; Homo_714,totalSample,immune-CD8_T; Homo_714,totalSample,immune-Exhausted; Homo_714,totalSample,immune-MAIT; Homo_633,totalSample,immune-Neutrophil; Homo_633,totalSample,immune-CD8_T; MCSA,totalSample,immune-Neutrophil; MCSA,totalSample,immune-CD8_T; MCSA,totalSample,immune-Gamma_delta; MCSA,totalSample,immune-Th1; MCSA,totalSample,immune-Th17; MCSA,totalSample,immune-Cytotoxic; MCSA,totalSample,immune-Exhausted; MCSA,totalSample,immune-Effector_memory; SRP223445,totalSample,immune-Neutrophil; SRP223445,totalSample,immune-Gamma_delta; SRP223445,totalSample,immune-CD4_naive;
LHFPL6	Homo_723,female,AD.vs.control; Homo_723,male,AD.vs.control; Homo_723,female,AD.vs.MCI; Homo_723,male,AD.vs.MCI; Homo_723,female,MCI.vs.control; Homo_714,female,AD.vs.control; Homo_714,male,AD.vs.control; Homo_714,female,AD.vs.MCI; Homo_714,male,AD.vs.MCI; Homo_714,female,MCI.vs.control; Homo_633,female,AD.vs.control; Homo_633,female,AD.vs.MCI; ROSMAP,female,AD.vs.control; ROSMAP,male,AD.vs.control; Emory,totalSample,immune-Th17; Homo_723,totalSample,immune-DC; Homo_723,totalSample,immune-Exhausted; Homo_723,totalSample,immune-CD8_T; Homo_714,totalSample,immune-Exhausted; MCSA,totalSample,immune-iTreg; MCSA,totalSample,immune-Th1; MCSA,totalSample,immune-Exhausted; ROSMAP,male-excludeAge90+,survival; ROSMAP,male-includeAge90+,survival
LAMC1	Homo_723,female,AD.vs.control; Homo_723,male,AD.vs.control; Homo_723,female,AD.vs.MCI; Homo_723,male,AD.vs.MCI; Homo_723,female,MCI.vs.control; Homo_723,male,MCI.vs.control; Homo_714,female,AD.vs.control; Homo_714,male,AD.vs.control; Homo_714,female,AD.vs.MCI; Homo_714,male,AD.vs.MCI; Homo_714,female,MCI.vs.control; Homo_714,male,MCI.vs.control; Homo_633,female,AD.vs.control; Homo_633,female,AD.vs.MCI; Homo_633,female,MCI.vs.control; Homo_633,male,MCI.vs.control; MCSA,totalSample,age; MCSA,control,age; ROSMAP,MCI,last.visit.age; ROSMAP,AD,MMSE; Emory,totalSample,immune-B_cell; Emory,totalSample,immune-Gamma_delta; Emory,totalSample,immune-InfiltrationScore; Homo_723,totalSample,immune-Th17; Homo_723,totalSample,immune-Exhausted; Homo_723,totalSample,immune-CD4_naive; Homo_723,totalSample,immune-CD4_T; Homo_723,totalSample,immune-Cytotoxic; Homo_723,totalSample,immune-nTreg; Homo_723,totalSample,immune-Th2; Homo_723,totalSample,immune-Central_memory; Homo_723,totalSample,immune-Tfh; Homo_723,totalSample,immune-CD8_T; Homo_723,totalSample,immune-Tr1; Homo_723,totalSample,immune-Th1; Homo_723,totalSample,immune-Gamma_delta; Homo_714,totalSample,immune-Th17; Homo_714,totalSample,immune-Exhausted; Homo_714,totalSample,immune-CD4_naive; Homo_714,totalSample,immune-Gamma_delta; Homo_714,totalSample,immune-CD4_T; Homo_714,totalSample,immune-Central_memory; Homo_714,totalSample,immune-Tfh; Homo_714,totalSample,immune-nTreg; Homo_714,totalSample,immune-Cytotoxic; Homo_633,totalSample,immune-Th2; Homo_633,totalSample,immune-Tfh; Homo_633,totalSample,immune-CD4_T; Homo_633,totalSample,immune-Gamma_delta; Homo_633,totalSample,immune-Central_memory; Homo_633,totalSample,immune-Exhausted; Homo_633,totalSample,immune-Th17; Homo_633,totalSample,immune-CD4_naive; Homo_633,totalSample,immune-Effector_memory; Homo_633,totalSample,immune-nTreg; Homo_633,totalSample,immune-Tr1; Homo_633,totalSample,immune-iTreg; MCSA,totalSample,immune-Bcell; MCSA,totalSample,immune-Monocyte; MCSA,totalSample,immune-CD4_T; MCSA,totalSample,immune-Gamma_delta; MCSA,totalSample,immune-CD4_naive; MCSA,totalSample,immune-Tr1; MCSA,totalSample,immune-nTreg; MCSA,totalSample,immune-iTreg; MCSA,totalSample,immune-Th17; [illegible]
OR6N1	Homo_723,female,AD.vs.control; Homo_723,male,AD.vs.control; Homo_723,female,AD.vs.MCI; Homo_723,male,AD.vs.MCI; Homo_723,female,MCI.vs.control; Homo_723,male,MCI.vs.control; Homo_714,female,AD.vs.control; Homo_714,male,AD.vs.control; Homo_714,female,AD.vs.MCI; Homo_714,male,AD.vs.MCI; Homo_714,female,MCI.vs.control; Homo_714,male,MCI.vs.control; Homo_633,female,AD.vs.control; Homo_633,female,AD.vs.MCI; Homo_633,female,MCI.vs.control; Homo_633,male,MCI.vs.control; Emory,MCI,age; MCSA,MCI,age; Emory,totalSample,immune-Macrophage; Emory,totalSample,immune-NK; Emory,totalSample,immune-Neutrophil; Emory,totalSample,immune-CD8_T; Emory,totalSample,immune-Gamma_delta; Emory,totalSample,immune-Th17; Emory,totalSample,immune-Tfh; Emory,totalSample,immune-CD8_naive; Emory,totalSample,immune-Cytotoxic; Emory,totalSample,immune-Exhausted; Emory,totalSample,immune-InfiltrationScore; Homo_723,totalSample,immune-Gamma_delta; Homo_723,totalSample,immune-iTreg; Homo_723,totalSample,immune-Neutrophil; Homo_723,totalSample,immune-CD8_naive; Homo_714,totalSample,immune-Gamma_delta; Homo_714,totalSample,immune-Tfh; Homo_714,totalSample,immune-iTreg; Homo_633,totalSample,immune-NKT; Homo_633,totalSample,immune-CD8_T; Homo_633,totalSample,immune-Gamma_delta; Homo_633,totalSample,immune-Cytotoxic; Homo_633,totalSample,immune-Neutrophil; MCSA,totalSample,immune-DC; MCSA,totalSample,immune-Neutrophil; MCSA,totalSample,immune-CD4_T; MCSA,totalSample,immune-CD8_T; MCSA,totalSample,immune-Gamma_delta; MCSA,totalSample,immune-CD4_naive; MCSA,totalSample,immune-Tr1; MCSA,totalSample,immune-nTreg; MCSA,totalSample,immune-iTreg; MCSA,totalSample,immune-Th1; MCSA,totalSample,immune-Th17; MCSA,totalSample,immune-Cytotoxic; MCSA,totalSample,immune-Exhausted; ROSMAP,male-excludeAge90+,survival
XRCC6	Homo_723,female,AD.vs.control; Homo_723,male,AD.vs.control; Homo_723,female,AD.vs.MCI; Homo_723,male,AD.vs.MCI; Homo_723,female,MCI.vs.control; Homo_723,male,MCI.vs.control; Homo_723,control,male.vs.female; Homo_723,MCI,male.vs.female; Homo_714,female,AD.vs.control; Homo_714,male,AD.vs.control; Homo_714,female,AD.vs.MCI; Homo_714,male,AD.vs.MCI; Homo_714,female,MCI.vs.control; Homo_714,male,MCI.vs.control; Homo_714,control,male.vs.female; Homo_714,MCI,male.vs.female; Homo_633,male,MCI.vs.control; Homo_633,control,male.vs.female; Homo_633,MCI,male.vs.female; ROSMAP,female,AD.vs.control; ROSMAP,female,MCI.vs.control; MCSA,totalSample,age; MCSA,control,age; ROSMAP,MCI,last.visit.age; ROSMAP,MCI,sample.age; Emory,totalSample,immune-Monocyte; Emory,totalSample,immune-Macrophage; Emory,totalSample,immune-NK; Emory,totalSample,immune-Neutrophil; Emory,totalSample,immune-CD4_T; Emory,totalSample,immune-CD8_T; Emory,totalSample,immune-Tr1; Emory,totalSample,immune-nTreg; Emory,totalSample,immune-iTreg; Emory,totalSample,immune-Exhausted; Emory,totalSample,immune-MAIT; Emory,totalSample,immune-Effector_memory; Emory,totalSample,immune-InfiltrationScore; Homo_723,totalSample,immune-Monocyte; Homo_723,totalSample,immune-CD4_naive; Homo_723,totalSample,immune-nTreg; Homo_723,totalSample,immune-Central_memory; Homo_723,totalSample,immune-DC; Homo_723,totalSample,immune-Th1; Homo_723,totalSample,immune-CD4_T; Homo_723,totalSample,immune-InfiltrationScore; Homo_723,totalSample,immune-Neutrophil; Homo_714,totalSample,immune-Monocyte; Homo_714,totalSample,immune-CD4_naive; Homo_714,totalSample,immune-Th1; Homo_714,totalSample,immune-InfiltrationScore; Homo_714,totalSample,immune-NKT; Homo_714,totalSample,immune-nTreg; Homo_714,totalSample,immune-iTreg; Homo_633,totalSample,immune-Monocyte; Homo_633,totalSample,immune-CD4_naive; Homo_633,totalSample,immune-InfiltrationScore; Homo_633,totalSample,immune-nTreg; Homo_633,totalSample,immune-Th1; Homo_633,totalSample,immune-iTreg; Homo_633,totalSample,immune-NKT; Homo_633,totalSample,immune-Tfh; Homo_633,totalSample,immune-CD4_T; MCSA,totalSample,immune-Monocyte; MCSA,totalSample,immune-Macrophage; MCSA,totalSample,immune-Neutrophil; MCSA,totalSample,immune-CD4_T; MCSA,totalSample,immune-CD4_naive; MCSA,totalSample,immune-Tr1; MCSA,totalSample,immune-nTreg; MCSA,totalSample,immune-iTreg; MCSA,totalSample,immune-Th17; MCSA,totalSample,immune-Tfh; MCSA,totalSample,immune-CD8_naive; MCSA,totalSample,immune-MAIT; MCSA,totalSample,immune-Central_memory; MCSA,totalSample,immune-Effector_memory; MCSA,totalSample,immune-InfiltrationScore; ROSMAP,totalSample,immune-CD8_naive; SRP223445,totalSample,immune-NK; SRP223445,totalSample,immune-Neutrophil; SRP223445,totalSample,immune-CD4_naive; SRP223445,totalSample,immune-Th17; SRP223445,totalSample,immune-Exhausted; SRP223445,totalSample,immune-InfiltrationScore; [illegible]

MUL1	Homo_723,female,AD.vs.control; Homo_723,male,AD.vs.control; Homo_723,female,AD.vs.MCI; Homo_723,male,AD.vs.MCI; Homo_714,female,AD.vs.control; Homo_714,male,AD.vs.control; Homo_714,female,AD.vs.MCI; Homo_714,male,AD.vs.MCI; Homo_633,female,AD.vs.control; Homo_633,female,AD.vs.MCI; ROSMAP,female,AD.vs.control; Emory,MCI,age; MCSA,totalSample,age; MCSA,control,age; Emory,totalSample,immune-Tr1; Emory,totalSample,immune-iTreg; Emory,totalSample,immune-CD8_naive; Emory,totalSample,immune-InfiltrationScore; Homo_723,totalSample,immune-InfiltrationScore; Homo_723,totalSample,immune-CD8_T; Homo_723,totalSample,immune-Monocyte; Homo_723,totalSample,immune-iTreg; Homo_723,totalSample,immune-Exhausted; Homo_723,totalSample,immune-Cytotoxic; Homo_723,totalSample,immune-Effector_memory; Homo_723,totalSample,immune-Bcell; Homo_723,totalSample,immune-Tfh; Homo_714,totalSample,immune-InfiltrationScore; Homo_714,totalSample,immune-Effector_memory; Homo_714,totalSample,immune-Monocyte; Homo_714,totalSample,immune-Tfh; Homo_714,totalSample,immune-nTreg; Homo_714,totalSample,immune-DC; Homo_633,totalSample,immune-InfiltrationScore; Homo_633,totalSample,immune-DC; Homo_633,totalSample,immune-Monocyte; Homo_633,totalSample,immune-nTreg; Homo_633,totalSample,immune-Effector_memory; MCSA,totalSample,immune-DC; MCSA,totalSample,immune-Monocyte; MCSA,totalSample,immune-CD8_T; MCSA,totalSample,immune-NKT; MCSA,totalSample,immune-CD4_naive; MCSA,totalSample,immune-nTreg; MCSA,totalSample,immune-Th2; MCSA,totalSample,immune-Tfh; MCSA,totalSample,immune-Cytotoxic; MCSA,totalSample,immune-Central_memory; MCSA,totalSample,immune-
PTPN13	Homo_723,female,AD.vs.control; Homo_723,male,AD.vs.control; Homo_723,female,AD.vs.MCI; Homo_723,male,AD.vs.MCI; Homo_723,female,MCI.vs.control; Homo_723,male,MCI.vs.control; Homo_714,female,AD.vs.control; Homo_714,male,AD.vs.control; Homo_714,female,AD.vs.MCI; Homo_714,male,AD.vs.MCI; Homo_633,female,AD.vs.control; Homo_633,female,AD.vs.MCI; ACOM,totalSample,age; Emory,totalSample,age; Emory,control,age; Emory,MCI,age; MCSA,totalSample,age; MCSA,control,age; ROSMAP,AD,last.visit.age; ROSMAP,MCI,last.visit.age; Emory,totalSample,immune-Macrophage; Emory,totalSample,immune-CD4_T; Emory,totalSample,immune-Tr1; Emory,totalSample,immune-iTreg; Emory,totalSample,immune-Th17; Emory,totalSample,immune-InfiltrationScore; Homo_723,totalSample,immune-Th17; Homo_723,totalSample,immune-Exhausted; Homo_723,totalSample,immune-CD4_naive; Homo_723,totalSample,immune-CD4_T; Homo_723,totalSample,immune-Cytotoxic; Homo_723,totalSample,immune-Tfh; Homo_723,totalSample,immune-Tr1; Homo_723,totalSample,immune-nTreg; Homo_723,totalSample,immune-Th2; Homo_723,totalSample,immune-Central_memory; Homo_723,totalSample,immune-Th1; Homo_723,totalSample,immune-InfiltrationScore; Homo_723,totalSample,immune-iTreg; Homo_723,totalSample,immune-CD8_T; Homo_723,totalSample,immune-Gamma_delta; Homo_714,totalSample,immune-Th17; Homo_714,totalSample,immune-CD4_naive; Homo_714,totalSample,immune-Exhausted; Homo_714,totalSample,immune-CD4_T; Homo_714,totalSample,immune-Tfh; Homo_714,totalSample,immune-InfiltrationScore; Homo_714,totalSample,immune-Central_memory; Homo_714,totalSample,immune-NKT; Homo_714,totalSample,immune-Tr1; Homo_714,totalSample,immune-Th1; Homo_714,totalSample,immune-iTreg; Homo_714,totalSample,immune-Gamma_delta; Homo_714,totalSample,immune-Cytotoxic; Homo_714,totalSample,immune-nTreg; Homo_714,totalSample,immune-Th2; Homo_714,totalSample,immune-Effector_memory; Homo_633,totalSample,immune-Tfh; Homo_633,totalSample,immune-CD4_T; Homo_633,totalSample,immune-Th2; Homo_633,totalSample,immune-Th17; Homo_633,totalSample,immune-CD4_naive; Homo_633,totalSample,immune-Tr1; Homo_633,totalSample,immune-Central_memory; Homo_633,totalSample,immune-Effector_memory; Homo_633,totalSample,immune-iTreg; Homo_633,totalSample,immune-Exhausted; Homo_633,totalSample,immune-Gamma_delta; Homo_633,totalSample,immune-InfiltrationScore; Homo_633,totalSample,immune-nTreg; Homo_633,totalSample,immune-NKT; MCSA,totalSample,immune-DC; MCSA,totalSample,immune-CD4_T; MCSA,totalSample,immune-CD8_T; MCSA,totalSample,immune-Gamma_delta; MCSA,totalSample,immune-CD4_naive; MCSA,totalSample,immune-Tr1; MCSA,totalSample,immune-nTreg; MCSA,totalSample,immune-iTreg; MCSA,totalSample,immune-Th17; MCSA,totalSample,immune-Tfh; MCSA,totalSample,immune-CD8_naive; MCSA,totalSample,immune-Cytotoxic; MCSA,totalSample,immune-Exhausted; MCSA,totalSample,immune-MAIT; MCSA,totalSample,immune- [illegible]
DAAM1	Homo_723,female,AD.vs.control; Homo_723,male,AD.vs.control; Homo_723,female,AD.vs.MCI; Homo_723,male,AD.vs.MCI; Homo_723,female,MCI.vs.control; Homo_723,male,MCI.vs.control; Homo_714,female,AD.vs.control; Homo_714,male,AD.vs.control; Homo_714,female,AD.vs.MCI; Homo_714,male,AD.vs.MCI; Homo_714,female,MCI.vs.control; Homo_714,male,MCI.vs.control; Homo_633,female,AD.vs.control; Homo_633,female,AD.vs.MCI; Homo_633,female,MCI.vs.control; MCSA,MCI,age; Emory,totalSample,immune-Macrophage; Emory,totalSample,immune-NK; Emory,totalSample,immune-Neutrophil; Emory,totalSample,immune-CD4_T; Emory,totalSample,immune-CD8_T; Emory,totalSample,immune-Tr1; Emory,totalSample,immune-iTreg; Emory,totalSample,immune-Tfh; Emory,totalSample,immune-InfiltrationScore; Homo_723,totalSample,immune-Th1; Homo_723,totalSample,immune-Central_memory; Homo_723,totalSample,immune-CD4_naive; Homo_723,totalSample,immune-Cytotoxic; Homo_723,totalSample,immune-DC; Homo_723,totalSample,immune-Monocyte; Homo_723,totalSample,immune-Neutrophil; Homo_723,totalSample,immune-NKT; Homo_723,totalSample,immune-Th17; Homo_714,totalSample,immune-Th1; Homo_714,totalSample,immune-NKT; Homo_714,totalSample,immune-CD4_naive; Homo_633,totalSample,immune-NKT; Homo_633,totalSample,immune-Th1; Homo_633,totalSample,immune-Tfh; Homo_633,totalSample,immune-Tr1; Homo_633,totalSample,immune-CD4_naive; Homo_633,totalSample,immune-InfiltrationScore; Homo_633,totalSample,immune-iTreg; Homo_633,totalSample,immune-CD4_T; MCSA,totalSample,immune-DC; MCSA,totalSample,immune-Bcell; MCSA,totalSample,immune-Monocyte; MCSA,totalSample,immune-Macrophage; MCSA,totalSample,immune-CD4_T; MCSA,totalSample,immune-CD8_T; MCSA,totalSample,immune-CD4_naive; MCSA,totalSample,immune-Tr1; MCSA,totalSample,immune-nTreg; MCSA,totalSample,immune-iTreg; MCSA,totalSample,immune-Th2; MCSA,totalSample,immune-Th17; MCSA,totalSample,immune-Tfh; MCSA,totalSample,immune-CD8_naive; MCSA,totalSample,immune-Cytotoxic; MCSA,totalSample,immune-Exhausted; MCSA,totalSample,immune-MAIT; MCSA,totalSample,immune-Central_memory; ROSMAP,totalSample,immune-Th17; ROSMAP,totalSample,immune-InfiltrationScore; SRP223445,totalSample,immune-CD4_naive; SRP223445,totalSample,immune-Exhausted; ROSMAP,male- [illegible]
DNMT3B	Homo_723,female,AD.vs.control; Homo_723,male,AD.vs.control; Homo_723,female,AD.vs.MCI; Homo_723,male,AD.vs.MCI; Homo_723,female,MCI.vs.control; Homo_723,male,MCI.vs.control; Homo_714,female,AD.vs.control; Homo_714,male,AD.vs.control; Homo_714,female,AD.vs.MCI; Homo_714,male,AD.vs.MCI; Homo_714,male,MCI.vs.control; Homo_633,female,AD.vs.control; Homo_633,female,AD.vs.MCI; MCSA,totalSample,age; MCSA,control,age; Emory,totalSample,immune-iTreg; Emory,totalSample,immune-Th17; Homo_723,totalSample,immune-CD4_naive; Homo_723,totalSample,immune-Central_memory; Homo_723,totalSample,immune-Th17; Homo_723,totalSample,immune-CD4_T; Homo_723,totalSample,immune-nTreg; Homo_723,totalSample,immune-Tr1; Homo_723,totalSample,immune-Cytotoxic; Homo_723,totalSample,immune-Tfh; Homo_723,totalSample,immune-Exhausted; Homo_723,totalSample,immune-Neutrophil; Homo_714,totalSample,immune-CD4_naive; Homo_714,totalSample,immune-Central_memory; Homo_714,totalSample,immune-CD4_T; Homo_714,totalSample,immune-Th17; Homo_714,totalSample,immune-nTreg; Homo_714,totalSample,immune-Tr1; Homo_714,totalSample,immune-Tfh; Homo_714,totalSample,immune-iTreg; Homo_633,totalSample,immune-Tfh; Homo_633,totalSample,immune-CD4_T; Homo_633,totalSample,immune-Central_memory; Homo_633,totalSample,immune-CD4_naive; Homo_633,totalSample,immune-Tr1; Homo_633,totalSample,immune-nTreg; Homo_633,totalSample,immune-iTreg; Homo_633,totalSample,immune-Effector_memory; Homo_633,totalSample,immune-Th2; Homo_633,totalSample,immune-InfiltrationScore; Homo_633,totalSample,immune-Gamma_delta; Homo_633,totalSample,immune-Th17; MCSA,totalSample,immune-Monocyte; MCSA,totalSample,immune-CD4_T; MCSA,totalSample,immune-Gamma_delta; MCSA,totalSample,immune-CD4_naive; MCSA,totalSample,immune-Tr1; MCSA,totalSample,immune-nTreg; MCSA,totalSample,immune-iTreg; MCSA,totalSample,immune-Th17; MCSA,totalSample,immune-Tfh; MCSA,totalSample,immune-CD8_naive; MCSA,totalSample,immune-Exhausted; MCSA,totalSample,immune-Central_memory; MCSA,totalSample,immune-Effector_memory; ROSMAP,totalSample,immune-InfiltrationScore; SRP223445,totalSample,immune-Exhausted; ROSMAP,female-includeAge90+,survival
ETV5	Homo_723,female,AD.vs.control; Homo_723,male,AD.vs.control; Homo_723,female,AD.vs.MCI; Homo_723,male,AD.vs.MCI; Homo_714,female,AD.vs.control; Homo_714,male,AD.vs.control; Homo_714,female,AD.vs.MCI; Homo_714,male,AD.vs.MCI; Homo_633,female,AD.vs.control; Homo_633,female,AD.vs.MCI; ROSMAP,female,AD.vs.control; ROSMAP,male,AD.vs.control; ROSMAP,female,AD.vs.MCI; MCSA,MCI,age; ROSMAP,MCI,last.visit.age; Emory,totalSample,immune-Th1; Emory,totalSample,immune-Tfh; Homo_723,totalSample,immune-Th17; Homo_723,totalSample,immune-CD8_T; Homo_723,totalSample,immune-Exhausted; Homo_714,totalSample,immune-Exhausted; Homo_714,totalSample,immune-CD8_T; Homo_714,totalSample,immune-Th17; Homo_633,totalSample,immune-InfiltrationScore; MCSA,totalSample,immune-Neutrophil; MCSA,totalSample,immune-CD8_T; MCSA,totalSample,immune-Th17; MCSA,totalSample,immune-Cytotoxic; MCSA,totalSample,immune-Exhausted; ROSMAP,male-excludeAge90+,survival
SPATA7	Homo_723,female,AD.vs.control; Homo_723,male,AD.vs.control; Homo_723,female,AD.vs.MCI; Homo_723,male,AD.vs.MCI; Homo_714,female,AD.vs.control; Homo_714,male,AD.vs.control; Homo_714,female,AD.vs.MCI; Homo_714,male,AD.vs.MCI; Homo_633,female,AD.vs.control; Homo_633,female,AD.vs.MCI; ROSMAP,control,male.vs.female; ACOM,AD,age; MCSA,MCI,age; ROSMAP,AD,sample.age; ROSMAP,MCI,last.visit.age; ROSMAP,MCI,sample.age; Emory,totalSample,immune-Tr1; Emory,totalSample,immune-Effector_memory; Homo_723,totalSample,immune-Exhausted; Homo_723,totalSample,immune-InfiltrationScore; Homo_723,totalSample,immune-Th17; Homo_723,totalSample,immune-CD4_naive; Homo_723,totalSample,immune-Tfh; Homo_723,totalSample,immune-CD4_T; Homo_723,totalSample,immune-Th2; Homo_723,totalSample,immune-Tr1; Homo_723,totalSample,immune-Neutrophil; Homo_723,totalSample,immune-Monocyte; Homo_714,totalSample,immune-InfiltrationScore; Homo_714,totalSample,immune-Monocyte; Homo_714,totalSample,immune-CD4_naive; Homo_714,totalSample,immune-Tfh; Homo_714,totalSample,immune-NK; Homo_633,totalSample,immune-Tfh; Homo_633,totalSample,immune-InfiltrationScore; Homo_633,totalSample,immune-Th2; Homo_633,totalSample,immune-Monocyte; Homo_633,totalSample,immune-Effector_memory; Homo_633,totalSample,immune-CD4_T; Homo_633,totalSample,immune-CD4_naive; Homo_633,totalSample,immune-Central_memory; Homo_633,totalSample,immune-Bcell; Homo_633,totalSample,immune-Tr1; MCSA,totalSample,immune-DC; MCSA,totalSample,immune-Monocyte; MCSA,totalSample,immune-Macrophage; MCSA,totalSample,immune-CD4_T; MCSA,totalSample,immune-CD4_naive; MCSA,totalSample,immune-Tr1; MCSA,totalSample,immune-nTreg; MCSA,totalSample,immune-iTreg; MCSA,totalSample,immune-Th2; MCSA,totalSample,immune-Th17; MCSA,totalSample,immune-Tfh; MCSA,totalSample,immune-CD8_naive; MCSA,totalSample,immune-Cytotoxic; MCSA,totalSample,immune-Exhausted; MCSA,totalSample,immune-MAIT;
ANO6	Homo_723,female,AD.vs.control; Homo_723,male,AD.vs.control; Homo_723,female,AD.vs.MCI; Homo_723,male,AD.vs.MCI; Homo_723,female,MCI.vs.control; Homo_723,male,MCI.vs.control; Homo_714,female,AD.vs.control; Homo_714,male,AD.vs.control; Homo_714,female,AD.vs.MCI; Homo_714,male,AD.vs.MCI; Homo_714,female,MCI.vs.control; Homo_714,male,MCI.vs.control; Homo_633,female,AD.vs.control; Homo_633,female,AD.vs.MCI; MCSA,MCI,age; Emory,totalSample,immune-DC; Emory,totalSample,immune-NKT; Emory,totalSample,immune-Th17; Homo_723,totalSample,immune-Th17; Homo_723,totalSample,immune-Exhausted; Homo_723,totalSample,immune-CD4_naive; Homo_723,totalSample,immune-Cytotoxic; Homo_723,totalSample,immune-Central_memory; Homo_723,totalSample,immune-Gamma_delta; Homo_723,totalSample,immune-CD8_T; Homo_723,totalSample,immune-CD4_T; Homo_723,totalSample,immune-InfiltrationScore; Homo_723,totalSample,immune-Tr1; Homo_723,totalSample,immune-Tfh; Homo_723,totalSample,immune-NKT; Homo_723,totalSample,immune-iTreg; Homo_714,totalSample,immune-CD4_naive; Homo_714,totalSample,immune-Exhausted; Homo_714,totalSample,immune-Th17; Homo_714,totalSample,immune-NKT; Homo_714,totalSample,immune-Gamma_delta; Homo_714,totalSample,immune-Cytotoxic; Homo_714,totalSample,immune-Central_memory; Homo_714,totalSample,immune-CD4_T; Homo_714,totalSample,immune-Tfh; Homo_714,totalSample,immune-InfiltrationScore; Homo_714,totalSample,immune-CD8_T; Homo_714,totalSample,immune-Tr1; Homo_633,totalSample,immune-Tfh; Homo_633,totalSample,immune-CD4_T; Homo_633,totalSample,immune-CD4_naive; Homo_633,totalSample,immune-Central_memory; Homo_633,totalSample,immune-Effector_memory; Homo_633,totalSample,immune-Gamma_delta; Homo_633,totalSample,immune-NKT; Homo_633,totalSample,immune-InfiltrationScore; Homo_633,totalSample,immune-Th2; Homo_633,totalSample,immune-Tr1; Homo_633,totalSample,immune-iTreg; Homo_633,totalSample,immune-Th17; Homo_633,totalSample,immune-Exhausted; Homo_633,totalSample,immune-nTreg; Homo_633,totalSample,immune-Cytotoxic; MCSA,totalSample,immune-DC; MCSA,totalSample,immune-CD4_T; MCSA,totalSample,immune-CD8_T; MCSA,totalSample,immune-Gamma_delta; MCSA,totalSample,immune-CD4_naive; MCSA,totalSample,immune-Tr1; MCSA,totalSample,immune-nTreg; MCSA,totalSample,immune-iTreg; MCSA,totalSample,immune-Th17; MCSA,totalSample,immune-Tfh; MCSA,totalSample,immune-CD8_naive; MCSA,totalSample,immune-Cytotoxic; MCSA,totalSample,immune-Exhausted; MCSA,totalSample,immune-MAIT; MCSA,totalSample,immune-Central_memory; [illegible]
LIPC	Homo_723,female,AD.vs.control; Homo_723,male,AD.vs.control; Homo_723,female,AD.vs.MCI; Homo_723,male,AD.vs.MCI; Homo_714,female,AD.vs.control; Homo_714,male,AD.vs.control; Homo_714,female,AD.vs.MCI; Homo_714,male,AD.vs.MCI; Homo_633,female,AD.vs.control; Homo_633,female,AD.vs.MCI; ACOM,totalSample,age; Homo_723,totalSample,immune-DC; Homo_723,totalSample,immune-InfiltrationScore; Homo_723,totalSample,immune-Central_memory; Homo_723,totalSample,immune-Th17; Homo_714,totalSample,immune-DC; Homo_714,totalSample,immune-NK; Homo_633,totalSample,immune-InfiltrationScore; Homo_633,totalSample,immune-Tfh; Homo_633,totalSample,immune-DC; Homo_633,totalSample,immune-NK; MCSA,totalSample,immune-Effector_memory; ROSMAP,totalSample,immune-InfiltrationScore; SRP223445,totalSample,immune-CD4_naive; SRP223445,totalSample,immune-Exhausted
LIN9	Homo_723,female,AD.vs.control; Homo_723,male,AD.vs.control; Homo_723,female,AD.vs.MCI; Homo_723,male,AD.vs.MCI; Homo_714,female,AD.vs.control; Homo_714,male,AD.vs.control; Homo_714,female,AD.vs.MCI; Homo_714,male,AD.vs.MCI; Homo_633,female,AD.vs.control; Homo_633,female,AD.vs.MCI; ROSMAP,female,AD.vs.control; MCSA,MCI,age; ROSMAP,MCI,last.visit.age; Emory,totalSample,immune-Macrophage; Emory,totalSample,immune-Neutrophil; Emory,totalSample,immune-CD4_T; Emory,totalSample,immune-CD8_T; Emory,totalSample,immune-Tr1; Emory,totalSample,immune-iTreg; Emory,totalSample,immune-Tfh; Emory,totalSample,immune-Central_memory; Emory,totalSample,immune-InfiltrationScore; Homo_723,totalSample,immune-CD4_naive; Homo_723,totalSample,immune-CD4_T; Homo_723,totalSample,immune-Exhausted; Homo_723,totalSample,immune-Cytotoxic; Homo_723,totalSample,immune-Tr1; Homo_723,totalSample,immune-Tfh; Homo_723,totalSample,immune-Th1; Homo_723,totalSample,immune-Central_memory; Homo_723,totalSample,immune-Th17; Homo_723,totalSample,immune-Th2; Homo_723,totalSample,immune-iTreg; Homo_723,totalSample,immune-Neutrophil; Homo_723,totalSample,immune-nTreg; Homo_723,totalSample,immune-InfiltrationScore; Homo_723,totalSample,immune-CD8_T; Homo_723,totalSample,immune-NKT; Homo_714,totalSample,immune-CD4_naive; Homo_714,totalSample,immune-Tfh; Homo_714,totalSample,immune-Central_memory; Homo_714,totalSample,immune-CD4_T; Homo_714,totalSample,immune-Th1; Homo_714,totalSample,immune-Tr1; Homo_714,totalSample,immune-NKT; Homo_714,totalSample,immune-Monocyte; Homo_714,totalSample,immune-Exhausted; Homo_714,totalSample,immune-Th17; Homo_714,totalSample,immune-InfiltrationScore; Homo_714,totalSample,immune-iTreg; Homo_633,totalSample,immune-Tfh; Homo_633,totalSample,immune-CD4_naive; Homo_633,totalSample,immune-CD4_T; Homo_633,totalSample,immune-Central_memory; Homo_633,totalSample,immune-Effector_memory; Homo_633,totalSample,immune-Tr1; Homo_633,totalSample,immune-NKT; Homo_633,totalSample,immune-Th2; Homo_633,totalSample,immune-InfiltrationScore; Homo_633,totalSample,immune-iTreg; Homo_633,totalSample,immune-Monocyte; Homo_633,totalSample,immune-Gamma_delta; Homo_633,totalSample,immune-DC; Homo_633,totalSample,immune-Exhausted; MCSA,totalSample,immune-DC; MCSA,totalSample,immune-Bcell; MCSA,totalSample,immune-Monocyte; MCSA,totalSample,immune-Macrophage; MCSA,totalSample,immune-CD4_T; MCSA,totalSample,immune-CD8_T; MCSA,totalSample,immune-CD4_naive; MCSA,totalSample,immune-Tr1; MCSA,totalSample,immune-nTreg; MCSA,totalSample,immune- [illegible]
RNF217	Homo_723,female,AD.vs.control; Homo_723,male,AD.vs.control; Homo_723,female,AD.vs.MCI; Homo_723,male,AD.vs.MCI; Homo_723,female,MCI.vs.control; Homo_723,male,MCI.vs.control; Homo_714,female,AD.vs.control; Homo_714,male,AD.vs.control; Homo_714,female,AD.vs.MCI; Homo_714,male,AD.vs.MCI; Homo_714,female,MCI.vs.control; Homo_714,male,MCI.vs.control; Homo_633,female,AD.vs.control; Homo_633,female,AD.vs.MCI; ROSMAP,female,AD.vs.control; ROSMAP,female,AD.vs.MCI; MCSA,MCI,age; Emory,totalSample,immune-Monocyte; Emory,totalSample,immune-Macrophage; Emory,totalSample,immune-CD8_T; Emory,totalSample,immune-Gamma_delta; Emory,totalSample,immune-Exhausted; Homo_723,totalSample,immune-Exhausted; Homo_723,totalSample,immune-CD8_T; Homo_723,totalSample,immune-Cytotoxic; Homo_723,totalSample,immune-Th17; Homo_723,totalSample,immune-Monocyte; Homo_723,totalSample,immune-Macrophage; Homo_723,totalSample,immune-CD4_naive; Homo_723,totalSample,immune-Gamma_delta; Homo_723,totalSample,immune-Th1; Homo_714,totalSample,immune-Monocyte; Homo_714,totalSample,immune-Exhausted; Homo_714,totalSample,immune-Th17; Homo_714,totalSample,immune-CD8_T; Homo_714,totalSample,immune-Cytotoxic; Homo_714,totalSample,immune-Macrophage; Homo_714,totalSample,immune-Gamma_delta; Homo_633,totalSample,immune-Monocyte; Homo_633,totalSample,immune-Exhausted; Homo_633,totalSample,immune-Gamma_delta; Homo_633,totalSample,immune-Tfh; Homo_633,totalSample,immune-Th2; Homo_633,totalSample,immune-Th17; Homo_633,totalSample,immune-NKT; Homo_633,totalSample,immune-CD4_T; Homo_633,totalSample,immune-DC; Homo_633,totalSample,immune-CD8_T; Homo_633,totalSample,immune-CD4_naive; Homo_633,totalSample,immune-InfiltrationScore; Homo_633,totalSample,immune-Cytotoxic; MCSA,totalSample,immune-DC; MCSA,totalSample,immune-Monocyte; MCSA,totalSample,immune-CD4_T; MCSA,totalSample,immune-CD8_T; MCSA,totalSample,immune-CD4_naive; MCSA,totalSample,immune-Tr1; MCSA,totalSample,immune-nTreg; MCSA,totalSample,immune-iTreg; MCSA,totalSample,immune-Th17; MCSA,totalSample,immune-CD8_naive; MCSA,totalSample,immune-Cytotoxic; MCSA,totalSample,immune-Exhausted; MCSA,totalSample,immune-Effector_memory; ROSMAP,totalSample,immune-Effector_memory
KIF20B	Homo_723,female,AD.vs.control; Homo_723,male,AD.vs.control; Homo_723,female,AD.vs.MCI; Homo_723,male,AD.vs.MCI; Homo_714,female,AD.vs.control; Homo_714,male,AD.vs.control; Homo_714,female,AD.vs.MCI; Homo_714,male,AD.vs.MCI; Homo_633,female,AD.vs.control; Homo_633,female,AD.vs.MCI; ROSMAP,female,AD.vs.control; MCSA,MCI,age; Emory,totalSample,immune-Macrophage; Emory,totalSample,immune-NK; Emory,totalSample,immune-Neutrophil; Emory,totalSample,immune-CD4_T; Emory,totalSample,immune-CD8_T; Emory,totalSample,immune-Tr1; Emory,totalSample,immune-iTreg; Emory,totalSample,immune-Tfh; Emory,totalSample,immune-Exhausted; Emory,totalSample,immune-Central_memory; Emory,totalSample,immune-Effector_memory; Emory,totalSample,immune-InfiltrationScore; Homo_723,totalSample,immune-Th1; Homo_723,totalSample,immune-Central_memory; Homo_723,totalSample,immune-Neutrophil; Homo_723,totalSample,immune-CD4_naive; Homo_723,totalSample,immune-Tr1; Homo_723,totalSample,immune-NKT; Homo_723,totalSample,immune-CD4_T; Homo_723,totalSample,immune-Cytotoxic; Homo_723,totalSample,immune-nTreg; Homo_723,totalSample,immune-Tfh; Homo_714,totalSample,immune-Th1; Homo_714,totalSample,immune-NKT; Homo_714,totalSample,immune-CD4_naive; Homo_714,totalSample,immune-Neutrophil; Homo_714,totalSample,immune-InfiltrationScore; Homo_714,totalSample,immune-Central_memory; Homo_714,totalSample,immune-NK; Homo_633,totalSample,immune-NKT; Homo_633,totalSample,immune-Tfh; Homo_633,totalSample,immune-CD4_naive; Homo_633,totalSample,immune-Th1; Homo_633,totalSample,immune-Tr1; Homo_633,totalSample,immune-Central_memory; Homo_633,totalSample,immune-iTreg; Homo_633,totalSample,immune-CD4_T; Homo_633,totalSample,immune-InfiltrationScore; Homo_633,totalSample,immune-Neutrophil; Homo_633,totalSample,immune-Effector_memory; Homo_633,totalSample,immune-MAIT; MCSA,totalSample,immune-DC; MCSA,totalSample,immune-Bcell; MCSA,totalSample,immune-Monocyte; MCSA,totalSample,immune-Macrophage; MCSA,totalSample,immune-CD4_T; MCSA,totalSample,immune-NKT; MCSA,totalSample,immune-CD4_naive; MCSA,totalSample,immune-Tr1; MCSA,totalSample,immune-nTreg; MCSA,totalSample,immune-iTreg; MCSA,totalSample,immune-Th2; MCSA,totalSample,immune-Tfh; MCSA,totalSample,immune-CD8_naive; [illegible]
SVIP	Homo_723,female,AD.vs.control; Homo_723,male,AD.vs.control; Homo_723,female,AD.vs.MCI; Homo_723,male,AD.vs.MCI; Homo_723,female,MCI.vs.control; Homo_723,male,MCI.vs.control; Homo_714,female,AD.vs.control; Homo_714,male,AD.vs.control; Homo_714,female,AD.vs.MCI; Homo_714,male,AD.vs.MCI; Homo_714,female,MCI.vs.control; Homo_714,male,MCI.vs.control; Homo_633,female,AD.vs.control; Homo_633,female,AD.vs.MCI; Homo_633,female,MCI.vs.control; ROSMAP,female,AD.vs.MCI; ROSMAP,control,male.vs.female; Emory,totalSample,age; Emory,MCI,age; MCSA,MCI,age; ROSMAP,MCI,MMSE; Emory,totalSample,immune-DC; Emory,totalSample,immune-Monocyte; Emory,totalSample,immune-Macrophage; Emory,totalSample,immune-Neutrophil; Emory,totalSample,immune-CD4_T; Emory,totalSample,immune-CD8_T; Emory,totalSample,immune-Tr1; Emory,totalSample,immune-iTreg; Emory,totalSample,immune-Tfh; Emory,totalSample,immune-CD8_naive; Emory,totalSample,immune-Central_memory; Homo_723,totalSample,immune-Exhausted; Homo_723,totalSample,immune-Monocyte; Homo_723,totalSample,immune-Th17; Homo_723,totalSample,immune-CD4_naive; Homo_723,totalSample,immune-Cytotoxic; Homo_723,totalSample,immune-Tfh; Homo_723,totalSample,immune-CD4_T; Homo_723,totalSample,immune-Neutrophil; Homo_723,totalSample,immune-Central_memory; Homo_723,totalSample,immune-Th1; Homo_723,totalSample,immune-Tr1; Homo_723,totalSample,immune-CD8_T; Homo_714,totalSample,immune-Monocyte; Homo_714,totalSample,immune-CD4_naive; Homo_714,totalSample,immune-Th17; Homo_714,totalSample,immune-Central_memory; Homo_714,totalSample,immune-Tfh; Homo_714,totalSample,immune-CD4_T; Homo_714,totalSample,immune-Exhausted; Homo_633,totalSample,immune-Tfh; Homo_633,totalSample,immune-Monocyte; Homo_633,totalSample,immune-Th2; Homo_633,totalSample,immune-Central_memory; Homo_633,totalSample,immune-CD4_T; Homo_633,totalSample,immune-CD4_naive; Homo_633,totalSample,immune-Tr1; Homo_633,totalSample,immune-Effector_memory; Homo_633,totalSample,immune-DC; Homo_633,totalSample,immune-Gamma_delta; MCSA,totalSample,immune-DC; MCSA,totalSample,immune-Bcell; MCSA,totalSample,immune-Monocyte; MCSA,totalSample,immune-Macrophage; MCSA,totalSample,immune-CD4_T; MCSA,totalSample,immune-CD4_naive; MCSA,totalSample,immune-Tr1; MCSA,totalSample,immune-nTreg; MCSA,totalSample,immune-iTreg; MCSA,totalSample,immune-Th2; MCSA,totalSample,immune-Tfh; MCSA,totalSample,immune-CD8_naive; MCSA,totalSample,immune-Cytotoxic; MCSA,totalSample,immune-Exhausted; MCSA,totalSample,immune-MAIT; MCSA,totalSample,immune-Central_memory; [illegible]
MTFR1	Homo_723,female,AD.vs.control; Homo_723,male,AD.vs.control; Homo_723,female,AD.vs.MCI; Homo_723,male,AD.vs.MCI; Homo_714,female,AD.vs.control; Homo_714,male,AD.vs.control; Homo_714,female,AD.vs.MCI; Homo_714,male,AD.vs.MCI; Homo_633,female,AD.vs.control; Homo_633,female,AD.vs.MCI; ACOM,totalSample,age; MCSA,MCI,age; Emory,totalSample,immune-CD4_T; Emory,totalSample,immune-Tr1; Emory,totalSample,immune-iTreg; Emory,totalSample,immune-Tfh; Emory,totalSample,immune-Central_memory; Emory,totalSample,immune-Effector_memory; Emory,totalSample,immune-InfiltrationScore; Homo_723,totalSample,immune-Th1; Homo_723,totalSample,immune-CD4_naive; Homo_723,totalSample,immune-Cytotoxic; Homo_723,totalSample,immune-Central_memory; Homo_723,totalSample,immune-CD4_T; Homo_723,totalSample,immune-Tr1; Homo_723,totalSample,immune-NKT; Homo_723,totalSample,immune-Neutrophil; Homo_723,totalSample,immune-Th17; Homo_723,totalSample,immune-Tfh; Homo_723,totalSample,immune-InfiltrationScore; Homo_714,totalSample,immune-NKT; Homo_714,totalSample,immune-Th1; Homo_714,totalSample,immune-CD4_naive; Homo_714,totalSample,immune-InfiltrationScore; Homo_714,totalSample,immune-Exhausted; Homo_633,totalSample,immune-NKT; Homo_633,totalSample,immune-Tfh; Homo_633,totalSample,immune-Tr1; Homo_633,totalSample,immune-CD4_T; Homo_633,totalSample,immune-Th1; Homo_633,totalSample,immune-CD4_naive; Homo_633,totalSample,immune-Central_memory; Homo_633,totalSample,immune-InfiltrationScore; Homo_633,totalSample,immune-iTreg; MCSA,totalSample,immune-DC; MCSA,totalSample,immune-Monocyte; MCSA,totalSample,immune-Macrophage; MCSA,totalSample,immune-CD4_T; MCSA,totalSample,immune-CD8_T; MCSA,totalSample,immune-CD4_naive; MCSA,totalSample,immune-Tr1; MCSA,totalSample,immune-nTreg; MCSA,totalSample,immune-iTreg; MCSA,totalSample,immune-Th2; MCSA,totalSample,immune-Tfh; MCSA,totalSample,immune-CD8_naive; MCSA,totalSample,immune-

RAB30	Homo_723,female,AD.vs.control; Homo_723,male,AD.vs.control; Homo_723,female,AD.vs.MCI; Homo_723,male,AD.vs.MCI; Homo_723,female,MCI.vs.control; Homo_723,male,MCI.vs.control; Homo_714,female,AD.vs.control; Homo_714,male,AD.vs.control; Homo_714,female,AD.vs.MCI; Homo_714,male,AD.vs.MCI; Homo_714,female,MCI.vs.control; Homo_714,male,MCI.vs.control; Homo_633,female,AD.vs.control; Homo_633,female,AD.vs.MCI; MCSA,totalSample,age; MCSA,control,age; Emory,totalSample,immune-B_cell; Emory,totalSample,immune-Macrophage; Emory,totalSample,immune-Neutrophil; Emory,totalSample,immune-CD4_T; Emory,totalSample,immune-CD8_T; Emory,totalSample,immune-Tr1; Emory,totalSample,immune-iTreg; Emory,totalSample,immune-Tfh; Emory,totalSample,immune-CD8_naive; Emory,totalSample,immune-Central_memory; Emory,totalSample,immune-InfiltrationScore; Homo_723,totalSample,immune-CD4_naive; Homo_723,totalSample,immune-Central_memory; Homo_723,totalSample,immune-CD4_T; Homo_723,totalSample,immune-nTreg; Homo_723,totalSample,immune-Th1; Homo_723,totalSample,immune-Tr1; Homo_723,totalSample,immune-Th17; Homo_723,totalSample,immune-Tfh; Homo_723,totalSample,immune-Cytotoxic; Homo_723,totalSample,immune-iTreg; Homo_723,totalSample,immune-Neutrophil; Homo_723,totalSample,immune-Th2; Homo_723,totalSample,immune-Gamma_delta; Homo_714,totalSample,immune-CD4_naive; Homo_714,totalSample,immune-Th1; Homo_714,totalSample,immune-CD4_T; Homo_714,totalSample,immune-Central_memory; Homo_714,totalSample,immune-Tfh; Homo_714,totalSample,immune-NKT; Homo_714,totalSample,immune-nTreg; Homo_714,totalSample,immune-Tr1; Homo_714,totalSample,immune-iTreg; Homo_714,totalSample,immune-Th17; Homo_714,totalSample,immune-Cytotoxic; Homo_714,totalSample,immune-Neutrophil; Homo_714,totalSample,immune-Gamma_delta; Homo_714,totalSample,immune-Exhausted; Homo_714,totalSample,immune-InfiltrationScore; Homo_633,totalSample,immune-Tfh; Homo_633,totalSample,immune-CD4_T; Homo_633,totalSample,immune-CD4_naive; Homo_633,totalSample,immune-Tr1; Homo_633,totalSample,immune-Central_memory; Homo_633,totalSample,immune-iTreg; Homo_633,totalSample,immune-NKT; Homo_633,totalSample,immune-nTreg; Homo_633,totalSample,immune-Th1; Homo_633,totalSample,immune-Effector_memory; Homo_633,totalSample,immune-Th2; Homo_633,totalSample,immune-Gamma_delta; Homo_633,totalSample,immune-InfiltrationScore; Homo_633,totalSample,immune-Neutrophil; Homo_633,totalSample,immune-Cytotoxic; Homo_633,totalSample,immune-Th17; Homo_633,totalSample,immune-Macrophage; MCSA,totalSample,immune-DC; MCSA,totalSample,immune-Bcell; MCSA,totalSample,immune-Macrophage; MCSA,totalSample,immune-CD4_T; MCSA,totalSample,immune-Gamma_delta; MCSA,totalSample,immune-CD4_naive; MCSA,totalSample,immune-Tr1; MCSA,totalSample,immune-nTreg; MCSA,totalSample,immune-iTreg; MCSA,totalSample,immune-Th17; MCSA,totalSample,immune-Tfh; MCSA,totalSample,immune-CD8_naive; MCSA,totalSample,immune-Cytotoxic; MCSA,totalSample,immune-Exhausted; MCSA,totalSample,immune-Central_memory; ROSMAP,totalSample,immune-InfiltrationScore; SRP223445,totalSample,immune-Neutrophil; SRP223445,totalSample,immune-CD4_naive; SRP223445,totalSample,immune-InfiltrationScore
MRM2	Homo_723,female,AD.vs.control; Homo_723,male,AD.vs.control; Homo_723,female,AD.vs.MCI; Homo_723,male,AD.vs.MCI; Homo_714,female,AD.vs.control; Homo_714,male,AD.vs.control; Homo_714,female,AD.vs.MCI; Homo_714,male,AD.vs.MCI; Homo_633,female,AD.vs.control; Homo_633,female,AD.vs.MCI; Emory,totalSample,immune-Macrophage; Emory,totalSample,immune-NK; Emory,totalSample,immune-Neutrophil; Emory,totalSample,immune-CD4_T; Emory,totalSample,immune-CD8_T; Emory,totalSample,immune-Tr1; Emory,totalSample,immune-iTreg; Emory,totalSample,immune-Tfh; Emory,totalSample,immune-Exhausted; Emory,totalSample,immune-Central_memory; Emory,totalSample,immune-Effector_memory; Emory,totalSample,immune-InfiltrationScore; Homo_723,totalSample,immune-Tfh; Homo_723,totalSample,immune-Th2; Homo_723,totalSample,immune-Effector_memory; Homo_723,totalSample,immune-iTreg; Homo_723,totalSample,immune-InfiltrationScore; Homo_723,totalSample,immune-CD4_T; Homo_723,totalSample,immune-Tr1; Homo_723,totalSample,immune-CD4_naive; Homo_723,totalSample,immune-nTreg; Homo_723,totalSample,immune-Monocyte; Homo_723,totalSample,immune-Macrophage; Homo_723,totalSample,immune-Central_memory; Homo_723,totalSample,immune-Bcell; Homo_714,totalSample,immune-Tfh; Homo_714,totalSample,immune-Th2; Homo_714,totalSample,immune-Effector_memory; Homo_714,totalSample,immune-CD4_T; Homo_714,totalSample,immune-Tr1; Homo_714,totalSample,immune-InfiltrationScore; Homo_714,totalSample,immune-Central_memory; Homo_714,totalSample,immune-CD4_naive; Homo_714,totalSample,immune-nTreg; Homo_714,totalSample,immune-iTreg; Homo_633,totalSample,immune-Tfh; Homo_633,totalSample,immune-Effector_memory; Homo_633,totalSample,immune-Th2; Homo_633,totalSample,immune-InfiltrationScore; Homo_633,totalSample,immune-CD4_T; Homo_633,totalSample,immune-CD4_naive; Homo_633,totalSample,immune-Central_memory; Homo_633,totalSample,immune-Tr1; Homo_633,totalSample,immune-nTreg; Homo_633,totalSample,immune-Monocyte; Homo_633,totalSample,immune-Neutrophil; Homo_633,totalSample,immune-Exhausted; Homo_633,totalSample,immune-Bcell; MCSA,totalSample,immune-Macrophage; MCSA,totalSample,immune-NK; MCSA,totalSample,immune-Neutrophil; MCSA,totalSample,immune-CD4_T; MCSA,totalSample,immune-CD4_naive; MCSA,totalSample,immune-Tr1; MCSA,totalSample,immune-nTreg; MCSA,totalSample,immune-iTreg; MCSA,totalSample,immune-Tfh; MCSA,totalSample,immune-CD8_naive; MCSA,totalSample,immune-MAIT; MCSA,totalSample,immune-Central_memory; MCSA,totalSample,immune-Effector_memory; MCSA,totalSample,immune-InfiltrationScore; ROSMAP,totalSample,immune-Th17; ROSMAP,totalSample,immune-Effector_memory; ROSMAP,totalSample,immune-InfiltrationScore; SRP223445,totalSample,immune-NK; SRP223445,totalSample,immune-CD4_naive; SRP223445,totalSample,immune-Th17; SRP223445,totalSample,immune-Exhausted; SRP223445,totalSample,immune-InfiltrationScore
GDPD1	Homo_723,female,AD.vs.control; Homo_723,male,AD.vs.control; Homo_723,female,AD.vs.MCI; Homo_723,male,AD.vs.MCI; Homo_723,female,MCI.vs.control; Homo_723,male,MCI.vs.control; Homo_714,female,AD.vs.control; Homo_714,male,AD.vs.control; Homo_714,female,AD.vs.MCI; Homo_714,male,AD.vs.MCI; Homo_714,female,MCI.vs.control; Homo_714,male,MCI.vs.control; Homo_633,female,AD.vs.control; Homo_633,female,AD.vs.MCI; ROSMAP,female,AD.vs.control; ROSMAP,male,AD.vs.control; ROSMAP,female,AD.vs.MCI; ROSMAP,female,MCI.vs.control; ROSMAP,control,male.vs.female; ROSMAP,MCI,male.vs.female; ROSMAP,MCI,sample.age; Emory,totalSample,immune-NK; Emory,totalSample,immune-Tr1; Emory,totalSample,immune-iTreg; Emory,totalSample,immune-InfiltrationScore; Homo_723,totalSample,immune-CD4_T; Homo_723,totalSample,immune-CD4_naive; Homo_723,totalSample,immune-Th2; Homo_723,totalSample,immune-Th17; Homo_723,totalSample,immune-Exhausted; Homo_723,totalSample,immune-Tfh; Homo_723,totalSample,immune-Tr1; Homo_723,totalSample,immune-Cytotoxic; Homo_723,totalSample,immune-iTreg; Homo_723,totalSample,immune-Central_memory; Homo_723,totalSample,immune-nTreg; Homo_723,totalSample,immune-Th1; Homo_723,totalSample,immune-InfiltrationScore; Homo_714,totalSample,immune-CD4_naive; Homo_714,totalSample,immune-Tfh; Homo_714,totalSample,immune-CD4_T; Homo_714,totalSample,immune-Th17; Homo_714,totalSample,immune-Th2; Homo_714,totalSample,immune-Tr1; Homo_714,totalSample,immune-Central_memory; Homo_714,totalSample,immune-InfiltrationScore; Homo_714,totalSample,immune-Monocyte; Homo_714,totalSample,immune-Exhausted; Homo_633,totalSample,immune-Tfh; Homo_633,totalSample,immune-Th2; Homo_633,totalSample,immune-CD4_T; Homo_633,totalSample,immune-CD4_naive; Homo_633,totalSample,immune-Effector_memory; Homo_633,totalSample,immune-InfiltrationScore; Homo_633,totalSample,immune-Central_memory; Homo_633,totalSample,immune-Tr1; Homo_633,totalSample,immune-Th17; Homo_633,totalSample,immune-NKT; Homo_633,totalSample,immune-Monocyte; Homo_633,totalSample,immune-iTreg; Homo_633,totalSample,immune-Exhausted; Homo_633,totalSample,immune-Bcell; Homo_633,totalSample,immune-nTreg; MCSA,totalSample,immune-DC; MCSA,totalSample,immune-Macrophage; MCSA,totalSample,immune-CD4_T; MCSA,totalSample,immune-CD4_naive; MCSA,totalSample,immune-Tr1; MCSA,totalSample,immune-nTreg; MCSA,totalSample,immune-iTreg; MCSA,totalSample,immune-Th17; MCSA,totalSample,immune-Tfh; MCSA,totalSample,immune-CD8_naive; MCSA,totalSample,immune-Cytotoxic; MCSA,totalSample,immune-Exhausted; [illegible]
GRB14	Homo_723,female,AD.vs.control; Homo_723,male,AD.vs.control; Homo_723,female,AD.vs.MCI; Homo_723,male,AD.vs.MCI; Homo_723,female,MCI.vs.control; Homo_723,male,MCI.vs.control; Homo_714,female,AD.vs.control; Homo_714,male,AD.vs.control; Homo_714,female,AD.vs.MCI; Homo_714,male,AD.vs.MCI; Homo_714,female,MCI.vs.control; Homo_714,male,MCI.vs.control; Homo_633,female,AD.vs.control; Homo_633,female,AD.vs.MCI; Homo_633,female,MCI.vs.control; ROSMAP,female,AD.vs.control; SRP223445,female,AD.vs.control; ROSMAP,totalSample,age; Homo_723,totalSample,immune-Exhausted; Homo_723,totalSample,immune-CD8_T; Homo_723,totalSample,immune-Th17; Homo_723,totalSample,immune-Monocyte; Homo_723,totalSample,immune-Cytotoxic; Homo_714,totalSample,immune-Monocyte; Homo_714,totalSample,immune-Exhausted; Homo_714,totalSample,immune-Th17; Homo_633,totalSample,immune-Monocyte; Homo_633,totalSample,immune-Th2; MCSA,totalSample,immune-DC; MCSA,totalSample,immune-Monocyte; MCSA,totalSample,immune-iTreg; ROSMAP,totalSample,immune-Th17; ROSMAP,totalSample,immune-InfiltrationScore; SRP223445,totalSample,immune-CD4_naive; SRP223445,totalSample,immune-Exhausted
FANCL	Homo_723,female,AD.vs.control; Homo_723,male,AD.vs.control; Homo_723,female,AD.vs.MCI; Homo_723,male,AD.vs.MCI; Homo_723,female,MCI.vs.control; Homo_723,male,MCI.vs.control; Homo_714,female,AD.vs.control; Homo_714,male,AD.vs.control; Homo_714,female,AD.vs.MCI; Homo_714,male,AD.vs.MCI; Homo_714,female,MCI.vs.control; Homo_714,male,MCI.vs.control; Homo_633,female,AD.vs.control; Homo_633,female,AD.vs.MCI; MCSA,MCI,age; Emory,totalSample,immune-Neutrophil; Emory,totalSample,immune-Tr1; Emory,totalSample,immune-Effector_memory; Emory,totalSample,immune-InfiltrationScore; Homo_723,totalSample,immune-Exhausted; Homo_723,totalSample,immune-Th17; Homo_723,totalSample,immune-Th1; Homo_723,totalSample,immune-CD4_naive; Homo_723,totalSample,immune-Neutrophil; Homo_723,totalSample,immune-Cytotoxic; Homo_723,totalSample,immune-CD8_T; Homo_723,totalSample,immune-InfiltrationScore; Homo_714,totalSample,immune-InfiltrationScore; Homo_714,totalSample,immune-CD4_naive; Homo_633,totalSample,immune-Effector_memory; Homo_633,totalSample,immune-InfiltrationScore; Homo_633,totalSample,immune-Tfh; Homo_633,totalSample,immune-CD4_T; Homo_633,totalSample,immune-CD4_naive; Homo_633,totalSample,immune-Central_memory; MCSA,totalSample,immune-DC; MCSA,totalSample,immune-CD4_T; MCSA,totalSample,immune-Tr1; MCSA,totalSample,immune-nTreg; MCSA,totalSample,immune-iTreg; MCSA,totalSample,immune-Cytotoxic; SRP223445,totalSample,immune-InfiltrationScore
APC2	Homo_723,female,AD.vs.control; Homo_723,male,AD.vs.control; Homo_723,female,AD.vs.MCI; Homo_723,male,AD.vs.MCI; Homo_714,female,AD.vs.control; Homo_714,female,AD.vs.MCI; Homo_633,female,AD.vs.control; Homo_633,female,AD.vs.MCI; Homo_633,female,MCI.vs.control; Homo_633,male,MCI.vs.control; ROSMAP,female,AD.vs.control; ROSMAP,control,male.vs.female; MCSA,MCI,age; ROSMAP,AD,last.visit.age; Homo_723,totalSample,immune-DC; Homo_723,totalSample,immune-Monocyte; Homo_723,totalSample,immune-Th2; Homo_723,totalSample,immune-Neutrophil; Homo_714,totalSample,immune-DC; Homo_714,totalSample,immune-Th2; Homo_714,totalSample,immune-Monocyte; Homo_714,totalSample,immune-InfiltrationScore; Homo_633,totalSample,immune-DC; Homo_633,totalSample,immune-Monocyte; Homo_633,totalSample,immune-Th2; MCSA,totalSample,immune-DC; MCSA,totalSample,immune-Monocyte; MCSA,totalSample,immune-Macrophage; MCSA,totalSample,immune-Neutrophil; MCSA,totalSample,immune-CD8_T; MCSA,totalSample,immune-NKT; MCSA,totalSample,immune-iTreg; MCSA,totalSample,immune-Th2; MCSA,totalSample,immune-Cytotoxic; MCSA,totalSample,immune-Effector_memory; MCSA,totalSample,immune-InfiltrationScore; SRP223445,totalSample,immune-InfiltrationScore
PRC1	Homo_723,female,AD.vs.control; Homo_723,male,AD.vs.control; Homo_723,female,AD.vs.MCI; Homo_723,male,AD.vs.MCI; Homo_723,female,MCI.vs.control; Homo_723,male,MCI.vs.control; Homo_714,female,AD.vs.control; Homo_714,male,AD.vs.control; Homo_714,female,AD.vs.MCI; Homo_714,male,AD.vs.MCI; Homo_714,female,MCI.vs.control; Homo_714,male,MCI.vs.control; Homo_633,female,AD.vs.control; Homo_633,female,AD.vs.MCI; MCSA,MCI,age; Emory,totalSample,immune-Neutrophil; Emory,totalSample,immune-CD4_T; Emory,totalSample,immune-Tr1; Emory,totalSample,immune-iTreg; Emory,totalSample,immune-Exhausted; Emory,totalSample,immune-InfiltrationScore; Homo_723,totalSample,immune-CD4_naive; Homo_723,totalSample,immune-Th17; Homo_723,totalSample,immune-Exhausted; Homo_723,totalSample,immune-CD4_T; Homo_723,totalSample,immune-Tfh; Homo_723,totalSample,immune-Tr1; Homo_723,totalSample,immune-InfiltrationScore; Homo_723,totalSample,immune-nTreg; Homo_723,totalSample,immune-Neutrophil; Homo_723,totalSample,immune-Central_memory; Homo_723,totalSample,immune-Th2; Homo_723,totalSample,immune-Effector_memory; Homo_723,totalSample,immune-Cytotoxic; Homo_723,totalSample,immune-Th1; Homo_714,totalSample,immune-CD4_naive; Homo_714,totalSample,immune-InfiltrationScore; Homo_714,totalSample,immune-Central_memory; Homo_714,totalSample,immune-CD4_T; Homo_714,totalSample,immune-Tfh; Homo_714,totalSample,immune-Th17; Homo_714,totalSample,immune-Effector_memory; Homo_714,totalSample,immune-Tr1; Homo_714,totalSample,immune-Neutrophil; Homo_633,totalSample,immune-Tfh; Homo_633,totalSample,immune-Effector_memory; Homo_633,totalSample,immune-CD4_T; Homo_633,totalSample,immune-Th2; Homo_633,totalSample,immune-InfiltrationScore; Homo_633,totalSample,immune-CD4_naive; Homo_633,totalSample,immune-Central_memory; Homo_633,totalSample,immune-Tr1; Homo_633,totalSample,immune-nTreg; Homo_633,totalSample,immune-iTreg; Homo_633,totalSample,immune-Gamma_delta; MCSA,totalSample,immune-CD4_T; MCSA,totalSample,immune-CD4_naive; MCSA,totalSample,immune-Tr1; MCSA,totalSample,immune-nTreg; MCSA,totalSample,immune-iTreg; MCSA,totalSample,immune-Th17; MCSA,totalSample,immune-Tfh; MCSA,totalSample,immune-CD8_naive; MCSA,totalSample,immune-Exhausted; [illegible]
BTNL8	Homo_723,female,AD.vs.control; Homo_723,male,AD.vs.control; Homo_723,female,AD.vs.MCI; Homo_723,male,AD.vs.MCI; Homo_714,female,AD.vs.control; Homo_714,male,AD.vs.control; Homo_714,female,AD.vs.MCI; Homo_714,male,AD.vs.MCI; Homo_633,female,AD.vs.control; Homo_633,female,AD.vs.MCI; SRP223445,male,AD.vs.control; Emory,MCI,age; Emory,totalSample,immune-DC; Emory,totalSample,immune-Monocyte; Emory,totalSample,immune-Neutrophil; Emory,totalSample,immune-CD4_T; Emory,totalSample,immune-CD8_T; Emory,totalSample,immune-Gamma_delta; Emory,totalSample,immune-Th1; Emory,totalSample,immune-Th17; Emory,totalSample,immune-Tfh; Emory,totalSample,immune-CD8_naive; Emory,totalSample,immune-Cytotoxic; Emory,totalSample,immune-Exhausted; Emory,totalSample,immune-MAIT; Emory,totalSample,immune-InfiltrationScore; Homo_723,totalSample,immune-Neutrophil; Homo_723,totalSample,immune-Monocyte; Homo_723,totalSample,immune-Gamma_delta; Homo_723,totalSample,immune-DC; Homo_723,totalSample,immune-Th1; Homo_723,totalSample,immune-Bcell; Homo_714,totalSample,immune-Neutrophil; Homo_714,totalSample,immune-Monocyte; Homo_714,totalSample,immune-Gamma_delta; Homo_714,totalSample,immune-Th1; Homo_714,totalSample,immune-Th2; Homo_633,totalSample,immune-Neutrophil; Homo_633,totalSample,immune-Exhausted; Homo_633,totalSample,immune-Monocyte; Homo_633,totalSample,immune-Th2; Homo_633,totalSample,immune-Gamma_delta; Homo_633,totalSample,immune-CD8_T; Homo_633,totalSample,immune-Th17; MCSA,totalSample,immune-Neutrophil; MCSA,totalSample,immune-CD8_T; MCSA,totalSample,immune-Th17; MCSA,totalSample,immune-Tfh; MCSA,totalSample,immune-Cytotoxic; MCSA,totalSample,immune-Exhausted; MCSA,totalSample,immune-Central_memory; MCSA,totalSample,immune-Effector_memory; MCSA,totalSample,immune-InfiltrationScore; ROSMAP,totalSample,immune-InfiltrationScore; SRP223445,totalSample,immune-Neutrophil
DENND5B	Homo_723,female,AD.vs.control; Homo_723,male,AD.vs.control; Homo_723,female,AD.vs.MCI; Homo_723,male,AD.vs.MCI; Homo_723,female,MCI.vs.control; Homo_723,male,MCI.vs.control; Homo_714,female,AD.vs.control; Homo_714,male,AD.vs.control; Homo_714,female,AD.vs.MCI; Homo_714,male,AD.vs.MCI; Homo_714,female,MCI.vs.control; Homo_633,female,AD.vs.control; Homo_633,female,AD.vs.MCI; ROSMAP,female,AD.vs.control; ROSMAP,male,AD.vs.control; ROSMAP,male,AD.vs.MCI; ROSMAP,female,MCI.vs.control; ROSMAP,AD,male.vs.female; MCSA,MCI,age; Emory,totalSample,immune-B_cell; Emory,totalSample,immune-Macrophage; Emory,totalSample,immune-Neutrophil; Emory,totalSample,immune-CD4_T; Emory,totalSample,immune-Gamma_delta; Emory,totalSample,immune-Tr1; Emory,totalSample,immune-iTreg; Emory,totalSample,immune-CD8_naive; Emory,totalSample,immune-InfiltrationScore; Homo_723,totalSample,immune-CD4_naive; Homo_723,totalSample,immune-CD4_T; Homo_723,totalSample,immune-Th1; Homo_723,totalSample,immune-Central_memory; Homo_723,totalSample,immune-nTreg; Homo_723,totalSample,immune-Th17; Homo_723,totalSample,immune-Tr1; Homo_723,totalSample,immune-Tfh; Homo_723,totalSample,immune-Cytotoxic; Homo_723,totalSample,immune-Neutrophil; Homo_723,totalSample,immune-iTreg; Homo_723,totalSample,immune-Th2; Homo_723,totalSample,immune-InfiltrationScore; Homo_723,totalSample,immune-Exhausted; Homo_714,totalSample,immune-CD4_naive; Homo_714,totalSample,immune-Th1; Homo_714,totalSample,immune-CD4_T; Homo_714,totalSample,immune-NKT; Homo_714,totalSample,immune-Tfh; Homo_714,totalSample,immune-Central_memory; Homo_714,totalSample,immune-nTreg; Homo_714,totalSample,immune-Tr1; Homo_714,totalSample,immune-iTreg; Homo_714,totalSample,immune-Th17; Homo_714,totalSample,immune-Neutrophil; Homo_714,totalSample,immune-InfiltrationScore; Homo_714,totalSample,immune-Cytotoxic; Homo_714,totalSample,immune-Exhausted; Homo_633,totalSample,immune-Tfh; Homo_633,totalSample,immune-CD4_T; Homo_633,totalSample,immune-CD4_naive; Homo_633,totalSample,immune-Tr1; Homo_633,totalSample,immune-NKT; Homo_633,totalSample,immune-Central_memory; Homo_633,totalSample,immune-iTreg; Homo_633,totalSample,immune-Th1; Homo_633,totalSample,immune-nTreg; Homo_633,totalSample,immune-Effector_memory; Homo_633,totalSample,immune-Gamma_delta; Homo_633,totalSample,immune-InfiltrationScore; Homo_633,totalSample,immune-Th2; Homo_633,totalSample,immune-Neutrophil; MCSA,totalSample,immune-DC; MCSA,totalSample,immune-Bcell; MCSA,totalSample,immune-Macrophage; MCSA,totalSample,immune-CD4_T; MCSA,totalSample,immune-Gamma_delta; MCSA,totalSample,immune-CD4_naive; MCSA,totalSample,immune-Tr1; MCSA,totalSample,immune-nTreg; MCSA,totalSample,immune-iTreg; MCSA,totalSample,immune-Th17; MCSA,totalSample,immune-Tfh; MCSA,totalSample,immune-CD8_naive; MCSA,totalSample,immune-Cytotoxic; MCSA,totalSample,immune-Exhausted; [illegible]
SLC8A3	Homo_723,female,AD.vs.control; Homo_723,male,AD.vs.control; Homo_723,female,AD.vs.MCI; Homo_723,male,AD.vs.MCI; Homo_723,female,MCI.vs.control; Homo_723,male,MCI.vs.control; Homo_714,female,AD.vs.control; Homo_714,male,AD.vs.control; Homo_714,female,AD.vs.MCI; Homo_714,male,AD.vs.MCI; Homo_714,female,MCI.vs.control; Homo_714,male,MCI.vs.control; Homo_633,female,AD.vs.control; Homo_633,female,AD.vs.MCI; Emory,totalSample,immune-Th1; Homo_723,totalSample,immune-DC; Homo_723,totalSample,immune-Th17; Homo_723,totalSample,immune-CD8_T; Homo_723,totalSample,immune-MAIT; Homo_714,totalSample,immune-Th17; Homo_633,totalSample,immune-InfiltrationScore; MCSA,totalSample,immune-CD4_naive; MCSA,totalSample,immune-nTreg; MCSA,totalSample,immune-Th17; ROSMAP,totalSample,immune-InfiltrationScore; SRP223445,totalSample,immune-CD4_naive; SRP223445,totalSample,immune-Exhausted
KMT5A	Homo_723,female,AD.vs.control; Homo_723,male,AD.vs.control; Homo_723,female,AD.vs.MCI; Homo_723,male,AD.vs.MCI; Homo_714,female,AD.vs.control; Homo_714,male,AD.vs.control; Homo_714,female,AD.vs.MCI; Homo_714,male,AD.vs.MCI; Homo_633,female,AD.vs.control; Homo_633,female,AD.vs.MCI; MCSA,totalSample,age; MCSA,control,age; Emory,totalSample,immune-CD4_T; Emory,totalSample,immune-Tr1; Emory,totalSample,immune-iTreg; Emory,totalSample,immune-InfiltrationScore; Homo_723,totalSample,immune-DC; Homo_723,totalSample,immune-Th17; Homo_723,totalSample,immune-nTreg; Homo_723,totalSample,immune-InfiltrationScore; Homo_723,totalSample,immune-CD4_naive; Homo_723,totalSample,immune-Central_memory; Homo_714,totalSample,immune-InfiltrationScore; Homo_714,totalSample,immune-Th17; Homo_714,totalSample,immune-Exhausted; Homo_633,totalSample,immune-InfiltrationScore; Homo_633,totalSample,immune-nTreg; Homo_633,totalSample,immune-Tfh; Homo_633,totalSample,immune-iTreg; Homo_633,totalSample,immune-CD4_T; Homo_633,totalSample,immune-Central_memory; Homo_633,totalSample,immune-Effector_memory; Homo_633,totalSample,immune-Tr1; MCSA,totalSample,immune-DC; MCSA,totalSample,immune-Monocyte; MCSA,totalSample,immune-CD4_T; MCSA,totalSample,immune-NKT; MCSA,totalSample,immune-Gamma_delta; MCSA,totalSample,immune-CD4_naive; MCSA,totalSample,immune-Tr1; MCSA,totalSample,immune-nTreg; MCSA,totalSample,immune-Th2; MCSA,totalSample,immune-Th17; MCSA,totalSample,immune-Tfh; MCSA,totalSample,immune-Exhausted; MCSA,totalSample,immune-Central_memory; MCSA,totalSample,immune-Effector_memory; MCSA,totalSample,immune-
ST8SIA6	Homo_723,female,AD.vs.control; Homo_723,male,AD.vs.control; Homo_723,female,AD.vs.MCI; Homo_723,male,AD.vs.MCI; Homo_714,female,AD.vs.control; Homo_714,male,AD.vs.control; Homo_714,female,AD.vs.MCI; Homo_714,male,AD.vs.MCI; Homo_633,female,AD.vs.control; Homo_633,female,AD.vs.MCI; ROSMAP,female,AD.vs.control; ROSMAP,female,MCI.vs.control; ROSMAP,control,male.vs.female; MCSA,MCI,age; Emory,totalSample,immune-NK; Emory,totalSample,immune-Neutrophil; Emory,totalSample,immune-CD8_T; Emory,totalSample,immune-Gamma_delta; Emory,totalSample,immune-Tr1; Emory,totalSample,immune-Cytotoxic; Emory,totalSample,immune-Exhausted; Emory,totalSample,immune-Effector_memory; Emory,totalSample,immune-InfiltrationScore; Homo_723,totalSample,immune-InfiltrationScore; Homo_723,totalSample,immune-NK; Homo_723,totalSample,immune-Neutrophil; Homo_723,totalSample,immune-CD4_naive; Homo_723,totalSample,immune-DC; Homo_714,totalSample,immune-InfiltrationScore; Homo_714,totalSample,immune-NK; Homo_714,totalSample,immune-Neutrophil; Homo_714,totalSample,immune-NKT; Homo_714,totalSample,immune-CD4_naive; Homo_633,totalSample,immune-InfiltrationScore; Homo_633,totalSample,immune-NK; Homo_633,totalSample,immune-Effector_memory; Homo_633,totalSample,immune-Tfh; Homo_633,totalSample,immune-CD4_naive; Homo_633,totalSample,immune-Neutrophil; Homo_633,totalSample,immune-NKT; Homo_633,totalSample,immune-Macrophage; Homo_633,totalSample,immune-Bcell; MCSA,totalSample,immune-DC; MCSA,totalSample,immune-CD4_T; MCSA,totalSample,immune-CD4_naive; MCSA,totalSample,immune-Tr1; MCSA,totalSample,immune-nTreg; MCSA,totalSample,immune-iTreg; MCSA,totalSample,immune-Tfh; MCSA,totalSample,immune-MAIT; SRP223445,totalSample,immune-CD4_naive; SRP223445,totalSample,immune-Exhausted
GABRR2	Homo_723,female,AD.vs.control; Homo_723,male,AD.vs.control; Homo_723,female,AD.vs.MCI; Homo_723,male,AD.vs.MCI; Homo_714,female,AD.vs.control; Homo_714,male,AD.vs.control; Homo_714,female,AD.vs.MCI; Homo_714,male,AD.vs.MCI; Homo_633,female,AD.vs.control; Homo_633,female,AD.vs.MCI; ROSMAP,male,AD.vs.control; ROSMAP,female,MCI.vs.control; Emory,totalSample,immune-Gamma_delta; Emory,totalSample,immune-Cytotoxic; Homo_723,totalSample,immune-Th2; Homo_723,totalSample,immune-Gamma_delta; Homo_723,totalSample,immune-DC; Homo_723,totalSample,immune-Monocyte; Homo_723,totalSample,immune-Tfh; Homo_723,totalSample,immune-iTreg; Homo_723,totalSample,immune-Neutrophil; Homo_723,totalSample,immune-CD4_T; Homo_723,totalSample,immune-Tr1; Homo_714,totalSample,immune-Th2; Homo_714,totalSample,immune-Tfh; Homo_714,totalSample,immune-Gamma_delta; Homo_714,totalSample,immune-Monocyte; Homo_714,totalSample,immune-CD4_T; Homo_714,totalSample,immune-Tr1; Homo_633,totalSample,immune-Th2; Homo_633,totalSample,immune-Monocyte; Homo_633,totalSample,immune-Gamma_delta; Homo_633,totalSample,immune-Th17; Homo_633,totalSample,immune-Exhausted; Homo_633,totalSample,immune-Tfh; Homo_633,totalSample,immune-CD4_T; Homo_633,totalSample,immune-Central_memory; MCSA,totalSample,immune-CD4_T; MCSA,totalSample,immune-CD8_T; MCSA,totalSample,immune-Gamma_delta; MCSA,totalSample,immune-CD4_naive; MCSA,totalSample,immune-Tr1; MCSA,totalSample,immune-nTreg; MCSA,totalSample,immune-iTreg; MCSA,totalSample,immune-Th17; MCSA,totalSample,immune-Cytotoxic; MCSA,totalSample,immune-Exhausted;
BIK	Homo_723,female,AD.vs.control; Homo_723,male,AD.vs.control; Homo_723,female,AD.vs.MCI; Homo_723,male,AD.vs.MCI; Homo_714,female,AD.vs.control; Homo_714,male,AD.vs.control; Homo_714,female,AD.vs.MCI; Homo_714,male,AD.vs.MCI; Homo_633,female,AD.vs.control; Homo_633,female,AD.vs.MCI; ROSMAP,male,AD.vs.control; ROSMAP,female,MCI.vs.control; ROSMAP,AD,male.vs.female; MCSA,MCI,age; Homo_723,totalSample,immune-Exhausted; Homo_723,totalSample,immune-CD8_T; Homo_723,totalSample,immune-Cytotoxic; Homo_633,totalSample,immune-DC; MCSA,totalSample,immune-DC; MCSA,totalSample,immune-Monocyte; MCSA,totalSample,immune-Macrophage;

TMEM67	Homo_723,female,AD.vs.control; Homo_723,male,AD.vs.control; Homo_723,female,AD.vs.MCI; Homo_723,male,AD.vs.MCI; Homo_723,female,MCI.vs.control; Homo_714,female,AD.vs.control; Homo_714,male,AD.vs.control; Homo_714,female,AD.vs.MCI; Homo_714,male,AD.vs.MCI; Homo_714,female,MCI.vs.control; Homo_633,female,AD.vs.control; Homo_633,female,AD.vs.MCI; ROSMAP,AD,last.visit.age; Emory,totalSample,immune-Macrophage; Emory,totalSample,immune-Neutrophil; Emory,totalSample,immune-CD4_T; Emory,totalSample,immune-CD8_T; Emory,totalSample,immune-Tr1; Emory,totalSample,immune-iTreg; Emory,totalSample,immune-Tfh; Emory,totalSample,immune-CD8_naive; Emory,totalSample,immune-InfiltrationScore; Homo_723,totalSample,immune-CD4_naive; Homo_723,totalSample,immune-CD4_T; Homo_723,totalSample,immune-Tr1; Homo_723,totalSample,immune-Tfh; Homo_723,totalSample,immune-Central_memory; Homo_723,totalSample,immune-iTreg; Homo_723,totalSample,immune-Th2; Homo_723,totalSample,immune-nTreg; Homo_723,totalSample,immune-Cytotoxic; Homo_723,totalSample,immune-Th17; Homo_723,totalSample,immune-Th1; Homo_723,totalSample,immune-Exhausted; Homo_723,totalSample,immune-Neutrophil; Homo_723,totalSample,immune-NKT; Homo_723,totalSample,immune-InfiltrationScore; Homo_714,totalSample,immune-CD4_naive; Homo_714,totalSample,immune-CD4_T; Homo_714,totalSample,immune-Tfh; Homo_714,totalSample,immune-Central_memory; Homo_714,totalSample,immune-Tr1; Homo_714,totalSample,immune-iTreg; Homo_714,totalSample,immune-NKT; Homo_714,totalSample,immune-Th2; Homo_714,totalSample,immune-nTreg; Homo_714,totalSample,immune-Th1; Homo_714,totalSample,immune-InfiltrationScore; Homo_714,totalSample,immune-Th17; Homo_714,totalSample,immune-Effector_memory; Homo_714,totalSample,immune-Gamma_delta; Homo_633,totalSample,immune-Tfh; Homo_633,totalSample,immune-CD4_T; Homo_633,totalSample,immune-CD4_naive; Homo_633,totalSample,immune-Tr1; Homo_633,totalSample,immune-Central_memory; Homo_633,totalSample,immune-Effector_memory; Homo_633,totalSample,immune-iTreg; Homo_633,totalSample,immune-NKT; Homo_633,totalSample,immune-Th2; Homo_633,totalSample,immune-nTreg; Homo_633,totalSample,immune-InfiltrationScore; Homo_633,totalSample,immune-DC; Homo_633,totalSample,immune-Gamma_delta; Homo_633,totalSample,immune-MAIT; Homo_633,totalSample,immune-Th1; Homo_633,totalSample,immune-Th17; MCSA,totalSample,immune-DC; MCSA,totalSample,immune-Bcell; MCSA,totalSample,immune-Macrophage; MCSA,totalSample,immune-CD4_T; MCSA,totalSample,immune-CD4_naive; MCSA,totalSample,immune-Tr1; MCSA,totalSample,immune-nTreg; MCSA,totalSample,immune-iTreg; MCSA,totalSample,immune-Th17; MCSA,totalSample,immune-Tfh; MCSA,totalSample,immune-CD8_naive; MCSA,totalSample,immune-Cytotoxic; MCSA,totalSample,immune-Exhausted; MCSA,totalSample,immune-MAIT; MCSA,totalSample,immune-Central_memory; [illegible]
FICD	Homo_723,female,AD.vs.control; Homo_723,male,AD.vs.control; Homo_723,female,AD.vs.MCI; Homo_723,male,AD.vs.MCI; Homo_714,female,AD.vs.control; Homo_714,male,AD.vs.control; Homo_714,female,AD.vs.MCI; Homo_714,male,AD.vs.MCI; Homo_633,female,AD.vs.control; Homo_633,female,AD.vs.MCI; ROSMAP,male,MCI.vs.control; MCSA,MCI,age; Emory,totalSample,immune-Macrophage; Emory,totalSample,immune-Neutrophil; Emory,totalSample,immune-CD4_T; Emory,totalSample,immune-Tr1; Emory,totalSample,immune-iTreg; Emory,totalSample,immune-Effector_memory; Emory,totalSample,immune-InfiltrationScore; Homo_723,totalSample,immune-InfiltrationScore; Homo_723,totalSample,immune-iTreg; Homo_723,totalSample,immune-Tfh; Homo_723,totalSample,immune-CD4_naive; Homo_723,totalSample,immune-Central_memory; Homo_723,totalSample,immune-Tr1; Homo_723,totalSample,immune-CD4_T; Homo_723,totalSample,immune-MAIT; Homo_723,totalSample,immune-CD8_T; Homo_723,totalSample,immune-Monocyte; Homo_723,totalSample,immune-Effector_memory; Homo_723,totalSample,immune-Exhausted; Homo_723,totalSample,immune-nTreg; Homo_723,totalSample,immune-Macrophage; Homo_723,totalSample,immune-Th2; Homo_723,totalSample,immune-CD8_naive; Homo_723,totalSample,immune-NKT; Homo_714,totalSample,immune-InfiltrationScore; Homo_714,totalSample,immune-Tfh; Homo_714,totalSample,immune-Effector_memory; Homo_714,totalSample,immune-iTreg; Homo_714,totalSample,immune-Tr1; Homo_714,totalSample,immune-CD4_T; Homo_714,totalSample,immune-CD4_naive; Homo_714,totalSample,immune-nTreg; Homo_714,totalSample,immune-Th2; Homo_714,totalSample,immune-Central_memory; Homo_714,totalSample,immune-Monocyte; Homo_714,totalSample,immune-Macrophage; Homo_714,totalSample,immune-NKT; Homo_633,totalSample,immune-InfiltrationScore; Homo_633,totalSample,immune-NKT; Homo_633,totalSample,immune-Tfh; Homo_633,totalSample,immune-CD4_naive; Homo_633,totalSample,immune-Effector_memory; Homo_633,totalSample,immune-Tr1; Homo_633,totalSample,immune-CD4_T; Homo_633,totalSample,immune-iTreg; Homo_633,totalSample,immune-nTreg; Homo_633,totalSample,immune-Central_memory; Homo_633,totalSample,immune-Monocyte; MCSA,totalSample,immune-Neutrophil; MCSA,totalSample,immune-CD4_T; MCSA,totalSample,immune-NKT; MCSA,totalSample,immune-CD4_naive; MCSA,totalSample,immune-Tr1; MCSA,totalSample,immune-nTreg; MCSA,totalSample,immune-iTreg; MCSA,totalSample,immune-Th17; MCSA,totalSample,immune-Tfh; MCSA,totalSample,immune-CD8_naive; MCSA,totalSample,immune-Exhausted; MCSA,totalSample,immune-MAIT; MCSA,totalSample,immune-[illegible]
LEFTY1	Homo_723,female,AD.vs.control; Homo_723,male,AD.vs.control; Homo_723,female,AD.vs.MCI; Homo_723,male,AD.vs.MCI; Homo_723,MCI,male.vs.female; Homo_714,female,AD.vs.control; Homo_714,male,AD.vs.control; Homo_714,female,AD.vs.MCI; Homo_714,male,AD.vs.MCI; Homo_714,MCI,male.vs.female; Homo_633,female,AD.vs.control; Homo_633,female,AD.vs.MCI; Homo_633,MCI,male.vs.female; ROSMAP,female,AD.vs.control; Emory,MCI,age; MCSA,MCI,age; ROSMAP,AD,last.visit.age; ROSMAP,AD,sample.age; Emory,totalSample,immune-Effector_memory; Homo_723,totalSample,immune-DC; Homo_714,totalSample,immune-DC; Homo_633,totalSample,immune-DC; MCSA,totalSample,immune-DC; MCSA,totalSample,immune-Monocyte; MCSA,totalSample,immune-Th2; SRP223445,totalSample,immune-CD4_naive; ROSMAP,male-excludeAge90+,survival
SALL2	Homo_723,female,AD.vs.control; Homo_723,male,AD.vs.control; Homo_723,female,AD.vs.MCI; Homo_723,male,AD.vs.MCI; Homo_723,female,MCI.vs.control; Homo_714,female,AD.vs.control; Homo_714,male,AD.vs.control; Homo_714,female,AD.vs.MCI; Homo_714,male,AD.vs.MCI; Homo_633,female,AD.vs.control; Homo_633,female,AD.vs.MCI; Homo_633,female,MCI.vs.control; Homo_633,male,MCI.vs.control; MCSA,totalSample,age; MCSA,control,age; MCSA,MCI,age; Emory,totalSample,immune-Monocyte; Emory,totalSample,immune-Neutrophil; Emory,totalSample,immune-CD4_T; Emory,totalSample,immune-CD8_T; Emory,totalSample,immune-CD4_naive; Emory,totalSample,immune-Tr1; Emory,totalSample,immune-nTreg; Emory,totalSample,immune-iTreg; Emory,totalSample,immune-Tfh; Emory,totalSample,immune-MAIT; Homo_723,totalSample,immune-Monocyte; Homo_723,totalSample,immune-DC; Homo_723,totalSample,immune-nTreg; Homo_723,totalSample,immune-Central_memory; Homo_723,totalSample,immune-CD4_naive; Homo_723,totalSample,immune-Neutrophil; Homo_723,totalSample,immune-Th1; Homo_723,totalSample,immune-CD4_T; Homo_714,totalSample,immune-Monocyte; Homo_714,totalSample,immune-Th1; Homo_714,totalSample,immune-CD4_naive; Homo_714,totalSample,immune-nTreg; Homo_714,totalSample,immune-NKT; Homo_633,totalSample,immune-Monocyte; Homo_633,totalSample,immune-nTreg; Homo_633,totalSample,immune-Tfh; Homo_633,totalSample,immune-CD4_naive; Homo_633,totalSample,immune-Tr1; Homo_633,totalSample,immune-iTreg; Homo_633,totalSample,immune-CD4_T; Homo_633,totalSample,immune-Central_memory; MCSA,totalSample,immune-Monocyte; MCSA,totalSample,immune-Neutrophil; MCSA,totalSample,immune-CD4_T; MCSA,totalSample,immune-CD8_T; MCSA,totalSample,immune-CD4_naive; MCSA,totalSample,immune-Tr1; MCSA,totalSample,immune-nTreg; MCSA,totalSample,immune-iTreg; MCSA,totalSample,immune-Th17; MCSA,totalSample,immune-Tfh; MCSA,totalSample,immune-Central_memory; MCSA,totalSample,immune-[illegible]
OLFM1	Homo_723,female,AD.vs.control; Homo_723,male,AD.vs.control; Homo_723,female,AD.vs.MCI; Homo_723,male,AD.vs.MCI; Homo_714,female,AD.vs.control; Homo_633,female,AD.vs.control; Homo_633,female,AD.vs.MCI; ROSMAP,female,AD.vs.control; ROSMAP,male,AD.vs.control; Emory,MCI,age; Homo_723,totalSample,immune-DC; Homo_714,totalSample,immune-DC; Homo_633,totalSample,immune-DC; SRP223445,totalSample,immune-Neutrophil; SRP223445,totalSample,immune-Exhausted; SRP223445,totalSample,immune-InfiltrationScore
DOCK7	Homo_723,female,AD.vs.control; Homo_723,male,AD.vs.control; Homo_723,female,AD.vs.MCI; Homo_723,male,AD.vs.MCI; Homo_723,female,MCI.vs.control; Homo_723,male,MCI.vs.control; Homo_714,female,AD.vs.control; Homo_714,male,AD.vs.control; Homo_714,female,AD.vs.MCI; Homo_714,male,AD.vs.MCI; Homo_714,female,MCI.vs.control; Homo_633,female,AD.vs.control; Homo_633,female,AD.vs.MCI; Emory,totalSample,immune-Neutrophil; Emory,totalSample,immune-CD4_T; Emory,totalSample,immune-Tr1; Emory,totalSample,immune-iTreg; Emory,totalSample,immune-InfiltrationScore; Homo_723,totalSample,immune-Exhausted; Homo_723,totalSample,immune-CD4_naive; Homo_723,totalSample,immune-Th17; Homo_723,totalSample,immune-CD4_T; Homo_723,totalSample,immune-Th2; Homo_723,totalSample,immune-Tfh; Homo_723,totalSample,immune-Tr1; Homo_723,totalSample,immune-Cytotoxic; Homo_723,totalSample,immune-Central_memory; Homo_723,totalSample,immune-nTreg; Homo_723,totalSample,immune-iTreg; Homo_723,totalSample,immune-Effector_memory; Homo_723,totalSample,immune-InfiltrationScore; Homo_723,totalSample,immune-CD8_T; Homo_723,totalSample,immune-Th1; Homo_723,totalSample,immune-Neutrophil; Homo_714,totalSample,immune-CD4_naive; Homo_714,totalSample,immune-Central_memory; Homo_714,totalSample,immune-CD4_T; Homo_714,totalSample,immune-Th17; Homo_714,totalSample,immune-Tfh; Homo_714,totalSample,immune-Exhausted; Homo_714,totalSample,immune-InfiltrationScore; Homo_714,totalSample,immune-Th2; Homo_714,totalSample,immune-Effector_memory; Homo_714,totalSample,immune-Tr1; Homo_714,totalSample,immune-iTreg; Homo_714,totalSample,immune-Monocyte; Homo_714,totalSample,immune-Gamma_delta; Homo_633,totalSample,immune-Tfh; Homo_633,totalSample,immune-Th2; Homo_633,totalSample,immune-CD4_T; Homo_633,totalSample,immune-Effector_memory; Homo_633,totalSample,immune-CD4_naive; Homo_633,totalSample,immune-Central_memory; Homo_633,totalSample,immune-InfiltrationScore; Homo_633,totalSample,immune-Tr1; Homo_633,totalSample,immune-Th17; Homo_633,totalSample,immune-Gamma_delta; Homo_633,totalSample,immune-Exhausted; Homo_633,totalSample,immune-iTreg; Homo_633,totalSample,immune-NKT; Homo_633,totalSample,immune-Monocyte; Homo_633,totalSample,immune-nTreg; Homo_633,totalSample,immune-Bcell; MCSA,totalSample,immune-DC; MCSA,totalSample,immune-Macrophage; MCSA,totalSample,immune-CD4_T; MCSA,totalSample,immune-CD4_naive; MCSA,totalSample,immune-Tr1; MCSA,totalSample,immune-nTreg; MCSA,totalSample,immune-iTreg; MCSA,totalSample,immune-Th17; MCSA,totalSample,immune-Tfh; MCSA,totalSample,immune-CD8_naive; MCSA,totalSample,immune-Cytotoxic; MCSA,totalSample,immune-Exhausted; MCSA,totalSample,immune-MAIT; MCSA,totalSample,immune-Central_memory; ROSMAP,totalSample,immune-Effector_memory; SRP223445,totalSample,immune-CD4_naive
NECTIN3	Homo_723,female,AD.vs.control; Homo_723,male,AD.vs.control; Homo_723,female,AD.vs.MCI; Homo_723,male,AD.vs.MCI; Homo_723,female,MCI.vs.control; Homo_723,male,MCI.vs.control; Homo_714,male,AD.vs.control; Homo_714,female,AD.vs.MCI; Homo_714,male,AD.vs.MCI; Homo_633,female,AD.vs.control; Homo_633,female,AD.vs.MCI; ROSMAP,female,AD.vs.control; Emory,totalSample,immune-Macrophage; Emory,totalSample,immune-Neutrophil; Emory,totalSample,immune-CD4_T; Emory,totalSample,immune-Tr1; Emory,totalSample,immune-iTreg; Emory,totalSample,immune-Tfh; Emory,totalSample,immune-CD8_naive; Homo_723,totalSample,immune-Tfh; Homo_723,totalSample,immune-CD4_T; Homo_723,totalSample,immune-CD4_naive; Homo_723,totalSample,immune-Th2; Homo_723,totalSample,immune-iTreg; Homo_723,totalSample,immune-Tr1; Homo_723,totalSample,immune-Th17; Homo_723,totalSample,immune-nTreg; Homo_723,totalSample,immune-Central_memory; Homo_723,totalSample,immune-Effector_memory; Homo_723,totalSample,immune-InfiltrationScore; Homo_723,totalSample,immune-Th1; Homo_723,totalSample,immune-Exhausted; Homo_723,totalSample,immune-Neutrophil; Homo_714,totalSample,immune-Tfh; Homo_714,totalSample,immune-CD4_T; Homo_714,totalSample,immune-CD4_naive; Homo_714,totalSample,immune-Th2; Homo_714,totalSample,immune-Central_memory; Homo_714,totalSample,immune-InfiltrationScore; Homo_714,totalSample,immune-Effector_memory; Homo_714,totalSample,immune-Tr1; Homo_714,totalSample,immune-iTreg; Homo_714,totalSample,immune-Th17; Homo_714,totalSample,immune-nTreg; Homo_714,totalSample,immune-MAIT; Homo_714,totalSample,immune-Neutrophil; Homo_714,totalSample,immune-NKT; Homo_714,totalSample,immune-Bcell; Homo_633,totalSample,immune-Tfh; Homo_633,totalSample,immune-CD4_T; Homo_633,totalSample,immune-CD4_naive; Homo_633,totalSample,immune-Central_memory; Homo_633,totalSample,immune-Effector_memory; Homo_633,totalSample,immune-Th2; Homo_633,totalSample,immune-InfiltrationScore; Homo_633,totalSample,immune-Tr1; Homo_633,totalSample,immune-MAIT; Homo_633,totalSample,immune-iTreg; Homo_633,totalSample,immune-Th17; Homo_633,totalSample,immune-nTreg; Homo_633,totalSample,immune-NKT; Homo_633,totalSample,immune-DC; Homo_633,totalSample,immune-Bcell; Homo_633,totalSample,immune-Neutrophil; MCSA,totalSample,immune-DC; MCSA,totalSample,immune-Macrophage; MCSA,totalSample,immune-CD4_T; MCSA,totalSample,immune-CD4_naive; MCSA,totalSample,immune-Tr1; MCSA,totalSample,immune-nTreg; MCSA,totalSample,immune-iTreg; MCSA,totalSample,immune-Th17; MCSA,totalSample,immune-Tfh; MCSA,totalSample,immune-CD8_naive; MCSA,totalSample,immune-Cytotoxic; MCSA,totalSample,immune-Exhausted; MCSA,totalSample,immune-MAIT; MCSA,totalSample,immune-Central_memory; MCSA,totalSample,immune-Effector_memory; ROSMAP,totalSample,immune-InfiltrationScore
SUCO	Homo_723,female,AD.vs.control; Homo_723,male,AD.vs.control; Homo_723,female,AD.vs.MCI; Homo_723,male,AD.vs.MCI; Homo_723,female,MCI.vs.control; Homo_714,female,AD.vs.control; Homo_714,male,AD.vs.control; Homo_714,female,AD.vs.MCI; Homo_714,male,AD.vs.MCI; Homo_714,female,MCI.vs.control; Homo_633,female,AD.vs.control; Homo_633,female,AD.vs.MCI; SRP223445,female,AD.vs.control; ACOM,totalSample,age; MCSA,MCI,age; ROSMAP,MCI,MMSE; Emory,totalSample,immune-DC; Emory,totalSample,immune-Monocyte; Emory,totalSample,immune-Macrophage; Emory,totalSample,immune-CD4_T; Emory,totalSample,immune-Tr1; Emory,totalSample,immune-iTreg; Emory,totalSample,immune-Tfh; Emory,totalSample,immune-Central_memory; Emory,totalSample,immune-Effector_memory; Emory,totalSample,immune-InfiltrationScore; Homo_723,totalSample,immune-Exhausted; Homo_723,totalSample,immune-Th17; Homo_723,totalSample,immune-Cytotoxic; Homo_723,totalSample,immune-CD4_naive; Homo_723,totalSample,immune-CD4_T; Homo_723,totalSample,immune-CD8_T; Homo_723,totalSample,immune-Tr1; Homo_723,totalSample,immune-Tfh; Homo_723,totalSample,immune-Central_memory; Homo_723,totalSample,immune-Th1; Homo_723,totalSample,immune-Th2; Homo_723,totalSample,immune-Monocyte; Homo_723,totalSample,immune-NKT; Homo_723,totalSample,immune-Gamma_delta; Homo_714,totalSample,immune-CD4_naive; Homo_714,totalSample,immune-Th17; Homo_714,totalSample,immune-Exhausted; Homo_714,totalSample,immune-Monocyte; Homo_714,totalSample,immune-Central_memory; Homo_714,totalSample,immune-CD4_T; Homo_714,totalSample,immune-Tfh; Homo_714,totalSample,immune-NKT; Homo_714,totalSample,immune-Tr1; Homo_714,totalSample,immune-Gamma_delta; Homo_714,totalSample,immune-Cytotoxic; Homo_714,totalSample,immune-Th1; Homo_633,totalSample,immune-Tfh; Homo_633,totalSample,immune-CD4_T; Homo_633,totalSample,immune-Th2; Homo_633,totalSample,immune-Central_memory; Homo_633,totalSample,immune-CD4_naive; Homo_633,totalSample,immune-Effector_memory; Homo_633,totalSample,immune-Monocyte; Homo_633,totalSample,immune-Tr1; Homo_633,totalSample,immune-NKT; Homo_633,totalSample,immune-Gamma_delta; Homo_633,totalSample,immune-Exhausted; Homo_633,totalSample,immune-InfiltrationScore; Homo_633,totalSample,immune-iTreg; Homo_633,totalSample,immune-DC; Homo_633,totalSample,immune-Th17; MCSA,totalSample,immune-DC; MCSA,totalSample,immune-Monocyte; MCSA,totalSample,immune-Macrophage; MCSA,totalSample,immune-CD4_T; MCSA,totalSample,immune-CD8_T; MCSA,totalSample,immune-CD4_naive; MCSA,totalSample,immune-Tr1; MCSA,totalSample,immune-nTreg; MCSA,totalSample,immune-iTreg; MCSA,totalSample,immune-Tfh; MCSA,totalSample,immune-CD8_naive; MCSA,totalSample,immune-Cytotoxic; MCSA,totalSample,immune-Exhausted; MCSA,totalSample,immune-MAIT; MCSA,totalSample,immune-Central_memory; SRP223445,totalSample,immune-Neutrophil; [illegible]
HECTD2	Homo_723,female,AD.vs.control; Homo_723,male,AD.vs.control; Homo_723,female,AD.vs.MCI; Homo_723,male,AD.vs.MCI; Homo_723,female,MCI.vs.control; Homo_723,male,MCI.vs.control; Homo_714,female,AD.vs.control; Homo_714,male,AD.vs.control; Homo_714,female,AD.vs.MCI; Homo_714,male,AD.vs.MCI; Homo_714,female,MCI.vs.control; Homo_714,male,MCI.vs.control; Homo_633,female,AD.vs.control; Homo_633,female,AD.vs.MCI; MCSA,MCI,age; Emory,totalSample,immune-Macrophage; Emory,totalSample,immune-NK; Emory,totalSample,immune-Neutrophil; Emory,totalSample,immune-CD4_T; Emory,totalSample,immune-Tr1; Homo_723,totalSample,immune-CD4_naive; Homo_723,totalSample,immune-CD4_T; Homo_723,totalSample,immune-Th1; Homo_723,totalSample,immune-Neutrophil; Homo_723,totalSample,immune-Cytotoxic; Homo_723,totalSample,immune-Th17; Homo_723,totalSample,immune-Exhausted; Homo_723,totalSample,immune-Tr1; Homo_723,totalSample,immune-Tfh; Homo_723,totalSample,immune-Central_memory; Homo_714,totalSample,immune-CD4_naive; Homo_714,totalSample,immune-NKT; Homo_714,totalSample,immune-Central_memory; Homo_714,totalSample,immune-CD4_T; Homo_714,totalSample,immune-Th1; Homo_714,totalSample,immune-Tfh; Homo_714,totalSample,immune-Neutrophil; Homo_714,totalSample,immune-InfiltrationScore; Homo_633,totalSample,immune-Tfh; Homo_633,totalSample,immune-CD4_T; Homo_633,totalSample,immune-CD4_naive; Homo_633,totalSample,immune-Central_memory; Homo_633,totalSample,immune-Effector_memory; Homo_633,totalSample,immune-NKT; Homo_633,totalSample,immune-Tr1; Homo_633,totalSample,immune-InfiltrationScore; Homo_633,totalSample,immune-Th2; Homo_633,totalSample,immune-iTreg; Homo_633,totalSample,immune-MAIT; Homo_633,totalSample,immune-Neutrophil; MCSA,totalSample,immune-DC; MCSA,totalSample,immune-Bcell; MCSA,totalSample,immune-Monocyte; MCSA,totalSample,immune-Macrophage; MCSA,totalSample,immune-CD4_T; MCSA,totalSample,immune-CD4_naive; MCSA,totalSample,immune-Tr1; MCSA,totalSample,immune-nTreg; MCSA,totalSample,immune-iTreg; MCSA,totalSample,immune-Tfh; MCSA,totalSample,immune-CD8_naive; MCSA,totalSample,immune-Cytotoxic; MCSA,totalSample,immune-Exhausted; MCSA,totalSample,immune-MAIT; MCSA,totalSample,immune-[illegible]
AR	Homo_723,female,AD.vs.control; Homo_723,male,AD.vs.control; Homo_723,female,AD.vs.MCI; Homo_723,male,AD.vs.MCI; Homo_723,female,MCI.vs.control; Homo_723,male,MCI.vs.control; Homo_723,MCI,male.vs.female; Homo_714,female,AD.vs.control; Homo_714,male,AD.vs.control; Homo_714,female,AD.vs.MCI; Homo_714,male,AD.vs.MCI; Homo_714,female,MCI.vs.control; Homo_714,male,MCI.vs.control; Homo_714,control,male.vs.female; Homo_714,MCI,male.vs.female; Homo_633,female,AD.vs.control; Homo_633,female,AD.vs.MCI; Homo_633,MCI,male.vs.female; ROSMAP,control,male.vs.female; ROSMAP,MCI,male.vs.female; Homo_723,totalSample,immune-Th17; Homo_723,totalSample,immune-Exhausted; Homo_723,totalSample,immune-Cytotoxic; Homo_723,totalSample,immune-CD8_T; Homo_723,totalSample,immune-CD4_naive; Homo_723,totalSample,immune-MAIT; Homo_723,totalSample,immune-DC; Homo_714,totalSample,immune-Th17; Homo_714,totalSample,immune-Exhausted; Homo_714,totalSample,immune-CD8_T; Homo_714,totalSample,immune-Cytotoxic; Homo_633,totalSample,immune-Tfh; Homo_633,totalSample,immune-Th2; Homo_633,totalSample,immune-Gamma_delta; Homo_633,totalSample,immune-Th17; Homo_633,totalSample,immune-CD4_T; Homo_633,totalSample,immune-Exhausted; MCSA,totalSample,immune-CD4_T; MCSA,totalSample,immune-Gamma_delta; MCSA,totalSample,immune-CD4_naive; MCSA,totalSample,immune-Tr1; MCSA,totalSample,immune-nTreg; MCSA,totalSample,immune-iTreg; MCSA,totalSample,immune-Th17; MCSA,totalSample,immune-Tfh; MCSA,totalSample,immune-Cytotoxic; MCSA,totalSample,immune-Exhausted; MCSA,totalSample,immune-Central_memory; ROSMAP,male and female-[illegible]
NRP2	Homo_723,female,AD.vs.control; Homo_723,male,AD.vs.control; Homo_723,female,AD.vs.MCI; Homo_723,male,AD.vs.MCI; Homo_723,female,MCI.vs.control; Homo_723,male,MCI.vs.control; Homo_714,female,AD.vs.control; Homo_714,male,AD.vs.control; Homo_714,female,AD.vs.MCI; Homo_714,male,AD.vs.MCI; Homo_714,female,MCI.vs.control; Homo_714,male,MCI.vs.control; Homo_633,female,AD.vs.control; Homo_633,female,AD.vs.MCI; ROSMAP,male,AD.vs.control; ROSMAP,AD,male.vs.female; MCSA,MCI,age; ROSMAP,MCI,last.visit.age; ROSMAP,AD,MMSE; Homo_723,totalSample,immune-Th17; Homo_723,totalSample,immune-Exhausted; Homo_723,totalSample,immune-Cytotoxic; Homo_723,totalSample,immune-CD4_naive; Homo_723,totalSample,immune-CD8_T; Homo_723,totalSample,immune-InfiltrationScore; Homo_723,totalSample,immune-CD4_T; Homo_714,totalSample,immune-Exhausted; Homo_714,totalSample,immune-Th17; Homo_714,totalSample,immune-InfiltrationScore; Homo_714,totalSample,immune-CD4_naive; Homo_714,totalSample,immune-NKT; Homo_633,totalSample,immune-Tfh; Homo_633,totalSample,immune-CD4_T; Homo_633,totalSample,immune-InfiltrationScore; Homo_633,totalSample,immune-Gamma_delta; Homo_633,totalSample,immune-Exhausted; Homo_633,totalSample,immune-Effector_memory; Homo_633,totalSample,immune-CD4_naive; Homo_633,totalSample,immune-Th17; Homo_633,totalSample,immune-Th2; Homo_633,totalSample,immune-NKT; Homo_633,totalSample,immune-Tr1; MCSA,totalSample,immune-CD4_T; MCSA,totalSample,immune-Gamma_delta; MCSA,totalSample,immune-CD4_naive; MCSA,totalSample,immune-Tr1; MCSA,totalSample,immune-nTreg; MCSA,totalSample,immune-iTreg; MCSA,totalSample,immune-Th17; MCSA,totalSample,immune-Tfh; MCSA,totalSample,immune-Cytotoxic; MCSA,totalSample,immune-Exhausted; MCSA,totalSample,immune-Central_memory; SRP223445,totalSample,immune-Neutrophil; SRP223445,totalSample,immune-CD4_naive; SRP223445,totalSample,immune-nTreg; SRP223445,totalSample,immune-[illegible]
MRPS10	Homo_723,female,AD.vs.control; Homo_723,male,AD.vs.control; Homo_723,female,AD.vs.MCI; Homo_723,male,AD.vs.MCI; Homo_714,female,AD.vs.control; Homo_714,male,AD.vs.control; Homo_714,female,AD.vs.MCI; Homo_714,male,AD.vs.MCI; Homo_633,female,AD.vs.control; Homo_633,female,AD.vs.MCI; ROSMAP,female,AD.vs.control; MCSA,MCI,age; ROSMAP,AD,last.visit.age; ROSMAP,MCI,last.visit.age; Emory,totalSample,immune-Macrophage; Emory,totalSample,immune-Neutrophil; Emory,totalSample,immune-CD4_T; Emory,totalSample,immune-CD8_T; Emory,totalSample,immune-Tr1; Emory,totalSample,immune-iTreg; Emory,totalSample,immune-Tfh; Emory,totalSample,immune-Central_memory; Emory,totalSample,immune-Effector_memory; Emory,totalSample,immune-InfiltrationScore; Homo_723,totalSample,immune-Th2; Homo_723,totalSample,immune-Tfh; Homo_723,totalSample,immune-iTreg; Homo_723,totalSample,immune-Monocyte; Homo_723,totalSample,immune-DC; Homo_723,totalSample,immune-Effector_memory; Homo_723,totalSample,immune-Tr1; Homo_723,totalSample,immune-CD4_T; Homo_723,totalSample,immune-InfiltrationScore; Homo_723,totalSample,immune-CD4_naive; Homo_723,totalSample,immune-MAIT; Homo_723,totalSample,immune-Bcell; Homo_723,totalSample,immune-Macrophage; Homo_714,totalSample,immune-Th2; Homo_714,totalSample,immune-Tfh; Homo_714,totalSample,immune-Effector_memory; Homo_714,totalSample,immune-Tr1; Homo_714,totalSample,immune-CD4_T; Homo_714,totalSample,immune-Monocyte; Homo_714,totalSample,immune-Central_memory; Homo_714,totalSample,immune-InfiltrationScore; Homo_714,totalSample,immune-CD4_naive; Homo_714,totalSample,immune-iTreg; Homo_633,totalSample,immune-Tfh; Homo_633,totalSample,immune-Monocyte; Homo_633,totalSample,immune-Th2; Homo_633,totalSample,immune-CD4_T; Homo_633,totalSample,immune-Effector_memory; Homo_633,totalSample,immune-Central_memory; Homo_633,totalSample,immune-CD4_naive; Homo_633,totalSample,immune-InfiltrationScore; Homo_633,totalSample,immune-Tr1; Homo_633,totalSample,immune-Exhausted; Homo_633,totalSample,immune-Bcell; Homo_633,totalSample,immune-Th17; MCSA,totalSample,immune-DC; MCSA,totalSample,immune-Monocyte; MCSA,totalSample,immune-Macrophage; MCSA,totalSample,immune-CD4_T; MCSA,totalSample,immune-CD4_naive; MCSA,totalSample,immune-Tr1; MCSA,totalSample,immune-nTreg; MCSA,totalSample,immune-iTreg; MCSA,totalSample,immune-Th2; MCSA,totalSample,immune-Tfh; MCSA,totalSample,immune-CD8_naive; MCSA,totalSample,immune-Cytotoxic; MCSA,totalSample,immune-Exhausted; MCSA,totalSample,immune-MAIT; MCSA,totalSample,immune-Central_memory; SRP223445,totalSample,immune-[illegible]

RETREG1	Homo_723,female,AD.vs.control; Homo_723,male,AD.vs.control; Homo_723,female,AD.vs.MCI; Homo_723,male,AD.vs.MCI; Homo_714,female,AD.vs.control; Homo_714,male,AD.vs.control; Homo_714,female,AD.vs.MCI; Homo_714,male,AD.vs.MCI; Homo_633,female,AD.vs.control; Homo_633,female,AD.vs.MCI; ROSMAP,female,MCI.vs.control; Emory,totalSample,age; Emory,control,age; Emory,MCI,age; MCSA,MCI,age; Emory,totalSample,immune-DC; Emory,totalSample,immune-Macrophage; Emory,totalSample,immune-Neutrophil; Emory,totalSample,immune-CD4_T; Emory,totalSample,immune-CD8_T; Emory,totalSample,immune-CD4_naive; Emory,totalSample,immune-Tr1; Emory,totalSample,immune-nTreg; Emory,totalSample,immune-iTreg; Emory,totalSample,immune-Th17; Emory,totalSample,immune-Tfh; Emory,totalSample,immune-CD8_naive; Emory,totalSample,immune-MAIT; Emory,totalSample,immune-Central_memory; Emory,totalSample,immune-InfiltrationScore; Homo_723,totalSample,immune-iTreg; Homo_723,totalSample,immune-Tfh; Homo_723,totalSample,immune-Th2; Homo_723,totalSample,immune-Tr1; Homo_723,totalSample,immune-CD4_T; Homo_723,totalSample,immune-CD4_naive; Homo_723,totalSample,immune-Central_memory; Homo_723,totalSample,immune-DC; Homo_723,totalSample,immune-Gamma_delta; Homo_723,totalSample,immune-nTreg; Homo_723,totalSample,immune-Macrophage; Homo_723,totalSample,immune-Effector_memory; Homo_723,totalSample,immune-MAIT; Homo_723,totalSample,immune-Monocyte; Homo_723,totalSample,immune-InfiltrationScore; Homo_714,totalSample,immune-Tfh; Homo_714,totalSample,immune-Th2; Homo_714,totalSample,immune-Tr1; Homo_714,totalSample,immune-CD4_T; Homo_714,totalSample,immune-iTreg; Homo_714,totalSample,immune-Central_memory; Homo_714,totalSample,immune-CD4_naive; Homo_714,totalSample,immune-nTreg; Homo_714,totalSample,immune-Gamma_delta; Homo_714,totalSample,immune-Effector_memory; Homo_714,totalSample,immune-DC; Homo_633,totalSample,immune-Tfh; Homo_633,totalSample,immune-CD4_T; Homo_633,totalSample,immune-Central_memory; Homo_633,totalSample,immune-Tr1; Homo_633,totalSample,immune-CD4_naive; Homo_633,totalSample,immune-Th2; Homo_633,totalSample,immune-iTreg; Homo_633,totalSample,immune-nTreg; Homo_633,totalSample,immune-Gamma_delta; Homo_633,totalSample,immune-Th17; Homo_633,totalSample,immune-Effector_memory; Homo_633,totalSample,immune-NKT; Homo_633,totalSample,immune-DC; Homo_633,totalSample,immune-Exhausted; Homo_633,totalSample,immune-Th1; Homo_633,totalSample,immune-Monocyte; Homo_633,totalSample,immune-Cytotoxic; MCSA,totalSample,immune-DC; MCSA,totalSample,immune-Monocyte; MCSA,totalSample,immune-Macrophage; MCSA,totalSample,immune-CD4_T; MCSA,totalSample,immune-CD4_naive; MCSA,totalSample,immune-Tr1; MCSA,totalSample,immune-nTreg; MCSA,totalSample,immune-iTreg; MCSA,totalSample,immune-Th17; MCSA,totalSample,immune-Tfh; MCSA,totalSample,immune-CD8_naive; MCSA,totalSample,immune-[illegible]
ZNF263	Homo_723,female,AD.vs.control; Homo_723,male,AD.vs.control; Homo_723,female,AD.vs.MCI; Homo_723,male,AD.vs.MCI; Homo_723,female,MCI.vs.control; Homo_714,female,AD.vs.control; Homo_714,male,AD.vs.control; Homo_714,female,AD.vs.MCI; Homo_714,male,AD.vs.MCI; Homo_714,female,MCI.vs.control; Homo_633,female,AD.vs.control; Homo_633,female,AD.vs.MCI; Homo_633,female,MCI.vs.control; MCSA,totalSample,age; MCSA,control,age; ROSMAP,MCI,last.visit.age; ROSMAP,MCI,sample.age; ROSMAP,MCI,MMSE; Emory,totalSample,immune-Macrophage; Emory,totalSample,immune-NK; Emory,totalSample,immune-Neutrophil; Emory,totalSample,immune-CD4_T; Emory,totalSample,immune-CD8_T; Emory,totalSample,immune-Tr1; Emory,totalSample,immune-iTreg; Emory,totalSample,immune-Tfh; Emory,totalSample,immune-Exhausted; Emory,totalSample,immune-Central_memory; Emory,totalSample,immune-Effector_memory; Emory,totalSample,immune-InfiltrationScore; Homo_723,totalSample,immune-Th2; Homo_723,totalSample,immune-Tfh; Homo_723,totalSample,immune-Effector_memory; Homo_723,totalSample,immune-iTreg; Homo_723,totalSample,immune-CD4_T; Homo_723,totalSample,immune-CD4_naive; Homo_723,totalSample,immune-Tr1; Homo_723,totalSample,immune-InfiltrationScore; Homo_723,totalSample,immune-nTreg; Homo_723,totalSample,immune-Macrophage; Homo_723,totalSample,immune-Bcell; Homo_723,totalSample,immune-Central_memory; Homo_723,totalSample,immune-Monocyte; Homo_723,totalSample,immune-DC; Homo_714,totalSample,immune-Th2; Homo_714,totalSample,immune-Tfh; Homo_714,totalSample,immune-Effector_memory; Homo_714,totalSample,immune-CD4_T; Homo_714,totalSample,immune-Tr1; Homo_714,totalSample,immune-Central_memory; Homo_714,totalSample,immune-nTreg; Homo_714,totalSample,immune-CD4_naive; Homo_714,totalSample,immune-InfiltrationScore; Homo_714,totalSample,immune-iTreg; Homo_714,totalSample,immune-Macrophage; Homo_714,totalSample,immune-CD8_T; Homo_633,totalSample,immune-Tfh; Homo_633,totalSample,immune-Th2; Homo_633,totalSample,immune-Effector_memory; Homo_633,totalSample,immune-CD4_T; Homo_633,totalSample,immune-Central_memory; Homo_633,totalSample,immune-CD4_naive; Homo_633,totalSample,immune-InfiltrationScore; Homo_633,totalSample,immune-nTreg; Homo_633,totalSample,immune-Tr1; Homo_633,totalSample,immune-Th17; Homo_633,totalSample,immune-Bcell; Homo_633,totalSample,immune-iTreg; MCSA,totalSample,immune-Monocyte; MCSA,totalSample,immune-Macrophage; MCSA,totalSample,immune-Neutrophil; MCSA,totalSample,immune-CD4_T; MCSA,totalSample,immune-CD8_T; MCSA,totalSample,immune-Gamma_delta; MCSA,totalSample,immune-CD4_naive; MCSA,totalSample,immune-Tr1; MCSA,totalSample,immune-nTreg; MCSA,totalSample,immune-iTreg; MCSA,totalSample,immune-Th17; MCSA,totalSample,immune-Tfh; MCSA,totalSample,immune-CD8_naive; MCSA,totalSample,immune-MAIT; MCSA,totalSample,immune-Central_memory; MCSA,totalSample,immune-[illegible]
ECHDC2	Homo_723,female,AD.vs.control; Homo_723,male,AD.vs.control; Homo_723,female,AD.vs.MCI; Homo_723,female,MCI.vs.control; Homo_723,male,MCI.vs.control; Homo_723,AD,male.vs.female; Homo_723,control,male.vs.female; Homo_714,female,AD.vs.control; Homo_714,male,AD.vs.control; Homo_714,female,AD.vs.MCI; Homo_714,female,MCI.vs.control; Homo_714,male,MCI.vs.control; Homo_714,control,male.vs.female; Homo_633,female,AD.vs.control; Homo_633,female,AD.vs.MCI; Homo_633,female,MCI.vs.control; Homo_633,male,MCI.vs.control; Homo_633,control,male.vs.female; ROSMAP,MCI,male.vs.female; MCSA,totalSample,age; MCSA,control,age; ROSMAP,AD,last.visit.age; Emory,totalSample,immune-Monocyte; Emory,totalSample,immune-Macrophage; Emory,totalSample,immune-NK; Emory,totalSample,immune-Neutrophil; Emory,totalSample,immune-CD4_T; Emory,totalSample,immune-CD8_T; Emory,totalSample,immune-Tr1; Emory,totalSample,immune-iTreg; Emory,totalSample,immune-Tfh; Emory,totalSample,immune-CD8_naive; Emory,totalSample,immune-Exhausted; Emory,totalSample,immune-MAIT; Emory,totalSample,immune-Central_memory; Emory,totalSample,immune-Effector_memory; Emory,totalSample,immune-InfiltrationScore; Homo_723,totalSample,immune-Monocyte; Homo_723,totalSample,immune-Exhausted; Homo_723,totalSample,immune-CD8_T; Homo_723,totalSample,immune-Neutrophil; Homo_723,totalSample,immune-DC; Homo_723,totalSample,immune-Central_memory; Homo_723,totalSample,immune-nTreg; Homo_723,totalSample,immune-CD4_naive; Homo_723,totalSample,immune-Tfh; Homo_714,totalSample,immune-Monocyte; Homo_714,totalSample,immune-Neutrophil; Homo_714,totalSample,immune-Macrophage; Homo_714,totalSample,immune-CD4_naive; Homo_714,totalSample,immune-nTreg; Homo_714,totalSample,immune-DC; Homo_714,totalSample,immune-CD8_T; Homo_714,totalSample,immune-Exhausted; Homo_633,totalSample,immune-Monocyte; Homo_633,totalSample,immune-Macrophage; Homo_633,totalSample,immune-nTreg; Homo_633,totalSample,immune-CD4_naive; Homo_633,totalSample,immune-Exhausted; Homo_633,totalSample,immune-Tfh; Homo_633,totalSample,immune-CD8_T; MCSA,totalSample,immune-Monocyte; MCSA,totalSample,immune-Macrophage; MCSA,totalSample,immune-Neutrophil; MCSA,totalSample,immune-CD4_T; MCSA,totalSample,immune-CD8_T; MCSA,totalSample,immune-CD4_naive; MCSA,totalSample,immune-Tr1; MCSA,totalSample,immune-nTreg; MCSA,totalSample,immune-iTreg; MCSA,totalSample,immune-Th17; MCSA,totalSample,immune-Tfh; MCSA,totalSample,immune-CD8_naive; MCSA,totalSample,immune-Cytotoxic; MCSA,totalSample,immune-Central_memory; MCSA,totalSample,immune-Effector_memory; MCSA,totalSample,immune-[illegible]
IL7R	Homo_723,female,AD.vs.control; Homo_723,male,AD.vs.control; Homo_723,female,AD.vs.MCI; Homo_723,male,AD.vs.MCI; Homo_723,female,MCI.vs.control; Homo_714,female,AD.vs.control; Homo_714,male,AD.vs.control; Homo_714,female,AD.vs.MCI; Homo_714,male,AD.vs.MCI; Homo_714,female,MCI.vs.control; Homo_633,female,AD.vs.control; Homo_633,female,AD.vs.MCI; Homo_633,female,MCI.vs.control; Emory,totalSample,age; Emory,control,age; Emory,MCI,age; MCSA,totalSample,age; MCSA,control,age; ROSMAP,AD,last.visit.age; ROSMAP,MCI,last.visit.age; ROSMAP,MCI,MMSE; Emory,totalSample,immune-Macrophage; Emory,totalSample,immune-Neutrophil; Emory,totalSample,immune-CD4_T; Emory,totalSample,immune-CD8_T; Emory,totalSample,immune-Tr1; Emory,totalSample,immune-nTreg; Emory,totalSample,immune-iTreg; Emory,totalSample,immune-Th17; Emory,totalSample,immune-Tfh; Emory,totalSample,immune-CD8_naive; Emory,totalSample,immune-Exhausted; Emory,totalSample,immune-MAIT; Emory,totalSample,immune-Central_memory; Emory,totalSample,immune-InfiltrationScore; Homo_723,totalSample,immune-Tfh; Homo_723,totalSample,immune-Th2; Homo_723,totalSample,immune-iTreg; Homo_723,totalSample,immune-CD4_T; Homo_723,totalSample,immune-Tr1; Homo_723,totalSample,immune-CD4_naive; Homo_723,totalSample,immune-DC; Homo_723,totalSample,immune-Effector_memory; Homo_723,totalSample,immune-Central_memory; Homo_723,totalSample,immune-nTreg; Homo_723,totalSample,immune-InfiltrationScore; Homo_723,totalSample,immune-Macrophage; Homo_723,totalSample,immune-Monocyte; Homo_723,totalSample,immune-Bcell; Homo_723,totalSample,immune-MAIT; Homo_723,totalSample,immune-Gamma_delta; Homo_714,totalSample,immune-Tfh; Homo_714,totalSample,immune-Th2; Homo_714,totalSample,immune-CD4_T; Homo_714,totalSample,immune-Tr1; Homo_714,totalSample,immune-Central_memory; Homo_714,totalSample,immune-Effector_memory; Homo_714,totalSample,immune-CD4_naive; Homo_714,totalSample,immune-iTreg; Homo_714,totalSample,immune-nTreg; Homo_714,totalSample,immune-MAIT; Homo_714,totalSample,immune-Macrophage; Homo_714,totalSample,immune-DC; Homo_714,totalSample,immune-Gamma_delta; Homo_714,totalSample,immune-Bcell; Homo_714,totalSample,immune-InfiltrationScore; Homo_633,totalSample,immune-Tfh; Homo_633,totalSample,immune-CD4_T; Homo_633,totalSample,immune-Th2; Homo_633,totalSample,immune-Central_memory; Homo_633,totalSample,immune-CD4_naive; Homo_633,totalSample,immune-Effector_memory; Homo_633,totalSample,immune-Tr1; Homo_633,totalSample,immune-Th17; Homo_633,totalSample,immune-nTreg; Homo_633,totalSample,immune-iTreg; Homo_633,totalSample,immune-InfiltrationScore; Homo_633,totalSample,immune-Bcell; Homo_633,totalSample,immune-Gamma_delta; Homo_633,totalSample,immune-Exhausted; Homo_633,totalSample,immune-Monocyte; Homo_633,totalSample,immune-NKT; Homo_633,totalSample,immune-MAIT; Homo_633,totalSample,immune-DC; MCSA,totalSample,immune-DC; MCSA,totalSample,immune-Macrophage; MCSA,totalSample,immune-Neutrophil; MCSA,totalSample,immune-CD4_T; MCSA,totalSample,immune-CD4_naive; MCSA,totalSample,immune-Tr1; MCSA,totalSample,immune-nTreg; MCSA,totalSample,immune-iTreg; MCSA,totalSample,immune-Th17; MCSA,totalSample,immune-Tfh; MCSA,totalSample,immune-CD8_naive; MCSA,totalSample,immune-Cytotoxic; MCSA,totalSample,immune-Exhausted; MCSA,totalSample,immune-MAIT; MCSA,totalSample,immune-Central_memory; MCSA,totalSample,immune-Effector_memory; ROSMAP,totalSample,immune-Macrophage; ROSMAP,totalSample,immune-NK; [illegible]
CZIB	Homo_723,female,AD.vs.control; Homo_723,male,AD.vs.control; Homo_723,female,AD.vs.MCI; Homo_723,male,AD.vs.MCI; Homo_714,female,AD.vs.control; Homo_714,male,AD.vs.control; Homo_714,female,AD.vs.MCI; Homo_714,male,AD.vs.MCI; Homo_633,female,AD.vs.control; Homo_633,female,AD.vs.MCI; Emory,totalSample,immune-Macrophage; Emory,totalSample,immune-NK; Emory,totalSample,immune-Neutrophil; Emory,totalSample,immune-CD4_T; Emory,totalSample,immune-CD8_T; Emory,totalSample,immune-Tr1; Emory,totalSample,immune-iTreg; Emory,totalSample,immune-Tfh; Emory,totalSample,immune-Effector_memory; Emory,totalSample,immune-InfiltrationScore; Homo_723,totalSample,immune-Th2; Homo_723,totalSample,immune-Tfh; Homo_723,totalSample,immune-Effector_memory; Homo_723,totalSample,immune-InfiltrationScore; Homo_723,totalSample,immune-iTreg; Homo_723,totalSample,immune-CD4_T; Homo_723,totalSample,immune-Bcell; Homo_723,totalSample,immune-Monocyte; Homo_723,totalSample,immune-Macrophage; Homo_723,totalSample,immune-Tr1; Homo_723,totalSample,immune-CD4_naive; Homo_714,totalSample,immune-Th2; Homo_714,totalSample,immune-Tfh; Homo_714,totalSample,immune-Effector_memory; Homo_714,totalSample,immune-InfiltrationScore; Homo_714,totalSample,immune-CD4_T; Homo_714,totalSample,immune-Tr1; Homo_714,totalSample,immune-Bcell; Homo_633,totalSample,immune-Th2; Homo_633,totalSample,immune-InfiltrationScore; Homo_633,totalSample,immune-Tfh; Homo_633,totalSample,immune-Effector_memory; Homo_633,totalSample,immune-CD4_T; Homo_633,totalSample,immune-Bcell; Homo_633,totalSample,immune-Monocyte; Homo_633,totalSample,immune-CD4_naive; Homo_633,totalSample,immune-Central_memory; MCSA,totalSample,immune-Monocyte; MCSA,totalSample,immune-Macrophage; MCSA,totalSample,immune-Neutrophil; MCSA,totalSample,immune-CD4_T; MCSA,totalSample,immune-CD4_naive; MCSA,totalSample,immune-Tr1; MCSA,totalSample,immune-nTreg; MCSA,totalSample,immune-iTreg; MCSA,totalSample,immune-Th17; MCSA,totalSample,immune-Tfh; MCSA,totalSample,immune-Effector_memory; MCSA,totalSample,immune-InfiltrationScore; ROSMAP,totalSample,immune-Cytotoxic; ROSMAP,totalSample,immune-Effector_memory; SRP223445,totalSample,immune-CD4_naive; SRP223445,totalSample,immune-Th17; SRP223445,totalSample,immune-[illegible]
PYCR1	Homo_723,female,AD.vs.control; Homo_723,female,AD.vs.MCI; Homo_633,female,AD.vs.control; Homo_633,female,AD.vs.MCI; ROSMAP,female,AD.vs.control; ROSMAP,female,MCI.vs.control; MCSA,totalSample,age; Emory,totalSample,immune-CD4_T; Emory,totalSample,immune-Tr1; Emory,totalSample,immune-iTreg; Emory,totalSample,immune-InfiltrationScore; Homo_723,totalSample,immune-nTreg; Homo_723,totalSample,immune-Central_memory; Homo_723,totalSample,immune-DC; Homo_723,totalSample,immune-Tfh; Homo_723,totalSample,immune-Neutrophil; Homo_723,totalSample,immune-CD4_naive; Homo_723,totalSample,immune-Monocyte; Homo_723,totalSample,immune-CD4_T; Homo_723,totalSample,immune-CD8_T; Homo_723,totalSample,immune-iTreg; Homo_714,totalSample,immune-nTreg; Homo_714,totalSample,immune-DC; Homo_714,totalSample,immune-Tfh; Homo_714,totalSample,immune-Central_memory; Homo_714,totalSample,immune-Neutrophil; Homo_714,totalSample,immune-CD4_naive; Homo_714,totalSample,immune-Monocyte; Homo_714,totalSample,immune-InfiltrationScore; Homo_714,totalSample,immune-iTreg; Homo_633,totalSample,immune-nTreg; Homo_633,totalSample,immune-DC; Homo_633,totalSample,immune-Central_memory; Homo_633,totalSample,immune-Monocyte; Homo_633,totalSample,immune-Tfh; Homo_633,totalSample,immune-CD4_naive; Homo_633,totalSample,immune-Effector_memory; Homo_633,totalSample,immune-InfiltrationScore; Homo_633,totalSample,immune-Neutrophil; Homo_633,totalSample,immune-Tr1; Homo_633,totalSample,immune-CD4_T; MCSA,totalSample,immune-DC; MCSA,totalSample,immune-Monocyte; MCSA,totalSample,immune-Neutrophil; MCSA,totalSample,immune-CD8_T; MCSA,totalSample,immune-NKT; MCSA,totalSample,immune-CD4_naive; MCSA,totalSample,immune-nTreg; MCSA,totalSample,immune-Th2; MCSA,totalSample,immune-Tfh; MCSA,totalSample,immune-CD8_naive; MCSA,totalSample,immune-Cytotoxic; MCSA,totalSample,immune-Central_memory; MCSA,totalSample,immune-Effector_memory; ROSMAP,female-[illegible]
CSPP1	Homo_723,female,AD.vs.control; Homo_723,male,AD.vs.control; Homo_723,female,AD.vs.MCI; Homo_723,male,AD.vs.MCI; Homo_714,female,AD.vs.control; Homo_714,male,AD.vs.control; Homo_714,female,AD.vs.MCI; Homo_714,male,AD.vs.MCI; Homo_633,female,AD.vs.control; Homo_633,female,AD.vs.MCI; MCSA,MCI,age; Emory,totalSample,immune-Macrophage; Emory,totalSample,immune-NK; Emory,totalSample,immune-Neutrophil; Emory,totalSample,immune-CD4_T; Emory,totalSample,immune-CD8_T; Emory,totalSample,immune-Tr1; Emory,totalSample,immune-iTreg; Emory,totalSample,immune-Tfh; Emory,totalSample,immune-Exhausted; Emory,totalSample,immune-Central_memory; Emory,totalSample,immune-Effector_memory; Emory,totalSample,immune-InfiltrationScore; Homo_723,totalSample,immune-CD4_naive; Homo_723,totalSample,immune-CD4_T; Homo_723,totalSample,immune-Th17; Homo_723,totalSample,immune-Tfh; Homo_723,totalSample,immune-Central_memory; Homo_723,totalSample,immune-Tr1; Homo_723,totalSample,immune-Exhausted; Homo_723,totalSample,immune-Cytotoxic; Homo_723,totalSample,immune-Th1; Homo_723,totalSample,immune-nTreg; Homo_723,totalSample,immune-InfiltrationScore; Homo_723,totalSample,immune-Th2; Homo_723,totalSample,immune-iTreg; Homo_723,totalSample,immune-Neutrophil; Homo_723,totalSample,immune-CD8_T; Homo_723,totalSample,immune-NKT; Homo_714,totalSample,immune-CD4_naive; Homo_714,totalSample,immune-CD4_T; Homo_714,totalSample,immune-Tfh; Homo_714,totalSample,immune-Central_memory; Homo_714,totalSample,immune-InfiltrationScore; Homo_714,totalSample,immune-Tr1; Homo_714,totalSample,immune-Th17; Homo_714,totalSample,immune-Th1; Homo_714,totalSample,immune-NKT; Homo_714,totalSample,immune-iTreg; Homo_714,totalSample,immune-Exhausted; Homo_714,totalSample,immune-Effector_memory; Homo_714,totalSample,immune-nTreg; Homo_633,totalSample,immune-Tfh; Homo_633,totalSample,immune-CD4_T; Homo_633,totalSample,immune-CD4_naive; Homo_633,totalSample,immune-Central_memory; Homo_633,totalSample,immune-Tr1; Homo_633,totalSample,immune-Effector_memory; Homo_633,totalSample,immune-Th2; Homo_633,totalSample,immune-InfiltrationScore; Homo_633,totalSample,immune-iTreg; Homo_633,totalSample,immune-NKT; Homo_633,totalSample,immune-nTreg; Homo_633,totalSample,immune-Bcell; Homo_633,totalSample,immune-Gamma_delta; Homo_633,totalSample,immune-Th17; Homo_633,totalSample,immune-Exhausted; Homo_633,totalSample,immune-DC; Homo_633,totalSample,immune-Th1; MCSA,totalSample,immune-DC; MCSA,totalSample,immune-Monocyte; MCSA,totalSample,immune-Macrophage; MCSA,totalSample,immune-CD4_T; MCSA,totalSample,immune-CD4_naive; MCSA,totalSample,immune-Tr1; MCSA,totalSample,immune-nTreg; MCSA,totalSample,immune-iTreg; MCSA,totalSample,immune-Th17; MCSA,totalSample,immune-Tfh; MCSA,totalSample,immune-CD8_naive; MCSA,totalSample,immune-Cytotoxic; MCSA,totalSample,immune-Exhausted; [illegible]
ARHGAP6	Homo_723,female,AD.vs.control; Homo_723,male,AD.vs.control; Homo_723,female,AD.vs.MCI; Homo_723,male,AD.vs.MCI; Homo_723,female,MCI.vs.control; Homo_723,male,MCI.vs.control; Homo_714,female,AD.vs.control; Homo_714,male,AD.vs.control; Homo_714,female,AD.vs.MCI; Homo_714,male,AD.vs.MCI; Homo_714,female,MCI.vs.control; Homo_714,male,MCI.vs.control; Homo_633,female,AD.vs.control; Homo_633,female,AD.vs.MCI; ROSMAP,female,AD.vs.control; ACOM,totalSample,age; Emory,totalSample,immune-CD8_T; Emory,totalSample,immune-Gamma_delta; Emory,totalSample,immune-Cytotoxic; Homo_723,totalSample,immune-DC; Homo_723,totalSample,immune-Cytotoxic; Homo_723,totalSample,immune-CD8_T; Homo_723,totalSample,immune-Th17; Homo_714,totalSample,immune-CD8_T; Homo_714,totalSample,immune-Exhausted; Homo_714,totalSample,immune-DC; Homo_714,totalSample,immune-NKT; Homo_633,totalSample,immune-NKT; Homo_633,totalSample,immune-Gamma_delta; MCSA,totalSample,immune-Tr1; MCSA,totalSample,immune-iTreg; MCSA,totalSample,immune-Cytotoxic; MCSA,totalSample,immune-Exhausted; SRP223445,totalSample,immune-CD4_naive; SRP223445,totalSample,immune-Exhausted
ZRANB3	Homo_723,female,AD.vs.control; Homo_723,male,AD.vs.control; Homo_723,female,AD.vs.MCI; Homo_723,male,AD.vs.MCI; Homo_723,female,MCI.vs.control; Homo_714,female,AD.vs.control; Homo_714,male,AD.vs.control; Homo_714,female,AD.vs.MCI; Homo_714,male,AD.vs.MCI; Homo_714,female,MCI.vs.control; Homo_633,female,AD.vs.control; Homo_633,female,AD.vs.MCI; ROSMAP,female,AD.vs.MCI; ACOM,totalSample,age; ROSMAP,MCI,last.visit.age; Emory,totalSample,immune-Macrophage; Emory,totalSample,immune-NK; Emory,totalSample,immune-Neutrophil; Emory,totalSample,immune-CD4_T; Emory,totalSample,immune-CD8_T; Emory,totalSample,immune-Tr1; Emory,totalSample,immune-iTreg; Emory,totalSample,immune-Tfh; Emory,totalSample,immune-CD8_naive; Emory,totalSample,immune-MAIT; Emory,totalSample,immune-Central_memory; Emory,totalSample,immune-InfiltrationScore; Homo_723,totalSample,immune-CD4_naive; Homo_723,totalSample,immune-CD4_T; Homo_723,totalSample,immune-Exhausted; Homo_723,totalSample,immune-Th17; Homo_723,totalSample,immune-Tfh; Homo_723,totalSample,immune-Tr1; Homo_723,totalSample,immune-Th2; Homo_723,totalSample,immune-Cytotoxic; Homo_723,totalSample,immune-Central_memory; Homo_723,totalSample,immune-Th1; Homo_723,totalSample,immune-nTreg; Homo_723,totalSample,immune-InfiltrationScore; Homo_723,totalSample,immune-iTreg; Homo_723,totalSample,immune-Neutrophil; Homo_723,totalSample,immune-Effector_memory; Homo_714,totalSample,immune-CD4_naive; Homo_714,totalSample,immune-CD4_T; Homo_714,totalSample,immune-Tfh; Homo_714,totalSample,immune-Central_memory; Homo_714,totalSample,immune-InfiltrationScore; Homo_714,totalSample,immune-Th17; Homo_714,totalSample,immune-Tr1; Homo_714,totalSample,immune-Th1; Homo_714,totalSample,immune-Effector_memory; Homo_714,totalSample,immune-Th2; Homo_714,totalSample,immune-NKT; Homo_714,totalSample,immune-Monocyte; Homo_714,totalSample,immune-iTreg; Homo_714,totalSample,immune-Exhausted; Homo_633,totalSample,immune-Tfh; Homo_633,totalSample,immune-CD4_T; Homo_633,totalSample,immune-Th2; Homo_633,totalSample,immune-CD4_naive; Homo_633,totalSample,immune-Effector_memory; Homo_633,totalSample,immune-Central_memory; Homo_633,totalSample,immune-Tr1; Homo_633,totalSample,immune-InfiltrationScore; Homo_633,totalSample,immune-iTreg; Homo_633,totalSample,immune-NKT; Homo_633,totalSample,immune-nTreg; Homo_633,totalSample,immune-Gamma_delta; Homo_633,totalSample,immune-Th17; Homo_633,totalSample,immune-Monocyte; Homo_633,totalSample,immune-Bcell; Homo_633,totalSample,immune-Exhausted; MCSA,totalSample,immune-DC; MCSA,totalSample,immune-Bcell; MCSA,totalSample,immune-Macrophage; MCSA,totalSample,immune-CD4_T; MCSA,totalSample,immune-CD4_naive; MCSA,totalSample,immune-Tr1; MCSA,totalSample,immune-nTreg; MCSA,totalSample,immune-iTreg; MCSA,totalSample,immune-Tfh; MCSA,totalSample,immune-CD8_naive; MCSA,totalSample,immune-Cytotoxic; MCSA,totalSample,immune-[illegible]
CA12	Homo_723,female,AD.vs.control; Homo_723,male,AD.vs.control; Homo_723,female,AD.vs.MCI; Homo_723,male,AD.vs.MCI; Homo_714,male,AD.vs.control; Homo_633,female,AD.vs.control; Homo_633,female,AD.vs.MCI; ROSMAP,male,AD.vs.control; ROSMAP,male,MCI.vs.control; ROSMAP,control,male.vs.female; ROSMAP,MCI,last.visit.age; Emory,totalSample,immune-Neutrophil; Emory,totalSample,immune-CD8_T; Emory,totalSample,immune-Gamma_delta; Emory,totalSample,immune-Th1; Emory,totalSample,immune-Tfh; Emory,totalSample,immune-Exhausted; Homo_714,totalSample,immune-Monocyte; Homo_633,totalSample,immune-Monocyte; Homo_633,totalSample,immune-Gamma_delta; Homo_633,totalSample,immune-Exhausted; MCSA,totalSample,immune-Neutrophil; MCSA,totalSample,immune-CD8_T; MCSA,totalSample,immune-Th17; MCSA,totalSample,immune-Cytotoxic; ROSMAP,totalSample,immune-InfiltrationScore
CFAP97D2	Homo_723,female,AD.vs.control; Homo_723,male,AD.vs.control; Homo_723,female,AD.vs.MCI; Homo_723,male,AD.vs.MCI; Homo_714,female,AD.vs.control; Homo_714,male,AD.vs.control; Homo_714,female,AD.vs.MCI; Homo_714,male,AD.vs.MCI; Homo_633,female,AD.vs.control; Homo_633,female,AD.vs.MCI; ROSMAP,female,AD.vs.control; ROSMAP,female,AD.vs.MCI; Emory,totalSample,immune-CD8_naive; Homo_723,totalSample,immune-Tfh; Homo_723,totalSample,immune-CD8_T; Homo_723,totalSample,immune-Macrophage; Homo_723,totalSample,immune-Th2; Homo_723,totalSample,immune-iTreg; Homo_723,totalSample,immune-Cytotoxic; Homo_723,totalSample,immune-Effector_memory; Homo_723,totalSample,immune-MAIT; Homo_714,totalSample,immune-Tfh; Homo_714,totalSample,immune-Th2; Homo_714,totalSample,immune-Macrophage; Homo_714,totalSample,immune-CD8_T; Homo_714,totalSample,immune-Effector_memory; Homo_633,totalSample,immune-InfiltrationScore; Homo_633,totalSample,immune-Tfh; MCSA,totalSample,immune-Tfh;
CLEC4D	Homo_723,female,AD.vs.control; Homo_723,male,AD.vs.control; Homo_723,female,AD.vs.MCI; Homo_723,male,AD.vs.MCI; Homo_723,female,MCI.vs.control; Homo_723,MCI,male.vs.female; Homo_714,female,AD.vs.control; Homo_714,male,AD.vs.control; Homo_714,female,AD.vs.MCI; Homo_714,male,AD.vs.MCI; Homo_714,female,MCI.vs.control; Homo_714,MCI,male.vs.female; Homo_633,female,AD.vs.control; Homo_633,female,AD.vs.MCI; Homo_633,female,MCI.vs.control; Homo_633,MCI,male.vs.female; ROSMAP,female,AD.vs.control; MCSA,totalSample,age; MCSA,control,age; MCSA,MCI,age; Emory,totalSample,immune-DC; Emory,totalSample,immune-Monocyte; Emory,totalSample,immune-Neutrophil; Emory,totalSample,immune-Gamma_delta; Emory,totalSample,immune-CD4_naive; Emory,totalSample,immune-nTreg; Emory,totalSample,immune-CD8_naive; Emory,totalSample,immune-Exhausted; Homo_723,totalSample,immune-Monocyte; Homo_723,totalSample,immune-DC; Homo_723,totalSample,immune-Neutrophil; Homo_714,totalSample,immune-Monocyte; Homo_714,totalSample,immune-DC; Homo_633,totalSample,immune-Monocyte; Homo_633,totalSample,immune-Exhausted; Homo_633,totalSample,immune-nTreg; Homo_633,totalSample,immune-iTreg; MCSA,totalSample,immune-DC; MCSA,totalSample,immune-Monocyte; MCSA,totalSample,immune-Neutrophil; MCSA,totalSample,immune-CD4_T; MCSA,totalSample,immune-CD8_T; MCSA,totalSample,immune-CD4_naive; MCSA,totalSample,immune-Tr1; MCSA,totalSample,immune-nTreg; MCSA,totalSample,immune-Th17; MCSA,totalSample,immune-Tfh; MCSA,totalSample,immune-Cytotoxic; MCSA,totalSample,immune-Central_memory; MCSA,totalSample,immune-Effector_memory;

VPS33A	Homo_723,female,AD.vs.control; Homo_723,male,AD.vs.control; Homo_723,female,AD.vs.MCI; Homo_723,male,AD.vs.MCI; Homo_714,female,AD.vs.control; Homo_714,male,AD.vs.control; Homo_714,female,AD.vs.MCI; Homo_714,male,AD.vs.MCI; Homo_633,female,AD.vs.control; Homo_633,female,AD.vs.MCI; Emory,MCI,age; MCSA,MCI,age; ROSMAP,MCI,last.visit.age; ROSMAP,MCI,sample.age; Emory,totalSample,immune-Macrophage; Emory,totalSample,immune-NK; Emory,totalSample,immune-Neutrophil; Emory,totalSample,immune-CD4_T; Emory,totalSample,immune-Tr1; Emory,totalSample,immune-iTreg; Emory,totalSample,immune-Effector_memory; Emory,totalSample,immune-InfiltrationScore; Homo_723,totalSample,immune-Th2; Homo_723,totalSample,immune-Tfh; Homo_723,totalSample,immune-iTreg; Homo_723,totalSample,immune-Effector_memory; Homo_723,totalSample,immune-InfiltrationScore; Homo_723,totalSample,immune-CD4_T; Homo_723,totalSample,immune-Tr1; Homo_723,totalSample,immune-CD4_naive; Homo_723,totalSample,immune-Bcell; Homo_723,totalSample,immune-DC; Homo_723,totalSample,immune-Monocyte; Homo_723,totalSample,immune-nTreg; Homo_723,totalSample,immune-Macrophage; Homo_723,totalSample,immune-Central_memory; Homo_723,totalSample,immune-Gamma_delta; Homo_714,totalSample,immune-Th2; Homo_714,totalSample,immune-Tfh; Homo_714,totalSample,immune-Effector_memory; Homo_714,totalSample,immune-CD4_T; Homo_714,totalSample,immune-Tr1; Homo_714,totalSample,immune-InfiltrationScore; Homo_714,totalSample,immune-Central_memory; Homo_714,totalSample,immune-CD4_naive; Homo_714,totalSample,immune-iTreg; Homo_714,totalSample,immune-nTreg; Homo_714,totalSample,immune-Bcell; Homo_633,totalSample,immune-Tfh; Homo_633,totalSample,immune-Th2; Homo_633,totalSample,immune-Effector_memory; Homo_633,totalSample,immune-InfiltrationScore; Homo_633,totalSample,immune-CD4_T; Homo_633,totalSample,immune-CD4_naive; Homo_633,totalSample,immune-Central_memory; Homo_633,totalSample,immune-Tr1; Homo_633,totalSample,immune-Bcell; Homo_633,totalSample,immune-Th17; Homo_633,totalSample,immune-nTreg; Homo_633,totalSample,immune-Exhausted; Homo_633,totalSample,immune-Monocyte; Homo_633,totalSample,immune-iTreg; Homo_633,totalSample,immune-Gamma_delta; MCSA,totalSample,immune-Macrophage; MCSA,totalSample,immune-CD4_T; MCSA,totalSample,immune-CD4_naive; MCSA,totalSample,immune-Tr1; MCSA,totalSample,immune-nTreg; MCSA,totalSample,immune-iTreg; MCSA,totalSample,immune-Th17; MCSA,totalSample,immune-Tfh; MCSA,totalSample,immune-CD8_naive; MCSA,totalSample,immune-Cytotoxic; MCSA,totalSample,immune-Exhausted; MCSA,totalSample,immune-MAIT; MCSA,totalSample,immune-Central_memory; MCSA,totalSample,immune-Effector_memory; ROSMAP,totalSample,immune-Th17; ROSMAP,totalSample,immune-Effector_memory; SRP223445,totalSample,immune-CD4_naive; SRP223445,totalSample,immune-Th17; SRP223445,totalSample,immune-Exhausted; [illegible]
PLCH1	Homo_723,female,AD.vs.control; Homo_723,male,AD.vs.control; Homo_723,female,AD.vs.MCI; Homo_723,male,AD.vs.MCI; Homo_723,female,MCI.vs.control; Homo_723,male,MCI.vs.control; Homo_714,female,AD.vs.control; Homo_714,male,AD.vs.control; Homo_714,female,AD.vs.MCI; Homo_714,male,AD.vs.MCI; Homo_714,female,MCI.vs.control; Homo_714,male,MCI.vs.control; Homo_633,female,AD.vs.control; Homo_633,female,AD.vs.MCI; ROSMAP,female,AD.vs.control; ROSMAP,MCI,sample.age; Homo_723,totalSample,immune-Th17; Homo_723,totalSample,immune-Exhausted; Homo_723,totalSample,immune-CD8_T; Homo_723,totalSample,immune-Cytotoxic; Homo_714,totalSample,immune-Exhausted; Homo_714,totalSample,immune-Th17; Homo_714,totalSample,immune-CD8_T; Homo_633,totalSample,immune-Gamma_delta; Homo_633,totalSample,immune-InfiltrationScore; Homo_633,totalSample,immune-Tfh; Homo_633,totalSample,immune-Th2; Homo_633,totalSample,immune-Th17; Homo_633,totalSample,immune-Exhausted; MCSA,totalSample,immune-CD4_T; MCSA,totalSample,immune-CD8_T; MCSA,totalSample,immune-Gamma_delta; MCSA,totalSample,immune-CD4_naive; MCSA,totalSample,immune-Tr1; MCSA,totalSample,immune-nTreg; MCSA,totalSample,immune-iTreg; MCSA,totalSample,immune-Th17; MCSA,totalSample,immune-Cytotoxic; MCSA,totalSample,immune-Exhausted; ROSMAP,totalSample,immune-[illegible]
PLXNA2	Homo_723,female,AD.vs.control; Homo_723,male,AD.vs.control; Homo_723,female,AD.vs.MCI; Homo_723,male,AD.vs.MCI; Homo_714,female,AD.vs.control; Homo_714,male,AD.vs.control; Homo_714,female,AD.vs.MCI; Homo_714,male,AD.vs.MCI; Homo_633,female,AD.vs.control; Homo_633,female,AD.vs.MCI; Homo_633,female,MCI.vs.control; ROSMAP,male,AD.vs.control; Emory,totalSample,age; Emory,MCI,age; MCSA,MCI,age; Emory,totalSample,immune-Neutrophil; Emory,totalSample,immune-CD8_T; Emory,totalSample,immune-Gamma_delta; Emory,totalSample,immune-Th1; Emory,totalSample,immune-Th17; Emory,totalSample,immune-Tfh; Emory,totalSample,immune-CD8_naive; Emory,totalSample,immune-Cytotoxic; Emory,totalSample,immune-Exhausted; Homo_723,totalSample,immune-DC; Homo_723,totalSample,immune-Monocyte; Homo_723,totalSample,immune-Th17; Homo_714,totalSample,immune-CD8_T; Homo_714,totalSample,immune-Exhausted; Homo_714,totalSample,immune-Th2; Homo_714,totalSample,immune-DC; Homo_714,totalSample,immune-MAIT; Homo_633,totalSample,immune-InfiltrationScore; Homo_633,totalSample,immune-Monocyte; Homo_633,totalSample,immune-Neutrophil; MCSA,totalSample,immune-DC; MCSA,totalSample,immune-Monocyte; MCSA,totalSample,immune-Macrophage; MCSA,totalSample,immune-Neutrophil; MCSA,totalSample,immune-Gamma_delta; MCSA,totalSample,immune-Th1; MCSA,totalSample,immune-Th2; MCSA,totalSample,immune-Th17; MCSA,totalSample,immune-Exhausted; SRP223445,totalSample,immune-InfiltrationScore
MOB3B	Homo_723,female,AD.vs.control; Homo_723,male,AD.vs.control; Homo_723,female,AD.vs.MCI; Homo_723,male,AD.vs.MCI; Homo_714,female,AD.vs.control; Homo_714,male,AD.vs.control; Homo_714,female,AD.vs.MCI; Homo_714,male,AD.vs.MCI; Homo_633,female,AD.vs.control; Homo_633,female,AD.vs.MCI; MCSA,totalSample,age; MCSA,control,age; Emory,totalSample,immune-B_cell; Emory,totalSample,immune-Gamma_delta; Emory,totalSample,immune-Tr1; Emory,totalSample,immune-iTreg; Emory,totalSample,immune-CD8_naive; Homo_723,totalSample,immune-Th17; Homo_723,totalSample,immune-Exhausted; Homo_723,totalSample,immune-CD4_naive; Homo_723,totalSample,immune-CD4_T; Homo_723,totalSample,immune-Cytotoxic; Homo_723,totalSample,immune-nTreg; Homo_723,totalSample,immune-Tfh; Homo_723,totalSample,immune-Tr1; Homo_723,totalSample,immune-Central_memory; Homo_723,totalSample,immune-Th1; Homo_723,totalSample,immune-Th2; Homo_723,totalSample,immune-InfiltrationScore; Homo_723,totalSample,immune-CD8_T; Homo_723,totalSample,immune-Neutrophil; Homo_723,totalSample,immune-iTreg; Homo_723,totalSample,immune-Gamma_delta; Homo_714,totalSample,immune-Th17; Homo_714,totalSample,immune-Exhausted; Homo_714,totalSample,immune-CD4_naive; Homo_714,totalSample,immune-CD4_T; Homo_714,totalSample,immune-Tfh; Homo_714,totalSample,immune-InfiltrationScore; Homo_714,totalSample,immune-nTreg; Homo_714,totalSample,immune-Gamma_delta; Homo_714,totalSample,immune-Th1; Homo_714,totalSample,immune-Central_memory; Homo_714,totalSample,immune-Cytotoxic; Homo_714,totalSample,immune-NKT; Homo_714,totalSample,immune-Tr1; Homo_633,totalSample,immune-Tfh; Homo_633,totalSample,immune-CD4_T; Homo_633,totalSample,immune-Th2; Homo_633,totalSample,immune-Tr1; Homo_633,totalSample,immune-CD4_naive; Homo_633,totalSample,immune-Gamma_delta; Homo_633,totalSample,immune-Central_memory; Homo_633,totalSample,immune-Effector_memory; Homo_633,totalSample,immune-Exhausted; Homo_633,totalSample,immune-Th17; Homo_633,totalSample,immune-nTreg; Homo_633,totalSample,immune-iTreg; Homo_633,totalSample,immune-InfiltrationScore; Homo_633,totalSample,immune-NKT; MCSA,totalSample,immune-Bcell; MCSA,totalSample,immune-Macrophage; MCSA,totalSample,immune-CD4_T; MCSA,totalSample,immune-CD8_T; MCSA,totalSample,immune-Gamma_delta; MCSA,totalSample,immune-CD4_naive; MCSA,totalSample,immune-Tr1; MCSA,totalSample,immune-nTreg; MCSA,totalSample,immune-iTreg; MCSA,totalSample,immune-Th17; MCSA,totalSample,immune-Tfh; MCSA,totalSample,immune-CD8_naive; MCSA,totalSample,immune-Cytotoxic; [illegible]
EPB41L5	Homo_723,female,AD.vs.control; Homo_723,male,AD.vs.control; Homo_723,female,AD.vs.MCI; Homo_723,male,AD.vs.MCI; Homo_723,female,MCI.vs.control; Homo_723,male,MCI.vs.control; Homo_714,female,AD.vs.control; Homo_714,male,AD.vs.control; Homo_714,female,AD.vs.MCI; Homo_714,male,AD.vs.MCI; Homo_633,female,AD.vs.control; Homo_633,female,AD.vs.MCI; Emory,totalSample,immune-Macrophage; Emory,totalSample,immune-CD4_T; Emory,totalSample,immune-Gamma_delta; Emory,totalSample,immune-Tr1; Emory,totalSample,immune-iTreg; Emory,totalSample,immune-Central_memory; Emory,totalSample,immune-InfiltrationScore; Homo_723,totalSample,immune-CD4_naive; Homo_723,totalSample,immune-Exhausted; Homo_723,totalSample,immune-Th17; Homo_723,totalSample,immune-CD4_T; Homo_723,totalSample,immune-Tfh; Homo_723,totalSample,immune-Cytotoxic; Homo_723,totalSample,immune-Tr1; Homo_723,totalSample,immune-Th2; Homo_723,totalSample,immune-Central_memory; Homo_723,totalSample,immune-nTreg; Homo_723,totalSample,immune-iTreg; Homo_723,totalSample,immune-Th1; Homo_723,totalSample,immune-InfiltrationScore; Homo_723,totalSample,immune-CD8_T; Homo_723,totalSample,immune-Effector_memory; Homo_714,totalSample,immune-CD4_naive; Homo_714,totalSample,immune-CD4_T; Homo_714,totalSample,immune-Tfh; Homo_714,totalSample,immune-Central_memory; Homo_714,totalSample,immune-Th17; Homo_714,totalSample,immune-Exhausted; Homo_714,totalSample,immune-Tr1; Homo_714,totalSample,immune-InfiltrationScore; Homo_714,totalSample,immune-Th2; Homo_714,totalSample,immune-iTreg; Homo_714,totalSample,immune-Effector_memory; Homo_714,totalSample,immune-Gamma_delta; Homo_714,totalSample,immune-nTreg; Homo_714,totalSample,immune-NKT; Homo_714,totalSample,immune-Monocyte; Homo_714,totalSample,immune-Th1; Homo_633,totalSample,immune-Tfh; Homo_633,totalSample,immune-CD4_T; Homo_633,totalSample,immune-Th2; Homo_633,totalSample,immune-CD4_naive; Homo_633,totalSample,immune-Central_memory; Homo_633,totalSample,immune-Effector_memory; Homo_633,totalSample,immune-Tr1; Homo_633,totalSample,immune-Gamma_delta; Homo_633,totalSample,immune-iTreg; Homo_633,totalSample,immune-Th17; Homo_633,totalSample,immune-InfiltrationScore; Homo_633,totalSample,immune-NKT; Homo_633,totalSample,immune-nTreg; Homo_633,totalSample,immune-Exhausted; Homo_633,totalSample,immune-DC; Homo_633,totalSample,immune-Monocyte; MCSA,totalSample,immune-DC; MCSA,totalSample,immune-Bcell; MCSA,totalSample,immune-Macrophage; MCSA,totalSample,immune-CD4_T; MCSA,totalSample,immune-CD8_T; MCSA,totalSample,immune-Gamma_delta; MCSA,totalSample,immune-CD4_naive; MCSA,totalSample,immune-Tr1; MCSA,totalSample,immune-nTreg; MCSA,totalSample,immune-iTreg; MCSA,totalSample,immune-Th17; MCSA,totalSample,immune-Tfh; MCSA,totalSample,immune-CD8_naive; MCSA,totalSample,immune-Cytotoxic; MCSA,totalSample,immune-[illegible]
SPSB1	Homo_723,female,AD.vs.control; Homo_723,male,AD.vs.control; Homo_723,female,AD.vs.MCI; Homo_723,male,AD.vs.MCI; Homo_714,female,AD.vs.control; Homo_714,male,AD.vs.control; Homo_714,female,AD.vs.MCI; Homo_714,male,AD.vs.MCI; Homo_633,female,AD.vs.control; Homo_633,female,AD.vs.MCI; Homo_633,female,MCI.vs.control; Homo_633,male,MCI.vs.control; ROSMAP,male,AD.vs.control; ROSMAP,MCI,male.vs.female; ACOM,totalSample,age; MCSA,totalSample,age; MCSA,control,age; Emory,totalSample,immune-Macrophage; Emory,totalSample,immune-NK; Emory,totalSample,immune-Neutrophil; Emory,totalSample,immune-CD4_T; Emory,totalSample,immune-Tr1; Emory,totalSample,immune-iTreg; Emory,totalSample,immune-Tfh; Emory,totalSample,immune-InfiltrationScore; Homo_723,totalSample,immune-DC; Homo_723,totalSample,immune-Monocyte; Homo_723,totalSample,immune-Neutrophil; Homo_723,totalSample,immune-Central_memory; Homo_723,totalSample,immune-nTreg; Homo_723,totalSample,immune-CD4_naive; Homo_723,totalSample,immune-InfiltrationScore; Homo_723,totalSample,immune-Th1; Homo_714,totalSample,immune-DC; Homo_714,totalSample,immune-Monocyte; Homo_714,totalSample,immune-InfiltrationScore; Homo_714,totalSample,immune-Th2; Homo_714,totalSample,immune-NKT; Homo_714,totalSample,immune-Th1; Homo_633,totalSample,immune-Monocyte; Homo_633,totalSample,immune-InfiltrationScore; Homo_633,totalSample,immune-nTreg; Homo_633,totalSample,immune-DC; Homo_633,totalSample,immune-Th2; Homo_633,totalSample,immune-iTreg; MCSA,totalSample,immune-DC; MCSA,totalSample,immune-Monocyte; MCSA,totalSample,immune-Neutrophil; MCSA,totalSample,immune-CD4_T; MCSA,totalSample,immune-CD8_T; MCSA,totalSample,immune-NKT; MCSA,totalSample,immune-CD4_naive; MCSA,totalSample,immune-Tr1; MCSA,totalSample,immune-nTreg; MCSA,totalSample,immune-Th2; MCSA,totalSample,immune-Tfh; MCSA,totalSample,immune-Cytotoxic; MCSA,totalSample,immune-Central_memory; MCSA,totalSample,immune-[illegible]
NFILZ	Homo_723,female,AD.vs.control; Homo_723,male,AD.vs.control; Homo_723,female,AD.vs.MCI; Homo_723,male,AD.vs.MCI; Homo_723,female,MCI.vs.control; Homo_723,male,MCI.vs.control; Homo_714,female,AD.vs.control; Homo_714,male,AD.vs.control; Homo_714,female,AD.vs.MCI; Homo_714,male,AD.vs.MCI; Homo_714,female,MCI.vs.control; Homo_714,male,MCI.vs.control; Homo_633,female,AD.vs.control; Homo_633,female,AD.vs.MCI; Homo_633,female,MCI.vs.control; Homo_633,male,MCI.vs.control; ROSMAP,male,AD.vs.control; ROSMAP,male,MCI.vs.control; MCSA,totalSample,age; MCSA,control,age; Emory,totalSample,immune-Tr1; Emory,totalSample,immune-iTreg; Emory,totalSample,immune-InfiltrationScore; Homo_723,totalSample,immune-Exhausted; Homo_723,totalSample,immune-CD8_T; Homo_723,totalSample,immune-Cytotoxic; Homo_723,totalSample,immune-InfiltrationScore; Homo_723,totalSample,immune-MAIT; Homo_723,totalSample,immune-Monocyte; Homo_723,totalSample,immune-iTreg; Homo_723,totalSample,immune-Macrophage; Homo_723,totalSample,immune-Tfh; Homo_723,totalSample,immune-Th17; Homo_723,totalSample,immune-Bcell; Homo_714,totalSample,immune-InfiltrationScore; Homo_714,totalSample,immune-Macrophage; Homo_714,totalSample,immune-Monocyte; Homo_714,totalSample,immune-Exhausted; Homo_714,totalSample,immune-Tfh; Homo_714,totalSample,immune-CD8_T; Homo_714,totalSample,immune-MAIT; Homo_714,totalSample,immune-Effector_memory; Homo_714,totalSample,immune-Cytotoxic; Homo_633,totalSample,immune-InfiltrationScore; Homo_633,totalSample,immune-Monocyte; Homo_633,totalSample,immune-Macrophage; Homo_633,totalSample,immune-Bcell; MCSA,totalSample,immune-Monocyte; MCSA,totalSample,immune-Macrophage; MCSA,totalSample,immune-CD4_T; MCSA,totalSample,immune-CD8_T; MCSA,totalSample,immune-CD4_naive; MCSA,totalSample,immune-Tr1; MCSA,totalSample,immune-nTreg; MCSA,totalSample,immune-Th17; MCSA,totalSample,immune-Tfh; MCSA,totalSample,immune-Cytotoxic; MCSA,totalSample,immune-MAIT;
TLR8	Homo_723,female,AD.vs.control; Homo_723,male,AD.vs.control; Homo_723,female,AD.vs.MCI; Homo_723,male,AD.vs.MCI; Homo_723,female,MCI.vs.control; SRP223445,female,AD.vs.control; MCSA,MCI,age; Emory,totalSample,immune-DC; Emory,totalSample,immune-Monocyte; Emory,totalSample,immune-NK; Emory,totalSample,immune-Neutrophil; Emory,totalSample,immune-CD8_T; Emory,totalSample,immune-Gamma_delta; Emory,totalSample,immune-nTreg; Emory,totalSample,immune-Th1; Emory,totalSample,immune-Th17; Emory,totalSample,immune-Tfh; Emory,totalSample,immune-CD8_naive; Emory,totalSample,immune-Cytotoxic; Emory,totalSample,immune-Exhausted; Homo_723,totalSample,immune-DC; Homo_723,totalSample,immune-Monocyte; Homo_723,totalSample,immune-Neutrophil; Homo_723,totalSample,immune-Th2; Homo_723,totalSample,immune-Gamma_delta; Homo_723,totalSample,immune-Exhausted; Homo_723,totalSample,immune-iTreg; Homo_723,totalSample,immune-Tfh; Homo_723,totalSample,immune-Effector_memory; Homo_723,totalSample,immune-Bcell; Homo_714,totalSample,immune-Neutrophil; Homo_714,totalSample,immune-Exhausted; Homo_714,totalSample,immune-CD8_T; Homo_714,totalSample,immune-Gamma_delta; Homo_714,totalSample,immune-Cytotoxic; Homo_714,totalSample,immune-DC; Homo_714,totalSample,immune-Monocyte; Homo_714,totalSample,immune-NKT; Homo_714,totalSample,immune-Macrophage; Homo_714,totalSample,immune-Th17; Homo_633,totalSample,immune-CD8_T; Homo_633,totalSample,immune-Exhausted; Homo_633,totalSample,immune-Gamma_delta; Homo_633,totalSample,immune-Neutrophil; Homo_633,totalSample,immune-Cytotoxic; Homo_633,totalSample,immune-NKT; Homo_633,totalSample,immune-DC; Homo_633,totalSample,immune-Th17; Homo_633,totalSample,immune-Th2; Homo_633,totalSample,immune-Macrophage; Homo_633,totalSample,immune-Monocyte; MCSA,totalSample,immune-DC; MCSA,totalSample,immune-Monocyte; MCSA,totalSample,immune-Neutrophil; MCSA,totalSample,immune-CD4_T; MCSA,totalSample,immune-CD8_T; MCSA,totalSample,immune-Gamma_delta; MCSA,totalSample,immune-Tr1; MCSA,totalSample,immune-nTreg; MCSA,totalSample,immune-iTreg; MCSA,totalSample,immune-Th1; MCSA,totalSample,immune-Th17; MCSA,totalSample,immune-Cytotoxic; MCSA,totalSample,immune-Exhausted; MCSA,totalSample,immune-Effector_memory; ROSMAP,totalSample,immune-Neutrophil; ROSMAP,totalSample,immune-CD8_naive; ROSMAP,totalSample,immune-Effector_memory; SRP223445,totalSample,immune-Monocyte; SRP223445,totalSample,immune-Neutrophil; SRP223445,totalSample,immune-Th17
SYTL4	Homo_723,female,AD.vs.control; Homo_723,male,AD.vs.control; Homo_723,female,AD.vs.MCI; Homo_723,male,AD.vs.MCI; Homo_723,female,MCI.vs.control; Homo_723,male,MCI.vs.control; Homo_714,female,AD.vs.control; Homo_714,male,AD.vs.control; Homo_714,female,AD.vs.MCI; Homo_714,male,AD.vs.MCI; Homo_714,female,MCI.vs.control; Homo_714,male,MCI.vs.control; Homo_633,female,AD.vs.control; Homo_633,female,AD.vs.MCI; Homo_723,totalSample,immune-Exhausted; Homo_723,totalSample,immune-Th17; Homo_723,totalSample,immune-CD8_T; Homo_723,totalSample,immune-Cytotoxic; Homo_723,totalSample,immune-DC; Homo_723,totalSample,immune-MAIT; Homo_714,totalSample,immune-Th17; Homo_714,totalSample,immune-Exhausted; Homo_714,totalSample,immune-CD8_T; Homo_633,totalSample,immune-Th2; Homo_633,totalSample,immune-Gamma_delta; Homo_633,totalSample,immune-Th17; Homo_633,totalSample,immune-Tfh; Homo_633,totalSample,immune-Exhausted; Homo_633,totalSample,immune-InfiltrationScore; Homo_633,totalSample,immune-Monocyte; MCSA,totalSample,immune-Tr1; MCSA,totalSample,immune-iTreg; MCSA,totalSample,immune-Th17; MCSA,totalSample,immune-Exhausted; ROSMAP,totalSample,immune-InfiltrationScore; SRP223445,totalSample,immune-CD4_naive; SRP223445,totalSample,immune-Exhausted
PRDM11	Homo_723,female,AD.vs.control; Homo_723,male,AD.vs.control; Homo_723,female,AD.vs.MCI; Homo_723,male,AD.vs.MCI; Homo_714,female,AD.vs.control; Homo_714,male,AD.vs.control; Homo_714,female,AD.vs.MCI; Homo_714,male,AD.vs.MCI; Homo_633,female,AD.vs.control; Homo_633,female,AD.vs.MCI; ROSMAP,male,AD.vs.control; MCSA,totalSample,age; MCSA,control,age; MCSA,MCI,age; ROSMAP,MCI,last.visit.age; Emory,totalSample,immune-Monocyte; Emory,totalSample,immune-Macrophage; Emory,totalSample,immune-NK; Emory,totalSample,immune-Neutrophil; Emory,totalSample,immune-CD4_T; Emory,totalSample,immune-Tr1; Emory,totalSample,immune-iTreg; Emory,totalSample,immune-Exhausted; Emory,totalSample,immune-Effector_memory; Emory,totalSample,immune-InfiltrationScore; Homo_723,totalSample,immune-DC; Homo_723,totalSample,immune-Monocyte; Homo_723,totalSample,immune-Neutrophil; Homo_723,totalSample,immune-nTreg; Homo_723,totalSample,immune-Central_memory; Homo_723,totalSample,immune-InfiltrationScore; Homo_723,totalSample,immune-CD4_naive; Homo_714,totalSample,immune-Monocyte; Homo_714,totalSample,immune-InfiltrationScore; Homo_714,totalSample,immune-DC; Homo_714,totalSample,immune-Neutrophil; Homo_714,totalSample,immune-nTreg; Homo_714,totalSample,immune-CD4_naive; Homo_633,totalSample,immune-Monocyte; Homo_633,totalSample,immune-InfiltrationScore; Homo_633,totalSample,immune-nTreg; Homo_633,totalSample,immune-DC; Homo_633,totalSample,immune-Effector_memory; Homo_633,totalSample,immune-Neutrophil; Homo_633,totalSample,immune-CD4_naive; Homo_633,totalSample,immune-Macrophage; Homo_633,totalSample,immune-iTreg; Homo_633,totalSample,immune-Tfh; MCSA,totalSample,immune-DC; MCSA,totalSample,immune-Monocyte; MCSA,totalSample,immune-Neutrophil; MCSA,totalSample,immune-CD4_T; MCSA,totalSample,immune-CD8_T; MCSA,totalSample,immune-NKT; MCSA,totalSample,immune-CD4_naive; MCSA,totalSample,immune-Tr1; MCSA,totalSample,immune-nTreg; MCSA,totalSample,immune-Th2; MCSA,totalSample,immune-Th17; MCSA,totalSample,immune-Tfh; MCSA,totalSample,immune-Cytotoxic; MCSA,totalSample,immune-Central_memory; MCSA,totalSample,immune-Effector_memory; MCSA,totalSample,immune-InfiltrationScore; SRP223445,totalSample,immune-Neutrophil; [illegible]
GPM6A	Homo_723,female,AD.vs.control; Homo_723,female,AD.vs.MCI; Homo_723,female,MCI.vs.control; Homo_723,male,MCI.vs.control; Homo_714,male,MCI.vs.control; Homo_633,female,AD.vs.control; Homo_633,female,AD.vs.MCI; Homo_633,male,MCI.vs.control; ROSMAP,control,male.vs.female; ACOM,totalSample,age; Emory,MCI,age; ROSMAP,MCI,last.visit.age; ROSMAP,MCI,sample.age; Homo_723,totalSample,immune-Th2; Homo_723,totalSample,immune-CD4_T; Homo_723,totalSample,immune-iTreg; Homo_723,totalSample,immune-Tfh; Homo_723,totalSample,immune-Tr1; Homo_723,totalSample,immune-nTreg; Homo_723,totalSample,immune-CD4_naive; Homo_723,totalSample,immune-Central_memory; Homo_714,totalSample,immune-Tfh; Homo_714,totalSample,immune-CD4_T; Homo_714,totalSample,immune-Th2; Homo_714,totalSample,immune-Tr1; Homo_714,totalSample,immune-iTreg; Homo_714,totalSample,immune-CD4_naive; Homo_714,totalSample,immune-Effector_memory; Homo_633,totalSample,immune-Tfh; Homo_633,totalSample,immune-CD4_T; Homo_633,totalSample,immune-Central_memory; Homo_633,totalSample,immune-CD4_naive; Homo_633,totalSample,immune-Tr1; Homo_633,totalSample,immune-Effector_memory; Homo_633,totalSample,immune-nTreg; Homo_633,totalSample,immune-Th2; Homo_633,totalSample,immune-iTreg; Homo_633,totalSample,immune-NKT; MCSA,totalSample,immune-DC; MCSA,totalSample,immune-Bcell; MCSA,totalSample,immune-Macrophage; MCSA,totalSample,immune-CD4_T; MCSA,totalSample,immune-CD4_naive; MCSA,totalSample,immune-Tr1; MCSA,totalSample,immune-nTreg; MCSA,totalSample,immune-iTreg; MCSA,totalSample,immune-Tfh; MCSA,totalSample,immune-CD8_naive; MCSA,totalSample,immune-MAIT; MCSA,totalSample,immune-Central_memory; ROSMAP,totalSample,immune-InfiltrationScore; SRP223445,totalSample,immune-NK; SRP223445,totalSample,immune-InfiltrationScore
PROSER2	Homo_723,female,AD.vs.control; Homo_723,male,AD.vs.control; Homo_723,female,AD.vs.MCI; Homo_723,male,AD.vs.MCI; Homo_714,female,AD.vs.control; Homo_714,male,AD.vs.control; Homo_714,female,AD.vs.MCI; Homo_714,male,AD.vs.MCI; Homo_633,female,AD.vs.control; Homo_633,female,AD.vs.MCI; Homo_633,female,MCI.vs.control; Homo_633,male,MCI.vs.control; ROSMAP,female,MCI.vs.control; ROSMAP,AD,male.vs.female; ROSMAP,control,male.vs.female; MCSA,MCI,age; ROSMAP,MCI,MMSE; Emory,totalSample,immune-Th1; Emory,totalSample,immune-Th17; Emory,totalSample,immune-Cytotoxic; Homo_723,totalSample,immune-DC; Homo_723,totalSample,immune-Monocyte; Homo_723,totalSample,immune-Th17; Homo_714,totalSample,immune-Th2; Homo_714,totalSample,immune-DC; Homo_714,totalSample,immune-CD8_T; MCSA,totalSample,immune-Th17; SRP223445,totalSample,immune-CD4_naive; SRP223445,totalSample,immune-Exhausted
SLC25A38	Homo_723,female,AD.vs.control; Homo_723,male,AD.vs.control; Homo_723,female,AD.vs.MCI; Homo_723,male,AD.vs.MCI; Homo_714,female,AD.vs.control; Homo_714,male,AD.vs.control; Homo_714,female,AD.vs.MCI; Homo_714,male,AD.vs.MCI; Homo_633,female,AD.vs.control; Homo_633,female,AD.vs.MCI; ROSMAP,female,AD.vs.control; ROSMAP,female,MCI.vs.control; Emory,totalSample,age; Emory,MCI,age; Emory,totalSample,immune-Macrophage; Emory,totalSample,immune-NK; Emory,totalSample,immune-Neutrophil; Emory,totalSample,immune-CD4_T; Emory,totalSample,immune-CD8_T; Emory,totalSample,immune-Gamma_delta; Emory,totalSample,immune-Tr1; Emory,totalSample,immune-iTreg; Emory,totalSample,immune-Tfh; Emory,totalSample,immune-CD8_naive; Emory,totalSample,immune-Cytotoxic; Emory,totalSample,immune-Exhausted; Emory,totalSample,immune-MAIT; Emory,totalSample,immune-Effector_memory; Emory,totalSample,immune-InfiltrationScore; Homo_723,totalSample,immune-Tfh; Homo_723,totalSample,immune-iTreg; Homo_723,totalSample,immune-CD8_T; Homo_723,totalSample,immune-Macrophage; Homo_723,totalSample,immune-InfiltrationScore; Homo_723,totalSample,immune-Exhausted; Homo_723,totalSample,immune-CD4_T; Homo_723,totalSample,immune-Th2; Homo_723,totalSample,immune-Central_memory; Homo_723,totalSample,immune-CD4_naive; Homo_723,totalSample,immune-nTreg; Homo_723,totalSample,immune-MAIT; Homo_723,totalSample,immune-Tr1; Homo_723,totalSample,immune-Effector_memory; Homo_723,totalSample,immune-Cytotoxic; Homo_714,totalSample,immune-Tfh; Homo_714,totalSample,immune-Macrophage; Homo_714,totalSample,immune-Th2; Homo_714,totalSample,immune-CD4_T; Homo_714,totalSample,immune-Effector_memory; Homo_714,totalSample,immune-InfiltrationScore; Homo_714,totalSample,immune-nTreg; Homo_714,totalSample,immune-CD8_T; Homo_714,totalSample,immune-Central_memory; Homo_714,totalSample,immune-iTreg; Homo_714,totalSample,immune-Tr1; Homo_714,totalSample,immune-Neutrophil; Homo_714,totalSample,immune-MAIT; Homo_714,totalSample,immune-CD4_naive; Homo_714,totalSample,immune-Exhausted; Homo_633,totalSample,immune-Tfh; Homo_633,totalSample,immune-InfiltrationScore; Homo_633,totalSample,immune-Neutrophil; Homo_633,totalSample,immune-nTreg; Homo_633,totalSample,immune-CD4_T; Homo_633,totalSample,immune-Central_memory; Homo_633,totalSample,immune-Macrophage; Homo_633,totalSample,immune-CD4_naive; Homo_633,totalSample,immune-Tr1; Homo_633,totalSample,immune-iTreg; Homo_633,totalSample,immune-Effector_memory; MCSA,totalSample,immune-Monocyte; MCSA,totalSample,immune-Neutrophil; MCSA,totalSample,immune-CD4_T; MCSA,totalSample,immune-CD8_T; MCSA,totalSample,immune-CD4_naive; MCSA,totalSample,immune-Tr1; MCSA,totalSample,immune-nTreg; MCSA,totalSample,immune-iTreg; MCSA,totalSample,immune-Th17; MCSA,totalSample,immune-Tfh; MCSA,totalSample,immune-CD8_naive; MCSA,totalSample,immune-Central_memory; MCSA,totalSample,immune-Effector_memory; MCSA,totalSample,immune-InfiltrationScore; ROSMAP,totalSample,immune-Th17; ROSMAP,totalSample,immune-Effector_memory; ROSMAP,totalSample,immune-InfiltrationScore; SRP223445,totalSample,immune-NK; SRP223445,totalSample,immune-Neutrophil; SRP223445,totalSample,immune-CD8_T; [illegible]

CSNK1G3	Homo_723,female,AD.vs.control; Homo_723,male,AD.vs.control; Homo_723,female,AD.vs.MCI; Homo_723,male,AD.vs.MCI; Homo_723,female,MCI.vs.control; Homo_723,male,MCI.vs.control; Homo_714,female,AD.vs.control; Homo_714,male,AD.vs.control; Homo_714,female,AD.vs.MCI; Homo_714,male,AD.vs.MCI; Homo_714,female,MCI.vs.control; Homo_714,male,MCI.vs.control; Homo_633,female,AD.vs.control; Homo_633,female,AD.vs.MCI; ROSMAP,female,AD.vs.control; MCSA,MCI,age; ROSMAP,MCI,sample.age; Emory,totalSample,immune-DC; Emory,totalSample,immune-Monocyte; Emory,totalSample,immune-Macrophage; Emory,totalSample,immune-CD4_T; Emory,totalSample,immune-Gamma_delta; Emory,totalSample,immune-Tr1; Emory,totalSample,immune-iTreg; Emory,totalSample,immune-Central_memory; Emory,totalSample,immune-Effector_memory; Emory,totalSample,immune-InfiltrationScore; Homo_723,totalSample,immune-Exhausted; Homo_723,totalSample,immune-Cytotoxic; Homo_723,totalSample,immune-CD4_naive; Homo_723,totalSample,immune-Th17; Homo_723,totalSample,immune-CD4_T; Homo_723,totalSample,immune-CD8_T; Homo_723,totalSample,immune-Central_memory; Homo_723,totalSample,immune-Tr1; Homo_723,totalSample,immune-Th1; Homo_723,totalSample,immune-Tfh; Homo_723,totalSample,immune-NKT; Homo_723,totalSample,immune-InfiltrationScore; Homo_714,totalSample,immune-CD4_naive; Homo_714,totalSample,immune-NKT; Homo_714,totalSample,immune-Monocyte; Homo_714,totalSample,immune-Exhausted; Homo_714,totalSample,immune-Central_memory; Homo_714,totalSample,immune-CD4_T; Homo_714,totalSample,immune-Tfh; Homo_714,totalSample,immune-Th17; Homo_714,totalSample,immune-Th1; Homo_714,totalSample,immune-InfiltrationScore; Homo_714,totalSample,immune-Tr1; Homo_633,totalSample,immune-Tfh; Homo_633,totalSample,immune-CD4_T; Homo_633,totalSample,immune-CD4_naive; Homo_633,totalSample,immune-NKT; Homo_633,totalSample,immune-Central_memory; Homo_633,totalSample,immune-Effector_memory; Homo_633,totalSample,immune-Monocyte; Homo_633,totalSample,immune-Th2; Homo_633,totalSample,immune-Tr1; Homo_633,totalSample,immune-DC; Homo_633,totalSample,immune-InfiltrationScore; Homo_633,totalSample,immune-Gamma_delta; Homo_633,totalSample,immune-iTreg; Homo_633,totalSample,immune-Exhausted; MCSA,totalSample,immune-DC; MCSA,totalSample,immune-Bcell; MCSA,totalSample,immune-Monocyte; MCSA,totalSample,immune-Macrophage; MCSA,totalSample,immune-CD4_T; MCSA,totalSample,immune-CD8_T; MCSA,totalSample,immune-CD4_naive; MCSA,totalSample,immune-Tr1; MCSA,totalSample,immune-nTreg; MCSA,totalSample,immune-iTreg; MCSA,totalSample,immune-Th2; [illegible]
DUSP11	Homo_723,female,AD.vs.control; Homo_723,male,AD.vs.control; Homo_723,female,AD.vs.MCI; Homo_723,male,AD.vs.MCI; Homo_714,female,AD.vs.control; Homo_714,male,AD.vs.control; Homo_714,female,AD.vs.MCI; Homo_714,male,AD.vs.MCI; Homo_633,female,AD.vs.control; Homo_633,female,AD.vs.MCI; ROSMAP,female,AD.vs.control; MCSA,MCI,age; ROSMAP,MCI,last.visit.age; ROSMAP,MCI,sample.age; Emory,totalSample,immune-DC; Emory,totalSample,immune-Monocyte; Emory,totalSample,immune-Macrophage; Emory,totalSample,immune-CD4_T; Emory,totalSample,immune-CD8_T; Emory,totalSample,immune-Tr1; Emory,totalSample,immune-iTreg; Emory,totalSample,immune-Effector_memory; Emory,totalSample,immune-InfiltrationScore; Homo_723,totalSample,immune-Tfh; Homo_723,totalSample,immune-iTreg; Homo_723,totalSample,immune-Th2; Homo_723,totalSample,immune-InfiltrationScore; Homo_723,totalSample,immune-Tr1; Homo_723,totalSample,immune-CD4_T; Homo_723,totalSample,immune-DC; Homo_723,totalSample,immune-Effector_memory; Homo_723,totalSample,immune-CD4_naive; Homo_723,totalSample,immune-Gamma_delta; Homo_723,totalSample,immune-Central_memory; Homo_723,totalSample,immune-Monocyte; Homo_723,totalSample,immune-MAIT; Homo_723,totalSample,immune-Bcell; Homo_723,totalSample,immune-Macrophage; Homo_714,totalSample,immune-Tfh; Homo_714,totalSample,immune-Th2; Homo_714,totalSample,immune-Tr1; Homo_714,totalSample,immune-CD4_T; Homo_714,totalSample,immune-Effector_memory; Homo_714,totalSample,immune-Central_memory; Homo_714,totalSample,immune-InfiltrationScore; Homo_714,totalSample,immune-iTreg; Homo_714,totalSample,immune-CD4_naive; Homo_714,totalSample,immune-DC; Homo_714,totalSample,immune-Monocyte; Homo_714,totalSample,immune-Gamma_delta; Homo_714,totalSample,immune-Bcell; Homo_633,totalSample,immune-Tfh; Homo_633,totalSample,immune-CD4_T; Homo_633,totalSample,immune-InfiltrationScore; Homo_633,totalSample,immune-CD4_naive; Homo_633,totalSample,immune-Central_memory; Homo_633,totalSample,immune-Th2; Homo_633,totalSample,immune-Effector_memory; Homo_633,totalSample,immune-Tr1; Homo_633,totalSample,immune-NKT; Homo_633,totalSample,immune-Monocyte; Homo_633,totalSample,immune-iTreg; Homo_633,totalSample,immune-Exhausted; Homo_633,totalSample,immune-Bcell; Homo_633,totalSample,immune-Gamma_delta; Homo_633,totalSample,immune-Th17; MCSA,totalSample,immune-DC; MCSA,totalSample,immune-Monocyte; MCSA,totalSample,immune-Macrophage; MCSA,totalSample,immune-CD4_T; MCSA,totalSample,immune-CD8_T; MCSA,totalSample,immune-CD4_naive; MCSA,totalSample,immune-Tr1; MCSA,totalSample,immune-nTreg; MCSA,totalSample,immune-iTreg; MCSA,totalSample,immune-Th17; MCSA,totalSample,immune-Tfh; MCSA,totalSample,immune-CD8_naive; MCSA,totalSample,immune-Cytotoxic; MCSA,totalSample,immune-Exhausted; MCSA,totalSample,immune-MAIT; MCSA,totalSample,immune-Central_memory; [illegible]
SCHIP1	Homo_723,female,AD.vs.control; Homo_723,male,AD.vs.control; Homo_723,female,AD.vs.MCI; Homo_723,male,AD.vs.MCI; Homo_723,female,MCI.vs.control; Homo_723,male,MCI.vs.control; Homo_714,female,AD.vs.control; Homo_714,male,AD.vs.control; Homo_714,female,AD.vs.MCI; Homo_714,male,AD.vs.MCI; Homo_714,female,MCI.vs.control; Homo_714,male,MCI.vs.control; Homo_633,female,AD.vs.control; Homo_633,female,AD.vs.MCI; ROSMAP,female,AD.vs.control; Emory,control,age; Homo_723,totalSample,immune-Exhausted; Homo_723,totalSample,immune-Neutrophil; Homo_723,totalSample,immune-Th17; Homo_714,totalSample,immune-InfiltrationScore; Homo_714,totalSample,immune-Exhausted; Homo_633,totalSample,immune-InfiltrationScore; Homo_633,totalSample,immune-Tfh; MCSA,totalSample,immune-DC; MCSA,totalSample,immune-Monocyte; MCSA,totalSample,immune-CD4_T; MCSA,totalSample,immune-NKT; MCSA,totalSample,immune-CD4_naive; MCSA,totalSample,immune-Tr1; MCSA,totalSample,immune-iTreg; MCSA,totalSample,immune-CD8_naive; MCSA,totalSample,immune-Exhausted; MCSA,totalSample,immune-MAIT; MCSA,totalSample,immune-Central_memory; ROSMAP,totalSample,immune-Effector_memory
ARHGAP33	Homo_723,female,AD.vs.control; Homo_723,male,AD.vs.control; Homo_723,female,AD.vs.MCI; Homo_723,male,AD.vs.MCI; Homo_714,female,AD.vs.control; Homo_714,female,AD.vs.MCI; Homo_633,female,AD.vs.control; Homo_633,female,AD.vs.MCI; Homo_633,female,MCI.vs.control; ROSMAP,female,AD.vs.control; ROSMAP,female,AD.vs.MCI; ROSMAP,male,MCI.vs.control; ROSMAP,MCI,male.vs.female; MCSA,totalSample,age; MCSA,control,age; MCSA,MCI,age; Homo_723,totalSample,immune-DC; Homo_723,totalSample,immune-Monocyte; Homo_723,totalSample,immune-Neutrophil; Homo_723,totalSample,immune-Exhausted; Homo_714,totalSample,immune-Monocyte; Homo_714,totalSample,immune-DC; Homo_714,totalSample,immune-Th2; Homo_633,totalSample,immune-Monocyte; Homo_633,totalSample,immune-DC; MCSA,totalSample,immune-DC; MCSA,totalSample,immune-Monocyte; MCSA,totalSample,immune-Neutrophil; MCSA,totalSample,immune-CD8_T; MCSA,totalSample,immune-iTreg; MCSA,totalSample,immune-Th2; MCSA,totalSample,immune-Cytotoxic; MCSA,totalSample,immune-Exhausted; MCSA,totalSample,immune-Effector_memory; MCSA,totalSample,immune-InfiltrationScore
NINL	Homo_723,female,AD.vs.control; Homo_723,male,AD.vs.control; Homo_723,female,AD.vs.MCI; Homo_723,male,AD.vs.MCI; Homo_714,female,AD.vs.control; Homo_714,male,AD.vs.control; Homo_714,male,AD.vs.MCI; Homo_633,female,AD.vs.control; Homo_633,female,AD.vs.MCI; Emory,totalSample,immune-Neutrophil; Emory,totalSample,immune-InfiltrationScore; Homo_723,totalSample,immune-InfiltrationScore; Homo_723,totalSample,immune-Neutrophil; Homo_723,totalSample,immune-CD4_naive; Homo_723,totalSample,immune-nTreg; Homo_723,totalSample,immune-NK; Homo_723,totalSample,immune-Effector_memory; Homo_723,totalSample,immune-CD4_T; Homo_723,totalSample,immune-Central_memory; Homo_723,totalSample,immune-Tfh; Homo_723,totalSample,immune-DC; Homo_714,totalSample,immune-InfiltrationScore; Homo_714,totalSample,immune-Neutrophil; Homo_714,totalSample,immune-CD4_naive; Homo_714,totalSample,immune-Effector_memory; Homo_714,totalSample,immune-NK; Homo_633,totalSample,immune-InfiltrationScore; Homo_633,totalSample,immune-Effector_memory; Homo_633,totalSample,immune-CD4_naive; Homo_633,totalSample,immune-Neutrophil; Homo_633,totalSample,immune-Tfh; Homo_633,totalSample,immune-Central_memory; Homo_633,totalSample,immune-CD4_T; MCSA,totalSample,immune-Macrophage; MCSA,totalSample,immune-Neutrophil; MCSA,totalSample,immune-CD4_T; MCSA,totalSample,immune-CD8_T; MCSA,totalSample,immune-NKT; MCSA,totalSample,immune-CD4_naive; MCSA,totalSample,immune-Tr1; MCSA,totalSample,immune-nTreg; MCSA,totalSample,immune-iTreg; MCSA,totalSample,immune-Tfh; MCSA,totalSample,immune-CD8_naive; MCSA,totalSample,immune-MAIT;
DST	Homo_723,female,AD.vs.control; Homo_723,male,AD.vs.control; Homo_723,female,AD.vs.MCI; Homo_723,male,AD.vs.MCI; Homo_723,female,MCI.vs.control; Homo_723,male,MCI.vs.control; Homo_714,female,AD.vs.control; Homo_714,male,AD.vs.control; Homo_714,female,AD.vs.MCI; Homo_714,male,AD.vs.MCI; Homo_714,female,MCI.vs.control; Homo_714,male,MCI.vs.control; Homo_633,female,AD.vs.control; Homo_633,female,AD.vs.MCI; MCSA,MCI,age; Emory,totalSample,immune-CD4_T; Emory,totalSample,immune-Gamma_delta; Emory,totalSample,immune-Tr1; Emory,totalSample,immune-iTreg; Emory,totalSample,immune-Th17; Emory,totalSample,immune-Cytotoxic; Emory,totalSample,immune-InfiltrationScore; Homo_723,totalSample,immune-Exhausted; Homo_723,totalSample,immune-Th17; Homo_723,totalSample,immune-CD4_naive; Homo_723,totalSample,immune-Cytotoxic; Homo_723,totalSample,immune-CD4_T; Homo_723,totalSample,immune-Central_memory; Homo_723,totalSample,immune-CD8_T; Homo_723,totalSample,immune-Tfh; Homo_723,totalSample,immune-Tr1; Homo_723,totalSample,immune-nTreg; Homo_723,totalSample,immune-InfiltrationScore; Homo_723,totalSample,immune-Th2; Homo_723,totalSample,immune-Gamma_delta; Homo_723,totalSample,immune-Th1; Homo_714,totalSample,immune-Th17; Homo_714,totalSample,immune-Exhausted; Homo_714,totalSample,immune-CD4_naive; Homo_714,totalSample,immune-Central_memory; Homo_714,totalSample,immune-CD4_T; Homo_714,totalSample,immune-Cytotoxic; Homo_714,totalSample,immune-Tfh; Homo_714,totalSample,immune-InfiltrationScore; Homo_714,totalSample,immune-Gamma_delta; Homo_714,totalSample,immune-CD8_T; Homo_714,totalSample,immune-Tr1; Homo_714,totalSample,immune-nTreg; Homo_714,totalSample,immune-NKT; Homo_633,totalSample,immune-Tfh; Homo_633,totalSample,immune-CD4_T; Homo_633,totalSample,immune-Th2; Homo_633,totalSample,immune-CD4_naive; Homo_633,totalSample,immune-Effector_memory; Homo_633,totalSample,immune-Central_memory; Homo_633,totalSample,immune-Tr1; Homo_633,totalSample,immune-Exhausted; Homo_633,totalSample,immune-Gamma_delta; Homo_633,totalSample,immune-Th17; Homo_633,totalSample,immune-InfiltrationScore; Homo_633,totalSample,immune-iTreg; Homo_633,totalSample,immune-nTreg; Homo_633,totalSample,immune-NKT; Homo_633,totalSample,immune-Cytotoxic; MCSA,totalSample,immune-CD4_T; MCSA,totalSample,immune-NKT; MCSA,totalSample,immune-Gamma_delta; MCSA,totalSample,immune-CD4_naive; MCSA,totalSample,immune-Tr1; MCSA,totalSample,immune-nTreg; MCSA,totalSample,immune-iTreg; MCSA,totalSample,immune-Th1; MCSA,totalSample,immune-Th17; MCSA,totalSample,immune-Tfh; MCSA,totalSample,immune-CD8_naive; MCSA,totalSample,immune-Cytotoxic; MCSA,totalSample,immune-Exhausted; MCSA,totalSample,immune-Central_memory; ROSMAP,totalSample,immune-InfiltrationScore; SRP223445,totalSample,immune-CD4_naive; SRP223445,totalSample,immune-InfiltrationScore; ROSMAP,male-includeAge90+,survival
HOMER1	Homo_723,female,AD.vs.control; Homo_723,male,AD.vs.control; Homo_723,female,AD.vs.MCI; Homo_723,male,AD.vs.MCI; Homo_723,male,MCI.vs.control; Homo_714,female,AD.vs.control; Homo_714,male,AD.vs.control; Homo_714,female,AD.vs.MCI; Homo_714,male,AD.vs.MCI; Homo_633,female,AD.vs.control; Homo_633,female,AD.vs.MCI; ROSMAP,AD,MMSE; Emory,totalSample,immune-Neutrophil; Emory,totalSample,immune-InfiltrationScore; Homo_723,totalSample,immune-CD4_naive; Homo_723,totalSample,immune-Th1; Homo_723,totalSample,immune-Central_memory; Homo_723,totalSample,immune-NKT; Homo_723,totalSample,immune-Neutrophil; Homo_723,totalSample,immune-CD4_T; Homo_723,totalSample,immune-InfiltrationScore; Homo_723,totalSample,immune-Tr1; Homo_723,totalSample,immune-Tfh; Homo_723,totalSample,immune-Cytotoxic; Homo_723,totalSample,immune-NK; Homo_714,totalSample,immune-NKT; Homo_714,totalSample,immune-CD4_naive; Homo_714,totalSample,immune-InfiltrationScore; Homo_714,totalSample,immune-Th1; Homo_714,totalSample,immune-Neutrophil; Homo_633,totalSample,immune-Tfh; Homo_633,totalSample,immune-CD4_naive; Homo_633,totalSample,immune-NKT; Homo_633,totalSample,immune-InfiltrationScore; Homo_633,totalSample,immune-CD4_T; Homo_633,totalSample,immune-Effector_memory; Homo_633,totalSample,immune-Central_memory; Homo_633,totalSample,immune-Tr1; Homo_633,totalSample,immune-iTreg; Homo_633,totalSample,immune-MAIT; MCSA,totalSample,immune-DC; MCSA,totalSample,immune-Monocyte; MCSA,totalSample,immune-Macrophage; MCSA,totalSample,immune-CD4_T; MCSA,totalSample,immune-CD4_naive; MCSA,totalSample,immune-Tr1; MCSA,totalSample,immune-nTreg; MCSA,totalSample,immune-iTreg; MCSA,totalSample,immune-Tfh; MCSA,totalSample,immune-CD8_naive; MCSA,totalSample,immune-Cytotoxic; MCSA,totalSample,immune-Exhausted; MCSA,totalSample,immune-MAIT; MCSA,totalSample,immune-Central_memory; ROSMAP,totalSample,immune-Th17; ROSMAP,totalSample,immune-InfiltrationScore
GUCY1A1	Homo_723,female,AD.vs.control; Homo_723,male,AD.vs.control; Homo_723,female,AD.vs.MCI; Homo_723,male,AD.vs.MCI; Homo_723,female,MCI.vs.control; Homo_723,male,MCI.vs.control; Homo_714,female,AD.vs.control; Homo_714,male,AD.vs.control; Homo_714,female,AD.vs.MCI; Homo_714,male,AD.vs.MCI; Homo_714,female,MCI.vs.control; Homo_714,male,MCI.vs.control; Homo_633,female,AD.vs.control; Homo_633,female,AD.vs.MCI; ROSMAP,female,MCI.vs.control; Emory,totalSample,immune-DC; Homo_723,totalSample,immune-DC; Homo_723,totalSample,immune-Th17; Homo_723,totalSample,immune-Cytotoxic; Homo_723,totalSample,immune-CD8_T; Homo_723,totalSample,immune-CD4_naive; Homo_723,totalSample,immune-Central_memory; Homo_723,totalSample,immune-Th1; Homo_723,totalSample,immune-NK; Homo_714,totalSample,immune-CD8_T; Homo_714,totalSample,immune-Exhausted; Homo_714,totalSample,immune-NKT; Homo_714,totalSample,immune-Th17; Homo_714,totalSample,immune-Cytotoxic; Homo_714,totalSample,immune-Th1; Homo_714,totalSample,immune-NK; Homo_633,totalSample,immune-NKT; Homo_633,totalSample,immune-Tfh; Homo_633,totalSample,immune-InfiltrationScore; Homo_633,totalSample,immune-Gamma_delta; Homo_633,totalSample,immune-CD4_naive; Homo_633,totalSample,immune-CD4_T; Homo_633,totalSample,immune-iTreg; MCSA,totalSample,immune-CD4_T; MCSA,totalSample,immune-CD8_T; MCSA,totalSample,immune-Gamma_delta; MCSA,totalSample,immune-CD4_naive; MCSA,totalSample,immune-Tr1; MCSA,totalSample,immune-nTreg; MCSA,totalSample,immune-iTreg; MCSA,totalSample,immune-Th17; MCSA,totalSample,immune-Tfh; MCSA,totalSample,immune-Cytotoxic; MCSA,totalSample,immune-Exhausted; MCSA,totalSample,immune-Central_memory; ROSMAP,totalSample,immune-Th17; ROSMAP,totalSample,immune-InfiltrationScore; SRP223445,totalSample,immune-CD4_naive; SRP223445,totalSample,immune-Exhausted
IL15	Homo_723,female,AD.vs.control; Homo_723,male,AD.vs.control; Homo_723,female,AD.vs.MCI; Homo_723,male,AD.vs.MCI; Homo_714,female,AD.vs.control; Homo_714,male,AD.vs.control; Homo_714,female,AD.vs.MCI; Homo_714,male,AD.vs.MCI; Homo_633,female,AD.vs.control; Homo_633,female,AD.vs.MCI; MCSA,MCI,age; ROSMAP,MCI,sample.age; Emory,totalSample,immune-Monocyte; Emory,totalSample,immune-NK; Emory,totalSample,immune-CD4_naive; Emory,totalSample,immune-nTreg; Emory,totalSample,immune-CD8_naive; Emory,totalSample,immune-Effector_memory; Emory,totalSample,immune-InfiltrationScore; Homo_723,totalSample,immune-Monocyte; Homo_723,totalSample,immune-InfiltrationScore; Homo_723,totalSample,immune-Tfh; Homo_723,totalSample,immune-Th2; Homo_723,totalSample,immune-DC; Homo_723,totalSample,immune-iTreg; Homo_723,totalSample,immune-Effector_memory; Homo_723,totalSample,immune-Gamma_delta; Homo_723,totalSample,immune-Tr1; Homo_723,totalSample,immune-CD4_T; Homo_723,totalSample,immune-Bcell; Homo_714,totalSample,immune-Th2; Homo_714,totalSample,immune-Monocyte; Homo_714,totalSample,immune-Tfh; Homo_714,totalSample,immune-Effector_memory; Homo_714,totalSample,immune-InfiltrationScore; Homo_714,totalSample,immune-CD4_T; Homo_633,totalSample,immune-Monocyte; Homo_633,totalSample,immune-Th2; Homo_633,totalSample,immune-Tfh; Homo_633,totalSample,immune-InfiltrationScore; Homo_633,totalSample,immune-Effector_memory; Homo_633,totalSample,immune-Exhausted; Homo_633,totalSample,immune-Gamma_delta; Homo_633,totalSample,immune-CD4_T; Homo_633,totalSample,immune-CD4_naive; Homo_633,totalSample,immune-NKT; Homo_633,totalSample,immune-Th17; Homo_633,totalSample,immune-Bcell; MCSA,totalSample,immune-DC; MCSA,totalSample,immune-Monocyte; MCSA,totalSample,immune-CD4_T; MCSA,totalSample,immune-CD8_T; MCSA,totalSample,immune-CD4_naive; MCSA,totalSample,immune-Tr1; MCSA,totalSample,immune-nTreg; MCSA,totalSample,immune-iTreg;
TRAT1	Homo_723,female,AD.vs.control; Homo_723,male,AD.vs.control; Homo_723,female,AD.vs.MCI; Homo_723,male,AD.vs.MCI; Homo_714,female,AD.vs.control; Homo_714,male,AD.vs.control; Homo_714,female,AD.vs.MCI; Homo_714,male,AD.vs.MCI; Homo_633,female,AD.vs.control; Homo_633,female,AD.vs.MCI; ROSMAP,female,AD.vs.control; Emory,totalSample,age; Emory,MCI,age; MCSA,MCI,age; Emory,totalSample,immune-DC; Emory,totalSample,immune-Monocyte; Emory,totalSample,immune-Macrophage; Emory,totalSample,immune-Neutrophil; Emory,totalSample,immune-CD4_T; Emory,totalSample,immune-CD8_T; Emory,totalSample,immune-Tr1; Emory,totalSample,immune-iTreg; Emory,totalSample,immune-Tfh; Emory,totalSample,immune-CD8_naive; Emory,totalSample,immune-MAIT; Emory,totalSample,immune-Central_memory; Emory,totalSample,immune-InfiltrationScore; Homo_723,totalSample,immune-Tfh; Homo_723,totalSample,immune-iTreg; Homo_723,totalSample,immune-Tr1; Homo_723,totalSample,immune-Th2; Homo_723,totalSample,immune-DC; Homo_723,totalSample,immune-CD4_T; Homo_723,totalSample,immune-CD4_naive; Homo_723,totalSample,immune-Central_memory; Homo_723,totalSample,immune-MAIT; Homo_723,totalSample,immune-Monocyte; Homo_723,totalSample,immune-nTreg; Homo_723,totalSample,immune-Effector_memory; Homo_723,totalSample,immune-CD8_T; Homo_723,totalSample,immune-InfiltrationScore; Homo_723,totalSample,immune-Macrophage; Homo_723,totalSample,immune-Th1; Homo_723,totalSample,immune-Gamma_delta; Homo_714,totalSample,immune-Tfh; Homo_714,totalSample,immune-Th2; Homo_714,totalSample,immune-Tr1; Homo_714,totalSample,immune-CD4_T; Homo_714,totalSample,immune-Central_memory; Homo_714,totalSample,immune-iTreg; Homo_714,totalSample,immune-CD4_naive; Homo_714,totalSample,immune-Effector_memory; Homo_714,totalSample,immune-MAIT; Homo_714,totalSample,immune-DC; Homo_714,totalSample,immune-nTreg; Homo_714,totalSample,immune-Th1; Homo_714,totalSample,immune-Neutrophil; Homo_714,totalSample,immune-Monocyte; Homo_633,totalSample,immune-Tfh; Homo_633,totalSample,immune-Central_memory; Homo_633,totalSample,immune-CD4_T; Homo_633,totalSample,immune-CD4_naive; Homo_633,totalSample,immune-Tr1; Homo_633,totalSample,immune-Th1; Homo_633,totalSample,immune-iTreg; Homo_633,totalSample,immune-Th2; Homo_633,totalSample,immune-Effector_memory; Homo_633,totalSample,immune-MAIT; Homo_633,totalSample,immune-Neutrophil; Homo_633,totalSample,immune-NKT; Homo_633,totalSample,immune-Monocyte; Homo_633,totalSample,immune-DC; Homo_633,totalSample,immune-nTreg; Homo_633,totalSample,immune-Bcell; MCSA,totalSample,immune-DC; MCSA,totalSample,immune-Monocyte; MCSA,totalSample,immune-Macrophage; MCSA,totalSample,immune-CD4_T; MCSA,totalSample,immune-CD4_naive; MCSA,totalSample,immune-Tr1; MCSA,totalSample,immune-nTreg; MCSA,totalSample,immune-iTreg; MCSA,totalSample,immune-Th2; MCSA,totalSample,immune-Tfh; [illegible]
MAPK13	Homo_723,female,AD.vs.control; Homo_723,male,AD.vs.control; Homo_723,female,AD.vs.MCI; Homo_723,male,AD.vs.MCI; Homo_714,female,AD.vs.control; Homo_714,male,AD.vs.control; Homo_714,female,AD.vs.MCI; Homo_714,male,AD.vs.MCI; Homo_633,female,AD.vs.control; Homo_633,female,AD.vs.MCI; Emory,MCI,age; MCSA,totalSample,age; MCSA,control,age; ROSMAP,MCI,last.visit.age; Emory,totalSample,immune-CD4_T; Emory,totalSample,immune-Gamma_delta; Emory,totalSample,immune-Tr1; Emory,totalSample,immune-iTreg; Homo_723,totalSample,immune-InfiltrationScore; Homo_723,totalSample,immune-Th2; Homo_723,totalSample,immune-Tfh; Homo_723,totalSample,immune-iTreg; Homo_723,totalSample,immune-nTreg; Homo_723,totalSample,immune-Effector_memory; Homo_723,totalSample,immune-Gamma_delta; Homo_723,totalSample,immune-CD4_T; Homo_723,totalSample,immune-Tr1; Homo_723,totalSample,immune-Bcell; Homo_723,totalSample,immune-Neutrophil; Homo_714,totalSample,immune-Th2; Homo_714,totalSample,immune-Tfh; Homo_714,totalSample,immune-InfiltrationScore; Homo_714,totalSample,immune-nTreg; Homo_714,totalSample,immune-Effector_memory; Homo_714,totalSample,immune-Tr1; Homo_714,totalSample,immune-CD4_T; Homo_714,totalSample,immune-Gamma_delta; Homo_714,totalSample,immune-iTreg; Homo_633,totalSample,immune-InfiltrationScore; Homo_633,totalSample,immune-nTreg; Homo_633,totalSample,immune-Th2; Homo_633,totalSample,immune-Exhausted; Homo_633,totalSample,immune-Tfh; Homo_633,totalSample,immune-Th17; Homo_633,totalSample,immune-Tr1; Homo_633,totalSample,immune-CD4_T; Homo_633,totalSample,immune-Gamma_delta; Homo_633,totalSample,immune-Effector_memory; Homo_633,totalSample,immune-Central_memory; MCSA,totalSample,immune-Monocyte; MCSA,totalSample,immune-Gamma_delta; MCSA,totalSample,immune-CD4_naive; MCSA,totalSample,immune-nTreg; MCSA,totalSample,immune-Th1; MCSA,totalSample,immune-Th2; MCSA,totalSample,immune-Th17; MCSA,totalSample,immune-Exhausted; SRP223445,totalSample,immune-NK; SRP223445,totalSample,immune-Neutrophil; SRP223445,totalSample,immune-CD8_T; SRP223445,totalSample,immune-Th17; SRP223445,totalSample,immune-InfiltrationScore
VPS13A	Homo_723,female,AD.vs.control; Homo_723,male,AD.vs.control; Homo_723,female,AD.vs.MCI; Homo_723,male,AD.vs.MCI; Homo_723,female,MCI.vs.control; Homo_723,male,MCI.vs.control; Homo_714,female,AD.vs.control; Homo_714,male,AD.vs.control; Homo_714,female,AD.vs.MCI; Homo_714,male,AD.vs.MCI; Homo_714,female,MCI.vs.control; Homo_714,male,MCI.vs.control; Homo_633,female,AD.vs.control; Homo_633,female,AD.vs.MCI; Homo_633,female,MCI.vs.control; ROSMAP,female,MCI.vs.control; ROSMAP,control,male.vs.female; MCSA,MCI,age; ROSMAP,AD,sample.age; Emory,totalSample,immune-Macrophage; Emory,totalSample,immune-Neutrophil; Emory,totalSample,immune-CD4_T; Emory,totalSample,immune-CD8_T; Emory,totalSample,immune-Tr1; Emory,totalSample,immune-iTreg; Emory,totalSample,immune-Tfh; Emory,totalSample,immune-CD8_naive; Emory,totalSample,immune-Exhausted; Emory,totalSample,immune-MAIT; Emory,totalSample,immune-Central_memory; Emory,totalSample,immune-Effector_memory; Emory,totalSample,immune-InfiltrationScore; Homo_723,totalSample,immune-Exhausted; Homo_723,totalSample,immune-Th17; Homo_723,totalSample,immune-CD4_naive; Homo_723,totalSample,immune-CD4_T; Homo_723,totalSample,immune-Tfh; Homo_723,totalSample,immune-Cytotoxic; Homo_723,totalSample,immune-Tr1; Homo_723,totalSample,immune-Central_memory; Homo_723,totalSample,immune-Th2; Homo_723,totalSample,immune-nTreg; Homo_723,totalSample,immune-CD8_T; Homo_723,totalSample,immune-InfiltrationScore; Homo_723,totalSample,immune-Th1; Homo_723,totalSample,immune-Effector_memory; Homo_723,totalSample,immune-Neutrophil; Homo_723,totalSample,immune-Monocyte; Homo_723,totalSample,immune-iTreg; Homo_714,totalSample,immune-CD4_naive; Homo_714,totalSample,immune-Th17; Homo_714,totalSample,immune-CD4_T; Homo_714,totalSample,immune-Tfh; Homo_714,totalSample,immune-Central_memory; Homo_714,totalSample,immune-InfiltrationScore; Homo_714,totalSample,immune-Monocyte; Homo_714,totalSample,immune-Exhausted; Homo_714,totalSample,immune-Tr1; Homo_714,totalSample,immune-Effector_memory; Homo_714,totalSample,immune-Th2; Homo_633,totalSample,immune-Tfh; Homo_633,totalSample,immune-CD4_T; Homo_633,totalSample,immune-Th2; Homo_633,totalSample,immune-CD4_naive; Homo_633,totalSample,immune-Effector_memory; Homo_633,totalSample,immune-Central_memory; Homo_633,totalSample,immune-InfiltrationScore; Homo_633,totalSample,immune-Tr1; Homo_633,totalSample,immune-Monocyte; Homo_633,totalSample,immune-iTreg; Homo_633,totalSample,immune-Th17; Homo_633,totalSample,immune-nTreg; Homo_633,totalSample,immune-Exhausted; Homo_633,totalSample,immune-NKT; Homo_633,totalSample,immune-DC; Homo_633,totalSample,immune-Bcell; Homo_633,totalSample,immune-Gamma_delta; Homo_633,totalSample,immune-MAIT; MCSA,totalSample,immune-DC; MCSA,totalSample,immune-Macrophage; MCSA,totalSample,immune-CD4_T; MCSA,totalSample,immune-CD4_naive; MCSA,totalSample,immune-Tr1; MCSA,totalSample,immune-nTreg; MCSA,totalSample,immune-iTreg; MCSA,totalSample,immune-Th17; MCSA,totalSample,immune-Tfh; MCSA,totalSample,immune-CD8_naive; MCSA,totalSample,immune-Cytotoxic; MCSA,totalSample,immune-Exhausted; MCSA,totalSample,immune-MAIT; MCSA,totalSample,immune-Central_memory; [illegible]

RPL24	Homo_723,female,AD.vs.control; Homo_723,male,AD.vs.control; Homo_723,female,AD.vs.MCI; Homo_723,male,AD.vs.MCI; Homo_723,female,MCI.vs.control; Homo_723,male,MCI.vs.control; Homo_714,female,AD.vs.control; Homo_714,male,AD.vs.control; Homo_714,female,AD.vs.MCI; Homo_714,male,AD.vs.MCI; Homo_714,female,MCI.vs.control; Homo_714,male,MCI.vs.control; Homo_714,MCI,male.vs.female; Homo_633,female,AD.vs.control; Homo_633,female,AD.vs.MCI; Homo_633,female,MCI.vs.control; Homo_633,male,MCI.vs.control; Homo_633,control,male.vs.female; Homo_633,MCI,male.vs.female; ROSMAP,female,AD.vs.control; ROSMAP,female,MCI.vs.control; Emory,MCI,age; MCSA,totalSample,age; MCSA,control,age; Emory,totalSample,immune-DC; Emory,totalSample,immune-Macrophage; Emory,totalSample,immune-Neutrophil; Emory,totalSample,immune-CD4_T; Emory,totalSample,immune-CD8_T; Emory,totalSample,immune-Gamma_delta; Emory,totalSample,immune-Tr1; Emory,totalSample,immune-iTreg; Emory,totalSample,immune-Th1; Emory,totalSample,immune-Tfh; Emory,totalSample,immune-CD8_naive; Emory,totalSample,immune-Cytotoxic; Emory,totalSample,immune-Exhausted; Emory,totalSample,immune-MAIT; Emory,totalSample,immune-Central_memory; Homo_723,totalSample,immune-Neutrophil; Homo_723,totalSample,immune-Exhausted; Homo_723,totalSample,immune-Th1; Homo_714,totalSample,immune-Th1; Homo_714,totalSample,immune-Neutrophil; Homo_633,totalSample,immune-Th1; MCSA,totalSample,immune-DC; MCSA,totalSample,immune-Monocyte; MCSA,totalSample,immune-Macrophage; MCSA,totalSample,immune-Neutrophil; MCSA,totalSample,immune-Gamma_delta; MCSA,totalSample,immune-Th1; MCSA,totalSample,immune-Th2; MCSA,totalSample,immune-Th17; MCSA,totalSample,immune-Exhausted; ROSMAP,totalSample,immune-CD4_T; ROSMAP,totalSample,immune-Th2; SRP223445,totalSample,immune-NK; SRP223445,totalSample,immune-CD4_naive; SRP223445,totalSample,immune-Th17; SRP223445,totalSample,immune-Exhausted;
MIIP	Homo_723,female,AD.vs.control; Homo_723,male,AD.vs.control; Homo_723,male,AD.vs.MCI; Homo_723,female,MCI.vs.control; Homo_723,MCI,male.vs.female; Homo_714,female,AD.vs.control; Homo_714,male,AD.vs.control; Homo_714,male,AD.vs.MCI; Homo_714,female,MCI.vs.control; Homo_714,MCI,male.vs.female; Homo_633,female,AD.vs.control; Homo_633,female,AD.vs.MCI; Homo_633,female,MCI.vs.control; Homo_633,male,MCI.vs.control; Homo_633,MCI,male.vs.female; MCSA,MCI,age; Emory,totalSample,immune-DC; Emory,totalSample,immune-NK; Emory,totalSample,immune-CD4_T; Emory,totalSample,immune-Tr1; Emory,totalSample,immune-iTreg; Emory,totalSample,immune-Th17; Homo_723,totalSample,immune-DC; Homo_723,totalSample,immune-Monocyte; Homo_723,totalSample,immune-Th2; Homo_714,totalSample,immune-DC; Homo_714,totalSample,immune-Th2; Homo_714,totalSample,immune-Monocyte; Homo_633,totalSample,immune-Monocyte; Homo_633,totalSample,immune-DC; Homo_633,totalSample,immune-Th2; MCSA,totalSample,immune-DC; MCSA,totalSample,immune-Monocyte; MCSA,totalSample,immune-Macrophage; MCSA,totalSample,immune-Neutrophil; MCSA,totalSample,immune-CD8_T; MCSA,totalSample,immune-NKT; MCSA,totalSample,immune-iTreg; MCSA,totalSample,immune-Th2; MCSA,totalSample,immune-Cytotoxic; MCSA,totalSample,immune-Effector_memory; MCSA,totalSample,immune-InfiltrationScore; ROSMAP,totalSample,immune-MAIT; ROSMAP,totalSample,immune-Effector_memory; SRP223445,totalSample,immune-CD4_naive; SRP223445,totalSample,immune-Th17; SRP223445,totalSample,immune-Exhausted; SRP223445,totalSample,immune-InfiltrationScore; ROSMAP,male and female-
XCL2	Homo_723,female,AD.vs.control; Homo_723,male,AD.vs.control; Homo_723,female,AD.vs.MCI; Homo_723,male,AD.vs.MCI; Homo_714,female,AD.vs.control; Homo_714,male,AD.vs.control; Homo_714,female,AD.vs.MCI; Homo_714,male,AD.vs.MCI; Homo_633,female,AD.vs.control; Homo_633,female,AD.vs.MCI; ROSMAP,MCI,MMSE; Emory,totalSample,immune-Macrophage; Emory,totalSample,immune-NK; Emory,totalSample,immune-Neutrophil; Emory,totalSample,immune-CD8_T; Emory,totalSample,immune-Gamma_delta; Emory,totalSample,immune-CD4_naive; Emory,totalSample,immune-Th17; Emory,totalSample,immune-Tfh; Emory,totalSample,immune-Cytotoxic; Emory,totalSample,immune-Exhausted; Emory,totalSample,immune-Effector_memory; Emory,totalSample,immune-InfiltrationScore; Homo_723,totalSample,immune-InfiltrationScore; Homo_723,totalSample,immune-Effector_memory; Homo_723,totalSample,immune-Cytotoxic; Homo_723,totalSample,immune-MAIT; Homo_723,totalSample,immune-Macrophage; Homo_723,totalSample,immune-Tfh; Homo_723,totalSample,immune-CD8_T; Homo_723,totalSample,immune-NK; Homo_714,totalSample,immune-Cytotoxic; Homo_714,totalSample,immune-InfiltrationScore; Homo_714,totalSample,immune-Effector_memory; Homo_714,totalSample,immune-CD8_T; Homo_714,totalSample,immune-MAIT; Homo_714,totalSample,immune-Macrophage; Homo_714,totalSample,immune-Neutrophil; Homo_714,totalSample,immune-Tfh; Homo_714,totalSample,immune-Gamma_delta; Homo_714,totalSample,immune-NK; Homo_714,totalSample,immune-Bcell; Homo_633,totalSample,immune-InfiltrationScore; Homo_633,totalSample,immune-Cytotoxic; Homo_633,totalSample,immune-Neutrophil; Homo_633,totalSample,immune-Effector_memory; Homo_633,totalSample,immune-NK; Homo_633,totalSample,immune-MAIT; Homo_633,totalSample,immune-Gamma_delta; Homo_633,totalSample,immune-CD8_T; Homo_633,totalSample,immune-Bcell; Homo_633,totalSample,immune-Macrophage; MCSA,totalSample,immune-Macrophage; MCSA,totalSample,immune-
HDAC1	Homo_723,female,AD.vs.control; Homo_723,male,AD.vs.control; Homo_723,female,AD.vs.MCI; Homo_723,male,AD.vs.MCI; Homo_714,female,AD.vs.control; Homo_714,male,AD.vs.control; Homo_714,female,AD.vs.MCI; Homo_714,male,AD.vs.MCI; Homo_633,female,AD.vs.control; Homo_633,female,AD.vs.MCI; Homo_633,female,MCI.vs.control; ROSMAP,female,AD.vs.control; ACOM,totalSample,age; MCSA,totalSample,age; MCSA,control,age; Emory,totalSample,immune-Macrophage; Emory,totalSample,immune-NK; Emory,totalSample,immune-Neutrophil; Emory,totalSample,immune-CD4_T; Emory,totalSample,immune-CD8_T; Emory,totalSample,immune-Tr1; Emory,totalSample,immune-iTreg; Emory,totalSample,immune-Exhausted; Emory,totalSample,immune-MAIT; Emory,totalSample,immune-Central_memory; Emory,totalSample,immune-Effector_memory; Emory,totalSample,immune-InfiltrationScore; Homo_723,totalSample,immune-Th2; Homo_723,totalSample,immune-Tfh; Homo_723,totalSample,immune-iTreg; Homo_723,totalSample,immune-Effector_memory; Homo_723,totalSample,immune-InfiltrationScore; Homo_723,totalSample,immune-CD4_T; Homo_723,totalSample,immune-CD4_naive; Homo_723,totalSample,immune-Tr1; Homo_723,totalSample,immune-Bcell; Homo_723,totalSample,immune-DC; Homo_723,totalSample,immune-nTreg; Homo_723,totalSample,immune-Macrophage; Homo_723,totalSample,immune-Monocyte; Homo_723,totalSample,immune-Central_memory; Homo_723,totalSample,immune-Gamma_delta; Homo_714,totalSample,immune-Th2; Homo_714,totalSample,immune-Tfh; Homo_714,totalSample,immune-Effector_memory; Homo_714,totalSample,immune-CD4_T; Homo_714,totalSample,immune-Tr1; Homo_714,totalSample,immune-Central_memory; Homo_714,totalSample,immune-InfiltrationScore; Homo_714,totalSample,immune-CD4_naive; Homo_714,totalSample,immune-iTreg; Homo_714,totalSample,immune-nTreg; Homo_714,totalSample,immune-Bcell; Homo_633,totalSample,immune-Th2; Homo_633,totalSample,immune-Tfh; Homo_633,totalSample,immune-CD4_T; Homo_633,totalSample,immune-Effector_memory; Homo_633,totalSample,immune-CD4_naive; Homo_633,totalSample,immune-InfiltrationScore; Homo_633,totalSample,immune-Central_memory; Homo_633,totalSample,immune-Tr1; Homo_633,totalSample,immune-nTreg; Homo_633,totalSample,immune-Th17; Homo_633,totalSample,immune-Bcell; Homo_633,totalSample,immune-iTreg; Homo_633,totalSample,immune-Gamma_delta; Homo_633,totalSample,immune-Exhausted; MCSA,totalSample,immune-Monocyte; MCSA,totalSample,immune-Macrophage; MCSA,totalSample,immune-CD4_T; MCSA,totalSample,immune-Gamma_delta; MCSA,totalSample,immune-CD4_naive; MCSA,totalSample,immune-Tr1; MCSA,totalSample,immune-nTreg; MCSA,totalSample,immune-iTreg; MCSA,totalSample,immune-Th17; MCSA,totalSample,immune-Tfh; MCSA,totalSample,immune-CD8_naive; MCSA,totalSample,immune-Exhausted; MCSA,totalSample,immune-MAIT; MCSA,totalSample,immune-Central_memory; MCSA,totalSample,immune-Effector_memory; MCSA,totalSample,immune-InfiltrationScore; ROSMAP,totalSample,immune-Effector_memory; SRP223445,totalSample,immune-NK; SRP223445,totalSample,immune-Neutrophil; SRP223445,totalSample,immune-CD4_naive; SRP223445,totalSample,immune-nTreg; SRP223445,totalSample,immune-Th17; SRP223445,totalSample,immune-Exhausted; [illegible]
KBTBD4	Homo_723,female,AD.vs.control; Homo_723,male,AD.vs.control; Homo_723,female,AD.vs.MCI; Homo_723,male,AD.vs.MCI; Homo_714,female,AD.vs.control; Homo_714,male,AD.vs.control; Homo_714,female,AD.vs.MCI; Homo_714,male,AD.vs.MCI; Homo_633,female,AD.vs.control; Homo_633,female,AD.vs.MCI; ROSMAP,female,AD.vs.control; ROSMAP,female,MCI.vs.control; ROSMAP,control,male.vs.female; MCSA,totalSample,age; MCSA,control,age; ROSMAP,MCI,last.visit.age; Emory,totalSample,immune-Macrophage; Emory,totalSample,immune-NK; Emory,totalSample,immune-Neutrophil; Emory,totalSample,immune-CD4_T; Emory,totalSample,immune-Tr1; Emory,totalSample,immune-iTreg; Emory,totalSample,immune-MAIT; Emory,totalSample,immune-Central_memory; Emory,totalSample,immune-Effector_memory; Emory,totalSample,immune-InfiltrationScore; Homo_723,totalSample,immune-Th2; Homo_723,totalSample,immune-Tfh; Homo_723,totalSample,immune-iTreg; Homo_723,totalSample,immune-Effector_memory; Homo_723,totalSample,immune-InfiltrationScore; Homo_723,totalSample,immune-CD4_T; Homo_723,totalSample,immune-Tr1; Homo_723,totalSample,immune-CD4_naive; Homo_723,totalSample,immune-nTreg; Homo_723,totalSample,immune-Central_memory; Homo_723,totalSample,immune-DC; Homo_723,totalSample,immune-Bcell; Homo_723,totalSample,immune-Gamma_delta; Homo_723,totalSample,immune-Macrophage; Homo_714,totalSample,immune-Th2; Homo_714,totalSample,immune-Tfh; Homo_714,totalSample,immune-Effector_memory; Homo_714,totalSample,immune-CD4_T; Homo_714,totalSample,immune-Tr1; Homo_714,totalSample,immune-InfiltrationScore; Homo_714,totalSample,immune-Central_memory; Homo_714,totalSample,immune-CD4_naive; Homo_714,totalSample,immune-iTreg; Homo_714,totalSample,immune-nTreg; Homo_714,totalSample,immune-Gamma_delta; Homo_633,totalSample,immune-Tfh; Homo_633,totalSample,immune-Effector_memory; Homo_633,totalSample,immune-Th2; Homo_633,totalSample,immune-CD4_T; Homo_633,totalSample,immune-InfiltrationScore; Homo_633,totalSample,immune-CD4_naive; Homo_633,totalSample,immune-Central_memory; Homo_633,totalSample,immune-nTreg; Homo_633,totalSample,immune-Tr1; Homo_633,totalSample,immune-Th17; Homo_633,totalSample,immune-Exhausted; Homo_633,totalSample,immune-iTreg; Homo_633,totalSample,immune-Gamma_delta; Homo_633,totalSample,immune-Bcell; MCSA,totalSample,immune-Monocyte; MCSA,totalSample,immune-Macrophage; MCSA,totalSample,immune-CD4_T; MCSA,totalSample,immune-Gamma_delta; MCSA,totalSample,immune-CD4_naive; MCSA,totalSample,immune-Tr1; MCSA,totalSample,immune-nTreg; MCSA,totalSample,immune-iTreg; MCSA,totalSample,immune-Th17; MCSA,totalSample,immune-Tfh; MCSA,totalSample,immune-CD8_naive; MCSA,totalSample,immune-Exhausted; MCSA,totalSample,immune-MAIT; MCSA,totalSample,immune-Central_memory; MCSA,totalSample,immune-Effector_memory; MCSA,totalSample,immune-InfiltrationScore; ROSMAP,totalSample,immune-Th17; [illegible]
FKBP2	Homo_723,female,AD.vs.control; Homo_723,male,AD.vs.control; Homo_723,female,AD.vs.MCI; Homo_723,male,AD.vs.MCI; Homo_714,female,AD.vs.control; Homo_714,male,AD.vs.control; Homo_714,female,AD.vs.MCI; Homo_714,male,AD.vs.MCI; Homo_633,female,AD.vs.control; Homo_633,female,AD.vs.MCI; ROSMAP,male,AD.vs.control; MCSA,totalSample,age; MCSA,control,age; MCSA,MCI,age; ROSMAP,MCI,last.visit.age; Emory,totalSample,immune-Neutrophil; Emory,totalSample,immune-CD4_T; Emory,totalSample,immune-CD8_T; Emory,totalSample,immune-Tr1; Emory,totalSample,immune-iTreg; Emory,totalSample,immune-Tfh; Emory,totalSample,immune-Exhausted; Emory,totalSample,immune-InfiltrationScore; Homo_723,totalSample,immune-CD8_T; Homo_723,totalSample,immune-Cytotoxic; Homo_723,totalSample,immune-InfiltrationScore; Homo_723,totalSample,immune-Effector_memory; Homo_723,totalSample,immune-Exhausted; Homo_723,totalSample,immune-MAIT; Homo_723,totalSample,immune-Th1; Homo_723,totalSample,immune-Macrophage; Homo_723,totalSample,immune-DC; Homo_723,totalSample,immune-Tfh; Homo_723,totalSample,immune-Bcell; Homo_723,totalSample,immune-Th17; Homo_714,totalSample,immune-CD8_T; Homo_714,totalSample,immune-InfiltrationScore; Homo_714,totalSample,immune-Cytotoxic; Homo_714,totalSample,immune-DC; Homo_714,totalSample,immune-Effector_memory; Homo_714,totalSample,immune-Th1; Homo_714,totalSample,immune-Macrophage; Homo_714,totalSample,immune-Exhausted; Homo_633,totalSample,immune-DC; Homo_633,totalSample,immune-InfiltrationScore; Homo_633,totalSample,immune-CD8_T; Homo_633,totalSample,immune-Cytotoxic; Homo_633,totalSample,immune-Neutrophil; Homo_633,totalSample,immune-NK; Homo_633,totalSample,immune-Effector_memory; Homo_633,totalSample,immune-nTreg; Homo_633,totalSample,immune-Th1; MCSA,totalSample,immune-DC; MCSA,totalSample,immune-Monocyte; MCSA,totalSample,immune-Neutrophil; MCSA,totalSample,immune-CD8_T; MCSA,totalSample,immune-NKT; MCSA,totalSample,immune-iTreg; MCSA,totalSample,immune-Th2; MCSA,totalSample,immune-Cytotoxic; MCSA,totalSample,immune-Exhausted; MCSA,totalSample,immune-Effector_memory; MCSA,totalSample,immune-InfiltrationScore; SRP223445,totalSample,immune-Exhausted; SRP223445,totalSample,immune-InfiltrationScore; ROSMAP,female-[illegible]
PIFO	Homo_723,female,AD.vs.control; Homo_723,male,AD.vs.control; Homo_723,female,AD.vs.MCI; Homo_723,male,AD.vs.MCI; Homo_714,male,AD.vs.control; Homo_714,male,AD.vs.MCI; ROSMAP,AD,last.visit.age; ROSMAP,AD,sample.age; Homo_723,totalSample,immune-Th2; Homo_723,totalSample,immune-iTreg; Homo_723,totalSample,immune-Tfh; Homo_723,totalSample,immune-CD4_T; Homo_723,totalSample,immune-Tr1; Homo_714,totalSample,immune-Th2; Homo_714,totalSample,immune-Tfh; Homo_714,totalSample,immune-Tr1; MCSA,totalSample,immune-Macrophage; MCSA,totalSample,immune-CD4_T; MCSA,totalSample,immune-CD4_naive; MCSA,totalSample,immune-Tr1; MCSA,totalSample,immune-nTreg; MCSA,totalSample,immune-iTreg; MCSA,totalSample,immune-Tfh; MCSA,totalSample,immune-Central_memory
FGF11	Homo_723,female,AD.vs.control; Homo_723,male,AD.vs.control; Homo_723,female,AD.vs.MCI; Homo_723,male,AD.vs.MCI; Homo_723,female,MCI.vs.control; Homo_723,male,MCI.vs.control; Homo_714,female,AD.vs.MCI; Homo_633,female,AD.vs.control; Homo_633,female,AD.vs.MCI; ROSMAP,female,AD.vs.control; ROSMAP,male,AD.vs.control; ROSMAP,female,AD.vs.MCI; ROSMAP,MCI,male.vs.female; MCSA,MCI,age; Homo_723,totalSample,immune-Th17; Homo_714,totalSample,immune-Th17; Homo_633,totalSample,immune-Th2; Homo_633,totalSample,immune-Monocyte; Homo_633,totalSample,immune-Th17; MCSA,totalSample,immune-Monocyte; MCSA,totalSample,immune-NKT; MCSA,totalSample,immune-Th17; MCSA,totalSample,immune-InfiltrationScore; ROSMAP,female-excludeAge90+,survival; ROSMAP,female-includeAge90+,survival
C21orf58	Homo_723,female,AD.vs.control; Homo_723,male,AD.vs.control; Homo_723,female,AD.vs.MCI; Homo_723,male,AD.vs.MCI; Homo_714,female,AD.vs.control; Homo_714,male,AD.vs.control; Homo_714,female,AD.vs.MCI; Homo_714,male,AD.vs.MCI; Homo_633,female,AD.vs.control; Homo_633,female,AD.vs.MCI; Homo_633,female,MCI.vs.control; Homo_633,male,MCI.vs.control; ROSMAP,female,AD.vs.control; MCSA,totalSample,age; MCSA,control,age; ROSMAP,MCI,MMSE; Emory,totalSample,immune-Monocyte; Emory,totalSample,immune-Neutrophil; Emory,totalSample,immune-CD8_T; Emory,totalSample,immune-Tfh; Emory,totalSample,immune-CD8_naive; Emory,totalSample,immune-Exhausted; Emory,totalSample,immune-InfiltrationScore; Homo_723,totalSample,immune-DC; Homo_723,totalSample,immune-Monocyte; Homo_723,totalSample,immune-Neutrophil; Homo_723,totalSample,immune-Central_memory; Homo_714,totalSample,immune-DC; Homo_714,totalSample,immune-Monocyte; Homo_714,totalSample,immune-Th2; Homo_633,totalSample,immune-Monocyte; Homo_633,totalSample,immune-DC; Homo_633,totalSample,immune-Th2; Homo_633,totalSample,immune-InfiltrationScore; MCSA,totalSample,immune-DC; MCSA,totalSample,immune-Monocyte; MCSA,totalSample,immune-Neutrophil; MCSA,totalSample,immune-CD8_T; MCSA,totalSample,immune-CD4_naive; MCSA,totalSample,immune-nTreg; MCSA,totalSample,immune-Th2; MCSA,totalSample,immune-Th17; MCSA,totalSample,immune-Tfh; MCSA,totalSample,immune-Cytotoxic; MCSA,totalSample,immune-Central_memory; MCSA,totalSample,immune-Effector_memory; MCSA,totalSample,immune-InfiltrationScore; ROSMAP,totalSample,immune-InfiltrationScore; SRP223445,totalSample,immune-
MAGED1	Homo_723,female,AD.vs.control; Homo_723,male,AD.vs.control; Homo_723,female,AD.vs.MCI; Homo_723,male,AD.vs.MCI; Homo_714,female,AD.vs.control; Homo_714,male,AD.vs.control; Homo_714,female,MCI.vs.control; Homo_714,male,MCI.vs.control; Homo_633,female,AD.vs.control; Homo_633,female,AD.vs.MCI; Homo_633,female,MCI.vs.control; Homo_633,male,MCI.vs.control; MCSA,totalSample,age; MCSA,control,age; MCSA,MCI,age; Emory,totalSample,immune-Monocyte; Emory,totalSample,immune-Macrophage; Emory,totalSample,immune-NK; Emory,totalSample,immune-Neutrophil; Emory,totalSample,immune-CD4_T; Emory,totalSample,immune-CD8_T; Emory,totalSample,immune-Tr1; Emory,totalSample,immune-nTreg; Emory,totalSample,immune-iTreg; Emory,totalSample,immune-Tfh; Emory,totalSample,immune-Exhausted; Emory,totalSample,immune-MAIT; Emory,totalSample,immune-InfiltrationScore; Homo_723,totalSample,immune-Monocyte; Homo_723,totalSample,immune-DC; Homo_723,totalSample,immune-Neutrophil; Homo_723,totalSample,immune-nTreg; Homo_723,totalSample,immune-Exhausted; Homo_723,totalSample,immune-Central_memory; Homo_723,totalSample,immune-CD4_naive; Homo_723,totalSample,immune-InfiltrationScore; Homo_714,totalSample,immune-Monocyte; Homo_714,totalSample,immune-DC; Homo_714,totalSample,immune-InfiltrationScore; Homo_714,totalSample,immune-Neutrophil; Homo_714,totalSample,immune-nTreg; Homo_714,totalSample,immune-CD4_naive; Homo_714,totalSample,immune-Th1; Homo_633,totalSample,immune-Monocyte; Homo_633,totalSample,immune-InfiltrationScore; Homo_633,totalSample,immune-nTreg; Homo_633,totalSample,immune-DC; Homo_633,totalSample,immune-Macrophage; Homo_633,totalSample,immune-Neutrophil; MCSA,totalSample,immune-DC; MCSA,totalSample,immune-Monocyte; MCSA,totalSample,immune-Neutrophil; MCSA,totalSample,immune-CD4_T; MCSA,totalSample,immune-CD8_T; MCSA,totalSample,immune-CD4_naive; MCSA,totalSample,immune-Tr1; MCSA,totalSample,immune-nTreg; MCSA,totalSample,immune-Th17; MCSA,totalSample,immune-Tfh; MCSA,totalSample,immune-Cytotoxic; MCSA,totalSample,immune-Exhausted; MCSA,totalSample,immune-Central_memory; MCSA,totalSample,immune-Effector_memory; MCSA,totalSample,immune-InfiltrationScore; ROSMAP,totalSample,immune-Cytotoxic; SRP223445,totalSample,immune-Neutrophil; [illegible]
TCEAL2	Homo_723,female,AD.vs.control; Homo_723,male,AD.vs.control; Homo_723,female,AD.vs.MCI; Homo_723,male,AD.vs.MCI; Homo_723,female,MCI.vs.control; Homo_723,male,MCI.vs.control; Homo_714,female,AD.vs.control; Homo_714,male,AD.vs.control; Homo_714,female,AD.vs.MCI; Homo_714,male,AD.vs.MCI; Homo_714,female,MCI.vs.control; Homo_714,male,MCI.vs.control; Homo_633,female,AD.vs.control; Homo_633,female,AD.vs.MCI; ACOM,totalSample,age; Emory,totalSample,immune-CD4_T; Emory,totalSample,immune-Tr1; Emory,totalSample,immune-iTreg; Emory,totalSample,immune-Th17; Emory,totalSample,immune-Tfh; Homo_723,totalSample,immune-Th17; Homo_723,totalSample,immune-CD4_naive; Homo_723,totalSample,immune-nTreg; Homo_723,totalSample,immune-Exhausted; Homo_723,totalSample,immune-CD4_T; Homo_723,totalSample,immune-Tfh; Homo_723,totalSample,immune-Central_memory; Homo_723,totalSample,immune-Th2; Homo_723,totalSample,immune-Cytotoxic; Homo_714,totalSample,immune-CD4_naive; Homo_714,totalSample,immune-Th17; Homo_714,totalSample,immune-Central_memory; Homo_714,totalSample,immune-Tfh; Homo_714,totalSample,immune-CD4_T; Homo_714,totalSample,immune-nTreg; Homo_633,totalSample,immune-Tfh; Homo_633,totalSample,immune-Th2; Homo_633,totalSample,immune-Central_memory; Homo_633,totalSample,immune-CD4_T; Homo_633,totalSample,immune-CD4_naive; Homo_633,totalSample,immune-Tr1; Homo_633,totalSample,immune-nTreg; Homo_633,totalSample,immune-Effector_memory; Homo_633,totalSample,immune-iTreg; Homo_633,totalSample,immune-Th17; MCSA,totalSample,immune-CD4_T; MCSA,totalSample,immune-CD4_naive; MCSA,totalSample,immune-Tr1; MCSA,totalSample,immune-nTreg; MCSA,totalSample,immune-iTreg; MCSA,totalSample,immune-Tfh; MCSA,totalSample,immune-CD8_naive; MCSA,totalSample,immune-Central_memory; SRP223445,totalSample,immune-CD4_naive; SRP223445,totalSample,immune-Exhausted
ARHGAP11A	Homo_723,female,AD.vs.control; Homo_723,male,AD.vs.control; Homo_723,female,AD.vs.MCI; Homo_723,male,AD.vs.MCI; Homo_723,female,MCI.vs.control; Homo_723,male,MCI.vs.control; Homo_714,female,AD.vs.control; Homo_714,male,AD.vs.control; Homo_714,female,AD.vs.MCI; Homo_714,male,AD.vs.MCI; Homo_714,female,MCI.vs.control; Homo_714,male,MCI.vs.control; Homo_633,female,AD.vs.control; Homo_633,female,AD.vs.MCI; ROSMAP,male,AD.vs.control; MCSA,MCI,age; Emory,totalSample,immune-Tr1; Emory,totalSample,immune-iTreg; Emory,totalSample,immune-InfiltrationScore; Homo_723,totalSample,immune-Exhausted; Homo_723,totalSample,immune-Th17; Homo_723,totalSample,immune-CD4_naive; Homo_723,totalSample,immune-CD8_T; Homo_723,totalSample,immune-Cytotoxic; Homo_723,totalSample,immune-InfiltrationScore; Homo_723,totalSample,immune-CD4_T; Homo_723,totalSample,immune-Tfh; Homo_723,totalSample,immune-Monocyte; Homo_723,totalSample,immune-Neutrophil; Homo_723,totalSample,immune-NK; Homo_714,totalSample,immune-Monocyte; Homo_714,totalSample,immune-InfiltrationScore; Homo_714,totalSample,immune-CD4_naive; Homo_714,totalSample,immune-Exhausted; Homo_714,totalSample,immune-Th17; Homo_633,totalSample,immune-Tfh; Homo_633,totalSample,immune-InfiltrationScore; Homo_633,totalSample,immune-Effector_memory; Homo_633,totalSample,immune-Monocyte; Homo_633,totalSample,immune-Th2; Homo_633,totalSample,immune-CD4_naive; Homo_633,totalSample,immune-CD4_T; Homo_633,totalSample,immune-Central_memory; Homo_633,totalSample,immune-Bcell; MCSA,totalSample,immune-DC; MCSA,totalSample,immune-Monocyte; MCSA,totalSample,immune-Macrophage; MCSA,totalSample,immune-CD4_T; MCSA,totalSample,immune-CD4_naive; MCSA,totalSample,immune-Tr1; MCSA,totalSample,immune-nTreg; MCSA,totalSample,immune-iTreg; MCSA,totalSample,immune-Tfh; MCSA,totalSample,immune-CD8_naive; MCSA,totalSample,immune-Cytotoxic; MCSA,totalSample,immune-Exhausted; MCSA,totalSample,immune-MAIT; MCSA,totalSample,immune-Central_memory; ROSMAP,totalSample,immune-Effector_memory; ROSMAP,totalSample,immune-InfiltrationScore; SRP223445,totalSample,immune-NK; SRP223445,totalSample,immune-Neutrophil; SRP223445,totalSample,immune-InfiltrationScore
NUDT18	Homo_723,female,AD.vs.control; Homo_723,male,AD.vs.control; Homo_723,female,AD.vs.MCI; Homo_723,male,AD.vs.MCI; Homo_714,female,AD.vs.control; Homo_714,male,AD.vs.control; Homo_714,female,AD.vs.MCI; Homo_714,male,AD.vs.MCI; Homo_633,female,AD.vs.control; Homo_633,female,AD.vs.MCI; ROSMAP,female,AD.vs.control; MCSA,MCI,age; Emory,totalSample,immune-Macrophage; Emory,totalSample,immune-NK; Emory,totalSample,immune-Neutrophil; Emory,totalSample,immune-Tr1; Emory,totalSample,immune-iTreg; Emory,totalSample,immune-Effector_memory; Emory,totalSample,immune-InfiltrationScore; Homo_723,totalSample,immune-Cytotoxic; Homo_723,totalSample,immune-InfiltrationScore; Homo_723,totalSample,immune-CD8_T; Homo_723,totalSample,immune-Effector_memory; Homo_723,totalSample,immune-DC; Homo_723,totalSample,immune-Exhausted; Homo_723,totalSample,immune-Th17; Homo_723,totalSample,immune-Th1; Homo_714,totalSample,immune-DC; Homo_714,totalSample,immune-InfiltrationScore; Homo_714,totalSample,immune-CD8_T; Homo_714,totalSample,immune-Effector_memory; Homo_714,totalSample,immune-Cytotoxic; Homo_714,totalSample,immune-Neutrophil; Homo_633,totalSample,immune-DC; Homo_633,totalSample,immune-InfiltrationScore; Homo_633,totalSample,immune-CD8_T; Homo_633,totalSample,immune-Neutrophil; Homo_633,totalSample,immune-Cytotoxic; Homo_633,totalSample,immune-Effector_memory; Homo_633,totalSample,immune-NK; MCSA,totalSample,immune-DC; MCSA,totalSample,immune-Monocyte; MCSA,totalSample,immune-Neutrophil; MCSA,totalSample,immune-CD8_T; MCSA,totalSample,immune-NKT; MCSA,totalSample,immune-iTreg; MCSA,totalSample,immune-Th2; MCSA,totalSample,immune-Cytotoxic; MCSA,totalSample,immune-Effector_memory; MCSA,totalSample,immune-InfiltrationScore; SRP223445,totalSample,immune-Neutrophil; SRP223445,totalSample,immune-CD4_naive; SRP223445,totalSample,immune-InfiltrationScore
FBP1	Homo_723,female,AD.vs.control; Homo_723,male,AD.vs.control; Homo_723,female,AD.vs.MCI; Homo_723,male,AD.vs.MCI; Homo_714,female,AD.vs.control; Homo_714,male,AD.vs.control; Homo_714,female,AD.vs.MCI; Homo_714,male,AD.vs.MCI; Homo_633,female,AD.vs.control; Homo_633,female,AD.vs.MCI; Emory,MCI,age; MCSA,MCI,age; Emory,totalSample,immune-DC; Emory,totalSample,immune-Macrophage; Homo_723,totalSample,immune-InfiltrationScore; Homo_723,totalSample,immune-Exhausted; Homo_723,totalSample,immune-Th17; Homo_723,totalSample,immune-Cytotoxic; Homo_723,totalSample,immune-DC; Homo_723,totalSample,immune-CD8_T; Homo_714,totalSample,immune-DC; Homo_714,totalSample,immune-InfiltrationScore; Homo_633,totalSample,immune-DC; Homo_633,totalSample,immune-InfiltrationScore; Homo_633,totalSample,immune-NK; MCSA,totalSample,immune-DC; MCSA,totalSample,immune-Monocyte; MCSA,totalSample,immune-Macrophage; MCSA,totalSample,immune-Gamma_delta; MCSA,totalSample,immune-iTreg; MCSA,totalSample,immune-Th2; MCSA,totalSample,immune-MAIT; MCSA,totalSample,immune-InfiltrationScore; SRP223445,totalSample,immune-CD4_naive; SRP223445,totalSample,immune-InfiltrationScore

FAM171A1	Homo_723,female,AD.vs.control; Homo_723,male,AD.vs.control; Homo_723,female,AD.vs.MCI; Homo_723,male,AD.vs.MCI; Homo_714,female,AD.vs.control; Homo_714,male,AD.vs.control; Homo_714,male,AD.vs.MCI; Homo_633,female,AD.vs.control; Homo_633,female,AD.vs.MCI; MCSA,totalSample,age; MCSA,control,age; MCSA,MCI,age; Emory,totalSample,immune-Monocyte; Emory,totalSample,immune-Macrophage; Emory,totalSample,immune-Neutrophil; Emory,totalSample,immune-CD4_T; Emory,totalSample,immune-CD8_T; Emory,totalSample,immune-CD4_naive; Emory,totalSample,immune-Tr1; Emory,totalSample,immune-nTreg; Emory,totalSample,immune-iTreg; Emory,totalSample,immune-Th2; Emory,totalSample,immune-Th17; Emory,totalSample,immune-Tfh; Emory,totalSample,immune-CD8_naive; Emory,totalSample,immune-Cytotoxic; Emory,totalSample,immune-MAIT; Emory,totalSample,immune-Central_memory; Emory,totalSample,immune-InfiltrationScore; Homo_723,totalSample,immune-nTreg; Homo_723,totalSample,immune-Central_memory; Homo_723,totalSample,immune-Monocyte; Homo_723,totalSample,immune-CD4_naive; Homo_723,totalSample,immune-CD4_T; Homo_723,totalSample,immune-Th1; Homo_723,totalSample,immune-DC; Homo_723,totalSample,immune-Neutrophil; Homo_723,totalSample,immune-Tfh; Homo_723,totalSample,immune-Tr1; Homo_723,totalSample,immune-iTreg; Homo_714,totalSample,immune-nTreg; Homo_714,totalSample,immune-Th1; Homo_714,totalSample,immune-CD4_naive; Homo_714,totalSample,immune-Monocyte; Homo_714,totalSample,immune-iTreg; Homo_714,totalSample,immune-Tfh; Homo_714,totalSample,immune-InfiltrationScore; Homo_714,totalSample,immune-CD4_T; Homo_714,totalSample,immune-Tr1; Homo_633,totalSample,immune-nTreg; Homo_633,totalSample,immune-Tr1; Homo_633,totalSample,immune-Tfh; Homo_633,totalSample,immune-Monocyte; Homo_633,totalSample,immune-iTreg; Homo_633,totalSample,immune-CD4_T; Homo_633,totalSample,immune-CD4_naive; Homo_633,totalSample,immune-Th1; Homo_633,totalSample,immune-Central_memory; Homo_633,totalSample,immune-Macrophage; MCSA,totalSample,immune-Monocyte; MCSA,totalSample,immune-Macrophage; MCSA,totalSample,immune-Neutrophil; MCSA,totalSample,immune-CD4_T; MCSA,totalSample,immune-CD8_T; MCSA,totalSample,immune-CD4_naive; MCSA,totalSample,immune-Tr1; MCSA,totalSample,immune-nTreg; MCSA,totalSample,immune-iTreg; MCSA,totalSample,immune-Th17; MCSA,totalSample,immune-Tfh; MCSA,totalSample,immune-CD8_naive; MCSA,totalSample,immune-Central_memory; MCSA,totalSample,immune-Effector_memory; MCSA,totalSample,immune-InfiltrationScore; ROSMAP,totalSample,immune-InfiltrationScore; SRP223445,totalSample,immune-[illegible]
ASB13	Homo_723,female,AD.vs.control; Homo_723,male,AD.vs.control; Homo_723,female,AD.vs.MCI; Homo_723,male,AD.vs.MCI; Homo_714,female,AD.vs.control; Homo_714,male,AD.vs.control; Homo_714,female,AD.vs.MCI; Homo_714,male,AD.vs.MCI; Homo_633,female,AD.vs.control; Homo_633,female,AD.vs.MCI; Emory,MCI,age; MCSA,totalSample,age; MCSA,control,age; ROSMAP,MCI,sample.age; Emory,totalSample,immune-CD4_T; Emory,totalSample,immune-Tr1; Emory,totalSample,immune-iTreg; Emory,totalSample,immune-MAIT; Emory,totalSample,immune-Effector_memory; Emory,totalSample,immune-InfiltrationScore; Homo_723,totalSample,immune-Effector_memory; Homo_723,totalSample,immune-Tfh; Homo_723,totalSample,immune-Th2; Homo_723,totalSample,immune-iTreg; Homo_723,totalSample,immune-InfiltrationScore; Homo_723,totalSample,immune-CD4_T; Homo_723,totalSample,immune-nTreg; Homo_723,totalSample,immune-CD4_naive; Homo_723,totalSample,immune-Tr1; Homo_723,totalSample,immune-Central_memory; Homo_723,totalSample,immune-CD8_T; Homo_723,totalSample,immune-Bcell; Homo_714,totalSample,immune-Effector_memory; Homo_714,totalSample,immune-Tfh; Homo_714,totalSample,immune-Th2; Homo_714,totalSample,immune-InfiltrationScore; Homo_714,totalSample,immune-Central_memory; Homo_714,totalSample,immune-Tr1; Homo_714,totalSample,immune-nTreg; Homo_714,totalSample,immune-CD4_T; Homo_714,totalSample,immune-iTreg; Homo_633,totalSample,immune-Effector_memory; Homo_633,totalSample,immune-InfiltrationScore; Homo_633,totalSample,immune-Tfh; Homo_633,totalSample,immune-Central_memory; Homo_633,totalSample,immune-nTreg; Homo_633,totalSample,immune-Exhausted; Homo_633,totalSample,immune-CD4_T; Homo_633,totalSample,immune-Tr1; Homo_633,totalSample,immune-CD4_naive; Homo_633,totalSample,immune-Th2; Homo_633,totalSample,immune-iTreg; Homo_633,totalSample,immune-Gamma_delta; MCSA,totalSample,immune-DC; MCSA,totalSample,immune-Monocyte; MCSA,totalSample,immune-Neutrophil; MCSA,totalSample,immune-CD4_T; MCSA,totalSample,immune-CD8_T; MCSA,totalSample,immune-NKT; MCSA,totalSample,immune-Gamma_delta; MCSA,totalSample,immune-CD4_naive; MCSA,totalSample,immune-Tr1; MCSA,totalSample,immune-nTreg; MCSA,totalSample,immune-Th2; MCSA,totalSample,immune-Tfh; MCSA,totalSample,immune-Central_memory; MCSA,totalSample,immune-Effector_memory; MCSA,totalSample,immune-InfiltrationScore; ROSMAP,totalSample,immune-Cytotoxic; ROSMAP,totalSample,immune-Effector_memory; SRP223445,totalSample,immune-CD4_naive; SRP223445,totalSample,immune-InfiltrationScore
RORB	Homo_723,female,AD.vs.control; Homo_723,male,AD.vs.control; Homo_723,female,AD.vs.MCI; Homo_723,male,AD.vs.MCI; Homo_714,female,AD.vs.control; ROSMAP,control,male.vs.female; Emory,totalSample,immune-MAIT; Homo_723,totalSample,immune-InfiltrationScore; Homo_714,totalSample,immune-InfiltrationScore; Homo_633,totalSample,immune-InfiltrationScore; MCSA,totalSample,immune-MAIT; ROSMAP,totalSample,immune-InfiltrationScore; SRP223445,totalSample,immune-CD4_naive
PLPP3	Homo_723,female,AD.vs.control; Homo_723,male,AD.vs.control; Homo_723,female,AD.vs.MCI; Homo_723,male,AD.vs.MCI; Homo_723,female,MCI.vs.control; Homo_723,male,MCI.vs.control; Homo_714,female,AD.vs.control; Homo_714,male,AD.vs.control; Homo_714,female,AD.vs.MCI; Homo_714,male,AD.vs.MCI; Homo_714,female,MCI.vs.control; Homo_714,male,MCI.vs.control; Homo_633,female,AD.vs.control; Homo_633,female,AD.vs.MCI; Homo_633,female,MCI.vs.control; Emory,MCI,age; MCSA,MCI,age; ROSMAP,MCI,last.visit.age; Emory,totalSample,immune-Neutrophil; Emory,totalSample,immune-CD4_T; Emory,totalSample,immune-CD8_T; Emory,totalSample,immune-Gamma_delta; Emory,totalSample,immune-Tfh; Emory,totalSample,immune-CD8_naive; Emory,totalSample,immune-Exhausted; Emory,totalSample,immune-MAIT; Homo_723,totalSample,immune-CD8_T; Homo_723,totalSample,immune-Cytotoxic; Homo_723,totalSample,immune-Th17; Homo_723,totalSample,immune-DC; Homo_723,totalSample,immune-Macrophage; Homo_714,totalSample,immune-CD8_T; Homo_714,totalSample,immune-Exhausted; Homo_714,totalSample,immune-Th2; Homo_633,totalSample,immune-CD8_T; MCSA,totalSample,immune-DC; MCSA,totalSample,immune-Neutrophil; MCSA,totalSample,immune-CD8_T; MCSA,totalSample,immune-Gamma_delta; MCSA,totalSample,immune-Tr1; MCSA,totalSample,immune-iTreg; MCSA,totalSample,immune-Th17; MCSA,totalSample,immune-Cytotoxic; MCSA,totalSample,immune-Exhausted; ROSMAP,totalSample,immune-Central_memory; ROSMAP,totalSample,immune-Effector_memory; SRP223445,totalSample,immune-CD4_naive; SRP223445,totalSample,immune-InfiltrationScore
CRELD2	Homo_723,female,AD.vs.control; Homo_723,male,AD.vs.control; Homo_723,female,AD.vs.MCI; Homo_723,male,AD.vs.MCI; Homo_714,female,AD.vs.control; Homo_714,male,AD.vs.control; Homo_714,female,AD.vs.MCI; Homo_714,male,AD.vs.MCI; Homo_633,female,AD.vs.control; Homo_633,female,AD.vs.MCI; ROSMAP,female,AD.vs.control; Emory,totalSample,immune-Macrophage; Emory,totalSample,immune-NK; Emory,totalSample,immune-Neutrophil; Emory,totalSample,immune-CD4_T; Emory,totalSample,immune-CD8_T; Emory,totalSample,immune-Tr1; Emory,totalSample,immune-iTreg; Emory,totalSample,immune-Exhausted; Emory,totalSample,immune-Effector_memory; Emory,totalSample,immune-InfiltrationScore; Homo_723,totalSample,immune-CD8_T; Homo_723,totalSample,immune-InfiltrationScore; Homo_723,totalSample,immune-Cytotoxic; Homo_723,totalSample,immune-Exhausted; Homo_723,totalSample,immune-Effector_memory; Homo_723,totalSample,immune-MAIT; Homo_723,totalSample,immune-Monocyte; Homo_723,totalSample,immune-Th17; Homo_723,totalSample,immune-DC; Homo_723,totalSample,immune-Tfh; Homo_723,totalSample,immune-iTreg; Homo_723,totalSample,immune-Macrophage; Homo_723,totalSample,immune-nTreg; Homo_723,totalSample,immune-Bcell; Homo_714,totalSample,immune-InfiltrationScore; Homo_714,totalSample,immune-Effector_memory; Homo_714,totalSample,immune-CD8_T; Homo_714,totalSample,immune-DC; Homo_714,totalSample,immune-Cytotoxic; Homo_714,totalSample,immune-Monocyte; Homo_714,totalSample,immune-Th17; Homo_714,totalSample,immune-Macrophage; Homo_714,totalSample,immune-nTreg; Homo_714,totalSample,immune-Tfh; Homo_714,totalSample,immune-MAIT; Homo_633,totalSample,immune-InfiltrationScore; Homo_633,totalSample,immune-DC; Homo_633,totalSample,immune-Effector_memory; Homo_633,totalSample,immune-Neutrophil; Homo_633,totalSample,immune-Monocyte; Homo_633,totalSample,immune-nTreg; Homo_633,totalSample,immune-NK; Homo_633,totalSample,immune-CD8_T; Homo_633,totalSample,immune-Cytotoxic; MCSA,totalSample,immune-DC; MCSA,totalSample,immune-Monocyte; MCSA,totalSample,immune-Neutrophil; MCSA,totalSample,immune-CD8_T; MCSA,totalSample,immune-NKT; MCSA,totalSample,immune-Th2; MCSA,totalSample,immune-Cytotoxic; MCSA,totalSample,immune-Central_memory; MCSA,totalSample,immune-Effector_memory; [illegible]
TSPAN13	Homo_723,female,AD.vs.control; Homo_723,male,AD.vs.control; Homo_723,female,AD.vs.MCI; Homo_723,male,AD.vs.MCI; Homo_714,female,AD.vs.control; Homo_714,male,AD.vs.control; Homo_714,female,AD.vs.MCI; Homo_714,male,AD.vs.MCI; Homo_633,female,AD.vs.control; Homo_633,female,AD.vs.MCI; SRP223445,female,AD.vs.control; ROSMAP,MCI,sample.age; Emory,totalSample,immune-DC; Emory,totalSample,immune-B_cell; Emory,totalSample,immune-Macrophage; Emory,totalSample,immune-Neutrophil; Emory,totalSample,immune-CD4_T; Emory,totalSample,immune-Tr1; Emory,totalSample,immune-iTreg; Emory,totalSample,immune-Tfh; Emory,totalSample,immune-CD8_naive; Homo_723,totalSample,immune-CD4_naive; Homo_723,totalSample,immune-Th1; Homo_723,totalSample,immune-CD4_T; Homo_723,totalSample,immune-Cytotoxic; Homo_723,totalSample,immune-Central_memory; Homo_723,totalSample,immune-Th17; Homo_723,totalSample,immune-Tr1; Homo_723,totalSample,immune-Tfh; Homo_723,totalSample,immune-Exhausted; Homo_723,totalSample,immune-nTreg; Homo_723,totalSample,immune-iTreg; Homo_723,totalSample,immune-Neutrophil; Homo_714,totalSample,immune-Th1; Homo_714,totalSample,immune-NKT; Homo_714,totalSample,immune-CD4_naive; Homo_714,totalSample,immune-Tfh; Homo_714,totalSample,immune-CD4_T; Homo_714,totalSample,immune-Exhausted; Homo_714,totalSample,immune-Tr1; Homo_714,totalSample,immune-Central_memory; Homo_714,totalSample,immune-Th17; Homo_714,totalSample,immune-Cytotoxic; Homo_633,totalSample,immune-Tfh; Homo_633,totalSample,immune-CD4_T; Homo_633,totalSample,immune-NKT; Homo_633,totalSample,immune-CD4_naive; Homo_633,totalSample,immune-Tr1; Homo_633,totalSample,immune-Central_memory; Homo_633,totalSample,immune-iTreg; Homo_633,totalSample,immune-Th2; Homo_633,totalSample,immune-Gamma_delta; Homo_633,totalSample,immune-Th1; Homo_633,totalSample,immune-InfiltrationScore; Homo_633,totalSample,immune-nTreg; Homo_633,totalSample,immune-Effector_memory; MCSA,totalSample,immune-DC; MCSA,totalSample,immune-Bcell; MCSA,totalSample,immune-Monocyte; MCSA,totalSample,immune-Macrophage; MCSA,totalSample,immune-CD4_T; MCSA,totalSample,immune-CD8_T; MCSA,totalSample,immune-CD4_naive; MCSA,totalSample,immune-Tr1; MCSA,totalSample,immune-nTreg; MCSA,totalSample,immune-iTreg; MCSA,totalSample,immune-Th2; MCSA,totalSample,immune-Tfh; MCSA,totalSample,immune-CD8_naive; MCSA,totalSample,immune-Cytotoxic; MCSA,totalSample,immune-Exhausted; MCSA,totalSample,immune-Central_memory; ROSMAP,totalSample,immune-InfiltrationScore
DDX47	Homo_723,female,AD.vs.control; Homo_723,female,AD.vs.MCI; Homo_723,female,MCI.vs.control; Homo_723,male,MCI.vs.control; Homo_723,AD,male.vs.female; Homo_723,control,male.vs.female; Homo_714,female,AD.vs.control; Homo_714,male,AD.vs.control; Homo_714,female,AD.vs.MCI; Homo_714,male,AD.vs.MCI; Homo_714,female,MCI.vs.control; Homo_714,male,MCI.vs.control; Homo_714,control,male.vs.female; Homo_633,female,AD.vs.control; Homo_633,female,AD.vs.MCI; Homo_633,female,MCI.vs.control; Homo_633,male,MCI.vs.control; Homo_633,MCI,male.vs.female; MCSA,MCI,age; ROSMAP,MCI,last.visit.age; Emory,totalSample,immune-Macrophage; Emory,totalSample,immune-NK; Emory,totalSample,immune-Neutrophil; Emory,totalSample,immune-CD4_T; Emory,totalSample,immune-CD8_T; Emory,totalSample,immune-Tr1; Emory,totalSample,immune-iTreg; Emory,totalSample,immune-Tfh; Emory,totalSample,immune-Exhausted; Emory,totalSample,immune-MAIT; Emory,totalSample,immune-Central_memory; Emory,totalSample,immune-Effector_memory; Emory,totalSample,immune-InfiltrationScore; Homo_723,totalSample,immune-CD4_naive; Homo_723,totalSample,immune-CD4_T; Homo_723,totalSample,immune-Central_memory; Homo_723,totalSample,immune-Th1; Homo_723,totalSample,immune-Tfh; Homo_723,totalSample,immune-Tr1; Homo_723,totalSample,immune-iTreg; Homo_723,totalSample,immune-nTreg; Homo_723,totalSample,immune-Neutrophil; Homo_723,totalSample,immune-Monocyte; Homo_723,totalSample,immune-InfiltrationScore; Homo_723,totalSample,immune-Exhausted; Homo_723,totalSample,immune-NKT; Homo_714,totalSample,immune-CD4_naive; Homo_714,totalSample,immune-Th1; Homo_714,totalSample,immune-iTreg; Homo_714,totalSample,immune-CD4_T; Homo_714,totalSample,immune-NKT; Homo_714,totalSample,immune-Tfh; Homo_714,totalSample,immune-Tr1; Homo_714,totalSample,immune-Neutrophil; Homo_714,totalSample,immune-nTreg; Homo_714,totalSample,immune-Central_memory; Homo_714,totalSample,immune-Monocyte; Homo_714,totalSample,immune-InfiltrationScore; Homo_633,totalSample,immune-Th1; Homo_633,totalSample,immune-CD4_naive; Homo_633,totalSample,immune-iTreg; Homo_633,totalSample,immune-NKT; Homo_633,totalSample,immune-Tfh; Homo_633,totalSample,immune-CD4_T; Homo_633,totalSample,immune-Tr1; Homo_633,totalSample,immune-Monocyte; Homo_633,totalSample,immune-nTreg; Homo_633,totalSample,immune-InfiltrationScore; Homo_633,totalSample,immune-Central_memory; Homo_633,totalSample,immune-Effector_memory; MCSA,totalSample,immune-DC; MCSA,totalSample,immune-Macrophage; MCSA,totalSample,immune-Neutrophil; MCSA,totalSample,immune-CD4_T; MCSA,totalSample,immune-CD4_naive; MCSA,totalSample,immune-Tr1; MCSA,totalSample,immune-nTreg; MCSA,totalSample,immune-iTreg; MCSA,totalSample,immune-Th17; MCSA,totalSample,immune-Tfh; MCSA,totalSample,immune-CD8_naive; MCSA,totalSample,immune-Cytotoxic; MCSA,totalSample,immune-Exhausted; MCSA,totalSample,immune-MAIT; MCSA,totalSample,immune-[illegible]
MFSD14B	Homo_723,female,AD.vs.control; Homo_723,male,AD.vs.control; Homo_723,female,AD.vs.MCI; Homo_723,male,AD.vs.MCI; Homo_714,female,AD.vs.control; Homo_714,male,AD.vs.control; Homo_714,female,AD.vs.MCI; Homo_714,male,AD.vs.MCI; Homo_633,female,AD.vs.control; Homo_633,female,AD.vs.MCI; Emory,MCI,age; MCSA,MCI,age; Emory,totalSample,immune-Monocyte; Emory,totalSample,immune-Gamma_delta; Emory,totalSample,immune-nTreg; Emory,totalSample,immune-Tfh; Emory,totalSample,immune-CD8_naive; Homo_723,totalSample,immune-iTreg; Homo_723,totalSample,immune-Gamma_delta; Homo_723,totalSample,immune-InfiltrationScore; Homo_723,totalSample,immune-Tfh; Homo_723,totalSample,immune-Th2; Homo_723,totalSample,immune-DC; Homo_723,totalSample,immune-Tr1; Homo_723,totalSample,immune-Neutrophil; Homo_723,totalSample,immune-Bcell; Homo_723,totalSample,immune-CD4_T; Homo_723,totalSample,immune-Effector_memory; Homo_723,totalSample,immune-NKT; Homo_723,totalSample,immune-Monocyte; Homo_723,totalSample,immune-CD4_naive; Homo_714,totalSample,immune-Tfh; Homo_714,totalSample,immune-Th2; Homo_714,totalSample,immune-Gamma_delta; Homo_714,totalSample,immune-Tr1; Homo_714,totalSample,immune-Effector_memory; Homo_714,totalSample,immune-InfiltrationScore; Homo_714,totalSample,immune-CD4_T; Homo_714,totalSample,immune-iTreg; Homo_714,totalSample,immune-Monocyte; Homo_633,totalSample,immune-InfiltrationScore; Homo_633,totalSample,immune-NKT; Homo_633,totalSample,immune-Tfh; Homo_633,totalSample,immune-Exhausted; Homo_633,totalSample,immune-Gamma_delta; Homo_633,totalSample,immune-Monocyte; Homo_633,totalSample,immune-Th2; Homo_633,totalSample,immune-CD4_naive; Homo_633,totalSample,immune-CD4_T; Homo_633,totalSample,immune-Effector_memory; Homo_633,totalSample,immune-Tr1; Homo_633,totalSample,immune-CD8_T; MCSA,totalSample,immune-DC; MCSA,totalSample,immune-Monocyte; MCSA,totalSample,immune-Neutrophil; MCSA,totalSample,immune-CD4_T; MCSA,totalSample,immune-CD8_T; MCSA,totalSample,immune-CD4_naive; MCSA,totalSample,immune-Tr1; MCSA,totalSample,immune-nTreg; MCSA,totalSample,immune-iTreg; MCSA,totalSample,immune-Th17; MCSA,totalSample,immune-CD8_naive; MCSA,totalSample,immune-Cytotoxic; MCSA,totalSample,immune-Exhausted; ROSMAP,totalSample,immune-Effector_memory; SRP223445,totalSample,immune-CD4_naive; ROSMAP,female-excludeAge90+,survival; ROSMAP,female-includeAge90+,survival; ROSMAP,male-includeAge90+,survival; ROSMAP,male and female-excludeAge90+,survival
CPS1	Homo_723,female,AD.vs.control; Homo_723,male,AD.vs.control; Homo_723,female,AD.vs.MCI; Homo_723,male,AD.vs.MCI; Homo_723,female,MCI.vs.control; Homo_723,male,MCI.vs.control; Homo_714,female,AD.vs.control; Homo_714,male,AD.vs.control; Homo_714,female,AD.vs.MCI; Homo_714,male,AD.vs.MCI; Homo_633,female,AD.vs.control; Homo_633,female,AD.vs.MCI; Homo_723,totalSample,immune-CD4_naive; Homo_723,totalSample,immune-CD4_T; Homo_723,totalSample,immune-Th17; Homo_723,totalSample,immune-Exhausted; Homo_723,totalSample,immune-Tfh; Homo_723,totalSample,immune-Cytotoxic; Homo_714,totalSample,immune-CD4_naive; Homo_714,totalSample,immune-InfiltrationScore; Homo_633,totalSample,immune-Tfh; Homo_633,totalSample,immune-CD4_T; Homo_633,totalSample,immune-CD4_naive; Homo_633,totalSample,immune-Effector_memory; Homo_633,totalSample,immune-InfiltrationScore; Homo_633,totalSample,immune-Th2; Homo_633,totalSample,immune-Central_memory; MCSA,totalSample,immune-DC; MCSA,totalSample,immune-Macrophage; MCSA,totalSample,immune-CD4_T; MCSA,totalSample,immune-CD4_naive; MCSA,totalSample,immune-Tr1; MCSA,totalSample,immune-nTreg; MCSA,totalSample,immune-iTreg; MCSA,totalSample,immune-Th17; MCSA,totalSample,immune-Tfh; MCSA,totalSample,immune-CD8_naive; MCSA,totalSample,immune-Exhausted; MCSA,totalSample,immune-MAIT; MCSA,totalSample,immune-Central_memory; MCSA,totalSample,immune-Effector_memory; SRP223445,totalSample,immune-InfiltrationScore; ROSMAP,male and female-excludeAge90+,survival; ROSMAP,male and female-includeAge90+,survival
TBC1D32	Homo_723,female,AD.vs.control; Homo_723,male,AD.vs.control; Homo_723,female,AD.vs.MCI; Homo_723,male,AD.vs.MCI; Homo_714,female,AD.vs.control; Homo_714,male,AD.vs.control; Homo_714,female,AD.vs.MCI; Homo_714,male,AD.vs.MCI; Homo_633,female,AD.vs.control; Homo_633,female,AD.vs.MCI; MCSA,MCI,age; Emory,totalSample,immune-Macrophage; Emory,totalSample,immune-Neutrophil; Emory,totalSample,immune-CD4_T; Emory,totalSample,immune-CD8_T; Emory,totalSample,immune-Tr1; Emory,totalSample,immune-iTreg; Emory,totalSample,immune-Tfh; Emory,totalSample,immune-CD8_naive; Emory,totalSample,immune-MAIT; Emory,totalSample,immune-Central_memory; Emory,totalSample,immune-InfiltrationScore; Homo_723,totalSample,immune-CD4_naive; Homo_723,totalSample,immune-Central_memory; Homo_723,totalSample,immune-CD4_T; Homo_723,totalSample,immune-Tr1; Homo_723,totalSample,immune-iTreg; Homo_723,totalSample,immune-Tfh; Homo_723,totalSample,immune-Th1; Homo_723,totalSample,immune-nTreg; Homo_723,totalSample,immune-Cytotoxic; Homo_723,totalSample,immune-Th2; Homo_723,totalSample,immune-NKT; Homo_723,totalSample,immune-Neutrophil; Homo_723,totalSample,immune-Th17; Homo_723,totalSample,immune-InfiltrationScore; Homo_714,totalSample,immune-CD4_naive; Homo_714,totalSample,immune-Tfh; Homo_714,totalSample,immune-CD4_T; Homo_714,totalSample,immune-Tr1; Homo_714,totalSample,immune-Central_memory; Homo_714,totalSample,immune-iTreg; Homo_714,totalSample,immune-NKT; Homo_714,totalSample,immune-Th1; Homo_714,totalSample,immune-nTreg; Homo_714,totalSample,immune-InfiltrationScore; Homo_714,totalSample,immune-Effector_memory; Homo_633,totalSample,immune-Tfh; Homo_633,totalSample,immune-CD4_naive; Homo_633,totalSample,immune-CD4_T; Homo_633,totalSample,immune-Central_memory; Homo_633,totalSample,immune-Tr1; Homo_633,totalSample,immune-NKT; Homo_633,totalSample,immune-iTreg; Homo_633,totalSample,immune-Effector_memory; Homo_633,totalSample,immune-nTreg; Homo_633,totalSample,immune-InfiltrationScore; Homo_633,totalSample,immune-Th1; Homo_633,totalSample,immune-MAIT; Homo_633,totalSample,immune-DC; Homo_633,totalSample,immune-Th2; Homo_633,totalSample,immune-Neutrophil; Homo_633,totalSample,immune-Gamma_delta; MCSA,totalSample,immune-DC; MCSA,totalSample,immune-Bcell; MCSA,totalSample,immune-Monocyte; MCSA,totalSample,immune-Macrophage; MCSA,totalSample,immune-CD4_T; MCSA,totalSample,immune-CD4_naive; MCSA,totalSample,immune-Tr1; MCSA,totalSample,immune-nTreg; MCSA,totalSample,immune-iTreg; MCSA,totalSample,immune-Th17; MCSA,totalSample,immune-Tfh; MCSA,totalSample,immune-CD8_naive; [illegible]
ELK4	Homo_723,female,AD.vs.control; Homo_723,male,AD.vs.control; Homo_723,female,AD.vs.MCI; Homo_723,male,AD.vs.MCI; Homo_723,female,MCI.vs.control; Homo_723,male,MCI.vs.control; Homo_723,AD,male.vs.female; Homo_723,MCI,male.vs.female; Homo_714,female,AD.vs.control; Homo_714,male,AD.vs.control; Homo_714,female,AD.vs.MCI; Homo_714,male,AD.vs.MCI; Homo_714,female,MCI.vs.control; Homo_714,male,MCI.vs.control; Homo_714,control,male.vs.female; Homo_714,MCI,male.vs.female; Homo_633,female,AD.vs.control; Homo_633,female,AD.vs.MCI; Homo_633,female,MCI.vs.control; Homo_633,male,MCI.vs.control; Homo_633,control,male.vs.female; Homo_633,MCI,male.vs.female; ROSMAP,female,AD.vs.control; ROSMAP,female,AD.vs.MCI; MCSA,MCI,age; ROSMAP,MCI,MMSE; Emory,totalSample,immune-Macrophage; Emory,totalSample,immune-Neutrophil; Emory,totalSample,immune-CD4_T; Emory,totalSample,immune-CD8_T; Emory,totalSample,immune-Tr1; Emory,totalSample,immune-iTreg; Emory,totalSample,immune-Tfh; Emory,totalSample,immune-CD8_naive; Emory,totalSample,immune-Exhausted; Emory,totalSample,immune-MAIT; Emory,totalSample,immune-Central_memory; Emory,totalSample,immune-Effector_memory; Emory,totalSample,immune-InfiltrationScore; Homo_723,totalSample,immune-CD4_naive; Homo_723,totalSample,immune-Central_memory; Homo_723,totalSample,immune-Th1; Homo_723,totalSample,immune-CD4_T; Homo_723,totalSample,immune-iTreg; Homo_723,totalSample,immune-Tr1; Homo_723,totalSample,immune-Tfh; Homo_723,totalSample,immune-Monocyte; Homo_723,totalSample,immune-NKT; Homo_714,totalSample,immune-Th1; Homo_714,totalSample,immune-CD4_naive; Homo_714,totalSample,immune-NKT; Homo_714,totalSample,immune-iTreg; Homo_714,totalSample,immune-CD4_T; Homo_714,totalSample,immune-Tfh; Homo_714,totalSample,immune-Tr1; Homo_714,totalSample,immune-Central_memory; Homo_633,totalSample,immune-Th1; Homo_633,totalSample,immune-NKT; Homo_633,totalSample,immune-CD4_naive; Homo_633,totalSample,immune-iTreg; Homo_633,totalSample,immune-Tfh; Homo_633,totalSample,immune-CD4_T; Homo_633,totalSample,immune-Tr1; Homo_633,totalSample,immune-Monocyte; Homo_633,totalSample,immune-Central_memory; MCSA,totalSample,immune-DC; MCSA,totalSample,immune-Bcell; MCSA,totalSample,immune-Macrophage; MCSA,totalSample,immune-CD4_T; MCSA,totalSample,immune-CD8_T; MCSA,totalSample,immune-CD4_naive; MCSA,totalSample,immune-Tr1; MCSA,totalSample,immune-nTreg; MCSA,totalSample,immune-iTreg; MCSA,totalSample,immune-Th17; MCSA,totalSample,immune-Tfh; MCSA,totalSample,immune-CD8_naive; MCSA,totalSample,immune-Cytotoxic; MCSA,totalSample,immune-Exhausted; MCSA,totalSample,immune-MAIT; MCSA,totalSample,immune-Central_memory; SRP223445,totalSample,immune-NK; SRP223445,totalSample,immune-Neutrophil; SRP223445,totalSample,immune-CD4_naive; SRP223445,totalSample,immune-Th17; SRP223445,totalSample,immune-Exhausted; SRP223445,totalSample,immune-[illegible]
ANK3	Homo_723,female,AD.vs.control; Homo_723,male,AD.vs.control; Homo_723,female,AD.vs.MCI; Homo_723,male,AD.vs.MCI; Homo_714,female,AD.vs.control; Homo_714,male,AD.vs.control; Homo_714,female,AD.vs.MCI; Homo_714,male,AD.vs.MCI; Homo_633,female,AD.vs.control; Homo_633,female,AD.vs.MCI; Emory,totalSample,immune-Macrophage; Emory,totalSample,immune-Neutrophil; Emory,totalSample,immune-CD4_T; Emory,totalSample,immune-CD8_T; Emory,totalSample,immune-CD4_naive; Emory,totalSample,immune-Tr1; Emory,totalSample,immune-nTreg; Emory,totalSample,immune-iTreg; Emory,totalSample,immune-Th17; Emory,totalSample,immune-Tfh; Emory,totalSample,immune-CD8_naive; Emory,totalSample,immune-MAIT; Emory,totalSample,immune-Central_memory; Emory,totalSample,immune-InfiltrationScore; Homo_723,totalSample,immune-Central_memory; Homo_723,totalSample,immune-CD4_naive; Homo_723,totalSample,immune-CD4_T; Homo_723,totalSample,immune-Tr1; Homo_723,totalSample,immune-Tfh; Homo_723,totalSample,immune-nTreg; Homo_723,totalSample,immune-iTreg; Homo_723,totalSample,immune-Th1; Homo_723,totalSample,immune-Cytotoxic; Homo_723,totalSample,immune-Th2; Homo_723,totalSample,immune-Th17; Homo_723,totalSample,immune-Gamma_delta; Homo_723,totalSample,immune-NKT; Homo_723,totalSample,immune-Monocyte; Homo_723,totalSample,immune-Neutrophil; Homo_723,totalSample,immune-InfiltrationScore; Homo_714,totalSample,immune-CD4_naive; Homo_714,totalSample,immune-CD4_T; Homo_714,totalSample,immune-Tfh; Homo_714,totalSample,immune-iTreg; Homo_714,totalSample,immune-Tr1; Homo_714,totalSample,immune-Central_memory; Homo_714,totalSample,immune-Th1; Homo_714,totalSample,immune-nTreg; Homo_714,totalSample,immune-NKT; Homo_714,totalSample,immune-Cytotoxic; Homo_714,totalSample,immune-InfiltrationScore; Homo_714,totalSample,immune-Th2; Homo_714,totalSample,immune-Th17; Homo_714,totalSample,immune-Gamma_delta; Homo_633,totalSample,immune-Tfh; Homo_633,totalSample,immune-CD4_T; Homo_633,totalSample,immune-Tr1; Homo_633,totalSample,immune-CD4_naive; Homo_633,totalSample,immune-iTreg; Homo_633,totalSample,immune-Central_memory; Homo_633,totalSample,immune-nTreg; Homo_633,totalSample,immune-NKT; Homo_633,totalSample,immune-Th1; Homo_633,totalSample,immune-Effector_memory; Homo_633,totalSample,immune-InfiltrationScore; Homo_633,totalSample,immune-Gamma_delta; Homo_633,totalSample,immune-Cytotoxic; Homo_633,totalSample,immune-Th2; MCSA,totalSample,immune-DC; MCSA,totalSample,immune-Macrophage; MCSA,totalSample,immune-CD4_T; MCSA,totalSample,immune-Gamma_delta; MCSA,totalSample,immune-CD4_naive; MCSA,totalSample,immune-Tr1; MCSA,totalSample,immune-nTreg; MCSA,totalSample,immune-iTreg; MCSA,totalSample,immune-Th17; MCSA,totalSample,immune-Tfh; MCSA,totalSample,immune-CD8_naive; MCSA,totalSample,immune-Cytotoxic; MCSA,totalSample,immune-Exhausted; MCSA,totalSample,immune-MAIT; MCSA,totalSample,immune-[illegible]

LAGE3	Homo_723,female,AD.vs.control; Homo_723,male,AD.vs.control; Homo_723,female,AD.vs.MCI; Homo_723,male,AD.vs.MCI; Homo_714,female,AD.vs.control; Homo_714,male,AD.vs.control; Homo_714,female,AD.vs.MCI; Homo_714,male,AD.vs.MCI; Homo_633,female,AD.vs.control; Homo_633,female,AD.vs.MCI; ROSMAP,female,AD.vs.control; SRP223445,female,AD.vs.control; MCSA,totalSample,age; MCSA,control,age; MCSA,MCI,age; Emory,totalSample,immune-Monocyte; Emory,totalSample,immune-Macrophage; Emory,totalSample,immune-NK; Emory,totalSample,immune-Neutrophil; Emory,totalSample,immune-CD4_T; Emory,totalSample,immune-CD8_T; Emory,totalSample,immune-Tr1; Emory,totalSample,immune-nTreg; Emory,totalSample,immune-iTreg; Emory,totalSample,immune-Th1; Emory,totalSample,immune-Tfh; Emory,totalSample,immune-CD8_naive; Emory,totalSample,immune-Exhausted; Emory,totalSample,immune-InfiltrationScore; Homo_723,totalSample,immune-CD8_T; Homo_723,totalSample,immune-Cytotoxic; Homo_723,totalSample,immune-nTreg; Homo_723,totalSample,immune-InfiltrationScore; Homo_723,totalSample,immune-Exhausted; Homo_723,totalSample,immune-Tfh; Homo_723,totalSample,immune-Effector_memory; Homo_723,totalSample,immune-Macrophage; Homo_723,totalSample,immune-Central_memory; Homo_723,totalSample,immune-DC; Homo_723,totalSample,immune-iTreg; Homo_723,totalSample,immune-Neutrophil; Homo_723,totalSample,immune-MAIT; Homo_723,totalSample,immune-Monocyte; Homo_714,totalSample,immune-CD8_T; Homo_714,totalSample,immune-DC; Homo_714,totalSample,immune-nTreg; Homo_714,totalSample,immune-Effector_memory; Homo_714,totalSample,immune-Tfh; Homo_714,totalSample,immune-InfiltrationScore; Homo_714,totalSample,immune-Cytotoxic; Homo_714,totalSample,immune-Neutrophil; Homo_714,totalSample,immune-Macrophage; Homo_714,totalSample,immune-Central_memory; Homo_714,totalSample,immune-Monocyte; Homo_714,totalSample,immune-Exhausted; Homo_714,totalSample,immune-MAIT; Homo_714,totalSample,immune-CD8_naive; Homo_633,totalSample,immune-DC; Homo_633,totalSample,immune-nTreg; Homo_633,totalSample,immune-CD8_T; Homo_633,totalSample,immune-Neutrophil; Homo_633,totalSample,immune-InfiltrationScore; Homo_633,totalSample,immune-Central_memory; Homo_633,totalSample,immune-Effector_memory; Homo_633,totalSample,immune-Monocyte; Homo_633,totalSample,immune-Cytotoxic; Homo_633,totalSample,immune-Macrophage; Homo_633,totalSample,immune-Tfh; Homo_633,totalSample,immune-NK; MCSA,totalSample,immune-DC; MCSA,totalSample,immune-Monocyte; MCSA,totalSample,immune-Neutrophil; MCSA,totalSample,immune-CD8_T; MCSA,totalSample,immune-NKT; MCSA,totalSample,immune-CD4_naive; MCSA,totalSample,immune-iTreg; MCSA,totalSample,immune-Th2; MCSA,totalSample,immune-Tfh; MCSA,totalSample,immune-Cytotoxic; MCSA,totalSample,immune-Exhausted; MCSA,totalSample,immune-Central_memory; MCSA,totalSample,immune-Effector_memory; MCSA,totalSample,immune-InfiltrationScore; ROSMAP,totalSample,immune-CD4_T; ROSMAP,totalSample,immune-Tr1; ROSMAP,totalSample,immune-nTreg; ROSMAP,totalSample,immune-iTreg; ROSMAP,totalSample,immune-Th2; ROSMAP,totalSample,immune-Th17; ROSMAP,totalSample,immune-InfiltrationScore; SRP223445,totalSample,immune-CD4_naive
DCUN1D4	Homo_723,female,AD.vs.control; Homo_723,male,AD.vs.control; Homo_723,female,AD.vs.MCI; Homo_723,male,AD.vs.MCI; Homo_723,female,MCI.vs.control; Homo_723,male,MCI.vs.control; Homo_714,female,AD.vs.control; Homo_714,male,AD.vs.control; Homo_714,female,AD.vs.MCI; Homo_714,male,AD.vs.MCI; Homo_714,female,MCI.vs.control; Homo_714,male,MCI.vs.control; Homo_633,female,AD.vs.control; Homo_633,female,AD.vs.MCI; Homo_633,female,MCI.vs.control; ROSMAP,female,AD.vs.control; MCSA,MCI,age; Emory,totalSample,immune-DC; Emory,totalSample,immune-Monocyte; Emory,totalSample,immune-Macrophage; Emory,totalSample,immune-Neutrophil; Emory,totalSample,immune-CD4_T; Emory,totalSample,immune-CD8_T; Emory,totalSample,immune-Tr1; Emory,totalSample,immune-iTreg; Emory,totalSample,immune-Tfh; Emory,totalSample,immune-Central_memory; Emory,totalSample,immune-Effector_memory; Emory,totalSample,immune-InfiltrationScore; Homo_723,totalSample,immune-Exhausted; Homo_723,totalSample,immune-Th17; Homo_723,totalSample,immune-CD4_naive; Homo_723,totalSample,immune-Cytotoxic; Homo_723,totalSample,immune-CD4_T; Homo_723,totalSample,immune-Tfh; Homo_723,totalSample,immune-Tr1; Homo_723,totalSample,immune-Monocyte; Homo_723,totalSample,immune-Central_memory; Homo_723,totalSample,immune-Th2; Homo_723,totalSample,immune-CD8_T; Homo_723,totalSample,immune-Th1; Homo_723,totalSample,immune-Neutrophil; Homo_723,totalSample,immune-nTreg; Homo_714,totalSample,immune-CD4_naive; Homo_714,totalSample,immune-Monocyte; Homo_714,totalSample,immune-Th17; Homo_714,totalSample,immune-CD4_T; Homo_714,totalSample,immune-Central_memory; Homo_714,totalSample,immune-Tfh; Homo_714,totalSample,immune-Exhausted; Homo_714,totalSample,immune-Th1; Homo_714,totalSample,immune-Tr1; Homo_633,totalSample,immune-Tfh; Homo_633,totalSample,immune-Th2; Homo_633,totalSample,immune-CD4_T; Homo_633,totalSample,immune-CD4_naive; Homo_633,totalSample,immune-Central_memory; Homo_633,totalSample,immune-Monocyte; Homo_633,totalSample,immune-Effector_memory; Homo_633,totalSample,immune-Tr1; Homo_633,totalSample,immune-DC; Homo_633,totalSample,immune-InfiltrationScore; Homo_633,totalSample,immune-NKT; Homo_633,totalSample,immune-Gamma_delta; Homo_633,totalSample,immune-Th17; Homo_633,totalSample,immune-iTreg; Homo_633,totalSample,immune-Exhausted; Homo_633,totalSample,immune-Bcell; MCSA,totalSample,immune-DC; MCSA,totalSample,immune-Bcell; MCSA,totalSample,immune-Monocyte; MCSA,totalSample,immune-Macrophage; MCSA,totalSample,immune-CD4_T; MCSA,totalSample,immune-CD8_T; MCSA,totalSample,immune-CD4_naive; MCSA,totalSample,immune-Tr1; MCSA,totalSample,immune-nTreg; MCSA,totalSample,immune-iTreg; MCSA,totalSample,immune-Th2; MCSA,totalSample,immune-Th17; MCSA,totalSample,immune-Tfh; MCSA,totalSample,immune-CD8_naive; MCSA,totalSample,immune-Cytotoxic; MCSA,totalSample,immune-Exhausted; [illegible]
KCNE1	Homo_723,female,AD.vs.control; Homo_723,male,AD.vs.control; Homo_723,female,AD.vs.MCI; Homo_723,male,AD.vs.MCI; Homo_714,female,AD.vs.control; Homo_714,male,AD.vs.control; Homo_714,female,AD.vs.MCI; Homo_714,male,AD.vs.MCI; Homo_633,female,AD.vs.control; Homo_633,female,AD.vs.MCI; Emory,totalSample,age; Emory,MCI,age; Emory,totalSample,immune-B_cell; Emory,totalSample,immune-Neutrophil; Emory,totalSample,immune-CD8_T; Emory,totalSample,immune-Gamma_delta; Emory,totalSample,immune-Th1; Emory,totalSample,immune-Tfh; Emory,totalSample,immune-CD8_naive; Emory,totalSample,immune-Exhausted; Homo_723,totalSample,immune-Neutrophil; Homo_723,totalSample,immune-Gamma_delta; Homo_723,totalSample,immune-InfiltrationScore; Homo_723,totalSample,immune-Th1; Homo_723,totalSample,immune-Bcell; Homo_714,totalSample,immune-Neutrophil; Homo_714,totalSample,immune-Th1; Homo_714,totalSample,immune-Gamma_delta; Homo_714,totalSample,immune-Exhausted; Homo_633,totalSample,immune-Exhausted; Homo_633,totalSample,immune-Neutrophil; Homo_633,totalSample,immune-CD8_T; Homo_633,totalSample,immune-InfiltrationScore; Homo_633,totalSample,immune-MAIT; Homo_633,totalSample,immune-Gamma_delta; Homo_633,totalSample,immune-Macrophage; MCSA,totalSample,immune-Macrophage; MCSA,totalSample,immune-Neutrophil; MCSA,totalSample,immune-CD8_T; MCSA,totalSample,immune-NKT; MCSA,totalSample,immune-Gamma_delta; MCSA,totalSample,immune-Th1; MCSA,totalSample,immune-Th17; MCSA,totalSample,immune-Exhausted; ROSMAP,totalSample,immune-Effector_memory
TUT1	Homo_723,female,AD.vs.control; Homo_723,male,AD.vs.MCI; Homo_723,female,MCI.vs.control; Homo_723,male,MCI.vs.control; Homo_723,control,male.vs.female; Homo_723,MCI,male.vs.female; Homo_714,female,AD.vs.control; Homo_714,male,AD.vs.MCI; Homo_714,female,MCI.vs.control; Homo_714,male,MCI.vs.control; Homo_714,control,male.vs.female; Homo_714,MCI,male.vs.female; Homo_633,female,AD.vs.control; Homo_633,female,AD.vs.MCI; Homo_633,female,MCI.vs.control; Homo_633,male,MCI.vs.control; Homo_633,control,male.vs.female; Homo_633,MCI,male.vs.female; ROSMAP,male,AD.vs.control; ROSMAP,female,AD.vs.MCI; MCSA,totalSample,age; MCSA,control,age; MCSA,MCI,age; ROSMAP,AD,last.visit.age; Emory,totalSample,immune-Monocyte; Emory,totalSample,immune-Macrophage; Emory,totalSample,immune-NK; Emory,totalSample,immune-Neutrophil; Emory,totalSample,immune-CD4_T; Emory,totalSample,immune-CD8_T; Emory,totalSample,immune-Tr1; Emory,totalSample,immune-nTreg; Emory,totalSample,immune-iTreg; Emory,totalSample,immune-Tfh; Emory,totalSample,immune-Exhausted; Emory,totalSample,immune-MAIT; Emory,totalSample,immune-Central_memory; Emory,totalSample,immune-InfiltrationScore; Homo_723,totalSample,immune-Monocyte; Homo_723,totalSample,immune-DC; Homo_723,totalSample,immune-Central_memory; Homo_723,totalSample,immune-Neutrophil; Homo_723,totalSample,immune-CD8_T; Homo_723,totalSample,immune-nTreg; Homo_723,totalSample,immune-Exhausted; Homo_723,totalSample,immune-CD4_naive; Homo_714,totalSample,immune-Monocyte; Homo_714,totalSample,immune-DC; Homo_714,totalSample,immune-nTreg; Homo_714,totalSample,immune-Neutrophil; Homo_633,totalSample,immune-Monocyte; Homo_633,totalSample,immune-nTreg; Homo_633,totalSample,immune-DC; Homo_633,totalSample,immune-Macrophage; MCSA,totalSample,immune-DC; MCSA,totalSample,immune-Monocyte; MCSA,totalSample,immune-Neutrophil; MCSA,totalSample,immune-CD8_T; MCSA,totalSample,immune-NKT; MCSA,totalSample,immune-CD4_naive; MCSA,totalSample,immune-nTreg; MCSA,totalSample,immune-Th2; MCSA,totalSample,immune-Tfh; MCSA,totalSample,immune-Cytotoxic; MCSA,totalSample,immune-Central_memory; MCSA,totalSample,immune-Effector_memory; MCSA,totalSample,immune-InfiltrationScore; ROSMAP,totalSample,immune-Effector_memory; [illegible]
CNTNAP2	Homo_723,female,AD.vs.control; Homo_723,male,AD.vs.control; Homo_723,female,AD.vs.MCI; Homo_723,male,AD.vs.MCI; Homo_723,female,MCI.vs.control; Homo_714,male,AD.vs.control; Homo_714,male,AD.vs.MCI; Homo_714,female,MCI.vs.control; Homo_633,female,AD.vs.control; Homo_633,female,AD.vs.MCI; MCSA,MCI,age; ROSMAP,AD,last.visit.age; Emory,totalSample,immune-B_cell; Emory,totalSample,immune-Gamma_delta; Emory,totalSample,immune-iTreg; Homo_723,totalSample,immune-nTreg; Homo_723,totalSample,immune-CD4_T; Homo_723,totalSample,immune-CD4_naive; Homo_723,totalSample,immune-Central_memory; Homo_723,totalSample,immune-Tfh; Homo_723,totalSample,immune-iTreg; Homo_723,totalSample,immune-Th2; Homo_723,totalSample,immune-Th1; Homo_723,totalSample,immune-Tr1; Homo_723,totalSample,immune-Neutrophil; Homo_714,totalSample,immune-CD4_naive; Homo_714,totalSample,immune-Tfh; Homo_714,totalSample,immune-CD4_T; Homo_714,totalSample,immune-nTreg; Homo_714,totalSample,immune-Th1; Homo_633,totalSample,immune-Tfh; Homo_633,totalSample,immune-CD4_T; Homo_633,totalSample,immune-CD4_naive; Homo_633,totalSample,immune-nTreg; Homo_633,totalSample,immune-iTreg; Homo_633,totalSample,immune-Tr1; Homo_633,totalSample,immune-Central_memory; Homo_633,totalSample,immune-NKT; Homo_633,totalSample,immune-Gamma_delta; MCSA,totalSample,immune-Bcell; MCSA,totalSample,immune-Macrophage; MCSA,totalSample,immune-Neutrophil; MCSA,totalSample,immune-CD4_T; MCSA,totalSample,immune-CD4_naive; MCSA,totalSample,immune-Tr1; MCSA,totalSample,immune-nTreg; MCSA,totalSample,immune-iTreg; MCSA,totalSample,immune-Tfh;
EVC	Homo_723,female,AD.vs.control; Homo_723,male,AD.vs.control; Homo_723,female,AD.vs.MCI; Homo_723,male,AD.vs.MCI; Homo_714,male,AD.vs.control; Homo_714,male,AD.vs.MCI; Homo_633,female,AD.vs.control; Homo_633,female,AD.vs.MCI; Emory,control,age; Homo_714,totalSample,immune-InfiltrationScore; Homo_633,totalSample,immune-InfiltrationScore; MCSA,totalSample,immune-Monocyte; SRP223445,totalSample,immune-Neutrophil
R3HCC1L	Homo_723,female,AD.vs.control; Homo_723,male,AD.vs.control; Homo_723,female,AD.vs.MCI; Homo_723,male,AD.vs.MCI; Homo_723,female,MCI.vs.control; Homo_714,female,AD.vs.control; Homo_714,male,AD.vs.control; Homo_714,female,AD.vs.MCI; Homo_714,male,AD.vs.MCI; Homo_714,female,MCI.vs.control; Homo_633,female,AD.vs.control; Homo_633,female,AD.vs.MCI; Homo_633,female,MCI.vs.control; ROSMAP,female,AD.vs.control; Emory,MCI,age; MCSA,totalSample,age; MCSA,control,age; MCSA,MCI,age; ROSMAP,MCI,last.visit.age; ROSMAP,MCI,sample.age; Emory,totalSample,immune-Macrophage; Emory,totalSample,immune-CD4_T; Emory,totalSample,immune-Gamma_delta; Emory,totalSample,immune-Tr1; Emory,totalSample,immune-iTreg; Emory,totalSample,immune-InfiltrationScore; Homo_723,totalSample,immune-Monocyte; Homo_723,totalSample,immune-Th2; Homo_723,totalSample,immune-Tfh; Homo_723,totalSample,immune-DC; Homo_723,totalSample,immune-iTreg; Homo_723,totalSample,immune-Effector_memory; Homo_723,totalSample,immune-InfiltrationScore; Homo_723,totalSample,immune-Tr1; Homo_723,totalSample,immune-CD4_T; Homo_723,totalSample,immune-Bcell; Homo_714,totalSample,immune-Th2; Homo_714,totalSample,immune-Tfh; Homo_714,totalSample,immune-Monocyte; Homo_714,totalSample,immune-Effector_memory; Homo_714,totalSample,immune-Tr1; Homo_714,totalSample,immune-CD4_T; Homo_633,totalSample,immune-Monocyte; Homo_633,totalSample,immune-Th2; Homo_633,totalSample,immune-Tfh; Homo_633,totalSample,immune-Exhausted; Homo_633,totalSample,immune-InfiltrationScore; Homo_633,totalSample,immune-Bcell; Homo_633,totalSample,immune-Effector_memory; Homo_633,totalSample,immune-CD4_T; Homo_633,totalSample,immune-Central_memory; MCSA,totalSample,immune-DC; MCSA,totalSample,immune-Monocyte; MCSA,totalSample,immune-Macrophage; MCSA,totalSample,immune-CD4_T; MCSA,totalSample,immune-CD8_T; MCSA,totalSample,immune-Tr1; MCSA,totalSample,immune-iTreg; MCSA,totalSample,immune-Th2; MCSA,totalSample,immune-Cytotoxic; MCSA,totalSample,immune-Exhausted; MCSA,totalSample,immune-MAIT; SRP223445,totalSample,immune-Exhausted; SRP223445,totalSample,immune-InfiltrationScore; ROSMAP,male-excludeAge90+,survival
TCEANC	Homo_723,female,AD.vs.control; Homo_723,male,AD.vs.control; Homo_723,female,AD.vs.MCI; Homo_723,male,AD.vs.MCI; Homo_714,female,AD.vs.control; Homo_714,male,AD.vs.control; Homo_714,female,AD.vs.MCI; Homo_714,male,AD.vs.MCI; Homo_633,female,AD.vs.control; Homo_633,female,AD.vs.MCI; MCSA,MCI,age; ROSMAP,AD,last.visit.age; ROSMAP,MCI,last.visit.age; ROSMAP,MCI,sample.age; ROSMAP,AD,MMSE; Emory,totalSample,immune-Macrophage; Emory,totalSample,immune-Neutrophil; Emory,totalSample,immune-CD4_T; Emory,totalSample,immune-CD8_T; Emory,totalSample,immune-Gamma_delta; Emory,totalSample,immune-Tr1; Emory,totalSample,immune-iTreg; Emory,totalSample,immune-Cytotoxic; Emory,totalSample,immune-Effector_memory; Emory,totalSample,immune-InfiltrationScore; Homo_723,totalSample,immune-Tfh; Homo_723,totalSample,immune-iTreg; Homo_723,totalSample,immune-Th2; Homo_723,totalSample,immune-Tr1; Homo_723,totalSample,immune-InfiltrationScore; Homo_723,totalSample,immune-CD4_T; Homo_723,totalSample,immune-Effector_memory; Homo_723,totalSample,immune-CD4_naive; Homo_723,totalSample,immune-DC; Homo_723,totalSample,immune-Central_memory; Homo_723,totalSample,immune-Macrophage; Homo_723,totalSample,immune-Gamma_delta; Homo_723,totalSample,immune-MAIT; Homo_723,totalSample,immune-nTreg; Homo_714,totalSample,immune-Tfh; Homo_714,totalSample,immune-Th2; Homo_714,totalSample,immune-CD4_T; Homo_714,totalSample,immune-Tr1; Homo_714,totalSample,immune-Effector_memory; Homo_714,totalSample,immune-Central_memory; Homo_714,totalSample,immune-InfiltrationScore; Homo_714,totalSample,immune-iTreg; Homo_714,totalSample,immune-CD4_naive; Homo_714,totalSample,immune-Macrophage; Homo_714,totalSample,immune-nTreg; Homo_633,totalSample,immune-Tfh; Homo_633,totalSample,immune-CD4_T; Homo_633,totalSample,immune-InfiltrationScore; Homo_633,totalSample,immune-CD4_naive; Homo_633,totalSample,immune-Effector_memory; Homo_633,totalSample,immune-Th2; Homo_633,totalSample,immune-Central_memory; Homo_633,totalSample,immune-Tr1; Homo_633,totalSample,immune-NKT; Homo_633,totalSample,immune-iTreg; Homo_633,totalSample,immune-Th17; Homo_633,totalSample,immune-Monocyte; Homo_633,totalSample,immune-Gamma_delta; MCSA,totalSample,immune-DC; MCSA,totalSample,immune-Macrophage; MCSA,totalSample,immune-CD4_T; MCSA,totalSample,immune-CD4_naive; MCSA,totalSample,immune-Tr1; MCSA,totalSample,immune-nTreg; MCSA,totalSample,immune-iTreg; MCSA,totalSample,immune-Th17; MCSA,totalSample,immune-Tfh; MCSA,totalSample,immune-CD8_naive; MCSA,totalSample,immune-Cytotoxic; MCSA,totalSample,immune-Exhausted; MCSA,totalSample,immune-MAIT; MCSA,totalSample,immune-Central_memory; ROSMAP,totalSample,immune-InfiltrationScore; SRP223445,totalSample,immune-CD4_naive; SRP223445,totalSample,immune-InfiltrationScore
PLCD4	Homo_723,female,AD.vs.control; Homo_723,male,AD.vs.control; Homo_723,female,AD.vs.MCI; Homo_723,male,AD.vs.MCI; Homo_723,female,MCI.vs.control; Homo_723,male,MCI.vs.control; Homo_714,female,AD.vs.control; Homo_714,male,AD.vs.control; Homo_714,female,AD.vs.MCI; Homo_714,male,AD.vs.MCI; Homo_714,female,MCI.vs.control; Homo_714,male,MCI.vs.control; ROSMAP,female,AD.vs.control; ROSMAP,male,AD.vs.control; ROSMAP,MCI,male.vs.female; ROSMAP,AD,sample.age; Homo_723,totalSample,immune-Th17; Homo_723,totalSample,immune-Exhausted; Homo_723,totalSample,immune-Cytotoxic; Homo_714,totalSample,immune-Th17; Homo_633,totalSample,immune-Th2; MCSA,totalSample,immune-Macrophage; MCSA,totalSample,immune-CD4_T; MCSA,totalSample,immune-CD4_naive; MCSA,totalSample,immune-Tr1; MCSA,totalSample,immune-nTreg; MCSA,totalSample,immune-iTreg; MCSA,totalSample,immune-Th17; MCSA,totalSample,immune-Tfh
LZTS3	Homo_723,female,AD.vs.control; Homo_723,male,AD.vs.control; Homo_723,female,AD.vs.MCI; Homo_723,male,AD.vs.MCI; Homo_723,male,MCI.vs.control; Homo_714,female,MCI.vs.control; Homo_714,male,MCI.vs.control; Homo_633,female,AD.vs.control; Homo_633,female,MCI.vs.control; Homo_633,male,MCI.vs.control; ROSMAP,female,AD.vs.control; ROSMAP,female,AD.vs.MCI; MCSA,totalSample,age; MCSA,control,age; MCSA,MCI,age; Emory,totalSample,immune-Monocyte; Emory,totalSample,immune-CD4_T; Emory,totalSample,immune-Gamma_delta; Emory,totalSample,immune-CD4_naive; Emory,totalSample,immune-Tr1; Emory,totalSample,immune-nTreg; Emory,totalSample,immune-iTreg; Emory,totalSample,immune-Th2; Emory,totalSample,immune-Th17; Emory,totalSample,immune-Tfh; Emory,totalSample,immune-Cytotoxic; Emory,totalSample,immune-Central_memory; Homo_723,totalSample,immune-Monocyte; Homo_723,totalSample,immune-nTreg; Homo_723,totalSample,immune-DC; Homo_723,totalSample,immune-Central_memory; Homo_723,totalSample,immune-Exhausted; Homo_723,totalSample,immune-CD8_T; Homo_723,totalSample,immune-CD4_naive; Homo_723,totalSample,immune-Neutrophil; Homo_723,totalSample,immune-CD4_T; Homo_714,totalSample,immune-Monocyte; Homo_714,totalSample,immune-nTreg; Homo_714,totalSample,immune-DC; Homo_633,totalSample,immune-Monocyte; Homo_633,totalSample,immune-nTreg; Homo_633,totalSample,immune-DC; Homo_633,totalSample,immune-iTreg; Homo_633,totalSample,immune-Tr1; MCSA,totalSample,immune-DC; MCSA,totalSample,immune-Monocyte; MCSA,totalSample,immune-Neutrophil; MCSA,totalSample,immune-CD4_T; MCSA,totalSample,immune-CD8_T; MCSA,totalSample,immune-CD4_naive; MCSA,totalSample,immune-Tr1; MCSA,totalSample,immune-nTreg; MCSA,totalSample,immune-Th2; MCSA,totalSample,immune-Th17; MCSA,totalSample,immune-Tfh; MCSA,totalSample,immune-Cytotoxic; MCSA,totalSample,immune-Central_memory; MCSA,totalSample,immune-[illegible]
HAS3	Homo_723,female,AD.vs.control; Homo_723,male,AD.vs.control; Homo_723,female,AD.vs.MCI; Homo_723,male,AD.vs.MCI; Homo_633,female,AD.vs.control; Homo_633,female,AD.vs.MCI; MCSA,totalSample,age; MCSA,control,age; ROSMAP,MCI,last.visit.age; ROSMAP,MCI,sample.age; Emory,totalSample,immune-CD4_T; Emory,totalSample,immune-Gamma_delta; Emory,totalSample,immune-Tr1; Emory,totalSample,immune-iTreg; Homo_723,totalSample,immune-Th2; Homo_723,totalSample,immune-iTreg; Homo_723,totalSample,immune-CD4_T; Homo_723,totalSample,immune-Tfh; Homo_723,totalSample,immune-nTreg; Homo_723,totalSample,immune-CD4_naive; Homo_723,totalSample,immune-Tr1; Homo_723,totalSample,immune-Central_memory; Homo_723,totalSample,immune-Macrophage; Homo_723,totalSample,immune-InfiltrationScore; Homo_714,totalSample,immune-Tfh; Homo_714,totalSample,immune-CD4_T; Homo_714,totalSample,immune-Th2; Homo_714,totalSample,immune-Tr1; Homo_714,totalSample,immune-nTreg; Homo_714,totalSample,immune-iTreg; Homo_714,totalSample,immune-CD4_naive; Homo_714,totalSample,immune-Effector_memory; Homo_714,totalSample,immune-InfiltrationScore; Homo_714,totalSample,immune-Central_memory; Homo_714,totalSample,immune-Macrophage; Homo_633,totalSample,immune-CD4_T; Homo_633,totalSample,immune-Tfh; Homo_633,totalSample,immune-Tr1; Homo_633,totalSample,immune-CD4_naive; Homo_633,totalSample,immune-nTreg; Homo_633,totalSample,immune-InfiltrationScore; Homo_633,totalSample,immune-iTreg; Homo_633,totalSample,immune-Central_memory; Homo_633,totalSample,immune-Effector_memory; Homo_633,totalSample,immune-Th2; Homo_633,totalSample,immune-NKT; Homo_633,totalSample,immune-Th1; MCSA,totalSample,immune-Monocyte; MCSA,totalSample,immune-Macrophage; MCSA,totalSample,immune-Neutrophil; MCSA,totalSample,immune-CD4_T; MCSA,totalSample,immune-Gamma_delta; MCSA,totalSample,immune-CD4_naive; MCSA,totalSample,immune-Tr1; MCSA,totalSample,immune-nTreg; MCSA,totalSample,immune-iTreg; MCSA,totalSample,immune-Th17; MCSA,totalSample,immune-Tfh; MCSA,totalSample,immune-CD8_naive; MCSA,totalSample,immune-Central_memory; MCSA,totalSample,immune-Effector_memory; MCSA,totalSample,immune-InfiltrationScore; ROSMAP,totalSample,immune-Th17; ROSMAP,totalSample,immune-Effector_memory
EXOSC10	Homo_723,female,AD.vs.control; Homo_723,male,AD.vs.control; Homo_723,female,AD.vs.MCI; Homo_723,male,AD.vs.MCI; Homo_723,female,MCI.vs.control; Homo_723,control,male.vs.female; Homo_714,female,AD.vs.control; Homo_714,male,AD.vs.control; Homo_714,female,AD.vs.MCI; Homo_714,male,AD.vs.MCI; Homo_714,female,MCI.vs.control; Homo_714,control,male.vs.female; Homo_633,female,AD.vs.control; Homo_633,female,AD.vs.MCI; Homo_633,female,MCI.vs.control; Homo_633,control,male.vs.female; ROSMAP,female,AD.vs.control; ACOM,AD,age; ACOM,totalSample,age; MCSA,totalSample,age; MCSA,control,age; Emory,totalSample,immune-Monocyte; Emory,totalSample,immune-Macrophage; Emory,totalSample,immune-NK; Emory,totalSample,immune-Neutrophil; Emory,totalSample,immune-CD4_T; Emory,totalSample,immune-CD8_T; Emory,totalSample,immune-Tr1; Emory,totalSample,immune-iTreg; Emory,totalSample,immune-Tfh; Emory,totalSample,immune-Exhausted; Emory,totalSample,immune-MAIT; Emory,totalSample,immune-Central_memory; Emory,totalSample,immune-Effector_memory; Emory,totalSample,immune-InfiltrationScore; Homo_723,totalSample,immune-Monocyte; Homo_723,totalSample,immune-CD4_naive; Homo_723,totalSample,immune-nTreg; Homo_723,totalSample,immune-DC; Homo_723,totalSample,immune-Central_memory; Homo_723,totalSample,immune-CD4_T; Homo_723,totalSample,immune-InfiltrationScore; Homo_723,totalSample,immune-Tfh; Homo_723,totalSample,immune-Neutrophil; Homo_723,totalSample,immune-iTreg; Homo_723,totalSample,immune-Th1; Homo_723,totalSample,immune-Exhausted; Homo_714,totalSample,immune-Monocyte; Homo_714,totalSample,immune-CD4_naive; Homo_714,totalSample,immune-nTreg; Homo_714,totalSample,immune-InfiltrationScore; Homo_714,totalSample,immune-CD4_T; Homo_714,totalSample,immune-Th1; Homo_714,totalSample,immune-iTreg; Homo_714,totalSample,immune-Tfh; Homo_714,totalSample,immune-Neutrophil; Homo_714,totalSample,immune-Central_memory; Homo_714,totalSample,immune-NKT; Homo_714,totalSample,immune-DC; Homo_714,totalSample,immune-Effector_memory; Homo_633,totalSample,immune-Monocyte; Homo_633,totalSample,immune-CD4_naive; Homo_633,totalSample,immune-InfiltrationScore; Homo_633,totalSample,immune-nTreg; Homo_633,totalSample,immune-Tfh; Homo_633,totalSample,immune-CD4_T; Homo_633,totalSample,immune-iTreg; Homo_633,totalSample,immune-Effector_memory; Homo_633,totalSample,immune-Th1; Homo_633,totalSample,immune-Central_memory; MCSA,totalSample,immune-Monocyte; MCSA,totalSample,immune-Neutrophil; MCSA,totalSample,immune-CD4_T; MCSA,totalSample,immune-Gamma_delta; MCSA,totalSample,immune-CD4_naive; MCSA,totalSample,immune-Tr1; MCSA,totalSample,immune-nTreg; MCSA,totalSample,immune-iTreg; MCSA,totalSample,immune-Th17; MCSA,totalSample,immune-Tfh; MCSA,totalSample,immune-CD8_naive; MCSA,totalSample,immune-Central_memory; MCSA,totalSample,immune-Effector_memory; MCSA,totalSample,immune-InfiltrationScore; ROSMAP,totalSample,immune-Central_memory; ROSMAP,totalSample,immune-Effector_memory; SRP223445,totalSample,immune-CD4_naive; SRP223445,totalSample,immune-nTreg; SRP223445,totalSample,immune-Th17; SRP223445,totalSample,immune-Exhausted; SRP223445,totalSample,immune-InfiltrationScore
PEX6	Homo_723,female,AD.vs.control; Homo_723,male,AD.vs.control; Homo_723,male,AD.vs.MCI; Homo_723,female,MCI.vs.control; Homo_723,control,male.vs.female; Homo_714,female,AD.vs.control; Homo_714,male,AD.vs.control; Homo_714,male,AD.vs.MCI; Homo_714,female,MCI.vs.control; Homo_714,control,male.vs.female; Homo_714,MCI,male.vs.female; Homo_633,female,AD.vs.control; Homo_633,female,AD.vs.MCI; Homo_633,female,MCI.vs.control; Homo_633,control,male.vs.female; Homo_633,MCI,male.vs.female; MCSA,totalSample,age; MCSA,control,age; MCSA,MCI,age; Emory,totalSample,immune-CD4_T; Emory,totalSample,immune-Tr1; Emory,totalSample,immune-iTreg; Emory,totalSample,immune-InfiltrationScore; Homo_723,totalSample,immune-DC; Homo_723,totalSample,immune-Monocyte; Homo_723,totalSample,immune-Central_memory; Homo_723,totalSample,immune-nTreg; Homo_723,totalSample,immune-CD8_T; Homo_714,totalSample,immune-Monocyte; Homo_714,totalSample,immune-DC; Homo_714,totalSample,immune-nTreg; Homo_714,totalSample,immune-InfiltrationScore; Homo_633,totalSample,immune-Monocyte; Homo_633,totalSample,immune-nTreg; Homo_633,totalSample,immune-InfiltrationScore; Homo_633,totalSample,immune-DC; MCSA,totalSample,immune-DC; MCSA,totalSample,immune-Monocyte; MCSA,totalSample,immune-Neutrophil; MCSA,totalSample,immune-CD8_T; MCSA,totalSample,immune-Th2; MCSA,totalSample,immune-Cytotoxic; MCSA,totalSample,immune-Central_memory; MCSA,totalSample,immune-Effector_memory; MCSA,totalSample,immune-InfiltrationScore; SRP223445,totalSample,immune-Monocyte; SRP223445,totalSample,immune-Neutrophil; SRP223445,totalSample,immune-nTreg

SLC22A4	Homo_723,female,AD.vs.control; Homo_723,male,AD.vs.control; Homo_723,female,AD.vs.MCI; Homo_723,male,AD.vs.MCI; Homo_714,female,AD.vs.control; Homo_714,male,AD.vs.control; Homo_714,female,AD.vs.MCI; Homo_714,male,AD.vs.MCI; Homo_633,female,AD.vs.control; Homo_633,female,AD.vs.MCI; Emory,control,age; Emory,totalSample,immune-DC; Emory,totalSample,immune-Monocyte; Emory,totalSample,immune-Neutrophil; Emory,totalSample,immune-CD8_T; Emory,totalSample,immune-Gamma_delta; Emory,totalSample,immune-nTreg; Emory,totalSample,immune-Th1; Emory,totalSample,immune-Tfh; Emory,totalSample,immune-Exhausted; Emory,totalSample,immune-InfiltrationScore; Homo_723,totalSample,immune-Neutrophil; Homo_723,totalSample,immune-Gamma_delta; Homo_723,totalSample,immune-DC; Homo_723,totalSample,immune-Monocyte; Homo_723,totalSample,immune-Th1; Homo_723,totalSample,immune-CD8_naive; Homo_714,totalSample,immune-Neutrophil; Homo_714,totalSample,immune-Gamma_delta; Homo_714,totalSample,immune-Th2; Homo_714,totalSample,immune-Exhausted; Homo_714,totalSample,immune-Th1; Homo_714,totalSample,immune-Monocyte; Homo_714,totalSample,immune-CD8_T; Homo_633,totalSample,immune-Neutrophil; Homo_633,totalSample,immune-Gamma_delta; Homo_633,totalSample,immune-CD8_T; Homo_633,totalSample,immune-Exhausted; Homo_633,totalSample,immune-Th17; Homo_633,totalSample,immune-MAIT; Homo_633,totalSample,immune-Monocyte; Homo_633,totalSample,immune-Cytotoxic; Homo_633,totalSample,immune-Th2; MCSA,totalSample,immune-Macrophage; MCSA,totalSample,immune-Neutrophil; MCSA,totalSample,immune-CD8_T; MCSA,totalSample,immune-Gamma_delta; MCSA,totalSample,immune-Th17; MCSA,totalSample,immune-Cytotoxic; MCSA,totalSample,immune-Exhausted; MCSA,totalSample,immune-MAIT; MCSA,totalSample,immune-Effector_memory; MCSA,totalSample,immune-InfiltrationScore; ROSMAP,male-includeAge90+,survival
AUTS2	Homo_723,female,AD.vs.control; Homo_723,male,AD.vs.control; Homo_723,female,AD.vs.MCI; Homo_723,male,AD.vs.MCI; Homo_714,female,AD.vs.control; Homo_714,male,AD.vs.control; Homo_714,female,AD.vs.MCI; Homo_714,male,AD.vs.MCI; Homo_633,female,AD.vs.control; Homo_633,female,AD.vs.MCI; ROSMAP,male,AD.vs.control; MCSA,control,age; Emory,totalSample,immune-Macrophage; Emory,totalSample,immune-NK; Emory,totalSample,immune-Neutrophil; Emory,totalSample,immune-CD4_T; Emory,totalSample,immune-CD8_T; Emory,totalSample,immune-Gamma_delta; Emory,totalSample,immune-Cytotoxic; Emory,totalSample,immune-Exhausted; Emory,totalSample,immune-Effector_memory; Emory,totalSample,immune-InfiltrationScore; Homo_723,totalSample,immune-InfiltrationScore; Homo_723,totalSample,immune-NK; Homo_723,totalSample,immune-Neutrophil; Homo_723,totalSample,immune-DC; Homo_723,totalSample,immune-Monocyte; Homo_723,totalSample,immune-CD4_naive; Homo_723,totalSample,immune-Th17; Homo_723,totalSample,immune-nTreg; Homo_723,totalSample,immune-Th1; Homo_714,totalSample,immune-InfiltrationScore; Homo_714,totalSample,immune-NK; Homo_714,totalSample,immune-Neutrophil; Homo_714,totalSample,immune-NKT; Homo_714,totalSample,immune-CD4_naive; Homo_633,totalSample,immune-InfiltrationScore; Homo_633,totalSample,immune-Macrophage; Homo_633,totalSample,immune-NKT; Homo_633,totalSample,immune-MAIT; Homo_633,totalSample,immune-NK; Homo_633,totalSample,immune-CD4_naive; Homo_633,totalSample,immune-Tfh; Homo_633,totalSample,immune-Monocyte; Homo_633,totalSample,immune-Effector_memory; Homo_633,totalSample,immune-Neutrophil; Homo_633,totalSample,immune-CD4_T; Homo_633,totalSample,immune-Bcell; MCSA,totalSample,immune-DC; MCSA,totalSample,immune-Macrophage; MCSA,totalSample,immune-CD4_T; MCSA,totalSample,immune-CD4_naive; MCSA,totalSample,immune-Tr1; MCSA,totalSample,immune-nTreg; MCSA,totalSample,immune-iTreg; MCSA,totalSample,immune-Th17; MCSA,totalSample,immune-Tfh; MCSA,totalSample,immune-MAIT; MCSA,totalSample,immune-Central_memory; MCSA,totalSample,immune-Effector_memory; MCSA,totalSample,immune-InfiltrationScore; SRP223445,totalSample,immune-Neutrophil; SRP223445,totalSample,immune-CD8_T; SRP223445,totalSample,immune- [illegible]
[truncated: 2,171,678 more chars]
